# Health care provider and client experiences of counselling on depot medroxyprogesterone acetate subcutaneous (DMPA-SC) for self-injection in Malawi

**DOI:** 10.1371/journal.pgph.0002057

**Published:** 2023-11-30

**Authors:** Chelsey Porter Erlank, Gracious Ali, Frehiwot Birhanu, Melinda Stanley, Jessie Salamba Chirwa, Fannie Kachale, Andrews Gunda

**Affiliations:** 1 Analytics and Implementation Research Team, Clinton Health Access Initiative, London, United Kingdom; 2 Sexual Reproductive Maternal and Newborn Health Team, Clinton Health Access Initiative, Lilongwe, Malawi; 3 Independent Contractor, Sydney, New South Wales, Australia; 4 Reproductive Health Directorate, Ministry of Health, Lilongwe, Malawi; 5 Clinton Health Access Initiative, Lilongwe, Malawi; Jhpiego, UNITED STATES

## Abstract

Since the introduction of subcutaneous depot medroxyprogesterone acetate (DMPA-SC) in 2018, Malawi has achieved national coverage of trained providers in the public sector and steady increases in uptake of DMPA-SC. However, the rate of clients opting to self-inject DMPA-SC has remained lower than early acceptability studies suggested. Providers play an instrumental role in building client confidence to self-inject through counselling/training. This cross-sectional qualitative study explored the perspectives of providers and injectable clients on the integration of self-injection into contraceptive counselling, to identify best practices and potential gaps. The study was conducted at public sector sites in three districts (Nkhotakota, Mzimba South, Zomba) in Malawi. In-depth interviews were conducted with provider-administered injectable clients, self-injecting clients, and DMPA-SC trained providers. All providers interviewed reported successfully integrating self-injection into their approach. During group health education sessions, some providers reported focusing on benefits of self-injection to spark interest in the method, and then follow that up with more in-depth information during individual counselling. Due to time pressures, a minority of providers reported replacing individual counselling with small-group counselling and limited use of elements such as visualizations and demonstrations. Most providers skipped client practice on inanimate objects, feeling this was either not necessary or inappropriate given stock constraints. Self-injection clients tended to credit their decision to take up SI to receiving lengthy, comprehensive counselling/training, often inclusive of reassuring messages, visualizations, demonstrations and sometimes repeated trainings over time. Provider-administered clients tended to credit their lack of uptake of self-injection to fear and lack of confidence, often blaming themselves instead of the quality of their counselling/training–even while many felt their counselling/training had been rushed or incomplete. Providers should be supported to overcome time- and resource-pressures to invest in counselling/training best practices, to ensure sufficient support is provided to clients interested in self-injection.

## Introduction

Injectable contraceptives are the most popular method used in Malawi–making up over half (52%) of the contraceptive method mix [1]. A new subcutaneous formulation (DMPA-SC) of the popular injectable depot medroxyprogesterone acetate intramuscular (DMPA-IM) was introduced in Malawi in 2018. DMPA-SC is safe, highly effective at preventing pregnancy, and administered every three months. DMPA-SC differs from DMPA-IM in that it comes in a pre-filled, ‘all-in-one’ Uniject syringe, it is injected subcutaneously, and contains a lower dose of DMPA. The Uniject syringe means DMPA-SC can be administered by any trained person, including community health workers, pharmacists and contraceptive clients themselves, where it is registered for use by these groups.

Research on DMPA-SC in Malawi has shown high acceptability rates for DMPA-SC being injected ‘at home’ as opposed to receiving either DMPA-SC or DMPA-IM in a clinic from a provider (70% preference for this option among injectable users when asked at three months—90% among a group already trained in self-injection and 50% among a group using provider-administered options) [2]. The research also showed high rates of willingness to continue to self-inject (SI) in future among SI trained clients when asked at nine months (98%); and high rates of willingness to SI in the future among provider-administered (PA) injectable clients when asked at nine months (78%) [2]. The SI option has also been found to be associated with increased continuation of contraceptive use among injectable clients–with 73% SI clients continuing with their method at 12 months compared to 45% among PA clients [3]. Qualitative studies in Malawi have demonstrated preference for DMPA-SC over DMPA-IM among both providers and clients, due to the ease of administration and the time- and travel-savings associated with SI versus PA [4]. Based on these positive results, the Ministry of Health (MOH) approved the national roll-out of DMPA-SC (both for PA and SI) in 2018. By 2021, all public sector facilities nationally had at least one DMPA-SC trained provider.

Since the national scale up, analysis of Health Management Information System (HMIS) data shows that uptake of DMPA-SC has steadily increased in Malawi, with the percentage of new injectables users taking up DMPA-SC (PA and SI combined) climbing from 20% in 2019 (when HMIS tools were adapted to integrate DMPA-SC) to 60% in 2020. However, the rate of new injectable users choosing the SI option over PA options has remained lower than rates of 50%+ preference for SI indicated by early acceptability studies [2, 5]. For example, in 2020 only 15% of DMPA new injectable users took up the SI option (versus 45% and 40% taking up DMPA-SC PA and DMPA-IM, respectively). SI accounted for just a quarter (25%) of total DMPA-SC uptake among new injectable users in 2020 (down from 31% in 2019) and continued to average 21% in 2021 [5].

While the discrepancy in preference rates may be partially explained by differences in DMPA-SC provision under pilot conditions compared to national scale-up conditions, these likely do not explain all of the disprepancy. For example, efforts were made during the original pilot to mimic actual provision conditions in the public sector as much as possible. Staff who received training in DMPA-SC (both how to administer it and how to train clients to SI) during the pilot were all existing public sector personnel and were already designated family planning providers in the study sites. Enrollment and training of study participants occurred within the normal FP service delivery context in public-sector family planning clinics, among women who visited those clinics as usual (i.e. no specific demand generation for DMPA-SC was conducted). However, there may be some differences between pilot and national scale context in terms of consistency of implementation around elements such as provider training and supportive supervision, potentially introducing variation in provider knowledge, skills, and attitudes towards DMPA-SC and SI provision.

Indications that provider-side factors might contribute towards the discrepancy in rates of preference for SI emerged during supervision visits to facilities across Malawi in 2020, when supervisors observed occasional provider bias against SI, which they attributed to a lack of provider knowledge and confidence to transfer SI skills to clients [6, 7]. Qualitative evidence from Malawi also shows training clients on SI may take providers longer than administering PA options, due to the addition of the SI demonstration, SI training and practice sessions on top of the standard method-specific counselling on advantages/disadvantages and side effects. According to SI clients in one study in Malawi, SI training took approximately 20 minutes, while providers reported 27 minutes on average (10–60 minutes range) [4]. Exactly *how* the SI option is introduced by providers can also influence client confidence to take up this option–for example, a recent study in Malawi found that standardizing evidence-based messaging on SI (including specific upfront reassurances about the common SI concerns) was associated with higher SI uptake [8].

A recent study in Uganda also found that SI training quality may affect adolescents’ confidence to SI independently [9], while mixed-methods research conducted by the Delivering Innovation through Self-Care (DISC) program in Nigeria and Uganda has identified that providers can ‘gatekeep’ the SI option (i.e. introduce barriers to information and support to SI for some clients) based on their own biases or lack of confidence with the product [10]. Researchers working on DISC project also found missed opportunities to provide clients with information on DMPA-SC in quantitative client exit interviews, even among clients expressing interest in learning about DMPA-SC SI. For example only 68.8% of clients in Uganda who were interested in learning about DMPA-SC SI were actually informed about the option by a provider [11]. A study in Zambia has also noted that in the context of a new product introduction, such as DMPA-SC, some providers may feel hesitant about a new method and withhold information about it, while other providers may believe the new method is ‘better’ than other methods and place undue emphasis on it during counselling [12].

This growing evidence base in Malawi and other contexts where DMPA-SC is being rolled out raises the hypothesis that provider-related factors, and specifically variation in the quality of counselling/training on SI provided to clients, may explain at least some of the discrepancy between the high preference rates for SI during the pilot compared to comparatively lower actual rates of SI uptake at national scale in Malawi. This study was designed to include a specific objective to investigate best practices and potential gaps in providers’ approaches to integrating the SI option into their contraceptive counselling/training for injectable users in Malawi. Other specific objectives of the study included aspects such as understanding perspectives of adolescents with unmet need for contraception on DMPA-SC SI, the results of which are published elsewhere [13].

## Materials and methods

### Ethics statement

The study was conducted in close partnership with the Reproductive Health Directorate, MOH Malawi, and ethical approval to conduct this research was granted by the National Health Science Research Committee (NHSRC)–an independent international review board in Malawi with Federal-Wide Assurance (IRB00003905, FWA00005976). All study participants were asked to provide voluntary written informed consent to participate in this study prior to interview and were compensated for their participation in line with NHSRC policy. Additional information regarding the ethical, cultural, and scientific considerations specific to inclusivity in global research is included in the Supporting Information Inclusivity in Global Research Questionnaire (S2 Text).

### Methods, sampling and data collection

MOH Malawi and Clinton Health Access Initiative (CHAI) collaborated in 2021 to conduct this cross-sectional qualitative study. Specific outcomes of interest included: provider perspectives on enablers and barriers of integrating DMPA-SC intro their contraceptive counselling approach; client recall of, and perspectives on, the information received about DMPA-SC from providers during counselling sessions; client perspectives on the quality of training and support provided during SI training; as well as provider and client perspectives on the length of time counselling/training on DMPA-SC takes.

Study sites were six randomly-sampled public sector facilities in three districts (Nkhotakota, Mzimba South, Zomba)–one district from each of Malawi’s three regions. Districts and facilities were sampled randomly to minimize selection bias, however only public facilities that provided 10 or more DMPA-SC services per month were included in the sampling frame, to ensure enough injectable clients could be sampled. At each site, providers and injectable clients were purposively sampled according to inclusion criteria (for providers, this meant having been trained in DMPA-SC; for clients, this meant having used either a self-injected or provider-administered injectable contraceptive in the last nine months).

Data was collected using semi-structured in-depth interview guides. Several participatory activities were integrated to elicit detail about the provider-client counselling interaction. Firstly, providers were asked to role-play injectable contraceptive counselling and SI training with the data collectors acting as ‘clients’ interested in injectables. Providers were reassured that this exercise was purely to understand variations in approaches between providers, and that their responses would be anonymized, to try and minimize possible Hawthorne effect.

All data was collected in October 2021. Data for this study was collected during a period of inconsistent DMPA-SC stock in Malawi and other countries, due to global disruption in supply. Cognizant that method stockouts may affect how providers counsel on these methods [14] and may impact quality of contraceptive services [15], research assistants were trained to probe during role plays on whether and how providers’ self-reported counselling approaches may vary in cases of DMPA-SC stockouts.

Secondly, recognizing that some clients may not always critically appraise the quality of their care when they are unaware of the standards expected [16], injectable clients in this study were shown one of two videos to establish a standard against which they could assess the quality of the injectables counselling and SI training received:

1. Video 1 showed two actors demonstrating a best practice injectables counselling session between provider and client (S1 Text contains the script for the video). This video was created for the study and approved by the MOH for use for study purposes. It was shown to PA injectable clients who had chosen not to take up the SI option to help them critically compare with their own experience of counselling on injectables. It did not cover the SI training steps in detail, as most PA clients would not have proceeded with SI training after individual counselling. PA clients were interviewed on the same day that they received counselling, to minimize the chance of recall bias influencing results.
2. Video 2 was developed by PATH International [17] and explains the critical SI information and SI steps that a self-injecting client needs to know. This animated video was shown to current/recent SI clients to help them identify any gaps in their knowledge/recall. SI clients were often interviewed weeks or even months after receiving their original counselling on DMPA-SC, introducing the chance of recall bias in their recollections of individual counselling messages originally received about elements such as side effects and safe disposal. However, it was determined that they should still be able to recall the key SI steps, as successful independent self-injection is dependent on recall of these steps.

As Nkhotakota and Zomba are predominantly Chichewa-speaking districts, while Mzimba South is predominantly Tumbuka-speaking, all study tools and videos were translated or dubbed into both languages for study purposes.

The final sample size was based on theoretical saturation, which refers to the point at which no new ideas arise from data from a sample diverse in relevant characteristics and experiences [18]. Clear variation in themes emerging from the two DMPA client populations (PA and SI) during early data collection led to the decision to slightly expand sample sizes in those two groups to ensure saturation could be reached. No identifying information about participants was collected other than broad descriptors relating to basic demographics or cadre. Quantitative descriptors were collected on paper by the study team and manually entered into Excel for descriptive analysis. Qualitative data was collected by trained research assistants, audio-recorded, transcribed verbatim in Chichewa or Tumbuka and then translated into English for analysis.

### Analysis

Data was coded deductively by three qualitative researchers using Dedoose software. To ensure alignment between coders, coding was conducted in three phases. Firstly, 20% (n = 8) client transcripts and 25% (n = 6) of provider transcripts were coded independently by all three members of the analysis team, then coded versions merged for comparison and discussion to generate a codebook. Then, a further 20% (n = 8) of the sample for client IDIs and 25% (n = 6) of the sample for provider IDIs were double-coded (i.e. coded by one member of the team and then checked by another) to ensure consistency and comprehensiveness of code application. Finally, and only once good agreement in coding between the three members of the team had been established, 60% (n = 24) of the sample of client IDIs and 50% (n = 12) of the sample for provider IDIs were single coded. Once data was fully coded, code reports (S1 Data contains code reports relevant to counselling/training) were exported from Dedoose for thematic analysis.

**Fig 1:**
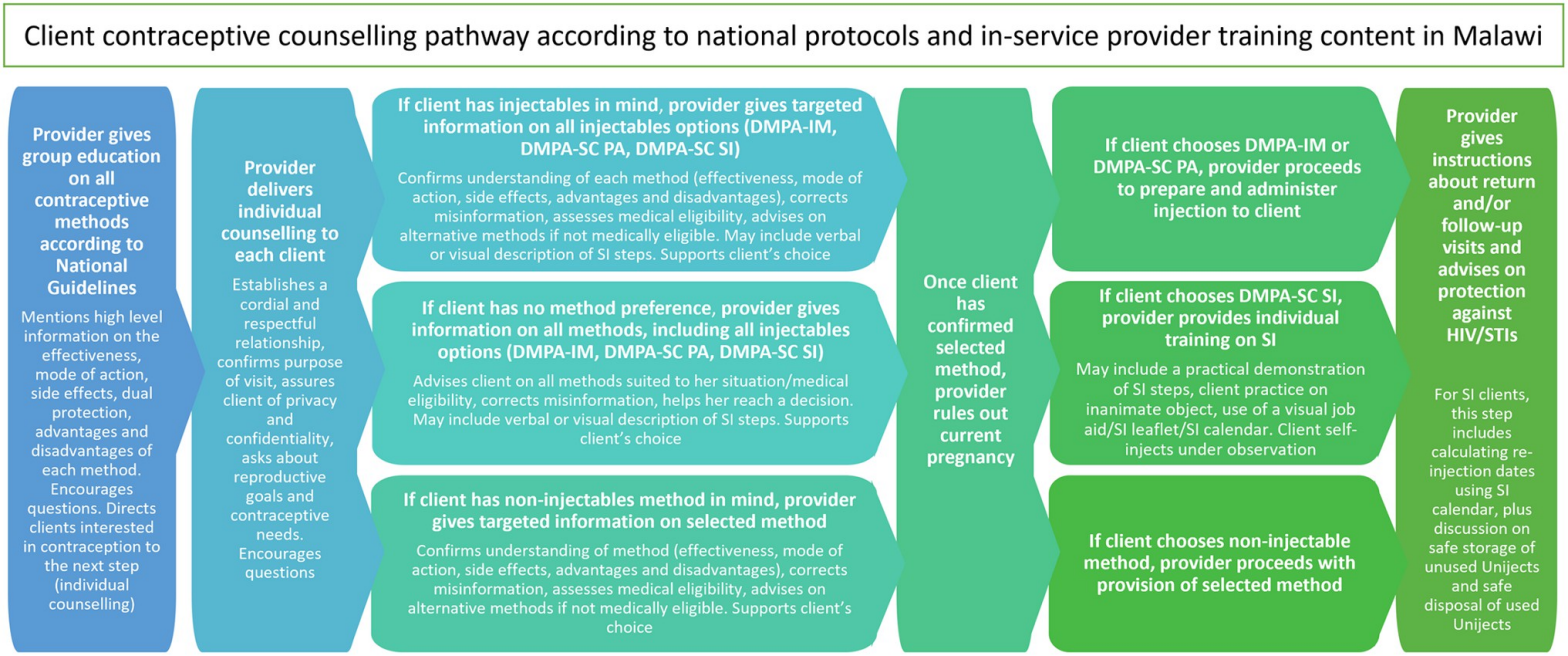
Client counselling pathways according to national protocols and provider in-service training content on DMPA-SC in Malawi.

For some themes (for example, the counselling messages covered during provider role-plays), a ‘framework approach’ was used, charting the coded data into a matrix to allow comparability across in-depth interviews and against the recommended client counselling pathways in national protocols and in-service training content on DMPA-SC provided by MOH (Fig 1). Where relevant, sub-group analyses were conducted by splitting code reports up between provider-administered and self-injection client groups or between health surveillance assistants (HSAs) and facility-based provider groups, to note any variation in themes or opinions between the sub-groups.

## Results

The final sample of 24 providers included 18 HSAs and 6 facility-based providers, most of whom reported receiving their DMPA-SC training a year or more ago. The final sample of 40 clients included 15 clients who had self-injected in the last nine months and 25 clients who had received a provider-administered injectable (DMPA-IM or DMPA-SC) in the last nine months. The average age of clients was 27 years, the majority were married and had 1–2 children. There were no substantial differences in demographic profile between PA and SI clients. All (100%) of the SI clients and most (80%) of the PA clients had heard of SI before the interview. Full details of the provider and client sample characteristics are outlined in Table 1.

**Table 1:** Participant characteristics.

**Provider sample (N = 24)**	**Facility-based providers**	**Health Surveillance Assistants (HSAs)**
**Time since trained**		
**Less than a year ago**	2 (33%)	4 (22%)
**A year or more ago**	3 (50%)	11 (61%)
**Data missing**	1 (17%)	3 (17%)
**Total**	6 (100%)	18 (100%)
**Client sample (N = 40)**	**DMPA-IM / DMPA-SC provider-administered clients**	**DMPA-SC self-injection clients**
**Age**		
**18–19 years old**	6 (24%)	4 (27%)
**20–29 years old**	9 (36%)	5 (33%)
**30–39 years old**	8 (32%)	3 (20%)
**40–49 years old**	1 (4%)	2 (13%)
**Data missing**	1 (4%)	1 (7%)
**Marital status**		
**Never married**	3 (12%)	1 (7%)
**Married**	18 (72%)	12 (80%)
**Divorced/separated/widowed**	3 (12%)	1 (7%)
**Data missing**	1 (4%)	1 (7%)
**Children**		
**No children**	1 (4%)	0 (0%)
**1–2 children**	16 (64%)	9 (60%)
**3–4 children**	6 (24%)	5 (33%)
**Data missing**	2 (8%)	1 (7%)
**Method used in last 9 months**		
**DMPA-IM**	20 (80%)	N/A
**DMPA-SC provider-administered**	5 (20%)	N/A
**DMPA-SC self-injected**	N/A	15 (100%)
**Heard of SI before the in-depth interview**		
**Yes**	20 (80%)	15 (100%)
**No**	5 (20%)	0 (0%)
**Total**	25 (100%)	15 (100%)

### Integrating self-injection into health education

All providers reported they had integrated high-level information on DMPA-SC and the SI option into their health education run-through of all the contraceptive methods (which most providers reportedly conducted as either a group session during a structured clinic or, for some HSAs, one-to-one with individual clients during home-based visits). Most providers said they still mentioned DMPA-SC and the SI option at this health education stage, even if they did not currently have DMPA-SC in stock, but advised that clients may need to receive PA options in the meantime until DMPA-SC was available again.

Some providers mentioned incorporating testimony from satisfied SI clients at the group health education stage, to demonstrate to other clients that it was possible for clients like them to SI. These providers felt this addition of peer testimonials was a powerful tool to support client confidence to SI.

Many providers reported emphasizing the benefits of SI at the health education stage; particularly that SI could reduce visits to facilities, which they felt would be the most appealing feature to clients. This is despite national protocol indicating both advantages and disadvantages of each method should be covered at group health education stage (Fig 1). One provider explained that the decision to focus on benefits was to pique client interest in DMPA-SC, knowing that the details of the method would be covered later in individual counselling:

*“[In health education sessions]… we cannot focus on the bad side because she will not choose the method*, *but we should explain to her the advantages of the method*. *Then she understood [sic] and makes her choice*. *And then we train her on this method [later]*.*”*

–*HSA*, *Nkhotakota*

Echoing this practice, most PA injectable clients mentioned having first heard some high-level messages on SI during either a health education session and/or sometimes through other clients. For these clients, their level of recall was also typically limited to only the high-level benefits of SI, indicating that they had either not received or did not recall more in-depth information on disadvantages, mode of action, and side effects during health education nor through subsequent individual counselling on injectables:

*“Yes*, *they [providers] told us that the injection is good*, *the self-injection*. *[Interviewer*: *Alright*, *did they say anything about the side effects of this injection*? *…] No*, *they did not say anything on that*, *they just explained about the benefits…”*

*–married PA client*, *30–39 years*, *Zomba*

A small group of PA injectable clients reported little or no awareness of even the high-level messages about SI, indicating they may have either not received or did not recall these messages from even a group health education session. These clients were mainly long-term DMPA-IM users who also reported that they tended to skip or not pay attention during group health education sessions and only spend a short time with providers one-on-one at their re-injection visits. They often cited satisfaction with DMPA-IM and lack of motivation to switch methods as their reasons for not paying attention to group health education:

*“I can’t explain [about DMPA-SC] because I did not pay attention [during health education] … [Interviewer*: *Why didn’t you pay attention*?*] It’s because I don’t want to be convinced with a new method… I did not pay attention [be]cause the one [contraceptive method] I am using works well on me*, *so I don’t want to hear other methods*.*”*

*–married PA client*, *30–39 years*, *Mzimba South*

### Integrating self-injection into individual method-specific counselling

If clients expressed interest in injectables after health education, providers then reported offering them in-depth individual counselling where they asked questions about contraceptive history, side effects and checked for contraindications, as well as presenting detailed information on all injectables options and exploring the client’s interest in the SI or PA options for mode of administration. This self-reported provider practice aligns with expected national protocol (Fig 1).

Most providers stressed the importance of having this one-to-one space with clients to tailor counselling to their needs. However, in a deviation from national protocol, a handful of providers reported grouping clients who expressed interest in a particular method to avoid repeating themselves and to save time–this practice included but did not seem to be exclusive to counselling on injectables. During stockouts of DMPA-SC, providers ranged in their approach with clients interested in SI at this stage: from encouraging clients to temporarily use other methods, to still proceeding with SI counselling/training but limiting clients’ opportunities to practice and/or their number of take-home doses, depending on stock availability.

During counselling role-plays, most providers interviewed demonstrated comprehensive counselling on DMPA-SC messages that aligned with national protocols and in-service training content, including proactively advising on re-injection dates, safe storage and disposal of the Unijects. Most providers were able to explain the benefits (for example, pregnancy prevention, reduced visits to facilities, discretion, etc.) and disadvantages/potential side effects (for example, menstrual changes, weight change, headaches, potential delayed return of fertility, etc.) of DMPA-SC, relative to other methods.

However, some messages from the national in-service training content that providers often missed during role-plays included advice on what clients should do:

- if they missed their re-injection window
- if they experienced any irritation or dimpling at the injection site
- to return unused units if they decided not to use them

In role-plays with research assistants, providers also shared their improvised techniques for familiarizing the concept of SI with clients early on. For example, some mentioned making comparisons between DMPA-SC and other needle-based concepts clients were familiar with–usually DMPA-IM, but also sometimes things like vaccines, malaria test kits, or insulin injections:

*“…we also tell them that the needle is small comparing with Depo Provera [DMPA-IM]*. *When you show the… the two needles*, *they say this is long and this small and easy just like malaria test kit*. *When we counsel her*, *they understand although they might still be afraid*.*”*

*–HSA*, *Nkhotakota*

Many providers noted the effectiveness of including elements that are typically associated with hands-on SI training (the next step for interested clients) into the individual counselling session early on–for example they had noticed that including a verbal/gestural description or visual of the SI steps into the information provided on DMPA-SC and/or showing clients the Uniject that this stage of the counselling was effective at alleviating concerns about the size of the needle or complexity of the SI process.

Some PA injectable clients were positive about the quality of individual counselling on injectables they received, even after comparing it to the best practice counselling session video (S1 Text). However, several others felt their own experience of counselling was less detailed than the session in the video:

*“[Interviewer*: *Ah why have you not tried it [self-injection]*?*] because I was not trained properly*, *I did not receive the proper training … but they also did not explain that this is how you perform self-injection very well …they also did not talk about the consequences [side effects]”*

*–PA client*, *missing age and marital status*, *Zomba*

For example, some PA clients picked up on topics covered in the best practice video that they felt had been missing (or not recalled) from their counselling, namely exactly how and where to self-inject, while others noted that they had not seen a visual aid/calendar that might have helped them remember the steps:

*“The provider said we have three places where we can self-inject but*, *in the video*, *they have said we have only two places where we can self-inject*.*”*

*–married PA client*, *30–39 years*, *Mzimba South*

*“…we were not given the calendar… it was a verbal calendar explaining that after 90 days you should do it…*. *But here [in the video] there is a calendar [with the job aid showing the SI steps] from washing hands to self-injecting*.*”*

*–married PA client*, *40–49 years*, *Zomba*,

From what they recalled from their original counselling on SI, most SI clients said the messages aligned with the key information covered in the PATH video. [17] Only a few SI clients reflected that the topic of possible side effects of DMPA-SC had been covered in more depth in the video than they recalled from their original counselling. A few others said they recalled receiving different disposal advice (for example, to throw used Unijects in a pit latrine) to the recommended advice mentioned in the video (i.e. to keep in a puncture-proof container and return to the facility):

*“The training I received from my provider is different [from the video] because [in the video] they have said that some get fat*, *some menstruate*, *some [have] stomach ache and some headache*. *They did not explain this*.*”*

*–married SI client*, *20–29 years*, *Nkhotakota*

*“[Interviewer*: *Anything else that you were not told that you have heard in the video*?*] … Oooh yes*, *we were told to throw the used injection in the toilets [pit latrine] whilst our friend [the client from the video] was told to through it in a Frozy bottle and bring it back to the facility*.*”*

*–married SI client*, *20–29 years*, *Mzimba South*

However, given most SI clients were interviewed weeks or months after their original counselling, these examples may be subject to recall bias.

All SI clients interviewed reported feeling reassured enough in their individual counselling to proceed to being trained in SI. By contrast, only a few PA clients reported that they had decided to proceed with training in SI after receiving counselling–the majority reported that they still had too many concerns about pain or doubt in their own ability to SI to even proceed with SI training.

### Conducting self-injection training

After counselling on DMPA-SC, answering client questions, and establishing no contraindications, providers reported offering clients interested in DMPA-SC the opportunity to be trained on SI. Several providers commented that, if they had spent enough time addressing concerns during counselling (and especially if they had included a visualization or demonstration element of the Uniject and/or SI steps early) most clients would choose to be trained in SI, while only a few clients would request PA instead:

*“…if you do the counselling very well*, *they do not have any concerns as long as they have understood every procedure on how they can self-inject and also know the next date to self-inject in doing so they will not forget*.*”*

*–Facility-based provider*, *Mzimba South*

If they had not done so already, most providers reported first conducting some sort of visual demonstration of the Uniject and SI steps to kick off SI training. This typically involved using a visual aid (for example, posters, job aids or the visuals on the back of SI calendars) and/or gestures and demonstrations with a real Uniject–where DMPA-SC stock availability allowed. Many providers emphasized the importance of clients being able to visualize the SI steps to reassure them of the simplicity of it:

*“…most people easily understand when they are able to see the things that they are being trained in*, *how it is operating…”*

*–HSA*, *Zomba*

Providers were generally very positive about the DMPA-SC SI calendars used in Malawi (these include a visual reminder of the SI steps on the back), which they felt served multiple purposes– 1) acting as a visual aid during counselling/training, 2) serving as a reminder of the SI steps for clients at home, and 3) helping clients keep track of their re-injection dates:

*“… calendars they are so helpful… It contains details on the steps on how self-injection should be done and also it has some pictures that help the clients to see the process*. *We also use the pictures when counselling clients … It helps especially when they are alone*, *they are able to refer to the instruction sheet*.*”*

*–HSA*, *Nkhotakota*

During role plays, most providers showed good knowledge of the critical SI steps from national protocol/provider in-service training content. The critical SI steps most commonly mentioned by providers during the role plays included:

- the importance of shaking and activating the Uniject
- pinching the skin at the injection site
- injecting at a downward angle
- not rubbing the injection site after injecting
- disposing of the product safely in a closed container and returning it to a heath official

A few of the SI steps that were less commonly mentioned by providers included:

- Handwashing before self-injecting
- Checking expiration dates before self-injecting
- Removing the needle cap and not replacing it
- Pressing the reservoir for 5–7 seconds
- The specific sequencing of removing the needle and releasing the ‘pinched’ skin
- Calculating and noting down the date for the next injection

From the client side, there were understandable differences between the level of recall of critical SI steps between PA and SI clients. Most PA clients had not proceeded to SI training and therefore recalled only high-level information (typically only shaking and activating the Uniject and pinching the skin before injecting on the thigh or stomach) from their individual counselling. It was not always clear if this lower recall of the SI steps was due to providers omitting SI steps during counselling, or due to lack of PA client interest or absorption of the information at this stage.

By contrast, current/recent SI clients understandably showed the most detailed spontaneous recall for most of the critical SI steps, typically able to walk through most or all the steps from memory:

*“This is how we hold Sayana [showing the interviewer using hands] then we shake it*, *after shaking it we press its neck and then the needle is pushed inside then we hold the place where we want to inject ourselves and then we inject the needle*. *The moment we realize that the needle is inside the skin then we press the medicine until all is finished then we start pulling out the needle little by little*.*”*

*–divorced/widowed SI client*, *20–29 years*, *Nkhotakota*

Most SI clients reported that the SI steps they recalled aligned mostly or completely with those covered in the PATH video: [17]

“… *everything that we were trained [on] is in the video… After I watched the video and the training that I received from the providers… I can see that [I] am able to do everything in order and there is no problem and the providers trained us well*”

*–married SI client*, *30–39 years*, *Zomba*

After the visual demonstration, most providers reportedly then encouraged clients to practice with a Uniject. While national protocol/provider in-service training content suggests clients should practice first on inanimate objects, such as a condom filled with sugar, only a few providers talked about doing this in practice (and, if they did, they typically used cheaper materials than sugar, such as condoms filled with sand, or oranges or tomatoes). Most providers in this study instead reported encouraging clients to ‘practice’ SI directly for the first time on their thighs or stomachs, under supervision. The skipping of the practice on inanimate objects was said to be due to concerns about ‘wasting’ scarce DMPA-SC stock; provider time constraints; concerns about the cost of sugar; or because providers felt clients were not ‘convinced’ by practicing on inanimate materials:

*“…most of the times the women are reluctant [they think] that this [condom filled with sugar] is not a real thing*, *and they want a demonstration on the actual body… so we don’t rely on it too much…”*

*–Facility-based provider*, *Zomba*

Providers often noted that practice was the most challenging part of SI training, requiring observation and repeated feedback to ensure clients self-injected correctly. Some providers seemed to treat this step as something of an ‘exam’ for clients, using the terms ‘pass’ and ‘fail’ to denote if they were happy or unhappy with the client’s attempt:

*“…we compare … how they are injecting themselves and see where they are doing wrong*, *and we help them to improve on that*. *And the ones who have passed [successfully self-injected]*, *we congratulate them and tell them to continue at home*. *But for the ones who have failed*, *we tell them*.*”*

*–HSA*, *Nkhotakota*

A few providers talked about revisiting demonstrations and training on SI over time (for example, over the course of several PA re-injection visits) to build the confidence of clients who had previously ‘failed’ practice sessions.

From the client perspective, SI clients were slightly more likely than their PA counterparts who had received SI training to mention having seen a visual demonstration of SI steps during SI training, while some SI clients said they had seen another client self-inject before being encouraged to ‘practice’ on themselves. By contrast, very few PA clients who proceeded to SI training mentioned seeing a visual demonstration of any kind, nor seeing other clients self-inject first before being asked to ‘practice’ on themselves. Reflecting the provider tendency to skip the practice on inanimate objects, very few clients in either the SI or PA group mentioned having practiced in this way–most said that their first ‘practice’ had been on their own thighs under provider observation:

*“We were taught how to inject ourselves*; *we were asked if [we] would manage and I agreed and attempted to inject myself while the HSA was watching*, *she confirmed that I had done it well and could manage to do it on my own*.*”*

–married *SI client*, *40–49 years*, *Zomba*

*“[Interviewer*: *Did they give you a chance to practice] Yes*, *they did [Interviewer*: *Did you self-inject*? *What did you use to practice*?*] They gave us a chance like me I practiced on my body”*

*–married PA client*, *under 20*, *Mzimba South*

In general, SI clients reflected positively on their SI training and credited the visualizations, demonstrations and practice under observation with helping them build confidence and overcome initial fears:

*“I had fears at first*. *But the fears went off because we were self-injecting under direct observation by the provider*, *they were instructing us on how to do it [Interviewer*: *What did you [do to] deal with the fears apart from providers being there for you*?*] It’s because of the counselling they gave us*. *When we started the procedure*, *everything was going as they had taught us*, *so this relieved us our fears*.*”*

*–married SI client*, *20–29 years*, *Mzimba South*

A few SI clients described how providers helped them overcome their fears and build their confidence over repeated PA visits, if they had initially been too afraid to try SI:

*“Like for me I asked them to inject me because I was not confident*. *But I was told it [DMPA-SC] was meant for self-injection*. *During the second visit*, *they counselled us again and this time I understood and managed to self-inject”*

*–married SI client*, *30–39 years*, *Mzimba South*

When asked why they had not taken up SI after receiving SI training, the few PA clients who had received the SI training tended to blame themselves rather than the quality of their training–despite also reporting their training as ‘rushed’. For example, they were more likely to talk about struggling to overcome initial fears; lacking confidence in their own ability; or mirroring the providers’ language about having ‘failed’ their practice:

*“I wanted to find out if I will be able to self-inject*. *If I will do it*, *I will be using that one*. *But I failed*. *[Interviewer*: *You failed*?… *How exactly did you fail*?*] I failed to open [activate the Uniject]*. *They say that for it to be opened [activated]*, *it has to make a sound and I failed to do that*. *I was like ‘I will end up destroying this thing’*.*”*

*–married PA client*, *30–39 years*, *Nkhotakota*

A few PA clients also reported feeling pressure or judgment from providers to take up SI:

*“[Interviewer*: *What was the nurse saying*?*] …[she] was saying*, *‘Why do you still want us to inject you*? *Why are you not getting the self-injecting one*?… *Why are you not injecting yourself*?*’ … [Interviewer*: *So what did you say*?*] I said I have never taken the self-injecting type before*.*”*

*–married PA client*, *20–29 years*, *Nkhotakota*

### Length and feasibility of comprehensive counselling and training

Most providers in this study reported that it takes between 15–20 minutes to counsel and then train a client on DMPA-SC SI. A few others said it could take more than 20 minutes and up to 45 minutes with some clients. Finding this additional time during busy days was not always easy for providers:

*“To explain to the client [about DMPA-SC SI]*, *you need to have uninterrupted time and also sometimes nurses are busy … you need to sit down and start explain[ing] all the procedures up until you are convinced that she can do it*. *So it’s not that difficult but you just need time to do it”*

–*Facility-based provider*, *Mzimba South*

A few providers mentioned other factors that could influence the length of counselling/training. For example, many providers felt that counselling/training a client who had previously used an injectable would be quicker, as she had existing knowledge to draw on, while clients with less knowledge of contraception may be more difficult to counsel and train, as they would have more questions to address:

*“Having previous experience [with injectables] does help*, *for instance*, *during counselling if we have clients who started the method some time [ago]… you will hear them commenting that they know the stuff … So … this makes the work simple because you know the counselling process will be easy and does not take a lot of time*. *Whilst if you have new clients it takes long to counsel them”*

*–HSA*, *Nkhotakota*

Providers generally did not mention educational status of the client influencing counselling time, but they did mention age. Most providers felt that adolescents (under 20) required more time to counsel, just because they were more likely to be starting from a low baseline of information about contraception:

“*Compared to an older woman*, *because older women already know the things*, *and maybe they have already heard the things from somebody else*, *but mostly the adolescents don’t know most of the information relating to family planning…”*

–*HSA*, *Zomba*

However, a few providers said that it was actually quicker to counsel adolescents because they would pick up new ideas quickly:

*“The understanding of adults is a bit complex… Hence it becomes difficult for them to grasp the information easily as such you have to say it again and again*. *It’s different from adolescents when you train them*, *they capture the info easily…”*

*–Facility-based provider*, *Nkhotakota*

A few providers said adolescents’ anxiety about being seen at the facility was a factor in them wanting to avoid group health education sessions or in them asking for faster counselling:

*“Most youth mostly don’t have time*. *It happens that sometimes the time we’re giving the talk*, *they’re rushing to do their own things and they sometimes try to avoid people … they think “If so and so sees me they will report them to their homes*.*”*

–*Facility-based provider*, *Zomba*

Several providers described their role in counselling/training on SI becoming quicker and easier over time, as their own experience increased and awareness of DMPA-SC at community-level grew:

“…*initially I think for the first three months … we would spend almost 45 minutes… 40 to 45 minutes… but after the first three months we spend 15 minutes*”

–*Facility-based provider*, *Zomba*

Despite the time pressures, most providers took their responsibility for counselling and training clients in SI very seriously and felt that investing in longer counselling/training facilitated comprehensive understanding, uptake of SI, and reduced likelihood of SI clients encountering problems later. Many providers also clearly articulated that they believed investing that time would increase SI uptake and reduce their workload later, in terms of reducing visits by repeat PA clients:

*“If the provider has counselled the clients so well in details*, *it becomes so easy for the clients when it comes for them to try to self-inject*. *But in cases where the provider does counsel the clients in a hurry without checking whether they are clear with the process*, *[then] it becomes a problem for them when doing the trial injection [practice]*.*”*

–*HSA*, *Nkhotakota*

*“[once trained in SI] … they will just come and collect*, *and they will not show up again for an entire year*. *For them [not] to come back*, *it means [in] the gap they create we can be serving other clients…”*

*- HSA*, *Zomba*

However, a minority of providers admitted that time pressures drove them to take shortcuts in counselling/training, most commonly turning to small-group method-specific counselling and small-group SI training approaches over individual approaches:

*“During busy days*, *we do shortcuts (laughs)… We wait for at least the women to be 10 or 8 or 7*, *where you feel that this a good number*. *You just teach them in one kick [one group session] and then give them the methods*. *But for you to start one-on-one [counselling/training] and [it] being a busy day*, *it does not work*.*”*

–*HSA*, *Nkhotakota*

On the clients’ side, SI clients were also more likely than their PA counterparts to say their training had taken a long time, even up to an hour, but typically they were satisfied with this length, as they felt it aided their understanding:

*“[Interviewer*: *… Did you feel the provider spent enough time training you*?*] Yes*. *we spent a lot of time at that place*. *[Interviewer*: *you understood what they trained you*?*] I understood everything they trained me about*.*”*

*–married SI client*, *20–29 years*, *Nkhotakota*

Meanwhile most PA clients who had not taken up SI felt that the counselling and training time they received was too short to fully help them understand:

*“[Interviewer*: *do you feel you received enough counselling [on DMPA-SC] from the provider*?*] no she was rushing she seemed to have other things to do”*

*–married PA client*, *20–29 years*, *Mzimba South*

Both PA and SI clients who reported having received at least some of their counselling and/or SI training in a small-group context did not explicitly note whether the group nature of this experience affected the quality of their counselling/training nor whether it influenced their decision to take up or not take up SI.

## Discussion

All providers in this study reported successfully integrating DMPA-SC and the SI option into at least the first stages of their health education and individual contraceptive counselling approach. During health education sessions, providers reported that they tended to focus only on the key benefits of self-injection, to spark client interest in the method, and then follow that up with more in-depth information during individual counselling (or sometimes in small-group method specific counselling). While national protocol states the need to cover both advantages and disadvantages of each method at a high-level in group health education, providers seemed to feel that clients’ initial fears at the idea of SI might put them off trying the method and tried to focus on the advantages of the SI option to engage clients in further conversations where they could provide more personalized reassurance than they could in a group health education context.

Most injectable clients interviewed had at least some high-level awareness of the SI option, indicating that it is routinely being mentioned at least in the group health education sessions. However, patchy recall of details about SI among most PA clients points to a lack of provision and/or absorption of detailed information about SI during individual counselling. In addition, the fact that a few returning DMPA-IM clients in this study had very limited awareness of the SI option suggests that in these cases providers may sometimes be missing an opportunity to confirm client satisfaction with their method and explore alternative injectable options for returning clients. This ‘status quo bias’ of defaulting to repeating provision of a client’s previous method is something that has been found in other studies on contraceptive counselling in Malawi [14], although in this study it seemed to be limited to only long-standing DMPA-IM users.

During role-plays of counselling and SI training, providers generally displayed knowledge and counselling practice on DMPA-SC that aligned with the information from national protocols and provider in-service training content, with a few exceptions where key messages/critical SI steps were missed. Future studies could investigate whether the number of ‘critical steps’ for SI could be reduced without compromising quality of care, and whether this could improve memory retention of the critical SI steps among providers and clients alike. In the meantime, post-training support visits for DMPA-SC trained providers should include emphasis on the commonly missed steps/messages outlined in this study to refresh providers’ memories. Use of standardized evidence-based messaging on laminated cards–such as the intervention by Burke at al. [8]—could be considered to ensure key messages are covered during counselling to ensure clients are fully informed.

Over time, many providers in this study had developed and innovated their own counselling/training techniques to reassure clients sufficiently to take up the SI option. For example, providers relied on 1) comparisons between DMPA-IM and DMPA-SC (and other needle-related concepts) to familiarize the product, 2) showing clients the Uniject early on in counselling to reassure them about needle size and simplicity, 3) visualization of the SI steps (either using visual aids or a demonstration with gestures) early on during individual counselling as well as during SI training, 4) hearing from other satisfied self-injectors (although providers varied in when and how they incorporated these peer testimonials), 5) giving clients the opportunity to practice SI, sometimes on demonstration materials but more commonly on themselves under observation, and 6) repeat SI trainings for the minority of clients who needed more time to build confidence.

For their part, clients who successfully took up the SI option typically credited this decision to receiving lengthy and in-depth counselling/training, which was supportive enough to build their confidence. SI clients flagged the following techniques as particularly effective at increasing their confidence: the provider taking their time during counselling/training; seeing a demonstration of the SI steps before practicing; and hearing from other SI clients. Repeated counselling/training was also noted to be necessary for a minority of clients wanting to try SI, to give them time to get used to the idea and build up their confidence.

While providers themselves demonstrated counselling in-depth on potential side effects and safe disposal of used Unijects during role plays, a few SI clients could not recall having been told about these elements, when comparing their retained knowledge to the video. It’s not clear whether this finding indicates gaps in provider counselling on this topic or recall bias on the part of SI clients, many of whom were interviewed long after their original DMPA-SC counselling. However, the request from SI clients to cover side-effects comprehensively during counselling echoes evidence from other contexts that clients value receiving full and clear information, especially about potential side effects, during counselling [19].

Many PA clients who did not take up the SI option tended to report being put off the idea of SI from the start due to fear and doubt in their own ability, and not feeling sufficiently reassured about the messages they may have heard in a group health education session or individual counselling to even progress to SI training. This aligns with evidence from other contexts suggesting fear of SI is a strong barrier for many clients at both the decision and initiation stages [10], and, combined with PA clients tending to report shorter/more rushed counselling, suggests that there could be opportunities to integrate best practices to make SI counselling more reassuring.

For those few PA clients who were sufficiently interested to proceed to SI training, they were slightly less likely than SI clients to recall receiving key elements such as visualizations and demonstrations, before being asked to ‘practice’ on themselves. It’s not always clear from the narratives in the transcripts whether these features were missed from training due to providers rushing; missed due to providers sensing lack of interest in SI from PA clients; or whether they were included by providers but simply not recalled by PA clients (though the latter is less likely, as PA client interviews were conducted soon after counselling/training). There may well be valid reasons providers chose to skip steps during training, for example because they felt certain steps did not work (for example, practice on inanimate objects), due to stock constraints, or because clients were not interested, or their reassurances were not working.

However, what was clear was that even in the few cases of PA clients who had received SI training, they tended to blame themselves rather than the quality of their training, particularly mirroring provider language about having ‘failed’—a negative framing likely to have reinforced their fear or lack of confidence. Instead of using narratives implying clients ‘passing’ or ‘failing’, providers should be encouraged to help unpack client fears and support clients who still want to self-inject to build confidence over time, as some of their colleagues are already doing. In some cases, multiple training sessions may be needed, something that was also found in the DISC program research [10].

Providers in this study said that it typically took them around 15–20 minutes (and up to 45 minutes) to counsel and train clients in SI. This is slightly shorter compared to estimates by providers in previous qualitative studies in Malawi [4], and may reflect providers finding ways to streamline their counselling/training approach over time as they learned the techniques that best reassured clients. Most providers reported investing time to train clients in-depth on SI, despite time-pressure and competing priorities, because they felt it was important to ensure clients fully understood the information and because they saw the benefit of reduced workload once clients were satisfied and established with using SI. This provider belief that DMPA-SC SI could reduce workloads and save them (and clients) time has been found in previous studies in Malawi [4].

However, by contrast, some providers in this study reported time pressures leading them to take counselling/training shortcuts; namely, counselling and training clients in SI in small groups, and sometimes skipping recommended steps such as visualizations and demonstrations. The practice of conducting group method-specific counselling or SI training is not recommended according to national protocol/in-service training content in Malawi, however it is occurring in practice–and future research could explore the impact of this approach on confidentiality and quality of care. Evidence from other contexts indicates that clients have mixed opinions about small-group counselling/trainings. For example, a recent study with adolescent SI users in Uganda found that around half of the adolescents interviewed would have preferred individual counselling/training versus around a third reported a preference for group counselling/training due to the benefits of peer support and shared experience [9]. Research from the DISC program also found mixed client perspectives on the group training practice in Uganda and Nigeria, with some users emphasizing the need for discretion and confidentiality during training, while other clients felt that ‘peer training’ and other group-based learning about SI may be appealing [10]. In addition, while providers in this study were confident that hearing from satisfied SI clients during group health education, counselling or training had a positive impact on client confidence, peer testimonial approaches should be cognizant of the preference for confidentiality among many SI users, as the researchers from the DISC program note [10].

By contrast to other studies conducted on provider counselling in Malawi that indicate providers tend to only focus their counselling only on methods currently in stock [14], this study did not find any evidence of omission of DMPA-SC from counselling during periods of stock-outs. However, providers did report telling clients interested in SI to take up PA options temporarily in cases of DMPA-SC stock out, and stock scarcity was said to influence practice opportunities and the number of take-home doses offered to clients.

Interestingly, most providers and clients receiving SI training in this study reported skipping the practice of SI on inanimate objects, despite previous research in Malawi finding evidence of this practice being implemented [4]. While this was partly said to be driven by challenges with DMPA-SC stock at the time of the data collection and maintaining expensive demonstration materials (i.e. condoms filled with sugar), other providers reported skipping this step because they felt it was not useful to clients. On their side, clients tended not to differentiate between practice on inanimate objects or ‘practice’ on themselves under observation and did not explicitly express a preference for one option over the other. This finding is interesting given that research in Uganda suggests that repeated practice on a prosthetic before self-injecting for the first time is an important step for providers to assess client competence [20]. The impact of skipping this step on client competence and confidence should be explored in future research.

Overall, this study has highlighted the critical role that providers play in ensuring clients interested in SI are confident to do so. There are likely many factors explaining the difference between the high SI preference rates from early acceptability studies under pilot conditions in Malawi compared to the relatively lower SI uptake rates at national scale, however provider-side variation in counselling/training approaches may well play an important role. Overall, DMPA-SC and the SI option seem to be well-integrated into routine group health education and individual counselling approaches among providers in this study, and many providers had innovated their own approaches and techniques to support clients to confidently take up SI. However, time and resource pressures were also said to sometimes affect the ability of providers to invest in comprehensive, supportive, in-depth counselling and SI training, which may well have a knock-on effect on client confidence to find out more about, and ultimately take up, SI.

Limitations of the study include:

- The sites chosen for the study and the people recruited at these sites may not generate results generalizable to all the providers/HSAs and clientele at all public sector sites in Malawi. The districts and facilities were sampled randomly to minimize this bias. Results aligning with other similar qualitative studies conducted in Malawi suggest the findings of this study were not unduly affected.
- The perspectives of 15–17-years-old contraceptive users were not included in this study, as the high likelihood of their contraceptive use being covert, combined with the IRB requirement for parental/guardian consent for this population, made their inclusion high-risk for accidental disclosure of their contraceptive use to parents/guardians. It is probable that their perspectives on counselling/training experience differ from the perspectives of adult clients–as was found in a recent study in Uganda [9].
- Providers were asked to role-play counselling and SI training sessions with interviewers during the data collection. While these role-plays were likely affected by the knowledge of being observed (Hawthorne effect), they still allowed the interviewers to understand the general approach and content of provider counselling, as well as how it was reported to have varied between providers and under certain conditions (for example, during stock outs of DMPA-SC).
- As this study relied on either hypothetical (in the case of provider role plays) or retrospective recall of counselling (in the case of client interviews), it was not always possible to assess exactly how provider-client interactions causally affected the flow of counselling/training on DMPA-SC and SI. While providers and clients themselves reported causal associations between counselling/training length and content and client confidence or lack of confidence with SI, it was not possible from the data to confirm these as causal relationships—for example, whether insufficient information or skipping of key steps by providers was a response to low client interest in SI or whether it played a causal role in clients not taking up SI.

## Conclusion

Public sector providers in this study generally demonstrated knowledge and skills on DMPA-SC counselling in line with national protocol and provider in-service training content and shared their improvised examples of techniques for building client confidence with the SI option. However, time- and resource-pressures can lead some providers to deviate from recommended approaches–particularly in terms of grouping clients for SI training, and/or skipping visualizations, demonstrations, and the step of practicing on inanimate objects. SI clients tended to credit their decision to take up SI to receiving lengthy, comprehensive counselling/training, often inclusive of reassuring messages, visualizations, demonstrations and sometimes repeated trainings over time. PA clients tended to credit their lack of uptake of SI to fear and lack of confidence, often blaming themselves instead of the quality of their counselling/training–even while many felt their counselling/training had been rushed or incomplete.

Based on these findings, public providers in Malawi should continue to receive post-training follow up support focused on honing their counselling and training skills, addressing any gaps in their knowledge, re-emphasizing that investing in SI counselling/training may save them time in the long-run, and sharing best practices for building client confidence to SI, such as integration of visualizations, demonstrations, and peer testimonials–although balancing the latter with client privacy and confidentiality. Use of standardized messaging during counselling could be considered to ensure key messages are covered. Providers should be encouraged to avoid narratives of ‘passing’ or ‘failing’ when training clients, instead focusing on helping to unpack client fears and support clients who still want to SI to build confidence over time.

Future research could explore the impact of removing client practice on inanimate objects before proceeding to SI for the first time; the impact on quality of care and privacy when implementing SI counselling/training individually versus in small groups; how best to support clients to freely choose between all injectable contraceptive options in an informed manner; how best to leverage voluntary peer testimonials during health education, counselling or training while respecting client confidentiality; and explore opportunities to incorporate voluntary SI client testimonials into broader community sensitization activities.

## Supporting information

S1 Data Malawi DMPA-SC counselling code reports. (ZIP)

S1 Text Best practice counselling video script. (DOCX)

S2 Text Inclusivity in global research questionnaire. (DOCX)
