# Supplementary material for: Health care provider and client experiences of counselling on depot medroxyprogesterone acetate subcutaneous (DMPA-SC) for self-injection in Malawi
Source: PLOS Glob Public Health. 2023 Nov 30;3(11):e0002057. doi: 10.1371/journal.pgph.0002057 (PMC10688740; doi:10.1371/journal.pgph.0002057)
Supplement: S1 Text — (DOCX) [file pgph.0002057.s002.docx]

## S1 Supporting information

**Script from the best practices DMPA-SC contraceptive counselling video used as a visual prompt for clients during the study to help them assess quality of counselling received**

| **Scene description:**   - Nurse Mary is in an FP room of a health centre where Grace has come to get her 3-month contraceptive re-injection. - The room has some government women’s health posters on the walls. There is the MOH FP flip chart on the table - Nurse Mary is about 40 years old and is wearing a nurse’s uniform. She is a warm and friendly nurse who always maintains eye contact and attention on Grace - Grace is about 25 years old and already has 2 children | |
| --- | --- |
| **Scene** | **Narrative** |
| Grace knocks on the door of the family planning clinic room. Nurse Mary warmly invites her in while seated behind her desk and points her to a designated seat. Mary takes her seat, facing Nurse Mary. | Nurse Mary (with a friendly and pleasant tone):   - *Welcome back Grace, how are you today?*   Grace:   - *I am well thank you – Sorry I am a little late. I had to go first to the garden, as you know the onset of the rains is near.* |
| Nurse Mary and Grace take a seat on either side of the table  Nurse Mary leans forward with open and friendly body language | Nurse Mary (with a friendly and pleasant tone):   - *That is no problem, please have a seat.* - *How can I help you today?*   Grace:   - *I have come to get my 3 month contraceptive injection*   Nurse Mary:   - *OK. Great. I can help you with that. Can I take a look at your health passport please?* |
| Grace hands over her health passport to Nurse Mary.  Nurse Mary looks at Grace’s health passport book and then looks back up at Grace | Nurse Mary:   - *I see that you have been using the injectable for 6 months now – how have you been finding it?*   Grace:   - *I think it has been going ok. I still have some spotting and my last period was 2 months ago. Is that normal?*   Nurse Mary:   - *Yes – irregular and less frequent bleeding is quite common for women using the injectable and is not a health concern.* - *Are the bleeding changes causing disruption to your life? We can talk about other options if you would like.*   Grace:   - *No – I like the convenience of the injectable and am OK with less bleeding if I know it is not harmful to me.*   Nurse Mary:   - *As I mentioned – bleeding changes are common and are in no way harmful to your health. Your menses will return to normal when you stop using the injectable.* - *One thing to remember is that there can be a delay in being able to get pregnant after you stop using the injectable. Are you planning another child any time soon?*   Grace:   - *We want to wait for another 1 to 2 years before getting pregnant again.*   Nurse Mary:   - *Ok – then the injectable is still a good option for you. Would you like to continue with the injectable today?*   Grace:   - *Yes please.* |
| Nurse Mary picks up the self-injection counselling aid and gets out a demo Uniject device | Nurse Mary:   - *Have you heard about a new type of injectable that you can self-inject in the privacy of your own home?*   Grace:   - *I heard some women at the markets talking about it and I was curious.* |
| Nurse Mary picks up the Uniject device and shows it to Grace | Nurse Mary:   - *This is the new version of the injectable contraceptive that you could self-inject.* - *It is pre-filled with the same contraceptive drug as you have used before, but as you can see it has a small needle, which is why you could use it at home, if you wanted to.* - *What happens is I would train you how to use it and you would give yourself the first injection here, so we make sure you are comfortable and doing it correctly.* - *Then I would give you 3 injections to take home with you, as well as a calendar to help you remember when to re-inject. This means you wouldn’t need to come back until after a year – no need to worry about missing your appointment or coming late, during the rainy season for example. It can be very convenient for many women.*   Grace:   - *That does sound convenient for me. It is so hard to find time to come to the clinic every three months and find someone to look after the children…*   Nurse Mary:   - *An important thing to note is that you do need to store the unused injections in a safe space at home away from children, and once you have used them, they need to be placed in a puncture-proof container with a lid and stored in a safe location. At your convenience, you can bring the container to a facility or health worker so the devices can be disposed of safely. Does that sound OK?*   Grace:   - *Yes – I could store them at the top of the cupboard in our bedroom.*   Nurse Mary:   - *And would you be able to safely dispose of the used units in a close container and bring them to a health care worker or facility at your convenient time?*   Grace:   - *Yes, I think that would be fine.* |
| Nurse Mary puts down the device and counselling tool | Nurse Mary:   - *So what do you think? Is this self-injection something you would be interested in trying?*   Grace (says hesitantly):   - *I am interested but I just don’t think I can inject myself! What if I can’t do it? What if I forget the training?*   Nurse Mary:   - *All women are a little hesitant at first but most women I train find it very easy once they have had a chance to practice.* - *If you still feel uncomfortable after training and practicing today, I can simply do the injection for you. It is no problem.* - *If you decide to take the self-injection option today but in three months’ time you forget the training, you can also come back and we can practice again, or I can do the injection for you. I’m here to support you.* |
| Grace nods her head | Nurse Mary:   - *Would you like me to train you how to self-inject? It usually takes about 10 minutes.*   Grace:   - *Yes please – I would like to give it a go.* |

For more information about this video, please email the corresponding author [cporter@clintonhealthaccess.org](about:blank)
