# Supplementary material for: Health care provider and client experiences of counselling on depot medroxyprogesterone acetate subcutaneous (DMPA-SC) for self-injection in Malawi
Source: PLOS Glob Public Health. 2023 Nov 30;3(11):e0002057. doi: 10.1371/journal.pgph.0002057 (PMC10688740; doi:10.1371/journal.pgph.0002057)
Supplement: S2 Text — (DOCX) [file pgph.0002057.s003.docx]

Inclusivity in global research

PLOS’ policy on inclusivity in global research aims to improve transparency in the reporting of research performed outside of researchers’ own country or community and ensures that PLOS publications reporting global research adhere to high standards for research ethics and authorship. Authors of relevant research articles may be asked to complete the questionnaire below, which outlines ethical, cultural, and scientific considerations specific to inclusivity in global research. This questionnaire may be requested when researchers have travelled to a different country to conduct research, if research uses samples collected in another country, research with Indigenous populations or their lands, or if research is on cultural artefacts. Researchers travelling to another country solely to use laboratory equipment will not normally be required to complete the questionnaire. However, the questionnaire can be requested at the journal’s discretion for any submission – if you have been requested to complete this questionnaire by the PLOS journal you submitted to, please do so.

Please complete the questionnaire below and include this as a Supporting Information file with your manuscript. Note that if your paper is accepted for publication, this checklist will be published with your article in the supporting information files. Please ensure that you reference the checklist in the main body of your manuscript. We suggest adding a subsection ‘Inclusivity in global research’ to your Methods section and adding the following sentence: “Additional information regarding the ethical, cultural, and scientific considerations specific to inclusivity in global research is included in the Supporting Information (SX Checklist)”

The questions have been designed to be applicable to a wide range of study types, and there are subsections for both human subjects research and non-human subjects research. If any of the questions are not relevant to your research please mark them as “N/A” as appropriate.

**Ethical considerations, permits and authorship**

*This section is applicable to all research types.*

Provide details as to who granted permissions and/or consent for the study to take place in the Methods section of your manuscript. This should include the names of **all** ethics boards, governmental organizations, community leaders or other bodies that provided approval for the study. If individuals provided approval refer to these people by their role or title but do not list their name(s).

The study was conducted in close partnership with the Reproductive Health Directorate, Ministry of Health Malawi and ethical approval to conduct this research was granted by the National Health Science Research Committee (NHSRC) – an independent international review board in Malawi with Federal-Wide Assurance (IRB00003905, FWA00005976). This is also outlined in the Methods section of the manuscript.

If there were any deviations from the study protocol after approval was obtained please provide details of these changes in the Methods section of your manuscript.
Did this study involve local collaborators that are residents of the country where the research was conducted or members of the community studied? If you do not have any authors from said communities, please provide an explanation for this below.

Five out of the seven co-authors (Gracious Ali, Frehiwot Birhanu, Jessie Salamba Chirwa, Fannie Kachale, Andrews Gunda) live and work in Malawi. In addition, facility in-charges, nurses/midwives and health surveillance assistants at the sampled public sector sites were briefed about the study and collaborated with the study team in recruiting eligible participants at each site.

N/A – no deviations from study protocol after approval

Everyone listed as an author should meet PLOS’ criteria for authorship and all individuals who meet these criteria should be included in the author byline, rather than the acknowledgements. For further information please see the journal’s Authorship Policy.

**Human subjects research (e.g. health research, medical research, cross-cultural psychology)**

Did you obtain written informed consent from a representative of the local community or region before the research took place? How did you establish who speaks for the community? Details of written informed consent obtained from study participants should be reported separately in the Methods section of your manuscript.

Written approval to conduct the study was obtained from the Reproductive Health Directorate, Ministry of Health Malawi, as well as the District Health Office of each of the three study districts: Zomba, Nkhotakota and Mzimba South. This approval authorized the study team to visit the health facilities and communities in each district to collect data. At the facilities, the study team first made a courtesy call to the facility in-charge of each study site who in turn handed the team over to the family planning clinic to commence participant recruitment and data collection. Details of written informed consent obtained from study participants are reported separately in the Methods section of the manuscript.

How did members of the local community provide input on the aims of the research investigation, its methodology, and its anticipated outcome(s)?

The research questions were developed between co-authors from the Reproductive Health Directorate of the Ministry of Health, Malawi, and co-authors from Clinton Health Access Initiative, to ensure these were aligned to national research priorities. In addition, during development of the study protocol, the study team conducted consultative meetings with the three District Health Offices (Nkhotakota, Mzimba South, Zomba), including their research committees. In these meetings, the draft methodology was presented and discussed with the local team who provided valuable inputs into the study design.

When engaging with the local community, how did you ensure that the informed consent documents and other materials could be understood by local stakeholders?

As Nkhotakota and Zomba are predominantly Chichewa-speaking districts, while Mzimba South is predominantly Tumbuka-speaking, all study tools, information sheets/informed consent forms, and videos were translated or dubbed into both languages for study purposes. Research assistants speaking both languages were hired, so participants could choose the language they felt most comfortable with during interview.

Will the findings of the research be made available in an understandable format to stakeholders in the community where the study was conducted (e.g. via a presentation, summary report, copies of publications, etc.)? Please provide details of how this will be achieved.

Findings from this study have been extensively shared with national level government, NGO partners involved in the roll-out of DMPA-SC in Malawi, district-level health officials (including the research committees at the districts) and facility-level health care providers. In addition, three workshops were conducted with representatives of each study target population (providers, clients, and adolescents), in the final stages of analysis to share draft study findings for validation and discussion. These workshops were conducted in Chichewa or Tumbuka, depending on the district.

**Non-human subjects research using specimens/ animals collected as part of the study, or those housed in archival collections. Examples include archaeology, paleontology, botany and zoology.**

Did the permission you obtained from a local authority to perform the study include an agreement on access to outputs and benefit sharing? This may include procedures to enable fair distribution of the benefits and resources arising from the research performed. Please include any details of Prior Informed Consent and Benefit Sharing Agreements obtained. These may be required by field-specific regulations, for example the Convention on Biological Diversity (CBD) and the associated Nagoya Protocol.

N/A – no specimens/animals collected

If the material used in your study was imported, please A) provide the year it was imported and B) indicate whether permits were obtained to import/export the materials used, C) provide details of any permits obtained. If this information is not available, please indicate this.

N/A – no imported materials

If you used archival specimens, please state how the material used in your study was acquired by the institute it is held in and provide details of any permits obtained for the original excavations/ sample collection. If this information is not available, please indicate this.

N/A – no archival specimens

How was the potential cultural significance of the materials collected in your study to local communities considered in your research design? Were Indigenous peoples and/or local researchers and institutions involved with archaeological excavations / collection of specimens? If so, please provide a description of their involvement.

N/A – no materials collected

If your manuscript includes photographs of human remains please indicate whether authors obtained permission from descendants or affiliated cultural communities to do so.

N/A – no photographs taken
