# Supplementary material for: Health care provider and client experiences of counselling on depot medroxyprogesterone acetate subcutaneous (DMPA-SC) for self-injection in Malawi
Source: PLOS Glob Public Health. 2023 Nov 30;3(11):e0002057. doi: 10.1371/journal.pgph.0002057 (PMC10688740; doi:10.1371/journal.pgph.0002057)
Supplement: S1 Data — (ZIP) [file pgph.0002057.s001.zip › S2/C0.3 client prev experience PAIM only.doc]

Title: KK_IM_3.docx

Descriptor Info:	District: Nkhotakota	Target Population: TP2 - DMPA-IM/DMPA-SC PI clients	Respondent type: DMPA-IM client	Aware of SI: Yes - had heard of SI before	Coding phase: 1. Single coded	Client marital status: Divorced/ separated/ widowed	Client age group: 20-29 years	Time since trained: N/A - client

Codes Applied:	C0.3 - Client previous use/experience of any FP method

INTERVIEWER: … I would now like to talk to you about your experiences with contraceptive methods. Other than injectables, have you used other family planning / contraception in the past? Tell me your experiences about these other methods

RESPONDENT: So far, I have used pills

INTERVIEWER: So what has been your experience?

RESPONDENT: Mainly it was nausea

INTERVIEWER: Nausea?

RESPONDENT: when taking pills, the mail problem is nausea

INTERVIEWER: What were the pros and cons of the method you used?

RESPONDENT: The better one is the injectable method

INTERVIEWER: No I want to hear about the pill method, are there any pros for the pills

RESPONDENT: The goodness

INTERVIEWER: Pros and cons

RESPONDENT: The good side is you cannot get pregnant

INTERVIEWER: You cannot get pregnant?

RESPONDENT: The bad side is that since you take them daily you may end up missing taking the pills

INTERVIEWER: You may forget?

RESPONDENT: Yes

INTERVIEWER: Alright

RESPONDENT: You may also have unplanned pregnancy

INTERVIEWER: If you stopped using the method, why did you stop?

RESPONDENT: Because I could sometimes miss the pills

INTERVIEWER: So you thought of dropping it

RESPONDENT: Yes (participant laugh) I felt I may be in danger

Title: KK_IM_3.docx

Descriptor Info:	District: Nkhotakota	Target Population: TP2 - DMPA-IM/DMPA-SC PI clients	Respondent type: DMPA-IM client	Aware of SI: Yes - had heard of SI before	Coding phase: 1. Single coded	Client marital status: Divorced/ separated/ widowed	Client age group: 20-29 years	Time since trained: N/A - client

Codes Applied:	C0.3 - Client previous use/experience of any FP method

INTERVIEWER: What was it that first drew you to using injectable contraceptives (10 minutes 4 seconds).

RESPONDENT: What drew me is the fact that I should have child spacing.

INTERVIEWER: Is that what drew you to using injectables?

RESPONDENT: Yes

INTERVIEWER: But pills also make one to have child spacing, what is it that drew to using injectables?

RESPONDENT: “Participant Laugh” what prompted me was that I should have enough time to care for my family

INTERVIEWER: You needed enough time to care for your family, does it mean that when using pills you could not care for your family?

RESPONDENT: “Participant laugh” it was because I could forget

INTERVIEWER: Ok our could forget ?

RESPONDENT: Yes

INTERVIEWER: Alright, what do you like about injectables?

RESPONDENT: like? aaaa “participant was not sure on what to respond”

INTERVIEWER: I thought you have used pills before, and now you are using injection. Let’s put it this way: how are you able to differentiate the good side and bad side, as you are using injection and the time you were using pills?

RESPONDENT: Mhh “participant not sure of what to answer”

INTERVIEWER: What is it that you like about injection method compared to pills may be u may say when using injection this is what I am experiencing over pills. May be that could be one of the explanations

RESPONDENT: Injection method is so helpful because there are no issues of forgetting / missing out some dates.

INTERVIEWER: What is it that you don’t forget to get injected

RESPONDENT: It helps since, when using pills, you take them every day so it’s easy to forget some days. Whilst the injectable one you are given dates for you to go back for another injection so you cannot forget.

INTERVIEWER: Alright, how long have you been using injectables?

RESPONDENT: For three years

Title: MZs_IM_8.docx

Descriptor Info:	District: Mzimba South	Target Population: TP2 - DMPA-IM/DMPA-SC PI clients	Respondent type: DMPA-IM client	Aware of SI: Yes - had heard of SI before	Coding phase: 1. Single coded	Client marital status: Married	Client age group: 20-29 years	Time since trained: N/A - client

Codes Applied:	C0.3 - Client previous use/experience of any FP method	C0.2.1 - Positive community/client perspectives on FP

Interviewer: now I would like to ask about your experience on using family planning methods. Apart from Depo-IM, have you used another family planning method before?

Respondent: no

Interviewer: since your first child?

Respondent: yes

Interviewer: what motivated you to use Depo-IM?

Respondent: I got married when I was young. I gave birth with complications. So I wanted to delay the next pregnancy. So I inquired from doctors.

Interviewer: what do you like most about Depo-IM?

Respondent: I have normal menses,

Interviewer: don’t you have heavy menses ?

Respondent: no

Interviewer: what else?

Respondent: I do not experience any problem

Interviewer: how long have you been using Depo-IM?

Respondent: I used from the birth of my first child until he was 4 years then I got pregnant for the second child then the second child was 2 years I started again until now

Title: MZs_IM_2.docx

Descriptor Info:	District: Mzimba South	Target Population: TP2 - DMPA-IM/DMPA-SC PI clients	Respondent type: DMPA-IM client	Aware of SI: Yes - had heard of SI before	Coding phase: 3. Triple coded	Client marital status: Married	Client age group: 30-39 years	Time since trained: N/A - client

Codes Applied:	C0.3 - Client previous use/experience of any FP method	C0.2.2 - Negative community/client perspectives on FP

Q5. Interviewer: Other than injectables, have you used other family planning/contraceptive in the past?

Respondent: Yes, I have ever used.

Interviewer: Which method were you using?

Respondent: I was using the one for 5 years [Jadelle, 5-year implant].

Interviewer: Mmmmmm. (following).

Respondent: But since I started using it, I was bleeding too much and, in a month, it was only 1 week without bleeding as a result I request for the removal. I only stayed 1 year with it because I thought my life was in danger then I opt for injectables and with this one all is well sometimes I stay 4 months no menstrual and when I start menstrual I do it only one week and I like this method very much.

Interviewer: So this method you were using, where did you receive it from?

Respondent: From the facility.

Interviewer: You have only mentioned the bad things about the method, what about the good ones?

Respondent: Mmmmm…. that time I wanted to rest from being injected (injectables), thus why I opt for this method, unfortunately the method didn`t work for me and I didn`t see anything good about the method and my fear was that I may end up having less blood in my body.

Title: MZs_IM_2.docx

Descriptor Info:	District: Mzimba South	Target Population: TP2 - DMPA-IM/DMPA-SC PI clients	Respondent type: DMPA-IM client	Aware of SI: Yes - had heard of SI before	Coding phase: 3. Triple coded	Client marital status: Married	Client age group: 30-39 years	Time since trained: N/A - client

Codes Applied:	C0.3 - Client previous use/experience of any FP method	C8.1 - Male involvement, general	C8.2 - Covert/ open use of injectables/SI

Q6. Interviewer: How is or difficult have you found it accessing family planning services in the past?

Respondent: At first it was difficult cause I did not open up to my husband because most of the times we were using condoms, then something happened, I get pregnant like this year and the other year another child thus why I decided to start using family planning method. (the 5 years one - Jadelle) and I thought it necessary for me to tell him. It was difficult because I did not tell him and about family planning, he was hearing it from other people and after telling him about it he said let’s go to the hospital so that they should explain to us in details.

Title: MZs_IM_2.docx

Descriptor Info:	District: Mzimba South	Target Population: TP2 - DMPA-IM/DMPA-SC PI clients	Respondent type: DMPA-IM client	Aware of SI: Yes - had heard of SI before	Coding phase: 3. Triple coded	Client marital status: Married	Client age group: 30-39 years	Time since trained: N/A - client

Codes Applied:	C0.3 - Client previous use/experience of any FP method

Interviewer: Tell me why did you choose injectables?

Respondent: The way doctor explained family planning methods, injectables was the best for me because I wanted to see if it will work well with me. I was thinking that if I take the method that is for more years and it doesn`t work for me then I will end up regretting. For pills I thought of I might forgot cause its every day, as a result I chose Jadelle that I will be using it for 3 months and if it doesn`t work for me I will change to another one. Since I started using injectables there is not any problem everything is working very well thus why I have been using it for about 13 years.

Interviewer: What do you like about injectables?

Respondent: What I like about injectables is that even if I go to my village and forgot the date am supposed to go the hospital for the injection or maybe I am busy with something and I forgot to go to the hospital I don’t get pregnant because this method works more days than expected. Rather than, if you forgot the date to go to the hospital for an injection that means if you sleep with your husband that means you are pregnant.

Interviewer: Mmmmm. (following)

Respondent: If you forgot the date and you remember the next day still, I don’t get bothered about it and you can have it with your husband without any worry that I might get pregnant.

Interviewer: Mmmmh?

Respondent: Yes, thus the good thing about this method.

Interviewer: Anything else?

Respondent: As for me I know that with this method everything is okay because whenever we are together with my husband everything works perfect and there is no different as before I started using it and now.

Interviewer: You said something on how you do your menstruation, would you tell me how you do your menstruation?

Respondent: This injectables is different from the one I was using (Jadelle). With Jadelle I used to bleed too much and with this one I do my menstrual normal and even if I stay one or two months without doing my menstrual, I don’t get worried cause I know for sure that next months I will.

Title: ZA_IM_2.docx

Descriptor Info:	District: Zomba	Target Population: TP2 - DMPA-IM/DMPA-SC PI clients	Respondent type: DMPA-IM client	Aware of SI: Yes - had heard of SI before	Coding phase: 1. Single coded	Client marital status: Married	Client age group: 30-39 years	Time since trained: N/A - client

Codes Applied:	C0.3 - Client previous use/experience of any FP method

Interviewer: Now I want to get into another section of questions, I want to talk to you about your experience using contraceptives, apart from the injection mentioned, have you used any other family planning methods in the past other than the injectables?

Respondent: Yes, I have used condoms.

Interviewer: Alright

Respondent: mm (Nods to show agreement) mmhh (Showing agreement)

Interviewer: Can you tell me your experience using this condom method.

Respondent: Ah, there is no problem that I have encountered

Title: ZA_IM_2.docx

Descriptor Info:	District: Zomba	Target Population: TP2 - DMPA-IM/DMPA-SC PI clients	Respondent type: DMPA-IM client	Aware of SI: Yes - had heard of SI before	Coding phase: 1. Single coded	Client marital status: Married	Client age group: 30-39 years	Time since trained: N/A - client

Codes Applied:	C0.3 - Client previous use/experience of any FP method

Interviewer: Alright, you have told me that this family planning method of condoms, there is no problem that you have encountered with I, are there any good things that you can say you have experienced with this family planning method?

Respondent: Yes, the good thing is that whenever I told my husband that we should use this method family planning he was not resisting, some people complain that they cannot afford to use a condom during sex. My husband was accepting to have sex using a condom as a family planning method.

Interviewer: Alright, you have told me that currently you are using injectables as a family planning method, why did you stop using condoms?

Respondent: I used condoms because my child as so small, it’s not that my child was too young for me not to have sex with my husband, no, we would have been able to have sex, but I had not yet made the decision to go and take the injectable method yet.

Title: ZA_IM_2.docx

Descriptor Info:	District: Zomba	Target Population: TP2 - DMPA-IM/DMPA-SC PI clients	Respondent type: DMPA-IM client	Aware of SI: Yes - had heard of SI before	Coding phase: 1. Single coded	Client marital status: Married	Client age group: 30-39 years	Time since trained: N/A - client

Codes Applied:	C0.3 - Client previous use/experience of any FP method

Interviewer: Alright, what made you start using the injectable contraceptives?

Respondent: The reason I started using injectable contraceptive is that I was feeling sorry for my husband that he might be upset so I saw that it was better that I take the injectables, so I can stay for a long period without having menstrual periods and I can be able to have sex with my husband without any problems and without using the condoms.

Interviewer: Alright, what do you like about the injectable contraceptives and what do you enjoy about this method?

Respondent: The thing that appeals to me on the injectable contraceptive method (A considerable pause) is that when I want to have sex with my husband, we can do that without any problems and also without any care that I will get pregnant, and also, I experience changed menstrual period schedules, I have lighter menstrual periods and they are not as random (month to month) compared to when one is not taking any contraceptive method.

Interviewer: mm (Showing that one is following the conversation)

Respondent: Yah

Interviewer: Alright, is there anything that you don’t like about the injectable contraceptive method?

Respondent: No there is nothing

Interviewer: Alright, Maybe just let me know, how long have you been using the injectable contraceptive method?

Respondent: I have been using the injectable contraceptive since 2015 up to 2021.

Interviewer: Alright, that means you have been using it for almost six years, right?

Respondent: Yes

Title: KK_IM_5.docx

Descriptor Info:	District: Nkhotakota	Target Population: TP2 - DMPA-IM/DMPA-SC PI clients	Respondent type: DMPA-IM client	Aware of SI: No - never heard of SI before	Coding phase: 3. Triple coded	Client marital status: Divorced/ separated/ widowed	Client age group: Under 20 (adolescent)	Time since trained: N/A - client

Codes Applied:	C0.3 - Client previous use/experience of any FP method

INTERVIEWER: Alright because she is taking contraceptives? Alright let’s continue with our discussions. I would now like to talk to you about your experiences with contraceptive methods. Other than injectables, have you used other family planning / contraception in the past?

RESPONDENT: No

Title: KK_IM_5.docx

Descriptor Info:	District: Nkhotakota	Target Population: TP2 - DMPA-IM/DMPA-SC PI clients	Respondent type: DMPA-IM client	Aware of SI: No - never heard of SI before	Coding phase: 3. Triple coded	Client marital status: Divorced/ separated/ widowed	Client age group: Under 20 (adolescent)	Time since trained: N/A - client

Codes Applied:	C0.3 - Client previous use/experience of any FP method

INTERVIEWER: Alright, we are proceeding, what factors have made it easy to take injectables?

(10 minutes 15 Seconds)

RESPONDENT: After I took the first contraception, I noted that I was having prolonged periods hence I decided to continue with it.

INTERVIEWER: Ook you were not having prolonged periods after the first injection?

RESPONDENT: Yes, so I thought of continuing with it.

INTERVIEWER: For how long have you been using the injectables?

RESPONDENT: For a year and 6 months

INTERVIEWER: For a year and 6 months ?

RESPONDENT: Yes

Title: ZA_IM_1.docx

Descriptor Info:	District: Zomba	Target Population: TP2 - DMPA-IM/DMPA-SC PI clients	Respondent type: DMPA-IM client	Aware of SI: Yes - had heard of SI before	Coding phase: 1. Single coded	Client marital status: Married	Client age group: 40-49 years	Time since trained: N/A - client

Codes Applied:	C0.3 - Client previous use/experience of any FP method	C0.2 - Community and client perspectives on FP - general

Interviewer: Alright thank you very much, I would like now to talk to you about your experiences with contraceptive methods. Other than injectables, have you used other family planning/ contraceptive in the past?

Respondent: Yes, I used the pills method

Interviewer: Alright, Tell me about your experiences with this method?

Respondent: At the time I was using pills I would forget to take my pills. I viewed this as a risk. I then made a decision to start using the injection method.

Interviewer: Alright, alright, where were you accessing this method from?

Respondent: Here at Zilindo Hospital

Interviewer: At this same hospital Zilindo?

Respondent: Yes

Interviewer: What were the pros and cons of the method you used? You earlier mentioned that you used pill. You have already mentioned about the pros and cons of using pills. What are the cons of using the injection method?

Respondent: The pros of using the injection method is that you get injected once and 3 months goes by without getting injected.

Interviewer: Mhhhh

Respondent: You are free person during the other days eeh

Interviewer: What are the cons?

Respondent: The cons are on the menstruating side

Interviewer: mhhhh

Respondent: They are irregular I sometimes go a month without seeing my periods. I sometimes just menstruate a little bit. Further to this, I experience backaches

Title: ZA_IM_1.docx

Descriptor Info:	District: Zomba	Target Population: TP2 - DMPA-IM/DMPA-SC PI clients	Respondent type: DMPA-IM client	Aware of SI: Yes - had heard of SI before	Coding phase: 1. Single coded	Client marital status: Married	Client age group: 40-49 years	Time since trained: N/A - client

Codes Applied:	C0.3 - Client previous use/experience of any FP method	C0.2 - Community and client perspectives on FP - general	C8.1 - Male involvement, general

Interviewer: How easy or difficult have you found it accessing family planning services in the past?

Respondent: Ahhh, in the past it was not really hard per se, because the time we made a choice to start using contraceptives the methods were available at the hospital. As I mentioned earlier I started using the injection because I used to forget to take my pills that’s when I started using this method. It was not really hard.

Interviewer: Alright, if it was easy, what factors made it easy?

Respondent: Ahhh, things that have helped are like openness with my husband as well as freely doing my work because I don’t have children who are not well spaced.

Interviewer: You explained that you easily accessed these methods, what enabled you to easily access these services? What made it easy for you to access the services?

Respondent: I heard that there are methods that’s when I can to the hospital to choose the suitable method that I can use.

Interviewer: Thank you very much, has there been any changes in accessing family planning services since the outbreak of Corona?

Respondent: No, there has been no changes.

Time stamp: 10:42

Interviewer: Alright, alright thank you very much. What was it that drew you to using injectable contraception?

Respondent: What drew me is that I saw that I had enough children and am supposed stay a long time without getting pregnant that’s why I started taking contraception that I should be able to freely do my work.

Interviewer: Alright, Alright, just to add on that, what is it that you like about injectables?

Respondent: I like the fact that once I get injected I rest for 3 months whilst the injection is still working in my body. I indeed stay for a long time.

Interviewer: Mhhhh

Respondent: Yes

Interviewer: Alright, alright, what is it that you don’t like about injectables?

Respondent: The thing that I don’t like is the issue about the body. As for me, my back aches a lot. People say that this is due to the injectable.

Interviewer: Mhhhh

Respondent: Yes

Interviewer: Is there anything you would like to add?

Respondent: I also experience headaches

Interviewer: Alright

Respondent: Indeed

Interviewer: Thank you very much. For how long have you been using injectables for?

Respondent: Ahh close to 6 years

Title: KK_SCPI_6.docx

Descriptor Info:	District: Nkhotakota	Target Population: TP2 - DMPA-IM/DMPA-SC PI clients	Respondent type: DMPA-SC PI client	Aware of SI: Yes - had heard of SI before	Coding phase: 1. Single coded	Client marital status: Married	Client age group: 20-29 years	Time since trained: N/A - client

Codes Applied:	C0.3 - Client previous use/experience of any FP method

INTERVIEWER: What do they say pills do?

RESPONDENT: Some say pills are good. However, it is not all people, people admire different things. Even Norplant, some say it is good but for me it is not, with the experience I have.

INTERVIEWER: okay.

RESPONDENT: I was menstruating the whole month. Then some say they menstruate normally. However, in the area I come from more people use family planning methods. In addition, they appreciate contraception.

Title: KK_SCPI_6.docx

Descriptor Info:	District: Nkhotakota	Target Population: TP2 - DMPA-IM/DMPA-SC PI clients	Respondent type: DMPA-SC PI client	Aware of SI: Yes - had heard of SI before	Coding phase: 1. Single coded	Client marital status: Married	Client age group: 20-29 years	Time since trained: N/A - client

Codes Applied:	C0.3 - Client previous use/experience of any FP method	C0.2.2 - Negative community/client perspectives on FP

INTERVIEWER: Alright. Now I would like to discuss your experiences in using family planning methods. Now, apart from the injection type, have you used any other type of contraception previously?

Time stamp: (10:02)

RESPONDENT: Yes. Norplant.

INTERVIEWER: Norplant?

RESPONDENT: mmmh.

INTERVIEWER: What else?

RESPONDENT: Pills

INTERVIEWER: Pills?

RESPONDENT: mmmh.

INTERVIEWER: What were your experiences using Norplant?

RESPONDENT: The disadvantages I encountered using Norplant were that I was menstruating everyday of a month as I already explained.

INTERVIEWER: mmmmhu.

RESPONDENT: mmmh.

INTERVIEWER: What are other problems?

RESPONDENT: The other problems were having serious back pains. These were the only problems I had.

INTERVIEWER: What were the problems you were facing while using pills?

RESPONDENT: I had serious back pains. When sleeping I had continuous back pains. And the other reason that made me to stop is that I was forgetting the days when I was supposed to take the pills.

INTERVIEWER: Pills?

RESPONDENT: mmmh. So I could take the pills yes, but you would find that the next day I forget that I take pills, then maybe I would take in the evening and then the following day I forget and may be remember two days later. Because of that I decided to change because I figured that I might end up getting pregnant unexpectedly.

INTERVIEWER: mmmhu.

RESPONDENT: Yes

Title: KK_SCPI_6.docx

Descriptor Info:	District: Nkhotakota	Target Population: TP2 - DMPA-IM/DMPA-SC PI clients	Respondent type: DMPA-SC PI client	Aware of SI: Yes - had heard of SI before	Coding phase: 1. Single coded	Client marital status: Married	Client age group: 20-29 years	Time since trained: N/A - client

Codes Applied:	C0.3 - Client previous use/experience of any FP method	C0.2.1 - Positive community/client perspectives on FP

INTERVIEWER: Alright, do you get these family planning methods from the hospital, health personnel or somewhere else? These methods, where do you get them?

RESPONDENT: The hospital.

INTERVIEWER: This same hospital?

RESPONDENT: yes

INTERVIEWER: Okay. You have told me the bad side of pills and Norplant that they are to do with the back and menstruation but what are the good sides of these two methods?

RESPONDENT: About Norplant?

INTERVIEWER: Yes, Norplant and pills. What are the good sides that you saw with these two methods?

RESPONDENT: Their advantages?

INTERVIEWER: mmmmh.

RESPONDENT: The good part with Norplant is when you have it planted you take three years before going to the hospital for the same cause unless you have some different reason for going to the hospital.

INTERVIEWER: okay.

RESPONDENT: mmmmh.

INTERVIEWER: Pills?

RESPONDENT: The good side I saw with pills is that when you get them you drink them at home.

Title: KK_SCPI_6.docx

Descriptor Info:	District: Nkhotakota	Target Population: TP2 - DMPA-IM/DMPA-SC PI clients	Respondent type: DMPA-SC PI client	Aware of SI: Yes - had heard of SI before	Coding phase: 1. Single coded	Client marital status: Married	Client age group: 20-29 years	Time since trained: N/A - client

Codes Applied:	C0.3 - Client previous use/experience of any FP method	C0.2.1 - Positive community/client perspectives on FP

INTERVIEWER: okay. Now, what motivated you to start using the injection type of family planning?

RESPONDENT: I was hearing from my friends that the injection type is good and that I should go get it given the experience I previously had with contraceptives. Therefore, they were guiding me to go get the injection type because I had thought of stopping family planning all together. Nevertheless, the people who were using the injection type were encouraging me not to stop rather I should get the injection contraception instead.

INTERVIEWER: okay.

RESPONDENT: Yes

INTERVIEWER: What do you like about the injection contraceptive?

RESPONDENT: Liking?

INTERVIEWER: Why do you like the injection contraceptive?

RESPONDENT: Because it does not keep you busy coming to the hospital frequently.

INTERVIEWER: okay. What is that you do not like about the injection contraceptive? Everything has its good and bad side. You have mentioned that you like it because it does not keep you busy frequenting to the hospital. How about the side that you do not like?

RESPONDENT: Aaah I... I do like it.

INTERVIEWER: okay. How long have you been using the injective contraception?

RESPONDENT: A year and ten months.

INTERVIEWER: A year and ten months.

RESPONDENT: Before the birth of this child, I was also using the injection contraceptive.

INTERVIEWER: Okay

RESPONDENT: Long time ago before I got pregnant

INTERVIEWER: aha.

RESPONDENT: I was using the injection type.

INTERVIEWER: It means the period in which you have been using the injective contraception is more than a year and ten months?

RESPONDENT: Yes

Title: KK_SCPI_4.docx

Descriptor Info:	District: Nkhotakota	Target Population: TP2 - DMPA-IM/DMPA-SC PI clients	Respondent type: DMPA-SC PI client	Aware of SI: Yes - had heard of SI before	Coding phase: 2. Double coded	Client marital status: Never married	Client age group: 20-29 years	Time since trained: N/A - client

Codes Applied:	C0.3 - Client previous use/experience of any FP method

INTERVIEWER: Okay, I am continuing. Now I want us to talk about what you are experiencing while using contraceptions. Apart from the injective contraception that you are currently using, have you used any other contraception method previously?

RESPONDENT: No. starting with the previous pregnancy I had, I miscarried.

INTERVIEWER: pepani. Sorry

RESPONDENT: mmmh. From there, I gave birth through operation.

INTERVIEWER: Sorry

RESPONDENT: So from there I decided to wait, so I got the injective contraception, that was before Sayana came in. Another injection contraceptive was being given. Whatever they were calling it.

INTERVIEWER: Depo?

RESPONDENT: I was taking that one. I took three of them. Thereafter I got pregnant. For this baby also I gave birth through operation. Thereafter I came here, and I was told that Sayana had come so I took it...I took the injection one as in continuing with this same method. However, these other family planning methods, I feel like with my body…because I started the injection one…there has been no negative effect on my body since I started using it.

INTERVIEWER: mmmhu.

RESPONDENT: mmmh.

INTERVIEWER: mmmhu. Okay, just to be clear, you said you use the injection one only?

RESPONDENT: Yes

INTERVIEWER: Nevertheless, there are different family planning methods…several. May be pills, Norplant, may be condoms, have…have you used anyone of those methods?

Time stamp: (10:01)

RESPONDENT: No. I have used condoms before.

INTERVIEWER: You have used?

RESPONDENT: Yes

INTERVIEWER: mmmhu. what did you experience using this method, the condoms?

RESPONDENT: Condoms?

INTERVIEWER: mmmmh.

RESPONDENT: I have now encountered any challenge about condoms.

Title: KK_SCPI_4.docx

Descriptor Info:	District: Nkhotakota	Target Population: TP2 - DMPA-IM/DMPA-SC PI clients	Respondent type: DMPA-SC PI client	Aware of SI: Yes - had heard of SI before	Coding phase: 2. Double coded	Client marital status: Never married	Client age group: 20-29 years	Time since trained: N/A - client

Codes Applied:	C0.3 - Client previous use/experience of any FP method

INTERVIEWER: Why did you stop using these condoms?

RESPONDENT: I (laughs). There were a few reasons right. Sometimes you are married and in the house. Can a man manage? the condoms? You cannot manage. That is why I started this injection method.

INTERVIEWER: Okay.

RESPONDENT: mmmh.

INTERVIEWER: As you were using these condoms, what were the advantages that you observed?

RESPONDENT: I observed that using condoms even in a relationship it means you will not get pregnant because you are protected.

INTERVIEWER: okay.

RESPONDENT: Yes

Title: KK_SCPI_4.docx

Descriptor Info:	District: Nkhotakota	Target Population: TP2 - DMPA-IM/DMPA-SC PI clients	Respondent type: DMPA-SC PI client	Aware of SI: Yes - had heard of SI before	Coding phase: 2. Double coded	Client marital status: Never married	Client age group: 20-29 years	Time since trained: N/A - client

Codes Applied:	C0.3 - Client previous use/experience of any FP method

INTERVIEWER: What about what does not please you about the injection contraception?

RESPONDENT: About the non-injective contraception; the one we put on the arm, is that what you mean?

INTERVIEWER: Any of it whether Sayana or whichever, what is it that you do not like about the injection that you use?

RESPONDENT: With this contraception method whether the loop one, does not please me

Title: KK_SCPI_4.docx

Descriptor Info:	District: Nkhotakota	Target Population: TP2 - DMPA-IM/DMPA-SC PI clients	Respondent type: DMPA-SC PI client	Aware of SI: Yes - had heard of SI before	Coding phase: 2. Double coded	Client marital status: Never married	Client age group: 20-29 years	Time since trained: N/A - client

Codes Applied:	C0.3 - Client previous use/experience of any FP method

INTERVIEWER: How about the injection that you use. Everything has its good …

RESPONDENT: Its good side

INTERVIEWER: And bad side. What makes you say for me I am not taking this one because of these reasons, what are the reasons.

RESPONDENT: No, as for me since I started taking the injection method, I cannot distinguish between the contraception methods.

INTERVIEWER: okay.

RESPONDENT: Yes. Because since I started taking the injection contraception, I have not been able to tell the good and bad sides of it may be in future things will change.

INTERVIEWER: okay. How long have you been using the injection contraception method?

RESPONDENT: I have just used it three times, one year I should say.

INTERVIEWER: One year?

RESPONDENT: mmmh

Title: ZA_IM_6.docx

Descriptor Info:	District: Zomba	Target Population: TP2 - DMPA-IM/DMPA-SC PI clients	Respondent type: DMPA-IM client	Aware of SI: Yes - had heard of SI before	Coding phase: 1. Single coded	Client marital status: Married	Client age group: 30-39 years	Time since trained: N/A - client

Codes Applied:	C0.3 - Client previous use/experience of any FP method

Interviewer: Alright, thank you so much. Now I would like us to talk about your experience using a contraceptive method. Other than the injection method, what other methods have you used in the past?

Respondent: No! it’s just the injection.

Interviewer: Where do you get your injection?

Respondent: Here in Matawale.

Interviewer: Oh! You did not get it from your health counselors?

Respondent: Mmhmm I come here.

Interviewer: Alright. What good and bad issues do you go through when using this method?

Respondent: At first there were no problems, I could not menstruate after using the method and I could stay through the days till my next injection. But as of now I feel nauseated, or I feel like vomiting and sometimes like am pregnant.

Interviewer: Mhmm

Respondent: Mhhmm

Interviewer: Sometimes am selective with food for instance I don’t eat nsima from fermented flour.

Respondent: Mmhhm

Interviewer: Eh! Because of the injection yes

Respondent: Alright

Interviewer: Anything to add? Any good results from the contraceptive you have used? What is good about the injection?

Respondent: The good thing about the injection is that you use it so that your child grows well.

Interviewer: Mmhhmm

Respondent: Mmhhm

Interviewer: Any other good thing?

Respondent: No

Title: ZA_IM_6.docx

Descriptor Info:	District: Zomba	Target Population: TP2 - DMPA-IM/DMPA-SC PI clients	Respondent type: DMPA-IM client	Aware of SI: Yes - had heard of SI before	Coding phase: 1. Single coded	Client marital status: Married	Client age group: 30-39 years	Time since trained: N/A - client

Codes Applied:	C0.3 - Client previous use/experience of any FP method

Interviewer: Alright thank you so much. What first drew you to using the injection method?

Respondent: Because the injection works for three months, it is easy to stop using any time and get pregnant after the three months.

Interviewer: Mmhhm

Respondent: Without contraception.

Interviewer: Mhmm

Respondent: So you get pregnant, yet the arm method takes five years so to reach the five years!

Interviewer: Mmhmm

Respondent: Which makes the injection good to me.

Interviewer: Alright.

Respondent: Mmhhm

Interviewer: What do you not like about the injection? What don’t you love about it?

Respondent: Nothing.

Time stamp: 9:22

Interviewer: For how long have you been using the injection method?

Respondent: mmhmm a very long time, even after my first child.

Interviewer: Mmhmm

Respondent: The second child as well.

Interviewer: mmhmm

Respondent: Since I became a mother.

Interviewer: Mhhmm

Respondent: Mmhhm

Interviewer: For how many years?

Respondent: A lot of years considering one child is twelve and the other is seven, so it is a lot of years.

Interviewer: So you have been injecting for twelve years?

Respondent: Yes, it’s been years.

Interviewer: Mhhmm almost twelve years?

Respondent: Mhhmm

Title: ZA_SCPI_3.docx

Descriptor Info:	District: Zomba	Target Population: TP2 - DMPA-IM/DMPA-SC PI clients	Respondent type: DMPA-SC PI client	Aware of SI: No - never heard of SI before	Coding phase: 3. Triple coded	Client marital status: Never married	Client age group: Under 20 (adolescent)	Time since trained: N/A - client

Codes Applied:	C0.3 - Client previous use/experience of any FP method

Interviewer: Ah other than injectables, have you used any other family planning methods in the past?

Respondent: Yes

Interviewer: Which methods have you used?

Respondent: Condoms

Interviewer: Okay alright.

Respondent: And pills

Interviewer: Pills

Respondent: mmmm (agreeing)

Title: ZA_SCPI_3.docx

Descriptor Info:	District: Zomba	Target Population: TP2 - DMPA-IM/DMPA-SC PI clients	Respondent type: DMPA-SC PI client	Aware of SI: No - never heard of SI before	Coding phase: 3. Triple coded	Client marital status: Never married	Client age group: Under 20 (adolescent)	Time since trained: N/A - client

Codes Applied:	C0.3 - Client previous use/experience of any FP method

Interviewer: Alright, what were the pros and cons of using these methods you have mentioned?

Respondent: Yes, the pills…

Interviewer: mmm (showing that one is following the conversation)

Respondent: I used to have heavy bleeding more often

Interviewer: Okay

Respondent: Sometimes I would bleed the whole month.

Interviewer: Okay

Respondent: Yes

Interviewer: Alright, what about the condoms, were there any pros and cons?

Respondent: there were disadvantages with condoms…

Interviewer: mmm (showing that one is following the conversation)

Respondent: During that time, I was using condoms before I started using family planning…

Interviewer: mmm (showing that one is following the conversation)

Respondent: I had a strange pain on my navel

Interviewer: Okay

Respondent: So, I had a strange illness, I used to urinate blood…

Interviewer: oh (showing concern)

Respondent: Yes

Interviewer: Alright, ah so it means you stopped using these methods because of the problems you have told me?

Respondent: Yes.

Interviewer: Are there any other reasons?

Respondent: No.

Interviewer: Alright, ah how old were you when you started using contraceptives?

Respondent: 16

Title: ZA_SCPI_3.docx

Descriptor Info:	District: Zomba	Target Population: TP2 - DMPA-IM/DMPA-SC PI clients	Respondent type: DMPA-SC PI client	Aware of SI: No - never heard of SI before	Coding phase: 3. Triple coded	Client marital status: Never married	Client age group: Under 20 (adolescent)	Time since trained: N/A - client

Codes Applied:	A5.2 - Stigma/judgement	A4 - Adol access to FP services	C0.3 - Client previous use/experience of any FP method	C0.2 - Community and client perspectives on FP - general

Interviewer: Alright, what was it that first drew you to start using injectable contraceptive?

Respondent: Okay, when I was in a relationship which I still am in now, yeah…

Interviewer: eh

Respondent: I got pregnant…

Interviewer: Okay

Respondent: so, when I got pregnant that time…

Interviewer: mmm (showing that one is following the conversation)

Respondent: I kept the pregnancy…

Interviewer: mmmm (showing that one is following the conversation)

Respondent: And then I miscarried.

Interviewer: Okay,

Respondent: mmm so when I went to the hospital…

Interviewer: mmm (showing that one is following the conversation)

Respondent: I stayed there for two weeks.

Interviewer: mmm (Showing that one is following the conversation)

Respondent: When I was being discharged, I was advised to be on contraceptives.

Interviewer: mmmm (showing that one is following the conversation)

Respondent: It was forced on me, yeah…

Interviewer: eh

Respondent: so, during that time I started using family planning methods…

Interviewer: Okay…

Respondent: so, after some time of using it, I saw that I should continue being on contraceptives.

Interviewer: mmm (showing that one is following the conversation)

Respondent: If I don’t continue being on contraceptives, then I will get another pregnancy.

Interviewer: Alright.

Title: ZA_SCPI_3.docx

Descriptor Info:	District: Zomba	Target Population: TP2 - DMPA-IM/DMPA-SC PI clients	Respondent type: DMPA-SC PI client	Aware of SI: No - never heard of SI before	Coding phase: 3. Triple coded	Client marital status: Never married	Client age group: Under 20 (adolescent)	Time since trained: N/A - client

Codes Applied:	C0.3 - Client previous use/experience of any FP method

Interviewer: Alright, what appealed most to you about injectables?

Respondent: I no longer have menstrual periods.

Interviewer: Alright, is there anything that you don’t like about injectables?

Respondent: No

Interviewer: Alright, how long have you used this method of family planning? The injectables.

Respondent: I have been injected three times.

Interviewer: Okay

Respondent: mmm (agreeing)

Interviewer: meaning that it’s been almost a year?

Respondent: Yes.

Interviewer: of using it...

Respondent: After doing three injections I changed

Interviewer: mmmm (showing that one is following the conversation)

Respondent: I took the pills

Title: MZs_IM_5.docx

Descriptor Info:	District: Mzimba South	Target Population: TP2 - DMPA-IM/DMPA-SC PI clients	Respondent type: DMPA-IM client	Aware of SI: Yes - had heard of SI before	Coding phase: 1. Single coded	Client marital status: Married	Client age group: 20-29 years	Time since trained: N/A - client

Codes Applied:	C0.3 - Client previous use/experience of any FP method

Interviewer: now I would like to ask on your experiences while using this family planning method apart from IM Depo, have you ever used another method?

Respondent: my husband used to refuse condom, so we were using withdrawal method but then he came to get condoms again

Interviewer: who told you about the withdrawal method?

Respondent: (giggles) no one we just tried

Interviewer: did you see any benefits of the method?

Respondent: it was generally risky.

Interviewer: what were the good parts of using condoms?

Respondent: generally, it was good but at some point, I was feeling the heat when we use condoms. But the good thing I did not got pregnant

Title: MZs_IM_5.docx

Descriptor Info:	District: Mzimba South	Target Population: TP2 - DMPA-IM/DMPA-SC PI clients	Respondent type: DMPA-IM client	Aware of SI: Yes - had heard of SI before	Coding phase: 1. Single coded	Client marital status: Married	Client age group: 20-29 years	Time since trained: N/A - client

Codes Applied:	C0.3 - Client previous use/experience of any FP method

Interviewer: what do you like most about Depo-IM?

Respondent: I like this one because I have difficulties and fears with the self-injection. I feel I might get pregnant after self-injection. I had those fears.

Interviewer: anything you liked on Depo-IM?

Respondent: I have not experienced problems like heavy menses and heart palpitations

Interviewer: what do you not like about Depo?

Respondent: nothing

Interviewer: how long have you used Depo-IM?

Respondent: since 2010

Title: MZs_IM_6.docx

Descriptor Info:	District: Mzimba South	Target Population: TP2 - DMPA-IM/DMPA-SC PI clients	Respondent type: DMPA-IM client	Aware of SI: Yes - had heard of SI before	Coding phase: 1. Single coded	Client marital status: Married	Client age group: Under 20 (adolescent)	Time since trained: N/A - client

Codes Applied:	C0.3 - Client previous use/experience of any FP method

Interviewer: At what age did you start using family planning method?

Respondent: At 16 years’ old

Interviewer: You mentioned that some come to get methods of family planning method. Which methods of family planning do they mention?

Respondent: So when we come, we meet providers from Banja La Mtsogolo they teach us on all family planning methods. Then we chose from there. Like me I choose the one which we inject after 3 months.

Interviewer: Thank you so much. Now we would like to talk about what you experienced when using this family planning method. Apart from the one you use (provider-injected) have you ever used other methods of family planning methods?

Respondent: Mmmmmh no

Interviewer: The one which you were injected? Where did you get it from?

Respondent: Here at the health facility

Interviewer: Here at the facility? how easy or difficult has it been for you to access this family planning method you use; I mean Depo?

Respondent: Come again?

Interviewer: Has it been easy? Or difficult?

respondent: No, it is not difficult

Interviewer: What factors made it easy for you to access Depo?

Respondent: It’s because the providers are readily available here at the health facility and they provide the services at any time you come

Interviewer: Did the coming of covid-19 have an impact on the accessibility of family planning services?

Respondent: With the coming of covid-19 we were told that each one of us should start self-injecting of-course am not very sure if that was the main reason

Interviewer: What made you choose intramuscular Depo Provera?

Respondent: I was influenced by my neighbor who uses same method and she encouraged me to use the same method

Interviewer: What method did you say you use? Provider injected or you inject yourself?

Respondent: Sayana….[stammer]…Depo... [giggles] it’s the provider who inject every 3 months

Interviewer: Every 3 months?

Respondent: Yes

Interviewer: What is it that you like on this method that you are using?

Respondent: What I like? Ever since I started using this method, I have never encountered any problem like getting pregnant or any other problem, so it is a good method

Interviewer: What else did you like about this method?

Respondent: What else?

Interviewer: Yes. If any.

Respondent: I do not experience any side effect every time I get injected

Interviewer: For how long have you used this method?

Respondent: Almost a year now

Interviewer: A year?

Respondent: Mmmmh (agreeing)

Title: ZA_IM_4.docx

Descriptor Info:	District: Zomba	Target Population: TP2 - DMPA-IM/DMPA-SC PI clients	Respondent type: DMPA-IM client	Aware of SI: Yes - had heard of SI before	Coding phase: 1. Single coded	Client marital status: Married	Client age group: Under 20 (adolescent)	Time since trained: N/A - client

Codes Applied:	C0.3 - Client previous use/experience of any FP method

Interviewer: Alright thank you so I want to talk to you about the experiences with different types of family planning apart from the injection. Have ever used any other type of family planning back then?

Respondent: Mmmh injection only

Interviewer: Injection only? Where do you get this kind of contraceptive?

Respondent: Here at the hospital

Interviewer: Alright how important is this method of family planning

Respondent: It is easier and not very difficult

Interviewer: Mmmh it is easier in what way?

Respondent: After you get an injection

Interviewer: Mhhh

Respondent: Then you are done

Interviewer: Mhhh alright thank you so much, are there any effects?

Respondent: Mhh you miss your periods

Interviewer: Mmmmmmh alright how old were you when you started using this kind of family planning method?

Respondent: 17

Title: MZs_IM_7.docx

Descriptor Info:	District: Mzimba South	Target Population: TP2 - DMPA-IM/DMPA-SC PI clients	Respondent type: DMPA-IM client	Aware of SI: Yes - had heard of SI before	Coding phase: 1. Single coded	Client marital status: Divorced/ separated/ widowed	Client age group: Under 20 (adolescent)	Time since trained: N/A - client

Codes Applied:	C0.3 - Client previous use/experience of any FP method

Interviewer: now I would like to ask about your experiences with the family planning method you are using. Apart from Depo IM, have you ever used another method?

Respondent: no

Interviewer: no?

Respondent: no

Interviewer: what have you experienced with Depo IM?

Respondent: sometimes I have shorter menses but sometimes longer periods

Interviewer: how old were you when you started using Depo?

Respondent: I was 18 years old

Title: MZs_IM_7.docx

Descriptor Info:	District: Mzimba South	Target Population: TP2 - DMPA-IM/DMPA-SC PI clients	Respondent type: DMPA-IM client	Aware of SI: Yes - had heard of SI before	Coding phase: 1. Single coded	Client marital status: Divorced/ separated/ widowed	Client age group: Under 20 (adolescent)	Time since trained: N/A - client

Codes Applied:	C0.3 - Client previous use/experience of any FP method

Interviewer: what made you start using family planning methods?

Respondent: I had delivered my child, so I wanted to do child spacing

Interviewer: what did you like about Depo?

Respondent: it’s easy to follow dates for subsequent doses. That’s why I liked this method

Interviewer: what else?

Respondent: I saw that this method is easy compared to pills and other methods

Interviewer: anything else?

Respondent: yes

Interviewer: would you tell me?

Respondent: It prevents pregnancy

Interviewer: any challenges with Depo?

Respondent: the main challenge I saw was I had longer periods, up to 2 weeks

Interviewer: mmmh

Respondent: that’s the only problem I saw

Interviewer: how long did you use Depo?

Respondent 6 months

Interviewer: how did you find Depo?

Respondent: as I have already said I came to the provider

Title: KK_IM_7.docx

Descriptor Info:	District: Nkhotakota	Target Population: TP2 - DMPA-IM/DMPA-SC PI clients	Respondent type: DMPA-IM client	Aware of SI: Yes - had heard of SI before	Coding phase: 1. Single coded	Client marital status: Married	Client age group: 30-39 years	Time since trained: N/A - client

Codes Applied:	C0.3 - Client previous use/experience of any FP method

INTERVIEWER: What do they say about these contraceptions?

RESPONDENT: The methods are different…if your view of the method is positive, you will find them good and vice versa. The bad thing about this is that some say…a method where when using it, some they have fast heartbeat, some they even faint. But for me, since I’ve been using this method, I have no side effects because back then when I had my 8-year-old child, I was using Norplant.

INTERVIEWER: okay.

RESPONDENT: Yes, when I got that contraception, I started fainting. I was just fainting, and I went to the hospital to be removed. For my 5-year-old, I was using an injection and I was fine, and I am using the same injection for my 2-year-old and everything is perfect.

INTERVIEWER: okay.

RESPONDENT: yes.

INTERVIEWER: When you went to the hospital, they told you that you were fainting because of Norplant?

RESPONDENT: Yea. They said that I was not supposed to have the implant because it was causing high blood pressure.

INTERVIEWER: mmmhu.

RESPONDENT: sure

Title: KK_IM_7.docx

Descriptor Info:	District: Nkhotakota	Target Population: TP2 - DMPA-IM/DMPA-SC PI clients	Respondent type: DMPA-IM client	Aware of SI: Yes - had heard of SI before	Coding phase: 1. Single coded	Client marital status: Married	Client age group: 30-39 years	Time since trained: N/A - client

Codes Applied:	C0.3 - Client previous use/experience of any FP method

INTERVIEWER: Alright. I would like to talk to you about your experiences with contraceptive methods.

RESPONDENT: mmmh

INTERVIEWER: Other than injectables, have you used other contraception methods in the past?

RESPONDENT: mmmh mmmh (no)

INTERVIEWER: Apart from the injectable mmmmh…

RESPONDENT: I have never used any other method. That’s the only one.

INTERVIEWER: The injectable

RESPONDENT: Yes.

INTERVIEWER: But you have told me about…Norplant

RESPONDENT: Oooh Norplant, that was a long time ago with my 8-year-old child. I used Norplant.

INTERVIEWER: Okay. Which mean you have only used Norplant and an injectable?

RESPONDENT: Yes.

INTERVIEWER: Mmmhu. What was your experience with this method?

RESPONDENT: When it was implanted, the first thing I noticed was fast heartbeat, when I stood up, I was feeling dizzy. I went to the hospital; they gave me medication but there was no change. One day I fainted, and the doctors said it was because of Norplant and they removed it.

INTERVIEWER: okay.

Title: KK_IM_7.docx

Descriptor Info:	District: Nkhotakota	Target Population: TP2 - DMPA-IM/DMPA-SC PI clients	Respondent type: DMPA-IM client	Aware of SI: Yes - had heard of SI before	Coding phase: 1. Single coded	Client marital status: Married	Client age group: 30-39 years	Time since trained: N/A - client

Codes Applied:	C0.3 - Client previous use/experience of any FP method

INTERVIEWER: So, what was it that drew you to using injectable conception?

RESPONDENT: Mmmh, it is a choice that I made, and I felt that it was a very good choice.

INTERVIEWER: Alright. What was it really that drew you to using the injectable?

RESPONDENT: For me to choose an injectable… as I have already said, I made this choice because Norplant was affecting me, and I thought of trying the injectable so that when it also affects me, I will try other methods because we have many contraception methods. And this one is good up to now.

INTERVIEWER: Mmmmh. So what do you like about injectables?

RESPONDENT: Aaah I like injectables because as I have already said, I don’t experience any side effects.

INTERVIEWER: mmmhu.

RESPONDENT: yea

INTERVIEWER: What is good about it?

RESPONDENT: I have no pain or what people say about menstruation, I menstruate normally. The injectable is good because my heartrate is normal than before.

INTERVIEWER: Okay. What do you not like about injectables?

RESPONDENT: Aaaah mmmm mmmm [no], there is nothing.

INTERVIEWER: All good?

RESPONDENT: To say the truth, all is well.

INTERVIEWER: Mmmmhu. For how long have you been using injectables?

RESPONDENT: Mmmmmmmh, for one year.

INTERVIEWER: One year?

RESPONDENT: yes, one year…

INTERVIEWER: You said you started using when your first born was how old?

RESPONDENT: He/she is 8 years old.

INTERVIEWER: You were using Norplant?

RESPONDENT: Yes.

INTERVIEWER: The second born?

RESPONDENT: He/she is 5 years old.

INTERVIEWER: What method were you using after the birth of this child?

RESPONDENT: The injectable.

INTERVIEWER: And this one, the same injectable.

RESPONDENT: The same. That is when my husband said I should continue with the same one.

Title: ZA_IM_5.docx

Descriptor Info:	District: Zomba	Target Population: TP2 - DMPA-IM/DMPA-SC PI clients	Respondent type: DMPA-IM client	Aware of SI: Yes - had heard of SI before	Coding phase: 2. Double coded	Client marital status: Married	Client age group: 30-39 years	Time since trained: N/A - client

Codes Applied:	C0.3 - Client previous use/experience of any FP method

nterviewer: Alright, Now I want to talk to you about your experiences with contraceptives. Ah other than injectables, have you used other contraceptive methods in the past?

Respondent: Yes, for me, at the very beginning I was using Depo.

Interviewer: Mmmm (showing that one is following the conversation)

Respondent: After some years, after my second child

Interviewer: Mmmm (showing that one is following the conversation)

Respondent: I used Depo

Interviewer: Mmmm (showing that one is following the conversation)

Respondent: But with Depo I was having excessive bleeding.

Interviewer: Mmmm (showing that one is following the conversation)

Respondent: 18 bleeding from the 1st of the month even up to the 18th

Interviewer: Mmmm (showing that one is following the conversation)

Respondent: Sometimes even the 20th

Interviewer: Mmmm (showing that one is following the conversation)

Respondent: I would go to the hospital to seek help

Interviewer: Mmmm (showing that one is following the conversation)

Respondent: I would take the medication, sometimes it would work

Interviewer: Mmmm (showing that one is following the conversation)

Respondent: and then it would resume the next month.

Interviewer: Mmmm (showing that one is following the conversation)

Respondent: I would take the medication, but it would not change.

Interviewer: Mmmm (showing that one is following the conversation)

Respondent: So I changed, I accepted that it’s not working for me. So I changed and started using the pills

Interviewer: Mmmm (showing that one is following the conversation)

Respondent: For the pills I was feeling just okay.

Interviewer: Mmmm (showing that one is following the conversation)

Respondent: But the problem that I had was consistent headaches.

Interviewer: Mmmm (showing that one is following the conversation)

Respondent: A headache that robbed my peace of mind.

Interviewer: Mmmm (showing that one is following the conversation)

Respondent: Everyday, so I stopped and had my next child, the last born of 3 years old.

Interviewer: Yah

Respondent: After I delivered that child, I started this method

Interviewer: Eh

Respondent: So I can see that now I don’t have any problem, no bleeding I just move around.

Interviewer: Alright

Respondent: Yes

Title: ZA_IM_5.docx

Descriptor Info:	District: Zomba	Target Population: TP2 - DMPA-IM/DMPA-SC PI clients	Respondent type: DMPA-IM client	Aware of SI: Yes - had heard of SI before	Coding phase: 2. Double coded	Client marital status: Married	Client age group: 30-39 years	Time since trained: N/A - client

Codes Applied:	C0.3 - Client previous use/experience of any FP method

Interviewer: Alright, ah where were you accessing the pills, from the facility?

Respondent: Yes, I was coming to get it from here.

Interviewer: Alright, are there any other problems in addition to the headache as you were using the pills?

Respondent: Yes, I was losing weight.

Interviewer: Okay

Respondent: Eh

Interviewer: Alright, can you tell me the good things that you experienced using the pills?

Respondent: Ah with pills the good thing was…

Interviewer: Mmmm (showing that one is following the conversation)

Respondent: The only problem was the headache, when I took the pills and am taking the last ones and am bleeding

Interviewer: Mmmm (showing that one is following the conversation)

Respondent: I was feeling my body become lighter

Interviewer: Alright

Respondent: That’s the good thing I experienced with pills.

Interviewer: Alright, are there any other factors that made you stop using pills and start using the injectable method?

Respondent: Ah the reason is that I just decided…

Interviewer: Mmmm (showing that one is following the conversation)

Respondent: I just stopped, in the middle of the usage of pills I just stopped, I would miss I don’t know what was happening.

Interviewer: Mmmm (showing that one is following the conversation)

Respondent: I could not feel my body the way I used to feel it, I came to the facility three times.

Interviewer: Mmmm (showing that one is following the conversation)

Respondent: So they told me to stop using the method

Interviewer: Mmmm (showing that one is following the conversation)

Respondent: When you stop you should wait for a few days and then come to the facility so that we can examine you, When I came back to the hospital, they examined me, and they found that I was pregnant, and they told me to completely stop using the pills.

Interviewer: Alright.

Respondent: When I stopped, I saw that this is very risky I would better go back to my old method of Depo, so now my body is used to Depo now.

Title: ZA_IM_5.docx

Descriptor Info:	District: Zomba	Target Population: TP2 - DMPA-IM/DMPA-SC PI clients	Respondent type: DMPA-IM client	Aware of SI: Yes - had heard of SI before	Coding phase: 2. Double coded	Client marital status: Married	Client age group: 30-39 years	Time since trained: N/A - client

Codes Applied:	C0.3 - Client previous use/experience of any FP method

Interviewer: Alright, what motivated you to start using injectable contraceptives?

Respondent: What motivated me to start using injectables...

Interviewer: Mmmm (showing that one is following the conversation)

Respondent: When I come here at the facility and the providers have assisted us…

Interviewer: Mmmm (showing that one is following the conversation)

Respondent: This month and then we have to come after three months, so we see that at least different from the other methods like pills, because for the pills you have to take them daily.

Interviewer: Mmmm (showing that one is following the conversation)

Respondent: Eh

Interviewer: Alright what do you like about injectable contraceptives?

Respondent: About contraceptive injectables?

Interviewer: Yes

Respondent: (silence)

Interviewer: What do you like about contraceptive injectables?

Respondent: I like injectable contraceptive because when I get the injection now…

Interviewer: Mmmm (showing that one is following the conversation)

Respondent: I am able to move around and at home we are able to live just okay without fear, sleep with my husband without fear of getting pregnant.

Interviewer: Alright, is there anything that you don’t like about injectables?

Respondent: Ah no

Interviewer: Alright, for how long have you been using injectable contraceptives?

Respondent: Eh I have used for some years now.

Interviewer: Mmmm (showing that one is following the conversation)

Respondent: Eh (thinks deeply) nine, ten years? (asking themselves) 11 years.

Interviewer: 11 years?

Respondent: Yes.

Interviewer: Alright

(RECORDING STOPS BECAUSE SOMEONE KNOCKS AT THE DOOR)

TIME INTO THE INTERVIEW: 13Mins

Interviewer: Sorry, you told me that you have used this method for 11 years

Respondent: Yes

Title: ZA_IM_9.docx

Descriptor Info:	District: Zomba	Target Population: TP2 - DMPA-IM/DMPA-SC PI clients	Respondent type: DMPA-IM client	Aware of SI: Yes - had heard of SI before	Coding phase: 1. Single coded	Client marital status: Married	Client age group: 20-29 years	Time since trained: N/A - client

Codes Applied:	A2.1 - Adol pros/cons of injectables	C0.3 - Client previous use/experience of any FP method

Interviewer: Alright. Was it hard or not to find contraception methods before you reached the age of 18?

Respondent: Before I reached 18 it was hard because I had not yet given birth.

Interviewer: Mmh.

Respondent: Right now after I gave birth, I have seen that I can go and get contraceptives so that I cannot constantly get pregnant.

Interviewer: Alright. When you first got your injection contraception at the age of 18, did you face any problems?

Respondent: Yes, I could menstruate constantly, after I came and told the doctor they gave me medicine and it stopped. And after I continued with the contraception, it stopped.

Title: ZA_IM_9.docx

Descriptor Info:	District: Zomba	Target Population: TP2 - DMPA-IM/DMPA-SC PI clients	Respondent type: DMPA-IM client	Aware of SI: Yes - had heard of SI before	Coding phase: 1. Single coded	Client marital status: Married	Client age group: 20-29 years	Time since trained: N/A - client

Codes Applied:	C0.3 - Client previous use/experience of any FP method	C0.2.1 - Positive community/client perspectives on FP

Interviewer: Alright, thank you so much. What encouraged you to start using the injection contraception method?

Respondent: Becau- hmm (Smiles) Because of,

Interviewer: Mmh. Because of what?

Respondent: Because I do not want to usually give birth and to also work without thinking I have a small child and also walk without thinking I have a baby.

Interviewer: Alright, thank you so much. So what were you interested with in the injection contraception method?

Respondent: I liked the injection contraception because when you get the injection right?

Interviewer: Mmh.

Respondent: You do not face any problems.

Interviewer: Mmh.

Respondent: Mmh.

Interviewer: Mmhhm. What, what don’t you like about the injection? What doesn’t interest you with the injection contraception method?

Respondent: Nothing. Nothing doesn’t interest me.

Interviewer: You said you used to menstruate twice.

Respondent: Mmh. As of now iii, it stopped.

Interviewer: You don’t face any problems?

Respondent: I do not face any problems.

Interviewer: Mmh.

Respondent: Mmh.

Interviewer: Alright alright, thank you so much. Just to assume, for how long have you been using this injection?

Respondent: Yes, I have been using for a long time.

Interviewer: Mmh.

Respondent: A long time, right?

Interviewer: Just to assume, how many years?

Respondent: Year? As of now it’s been almost 5 years, right?

Interviewer: Mmh.

Respondent: I have been using the injection contraception.

Interviewer: But you explained that you started using after your child was born, your first child?

Respondent: Mmh.

Interviewer: First born?

Respondent: first born; I used a few injections, a few years, right?

Interviewer: Mmh.

Respondent: Then I got pregnant again.

Interviewer: Mmh.

Respondent: And now am still getting injected.

Interviewer: Alright, how old is your first child?

Respondent: 7 years old.

Interviewer: so we should say you have been using for 7 years?

Respondent: Mmh.

Title: MZs_IM_1.docx

Descriptor Info:	District: Mzimba South	Target Population: TP2 - DMPA-IM/DMPA-SC PI clients	Respondent type: DMPA-IM client	Aware of SI: Yes - had heard of SI before	Coding phase: 1. Single coded	Client marital status: Married	Client age group: 30-39 years	Time since trained: N/A - client

Codes Applied:	C0.3 - Client previous use/experience of any FP method	C2.1.1 - Community availability of injectables/SI

Q5. Interviewer: Other than injectables, have you used any other family planning method in the past:

Respondent: No, I started with injectables, and I have never switched to another method because I have never experienced any challenge since I started.

Interviewer: Where do you receive it?

Respondent: Most of the time I receive it from the HSA who is in our area,

Interviewer: Tell me the good things about this method?

Respondent: The good thing about this method is that where we get the method is not far from where we stay and the HSA always visit us whenever they have promised to do so.

Interviewer: What about the bad things about the method?

Respondent: I have never experienced anything bad, that is why since I started I have never changed the method.

Title: MZs_IM_1.docx

Descriptor Info:	District: Mzimba South	Target Population: TP2 - DMPA-IM/DMPA-SC PI clients	Respondent type: DMPA-IM client	Aware of SI: Yes - had heard of SI before	Coding phase: 1. Single coded	Client marital status: Married	Client age group: 30-39 years	Time since trained: N/A - client

Codes Applied:	C0.3 - Client previous use/experience of any FP method	C8.1 - Male involvement, general	C0.2.1 - Positive community/client perspectives on FP

Q7. Interviewer: What was it that first drew you into using injectable contraception?

Respondent: In my marriage whenever we have a child we always fight (me and my husband), so it was this other day when I came to scale with my child I heard that there is family planning methods, then I said to myself let me get the method so that may I get out from that man`s bondage thus when I started using the family planning method, and later I find out that all the fight we used to have are gone.

Interviewer: What do you like about the method?

Respondent: The only thing I like about the method is that once the provider injects me, I stay at home for 3 months without visiting the facility.

Interviewer: Is there anything that you don’t like about the injectables?

Respondent: There is nothing that I don’t like about the injectables.

Interviewer: For how long have you been using this method?

Respondent: Since I started, I have never changed, and I now have 4 children and its now18 years since I started.

Title: KK_IM_1.docx

Descriptor Info:	District: Nkhotakota	Target Population: TP2 - DMPA-IM/DMPA-SC PI clients	Respondent type: DMPA-IM client	Aware of SI: No - never heard of SI before	Coding phase: 3. Triple coded	Client marital status: Married	Client age group: Under 20 (adolescent)	Time since trained: N/A - client

Codes Applied:	C0.3 - Client previous use/experience of any FP method

INTERVIEWER: alright fine, so now I would like to talk to about your experiences with contraceptive methods. What are your experiences in using contraceptives? Other than injectables have you used other family planning methods in the past? Tell me about your experiences with these methods.

RESPONDENT: I have never used any other family planning methods I have just started using contraceptives. I just started with injectable.

(10 minutes and 2 seconds)

INTERVIEWER: Okay you never used any other family planning method since you have just started

RESPONDENT: Yes

INTERVIEWER: You have just used this injectable method so far?

RESPONDENT: Yes

INTERVIEWER: You mean Depo not so?

RESPONDENT: Yes

INTERVIEWER: What were the pros and cons of the methods used

RESPONDENT: I have not experienced anything so far

INTERVIEWER: Since they are talking about other methods hence, there is nothing for you to comment as you have said earlier.

RESPONDENT: Yes

Title: KK_IM_1.docx

Descriptor Info:	District: Nkhotakota	Target Population: TP2 - DMPA-IM/DMPA-SC PI clients	Respondent type: DMPA-IM client	Aware of SI: No - never heard of SI before	Coding phase: 3. Triple coded	Client marital status: Married	Client age group: Under 20 (adolescent)	Time since trained: N/A - client

Codes Applied:	C0.3 - Client previous use/experience of any FP method

INTERVIEWER: Aright, but how do you know that, that’s the one that fits you well, yet you have not tried other methods before (interviewer laughing). Okay you felt that’s the right one for you?

RESPONDENT: Yes

INTERVIEWER: How long have you been using injectables?

RESPONDENT: I just started last month

INTERVIEWER: You just started last month after having this child?

RESPONDENT: Yes

Title: ZA_SCPI_8.docx

Descriptor Info:	District: Zomba	Target Population: TP2 - DMPA-IM/DMPA-SC PI clients	Respondent type: DMPA-SC PI client	Aware of SI: Yes - had heard of SI before	Coding phase: 1. Single coded	Client marital status:	Client age group:	Time since trained: N/A - client

Codes Applied:	C0.3 - Client previous use/experience of any FP method

Interviewer: alright, now I want to talk to you about your experiences in using family planning methods. Other than injectables have you used other family planning methods in the past?

Respondent: yes, I have, I once had Norplant

Interviewer: mmmm (showing that one is following the conversation)

Respondent: the one for 5 years, but I was feeling sick with it, I would also feel dizzy

Interviewer: mmmm (showing that one is following the conversation)

Respondent: eh

Interviewer: alright.

Respondent: then I came to the hospital to complain

Interviewer: mmmm (showing that one is following the conversation)

Respondent: I told them the problem that I was having, so they removed it

Interviewer: okay

Respondent: after they removed it, I was out on injectables, and since then I have not had any problems.

Interviewer: alright.

Respondent: mmmm (showing that one is following the conversation)

Interviewer: did you access Norplant from the facility?

Respondent: yes

Interviewer: alright, any good things about this method?

Respondent: yes, but it just depends on one’s body type.

Interviewer: mmmm (showing that one is following the conversation)

Respondent: some women have it and don’t have any problems with it.

Interviewer: mmmm (showing that one is following the conversation)

Respondent: but some people will have it and face so many problems with it

Interviewer: alright.

Respondent: yes

Interviewer: were there any other problems apart from the ones that you mentioned?

Respondent: no

Title: ZA_SCPI_8.docx

Descriptor Info:	District: Zomba	Target Population: TP2 - DMPA-IM/DMPA-SC PI clients	Respondent type: DMPA-SC PI client	Aware of SI: Yes - had heard of SI before	Coding phase: 1. Single coded	Client marital status:	Client age group:	Time since trained: N/A - client

Codes Applied:	C0.3 - Client previous use/experience of any FP method

Interviewer: what motivated you to start using injectables?

Respondent: what motivated me

Interviewer: mmmm (showing that one is following the conversation)

Respondent: when I told my friends that Norplant was giving me problems

Interviewer: mmmm (showing that one is following the conversation)

Respondent: they told me that it depends on one’s blood type

Interviewer: mmmm (showing that one is following the conversation)

Respondent: some can be okay with it. So they helped me that I should come to the facility and get it removed, after that they can out me on another method of injectables.

Interviewer: mmmm (showing that one is following the conversation)

Respondent: yes

Interviewer: alright.

Respondent: so I tried injectables, and there were not any problems.

Interviewer: alright.

Respondent: yes

Interviewer: what do you like about the injectables that you use?

Respondent: I like that am planning my children’s future well,

Interviewer: mmmm (showing that one is following the conversation)

Respondent: yes

Interviewer: is there anything that you don’t like about injectables?

Respondent: I cannot know.

Interviewer: ok

Respondent: eh

Interviewer: for how long have you been using injectables?

Respondent: I have just started using this year.

Interviewer: ok

Respondent: eh

Title: ZA_IM_7.docx

Descriptor Info:	District: Zomba	Target Population: TP2 - DMPA-IM/DMPA-SC PI clients	Respondent type: DMPA-IM client	Aware of SI: No - never heard of SI before	Coding phase: 1. Single coded	Client marital status: Never married	Client age group: 20-29 years	Time since trained: N/A - client

Codes Applied:	C0.3 - Client previous use/experience of any FP method

Interviewer: Mmmmmh alright thank you. Now I want to talk to you about the challenges that you meet when using contraceptives. Apart from the injection method have you ever used any other methods before in the past?

Respondent: No only the injection method

Title: ZA_IM_7.docx

Descriptor Info:	District: Zomba	Target Population: TP2 - DMPA-IM/DMPA-SC PI clients	Respondent type: DMPA-IM client	Aware of SI: No - never heard of SI before	Coding phase: 1. Single coded	Client marital status: Never married	Client age group: 20-29 years	Time since trained: N/A - client

Codes Applied:	C0.3 - Client previous use/experience of any FP method

Interviewer: Thank you? What made you start using the injection method?

Respondent: I thought it wise to start using contraceptives since I have a small child.

Interviewer: Alright, what is it that you like about the injection method?

Respondent: I just feel like I like it and I differentiate it from the one you put on your arm.

Interviewer: Mmmmh

Respondent: A lot of women complain about the one you put on the arm so I feel like I would also face problems.

Interviewer: Have you ever used the one on the arm?

Respondent: No

Interviewer: But the first time I asked you what do women say about methods of contraception?

Respondent: I heard them here at the hospital.

Interviewer: Alright alright thank you, what is it that you don’t like about the injection method?

Respondent: Mmmmh nothing really the only thing I face with the injection method is the back pain. When the injection is overdue it’s when I feel pain.

Interviewer: Do you menstruate properly?

Respondent: No, because ever since I gave birth to my child I haven’t menstruated.

Interviewer: Mmmmmh

Respondent: When I first gave birth, I stayed for almost 4 months without menstruating.

Interviewer: So since you gave birth you haven’t menstruated?

Respondent: Yes

Interviewer: Alright, thank you. How long have you been using the injection method?

Respondent: Mmmmmh I can’t really tell but right now I haven’t received my number 7th injection.

Interviewer: Mmmmh you haven’t been injected the 7th injection?

Respondent: Yes

Interviewer: How many injections have you had?

Respondent: Mmmmh I have forgotten let me check in my book

Interviewer: Mmmmmh

Respondent: I started on the 30th of September 2021.

Interviewer: So if you started on the 30th it means you have been doing it for almost a year?

Respondent: Yes.

Title: MZs_SCPI_3.docx

Descriptor Info:	District: Mzimba South	Target Population: TP2 - DMPA-IM/DMPA-SC PI clients	Respondent type: DMPA-SC PI client	Aware of SI: Yes - had heard of SI before	Coding phase: 1. Single coded	Client marital status: Married	Client age group: 20-29 years	Time since trained: N/A - client

Codes Applied:	C0.3 - Client previous use/experience of any FP method

Q5. Interviewer: Other than injectables, have you used other family planning in the past?

Respondent: No

Interviewer: Did you start with injectables?

Respondent: Yes.

Q6. Interviewer: How easy or difficult have you found it accessing family planning in the past?

Respondent: It was easy

Interviewer: You have said it was easy, so what factors have made it easy?

Respondent: No, it was not easy.

Interviewer: What factors made it difficult?

Respondent: I was scared to come and access family planning and because I was afraid thus why am saying it was difficult.

Interviewer: Why were you afraid?

Respondent: I was thinking that may be method will not be good on me thus why I was afraid.

Interviewer: So do you still have that fear?

Respondent: No

Interviewer: Why don’t you have the fear anymore?

Respondent: After seeing friends doing it, then decided to go to the facility and start the method and it’s a good one.

Interviewer: What are the good thing about the method?

Respondent: The good thing is that as of now am able to have child spacing .

Interviewer: Anything else?

Respondent: [Quiet….]

Interviewer: If there is?

Respondent: Nothing else.

Title: MZs_SCPI_3.docx

Descriptor Info:	District: Mzimba South	Target Population: TP2 - DMPA-IM/DMPA-SC PI clients	Respondent type: DMPA-SC PI client	Aware of SI: Yes - had heard of SI before	Coding phase: 1. Single coded	Client marital status: Married	Client age group: 20-29 years	Time since trained: N/A - client

Codes Applied:	C0.3 - Client previous use/experience of any FP method

Q7. Interviewer: What was it that drew you to using injectable contraception?

Respondent: I wanted to have child spacing.

Interviewer: Anything else?

Respondent: No

Interviewer: What do you like about injectables?

Respondent: The good thing is that am able to have child spacing.

Interviewer: Is that all?

Respondent: Yes.

Interviewer: For how long have you been using injectables?

Respondent: It’s a year now

Interviewer: If you say it’s a year now, when did you start?

Respondent: I started last year but I can’t remember the month.

Title: MZs_IM_4.docx

Descriptor Info:	District: Mzimba South	Target Population: TP2 - DMPA-IM/DMPA-SC PI clients	Respondent type: DMPA-IM client	Aware of SI: Yes - had heard of SI before	Coding phase: 2. Double coded	Client marital status: Married	Client age group: 30-39 years	Time since trained: N/A - client

Codes Applied:	C0.3 - Client previous use/experience of any FP method

Q5. Interviewer: I would like now to talk to you about your experiences with contraceptive methods Other than injectables have you used other family planning/contraceptives in the past?

Respondent: My first family planning method was pills but because I was heavily bleeding, I switched to injectables and with injectables I was also breeding too much then I went to the provider, and he gave me some medicine and after taking those medicine I stopped bleeding too much then I went back to the provider, and he said that everything will be okay and after some time the body will start responding to the medicine

Interviewer: You have said that at first the bleeding was too much so tell me what method where you using?

Respondent: I was using injectables

Interviewer: Where you using self – injection or providers injection?

Respondent: It was provider’s injection

Interviewer: Where did you receive these injectables, from the hospital or HSA?

Respondent: It was from the hospital

Interviewer: What are the advantages of the injectables?

Respondent: The good thing about the injectables it is reliable you can have sex with your husband anytime you want.

Interviewer: What are the disadvantages of injectables?

Respondent: The bad thing is that the bleeding was too much

Title: MZs_IM_4.docx

Descriptor Info:	District: Mzimba South	Target Population: TP2 - DMPA-IM/DMPA-SC PI clients	Respondent type: DMPA-IM client	Aware of SI: Yes - had heard of SI before	Coding phase: 2. Double coded	Client marital status: Married	Client age group: 30-39 years	Time since trained: N/A - client

Codes Applied:	C0.2.1 - Positive community/client perspectives on FP	C0.3 - Client previous use/experience of any FP method

Interviewer: Mmmmh. Q7. Interviewer: What was it first that drew you to use injectable contraception?

Respondent: After I gave birth to my two children it’s when I realize that I need to give space to my children so that they should grow

Interviewer: You said that the time you where using pills you had heavy bleeding was this the reason why you switched from pills to injectables

Respondent: Yes

Interviewer: Mmmh (following)

Respondent: Sure

Interviewer: What do you like about injectables?

Respondent: Injectables makes ovulation weak that you cannot get pregnant

Interviewer: So before you proceed, I want you to tell me about injectables not as a volunteer but someone who uses it.

Respondent: The good thing about this method is that we can stay together with my husband anytime we want

Interviewer: Tell me its goodness?

Respondent: The good thing about this method is that when I want to do menstruation I do it without any problem

Interviewer: Do you have anything to add

Respondent: No, there is nothing to add

Title: MZs_IM_4.docx

Descriptor Info:	District: Mzimba South	Target Population: TP2 - DMPA-IM/DMPA-SC PI clients	Respondent type: DMPA-IM client	Aware of SI: Yes - had heard of SI before	Coding phase: 2. Double coded	Client marital status: Married	Client age group: 30-39 years	Time since trained: N/A - client

Codes Applied:	C0.3 - Client previous use/experience of any FP method

Interviewer: Now tell me what you dislike about this method?

Respondent: What I dislike about this method is that sometimes it makes me to relish as if I am pregnant while am just okay the other thing is that sometimes I feel like vomiting some people also do think that am pregnant while not.

Interviewer: How long have you been using injectables?

Respondent: It has been 4 years since I started the injectables

Interviewer: 4 years using injectables

Respondent: Yes

Title: KK_IM_2.docx

Descriptor Info:	District: Nkhotakota	Target Population: TP2 - DMPA-IM/DMPA-SC PI clients	Respondent type: DMPA-IM client	Aware of SI: Yes - had heard of SI before	Coding phase: 2. Double coded	Client marital status: Married	Client age group: 30-39 years	Time since trained: N/A - client

Codes Applied:	C0.3 - Client previous use/experience of any FP method

INTERVIEWER: Now we want to know more about what you experience. Now, apart from the injection method, what other contraceptive methods have you used previously?

RESPONDENT: No, just the injection one.

INTERVIEWER: Just the injection?

RESPONDENT: mmmh.

INTERVIEWER: Since you started.

RESPONDENT: Since I started, after giving birth to my first child, my husband would not allow me to go for contraceptions. Therefore, I was going by myself.

INTERVIEWER: mmmh.

RESPONDENT: I was going for an injection. I had 5 injections, but I had a child while the other one was a year and two months old that is when I said I should rest because I was having heavy menstruations. I said may be if I take a break I will rest from the heavy menstruations as well that is when I will go again for an injection. So the same month I stopped menstruating that is the month I found myself pregnant. After some time, I gave birth to my second child. After giving birth to my second child, I used this same contraceptive method, I did not change.

INTERVIEWER: The same injection…Depo?

RESPONDENT: Yes, the same Injection. I injected for a while. I injected up to 15 of them. I had another baby after the child was three years old. Mmmhu, now, for the third one, my husband had first gone away.

INTERVIEWER: okay.

RESPONDENT: When he got back from where he went, I started using contraceptives again. That is the one who took eight years to be born.

INTERVIEWER: owo, okay.

RESPONDENT: Sure.

Title: KK_IM_2.docx

Descriptor Info:	District: Nkhotakota	Target Population: TP2 - DMPA-IM/DMPA-SC PI clients	Respondent type: DMPA-IM client	Aware of SI: Yes - had heard of SI before	Coding phase: 2. Double coded	Client marital status: Married	Client age group: 30-39 years	Time since trained: N/A - client

Codes Applied:	C0.3 - Client previous use/experience of any FP method	C0.2 - Community and client perspectives on FP - general

INTERVIEWER: okay. there are several contraceptive methods right. You know them, right? May be there is loop, there are tablets right. What what made you to choose injection and leave all of them?

RESPONDENT: Injection is the method that is compatible with my body.

INTERVIEWER: Is compatible with your body? Have you tried other methods?

RESPONDENT: Yes, I tried tablets, but it did not help me.

INTERVIEWER: I did ask you that before you started whatchamacallit …

RESPONDENT: I have taken tablets before. What happened is that, my husband went to South Africa, and he came and I took the tablets. After taking the tablets, I could not see whatchamacallit …enough encouragement. I just stopped after taking them once and I reverted to my usual method.

INTERVIEWER: okay?

RESPONDENT: mmmmh

INTERVIEWER: What happened for you to stop using whatchamacallit…

RESPONDENT: I was not menstruating

INTERVIEWER: The tablets?

RESPONDENT: mmmmh.

INTERVIEWER: taking them well enough…

RESPONDENT: Yes, I had taken them, but I was not menstruating… I took three months without menstruating

INTERVIEWER: Okay. So, you reverted.

RESPONDENT: After that, I doubted myself, I thought I had gotten pregnant and went to the hospital where my they tested my urine, but it came out negative.

INTERVIEWER: okay. now you are back to Whatchamacallit?

RESPONDENT: Yes

INTERVIEWER: To the injection method.

RESPONDENT: Yes

INTERVIEWER: Now you …I want to know what made you to choose Injection over the pills

RESPONDENT: Whenever I used injection, I was able to menstruate.

INTERVIEWER: okay.

RESPONDENT: mmmmh. When I get the injection, I menstruate for a week, sometimes two weeks or sometimes I menstruate for my normal menstruating days, which are five days.

INTERVIEWER: okay.

RESPONDENT: mmmmh.

INTERVIEWER: Everything has its good and bad sides, most things. What do you think is the bad side of injection?

RESPONDENT: Sexual desire.

INTERVIEWER: Sexual desire… can you explain further.

RESPONDENT: (laughs) When I am injected, I am never interested in a man, the sexual feelings do not come.

INTERVIEWER: completely gone?

RESPONDENT: Mmmh, even for a man to sleep with me, I just do it because it is a family, just to help my husband (laughs). However, the time I took tablets I was having my normal sexual feelings.

INTERVIEWER: okay.

RESPONDENT: Yes, but the injection I do not get sexual feelings.

INTERVIEWER: You mean as of now there is nothing?

RESPONDENT: Yes, once am injected the sexual desire is all gone. The moment I get injected the desire to sleep with a man is all gone.

INTERVIEWER: mmmhu.

RESPONDENT: yes.

Title: KK_IM_8.docx

Descriptor Info:	District: Nkhotakota	Target Population: TP2 - DMPA-IM/DMPA-SC PI clients	Respondent type: DMPA-IM client	Aware of SI: No - never heard of SI before	Coding phase: 1. Single coded	Client marital status: Married	Client age group: 20-29 years	Time since trained: N/A - client

Codes Applied:	C0.3 - Client previous use/experience of any FP method	C8.1 - Male involvement, general	C8.1.1 - Decision making with partner

INTERVIEWER: I would like to discuss about your experience in using contraceptive methods.

RESPONDENT: yes.

INTERVIEWER: Apart from injectable, Depo right, have you used other contraceptives in the past?

RESPONDENT: yes.

INTERVIEWER: what methods have you used?

RESPONDENT: Norplant.

INTERVIEWER: Norplant. Norplant is this?

RESPONDENT: they implant at the hospital

Time stamp: (10:03).

INTERVIEWER: Okay. What were your experiences with this method?

RESPONDENT: What I experiences was…I stayed with the implant for one year and 9 months. I was not menstruating. On the 9 month began menstruating from the 1st of the month to the 30th. Then I went to the hospital, and they gave me medication. And after the dose, the period would start again. and I was getting worse because I was now looking pale, my feet were hot and my heard was also hot. That is when I went to the hospital for the Norplant to be removed.

INTERVIEWER: When was the Norplant implanted?

RESPONDENT: 2017.

INTERVIEWER: 2017?

RESPONDENT: yes.

INTERVIEWER: okay. You were implanted at the hospital or from an HSA.

RESPONDENT: it was the hospital

INTERVIEWER: ok, the same hospital

RESPONDENT: yes, the same Benga by Banja la Mtsogolo.

INTERVIEWER: oh, so its Benga (laughs).

RESPONDENT: yes.

INTERVIEWER: not Benga right?

RESPONDENT: no Benga (laughs).

INTERVIEWER: okay. Mmmh you have explained what you experienced when you were using this method right?

RESPONDENT: yes.

INTERVIEWER: Apart from this, did you experience any bad things?

RESPONDENT: no. those are the only ones

INTERVIEWER: it’s only those?

RESPONDENT: mmmh.

INTERVIEWER: What was the good part of using Norplant?

RESPONDENT: I can say that the first part it was good because I was ok. That was the good part in the first part.

INTERVIEWER: mmmhu.

RESPONDENT: yes.

INTERVIEWER: when you say you were okay what do you mean?

RESPONDENT: I was not sick. I was doing my chores without any pain.

INTERVIEWER: okay.

RESPONDENT: sure.

INTERVIEWER: For how long were you sick?

RESPONDENT: for two months.

INTERVIEWER: feeling like that?

RESPONDENT: yes.

INTERVIEWER: then you went to the hospital?

RESPONDENT: yes.

INTERVIEWER: Was the decision to be removed yours or you were told?

RESPONDENT: We agreed because when I was going for this method, we also agreed. When we faced this problem, my husband said that the Norplant should be removed because I might end up losing a lot of blood.

INTERVIEWER: mmmhu.

RESPONDENT: yes.

Title: KK_IM_8.docx

Descriptor Info:	District: Nkhotakota	Target Population: TP2 - DMPA-IM/DMPA-SC PI clients	Respondent type: DMPA-IM client	Aware of SI: No - never heard of SI before	Coding phase: 1. Single coded	Client marital status: Married	Client age group: 20-29 years	Time since trained: N/A - client

Codes Applied:	C0.3 - Client previous use/experience of any FP method	C0.2 - Community and client perspectives on FP - general

INTERVIEWER: Okay. What drew you exactly to using injectable contraception?

RESPONDENT: What drew me I can say that its motherhood. I can say that women have different bodies. Some when they give birth, they can stay up to a year and some months without menstruating while for me I can give birth this month and the next month, I menstruate. So our parents in the community say that for that who menstruates immediately, she can get pregnant easily. So to avoid this, I opted for an injectable.

INTERVIEWER: okay.

RESPONDENT: yes.

INTERVIEWER: So there are different methods; there is loop, condoms etc. why were you interested in an injectable?

RESPONDENT: I do not face any side effects when using this method.

INTERVIEWER: okay.

RESPONDENT: sure.

INTERVIEWER: what side effects does an injection bring about?

RESPONDENT: I can say that I have not faced any problem. Like it used to happen with Norplant for example getting sick and menstruation. I menstruate the way I used to when I had just started my period. And that is how I am menstruating up to now.

INTERVIEWER: So you like the injectable because of what you have said?

RESPONDENT: yes.

INTERVIEWER: But it might happen that you don’t like it because everything has a positive and negative side

RESPONDENT: yes.

INTERVIEWER: What don’t you like about an injection?

RESPONDENT: aaah…(laughs).

INTERVIEWER: everything has good and bad...

RESPONDENT: that is true.

INTERVIEWER: right?

RESPONDENT: What I do not like about this method is that it reduces my sexual feeling to be with a man. It is what I do not like about the injection.

INTERVIEWER: this happens to you?

RESPONDENT: yes.

INTERVIEWER: Do the sexual feelings go when you have just injected…or the whole three months or it varies?

RESPONDENT: It changes.

INTERVIEWER: mmmhu.

RESPONDENT: mmmh

INTERVIEWER: explain to me.

RESPONDENT: (laughs) It happens at different times. When you have just been injected and then you go home and when the drugs have started working, that is when the sexual desire goes. And when the drug is welling off, that is when my sexual desire comes back.

INTERVIEWER: okay.

RESPONDENT: When the drug is welling off, that is when my body starts to have a sexual desire frequently.

INTERVIEWER: okay.

RESPONDENT: yes.

INTERVIEWER: For how long have you been using the injectable contraceptive?

RESPONDENT: I can say that for one year.

INTERVIEWER: one year?

RESPONDENT: yes.

INTERVIEWER: but you removed loop….

RESPONDENT: I was not on any method.

INTERVIEWER: oh ok. You were not using any method?

RESPONDENT: yes.

INTERVIEWER: ok. Then?

RESPONDENT: I got pregnant for this child.

INTERVIEWER: ok. And when he was born...

RESPONDENT: That is when I started using an injectable contraceptive.
