# Supplementary material for: Health care provider and client experiences of counselling on depot medroxyprogesterone acetate subcutaneous (DMPA-SC) for self-injection in Malawi
Source: PLOS Glob Public Health. 2023 Nov 30;3(11):e0002057. doi: 10.1371/journal.pgph.0002057 (PMC10688740; doi:10.1371/journal.pgph.0002057)
Supplement: S1 Data — (ZIP) [file pgph.0002057.s001.zip › S2/C0.3 client prev experience SI only.doc]

Title: KK_SCSI_5.docx

Descriptor Info:	District: Nkhotakota	Target Population: TP3 - DMPA-SC SI clients	Respondent type: DMPA-SC SI client	Aware of SI: Yes - had heard of SI before	Coding phase: 2. Double coded	Client marital status: Married	Client age group: 20-29 years	Time since trained: N/A - client

Codes Applied:	C0.3 - Client previous use/experience of any FP method

INTERVIEWER: Ok thank you. Alright, may we now go to another topic; now I would like to talk to you about your experience with contraceptive methods. On this topic we have few questions. The first question is other than injectables, have you used other family planning methods in the past?

RESPONDENT: Yes, firstly I started with Norplant, but with Norplant I was having prolonged period hence, I went to the nurse and told him that the type of contraceptive was not good. So I changed to Sayana.

INTERVIEWER: Ok

RESPONDENT: Yes, am now ok and I don’t have my periods if I do, they are very light not as heavy as used to do when I was taking Norplant. Yes

Title: KK_SCSI_5.docx

Descriptor Info:	District: Nkhotakota	Target Population: TP3 - DMPA-SC SI clients	Respondent type: DMPA-SC SI client	Aware of SI: Yes - had heard of SI before	Coding phase: 2. Double coded	Client marital status: Married	Client age group: 20-29 years	Time since trained: N/A - client

Codes Applied:	C0.3 - Client previous use/experience of any FP method

INTERVIEWER: What were the pros and cons of the method you used? If yes, what are these? I think you have already answered this question

RESPONDENT: Yes, I did answer it

INTERVIEWER: If stopped using the method, why did you stop? I think as for you, you didn’t stop.

RESPONDENT: Yes, I didn’t stop I just switched to a new one

Title: MZs_SCSI_3.docx

Descriptor Info:	District: Mzimba South	Target Population: TP3 - DMPA-SC SI clients	Respondent type: DMPA-SC SI client	Aware of SI: Yes - had heard of SI before	Coding phase: 1. Single coded	Client marital status: Married	Client age group: 30-39 years	Time since trained: N/A - client

Codes Applied:	C0.3 - Client previous use/experience of any FP method

Interviewer: now I would like to talk about what people in your community talk about on family planning. What do you and fellow women discuss on family planning methods?

Respondent: I was gaining weight; some women were saying that I will not conceive again. After first dose I had overstayed without conceiving so I was worried I went to see a health worker who reassured us that she will be fine.

Title: MZs_SCSI_3.docx

Descriptor Info:	District: Mzimba South	Target Population: TP3 - DMPA-SC SI clients	Respondent type: DMPA-SC SI client	Aware of SI: Yes - had heard of SI before	Coding phase: 1. Single coded	Client marital status: Married	Client age group: 30-39 years	Time since trained: N/A - client

Codes Applied:	C0.3 - Client previous use/experience of any FP method

Interviewer: do women talk about changes in their bodies due to family planning methods?

Respondent: I was one of them who had weight gain. I had increased appetite. Those who used Depo have increased appetite.

Title: MZs_SCSI_3.docx

Descriptor Info:	District: Mzimba South	Target Population: TP3 - DMPA-SC SI clients	Respondent type: DMPA-SC SI client	Aware of SI: Yes - had heard of SI before	Coding phase: 1. Single coded	Client marital status: Married	Client age group: 30-39 years	Time since trained: N/A - client

Codes Applied:	C0.3 - Client previous use/experience of any FP method

Interviewer: now I would like to talk about what you experience with Depo you are using. Apart from Depo, have you used any other method of family planning?

Respondent: yes, an implant for 5 years

Interviewer: what was your experience with the implant?

Respondent: it made me gain weight. One of the things I liked was that I was not having my menses my husband was happy with that.

Interviewer: where did you find the method?

Respondent: at the facility

Interviewer: why did you stop using the implant?

Respondent: I was gaining too much weight

Interviewer: did you ever use Depo IM?

Respondent: yes

TIME STAMP: 24:00MIN-34:00 MIN

Interviewer: how long did you use Depo IM before starting Sayana Press

Respondent: from 2009 to 2020 but in between I had used an implant. So for Depo IM I used for 7 years

Interviewer: how long did you use implant?

Respondent: 4 years

Title: MZs_SCSI_3.docx

Descriptor Info:	District: Mzimba South	Target Population: TP3 - DMPA-SC SI clients	Respondent type: DMPA-SC SI client	Aware of SI: Yes - had heard of SI before	Coding phase: 1. Single coded	Client marital status: Married	Client age group: 30-39 years	Time since trained: N/A - client

Codes Applied:	C0.3 - Client previous use/experience of any FP method

Interviewer: what challenges did you meet with Depo IM?

Respondent: for the implant I gained weight. For Depo IM the challenge was sometimes we could not find the provider at the facility, yet we were walking a long distance.

Interviewer: what benefits did you see?

Respondent: for the implant I stayed with it for a long period of time. We were just coming for check up

Title: MZs_SCSI_1.docx

Descriptor Info:	District: Mzimba South	Target Population: TP3 - DMPA-SC SI clients	Respondent type: DMPA-SC SI client	Aware of SI: Yes - had heard of SI before	Coding phase: 1. Single coded	Client marital status: Married	Client age group: Under 20 (adolescent)	Time since trained: N/A - client

Codes Applied:	C0.3 - Client previous use/experience of any FP method

Q5. Interviewer: I would like to talk to you about your experiences with contraceptive methods. Other than injectables have used other family planning methods in the past

Respondent: No

Q6. Interviewer: Before starting to self – inject DMPA –SC, have you previously used injectables administered by the provider?

Respondent: Yes

Interviewer: Did you receive your injections in a facility or HSA?

Respondent: At the hospital

Interviewer: For how long where you using injectables administered by a provider before you chose to self-inject?

Respondent: I started on 21/11/2018 and I used the method for about 3 years

Interviewer: How old where you when first started using injectables

Respondent: I was 17 years old

Title: KK_SCSI_2.docx

Descriptor Info:	District: Nkhotakota	Target Population: TP3 - DMPA-SC SI clients	Respondent type: DMPA-SC SI client	Aware of SI: Yes - had heard of SI before	Coding phase: 1. Single coded	Client marital status: Divorced/ separated/ widowed	Client age group: 20-29 years	Time since trained: N/A - client

Codes Applied:	C0.3 - Client previous use/experience of any FP method

INTERVIEWER: Alright, now I would like to talk to you about your experience with contraceptive methods. Other than injectables, have you used other family planning methods in the past

RESPONDENT : No

INTERVIEWER: You have not used any other family planning methods other than injectables?

RESPONDENT : Yes

Title: KK_SCSI_2.docx

Descriptor Info:	District: Nkhotakota	Target Population: TP3 - DMPA-SC SI clients	Respondent type: DMPA-SC SI client	Aware of SI: Yes - had heard of SI before	Coding phase: 1. Single coded	Client marital status: Divorced/ separated/ widowed	Client age group: 20-29 years	Time since trained: N/A - client

Codes Applied:	C0.3 - Client previous use/experience of any FP method

INTERVIEWER: Before starting to self- inject DMPA-SC, had you previously used injectables administered by a provider.

RESPONDENT: Yes

INTERVIEWER: You used it not so

RESPONDENT: Yes, I have ever used

INTERVIEWER: If yes did you receive your vaccine in a facility or from an HSA

RESPONDENT: From the HSA

INTERVIEWER: Ok from the HSA?

RESPONDENT : Yes

INTERVIEWER: If yes, how long were you using injectables (administered by a provider) before you chose to self-inject.

RESPONDET: Three months

INTERVIEWER: Three months, the one that you are administered by the provider called Depo not so

RESPONDENT: yes

Title: KK_SCSI_3.doc

Descriptor Info:	District: Nkhotakota	Target Population: TP3 - DMPA-SC SI clients	Respondent type: DMPA-SC SI client	Aware of SI: Yes - had heard of SI before	Coding phase: 1. Single coded	Client marital status: Married	Client age group: 20-29 years	Time since trained: N/A - client

Codes Applied:	C0.3 - Client previous use/experience of any FP method

INTERVIEWER: okay. So apart from injectable, have you ever used other family planning methods in the past?

RESPONDENT: mmmm (yes)

INTERVIEWER: what methods have you ever used?

RESPONDENT: in the past I was using pills.

INTERVIEWER: Pills?

RESPONDENT: yes

INTERVIEWER: mmmh, beside pills?

RESPONDENT: beside pills then it's this Norplant

INTERVIEWER: you used Norplant?

RESPONDENT: I did.

INTERVIEWER: you have used?

RESPONDENT: mmmm [yes]

INTERVIEWER: okay. Tell me the first method you ever used.

RESPONDENT: the first method I used was Depo.

INTERVIEWER okay and then?

RESPONDENT: I was using Depo. I never used any method after my first born until I got pregnant for this child. But I got 12 Depo injections. After this child, that is when I decided to get Norplant. Norplant affected me and I removed it and I was using an injectable.

INTERVIEWER: when did you use the pills? Because you have also told me about pills.

RESPONDENT: (laughs) on my first born when I go to the hospital for contraception, they would tell me that there is no injection and that I should just use pills. I would use the pills as I wait for injections.

INTERVIEWER: mmmmhu.

RESPONDENT: I wanted to have child spacing (laughs).

INTERVIEWER okay. so you said what was the reason you removed your Norplant?

RESPONDENT: because of my menstruation and headache

INTERVIEWER: did you receive these methods form the hospital or an HSA?

RESPONDENT: Hospital

INTERVIEWER: here?

RESPONDENT: mmmmh

INTERVIEWER: where we are?

RESPONDENT: mmmm

INTERVIEWER: besides that, it gave you problems of your menstruation and headache, what goodness did you encounter with Norplant?

RESPONDENT: honestly, I can't say anything good about Norplant.

INTERVIEWER: (laughs) it was problems throughout?

RESPONDENT: Mmmmmh [yes].

INTERVIEWER: what of....

RESPONDENT: I can’t say anything good about Norplant because soon after I got the implant, after just a week, I was having headache all the time. Finally, it's when menstruating started before it was even the year of removal. it's when I told myself that I should remove it and use another method. [she was serious when she was explaining this]

Time stamp: (10:19)

INTERVIEWER: what of the pills? What did you think were their importance?

RESPONDENT: (laughs) the pills there is no importance really for me.

INTERVIEWER: None?

RESPONDENT: mmmm (yes) it is the same with the Norplant

INTERVIEWER: what problems were they giving you?

RESPONDENT: Pills, menstruation.

INTERVIEWER: Same as this one?

RESPONDENT: yes

INTERVIEWER: mmmh alright. Before starting to self-inject, had you previously used injectable administered by a provider? We should just say Depo, right?

RESPONDENT: mmmm

INTERVIEWER: yeah?

RESPONDENT: Yes

INTERVIEWER: so where were you receiving this injection from?

RESPONDENT: I was receiving from this very hospital

INTERVIEWER: okay

RESPONDENT: Yes

INTERVIEWER: mmmhu. How long have you been taking this provider injection before you started Sayana?

RESPONDENT: before starting Sayana I would say I have been using for one month, two months

INTERVIEWER: Two months?

RESPONDENT: yes.

INTERVIEWER: okay, you implanted a Norplant, did you last for a year?

RESPONDENT: The Norplant lasted for a year because I inserted 2019 and removed end of 2018.

INTERVIEWER: when was it implanted?

RESPONDENT: It was implanted in 2019, then the end of 2019 is when I started period and then I removed it. [she was confused with the years].

INTERVIEWER: When you removed... When you removed the Norplant, you started getting injections?

RESPONDENT: Mmmh

INTERVIEWER: so how long have you been getting injections?

RESPONDENT: the injection?

INTERVIEWER: mmmmm

RESPONDENT: I’ve been injecting for three months.

INTERVIEWER: You have come for three consecutive times before starting Sayana?

RESPONDENT: no

INTERVIEWER: Okay explain. Explain to me so that I should understand.

RESPONDENT: when I removed Norplant, I got Depo for two months (two times). The third month, that is when there was Depo stock out, there was only Do you understand?

INTERVIEWER: mmmhu...

RESPONDENT: there was no Depo the third month, I found that there was no Depo but there was Sayana. I told them that I will use Sayana because they are all contraceptives. Yes, that’s how I got Sayana.

Title: KK_SCSI_4.docx

Descriptor Info:	District: Nkhotakota	Target Population: TP3 - DMPA-SC SI clients	Respondent type: DMPA-SC SI client	Aware of SI: Yes - had heard of SI before	Coding phase: 2. Double coded	Client marital status: Married	Client age group: Under 20 (adolescent)	Time since trained: N/A - client

Codes Applied:	C0.3 - Client previous use/experience of any FP method

INTERVIEWER: Other than injectable, have you used other family planning/ contraception in the past

RESPONDENT: No this is my first contraception

INTERVIEWER: Is this the first time? have you not used condoms before

RESPONDENT: No

Title: KK_SCSI_4.docx

Descriptor Info:	District: Nkhotakota	Target Population: TP3 - DMPA-SC SI clients	Respondent type: DMPA-SC SI client	Aware of SI: Yes - had heard of SI before	Coding phase: 2. Double coded	Client marital status: Married	Client age group: Under 20 (adolescent)	Time since trained: N/A - client

Codes Applied:	C0.3 - Client previous use/experience of any FP method

INTERVIEWER: Alright, then we will not touch these other questions since they are about these other family planning methods. The next question, before starting to self-inject DMPA-SC, had you previously used injectables administered by the provider? Before you started using Sayana

RESPONDENT: Yes, once

INTERVIEWER: Used the on administered by the provider once

RESPONDENT: Yes

INTERVIEWER: Was it the doctor who injected you or yourself

RESPONDENT: It was the doctor who injected me the first

INTERVIEWER: Alright, were you injected by the doctor or the HSA?

RESPONDENT: It was at the hospital; I met the one who injects us when we visit a facility.

INTERVIEWER: Alright you were injected here at the facility? Alright, for how long were you using injectables administered by the provider before you chose to self-inject?

RESPONDENT: I used for two months and come the third month I injected myself.

Title: KK_SCSI_4.docx

Descriptor Info:	District: Nkhotakota	Target Population: TP3 - DMPA-SC SI clients	Respondent type: DMPA-SC SI client	Aware of SI: Yes - had heard of SI before	Coding phase: 2. Double coded	Client marital status: Married	Client age group: Under 20 (adolescent)	Time since trained: N/A - client

Codes Applied:	C0.3 - Client previous use/experience of any FP method

INTERVIEWER: How old were you when you first started self-injection

RESPONDENT: 18

INTERVIEWER: 18 Years?

RESPONDENT: So I really don’t know does the years change, since 3 years have passed since I received the first injectable, but I still use the same years (18) that I mentioned when I was pregnant

INTERVIEWER: Alright you were 18 when you were pregnant, when did you get pregnant?

RESPONDENT: I got pregnant when I was 18 years

INTERVIEWER: When was that

RESPONDENT: 2020, 2021

INTERVIEWER: 2020 its last year

RESPONDENT: Yes

INTERVEIWER: That means you are now 19 years because years are added every year

RESPONDENT: Yes

INTERVIEWER: Which means if it was last year that you registered for ante-natal then it should be 18 but if its 2021 then its 18

RESPONDENT: No its last year

INTERVIEWER: That means you were 18 and now you are 19. Hence you started taking injectables when you were 19. When did you start using the injectables, I think at 19 years not so?

RESPONDENT: Yes

Title: KK_SCSI_4.docx

Descriptor Info:	District: Nkhotakota	Target Population: TP3 - DMPA-SC SI clients	Respondent type: DMPA-SC SI client	Aware of SI: Yes - had heard of SI before	Coding phase: 2. Double coded	Client marital status: Married	Client age group: Under 20 (adolescent)	Time since trained: N/A - client

Codes Applied:	C0.3 - Client previous use/experience of any FP method

INTERVIEWER: Before starting to self-inject DMPA-SC, how easy or difficult have you found it accessing family planning services in the past? What do think was it difficult to access family planning methods?

RESPONDENT: No

INTERVIEWER: It is not difficult?

RESPONDENT: Yes

INTERVIEWER: If easy what factors have made it most easy?

RESPONDENT: I stopped having my periods

INTERVIEWER: You are not having your periods?

RESPONDENT: Since I started to inject myself, I have had no periods

INTERVIEWER: Since you started self- injection you have not had your periods?

RESPONDENT: Yes

INTERVIEWER: Alright, so you feel that has helped

RESPONDENT: Yes

Title: ZA_SCSI_3.docx

Descriptor Info:	District: Zomba	Target Population: TP3 - DMPA-SC SI clients	Respondent type: DMPA-SC SI client	Aware of SI: Yes - had heard of SI before	Coding phase: 3. Triple coded	Client marital status: Married	Client age group: 40-49 years	Time since trained: N/A - client

Codes Applied:	C0.3 - Client previous use/experience of any FP method

Interviewer: Okay. Now, I want to discuss with you your experiences while using the family planning methods. Besides the injection method, have you ever used any other type of family planning methods?

Respondent: No.

Interviewer: When did you start using the injection method of family planning?

Respondent: I’ve used it after giving birth to all my children. I started using it again six weeks after my last born who is 2 years and seven months old was born. So I can say I have used it for 2 years now.

Title: ZA_SCSI_3.docx

Descriptor Info:	District: Zomba	Target Population: TP3 - DMPA-SC SI clients	Respondent type: DMPA-SC SI client	Aware of SI: Yes - had heard of SI before	Coding phase: 3. Triple coded	Client marital status: Married	Client age group: 40-49 years	Time since trained: N/A - client

Codes Applied:	C0.3 - Client previous use/experience of any FP method	C0.2.2 - Negative community/client perspectives on FP	C1.1.1 - Positive client attitudes to SI

Interviewer: Okay. Are there advantages and disadvantages of using family planning methods?

Respondent: Yes, when I was being injected at the hospital on my hand, I would feel pain or numbness in my whole arm so if I got injected on my left arm, it made me unable to do my household chores because am left-handed. So I was given another option of injecting myself on my belly button. Since I started injecting there, I haven’t experienced any problems at all.

Title: ZA_SCSI_2.docx

Descriptor Info:	District: Zomba	Target Population: TP3 - DMPA-SC SI clients	Respondent type: DMPA-SC SI client	Aware of SI: Yes - had heard of SI before	Coding phase: 1. Single coded	Client marital status:	Client age group:	Time since trained: N/A - client

Codes Applied:	C0.3 - Client previous use/experience of any FP method

Interviewer: Alright, I would now like to talk to you about your experiences with contraceptive methods. Ah apart from the injectable contraceptives, have you used other family planning methods?

Respondent: I have never used other methods.

Interviewer: Alright, ah how old were you when you first used contraceptives?

Respondent: I have just started using last year.

Interviewer: Alright.

Respondent: 2020

Interviewer: Alright, have you used injectables administered by a provider before you started using DMPA-SC?

Respondent: Yes, I have used the provider administered injectables.

Title: ZA_SCSI_2.docx

Descriptor Info:	District: Zomba	Target Population: TP3 - DMPA-SC SI clients	Respondent type: DMPA-SC SI client	Aware of SI: Yes - had heard of SI before	Coding phase: 1. Single coded	Client marital status:	Client age group:	Time since trained: N/A - client

Codes Applied:	C2.1.1 - Community availability of injectables/SI	C0.3 - Client previous use/experience of any FP method

Interviewer: Ah you have said that you have used the provider administered injectables, ah did you receive this from the facility or from the HSAs?

Respondent: I received from the HSAs

Interviewer: Alright.

Respondent: In my community.

Interviewer: Alright, you have told me that you have used the provider administered injectables, for how long have you used this injectable before you started using DMPA-SC?

Respondent: the provider administered I have taken it twice, and with this one its three times.

Interviewer: Okay

Respondent: Yes.

Interviewer: so we can say approximately nine months?

Respondent: Yes, nine months

Interviewer: Alright

Respondent: This one I have just gotten it here

Title: ZA_SCSI_5.docx

Descriptor Info:	District: Zomba	Target Population: TP3 - DMPA-SC SI clients	Respondent type: DMPA-SC SI client	Aware of SI: Yes - had heard of SI before	Coding phase: 3. Triple coded	Client marital status: Married	Client age group: 30-39 years	Time since trained: N/A - client

Codes Applied:	C0.3 - Client previous use/experience of any FP method

Interviewer: Now I want to talk to you about your experiences with contraceptives. Ah other than the injectable contraceptives, have you used any other contraceptive methods in the past?

Respondent: Yes, I have used.

Interviewer: Can you tell me these methods.

Respondent: Yes, I can tell you

Interviewer: Tell me

Respondent: At first I used pills

Interviewer: Mmm (showing that one is following the conversation)

Respondent: When I took the pills, they explained to me that…

Interviewer: Mmm (showing that one is following the conversation)

Respondent: You should take the pills in this line, when you reach this red line

Interviewer: Mmm (showing that one is following the conversation)

Respondent: You will start your period

Interviewer: Alright.

Respondent: So when you start your period you should stop there.

Interviewer: Mmm (showing that one is following the conversation)

Respondent: So they gave me three months dosage.

Interviewer: Alright.

Respondent: Yes.

Interviewer: Alright.

Respondent: Sure

Interviewer: Ah Can you tell me your experiences using this method of pills?

Respondent: I did not have any problems using this method of pills

Interviewer: Mmm (showing that one is following the conversation)

Respondent: No

Interviewer: Alright.

Respondent: It was just okay

Interviewer: Mmm (showing that one is following the conversation)

Respondent: But when I sat down with my husband

Interviewer: Mmm (showing that one is following the conversation)

Respondent: That’s when I said that I think we should change the method

Interviewer: Mmm (showing that one is following the conversation)

Respondent: We should go and get another method.

Title: ZA_SCSI_5.docx

Descriptor Info:	District: Zomba	Target Population: TP3 - DMPA-SC SI clients	Respondent type: DMPA-SC SI client	Aware of SI: Yes - had heard of SI before	Coding phase: 3. Triple coded	Client marital status: Married	Client age group: 30-39 years	Time since trained: N/A - client

Codes Applied:	C0.3 - Client previous use/experience of any FP method

Interviewer: Are there any good pros about this method of pills that you used?

Respondent: Ah I was not having any problem

Interviewer: Alright.

Respondent: Sure

Interviewer: Alright, why did you stop using the pills?

Respondent: I stopped because it was making me busy.

Interviewer: Mmm (showing that one is following the conversation)

Respondent: Sometimes in the evening I would forget to take the pills.

Interviewer: Eh

Respondent: You take them daily.

Interviewer: Mmm (showing that one is following the conversation)

Respondent: So sometimes I could miss, sometimes I could just get lazy to take them

Interviewer: Yes

Respondent: So looking at that, I just saw that I will keep on forgetting

Interviewer: Mmm (showing that one is following the conversation)

Respondent: But I should stop and just go and get the injectables so that I won’t have the problem of forgetting

Title: ZA_SCSI_5.docx

Descriptor Info:	District: Zomba	Target Population: TP3 - DMPA-SC SI clients	Respondent type: DMPA-SC SI client	Aware of SI: Yes - had heard of SI before	Coding phase: 3. Triple coded	Client marital status: Married	Client age group: 30-39 years	Time since trained: N/A - client

Codes Applied:	C0.3 - Client previous use/experience of any FP method

Interviewer: Alright, for how long did you use provider administered injectable before you started using DMPA-SC for self-injection?

Respondent: I have used for one year.

Interviewer: Mmm (showing that one is following the conversation)

Respondent: The provider administered injectable

Title: ZA_SCSI_5.docx

Descriptor Info:	District: Zomba	Target Population: TP3 - DMPA-SC SI clients	Respondent type: DMPA-SC SI client	Aware of SI: Yes - had heard of SI before	Coding phase: 3. Triple coded	Client marital status: Married	Client age group: 30-39 years	Time since trained: N/A - client

Codes Applied:	C0.4 - Access to or process of obtaining FP services	C0.3 - Client previous use/experience of any FP method

Interviewer: Alright, in the past before you started using DMPA SC for self-injection, how difficult or easy was it to access contraceptives?

Respondent: Sayana?

Interviewer: Before you started using Sayana

Respondent: Mmm (showing that one is following the conversation)

Interviewer: How difficult was it to access family planning methods in the past?

Respondent: Ah it was not difficult.

Interviewer: Mmm (showing that one is following the conversation)

Respondent: Eh that’s why I would just change among the methods.

Interviewer: Mmm (showing that one is following the conversation)

Respondent: Change again

Interviewer: Alright.

Respondent: Yes, so when I started using this one that’s when I decided that I will be using this one

Title: ZA_SCSI_5.docx

Descriptor Info:	District: Zomba	Target Population: TP3 - DMPA-SC SI clients	Respondent type: DMPA-SC SI client	Aware of SI: Yes - had heard of SI before	Coding phase: 3. Triple coded	Client marital status: Married	Client age group: 30-39 years	Time since trained: N/A - client

Codes Applied:	C0.4 - Access to or process of obtaining FP services	C0.3 - Client previous use/experience of any FP method

Interviewer: Alright, can you explain to me other difficult things that you were experiencing when you were using the provider administered injectables, not the problems in your body but maybe logistical issues, can you explain to me?

Respondent: Yes, for Depo it was difficult.

Interviewer: Mmm (showing that one is following the conversation)

Respondent: Because when the months were getting closer.

Interviewer: Mmm (showing that one is following the conversation)

Respondent: Oh they gave me this date so I should rush.

Interviewer: Mmm (showing that one is following the conversation)

Respondent: And sometimes I would forget the date.

Interviewer: Mmm (showing that one is following the conversation)

Respondent: What was the date that they gave me, when I came here, they would send me back saying that my date is not yet due.

Interviewer: Mmm (showing that one is following the conversation)

Respondent: So I was abandoning chores at home

Interviewer: Mmm (showing that one is following the conversation)

Respondent: And coming here just to go back home

Interviewer: Mmm (showing that one is following the conversation)

Respondent: So from that I saw that its that I change

Interviewer: Mmm (showing that one is following the conversation)

Respondent: I should start using Sayana

Interviewer: Mmm (showing that one is following the conversation)

Respondent: Because that one was getting me busy

Title: MZs_SCSI_2.docx

Descriptor Info:	District: Mzimba South	Target Population: TP3 - DMPA-SC SI clients	Respondent type: DMPA-SC SI client	Aware of SI: Yes - had heard of SI before	Coding phase: 3. Triple coded	Client marital status: Married	Client age group: Under 20 (adolescent)	Time since trained: N/A - client

Codes Applied:	C0.3 - Client previous use/experience of any FP method

Q5. Interviewer: Other than injectables have you used other family planning/contraception in the past?

Respondent: No

Interviewer: How old were you when you first started using contraception?

Respondent: I can`t remember, but my child now is two years and I started using the method immediately after delivery.

Interviewer: Did you start family planning immediately after delivery?

Respondent: When I came for under five clinic it’s when they counseled me on family planning methods, and this was after 6 weeks of my delivery.

Interviewer: So if you are saying a child now is 2 years that means you started at the age of 17.

Respondent: Yes.

Interviewer: Are you saying you started family planning after delivery of your child?

Respondent: Mmmmm (yes).

Q6. Interviewer: Before you started self-inject DMPA-SC, have previously used injectables administered by the provider?

Respondent: Yes.

Interviewer: Were you receiving this method at the facility or at the HSA?

Respondent: At the facility.

Interviewer: How long did you use the method administered by the provider before you started self-injection?

Respondent: Mmmmm, I can lie

Interviewer: Okay, when did you start this self-injection method?

Respondent: This year January.

Interviewer: Since you gave birth you have been using providers injection?

Respondent: Yes.

Title: MZs_SCSI_2.docx

Descriptor Info:	District: Mzimba South	Target Population: TP3 - DMPA-SC SI clients	Respondent type: DMPA-SC SI client	Aware of SI: Yes - had heard of SI before	Coding phase: 3. Triple coded	Client marital status: Married	Client age group: Under 20 (adolescent)	Time since trained: N/A - client

Codes Applied:	C1.1.1 - Positive client attitudes to SI	C0.2.2 - Negative community/client perspectives on FP	C0.3 - Client previous use/experience of any FP method

Q7. Interviewer: Thanks a lot, as you have said before you started self-injection you have been using providers injection, did you find it difficult or not?

Respondent: No it was easy, but when I came for providers injection it’s when they told us that it’s out of stock, rather they told us there is new method and provider said that with this method we can be able to inject ourselves.

Interviewer: You have said it was easy can you explain how easy it was?

Respondent: I achieved what I wanted, even though some women were saying that they bleed too much but to I was not bleeding a lot and my first time using it I was not bleeding at all.

Title: MZs_SCSI_5.docx

Descriptor Info:	District: Mzimba South	Target Population: TP3 - DMPA-SC SI clients	Respondent type: DMPA-SC SI client	Aware of SI: Yes - had heard of SI before	Coding phase: 1. Single coded	Client marital status: Married	Client age group: 20-29 years	Time since trained: N/A - client

Codes Applied:	C0.3 - Client previous use/experience of any FP method

Interviewer: Mmmmh aaah (thinking out loud) now I would like to ask you on your experiences with self-injection.

Respondent: Mmmh (agreeing)

Interviewer: Apart from Sayana Press self-injection, have you used any other family planning method?

Respondent: Pardon?

Interviewer: Apart from self-inject, have you used any other family planning method before?

Respondent: Mmmh mmmh (denying) like I said I took first method after I had a miscarriage at Mzimba DHO. But voluntarily its after my third born child.

Interviewer: You have never used any other method?

Respondent: Mmmh mmmmh (denying)

Interviewer: Only self-inject?

Respondent: Yes

Interviewer: How many times did you get Depo Provera after using self-inject?

Respondent: Once

Interviewer: Mmmhu (probing)

Respondent: Yes

Title: MZs_SCSI_5.docx

Descriptor Info:	District: Mzimba South	Target Population: TP3 - DMPA-SC SI clients	Respondent type: DMPA-SC SI client	Aware of SI: Yes - had heard of SI before	Coding phase: 1. Single coded	Client marital status: Married	Client age group: 20-29 years	Time since trained: N/A - client

Codes Applied:	C0.3 - Client previous use/experience of any FP method	C5.2.1 - Discontinuation of SI (including reasons)

Interviewer: Would you tell me what you experienced after self-injecting?

Respondent: What I experienced?

Interviewer: Yes, if you met any challenges after self-injecting.

Respondent: In my body?

Interviewer: Yes, if at all you saw benefits as well

Respondent: I did not experience any challenges when I got my first dose. We have other illnesses, but I don’t think they were related to Sayana Press. But when I got my second dose I had several symptoms: I had dizziness, heart palpitations. It usually occurred after waking up so I could lie down a bit until the symptoms subside, I would simply take a bed rest. Upon reporting to the health facility I was told that I did not have any problems after their assessments. I also had general body weakness and joint pains. But I started feeling better after I got a dose of Depo Provera.

Title: KK_SCSI_1.docx

Descriptor Info:	District: Nkhotakota	Target Population: TP3 - DMPA-SC SI clients	Respondent type: DMPA-SC SI client	Aware of SI: Yes - had heard of SI before	Coding phase: 3. Triple coded	Client marital status: Never married	Client age group: Under 20 (adolescent)	Time since trained: N/A - client

Codes Applied:	C0.3 - Client previous use/experience of any FP method

INTERVIEWER: Alright. I would like to talk to you about your experiences in using contraceptives.

RESPONDENT: Alright.

INTERVIEWER: Mmmmhu. Apart from injectables, have you used other contraceptives before?

RESPONDENT: No it’s the only one.

INTERVIEWER: The only one?

RESPONDENT: mmmhu.

INTERVIEWER: Aaah apart from this one…the one you are using now, you started with this one or you have used a different method before?

RESPONDENT: I have never used a different method; it is the only one.

INTERVIEWER: It’s the only one?

RESPONDENT: mmmmmh.

INTERVIEWER: Okay. Okay so we are proceeding, and I will ask you again, right?

RESPONDENT: mmmmh.

INTERVIEWER: apart from this injectable you are using now, aaah have you ever used other contraceptive methods before?

RESPONDENT: Yes, I have.

INTERVIEWER: Aaah…aa…what were you using?

RESPONDENT: I was using condoms.

Title: KK_SCSI_1.docx

Descriptor Info:	District: Nkhotakota	Target Population: TP3 - DMPA-SC SI clients	Respondent type: DMPA-SC SI client	Aware of SI: Yes - had heard of SI before	Coding phase: 3. Triple coded	Client marital status: Never married	Client age group: Under 20 (adolescent)	Time since trained: N/A - client

Codes Applied:	C0.3 - Client previous use/experience of any FP method

INTERVIEWER: What are the pros and cons of…what were the pros and cons right, that you experienced when using the condoms? What problems did you face?

RESPONDENT: The problems are that some people hate condoms right…for you to be using condoms. For example men, they don’t want to be using condoms, that’s the problem. But the good thing is that you are protecting yourself from diseases and pregnancies.

INTERVIEWER: Okay. Aaa...why did you stop using condoms? Why?

Time stamp: (10:05)

RESPONDENT: I wanted to…I stopped using condoms because I wanted to find a more suitable method.

INTERVIEWER: okay.

RESPONDENT: mmmhu.

INTERVIEWER: you stopped using condoms because you wanted to find…

RESPONDENT: Another method that is more suitable and trusted.

INTERVIEWER: Okay?

RESPONDENT: Yes.

Title: KK_SCSI_1.docx

Descriptor Info:	District: Nkhotakota	Target Population: TP3 - DMPA-SC SI clients	Respondent type: DMPA-SC SI client	Aware of SI: Yes - had heard of SI before	Coding phase: 3. Triple coded	Client marital status: Never married	Client age group: Under 20 (adolescent)	Time since trained: N/A - client

Codes Applied:	C0.3 - Client previous use/experience of any FP method

INTERVIEWER: Okay. Did you first start using self-injectable…provider injectable before using self-injectable?

RESPONDENT: Yes. They injected me; it was in November when they injected me. I first used provider injectable when I started.

INTERVIEWER: Alright. Depo?

RESPONDENT: Mmmmh at first.

INTERVIEWER: okay. Explain in detail.

RESPONDENT: When I was going to start contraceptives at the hospital, they injected me…they injected me Depo not Sayana. They injected me here [pointing on her arm]

INTERVIEWER: Okay they injected you here?

RESPONDENT: Mmmmhu.

INTERVIEWER: Alright. Were you injected by an HSA or at the hospital?

RESPONDENT: A nurse injected me at the hospital.

INTERVIEWER: okay. For how long were you injected this one administered by a nurse right, how long did you receive before Sayana?

RESPONDENT: I only received once.

INTERVIEWER: Once?

RESPONDENT: mmmmh

INTERVIEWER: which means you received once and then started…

RESPONDENT: Sayana

INTERVIEWER: Sayana?

RESPONDENT: mmmh.

INTERVIEWER: so how old were you this time?

RESPONDENT: I was 18 years old because I started injectable last year.

INTERVIEWER: You started…you started injectables last year when you were 18?

RESPONDENT: mmmh.

INTERVIEWER: Depo?

RESPONDENT: mmmh.

INTERVIEWER: How about condoms, the same 18?

RESPONDENT: Aaa condoms…it was back then with condoms. Around 16.

INTERVIEWER: 16?

RESPONDENT: mmmh.

Title: ZA_SCSI_4.docx

Descriptor Info:	District: Zomba	Target Population: TP3 - DMPA-SC SI clients	Respondent type: DMPA-SC SI client	Aware of SI: Yes - had heard of SI before	Coding phase: 2. Double coded	Client marital status: Married	Client age group: 30-39 years	Time since trained: N/A - client

Codes Applied:	C0.3 - Client previous use/experience of any FP method

Interviewer: Now I would like to talk about your experiences with contraceptive methods. Other than injectables, have you used other family planning methods in the past?

Respondent: No, I have just used this provider administered injectable.

Interviewer: mmm (showing that one is following the conversation)

Respondent: I have also used the self-injection injectable.

Title: ZA_SCSI_4.docx

Descriptor Info:	District: Zomba	Target Population: TP3 - DMPA-SC SI clients	Respondent type: DMPA-SC SI client	Aware of SI: Yes - had heard of SI before	Coding phase: 2. Double coded	Client marital status: Married	Client age group: 30-39 years	Time since trained: N/A - client

Codes Applied:	C0.3 - Client previous use/experience of any FP method	C0.2.2 - Negative community/client perspectives on FP

Interviewer: Alright, are there any pros and cons on this method?

Respondent: Ah on my side I didn’t see any problems

Interviewer: Alright.

Respondent: I just heard other women saying that they came to the facility to meet with the doctor that they were facing some problems, but for me

Interviewer: yah

Respondent: All those years up until now

Interviewer: mmm (showing that one is following the conversation)

Respondent: I just hear from people that the injection has a problem, but for me I have never faced any of the problems.

Interviewer: Alright.

Respondent: mmm (agreeing)

Title: ZA_SCSI_4.docx

Descriptor Info:	District: Zomba	Target Population: TP3 - DMPA-SC SI clients	Respondent type: DMPA-SC SI client	Aware of SI: Yes - had heard of SI before	Coding phase: 2. Double coded	Client marital status: Married	Client age group: 30-39 years	Time since trained: N/A - client

Codes Applied:	C0.4 - Access to or process of obtaining FP services	C0.3 - Client previous use/experience of any FP method

Interviewer You have shared with me that you were using provider administered injectables

Respondent: mmm (showing that one is following the conversation)

Interviewer: Why did you stop using this injectable?

Respondent: I stopped using this injectable because in this period of covid

Interviewer: mmmm (showing that one is following the conversation)

Respondent: So they told us that we should be social distancing, because here at the hospital we are a lot of women.

Interviewer: eh

Respondent: So we are always crowded so they told us that starting from that day everyone should be trained.

Interviewer: mmm (showing that one is following the conversation)

Respondent: in self injection

Interviewer: mmmm (showing that one is following the conversation)

Respondent: Everyone should start using the injectable. So the day that we were trained everyone too self-injection doses for one year and went home with it

Title: ZA_SCSI_4.docx

Descriptor Info:	District: Zomba	Target Population: TP3 - DMPA-SC SI clients	Respondent type: DMPA-SC SI client	Aware of SI: Yes - had heard of SI before	Coding phase: 2. Double coded	Client marital status: Married	Client age group: 30-39 years	Time since trained: N/A - client

Codes Applied:	C0.3 - Client previous use/experience of any FP method	C1.1.3 - Awareness of DMPA-SC SI

Interviewer: Alright, how long did you use the provider administered injectable contraceptive?

Respondent: I have used it for one year

Interviewer: mmm (showing that one is following the conversation)

Respondent: After one year I came to get more

Interviewer: mmm

Respondent: eh

Interviewer: That one is the self-injection, right?

Respondent: Yes, the self-injecting one

Interviewer: Alright, what about the provider administered one, how long did you use it for?

Respondent: iiii (thinking deeply) 16 years

Interviewer: Okay

Respondent: mmm

Title: MZs_SCSI_4.docx

Descriptor Info:	District: Mzimba South	Target Population: TP3 - DMPA-SC SI clients	Respondent type: DMPA-SC SI client	Aware of SI: Yes - had heard of SI before	Coding phase: 2. Double coded	Client marital status: Married	Client age group: 20-29 years	Time since trained: N/A - client

Codes Applied:	C0.3 - Client previous use/experience of any FP method

Interviewer: now we would like to talk about your experiences in using family planning methods. Apart from this method which you self-inject, have you ever used other family planning methods?

Respondent: Yes. I once used provider injected method (showed where she was given)

Interviewer: On the arm?

Respondent: Yes

Interviewer: What name was the family planning method?

Respondent: They said….(thinking out loud) …which one is called loop?

Interviewer: The one with a string they insert in the vagina. Then there is Jadelle or Norplant which they insert in the arm

Respondent: Eee

Interviewer: Did you get Jadelle? How many years was it?

Respondent: No they did not insert but inject

Interviewer: Did they inject? Did they tell you the name?

Respondent: Aaah aaah (denying)

Interviewer: It could be Depo

Respondent: Alright

Interviewer: What was your experience using this type of method?

Respondent: I had excessive menstrual bleeding

Interviewer: Mmh (agreeing)

Respondent: Yes

Interviewer: What else did you experience?

Respondent: Excessive bleeding and dizziness

Interviewer: Dizziness?

Respondent: Yes

Interviewer: Is there anything else?

Respondent: No

Interviewer: This self-inject method did you self-inject or you were injected by HSAs in your catchment area?

Respondent: HSA in our catchment area

Interviewer: Have you ever received from else-where apart from the health facility or from HSA?

Respondent: Mmmmh mmmmh (denying)

Interviewer: You said you experienced excessive bleeding and dizziness? Do you experience any side effects apart from these?

Respondent: Aaaah no

Interviewer: What about the benefits you experienced

Respondent: The outstanding benefit is that I didn’t get pregnant

Interviewer: Any other benefit you experience

Respondent: Nothing else

Title: MZs_SCSI_4.docx

Descriptor Info:	District: Mzimba South	Target Population: TP3 - DMPA-SC SI clients	Respondent type: DMPA-SC SI client	Aware of SI: Yes - had heard of SI before	Coding phase: 2. Double coded	Client marital status: Married	Client age group: 20-29 years	Time since trained: N/A - client

Codes Applied:	C0.3 - Client previous use/experience of any FP method

Interviewer: What made you change from provider Depo to Sayana Press

Respondent: I had excesses menstrual bleeding, so I changed to self-inject

Interviewer: Is there any reason that made you stop

Respondent: None

Interviewer: How long did you use provider Depo?

Respondent: I used for three months as advised by the provider

Interviewer: Mmmh (agreeing)

Respondent: That’s when I changed to self-inject

Title: ZA_SCSI_1.docx

Descriptor Info:	District: Zomba	Target Population: TP3 - DMPA-SC SI clients	Respondent type: DMPA-SC SI client	Aware of SI: Yes - had heard of SI before	Coding phase: 1. Single coded	Client marital status: Married	Client age group: 40-49 years	Time since trained: N/A - client

Codes Applied:	C0.3 - Client previous use/experience of any FP method

Interviewer: Thank you very much. Let’s enter another section, I want you to share with me what you have encountered for using family planning methods? Apart from injection have you used any other method?

Respondent: No I haven’t used any other.

Title: ZA_SCSI_1.docx

Descriptor Info:	District: Zomba	Target Population: TP3 - DMPA-SC SI clients	Respondent type: DMPA-SC SI client	Aware of SI: Yes - had heard of SI before	Coding phase: 1. Single coded	Client marital status: Married	Client age group: 40-49 years	Time since trained: N/A - client

Codes Applied:	C0.3 - Client previous use/experience of any FP method

Interviewer: Have you ever used before an injection from the hospital?

Respondent: Yes, I have used it before.

Interviewer: So before you started?

Respondent: I used to go to the hospital but after introducing self-injection then I opted for that one.

Interviewer: Did you receive the injection from medical assistant or hospital or community medical assistant?

Respondent: Community medical assistants are the ones who gave us.

Interviewer: At first you were saying?

Respondent: We used to get an injection from the hospital.

Interviewer: Okay, okay, for how long have you been using hospital injections before you started using self-injections?

Respondent: Used it for a year then I started using self-injections.
