# Supplementary material for: Health care provider and client experiences of counselling on depot medroxyprogesterone acetate subcutaneous (DMPA-SC) for self-injection in Malawi
Source: PLOS Glob Public Health. 2023 Nov 30;3(11):e0002057. doi: 10.1371/journal.pgph.0002057 (PMC10688740; doi:10.1371/journal.pgph.0002057)
Supplement: S1 Data — (ZIP) [file pgph.0002057.s001.zip › S2/C0.4 client access to FP services.doc]

Title: KK_IM_3.docx

Descriptor Info:	District: Nkhotakota	Target Population: TP2 - DMPA-IM/DMPA-SC PI clients	Respondent type: DMPA-IM client	Aware of SI: Yes - had heard of SI before	Coding phase: 1. Single coded	Client marital status: Divorced/ separated/ widowed	Client age group: 20-29 years	Time since trained: N/A - client

Codes Applied:	C0.4 - Access to or process of obtaining FP services

INTERVIEWER: Ok, we are proceeding with our questions, how easy or difficult have you found it accessing family planning services in the past

RESPONDENT: As for me I did not find it difficult to access family planning services because I visited the hospital and that is where I was advised on different methods and that where I have been getting my contraceptives.

INTERVIEWER: Where have you accessed contraception in the past?

RESPONDENT: I get most of the contraceptives from the hospital

INTERVIEWER: At the facility?

RESPONDENT: Yes

INTERVIEWER: If easy, what factors have made it easy? I recall you have just said you did not found it difficult to access contraceptives?

RESPONDENT: Yes

INTERVIEWER: So what factors have made it easy?

RESPONDENT: It helps in keeping my life healthy, and also my body to be strong

INTERVIEWER: If you found accessing family planning difficult, what has made it most difficult? I think this question does not apply to you since you have already responded that you did not find it difficult to access contraceptives not so?

RESPONDENT: Yes

INTERVIEWER: Has the ease/ difficult of accessing family planning services changed since the outbreak of corona virus? Accessing family planning now and before coronavirus outbreak has there been a change?

RESPONDENT: No there has never been a change in accessing family planning services due to coronavirus outbreak

INTERVIEWER: Nothing has changed?

RESPONDENT: Yes, everything is just normal

Title: KK_IM_3.docx

Descriptor Info:	District: Nkhotakota	Target Population: TP2 - DMPA-IM/DMPA-SC PI clients	Respondent type: DMPA-IM client	Aware of SI: Yes - had heard of SI before	Coding phase: 1. Single coded	Client marital status: Divorced/ separated/ widowed	Client age group: 20-29 years	Time since trained: N/A - client

Codes Applied:	C0.4 - Access to or process of obtaining FP services

INTERVIEWER: Ok for 3 years fine, can you describe the process of obtaining your 3 – monthly injection? To start, how do you remember to re-inject?

RESPONDENT: We remember because we are told at the hospital on the next date to report for another injection, however they also indicate in our health passport books on the next date to come and get injected.

INTERVIEWER: So, do you have to organize anything in advance before you can go for your injection? How do u organize yourself? What do you normally do?

RESPONDENT: We make sure that we have our health passport book so that the next date to go back to the hospital to get injected should be indicated.

INTERVIEWER: Ok so you make sure that before you leave you have packed your health pass port book?

RESPONDENT: Yes

INTERVIEWER: So that you may take it as you go to the hospital

RESPONDENT: But also, to ensure that the date has been verified before getting injected

INTERVIEWER: Alright

RESPONDENT: Because some may just go yet they forgot their actual date.

INTERVIEWER: Alright fine, where do you usually go for your injection? How do you get there and how long does it take?

RESPONDENT: At Benga health center

INTERVIEWER: That’s where you get injected?

RESPONDENT: Yes, at the health center

INTERVIEWER: How do you get there from your home to the facility?

RESPONDENT: It is closer, so I normally walk

INTERVIEWER: You go by foot do how long does it take you to reach the facility?

RESPONDENT: It could be 30 minutes

INTERVIEWER: 30 minutes? Okay

RESPONDENT: Yes

INTERVIEWER: What happens at the facility /with the HSA? How much time does the provider spend with you and what do they say?

RESPONDENT: When we get to the health center, they usually ask us how we have coped up with the previous contraceptive that we took, I would say they take us through a counselling session. So if anyone wants to change the method she can do so, whether you want to get no-plant or to maintain the previous one depending on your experience with the previous contraceptive.

INTERVIEWER: Alright, so that means every visit you make the provider offers a counselling session

RESPONDENT: Yes

INTERVIEWER: So one has a choice?

RESPONDENT: Yes

Title: KK_SCSI_5.docx

Descriptor Info:	District: Nkhotakota	Target Population: TP3 - DMPA-SC SI clients	Respondent type: DMPA-SC SI client	Aware of SI: Yes - had heard of SI before	Coding phase: 2. Double coded	Client marital status: Married	Client age group: 20-29 years	Time since trained: N/A - client

Codes Applied:	C0.4 - Access to or process of obtaining FP services

INTERVIEWER: That good then. Did you receive these from the facility, from am HSA or from other sources?

Time stamp: (10 minutes 8 seconds)

RESPONDENT: From a facility

INTERVIEWER: Ok you were getting the Norplant form a facility?

Title: KK_SCSI_5.docx

Descriptor Info:	District: Nkhotakota	Target Population: TP3 - DMPA-SC SI clients	Respondent type: DMPA-SC SI client	Aware of SI: Yes - had heard of SI before	Coding phase: 2. Double coded	Client marital status: Married	Client age group: 20-29 years	Time since trained: N/A - client

Codes Applied:	C0.4 - Access to or process of obtaining FP services

INTERVIEWER: Alright let’s proceed, before starting to self- inject DMPA-SC, had you previously used injectables administered by a provider

RESPONDENT: Yes, I used it

INTERVIEWER: Ok you used it, were you getting from a facility or from an HSA?

RESPONDENT: From a facility?

INTERVIEWER: You were getting it from a facility alright. So how long were you using injectables before you chose to self-inject? That is only once?

RESPONDENT: Yes, I didn’t use it for a long period it’s when I started using Sayana

INTERVIEWER: Ok, before starting to self-inject DMPA-SC, how easy or difficult did you found it accessing family planning services in the past? Was it easy to access family planning methods or it was difficult? If you say you say it was difficult, how difficult was it and if you say it was easy, how easy was it

RESPONDENT: Yes, it was easy because a facility where I use to get family planning methods is close by

INTERVIEWER: Okay

RESPONDENT: Yes

Title: KK_SCSI_5.docx

Descriptor Info:	District: Nkhotakota	Target Population: TP3 - DMPA-SC SI clients	Respondent type: DMPA-SC SI client	Aware of SI: Yes - had heard of SI before	Coding phase: 2. Double coded	Client marital status: Married	Client age group: 20-29 years	Time since trained: N/A - client

Codes Applied:	C0.4 - Access to or process of obtaining FP services

INTERVIEWER: Alright, we may proceed a bit because you have already answered some of the questions. Has the ease/ difficult of accessing FP services changed since the outbreak of corona virus?

RESPONDENT: No it has not changed; accessibility has been easy since then

INTERVIEWER: That means accessibility has been easy bore and even after the outbreak of coronavirus it has not changed ?

RESPONDENT: Yes, it is the same

INTERVIEWER: Coronavirus has not affected anything?

RESPONDENT: No everything is normal

INTERVIEWER: Alright that one is clear, we are continuing with our discussions.

Title: MZs_IM_8.docx

Descriptor Info:	District: Mzimba South	Target Population: TP2 - DMPA-IM/DMPA-SC PI clients	Respondent type: DMPA-IM client	Aware of SI: Yes - had heard of SI before	Coding phase: 1. Single coded	Client marital status: Married	Client age group: 20-29 years	Time since trained: N/A - client

Codes Applied:	C0.4 - Access to or process of obtaining FP services

Interviewer: how did you access Depo-IM?

Respondent: I went to the health facility. They recorded the next date of appointment in my health passport book. They tell me the date because some of us may not read

Interviewer: do you prepare for anything before you come to the facility?

Respondent: nothing I just take a bathe, and get my health passport book

Interviewer: what do you do with your business when you are due?

Respondent: that time I do not do business. I go to the business after getting my family planning method

Interviewer: where to you get your Depo?

Respondent: at the facility

Interviewer: how to you travel?

Respondent: I walk

Interviewer: how long do you travel?

Respondent: between 40 minutes to 1 hour

Interviewer: what happens when you arrive at the facility?

Respondent: when we come here at the facility, we wait until they start the clinic at 8 am. We queue before the clinic starts. Then we start entering one by one.

Interviewer: how long does the provider take with each one of you?

Respondent: they start the clinic at 8 am. We enter the room one by one by we don’t take much time

Title: MZs_SCSI_3.docx

Descriptor Info:	District: Mzimba South	Target Population: TP3 - DMPA-SC SI clients	Respondent type: DMPA-SC SI client	Aware of SI: Yes - had heard of SI before	Coding phase: 1. Single coded	Client marital status: Married	Client age group: 30-39 years	Time since trained: N/A - client

Codes Applied:	C0.4 - Access to or process of obtaining FP services

Interviewer: where did you find the method?

Respondent: at the facility

Title: MZs_SCSI_3.docx

Descriptor Info:	District: Mzimba South	Target Population: TP3 - DMPA-SC SI clients	Respondent type: DMPA-SC SI client	Aware of SI: Yes - had heard of SI before	Coding phase: 1. Single coded	Client marital status: Married	Client age group: 30-39 years	Time since trained: N/A - client

Codes Applied:	C0.4 - Access to or process of obtaining FP services

Interviewer: what challenges did you meet with Depo IM?

Respondent: for the implant I gained weight. For Depo IM the challenge was sometimes we could not find the provider at the facility, yet we were walking a long distance.

Title: MZs_SCSI_3.docx

Descriptor Info:	District: Mzimba South	Target Population: TP3 - DMPA-SC SI clients	Respondent type: DMPA-SC SI client	Aware of SI: Yes - had heard of SI before	Coding phase: 1. Single coded	Client marital status: Married	Client age group: 30-39 years	Time since trained: N/A - client

Codes Applied:	C0.4 - Access to or process of obtaining FP services

Interviewer: what do you think a woman needs to know on self-injection?

Respondent: they need to know that it saves time. And also it helps to prevent covid-19 since we do not come here

Title: MZs_IM_2.docx

Descriptor Info:	District: Mzimba South	Target Population: TP2 - DMPA-IM/DMPA-SC PI clients	Respondent type: DMPA-IM client	Aware of SI: Yes - had heard of SI before	Coding phase: 3. Triple coded	Client marital status: Married	Client age group: 30-39 years	Time since trained: N/A - client

Codes Applied:	C0.4 - Access to or process of obtaining FP services

Interviewer: So this method you were using, where did you receive it from?

Respondent: From the facility.

Title: MZs_IM_2.docx

Descriptor Info:	District: Mzimba South	Target Population: TP2 - DMPA-IM/DMPA-SC PI clients	Respondent type: DMPA-IM client	Aware of SI: Yes - had heard of SI before	Coding phase: 3. Triple coded	Client marital status: Married	Client age group: 30-39 years	Time since trained: N/A - client

Codes Applied:	C0.4 - Access to or process of obtaining FP services

Interviewer: Have it been easy or difficult of accessing family planning services changed since the outbreak of coronavirus?

Respondent: Mmmm…. (thinking), change…… mmmm… no, since I come here to be injected the only change I see is that when visiting the facility we should make sure we put on mask, failing which they send us away to go and put on a mask and thus the only change I see.

Title: MZs_IM_2.docx

Descriptor Info:	District: Mzimba South	Target Population: TP2 - DMPA-IM/DMPA-SC PI clients	Respondent type: DMPA-IM client	Aware of SI: Yes - had heard of SI before	Coding phase: 3. Triple coded	Client marital status: Married	Client age group: 30-39 years	Time since trained: N/A - client

Codes Applied:	C0.4 - Access to or process of obtaining FP services

Interviewer: What do you like about injectables?

Respondent: What I like about injectables is that even if I go to my village and forgot the date am supposed to go the hospital for the injection or maybe I am busy with something and I forgot to go to the hospital I don’t get pregnant because this method works more days than expected. Rather than, if you forgot the date to go to the hospital for an injection that means if you sleep with your husband that means you are pregnant.

Title: MZs_IM_2.docx

Descriptor Info:	District: Mzimba South	Target Population: TP2 - DMPA-IM/DMPA-SC PI clients	Respondent type: DMPA-IM client	Aware of SI: Yes - had heard of SI before	Coding phase: 3. Triple coded	Client marital status: Married	Client age group: 30-39 years	Time since trained: N/A - client

Codes Applied:	C0.4 - Access to or process of obtaining FP services

Q8. Interviewer: Can you describe the process for obtaining your 3 months injection? How do you remember to re-inject?

Respondent: As for me I do come here to the facility to be re-injected by the doctor.

Interviewer: At first how do you remember to re-inject

Respondent: They always tell us the date we are supposed to come and re-inject, like the day before yesterday they told me to come on 9th November for re-injection but all the time we do remember even though they do document in our health passport, and I do check the date I am supposed to go for re-injection.

Interviewer: Do you have to organize anything in advance before you can go for your injection?

Respondent: Yes I do organize myself because I have a grocery, whenever I want to go for injection I do close my grocery, or sometimes I do carry my mandasi business to sell at the facility as if I have just come for business yet I have come for family planning in order to hind it from my mother in-law for her not to know that I have come for family planning cause she always ask why am I coming to the facility as if am sick.

Interviewer: Where do you usually go for injection?

Respondent: At the facility.

Interviewer: Which facility?

Respondent: Mzalangwe

Interviewer: How do you get here?

Respondent: By foot.

Interviewer: By foot?

Respondent: Mmmmmm (yes)

Interviewer: If by foot how long does it take?

Respondent: Aaaaaa…. like 1 hour.

Interviewer: From home to the facility you take 1 hour?

Respondent: Yes, it`s not far

Title: ZA_IM_2.docx

Descriptor Info:	District: Zomba	Target Population: TP2 - DMPA-IM/DMPA-SC PI clients	Respondent type: DMPA-IM client	Aware of SI: Yes - had heard of SI before	Coding phase: 1. Single coded	Client marital status: Married	Client age group: 30-39 years	Time since trained: N/A - client

Codes Applied:	C0.4 - Access to or process of obtaining FP services

Interviewer: Alright, where did you get these condoms from? Did you get them from the hospital, or from the health surveillance assistants, or where exactly did you receive these condoms from, or how did you find them?

Respondent: I received them from the hospital.

Title: ZA_IM_2.docx

Descriptor Info:	District: Zomba	Target Population: TP2 - DMPA-IM/DMPA-SC PI clients	Respondent type: DMPA-IM client	Aware of SI: Yes - had heard of SI before	Coding phase: 1. Single coded	Client marital status: Married	Client age group: 30-39 years	Time since trained: N/A - client

Codes Applied:	C0.4 - Access to or process of obtaining FP services

Interviewer: How difficult or easy has it been for you to find condoms, have you faced any problems in trying to find condoms?

Respondent: No, the condoms were easy to find because when we come to the hospital the health professionals were able to explain of the methods that were available and the condoms were among those methods including injectables and no plant, so it was not difficult to find, we were able to meet the health workers and tell them that we want to use the condoms for family planning.

Interviewer: Alright

Respondent: yah

Interviewer: Thank you, have you used this method of condoms during the covid-19 era?

Respondent: No

Title: ZA_IM_2.docx

Descriptor Info:	District: Zomba	Target Population: TP2 - DMPA-IM/DMPA-SC PI clients	Respondent type: DMPA-IM client	Aware of SI: Yes - had heard of SI before	Coding phase: 1. Single coded	Client marital status: Married	Client age group: 30-39 years	Time since trained: N/A - client

Codes Applied:	C0.4 - Access to or process of obtaining FP services

Interviewer: Alright, ah can you explain to me how ah you get the contraceptive method every three months? Are there any other ways other than from the hospital here?

Respondent: Yes, there are some other ways (pause) when the health workers do communications with their bosses, they give a chance to some people who are capable to do the job from their place of residence to inject women, we are able to find a chance due to arrangements like these.

Interviewer: Alright, whenever you want to come to the hospital to get your injection do you prepare for anything before you come, for example arranging for a babysitter when you leave, or maybe you have mentioned of piecework, how it is going to go that particular day at your work, do you get prepared in any way before you come?

Respondent: Yes, before I come to get an injection, I make sure to prepare the kids for school, when they start off for school I also make sure to get ready so that I should come to the hospital, when I get here at the hospital especially for family planning I don’t take much time compared to when you have come for other thing like seeking medical attention on an illness, wen we are here they ask us questions and if we have questions we also ask and then they assist you and now you can return home.

Interviewer: Alright, how do you remember to come back to the hospital for reinjection?

Respondent: When we get here at the hospital, they write in our health passport the date on which we are at the hospital, and then they also write the date that we are supposed to come back for reinjection, so we focus much on the date that we are supposed to come back here, and we check that when the day has come, we return for reinjection.

Interviewer: Alright, can you explain to me about your transportation to get to the hospital to get your injection?

Respondent: When its reinjection time we try to have transport money because the clinic is a long distance from home, so when we have transport money, we hire a bicycle taxi so that it takes us to the clinic, when we are done at the clinic, we also use the bicycle taxi to go back home.

Interviewer: Alright, thank you, what happens when you get to the hospital for your injection?

Respondent: When we get to the hospital the first thing we are given a health talk, we start with a prayer then the health talk, when it’s time to start getting the injection they tell us that we cannot get into the doctors room with other clients, they have to talk to us one on one because maybe other people might have questions that they want to ask, so they may be scared or they may be shy and they may not afford to ask the questions in a group, so we meet with the person who injects us and we tell them about our issues and then they help and us and we go back home.

Interviewer: Alright, how long does this probably take, how long does your meeting with the health professional take?

Respondent: It doesn’t take long.

Interviewer: Approximately how minutes do you take?

Respondent: Maybe five minutes, if you have questions, you can take even ten minutes so that your questions get addressed.

Title: MZs_SCSI_1.docx

Descriptor Info:	District: Mzimba South	Target Population: TP3 - DMPA-SC SI clients	Respondent type: DMPA-SC SI client	Aware of SI: Yes - had heard of SI before	Coding phase: 1. Single coded	Client marital status: Married	Client age group: Under 20 (adolescent)	Time since trained: N/A - client

Codes Applied:	C0.4 - Access to or process of obtaining FP services	C5.1.1 - Continuation of SI (including reasons)

Q7. Interviewer: Before starting to self-inject DMPA-SC how easy or difficult have you found it accessing family planning services in the past

Respondent: It was not difficult

Interviewer: What factors made it easy?

Respondent: I was able to get to the method and my menstrual was just normal

Interviewer: Do you have anything to add?

Respondent: No

Interviewer: Has it been difficult or easy in accessing family planning services changed since the outbreak of coronavirus?

Respondent: There is no change, and it has been easy

Title: KK_IM_5.docx

Descriptor Info:	District: Nkhotakota	Target Population: TP2 - DMPA-IM/DMPA-SC PI clients	Respondent type: DMPA-IM client	Aware of SI: No - never heard of SI before	Coding phase: 3. Triple coded	Client marital status: Divorced/ separated/ widowed	Client age group: Under 20 (adolescent)	Time since trained: N/A - client

Codes Applied:	C0.4 - Access to or process of obtaining FP services

INTERVIEWER: You have never used any? Hence, we will proceed to another question since you have never used other FP methods. How easy or difficult have you found it accessing family planning services in the past?

RESPONDENT: (participant quite seems she has nothing to answer)

INTERVIEWER: how easy or difficult have you found it accessing family planning services in the past?

RESPONDENT: It wasn’t difficult

INTERVIEWER: How was it not difficult?

RESPONDENT: Because it was near so I could make some delays that I will go later

INTERVIEWER: So you mean he distance from your home to the facility was near hence you could just walk if you wanted to get FP?

RESPONDENT: Yes

INTERVIEWER: Is that what you mean?

RESPONDENT: Yes

INTERVIEWER: Alright, where have you accessed contraception in the past? Is it at the facility?

RESPONDENT: Yes

INTERVIEWER: Or else from the HSA

RESPONDENT: From the HSA they bring in our area.

Title: KK_IM_5.docx

Descriptor Info:	District: Nkhotakota	Target Population: TP2 - DMPA-IM/DMPA-SC PI clients	Respondent type: DMPA-IM client	Aware of SI: No - never heard of SI before	Coding phase: 3. Triple coded	Client marital status: Divorced/ separated/ widowed	Client age group: Under 20 (adolescent)	Time since trained: N/A - client

Codes Applied:	C0.4 - Access to or process of obtaining FP services

INTERVIEWER: Alright fine, where do you usually go for your injection in every three months? I recall you mentioned that you get injected by the HSA, does it mean that you also get your injection from the HSA in every three months?

RESPONDENT: Yes

INTERVIEWER: Do you have to organize anything in advance before you can go for your injection?

RESPONDENT: Yes

INTERVIEWER: Like doing what?

RESPONDENT: Getting a card

INTERVIEWER: Yes

RESPONDENT: Mask

INTERVIEWER: Card and Mask?

RESPONDENT: Yes

INTERVIEWER: What about taking care of the kids, so since you only have one you usually take her along with you.

RESPONDENT: Yes

INTERVIEWER: Where is it that you get injected?

RESPONDENT: At the HSA

INTERVIEWER: In your area at the HSA?

RESPONDENT: Yes

INTERVIEWER: And not at the facility?

RESPONDENT: Yes

Title: ZA_IM_1.docx

Descriptor Info:	District: Zomba	Target Population: TP2 - DMPA-IM/DMPA-SC PI clients	Respondent type: DMPA-IM client	Aware of SI: Yes - had heard of SI before	Coding phase: 1. Single coded	Client marital status: Married	Client age group: 40-49 years	Time since trained: N/A - client

Codes Applied:	C0.4 - Access to or process of obtaining FP services

Interviewer: Can you describe the process for obtaining your 3 monthly injection?

Respondent: We obtain this method through health officers who work here at Zilondo hospital

Interviewer: Yes

Respondent: Yes

Interviewer: Alright, alright, do you have to organize anything in advance before you can go for your injection? For instance, like house chores. What are you supposed at home before you come here?

Respondent: I am supposed to do my usual chores like taking care of my home then leave for the hospital to obtain my injectable.

Interviewer: Mhhhh

Respondent: Eya

Interviewer: Is there anything else that you do before coming here at the hospital?

Respondent: Yes, as I previously mentioned, I go to the fields as well as the nursery where I work part time. I firstly go to the nursery school and come here at the hospital once kids knock off.

Interviewer: What time do kids knock off?

Respondent: 11 o’clock

Interviewer: Oh, so you have knocked off early today?

Respondent: I didn’t go to work today

Interviewer: Oh, where do you usually receive your method from?

Respondent: Here at Zilindo hospital

Interviewer: How do you usually get here at the hospital? How do you usually get here?

Respondent: Often times I walk. There is some distance from my house to the hospital. I still walk though

Interviewer: Mmmhhh alright, alright, do you always walk?

Respondent: We usually walk, however we sometimes we get lucky when we meet cyclists they give us lifts.

Interviewer: Alright, for instance how long does it take you to get here? Just give us an estimate.

Respondent: When walking or on a bike?

Interviewer: How long does it take you when walking?

Respondent: When walking from our house it takes us approximately an hour.

Interviewer: When lucky when a cyclist picks you?

Respondent: Maybe in 30min we would have arrived here.

Interviewer: Thank you very much, what happens at the facility? How long do providers take before they start helping you?

Respondent: When we get here at the hospital, we explain to the providers that we have come to access family planning services.

Interviewer: Mmmhhh

Respondent: So they take our health passport and look at the date we were supposed to come. If the dates are matching, the take the health passport and fill in the register. The start with injecting us then they write in the health passports when we are supposed to return to the hospital to get our injectable.

Interviewer: When you come to the hospital, how long does it take for providers to inject you, write in your health passport and fill in the register? How long does this take?

Respondent: This really doesn’t take time; about 5 to 10 mins everything is done.

Title: KK_SCPI_6.docx

Descriptor Info:	District: Nkhotakota	Target Population: TP2 - DMPA-IM/DMPA-SC PI clients	Respondent type: DMPA-SC PI client	Aware of SI: Yes - had heard of SI before	Coding phase: 1. Single coded	Client marital status: Married	Client age group: 20-29 years	Time since trained: N/A - client

Codes Applied:	C0.4 - Access to or process of obtaining FP services

INTERVIEWER: yes, mmmh mmmh [no]. Let me ask it this way, how easy or difficult was it for you to access family planning methods?

RESPONDENT: It was not difficult

INTERVIEWER: It was not difficult?

RESPONDENT: mmmh.

INTERVIEWER: What are the things that helped you to easily access these methods?

RESPONDENT: Right here at the hospital

INTERVIEWER: here at the hospital?

RESPONDENT: yes

INTERVIEWER: How was it

RESPONDENT: The doctor was advising us to take on family planning methods.

INTERVIEWER: mmmmhu.

RESPONDENT: yes

INTERVIEWER: Now since COVID 19 started, has there been any change in the availability of family planning methods? Since the coming of COVID 19 have you observed any change in the availability of family planning methods?

RESPONDENT: No

INTERVIEWER: It is the same?

RESPONDENT: mmmmh.

Title: KK_SCPI_6.docx

Descriptor Info:	District: Nkhotakota	Target Population: TP2 - DMPA-IM/DMPA-SC PI clients	Respondent type: DMPA-SC PI client	Aware of SI: Yes - had heard of SI before	Coding phase: 1. Single coded	Client marital status: Married	Client age group: 20-29 years	Time since trained: N/A - client

Codes Applied:	C0.4 - Access to or process of obtaining FP services	C8.1 - Male involvement, general

INTERVIEWER: mmmhu, Alright. Alright. Can you explain how you access the injection in every three months? However, to begin with how do you remember to go and be injected?

RESPONDENT: Remembering?

INTERVIEWER: mmmh. How do you remember?

RESPONDENT: aaaa, I remember, I always count the dates down to say on such a date I have to go get the injection.

INTERVIEWER: okay.

RESPONDENT: mmmh.

INTERVIEWER: When do you exactly start this counting?

RESPONDENT: We start from the day we get the injection; we just keep counting to the day that tomorrow we are supposed to go and get the injection. In addition, at the same time the husband at home is busy with the same as well.

INTERVIEWER: Your husband assists you?

RESPONDENT: Yes, he does.

INTERVIEWER: mmmmhu. Are you supposed to get organize anything…anything before going for an injection? Let us take for example, taking care of the children. Maybe you check on the state you have left your house. Are you supposed to be prepared in any way before coming to the hospital?

RESPONDENT: Yes. I prepare.

INTERVIEWER: What do you do?

RESPONDENT: We sweep around the hose, fetch water, heating the water, bathing the children, making sure also that the husband has taken a bath. There after I get my card and head here.

INTERVIEWER: …To the hospital?

RESPONDENT: When getting prepared we make sure that the card is ready, put somewhere just waiting for the time.

INTERVIEWER: mmmh.

RESPONDENT: Yes

INTERVIEWER: okay. Mmmh, where do you often get the injection? Because some people do find…some go to the HSAs in the area where they come from, some that administer contraception door to door and some come to the hospital. Where do you often get yours?

RESPONDENT: aaah for I like coming here at the hospital

INTERVIEWER: mmmh.

RESPONDENT: yes

INTERVIEWER: All the time?

RESPONDENT: yes, I always come here I have never gone to HSAs before.

INTERVIEWER: okay. What means of transport do you use from where you stay to get here?

RESPONDENT: Motor cycle

INTERVIEWER: How long does it take to get here?

RESPONDENT: The motor cycle?

INTERVIEWER: mmmmh. Let us assume you started at …What time do you start from home?

RESPONDENT: From home I start at …like today I started at half past seven, and we got here eight o’clock.

INTERVIEWER: Eight o’clock?

RESPONDENT: Yes

Title: KK_SCSI_2.docx

Descriptor Info:	District: Nkhotakota	Target Population: TP3 - DMPA-SC SI clients	Respondent type: DMPA-SC SI client	Aware of SI: Yes - had heard of SI before	Coding phase: 1. Single coded	Client marital status: Divorced/ separated/ widowed	Client age group: 20-29 years	Time since trained: N/A - client

Codes Applied:	C0.4 - Access to or process of obtaining FP services

INTERVIEWER: Alright thanks, Has the ease/ difficult of accessing FP services changed since the outbreak of corona virus?

RESPONDENT: No

Title: KK_SCPI_4.docx

Descriptor Info:	District: Nkhotakota	Target Population: TP2 - DMPA-IM/DMPA-SC PI clients	Respondent type: DMPA-SC PI client	Aware of SI: Yes - had heard of SI before	Coding phase: 2. Double coded	Client marital status: Never married	Client age group: 20-29 years	Time since trained: N/A - client

Codes Applied:	C0.4 - Access to or process of obtaining FP services

INTERVIEWER: wh…Where were you receiving them?

RESPONDENT: At the hospital.

INTERVIEWER: Hospital?

RESPONDENT: mmmh.

Title: KK_SCPI_4.docx

Descriptor Info:	District: Nkhotakota	Target Population: TP2 - DMPA-IM/DMPA-SC PI clients	Respondent type: DMPA-SC PI client	Aware of SI: Yes - had heard of SI before	Coding phase: 2. Double coded	Client marital status: Never married	Client age group: 20-29 years	Time since trained: N/A - client

Codes Applied:	C0.4 - Access to or process of obtaining FP services

INTERVIEWER: Now, I just want to know how easy or difficult it was for you to access contraceptive methods in the past.

RESPONDENT: yea.

INTERVIEWER: Was it difficult or easy for you to access contraceptions in the past?

RESPONDENT: These others?

INTERVIEWER: mmmh.

RESPONDENT: Yes, we knew that we were girls who had never given birth before, so how could we get these other contraceptions; we decided it was better for us to use condoms.

INTERVIEWER: okay.

RESPONDENT: yea.

INTERVIEWER: Now about these condoms, were you struggling to find them, or how was it?

RESPONDENT: After finding these condoms?

INTERVIEWER: No. to find them

RESPONDENT: For us to find them?

INTERVIEWER: mmmh.

RESPONDENT: We had problems to find them at home, but when we come to the hospital, we could ask the doctors for condoms. Some understanding doctors would give us.

INTERVIEWER: mmmhu.

RESPONDENT: yea.

INTERVIEWER: Being at home, where would you get the condoms?

RESPONDENT: When at home? On that one, I would not know. Sometimes the boyfriend would bring (laughs).

INTERVIEWER: Now, you have told me that you could find them easily, right?

RESPONDENT: Yes

INTERVIEWER: You could find them at the hospital.

RESPONDENT: eeh. Yes.

INTERVIEWER: You were collecting them at the hospital.

RESPONDENT: Yes

INTERVIEWER: What are the things that you think were making it easy for you to access the condoms here at the hospital? Whenever you came here at the hospital, what are the things that were helping you to easily access these condoms?

RESPONDENT: We could go like here at the hospital…we used to come to the hospital frequently and complain to the doctors. Some could not accept…they were denying us the access to condoms.

INTERVIEWER: Okay, they were denying giving you?

RESPONDENT: As it is here at the hospital. As there are groceries, they say for you to buy the condoms and you are found, you will arrested.

INTERVIEWER: Who said that?

RESPONDENT: People were saying that, so you could just listen to what your friends are discussing.

INTERVIEWER: Okay, what were they really saying about the hospital?

RESPONDENT: Whenever we got here at the hospital, we could simply ask for the condoms.

INTERVIEWER: mmmhu.

RESPONDENT: Mainly, we were able to have access the condoms; we were accessing them as a family planning method. When they give you whether the injection, they were also giving you the condoms for you to be using as well.

INTERVIEWER: How about Depo, it means previously you were on…

RESPONDENT: yea.

INTERVIEWER: Was it difficult or easy for you to access Depo?

RESPONDENT: It was difficult.

INTERVIEWER: why?

RESPONDENT: As I have already stated, that for you a woman to go and ask for condoms at the hospital, we were shy on how to do it.

INTERVIEWER: mmmmhu. Alright, how about injection?

RESPONDENT: Injection?

INTERVIEWER: The one for here

RESPONDENT: Injection? They were also asking and we were giving out cards.

INTERVIEWER: Were you able to accessit easily or there were difficulties?

RESPONDENT: The access was easy, but sometimes the stock was not available but whenever the stock was available, access was easy.

INTERVIEWER: Whenever the stock was not available, what were you doing?

RESPONDENT: Whenever they told us that the stock was not available.

INTERVIEWER: mmmhu.

RESPONDENT: We could just go back home. Sometimes when condoms were scarce, it meant that nothing would happen in the house when the woman refuses (laughs).

INTERVIEWER: okay. Would you tell me; how often have you been sent back on condition that there was no injective contraception?

RESPONDENT: The times I have been sent back, here at Malowa Hospital, I have never been sent back here. It has always been available, because this is the third one.

INTERVIEWER: where else?

RESPONDENT: The other one, the first one whatever they called it. It had a name. I also just took three.

INTERVIEWER: The injectable one… Depo?

RESPONDENT: I think it is that one, Depo, the old one.

INTERVIEWER: The one they inject on the arm.

RESPONDENT: eya eeh. Yes.

INTERVIEWER: okay. You were getting it at this hospital or another hospital.

RESPONDENT: mmmh. Yes, there is another hospital where we were staying, its Mpamantha hospital.

INTERVIEWER: That is where you were sent sent back?

RESPONDENT: They started by saying the stock is not enough, because there were several people in need of the same.

INTERVIEWER: So they were sending you back.

RESPONDENT: No, we managed to get it

INTERVIEWER: You took

RESPONDENT: Depending on whose card was collected first.

INTERVIEWER: okay. some of your friends were sent back?

RESPONDENT: They also took; I would not know what happened after I had left. Because once you are assisted, you go straight home.

Title: KK_SCPI_4.docx

Descriptor Info:	District: Nkhotakota	Target Population: TP2 - DMPA-IM/DMPA-SC PI clients	Respondent type: DMPA-SC PI client	Aware of SI: Yes - had heard of SI before	Coding phase: 2. Double coded	Client marital status: Never married	Client age group: 20-29 years	Time since trained: N/A - client

Codes Applied:	C0.4 - Access to or process of obtaining FP services

INTERVIEWER: mmmhu. Now, we know that there is COVID-19 pandemic in the world. Now since COVID-19 started, do you think there is a change in the availability of family planning methods?

RESPONDENT: No.

INTERVIEWER: No change?

RESPONDENT: None, not even a thing.

INTERVIEWER: Everything is the same?

RESPONDENT: mmmmh.

Title: KK_SCPI_4.docx

Descriptor Info:	District: Nkhotakota	Target Population: TP2 - DMPA-IM/DMPA-SC PI clients	Respondent type: DMPA-SC PI client	Aware of SI: Yes - had heard of SI before	Coding phase: 2. Double coded	Client marital status: Never married	Client age group: 20-29 years	Time since trained: N/A - client

Codes Applied:	C0.4 - Access to or process of obtaining FP services

INTERVIEWER: Can you explain to me how you access the injection in every three months? How do you access it?

RESPONDENT: We get it here at the hospital, when we come.

INTERVIEWER: Before you here at the hospital….before you come here, aah let us say what did you do yesterday in preparation of coming to the hospital today?

RESPONDENT: lero mdapita kuchipatala Today when going to the hospital.

INTERVIEWER: Yesterday.

RESPONDENT: Yes, I have understood.

INTERVIEWER: You have understood right?

RESPONDENT: yes. I just slept (laughs)

INTERVIEWER: That is all

RESPONDENT: What else can you do the contraception is worn off. We just sleep because [not clear] it’s a woman right. Or you will pretend to have menstruated, it means you will not do.

INTERVIEWER: okay. Before you go for an injection the following day, do you prepare yourself in any way?

RESPONDENT: No

INTERVIEWER: No?

RESPONDENT: Yes

INTERVIEWER: May be taking care of things at home.

RESPONDENT: eeeh kumagwiragwirako tintchito eeh mmawa ndipita ku Of course, just a few things given that tomorrow you are going to the hospital.

INTERVIEWER: What do you do?

RESPONDENT: We do the usual house chores. We do the chores for us to just mind about going to the hospital in the morning. More especially for us, there is a distance between where we come from and where this hospital is, so we start very early whether on foot.

INTERVIEWER: How do you remember that it is time for you to take another injection?

RESPONDENT: They tell us here at the hospital.

INTERVIEWER: They tell you.

RESPONDENT: mmmmh.

INTERVIEWER: They tell you at the hospital but when you get home, you stay for three months.

RESPONDENT: Yes

INTERVIEWER: So how do you remember?

RESPONDENT: We remember based on the card.

INTERVIEWER: On the card?

RESPONDENT: When they tell us the date, for example 12 October you need to come or on 20 October, then we remember the month they told us.

INTERVIEWER: So where you frequently go for your injections. There are other health personnel, who go door to door, right, whether here at the hospital or elsewhere. Where do you regularly go for your injections?

RESPONDENT: As it is here, there are no health surveillance assistants. They are there but maybe they have not started working. Because when I went for under-five clinic with my baby, they said there are new HSAs to start working, but they are still looking for houses but for now, houses are not available. Therefore, we walk long distances to access contraceptions.

INTERVIEWER: Now, you have told me that you come from a long distance away from here, so how do you travel to get here?

RESPONDENT: The different ways?

INTERVIEWER: How do you move to get here?

RESPONDENT: If you have money, you can hire a motorcycle, if you do not have money, you borrow a bike, if the bike cannot be found you just walk.

INTERVIEWER: okay.

RESPONDENT: Yes

INTERVIEWER: As of today, I have walked all the way from home to here.

RESPONDENT: Let us assume, you started at 8 AM walking what time would you get here?

INTERVIEWER: We have phones that we can be checking the time, so when we get here, we just walk into the hospital.

RESPONDENT: nevertheless, yes, the distance is there

INTERVIEWER: Do you sometimes know what time you have started for the hospital?

RESPONDENT: Like for today, I would say I started when the sun was already up, may be around seven depending on the time I wake up. Then I was just walking along the way.

Title: ZA_IM_6.docx

Descriptor Info:	District: Zomba	Target Population: TP2 - DMPA-IM/DMPA-SC PI clients	Respondent type: DMPA-IM client	Aware of SI: Yes - had heard of SI before	Coding phase: 1. Single coded	Client marital status: Married	Client age group: 30-39 years	Time since trained: N/A - client

Codes Applied:	C0.4 - Access to or process of obtaining FP services

Interviewer: Alright, thank you so much. How hard or easy has it been for you to find family planning methods?

Respondent: No, it has not been hard.

Interviewer: Where do you get your family planning methods?

Respondent: Here in Matawale.

Interviewer: Mmhhmm you have said it is easy to access the methods. What makes it so easy?

Respondent: Because I come to a public(government) hospital.

Interviewer: Mmhhmm

Respondent: So, it is available most of the times.

Interviewer: Alright.

Respondent: Because every time I come, I have never returned due to unavailability of the injection.

Interviewer: Mhhmm

Respondent: I find it.

Interviewer: Mmhhm Alright, is there anything else you would like to add? Ok you have said it is easy to access the injection, right?

Respondent: Mhhmm

Interviewer: Since the COVID outbreak has there been any changes in accessing family planning methods?

Respondent: No.

Interviewer: Are you offered help every time you come to this hospital?

Respondent: Yes, but due to fear a lot of people do not come.

Interviewer: They do not come?

Respondent: Mhhmm they say if they come, they might be injected with a COVID vaccine.

Interviewer: Mmhhmm

Respondent: They will not get the contraceptive injection, but I still come.

Interviewer: Alright, is the help sufficient?

Respondent: Yes, it is sufficient.

Title: ZA_IM_6.docx

Descriptor Info:	District: Zomba	Target Population: TP2 - DMPA-IM/DMPA-SC PI clients	Respondent type: DMPA-IM client	Aware of SI: Yes - had heard of SI before	Coding phase: 1. Single coded	Client marital status: Married	Client age group: 30-39 years	Time since trained: N/A - client

Codes Applied:	C0.4 - Access to or process of obtaining FP services

Interviewer: Alright thank you so much. Could you explain how you access the injection after every three months? Do you have to prepare anything before you come to the hospital?

Respondent: What sought of preparation? I just know the day to get my injection then I come here.

Interviewer: Like house chores, are you supposed to do them all before you get here, at the hospital?

Respondent: Yes! I start with doing dishes then mopping and drawing water.

Interviewer: Mhhmm

Respondent: And then get here.

Interviewer: How about the piece works?

Respondent: Like today, I started with the piece work and then came here.

Interviewer: Mhhmm

Respondent: Mmhhm

Interviewer: Alright. Where do you usually get your injection?

Respondent: Here in Matawale.

Interviewer: Owoo! As you said your home is not far from here?

Respondent: Yes, it is not far, its Chinamwali.

Interviewer: Mmhhm, how to you travel to get here.

Respondent: We walk then find the tarmac road.

Interviewer: No, do you walk or come on a bicycle?

Respondent: Oh! I walk.

Interviewer: How long does it take you to get here?

Respondent: Iii a couple of minutes.

Interviewer: For instance, how many minutes?

Respondent: If I leave home at 9 o’clock I could get here around half 9.

Interviewer: Mhhm

Respondent: Mhhmm

Title: KK_SCSI_3.doc

Descriptor Info:	District: Nkhotakota	Target Population: TP3 - DMPA-SC SI clients	Respondent type: DMPA-SC SI client	Aware of SI: Yes - had heard of SI before	Coding phase: 1. Single coded	Client marital status: Married	Client age group: 20-29 years	Time since trained: N/A - client

Codes Applied:	C0.4 - Access to or process of obtaining FP services

INTERVIEWER: yeah, alright so before you started using Sayana, how difficult was it for you to access family planning methods?

RESPONDENT: nothing was difficult

INTERVIEWER: nothing was difficult for you everything was just smooth... it wasn't difficult?

RESPONDENT: (laughs)

INTERVIEWER: So... So what are the factors that helped that you should easily get these methods?

RESPONDENT: I didn't get the question, repeat

INTERVIEWER: you have told me that you didn't face any problems when accessing these family planning methods... you have told me about Depo, Norplant, pills right? So what assisted so that you should be able to access these methods with easiness?

RESPONDENT: aaa these methods are easy for me because I used other methods before and when they failed, it's when I decided to be using the one I am using currently, and on this I am not facing any problems.

INTERVIEWER: it's not hard?

RESPONDENT: mmmmm

INTERVIEWER: okay, for you to get the pills, implant the Norplant, inject Depo, was it difficult for you for you to get the injection or get the pills, was it difficult?

RESPONDENT: no

INTERVIEWER: no, what was helping? Mmmmm, what was helping for these methods to be easily accessible? What was helping?

RESPONDENT: (laughs) I have failed to answer this question [she did not understand this question although I tried to probe and ask in different ways].

INTERVIEWER: you have failed. we all know that there is Covid, so ever since Covid outbreak, have you noticed any change in the accessing of family planning methods?

RESPONDENT: there's a change

INTERVIEWER: what changed?

RESPONDENT: Because in the past we will get the injection and actually meet up with the nurse without a face mask whilst now they're saying you should have a face mask before meeting up with the doctor.

INTERVIEWER: okay

RESPONDENT: yes

INTERVIEWER: but were you accessing the family planning services with the outbreak?

RESPONDENT: yes

INTERVIEWER: there was no change at all?

RESPONDENT: no.

Title: ZA_SCPI_3.docx

Descriptor Info:	District: Zomba	Target Population: TP2 - DMPA-IM/DMPA-SC PI clients	Respondent type: DMPA-SC PI client	Aware of SI: No - never heard of SI before	Coding phase: 3. Triple coded	Client marital status: Never married	Client age group: Under 20 (adolescent)	Time since trained: N/A - client

Codes Applied:	C0.4 - Access to or process of obtaining FP services

Interviewer: Alright, ah where were you accessing these methods from, these condoms and pills where were you getting them from?

Respondent: The condoms and pills I was getting them..., we have this other organization named YONECO, MACRA,

Interviewer: Yes.

Respondent: Yes, when they come, they would give us.

Interviewer: Alright.

Respondent: mmm (showing agreement)

Interviewer: What about the pills?

Respondent: All of those.

Interviewer: Oh, they were giving you all of these?

Respondent: Yes, they were.

Title: ZA_SCPI_3.docx

Descriptor Info:	District: Zomba	Target Population: TP2 - DMPA-IM/DMPA-SC PI clients	Respondent type: DMPA-SC PI client	Aware of SI: No - never heard of SI before	Coding phase: 3. Triple coded	Client marital status: Never married	Client age group: Under 20 (adolescent)	Time since trained: N/A - client

Codes Applied:	C0.4 - Access to or process of obtaining FP services

Interviewer: I just wanted to know if there are any problems that you have faced…

Respondent: mmm (showing that they are following the conversation)

Interviewer: In trying to access the family planning methods that you have mentioned to me…

Respondent: mmm (showing that they are following the conversation)

Interviewer: Yah

Respondent: There were not any problems.

Interviewer: Alright, ah let’s just assume, the way you have told me that the pills and condoms were given to you by YONECO or MACRO…

Respondent: mmm (showing that they are following the conversation)

Interviewer: Whenever they came in your area…

Respondent: mmmm (showing that they are following the conversation)

Interviewer: Were there any other factors that were making this easy?

Respondent: Yes

Interviewer: What factors?

Respondent: …. (a pause) eh these factors…

Interviewer: The factors that were making it easy to find these methods…

Respondent: The facility is very close to us…

Interviewer: Oh alright…

Respondent: Yes…

Interviewer: Alright, before you turned 18, was it easy or difficult to access family planning methods?

Respondent: It was difficult.

Interviewer: How difficult was it?

Respondent: That time I was too young, and I didn’t know what I was doing…

Title: ZA_SCPI_3.docx

Descriptor Info:	District: Zomba	Target Population: TP2 - DMPA-IM/DMPA-SC PI clients	Respondent type: DMPA-SC PI client	Aware of SI: No - never heard of SI before	Coding phase: 3. Triple coded	Client marital status: Never married	Client age group: Under 20 (adolescent)	Time since trained: N/A - client

Codes Applied:	C0.4 - Access to or process of obtaining FP services

Interviewer: Alright, can you explain to me how you find injectable contraceptive in every three months?

Respondent: Yes

Interviewer: can you explain to me how you find them?

Respondent: (trying to think around the question)

Interviewer: Where do you find them, is it from HSAs, or maybe you come at the facility?

Respondent: sometimes I find them here at the facility.

Interviewer: mmm (showing that one is following the conversation)

Respondent: But mostly in my community I go to the HSAs.

Interviewer: Okay.

Respondent: mmmm

Interviewer: Alright how do you remember that the dates for you to reinject have come, how do you remember?

Respondent: ah when they inject us, they tell us the day that we are supposed to come back for reinjection.

Interviewer: Okay.

Respondent: Yes.

Interviewer: Alright, when you are coming at the hospital to get the injection or maybe you want to meet the HSAs, do you have to prepare in any way, for example that you won’t have to go to school, does this happen?

Respondent: No, it does not happen, I try to arrange a day prior, say Saturday…

Interviewer: Yes.

Respondent: so, we find that we are going to the HSAs.

Interviewer: mmm (showing that one is following the conversation)

Respondent: in the late afternoon

Interviewer: mmm (showing that one is following the conversation)

Respondent: Yes.

Interviewer: Alright, where do you usually get your injection from?

Respondent: From HSAs

Interviewer: From HSAs?

Respondent: mmm (agreeing)

Interviewer: Okay, how do you get there?

Respondent: Transportation?

Interviewer: Yes, what do you use for transportation, how long does it take to get there?

Respondent: It’s not that far…

Interviewer: Okay.

Respondent: Where we go…

Title: MZs_IM_5.docx

Descriptor Info:	District: Mzimba South	Target Population: TP2 - DMPA-IM/DMPA-SC PI clients	Respondent type: DMPA-IM client	Aware of SI: Yes - had heard of SI before	Coding phase: 1. Single coded	Client marital status: Married	Client age group: 20-29 years	Time since trained: N/A - client

Codes Applied:	C0.4 - Access to or process of obtaining FP services

Interviewer: when you are here at the facility, how easy was it to access family planning methods?

Respondent: it was difficult

Interviewer: how difficult?

Respondent: most of the times they said it was out of stock

Interviewer: anything else?

Respondent: no

Interviewer: has covid-19 affected the accessibility of family planning methods?

Respondent: we were sent back and given condoms because they discouraged gatherings. But after the ban was lifted everything went on as we used to

Title: MZs_IM_5.docx

Descriptor Info:	District: Mzimba South	Target Population: TP2 - DMPA-IM/DMPA-SC PI clients	Respondent type: DMPA-IM client	Aware of SI: Yes - had heard of SI before	Coding phase: 1. Single coded	Client marital status: Married	Client age group: 20-29 years	Time since trained: N/A - client

Codes Applied:	C0.4 - Access to or process of obtaining FP services

Interviewer: how did you access this method?

Respondent: I came to the health facility. I told the provider that I wanted the 3 months’ interval. He asked me question like why I had taken the family planning method and if I came with my spouse. We had come together.

Interviewer: do you have any reminders on the next dose?

Respondent: they record a date for next appointment

Interviewer: do you prepare for anything before coming to get your next dose?

Respondent: I sweep the surrounding, mop the house, and wash clothes. That’s when I come here

Interviewer: where do you get Depo?

Respondent: at the health facility they then changed that we should self-inject with coming of Sayana

Interviewer: have you ever accessed it from your HSA?

Respondent: no I used to come here

Interviewer: what mode of transport did you use to come here?

Respondent: I walk

Interviewer: what happens when you come to the health facility?

TIME STAMP 21:23MIN-32:50MIN

Respondent: when I arrive, I found other women already here, so I sit on then chair. Then when the clinic starts, we enter the room one by one

Interviewer: how long does it take for the provider to assist you?

Respondent: sometimes I find that they have already started. They start the clinic at around 8 am

Interviewer: what do they say when you are here?

Respondent: most of the times they start the clinic late we wait for them to do other things. When she starts the clinic, they assist us one by one counselling

Title: ZA_SCSI_3.docx

Descriptor Info:	District: Zomba	Target Population: TP3 - DMPA-SC SI clients	Respondent type: DMPA-SC SI client	Aware of SI: Yes - had heard of SI before	Coding phase: 3. Triple coded	Client marital status: Married	Client age group: 40-49 years	Time since trained: N/A - client

Codes Applied:	C0.4 - Access to or process of obtaining FP services	C2.1.1 - Community availability of injectables/SI

Interviewer: Fine. Have you ever used the injection method at the hospital before starting self-injection?

Respondent: Yes.

Interviewer: Where were you receiving this method?

Respondent: In Matawale.

Interviewer: Matawale?

Respondent: Here at the hospital in Matawale.

Interviewer: So you got the methods here at the hospital?

Respondent: Yes.

Interviewer: How long did you use the injection method before you started self-injecting?

Respondent: I have used it for 6 years

Interviewer: In the past, before you started using the self-injecting method, how easy or hard was it to access the family planning method?

Respondent: To my part, it was hard because sometimes we’d come here at the hospital and stay for a long time. When we gave this complaint to the HSA’s, they told us that they would start bringing the injections to our different areas when they come to deliver antenatal services. So what we did was that each one of us would keep their date and when the HSA’s come to deliver the antenatal services, we’d go there to get our injections.

Interviewer: Okay thank you very much. Has there been any change in the availability of the family planning method with the coming of COVID 19?

Respondent: No, since we would just go right where we go when accessing antenatal services to get the method, so it has had no effect in the way we access the method. In addition, we are a fewer number of people who go there.

Title: MZs_IM_6.docx

Descriptor Info:	District: Mzimba South	Target Population: TP2 - DMPA-IM/DMPA-SC PI clients	Respondent type: DMPA-IM client	Aware of SI: Yes - had heard of SI before	Coding phase: 1. Single coded	Client marital status: Married	Client age group: Under 20 (adolescent)	Time since trained: N/A - client

Codes Applied:	C0.4 - Access to or process of obtaining FP services	C1.1.3 - Awareness of DMPA-SC SI

Interviewer: Thank you so much. Now we would like to talk about what you experienced when using this family planning method. Apart from the one you use (provider-injected) have you ever used other methods of family planning methods?

Respondent: Mmmmmh no

Interviewer: The one which you were injected? Where did you get it from?

Respondent: Here at the health facility

Interviewer: Here at the facility? how easy or difficult has it been for you to access this family planning method you use; I mean Depo?

Respondent: Come again?

Interviewer: Has it been easy? Or difficult?

respondent: No, it is not difficult

Interviewer: What factors made it easy for you to access Depo?

Respondent: It’s because the providers are readily available here at the health facility and they provide the services at any time you come

Interviewer: Did the coming of covid-19 have an impact on the accessibility of family planning services?

Respondent: With the coming of covid-19 we were told that each one of us should start self-injecting of-course am not very sure if that was the main reason

Title: MZs_IM_6.docx

Descriptor Info:	District: Mzimba South	Target Population: TP2 - DMPA-IM/DMPA-SC PI clients	Respondent type: DMPA-IM client	Aware of SI: Yes - had heard of SI before	Coding phase: 1. Single coded	Client marital status: Married	Client age group: Under 20 (adolescent)	Time since trained: N/A - client

Codes Applied:	C0.4 - Access to or process of obtaining FP services	C0.2.1 - Positive community/client perspectives on FP

Interviewer: What made you choose intramuscular Depo Provera?

Respondent: I was influenced by my neighbor who uses same method and she encouraged me to use the same method

Interviewer: What method did you say you use? Provider injected or you inject yourself?

Respondent: Sayana….[stammer]…Depo... [giggles] it’s the provider who inject every 3 months

Interviewer: Every 3 months?

Respondent: Yes

Interviewer: What is it that you like on this method that you are using?

Respondent: What I like? Ever since I started using this method, I have never encountered any problem like getting pregnant or any other problem, so it is a good method

Interviewer: What else did you like about this method?

Respondent: What else?

Interviewer: Yes. If any.

Respondent: I do not experience any side effect every time I get injected

Interviewer: For how long have you used this method?

Respondent: Almost a year now

Interviewer: A year?

Respondent: Mmmmh (agreeing)

Title: MZs_IM_6.docx

Descriptor Info:	District: Mzimba South	Target Population: TP2 - DMPA-IM/DMPA-SC PI clients	Respondent type: DMPA-IM client	Aware of SI: Yes - had heard of SI before	Coding phase: 1. Single coded	Client marital status: Married	Client age group: Under 20 (adolescent)	Time since trained: N/A - client

Codes Applied:	C0.4 - Access to or process of obtaining FP services	C3.3 - Counselling / training approach for injectable users

Interviewer: Would you tell me how you accessed this method you are using?

Respondent: How I accessed it?

Interviewer: Mmmmh (agreeing)

Respondent: When we reach the facility…[stammer] the provider explained to us the pros and cons of the family planning method then they inject me and record in my health passport book and give me the next date of appointment. When I am due, I come for subsequent doses.

Interviewer: When did you start receiving the first dose if you can recall

Respondent: It was last year I think in November.

Interviewer: Before coming to the health facility to receive services did you do anything at home?

Respondent: Concerning what?

Interviewer: Concerning your household. What did you do before coming to the facility?

Respondent: I do household chores. Then I take a bathe that’s when I come here.

Interviewer: How do you come here?

Respondent: I use a hired bicycle because it’s a bit far from where I come from

Interviewer: How long does it take for you to travel from your home to this facility

Respondent: Mmmmmh (thinking out loud) about 30 minutes when I used the hired bicycle

Interviewer: Mmmmh (thinking out loud) what happens when you come here to get the family planning method you use?

Respondent: They start with health talk. after that they administer

Interviewer: Would you explain detail what happens from the time you arrive

Respondent: When we arrive, they greet us. We also greet them. Then we give them the health passport book then they check, and they then administer

TIME STAMP 2: FROM 10 MINUTES 13SEC

Interviewer: Mmmhu (agreeing) when they are giving you a talk, do they spend enough time with you?

Respondent: Yes, they take time and ask questions as well

Interviewer: Mmmhu (agreeing) how many minutes do they take

Respondent: From the time they start the clinic?

Interviewer: Mmmh (agreeing)

Respondent: Most of them it takes them hours

Title: MZs_IM_6.docx

Descriptor Info:	District: Mzimba South	Target Population: TP2 - DMPA-IM/DMPA-SC PI clients	Respondent type: DMPA-IM client	Aware of SI: Yes - had heard of SI before	Coding phase: 1. Single coded	Client marital status: Married	Client age group: Under 20 (adolescent)	Time since trained: N/A - client

Codes Applied:	A4 - Adol access to FP services	C0.4 - Access to or process of obtaining FP services

Interviewer: At what age did you start using family planning method?

Respondent: At 16 years’ old

Interviewer: How easy or difficult was it for you to come at the facility then?

Respondent: It was easy for me [giggles]

Interviewer: What made it easy?

Respondent: I just decided and got confident to come and access the family planning services

Interviewer: Were you judged for coming her at 16 years’ old?

Respondent: No

Title: ZA_IM_4.docx

Descriptor Info:	District: Zomba	Target Population: TP2 - DMPA-IM/DMPA-SC PI clients	Respondent type: DMPA-IM client	Aware of SI: Yes - had heard of SI before	Coding phase: 1. Single coded	Client marital status: Married	Client age group: Under 20 (adolescent)	Time since trained: N/A - client

Codes Applied:	C0.4 - Access to or process of obtaining FP services

Interviewer: Mmh alright thank you very much. How has it been difficult or easy to find this family planning method back then?

Respondent: It has always been easier

Interviewer: Mhhh

Respondent: Because we find everything here at the hospital

Interviewer: Mhhh alright ever found this kind of method of family planning in other areas?

Respondent: Mmhh mmh

Interviewer: All the time you find it here at the hospital? Alright so what really helped and made you find this family planning easier?

Respondent: We find it here when come with a baby for postnatal check up

Interviewer: Mhhh

Respondent: So it’s easier

Interviewer: Uhm alright thank you very much. is there any change on how you can access family planning methods since corona virus started?

Respondent: No

Interviewer: Alright everything goes as it was?

Respondent: Mhh

Interviewer: Ever came back without being injected?

Respondent: Mhh never came back (baby talks)

Title: ZA_IM_4.docx

Descriptor Info:	District: Zomba	Target Population: TP2 - DMPA-IM/DMPA-SC PI clients	Respondent type: DMPA-IM client	Aware of SI: Yes - had heard of SI before	Coding phase: 1. Single coded	Client marital status: Married	Client age group: Under 20 (adolescent)	Time since trained: N/A - client

Codes Applied:	C0.4 - Access to or process of obtaining FP services

Interviewer: The nurses were not saying anything? Alright thank you very much. What encouraged you to start using this injection method?

Respondent: Because we heard from the people who already started it that it’s one of the easiest method and that is why we also started it.

Interviewer: Alright what do you really like on this injection method?

Respondent: like what?

Interviewer: What do you really love about this self-injectable family planning method? (Sayana)

Respondent: This is because during the time we come (Baby screams) here to this hospital we also have other things to do so if there is this particular injection means the time we wasted coming here we would have been spent on other things.

Title: ZA_IM_4.docx

Descriptor Info:	District: Zomba	Target Population: TP2 - DMPA-IM/DMPA-SC PI clients	Respondent type: DMPA-IM client	Aware of SI: Yes - had heard of SI before	Coding phase: 1. Single coded	Client marital status: Married	Client age group: Under 20 (adolescent)	Time since trained: N/A - client

Codes Applied:	C0.4 - Access to or process of obtaining FP services

Interviewer: Eeeeh alright alright for how long have you been using this injection method?

Respondent: Not for very long only 3 times

Interviewer: Mhh alright may you explain how you find this injection method of family planning during 3 months?

Respondent: How we find?

Interviewer: The injection

Respondent: Mhu

Interviewer: During every 3 months are you supposed to get prepared for everything before the injection?

Respondent: Mmm

Interviewer: How do you get prepared?

Respondent: When the day has passed

Interviewer: Ah the day that you are supposed to come for the injection. What kind of chores do you do? at home?

Respondent: We clean the dishes

Interviewer: Mmmh

Respondent: Mopping

Interviewer: Mmh

Respondent: We draw water and take a shower

Interviewer: Mhhh the baby?

Respondent: We bath the baby then come here

Interviewer: Mmh Alright so where do you frequently get this injection method of family planning?

Respondent: Here at the hospital

Interviewer: What kind of transport do you use when coming here?

Respondent: By foot

Interviewer: How much time does it take when you are coming from area?

Respondent: Maybe 30minutes

Interviewer: You walk all the times?

Respondent: Mhhh we walk all the times

Title: ZA_SCSI_5.docx

Descriptor Info:	District: Zomba	Target Population: TP3 - DMPA-SC SI clients	Respondent type: DMPA-SC SI client	Aware of SI: Yes - had heard of SI before	Coding phase: 3. Triple coded	Client marital status: Married	Client age group: 30-39 years	Time since trained: N/A - client

Codes Applied:	C0.4 - Access to or process of obtaining FP services

Interviewer: Did you access this method from the facility or from the HSAs?

Respondent: I accessed it from the HSAs

Title: ZA_SCSI_5.docx

Descriptor Info:	District: Zomba	Target Population: TP3 - DMPA-SC SI clients	Respondent type: DMPA-SC SI client	Aware of SI: Yes - had heard of SI before	Coding phase: 3. Triple coded	Client marital status: Married	Client age group: 30-39 years	Time since trained: N/A - client

Codes Applied:	C0.4 - Access to or process of obtaining FP services

Interviewer: Thank you. Ah this provider administered injectable, did you access it from the facility or from the HSA?

Respondent: No I accessed it from the facility.

Title: ZA_SCSI_5.docx

Descriptor Info:	District: Zomba	Target Population: TP3 - DMPA-SC SI clients	Respondent type: DMPA-SC SI client	Aware of SI: Yes - had heard of SI before	Coding phase: 3. Triple coded	Client marital status: Married	Client age group: 30-39 years	Time since trained: N/A - client

Codes Applied:	C0.4 - Access to or process of obtaining FP services	C0.3 - Client previous use/experience of any FP method

Interviewer: Alright, in the past before you started using DMPA SC for self-injection, how difficult or easy was it to access contraceptives?

Respondent: Sayana?

Interviewer: Before you started using Sayana

Respondent: Mmm (showing that one is following the conversation)

Interviewer: How difficult was it to access family planning methods in the past?

Respondent: Ah it was not difficult.

Interviewer: Mmm (showing that one is following the conversation)

Respondent: Eh that’s why I would just change among the methods.

Interviewer: Mmm (showing that one is following the conversation)

Respondent: Change again

Interviewer: Alright.

Respondent: Yes, so when I started using this one that’s when I decided that I will be using this one

Title: ZA_SCSI_5.docx

Descriptor Info:	District: Zomba	Target Population: TP3 - DMPA-SC SI clients	Respondent type: DMPA-SC SI client	Aware of SI: Yes - had heard of SI before	Coding phase: 3. Triple coded	Client marital status: Married	Client age group: 30-39 years	Time since trained: N/A - client

Codes Applied:	C0.4 - Access to or process of obtaining FP services	C0.3 - Client previous use/experience of any FP method

Interviewer: Alright, can you explain to me other difficult things that you were experiencing when you were using the provider administered injectables, not the problems in your body but maybe logistical issues, can you explain to me?

Respondent: Yes, for Depo it was difficult.

Interviewer: Mmm (showing that one is following the conversation)

Respondent: Because when the months were getting closer.

Interviewer: Mmm (showing that one is following the conversation)

Respondent: Oh they gave me this date so I should rush.

Interviewer: Mmm (showing that one is following the conversation)

Respondent: And sometimes I would forget the date.

Interviewer: Mmm (showing that one is following the conversation)

Respondent: What was the date that they gave me, when I came here, they would send me back saying that my date is not yet due.

Interviewer: Mmm (showing that one is following the conversation)

Respondent: So I was abandoning chores at home

Interviewer: Mmm (showing that one is following the conversation)

Respondent: And coming here just to go back home

Interviewer: Mmm (showing that one is following the conversation)

Respondent: So from that I saw that its that I change

Interviewer: Mmm (showing that one is following the conversation)

Respondent: I should start using Sayana

Interviewer: Mmm (showing that one is following the conversation)

Respondent: Because that one was getting me busy

Title: ZA_SCSI_5.docx

Descriptor Info:	District: Zomba	Target Population: TP3 - DMPA-SC SI clients	Respondent type: DMPA-SC SI client	Aware of SI: Yes - had heard of SI before	Coding phase: 3. Triple coded	Client marital status: Married	Client age group: 30-39 years	Time since trained: N/A - client

Codes Applied:	C0.4 - Access to or process of obtaining FP services

Interviewer: Since the coronavirus disease started, have you seen any difficulties or no difficulties in accessing contraceptive methods?

Respondent: Ah I don’t see any difficulties.

Interviewer: Mmm (showing that one is following the conversation)

Respondent: When we meet here, maybe I have not worn a mask, maybe you have forgotten the mask

Interviewer: Mmm (showing that one is following the conversation)

Respondent: And other women have not worn a mask, so I see that as a problem.

Title: MZs_IM_7.docx

Descriptor Info:	District: Mzimba South	Target Population: TP2 - DMPA-IM/DMPA-SC PI clients	Respondent type: DMPA-IM client	Aware of SI: Yes - had heard of SI before	Coding phase: 1. Single coded	Client marital status: Divorced/ separated/ widowed	Client age group: Under 20 (adolescent)	Time since trained: N/A - client

Codes Applied:	C0.4 - Access to or process of obtaining FP services

Interviewer: how easy or difficult was it to access family planning methods at your age?

Respondent: the one I am using

Interviewer: you said you are using Depo, right?

Respondent: yes

Interviewer: how easy or difficult was it to access Depo?

Respondent: I met the provider

Interviewer: where did you meet?

Respondent: right here at our community

Interviewer: what made it easily accessible from the provider?

Respondent: for me I did not have challenges. When I arrived, I met the provider then he administered.

Interviewer: when you started using it at the age of 18, how easy or difficult was it to access family planning methods?

Respondent: my friends had already tipped me

Interviewer: they tipped you?

Respondent: yes

Interviewer: so where did you access the family planning method?

Respondent: right here from the provider

Interviewer: was it easy to access it at the age of 18?

Respondent: yes, I did not have any challenges

Title: MZs_IM_7.docx

Descriptor Info:	District: Mzimba South	Target Population: TP2 - DMPA-IM/DMPA-SC PI clients	Respondent type: DMPA-IM client	Aware of SI: Yes - had heard of SI before	Coding phase: 1. Single coded	Client marital status: Divorced/ separated/ widowed	Client age group: Under 20 (adolescent)	Time since trained: N/A - client

Codes Applied:	C0.4 - Access to or process of obtaining FP services

Interviewer: when coming to the provider, did you prepare anything?

Respondent: no

Interviewer: did you not do any household chores before coming?

Respondent: I did household chores in the morning

Interviewer: what were you doing before coming?

Respondent: sweep the surrounding, fetch water and prepare breakfast

Interviewer: where did you access Depo?

Respondent: right here at Sasa village

TIME STAMP: 10:00 MIN- 25:58MIN

Interviewer: what mode of transport did you use?

Respondent: I would meet the provider then get Depo

Interviewer: what was the mode of transport?

Respondent: I walked

Interviewer: have you access Depo at the health facility?

Respondent: no

Interviewer: how long did it take for you to access Depo?

Respondent: he was not taking much time. Sometimes when he is busy, we would wait for a short time then enter the room and be given Depo

Title: MZs_SCSI_2.docx

Descriptor Info:	District: Mzimba South	Target Population: TP3 - DMPA-SC SI clients	Respondent type: DMPA-SC SI client	Aware of SI: Yes - had heard of SI before	Coding phase: 3. Triple coded	Client marital status: Married	Client age group: Under 20 (adolescent)	Time since trained: N/A - client

Codes Applied:	C0.4 - Access to or process of obtaining FP services

Q7. Interviewer: Thanks a lot, as you have said before you started self-injection you have been using providers injection, did you find it difficult or not?

Respondent: No it was easy, but when I came for providers injection it’s when they told us that it’s out of stock, rather they told us there is new method and provider said that with this method we can be able to inject ourselves.

Interviewer: You have said it was easy can you explain how easy it was?

Respondent: I achieved what I wanted, even though some women were saying that they bleed too much but to I was not bleeding a lot and my first time using it I was not bleeding at all.

Interviewer: Has it been easy or difficult accessing family planning services because of corona virus?

Respondent: I don’t think so.

Interviewer: Alright, thanks a lot. Was it easier or difficult for you to access contraception when you were under 18?

Respondent: No it was not difficult cause I was a member of youth club, so as a youth we were told where we can find family planning methods.

Title: MZs_SCSI_2.docx

Descriptor Info:	District: Mzimba South	Target Population: TP3 - DMPA-SC SI clients	Respondent type: DMPA-SC SI client	Aware of SI: Yes - had heard of SI before	Coding phase: 3. Triple coded	Client marital status: Married	Client age group: Under 20 (adolescent)	Time since trained: N/A - client

Codes Applied:	A4 - Adol access to FP services	C0.4 - Access to or process of obtaining FP services

Interviewer: Alright, thanks a lot. Was it easier or difficult for you to access contraception when you were under 18?

Respondent: No it was not difficult cause I was a member of youth club, so as a youth we were told where we can find family planning methods.

Title: KK_IM_7.docx

Descriptor Info:	District: Nkhotakota	Target Population: TP2 - DMPA-IM/DMPA-SC PI clients	Respondent type: DMPA-IM client	Aware of SI: Yes - had heard of SI before	Coding phase: 1. Single coded	Client marital status: Married	Client age group: 30-39 years	Time since trained: N/A - client

Codes Applied:	C0.4 - Access to or process of obtaining FP services

RESPONDENT: mmmh.

INTERVIEWER: Where did you get the Norplant?

RESPONDENT: At Malomo.

INTERVIEWER: At Malomo. Here?

RESPONDENT: In Ntchisi. I was in Ntchisi, my first marriage.

INTERVIEWER: Oh ok. You have told me the problems you faced because of Norplant. What was good about this method?

RESPONDENT: The good outcome of this method was that I was not frequently visiting the hospital. I was just waiting to go for removal after the number of years they told me. But because of this problem, it was removed.

INTERVIEWER: okay.

RESPONDENT: yes

INTERVIEWER: Alright. How easy or difficult have you found it accessing family planning in the past?

RESPONDENT: From the past, there was no problem with family planning.

INTERVIEWER: Was it easy or difficult to access family planning?

RESPONDENT: It was easy.

INTERVIEWER: Where were you accessing contraception in the past?

RESPONDENT: At the hospital

INTERVIEWER: What were the factors that helped for you to access the contraceptives easily?

RESPONDENT: The factors that were helping were that at the hospital, we could tell the nurse and he/she would give us the method we wanted.

INTERVIEWER: okay.

RESPONDENT: mmmhu.

Title: KK_IM_7.docx

Descriptor Info:	District: Nkhotakota	Target Population: TP2 - DMPA-IM/DMPA-SC PI clients	Respondent type: DMPA-IM client	Aware of SI: Yes - had heard of SI before	Coding phase: 1. Single coded	Client marital status: Married	Client age group: 30-39 years	Time since trained: N/A - client

Codes Applied:	C0.4 - Access to or process of obtaining FP services

INTERVIEWER: So there was a Covid outbreak, right?

RESPONDENT: mmmh.

INTERVIEWER: Mmmh. Did you see any change in accessing contraception because of Covid outbreak?

RESPONDENT: Aaah, there was a change in the contraception method because of Covid. Because of the outbreak, they gave me Sayana, and I was not able to self-inject at home. They took back the Sayana and told me to be coming for an injection and I should be masking up.

Title: KK_IM_7.docx

Descriptor Info:	District: Nkhotakota	Target Population: TP2 - DMPA-IM/DMPA-SC PI clients	Respondent type: DMPA-IM client	Aware of SI: Yes - had heard of SI before	Coding phase: 1. Single coded	Client marital status: Married	Client age group: 30-39 years	Time since trained: N/A - client

Codes Applied:	C0.4 - Access to or process of obtaining FP services

INTERVIEWER: Mmmmmhu. Can you describe the process of obtaining your injection every 3 months? Because we know that you are supposed to come here every three months for an injection, right? So how do you obtain the injection every three months?

RESPONDENT: When we visit, we access the injection easily because we always find the injection in stock. So when we arrive, they ask us two or three questions and then they inject us and then we go back home.

INTERVIEWER: Mmmhu. What about on your appointment date, today was your appointment date?

RESPONDENT: Yes.

INTERVIEWER: For your injection?

RESPONDENT: mmmh.

INTERVIEWER: Do you have to organize anything in advance before you come here?

Time stamp: (20:11)

RESPONDENT: Organizing?

INTERVIEWER: For example, doing household chores or you close your business if you have one so that you should come here. What happens on your appointment day?

RESPONDENT: Yes. When it’s my visiting day, I wake up in the morning and go to the garden. My husband then tells me to go home after some time, take a bath and go to the hospital.

INTERVIEWER: mmmhu.

RESPONDENT: That is what we do. That is also what we have done today. We went to the garden and then he told me to go home because my time was up. I arrived at home, took a bath and came here.

INTERVIEWER: okay.

RESPONDENT: And that was when the doctor told me not to go home (laughs).

INTERVIEWER: okay.

RESPONDENT: sure

INTERVIEWER: you were with your husband at the garden?

RESPONDENT: indeed. Yes.

INTERVIEWER: okay, alright. We know that there are different places where people go for family planning right, for an injectable. For example, an HSA, at the hospital or community-based distribution agent. Where do you usually go for your injection?

RESPONDENT: I inject here [the hospital] …

INTERVIEWER: all the time?

RESPONDENT: Because it is close.

INTERVIEWER: Ok. You are close?

RESPONDENT: Yes, it’s close. It’s not far.

INTERVIEWER: Mmmmmmhu. Mmmmh, you have said that it’s close, do you walk?

RESPONDENT: Yes.

INTERVIEWER: Okay. How many minutes if you know?

RESPONDENT: Mmmmh, a few minutes. Maybe we arrive within an hour.

INTERVIEWER: mwafika? You arrive?

RESPONDENT: eeeh. Yes.

Title: KK_IM_7.docx

Descriptor Info:	District: Nkhotakota	Target Population: TP2 - DMPA-IM/DMPA-SC PI clients	Respondent type: DMPA-IM client	Aware of SI: Yes - had heard of SI before	Coding phase: 1. Single coded	Client marital status: Married	Client age group: 30-39 years	Time since trained: N/A - client

Codes Applied:	C0.4 - Access to or process of obtaining FP services	C2.1.2 - Reduced trips to facility

INTERVIEWER: Not only complaints. Anything you heard people talking about Sayana. What were they saying?

RESPONDENT: Aaaah, they were saying that Sayana is good.

INTERVIEWER: Okay. Why were they saying Sayana is good?

RESPONDENT: Because you have few visits to the hospital.

INTERVIEWER: mmmhu.

RESPONDENT: Yea.

Title: KK_IM_7.docx

Descriptor Info:	District: Nkhotakota	Target Population: TP2 - DMPA-IM/DMPA-SC PI clients	Respondent type: DMPA-IM client	Aware of SI: Yes - had heard of SI before	Coding phase: 1. Single coded	Client marital status: Married	Client age group: 30-39 years	Time since trained: N/A - client

Codes Applied:	C1.1.1 - Positive client attitudes to SI	C0.4 - Access to or process of obtaining FP services

INTERVIEWER: It’s not a test that you will pass or fail, I just want to hear your reactions.

RESPONDENT: Yes.

INTERVIEWER: mmmh.

[video from 33: 11-44:21]

INTERVIEWER: It’s finished.

RESPONDENT: It’s good.

INTERVIEWER: good right? okay

RESPONDENT: You will rest. Every three months you have to come to the hospital. I have seen that it is good because you will concentrate on farming and self-inject every three months. But currently, you have to hurry every three months to avoid being sent back (laughing).

INTERVIEWER: okay.

RESPONDENT: sure

Title: ZA_IM_5.docx

Descriptor Info:	District: Zomba	Target Population: TP2 - DMPA-IM/DMPA-SC PI clients	Respondent type: DMPA-IM client	Aware of SI: Yes - had heard of SI before	Coding phase: 2. Double coded	Client marital status: Married	Client age group: 30-39 years	Time since trained: N/A - client

Codes Applied:	C0.4 - Access to or process of obtaining FP services

Interviewer: Alright, ah where were you accessing the pills, from the facility?

Respondent: Yes, I was coming to get it from here.

Title: ZA_IM_5.docx

Descriptor Info:	District: Zomba	Target Population: TP2 - DMPA-IM/DMPA-SC PI clients	Respondent type: DMPA-IM client	Aware of SI: Yes - had heard of SI before	Coding phase: 2. Double coded	Client marital status: Married	Client age group: 30-39 years	Time since trained: N/A - client

Codes Applied:	C0.4 - Access to or process of obtaining FP services

Interviewer: Alright, when you were using the pills how difficult or easy was it to find the pills?

Respondent: It was easy.

Interviewer: Mmmm (showing that one is following the conversation)

Respondent: It was easy because when I saw that problem and came to the hospital, the providers accepted that yeah you can change the method, so which one should we give you?

Interviewer: Mmmm (showing that one is following the conversation)

Respondent: This one

Interviewer: Alright.

Respondent: So I saw that it was not hard to take this method.

Interviewer: Alright, ah so in the past you were finding the pills only here at the hospital?

Respondent: Yes, here at the hospital.

Interviewer: Alright.

Respondent: And collect

Interviewer: Alright.

Respondent: Kutchito when the providers are coming to work

Interviewer: Mmmm (showing that one is following the conversation)

Respondent: Sometimes they don’t have.

Interviewer: Mmmm (showing that one is following the conversation)

Respondent: So we start off to go to the facility

Interviewer: And collect.

Respondent: And collect so we come here to collect

Interviewer: Alright.

Title: ZA_IM_5.docx

Descriptor Info:	District: Zomba	Target Population: TP2 - DMPA-IM/DMPA-SC PI clients	Respondent type: DMPA-IM client	Aware of SI: Yes - had heard of SI before	Coding phase: 2. Double coded	Client marital status: Married	Client age group: 30-39 years	Time since trained: N/A - client

Codes Applied:	C0.4 - Access to or process of obtaining FP services

Interviewer: Ah what other things have made it easy for you to find contraceptives?

Respondent: The things that have made it easy to find contraceptives easy

Interviewer: Mmmm (showing that one is following the conversation)

Respondent: Many women

Interviewer: Mmmm (showing that one is following the conversation)

Respondent: In the past there were only a few methods of family planning.

Interviewer: Mmmm (showing that one is following the conversation)

Respondent: Maybe you could use condoms

Interviewer: Mmmm (showing that one is following the conversation)

Respondent: So the providers thought that there should be another method because the condoms…

Interviewer: Mmmm (showing that one is following the conversation)

Respondent: We were not doing family planning well.

Interviewer: Mmmm (showing that one is following the conversation)

Respondent: (pointing to the arm), implant, even with the pills we were at risk that ah, so when they brought us this method of Depo and implants.

Interviewer: Mmmm (showing that one is following the conversation)

Respondent: So that’s why we saw that it was easy to find the method, even if you want to change to another method

Interviewer: Mmmm (showing that one is following the conversation)

Respondent: Eh

Interviewer: Alright, since the COVID-19 pandemic started do you think there has been a change in the way that you access contraceptives?

Respondent: No

Title: ZA_IM_5.docx

Descriptor Info:	District: Zomba	Target Population: TP2 - DMPA-IM/DMPA-SC PI clients	Respondent type: DMPA-IM client	Aware of SI: Yes - had heard of SI before	Coding phase: 2. Double coded	Client marital status: Married	Client age group: 30-39 years	Time since trained: N/A - client

Codes Applied:	C0.4 - Access to or process of obtaining FP services

Interviewer: Ah can you tell me how you were accessing injectable contraceptives in the last three months?

Respondent: How I was accessing in the last three months?

Interviewer: Yes.

Respondent: In the three months we use to follow the dates.

Interviewer: Mmmm (showing that one is following the conversation)

Respondent: For example the Sayana option has similar dates.

Interviewer: Mmmm (showing that one is following the conversation)

Respondent: When they give us the injection this month on the 20th.

Interviewer: Mmmm (showing that one is following the conversation)

Respondent: Then the next one will end on December and they will inject us again, we will be injected on the 20th

Interviewer: On the 20th

Respondent: If its Depo then it depends that it just differs a little maybe this month they will inject us today on the 20th.

Interviewer: Mmmm (showing that one is following the conversation)

Respondent: Then next month we will not reach the, next month will pass

Interviewer: Mmmm (showing that one is following the conversation)

Respondent: Then the other one we will not reach the 20th

Interviewer: Alright, how do you remember that my date is due I need to get the injection?

Respondent: Yes, for example Sayana.

Interviewer: Mmmm (showing that one is following the conversation)

Respondent: We see the thing where you hold, yeah

Interviewer: Mmmm (showing that one is following the conversation)

Respondent: There is something like a paper

Interviewer: Mmmm (showing that one is following the conversation)

Respondent: So on that side they write the date

Interviewer: Mmmm (showing that one is following the conversation)

Respondent: If its Depo then when we come here at the hospital and the providers have assisted us yeah

Interviewer: Mmmm (showing that one is following the conversation)

Respondent: They write in our book

Interviewer: Alright.

Respondent: The date that we should what…

Interviewer: The date that you should come again

Respondent: The date that we should come

Interviewer: Alright

Respondent: So we see in the book

Interviewer: Alright, when you come here at the hospital on the date that you are supposed to get the injection, do you prepare in any way at home? For example, ah finding someone to take care of the house, or maybe at your workplace how will it be that day, do you prepare in any way?

Respondent: We don’t have to prepare up to that, because it is a school day, and the children are at school.

Interviewer: Mmmm (showing that one is following the conversation)

Respondent: When they go to school then you also get ready and come to the what?

Interviewer: To the facility

Respondent: To the facility

Interviewer: Alright.

Respondent: When we come here at the facility, we don’t spend so much time. No

Interviewer: Mmmm (showing that one is following the conversation)

Respondent: We don’t take time; they welcome us and assist us then we what…

Interviewer: Go back home

Respondent: Go back home.

Interviewer: Alright.

Respondent: So when we are getting home, the children are about to knock off from school…

Interviewer: Alright.

Respondent: Yes

Title: ZA_IM_5.docx

Descriptor Info:	District: Zomba	Target Population: TP2 - DMPA-IM/DMPA-SC PI clients	Respondent type: DMPA-IM client	Aware of SI: Yes - had heard of SI before	Coding phase: 2. Double coded	Client marital status: Married	Client age group: 30-39 years	Time since trained: N/A - client

Codes Applied:	C0.4 - Access to or process of obtaining FP services

Interviewer: Alright, where do you usually get your injection, is it at the facility or at the HSA?

Respondent: Most of the times I go to the HAS.

Interviewer: Mmmm (showing that one is following the conversation)

Respondent: Maybe when it’s the day for community clinic, since they distribute days for community clinics in the villages…

Interviewer: Mmmm (showing that one is following the conversation)

Respondent: So a place like that one, the centers like those they assist women on contraceptives…

Interviewer: They should get injected

Respondent: Children should also be assisted

Interviewer: Alright.

Respondent: That’s how it works

Interviewer: Alright, when you are coming here at the facility to get you injection, how is your mobility like?

Respondent: When we are coming here at the hospital to get the injection, our mobility…

Interviewer: Mmmm (showing that one is following the conversation)

Respondent: Sometimes we have money, and we can tell a motorcycle taxi guy to pick us, when we come here, we don’t take time as I have already said, yeah

Interviewer: Yah yah yah

Respondent: The motorcycle taxi can actually wait for us right here and we get in the facility then

Interviewer: Mmmm (showing that one is following the conversation)

Respondent: Yes

Interviewer: Alright.

Respondent: Sometimes we walk, we just say let’s walk and stretch out legs, so we walk.

Interviewer: Alright, how long do you take to travel from home to the facility and also going back home, how long does it take?

Respondent: Ah it does not take time, depending on the way we have gotten here

Interviewer: Yah

Respondent: But also along the way, you can stop to greet your friends you know women.

Interviewer: Yah yah

Respondent: Yeah, we don’t take time

Interviewer: Alright.

Respondent: Yes

Title: MZs_SCSI_5.docx

Descriptor Info:	District: Mzimba South	Target Population: TP3 - DMPA-SC SI clients	Respondent type: DMPA-SC SI client	Aware of SI: Yes - had heard of SI before	Coding phase: 1. Single coded	Client marital status: Married	Client age group: 20-29 years	Time since trained: N/A - client

Codes Applied:	C3.3 - Counselling / training approach for injectable users	C0.4 - Access to or process of obtaining FP services

Interviewer: Who first taught you on how to self-inject?

Respondent: I can’t know their name, but it was one of the providers

Interviewer: Was it a provider who taught you at first

Respondent: Yes

Interviewer: Did your friends ever taught you

Respondent: I came here at the facility that’s where I heard all about self-inject method of family planning.

Interviewer: Were you given anything to take home?

Respondent: As I said earlier on, no. we were just taught on how to self-inject. We were not given to self-inject at home. On the third visit we found that it was out of stock

Title: KK_SCSI_1.docx

Descriptor Info:	District: Nkhotakota	Target Population: TP3 - DMPA-SC SI clients	Respondent type: DMPA-SC SI client	Aware of SI: Yes - had heard of SI before	Coding phase: 3. Triple coded	Client marital status: Never married	Client age group: Under 20 (adolescent)	Time since trained: N/A - client

Codes Applied:	C0.4 - Access to or process of obtaining FP services

INTERVIEWER: mmmh , where were you receiving this method from?

RESPONDENT: I was receiving from the hospital…the condoms.

INTERVIEWER: Mmmhu. How about HSA’S…were you receiving from HSA’S…only the hospital, or maybe from an HSA’s? or maybe from somewhere else.

RESPONDENT: I was only receiving from the hospital because I have never been to an HSA.

INTERVIEWER: Okay. You…you were receiving only form the hospital?

RESPONDENT: mmmmh.

Title: KK_SCSI_1.docx

Descriptor Info:	District: Nkhotakota	Target Population: TP3 - DMPA-SC SI clients	Respondent type: DMPA-SC SI client	Aware of SI: Yes - had heard of SI before	Coding phase: 3. Triple coded	Client marital status: Never married	Client age group: Under 20 (adolescent)	Time since trained: N/A - client

Codes Applied:	C0.4 - Access to or process of obtaining FP services

INTERVIEWER: Okay. Alright. Before starting self-injection, how easy or difficult was it to access family planning services? You’ve told me that you began with condoms rights? How difficult was it to access contraceptives…how difficult or easy was it to access right, for you to access these family planning services?

RESPONDENT: It was easy because some HSAs encourage…they encourage that there are family planning services at the hospital. So the messa…whe I heard the message, I said to myself that I should go to the hospital and receive these methods so that I should not…I should not get pregnant like I wasn’t expecting back then.

INTERVIEWER: okay.

RESPONDENT: Yes.

INTERVIEWER: Where did you hear this message?

RESPONDENT: I heard for…from an HSA because they inform that there are family planning services at the hospital. You should be going to the hospital to get the contraceptives.

INTERVIEWER: Mmmh, alright. Was it difficult or easy for you to access condoms?

RESPONDENT: It was easy because I was going to the hospital.

INTERVIEWER: Okay, alright. With the outbreak of Covid right. We all know that…

RESPONDENT: Yes, true.

INTERVIEWER: Eee yes. With the outbreak of Covid, was it easy or difficult to access family planning services?

RESPONDENT: It was difficult because in hospitals, things were not the way they used to be. So it was difficult because when you go to the hospital, people were discriminating each other, with the way things were, it was difficult. Sometimes they would tell us they stopped…for example Norplant, they would say they stopped providing this method because of the outbreak. So it was difficult for people to be assisted because of the Corona outbreak.

INTERVIEWER: okay.

RESPONDENT: mmmh.

INTERVIEWER: Alright. You have told me that you started using condoms when you were 18 right?

RESPONDENT: mmh.

INTERVIEWER: Was it difficult/easy for you to access these condoms?

RESPONDENT: Sometimes it was difficult for you to access the condoms because you would go to the hospital and told they are out of stock. So sometimes it was difficult. But sometimes we were assisted when we could find them in stock.

Title: ZA_IM_9.docx

Descriptor Info:	District: Zomba	Target Population: TP2 - DMPA-IM/DMPA-SC PI clients	Respondent type: DMPA-IM client	Aware of SI: Yes - had heard of SI before	Coding phase: 1. Single coded	Client marital status: Married	Client age group: 20-29 years	Time since trained: N/A - client

Codes Applied:	C0.4 - Access to or process of obtaining FP services

Interviewer: Alright alright. Thank you so much. (background noise followed by a crowing rooster) We are now proceeding to another section; I would like us to talk about what you experience when using contraception method. Besides the injection contraception method, have you used another different contraception method in the past?

Respondent: No (Groans) It’s the injection only.

Interviewer: Alright alright. At what age did you start contraception?

Respondent: After I gave birth to my first child, I started contraception.

Interviewer: How old were you when you gave birth to the child?

Respondent: 18 years old.

Interviewer: 18 years?

Respondent: Mmh.

Interviewer: So how old were you the time you started using contraception methods?

Respondent: 18 years old.

Interviewer: You started using when you were 18 years old? Alright alright. Has it been hard or not to find contraception methods in the past?

Respondent: No.

Interviewer: Alright. Where do you find the contraception methods?

Respondent: We find them here at the hospital.

Interviewer: Mmh. You find them easily or it is hard?

Respondent: Easily.

Interviewer: Alright. What makes you find the methods easily?

Respondent: Because the hospital is close by.

Interviewer: Mmh.

Respondent: Mmh.

Interviewer: Alright alright, thank you so much. Have there been any changes in the availability of contraception methods since the Corona virus outbreak?

Respondent: No. No problem has occurred

Interviewer: When you come to the hospital, are you assisted?

Respondent: We are assisted with contraception methods.

Title: ZA_IM_9.docx

Descriptor Info:	District: Zomba	Target Population: TP2 - DMPA-IM/DMPA-SC PI clients	Respondent type: DMPA-IM client	Aware of SI: Yes - had heard of SI before	Coding phase: 1. Single coded	Client marital status: Married	Client age group: 20-29 years	Time since trained: N/A - client

Codes Applied:	C0.4 - Access to or process of obtaining FP services

Interviewer: mmh. Alright. Could you explain to me how you find the injection contraception method every three months? Do you have to prepare everything before you come and get your injection here at the hospital? Like taking care of your kids, mmh.

Respondent: Taking care of children.

Interviewer: Mmh.

Respondent: Mmh.

Interviewer: What else? What do you also do in the morning before you come here at the hospital to get your contraception?

Respondent: Cooking for my children before they go to school, mmh.

Interviewer: Mmh, your husband?

Respondent: I also cook for him food in the morning before he goes to work.

Interviewer: Mmh mmh. Alright where do you usually get your injection contraception?

Respondent: We usually get our injection at the hospital.

Interviewer: Which hospital?

Respondent: Zirindo hospital.

Interviewer: Alright, thank you. So how do you travel coming here to Zirindo? How do you travel? Do you walk? Ride a bicycle? Ride a car?

Respondent: Hmm (Smiles) we walk because my home is close, close to the hospital, so we walk on foot.

Interviewer: Mmh. It is close to here?

Respondent: Yes.

Interviewer Alright alright, how long does it take you to reach here at the hospital?

Respondent: We walk quickly to get here at the hospital.

Interviewer: For instance; how many minutes does it you?

Respondent: 2 minutes, ummh 2 hours.

Interviewer: 2 hours just to get here?

Respondent: Mmh.

Interviewer: Let’s say you leave home at 10 o’clock, what time will you get here?

Respondent: If we depart at 10 it means will get here at maybe 11.

Interviewer: Yes.

Respondent: Mmh

Interviewer: Alright alright. We should just say one hour?

Respondent: Mmh.

Interviewer: But you said it is close by, where exactly?

Respondent: (Smile) yes it really is close by.

Title: ZA_SCSI_4.docx

Descriptor Info:	District: Zomba	Target Population: TP3 - DMPA-SC SI clients	Respondent type: DMPA-SC SI client	Aware of SI: Yes - had heard of SI before	Coding phase: 2. Double coded	Client marital status: Married	Client age group: 30-39 years	Time since trained: N/A - client

Codes Applied:	C0.4 - Access to or process of obtaining FP services

Interviewer: Now I would like to talk about your experiences with contraceptive methods. Other than injectables, have you used other family planning methods in the past?

Respondent: No, I have just used this provider administered injectable.

Interviewer: mmm (showing that one is following the conversation)

Respondent: I have also used the self-injection injectable.

Interviewer: Alright.

Respondent: mmmm

Interviewer: for the provider administered injectable, were you accessing this from the facility or from other ways?

Respondent: I was accessing it from the chief in our community because they set up like, they would come once every month

Interviewer: mmm (showing that one is following the conversation)

Respondent: but when we miss the dates of going to the chief

Interviewer: mmmm (showing that one is following the conversation)

Respondent: The date that they have given us is not the one we go to the chief, then we would start off and come to the facility to get our injection.

Title: ZA_SCSI_4.docx

Descriptor Info:	District: Zomba	Target Population: TP3 - DMPA-SC SI clients	Respondent type: DMPA-SC SI client	Aware of SI: Yes - had heard of SI before	Coding phase: 2. Double coded	Client marital status: Married	Client age group: 30-39 years	Time since trained: N/A - client

Codes Applied:	C0.4 - Access to or process of obtaining FP services	C0.3 - Client previous use/experience of any FP method

Interviewer You have shared with me that you were using provider administered injectables

Respondent: mmm (showing that one is following the conversation)

Interviewer: Why did you stop using this injectable?

Respondent: I stopped using this injectable because in this period of covid

Interviewer: mmmm (showing that one is following the conversation)

Respondent: So they told us that we should be social distancing, because here at the hospital we are a lot of women.

Interviewer: eh

Respondent: So we are always crowded so they told us that starting from that day everyone should be trained.

Interviewer: mmm (showing that one is following the conversation)

Respondent: in self injection

Interviewer: mmmm (showing that one is following the conversation)

Respondent: Everyone should start using the injectable. So the day that we were trained everyone too self-injection doses for one year and went home with it

Title: ZA_SCSI_4.docx

Descriptor Info:	District: Zomba	Target Population: TP3 - DMPA-SC SI clients	Respondent type: DMPA-SC SI client	Aware of SI: Yes - had heard of SI before	Coding phase: 2. Double coded	Client marital status: Married	Client age group: 30-39 years	Time since trained: N/A - client

Codes Applied:	C0.4 - Access to or process of obtaining FP services

Interviewer: Alright, in the past before you started using the self-injecting contraceptive , how difficult or easy was it to access contraceptives?

Respondent: no it wasn’t difficult because we were trying that no matter what even if the facility is far, but we should walk so that we can protect ourselves.

Interviewer: mmmm (showing that one is following the conversation)

Respondent: And also that our children should grow well

Interviewer: Alright, ah when the covid-19 pandemic started have you seen any changes on accessing contraceptives?

Respondent: No I have not seen any changes because I have seen that it’s the same.

Interviewer: mmmm (showing that one is following the conversation)

Respondent: Even though they encouraged us to get injectables.

Interviewer: mmm (showing that one is following the conversation)

Respondent: But some fail to self-inject, they come back to the facility to have the injection administered to them. Those of us who are able to self-inject get the injectables and go home with it.

Interviewer: Alright.

Respondent: Sure.

Title: MZs_IM_1.docx

Descriptor Info:	District: Mzimba South	Target Population: TP2 - DMPA-IM/DMPA-SC PI clients	Respondent type: DMPA-IM client	Aware of SI: Yes - had heard of SI before	Coding phase: 1. Single coded	Client marital status: Married	Client age group: 30-39 years	Time since trained: N/A - client

Codes Applied:	C0.4 - Access to or process of obtaining FP services	C2.1.1 - Community availability of injectables/SI	C2.2 - Barriers, general/other

Q6. Interviewer: How easy or difficult have you found it accessing family planning services in the past?

Respondent: It is very easy because every Tuesday our HSA visit the community.

Interviewer: You have said that you receive family planning method from HSA?

Respondent: Yes

Interviewer: Since you started, have you been receiving the method from an HSA or from the facility?

Respondent: Most of the times I have been receiving it from the HSA, but sometimes it happens that the HSA have failed to show up as they planned then we do go to Jenda health for the method. But most of the times they do show up every Tuesday.

Interviewer: You have said that, if an HSA failed to show up you do visit the facility for the method, So have you done that before?

Respondent: Yes, and in have done it in different occasions. If an HSA has not shown up maybe he is busy we do go to the facility for the method, also may be as a client I did not manage to visit the clinic then I need to find time to visit the facility for the method.

Interviewer: Has there been easy or difficult in accessing family planning services changed since the outbreak of coronavirus?

Respondent: No, there is no change.

Title: MZs_IM_1.docx

Descriptor Info:	District: Mzimba South	Target Population: TP2 - DMPA-IM/DMPA-SC PI clients	Respondent type: DMPA-IM client	Aware of SI: Yes - had heard of SI before	Coding phase: 1. Single coded	Client marital status: Married	Client age group: 30-39 years	Time since trained: N/A - client

Codes Applied:	C0.4 - Access to or process of obtaining FP services	C2.1.1 - Community availability of injectables/SI

Interviewer: Can you describe the process for obtaining your 3 months injection, to start how do you remember to re-inject?

Respondent: For me to remember the days I am supposed to re-inject, is that every time we receive the injection the providers do document the dates in our health passport book and also I do count the months for example if I get the injection on January that means I will stay February, March and then I will re-inject on April and check the exactly date I am supposed to go for the injection.

Interviewer: Where do you check the dates?

Respondent: I do check in my health passport book.

Interviewer: Do you have to organize anything in advance before you can go for your injection?

Respondent: There is nothing to prepare for our body are very strong as a village people.

Interviewer: What I mean is that, may be if you want to prepare the children before going or if you are doing business maybe you have to close the business first before going?

Respondent: If the date for injection is due for family planning that means that means I have to stop whatever makes me busy and go to the clinic or facility for injection

Interviewer: Where do you usually get family planning method?

Respondent: Most of the times I do receive family planning from the HSA who is in our area.

Interviewer: How do you get there and how long does it take?

Respondent: Aaaah…. (thinking), it is a walkable distance and it takes us 5 kilometers for us to reach at the clinic, we don`t use a car.

Interviewer: How long does it take for you to reach at the clinic?

Respondent: 30 minutes.

Interviewer: What happens at the community with an HSA?

Respondent: First of all when we reach at the clinic an HSA starts with counseling and then provide the injection and at the end, they do document in our health passport book.

Interviewer: Then what happens next?

Respondent: Then they ask us to say what we discuss last we met for us to remind each other.

Interviewer: How much time do the provider spend with you?

Respondent: I think 1 hour, for them to finish all the work.

Title: KK_IM_1.docx

Descriptor Info:	District: Nkhotakota	Target Population: TP2 - DMPA-IM/DMPA-SC PI clients	Respondent type: DMPA-IM client	Aware of SI: No - never heard of SI before	Coding phase: 3. Triple coded	Client marital status: Married	Client age group: Under 20 (adolescent)	Time since trained: N/A - client

Codes Applied:	C0.4 - Access to or process of obtaining FP services

INTERVIEWER: Alright, another question is, how easy or difficult have you found it accessing family planning services in the past?

RESPONDENT: Come again

INTERVIEWER: Has it been difficult for you to access family planning methods, or it has not been difficult?

RESPONDENT: It has not been difficult

INTERVIEWER: It has not been difficult not so

RESPONDENT: Yes

INTERVIEWER: If it has not been difficult that’s good. Where have you accessed contraceptives in the past

RESPONDENT: At the health center

INTERVIEWER: You get them here at the hospital?

RESPONDENT: Yes

Title: KK_IM_1.docx

Descriptor Info:	District: Nkhotakota	Target Population: TP2 - DMPA-IM/DMPA-SC PI clients	Respondent type: DMPA-IM client	Aware of SI: No - never heard of SI before	Coding phase: 3. Triple coded	Client marital status: Married	Client age group: Under 20 (adolescent)	Time since trained: N/A - client

Codes Applied:	C0.4 - Access to or process of obtaining FP services

INTERVIEWER: Has the ease/difficult of accessing FP services changed since the outbreak of corona virus?

RESPONDENT: Come again.

INTERVIEWER: the question wants to find out since corona virus started and before it started, has there been any change in accessing FP methods? Like are you now having difficulties in accessing FP because of the outbreak of corona virus?

RESPONDENT: No

INTERVIEWER: There has never been a change not so?

RESPONDENT: Yes

INTERVIEWER: Access to FP before corona virus out break and now is it the same?

RESPONDENT: Yes

Title: KK_IM_1.docx

Descriptor Info:	District: Nkhotakota	Target Population: TP2 - DMPA-IM/DMPA-SC PI clients	Respondent type: DMPA-IM client	Aware of SI: No - never heard of SI before	Coding phase: 3. Triple coded	Client marital status: Married	Client age group: Under 20 (adolescent)	Time since trained: N/A - client

Codes Applied:	C0.4 - Access to or process of obtaining FP services

INTERVIEWER: Can you describe the process for obtaining your 3-monthly injection? To start with, how do you remember to inject yourself

RESPONDENT: At the hospital

INTERVIEWER: Is it at the hospital or through the HSA or door to door advisors or at the youth club?

RESPONDENT: No at the hospital

INTERVIEWER: You normally come here at the hospital for your injection?

RESPONDENT: Yes

INTERVIEWER: Do you have to organize anything in advance before you can go for your injection?

RESPOINDENT: Yes

INTERVIEWER: How do you organize yourself

RESPINDENT: Doing all house chores

INTERVIEWER: Doing all house chores?

RESPONDENT: Yes

INTERVIEWER: Alright, where do u usually go for your injection

RESPONDENT: At the health facility

INTERVIEWER: At the health facility?

RESPONDENT: Yes

INTERVIEWER: Now how do you go there and how long does it take?

RESPONDENT: We use a motor bike

INTERVIEWER: For you to come here?

RESPONDENT: Yes

INTERVIEWER: So how long does I take you to come here from home?

RESPONDENT: 30 minutes

Title: MZs_FAC_1.docx

Descriptor Info:	District: Mzimba South	Target Population: TP1 - Providers/HSAs	Respondent type: Facility-based provider	Aware of SI: Yes - had heard of SI before	Coding phase: 3. Triple coded	Client marital status: N/A - provider	Client age group: N/A - provider	Time since trained: A year or more ago

Codes Applied:	C0.4 - Access to or process of obtaining FP services

Interviewer: Okay, what about any challenges because of covid 19?

Respondent: The challenge may be the masking itself, we could receive a clients who just come for family planning and they didn’t wear mask and we say go and get the mask so that ewe assist you and I think this is some of the painful decision we could make and we are not sure if she goes and come back or not.

Interviewer: Mmmmmh.

Respondent: And I think that is some of the challenge, the masking itself is a challenge to some client.

Title: ZA_SCPI_8.docx

Descriptor Info:	District: Zomba	Target Population: TP2 - DMPA-IM/DMPA-SC PI clients	Respondent type: DMPA-SC PI client	Aware of SI: Yes - had heard of SI before	Coding phase: 1. Single coded	Client marital status:	Client age group:	Time since trained: N/A - client

Codes Applied:	C0.4 - Access to or process of obtaining FP services

Interviewer: did you access Norplant from the facility?

Respondent: yes

Title: ZA_SCPI_8.docx

Descriptor Info:	District: Zomba	Target Population: TP2 - DMPA-IM/DMPA-SC PI clients	Respondent type: DMPA-SC PI client	Aware of SI: Yes - had heard of SI before	Coding phase: 1. Single coded	Client marital status:	Client age group:	Time since trained: N/A - client

Codes Applied:	C0.4 - Access to or process of obtaining FP services

Interviewer: alright, can you tell me how you access the injectables every three months?

Respondent: yes, when the facility seems far, we go to the HSAs that are in the communities.

Interviewer: yah

Respondent: they encourage us about injectables, and they also inject us, when they don’t have the injectables then we come to the facility.

Interviewer: alright

Respondent: to get the injection

Interviewer: alright, how do you remember to get the injection?

Respondent: they give us a date, like the way I have come today, and they have injected me they have given me the next date that I will come on.

Title: ZA_SCPI_8.docx

Descriptor Info:	District: Zomba	Target Population: TP2 - DMPA-IM/DMPA-SC PI clients	Respondent type: DMPA-SC PI client	Aware of SI: Yes - had heard of SI before	Coding phase: 1. Single coded	Client marital status:	Client age group:	Time since trained: N/A - client

Codes Applied:	C0.4 - Access to or process of obtaining FP services

Interviewer: Alright, when you are coming to the facility to get the injection, do you have to prepare in any way, for example who will be at home that day?

Respondent: yes, I have relatives

Interviewer: alright, where do you usually get your injection from?

Respondent: mostly at the facility

Interviewer: alright, how is your transportation like when coming to the facility to get injected?

Respondent: we travel a long distance from [DZINA] to get here at the facility.

Interviewer: mmmm (showing that one is following the conversation) How long do you take to get here at the facility?

Respondent: we take some time to get here.

Interviewer: mmmm (showing that one is following the conversation)

Respondent: there is some long distance

Title: ZA_IM_7.docx

Descriptor Info:	District: Zomba	Target Population: TP2 - DMPA-IM/DMPA-SC PI clients	Respondent type: DMPA-IM client	Aware of SI: No - never heard of SI before	Coding phase: 1. Single coded	Client marital status: Never married	Client age group: 20-29 years	Time since trained: N/A - client

Codes Applied:	C0.4 - Access to or process of obtaining FP services

Interviewer: Thank you. Has it been hard or not hard for you to access the contraceptives?

Respondent: This will be hard for me to answer.

Interviewer: Okay let me ask in a different way. Where do you find the injection method?

Respondent: Here at the hospital.

Interviewer: Alright, how do you find this injection method here at the hospital? Is it hard to get access or not?

Respondent: Most of the times there are challenges for me access it because there is always a lot of people.

Interviewer: You have explained that you face challenges accessing this injection method? What are some of the challenges that you meet when you come here to the hospital?

Respondent: Like I explained the challenge is that when I come here there is always a lot of people as I speak people are still inside since morning, so this leads to the place being overcrowded and it happens that up to 2 o’clock we still here.

Interviewer: mmmmmh

Respondent: sure

Interviewer: Mmmmmh alright, thank you. Has there been any change to the way you access ways of contraception since COVID-19 started?

Respondent: No

Title: ZA_IM_7.docx

Descriptor Info:	District: Zomba	Target Population: TP2 - DMPA-IM/DMPA-SC PI clients	Respondent type: DMPA-IM client	Aware of SI: No - never heard of SI before	Coding phase: 1. Single coded	Client marital status: Never married	Client age group: 20-29 years	Time since trained: N/A - client

Codes Applied:	C0.4 - Access to or process of obtaining FP services

Interviewer: Alright thank you. Can you explain to me how you access the injection method after every 3 months? Before you come to the hospital to be injected what chores are you supposed to do?

Respondent: The same chores we do at home?

Interviewer: yes

Respondent: Like I said before I wash plates, fetch water, cleaning the house etc.

Interviewer: How about the child?

Respondent: I wash clothes for him, bath him and feed him but most of the times he is with his grandmother.

Interviewer: Alright thank you, where do you get the contraception frequently?

Respondent: Here at Matawale hospital.

Interviewer: Mmmmh, how do you travel from your village to here?

Respondent: I walk on foot it’s not far from the village.

Interviewer: How many minutes do you take?

Respondent: Mmmmh it’s not really far it’s just after chinamwali.

Interviewer: Oooh alright thank you. You walk on foot?

Respondent: Yes

Interviewer: Alright

Respondent: When I have transport money, I use a motor bicycle if not I walk on foot.

Interviewer: You are able to board a min-bus or a motorcycle?

Respondent: Bicycle or motorcycle.

Interviewer: Alright, when you use a bicycle how many minutes does it take for you to arrive?

Respondent: Just a few minutes.

Interviewer: like how many just an estimate?

Respondent: Aaaa I can’t really tell but it’s just a few minutes

Interviewer: Alright thank you, what happens when you come here to get the contraceptives?

Respondent: ummm what do you mean?

Interviewer: When you come here to the hospital to get your injection what happens?

Respondent: When we come, we first put our books on the queue then they call one by one to go inside.

Interviewer: How long does it take for the doctors or nurses to help you?

Respondent: Sometimes it really takes long because it happens that we women are outside waiting while the doctors and nurses are inside just staying.

Interviewer: Mmmmh

Respondent: Sure

Interviewer: Alright thank you, but in most cases when you come how long does it take for you to get injected?

Respondent: For instance, the other month that I came it didn’t take long but this month it has taken time.

Interviewer: How many minutes did you stay the other month?

Respondent: Ummmh I came here around 8 in the morning and by 10:00 I was done.

Interviewer: Mmmmh.

Respondent: So today I came around 8 but up to now we not yet done

Interviewer: Mmmmh now it’s not yet 10:00.

Respondent: mmmmmh

Title: MZs_SCSI_4.docx

Descriptor Info:	District: Mzimba South	Target Population: TP3 - DMPA-SC SI clients	Respondent type: DMPA-SC SI client	Aware of SI: Yes - had heard of SI before	Coding phase: 2. Double coded	Client marital status: Married	Client age group: 20-29 years	Time since trained: N/A - client

Codes Applied:	C0.4 - Access to or process of obtaining FP services	C2.1.1 - Community availability of injectables/SI

Interviewer: This self-inject method did you self-inject or you were injected by HSAs in your catchment area?

Respondent: HSA in our catchment area

Interviewer: Have you ever received from else-where apart from the health facility or from HSA?

Respondent: Mmmmh mmmmh (denying)

Title: MZs_SCSI_4.docx

Descriptor Info:	District: Mzimba South	Target Population: TP3 - DMPA-SC SI clients	Respondent type: DMPA-SC SI client	Aware of SI: Yes - had heard of SI before	Coding phase: 2. Double coded	Client marital status: Married	Client age group: 20-29 years	Time since trained: N/A - client

Codes Applied:	C0.4 - Access to or process of obtaining FP services

Interviewer: Previously before you started using self-inject, how easy or difficult was it to find provider Depo at a facility

Respondent: It was not difficult because they gave us a chance to choose the family planning method we want

TIME STAMP 2: FROM 10:01MIN TO 19:22 min

Interviewer: How difficult or easy was it for you to access provider Depo whenever you come to the facility?

Respondent: It was not difficult when I come here at the facility

Interviewer: Mmhu (agreeing)

Respondent: Yes

Interviewer: Where did you access the family planning methods you used before?

Respondent: At our health facility

Interviewer: Mmmhu (agreeing) where else did you access them?

Respondent: It was only here at the health facility

Interviewer: Mmmhu (agreeing) you have previously said that it was not difficult to access provider given Depo, what facilitated its accessibility at the health facility?

Respondent: Whenever we come here, we met providers who were assisting us promptly

Interviewer: Mmmhuuu (agreeing to probe more)

Respondent: Yes

Interviewer: What else facilitated the its accessibility?

Respondent: Come again

Interviewer: Ok, with the coming of covid-19, did it affect how you accessed family planning methods?

Respondent: No

Title: MZs_SCSI_4.docx

Descriptor Info:	District: Mzimba South	Target Population: TP3 - DMPA-SC SI clients	Respondent type: DMPA-SC SI client	Aware of SI: Yes - had heard of SI before	Coding phase: 2. Double coded	Client marital status: Married	Client age group: 20-29 years	Time since trained: N/A - client

Codes Applied:	C5.1.4 - Timely injection due to not having to rely on external factors	C0.4 - Access to or process of obtaining FP services

Interviewer: Would you tell me your first experience upon starting using Sayana Press?

Respondent: At first, we had to travel a long distance for us to access the Sayana Press. Sometimes we would go on our due dates to get another dose, but we could not find the provider whom would be attending other work at DHO or conducting other clinics like under-five. With these we would miss the doses.

Title: MZs_SCPI_3.docx

Descriptor Info:	District: Mzimba South	Target Population: TP2 - DMPA-IM/DMPA-SC PI clients	Respondent type: DMPA-SC PI client	Aware of SI: Yes - had heard of SI before	Coding phase: 1. Single coded	Client marital status: Married	Client age group: 20-29 years	Time since trained: N/A - client

Codes Applied:	C0.4 - Access to or process of obtaining FP services

5. Interviewer: Other than injectables, have you used other family planning in the past?

Respondent: No

Interviewer: Did you start with injectables?

Respondent: Yes.

Q6. Interviewer: How easy or difficult have you found it accessing family planning in the past?

Respondent: It was easy

Interviewer: You have said it was easy, so what factors have made it easy?

Respondent: No, it was not easy.

Interviewer: What factors made it difficult?

Respondent: I was scared to come and access family planning and because I was afraid thus why am saying it was difficult.

Interviewer: Why were you afraid?

Respondent: I was thinking that may be method will not be good on me thus why I was afraid.

Interviewer: So do you still have that fear?

Respondent: No

Interviewer: Why don’t you have the fear anymore?

Respondent: After seeing friends doing it, then decided to go to the facility and start the method and it’s a good one.

Interviewer: What are the good thing about the method?

Respondent: The good thing is that as of now am able to have child spacing .

Interviewer: Anything else?

Respondent: [Quiet….]

Interviewer: If there is?

Respondent: Nothing else.

Interviewer: Has there been easy/difficult accessing family planning services changed since the out beak of coronavirus?

Respondent: Yes,

Interviewer: What are the changes?

Respondent: The change I see is that we now wear something like the one you have put on (mask) and also they say that every time we should washing our hands every time.

Interviewer: Anything else?

Respondent: There is nothing else.

Interviewer: So you mean the only change you have seen is that you have been told that you should be putting on your mask and also you should be washing hands?

Respondent: Yes.

Title: MZs_SCPI_3.docx

Descriptor Info:	District: Mzimba South	Target Population: TP2 - DMPA-IM/DMPA-SC PI clients	Respondent type: DMPA-SC PI client	Aware of SI: Yes - had heard of SI before	Coding phase: 1. Single coded	Client marital status: Married	Client age group: 20-29 years	Time since trained: N/A - client

Codes Applied:	C0.4 - Access to or process of obtaining FP services

Q8. Interviewer: Can you describe the process for obtaining your 3 months injection?

Respondent: I visit the facility every 3 months.

Interviewer: Do you have to organize anything in advance before you can go for your injection?

Respondent: No

Interviewer: For example do you take care of the children or if you are doing business, you close your business first before coming for an injection?

Respondent: Yes, if it is business we do close so that we should do the injection first.

Interviewer: Where do you usually go for injection?

Respondent: At the hospital.

Interviewer: Please when talking raise your voice. How do you get to the facility and how long does it take?

Respondent: We usually walk, and it takes us to reach at the facility within 30 minutes.

Title: ZA_SCSI_1.docx

Descriptor Info:	District: Zomba	Target Population: TP3 - DMPA-SC SI clients	Respondent type: DMPA-SC SI client	Aware of SI: Yes - had heard of SI before	Coding phase: 1. Single coded	Client marital status: Married	Client age group: 40-49 years	Time since trained: N/A - client

Codes Applied:	C0.4 - Access to or process of obtaining FP services	C1.1.1 - Positive client attitudes to SI

Interviewer: Okay thanks a lot, so in the past before you started using self-injection method how hard was to get access to family planning method?

Respondent: Well it wasn’t that hard but still coming in of self-injection was a relief.

Interviewer: So in the past, where were you getting family planning methods?

Respondent: Only had them from the hospital

Interviewer: So you see it as not being hard?

Respondent: Well maybe the hard part was travelling long distance to have an injection but now we just visit a close medical assistant.

Title: ZA_SCSI_1.docx

Descriptor Info:	District: Zomba	Target Population: TP3 - DMPA-SC SI clients	Respondent type: DMPA-SC SI client	Aware of SI: Yes - had heard of SI before	Coding phase: 1. Single coded	Client marital status: Married	Client age group: 40-49 years	Time since trained: N/A - client

Codes Applied:	C0.4 - Access to or process of obtaining FP services

Interviewer: In your point of view, do you think the coming in of corona virus has interrupted the accessibility of family planning methods?

Respondent: It has changed yet because they still encourage us to have the injection.

Interviewer: So you still come and get the injection?

Respondent: Yes, we still come and get the injection.

Title: MZs_IM_4.docx

Descriptor Info:	District: Mzimba South	Target Population: TP2 - DMPA-IM/DMPA-SC PI clients	Respondent type: DMPA-IM client	Aware of SI: Yes - had heard of SI before	Coding phase: 2. Double coded	Client marital status: Married	Client age group: 30-39 years	Time since trained: N/A - client

Codes Applied:	C0.4 - Access to or process of obtaining FP services	C2.1.1 - Community availability of injectables/SI

Q6. Interviewer: How easy or difficult have you found it accessing family planning methods?

Respondent: It used to be difficult but not anymore

Interviewer: Why do you think it was difficult?

Respondent: It was difficult because the places where the HSA Where providing family planning methods where very far and sometimes they promised that they could come on this date but when the date comes, they couldn’t show up, so we had no choice but to walk to the facility

Interviewer: Do you want to add anything on difficulties on how to get the family planning services

Respondent: In the past we were walking long distances for us to reach the clinic where an HSA provide the family planning services but as of now at least we have them in different areas

Interviewer: Has the easy/difficult of accessing the family planning services changed since the outbreak of coronavirus?

Respondent: Things have been the same, the only different is that as of now we once visit the facility they say that before entering into providers room we need to wash hands

Interviewer: Is there any change that you think it has come because of coronavirus?

Respondent: The other change is that they advise us to wear mask all the times and whenever we want to see the doctor

Title: MZs_IM_4.docx

Descriptor Info:	District: Mzimba South	Target Population: TP2 - DMPA-IM/DMPA-SC PI clients	Respondent type: DMPA-IM client	Aware of SI: Yes - had heard of SI before	Coding phase: 2. Double coded	Client marital status: Married	Client age group: 30-39 years	Time since trained: N/A - client

Codes Applied:	C0.4 - Access to or process of obtaining FP services

Q8. Interviewer: Can you describe the process for obtaining your 3 monthly injection? How do you remember to re- inject?

Respondent: During stock out?

Interviewer: Not during stock out, but I want to know where do you find your 3 months injection?

Respondent: Most of the times I get it from the HSA`s, but if the HSA failed to show up then I just to to the facility.

Interviewer: How do you remember to re-inject?

Respondent: Once I get injected, I allows document it in the calendar for me to remember easily.

Interviewer: Do you have to organize anything in advance before you can go for your injection?

Respondent: Yes, I make sure that I have the mask ready and also my health passport book is ready

Interviewer: Anything else-like childcare or may be if you are doing business that means the business will suffer because you want to go to the facility

Respondent: Before I go for injection, I make sure I prepare food for my children and also help them to take a bath, then let them know that I will be out for some time and I will find them.

Interviewer: Where do you usually go for injection?

Respondent: Sometimes I get my injection at Chafesi within our area, but if the HSA did not show up, then I have to come here to get the method.

Interviewer: Mostly where do you get it from?

Respondent: Most of the time it’s here at the facility

Interviewer: How do you get here at the facility?

Respondent: I use public transport like minibus or taxi.

Interviewer: So how long does it take for you to reach here at the facility?

Respondent: Its 30 minutes

Title: KK_IM_2.docx

Descriptor Info:	District: Nkhotakota	Target Population: TP2 - DMPA-IM/DMPA-SC PI clients	Respondent type: DMPA-IM client	Aware of SI: Yes - had heard of SI before	Coding phase: 2. Double coded	Client marital status: Married	Client age group: 30-39 years	Time since trained: N/A - client

Codes Applied:	C0.4 - Access to or process of obtaining FP services

INTERVIEWER: Yes, so was it easy or difficult for you to access contraceptive methods?

RESPONDENT: It was not difficult because there were several contraceptive methods at the hospital.

INTERVIEWER: You were finding them at the hospital?

RESPONDENT: eya. Yes

INTERVIEWER: What do you think are the things that helped you to have an easy access to contraceptives?

RESPONDENT: Things? Methods?

INTERVIEWER: Things that helped you, you said it was easy for you to access contraceptives, right?

RESPONDENT: Yes

INTERVIEWER: What are the things that helped you to easily access contraceptive methods, the one you use to access it easily?

RESPONDENT: It’s because we are close to the hospital.

INTERVIEWER: Okay, the hospital is close?

RESPONDENT: mmmmhu.

INTERVIEWER: mmmhu. Now, we know that there is COVID 19 pandemic in the world. Now since this pandemic started, has there been any change in accessing contraceptive methods.

RESPONDENT: No, there is no change.

Title: KK_IM_2.docx

Descriptor Info:	District: Nkhotakota	Target Population: TP2 - DMPA-IM/DMPA-SC PI clients	Respondent type: DMPA-IM client	Aware of SI: Yes - had heard of SI before	Coding phase: 2. Double coded	Client marital status: Married	Client age group: 30-39 years	Time since trained: N/A - client

Codes Applied:	C0.4 - Access to or process of obtaining FP services

INTERVIEWER: Would you please tell me how you access the injection in every three months? Where do you get it?

RESPONDENT: I get it at the hospital.

INTERVIEWER: At the hospital. Let’s assume you were supposed to get the injection today, how do you prepare yourself for the injection? Because...Mm... Do you need to prepare yourself in any other way?

RESPONDENT: No when I am…when it is the day that I want to get the injection, I make sure not to have sex with a man.

INTERVIEWER: okay.

RESPONDENT: Mmmh. Because I easily get pregnant.

INTERVIEWER: okay. how do you remember that it is time for you to have another injection?

RESPONDENT: I check the calendar.

INTERVIEWER: You check the calendar

RESPONDENT: When they tell me the date, I make sure to keep that date keenly. Then I know that today is on fourteenth I have to go and have an injection.

INTERVIEWER: mmmhu. Apart from here at the hospital, is there any other place where you go for an injection?

RESPONDENT: Aah, we go to the HSAs

INTERVIEWER: The HSAs, from your area?

RESPONDENT: mmmh.

INTERVIEWER: Now, between here at the hospital and at the HSAs in your area, where do you frequently for for an injection

RESPONDENT: At the hospital.

INTERVIEWER: Here?

RESPONDENT: mmmnmh.

INTERVIEWER: okay. How do you travel from home to here for an injection? Mode of transport, how do you move?

RESPONDENT: I use a bike.

INTERVIEWER: You use a bike, mmmh is this a hired bike or yours?

RESPONDENT: Sometimes we hire. Sometimes my husband brings me by car.

INTERVIEWER: By car?

RESPONDENT: mmmmh.

INTERVIEWER: aaah, how many minutes from where you live to here?

RESPONDENT: It is just close.

INTERVIEWER: It is close?

RESPONDENT: mmmmh.

INTERVIEWER: When walking… okay let us assume on a bike, how much do they charge you?

RESPONDENT: I pay K200.00 for a bike.

INTERVIEWER: K200.00 aah then its… I just wanted to know the distance, with money right, if you pay K200.00 then its close right.

RESPONDENT: Yes, it is close.

INTERVIEWER: okay. because you just said it is close (Laughs)

RESPONDENT: (laughs).

Title: KK_IM_8.docx

Descriptor Info:	District: Nkhotakota	Target Population: TP2 - DMPA-IM/DMPA-SC PI clients	Respondent type: DMPA-IM client	Aware of SI: No - never heard of SI before	Coding phase: 1. Single coded	Client marital status: Married	Client age group: 20-29 years	Time since trained: N/A - client

Codes Applied:	C0.4 - Access to or process of obtaining FP services	C2.1.1 - Community availability of injectables/SI

INTERVIEWER: Alright. How easy or difficult was it for you to access contraceptive methods in the past? Was it easy or difficult? For you to access contraceptive methods?

RESPONDENT: aaah I can say that it is easy because of where I stay. I live near the hospital.

INTERVIEWER: oh ok. You travel easily?

RESPONDENT: yes.

INTERVIEWER: okay. Is it very close?

RESPONDENT: eeh. It’s this village.

INTERVIEWER: oh, there?

RESPONDENT: yes.

INTERVIEWER: Ok. Do you get the contraceptives at this hospital or also somewhere else??

RESPONDENT: There is another place for example HAS’s.

INTERVIEWER: HSA’s.

RESPONDENT: yes.

INTERVIEWER: where do you go?

RESPONDENT: Its close to the market. It Mrs [name] she stays close to the school.

INTERVIEWER: She is also an HSA?

RESPONDENT: yes.

INTERVIEWER: oh ok. Which means you get you contraceptives form…

RESPONDENT: two places.

INTERVIEWER: okay.

RESPONDENT: yes

INTERVIEWER: here and…

RESPONDENT: HSA.

INTERVIEWER: okay.

RESPONDENT: yes.

INTERVIEWER: Alright. You have said that that you access the contraceptives easily, right?

RESPONDENT: yes.

INTERVIEWER: What factors have helped for you to access the methods easily?

RESPONDENT: It is because I stay close to the hospital. If I was far from the hospital, it could have been difficult. But because I am close to both the hospital and the HSA.

INTERVIEWER: okay.

RESPONDENT: sure.

INTERVIEWER: Okay. Alright. Have you noticed any change in the availability of contraceptives because of Covid? Because there is covid right?

RESPONDENT: yes.

INTERVIEWER: Since the outbreak of covid, have you seen any changes in the availability of contraceptives?

RESPONDENT: no.

INTERVIEWER: it’s the same?

RESPONDENT: yes.

Title: KK_IM_8.docx

Descriptor Info:	District: Nkhotakota	Target Population: TP2 - DMPA-IM/DMPA-SC PI clients	Respondent type: DMPA-IM client	Aware of SI: No - never heard of SI before	Coding phase: 1. Single coded	Client marital status: Married	Client age group: 20-29 years	Time since trained: N/A - client

Codes Applied:	C0.4 - Access to or process of obtaining FP services

INTERVIEWER: Depo, ok. Can you describe the process for obtaining you 3-month injection? How do you obtain an injection on every three months?

RESPONDENT: I get it from the hospital.

INTERVIEWER: for the hospital?

RESPONDENT: Yes. When we come, we ask that we want an injectable contraception. Sometimes when they say that there is stock out, we go back.

INTERVIEWER: okay?

RESPONDENT: yes

INTERVIEWER: that really happens?

RESPONDENT: Yes. Because sometimes when you come, you are told that the injectable you are looking for, Depo is out of stock.

INTERVIEWER: so when that happens you go back?

RESPONDENT: Yes, we go back and use other methods at home.

INTERVIEWER: Okay. So how do you know that the injectable now is available I should go for an injection?

RESPONDENT: We come to ask. And sometimes we go to that HSA I have mentioned to ask. And she says it’s still not available and I’ll tell you when available. Because we stay close to them, when they are passing by, the tell me that the injection is now available I should go for an injection

Time stamp: (20:14).

INTERVIEWER: okay.

RESPONDENT: sure.

INTERVIEWER: How many times have you been sent back because of Depo stock out?

RESPONDENT: once

INTERVIEWER: once

RESPONDENT: yes, since I started this method.

INTERVIEWER: Alright. When it’s time for your injection, for example today, do you have to organize in advance before you can go for your injection? For example, taking care of your children or maybe your business, what normally happens?

RESPONDENT: I manage everything because I wake up in the morning, I do my household chores and I leave my business with my mother because I stay with her. And then I come to the hospital.

INTERVIEWER: How do you remember to go for your next injection for example if you are supposed to go today or any other date?

RESPONDENT: they tell us the next visit date when we go for an injection.

INTERVIEWER: they tell you right?

RESPONDENT: Yes. They also write in the health passport.

INTERVIEWER: Alright. Of course they tell you and they write in the health passport, but three months is a long time, right?

RESPONDENT: yes, it’s a long time.

INTERVIEWER: So at home, how do you remember that on such a date I have to go for testing, I mean for an injection.

RESPONDENT: Because I got an education, I count the months and mark the day I am supposed to go. I keep in memory that I have to go on this month. When you forget the date, you check in your health passport. And sometimes you check the health passport to avoid forgetting.

INTERVIEWER: Mmmhu, okay. So you have said that you get an injection from the hospital or form an HSA, right?

RESPONDENT: yes

INTERVIEWER: Where do you usually get your injection?

RESPONDENT: at the hospital

INTERVIEWER: at this hospital?

RESPONDENT: yes.

INTERVIEWER: okay. How do you get there?

RESPONDENT: aah because it is close, I just walk.

INTERVIEWER: Mmmhu. What happens when you arrive at the hospital? It’s your day to get an injection, what happen when you arrive?

RESPONDENT: The nurses are the ones who inject us and sometimes we find them doing other work. There are four days in a week for family planning. The days are Wednesday, Thursday…oh the days should be three. From Wednesday to Friday. And on these days, on Wednesday, there are also other activities, on Thursday its under-five clinic and other activities. And when we arrive and tell them that we are here for contraceptives, they tell us to wait for them because they are doing other things. And sometimes we go home late because we were waiting for them to finish what they were doing.

INTERVIEWER: okay. How long do you wait for them?

RESPONDENT: Sometimes we wait for an hour.

INTERVIEWER: Okay. So, how do they assist you when you arrive? Can you explain to me what happens up to the point of you being injected?

RESPONDENT: When we arrive, they give us a place to sit. Everyone who has come for family planning is supposed to sit there. We wait until they finish what they are doing for us to get help.

INTERVIEWER: Mmmhu. How do they help you?

RESPONDENT: One person at a time goes in the room.
