# Supplementary material for: Health care provider and client experiences of counselling on depot medroxyprogesterone acetate subcutaneous (DMPA-SC) for self-injection in Malawi
Source: PLOS Glob Public Health. 2023 Nov 30;3(11):e0002057. doi: 10.1371/journal.pgph.0002057 (PMC10688740; doi:10.1371/journal.pgph.0002057)
Supplement: S1 Data — (ZIP) [file pgph.0002057.s001.zip › S2/C2.1.1 community avail of injectables.doc]

Title: ZA_HSA_2.docx

Descriptor Info:	District: Zomba	Target Population: TP1 - Providers/HSAs	Respondent type: HSA	Aware of SI: Yes - had heard of SI before	Coding phase: 3. Triple coded	Client marital status: N/A - provider	Client age group: N/A - provider	Time since trained: Less than a year ago

Codes Applied:	C2.1.1 - Community availability of injectables/SI	C2.1.2 - Reduced trips to facility	P1.1.1 - Positive provider attitudes towards SI	P88 - Provider great quotes

Interviewer: ah do you think injectable contraceptives are more common than the other methods you mentioned?

Respondent: Okay, these methods are very easy to find

Interviewer: mmm (Showing that one is following the conversation)

Respondent: Because most of the HSAs in the communities have been trained in self-injection…

Interviewer: mmm (Showing that one is following the conversation)

Respondent: So, we serve most of the clients in the communities so that they don’t have to spend so much time coming to the facility.

Interviewer: mmm (Showing that one is following the conversation)

Respondent: Eh, we are able to serve so many clients in the communities different from these other methods like Norplant which require that there should be someone properly trained in providing those methods.

Title: MZs_IM_2.docx

Descriptor Info:	District: Mzimba South	Target Population: TP2 - DMPA-IM/DMPA-SC PI clients	Respondent type: DMPA-IM client	Aware of SI: Yes - had heard of SI before	Coding phase: 3. Triple coded	Client marital status: Married	Client age group: 30-39 years	Time since trained: N/A - client

Codes Applied:	C1.1.1 - Positive client attitudes to SI	C2.1.1 - Community availability of injectables/SI

Q16. Interviewer: After hearing this information, do you think that women who are already using injectable methods might be more, less or equally interested in the self-injection option?

Respondent: Yes, a lot of women we will be interested in the self-injection because they have the knowledge on how this works.

Interviewer: Mmmmmh (following)

Respondent: Women will just be going to the HSAs to collect the method.

Title: KK_HSA_1.docx

Descriptor Info:	District: Nkhotakota	Target Population: TP1 - Providers/HSAs	Respondent type: HSA	Aware of SI: Yes - had heard of SI before	Coding phase: 2. Double coded	Client marital status: N/A - provider	Client age group: N/A - provider	Time since trained: A year or more ago

Codes Applied:	C2.1.2 - Reduced trips to facility	C2.1.1 - Community availability of injectables/SI

Interviewer: Apart form these role models who encourage them to self inject what is…what are…what are the other factors you think have encouraged?

Respondent: Walking right?

Interviewer: mmmm

Respondent: Distance from the community for them to come here to get Sayana yet an HSA right in the community is helping…you are giving them the methods. So they see that they should be helped and not loose time going to the health centre mmhu. This is an advantage, when you give them and when they have run out, they will meet the HSA, my supply is finished.

Title: KK_SCSI_2.docx

Descriptor Info:	District: Nkhotakota	Target Population: TP3 - DMPA-SC SI clients	Respondent type: DMPA-SC SI client	Aware of SI: Yes - had heard of SI before	Coding phase: 1. Single coded	Client marital status: Divorced/ separated/ widowed	Client age group: 20-29 years	Time since trained: N/A - client

Codes Applied:	C2.1.1 - Community availability of injectables/SI

INTERVIEWER: Alright, where have you accessed contraceptives in the past? Is it at the facility, from the HSA or from community-based distribution agent?

RESPONDENT: HSA

INTERVIEWER: From HSA not so? Alright, so did you say it was not difficult for you to access family planning methods?

RESPONDENT : Yes

INTERVIEWER: We talked about accessibility of family planning methods especially the self- injection one, was it difficult to access it?

RESPONDENT: HSA were assisting on that one

INTERVIEWER: Ok HSA were assisting?

RESPONDENT: YES

Title: KK_HSA_3.docx

Descriptor Info:	District: Nkhotakota	Target Population: TP1 - Providers/HSAs	Respondent type: HSA	Aware of SI: Yes - had heard of SI before	Coding phase: 3. Triple coded	Client marital status: N/A - provider	Client age group: N/A - provider	Time since trained: A year or more ago

Codes Applied:	C2.1.1 - Community availability of injectables/SI

INTERVIEWER: When you say they easily get the services…where do they get the services?

RESPONDENT: When we say they easily get the services; when they need the service whether it’s a woman or an adolescent, when they get to us HSA’s, they are assisted because we work in the community?

Title: KK_HSA_3.docx

Descriptor Info:	District: Nkhotakota	Target Population: TP1 - Providers/HSAs	Respondent type: HSA	Aware of SI: Yes - had heard of SI before	Coding phase: 3. Triple coded	Client marital status: N/A - provider	Client age group: N/A - provider	Time since trained: A year or more ago

Codes Applied:	C2.1.1 - Community availability of injectables/SI

INTERVIEWER: So on the training, what were the good parts of the training?

RESPONDENT: this training was good because the method of self-injection, they…the women they are helped because when we provide the method to them, they help themselves without any problem because…the method is easily available. But for them to come to the hospital maybe some travel long distances and to come to the hospital the nurse to help them get that method might be busy with other patients and clients. But when HSAs are in the community, it’s a good method. They have been praising the method because they are getting help easily.

INTERVIEWER: mmmhu.

RESPONDENT: mmhu.

INTERVIEWER: what about…

RESPONDENT: and in good time.

Title: KK_HSA_3.docx

Descriptor Info:	District: Nkhotakota	Target Population: TP1 - Providers/HSAs	Respondent type: HSA	Aware of SI: Yes - had heard of SI before	Coding phase: 3. Triple coded	Client marital status: N/A - provider	Client age group: N/A - provider	Time since trained: A year or more ago

Codes Applied:	P1.2.1 - Negative provider attitudes towards SI	P1.1.1 - Positive provider attitudes towards SI	C2.1.2 - Reduced trips to facility	C2.1.1 - Community availability of injectables/SI

INTERVIEWER: In its first stages before it was implemented, when you had just heard. Were you in support that Sayana should be introduced?

RESPONDENT: it was difficult for us to accept it because we did not know its end results. But after it was introduced, we saw that it was a good thing and then we accepted it.

INTERVIEWER: mmmhu. So what made you change your mind? What made you to accept it?

RESPONDENT: What made me change my mind was that I realized that Sayana is a good method because with other methods, women were walking long distances to access contraceptives. But now a lot of women see the importance of this method because they access it right there in their community, in the field.

Title: KK_HSA_3.docx

Descriptor Info:	District: Nkhotakota	Target Population: TP1 - Providers/HSAs	Respondent type: HSA	Aware of SI: Yes - had heard of SI before	Coding phase: 3. Triple coded	Client marital status: N/A - provider	Client age group: N/A - provider	Time since trained: A year or more ago

Codes Applied:	C2.1.1 - Community availability of injectables/SI

INTERVIEWER: Where do women say they heard about Sayana?

RESPONDENT: a lot of get the information from an HSA. They get if from their HSA in the community and after these women tell their friends. And the friends come to the HSA to ask. Like in my community they come to ask me.

Title: ZA_SCSI_3.docx

Descriptor Info:	District: Zomba	Target Population: TP3 - DMPA-SC SI clients	Respondent type: DMPA-SC SI client	Aware of SI: Yes - had heard of SI before	Coding phase: 3. Triple coded	Client marital status: Married	Client age group: 40-49 years	Time since trained: N/A - client

Codes Applied:	C2.1.1 - Community availability of injectables/SI

Interviewer: Okay, where do you get these family planning methods?

Respondent: The health officials that come to my area.

Interviewer: You don’t get them from the hospital?

Respondent: No.

Title: ZA_SCSI_3.docx

Descriptor Info:	District: Zomba	Target Population: TP3 - DMPA-SC SI clients	Respondent type: DMPA-SC SI client	Aware of SI: Yes - had heard of SI before	Coding phase: 3. Triple coded	Client marital status: Married	Client age group: 40-49 years	Time since trained: N/A - client

Codes Applied:	C0.4 - Access to or process of obtaining FP services	C2.1.1 - Community availability of injectables/SI

Interviewer: Fine. Have you ever used the injection method at the hospital before starting self-injection?

Respondent: Yes.

Interviewer: Where were you receiving this method?

Respondent: In Matawale.

Interviewer: Matawale?

Respondent: Here at the hospital in Matawale.

Interviewer: So you got the methods here at the hospital?

Respondent: Yes.

Interviewer: How long did you use the injection method before you started self-injecting?

Respondent: I have used it for 6 years

Interviewer: In the past, before you started using the self-injecting method, how easy or hard was it to access the family planning method?

Respondent: To my part, it was hard because sometimes we’d come here at the hospital and stay for a long time. When we gave this complaint to the HSA’s, they told us that they would start bringing the injections to our different areas when they come to deliver antenatal services. So what we did was that each one of us would keep their date and when the HSA’s come to deliver the antenatal services, we’d go there to get our injections.

Interviewer: Okay thank you very much. Has there been any change in the availability of the family planning method with the coming of COVID 19?

Respondent: No, since we would just go right where we go when accessing antenatal services to get the method, so it has had no effect in the way we access the method. In addition, we are a fewer number of people who go there.

Title: ZA_SCSI_2.docx

Descriptor Info:	District: Zomba	Target Population: TP3 - DMPA-SC SI clients	Respondent type: DMPA-SC SI client	Aware of SI: Yes - had heard of SI before	Coding phase: 1. Single coded	Client marital status:	Client age group:	Time since trained: N/A - client

Codes Applied:	C2.1.1 - Community availability of injectables/SI	C0.3 - Client previous use/experience of any FP method

Interviewer: Ah you have said that you have used the provider administered injectables, ah did you receive this from the facility or from the HSAs?

Respondent: I received from the HSAs

Interviewer: Alright.

Respondent: In my community.

Interviewer: Alright, you have told me that you have used the provider administered injectables, for how long have you used this injectable before you started using DMPA-SC?

Respondent: the provider administered I have taken it twice, and with this one its three times.

Interviewer: Okay

Respondent: Yes.

Interviewer: so we can say approximately nine months?

Respondent: Yes, nine months

Interviewer: Alright

Respondent: This one I have just gotten it here

Title: ZA_SCSI_5.docx

Descriptor Info:	District: Zomba	Target Population: TP3 - DMPA-SC SI clients	Respondent type: DMPA-SC SI client	Aware of SI: Yes - had heard of SI before	Coding phase: 3. Triple coded	Client marital status: Married	Client age group: 30-39 years	Time since trained: N/A - client

Codes Applied:	C2.1.1 - Community availability of injectables/SI

Interviewer: Can you tell me your experiences in being trained how to use DMPA SC? Firstly I want you to tell me who trained you.

Respondent: Ah this injection, the person who trained me…

Interviewer: Mmm (showing that one is following the conversation)

Respondent: Is the HSA

Interviewer: Alright.

Respondent: That’s the one who trained me

Title: MZs_HSA_3.docx

Descriptor Info:	District: Mzimba South	Target Population: TP1 - Providers/HSAs	Respondent type: HSA	Aware of SI: Yes - had heard of SI before	Coding phase: 3. Triple coded	Client marital status: N/A - provider	Client age group: N/A - provider	Time since trained: Less than a year ago

Codes Applied:	P2.2 - Barriers, general/other	C2.1.1 - Community availability of injectables/SI

Interviewer: what were the good parts of the training?

Respondent: the good parts of that training were:

1. At… at… at least at first the job was difficult because the cadre which was taking that one could not move everywhere because they are… you said I should not mention the [CADRE]…or that one…other cadres could come in they were there to provide but later on they said we have to expand this programme we should include more people so that train all HSAs every HSA should be providing so it easier... it made it easy for the people to access the services because those were at the outreach those who are here could get here those who are at outreach there are HSAs there are HSAs there they collect the family planning methods they go with them there they give them… yah so people had access to the services.

2. the other one was we received the per diem whereby we had to sacrifice ourselves like buying some of things yah

Title: ZA_HSA_6.docx

Descriptor Info:	District: Zomba	Target Population: TP1 - Providers/HSAs	Respondent type: HSA	Aware of SI: Yes - had heard of SI before	Coding phase: 1. Single coded	Client marital status: N/A - provider	Client age group: N/A - provider	Time since trained:

Codes Applied:	P2.3 - Provider training content	C2.1.1 - Community availability of injectables/SI	P2.1.2 - Sufficient original provider training

Interviewer: Alright, Now I would like to ask you on your experiences in being trained on DMPA-SC or Sayana press. Ah would you tell me a bit about the training that you received relating to providing self-injection?

Respondent: ah we went to training, mostly we were targeting HSAs who are in hard-to-reach areas and those who do village clinics, village clinics are situated in areas that are far from the facility where most of the services from the hospital d does not reach, so many of them were trained because their catchment areas are very far from the facility. So that the family planning services can reach them without any problems. So, we were trained, we went to Mponela, and we were there for some days, I think they were three days if am not mistaken, but I think it was three days if am not mistaken, we were trained on how to train clients on the method, the benefits of the method and also problems which can be there due to the method and also the technic on how they can self-inject.

Interviewer: mmmm (showing that one is following the conversation)

Respondent: We were trained, and also when we came back here, we trained the committees of volunteers which are close to us so that they can be helping us spread the message.

Interviewer: Alright. What are your thoughts on the training you received?

Respondent: ah this training that we received I can say that we should be grateful, the training was good indeed, because of course we already knew about family planning.

Title: ZA_HSA_6.docx

Descriptor Info:	District: Zomba	Target Population: TP1 - Providers/HSAs	Respondent type: HSA	Aware of SI: Yes - had heard of SI before	Coding phase: 1. Single coded	Client marital status: N/A - provider	Client age group: N/A - provider	Time since trained:

Codes Applied:	P2.2 - Barriers, general/other	C2.1.1 - Community availability of injectables/SI

Interviewer: Okay fine, alright are there any specific challenges that you have faced due to lack of training in recording DMPA-SC?

TIME INTO THE INTERVIEW: 19Mins 51Secs

Respondent: ah because there are any of us

Interviewer: mmmm (showing that one is following the conversation)

Respondent: and all of us have catchment areas

Interviewer: mmmm (showing that one is following the conversation)

Respondent: so, if there are some HSAs that have not been trained, let’s just assume yeah

Interviewer: yah yah

Respondent: then it seems that in their catchment area the clients will be at a disadvantage because the benefits of the training or maybe of the resources that would have been received in their area…

Interviewer: yah yah

Respondent: let’s just assume that an HSA is around this hospital but is working in the village, but the person does not want to come to the hospital to access family planning and the HSA in that area has not been trained In DMPA-SC let’s just assume yeah

Interviewer: mmmm (showing that one is following the conversation)

Respondent: it means the HSAs are not supposed to take Sayana at the hospital and go distribute in the community

Interviewer: mmmm (showing that one is following the conversation)

Respondent: but the client wants to access the contraceptive and also, she cannot come here at the facility, maybe she is concerned about her family and because of other reasons. So, I see that if the HSA Is trained in DMPA-SC

Interviewer: mmmm (showing that one is following the conversation)

Respondent: it can help a lot.

Title: ZA_HSA_6.docx

Descriptor Info:	District: Zomba	Target Population: TP1 - Providers/HSAs	Respondent type: HSA	Aware of SI: Yes - had heard of SI before	Coding phase: 1. Single coded	Client marital status: N/A - provider	Client age group: N/A - provider	Time since trained:

Codes Applied:	P3.2.2 - Stockouts/stocks near expiry dates	P2.2 - Barriers, general/other	C2.1.1 - Community availability of injectables/SI

Interviewer: Alright, what factors do you think prevent women from using DMPA-SC?

Respondent: Stock outs of the commodity, it seems that it’s a major factor

Interviewer: mmmm (showing that one is following the conversation)

Respondent: ah and maybe the cancellation of clinics in the communities can be another factor

Interviewer: mmmm (showing that one is following the conversation)

Respondent: another factor is the HSAs that have been trained, if these are not available at the community clinic in a particular week

Interviewer: eh

Respondent: If only those that have gone to the community clinic are the ones that were not trained, this can affect the uptake of DMPA-SC, if all the HSAs are trained it means the clinics will still be done even if some HSAs are busy. It could be that 5 HSAs were supposed to go to the community clinic but two out of the five have been invited to a TB program, it means only three will go to the community clinic and if these three have not been trained in DMPA-SC and only the two that have been called to a different program were trained, then it could be a mess

Title: MZs_IM_1.docx

Descriptor Info:	District: Mzimba South	Target Population: TP2 - DMPA-IM/DMPA-SC PI clients	Respondent type: DMPA-IM client	Aware of SI: Yes - had heard of SI before	Coding phase: 1. Single coded	Client marital status: Married	Client age group: 30-39 years	Time since trained: N/A - client

Codes Applied:	C0.3 - Client previous use/experience of any FP method	C2.1.1 - Community availability of injectables/SI

Q5. Interviewer: Other than injectables, have you used any other family planning method in the past:

Respondent: No, I started with injectables, and I have never switched to another method because I have never experienced any challenge since I started.

Interviewer: Where do you receive it?

Respondent: Most of the time I receive it from the HSA who is in our area,

Interviewer: Tell me the good things about this method?

Respondent: The good thing about this method is that where we get the method is not far from where we stay and the HSA always visit us whenever they have promised to do so.

Interviewer: What about the bad things about the method?

Respondent: I have never experienced anything bad, that is why since I started I have never changed the method.

Title: MZs_IM_1.docx

Descriptor Info:	District: Mzimba South	Target Population: TP2 - DMPA-IM/DMPA-SC PI clients	Respondent type: DMPA-IM client	Aware of SI: Yes - had heard of SI before	Coding phase: 1. Single coded	Client marital status: Married	Client age group: 30-39 years	Time since trained: N/A - client

Codes Applied:	C0.4 - Access to or process of obtaining FP services	C2.1.1 - Community availability of injectables/SI	C2.2 - Barriers, general/other

Q6. Interviewer: How easy or difficult have you found it accessing family planning services in the past?

Respondent: It is very easy because every Tuesday our HSA visit the community.

Interviewer: You have said that you receive family planning method from HSA?

Respondent: Yes

Interviewer: Since you started, have you been receiving the method from an HSA or from the facility?

Respondent: Most of the times I have been receiving it from the HSA, but sometimes it happens that the HSA have failed to show up as they planned then we do go to Jenda health for the method. But most of the times they do show up every Tuesday.

Interviewer: You have said that, if an HSA failed to show up you do visit the facility for the method, So have you done that before?

Respondent: Yes, and in have done it in different occasions. If an HSA has not shown up maybe he is busy we do go to the facility for the method, also may be as a client I did not manage to visit the clinic then I need to find time to visit the facility for the method.

Interviewer: Has there been easy or difficult in accessing family planning services changed since the outbreak of coronavirus?

Respondent: No, there is no change.

Title: MZs_IM_1.docx

Descriptor Info:	District: Mzimba South	Target Population: TP2 - DMPA-IM/DMPA-SC PI clients	Respondent type: DMPA-IM client	Aware of SI: Yes - had heard of SI before	Coding phase: 1. Single coded	Client marital status: Married	Client age group: 30-39 years	Time since trained: N/A - client

Codes Applied:	C0.4 - Access to or process of obtaining FP services	C2.1.1 - Community availability of injectables/SI

Interviewer: Can you describe the process for obtaining your 3 months injection, to start how do you remember to re-inject?

Respondent: For me to remember the days I am supposed to re-inject, is that every time we receive the injection the providers do document the dates in our health passport book and also I do count the months for example if I get the injection on January that means I will stay February, March and then I will re-inject on April and check the exactly date I am supposed to go for the injection.

Interviewer: Where do you check the dates?

Respondent: I do check in my health passport book.

Interviewer: Do you have to organize anything in advance before you can go for your injection?

Respondent: There is nothing to prepare for our body are very strong as a village people.

Interviewer: What I mean is that, may be if you want to prepare the children before going or if you are doing business maybe you have to close the business first before going?

Respondent: If the date for injection is due for family planning that means that means I have to stop whatever makes me busy and go to the clinic or facility for injection

Interviewer: Where do you usually get family planning method?

Respondent: Most of the times I do receive family planning from the HSA who is in our area.

Interviewer: How do you get there and how long does it take?

Respondent: Aaaah…. (thinking), it is a walkable distance and it takes us 5 kilometers for us to reach at the clinic, we don`t use a car.

Interviewer: How long does it take for you to reach at the clinic?

Respondent: 30 minutes.

Interviewer: What happens at the community with an HSA?

Respondent: First of all when we reach at the clinic an HSA starts with counseling and then provide the injection and at the end, they do document in our health passport book.

Interviewer: Then what happens next?

Respondent: Then they ask us to say what we discuss last we met for us to remind each other.

Interviewer: How much time do the provider spend with you?

Respondent: I think 1 hour, for them to finish all the work.

Title: MZs_SCSI_4.docx

Descriptor Info:	District: Mzimba South	Target Population: TP3 - DMPA-SC SI clients	Respondent type: DMPA-SC SI client	Aware of SI: Yes - had heard of SI before	Coding phase: 2. Double coded	Client marital status: Married	Client age group: 20-29 years	Time since trained: N/A - client

Codes Applied:	C2.1.1 - Community availability of injectables/SI

Interviewer: This self-inject method did you self-inject or you were injected by HSAs in your catchment area?

Respondent: HSA in our catchment area

Title: MZs_SCSI_4.docx

Descriptor Info:	District: Mzimba South	Target Population: TP3 - DMPA-SC SI clients	Respondent type: DMPA-SC SI client	Aware of SI: Yes - had heard of SI before	Coding phase: 2. Double coded	Client marital status: Married	Client age group: 20-29 years	Time since trained: N/A - client

Codes Applied:	C0.4 - Access to or process of obtaining FP services	C2.1.1 - Community availability of injectables/SI

Interviewer: This self-inject method did you self-inject or you were injected by HSAs in your catchment area?

Respondent: HSA in our catchment area

Interviewer: Have you ever received from else-where apart from the health facility or from HSA?

Respondent: Mmmmh mmmmh (denying)

Title: MZs_SCSI_4.docx

Descriptor Info:	District: Mzimba South	Target Population: TP3 - DMPA-SC SI clients	Respondent type: DMPA-SC SI client	Aware of SI: Yes - had heard of SI before	Coding phase: 2. Double coded	Client marital status: Married	Client age group: 20-29 years	Time since trained: N/A - client

Codes Applied:	C2.1.1 - Community availability of injectables/SI

Interviewer: You were told? Were you given Sayana at the health facility or at your community?

Respondent: At our community

Interviewer: What motivated you to keep on using Sayana Press from the provider?

Respondent: It’s because you use it at home, so you have all the time to do household chores without interruptions

Title: ZA_HSA_1.docx

Descriptor Info:	District: Zomba	Target Population: TP1 - Providers/HSAs	Respondent type: HSA	Aware of SI: Yes - had heard of SI before	Coding phase: 1. Single coded	Client marital status: N/A - provider	Client age group: N/A - provider	Time since trained: Less than a year ago

Codes Applied:	C2.1.1 - Community availability of injectables/SI

Respondent: (Giggles) We go for outreach where we offer under 5 clinic

Interviewer: Mhhhhmmm

Respondent: When we go for outreach clinics we also take vaccines and Sayana

Interviewer: Mhhhmmm

Respondent: To inject women, to shorten their distance as the place is far. We follow them and inject them there

Title: MZs_IM_4.docx

Descriptor Info:	District: Mzimba South	Target Population: TP2 - DMPA-IM/DMPA-SC PI clients	Respondent type: DMPA-IM client	Aware of SI: Yes - had heard of SI before	Coding phase: 2. Double coded	Client marital status: Married	Client age group: 30-39 years	Time since trained: N/A - client

Codes Applied:	C0.4 - Access to or process of obtaining FP services	C2.1.1 - Community availability of injectables/SI

Q6. Interviewer: How easy or difficult have you found it accessing family planning methods?

Respondent: It used to be difficult but not anymore

Interviewer: Why do you think it was difficult?

Respondent: It was difficult because the places where the HSA Where providing family planning methods where very far and sometimes they promised that they could come on this date but when the date comes, they couldn’t show up, so we had no choice but to walk to the facility

Interviewer: Do you want to add anything on difficulties on how to get the family planning services

Respondent: In the past we were walking long distances for us to reach the clinic where an HSA provide the family planning services but as of now at least we have them in different areas

Interviewer: Has the easy/difficult of accessing the family planning services changed since the outbreak of coronavirus?

Respondent: Things have been the same, the only different is that as of now we once visit the facility they say that before entering into providers room we need to wash hands

Interviewer: Is there any change that you think it has come because of coronavirus?

Respondent: The other change is that they advise us to wear mask all the times and whenever we want to see the doctor

Title: MZs_IM_4.docx

Descriptor Info:	District: Mzimba South	Target Population: TP2 - DMPA-IM/DMPA-SC PI clients	Respondent type: DMPA-IM client	Aware of SI: Yes - had heard of SI before	Coding phase: 2. Double coded	Client marital status: Married	Client age group: 30-39 years	Time since trained: N/A - client

Codes Applied:	C2.1.1 - Community availability of injectables/SI

Q8. Interviewer: Can you describe the process for obtaining your 3 monthly injection? How do you remember to re- inject?

Respondent: During stock out?

Interviewer: Not during stock out, but I want to know where do you find your 3 months injection?

Respondent: Most of the times I get it from the HSA`s, but if the HSA failed to show up then I just to to the facility.

Title: KK_IM_2.docx

Descriptor Info:	District: Nkhotakota	Target Population: TP2 - DMPA-IM/DMPA-SC PI clients	Respondent type: DMPA-IM client	Aware of SI: Yes - had heard of SI before	Coding phase: 2. Double coded	Client marital status: Married	Client age group: 30-39 years	Time since trained: N/A - client

Codes Applied:	C2.1.1 - Community availability of injectables/SI

INTERVIEWER: mmmhu. Apart from here at the hospital, is there any other place where you go for an injection?

RESPONDENT: Aah, we go to the HSAs

INTERVIEWER: The HSAs, from your area?

RESPONDENT: mmmh.

INTERVIEWER: Now, between here at the hospital and at the HSAs in your area, where do you frequently for for an injection

RESPONDENT: At the hospital.

INTERVIEWER: Here?

RESPONDENT: mmmnmh.

Title: KK_IM_8.docx

Descriptor Info:	District: Nkhotakota	Target Population: TP2 - DMPA-IM/DMPA-SC PI clients	Respondent type: DMPA-IM client	Aware of SI: No - never heard of SI before	Coding phase: 1. Single coded	Client marital status: Married	Client age group: 20-29 years	Time since trained: N/A - client

Codes Applied:	C0.4 - Access to or process of obtaining FP services	C2.1.1 - Community availability of injectables/SI

INTERVIEWER: Alright. How easy or difficult was it for you to access contraceptive methods in the past? Was it easy or difficult? For you to access contraceptive methods?

RESPONDENT: aaah I can say that it is easy because of where I stay. I live near the hospital.

INTERVIEWER: oh ok. You travel easily?

RESPONDENT: yes.

INTERVIEWER: okay. Is it very close?

RESPONDENT: eeh. It’s this village.

INTERVIEWER: oh, there?

RESPONDENT: yes.

INTERVIEWER: Ok. Do you get the contraceptives at this hospital or also somewhere else??

RESPONDENT: There is another place for example HAS’s.

INTERVIEWER: HSA’s.

RESPONDENT: yes.

INTERVIEWER: where do you go?

RESPONDENT: Its close to the market. It Mrs [name] she stays close to the school.

INTERVIEWER: She is also an HSA?

RESPONDENT: yes.

INTERVIEWER: oh ok. Which means you get you contraceptives form…

RESPONDENT: two places.

INTERVIEWER: okay.

RESPONDENT: yes

INTERVIEWER: here and…

RESPONDENT: HSA.

INTERVIEWER: okay.

RESPONDENT: yes.

INTERVIEWER: Alright. You have said that that you access the contraceptives easily, right?

RESPONDENT: yes.

INTERVIEWER: What factors have helped for you to access the methods easily?

RESPONDENT: It is because I stay close to the hospital. If I was far from the hospital, it could have been difficult. But because I am close to both the hospital and the HSA.

INTERVIEWER: okay.

RESPONDENT: sure.

INTERVIEWER: Okay. Alright. Have you noticed any change in the availability of contraceptives because of Covid? Because there is covid right?

RESPONDENT: yes.

INTERVIEWER: Since the outbreak of covid, have you seen any changes in the availability of contraceptives?

RESPONDENT: no.

INTERVIEWER: it’s the same?

RESPONDENT: yes.
