# Supplementary material for: Health care provider and client experiences of counselling on depot medroxyprogesterone acetate subcutaneous (DMPA-SC) for self-injection in Malawi
Source: PLOS Glob Public Health. 2023 Nov 30;3(11):e0002057. doi: 10.1371/journal.pgph.0002057 (PMC10688740; doi:10.1371/journal.pgph.0002057)
Supplement: S1 Data — (ZIP) [file pgph.0002057.s001.zip › S2/C2.1.2 reduced trips to facility.doc]

Title: KK_IM_3.docx

Descriptor Info:	District: Nkhotakota	Target Population: TP2 - DMPA-IM/DMPA-SC PI clients	Respondent type: DMPA-IM client	Aware of SI: Yes - had heard of SI before	Coding phase: 1. Single coded	Client marital status: Divorced/ separated/ widowed	Client age group: 20-29 years	Time since trained: N/A - client

Codes Applied:	C1.1.1 - Positive client attitudes to SI	C2.1.2 - Reduced trips to facility

INTERVIEWER: If yes what influenced you to try the self-injection option? What were the main reason?

RESPONDENT: The major factor was that I can take the other doses and use them home, since one dose is for 3 months, that means I would take some months whilst at home.

INTERVIEWER: Yes

RESPONDENT: For instance, if you are given two doses it means you will not visit the hospital for some time since you have the injections.

INTERVIEWER: Yes, it means you will take time

RESPONDENT: You will take some time before you visit the hospital

Title: KK_IM_3.docx

Descriptor Info:	District: Nkhotakota	Target Population: TP2 - DMPA-IM/DMPA-SC PI clients	Respondent type: DMPA-IM client	Aware of SI: Yes - had heard of SI before	Coding phase: 1. Single coded	Client marital status: Divorced/ separated/ widowed	Client age group: 20-29 years	Time since trained: N/A - client

Codes Applied:	C2.1.2 - Reduced trips to facility	C1.1.1 - Positive client attitudes to SI

INTERVIEWER: Alright, are you not going to try one day?

RESPONDENT: I will try since it’s a good method

INTERVIEWER: Why are you saying it is a good method? What good thing have you learnt?

RESPONDENT: The good side is the reduction in visits to the hospital

INTERVIEWER: Visits not so?

RESPONDENT: Yes

Title: KK_SCSI_5.docx

Descriptor Info:	District: Nkhotakota	Target Population: TP3 - DMPA-SC SI clients	Respondent type: DMPA-SC SI client	Aware of SI: Yes - had heard of SI before	Coding phase: 2. Double coded	Client marital status: Married	Client age group: 20-29 years	Time since trained: N/A - client

Codes Applied:	C2.1.2 - Reduced trips to facility	C1.1.1 - Positive client attitudes to SI

INTERVIEWER: What interested you the most about self-injection

RESPONDENT: What interested me the most was that when you take Sayana you have a year still at home , whilst when you take this one that is injected by the provider you have to go to a facility every three moth that doesn’t feel good. Hence, I felt Sayana is a good contraceptive

Title: KK_SCSI_5.docx

Descriptor Info:	District: Nkhotakota	Target Population: TP3 - DMPA-SC SI clients	Respondent type: DMPA-SC SI client	Aware of SI: Yes - had heard of SI before	Coding phase: 2. Double coded	Client marital status: Married	Client age group: 20-29 years	Time since trained: N/A - client

Codes Applied:	C2.1.2 - Reduced trips to facility	C1.1.1 - Positive client attitudes to SI

INTERVIEWER: What are the things that encouraged you so you can be convinced to try self-injection method, because some would respond in a group, but they cannot be strong enough to take this method. But you were strong enough to tell yourself to try this method, what is it that motivated you to try it after being trained on the method. Maybe they mentioned something good about this method, what was it.

RESPONDENT: Yes, there was something good about the method that heard from my friend

INTERVIEWER: What was it?

RESPONDENT: The good side of self-injection is that you stay home for a year. Furthermore…

INTERVIEWER: Yes.

Title: KK_SCSI_5.docx

Descriptor Info:	District: Nkhotakota	Target Population: TP3 - DMPA-SC SI clients	Respondent type: DMPA-SC SI client	Aware of SI: Yes - had heard of SI before	Coding phase: 2. Double coded	Client marital status: Married	Client age group: 20-29 years	Time since trained: N/A - client

Codes Applied:	C2.1.2 - Reduced trips to facility	C1.1.1 - Positive client attitudes to SI

INTERVIEWER: Alright, what elements do like about using DMPA-SC for self-injection? What are the main factors you like? you have mentioned some of them already

RESPONDENT: yes, I mentioned

INTERVIEWER: What did you say let’s just remind each other

RESPONDENT: I said this method is good since you take a year without visiting the hospital

INTERVIEWER: Alright

RESPONDENT: Yes

Title: ZA_HSA_2.docx

Descriptor Info:	District: Zomba	Target Population: TP1 - Providers/HSAs	Respondent type: HSA	Aware of SI: Yes - had heard of SI before	Coding phase: 3. Triple coded	Client marital status: N/A - provider	Client age group: N/A - provider	Time since trained: Less than a year ago

Codes Applied:	C2.1.1 - Community availability of injectables/SI	C2.1.2 - Reduced trips to facility	P1.1.1 - Positive provider attitudes towards SI	P88 - Provider great quotes

Interviewer: ah do you think injectable contraceptives are more common than the other methods you mentioned?

Respondent: Okay, these methods are very easy to find

Interviewer: mmm (Showing that one is following the conversation)

Respondent: Because most of the HSAs in the communities have been trained in self-injection…

Interviewer: mmm (Showing that one is following the conversation)

Respondent: So, we serve most of the clients in the communities so that they don’t have to spend so much time coming to the facility.

Interviewer: mmm (Showing that one is following the conversation)

Respondent: Eh, we are able to serve so many clients in the communities different from these other methods like Norplant which require that there should be someone properly trained in providing those methods.

Title: ZA_HSA_2.docx

Descriptor Info:	District: Zomba	Target Population: TP1 - Providers/HSAs	Respondent type: HSA	Aware of SI: Yes - had heard of SI before	Coding phase: 3. Triple coded	Client marital status: N/A - provider	Client age group: N/A - provider	Time since trained: Less than a year ago

Codes Applied:	C2.1.2 - Reduced trips to facility	C5.1.4 - Timely injection due to not having to rely on external factors	P88 - Provider great quotes

Respondent: Yes, for the women instead of them finding time to come to the hospital, they can just self-inject right at home, and they can continue doing other chores, so DMPA-SC (Sayana press) is helping our clients not to travel longer distances or for more days to access family planning methods.

Interviewer: Alright.

Respondent: But when they go once and collect Sayana for use in a longer period, they will inject themselves first here at the facility and then take three more doses that they can use at home, this means that they can be able to perform their chores without disruptions.

Title: ZA_HSA_2.docx

Descriptor Info:	District: Zomba	Target Population: TP1 - Providers/HSAs	Respondent type: HSA	Aware of SI: Yes - had heard of SI before	Coding phase: 3. Triple coded	Client marital status: N/A - provider	Client age group: N/A - provider	Time since trained: Less than a year ago

Codes Applied:	C2.1.2 - Reduced trips to facility	C1.1.1 - Positive client attitudes to SI

Interviewer: Alright, what do you think women find most appealing about DMPA-SC? What exactly attracts them to start using the self-injectable alternative after you finish explaining to them?

Respondent: Yes, most of the times when the women come to the clinic, they see that the facility is busy most of the times….

Interviewer: Yes

Respondent: The same providers who are supposed to administer the injectable contraceptive to the clients at the facility are busy with other things like antenatal clinic, and do many other things, yeah…

Interviewer: mmm (showing that one is following the conversation)

Respondent: So, when the time to assist these clients comes and they are given three extra Sayana commodity in addition to the one that they have been injected with, the clients see that it’s a good thing, because in a year they will just have to travel once to the facility…

Interviewer: mmm (showing that one is following the conversation)

Respondent: Because, in every three months they have to go to the facility and receive the family planning service, so the women see that this method of Sayana is helpful

Interviewer: mmm (showing that one is following the conversation)

Respondent: Because of this, the method could be similar to some of these longer-term family planning methods.

Title: ZA_HSA_2.docx

Descriptor Info:	District: Zomba	Target Population: TP1 - Providers/HSAs	Respondent type: HSA	Aware of SI: Yes - had heard of SI before	Coding phase: 3. Triple coded	Client marital status: N/A - provider	Client age group: N/A - provider	Time since trained: Less than a year ago

Codes Applied:	C1.1.1 - Positive client attitudes to SI	C2.1.2 - Reduced trips to facility	C8.2 - Covert/ open use of injectables/SI	C8.1 - Male involvement, general

Interviewer: What are the main factors that would support a client to take up the self-injection option with DMPA-SC?

Respondent: The first thing…

Interviewer: mmmhhh (showing that one is following the conversation)

Respondent: Aah it’s because the method seems like a longer one…

Interviewer: mmm (showing agreement)

Respondent: If the client has managed to self-inject it means they are reducing the number of times they have to travel to the facility compared with DMPA-IM, yeah? Even the same DMPA-SC-PI but that they will have more time at home. When they understand how to self-inject, they will do it themselves at home which means they have more time for taking care of their families than coming to the hospital to get the injection within a short period of time. Yeah so, it’s a very important method. There are some men who don’t allow their wives to take family planning. If the client has taken the doses for future usage, it means they will keep at a secret place, when the day to self-inject comes they will just take the injection and administer to themselves without their men finding out that my wife went to get family planning, yeah.

Title: ZA_HSA_2.docx

Descriptor Info:	District: Zomba	Target Population: TP1 - Providers/HSAs	Respondent type: HSA	Aware of SI: Yes - had heard of SI before	Coding phase: 3. Triple coded	Client marital status: N/A - provider	Client age group: N/A - provider	Time since trained: Less than a year ago

Codes Applied:	C1.1.1 - Positive client attitudes to SI	C5.1.4 - Timely injection due to not having to rely on external factors	C5.2.1 - Discontinuation of SI (including reasons)	P3.2.2 - Stockouts/stocks near expiry dates	C2.1.2 - Reduced trips to facility	C7 - Resupply

Respondent: According to the preferences of the clients in the communities, some prefer Sayana…

Interviewer: mmmhhh (showing that one is following the conversation)

Respondent: … and some prefer Depo

Interviewer: mmmmmhhh (showing that one is following the conversation)

Respondent: Yes, so when all the commodities are available, especially Sayana is available without hustles…

Interviewer: mmmhhh (showing that one is following the conversation)

Respondent: Yeah, it means it will give assurance to the clients because they will be coming to access the commodities…

Interviewer: mmmm (showing that one is following the conversation)

Respondent: Because when the commodity has run out, and we tell them that we will give them Depo, that Sayana is not available…

Interviewer: mmm (showing that one is following the conversation)

Respondent: yeah, so some of the clients are discouraged, they say it was good with Sayana, should we be coming often to the facility, we are working women…

Interviewer: mmm (Showing that one is following the conversation)

Respondent: If we have the dosage, we just inject ourselves after we come back from work, or before we go to work in the morning, we just inject ourselves when we are due for the injection.

Interviewer: mmmm (showing that one is following the conversation)

Respondent: Yeah, so you should try and find for us, then we say I will try to find if it is available at the DHO, I don’t know how the DHO does it, but the one available there is not enough…

Title: MZs_IM_8.docx

Descriptor Info:	District: Mzimba South	Target Population: TP2 - DMPA-IM/DMPA-SC PI clients	Respondent type: DMPA-IM client	Aware of SI: Yes - had heard of SI before	Coding phase: 1. Single coded	Client marital status: Married	Client age group: 20-29 years	Time since trained: N/A - client

Codes Applied:	C3.3.1 - Recos for what should be covered in counselling / training	C1.1.1 - Positive client attitudes to SI	C2.1.2 - Reduced trips to facility

Interviewer: what should a woman planning to get Sayana for self-injection know?

Respondent: how to hold a needle. It is difficult to do it if you have not been taught. But if they do it is easy.

Interviewer: what exactly are they supposed to know?

Respondent: she should be counselled

Interviewer: after watching the video, has your perception on self-injection changed?

Respondent: yes, I have learnt some new things. The way the woman explained self-injection is good especially in this era of COVID-19 and also you have time at home to do your things. It also reduces costs of travelling.

Title: MZs_IM_8.docx

Descriptor Info:	District: Mzimba South	Target Population: TP2 - DMPA-IM/DMPA-SC PI clients	Respondent type: DMPA-IM client	Aware of SI: Yes - had heard of SI before	Coding phase: 1. Single coded	Client marital status: Married	Client age group: 20-29 years	Time since trained: N/A - client

Codes Applied:	C2.1.2 - Reduced trips to facility	C3.3.1 - Recos for what should be covered in counselling / training	C1.2.2 - Fear of SI (idea or reality)

Interviewer: have you ever used self-injection?

Respondent: no

Interviewer: why not?

Respondent: I had fears on self-injecting

Interviewer: did you ever ask the provider to help you on your fears?

Respondent: no

Interviewer: do you think women on Depo-IM might be more interested with Sayana?

Respondent: yes, a lot

Interviewer: why do you think many switch to self-injection?

Respondent: they see it as a way of protecting themselves from COVID-19 because those who get Depo-IM come in large numbers

Interviewer: do you think different women may have different views on self-injection?

Respondent: yes, because it depends with the understanding of each one of them

Interviewer: what do you think should a woman know before getting self-injection?

Respondent: she just needs to be counselled that’s when she would know

Title: MZs_SCSI_3.docx

Descriptor Info:	District: Mzimba South	Target Population: TP3 - DMPA-SC SI clients	Respondent type: DMPA-SC SI client	Aware of SI: Yes - had heard of SI before	Coding phase: 1. Single coded	Client marital status: Married	Client age group: 30-39 years	Time since trained: N/A - client

Codes Applied:	C2.1.2 - Reduced trips to facility

Interviewer: what did you like about self-injection?

Respondent: we come from a far. So they said those who will manage to self-inject will be given 3 doses to use at home for the whole year.

Interviewer: anything else?

Respondent: it’s the same reason, I would save costs of travelling

Title: MZs_SCSI_3.docx

Descriptor Info:	District: Mzimba South	Target Population: TP3 - DMPA-SC SI clients	Respondent type: DMPA-SC SI client	Aware of SI: Yes - had heard of SI before	Coding phase: 1. Single coded	Client marital status: Married	Client age group: 30-39 years	Time since trained: N/A - client

Codes Applied:	C2.1.2 - Reduced trips to facility

Interviewer: what do you think a woman needs to know on self-injection?

Respondent: they need to know that it saves time. And also it helps to prevent covid-19 since we do not come here

Title: MZs_IM_2.docx

Descriptor Info:	District: Mzimba South	Target Population: TP2 - DMPA-IM/DMPA-SC PI clients	Respondent type: DMPA-IM client	Aware of SI: Yes - had heard of SI before	Coding phase: 3. Triple coded	Client marital status: Married	Client age group: 30-39 years	Time since trained: N/A - client

Codes Applied:	C1.1.1 - Positive client attitudes to SI	C2.1.2 - Reduced trips to facility

Interviewer: How have you seen this video?

Respondent: The video is good and may be the provider saw that I don’t show any interest. I have seen that there is no different with the one I am using even though I didn’t want to change but I have seen that this is good cause there is no need to close my grocery and go to the facility to be injected, while this one you can just self-inject even in the shop.

Title: MZs_IM_2.docx

Descriptor Info:	District: Mzimba South	Target Population: TP2 - DMPA-IM/DMPA-SC PI clients	Respondent type: DMPA-IM client	Aware of SI: Yes - had heard of SI before	Coding phase: 3. Triple coded	Client marital status: Married	Client age group: 30-39 years	Time since trained: N/A - client

Codes Applied:	C1.1.1 - Positive client attitudes to SI	C2.1.2 - Reduced trips to facility	C5.1.4 - Timely injection due to not having to rely on external factors

Interviewer: Do you think some types of women may be more interested than others?

Respondent: Yes, some women might be more interested

Interviewer: Why do you think so?

Respondent: The method will help them to have child spacing and also the time they would have used to go to the facility they will use it for other things, like if in that area there is funeral and it’s their date to go for re-inject, they will just self-inject and go to the funeral.

Title: KK_HSA_1.docx

Descriptor Info:	District: Nkhotakota	Target Population: TP1 - Providers/HSAs	Respondent type: HSA	Aware of SI: Yes - had heard of SI before	Coding phase: 2. Double coded	Client marital status: N/A - provider	Client age group: N/A - provider	Time since trained: A year or more ago

Codes Applied:	C2.1.2 - Reduced trips to facility	C2.1.1 - Community availability of injectables/SI

Interviewer: Apart form these role models who encourage them to self inject what is…what are…what are the other factors you think have encouraged?

Respondent: Walking right?

Interviewer: mmmm

Respondent: Distance from the community for them to come here to get Sayana yet an HSA right in the community is helping…you are giving them the methods. So they see that they should be helped and not loose time going to the health centre mmhu. This is an advantage, when you give them and when they have run out, they will meet the HSA, my supply is finished.

Title: KK_HSA_1.docx

Descriptor Info:	District: Nkhotakota	Target Population: TP1 - Providers/HSAs	Respondent type: HSA	Aware of SI: Yes - had heard of SI before	Coding phase: 2. Double coded	Client marital status: N/A - provider	Client age group: N/A - provider	Time since trained: A year or more ago

Codes Applied:	C2.1.2 - Reduced trips to facility	C5.1.4 - Timely injection due to not having to rely on external factors

Respondent: So I have said distance right?

Interviewer: Distance.

Respondent: Distance, secre…secre…confidentiality. Another one, it is easy. It is easy because as we are saying, they are not supposed to stay on the line or waiting so that they should do this and that. They already have the drug at home, they will just take the Sayana and self-inject, so it is easy.

Interviewer: mmmmhu

Respondent: mmmh

Title: ZA_HSA_5.docx

Descriptor Info:	District: Zomba	Target Population: TP1 - Providers/HSAs	Respondent type: HSA	Aware of SI: Yes - had heard of SI before	Coding phase: 2. Double coded	Client marital status: N/A - provider	Client age group: N/A - provider	Time since trained: Less than a year ago

Codes Applied:	C1.1.1 - Positive client attitudes to SI	C2.1.2 - Reduced trips to facility	C5.1.4 - Timely injection due to not having to rely on external factors

Respondent: They say that with this method a woman can no longer bleed

Interviewer: Mmm (showing that one is following the conversation)

Respondent: You just live but also, it’s a method that takes more time for one to go back to the hospital.

Interviewer: Yah

Respondent: Especially for Sayana since we give them, and it last them a whole year

Interviewer: Mmm (showing that one is following the conversation)

Respondent: Until they come to the hospital.

Interviewer: Yah yah

Respondent: So, they are able to do other chores instead of coming here to be in large crowds so that they get Depo or Sayana

Interviewer: Okay

Respondent: Eh

Title: ZA_HSA_5.docx

Descriptor Info:	District: Zomba	Target Population: TP1 - Providers/HSAs	Respondent type: HSA	Aware of SI: Yes - had heard of SI before	Coding phase: 2. Double coded	Client marital status: N/A - provider	Client age group: N/A - provider	Time since trained: Less than a year ago

Codes Applied:	C2.1.2 - Reduced trips to facility	C1.1.1 - Positive client attitudes to SI

Interviewer: Alright, alright, wat do you think attracts women more about DMPA-SC?

Respondent: What attracts women is that, since the areas we work in are hard to reach areas yeah

Interviewer: Mmm (showing that one is following the conversation)

Respondent: So, when we start our work, we have so many clients.

Interviewer: Mmm (showing that one is following the conversation)

Respondent: So, they spend more time there before they get back home.

Interviewer: Mmm (showing that one is following the conversation)

Respondent: That’s what was attracting more women yesterday that no, we cannot be doing it like this it’s better that you train me so I can self-inject. You say I will only come back next year, which is way long ahead, so that is when the women accept it because they spend more time waiting to get back home to their chores.

Interviewer: Alright.

Respondent: Yes

Interviewer: Alright, do women have concerns on using DMPA-SC?

Respondent: No, they don’t.

Title: ZA_HSA_5.docx

Descriptor Info:	District: Zomba	Target Population: TP1 - Providers/HSAs	Respondent type: HSA	Aware of SI: Yes - had heard of SI before	Coding phase: 2. Double coded	Client marital status: N/A - provider	Client age group: N/A - provider	Time since trained: Less than a year ago

Codes Applied:	C2.1.2 - Reduced trips to facility	C5.1.4 - Timely injection due to not having to rely on external factors

Interviewer: Alright, Now I would like to hear your thoughts, in your view what are the factors that would support a client to take up the self-injection option.

TIME INTO INTERVIEW: 40Mins 4Secs

Respondent: What could support…

Interviewer: Mmm (showing that one is following the conversation)

Respondent: The first thing is what I have said that it makes them to have so much time before they come back to the hospital, maybe some have moved on short notice from the area, yeah

Interviewer: Mmm (showing that one is following the conversation)

Respondent: So, they will not have time to come back to the hospital.

Interviewer: Yah

Respondent: So, the due dates for reinjection have already passed

Interviewer: Mmm (showing that one is following the conversation)

Respondent: That’s an advantage.

Interviewer: Mmm (showing that one is following the conversation)

Respondent: The time factor, but also coming here to the facility in this period of covid-19

Interviewer: Mmm (showing that one is following the conversation)

Respondent: There won’t be so many clients, so many of the women will have the injection units at home anytime that they want to inject, they can self-inject. Tats another advantage.

Title: ZA_HSA_5.docx

Descriptor Info:	District: Zomba	Target Population: TP1 - Providers/HSAs	Respondent type: HSA	Aware of SI: Yes - had heard of SI before	Coding phase: 2. Double coded	Client marital status: N/A - provider	Client age group: N/A - provider	Time since trained: Less than a year ago

Codes Applied:	C1.1.1 - Positive client attitudes to SI	C2.1.2 - Reduced trips to facility	C5.1.2 - Clients correctly administering SI at home

Interviewer: Alright, what do you think would discourage women from using DMPA-SC?

Respondent: What can discourage women?

Interviewer: yes

Respondent: Ah I don’t see anything discouraging.

Interviewer: yah

Respondent: Because the women have accepted the method because of Depo which they already know yeah

Interviewer: Mmm (showing that one is following the conversation)

Respondent: This was just like an addition so that the women should not be busied by going to the facility.

Interviewer: Mmm (showing that one is following the conversation)

Respondent: Only if they encountered a problem, then that would discourage them

Interviewer: Yah

Respondent: Because if we are injecting 100 women and there is no one who is coming back to us to complain about Sayana Press, then it means there is no problem, so they cannot be discouraged, but if they are bringing in issues then they would have been discouraged

Title: MZs_SCSI_1.docx

Descriptor Info:	District: Mzimba South	Target Population: TP3 - DMPA-SC SI clients	Respondent type: DMPA-SC SI client	Aware of SI: Yes - had heard of SI before	Coding phase: 1. Single coded	Client marital status: Married	Client age group: Under 20 (adolescent)	Time since trained: N/A - client

Codes Applied:	C2.1.2 - Reduced trips to facility

Interviewer: What was your first reaction when you heard about woman can inject herself?

Respondent: As for me it was a good thing because I didn’t like the idea of coming to the facility each and every after 3 months because of this I was very happy that I will be injecting myself.

Interviewer: What concerns did you have?

Respondent: I had no concerns

Interviewer: What where the important factors motivating you to try it?

Respondent: I saw it was a good idea for me to take this self – injection method so that I will not be visiting the hospital regularly and I was also told that at the hospital they use a very big syringe compared to Sayana Press the needle is very small

Title: MZs_SCSI_1.docx

Descriptor Info:	District: Mzimba South	Target Population: TP3 - DMPA-SC SI clients	Respondent type: DMPA-SC SI client	Aware of SI: Yes - had heard of SI before	Coding phase: 1. Single coded	Client marital status: Married	Client age group: Under 20 (adolescent)	Time since trained: N/A - client

Codes Applied:	C1.1.1 - Positive client attitudes to SI	C2.1.2 - Reduced trips to facility

Interviewer: Tell me the things you like about this self-inject

Respondent: The self-inject is better than me going to the facility and get injection

Interviewer: Anything you want to add?

Respondent: No

Title: KK_HSA_5.docx

Descriptor Info:	District: Nkhotakota	Target Population: TP1 - Providers/HSAs	Respondent type: HSA	Aware of SI: Yes - had heard of SI before	Coding phase: 1. Single coded	Client marital status: N/A - provider	Client age group: N/A - provider	Time since trained: A year or more ago

Codes Applied:	C1.1.1 - Positive client attitudes to SI	C2.1.2 - Reduced trips to facility	C1.1.3 - Awareness of DMPA-SC SI	P1.1.1 - Positive provider attitudes towards SI

INTERVIEWER: What specifically has influenced your opinion changing or not changing regarding DMPA-SC?

RESPONDENT: Yes, there is something that has influenced my opinion.

INTERVIEWER: Ok

RESPONDENT: The thing that has changed my opinion is the way women have welcome method. A thing we thought would not turn this way.

INTERVIEWER: Ok.

RESPONDENT: We have noticed that it is very possible. Most of the people in this community are farmers

INTERVIEWER: Okay

RESPONDENT: There has been fights in most of the homes during rainy season as their wives have been spending much of their time at the hospital. However, upon the coming of DMPA-SC the few women who started to take this method had communicated to their friends about this it, that it doesn’t require one to be visiting a facility now and then, that is why they have been home most of the times. This has prompted some more clients to come and try this method. In no time we just noticed that more women are coming for this method as it gives them ample time to do other things at home.

Title: KK_HSA_5.docx

Descriptor Info:	District: Nkhotakota	Target Population: TP1 - Providers/HSAs	Respondent type: HSA	Aware of SI: Yes - had heard of SI before	Coding phase: 1. Single coded	Client marital status: N/A - provider	Client age group: N/A - provider	Time since trained: A year or more ago

Codes Applied:	C1.2.2 - Fear of SI (idea or reality)	C1.1.3 - Awareness of DMPA-SC SI	C8.1 - Male involvement, general	C2.1.2 - Reduced trips to facility	P3.1.1 - Reduced provider workload

NTERVIEWER: Would you say the trend in DMPA-S uptake for self-injection has changed over time? What factors do you think have influenced this trend?

RESPONDENT: Yes, there has been a good change, the first one being that those women who were very much afraid to inject themselves are now able to do so on their own, because of how they have been strengthened by their fellow women are doing it.

INTERVIEWER: Alright

RESPONDENT: It has reduced tension in the homes by taking away bad thought that men sed to think that their wives were coming to the hospital not for treatment or family planning but were interested in male doctors.

INTERVIEWER: [interviewer laugh]

RESPONDENT: And male HSA’s. But now most of them know that there is a new method that allows their wives to visit the hospital only once in 6 months. And if they do it’s because a child is sick, whilst in the past women used to visit the hospital regularly that caused fights in the homes. They thought the nurses or HSA’s were delaying their wives deliberately, not knowing that the counselling process takes time. But now all that is history, hence men are encouraging their wives to go for this method so that most of the times are home. This change has done good as our work load has reduced than in the past that we used to have a lot of clients at once.

INTERVIEWER: Okay

RESPONDENT: These are some of the changes that has taken pace in this community.

Title: KK_HSA_5.docx

Descriptor Info:	District: Nkhotakota	Target Population: TP1 - Providers/HSAs	Respondent type: HSA	Aware of SI: Yes - had heard of SI before	Coding phase: 1. Single coded	Client marital status: N/A - provider	Client age group: N/A - provider	Time since trained: A year or more ago

Codes Applied:	C1.1.1 - Positive client attitudes to SI	C2.1.2 - Reduced trips to facility

INTERVIEWER: What in your experience, is the response when you explain to clients that they could self-inject this method. What do you think women find it most appealing about DMPA-SC for self-injection?

RESPONDENT: The most important thing is the fact that they the method has created an ample time for them to do other things in their homes. In addition is the size of the injection that is small that can easily be handled that results in less pain when doing self-injection.

INTERVIEWER: Alright

RESPONDENT: Sure

Title: MZs_HSA_1.docx

Descriptor Info:	District: Mzimba South	Target Population: TP1 - Providers/HSAs	Respondent type: HSA	Aware of SI: Yes - had heard of SI before	Coding phase: 2. Double coded	Client marital status: N/A - provider	Client age group: N/A - provider	Time since trained:

Codes Applied:	C2.1.2 - Reduced trips to facility	C0.2 - Community and client perspectives on FP - general

Interviewer: Which methods are the common ones among adults and adolescents?

Respondent: You mean the ones they use frequently?

Interviewer: Yes, the frequent ones for both adults and adolescents?

Respondent: For the adolescents mostly they use providers injectables and this is so because most of them they use it secretly thus why they use providers injection they don’t want self-injection.

Interviewer: You have just mention adolescents what about adults?

Respondent: For adults most of them they use Sayana press-self injection, so that they should reduce visiting the facility/HSA`s regularly, thus why they love self-injection.

Title: MZs_HSA_1.docx

Descriptor Info:	District: Mzimba South	Target Population: TP1 - Providers/HSAs	Respondent type: HSA	Aware of SI: Yes - had heard of SI before	Coding phase: 2. Double coded	Client marital status: N/A - provider	Client age group: N/A - provider	Time since trained:

Codes Applied:	P1.1.1 - Positive provider attitudes towards SI	C2.1.2 - Reduced trips to facility	P4.3 - Follow up approach

Interviewer: So tell me about it. The good as well as the bad ones that someone can come across upon using Sayana self-injection and also how can a women self-inject herself.

Respondent: A women who uses Sayana self-injection will only be visiting the facility once a year and this method is very convenient because once a woman chooses to use this method the chances of getting pregnant are very low.

Interviewer: Mmmmh. (following)

Respondent: Yes. And this Sayana self-injection will give a chance to women to do other things since they will only be visiting the facility once a year. We also tell them that with this method women can experience lighter or heavy periods or sometimes no periods at all. But if women can experience overflowing of periods, then she should visit the facility immediately for help.

Title: MZs_HSA_1.docx

Descriptor Info:	District: Mzimba South	Target Population: TP1 - Providers/HSAs	Respondent type: HSA	Aware of SI: Yes - had heard of SI before	Coding phase: 2. Double coded	Client marital status: N/A - provider	Client age group: N/A - provider	Time since trained:

Codes Applied:	P1.1.1 - Positive provider attitudes towards SI	C2.1.2 - Reduced trips to facility	C1.1.1 - Positive client attitudes to SI	C3.1 - Enablers, general/other

Q17. Interviewer: In your view, what are the main factors that would support a client to take up the self-injection option with DMPA-SC?

Respondent: The main factor is that it saves time to a client by not visiting the facility regularly and spending her time on other things

Interviewer: Mmmmh (following)

Respondent: Yes

Interviewer: Do you have anything to add?

Respondent: The Sayana needle is very small thus why women do accept to self-inject.

Title: MZs_HSA_1.docx

Descriptor Info:	District: Mzimba South	Target Population: TP1 - Providers/HSAs	Respondent type: HSA	Aware of SI: Yes - had heard of SI before	Coding phase: 2. Double coded	Client marital status: N/A - provider	Client age group: N/A - provider	Time since trained:

Codes Applied:	C2.1.2 - Reduced trips to facility

Interviewer: Are there enabling factors that can make a woman choose self-injection?

Respondent: There are some women who come very far for them to reach where family planning methods are offered and others who are doing business, they might like the self-inject so that they save time by not visiting the facility or HAS’s regularly

Title: KK_FAC_1.docx

Descriptor Info:	District: Nkhotakota	Target Population: TP1 - Providers/HSAs	Respondent type: Facility-based provider	Aware of SI: Yes - had heard of SI before	Coding phase: 3. Triple coded	Client marital status: N/A - provider	Client age group: N/A - provider	Time since trained: A year or more ago

Codes Applied:	C2.1.2 - Reduced trips to facility	C1.1.1 - Positive client attitudes to SI

INTERVIEWER: Okay , what do you think women find most appealing about DMPA-SC for self -injection?

RESPONDENT: The issue is that since we give them enough doses to take them a year, they feel they have saves time to visit the facility as they will be just doing it at home.

INTERVIEWER: Okay

RESPONDENT: So most of them get excited that they have a dose for a year

Title: KK_IM_5.docx

Descriptor Info:	District: Nkhotakota	Target Population: TP2 - DMPA-IM/DMPA-SC PI clients	Respondent type: DMPA-IM client	Aware of SI: No - never heard of SI before	Coding phase: 3. Triple coded	Client marital status: Divorced/ separated/ widowed	Client age group: Under 20 (adolescent)	Time since trained: N/A - client

Codes Applied:	C2.1.2 - Reduced trips to facility	C8.3 - Discretion of injectables/SI

INTERVIEWER: That’s why I said think of those adolescents who are 16, 17 and 15 if they can be told about self-injection method, would this method appeal them?

RESPONDENT: Yes, it can

INTERVIEWER: What is it that can appeal them?

RESPONDENT: Because they visit the hospital only once

INTERVIEWER: Yes

RESPONDENT: They will be given some more to take home

INTERVIEWER: Yes

RESPONDENT: Hence nobody would know that they are on family planning

INTERVIEWER: What else do you can appeal them?

RESPONDENT: Because they will avoid unplanned pregnancy

Title: ZA_IM_1.docx

Descriptor Info:	District: Zomba	Target Population: TP2 - DMPA-IM/DMPA-SC PI clients	Respondent type: DMPA-IM client	Aware of SI: Yes - had heard of SI before	Coding phase: 1. Single coded	Client marital status: Married	Client age group: 40-49 years	Time since trained: N/A - client

Codes Applied:	C3.3 - Counselling / training approach for injectable users	C1.2.2 - Fear of SI (idea or reality)	C3.1.2 - Confidence/comfort to inject after counselling/ training	C2.1.2 - Reduced trips to facility	C3.2.1 - Lack of SI practice during training	C3.4 - Time to train clients

Interviewer: How did you first hear about it?

Respondent: When I first heard this, I was nervous that would I be able to inject myself because we were told that we have to inject ourselves below the belly button or on the thigh. I was afraid about the belly button thinking I would inject my intestines or anything else. We were then properly taught on how to inject ourselves.

Interviewer: Mmmhh

Respondent: So ahh I accepted it and I used it.

Interviewer: Do you know anyone who is using self-injection?

Respondent: Self injection is currently out of stock, so I would not know

Interviewer: Mmmmhhh

Respondent: Yes

Interviewer: At the time it was available did you know anyone who was using it?

Respondent: Yes, I knew someone who was using it at that time

Interviewer: Alright, alright, have you spoken about DMPA-SC for self-injection with any provider?

Respondent: Yes, we have spoken about it

Interviewer: Mmmmhhh

Respondent: We were taught on how we can use the injection, how we can inject as well as the days when we are supposed to wait till the injection is ineffective in our bodies so that we can re inject the second one.

Interviewer: Can you tell me more about those conversations? How did the provider describe it to you? Tell me in detail what you were told?

Respondent: The provider explained that with this type of injection everyone is supposed to self-inject and the position is either below the belly button or on the thigh. So they explained on how to inject as well as the days we are supposed to wait for the medicine to be effective when re injecting.

Interviewer: Mmmmhhh

Respondent: They explained that when we first inject ourselves we should wait for 90days which is three months. After the 90 days we should inject and so on.

Interviewer: Mmmhhh alright, did they tell you the pros and cons of self-injection?

Respondent: They told us about the pros that sometimes people get lazy or get busy to get injected by provider. With self-injection there are no problems to inject yourself. These are the pros

Interviewer: Did they explain the cons of self-injection?

Respondent: No, they did not explain

Time stamp: 19 mins 38 secs

Interviewer: Alright thank you very much. As a result of the discussion you had with a provider, did you agree to try self-injection?

Respondent: Yes, I agreed and I tried it

Interviewer: Mmmmhhh What influenced you to try the self-injection?

Respondent: I saw that self-injection was a way that I could freely self-inject. The places where I inject are places were am free and no one is there.

Interviewer: What influenced you to self-inject?

Respondent: I was influenced with the fact that I want to family plan and avoid getting pregnant

Interviewer: Alright, alright, can you describe your experience of being trained in self-injection?

Respondent: The time they were explaining about self-injection

Interviewer: mmmhhh

Respondent: I was nervous that they are possibilities of hurting myself as well as not fully injecting due to pain as well as the medicine not getting inside. Once I fully understood I found that I accepted it and I started self-injecting without any difficulties

Interviewer: Alright, alright, in your view do you feel the provider spent enough time training you?

Respondent: Yes, the time was adequate

Interviewer: Did you get the chance to practice on what you were taught? For instance, pricking a condom filled with sugar or sand?

Respondent: No, we didn’t do that

Interviewer: Alright, did you self-inject?

Respondent: Yes

Interviewer: How can you describe the friendliness of the training you received?

Respondent: The friendliness of the training on self-injection?

Interviewer: Mmmmhhh

Respondent: It was easy because it was an easy way, it’s not like it was hard to understand when being taught. It was quite easy, and we practiced and got it.

Interviewer: Yes, what were you given to take home after your training for instance how many injectables?

Respondent: 3

Interviewer: Were you given any paper with instructions on how to self-inject at home?

Respondent: No, the just explained on how we can self-inject

Interviewer: Alright, were you given a calendar?

Respondent: Yes, we were given a calendar

Interviewer: So you got all these?

Respondent: Yes, we got them at that time

Title: ZA_IM_1.docx

Descriptor Info:	District: Zomba	Target Population: TP2 - DMPA-IM/DMPA-SC PI clients	Respondent type: DMPA-IM client	Aware of SI: Yes - had heard of SI before	Coding phase: 1. Single coded	Client marital status: Married	Client age group: 40-49 years	Time since trained: N/A - client

Codes Applied:	C1.1.1 - Positive client attitudes to SI	C3.3.1 - Recos for what should be covered in counselling / training	C2.1.2 - Reduced trips to facility

Interviewer: What else do you think women need to know about self-injection before trying it?

Respondent: They are supposed to the taught about the benefits because you take time without going to the hospital. You will have the injection with you at your house, when time arises to self-inject you do it. You take take before returning to the hospital to get more self-injection kits

Interviewer: Alright thank you. After watching the video, would you say you feel the same, more positive, or more negative about self-injection?

[Kids making noise in the background]

Respondent: No, they didn’t change I still want self-injection

Title: ZA_IM_1.docx

Descriptor Info:	District: Zomba	Target Population: TP2 - DMPA-IM/DMPA-SC PI clients	Respondent type: DMPA-IM client	Aware of SI: Yes - had heard of SI before	Coding phase: 1. Single coded	Client marital status: Married	Client age group: 40-49 years	Time since trained: N/A - client

Codes Applied:	C1.1.1 - Positive client attitudes to SI	C2.1.2 - Reduced trips to facility	C2.2.1 - Stockouts/stocks near expiry dates

Respondent: It’s not available at the moment, am asking that it should be made available its very good.

Interviewer: Alright thank you very much. You explained that you previously used self-injection, how many times did you use it?

Respondent: I used it twice

Interviewer: You were given 3?

Respondent: Yes, 3

Interviewer: You used all three?

Respondent: Yes I used them all, that was in 2019

Interviewer: Did you inject all 3?

Respondent: Yes

Interviewer: How do you view this method?

Respondent: I find this method to be very good. As I previously mentioned you rest from going to the hospital. We come from a distance, so you just inject yourself when time arises

Interviewer: So what made you to go back to receiving your injection from the hospital?

Respondent: This is because that self-injection is not available at this hospital, the only available one is the one that injectable at the hospital.

Interviewer: Alright alright, we have come to the end of the questions I had for you today. Do you have any other thoughts or information you would like to share on the topics we have discussed today?

Respondent: I have additions, as I earlier mentioned self-injection is not currently available. It is my plea we still want self-injection.

Interviewer: Alright alright, you last self-injected in 2019 you said?

Respondent: No, I last self-injected in 2020 then started receiving from from the hospital the the one that is administered on the hand.

Title: KK_SCPI_6.docx

Descriptor Info:	District: Nkhotakota	Target Population: TP2 - DMPA-IM/DMPA-SC PI clients	Respondent type: DMPA-SC PI client	Aware of SI: Yes - had heard of SI before	Coding phase: 1. Single coded	Client marital status: Married	Client age group: 20-29 years	Time since trained: N/A - client

Codes Applied:	C3.1.2 - Confidence/comfort to inject after counselling/ training	C1.2.2 - Fear of SI (idea or reality)	C2.1.2 - Reduced trips to facility	C1.1.1 - Positive client attitudes to SI

NTERVIEWER: okay. So you said the fears are gone after watching the video?

RESPONDENT: mmmh Yes, they are gone?

INTERVIEWER: You will start injecting yourself?

RESPONDENT: mmh Yes, I will be collecting.

INTERVIEWER: When are you coming again?

RESPONDENT: I will come on nineteen.

INTERVIEWER: Nineteenth of which month?

RESPONDENT: January.

INTERVIEWER: When you come in January you are going to get Sayana?

RESPONDENT: I will get. Maybe they will give me three and that will make me get settled at home without frequent visiting the hospital to be injected.

INTERVIEWER: okay.

RESPONDENT: mmmh.

INTERVIEWER: I have this question, have you tried to inject yourself with Sayana more than once? I should just say have you tried to inject yourself before?

RESPONDENT: No, I have never tried.

INTERVIEWER: You have never tried?

RESPONDENT: mmmh.

INTERVIEWER: Have they never tried to give you to try?

RESPONDENT: They gave me, but I said no.

INTERVIEWER: You refused?

RESPONDENT: mmmh.

INTERVIEWER: You said you were afraid right?

RESPONDENT: Yes, I was afraid.

INTERVIEWER: As you have watched the video, have your thoughts about Sayana changed?

RESPONDENT: mmmh.

INTERVIEWER: Yes

RESPONDENT: mmmh.

INTERVIEWER: They have completely changed?

RESPONDENT: Yes, they have changed a lot.

INTERVIEWER: the next appointment.

RESPONDENT: aaah I will say, just give me I should be going home, I should be at home in good time. Sometimes I could leave other things to do and rush to the hospital to be injected.

INTERVIEWER: okay. Will you allow to try while the doctor is watching?

RESPONDENT: Yes, I will allow.

INTERVIEWER: You will really inject yourself?

RESPONDENT: mmmh.

INTERVIEWER: All the shivering will.

RESPONDENT: Will completely go.

INTERVIEWER: All gone

RESPONDENT: mmmh.

INTERVIEWER: What will make you lose all the shivering?

RESPONDENT: The video is what has made me.

INTERVIEWER: You have really loved the video?

RESPONDENT: Aah I have loved it.

INTERVIEWER: Why?

RESPONDENT: The nurse is also good explaining everything.

Title: KK_SCSI_2.docx

Descriptor Info:	District: Nkhotakota	Target Population: TP3 - DMPA-SC SI clients	Respondent type: DMPA-SC SI client	Aware of SI: Yes - had heard of SI before	Coding phase: 1. Single coded	Client marital status: Divorced/ separated/ widowed	Client age group: 20-29 years	Time since trained: N/A - client

Codes Applied:	C1.1.1 - Positive client attitudes to SI	C2.1.2 - Reduced trips to facility	C1.2.2 - Fear of SI (idea or reality)	C1.2.1 - Negative client attitudes to SI	C5.1.4 - Timely injection due to not having to rely on external factors

INTERVIEWER: Alright, what was your reaction

RESPONDENT: I welcomed the idea since they said it is a good method because one can take two doses where by one would inject herself right away at the hospital whilst another dosage would be taken home to be injected later. Secondly, I stay far from the hospital and also it was rainy season so I felt it would do me good to use this method.

INTERVIEWER: What appealed to you about the ability to self-injection. I can see you have explained about the distance from hospital to your home is far so you thought it was better for you that you will not come back for another dosage in a short period of time.

RESPONDENT: Yes

INTERVIEWER: What concerns did you have, if any.

RESPONDENT: Yes, I had some concerns

INTERVIEWER: What type of concerns

RESPONDENT: My concern was what if I make a mistake in administering the dosage will I not hurt myself

INTERVIEWER: hehehe (laughing) was it the only concern?

RESPONDENT: It was a big one

INTERVIEWER: What was it all about

RESPONDENT: If I make a mistake in administering it, different to what the doctor said and then I go back to report to the doctor will they not shout at me.

INTERVIEWER: Alright, that you have missed the doctor’s advice

RESPONDENT: Yes

INTERVIEWER: Alright, what were the most important factors motivating you to try self-injection method? What factors motivated you though you had some concerns about the method as you did put it that you had some concerns as to whether you will not hurt yourself when injecting yourself.

RESPONDENT: Yes

INTERVIEWER: But what motivated you the most to try Sayana

RESPONDENT: the reason was simple as I have already said, it will be like am saying the same thing that if I get the dosage, I will use one at the hospital whilst keep the other one to take home. I will just be checking when am I supposed to inject myself. Because in most cases you found out that you need to go for a family planning method, but you are busy, you end up missing the date there by resulting in getting pregnant.

INERVIEWER: Alright fine. Has your perspective changed over time on taking self-injection method? You decided to take self-injection method not so? Has your perspective changed over time?

RESPONDENT: No has not changed

Title: KK_SCSI_2.docx

Descriptor Info:	District: Nkhotakota	Target Population: TP3 - DMPA-SC SI clients	Respondent type: DMPA-SC SI client	Aware of SI: Yes - had heard of SI before	Coding phase: 1. Single coded	Client marital status: Divorced/ separated/ widowed	Client age group: 20-29 years	Time since trained: N/A - client

Codes Applied:	C5.1.1 - Continuation of SI (including reasons)	C2.1.2 - Reduced trips to facility

INTERVIEWER: Are you currently still using DMPA-SC for self-injection?

RESPONDENT: I have temporary stopped

INTERVIEWER: Why is it so? (the silence is due to interruption by an intruder who was enquiring about ART services in the room where we had the interviews ) you said you stopped

RESPONDENT : Yes

INTERVIEWER: Why did you discontinue?

RESPONDENT: It is because I do not have a husband at the moment.

INTERVIEWER: Alright, would you still consider using DMPA-SC again in future?

RESPONDENT: Yes

INTERVIEWER: why is it so?

RESPONDENT: Because it is so helpful

INTERVIEWER: How helpful is it?

RESPONDENT: It is helpful in a way that if you inject yourself today , it will take some time before you visit the hospital since you will be just checking time for next dose and do again.

Title: MZs_HSA_5.docx

Descriptor Info:	District: Mzimba South	Target Population: TP1 - Providers/HSAs	Respondent type: HSA	Aware of SI: Yes - had heard of SI before	Coding phase: 1. Single coded	Client marital status: N/A - provider	Client age group: N/A - provider	Time since trained: A year or more ago

Codes Applied:	C2.1.2 - Reduced trips to facility	C8.1 - Male involvement, general	P3.5.1 - Trends/numbers of SI

Interviewer: in the past when you had it in stock, for the 100 women who took Sayana, how many took for self-injection?

Respondent: at first there were a few who were confident to get self-injection

Interviewer: so on 100 women who took Sayana, how many took for self-injection?

Respondent: 70-75 only 25 would ask us to inject them

Interviewer: what made more women choose Sayana?

Respondent: most women like it because they have reduced number of clinic visits which brings trust to their spouse unlike when they visit clinics frequently

Title: MZs_HSA_5.docx

Descriptor Info:	District: Mzimba South	Target Population: TP1 - Providers/HSAs	Respondent type: HSA	Aware of SI: Yes - had heard of SI before	Coding phase: 1. Single coded	Client marital status: N/A - provider	Client age group: N/A - provider	Time since trained: A year or more ago

Codes Applied:	C1.1.1 - Positive client attitudes to SI	C2.1.2 - Reduced trips to facility

Interviewer: you said out of 100, about 75 could get Sayana, what do you think were factors that influenced them?

Respondent: firstly, it’s easy to self-inject because the needle is small. Second is that we give them three doses to take home, so they stay for the whole year without coming again.

Title: MZs_HSA_5.docx

Descriptor Info:	District: Mzimba South	Target Population: TP1 - Providers/HSAs	Respondent type: HSA	Aware of SI: Yes - had heard of SI before	Coding phase: 1. Single coded	Client marital status: N/A - provider	Client age group: N/A - provider	Time since trained: A year or more ago

Codes Applied:	P1.1.1 - Positive provider attitudes towards SI	C2.1.2 - Reduced trips to facility	C1.1.1 - Positive client attitudes to SI

Interviewer: we are now going to talk about what makes other women choose Sayana compared to others. In your view, what makes other women choose Sayana compared to others?

Respondent: like I have already said it is easy to use you do not visit the facility regularly. It is easy to self-inject.

Title: KK_HSA_3.docx

Descriptor Info:	District: Nkhotakota	Target Population: TP1 - Providers/HSAs	Respondent type: HSA	Aware of SI: Yes - had heard of SI before	Coding phase: 3. Triple coded	Client marital status: N/A - provider	Client age group: N/A - provider	Time since trained: A year or more ago

Codes Applied:	P1.2.1 - Negative provider attitudes towards SI	P1.1.1 - Positive provider attitudes towards SI	C2.1.2 - Reduced trips to facility	C2.1.1 - Community availability of injectables/SI

INTERVIEWER: In its first stages before it was implemented, when you had just heard. Were you in support that Sayana should be introduced?

RESPONDENT: it was difficult for us to accept it because we did not know its end results. But after it was introduced, we saw that it was a good thing and then we accepted it.

INTERVIEWER: mmmhu. So what made you change your mind? What made you to accept it?

RESPONDENT: What made me change my mind was that I realized that Sayana is a good method because with other methods, women were walking long distances to access contraceptives. But now a lot of women see the importance of this method because they access it right there in their community, in the field.

Title: KK_HSA_3.docx

Descriptor Info:	District: Nkhotakota	Target Population: TP1 - Providers/HSAs	Respondent type: HSA	Aware of SI: Yes - had heard of SI before	Coding phase: 3. Triple coded	Client marital status: N/A - provider	Client age group: N/A - provider	Time since trained: A year or more ago

Codes Applied:	C2.1.2 - Reduced trips to facility	C5.1.4 - Timely injection due to not having to rely on external factors

INTERVIEWER: Okay. What do you think motivates them to say yes to self-injection?

RESPONDENT: Mmmhu. What motivates them for self-injection is that they don’t waste their time going to an HSA. For example, someone on self-injection and someone who is not. The latter will be looking for an HSA and she might not find him or might find him busy, but it is her date for an injection. But when they have Sayana at home, they just self-inject.

Title: KK_SCPI_4.docx

Descriptor Info:	District: Nkhotakota	Target Population: TP2 - DMPA-IM/DMPA-SC PI clients	Respondent type: DMPA-SC PI client	Aware of SI: Yes - had heard of SI before	Coding phase: 2. Double coded	Client marital status: Never married	Client age group: 20-29 years	Time since trained: N/A - client

Codes Applied:	C2.1.2 - Reduced trips to facility	C1.1.1 - Positive client attitudes to SI

INTERVIEWER: mmmhu. What do you think are the most important things that are in the video?

RESPONDENT: The most important things are that, when they give you Sayana it is like they are saving you on the distance to come here to get more. Because they give you three of them that means the whole year. So when you come again, they also give you three that means you have added another year that is two years.

Title: ZA_HSA_4.docx

Descriptor Info:	District: Zomba	Target Population: TP1 - Providers/HSAs	Respondent type: HSA	Aware of SI: Yes - had heard of SI before	Coding phase: 1. Single coded	Client marital status: N/A - provider	Client age group: N/A - provider	Time since trained: A year or more ago

Codes Applied:	P1.1.1 - Positive provider attitudes towards SI	P3.1.1 - Reduced provider workload	C2.1.2 - Reduced trips to facility

Interviewer: Aah in your community when you first heard about the self-injection how did you and your colleagues feel?

Respondent: I felt very good because it minimized our workload and also time spent by women coming here to get the injection. They have time to do other things because they will just inject themselves doing other things whilst coming here is time consuming.

Interviewer: Has your opinion or your colleagues on self-injection changed over time?

Respondent: What kind of change?

Interviewer: You said that you accepted this method and it’s good, did your thoughts change since administering this injection?

Respondent: No, they did not change

Title: ZA_HSA_4.docx

Descriptor Info:	District: Zomba	Target Population: TP1 - Providers/HSAs	Respondent type: HSA	Aware of SI: Yes - had heard of SI before	Coding phase: 1. Single coded	Client marital status: N/A - provider	Client age group: N/A - provider	Time since trained: A year or more ago

Codes Applied:	C2.1.2 - Reduced trips to facility	C5.1.2 - Clients correctly administering SI at home

Interviewer: What do you think is the reason why women opt for the self-injecting method according to the three women who came for this method?

Respondent: They said that they will no longer have to walk to get the contraception methods from their homes to the hospital.

Interviewer: Alright. Thank you so much. Do women have any concerns upon the self-injecting method?

Respondent: Aaah when I asked them, they said they no longer have any fear because they are used to it, and they said that they are experts now. So, we tell them to be encouraging their fellow women.

Title: ZA_HSA_4.docx

Descriptor Info:	District: Zomba	Target Population: TP1 - Providers/HSAs	Respondent type: HSA	Aware of SI: Yes - had heard of SI before	Coding phase: 1. Single coded	Client marital status: N/A - provider	Client age group: N/A - provider	Time since trained: A year or more ago

Codes Applied:	C2.1.2 - Reduced trips to facility	P1.1.1 - Positive provider attitudes towards SI	C4 - Storage of unused units

Interviewer: What do you think is the reason why women may go for the self-injecting contraception method?

Respondent: They choose the method to save time, the way this vaccine is injected is simple as compared to Depo, the storage is easy.

Title: ZA_IM_6.docx

Descriptor Info:	District: Zomba	Target Population: TP2 - DMPA-IM/DMPA-SC PI clients	Respondent type: DMPA-IM client	Aware of SI: Yes - had heard of SI before	Coding phase: 1. Single coded	Client marital status: Married	Client age group: 30-39 years	Time since trained: N/A - client

Codes Applied:	C2.1.2 - Reduced trips to facility

Interviewer: After hearing this information, do you think women who are already using injectable methods might be interested in self injection Do you think they would be interested, or would women like you be interested in going for this contraceptive method?

Respondent: Yes, they would, since we will be injecting ourselves at home.

Interviewer: Mhhmm

Respondent: We will not have to come all the way here.

Title: ZA_SCSI_3.docx

Descriptor Info:	District: Zomba	Target Population: TP3 - DMPA-SC SI clients	Respondent type: DMPA-SC SI client	Aware of SI: Yes - had heard of SI before	Coding phase: 3. Triple coded	Client marital status: Married	Client age group: 40-49 years	Time since trained: N/A - client

Codes Applied:	C1.1.1 - Positive client attitudes to SI	C2.1.2 - Reduced trips to facility	C5.1.4 - Timely injection due to not having to rely on external factors

Interviewer: Okay, now I would like to discuss with you about your experiences while using the self-injection method. Can you tell me where you first heard of the self-injection method of family planning and how did you react or what did you do about it?

Respondent: I heard from the HSA of our area who explained to us about it and asked who would manage among us. I was one of those who agreed and since I started self-injecting, I haven’t experienced any problems. I also find it easy since I don’t have to go to the place where they provide the antenatal services just so I can access the family planning method because I have it at home and when my day comes, I just inject myself at home. It also helps in times when the day I am supposed to inject myself is on a weekend that means I can’t miss any days because I have the drug back home.

Title: ZA_SCSI_3.docx

Descriptor Info:	District: Zomba	Target Population: TP3 - DMPA-SC SI clients	Respondent type: DMPA-SC SI client	Aware of SI: Yes - had heard of SI before	Coding phase: 3. Triple coded	Client marital status: Married	Client age group: 40-49 years	Time since trained: N/A - client

Codes Applied:	C2.1.2 - Reduced trips to facility	C5.1.4 - Timely injection due to not having to rely on external factors

Interviewer: Thank you very much. What are the things that encouraged you to start taking the self-injection method?

Respondent: The fact that the distance of coming to the hospital is long, sometimes the day to inject oneself would be on weekends and hospitals are not open on weekends meaning that if one can miss the day she is supposed to get the injection. So I thought it would be easier if I can just inject myself at my own time while having the time to do other things and I can also be assured that even if my injection day is on a week end, I can still inject myself at home.

Interviewer: Thank you very much. Have your views or thoughts towards self-injecting changed recently?

Respondent: No, they are the same.

Title: ZA_SCSI_3.docx

Descriptor Info:	District: Zomba	Target Population: TP3 - DMPA-SC SI clients	Respondent type: DMPA-SC SI client	Aware of SI: Yes - had heard of SI before	Coding phase: 3. Triple coded	Client marital status: Married	Client age group: 40-49 years	Time since trained: N/A - client

Codes Applied:	C1.1.1 - Positive client attitudes to SI	C2.1.2 - Reduced trips to facility	C88 - Client great quotes	C3.3.1 - Recos for what should be covered in counselling / training

Interviewer: What other thing do you think women need to know about self-injection?

Respondent: What women can know about this method is that it reduces the time we spend going to the hospital. For example, I and the women from my area have to come a long way just to reach the hospital. It is hard for us to make time for other farming activities and coming to the hospital, on top of that some of us do some peace works, so it is difficult to divide our time. In the end, we end up postponing the days we are supposed to visit the hospital. All this can however be avoided if we have the injections at our homes.

Interviewer: So what are women supposed to know about the self-injection method?

Respondent: They should know that this method saves one’s time of visiting the hospital. So it is a good method.

Title: ZA_SCSI_3.docx

Descriptor Info:	District: Zomba	Target Population: TP3 - DMPA-SC SI clients	Respondent type: DMPA-SC SI client	Aware of SI: Yes - had heard of SI before	Coding phase: 3. Triple coded	Client marital status: Married	Client age group: 40-49 years	Time since trained: N/A - client

Codes Applied:	C3.3.2 - Good (sufficient/ non-judgemental) counselling/training	C2.1.2 - Reduced trips to facility	C1.2.1 - Negative client attitudes to SI	C1.1.1 - Positive client attitudes to SI

Interviewer: After finishing watching this video, what do you feel about the teaching you got?

Time stamp: 30:46

Respondent: After watching, I am now convinced that what we learnt is actually true and they also told that the things explained in the video happen. At first, I thought they were just trying to reduce their work or burden us with the job of injecting ourselves. Now I have been encouraged that this method is actually helpful since it helps reduce our trips to the hospital since we can check the calendars for our days and inject ourselves. This method is good, and a woman can successfully do family planning using it.

Title: ZA_SCSI_3.docx

Descriptor Info:	District: Zomba	Target Population: TP3 - DMPA-SC SI clients	Respondent type: DMPA-SC SI client	Aware of SI: Yes - had heard of SI before	Coding phase: 3. Triple coded	Client marital status: Married	Client age group: 40-49 years	Time since trained: N/A - client

Codes Applied:	C1.1.1 - Positive client attitudes to SI	C2.1.2 - Reduced trips to facility	C5.1.4 - Timely injection due to not having to rely on external factors

Interviewer: Okay fine. What do you find interesting when using the self-injecting method?

Respondent: Like I said, it reduces the trips that I make to the hospital as well as the time I spend there on a queue when at home I can just choose if I will inject myself when I just wake up or when am planning to sleep.

Interviewer: Is there anything that you don’t like about using the self-injection method?

Respondent: Nothing.

Interviewer: Now we have reached the final part of our interview. But before we part, I have some few final questions. Would you advise an encourage women in your area to do family planning?

Respondent: Yes, I would encourage them very much to do so.

Interviewer: Why would you encourage these women to take self-injection family planning?

Respondent: I do so because I and the women from my area live very far from the hospital as such takes time to reach here. And when the HSA of our area does not come that day for instance due to a funeral, it means that the women will not get injected. But if you have the injection at your home, there is no need to go the hospital. Even if the funeral is at your house, you can just go to your room and inject yourself there.

Title: ZA_SCSI_2.docx

Descriptor Info:	District: Zomba	Target Population: TP3 - DMPA-SC SI clients	Respondent type: DMPA-SC SI client	Aware of SI: Yes - had heard of SI before	Coding phase: 1. Single coded	Client marital status:	Client age group:	Time since trained: N/A - client

Codes Applied:	C1.1.1 - Positive client attitudes to SI	C1.1.3 - Awareness of DMPA-SC SI	C8.3 - Discretion of injectables/SI	C2.1.2 - Reduced trips to facility

Interviewer: What do they say about family planning, you say they like family planning, what do they say?

Respondent: Self-injection is good because people don’t know that you are using contraceptives.

Interviewer: mmmm (showing that one is following the conversation) Do women and girls in your community talk about the family planning methods that they are using?

Respondent: Yes, they are able to discuss, when this method just came the HSAs explained that there is a new method, so the people started using that method, so it is good, you don’t have to go and get injected.

Interviewer: mmmm (showing that one is following the conversation)

Respondent: you just take it and go to self-inject at home.

Title: ZA_SCSI_2.docx

Descriptor Info:	District: Zomba	Target Population: TP3 - DMPA-SC SI clients	Respondent type: DMPA-SC SI client	Aware of SI: Yes - had heard of SI before	Coding phase: 1. Single coded	Client marital status:	Client age group:	Time since trained: N/A - client

Codes Applied:	C1.1.1 - Positive client attitudes to SI	C8.3 - Discretion of injectables/SI	C2.1.2 - Reduced trips to facility

Interviewer: Alright, when you first heard about this, what was your reaction?

Respondent: I saw that it was a good method because you cannot struggle to go to someone to inject you.

Interviewer: mmmm (showing that one is following the conversation)

Respondent: Yes.

Interviewer: Alright, what appealed to you about the ability to self-inject?

Respondent: What appealed to me was that no one can know that you have self-injected or that someone has injected you

Interviewer: oho

Respondent: It’s a good method.

TIME INTO THE INTERVIEW: 10Mins

Interviewer: Alright, What concerns did you have if any when you heard that women could self-inject.

Respondent: no I did not have any concerns. Because I saw that this method was good there was no need to go to anyone else or to the hospital to get the injection.

Interviewer: alright

Respondent: yes

Interviewer: ah What were the most important factors motivating you to try DMPA SC?

Respondent: I saw that as a girl you can be able to do school and be in a relationship and take family planning to self-inject

Title: MZs_IM_6.docx

Descriptor Info:	District: Mzimba South	Target Population: TP2 - DMPA-IM/DMPA-SC PI clients	Respondent type: DMPA-IM client	Aware of SI: Yes - had heard of SI before	Coding phase: 1. Single coded	Client marital status: Married	Client age group: Under 20 (adolescent)	Time since trained: N/A - client

Codes Applied:	C1.1.1 - Positive client attitudes to SI	C2.1.2 - Reduced trips to facility

Interviewer: Did you ever use this method of self-inject?

Respondent: Eeeh (agreeing) we self-injected right there at the facility. they firstly taught us how to do it then gave us to self-inject

Interviewer: After you self-injected, how did you feel about this method?

Respondent: I did not experience pain since the needle is smaller compared to the one they use for intramuscular Depo [giggles]

Interviewer: Mmmhu (agreeing) as you have said that now you are using provider injected Depo. What would have made you go back to self-inject method

Respondent: Because of its benefits of saving costs and reduced number of visits since I would be required to come once a year

Interviewer: Any additions?

Respondent: No

Title: MZs_IM_6.docx

Descriptor Info:	District: Mzimba South	Target Population: TP2 - DMPA-IM/DMPA-SC PI clients	Respondent type: DMPA-IM client	Aware of SI: Yes - had heard of SI before	Coding phase: 1. Single coded	Client marital status: Married	Client age group: Under 20 (adolescent)	Time since trained: N/A - client

Codes Applied:	C1.1.1 - Positive client attitudes to SI	C2.1.2 - Reduced trips to facility	C3.3.1 - Recos for what should be covered in counselling / training

Interviewer: [interrupted by a visitor] as you have watched the video. Do you think other women who gets provider Depo would opt for self-inject after watching this video?

Respondent: Yes

Interviewer: Why?

Respondent: Because it is well explained

Interviewer: What else?

Respondent: They will be attracted by the fact that they will reduce the number of visits to the facility

Interviewer: Mmmh [thinking out loud] what else?

Respondent: That’s all

Interviewer: What do you think is the most crucial information for women who take this method of self-injection

Respondent: They have to know the procedure for using it because if they don’t have adequate information, they might end up doing it wrongly at home but if they are given all the information, they can use it easily

Interviewer: What about for adolescents?

Respondent: The benefit to them is that they will protect themselves from getting pregnant

Title: KK_HSA_4.docx

Descriptor Info:	District: Nkhotakota	Target Population: TP1 - Providers/HSAs	Respondent type: HSA	Aware of SI: Yes - had heard of SI before	Coding phase: 1. Single coded	Client marital status: N/A - provider	Client age group: N/A - provider	Time since trained: A year or more ago

Codes Applied:	C2.1.2 - Reduced trips to facility

RESPONDENT: Ok, if we consider injectables , Sayana is the most common one among them all, because since we started providing it to clients most women are more interested in it. This is so because whenever we give them this method,

INTERVIEWER: Yes

RESPONDENT: We ensure that we give them some more units to use at home. So most people like this method because it has reduced the visits one makes to a facility to meet the provider.

INTERVIEWER: Alright

Title: KK_HSA_4.docx

Descriptor Info:	District: Nkhotakota	Target Population: TP1 - Providers/HSAs	Respondent type: HSA	Aware of SI: Yes - had heard of SI before	Coding phase: 1. Single coded	Client marital status: N/A - provider	Client age group: N/A - provider	Time since trained: A year or more ago

Codes Applied:	P1.1.1 - Positive provider attitudes towards SI	P3.1.1 - Reduced provider workload	C2.1.2 - Reduced trips to facility	P2.1.2 - Sufficient original provider training

INTERVIEWER: Alright thank you so much. To continue with our conversation, I would like to ask you some questions about your experience with being trained in DMPA-SC. The first question on this topic is; may you please tell me a bit about the training you received in providing DMPA-SC? Overall what did you think of the training?

RESPONDENT: From the time we received the training in DMPA-SC for self-injection

INTERVIEWER: Yes

RESPONDENT: In would say that after the training I felt it was so good that I did undergone such training because as an HSA, it meant that I have added knowledge in another type family planning method. Secondly, I also considered the distance that most clients travel to come and meet me just to take contraceptives. Furthermore, the fact that the method do require clients to take some more units home to use afterwards, I felt this will do me good since my work load will reduce because I won’t be meeting clients regularly as before.

Title: KK_HSA_4.docx

Descriptor Info:	District: Nkhotakota	Target Population: TP1 - Providers/HSAs	Respondent type: HSA	Aware of SI: Yes - had heard of SI before	Coding phase: 1. Single coded	Client marital status: N/A - provider	Client age group: N/A - provider	Time since trained: A year or more ago

Codes Applied:	P1.1.1 - Positive provider attitudes towards SI	C1.1.1 - Positive client attitudes to SI	C8.3 - Discretion of injectables/SI	C5.1.4 - Timely injection due to not having to rely on external factors	C2.1.2 - Reduced trips to facility

INTERVIEWER: Ok, would you say the trend in DMPA-S uptake for self-injection has changed over time? What factors do you think have influenced this trend? From the time you introduced the method to now and please explain how the change has been.

RESPONDENT: I will explain more on a better change.

INTERVIEWER: Ok fine

RESPONDENT: Things have really changed for better

INTERVIEWER: Alright

RESPONDENT: As providers as well as clients we can see that there is a great change from the time the method was introduced compared to now in that; for the injection that was being administered by the provider every three months

INTERVIEWER: Yes

RESPONDENT: but for the new method After initial injection, the client is given extra units to use at home. This has led to more women getting interested in this method since it reduces visits that a client is supposed to make for her to go access the contraceptive.

INTERVIEWER: Alright

RESPONDENT: As such she is able to do other chores at home instead of spending much of her time at a facility. The other reason could be that the method has privacy, in that the client may use the method for a period of a year without visiting the facility ad people won’t notice that she is on taking any family planning method, because she will not be visiting the HSA.

INTERVIEWER: Yes, true

RESPONDENT: Yes

Title: ZA_IM_4.docx

Descriptor Info:	District: Zomba	Target Population: TP2 - DMPA-IM/DMPA-SC PI clients	Respondent type: DMPA-IM client	Aware of SI: Yes - had heard of SI before	Coding phase: 1. Single coded	Client marital status: Married	Client age group: Under 20 (adolescent)	Time since trained: N/A - client

Codes Applied:	C1.1.1 - Positive client attitudes to SI	C2.1.2 - Reduced trips to facility

Interviewer: Alright uhhmm (Baby coughs) How did you feel about this when you heard that (baby calls the mother) women inject themselves, how did you feel about this?

Respondent: It amazed me

Interviewer: Mhh

Respondent: I realised that I will spend lesser time coming to the hospital

Interviewer: Mmh

Respondent: Mhh

Title: ZA_IM_4.docx

Descriptor Info:	District: Zomba	Target Population: TP2 - DMPA-IM/DMPA-SC PI clients	Respondent type: DMPA-IM client	Aware of SI: Yes - had heard of SI before	Coding phase: 1. Single coded	Client marital status: Married	Client age group: Under 20 (adolescent)	Time since trained: N/A - client

Codes Applied:	C1.1.1 - Positive client attitudes to SI	C5.1.4 - Timely injection due to not having to rely on external factors	C2.1.2 - Reduced trips to facility

Interviewer: So, when you heard about this (baby calls the mother) injection method of family planning do you see women using this injection method of family planning from the hospitals or heath advisers being interested to use this method? (baby calls the mother)

Respondent: Mmmh they would be interested (baby calls the mother) because they be will managing their time instead of coming here, they will be able to do other things. (Baby cries)

Interviewer: Yes, that’s true thank you (baby cries) do think women will have different interest in this self-injection method?

Respondent: No they wouldn’t lose interest

Interviewer: Why wouldn’t they lose interest?

Respondent: Because they will not get lazy, they will simply inject themselves while others get lazy to go to the hospital that is why others end up getting pregnant

Title: MZs_HSA_4.docx

Descriptor Info:	District: Mzimba South	Target Population: TP1 - Providers/HSAs	Respondent type: HSA	Aware of SI: Yes - had heard of SI before	Coding phase: 1. Single coded	Client marital status: N/A - provider	Client age group: N/A - provider	Time since trained:

Codes Applied:	C2.1.2 - Reduced trips to facility	C1.1.1 - Positive client attitudes to SI	C5.1.4 - Timely injection due to not having to rely on external factors

Interviewer: do you encourage the clients to use the injectable methods?

Respondent: yes.

Interviewer: why?

Respondent: by using this method, the clients concentrate on other business rather than always thinking about when to go to the facility to self-inject. in using the self-injection they are not required to travel to the hospital but rather, they self-inject while at home in their bedrooms. this helps them to concentrate on development projects at home.

Interviewer: Mmmmmh.

Respondent: yes.

Title: MZs_HSA_4.docx

Descriptor Info:	District: Mzimba South	Target Population: TP1 - Providers/HSAs	Respondent type: HSA	Aware of SI: Yes - had heard of SI before	Coding phase: 1. Single coded	Client marital status: N/A - provider	Client age group: N/A - provider	Time since trained:

Codes Applied:	P3.3 - Counselling/ training approach, general	C2.1.2 - Reduced trips to facility	C5.1.1 - Continuation of SI (including reasons)

Interviewer: do you have any difficulties in differentiating self-injection form other injectables?

Respondent: most people prefer self-injection because they do not like to travel to the facility. There was one client who said that by using Depo she hard an abnormal menstruation while Sayana does not.

Interviewer: how did you interact with this client?

Respondent: I explained to the client that the two methods are similar. I asked her if she went to the hospital after experiencing such condition? She also explained that she believed that it was the Depo method which made her menstruate abnormally while Sayana did not. So I advised her to go to the hospital and seek clarification of whether this was caused by the method or not.

Title: ZA_FAC_2.docx

Descriptor Info:	District: Zomba	Target Population: TP1 - Providers/HSAs	Respondent type: Facility-based provider	Aware of SI: Yes - had heard of SI before	Coding phase: 3. Triple coded	Client marital status: N/A - provider	Client age group: N/A - provider	Time since trained: Less than a year ago

Codes Applied:	P2.3 - Provider training content	C2.1.2 - Reduced trips to facility	C1.1.1 - Positive client attitudes to SI

Interviewer: So, still on the training; what are the good aspects of the training? What is it that you really liked about the training? What is it that appealed to you about the training?

Time stamp: 10:04

Respondent: Okay. Its good because as I already said, when I educate my clients, a lot of them are willing to inject themselves at home. Because you know with this issue of COVID 19. So I think it’s good with Sayana press because when a client come, you can provide four doses of Sayana meaning the client will come again the other year which is good in the context of COVID 19.

Interviewer: So we’re talking about the interview now. What was the good part about the training you had about Sayana?

Respondent: The good part?

Interviewer: Mmh.

Respondent: The good part was I gained the knowledge about how to apply the Sayana press. Yeah.

Title: ZA_FAC_2.docx

Descriptor Info:	District: Zomba	Target Population: TP1 - Providers/HSAs	Respondent type: Facility-based provider	Aware of SI: Yes - had heard of SI before	Coding phase: 3. Triple coded	Client marital status: N/A - provider	Client age group: N/A - provider	Time since trained: Less than a year ago

Codes Applied:	P88 - Provider great quotes	C2.1.2 - Reduced trips to facility	P1.1.1 - Positive provider attitudes towards SI

Interviewer: Thank you very much. Is there any reason why you have changed or unchanged views towards the method?

Respondent: The reason for the change is that before the Sayana method was introduced, women after getting the depo method would go home and come back after 3months but with the introduction of the Sayana method once a woman gets it, she will have to visit the hospital again the following year which is also like a long-term method since the woman stays at home and comes back the other year.

Title: ZA_FAC_2.docx

Descriptor Info:	District: Zomba	Target Population: TP1 - Providers/HSAs	Respondent type: Facility-based provider	Aware of SI: Yes - had heard of SI before	Coding phase: 3. Triple coded	Client marital status: N/A - provider	Client age group: N/A - provider	Time since trained: Less than a year ago

Codes Applied:	C1.1.1 - Positive client attitudes to SI	C2.1.2 - Reduced trips to facility	P4.1.1 - Follow up materials for client to take home

Interviewer: According to what you’ve seen, what do women say about self-injection method?

Respondent: Many women are very happy because some come from far, and they see that when we teach them and they administer the self-injection on their own, they have enough time to do their household chores and thus they won’t be visiting the hospital very often. As such most women are happy because they can rest after getting the drugs and they can be at home and administer the drug after 3 months because we also give out calendars so that they should know the date when they should inject themselves.

Title: ZA_FAC_2.docx

Descriptor Info:	District: Zomba	Target Population: TP1 - Providers/HSAs	Respondent type: Facility-based provider	Aware of SI: Yes - had heard of SI before	Coding phase: 3. Triple coded	Client marital status: N/A - provider	Client age group: N/A - provider	Time since trained: Less than a year ago

Codes Applied:	C1.1.1 - Positive client attitudes to SI	C2.1.2 - Reduced trips to facility	C5.1.4 - Timely injection due to not having to rely on external factors

Interviewer: Okay, thank you. What do you think attracts women to choose the self-injection method? What attracts women?

Respondent: The issue is that the women do not want to be frequently visiting the hospital. They see that if they get it and apply it at home, then they are able to do their household chores and carry out their other plans.

Title: ZA_SCSI_5.docx

Descriptor Info:	District: Zomba	Target Population: TP3 - DMPA-SC SI clients	Respondent type: DMPA-SC SI client	Aware of SI: Yes - had heard of SI before	Coding phase: 3. Triple coded	Client marital status: Married	Client age group: 30-39 years	Time since trained: N/A - client

Codes Applied:	C2.1.2 - Reduced trips to facility	C1.1.1 - Positive client attitudes to SI

Interviewer: Since the coronavirus disease started, have you seen any difficulties or no difficulties in accessing contraceptive methods?

Respondent: Ah I don’t see any difficulties.

Interviewer: Mmm (showing that one is following the conversation)

Respondent: When we meet here, maybe I have not worn a mask, maybe you have forgotten the mask

Interviewer: Mmm (showing that one is following the conversation)

Respondent: And other women have not worn a mask, so I see that as a problem.

Interviewer: Alright.

Respondent: But when we take Sayana we are just at home, and we don’t go out unnecessarily

Title: ZA_SCSI_5.docx

Descriptor Info:	District: Zomba	Target Population: TP3 - DMPA-SC SI clients	Respondent type: DMPA-SC SI client	Aware of SI: Yes - had heard of SI before	Coding phase: 3. Triple coded	Client marital status: Married	Client age group: 30-39 years	Time since trained: N/A - client

Codes Applied:	C88 - Client great quotes	C2.1.2 - Reduced trips to facility	C5.1.4 - Timely injection due to not having to rely on external factors

Interviewer: When you first heard about that, how did you feel?

Respondent: When I heard about this, I saw that it was good.

Interviewer: Mmm (showing that one is following the conversation)

Respondent: Because we will just be at home

Interviewer: Mmm (showing that one is following the conversation)

Respondent: That means we will just self-inject and do our chores. We will not struggle to travel long distances to go to the facility.

Interviewer: Mmm (showing that one is following the conversation)

Respondent: So we saw that this method is good

Title: ZA_SCSI_5.docx

Descriptor Info:	District: Zomba	Target Population: TP3 - DMPA-SC SI clients	Respondent type: DMPA-SC SI client	Aware of SI: Yes - had heard of SI before	Coding phase: 3. Triple coded	Client marital status: Married	Client age group: 30-39 years	Time since trained: N/A - client

Codes Applied:	C1.1.1 - Positive client attitudes to SI	C2.1.2 - Reduced trips to facility

Interviewer: Alright what appealed to you a lot about the possibility of women to self-inject?

Respondent: What appealed to me was as I have said it’s a good method.

Interviewer: Mmm (showing that one is following the conversation)

Respondent: Even better than all these that I been using in the past.

Interviewer: Mmm (showing that one is following the conversation)

Respondent: Because this method is the best and I cannot take another method.

Interviewer: Yes

Respondent: I will be using Sayana.

Interviewer: Alright.

Respondent: The advantage is the one that I have explained that you will just be at home.

Title: ZA_SCSI_5.docx

Descriptor Info:	District: Zomba	Target Population: TP3 - DMPA-SC SI clients	Respondent type: DMPA-SC SI client	Aware of SI: Yes - had heard of SI before	Coding phase: 3. Triple coded	Client marital status: Married	Client age group: 30-39 years	Time since trained: N/A - client

Codes Applied:	C8.1 - Male involvement, general	C5.1.4 - Timely injection due to not having to rely on external factors	C2.1.2 - Reduced trips to facility

Interviewer: Alright, is there anything that motivated you that you should try this method, that for me I have chosen this method of Sayana?

Respondent: Yes, there is something that motivated me.

Interviewer: Mmm (showing that one is following the conversation)

Respondent: The motivation was that when I came here, I told my husband

Interviewer: Mmm (showing that one is following the conversation)

Respondent: When I explained to him, he said that I should just get that method

Interviewer: Mmm (showing that one is following the conversation)

Respondent: It will be good if you take that method.

Interviewer: Alright.

Respondent: He also agreed with me that other chores can be done without having to travel a long distance to get to the facility.

Interviewer: Alright.

Respondent: We encouraged each other with my husband. My husband also motivated me.

Interviewer: Alright.

Respondent: Sure

Interviewer: Have your thoughts about Sayana changed overtime?

Respondent: No they have not changed.

Title: ZA_SCSI_5.docx

Descriptor Info:	District: Zomba	Target Population: TP3 - DMPA-SC SI clients	Respondent type: DMPA-SC SI client	Aware of SI: Yes - had heard of SI before	Coding phase: 3. Triple coded	Client marital status: Married	Client age group: 30-39 years	Time since trained: N/A - client

Codes Applied:	C3.3.1 - Recos for what should be covered in counselling / training	C2.1.2 - Reduced trips to facility

Interviewer: What other thigs do you think women need to know about self-injection?

Respondent: Women need to know that we are encouraging them that there is a new injectable Sayana which you can self-inject.

Interviewer: Mmm (showing that one is following the conversation)

Respondent: So you can just go and find out more about it at the hospital.

Interviewer: Mmm (showing that one is following the conversation)

Respondent: If you like it, you can access it or you can choose not to.

Interviewer: Mmm (showing that one is following the conversation)

Respondent: So the women when they come here, we see that they access it

Interviewer: Alright.

Respondent: They say that its good. It does not keep you busy

Interviewer: Mmm (showing that one is following the conversation)

Respondent: Yes so many women are accessing it

Title: MZs_IM_7.docx

Descriptor Info:	District: Mzimba South	Target Population: TP2 - DMPA-IM/DMPA-SC PI clients	Respondent type: DMPA-IM client	Aware of SI: Yes - had heard of SI before	Coding phase: 1. Single coded	Client marital status: Divorced/ separated/ widowed	Client age group: Under 20 (adolescent)	Time since trained: N/A - client

Codes Applied:	C5.1.4 - Timely injection due to not having to rely on external factors	C2.1.2 - Reduced trips to facility

Interviewer: may you tell me what I said?

Respondent: I would tell them that they after using this method it’s not difficult to get pregnant again but also, they give you to use home for 9 months

Title: MZs_HSA_3.docx

Descriptor Info:	District: Mzimba South	Target Population: TP1 - Providers/HSAs	Respondent type: HSA	Aware of SI: Yes - had heard of SI before	Coding phase: 3. Triple coded	Client marital status: N/A - provider	Client age group: N/A - provider	Time since trained: Less than a year ago

Codes Applied:	C8.1 - Male involvement, general	C2.1.2 - Reduced trips to facility	A6 - Adol Perspectives/preferences for DMPA-SC SI	C4 - Storage of unused units

Interviewer: what about specifically for adolescent?

Respondent: mmmmmh those adolescent…. most of them I should they say no just give me because I have nowhere to keep this one…safety… safety at home, because they know where we stay we are 3 so for me to keep this one it will be difficult so therefore I should be coming and you should give me as a provider, yah that is what most of them they say. because even these big women the old ones when you say there is Sayana here now they say aaah but my husband will see this one I don’t have… may be I have got so many children I.. I will not be able to keep this one safe then I I choose instead of getting this one at home I will be coming here. You say no this is good there you reduce some of the problems which you are facing up to now you are still here but if it was at your house where you keep it could have just taken some minutes you are through with it so they say nowhere to keep is a problem. Yah because others you know they are free people others are accepted by the husbands, others they are not accepted by the husbands as we are saying maybe they have got zibwenzi [meaning extra marital affairs/partners] so to keep that one in the house they say aah its difficult some of them but most of them they like because it reduces the period of coming here.

Title: MZs_HSA_3.docx

Descriptor Info:	District: Mzimba South	Target Population: TP1 - Providers/HSAs	Respondent type: HSA	Aware of SI: Yes - had heard of SI before	Coding phase: 3. Triple coded	Client marital status: N/A - provider	Client age group: N/A - provider	Time since trained: Less than a year ago

Codes Applied:	C2.1.2 - Reduced trips to facility	C3.2.2 - Lack of inclination or confidence to inject, even after training	C8.1 - Male involvement, general	C4 - Storage of unused units

Q.17 Interviewer: in your view, what are the main factors that would support a client to take up the self- injection option with DMPA-SC?

Respondent: distance where she comes from, if it is very far, she says I have to get Sayana so that I should come I should not be coming here frequently to take almost a year before I come here. The other one could be I heard there is a new vaccine which whereby people inject themselves so I would like to be one of those so today I want to get that Sayana we say here it is we will train you after being trained we see what you do then later on you will be given to carry at home. So she says ok it’s easy for me to stay at home and come back at a later date instead of coming here every 3 months. But for the barriers, others would say that one I will have problems where to keep because my husband will either throw away or my children will may take it or I can’t do… I can’t do. You train her you say now I have finished have you understood she says yes do it then she says I can’t make it myself just give me so she says give me I have understood but I cannot take it home you have to give me yah.

Title: MZs_HSA_3.docx

Descriptor Info:	District: Mzimba South	Target Population: TP1 - Providers/HSAs	Respondent type: HSA	Aware of SI: Yes - had heard of SI before	Coding phase: 3. Triple coded	Client marital status: N/A - provider	Client age group: N/A - provider	Time since trained: Less than a year ago

Codes Applied:	P3.4 - Time to counsel/train, general	C1.1.3 - Awareness of DMPA-SC SI	C2.1.2 - Reduced trips to facility

Interviewer: Does the length of training influence whether some women choose to take up self-injection?

Respondent: yes. Yah! Because the they might be 2 so seeing that the other one is getting Sayana the other one got depo. So when she says what what about this one so we say this is Sayana has got these these advantages it’s like this it’s done like these somebody just gets it at home you will inject it at home so those who got depo somewhere they say aaah no therefore am am also able I can go and get.. [interrupted by noise the recording was paused] ndipitilize? ….where I was, I got Depo but today am hearing about Sayana the advantages which are there to I may stay home for almost a year without coming here now let me try Sayana.

Title: MZs_SCSI_2.docx

Descriptor Info:	District: Mzimba South	Target Population: TP3 - DMPA-SC SI clients	Respondent type: DMPA-SC SI client	Aware of SI: Yes - had heard of SI before	Coding phase: 3. Triple coded	Client marital status: Married	Client age group: Under 20 (adolescent)	Time since trained: N/A - client

Codes Applied:	C5.1.4 - Timely injection due to not having to rely on external factors	C2.1.2 - Reduced trips to facility

Interviewer: What appealed to you about the ability to self-inject?

Respondent: You have the freedom to do it at your own convenient time at home and also the provider did not spend a lot of time because they are just the same as Depo.

Title: MZs_SCSI_2.docx

Descriptor Info:	District: Mzimba South	Target Population: TP3 - DMPA-SC SI clients	Respondent type: DMPA-SC SI client	Aware of SI: Yes - had heard of SI before	Coding phase: 3. Triple coded	Client marital status: Married	Client age group: Under 20 (adolescent)	Time since trained: N/A - client

Codes Applied:	C1.1.1 - Positive client attitudes to SI	C2.1.2 - Reduced trips to facility

Q15. Interviewer: Would you recommend DMPA-SC for self-injection to other women in the community?

Respondent: Yes.

Interviewer: Why would you recommend them?

Respondent: So that they also save time of not going to the facility every 3 months for providers injection.

Interviewer: Do you think some women in your community may be more interested in DMPA-SC (Sayana press) than others?

Respondent: Yes, so that they should minimize their visiting to the facility.

Title: KK_HSA_2.docx

Descriptor Info:	District: Nkhotakota	Target Population: TP1 - Providers/HSAs	Respondent type: HSA	Aware of SI: Yes - had heard of SI before	Coding phase: 2. Double coded	Client marital status: N/A - provider	Client age group: N/A - provider	Time since trained: A year or more ago

Codes Applied:	C1.1.1 - Positive client attitudes to SI	C2.1.2 - Reduced trips to facility	C0.2 - Community and client perspectives on FP - general

RESPONDENT: aaamhhh Depo as one of the common family planning methods requires us to administer to women after every three months whilst Sayana we just advice women to take it home and do it on their own in their homes. As such they feel Sayana is better than Depo.

INTERVIEWER: Mhhhh but which ones are the most common ones, is it Depo or…?

RESPONDENT: Depo is very common and Sayana

INTERVIEWER: What about the one which is most commonly used by women

RESPONDENT: Sayana

INTERVIEWER: So its Sayana which is commonly used by women?

RESPONDENT: Yes most women are using this one

INTERVIEWER: Okay, so why do you think that Sayana is mostly used by women?

RESPONDENT: Mhhh mostly it's because they administer themselves at home

INTERVIEWER: Mhh okay

RESPONDENT: They don't walk long distances to go and get Sayana

INTERVIEWER: Eheeee

RESPONDENT: They administer it themselves in their homes

INTERVIEWER: Ok fine

Title: KK_HSA_2.docx

Descriptor Info:	District: Nkhotakota	Target Population: TP1 - Providers/HSAs	Respondent type: HSA	Aware of SI: Yes - had heard of SI before	Coding phase: 2. Double coded	Client marital status: N/A - provider	Client age group: N/A - provider	Time since trained: A year or more ago

Codes Applied:	P1.1.1 - Positive provider attitudes towards SI	C2.1.2 - Reduced trips to facility

INTERVIEWER: Mhh so would you say that the trend in DMPA-SC uptake of injection has changed over time. If so what factors do you think have influenced this trend.

RESPONDENT: Yes there has been a change for instance if one takes Depo she would be visiting the facility every three months whilst DMPA _Sc its only once a year. Hence she would prefer Sayana.

Title: KK_IM_7.docx

Descriptor Info:	District: Nkhotakota	Target Population: TP2 - DMPA-IM/DMPA-SC PI clients	Respondent type: DMPA-IM client	Aware of SI: Yes - had heard of SI before	Coding phase: 1. Single coded	Client marital status: Married	Client age group: 30-39 years	Time since trained: N/A - client

Codes Applied:	C0.4 - Access to or process of obtaining FP services	C2.1.2 - Reduced trips to facility

INTERVIEWER: Not only complaints. Anything you heard people talking about Sayana. What were they saying?

RESPONDENT: Aaaah, they were saying that Sayana is good.

INTERVIEWER: Okay. Why were they saying Sayana is good?

RESPONDENT: Because you have few visits to the hospital.

INTERVIEWER: mmmhu.

RESPONDENT: Yea.

Title: KK_IM_7.docx

Descriptor Info:	District: Nkhotakota	Target Population: TP2 - DMPA-IM/DMPA-SC PI clients	Respondent type: DMPA-IM client	Aware of SI: Yes - had heard of SI before	Coding phase: 1. Single coded	Client marital status: Married	Client age group: 30-39 years	Time since trained: N/A - client

Codes Applied:	C2.1.2 - Reduced trips to facility

INTERVIEWER: It’s not a test that you will pass or fail, I just want to hear your reactions.

RESPONDENT: Yes.

INTERVIEWER: mmmh.

[video from 33: 11-44:21]

INTERVIEWER: It’s finished.

RESPONDENT: It’s good.

INTERVIEWER: good right? okay

RESPONDENT: You will rest. Every three months you have to come to the hospital. I have seen that it is good because you will concentrate on farming and self-inject every three months. But currently, you have to hurry every three months to avoid being sent back (laughing).

INTERVIEWER: okay.

RESPONDENT: sure

Title: KK_IM_7.docx

Descriptor Info:	District: Nkhotakota	Target Population: TP2 - DMPA-IM/DMPA-SC PI clients	Respondent type: DMPA-IM client	Aware of SI: Yes - had heard of SI before	Coding phase: 1. Single coded	Client marital status: Married	Client age group: 30-39 years	Time since trained: N/A - client

Codes Applied:	C2.1.2 - Reduced trips to facility

INTERVIEWER: Okay. Another important thing?

RESPONDENT: The other important thing is that you will have few visits to the hospital, mmmmmh.

Title: ZA_IM_5.docx

Descriptor Info:	District: Zomba	Target Population: TP2 - DMPA-IM/DMPA-SC PI clients	Respondent type: DMPA-IM client	Aware of SI: Yes - had heard of SI before	Coding phase: 2. Double coded	Client marital status: Married	Client age group: 30-39 years	Time since trained: N/A - client

Codes Applied:	C2.1.2 - Reduced trips to facility	C3.3.1 - Recos for what should be covered in counselling / training

Interviewer: Alright, what do you think are the most important things in the video?

Respondent: The most important things in the video like they have said that it is reducing

Interviewer: Mmmm (showing that one is following the conversation)

Respondent: Going to the hospital more often because when you get the injection then they will give you three units, it will last you for 9 months.

Interviewer: Mmmm (showing that one is following the conversation)

Respondent: 9 months yeah

Interviewer: Mmmm (showing that one is following the conversation)

Respondent: Yes, which is like a year, just remaining with two months to end the year.

Interviewer: Mmmm (showing that one is following the conversation)

Respondent: Just remaining with a few months in the future.

Interviewer: Yah yah

Respondent: Yes.

Interviewer: Alright.

Respondent: So in a year you can just be coming to the hospital only two times.

Interviewer: Mmmm (showing that one is following the conversation)

Respondent: Yeah

Title: ZA_IM_5.docx

Descriptor Info:	District: Zomba	Target Population: TP2 - DMPA-IM/DMPA-SC PI clients	Respondent type: DMPA-IM client	Aware of SI: Yes - had heard of SI before	Coding phase: 2. Double coded	Client marital status: Married	Client age group: 30-39 years	Time since trained: N/A - client

Codes Applied:	C2.1.2 - Reduced trips to facility

Respondent: But we can, we can encourage them for example the mobility.

Interviewer: Mmmm (showing that one is following the conversation)

Respondent: Since we will stay after months they will come to the facility

Title: ZA_IM_5.docx

Descriptor Info:	District: Zomba	Target Population: TP2 - DMPA-IM/DMPA-SC PI clients	Respondent type: DMPA-IM client	Aware of SI: Yes - had heard of SI before	Coding phase: 2. Double coded	Client marital status: Married	Client age group: 30-39 years	Time since trained: N/A - client

Codes Applied:	C2.1.2 - Reduced trips to facility

Respondent: Maybe the difference, the liquid inside is the same but the difference is the times you go to the hospital, when we get the units, it means we have months, the months are the same with when they inject us at the hospital.

Interviewer: Yah yah yah

Respondent: But it reduces the times you travel, the way we have taken the two units, then it will last us for six months.

Interviewer: Alright.

Respondent: Yes.

Title: ZA_HSA_3.docx

Descriptor Info:	District: Zomba	Target Population: TP1 - Providers/HSAs	Respondent type: HSA	Aware of SI: Yes - had heard of SI before	Coding phase: 1. Single coded	Client marital status: N/A - provider	Client age group: N/A - provider	Time since trained: A year or more ago

Codes Applied:	C2.1.2 - Reduced trips to facility	P3.1.1 - Reduced provider workload

Interviewer: Alright, what are your thoughts about the training you got?

Respondent: From what I learnt, I believe Sayana press reduced the number of people in hospitals since people inject themselves at home and sometimes we give them three syringes to use themselves at home and that reduced queues in the hospital and also enhanced safety as people do not meet this prevent getting the corona virus.

Title: ZA_HSA_3.docx

Descriptor Info:	District: Zomba	Target Population: TP1 - Providers/HSAs	Respondent type: HSA	Aware of SI: Yes - had heard of SI before	Coding phase: 1. Single coded	Client marital status: N/A - provider	Client age group: N/A - provider	Time since trained: A year or more ago

Codes Applied:	P3.3 - Counselling/ training approach, general	C2.1.2 - Reduced trips to facility	C5.1.4 - Timely injection due to not having to rely on external factors

Interviewer: In what ways do you encourage?

Respondent: We encourage them by teaching them that they can inject themselves at their free time at home and the clients also get excited because it is simpler for them than setting out a day to come to the hospital which is tiresome and takes the whole morning up to noon hence they can do other things instead of doing one thing.

Title: ZA_HSA_3.docx

Descriptor Info:	District: Zomba	Target Population: TP1 - Providers/HSAs	Respondent type: HSA	Aware of SI: Yes - had heard of SI before	Coding phase: 1. Single coded	Client marital status: N/A - provider	Client age group: N/A - provider	Time since trained: A year or more ago

Codes Applied:	C2.1.2 - Reduced trips to facility

Interviewer: For what reasons do you think women are encouraged to use this method?

Respondent: Just like I have already said, it is an easy method to use and does not require going to the hospital and the only thing required is to find time to inject themselves

Title: MZs_SCSI_5.docx

Descriptor Info:	District: Mzimba South	Target Population: TP3 - DMPA-SC SI clients	Respondent type: DMPA-SC SI client	Aware of SI: Yes - had heard of SI before	Coding phase: 1. Single coded	Client marital status: Married	Client age group: 20-29 years	Time since trained: N/A - client

Codes Applied:	C3.1 - Enablers, general/other	C2.1.2 - Reduced trips to facility

Interviewer: I would like to know if the time you came there were other family planning methods apart from self-inject

Respondent: Yes, there were pills and condoms

Interviewer: But you chose self-inject?

Respondent: Yes

Interviewer: I would like to know why you chose self-inject over other methods of family planning

Respondent: Self-inject reduces number of facility visits. For pills I would forget to them according to prescription. For condoms I wouldn’t force my husband to use them because he denies using them saying they are for the unmarried. So the only option was to go for self-inject

Interviewer: Any other reasons?

Respondent: Pardon?

Interviewer: Was there any factor that led you choose Sayana Press?

Respondent: It was a method I liked since I couldn’t get pills and condoms

Title: MZs_SCSI_5.docx

Descriptor Info:	District: Mzimba South	Target Population: TP3 - DMPA-SC SI clients	Respondent type: DMPA-SC SI client	Aware of SI: Yes - had heard of SI before	Coding phase: 1. Single coded	Client marital status: Married	Client age group: 20-29 years	Time since trained: N/A - client

Codes Applied:	C2.1.2 - Reduced trips to facility

Interviewer: Mmmmh aaah (thinking out loud) what do you think is most crucial in the video that you have just seen

Respondent: On family planning?

Interviewer: Concerning Sayana Press based on the video you have just watched. What is the most crucial part that the nurse explained?

Respondent: The most crucial thing based on the video and not my what I already know?

Interviewer: Based on the video

Respondent: From what I heard and what I think, it relives us from frequent visits to the facility. it is just the same us they taught us the only challenge is that we found few stock of Sayana so we self-injected right here at the facility. but for those who took 5 or 4 doses they had a break from coming to the facility. but it is really helpful in reducing number of visits which helps to concentrate on other things at home.

Title: KK_SCSI_1.docx

Descriptor Info:	District: Nkhotakota	Target Population: TP3 - DMPA-SC SI clients	Respondent type: DMPA-SC SI client	Aware of SI: Yes - had heard of SI before	Coding phase: 3. Triple coded	Client marital status: Never married	Client age group: Under 20 (adolescent)	Time since trained: N/A - client

Codes Applied:	C5.1.4 - Timely injection due to not having to rely on external factors	C2.1.2 - Reduced trips to facility

INTERVIEWER: What appealed to you right, about the ability for a girl or woman right to self-inject at home? What appealed to you?

RESPONDENT: What appealed to me was that when my dose is welling off is maybe you stay far. But when you have it, you just self-inject. Because when you don’t have and you can to inject yourself, and you don’t have the time to go, you can get pregnant.

INTERVIEWER: [disturbed by a phone. Was conducting the interview in a car and the phone was for one of the team members. Did not know that there was a phone. Came up with the car idea after we had failed to secure a place] continue.

RESPONDENT: How did I…what did I do…what appealed to me when I heard about Sayana?

INTERVIEWER: mmmh.

RESPONDENT: I said that it a quick way because when the Sayana is wearing off, you don’t have to go to the hospital because they gave you Sayana. They can give you 4 sachets for self-injecting. When it’s wearing off [when the contraception has worn off at the end of 3 months], you self-inject. Other than you going to the hospital. You can also be lazy and by the time you think of going to the hospital, you might be pregnant. But now, when you see that it’s wearing off, “what should I do, I should inject myself” because you already have some.

INTERVIEWER: mmmhu.

RESPONDENT: mmmh.

Title: KK_SCSI_1.docx

Descriptor Info:	District: Nkhotakota	Target Population: TP3 - DMPA-SC SI clients	Respondent type: DMPA-SC SI client	Aware of SI: Yes - had heard of SI before	Coding phase: 3. Triple coded	Client marital status: Never married	Client age group: Under 20 (adolescent)	Time since trained: N/A - client

Codes Applied:	C2.1.2 - Reduced trips to facility

INTERVIEWER: mmh, why do you like Sayana? Why did you choose Sayana?

RESPONDENT: The other reason is that, may be because it does not stay too long, if we compare with another method that take very long, so I go to the hospital every three months and it’s not long. So I say let me choose this because may be after some time it’s not difficult to quit

Title: KK_SCSI_1.docx

Descriptor Info:	District: Nkhotakota	Target Population: TP3 - DMPA-SC SI clients	Respondent type: DMPA-SC SI client	Aware of SI: Yes - had heard of SI before	Coding phase: 3. Triple coded	Client marital status: Never married	Client age group: Under 20 (adolescent)	Time since trained: N/A - client

Codes Applied:	C1.1.1 - Positive client attitudes to SI	C2.1.2 - Reduced trips to facility

INTERVIEWER: As for you, would you advise and encourage other women in your area to use Sayana?

RESPONDENT: Yes, I would encourage them.

INTERVIEWER: Would you encourage them?

RESPONDENT: mmmmhu.

INTERVIEWER: Why would you encourage them?

RESPONDENT: I would encourage them because this method is important…its good and its mmh mmh [sigh, thinking of what to say] …I would encourage them to inject themselves with this method because it is not going to affect them, as do the contraceptive methods. In addition, maybe you will be going to the hospital after three months but also if they give you the self-injecting one it means you have already collected for the subsequent three-month periods while the other methods require that you go to the hospital each time they way off. Nevertheless, for this, if they gave you enough you will just be injecting yourself, no need to go to the hospital. Therefore, I would encourage them because they will get the self-injecting one and inject themselves at home.

Title: KK_SCSI_1.docx

Descriptor Info:	District: Nkhotakota	Target Population: TP3 - DMPA-SC SI clients	Respondent type: DMPA-SC SI client	Aware of SI: Yes - had heard of SI before	Coding phase: 3. Triple coded	Client marital status: Never married	Client age group: Under 20 (adolescent)	Time since trained: N/A - client

Codes Applied:	C1.1.1 - Positive client attitudes to SI	C2.1.2 - Reduced trips to facility

INTERVIEWER: Mmmmhu, alright, do you think other women from your area have an interest in Sayana?

RESPONDENT: yes…

INTERVIEWER: More than other women? Other women have... In your area, where you come from, right, do you think other women are more interested in Sayana than others are?

RESPONDENT: Yes, I think the interest is there, because it is… there are more women from my area that are using this method.

INTERVIEWER: Is that so?

RESPONDENT: That means the interest is there.

INTERVIEWER: Why do you think these women are more interested?

RESPONDENT: May be because they do not have a long time to go to the hospital because they collect in advance that they can use, they do not really get busy to go to the hospital for an injection or to get another method because they are given in advance.

INTERVIEWER: mmmhu.

RESPONDENT: mmmmh. They inject themselves, so I think the interest is there.

Title: ZA_HSA_6.docx

Descriptor Info:	District: Zomba	Target Population: TP1 - Providers/HSAs	Respondent type: HSA	Aware of SI: Yes - had heard of SI before	Coding phase: 1. Single coded	Client marital status: N/A - provider	Client age group: N/A - provider	Time since trained:

Codes Applied:	C2.1.2 - Reduced trips to facility

Interviewer: Alright, ah why do you think the Depo and Sayana methods are more popular?

Respondent: these methods are more popular because they are easy to use, for Depo it takes almost 90 days which looks like it’s a long time in which the client can be doing other things other than other methods that they are supposed to take daily. In addition to that the Sayana option looks like is easy especially for those who have accepted that they can be able to self-inject.

Interviewer: mmmm (showing that one is following the conversation)

Respondent: they can use these methods for the entire year.

Interviewer: mmmm (showing that one is following the conversation)

Respondent: they will take four doses which they will take once every three months and they will inject themselves, so they will stay for 12 months without coming to the hospital to access family planning.

Title: MZs_FAC_2.docx

Descriptor Info:	District: Mzimba South	Target Population: TP1 - Providers/HSAs	Respondent type: Facility-based provider	Aware of SI: Yes - had heard of SI before	Coding phase: 2. Double coded	Client marital status: N/A - provider	Client age group: N/A - provider	Time since trained:

Codes Applied:	C2.1.2 - Reduced trips to facility	P3.1.3 - IEC/demo materials for counselling/training

Interviewer: Mmmmmhh (following). What were the good parts of the training?

Respondent: During the training, we was given a flip chart and on this flip chart everything was well demonstrated, and because of this flip chart it has been easy to train women. And the needle is very small which makes the client to feel that they can do it and with this Sayana method clients will only be visiting the facility once a year once they have made a decision of self – injecting themselves and even our job has been reduced since we will be meeting them once a year rather than each and every 3 months.

Interviewer: Alright.

Respondent: Mmmmm. (yes)

Title: MZs_FAC_2.docx

Descriptor Info:	District: Mzimba South	Target Population: TP1 - Providers/HSAs	Respondent type: Facility-based provider	Aware of SI: Yes - had heard of SI before	Coding phase: 2. Double coded	Client marital status: N/A - provider	Client age group: N/A - provider	Time since trained:

Codes Applied:	C2.1.2 - Reduced trips to facility

Respondent: Yes. Then we tell them advantages of Sayana compared to Depo that with this one you will be visiting the facility every 3 months while this Sayana will only visit the facility once a year. Or if you choose jakison you will be taking it every day and with these pills you can easily forget. With Sayana method once you have injected or being injected by the provider then thus okay you will just document on the calendar on the next months that you are going to inject.

Title: MZs_FAC_2.docx

Descriptor Info:	District: Mzimba South	Target Population: TP1 - Providers/HSAs	Respondent type: Facility-based provider	Aware of SI: Yes - had heard of SI before	Coding phase: 2. Double coded	Client marital status: N/A - provider	Client age group: N/A - provider	Time since trained:

Codes Applied:	C2.1.2 - Reduced trips to facility

Q17. Interviewer: In your view, what are the main factors that would support a client to take up the self-injection option with DMPA-SC?

Respondent: The method is good since it makes a client not to visit the facility regularly. The only challenge can be to those who cannot inject themselves

Interviewer: Mmmmmhh….? (Really)

Respondent: Yes, thus the problem.

Interviewer: Alright. In your view, what are the main factors that would support a client to take up the self-injection option?

Respondent: Less number of visiting the facility

Title: MZs_HSA_6.docx

Descriptor Info:	District: Mzimba South	Target Population: TP1 - Providers/HSAs	Respondent type: HSA	Aware of SI: Yes - had heard of SI before	Coding phase: 1. Single coded	Client marital status: N/A - provider	Client age group: N/A - provider	Time since trained: A year or more ago

Codes Applied:	P1.1.1 - Positive provider attitudes towards SI	P3.1.1 - Reduced provider workload	C2.1.2 - Reduced trips to facility

Interviewer: When you first heard that Sayana is for self-injection, what was your reaction with your colleagues.

Respondent: it was a good thing because we would provide a woman with Sayana for self-injection then she would not be coming to the provider frequently

Interviewer: what was your reaction before starting providing after hearing the rumors that women will self-inject

Respondent: it was a welcome development

Interviewer: did you talk with your colleagues at this facility upon hearing that women will self-inject at their homes

Respondent: we were wondering what kind of the method it was for a woman to self-inject. So we had agreed to go to the training to learn more about it.

Title: MZs_HSA_6.docx

Descriptor Info:	District: Mzimba South	Target Population: TP1 - Providers/HSAs	Respondent type: HSA	Aware of SI: Yes - had heard of SI before	Coding phase: 1. Single coded	Client marital status: N/A - provider	Client age group: N/A - provider	Time since trained: A year or more ago

Codes Applied:	P3.2.2 - Stockouts/stocks near expiry dates	C2.1.2 - Reduced trips to facility	C5.1.4 - Timely injection due to not having to rely on external factors

Interviewer: with passing of time, did the demand for Sayana increase?

Respondent: the demand kept increasing because of thee counselling we were giving them so many understood and chose Sayana because they would get for a year and stay home which reduces the costs of travelling.

Interviewer: what increased the demand for Sayana?

Respondent: the counselling we gave them

Interviewer: what else

Respondent: women were happy with the idea of self-injecting at their homes. They could come to the provider and found him not their so self-injecting at home solved that challenge. Women are complaining now that it is out of stock

Title: MZs_HSA_6.docx

Descriptor Info:	District: Mzimba South	Target Population: TP1 - Providers/HSAs	Respondent type: HSA	Aware of SI: Yes - had heard of SI before	Coding phase: 1. Single coded	Client marital status: N/A - provider	Client age group: N/A - provider	Time since trained: A year or more ago

Codes Applied:	C1.1.1 - Positive client attitudes to SI	C2.1.2 - Reduced trips to facility

Interviewer: when you first tell a woman that she will self-inject, what are their reaction

Respondent: it’s a welcome development

Interviewer: don’t they have fears

Respondent: no because we start with the counselling, so they understand

Interviewer: what makes a lot of women choose Sayana

Respondent: most of them walk long distance to the community so if you supply for one year, they would be visiting the facility of the provider after a while.

Interviewer: what else

Respondent: they have few side effects

Title: MZs_HSA_6.docx

Descriptor Info:	District: Mzimba South	Target Population: TP1 - Providers/HSAs	Respondent type: HSA	Aware of SI: Yes - had heard of SI before	Coding phase: 1. Single coded	Client marital status: N/A - provider	Client age group: N/A - provider	Time since trained: A year or more ago

Codes Applied:	C2.1.2 - Reduced trips to facility	C5.1.1 - Continuation of SI (including reasons)

Interviewer: what would make other women choose Sayana over the other women

Respondent: those who choose Sayana they reduce number of visits to the facility/provider because most of them travel long distances

Interviewer: what else

Respondent: they see it as a good method because they have not experienced much side effects

Title: ZA_IM_9.docx

Descriptor Info:	District: Zomba	Target Population: TP2 - DMPA-IM/DMPA-SC PI clients	Respondent type: DMPA-IM client	Aware of SI: Yes - had heard of SI before	Coding phase: 1. Single coded	Client marital status: Married	Client age group: 20-29 years	Time since trained: N/A - client

Codes Applied:	C2.1.2 - Reduced trips to facility

Interviewer: So ummh, in addition, this method is found here at the hospital and also with health counsellors. This method is not found with health counsellors who take contraceptives door to door or even Youth centers nor pharmacies. So after hearing this, do you think you can start using this self-injecting injection?

Respondent: Mmh.

Interviewer: What has made you make that decision? For you to start using this method.

Respondent: Because it is good, unlike the other injection, you don’t have to keep on coming to the hospital.

Interviewer: Mmh.

Respondent: Mmh

Interviewer: Alright alright. So after hearing about this self-injecting contraception method, do you think the women using the injection you get at the hospital or given by health counsellors would be interested in this injection?

Respondent: Yes, they would be interested, because they will be able to, ummh, on the day the want the injection, they won’t need to come here at the hospital.

Interviewer: Mmh. Alright alright, thank you so much. Do you think the level of interest in the self-injecting method would be different among several different women?

Respondent: Yes, it would be different.

Interviewer: In what way would it be different?

Respondent: In the ways that for this one you have to keep on coming, and this one you will self-inject, and you won’t have to be coming over and over.

Interviewer: What would make women be more interested in the self-injecting contraception method?

Respondent: Interests would change because they differ in the journeys you have to make coming here, right?

Interviewer: Mmh.

Respondent: Mmh.

Interviewer: Explain clearly there.

Respondent: They would be different.

Interviewer: Mmh.

Respondent: Because the other one we will be getting for 9 months,

Interviewer: Mmh

Respondent: We will be self-injecting at home.

Interviewer: Mmh.

Respondent: You won’t have to come to the hospital.

Interviewer: Mmh. Alright.

Respondent: And the other you have to come here after 3 months here at the hospital. After the end of 3 months you come here.

Title: ZA_IM_9.docx

Descriptor Info:	District: Zomba	Target Population: TP2 - DMPA-IM/DMPA-SC PI clients	Respondent type: DMPA-IM client	Aware of SI: Yes - had heard of SI before	Coding phase: 1. Single coded	Client marital status: Married	Client age group: 20-29 years	Time since trained: N/A - client

Codes Applied:	C5.1.4 - Timely injection due to not having to rely on external factors	C2.1.2 - Reduced trips to facility

Interviewer: So now what has made you want to start using this method?

Respondent: What has made me is that I will not keep on coming to the hospital usually to get injected, I could be self-injecting at home.

Title: KK_HSA_6.docx

Descriptor Info:	District: Nkhotakota	Target Population: TP1 - Providers/HSAs	Respondent type: HSA	Aware of SI: Yes - had heard of SI before	Coding phase: 1. Single coded	Client marital status: N/A - provider	Client age group: N/A - provider	Time since trained: A year or more ago

Codes Applied:	P3.1.5 - Ease of training clients in SI	C2.1.2 - Reduced trips to facility	C1.1.1 - Positive client attitudes to SI	C8.3 - Discretion of injectables/SI

INTERVIEWER: Why do you think Sayana is the most common among women?

RESPONDENT: Mmmhu, Sayana is the most common because when we counsel the women, they feel that it is an easy method for them. There are also some who are on contraceptives without the knowledge of the husband and they feel that when we train them, it is easy for them to hide the drugs when we give them. But for the provider injectable, there is no privacy. It is also good because when we train them and they understand, sometimes they ask for more doses maybe for 9 months because during the rainy season, they have to cross rivers to get to an HSA. It’s only a handful that are provider injected these days. Most women are self-injecting at home. It is simple than Depo Provera and that is why it is common.

Title: KK_HSA_6.docx

Descriptor Info:	District: Nkhotakota	Target Population: TP1 - Providers/HSAs	Respondent type: HSA	Aware of SI: Yes - had heard of SI before	Coding phase: 1. Single coded	Client marital status: N/A - provider	Client age group: N/A - provider	Time since trained: A year or more ago

Codes Applied:	C2.1.2 - Reduced trips to facility	C1.1.1 - Positive client attitudes to SI

INTERVIEWER: What do you think women find most appealing about DMPA-SC for self-injection?

Time stamp: (40:02)

RESPONDENT: It is because they go home with 2,3 doses. They now have fewer visits to an HSA. And this time of the year, people in the village are farming and they feel that it is good for them to take 2 or 3 doses. When a client comes in October, she will come again maybe next year in April or May. She has Sayana for 6 or 9 months. To me, that client will only come maybe if she has other problems.

INTERVIEWER: mmmhu.

RESPONDENT: yea.

Title: KK_HSA_6.docx

Descriptor Info:	District: Nkhotakota	Target Population: TP1 - Providers/HSAs	Respondent type: HSA	Aware of SI: Yes - had heard of SI before	Coding phase: 1. Single coded	Client marital status: N/A - provider	Client age group: N/A - provider	Time since trained: A year or more ago

Codes Applied:	C2.1.2 - Reduced trips to facility	C1.1.1 - Positive client attitudes to SI

INTERVIEWER: Okay. Now I would like to get your perspectives on why you think clients may or may not choose self-injection option. So in your view, what are the main factors that would support a client take up self-injection option?

RESPONDENT: Most of them choose Sayana for self-injection because they of fewer visits to the hospital. We rely mostly on farming in the village and with the rainy season, which is fast approaching, they stay at home for 2 to 3 months and you see them after the rainy season. Some rivers they flood and stay like that for 2 to 3 weeks waiting for water to go down for people to start crossing. So a lot of them take up this method because they understand, and they take with them doses for a year.

INTERVIEWER: okay.

RESPONDENT: Yea. It is easy for them than the provider injected one. They take up this method because we give them Sayana and tell them to come some months later.

Title: ZA_SCSI_4.docx

Descriptor Info:	District: Zomba	Target Population: TP3 - DMPA-SC SI clients	Respondent type: DMPA-SC SI client	Aware of SI: Yes - had heard of SI before	Coding phase: 2. Double coded	Client marital status: Married	Client age group: 30-39 years	Time since trained: N/A - client

Codes Applied:	C2.1.2 - Reduced trips to facility	C5.1.4 - Timely injection due to not having to rely on external factors

Respondent: when I heard, I was excited even though I had not seen the injectable yet.

Interviewer: mmm (showing that one is following the conversation)

Respondent: because I had seen that I was finding it hard to go to the hospital to access family planning.

Interviewer: mmmm (showing that one is following the conversation)

Respondent: When we heard about it, I knew that I will start using that one, so we will be

Interviewer: Mostly at home

Respondent: Mostly at home and also during this time we will be doing other chores

Interviewer: mmm (showing that one is following the conversation)

Respondent: After that we go and self-inject

Interviewer: mmm (showing that one is following the conversation)

Respondent: And then we continue doing our chores,

Interviewer: Alright.

Respondent: Sure,

Title: ZA_SCSI_4.docx

Descriptor Info:	District: Zomba	Target Population: TP3 - DMPA-SC SI clients	Respondent type: DMPA-SC SI client	Aware of SI: Yes - had heard of SI before	Coding phase: 2. Double coded	Client marital status: Married	Client age group: 30-39 years	Time since trained: N/A - client

Codes Applied:	C1.1.1 - Positive client attitudes to SI	C5.1.4 - Timely injection due to not having to rely on external factors	C2.1.2 - Reduced trips to facility

Interviewer: Alright, ah what factors encouraged you that you should try self-injection contraceptive?

Respondent: What encouraged me was that we could miss dates sometimes…

Interviewer: mmm (showing that one is following the conversation)

Respondent: because of visiting the facility

Interviewer: Yah yah yah

Respondent: So we could miss dates sometimes, so the bodies are different, if you miss a date, one would find themselves pregnant on the day that you are going to get your injection.

Interviewer: Alright.

Respondent: So I thought it wise that I should, I should try and that my time I will be using it for other chores, when the date is due for getting an injection… I will self-inject.

Title: ZA_SCSI_4.docx

Descriptor Info:	District: Zomba	Target Population: TP3 - DMPA-SC SI clients	Respondent type: DMPA-SC SI client	Aware of SI: Yes - had heard of SI before	Coding phase: 2. Double coded	Client marital status: Married	Client age group: 30-39 years	Time since trained: N/A - client

Codes Applied:	C1.1.1 - Positive client attitudes to SI	C5.1.4 - Timely injection due to not having to rely on external factors	C2.1.2 - Reduced trips to facility

Interviewer: Alright, ah now we have reached the end of the questions that I had today, but I just have a few questions. Would you recommend DMPA-SC for self-injection to other women?

Respondent: Yes,

Interviewer: Alright, what is the main reason you would recommend it for them?

Respondent: Because I can recommend to my fellow women…

Interviewer: mmm (showing that one is following the conversation)

Respondent: That they should get the Sayana injection

Interviewer: mmm (showing that one is following the conversation)

Respondent: for self-injection

Interviewer: mmm (showing that one is following the conversation)

Respondent: because when they take this injection, they will be unburdened in many ways

Interviewer: mmm (showing that one is following the conversation)

Respondent: Because a woman is a busy person

Interviewer: mmm (showing that one is following the conversation)

Respondent: She easily forgets.

Interviewer: Yah.

Respondent: When taking this injection they should remember whenever they see the calendar since they will put it somewhere on display

Interviewer: mmm (showing that one is following the conversation)

Respondent: Whenever they see the calendar, they will know the date they need to get the injection. As a consequence they will not miss the injection. But when they do not get this injection, then they will have children without spacing

Interviewer: Alright

Respondent: In the end they will be worried

Interviewer: mmm (showing that one is following the conversation)

Respondent: When they have a contraceptive method they can use

Interviewer: eh. Alright, do you think some women in your community may be interested in DMPA-SC?

Respondent: Yes, they would be interested.

Interviewer: mmm (showing that one is following the conversation)

Respondent: Sure.

Interviewer: Alright, do you have any other thoughts or information you would like to share on the topics we have discussed today?

Respondent: Ah my thoughts are that this method of Sayana

Interviewer: eh

Respondent: It should not be phased out.

Interviewer: mmm (showing that one is following the conversation)

Respondent: It should not be ended, it should continue

Interviewer: mmm (showing that one is following the conversation)

Respondent: If there are plans to phase it out

Interviewer: mmm (showing that one is following the conversation)

Respondent: It should not be phased out; it should still continue because it is helping us in many ways.

Interviewer: mmm (showing that one is following the conversation)

Respondent: Sure.

Title: KK_IM_1.docx

Descriptor Info:	District: Nkhotakota	Target Population: TP2 - DMPA-IM/DMPA-SC PI clients	Respondent type: DMPA-IM client	Aware of SI: No - never heard of SI before	Coding phase: 3. Triple coded	Client marital status: Married	Client age group: Under 20 (adolescent)	Time since trained: N/A - client

Codes Applied:	C1.1.1 - Positive client attitudes to SI	C2.1.2 - Reduced trips to facility

INTERVIEWER: So my question is after hearing my explanation on the new Depo which is called Sayana

RESPONDENT: Yes

INTERVEIWER: It is different since a woman does self-injection. So after hearing about this do you think women in your area would be interested in this method?

RESPONDENT: Yes, they may be interested

INTERVIEWER: They may be interested?

RESPONDENT : Yes

INTERVIEWER: Do you think some types of women may be more interested than others? Why? Interested in the self-injection option. You said women may be interested to take this new method not so?

REPONDENT : Yes

INTERVIEWER: would the interest differ, for instance we have explained to a number of women, would some be interested and some would not be interested. If the interest may differ, why do you think so?

RESPONDENT: Because the method is different in that this new one you just go to for collection once a year, whilst the one that is administered by the provider you go regularly.

INTERVIEWER: Yes, we are indeed discussing about this new method that women come once in a year at the facility to collect

RESPONDENT: Yes

Title: KK_IM_1.docx

Descriptor Info:	District: Nkhotakota	Target Population: TP2 - DMPA-IM/DMPA-SC PI clients	Respondent type: DMPA-IM client	Aware of SI: No - never heard of SI before	Coding phase: 3. Triple coded	Client marital status: Married	Client age group: Under 20 (adolescent)	Time since trained: N/A - client

Codes Applied:	A6.1.1 - Reasons for potential interest in SI	A5.1 - Covert/ discreet use	C2.1.2 - Reduced trips to facility	A6.1.2 - Potential concerns about SI

INTERVIEWER: What about younger adolescents- do you think this method would appeal to them?

RESPONDENT: To some it would not be appealing since they will feel that they may be discovered

INTERVIEWER: Okay because they keep the injections at home? What would some say?

RESPONDENT: Mhhh (respondent not sure of what to answer) some would prefer it

INTERVIEWER: Why?

RESPONDENT: Because it has less visits

IINTERVIEWER: what do mean by less visits?

RESPONDNET: Going to the hospital now and then

INTERVIEWER: Ok going to the hospital now and the

RESPONDENT: Yes

Title: MZs_FAC_1.docx

Descriptor Info:	District: Mzimba South	Target Population: TP1 - Providers/HSAs	Respondent type: Facility-based provider	Aware of SI: Yes - had heard of SI before	Coding phase: 3. Triple coded	Client marital status: N/A - provider	Client age group: N/A - provider	Time since trained: A year or more ago

Codes Applied:	C1.1.1 - Positive client attitudes to SI	C2.1.2 - Reduced trips to facility

Interviewer: You said it has really changed, what could be the factor that has influenced this trend?

Respondent: Aaaah, I could see even the women themselves they could find it easier to take self-injection and continue at home and not reporting back for a year and I think to some it was a burden for them to come to the hospital only for injection, so they warmly welcomed it.

Title: ZA_IM_7.docx

Descriptor Info:	District: Zomba	Target Population: TP2 - DMPA-IM/DMPA-SC PI clients	Respondent type: DMPA-IM client	Aware of SI: No - never heard of SI before	Coding phase: 1. Single coded	Client marital status: Never married	Client age group: 20-29 years	Time since trained: N/A - client

Codes Applied:	C1.1.1 - Positive client attitudes to SI	C2.1.2 - Reduced trips to facility

Interviewer: Alright so after hearing this do you think women that are using injections administered to them at the hospital be interested to get the self-injected one?

Respondent: Yes.

Interviewer: Why?

Respondent: Because the self-injected method is good you will not have to come here at the hospital in order to get injected.

Title: MZs_SCSI_4.docx

Descriptor Info:	District: Mzimba South	Target Population: TP3 - DMPA-SC SI clients	Respondent type: DMPA-SC SI client	Aware of SI: Yes - had heard of SI before	Coding phase: 2. Double coded	Client marital status: Married	Client age group: 20-29 years	Time since trained: N/A - client

Codes Applied:	C1.1.1 - Positive client attitudes to SI	C2.1.2 - Reduced trips to facility

Interviewer: Do you ever think of resuming to self-inject in future when your husband is back?

Respondent: Yes

Interviewer: Why?

Respondent: It’s easy to follow dates which we are told for next doses

Interviewer: Did you ever go back to the provider for clarifications on the counselling the time you were using Sayana?

Respondent: On self-inject or?

Interviewer: Yes, on self-inject did you ever go to your provider for clarification

Respondent: No, we were given counselling on how to use it on the first day

Interviewer: You were told? Were you given Sayana at the health facility or at your community?

Respondent: At our community

Interviewer: What motivated you to keep on using Sayana Press from the provider?

Respondent: It’s because you use it at home, so you have all the time to do household chores without interruptions

Title: MZs_SCPI_3.docx

Descriptor Info:	District: Mzimba South	Target Population: TP2 - DMPA-IM/DMPA-SC PI clients	Respondent type: DMPA-SC PI client	Aware of SI: Yes - had heard of SI before	Coding phase: 1. Single coded	Client marital status: Married	Client age group: 20-29 years	Time since trained: N/A - client

Codes Applied:	C2.1.2 - Reduced trips to facility

Interviewer: So the way you see it, you still have that fear or it’s gone?

Respondent: Not anymore.

Interviewer: As of now can you be able to use self-injection?

Respondent: Yes.

Interviewer: So if they can counsel you on self-injection and ask you if you to use it, will you be able to use it?

Respondent: Yes.

Interviewer: Why would you opt for it?

Respondent: For child spacing and also for not visiting the facility every 3 months.

Interviewer: Anything else?

Respondent: The other thing is that I no longer have fear as before.

Title: MZs_SCPI_3.docx

Descriptor Info:	District: Mzimba South	Target Population: TP2 - DMPA-IM/DMPA-SC PI clients	Respondent type: DMPA-SC PI client	Aware of SI: Yes - had heard of SI before	Coding phase: 1. Single coded	Client marital status: Married	Client age group: 20-29 years	Time since trained: N/A - client

Codes Applied:	C2.1.2 - Reduced trips to facility

Interviewer: What do you think are the most important things covered in the video?

Respondent: The important thing is that once you have chosen Sayana self-injection, you will stay one year without visiting the facility for family planning.

Title: ZA_HSA_1.docx

Descriptor Info:	District: Zomba	Target Population: TP1 - Providers/HSAs	Respondent type: HSA	Aware of SI: Yes - had heard of SI before	Coding phase: 1. Single coded	Client marital status: N/A - provider	Client age group: N/A - provider	Time since trained: Less than a year ago

Codes Applied:	C1.1.1 - Positive client attitudes to SI	C8.2 - Covert/ open use of injectables/SI	C8.3 - Discretion of injectables/SI	C2.1.2 - Reduced trips to facility

Interviewer: Alright alright thank you very much. What is your experience in the response when you explain to clients that they could self-inject this method?

Respondent: Women do agree saying that they will shorten their traveling and time spent at the hospital

Interviewer: Alright alright what do you think women find most appealing about DMPA-SC for self-injection?

Respondent: What appeals to them is that they leave their homes and come here and find that there are a lot of people. When you do it on your own its more private

Interviewer: Mmmh

Respondent: People wouldn’t know that you came to the hospital for family planning. Others are not allowed by their husbands they just come in their own capacity. No one sees you on the queue, this is more confidential

Title: ZA_HSA_1.docx

Descriptor Info:	District: Zomba	Target Population: TP1 - Providers/HSAs	Respondent type: HSA	Aware of SI: Yes - had heard of SI before	Coding phase: 1. Single coded	Client marital status: N/A - provider	Client age group: N/A - provider	Time since trained: Less than a year ago

Codes Applied:	C2.1.2 - Reduced trips to facility	C1.1.1 - Positive client attitudes to SI

Interviewer: In your view, what are the main factors that would support a client to take up the self-injection option with DMPA-SC

Respondent: The time they would spend coming to the hospital, it would take them a few mins even seconds to self-inject from home

Interviewer: Alright

Respondent: They spend a lot of time coming to the hospital, that means they will be saving their time

Title: MZs_HSA_2.docx

Descriptor Info:	District: Mzimba South	Target Population: TP1 - Providers/HSAs	Respondent type: HSA	Aware of SI: Yes - had heard of SI before	Coding phase: 1. Single coded	Client marital status: N/A - provider	Client age group: N/A - provider	Time since trained: A year or more ago

Codes Applied:	C1.1.1 - Positive client attitudes to SI	C2.1.2 - Reduced trips to facility

Interviewer: from your experience, what do women say when you tell them to self-inject

Respondent: many women like the idea that they will self-inject at home, so it relieves costs of coming to the health facility

Interviewer: what do you think attract women to opt for Sayana if you have any additions

Respondent: they are relieved from the burden of coming to the health facility

Interviewer: anything else? Do women have fears on self-injection

Respondent: no

Title: MZs_HSA_2.docx

Descriptor Info:	District: Mzimba South	Target Population: TP1 - Providers/HSAs	Respondent type: HSA	Aware of SI: Yes - had heard of SI before	Coding phase: 1. Single coded	Client marital status: N/A - provider	Client age group: N/A - provider	Time since trained: A year or more ago

Codes Applied:	C8.1 - Male involvement, general	C8.2 - Covert/ open use of injectables/SI	C2.1.2 - Reduced trips to facility	C1.2.1 - Negative client attitudes to SI	C1.1.1 - Positive client attitudes to SI	C4 - Storage of unused units

Interviewer: we are now in the last part where I would like to hear about you views on why some women opt for Sayana Press compared to others. What do you think would motivate a woman to get Sayana Press for self-injection?

Respondent: most women like this method. Like I said more especially to women who travels since they cannot manage to come frequently at the health facility to get Sayana Press. If they get enough Sayana Press they reduce the frequency of coming at the health facility more especially for those who travels to South Africa if they get in bulk, it sustains them for a longer time. But there are some women who are not willing to get Sayana Press for self-injection because their husband does not support the idea of using family planning methods so it’s difficult for them to take home and store it because the husband might throw it in the toilet once found. Some are afraid to self-inject so they come we give provider injected Sayana Press at the health facility or at the community.

Title: MZs_IM_4.docx

Descriptor Info:	District: Mzimba South	Target Population: TP2 - DMPA-IM/DMPA-SC PI clients	Respondent type: DMPA-IM client	Aware of SI: Yes - had heard of SI before	Coding phase: 2. Double coded	Client marital status: Married	Client age group: 30-39 years	Time since trained: N/A - client

Codes Applied:	C2.1.2 - Reduced trips to facility

Q12. Interviewer: Can you describe your experience of being trained in self-injection?

Respondent: You mean the good ones or the bad ones?

Interviewer: Both good and the bad ones

Respondent: The good thing about this method is that it gives a woman time to other things freely

Title: MZs_IM_4.docx

Descriptor Info:	District: Mzimba South	Target Population: TP2 - DMPA-IM/DMPA-SC PI clients	Respondent type: DMPA-IM client	Aware of SI: Yes - had heard of SI before	Coding phase: 2. Double coded	Client marital status: Married	Client age group: 30-39 years	Time since trained: N/A - client

Codes Applied:	C2.1.2 - Reduced trips to facility

Interviewer: What do you think are the most important things covered in the video?

Respondent: The information that is there in proving training session to a client for self-injection method. The other thing is that a woman will be able to stay at home her children for some time without visiting the facility.

Title: MZs_IM_4.docx

Descriptor Info:	District: Mzimba South	Target Population: TP2 - DMPA-IM/DMPA-SC PI clients	Respondent type: DMPA-IM client	Aware of SI: Yes - had heard of SI before	Coding phase: 2. Double coded	Client marital status: Married	Client age group: 30-39 years	Time since trained: N/A - client

Codes Applied:	C1.1.1 - Positive client attitudes to SI	C2.1.2 - Reduced trips to facility

Interviewer: After seeing the video would you feel the same, more positive or more negative about self-injection?

Respondent: After watching the video, I feel more positive and the Sayana method is very good.

Q15. Interviewer: Did you try to self-inject?

Respondent: Yes, I did

Interviewer: What was your experience?

Respondent: It was a good method of family planning and it is helpful cause those who are using self-injection will no longer visit the facility regularly

Interviewer: What made you to try the method?

Respondent: After the training and hear that the method is good and simple thus when I decided to try the method by self-injecting myself before the provider and then he gave me 3 extra packets so that I can use it at home.

Title: KK_IM_2.docx

Descriptor Info:	District: Nkhotakota	Target Population: TP2 - DMPA-IM/DMPA-SC PI clients	Respondent type: DMPA-IM client	Aware of SI: Yes - had heard of SI before	Coding phase: 2. Double coded	Client marital status: Married	Client age group: 30-39 years	Time since trained: N/A - client

Codes Applied:	C2.1.2 - Reduced trips to facility	C1.1.1 - Positive client attitudes to SI

INTERVIEWER: Did the doctor say when the injection you wanted would be available, what did the doctor say?

RESPONDENT: The doctor said I would be called to come get it whenever another stock arrived.

INTERVIEWER: mmmmhu. What motivated you for you to say I will get Sayana?

RESPONDENT: What motivate…it was the motivation?

INTERVIEWER: mmmhu.

RESPONDENT: Anyhow, I would save the distance to come here to the hospital.

INTERVIEWER: okay.

RESPONDENT: Because once you get it, inject yourself at home

INTERVIEWER: mmmh.

RESPONDENT: Yes, once the injection ways off, you inject yourself.

INTERVIEWER: okay.

RESPONDENT: mmmh.

Title: KK_FAC_2.docx

Descriptor Info:	District: Nkhotakota	Target Population: TP1 - Providers/HSAs	Respondent type: Facility-based provider	Aware of SI: Yes - had heard of SI before	Coding phase: 1. Single coded	Client marital status: N/A - provider	Client age group: N/A - provider	Time since trained: A year or more ago

Codes Applied:	P2.3 - Provider training content	P3.1.1 - Reduced provider workload	C2.1.2 - Reduced trips to facility	P3.1.5 - Ease of training clients in SI

INTERVIEWER: Aaa we are continuing. I will ask you questions on your experiences during DMPA-SC training.

RESPONDENT: mmmhu.

INTERVIEWER: Aaah, can you tell me a little bit about the training you received in providing DMPA-SC? What training did you receive?

RESPONDENT: The training that we received regarding the self-injectable is how to counsel a client. On this on the counselling also training them on how they can self-inject and also how the self-injecting kit is so that when self-injecting they should not face any problems.

INTERVIEWER: okay.

RESPONDENT: mmmh.

INTERVIEWER: Alright. So what were the good parts of the training? [someone came in and disturbed us] continue, you can continue. Yeah.

RESPONDENT: Where were we?

INTERVIEWER: I asked about, wait [he came in again]. I asked you about the good parts of the training…training about Sayana?

RESPONDENT: Okay. There are a lot of good things; firstly, the kit for injecting the Sayana drug is very interesting because it has different parts that interests when learning. The other is that through that training, it reduced our workload because when we teach them and they accept to be self-injecting, we can give them up to four doses including the one they have injected at the facility. Which means they will have fewer visits to the hospital and also we have reduced our work load.

INTERVIEWER: Okay.

RESPONDENT: mmmh.

INTERVIEWER: Mmmhu. If there could be another training for instance right, what would you like to be improved in the training?

Time stamp: (10:09)

RESPONDENT: In my view, that training was just fine.

INTERVIEWER: mmmhu.

RESPONDENT: Because after the training we came to the facility and we started providing, we did not face any problems.

INTERVIEWER: okay.

RESPONDENT: yes.

INTERVIEWER: everything was…

RESPONDENT: And the questions that people were asking we were answering them very well.

Title: KK_FAC_2.docx

Descriptor Info:	District: Nkhotakota	Target Population: TP1 - Providers/HSAs	Respondent type: Facility-based provider	Aware of SI: Yes - had heard of SI before	Coding phase: 1. Single coded	Client marital status: N/A - provider	Client age group: N/A - provider	Time since trained: A year or more ago

Codes Applied:	C2.1.2 - Reduced trips to facility

INTERVIEWER: Alright. What do you think women find most appealing about DMPA-SC? What appeals to them?

RESPONDENT: There are a couple of things that appeals to them. First, they feel that when they self-inject, they will be doing their daily chores. Because most of the people here are farmers, they cultivate rice, maize and cassava. So for them to be frequently visiting the hospital, they feel that they are wasting their time. But when we give them Sayana maybe four for a year right, they will have enough free time.

Title: KK_FAC_2.docx

Descriptor Info:	District: Nkhotakota	Target Population: TP1 - Providers/HSAs	Respondent type: Facility-based provider	Aware of SI: Yes - had heard of SI before	Coding phase: 1. Single coded	Client marital status: N/A - provider	Client age group: N/A - provider	Time since trained: A year or more ago

Codes Applied:	C1.1.1 - Positive client attitudes to SI	C2.1.2 - Reduced trips to facility

INTERVIEWER: why do you think women are more interested in this method?

RESPONDENT: Firstly, aaa the syringe or needle of Sayana is small compared to Depo which has a big syringe. Even if the client is on Depo, but she is able to see that it has a big syringe. So women are attracted with that small needle. If I inject myself, it will be as if I am removing a small thorn and it is not painful, I will take this method. This is another reason that attracts them. (50:10)

INTERVIEWER: mmmhu.

RESPONDENT: mmmh.

INTERVIEWER: What is the other reason women at Malowa are more interested in Sayana?

RESPONDENT: I already explained the other reason. I have said that there are a lot of farmers and they don’t want to lose not even a day. They just want to be working continuously. So when they are using Sayana, if they self-inject, they will wait for three months and if we give them another three doses, that’s a year. It means they won’t be coming to the hospital unless they are sick that’s when they will come. Yeah.

Title: KK_IM_8.docx

Descriptor Info:	District: Nkhotakota	Target Population: TP2 - DMPA-IM/DMPA-SC PI clients	Respondent type: DMPA-IM client	Aware of SI: No - never heard of SI before	Coding phase: 1. Single coded	Client marital status: Married	Client age group: 20-29 years	Time since trained: N/A - client

Codes Applied:	C1.1.1 - Positive client attitudes to SI	C2.1.2 - Reduced trips to facility	C5.1.4 - Timely injection due to not having to rely on external factors

INTERVIEWER: The method is currently available from public health facilities in Malawi and from HSA’s. it is available and if you are interested…you have seen its advantages, right?

RESPONDENT: yes

INTERVIEWER: You can come here and talk with a nurse or you can go to that HSA and they will train you

RESPONDENT: Alright alright. You can have fewer visits to the hospital because during the rainy season, sometimes I fail to come to the hospital because of rain although I stay close to the hospital.

INTERVIEWER: Yes. After hearing what I have explained to you, do you think that women who are already using injectable method from the hospital or from an HSA might be more interested in self-injection method?

RESPONDENT: Yes, they might be more interested.

INTERVIEWER: or I should ask you, are you interested?

RESPONDENT: yes, I am interested because with the way you have explained, it’s a good thing. You will have Sayana at home and self-inject after every 3 months. Sometimes you are busy and you are also required to go the hospital the same day and you might not go. But when you have Sayana at home, you know that I will inject myself when I get back even at night.

INTERVIEWER: okay.

RESPONDENT: sure.
