# Supplementary material for: Health care provider and client experiences of counselling on depot medroxyprogesterone acetate subcutaneous (DMPA-SC) for self-injection in Malawi
Source: PLOS Glob Public Health. 2023 Nov 30;3(11):e0002057. doi: 10.1371/journal.pgph.0002057 (PMC10688740; doi:10.1371/journal.pgph.0002057)
Supplement: S1 Data — (ZIP) [file pgph.0002057.s001.zip › S2/C2.2 other barriers to client access.doc]

Title: ZA_HSA_2.docx

Descriptor Info:	District: Zomba	Target Population: TP1 - Providers/HSAs	Respondent type: HSA	Aware of SI: Yes - had heard of SI before	Coding phase: 3. Triple coded	Client marital status: N/A - provider	Client age group: N/A - provider	Time since trained: Less than a year ago

Codes Applied:	C2.2 - Barriers, general/other

Respondent: So, it depends what time the client has come to the facility, some of the clients come at dusk…

Interviewer: mm (showing that one is following the conversation)

Respondent: Yes, so at dusk it is very hard for us to train the client. But we are still able to assist the client because some of the clients come late because they already know the methods, yeah…

Interviewer: mm (Showing that one is following the conversation)

Respondent: and some of them have jobs, they go to work early and knock off late so we accept them, that they can come late here at the facility. But if they need to be trained and they want to know more…

Interviewer: mm (showing that one is following the conversation)

Respondent: We can still discuss with them,

Title: ZA_FAC_2.docx

Descriptor Info:	District: Zomba	Target Population: TP1 - Providers/HSAs	Respondent type: Facility-based provider	Aware of SI: Yes - had heard of SI before	Coding phase: 3. Triple coded	Client marital status: N/A - provider	Client age group: N/A - provider	Time since trained: Less than a year ago

Codes Applied:	C2.2 - Barriers, general/other	P3.5.1 - Trends/numbers of SI

Interviewer: Per 100 women, how many would you say come to your facility to get the self-injection method?

Time stamp: 20:12

Respondent: Per 100 women, about 50 get the self-injection method.

Interviewer: So you mean half of those administer the method on their own?

Respondent: Yes.

Interviewer: Okay, thank you. Can you say that the way the self-injection method is taken up by women has changed recently?

Respondent: Yes. Mainly because of the covid 19 pandemic as most women were afraid to come to the hospital.

Title: ZA_HSA_6.docx

Descriptor Info:	District: Zomba	Target Population: TP1 - Providers/HSAs	Respondent type: HSA	Aware of SI: Yes - had heard of SI before	Coding phase: 1. Single coded	Client marital status: N/A - provider	Client age group: N/A - provider	Time since trained:

Codes Applied:	C2.2 - Barriers, general/other	P3.5.1 - Trends/numbers of SI

Interviewer: Alright, in your job have you experienced any problems in the past three months?

Respondent: ah the turn up for clients in the village clinics

Interviewer: mmmm (showing that one is following the conversation)

Respondent: there was not any problem

Interviewer: mmmm (showing that one is following the conversation)

Respondent: but the turn up in the static clinic at this facility… there was a problem, the numbers of people coming to the hospital specially to access family planning services went down.

Interviewer: mmmm (showing that one is following the conversation)

Respondent: because of the time that they introduced covid-19 vaccine

Interviewer: okay

Respondent: but also, because they were conducting covid-19 testing at this facility

Interviewer: mmmm (showing that one is following the conversation)

Respondent: So, the people were scared that we should not go to the hospital because of particular reasons.

Interviewer: mmmm (showing that one is following the conversation)

Respondent: So, for the past three months we can say that the turn up went down a little bit but now it is just okay.

Interviewer: alright.

Respondent: sure

Title: MZs_IM_1.docx

Descriptor Info:	District: Mzimba South	Target Population: TP2 - DMPA-IM/DMPA-SC PI clients	Respondent type: DMPA-IM client	Aware of SI: Yes - had heard of SI before	Coding phase: 1. Single coded	Client marital status: Married	Client age group: 30-39 years	Time since trained: N/A - client

Codes Applied:	C0.4 - Access to or process of obtaining FP services	C2.1.1 - Community availability of injectables/SI	C2.2 - Barriers, general/other

Q6. Interviewer: How easy or difficult have you found it accessing family planning services in the past?

Respondent: It is very easy because every Tuesday our HSA visit the community.

Interviewer: You have said that you receive family planning method from HSA?

Respondent: Yes

Interviewer: Since you started, have you been receiving the method from an HSA or from the facility?

Respondent: Most of the times I have been receiving it from the HSA, but sometimes it happens that the HSA have failed to show up as they planned then we do go to Jenda health for the method. But most of the times they do show up every Tuesday.

Interviewer: You have said that, if an HSA failed to show up you do visit the facility for the method, So have you done that before?

Respondent: Yes, and in have done it in different occasions. If an HSA has not shown up maybe he is busy we do go to the facility for the method, also may be as a client I did not manage to visit the clinic then I need to find time to visit the facility for the method.

Interviewer: Has there been easy or difficult in accessing family planning services changed since the outbreak of coronavirus?

Respondent: No, there is no change.

Title: ZA_IM_7.docx

Descriptor Info:	District: Zomba	Target Population: TP2 - DMPA-IM/DMPA-SC PI clients	Respondent type: DMPA-IM client	Aware of SI: No - never heard of SI before	Coding phase: 1. Single coded	Client marital status: Never married	Client age group: 20-29 years	Time since trained: N/A - client

Codes Applied:	C1.1.1 - Positive client attitudes to SI	C2.2 - Barriers, general/other

Interviewer: What made you decide to take this method? because I asked you if you would be comfortable using this and you said yes?

Respondent: Like I explained before I decided to take this method because most of the time I walk to come here and when am here it takes a long time for me to be helped.
