# Supplementary material for: Health care provider and client experiences of counselling on depot medroxyprogesterone acetate subcutaneous (DMPA-SC) for self-injection in Malawi
Source: PLOS Glob Public Health. 2023 Nov 30;3(11):e0002057. doi: 10.1371/journal.pgph.0002057 (PMC10688740; doi:10.1371/journal.pgph.0002057)
Supplement: S1 Data — (ZIP) [file pgph.0002057.s001.zip › S2/C2.2.1 stockouts + client access.doc]

Title: ZA_HSA_2.docx

Descriptor Info:	District: Zomba	Target Population: TP1 - Providers/HSAs	Respondent type: HSA	Aware of SI: Yes - had heard of SI before	Coding phase: 3. Triple coded	Client marital status: N/A - provider	Client age group: N/A - provider	Time since trained: Less than a year ago

Codes Applied:	C2.2.1 - Stockouts/stocks near expiry dates	C1.2.2 - Fear of SI (idea or reality)	C7 - Resupply

Interviewer: Ah In your view, what are the main reasons that discourage clints to take up self-injection option with DMPA-SC?

Respondent: The first reason is the commodity stock-outs.

Interviewer: Alright.

Respondent: Yes.

Interviewer: Alright.

Respondent: Yes, the commodity stock-outs.

Interviewer: mmm (showing that one is following the conversation)

Respondent: Of course, some would say the injection is painful, but a needle is a needle, it is supposed to be painful a little bit.

Interviewer: mmm (showing that one is following the conversation)

Respondent: Yeah, but the main issue is the commodity stock-outs…

Interviewer: mmmm (showing that one is following the conversation)

Respondent: Because a woman can choose the method but then come four months the commodity is no longer available,

Interviewer: mmm (showing that one is following the conversation)

Respondent: we can talk to them, they select the method, and we provide them with doses for the entire year…

Interviewer: mmm (showing that one is following the conversation)

Respondent: Yeah for those ones it would be good, but for those who have chosen the method and at the time they do we inject them then we say we no longer have the commodity , when they come back they resort back to Depo-IM, when the commodity is now available we shift back to it, when this happens the clients are always reluctant, they will complain that DMPA-SC is rarely in stock so they would rather get DMPA-IM, but if the commodity is available

Interviewer: mmm (showing that none is following the conversation)

Respondent: Yeah, for example for the past three months yeah…

Interviewer: mmm (showing that one is following the conversation)

Respondent: The commodity is not available, so the clients that we have given in the past came and said, the method that you trained us in is good, so we have come to collect more of that commodity, so we tell them that right now I have Depo that I will inject you

Interviewer: mmmm (showing that one is following the conversation)

Respondent: The other one that you self-inject is not available…

Interviewer: Its out of stock.

Respondent: But when it is available I will what, I will tell you the date that you can, the last date that you will inject, then…

Interviewer: mmm (showing that one is following the conversation)

Respondent: You will take that one…

Title: MZs_SCSI_3.docx

Descriptor Info:	District: Mzimba South	Target Population: TP3 - DMPA-SC SI clients	Respondent type: DMPA-SC SI client	Aware of SI: Yes - had heard of SI before	Coding phase: 1. Single coded	Client marital status: Married	Client age group: 30-39 years	Time since trained: N/A - client

Codes Applied:	C2.2.1 - Stockouts/stocks near expiry dates	C1.1.2 - Baseline correct knowledge/ awareness of injectables

Interviewer: why did you switch to self-injection?

Respondent: Depo IM was out of stock, so the only option was self-injection

Title: MZs_SCSI_1.docx

Descriptor Info:	District: Mzimba South	Target Population: TP3 - DMPA-SC SI clients	Respondent type: DMPA-SC SI client	Aware of SI: Yes - had heard of SI before	Coding phase: 1. Single coded	Client marital status: Married	Client age group: Under 20 (adolescent)	Time since trained: N/A - client

Codes Applied:	C2.2.1 - Stockouts/stocks near expiry dates

Q13. Interviewer: Are you currently still using DMPA-SC for self-injection?

Respondent: No

Interviewer: Why did you stop?

Respondent: The time I went to get the method I was told that Sayana is out of stock

Interviewer: What factors influenced you to stop using DMPA-SC for self-inject

Respondent: The Sayana Press is out of stock

Title: KK_FAC_1.docx

Descriptor Info:	District: Nkhotakota	Target Population: TP1 - Providers/HSAs	Respondent type: Facility-based provider	Aware of SI: Yes - had heard of SI before	Coding phase: 3. Triple coded	Client marital status: N/A - provider	Client age group: N/A - provider	Time since trained: A year or more ago

Codes Applied:	C2.2.1 - Stockouts/stocks near expiry dates	P3.2.2 - Stockouts/stocks near expiry dates

INTERVIEWER: Alright fine. Our last question, in your view what are the main factors that would discourage clients to take the self-injection option with DMPA-SC? Factors that can discourage them.

RESPONDENT: The issue is the one that I said at some point on stockouts, this affects so much

INTERVIEWER: Okay

RESPONDENT: Yes, like the time we hard short life Sayana , we had clients asking to take it but since the expiry date was so close

INTERVIEWER: Yes

RESPONDENT: So we could not give out the method since we knew that the medication would expire at home

INTERVIEWER: Yes

RESPONDENT: So when procuring the medicines we should consider expiry dates.

INTERVIEWER: Ok

RESPONDENT: Sure

INTERVIEWER: Fine

RESPONDENT: Because if a client comes to request for the method and we tell them we have expired stuff they get disappointed, then next time they will not have the courage to ask for it again.

INTERVIEWER: So it means the client would go back to depo?

RESPONDENT: Yes, to the previous one depo

Title: ZA_IM_1.docx

Descriptor Info:	District: Zomba	Target Population: TP2 - DMPA-IM/DMPA-SC PI clients	Respondent type: DMPA-IM client	Aware of SI: Yes - had heard of SI before	Coding phase: 1. Single coded	Client marital status: Married	Client age group: 40-49 years	Time since trained: N/A - client

Codes Applied:	C1.1.1 - Positive client attitudes to SI	C2.1.2 - Reduced trips to facility	C2.2.1 - Stockouts/stocks near expiry dates

Respondent: It’s not available at the moment, am asking that it should be made available its very good.

Interviewer: Alright thank you very much. You explained that you previously used self-injection, how many times did you use it?

Respondent: I used it twice

Interviewer: You were given 3?

Respondent: Yes, 3

Interviewer: You used all three?

Respondent: Yes I used them all, that was in 2019

Interviewer: Did you inject all 3?

Respondent: Yes

Interviewer: How do you view this method?

Respondent: I find this method to be very good. As I previously mentioned you rest from going to the hospital. We come from a distance, so you just inject yourself when time arises

Interviewer: So what made you to go back to receiving your injection from the hospital?

Respondent: This is because that self-injection is not available at this hospital, the only available one is the one that injectable at the hospital.

Interviewer: Alright alright, we have come to the end of the questions I had for you today. Do you have any other thoughts or information you would like to share on the topics we have discussed today?

Respondent: I have additions, as I earlier mentioned self-injection is not currently available. It is my plea we still want self-injection.

Interviewer: Alright alright, you last self-injected in 2019 you said?

Respondent: No, I last self-injected in 2020 then started receiving from from the hospital the the one that is administered on the hand.

Title: KK_SCPI_6.docx

Descriptor Info:	District: Nkhotakota	Target Population: TP2 - DMPA-IM/DMPA-SC PI clients	Respondent type: DMPA-SC PI client	Aware of SI: Yes - had heard of SI before	Coding phase: 1. Single coded	Client marital status: Married	Client age group: 20-29 years	Time since trained: N/A - client

Codes Applied:	C2.2.1 - Stockouts/stocks near expiry dates

INTERVIEWER: Why did you not get and do it at home?

RESPONDENT: No, I said I would do it on the next injection.

INTERVIEWER: mmmmhu. So what happened the second time you came?

RESPONDENT: I just saw that they injected me with the other type because Sayana was out of stock.

Title: KK_SCSI_3.doc

Descriptor Info:	District: Nkhotakota	Target Population: TP3 - DMPA-SC SI clients	Respondent type: DMPA-SC SI client	Aware of SI: Yes - had heard of SI before	Coding phase: 1. Single coded	Client marital status: Married	Client age group: 20-29 years	Time since trained: N/A - client

Codes Applied:	C5.2.3 - Lack of resources/follow up materials to aid timely re-injection	C2.2.1 - Stockouts/stocks near expiry dates

INTERVIEWER: What else did they give you to take home after your training?

RESPONDENT: After the training, they gave me Sayana and I self-injected. They also gave me condoms.

INTERVIEWER: they gave you condoms?

RESPONDENT: yes. Because they said the drug starts working after seven days.

INTERVIEWER: okay.

RESPONDENT: sure

INTERVIEWER: how about Sayana?

RESPONDENT: sa…Sayana, no?

INTERVIEWER: for self-injecting at home.

RESPONDENT: they did not give me.

INTERVIEWER: why?

RESPONDENT: They said they were out of stock; they only had the one we used. During that time, they only had Sayana on the method of injectable.

INTERVIEWER: Did they tell you when you had to come back?

RESPONDENT: they did not

INTERVIEWER: they did not?

RESPONDENT: mmmmh.

Title: ZA_SCSI_2.docx

Descriptor Info:	District: Zomba	Target Population: TP3 - DMPA-SC SI clients	Respondent type: DMPA-SC SI client	Aware of SI: Yes - had heard of SI before	Coding phase: 1. Single coded	Client marital status:	Client age group:	Time since trained: N/A - client

Codes Applied:	C2.2.1 - Stockouts/stocks near expiry dates	C5.1.1 - Continuation of SI (including reasons)

Interviewer: Alright, are you still using DMPA SC for self-injection?

Respondent: No, currently they are saying it’s not available.

Interviewer: Alright.

Respondent: so currently we just use DMPA IM

Interviewer: Alright, are there any other reasons why you stopped using DMPA SC apart from the one that you have told me?

Respondent: Another reason?

Interviewer: mmmm (agreeing)

Respondent: there is no other reason apart from that it is not available in the government facilities.

Interviewer: Alright.

Respondent: maybe in the private ones.

Interviewer: Alright.

Respondent: but this commodity was good, it’s not available.

Title: ZA_SCSI_2.docx

Descriptor Info:	District: Zomba	Target Population: TP3 - DMPA-SC SI clients	Respondent type: DMPA-SC SI client	Aware of SI: Yes - had heard of SI before	Coding phase: 1. Single coded	Client marital status:	Client age group:	Time since trained: N/A - client

Codes Applied:	C1.1.1 - Positive client attitudes to SI	C2.2.1 - Stockouts/stocks near expiry dates

Interviewer: Do you have any thoughts or other things that you would like to share with me on the topics that we have discussed?

Respondent: Concerning DMPA SC?

Interviewer: mmm (saying yes)

Respondent: we like this injection

Interviewer: mmm (showing that one is following the conversation)

Respondent: but now it is not available.

Interviewer: alright.

Respondent: So we are asking if it could be back in stock

Interviewer: Alright.

Respondent: Yes

Title: KK_IM_7.docx

Descriptor Info:	District: Nkhotakota	Target Population: TP2 - DMPA-IM/DMPA-SC PI clients	Respondent type: DMPA-IM client	Aware of SI: Yes - had heard of SI before	Coding phase: 1. Single coded	Client marital status: Married	Client age group: 30-39 years	Time since trained: N/A - client

Codes Applied:	C2.2.1 - Stockouts/stocks near expiry dates

INTERVIEWER: Mmmmhu, alright. When you failed to self-inject at home, you came back the next day right? Did they train you again?

RESPONDENT: Aaaah they did, and they said that with what they have seen, you have to go without Sayana. On your next visit date, you will be trained again. on my next visit date, there was stockout of Sayana.

INTERVIEWER: okay.

RESPONDENT: Yes. I think I would have been able to self-inject after training during that visit. They said there was a stockout and the provider injected me.

Title: KK_IM_7.docx

Descriptor Info:	District: Nkhotakota	Target Population: TP2 - DMPA-IM/DMPA-SC PI clients	Respondent type: DMPA-IM client	Aware of SI: Yes - had heard of SI before	Coding phase: 1. Single coded	Client marital status: Married	Client age group: 30-39 years	Time since trained: N/A - client

Codes Applied:	C2.2.1 - Stockouts/stocks near expiry dates

RESPONDENT: Yes, I managed and they were saying why did you fail (laughs). And they told us to go home, and you will get Sayana on your next visit because by the you will be able to do it.

INTERVIEWER: okay.

RESPONDENT: mmmh.

INTERVIEWER: chabwino. Alright.

RESPONDENT: On my next visit, there was a stockout.

Title: ZA_IM_5.docx

Descriptor Info:	District: Zomba	Target Population: TP2 - DMPA-IM/DMPA-SC PI clients	Respondent type: DMPA-IM client	Aware of SI: Yes - had heard of SI before	Coding phase: 2. Double coded	Client marital status: Married	Client age group: 30-39 years	Time since trained: N/A - client

Codes Applied:	C2.2.1 - Stockouts/stocks near expiry dates	C7 - Resupply

Interviewer: Alright, ah after you watched the video do you think your perspective on self-injection has changed?

Respondent: Ah self-injection.

Interviewer: Mmmm (showing that one is following the conversation)

Respondent: They have not changed, during this period we changed because the commodity was out of stock, they were not making it available for us.

Title: ZA_IM_5.docx

Descriptor Info:	District: Zomba	Target Population: TP2 - DMPA-IM/DMPA-SC PI clients	Respondent type: DMPA-IM client	Aware of SI: Yes - had heard of SI before	Coding phase: 2. Double coded	Client marital status: Married	Client age group: 30-39 years	Time since trained: N/A - client

Codes Applied:	C2.2.1 - Stockouts/stocks near expiry dates	C5.2.1 - Discontinuation of SI (including reasons)

Interviewer: Ah what made you go back to the previous method of family planning, the provider administered injectable? After you used Sayana you went back to the provider administered injectable, what caused that change?

Respondent: What made me change is that when I went to get Depo

Interviewer: Mmmm (showing that one is following the conversation)

Respondent: I found that the injection was not there.

Interviewer: Eh eh

Respondent: So I had to go back to use Depo.

Interviewer: Eh

Respondent: Which seemed like I was just using Depo and I just continued…

Title: MZs_SCSI_5.docx

Descriptor Info:	District: Mzimba South	Target Population: TP3 - DMPA-SC SI clients	Respondent type: DMPA-SC SI client	Aware of SI: Yes - had heard of SI before	Coding phase: 1. Single coded	Client marital status: Married	Client age group: 20-29 years	Time since trained: N/A - client

Codes Applied:	C3.1.1 - Practice of SI during training	C2.2.1 - Stockouts/stocks near expiry dates

Interviewer: Did you have any fears after being told that you will self-inject?

Respondent: What kind of fears?

Interviewer: Did you have fears after being told to self-inject?

Respondent: I did not have fears because I was self-injecting under direct observation by the provider. But they did not give us to take home because they had limited stock. I have never self-injected alone it was under direct observation by the provider

Title: MZs_SCSI_5.docx

Descriptor Info:	District: Mzimba South	Target Population: TP3 - DMPA-SC SI clients	Respondent type: DMPA-SC SI client	Aware of SI: Yes - had heard of SI before	Coding phase: 1. Single coded	Client marital status: Married	Client age group: 20-29 years	Time since trained: N/A - client

Codes Applied:	C2.2.1 - Stockouts/stocks near expiry dates

Interviewer: Did you ever ask providers why it’s out of stock?

Respondent: We do ask they tell us they have not received from Mzimba DHO

Interviewer: Mmmh (agreeing)

Respondent: We usually ask because it’s the family planning method we want not condoms and pills

Title: ZA_IM_9.docx

Descriptor Info:	District: Zomba	Target Population: TP2 - DMPA-IM/DMPA-SC PI clients	Respondent type: DMPA-IM client	Aware of SI: Yes - had heard of SI before	Coding phase: 1. Single coded	Client marital status: Married	Client age group: 20-29 years	Time since trained: N/A - client

Codes Applied:	C2.2.1 - Stockouts/stocks near expiry dates

Interviewer: Alright, thank you so much. So you said when you came here at the hospital, they told you about the self-injecting injection. So why didn’t you want to start using the method?

Respondent: Because right now it is not available. Mmh.

Interviewer: So if available, would you be happy using this method?

Respondent Yes, I would come to use this contraception method.

Title: ZA_SCSI_4.docx

Descriptor Info:	District: Zomba	Target Population: TP3 - DMPA-SC SI clients	Respondent type: DMPA-SC SI client	Aware of SI: Yes - had heard of SI before	Coding phase: 2. Double coded	Client marital status: Married	Client age group: 30-39 years	Time since trained: N/A - client

Codes Applied:	C2.2.1 - Stockouts/stocks near expiry dates

Interviewer: Alright, ah before you took self-injection, before the corona virus pandemic have you ever used this self-injection contraceptive?

Respondent: No I can say that I have never used, that time I started using that period

Interviewer: mmmm (showing that ne is following the conversation)

Respondent: Because we just used to hear that there is that injectable but it was what... it wasn’t available it was scarce.

Interviewer: mmm (showing that one is following the conversation)

Respondent: it wasn’t yet available at out facility.

Interviewer: yah

Respondent: It became available mostly during that period.

Title: ZA_SCPI_8.docx

Descriptor Info:	District: Zomba	Target Population: TP2 - DMPA-IM/DMPA-SC PI clients	Respondent type: DMPA-SC PI client	Aware of SI: Yes - had heard of SI before	Coding phase: 1. Single coded	Client marital status:	Client age group:	Time since trained: N/A - client

Codes Applied:	C2.2.1 - Stockouts/stocks near expiry dates

Interviewer: alright, After you heard about this, how did you feel?

Respondent: when I first heard about this.

Interviewer: mmmm (showing that one is following the conversation)

Respondent: since they just said that they will inject me with the usual injectable that was available then, I was told that when I come back in October, I will find the self-injecting injectable, so when I went back to the HSAs, I was told that it is out of stock, and I was not able to collect from the facility to have it at home so just wait for new stock.

Interviewer: alright.

Respondent: so I did not want to wait, that’s why I came here at [NAME] to get the contraceptive.

Title: MZs_IM_4.docx

Descriptor Info:	District: Mzimba South	Target Population: TP2 - DMPA-IM/DMPA-SC PI clients	Respondent type: DMPA-IM client	Aware of SI: Yes - had heard of SI before	Coding phase: 2. Double coded	Client marital status: Married	Client age group: 30-39 years	Time since trained: N/A - client

Codes Applied:	C2.2.1 - Stockouts/stocks near expiry dates

Q11. Interviewer: As a result of the discussion you had with the provider, did you agree to try self-injection?

Respondent: Yes, I did though it was only once.

Interviewer: Mmmmmh….(following)

Respondent: Yes

Interviewer: Then why did you stop using it?

Respondent: After using the first Sayana that I was given, I went back to the provider to get another doses but I was told that Sayana is out of stock, and I came back the other day and I was also told the same thing, then I later decided that I should just get Depo, for me to avoid pregnancy.

Title: KK_IM_2.docx

Descriptor Info:	District: Nkhotakota	Target Population: TP2 - DMPA-IM/DMPA-SC PI clients	Respondent type: DMPA-IM client	Aware of SI: Yes - had heard of SI before	Coding phase: 2. Double coded	Client marital status: Married	Client age group: 30-39 years	Time since trained: N/A - client

Codes Applied:	C2.2.1 - Stockouts/stocks near expiry dates	C3.3 - Counselling / training approach for injectable users

INTERVIEWER: mmmhu. You have told me that you had discussed with the doctor and the HSAs right. Did you try to inject yourself to accept that you will take this method?

RESPONDENT: No

INTERVIEWER: Why?

RESPONDENT: I did not find it at the hospital that time.

INTERVIEWER: It was not there…

RESPONDENT: The self-injecting one.

INTERVIEWER: Okay…you should have…

RESPONDENT: The one I found is the one for you to be injected by the doctor

INTERVIEWER: Okay. Had you found it…

RESPONDENT: I would have taken it.

INTERVIEWER: You would have taken it?

RESPONDENT: mmmh.

INTERVIEWER: Now, what did the doctor tell you? Did you tell the doctor that you would want to take?

RESPONDENT: Yes. I had wanted to change the method right. I wanted to get a five-year Norplant, but the five-year Norplant was not available as well at that time. Then I said I would get the self-injecting one which they said the stock had ended the day before.

INTERVIEWER: and they…

RESPONDENT: And the one available is for you to be injected right here, so I waited, and I was injected.

INTERVIEWER: Did the doctor say when the injection you wanted would be available, what did the doctor say?

RESPONDENT: The doctor said I would be called to come get it whenever another stock arrived.

Title: KK_IM_8.docx

Descriptor Info:	District: Nkhotakota	Target Population: TP2 - DMPA-IM/DMPA-SC PI clients	Respondent type: DMPA-IM client	Aware of SI: No - never heard of SI before	Coding phase: 1. Single coded	Client marital status: Married	Client age group: 20-29 years	Time since trained: N/A - client

Codes Applied:	C2.2.1 - Stockouts/stocks near expiry dates

INTERVIEWER: Depo, ok. Can you describe the process for obtaining you 3-month injection? How do you obtain an injection on every three months?

RESPONDENT: I get it from the hospital.

INTERVIEWER: for the hospital?

RESPONDENT: Yes. When we come, we ask that we want an injectable contraception. Sometimes when they say that there is stock out, we go back.

INTERVIEWER: okay?

RESPONDENT: yes

INTERVIEWER: that really happens?

RESPONDENT: Yes. Because sometimes when you come, you are told that the injectable you are looking for, Depo is out of stock.

INTERVIEWER: so when that happens you go back?

RESPONDENT: Yes, we go back and use other methods at home.

INTERVIEWER: Okay. So how do you know that the injectable now is available I should go for an injection?

RESPONDENT: We come to ask. And sometimes we go to that HSA I have mentioned to ask. And she says it’s still not available and I’ll tell you when available. Because we stay close to them, when they are passing by, the tell me that the injection is now available I should go for an injection

Time stamp: (20:14).

INTERVIEWER: okay.

RESPONDENT: sure.

INTERVIEWER: How many times have you been sent back because of Depo stock out?

RESPONDENT: once

INTERVIEWER: once

RESPONDENT: yes, since I started this method.
