# Supplementary material for: Health care provider and client experiences of counselling on depot medroxyprogesterone acetate subcutaneous (DMPA-SC) for self-injection in Malawi
Source: PLOS Glob Public Health. 2023 Nov 30;3(11):e0002057. doi: 10.1371/journal.pgph.0002057 (PMC10688740; doi:10.1371/journal.pgph.0002057)
Supplement: S1 Data — (ZIP) [file pgph.0002057.s001.zip › S2/C3.1 other enablers of client uptake.doc]

Title: KK_SCSI_5.docx

Descriptor Info:	District: Nkhotakota	Target Population: TP3 - DMPA-SC SI clients	Respondent type: DMPA-SC SI client	Aware of SI: Yes - had heard of SI before	Coding phase: 2. Double coded	Client marital status: Married	Client age group: 20-29 years	Time since trained: N/A - client

Codes Applied:	C3.1 - Enablers, general/other	C1.2.2 - Fear of SI (idea or reality)

INTERVIEWER: Is it so?

RESPONDENT: You just inject yourself. And it’s not that difficult and its nit painful. Most people say it’s painful but it’s not, but the one that is administered by the provider it is painful than the one you self-inject.

INTERVIEWER: Ok

RESPONDENT: Yes, that one is more painful but the self -injection one it is very simple

INTERVIEWER: It’s not painful?

RESPONDENT: Not at all

Title: KK_SCSI_5.docx

Descriptor Info:	District: Nkhotakota	Target Population: TP3 - DMPA-SC SI clients	Respondent type: DMPA-SC SI client	Aware of SI: Yes - had heard of SI before	Coding phase: 2. Double coded	Client marital status: Married	Client age group: 20-29 years	Time since trained: N/A - client

Codes Applied:	C3.1 - Enablers, general/other	C1.1.1 - Positive client attitudes to SI

RESPONDENT: Furthermore, the injection that is used for self-injection is smaller than the one used by the provider. Hence when you look at it size you say I can manage to inject myself

INTERVIEWER: Ok

RESPONDENT: Yes

Title: MZs_HSA_1.docx

Descriptor Info:	District: Mzimba South	Target Population: TP1 - Providers/HSAs	Respondent type: HSA	Aware of SI: Yes - had heard of SI before	Coding phase: 2. Double coded	Client marital status: N/A - provider	Client age group: N/A - provider	Time since trained:

Codes Applied:	P1.1.1 - Positive provider attitudes towards SI	C2.1.2 - Reduced trips to facility	C1.1.1 - Positive client attitudes to SI	C3.1 - Enablers, general/other

Q17. Interviewer: In your view, what are the main factors that would support a client to take up the self-injection option with DMPA-SC?

Respondent: The main factor is that it saves time to a client by not visiting the facility regularly and spending her time on other things

Interviewer: Mmmmh (following)

Respondent: Yes

Interviewer: Do you have anything to add?

Respondent: The Sayana needle is very small thus why women do accept to self-inject.

Title: KK_SCPI_4.docx

Descriptor Info:	District: Nkhotakota	Target Population: TP2 - DMPA-IM/DMPA-SC PI clients	Respondent type: DMPA-SC PI client	Aware of SI: Yes - had heard of SI before	Coding phase: 2. Double coded	Client marital status: Never married	Client age group: 20-29 years	Time since trained: N/A - client

Codes Applied:	C3.1 - Enablers, general/other	C1.2.2 - Fear of SI (idea or reality)

INTERVIEWER: okay. For today, what injection are you taking?

RESPONDENT: I will get Sayana

INTERVIEWER: Are you going to inject yourself?

RESPONDENT: I will, I will try to gather some confidence and inject myself.

Title: MZs_HSA_4.docx

Descriptor Info:	District: Mzimba South	Target Population: TP1 - Providers/HSAs	Respondent type: HSA	Aware of SI: Yes - had heard of SI before	Coding phase: 1. Single coded	Client marital status: N/A - provider	Client age group: N/A - provider	Time since trained:

Codes Applied:	C3.1 - Enablers, general/other	C1.1.1 - Positive client attitudes to SI

Interviewer: what are the clients attracted to when it comes to self-injection?

Respondent: the clients are attracted to self-injection because nobody touches their bodies.

Title: MZs_IM_7.docx

Descriptor Info:	District: Mzimba South	Target Population: TP2 - DMPA-IM/DMPA-SC PI clients	Respondent type: DMPA-IM client	Aware of SI: Yes - had heard of SI before	Coding phase: 1. Single coded	Client marital status: Divorced/ separated/ widowed	Client age group: Under 20 (adolescent)	Time since trained: N/A - client

Codes Applied:	C1.1.1 - Positive client attitudes to SI	C3.1 - Enablers, general/other

Interviewer: so now I would like to ask you questions. After hearing about Sayana Press do you think more women may be interested in using this method?

Respondent: to give them?

Interviewer: I was explaining to you about Sayana where by a woman self-inject. Upon hearing do you think more women would be interested with this method?

Respondent: yes

Interviewer: why do you think so?

Respondent: like you said it prevent pregnancy and some may gain weight. I have forgotten others

Interviewer: repeated on the benefits…so upon hearing do you think women in your area who are using Depo may want to use Sayana?

Respondent: yes

Interviewer: why?

Respondent: as you said when you want to get pregnant again it is not difficult, but also the needle is smaller compared to Depo IM.

Title: MZs_SCSI_2.docx

Descriptor Info:	District: Mzimba South	Target Population: TP3 - DMPA-SC SI clients	Respondent type: DMPA-SC SI client	Aware of SI: Yes - had heard of SI before	Coding phase: 3. Triple coded	Client marital status: Married	Client age group: Under 20 (adolescent)	Time since trained: N/A - client

Codes Applied:	C3.1.2 - Confidence/comfort to inject after counselling/ training	C3.1 - Enablers, general/other	C1.1.1 - Positive client attitudes to SI

Interviewer: What were the most important factors that motivating you to try?

Respondent: The only thing that made me to try this method of self-injection, it was after seeing my friends doing it and they were also saying that there is no effect, and it is better than the Depo, cause with Depo sometimes the syringe hurts because of the hands of the person who is doing it, but with this one there is nothing like that, thus why I wanted to try this self-injection.

Interviewer: Has your perspective changed over time?

Respondent: Yes, my perspective has changed and all the concerns about the injection that I had are no longer and I do not have any worry about this self-injection.

Title: KK_IM_7.docx

Descriptor Info:	District: Nkhotakota	Target Population: TP2 - DMPA-IM/DMPA-SC PI clients	Respondent type: DMPA-IM client	Aware of SI: Yes - had heard of SI before	Coding phase: 1. Single coded	Client marital status: Married	Client age group: 30-39 years	Time since trained: N/A - client

Codes Applied:	C3.1 - Enablers, general/other

INTERVIEWER: Alright. As a result of the discussion you had with a provider, did you agree to try self-injection?

RESPONDENT: mmmh [yes].

INTERVIEWER: You agreed?

RESPONDENT: I did.

INTERVIEWER: What influenced you to try the self-injection?

RESPONDENT: I wanted to know if I will be able to self-inject or not at home. And I proved that I couldn’t I had to do some more training.

INTERVIEWER: okay.

RESPONDENT: mmmhu.

INTERVIEWER: What were the main reasons that influenced you to say yes to try self-injecting at home?

RESPONDENT: Aaah the reasons were that I wanted to find out if I will be able to self-inject. If I will do it, I will be using that one. But I failed.

INTERVIEWER: zakanika. You failed

RESPONDENT: (Laughs), we should not force ourselves on some of these contraception.

INTERVIEWER: How exactly did you fail?

RESPONDENT: (laughs). I failed to open. They say that for it to be opened, it has to make a sound and I failed to do that. I was like I will end up destroying this thing. Let me go back doctors are good people they understand us, and I will discuss with them, we will see what we will do. And they said that some women indeed fail to do it.

Time stamp: (30:37)

INTERVIEWER: mmmhu.

RESPONDENT: mmmh.

Title: MZs_SCSI_5.docx

Descriptor Info:	District: Mzimba South	Target Population: TP3 - DMPA-SC SI clients	Respondent type: DMPA-SC SI client	Aware of SI: Yes - had heard of SI before	Coding phase: 1. Single coded	Client marital status: Married	Client age group: 20-29 years	Time since trained: N/A - client

Codes Applied:	C3.3 - Counselling / training approach for injectable users	C3.1 - Enablers, general/other	C1.1.3 - Awareness of DMPA-SC SI

Interviewer: Where did you hear about this method of self-inject?

Respondent: It was here at the health facility. I just came here to start family planning because my third child had grown a bit so I wanted to avoid another pregnancy since I have had a flesh scar from caesarean section. Of course I first heard from my friends that they are self-injecting but I did not see what it looked like. So when I came here I gave them my health passport book. Then they started calling one by one. They told us they only had self-inject where a woman self-inject on the stomach or thighs. They taught us on how to self-inject. So I accepted to get it since I had come for family planning so I self-injected and went home

Interviewer: What motivated you to get self-inject method?

Respondent: I did not have any choice because it was the only option. I took time to self-inject because I thought it was a bigger needle which the provider uses to inject. But because I didn’t have a choice, I just took it. But because I did not experience any problems, I came for the second dose, and they told us they had few in stock so we self-injected right here at the facility. The third time it was out of stock, so I was given Depo Provera

Title: MZs_SCSI_5.docx

Descriptor Info:	District: Mzimba South	Target Population: TP3 - DMPA-SC SI clients	Respondent type: DMPA-SC SI client	Aware of SI: Yes - had heard of SI before	Coding phase: 1. Single coded	Client marital status: Married	Client age group: 20-29 years	Time since trained: N/A - client

Codes Applied:	C3.1 - Enablers, general/other	C2.1.2 - Reduced trips to facility

Interviewer: I would like to know if the time you came there were other family planning methods apart from self-inject

Respondent: Yes, there were pills and condoms

Interviewer: But you chose self-inject?

Respondent: Yes

Interviewer: I would like to know why you chose self-inject over other methods of family planning

Respondent: Self-inject reduces number of facility visits. For pills I would forget to them according to prescription. For condoms I wouldn’t force my husband to use them because he denies using them saying they are for the unmarried. So the only option was to go for self-inject

Interviewer: Any other reasons?

Respondent: Pardon?

Interviewer: Was there any factor that led you choose Sayana Press?

Respondent: It was a method I liked since I couldn’t get pills and condoms
