# Supplementary material for: Health care provider and client experiences of counselling on depot medroxyprogesterone acetate subcutaneous (DMPA-SC) for self-injection in Malawi
Source: PLOS Glob Public Health. 2023 Nov 30;3(11):e0002057. doi: 10.1371/journal.pgph.0002057 (PMC10688740; doi:10.1371/journal.pgph.0002057)
Supplement: S1 Data — (ZIP) [file pgph.0002057.s001.zip › S2/C3.1.1 practice of SI in training.doc]

Title: KK_SCSI_5.docx

Descriptor Info:	District: Nkhotakota	Target Population: TP3 - DMPA-SC SI clients	Respondent type: DMPA-SC SI client	Aware of SI: Yes - had heard of SI before	Coding phase: 2. Double coded	Client marital status: Married	Client age group: 20-29 years	Time since trained: N/A - client

Codes Applied:	C3.1.1 - Practice of SI during training

INTERVIEWER: Did you get the chance to practice what you were trained?

RESPONDENT: Yes, we were given that time

INTERVIEWER: You were given the chance?

Title: MZs_SCSI_1.docx

Descriptor Info:	District: Mzimba South	Target Population: TP3 - DMPA-SC SI clients	Respondent type: DMPA-SC SI client	Aware of SI: Yes - had heard of SI before	Coding phase: 1. Single coded	Client marital status: Married	Client age group: Under 20 (adolescent)	Time since trained: N/A - client

Codes Applied:	C3.1.1 - Practice of SI during training

Interviewer: Did you have a chance to practice e.g. on condom filled with sugar/sand?

Respondent: Yes, we did they gave us the first one for us to inject ourselves for them to see if we can manage to do that

Title: KK_SCSI_2.docx

Descriptor Info:	District: Nkhotakota	Target Population: TP3 - DMPA-SC SI clients	Respondent type: DMPA-SC SI client	Aware of SI: Yes - had heard of SI before	Coding phase: 1. Single coded	Client marital status: Divorced/ separated/ widowed	Client age group: 20-29 years	Time since trained: N/A - client

Codes Applied:	C3.3 - Counselling / training approach for injectable users	C3.3.2 - Good (sufficient/ non-judgemental) counselling/training	C3.1.1 - Practice of SI during training	C3.4 - Time to train clients

INTERVIEWER: Thank you. The next question is, would you please describe your experience of being trained in self injection? To start with please describe who trained you.

RESPONDET: I was trained by the HSA

INTERVIEWER: It was the HSA not so

RESPONDENT: Yes

INTERVIEWER: So would you please describe what has been your experience of being trained in self-injection

RESPONDENT: We were told that after injecting ourselves we need to keep the injections safe. After injecting ourselves we should put the injection in a bottle, then after finish all the dosage we should take back to the hospital as we go to collect the next injection, to show the health personnel to prove that we used it instead of keeping it and deny the opportunity for others to access the injection.

INTERVIEWER: What did the HSA explain about self-injection, I think you have already explained something on this one

RESPOINDENT : Yes

INTERVIEWER: Is there anything that you forgot? that the HSA explained to you about self-injection

RESPONDENT: How to hold it

INTERVIEWER: yes

RESPONDENT: This is how we hold Sayana (showing the interviewer using hands) then we shake it, after shaking it we press its neck and then the needle is pushed inside then we hold the place where we want to inject ourselves and then we inject the needle. The moment we rearise that the needle is inside the skin then we press the medicine until all is finished then we start pulling out the needle little by little.

INTERVIEWER: Thanks you so much this is well explained.

RESPONDENT: Thanks

INTERVIEWER: Did you feel the provider spent enough time training you?

REPONDENT: No we were trained twice

INTERVIEWER: they just trained you twice, but was it enough time?

RESPONDENT: Yes, it was enough time

INTERVIEWER: Maybe it was just, they just took some time

RESPONDENT: Yes

INTERVIEWER: Did you get the chance to practice what you were trained?

RESPONDNET: Yes

Title: KK_SCPI_4.docx

Descriptor Info:	District: Nkhotakota	Target Population: TP2 - DMPA-IM/DMPA-SC PI clients	Respondent type: DMPA-SC PI client	Aware of SI: Yes - had heard of SI before	Coding phase: 2. Double coded	Client marital status: Never married	Client age group: 20-29 years	Time since trained: N/A - client

Codes Applied:	C3.3 - Counselling / training approach for injectable users	C3.1.1 - Practice of SI during training

INTERVIEWER: Were you given an opportunity to try what you were taught?

RESPONDENT: I tried and I failed to do it.

INTERVIEWER: So you just tried on your arm, or you were given… because some were given a condom full of sugar to try. Where did you try?

RESPONDENT: On the arm

INTERVIEWER: Oh... Here right?

RESPONDENT: Yes, on the thigh

INTERVIEWER: On the thigh

RESPONDENT: mmmmh.

INTERVIEWER: mmmhu. What did you think about these lessons?

RESPONDENT: The lessons?

INTERVIEWER: Were the lessons easy or difficult?

RESPONDENT: The lessons about contraceptions?

INTERVIEWER: The lessons

RESPONDENT: The lessons are simple, could say they are, but sometimes we may say are difficult.

INTERVIEWER: They are simple, in what way?

RESPONDENT: When you get it, as long as you hand is steady and you inject yourself, you get used.

Title: KK_SCSI_3.doc

Descriptor Info:	District: Nkhotakota	Target Population: TP3 - DMPA-SC SI clients	Respondent type: DMPA-SC SI client	Aware of SI: Yes - had heard of SI before	Coding phase: 1. Single coded	Client marital status: Married	Client age group: 20-29 years	Time since trained: N/A - client

Codes Applied:	C3.3 - Counselling / training approach for injectable users	C3.3.2 - Good (sufficient/ non-judgemental) counselling/training	C3.1.1 - Practice of SI during training

INTERVIEWER: You came here right, and you were trained…the training from the HSA was all about Sayana and what it’s all about and here you were actually trained on how you can self-inject. Did you get the chance to practice on what you were trained about?

RESPONDENT: mmmmh.

INTERVIEWER: what were you doing in this practice session?

RESPONDENT: (laughs). Practicing what they were doing.

INTERVIEWER: mmmhu.

RESPONDENT: hold like this and then inject.

INTERVIEWER: Ok. Did they give you a chance to…because when others are conducting the training, they use a condom filled with sugar as a thigh or around the abdomen and a person should try on that. Did that happen?

RESPONDENT: mmmh mmmh[no].

INTERVIEWER: Did they train you using the condom or the actual thigh?

RESPONDENT: real thigh.

INTERVIEWER: yours?

RESPONDENT: yes

INTERVIEWER: it was a group session or one to one?

RESPONDENT: one to one.

INTERVIEWER: one to one?

RESPONDENT: mmmh.

INTERVIEWER: okay. When they were training you, were you judged, when I say judged do you know the meaning?

RESPONDENT: mmmh mmmh [no]

INTERVIEWER: Maybe mocking you. Did you feel any judgement during the training?

RESPONDENT: no.

INTERVIEWER: everything went well?

RESPONDENT: mmmmh.

INTERVIEWER: okay. So they gave you a chance to try self-injection and you did very well, right?

RESPONDENT: mmmmh.

Title: KK_SCSI_4.docx

Descriptor Info:	District: Nkhotakota	Target Population: TP3 - DMPA-SC SI clients	Respondent type: DMPA-SC SI client	Aware of SI: Yes - had heard of SI before	Coding phase: 2. Double coded	Client marital status: Married	Client age group: Under 20 (adolescent)	Time since trained: N/A - client

Codes Applied:	C3.3 - Counselling / training approach for injectable users	C3.1.1 - Practice of SI during training

INTERVIEWER: Alright, as we continue with our discussions; would you please describe your experience of being trained in self-injection? How did the provider describe self-injection to you?

RESPONDENT: They said get the injection tear it and shake it well and press at the middle and remove the cover in the front and then inject yourself on the thigh.

INTERVIEWER: Is that what they said?

RESPONDENT: After inject yourself then press squeeze the medicine and pull the injection slowly and release the thigh.

INTERVIEWER: Ok that’s what was explained?

RESPONDENT: Yes

INTERVIEWER: Who trained you the doctor at the facility or the HSA?

RESPONDENT: The HSA

Time stamp: (20 minutes 5 seconds)

INTERVIEWER: the HSA? Alright, did you feel the provider spent enough time training you?

RESPONDENT: No they didn’t take too much time

INTERVIEWER: They didn’t too much time, did you get the chance to practice?

RESPONDENT: yes

INTERVIEWER: You got the chance?

RESPONDENT: Yes

INTERVIEWER: For you to try and you did it?

RESPONDENT: Yes

INTERVIEWER: Please raise your voice, did you ever feel judged during the training?

RESPONDENT: (Participant not sure)

INTERVIEWER: Did you ever feel judged during the training

RESPONDENT: No

Title: ZA_SCSI_3.docx

Descriptor Info:	District: Zomba	Target Population: TP3 - DMPA-SC SI clients	Respondent type: DMPA-SC SI client	Aware of SI: Yes - had heard of SI before	Coding phase: 3. Triple coded	Client marital status: Married	Client age group: 40-49 years	Time since trained: N/A - client

Codes Applied:	C3.1.1 - Practice of SI during training	C3.1.2 - Confidence/comfort to inject after counselling/ training	C3.3 - Counselling / training approach for injectable users

Interviewer: Okay. So when did you start using the self-injection method?

Respondent: I started in 2020 after we were given the chance to learn about self-injection and I attempted it right there where it was a success so I was told that if I meet any problems, I should call the hospital. The following day after I was given the medication, the health official called me and asked me if I had come across any problems, I told her that I didn’t meet any problems. So she told me to go to the hospital so that they can give me more injections.

Title: ZA_SCSI_3.docx

Descriptor Info:	District: Zomba	Target Population: TP3 - DMPA-SC SI clients	Respondent type: DMPA-SC SI client	Aware of SI: Yes - had heard of SI before	Coding phase: 3. Triple coded	Client marital status: Married	Client age group: 40-49 years	Time since trained: N/A - client

Codes Applied:	C3.3 - Counselling / training approach for injectable users	C3.1.1 - Practice of SI during training

Interviewer: Okay. So what made you stop going to the hospital to get injected and start injecting yourself?

Respondent: We were taught how to inject ourselves; we were asked if would manage and I agreed and attempted to inject myself while the HSA was watching, she confirmed that I had done it well and could manage to do it on my own. I have been injecting myself for 2 years now.

Title: ZA_SCSI_3.docx

Descriptor Info:	District: Zomba	Target Population: TP3 - DMPA-SC SI clients	Respondent type: DMPA-SC SI client	Aware of SI: Yes - had heard of SI before	Coding phase: 3. Triple coded	Client marital status: Married	Client age group: 40-49 years	Time since trained: N/A - client

Codes Applied:	C3.1.1 - Practice of SI during training	C3.1.2 - Confidence/comfort to inject after counselling/ training	C1.2.2 - Fear of SI (idea or reality)

Interviewer: Can you explain to me your experiences when being taught the self-injection. Firstly, please explain to me what they taught you.

Respondent: They taught us and asked if we could manage. A few of us agreed and we were taken to a separate room where we were asked to try it out. Since I am left-handed, I failed to inject myself on my left arm, so I asked them to give me another option and they told me I can inject myself on my stomach. I sat down and I reached for my belly button where I injected myself and the medicine went in. so they asked me if it was going to be possible and I saw that I would do it. Some were really afraid if they would be able to do it, some thought that by injecting on the stomach they would accidentally inject the intestines. But I saw that the way I had injected myself, I didn’t reach my intestines at all.

Title: ZA_SCSI_3.docx

Descriptor Info:	District: Zomba	Target Population: TP3 - DMPA-SC SI clients	Respondent type: DMPA-SC SI client	Aware of SI: Yes - had heard of SI before	Coding phase: 3. Triple coded	Client marital status: Married	Client age group: 40-49 years	Time since trained: N/A - client

Codes Applied:	C3.1.1 - Practice of SI during training

Interviewer: Okay fine. Were you given the chance to try to inject yourselves at the hospital before you were given the injections to take home?

Respondent: Yes

Interviewer: For example, did they teach you using a condom filled with sand?

Respondent: Yes, we used a packet of sugar. And I did it, so they told me I can do it. The main problem was to squeeze the drug from the syringe it needed skill because some when pressing the injection would end up breaking the syringe. They really had to bear with us so that we could master the process of injecting ourselves.

Title: MZs_IM_6.docx

Descriptor Info:	District: Mzimba South	Target Population: TP2 - DMPA-IM/DMPA-SC PI clients	Respondent type: DMPA-IM client	Aware of SI: Yes - had heard of SI before	Coding phase: 1. Single coded	Client marital status: Married	Client age group: Under 20 (adolescent)	Time since trained: N/A - client

Codes Applied:	C3.3 - Counselling / training approach for injectable users	C3.1.1 - Practice of SI during training	C1.1.3 - Awareness of DMPA-SC SI	C5.2.3 - Lack of resources/follow up materials to aid timely re-injection	C1.2.2 - Fear of SI (idea or reality)

Interviewer: Mmmhu (agreeing) aaaah (thinking out loud) now I would like to talk about another type of type of injectable family planning method maybe you have heard of it before. It is also called Sayana Press. It is similar to intramuscular Depo only that with Sayana the woman self-inject. How you ever heard of this family planning method?

Respondent: Yes

Interviewer: Where did you hear it from?

Respondent: At the health facility

Interviewer: At the facility? after hearing that a woman will self-inject what was your reaction?

Respondent: At first, I had fears since I had never used before

Interviewer: When you first heard about it. What specific information did you hear about it?

Respondent: They explained to us

Interviewer: What did they say specifically?

Respondent: I heard that there was a new injectable family planning whereby a woman will self-inject, and they taught us how they self-inject

Interviewer: Do you know anyone who uses this method?

Respondent: Eeeh (agreeing) there are some I know)

Interviewer: Aaah (thinking out loud) have you ever talked with the provider on this new method of self-inject?

Respondent: No I haven’t talked to them about it

Interviewer: Have you ever talked to them in previous visits?

Respondent: No I haven’t talked to them on self-inject

Interviewer: Haven’t you talked to them on self-inject?

Respondent: Mmmmh (denying)

Interviewer: But at first, I asked you said you have ever heard about self-inject from the providers

Respondent: I thought you said me asking them. But they really talked about it but I have never used self-inject I use intramuscular Depo

Interviewer: How many times did he talk about it?

Respondent: A couple of times

Interviewer: Did they mention it when you come for family planning services or anytime you visit the health facility

Respondent: When I come for family planning clinic

Interviewer: So would you tell me what was discussed with the provider in detail what you discussed about Sayana Press?

Respondent: They told us that this method is good because you use it at home there in no need to frequently visit the health facility so long as they teach us on how to do it at home

Interviewer: Mmmh (agreeing) did they ever mentioned on the pros and cons of using Sayana Press?

Respondent: The advantages is what I have already said. Overall they said it does not have any cons

Interviewer: They said no cons for using it? Did you feel judged when they were teaching you?

Respondent: Judged?

Interviewer: Yes, like being rude to you

Respondent: No

Interviewer: After they taught you about Sayana Press that a woman will be required to self-inject, aaaah (thinking out loud) did you ever agreed to self-inject at home?

Respondent: Yes

Interviewer: Mmmmhu (agreeing) what made you agree to use it

Respondent: Because I’ve seen that all methods are safe so that’s why I agreed to try it

Interviewer: What else motivated you?

Respondent: Another factor is that I was motivated by my neighbor who also uses it

Interviewer: Mmmh (agreeing) ….aaaaah (thinking out loud) have you ever used this method of self-inject?

Respondent: No I haven’t

Interviewer: You haven’t used it before

Respondent: No

Interviewer: Would you tell me your experiences during the lessons on Sayana Press by the providers. What were the lessons like on this method of self-inject?

Respondent: Quite moment …seemed not to have understood the question

Interviewer: Did they spend enough time with you

Respondent: Yes, they spent enough time

Interviewer: Did they give you a chance to practice

Respondent: Yes, they did

Interviewer: Did you self-inject? What did you use to practice?

Respondent: They gave us a chance like me I practiced on my body

Interviewer: The lessons which you got would you describe it? Was it easy or difficult?

Respondent: It was not easy

Interviewer: It wasn’t easy?

Respondent: Yes, especially to self-inject. It was the first experience, so it was not easy

Interviewer: Mmmhu (agreeing) after self-injecting, what else did they give you to take home

Respondent: They gave us condoms

Interviewer: What else were you given?

Respondent: Sayana

Interviewer: How many doses?

Respondent: They gave us 2 doses for self-injection

Interviewer: What else were you given?

Respondent: That’s all

Title: KK_IM_7.docx

Descriptor Info:	District: Nkhotakota	Target Population: TP2 - DMPA-IM/DMPA-SC PI clients	Respondent type: DMPA-IM client	Aware of SI: Yes - had heard of SI before	Coding phase: 1. Single coded	Client marital status: Married	Client age group: 30-39 years	Time since trained: N/A - client

Codes Applied:	C3.1.1 - Practice of SI during training

INTERVIEWER: Okay. Did you get a chance to practice what they were training you? Did they give a chance to practice how to self-inject?

RESPONDENT: Yes, they gave me a chance to practice. they were hold my hand and say do like this, and I would do that. And after that we squeeze here and inject on the area around the belly button.

INTERVIEWER: When others are training women, they use a condom filled with sugar. Did they use the condom or the real thigh?

RESPONDENT: Mmmh, they did not.

Title: MZs_SCSI_5.docx

Descriptor Info:	District: Mzimba South	Target Population: TP3 - DMPA-SC SI clients	Respondent type: DMPA-SC SI client	Aware of SI: Yes - had heard of SI before	Coding phase: 1. Single coded	Client marital status: Married	Client age group: 20-29 years	Time since trained: N/A - client

Codes Applied:	C3.1.1 - Practice of SI during training	C2.2.1 - Stockouts/stocks near expiry dates

Interviewer: Did you have any fears after being told that you will self-inject?

Respondent: What kind of fears?

Interviewer: Did you have fears after being told to self-inject?

Respondent: I did not have fears because I was self-injecting under direct observation by the provider. But they did not give us to take home because they had limited stock. I have never self-injected alone it was under direct observation by the provider

Title: KK_SCSI_1.docx

Descriptor Info:	District: Nkhotakota	Target Population: TP3 - DMPA-SC SI clients	Respondent type: DMPA-SC SI client	Aware of SI: Yes - had heard of SI before	Coding phase: 3. Triple coded	Client marital status: Never married	Client age group: Under 20 (adolescent)	Time since trained: N/A - client

Codes Applied:	C3.1.1 - Practice of SI during training

INTERVIEWER: Mmmhu. Did you get the chance to practice…practice on what you were trained about?

RESPONDENT: Yes, I was given a chance to practice because they gave us…it was a group, right, and for everyone who went to be assisted with this method, they said that everyone should do what a friend is doing close by is doing so that you should learn because once you learn, you will be able to self-inject at home. After practicing, I found myself that I was able to self-inject.

INTERVIEWER: What were you practicing on?

RESPONDENT: Because everyone wants to inject…when training us, everyone has to practice on a thi…on her thigh.

INTERVIEWER: Mmmmhu. Everyone practices…when they have trained you.

RESPONDENT: Mmmhu. They say that everyone should inject herself. When they trained us and showed us what should be done, they they told us to self-inject.

INTERVIEWER: okay.

RESPONDENT: mmhu.

Title: MZs_SCSI_4.docx

Descriptor Info:	District: Mzimba South	Target Population: TP3 - DMPA-SC SI clients	Respondent type: DMPA-SC SI client	Aware of SI: Yes - had heard of SI before	Coding phase: 2. Double coded	Client marital status: Married	Client age group: 20-29 years	Time since trained: N/A - client

Codes Applied:	C3.1.1 - Practice of SI during training

Interviewer: Mmmhu (agreeing) did they give you a chance to practice on how to self-inject?

Respondent: Yes, the very same day they taught us on how to self-inject it’s the same day we self-injected

Interviewer: Did they give you anything to practice on self-injecting?

Respondent: It was a day we were due for injection

Interviewer: After teaching you, did they give you anything to practice on self-injecting? Or not?

Respondent: No they did not provide us with anything we went straight to self-inject

Title: ZA_SCSI_1.docx

Descriptor Info:	District: Zomba	Target Population: TP3 - DMPA-SC SI clients	Respondent type: DMPA-SC SI client	Aware of SI: Yes - had heard of SI before	Coding phase: 1. Single coded	Client marital status: Married	Client age group: 40-49 years	Time since trained: N/A - client

Codes Applied:	C3.1.1 - Practice of SI during training

Interviewer: Were you given a chance to try it on yourself?

Respondent: Yes, we tried it and after seeing its working then we were good to go.

Title: MZs_IM_4.docx

Descriptor Info:	District: Mzimba South	Target Population: TP2 - DMPA-IM/DMPA-SC PI clients	Respondent type: DMPA-IM client	Aware of SI: Yes - had heard of SI before	Coding phase: 2. Double coded	Client marital status: Married	Client age group: 30-39 years	Time since trained: N/A - client

Codes Applied:	C3.1.1 - Practice of SI during training

Interviewer: Did you get the chance to practice?

Respondent: Yes

Interviewer: How did you practice the method?

Respondent: One woman volunteers herself that we should practice on her body

Interviewer: Did the provider gave you a condom filled with sand or sugar for you inject as part of the training session?

Respondent: Yes, he did.

Interviewer: Explain what happened?

Respondent: He took a condom and it was filled with sand

Interviewer: And after that what did he do?

Respondent: Then he took a syringe and inject the condom that was filled with sand.

Interviewer: You have said that you’re one of the women volunteered herself that you should practice on her body, so between the condom and the women which one did the provider started with?

Respondent: He started with the condom that was filled with sand and then he ended with the woman.
