# Supplementary material for: Health care provider and client experiences of counselling on depot medroxyprogesterone acetate subcutaneous (DMPA-SC) for self-injection in Malawi
Source: PLOS Glob Public Health. 2023 Nov 30;3(11):e0002057. doi: 10.1371/journal.pgph.0002057 (PMC10688740; doi:10.1371/journal.pgph.0002057)
Supplement: S1 Data — (ZIP) [file pgph.0002057.s001.zip › S2/C3.1.2 client confidence after couns.doc]

Title: KK_SCSI_5.docx

Descriptor Info:	District: Nkhotakota	Target Population: TP3 - DMPA-SC SI clients	Respondent type: DMPA-SC SI client	Aware of SI: Yes - had heard of SI before	Coding phase: 2. Double coded	Client marital status: Married	Client age group: 20-29 years	Time since trained: N/A - client

Codes Applied:	C1.2.2 - Fear of SI (idea or reality)	C3.1.2 - Confidence/comfort to inject after counselling/ training

INTERVIEWER: What concern did you have if any when you heard about self-injection? When you heard about self-injection did you have any concern?

RESPONDENT: Yes, I had concern in the first time because I have never done such a thing, but after I did is all the concerns were gone

INTERVIEWER: What concern did you have when you just heard

RESPONDENT: The concern was about fear, I was afraid, thinking can I inject myself

Title: ZA_HSA_2.docx

Descriptor Info:	District: Zomba	Target Population: TP1 - Providers/HSAs	Respondent type: HSA	Aware of SI: Yes - had heard of SI before	Coding phase: 3. Triple coded	Client marital status: N/A - provider	Client age group: N/A - provider	Time since trained: Less than a year ago

Codes Applied:	P1.2.1 - Negative provider attitudes towards SI	C1.2.2 - Fear of SI (idea or reality)	C3.1.2 - Confidence/comfort to inject after counselling/ training

Interviewer: Ah and I wanted to know the first time that you heard about this injection that women can self-inject, did you have any concerns on the possibility of women to self-inject?

Respondent: Yes, I had concerns, it was that for most people to self-inject, even any type of injection, we tend to be scared, the same thing when at the hospital they tell you that we will give you an injection, we are scared and concerned…

Interviewer: Mmmm (Showing that one is following the conversation)

Respondent: The same concerns and fears we had them, we thought, will these women accept to self-inject? most of them are not as brave, but now these women no longer have those fears because at first, they thought the injection was painful…

Interviewer: mmm (Showing agreement)

Respondent: But after they were trained and they started using the injection, they appreciated it a lot.

Title: ZA_HSA_2.docx

Descriptor Info:	District: Zomba	Target Population: TP1 - Providers/HSAs	Respondent type: HSA	Aware of SI: Yes - had heard of SI before	Coding phase: 3. Triple coded	Client marital status: N/A - provider	Client age group: N/A - provider	Time since trained: Less than a year ago

Codes Applied:	C1.2.2 - Fear of SI (idea or reality)	C1.1.1 - Positive client attitudes to SI	C3.1.2 - Confidence/comfort to inject after counselling/ training

Interviewer: Ah do women that you provide services to have concerns or fears on the possibility for them to self-inject?

Respondent: At first, they can have fears…

Interviewer: mmm (showing that one is following the conversation)

Respondent: … that will I manage to self-inject, then when they inject themselves, we have injected them on the first time, the second time they inject themselves…

Interviewer: mm (Showing that one is following the conversation)

Respondent: After that they become used…

Interviewer: mmmh (Showing that one is following the conversation)

Respondent: eh let me give an example, for some women they see it like it’s difficult but after we inject them and encourage them how it is supposed to be done, up to now they no longer need to be reminded about what we trained them they come back to stock up on Sayana press for their home usage.

Title: ZA_HSA_2.docx

Descriptor Info:	District: Zomba	Target Population: TP1 - Providers/HSAs	Respondent type: HSA	Aware of SI: Yes - had heard of SI before	Coding phase: 3. Triple coded	Client marital status: N/A - provider	Client age group: N/A - provider	Time since trained: Less than a year ago

Codes Applied:	A6.1.2 - Potential concerns about SI	C1.2.2 - Fear of SI (idea or reality)	C3.1.2 - Confidence/comfort to inject after counselling/ training

Interviewer: Alright, for adolescents or I should say younger women…

Respondent: mmm (showing that one is following the conversation)

Interviewer: Do they have fears when you tell them about this self-injecting contraceptive?

Respondent: When it comes to the adolescents, the fears are always there…

Interviewer: mmm (showing that one is following the conversation)

Respondent: Yes, the fears are indeed there…

Interviewer: mmm (showing that one is following the conversation)

Respondent: But when we spend some time with them in their first visit, we inject them, the second time we also inject them, because we don’t just tell them to go and inject themselves, no…

Interviewer: mmm (showing that one is following the conversation)

Respondent: But we first see the clients who have the capability that they have accepted the training, and they accept that I will be able to self-inject at home…

Interviewer: mmm (showing that one is following the conversation)

Respondent: Yes so, we still continue, even though the person has come, and we have trained them…

Interviewer: mmm (showing that one is following the conversation)

Respondent: If it’s the case that they are not satisfied that they can be able to self-inject, when they come again, we are able to tell them, when they accept that now, because some of them do things because their friends have done them…

Interviewer: mmm (showing that one is following the conversation)

Respondent: Yes, when they hear that one of their friends, I do self-inject, they ask how do you do it? So, the friends explain to them that this is how we do it, when they explain you will hear them saying that I also need to use DMPA-SC so that I should what…

Interviewer: I should self-inject.

Respondent: I should self-inject, yes because some things happen in groups, yeah…

Interviewer: mmm (showing that one is following the conversation)

Respondent: Yes, so in groups most people learn faster than when they are being trained individually because in groups, they are able to see their friends, the way that they are doing…

Title: MZs_SCSI_3.docx

Descriptor Info:	District: Mzimba South	Target Population: TP3 - DMPA-SC SI clients	Respondent type: DMPA-SC SI client	Aware of SI: Yes - had heard of SI before	Coding phase: 1. Single coded	Client marital status: Married	Client age group: 30-39 years	Time since trained: N/A - client

Codes Applied:	C3.1.2 - Confidence/comfort to inject after counselling/ training

Interviewer: would you tell me your experience with what you were taught on self-injection?

Respondent: the counselling was all well it’s just us we had fears that it cannot be possible to self-inject. Like for me I asked them to inject me because I was not confident. But I was told it was meant for self-injection. During the second visit, they counselled us again and this time I understood and managed to self-inject

Title: MZs_HSA_1.docx

Descriptor Info:	District: Mzimba South	Target Population: TP1 - Providers/HSAs	Respondent type: HSA	Aware of SI: Yes - had heard of SI before	Coding phase: 2. Double coded	Client marital status: N/A - provider	Client age group: N/A - provider	Time since trained:

Codes Applied:	P3.1.5 - Ease of training clients in SI	C1.2.2 - Fear of SI (idea or reality)	C3.1.2 - Confidence/comfort to inject after counselling/ training

Q12B. Interviewer: In your experience what is the response when you explain to clients that they could self-inject this method?

Respondent: At first, they show some resistance that they cannot manage to self-inject, but after the counselling and ask them to do it while we are watching them at least they find it simple and easy.

Title: MZs_HSA_1.docx

Descriptor Info:	District: Mzimba South	Target Population: TP1 - Providers/HSAs	Respondent type: HSA	Aware of SI: Yes - had heard of SI before	Coding phase: 2. Double coded	Client marital status: N/A - provider	Client age group: N/A - provider	Time since trained:

Codes Applied:	P3.1.5 - Ease of training clients in SI	C1.2.2 - Fear of SI (idea or reality)	C3.1.2 - Confidence/comfort to inject after counselling/ training

Interviewer: Do women have concerns about self-injection?

Respondent: At first yes, but once we have counsel them on how they can self-inject themselves they no longer have concerns.

Interviewer: Alright.

Respondent: They only have concerns before the counselling.

Title: MZs_HSA_1.docx

Descriptor Info:	District: Mzimba South	Target Population: TP1 - Providers/HSAs	Respondent type: HSA	Aware of SI: Yes - had heard of SI before	Coding phase: 2. Double coded	Client marital status: N/A - provider	Client age group: N/A - provider	Time since trained:

Codes Applied:	C1.2.1 - Negative client attitudes to SI	C3.1.2 - Confidence/comfort to inject after counselling/ training

Q13. Interviewer: How easy or difficult it is to train clients in DMPA-SC for self-injection?

Respondent: The only problem we encounter when counselling them is the resistance they show at first before self-injection but after trying they show interest, and they do like the method.

Title: MZs_HSA_1.docx

Descriptor Info:	District: Mzimba South	Target Population: TP1 - Providers/HSAs	Respondent type: HSA	Aware of SI: Yes - had heard of SI before	Coding phase: 2. Double coded	Client marital status: N/A - provider	Client age group: N/A - provider	Time since trained:

Codes Applied:	C3.1.2 - Confidence/comfort to inject after counselling/ training

Interviewer: Are women not comfortable or not confident to inject themselves?

Respondent: Women are comfortable

Title: ZA_IM_1.docx

Descriptor Info:	District: Zomba	Target Population: TP2 - DMPA-IM/DMPA-SC PI clients	Respondent type: DMPA-IM client	Aware of SI: Yes - had heard of SI before	Coding phase: 1. Single coded	Client marital status: Married	Client age group: 40-49 years	Time since trained: N/A - client

Codes Applied:	C3.3 - Counselling / training approach for injectable users	C1.2.2 - Fear of SI (idea or reality)	C3.1.2 - Confidence/comfort to inject after counselling/ training	C2.1.2 - Reduced trips to facility	C3.2.1 - Lack of SI practice during training	C3.4 - Time to train clients

Interviewer: How did you first hear about it?

Respondent: When I first heard this, I was nervous that would I be able to inject myself because we were told that we have to inject ourselves below the belly button or on the thigh. I was afraid about the belly button thinking I would inject my intestines or anything else. We were then properly taught on how to inject ourselves.

Interviewer: Mmmhh

Respondent: So ahh I accepted it and I used it.

Interviewer: Do you know anyone who is using self-injection?

Respondent: Self injection is currently out of stock, so I would not know

Interviewer: Mmmmhhh

Respondent: Yes

Interviewer: At the time it was available did you know anyone who was using it?

Respondent: Yes, I knew someone who was using it at that time

Interviewer: Alright, alright, have you spoken about DMPA-SC for self-injection with any provider?

Respondent: Yes, we have spoken about it

Interviewer: Mmmmhhh

Respondent: We were taught on how we can use the injection, how we can inject as well as the days when we are supposed to wait till the injection is ineffective in our bodies so that we can re inject the second one.

Interviewer: Can you tell me more about those conversations? How did the provider describe it to you? Tell me in detail what you were told?

Respondent: The provider explained that with this type of injection everyone is supposed to self-inject and the position is either below the belly button or on the thigh. So they explained on how to inject as well as the days we are supposed to wait for the medicine to be effective when re injecting.

Interviewer: Mmmmhhh

Respondent: They explained that when we first inject ourselves we should wait for 90days which is three months. After the 90 days we should inject and so on.

Interviewer: Mmmhhh alright, did they tell you the pros and cons of self-injection?

Respondent: They told us about the pros that sometimes people get lazy or get busy to get injected by provider. With self-injection there are no problems to inject yourself. These are the pros

Interviewer: Did they explain the cons of self-injection?

Respondent: No, they did not explain

Time stamp: 19 mins 38 secs

Interviewer: Alright thank you very much. As a result of the discussion you had with a provider, did you agree to try self-injection?

Respondent: Yes, I agreed and I tried it

Interviewer: Mmmmhhh What influenced you to try the self-injection?

Respondent: I saw that self-injection was a way that I could freely self-inject. The places where I inject are places were am free and no one is there.

Interviewer: What influenced you to self-inject?

Respondent: I was influenced with the fact that I want to family plan and avoid getting pregnant

Interviewer: Alright, alright, can you describe your experience of being trained in self-injection?

Respondent: The time they were explaining about self-injection

Interviewer: mmmhhh

Respondent: I was nervous that they are possibilities of hurting myself as well as not fully injecting due to pain as well as the medicine not getting inside. Once I fully understood I found that I accepted it and I started self-injecting without any difficulties

Interviewer: Alright, alright, in your view do you feel the provider spent enough time training you?

Respondent: Yes, the time was adequate

Interviewer: Did you get the chance to practice on what you were taught? For instance, pricking a condom filled with sugar or sand?

Respondent: No, we didn’t do that

Interviewer: Alright, did you self-inject?

Respondent: Yes

Interviewer: How can you describe the friendliness of the training you received?

Respondent: The friendliness of the training on self-injection?

Interviewer: Mmmmhhh

Respondent: It was easy because it was an easy way, it’s not like it was hard to understand when being taught. It was quite easy, and we practiced and got it.

Interviewer: Yes, what were you given to take home after your training for instance how many injectables?

Respondent: 3

Interviewer: Were you given any paper with instructions on how to self-inject at home?

Respondent: No, the just explained on how we can self-inject

Interviewer: Alright, were you given a calendar?

Respondent: Yes, we were given a calendar

Interviewer: So you got all these?

Respondent: Yes, we got them at that time

Title: KK_SCPI_6.docx

Descriptor Info:	District: Nkhotakota	Target Population: TP2 - DMPA-IM/DMPA-SC PI clients	Respondent type: DMPA-SC PI client	Aware of SI: Yes - had heard of SI before	Coding phase: 1. Single coded	Client marital status: Married	Client age group: 20-29 years	Time since trained: N/A - client

Codes Applied:	C1.2.2 - Fear of SI (idea or reality)	C3.1.2 - Confidence/comfort to inject after counselling/ training	C1.1.1 - Positive client attitudes to SI

INTERVIEWER: okay. Based on your discussions with the HSAs did you agree to try Sayana?

RESPONDENT: eee. Yes

INTERVIEWER: What motivated you to try?

RESPONDENT: The way they were saying that, it is good; you can inject yourself at home. Then some were saying may be your husbands are jealousy. In response, I said we go and give birth there at the hospital what else is an injection. I decided to just come, get the self-injective one, and go home in good time.

INTERVIEWER: mmmhu.

RESPONDENT: yes

INTERVIEWER: How was it after trying?

RESPONDENT: This time around, I did not try.

INTERVIEWER: You did not try.

RESPONDENT: Getting to inject myself.

INTERVIEWER: How about trying here at the hospital?

RESPONDENT: Oh, trying?

INTERVIEWER: mmmmh.

RESPONDENT: It was just good.

INTERVIEWER: Did you manage to inject yourself?

RESPONDENT: mmmmh.

INTERVIEWER: mmmhu?

RESPONDENT: Yes, managed just fine.

INTERVIEWER: Why did you not get and do it at home?

RESPONDENT: No, I said I would do it on the next injection.

INTERVIEWER: mmmmhu. So what happened the second time you came?

RESPONDENT: I just saw that they injected me with the other type because Sayana was out of stock.

INTERVIEWER: You mean to tell me that they gave you the opportunity, do you know the Sayana… they gave you the chance?

RESPONDENT: They just advised me, that we should be injecting ourselves with this. They said that it is possible for us to change because there were some who were injecting themselves at home. I said then I will get it the next time just inject me with the other type.

INTERVIEWER: Oh, you said so?

RESPONDENT: Yes, that is what I said.

INTERVIEWER: Okay, and today you have not tried.

RESPONDENT: I did not try but my friends were saying we will not cause trouble.

INTERVIEWER: So why did you not want to try and you chose the doctor to be injecting you?

RESPONDENT: I said just be injecting me because I do it here, next time I will get Sayana.

INTERVIEWER: I just want to know the real reason that made you refuse to try.

RESPONDENT: It was just some fear (laughs)

INTERVIEWER: Fear?

RESPONDENT: Just some fears to inject myself.

INTERVIEWER: mmmhu.

RESPONDENT: However, I got courage because of my friends who were saying that it’s very easy. Therefore I got encouraged and I was ready but because I had already been injected.

Title: KK_SCPI_6.docx

Descriptor Info:	District: Nkhotakota	Target Population: TP2 - DMPA-IM/DMPA-SC PI clients	Respondent type: DMPA-SC PI client	Aware of SI: Yes - had heard of SI before	Coding phase: 1. Single coded	Client marital status: Married	Client age group: 20-29 years	Time since trained: N/A - client

Codes Applied:	C3.3.3 - Poor (insufficient/ judgemental) counselling/training	C3.2.1 - Lack of SI practice during training	C3.2.2 - Lack of inclination or confidence to inject, even after training	C3.1.2 - Confidence/comfort to inject after counselling/ training	C1.2.2 - Fear of SI (idea or reality)

INTERVIEWER: okay. Are there any new things in the video that you have never heard about?

RESPONDENT: aaaah. No. there is nothing new.

INTERVIEWER: Nothing new?

RESPONDENT: mmmh.

INTERVIEWER: What do you think are the most important things that are in the video?

RESPONDENT: The idea to inject yourself, Sayana.

INTERVIEWER: mmmhu. What about it?

RESPONDENT: The most important thing is the injection; the way I have seen it as the woman explained that at home…when you collect three injections you will take a year before you go back for the same.

INTERVIEWER: okay. Are there things you remember to have been taught by the providers but are not in the video? Are there things that the provider or may be a nurse taught you about Sayana but are not there in the video?

RESPONDENT: Aaah no.

INTERVIEWER: All of them?

RESPONDENT: mmmh.

INTERVIEWER: What else do you think women need to know about Sayana before they start using it? What else do women need to know before they start using Sayana?

RESPONDENT: They need to know how to open the medicine, how to shake it the way they do. Yes, they need to know about that and also how to inject themselves before they collect it. They need to follow everything as it has been taught by the provider; we need to follow it before we collect Sayana.

INTERVIEWER: mmmhu. Now that you have finished watching the video, have your thoughts about Sayana changed?

RESPONDENT: Yes, they have changed.

INTERVIEWER: Changed, how?

RESPONDENT: Have changed… here they have explained that… we should say the fears I had are gone.

INTERVIEWER: okay.

RESPONDENT: I was afraid. However, the way it has been taught here about shaking and everything and what to do when you reach that day my thoughts have changed and there are no more worries.

INTERVIEWER: oh…okay. As you have watched the video, all the fears are gone?

RESPONDENT: Yes. they are gone.

INTERVIEWER: Is it different from how the nurse or HSAS taught you?

RESPONDENT: A little different.

INTERVIEWER: How little is it different?

RESPONDENT: As they were teaching, they did not remove the Sayana.

INTERVIEWER: ka…

RESPONDENT: They did not show us the injection. They were just explaining theoretically. They did not demonstrate the way it is demonstrated in the video.

INTERVIEWER: ok.

RESPONDENT: yes. They did not take the injection they were just explaining theoretically.

INTERVIEWER: okay.

RESPONDENT: eyetu. Sure.

Title: KK_SCPI_6.docx

Descriptor Info:	District: Nkhotakota	Target Population: TP2 - DMPA-IM/DMPA-SC PI clients	Respondent type: DMPA-SC PI client	Aware of SI: Yes - had heard of SI before	Coding phase: 1. Single coded	Client marital status: Married	Client age group: 20-29 years	Time since trained: N/A - client

Codes Applied:	C3.1.2 - Confidence/comfort to inject after counselling/ training	C1.2.2 - Fear of SI (idea or reality)	C2.1.2 - Reduced trips to facility	C1.1.1 - Positive client attitudes to SI

INTERVIEWER: okay. So you said the fears are gone after watching the video?

RESPONDENT: mmmh Yes, they are gone?

INTERVIEWER: You will start injecting yourself?

RESPONDENT: mmh Yes, I will be collecting.

INTERVIEWER: When are you coming again?

RESPONDENT: I will come on nineteen.

INTERVIEWER: Nineteenth of which month?

RESPONDENT: January.

INTERVIEWER: When you come in January you are going to get Sayana?

RESPONDENT: I will get. Maybe they will give me three and that will make me get settled at home without frequent visiting the hospital to be injected.

INTERVIEWER: okay.

RESPONDENT: mmmh.

INTERVIEWER: I have this question, have you tried to inject yourself with Sayana more than once? I should just say have you tried to inject yourself before?

RESPONDENT: No, I have never tried.

INTERVIEWER: You have never tried?

RESPONDENT: mmmh.

INTERVIEWER: Have they never tried to give you to try?

RESPONDENT: They gave me, but I said no.

INTERVIEWER: You refused?

RESPONDENT: mmmh.

INTERVIEWER: You said you were afraid right?

RESPONDENT: Yes, I was afraid.

INTERVIEWER: As you have watched the video, have your thoughts about Sayana changed?

RESPONDENT: mmmh.

INTERVIEWER: Yes

RESPONDENT: mmmh.

INTERVIEWER: They have completely changed?

RESPONDENT: Yes, they have changed a lot.

INTERVIEWER: the next appointment.

RESPONDENT: aaah I will say, just give me I should be going home, I should be at home in good time. Sometimes I could leave other things to do and rush to the hospital to be injected.

INTERVIEWER: okay. Will you allow to try while the doctor is watching?

RESPONDENT: Yes, I will allow.

INTERVIEWER: You will really inject yourself?

RESPONDENT: mmmh.

INTERVIEWER: All the shivering will.

RESPONDENT: Will completely go.

INTERVIEWER: All gone

RESPONDENT: mmmh.

INTERVIEWER: What will make you lose all the shivering?

RESPONDENT: The video is what has made me.

INTERVIEWER: You have really loved the video?

RESPONDENT: Aah I have loved it.

INTERVIEWER: Why?

RESPONDENT: The nurse is also good explaining everything.

Title: KK_SCPI_4.docx

Descriptor Info:	District: Nkhotakota	Target Population: TP2 - DMPA-IM/DMPA-SC PI clients	Respondent type: DMPA-SC PI client	Aware of SI: Yes - had heard of SI before	Coding phase: 2. Double coded	Client marital status: Never married	Client age group: 20-29 years	Time since trained: N/A - client

Codes Applied:	C3.1.2 - Confidence/comfort to inject after counselling/ training

INTERVIEWER: mmmhu. After watching the video, has you view of Sayana changed?

RESPONDENT: No, they have not changed.

INTERVIEWER: mmmmhu.

RESPONDENT: Will be injecting myself.

INTERVIEWER: You will be injecting yourself.

RESPONDENT: yes. This contraception is good, depending on the way I have seen it.

INTERVIEWER: You will be injecting yourself.

RESPONDENT: yes.

Title: ZA_IM_6.docx

Descriptor Info:	District: Zomba	Target Population: TP2 - DMPA-IM/DMPA-SC PI clients	Respondent type: DMPA-IM client	Aware of SI: Yes - had heard of SI before	Coding phase: 1. Single coded	Client marital status: Married	Client age group: 30-39 years	Time since trained: N/A - client

Codes Applied:	C1.2.2 - Fear of SI (idea or reality)	C3.1.2 - Confidence/comfort to inject after counselling/ training	C1.1.1 - Positive client attitudes to SI

Interviewer: Do you think the level of interest in the self-injecting method would be different among several women?

Respondent: Mmhhm yes it would be.

Interviewer: You say interests would differ among different women

Respondent: Mmhhmm

Interviewer: On the self-injecting contraception?

Respondent: Yes, it would be different.

Interviewer: So to you it is a good method, but other women would see it differently?

Respondent: Yes, others could think it is not good, but to me it good to with how you have explained it.

Interviewer: Mhhmm.

Respondent: Mhhmm

Interviewer: Why do you think other women other women would see it as a bad method?

Respondent: Because others would be fearing the self-injecting just like how I fear injecting myself eh.

Interviewer: Mhmm. Is your fear now lessened?

Respondent: yes, I could inject myself mhmm.

Interviewer: You can inject yourself?

Respondent: Mhhmm.

Interviewer: Alright.

Respondent: yes, I could inject myself since you are told how it is to be done.

Title: ZA_IM_6.docx

Descriptor Info:	District: Zomba	Target Population: TP2 - DMPA-IM/DMPA-SC PI clients	Respondent type: DMPA-IM client	Aware of SI: Yes - had heard of SI before	Coding phase: 1. Single coded	Client marital status: Married	Client age group: 30-39 years	Time since trained: N/A - client

Codes Applied:	C3.1.2 - Confidence/comfort to inject after counselling/ training	C1.1.1 - Positive client attitudes to SI

Interviewer Yes, they tell you everything it’s not like they just give it to you to go and just inject yourself at home. They teach you and then give you to try it out while the nurse is right there.

Respondent: Mhhmm

Interviewer When going back home it means…

Respondent: You have self-injected!

Interviewer: You have self-injected, yes!

Respondent: You have self-injected mmhhmm

Interviewer: And the other you will self-inject alone. So what else do you think women would like to know before ummhm about the method before opting for it?

Respondent: Before choosing to go for it!

Interviewer: Mhhmm what do think women would want to know? For example, you, after hearing of the injection method.

Respondent: contraception!

Interviewer: Yes, what would you really want to know at first?

Respondent: I would like to know it after being taught very well

Interviewer: Mhhmm

Respondent: Just like how you have told me.

Interviewer: Mhhmm

Respondent: Mmmhhm since the others just talk.

Interviewer: Mmmhhm

Respondent: They did not explain mhhmm

Interviewer: So what exactly would women want to know?

Respondent: They would like to know why you have to self-inject.

Interviewer: Eh! Alright is there any additional thing you would like to say?

Respondent: No, there isn’t.

Interviewer: Mmmhmm Alright, so now I have reached to the questions I had today, so do you have anything you would love to share on what we have talked about?

Respondent: No there isn’t, you have already explained a lot.

Interviewer: Eh! Any questions?

Respondent: No there isn’t, I understand.

Title: KK_SCSI_3.doc

Descriptor Info:	District: Nkhotakota	Target Population: TP3 - DMPA-SC SI clients	Respondent type: DMPA-SC SI client	Aware of SI: Yes - had heard of SI before	Coding phase: 1. Single coded	Client marital status: Married	Client age group: 20-29 years	Time since trained: N/A - client

Codes Applied:	C3.1.2 - Confidence/comfort to inject after counselling/ training

INTERVIEWER: okay. So they gave you a chance to try self-injection and you did very well, right?

RESPONDENT: mmmmh.

Title: KK_SCSI_3.doc

Descriptor Info:	District: Nkhotakota	Target Population: TP3 - DMPA-SC SI clients	Respondent type: DMPA-SC SI client	Aware of SI: Yes - had heard of SI before	Coding phase: 1. Single coded	Client marital status: Married	Client age group: 20-29 years	Time since trained: N/A - client

Codes Applied:	C3.1.2 - Confidence/comfort to inject after counselling/ training

INTERVIEWER: If there was no stock out, would you have any concerns of fears about doing your first self-injection at home?

RESPONDENT: no.

INTERVIEWER: why?

RESPONDENT: Because I was trained in every aspect

INTERVIEWER: mmhu.

RESPONDENT: yes.

Title: KK_SCSI_4.docx

Descriptor Info:	District: Nkhotakota	Target Population: TP3 - DMPA-SC SI clients	Respondent type: DMPA-SC SI client	Aware of SI: Yes - had heard of SI before	Coding phase: 2. Double coded	Client marital status: Married	Client age group: Under 20 (adolescent)	Time since trained: N/A - client

Codes Applied:	C1.1.1 - Positive client attitudes to SI	C1.1.3 - Awareness of DMPA-SC SI	C3.1.2 - Confidence/comfort to inject after counselling/ training

INTERVIEWER: Where did you hear first about contraceptive method that you could inject yourself? where did you hear?

RESPONDENT: At ante- natal clinic

INTERVIEWER: Alright that means it was where you do ante- natal clinic

RESPONDNET: At Thundu

INTERVIEWER: Is it at the community or at facility?

RESPONDENT: That were we usually have ante- natal clinics

INTERVIEWER: Alright, so ante-natal clinics are conducted here? So you heard from the doctor or HSA

RESPONDENT: From the HSA

INTERVIEWER: So firstly you heard from the HSA?

RESPONDENT: yes

INTERVIEWER: What was your reaction and what did you do?

RESPONDENT: When I first heard, I was told that we should go to the hospital for practical. When I watched some of my friends doing it, I thought to myself, I should take the self-injection one, this provider injection will just waste my time

INTERVIEWER: ok fine, fine. So what appealed you about the ability to self-inject?

RESPONDENT: It’s that painful, the self-inject one

INTERVIEWER: Self –injection is not that painful?

RESPONDENT: Yes

INTERVIEWER: Its different form the on administered by the provider

RESPONDENT: Yes

INTERVIEWER: What appealed the most?

RESPONDENT: Nothing

INTERVIEWER: Alight you said nothing, so should we say that what appealed is that is less painful.

RESPONDENT: (respondent just laugh)

INTERVIEWER: ok may be at some point you may remember something. What concern did you have if any?

RESPONDENT: Nothing

INTERVIEWER: Firstly when you heard there is a contraceptive for self-injection, did you have any concern?

RESPONDENT: No, I heard from my friend that there is this self-injection contraceptive that is less painful than the one we get injected by the provider

INTERVIEWER: Ok so you

RESPONDENT: Will you not wait for me? [An interruption respondent was talking to her friends to wait for her.]

INTERVIEWER: So we were discussing that you hear from the HSA?

RESPONDENT: Yes

INTERVIEWER: So when you heard from the HSA you did not have any concern?

RESPONDENT: Yes, I did not have any concern, when I was told how we should do it, I just held the injection and did it.

Title: KK_SCSI_4.docx

Descriptor Info:	District: Nkhotakota	Target Population: TP3 - DMPA-SC SI clients	Respondent type: DMPA-SC SI client	Aware of SI: Yes - had heard of SI before	Coding phase: 2. Double coded	Client marital status: Married	Client age group: Under 20 (adolescent)	Time since trained: N/A - client

Codes Applied:	C3.1.2 - Confidence/comfort to inject after counselling/ training

INTERVIEWER: cay you describe your experience giving your first self-injection at home?

RESPONDENT: I experienced nothing

INTERVIEWER: You did not have any experience? What about any concern when you first used the injection?

RESPONDENT: No I was not afraid

INTERVIEWER: You have no concerns?

RESPONDENT: Yes

Title: ZA_SCSI_3.docx

Descriptor Info:	District: Zomba	Target Population: TP3 - DMPA-SC SI clients	Respondent type: DMPA-SC SI client	Aware of SI: Yes - had heard of SI before	Coding phase: 3. Triple coded	Client marital status: Married	Client age group: 40-49 years	Time since trained: N/A - client

Codes Applied:	C3.1.1 - Practice of SI during training	C3.1.2 - Confidence/comfort to inject after counselling/ training	C3.3 - Counselling / training approach for injectable users

Interviewer: Okay. So when did you start using the self-injection method?

Respondent: I started in 2020 after we were given the chance to learn about self-injection and I attempted it right there where it was a success so I was told that if I meet any problems, I should call the hospital. The following day after I was given the medication, the health official called me and asked me if I had come across any problems, I told her that I didn’t meet any problems. So she told me to go to the hospital so that they can give me more injections.

Title: ZA_SCSI_3.docx

Descriptor Info:	District: Zomba	Target Population: TP3 - DMPA-SC SI clients	Respondent type: DMPA-SC SI client	Aware of SI: Yes - had heard of SI before	Coding phase: 3. Triple coded	Client marital status: Married	Client age group: 40-49 years	Time since trained: N/A - client

Codes Applied:	C3.1.2 - Confidence/comfort to inject after counselling/ training	C1.1.1 - Positive client attitudes to SI

Interviewer: What interested you about the ability to inject yourself?

Respondent: I simply liked the idea because I just wanted to attempt to inject myself to see what it would be like and when I did it was successful, so I saw it was possible to inject myself and I have been doing so ever since.

Interviewer: Were you not worried or afraid in any way?

Respondent: No.

Interviewer: Are you sure?

Respondent: Yes, we were taught so I wasn’t afraid.

Interviewer: Other women are afraid to do it, weren’t you?

Respondent: No. I knew that if something goes wrong, I will follow up with the health officials

Title: ZA_SCSI_3.docx

Descriptor Info:	District: Zomba	Target Population: TP3 - DMPA-SC SI clients	Respondent type: DMPA-SC SI client	Aware of SI: Yes - had heard of SI before	Coding phase: 3. Triple coded	Client marital status: Married	Client age group: 40-49 years	Time since trained: N/A - client

Codes Applied:	C3.1.1 - Practice of SI during training	C3.1.2 - Confidence/comfort to inject after counselling/ training	C1.2.2 - Fear of SI (idea or reality)

Interviewer: Can you explain to me your experiences when being taught the self-injection. Firstly, please explain to me what they taught you.

Respondent: They taught us and asked if we could manage. A few of us agreed and we were taken to a separate room where we were asked to try it out. Since I am left-handed, I failed to inject myself on my left arm, so I asked them to give me another option and they told me I can inject myself on my stomach. I sat down and I reached for my belly button where I injected myself and the medicine went in. so they asked me if it was going to be possible and I saw that I would do it. Some were really afraid if they would be able to do it, some thought that by injecting on the stomach they would accidentally inject the intestines. But I saw that the way I had injected myself, I didn’t reach my intestines at all.

Title: ZA_SCSI_3.docx

Descriptor Info:	District: Zomba	Target Population: TP3 - DMPA-SC SI clients	Respondent type: DMPA-SC SI client	Aware of SI: Yes - had heard of SI before	Coding phase: 3. Triple coded	Client marital status: Married	Client age group: 40-49 years	Time since trained: N/A - client

Codes Applied:	C1.2.2 - Fear of SI (idea or reality)	C3.1.2 - Confidence/comfort to inject after counselling/ training

Interviewer: Can you explain to me what you experienced when you injected yourself for the first time?

Respondent: I was afraid somehow. I had assured the nurses that I would do it, but I didn’t know if I was going to be able to do it. I attempted to inject myself on the thigh, I felt pain, but I accepted it and when I finished my leg was hurting so the next time, I decided to inject myself on the stomach and when I did it, I did not encounter any problems, so I’ve been injecting myself on the stomach since then.

Interviewer: So how did you deal with these fears?

Respondent: I just strengthened myself and removed all fear because I knew that going to the hospital to look for family planning methods gives unnecessary busy-ness. Sometimes I have to explain to people where am going with my child who isn’t sick. It gets uncomfortable to explain to people that am going to get family planning but when I have the injection at home, I don’t have to explain to anyone that am doing family planning.

Title: MZs_HSA_4.docx

Descriptor Info:	District: Mzimba South	Target Population: TP1 - Providers/HSAs	Respondent type: HSA	Aware of SI: Yes - had heard of SI before	Coding phase: 1. Single coded	Client marital status: N/A - provider	Client age group: N/A - provider	Time since trained:

Codes Applied:	C1.1.2 - Baseline correct knowledge/ awareness of injectables	C3.1.2 - Confidence/comfort to inject after counselling/ training	C1.2.2 - Fear of SI (idea or reality)

Interviewer: you have talked about the general overview above, but specifically what do clients say about self-injection?

Respondent: at first the clients are afraid but when they have been taught the say that the method is good, and the needle is not painful as compared to the Depo IM. as such most clients recommend Sayana.

Title: ZA_FAC_2.docx

Descriptor Info:	District: Zomba	Target Population: TP1 - Providers/HSAs	Respondent type: Facility-based provider	Aware of SI: Yes - had heard of SI before	Coding phase: 3. Triple coded	Client marital status: N/A - provider	Client age group: N/A - provider	Time since trained: Less than a year ago

Codes Applied:	C3.1.2 - Confidence/comfort to inject after counselling/ training	P4.1.3 - Provider availability/ willingness to repeat SI training	P1.2.1 - Negative provider attitudes towards SI	P3.1.2 - Provider skill/ability to effectively counsel on SI

Interviewer: Do women have any fears concerning their ability to apply the drug themselves?

Respondent: Yes, some are worried. They say, “Will I manage this? Will I not get pregnant? What if I get lost when administering the drug?” these things make us providers ask questions like, “will these people really manage to do this on their own since they are doubting?”, “Do they really qualify to be given the drugs so as to administer them on their own?”. So here is where we sense this. But when the person seems to understand and is able to explain the things well, then we’re sure that this person will do it properly and it will be effective.

Interviewer: So how do you deal with these worries? I’ve shown you that am worried and unable to administer the self-injection on my own, how do you deal with it?

Respondent: We re-do the teaching and try to find out why the fears of self-injecting are coming? So they respond, for example, “the way the bottle is supposed to be held..., or maybe on the stomach the place where am supposed to inject how do I do it?”. So we re-explain the process and how they’re supposed to do it. We ask, “is it clear?”, “is there a question?”

Title: ZA_SCSI_5.docx

Descriptor Info:	District: Zomba	Target Population: TP3 - DMPA-SC SI clients	Respondent type: DMPA-SC SI client	Aware of SI: Yes - had heard of SI before	Coding phase: 3. Triple coded	Client marital status: Married	Client age group: 30-39 years	Time since trained: N/A - client

Codes Applied:	C3.1.2 - Confidence/comfort to inject after counselling/ training

Interviewer: Ah did you have any concerns when you heard about this that you can self-inject?

Respondent: No I did not have any concerns.

Interviewer: Mmm (showing that one is following the conversation)

Respondent: Because when they were telling us that we should self-inject below the navel, we followed the same advice

Title: ZA_SCSI_5.docx

Descriptor Info:	District: Zomba	Target Population: TP3 - DMPA-SC SI clients	Respondent type: DMPA-SC SI client	Aware of SI: Yes - had heard of SI before	Coding phase: 3. Triple coded	Client marital status: Married	Client age group: 30-39 years	Time since trained: N/A - client

Codes Applied:	C3.3.2 - Good (sufficient/ non-judgemental) counselling/training	C3.1.2 - Confidence/comfort to inject after counselling/ training

Interviewer: Did you feel judged when you were being trained?

Respondent: No they just trained us like that

Interviewer: Mmm (showing that one is following the conversation)

Respondent: They trained us, and we got home, and we were able to do what we were taught and we saw that it worked.

Interviewer: Alright.

Respondent: So when I came here, I just told them that what you trained me I have been able to do

Title: ZA_SCSI_5.docx

Descriptor Info:	District: Zomba	Target Population: TP3 - DMPA-SC SI clients	Respondent type: DMPA-SC SI client	Aware of SI: Yes - had heard of SI before	Coding phase: 3. Triple coded	Client marital status: Married	Client age group: 30-39 years	Time since trained: N/A - client

Codes Applied:	C1.2.2 - Fear of SI (idea or reality)	C3.1.2 - Confidence/comfort to inject after counselling/ training

Interviewer: Alright, ah can you tell me your experiences the first time of using DMPA SC for self-injection?

Respondent: No I did not face any problems.

Interviewer: Alright.

Respondent: Eh

Interviewer: Alright, did you have any concerns on the first time that you used the injection?

Respondent: Yes, I had concerns and fears on how it will all end.

Interviewer: Mmm (showing that one is following the conversation)

Respondent: But even though I was scared I saw that it was okay.

Title: ZA_SCSI_5.docx

Descriptor Info:	District: Zomba	Target Population: TP3 - DMPA-SC SI clients	Respondent type: DMPA-SC SI client	Aware of SI: Yes - had heard of SI before	Coding phase: 3. Triple coded	Client marital status: Married	Client age group: 30-39 years	Time since trained: N/A - client

Codes Applied:	C1.1.1 - Positive client attitudes to SI	C1.2.2 - Fear of SI (idea or reality)	C5.1.2 - Clients correctly administering SI at home	C88 - Client great quotes	C3.1.2 - Confidence/comfort to inject after counselling/ training

Interviewer: Alright, ah do you think women in your community would be interested in DMPA-SC?

Respondent: Yes, they have interest

Interviewer: Alright

Respondent: When they got home and self-injected, they said that we were just scared of nothing

Interviewer: Mmm (showing that one is following the conversation)

Respondent: But it is good just like you explained to me

Interviewer: Mmm (showing that one is following the conversation)

Respondent: At the beginning we used to be sacred maybe the hand was just shaking, but when we found out that the medicine has ended in the tube, we did it properly without fear the second time.

Title: MZs_HSA_3.docx

Descriptor Info:	District: Mzimba South	Target Population: TP1 - Providers/HSAs	Respondent type: HSA	Aware of SI: Yes - had heard of SI before	Coding phase: 3. Triple coded	Client marital status: N/A - provider	Client age group: N/A - provider	Time since trained: Less than a year ago

Codes Applied:	C1.2.1 - Negative client attitudes to SI	C3.1.2 - Confidence/comfort to inject after counselling/ training	P88 - Provider great quotes	P3.3.2 - Inclusion of practice opportunities for clients in training

Q.12b Interviewer: what, in your experience is the response when you explain to clients that they could self-inject this method?

Respondent: most of them they refuse at first they say we don’t know then you say although you don’t know but we can explain to you I tell you what to do at the end you will know what to do. So you start explaining to her you show that chart she looks at that one you move together now we are here now we are here now you can get it. Can you do this one? So you do it she says no I will just do at home but no without practicing you cannot do at home but after I practice here you get it here you will do it at home so those who are unable to do what told them they fail to give themselves even if you can talk to them they will say aaah no aah ahh just give me I will come back and get it here. But those who can catch up early they say aaah let me do. After doing they say aah its simple I will go.

Title: MZs_SCSI_2.docx

Descriptor Info:	District: Mzimba South	Target Population: TP3 - DMPA-SC SI clients	Respondent type: DMPA-SC SI client	Aware of SI: Yes - had heard of SI before	Coding phase: 3. Triple coded	Client marital status: Married	Client age group: Under 20 (adolescent)	Time since trained: N/A - client

Codes Applied:	C3.1.2 - Confidence/comfort to inject after counselling/ training	C3.1 - Enablers, general/other	C1.1.1 - Positive client attitudes to SI

Interviewer: What were the most important factors that motivating you to try?

Respondent: The only thing that made me to try this method of self-injection, it was after seeing my friends doing it and they were also saying that there is no effect, and it is better than the Depo, cause with Depo sometimes the syringe hurts because of the hands of the person who is doing it, but with this one there is nothing like that, thus why I wanted to try this self-injection.

Interviewer: Has your perspective changed over time?

Respondent: Yes, my perspective has changed and all the concerns about the injection that I had are no longer and I do not have any worry about this self-injection.

Title: KK_IM_7.docx

Descriptor Info:	District: Nkhotakota	Target Population: TP2 - DMPA-IM/DMPA-SC PI clients	Respondent type: DMPA-IM client	Aware of SI: Yes - had heard of SI before	Coding phase: 1. Single coded	Client marital status: Married	Client age group: 30-39 years	Time since trained: N/A - client

Codes Applied:	C3.1.2 - Confidence/comfort to inject after counselling/ training

INTERVIEWER: Back then, you tried self-injecting and you failed right? Do you now feel more positive or negative about self-injection? Will you be able to overcome what made you fail to self-inject that time?

RESPONDENT: Aaah I feel more positive because as I have watched here, they have said that when you get the Sayana, you will be doing your daily work at home as you will be self-injecting.

INTERVIEWER: okay.

RESPONDENT: Yes. I feel that it is good.

Title: KK_IM_7.docx

Descriptor Info:	District: Nkhotakota	Target Population: TP2 - DMPA-IM/DMPA-SC PI clients	Respondent type: DMPA-IM client	Aware of SI: Yes - had heard of SI before	Coding phase: 1. Single coded	Client marital status: Married	Client age group: 30-39 years	Time since trained: N/A - client

Codes Applied:	C3.1.2 - Confidence/comfort to inject after counselling/ training

INTERVIEWER: Back then, you tried self-injecting and you failed right? Do you now feel more positive or negative about self-injection? Will you be able to overcome what made you fail to self-inject that time?

RESPONDENT: Aaah I feel more positive because as I have watched here, they have said that when you get the Sayana, you will be doing your daily work at home as you will be self-injecting.

INTERVIEWER: okay.

RESPONDENT: Yes. I feel that it is good.

INTERVIEWER: What exactly made you fail to self-inject at home?

RESPONDENT: (laughs), fear.

INTERVIEWER: Fear?

RESPONDENT: I was afraid (laughs).

INTERVIEWER: After watching the video, are you still afraid?

RESPONDENT: Aaa no, I’ve seen that it is not difficult.

INTERVIEWER: So what are you going to do?

RESPONDENT: (laughing) aaah, I can do it.

INTERVIEWER: mmmhu.

RESPONDENT: mmmh.

INTERVIEWER: Mmm so what are you going to do? Mmmmm.

RESPONDENT: I will change to self-injection. I will get Sayana

Time stamp: (50:00)

INTERVIEWER: mmmhu.

RESPONDENT: mmmh.

INTERVIEWER: On your next visit?

RESPONDENT: aaah [yes]

INTERVIEWER: The fear is really gone?

RESPONDENT: (laughing)

INTERVIEWER: It’s gone?

RESPONDENT: Ah [yes], I feel it’s really gone.

INTERVIEWER: When is your next visit?

RESPONDENT: They say on 20th January.

INTERVIEWER: So in January you will…you will change the method?

RESPONDENT: mmmmh.

INTERVIEWER: For provider injected to self-injection?

RESPONDENT: I now understand that provider injection and self-injection is the same. People explain that they have the same amount of drugs. It’s because we are women, and we were afraid. But people on self-injection say that the amount of drugs are the same

INTERVIEWER: Mmmhu. Which means in January you will switch?

RESPONDENT: Aaah, I am changing in January.

INTERVIEWER: You are not changing your mind?

RESPONDENT: No. I will get Sayana

INTERVIEWER: What will encourage you this time around?

RESPONDENT: What will encourage me this time around?

INTERVIEWER: mmmh.

RESPONDENT: Because what I have seen in the video and what the provider was telling me is the same. And now I have the courage to do it.

INTERVIEWER: okay.

RESPONDENT: I will, I will

Title: KK_IM_7.docx

Descriptor Info:	District: Nkhotakota	Target Population: TP2 - DMPA-IM/DMPA-SC PI clients	Respondent type: DMPA-IM client	Aware of SI: Yes - had heard of SI before	Coding phase: 1. Single coded	Client marital status: Married	Client age group: 30-39 years	Time since trained: N/A - client

Codes Applied:	C1.1.1 - Positive client attitudes to SI	C3.1.2 - Confidence/comfort to inject after counselling/ training

INTERVIEWER: Okay, alright. We have now come to the end of the questions I had for you today. Do you have any other information you would like to share on the topics we have discussed?

RESPONDENT: Aaah, what we have discussed here is very good and important. You should not stop doing this because we learn something when you involve us. We learn what other people are doing. If we are doing something wrong, you help us to do right. Maybe when providers teach us, we go home and do it wrong like what happed to me. And with that video, I have seen that it’s not difficult and it is a good thing.

INTERVIEWER: your thoughts….

RESPONDENT: On 20th January, I will change to self-injection.

INTERVIEWER: What has encouraged you?

RESPONDENT: I am encouraged.

INTERVIEWER: What has encouraged you?

RESPONDENT: What has happened here.

INTERVIEWER: okay.

RESPONDENT: I was afraid, I thought it could kill me or I had so many thoughts. But I have seen that it is a good thing.

INTERVIEWER: okay.

RESPONDENT: mmmh.

INTERVIEWER: Alright. Thank you for taking the time to participate in this study…

RESPONDENT: I should also thank you for including us people in the village because sometimes we know things through this inclusion. We know through what?

INTERVIEWER: In that way.

RESPONDENT: aha. Yes.

INTERVIEWER: Alright. I really appreciate for your inputs. I have already told you that your responses will be anonymized right, mmmhu. Do you have any final questions before we finish?

RESPONDENT: Aaaaaah, I don’t have any questions. Everything is well. With what I have seen, I should not lie, everything is good. This is what we call teaching. You have taught me new things. The nurse kept my card and told me not to go. I was afraid and he told me not to be afraid and I will be able to respond. And I have seen that it is a good thing.

INTERVIEWER: Alright. Thank you so much.

RESPONDENT: Thank you.

INTERVIEWER: zikomo. Thank you.

RESPONDENT: mmmh.

Title: ZA_IM_5.docx

Descriptor Info:	District: Zomba	Target Population: TP2 - DMPA-IM/DMPA-SC PI clients	Respondent type: DMPA-IM client	Aware of SI: Yes - had heard of SI before	Coding phase: 2. Double coded	Client marital status: Married	Client age group: 30-39 years	Time since trained: N/A - client

Codes Applied:	C3.1.2 - Confidence/comfort to inject after counselling/ training

Respondent: But still after they encouraged us like they were doing our friend in the video…

Interviewer: Mmmm (showing that one is following the conversation)

Respondent: We also gotten used.

Title: ZA_HSA_3.docx

Descriptor Info:	District: Zomba	Target Population: TP1 - Providers/HSAs	Respondent type: HSA	Aware of SI: Yes - had heard of SI before	Coding phase: 1. Single coded	Client marital status: N/A - provider	Client age group: N/A - provider	Time since trained: A year or more ago

Codes Applied:	P3.1.3 - IEC/demo materials for counselling/training	C3.1.2 - Confidence/comfort to inject after counselling/ training	P3.3.2 - Inclusion of practice opportunities for clients in training

Interviewer: Alright, Do the women feel uncomfortable to inject themselves?

Respondent: Ahhhh... Many women do not feel uncomfortable about it since we teach them enough, we also use all the available resources to teach and they inject themselves first whole we are watching them, so they don't feel uncomfortable doing it.

Interviewer: Alright alright, what about adolescents?

Respondent: Same goes for the young women because they are free and comfortable because we teach them enough and during demonstration

Title: MZs_SCSI_5.docx

Descriptor Info:	District: Mzimba South	Target Population: TP3 - DMPA-SC SI clients	Respondent type: DMPA-SC SI client	Aware of SI: Yes - had heard of SI before	Coding phase: 1. Single coded	Client marital status: Married	Client age group: 20-29 years	Time since trained: N/A - client

Codes Applied:	C3.1.2 - Confidence/comfort to inject after counselling/ training

Interviewer: Did you have fears upon hearing that you will self-inject?

Respondent: Yes, I had fears at first. But the fears went off because we were self-injecting under direct observation by the provider, they were instructing us on how to do it

Interviewer: What did you deal with the fears apart from providers being there for you?

Respondent: It’s because of the counselling they gave us. When we started the procedure, everything was going as they had taught us, so this relieved us our fears.

Title: ZA_HSA_6.docx

Descriptor Info:	District: Zomba	Target Population: TP1 - Providers/HSAs	Respondent type: HSA	Aware of SI: Yes - had heard of SI before	Coding phase: 1. Single coded	Client marital status: N/A - provider	Client age group: N/A - provider	Time since trained:

Codes Applied:	C1.2.2 - Fear of SI (idea or reality)	C3.1.2 - Confidence/comfort to inject after counselling/ training

Interviewer: Are women comfortable to self-inject?

TIME INTO THE INTERVIEW: 51 Minutes 10 Seconds

Respondent: to those that have been self-injecting, they are comfortable, most of the women that seem to be scared are just afraid for the injection to get on their body, they are not scared of the method, but they are scared of self-injecting, but it’s not like there is any problem but for them to take a syringe and self-inject they see it like some challenge.

Title: MZs_HSA_6.docx

Descriptor Info:	District: Mzimba South	Target Population: TP1 - Providers/HSAs	Respondent type: HSA	Aware of SI: Yes - had heard of SI before	Coding phase: 1. Single coded	Client marital status: N/A - provider	Client age group: N/A - provider	Time since trained: A year or more ago

Codes Applied:	C3.1.2 - Confidence/comfort to inject after counselling/ training	A6.1.1 - Reasons for potential interest in SI

Interviewer: do women have fears

Respondent: no

Interviewer: what about adolescents

Respondent: they are also free

Title: ZA_IM_9.docx

Descriptor Info:	District: Zomba	Target Population: TP2 - DMPA-IM/DMPA-SC PI clients	Respondent type: DMPA-IM client	Aware of SI: Yes - had heard of SI before	Coding phase: 1. Single coded	Client marital status: Married	Client age group: 20-29 years	Time since trained: N/A - client

Codes Applied:	C3.1.2 - Confidence/comfort to inject after counselling/ training

Interviewer: So now what has made you want to start using this method?

Respondent: What has made me is that I will not keep on coming to the hospital usually to get injected, I could be self-injecting at home.

Interviewer: Your fear is gone?

Respondent: The fear has gone.

Interviewer: How has your fear ended?

Respondent: (Smiles) Because you have given us courage.

Interviewer: Mmh.

Respondent: We will not be having a lot of fear. We could be going to get and injecting ourselves after giving us advice on how to inject ourselves.

Title: KK_HSA_6.docx

Descriptor Info:	District: Nkhotakota	Target Population: TP1 - Providers/HSAs	Respondent type: HSA	Aware of SI: Yes - had heard of SI before	Coding phase: 1. Single coded	Client marital status: N/A - provider	Client age group: N/A - provider	Time since trained: A year or more ago

Codes Applied:	P1.1.1 - Positive provider attitudes towards SI	A6.1.2 - Potential concerns about SI	C3.1.2 - Confidence/comfort to inject after counselling/ training

INTERVIEWER: mmmh. Did you discuss with your colleagues about this when you first heard?

RESPONDENT: We discussed with my colleagues in the community if it was going to work. We had opinions and one of them was that it is possible depending on how we will be trained and how we will be training the women. And then we saw that it worked and people were self-injecting. It’s only a few women maybe a young mother of 16 or 17, are the ones when trained, they still remain afraid. But the majority responds responds positively when trained.

Title: KK_HSA_6.docx

Descriptor Info:	District: Nkhotakota	Target Population: TP1 - Providers/HSAs	Respondent type: HSA	Aware of SI: Yes - had heard of SI before	Coding phase: 1. Single coded	Client marital status: N/A - provider	Client age group: N/A - provider	Time since trained: A year or more ago

Codes Applied:	C3.1.2 - Confidence/comfort to inject after counselling/ training

RESPONDENT: No, injecting myself, no. but now, they are used to it. When you train them properly, it is not an issue. Maybe they will be afraid for a day, but when they are used, they self-inject without any problem. But on the first day, they are afraid.

INTERVIEWER: ok.

RESPONDENT: mmmhu.

Title: ZA_SCSI_4.docx

Descriptor Info:	District: Zomba	Target Population: TP3 - DMPA-SC SI clients	Respondent type: DMPA-SC SI client	Aware of SI: Yes - had heard of SI before	Coding phase: 2. Double coded	Client marital status: Married	Client age group: 30-39 years	Time since trained: N/A - client

Codes Applied:	C3.2.2 - Lack of inclination or confidence to inject, even after training	C5.2.2 - Clients incorrectly / failing to administer at home	C5.1.2 - Clients correctly administering SI at home	C3.1.2 - Confidence/comfort to inject after counselling/ training

Interviewer: Alright, ah when the covid-19 pandemic started have you seen any changes on accessing contraceptives?

Respondent: No I have not seen any changes because I have seen that it’s the same.

Interviewer: mmmm (showing that one is following the conversation)

Respondent: Even though they encouraged us to get injectables.

Interviewer: mmm (showing that one is following the conversation)

Respondent: But some fail to self-inject, they come back to the facility to have the injection administered to them. Those of us who are able to self-inject get the injectables and go home with it.

Interviewer: Alright.

Respondent: Sure.

Title: ZA_SCSI_4.docx

Descriptor Info:	District: Zomba	Target Population: TP3 - DMPA-SC SI clients	Respondent type: DMPA-SC SI client	Aware of SI: Yes - had heard of SI before	Coding phase: 2. Double coded	Client marital status: Married	Client age group: 30-39 years	Time since trained: N/A - client

Codes Applied:	C3.1.2 - Confidence/comfort to inject after counselling/ training

Respondent: they took us, we wanted to go and get the injection, so they took the injection and do whatever they do to it

Interviewer: mmmm mmm (showing that one is following the conversation)

Respondent: They told us where to get injected

Interviewer: mmm (showing that one is following the conversation)

Respondent: So when we saw like that we told them that, we can manage to do this

Interviewer: we can manage to do this.

Respondent: we van manage to do this the way that they trained us because they trained us well.

Title: ZA_SCSI_4.docx

Descriptor Info:	District: Zomba	Target Population: TP3 - DMPA-SC SI clients	Respondent type: DMPA-SC SI client	Aware of SI: Yes - had heard of SI before	Coding phase: 2. Double coded	Client marital status: Married	Client age group: 30-39 years	Time since trained: N/A - client

Codes Applied:	C3.1.2 - Confidence/comfort to inject after counselling/ training

Interviewer: Thank you, can you explain to me what you have experienced the first time using self-injection contraceptive?

Respondent: Ah My experiences like what?

Interviewer: Maybe if you had any fears on the day that you first self-injected, things like those. Or any issues that you had of the day that you first self-injected.

Respondent: I did not face any problems, but from the time that I started using the injection I don’t experience any bleeding during my menstrual period I just live

Title: MZs_FAC_1.docx

Descriptor Info:	District: Mzimba South	Target Population: TP1 - Providers/HSAs	Respondent type: Facility-based provider	Aware of SI: Yes - had heard of SI before	Coding phase: 3. Triple coded	Client marital status: N/A - provider	Client age group: N/A - provider	Time since trained: A year or more ago

Codes Applied:	C3.1.2 - Confidence/comfort to inject after counselling/ training

Interviewer: Thank you. Are women uncomfortable/not confident to inject themselves?

Respondent: No, actually they are comfortable.

Interviewer: What about the adolescents?

Respondent: They are comfortable.

Title: MZs_SCSI_4.docx

Descriptor Info:	District: Mzimba South	Target Population: TP3 - DMPA-SC SI clients	Respondent type: DMPA-SC SI client	Aware of SI: Yes - had heard of SI before	Coding phase: 2. Double coded	Client marital status: Married	Client age group: 20-29 years	Time since trained: N/A - client

Codes Applied:	C1.2.2 - Fear of SI (idea or reality)	C3.1.2 - Confidence/comfort to inject after counselling/ training

Interviewer: Did you have any fears after hearing that you will self-inject?

Respondent: Yes, I had fears that I won’t manage to prick myself I was not sure if I would manage to self-inject so I was just optimistic that I would do it.

Interviewer: Mmmhu (agreeing)

Respondent: Yes

Interviewer: What made you so confident that you would self-inject?

Respondent: After first dose which I self-injected I found out that my menstrual cycle was normal, so this made me put much effort on this method

Interviewer: Mmmhu

Respondent: Yes

Interviewer: Has your perception changed now that you are using this method of self-inject?

Respondent: No, it has not changed

Title: MZs_SCSI_4.docx

Descriptor Info:	District: Mzimba South	Target Population: TP3 - DMPA-SC SI clients	Respondent type: DMPA-SC SI client	Aware of SI: Yes - had heard of SI before	Coding phase: 2. Double coded	Client marital status: Married	Client age group: 20-29 years	Time since trained: N/A - client

Codes Applied:	C1.2.2 - Fear of SI (idea or reality)	C5.1.4 - Timely injection due to not having to rely on external factors	C3.1.2 - Confidence/comfort to inject after counselling/ training

Interviewer: Did you have any fears at first when you started using Sayana Press?

Respondent: Yes. We were afraid to prick ourselves “giggles” we would tremble

Interviewer: How did you deal with your fears?

Respondent: The lessons that they provider gave us gave me confidence. But also, I thought it was better to get Sayana Press home so that I have them in stock at home and just follow the dates without facing problems we had the time we were receiving at the health center because sometimes we would not find the provider there.

Title: MZs_SCPI_3.docx

Descriptor Info:	District: Mzimba South	Target Population: TP2 - DMPA-IM/DMPA-SC PI clients	Respondent type: DMPA-SC PI client	Aware of SI: Yes - had heard of SI before	Coding phase: 1. Single coded	Client marital status: Married	Client age group: 20-29 years	Time since trained: N/A - client

Codes Applied:	C3.1.2 - Confidence/comfort to inject after counselling/ training

Interviewer: Are you still scared?

Respondent: No, right now I don’t have any fear.

Interviewer: And if I can give you to go and do it at home, are you going to accept it?

Respondent: Yes, I can.

Interviewer: Why do you want to try now?

Respondent: Because I have been seeing the way the doctors do it.

Title: ZA_SCSI_1.docx

Descriptor Info:	District: Zomba	Target Population: TP3 - DMPA-SC SI clients	Respondent type: DMPA-SC SI client	Aware of SI: Yes - had heard of SI before	Coding phase: 1. Single coded	Client marital status: Married	Client age group: 40-49 years	Time since trained: N/A - client

Codes Applied:	C1.1.1 - Positive client attitudes to SI	C5.1.4 - Timely injection due to not having to rely on external factors	C3.1.2 - Confidence/comfort to inject after counselling/ training

Interviewer: So did you have any doubts?

Respondent: No I had no doubts actually was just happy that I have the injection

Interviewer: So what encouraged you to have a self-injection method?

Respondent: I just thought it was a good way compared to the rest.

Interviewer: So what encouraged you to inject yourself?

Respondent: What encouraged me is that one day you might hear medical assistants have moved or gone for further studies then we going to be stranded so just had to do it.

Interviewer: So has your thought changed overtime concerning your method of planning?

Respondent: Nothing has changed actually, when we started self-injections, it was all okay.

Interviewer: Nothing changed in your mind?

Respondent: No, actually the only thing that changed is us starting to inject ourselves.

Title: ZA_SCSI_1.docx

Descriptor Info:	District: Zomba	Target Population: TP3 - DMPA-SC SI clients	Respondent type: DMPA-SC SI client	Aware of SI: Yes - had heard of SI before	Coding phase: 1. Single coded	Client marital status: Married	Client age group: 40-49 years	Time since trained: N/A - client

Codes Applied:	C3.1.2 - Confidence/comfort to inject after counselling/ training

Interviewer: Yes.

Respondent: Only given one that we used to inject ourselves then told we have passed eyyyyy.

Title: ZA_SCSI_1.docx

Descriptor Info:	District: Zomba	Target Population: TP3 - DMPA-SC SI clients	Respondent type: DMPA-SC SI client	Aware of SI: Yes - had heard of SI before	Coding phase: 1. Single coded	Client marital status: Married	Client age group: 40-49 years	Time since trained: N/A - client

Codes Applied:	C3.1.2 - Confidence/comfort to inject after counselling/ training

Interviewer: They gave you two?

Respondent: Eeeeeeee maybe you might be scared but they saw that we were not afraid.

Title: ZA_SCSI_1.docx

Descriptor Info:	District: Zomba	Target Population: TP3 - DMPA-SC SI clients	Respondent type: DMPA-SC SI client	Aware of SI: Yes - had heard of SI before	Coding phase: 1. Single coded	Client marital status: Married	Client age group: 40-49 years	Time since trained: N/A - client

Codes Applied:	C3.1.2 - Confidence/comfort to inject after counselling/ training	C1.1.1 - Positive client attitudes to SI

Interviewer: Can you explain to me what you encountered the first time you started using the injection?

Respondent: They is no problem I encountered rather just had to remove the fear then it was all okay.

Interviewer: You had no doubts/fear?

Respondent: No I had no doubts.

Title: MZs_HSA_2.docx

Descriptor Info:	District: Mzimba South	Target Population: TP1 - Providers/HSAs	Respondent type: HSA	Aware of SI: Yes - had heard of SI before	Coding phase: 1. Single coded	Client marital status: N/A - provider	Client age group: N/A - provider	Time since trained: A year or more ago

Codes Applied:	C1.2.2 - Fear of SI (idea or reality)	C3.1.2 - Confidence/comfort to inject after counselling/ training

Interviewer: do women have fears to self-inject

Respondent: some yes. But most of them not because we teach them how to do it. We give them an opportunity to ask questions. some do ask but still more some are not open.
