# Supplementary material for: Health care provider and client experiences of counselling on depot medroxyprogesterone acetate subcutaneous (DMPA-SC) for self-injection in Malawi
Source: PLOS Glob Public Health. 2023 Nov 30;3(11):e0002057. doi: 10.1371/journal.pgph.0002057 (PMC10688740; doi:10.1371/journal.pgph.0002057)
Supplement: S1 Data — (ZIP) [file pgph.0002057.s001.zip › S2/C3.2 other barriers to client uptake.doc]

Title: KK_HSA_5.docx

Descriptor Info:	District: Nkhotakota	Target Population: TP1 - Providers/HSAs	Respondent type: HSA	Aware of SI: Yes - had heard of SI before	Coding phase: 1. Single coded	Client marital status: N/A - provider	Client age group: N/A - provider	Time since trained: A year or more ago

Codes Applied:	C1.2.2 - Fear of SI (idea or reality)	C3.2 - Barriers, general/other	C88 - Client great quotes

INTERVIEWER: Are women uncomfortable to inject themselves

RESPONDENT: Firstly they get uncomfortable due to fear

INTERVIEWER: Alright

RESPONDENT: Secondly, they become uncomfortable when it comes to injecting themselves because most of the providers are men, hence if they are told that the places where they have to inject themselves are a bit private ones, they become skeptical to take off their clothes and do self-injection. It takes us to remind them that we are health personnel hence we still abide by the rule of privacy, so we can nit tell anyone about anything that may be encountered during the process of administering the method. These are some of the issues that do make women uncomfortable.

INTERVIEWER: Alright

RESPONDENT: Sure

Title: ZA_SCSI_2.docx

Descriptor Info:	District: Zomba	Target Population: TP3 - DMPA-SC SI clients	Respondent type: DMPA-SC SI client	Aware of SI: Yes - had heard of SI before	Coding phase: 1. Single coded	Client marital status:	Client age group:	Time since trained: N/A - client

Codes Applied:	C3.2 - Barriers, general/other

Interviewer: Alright, How did they explain to you about DMPA-SC?

Respondent: I have forgotten some of the things.

Interviewer: You forgot?

Respondent: Yes

Interviewer: Okay

Respondent: (laughs in a shy way)
