# Supplementary material for: Health care provider and client experiences of counselling on depot medroxyprogesterone acetate subcutaneous (DMPA-SC) for self-injection in Malawi
Source: PLOS Glob Public Health. 2023 Nov 30;3(11):e0002057. doi: 10.1371/journal.pgph.0002057 (PMC10688740; doi:10.1371/journal.pgph.0002057)
Supplement: S1 Data — (ZIP) [file pgph.0002057.s001.zip › S2/C3.2.1 lack of SI practice in training.doc]

Title: KK_IM_3.docx

Descriptor Info:	District: Nkhotakota	Target Population: TP2 - DMPA-IM/DMPA-SC PI clients	Respondent type: DMPA-IM client	Aware of SI: Yes - had heard of SI before	Coding phase: 1. Single coded	Client marital status: Divorced/ separated/ widowed	Client age group: 20-29 years	Time since trained: N/A - client

Codes Applied:	C3.3.3 - Poor (insufficient/ judgemental) counselling/training	C3.2.1 - Lack of SI practice during training

INTERVIEWER: Alright, next question is: would you please describe your experience of being trained in self-injection? What was your experience? For instance, did the provider spent enough time training you?

RESPONDENT: It did not take much time; I would not say I understood the method deeply no

INTERVIEWER: You were not fully trained?

RESPONDENT: Yes, I would not say I know it 100% then I might be lying.

INTERVIEWER: You did not undergo the training fully? Did you get any chance to practice on what you were trained?

RESPONDENT: No

INTERVIEWER: you did not?

RESPONDENT: Yes

INTERVIEWER: Ok, how would you describe the friendliness of the training you received. I recall you said you did not undergo a full training? Like explaining in details on how the method works.

RESPONDENT: Yes

Title: MZs_IM_8.docx

Descriptor Info:	District: Mzimba South	Target Population: TP2 - DMPA-IM/DMPA-SC PI clients	Respondent type: DMPA-IM client	Aware of SI: Yes - had heard of SI before	Coding phase: 1. Single coded	Client marital status: Married	Client age group: 20-29 years	Time since trained: N/A - client

Codes Applied:	C3.3 - Counselling / training approach for injectable users	C3.2.1 - Lack of SI practice during training	C5.1.3 - Resources/follow up materials (e.g. calendar) to aid timely re-injection	C6 - Waste management

Interviewer: now I would like to talk about new method which is called Sayana which a woman self-inject. For those who cannot manage to self-inject the provider inject. Have you ever heard of this method of family planning?

TIME STAMP: 24:24MIN- 36: 34 MIN

Respondent: My friends once told me about the self-injection. But I had already taken Depo-IM which I did not encounter any challenges. I was just wondering how it could be because I had already taken one.

Interviewer: would you take Sayana for self-injection if they tell you that you can?

Respondent: yes, I can take depending with the counselling they can give me

Interviewer: you said you have talked about this method of self-injection from the provider. Tell me more what you said from the start to the time they made a decision to self-inject?

Respondent: when we were here, we were told that there is now a new method of family planning whereby a woman will self-inject. We were told that it was not difficult to self-inject and that we should not have fears. It would help because sometimes providers are not always available. so we will just get and keep it at a good place.

Interviewer: I would like you to tell me from the start how they counselled you?

Respondent: they told us that it is a good method. They told us the benefits of this method. Then they called us to self-inject. They assured us that it was not difficult to self-inject. But I was not comfortable to get it

Interviewer: Did they counsel you on the procedure of self-injection, on the benefits and disadvantages of using the method?

Respondent: they gave us Sayana, cotton to take home. They told us to first clean the area then shake the content. Then to press on the neck, then to pinch the skin and inject. After that then you throw the used needle into the toilet. Then sign on the date on the calendar. Then to bring the calendar to the provider as a proof that you self-injected

Interviewer: you were told to show the calendar?

Respondent: yes

Interviewer: you said after using you threw in…?

Respondent: in the toilet

Interviewer: did you accept to self-inject?

Respondent: no

Interviewer: why not?

Respondent: I thought if I get Sayana it will be difficult for me to go back to Depo-IM it would be like I mix things

Interviewer: did you not ask the provider if it was possible for you to get Sayana?

Respondent: no I did not ask

Interviewer: if they had counselled you on Sayana, would you self-inject?

Respondent: if well counselled I can

Interviewer: did the provider took enough time on counselling?

Respondent: yes, those who had questions were given a chance to ask

Interviewer: were you given a chance to practice before self-injecting?

Respondent: no we were told to pinch our skin and self-inject

Interviewer: were you not given a chance to try it on a condom before self-injecting?

Respondent: no

Interviewer: what were you given to take home after self-injecting?

Respondent: yes, they told us that those who choose to self-inject we will give them something to take home

Interviewer: what sort of things?

Respondent: 2 vials of Sayana and a calendar

Interviewer: how many vials of Sayana did they supply?

Respondent: 2 one was used to self-inject right here at the facility

Title: ZA_IM_2.docx

Descriptor Info:	District: Zomba	Target Population: TP2 - DMPA-IM/DMPA-SC PI clients	Respondent type: DMPA-IM client	Aware of SI: Yes - had heard of SI before	Coding phase: 1. Single coded	Client marital status: Married	Client age group: 30-39 years	Time since trained: N/A - client

Codes Applied:	C3.2.1 - Lack of SI practice during training

Interviewer: Alright, did the provider give you a chance to practice injecting yourself while the provider was there?

Respondent: No, they did not give me the opportunity to practice self-injection while he was there, this was because we were already on family planning.

Interviewer: Alright, did the provider do any demonstration on self-injection, for example, maybe taking an injection and injecting a condom full of sand or sugar?

Respondent: No.

Title: ZA_IM_1.docx

Descriptor Info:	District: Zomba	Target Population: TP2 - DMPA-IM/DMPA-SC PI clients	Respondent type: DMPA-IM client	Aware of SI: Yes - had heard of SI before	Coding phase: 1. Single coded	Client marital status: Married	Client age group: 40-49 years	Time since trained: N/A - client

Codes Applied:	C3.3 - Counselling / training approach for injectable users	C1.2.2 - Fear of SI (idea or reality)	C3.1.2 - Confidence/comfort to inject after counselling/ training	C2.1.2 - Reduced trips to facility	C3.2.1 - Lack of SI practice during training	C3.4 - Time to train clients

Interviewer: How did you first hear about it?

Respondent: When I first heard this, I was nervous that would I be able to inject myself because we were told that we have to inject ourselves below the belly button or on the thigh. I was afraid about the belly button thinking I would inject my intestines or anything else. We were then properly taught on how to inject ourselves.

Interviewer: Mmmhh

Respondent: So ahh I accepted it and I used it.

Interviewer: Do you know anyone who is using self-injection?

Respondent: Self injection is currently out of stock, so I would not know

Interviewer: Mmmmhhh

Respondent: Yes

Interviewer: At the time it was available did you know anyone who was using it?

Respondent: Yes, I knew someone who was using it at that time

Interviewer: Alright, alright, have you spoken about DMPA-SC for self-injection with any provider?

Respondent: Yes, we have spoken about it

Interviewer: Mmmmhhh

Respondent: We were taught on how we can use the injection, how we can inject as well as the days when we are supposed to wait till the injection is ineffective in our bodies so that we can re inject the second one.

Interviewer: Can you tell me more about those conversations? How did the provider describe it to you? Tell me in detail what you were told?

Respondent: The provider explained that with this type of injection everyone is supposed to self-inject and the position is either below the belly button or on the thigh. So they explained on how to inject as well as the days we are supposed to wait for the medicine to be effective when re injecting.

Interviewer: Mmmmhhh

Respondent: They explained that when we first inject ourselves we should wait for 90days which is three months. After the 90 days we should inject and so on.

Interviewer: Mmmhhh alright, did they tell you the pros and cons of self-injection?

Respondent: They told us about the pros that sometimes people get lazy or get busy to get injected by provider. With self-injection there are no problems to inject yourself. These are the pros

Interviewer: Did they explain the cons of self-injection?

Respondent: No, they did not explain

Time stamp: 19 mins 38 secs

Interviewer: Alright thank you very much. As a result of the discussion you had with a provider, did you agree to try self-injection?

Respondent: Yes, I agreed and I tried it

Interviewer: Mmmmhhh What influenced you to try the self-injection?

Respondent: I saw that self-injection was a way that I could freely self-inject. The places where I inject are places were am free and no one is there.

Interviewer: What influenced you to self-inject?

Respondent: I was influenced with the fact that I want to family plan and avoid getting pregnant

Interviewer: Alright, alright, can you describe your experience of being trained in self-injection?

Respondent: The time they were explaining about self-injection

Interviewer: mmmhhh

Respondent: I was nervous that they are possibilities of hurting myself as well as not fully injecting due to pain as well as the medicine not getting inside. Once I fully understood I found that I accepted it and I started self-injecting without any difficulties

Interviewer: Alright, alright, in your view do you feel the provider spent enough time training you?

Respondent: Yes, the time was adequate

Interviewer: Did you get the chance to practice on what you were taught? For instance, pricking a condom filled with sugar or sand?

Respondent: No, we didn’t do that

Interviewer: Alright, did you self-inject?

Respondent: Yes

Interviewer: How can you describe the friendliness of the training you received?

Respondent: The friendliness of the training on self-injection?

Interviewer: Mmmmhhh

Respondent: It was easy because it was an easy way, it’s not like it was hard to understand when being taught. It was quite easy, and we practiced and got it.

Interviewer: Yes, what were you given to take home after your training for instance how many injectables?

Respondent: 3

Interviewer: Were you given any paper with instructions on how to self-inject at home?

Respondent: No, the just explained on how we can self-inject

Interviewer: Alright, were you given a calendar?

Respondent: Yes, we were given a calendar

Interviewer: So you got all these?

Respondent: Yes, we got them at that time

Title: KK_SCPI_6.docx

Descriptor Info:	District: Nkhotakota	Target Population: TP2 - DMPA-IM/DMPA-SC PI clients	Respondent type: DMPA-SC PI client	Aware of SI: Yes - had heard of SI before	Coding phase: 1. Single coded	Client marital status: Married	Client age group: 20-29 years	Time since trained: N/A - client

Codes Applied:	C3.3.3 - Poor (insufficient/ judgemental) counselling/training	C3.2.1 - Lack of SI practice during training	C3.2.2 - Lack of inclination or confidence to inject, even after training	C3.1.2 - Confidence/comfort to inject after counselling/ training	C1.2.2 - Fear of SI (idea or reality)

INTERVIEWER: okay. Are there any new things in the video that you have never heard about?

RESPONDENT: aaaah. No. there is nothing new.

INTERVIEWER: Nothing new?

RESPONDENT: mmmh.

INTERVIEWER: What do you think are the most important things that are in the video?

RESPONDENT: The idea to inject yourself, Sayana.

INTERVIEWER: mmmhu. What about it?

RESPONDENT: The most important thing is the injection; the way I have seen it as the woman explained that at home…when you collect three injections you will take a year before you go back for the same.

INTERVIEWER: okay. Are there things you remember to have been taught by the providers but are not in the video? Are there things that the provider or may be a nurse taught you about Sayana but are not there in the video?

RESPONDENT: Aaah no.

INTERVIEWER: All of them?

RESPONDENT: mmmh.

INTERVIEWER: What else do you think women need to know about Sayana before they start using it? What else do women need to know before they start using Sayana?

RESPONDENT: They need to know how to open the medicine, how to shake it the way they do. Yes, they need to know about that and also how to inject themselves before they collect it. They need to follow everything as it has been taught by the provider; we need to follow it before we collect Sayana.

INTERVIEWER: mmmhu. Now that you have finished watching the video, have your thoughts about Sayana changed?

RESPONDENT: Yes, they have changed.

INTERVIEWER: Changed, how?

RESPONDENT: Have changed… here they have explained that… we should say the fears I had are gone.

INTERVIEWER: okay.

RESPONDENT: I was afraid. However, the way it has been taught here about shaking and everything and what to do when you reach that day my thoughts have changed and there are no more worries.

INTERVIEWER: oh…okay. As you have watched the video, all the fears are gone?

RESPONDENT: Yes. they are gone.

INTERVIEWER: Is it different from how the nurse or HSAS taught you?

RESPONDENT: A little different.

INTERVIEWER: How little is it different?

RESPONDENT: As they were teaching, they did not remove the Sayana.

INTERVIEWER: ka…

RESPONDENT: They did not show us the injection. They were just explaining theoretically. They did not demonstrate the way it is demonstrated in the video.

INTERVIEWER: ok.

RESPONDENT: yes. They did not take the injection they were just explaining theoretically.

INTERVIEWER: okay.

RESPONDENT: eyetu. Sure.

Title: KK_SCSI_2.docx

Descriptor Info:	District: Nkhotakota	Target Population: TP3 - DMPA-SC SI clients	Respondent type: DMPA-SC SI client	Aware of SI: Yes - had heard of SI before	Coding phase: 1. Single coded	Client marital status: Divorced/ separated/ widowed	Client age group: 20-29 years	Time since trained: N/A - client

Codes Applied:	C3.2.1 - Lack of SI practice during training

Interviewer: … did you get the chance to practice what you were trained like doing it on a condom filled with sugar / sand?

RESPONDENT : No

INTERVIEWER: On how to do self -injection you didn’t do that?

RESPONDENT: Yes

Title: MZs_IM_5.docx

Descriptor Info:	District: Mzimba South	Target Population: TP2 - DMPA-IM/DMPA-SC PI clients	Respondent type: DMPA-IM client	Aware of SI: Yes - had heard of SI before	Coding phase: 1. Single coded	Client marital status: Married	Client age group: 20-29 years	Time since trained: N/A - client

Codes Applied:	C3.2.1 - Lack of SI practice during training

Interviewer: were you given a chance to practice before self-injecting?

Respondent: he just demonstrated and asked us to self-inject below the navel. I did not understand very well so I went to ask to our provider at the community

Interviewer: were you given a chance to practice on something like a plastic filled with soil?

Respondent: no

Title: ZA_SCSI_2.docx

Descriptor Info:	District: Zomba	Target Population: TP3 - DMPA-SC SI clients	Respondent type: DMPA-SC SI client	Aware of SI: Yes - had heard of SI before	Coding phase: 1. Single coded	Client marital status:	Client age group:	Time since trained: N/A - client

Codes Applied:	C3.2.1 - Lack of SI practice during training

Interviewer: Did you get a chance to practice on what you had been trained?

Respondent: yes, someone tried, the women that were training us tried it…

Interviewer: Okay.

Respondent: so we watched them do it.

Interviewer: Okay.

Respondent: We watched

Interviewer: Did they give you a chance to practice?

Respondent: we were not given a chance to practice.

Interviewer: Okay.

Respondent: Yes

Interviewer: Alright, did they give you a condom filled with sugar or soil to practice injecting on it?

Respondent: No they did not do that, they just practiced on their body.

Interviewer: Alright.

Respondent: Yes

Title: ZA_SCSI_5.docx

Descriptor Info:	District: Zomba	Target Population: TP3 - DMPA-SC SI clients	Respondent type: DMPA-SC SI client	Aware of SI: Yes - had heard of SI before	Coding phase: 3. Triple coded	Client marital status: Married	Client age group: 30-39 years	Time since trained: N/A - client

Codes Applied:	C3.2.1 - Lack of SI practice during training

Interviewer: Alright, after they trained you, did they give you a chance that you should practice on what you had been trained?

Respondent: No

Title: ZA_SCSI_5.docx

Descriptor Info:	District: Zomba	Target Population: TP3 - DMPA-SC SI clients	Respondent type: DMPA-SC SI client	Aware of SI: Yes - had heard of SI before	Coding phase: 3. Triple coded	Client marital status: Married	Client age group: 30-39 years	Time since trained: N/A - client

Codes Applied:	C3.2.1 - Lack of SI practice during training

Interviewer: Alright, did they use any resources, for example taking soil and filling it in a condom and then practicing on it?

Respondent: Ah no

Title: MZs_SCSI_2.docx

Descriptor Info:	District: Mzimba South	Target Population: TP3 - DMPA-SC SI clients	Respondent type: DMPA-SC SI client	Aware of SI: Yes - had heard of SI before	Coding phase: 3. Triple coded	Client marital status: Married	Client age group: Under 20 (adolescent)	Time since trained: N/A - client

Codes Applied:	C3.2.1 - Lack of SI practice during training

Interviewer: Did you get the chance to practice what you were trained like filled condom with sugar or sand?

Respondent: No

Title: KK_IM_7.docx

Descriptor Info:	District: Nkhotakota	Target Population: TP2 - DMPA-IM/DMPA-SC PI clients	Respondent type: DMPA-IM client	Aware of SI: Yes - had heard of SI before	Coding phase: 1. Single coded	Client marital status: Married	Client age group: 30-39 years	Time since trained: N/A - client

Codes Applied:	C3.2.1 - Lack of SI practice during training

INTERVIEWER: They gave you Sayana?

RESPONDENT: Yes.

INTERVIEWER: At the hospital you were able to self-inject?

RESPONDENT: Here?

INTERVIEWER: mmmh.

RESPONDENT: They trained me here, but I failed at home (laughs). I was like no, this is a new thing, and I should not kill myself [hurt myself].

INTERVIEWER: But at the hospital you really managed to self-inject?

RESPONDENT: They trained me here, but I failed at home (laughing).

INTERVIEWER: When did this happen?

RESPONDENT: Aaaah, this year.

INTERVIEWER: This year or soon after the outbreak?

RESPONDENT: Yes, soon after the outbreak.

INTERVIEWER: What did they say was the reason for switching you to Sayana?

RESPONDENT: For our protection from the outbreak. We should be self-injecting at home.

INTERVIEWER: So you failed on your first attempt?

RESPONDENT: (laughs). Yes.

INTERVIEWER: After you failed, when did you go back to the hospital?

RESPONDENT: At the hospital I… [she was trying to remember].

INTERVIEWER: Did you come the same day, or you came after some days?

RESPONDENT: Aaaaa, I came after a day.

INTERVIEWER: mmmmhu.

RESPONDENT: Yes.

Title: KK_IM_7.docx

Descriptor Info:	District: Nkhotakota	Target Population: TP2 - DMPA-IM/DMPA-SC PI clients	Respondent type: DMPA-IM client	Aware of SI: Yes - had heard of SI before	Coding phase: 1. Single coded	Client marital status: Married	Client age group: 30-39 years	Time since trained: N/A - client

Codes Applied:	C3.2.1 - Lack of SI practice during training

INTERVIEWER: Okay, alright. As far as you remember, did the provider teach you anything that was not in the video?

RESPONDENT: Yes.

INTERVIEWER: What is that?

RESPONDENT: What I have seen in the video is that they took the drug when teaching. They did not do this with us for us to see how it is supposed to be done.

INTERVIEWER: Ok, about showing you the drug?

RESPONDENT: Yes.

Title: KK_IM_7.docx

Descriptor Info:	District: Nkhotakota	Target Population: TP2 - DMPA-IM/DMPA-SC PI clients	Respondent type: DMPA-IM client	Aware of SI: Yes - had heard of SI before	Coding phase: 1. Single coded	Client marital status: Married	Client age group: 30-39 years	Time since trained: N/A - client

Codes Applied:	C3.2.1 - Lack of SI practice during training

INTERVIEWER: Okay, alright. As far as you remember, did the provider teach you anything that was not in the video?

RESPONDENT: Yes.

INTERVIEWER: What is that?

RESPONDENT: What I have seen in the video is that they took the drug when teaching. They did not do this with us for us to see how it is supposed to be done.

INTERVIEWER: Ok, about showing you the drug?

RESPONDENT: Yes.

Title: ZA_SCSI_4.docx

Descriptor Info:	District: Zomba	Target Population: TP3 - DMPA-SC SI clients	Respondent type: DMPA-SC SI client	Aware of SI: Yes - had heard of SI before	Coding phase: 2. Double coded	Client marital status: Married	Client age group: 30-39 years	Time since trained: N/A - client

Codes Applied:	C3.2.1 - Lack of SI practice during training

Interviewer: Alright.

Respondent: Sure.

Interviewer: Alright, did they give you the chance to practice self-injecting?

Respondent: mmm (showing that one is following the conversation)

Interviewer: Did they give you a chance to use things like injecting on a condom filled with sugar or sand so that you could practice?

Respondent: No

Title: ZA_SCPI_8.docx

Descriptor Info:	District: Zomba	Target Population: TP2 - DMPA-IM/DMPA-SC PI clients	Respondent type: DMPA-SC PI client	Aware of SI: Yes - had heard of SI before	Coding phase: 1. Single coded	Client marital status:	Client age group:	Time since trained: N/A - client

Codes Applied:	C3.2.1 - Lack of SI practice during training

Interviewer: Alright, in these conversations, were you given a chance to practice on what you were trained?

Respondent: no I was not given a chance

Interviewer: alright.

Respondent: yes
