# Supplementary material for: Health care provider and client experiences of counselling on depot medroxyprogesterone acetate subcutaneous (DMPA-SC) for self-injection in Malawi
Source: PLOS Glob Public Health. 2023 Nov 30;3(11):e0002057. doi: 10.1371/journal.pgph.0002057 (PMC10688740; doi:10.1371/journal.pgph.0002057)
Supplement: S1 Data — (ZIP) [file pgph.0002057.s001.zip › S2/C3.2.2 lack of client conf after couns.doc]

Title: KK_IM_3.docx

Descriptor Info:	District: Nkhotakota	Target Population: TP2 - DMPA-IM/DMPA-SC PI clients	Respondent type: DMPA-IM client	Aware of SI: Yes - had heard of SI before	Coding phase: 1. Single coded	Client marital status: Divorced/ separated/ widowed	Client age group: 20-29 years	Time since trained: N/A - client

Codes Applied:	C3.2.2 - Lack of inclination or confidence to inject, even after training	C3.3.2 - Good (sufficient/ non-judgemental) counselling/training

INTERVIEWER: did you ever feel judged during these conversations?

RESPONDENT: No I have not

INTERVIEWER: As a result of the discussion, you had with the provider, did you agree to try self-injection?

RESPONDENT: Yes, I agreed but I did not take the method. But I recall they said it is a good method since anyone can use it, however, I have not used it so far.

INTERVIEWER: You have tried the method, but you agreed that you can use it

RESPONDENT: Ye I agreed that I can use it

Title: KK_IM_3.docx

Descriptor Info:	District: Nkhotakota	Target Population: TP2 - DMPA-IM/DMPA-SC PI clients	Respondent type: DMPA-IM client	Aware of SI: Yes - had heard of SI before	Coding phase: 1. Single coded	Client marital status: Divorced/ separated/ widowed	Client age group: 20-29 years	Time since trained: N/A - client

Codes Applied:	C1.2.2 - Fear of SI (idea or reality)	C3.2.2 - Lack of inclination or confidence to inject, even after training

INTERVIEWER: Alright, the last part on this topic, did you try self-injection at least once?

RESPONDENT: No I have never tried

INTERVIEWER: you have never tried? What stopped you from trying self-injection after your training

RESPONDENT: Was afraid to inject myself (participant break down into a laughter?)

INTERVIEWER: Afraid to inject yourself?

RESPONDENT: Ye since if the provider injects you, you turn away your face

INTERVIEWER: You may also do the same, turning away

RESPONDENT: Maybe since you learn as time goes

Title: ZA_HSA_2.docx

Descriptor Info:	District: Zomba	Target Population: TP1 - Providers/HSAs	Respondent type: HSA	Aware of SI: Yes - had heard of SI before	Coding phase: 3. Triple coded	Client marital status: N/A - provider	Client age group: N/A - provider	Time since trained: Less than a year ago

Codes Applied:	C3.2.2 - Lack of inclination or confidence to inject, even after training	C5.2.2 - Clients incorrectly / failing to administer at home	P3.3 - Counselling/ training approach, general	P4.1.3 - Provider availability/ willingness to repeat SI training

Interviewer: Alright, are the women that you serve uncomfortable or not confident to inject themselves?

Respondent: Some are uncomfortable, but only a few.

Interviewer: mmmm (showing that one is following the conversation)

Respondent: Those ones are the ones who when its time we are giving counselling about self-injection…

Interviewer: mmm (showing that one is following the conversation)

Respondent: Some of them are those that have completely failed to self-inject…

Interviewer: mmm (showing that one is following the conversation)

Respondent: They say that, ah I cannot manage…

Interviewer: I can not manage…

Respondent: I cannot manage, eh, those that cannot manage we just try to guide them and another time when they come back but we don’t force them that they should self-inject because if we do that, they might end up taking the doses and not use them…

Interviewer: mmmm (showing agreement)

Respondent: Yea, without using them, or they might just switch to another facility and get the provider administered injection there and they won’t use the doses that we have given them at this facility.

Interviewer: mmmm (showing that one is following the conversation)

Respondent: Yeah, and another client may end up lacking the doses while the other client is just keeping the doses without using them…

Interviewer: mmm (showing that one is following the conversation)

Respondent: So, we want that at first the client has been guided, and when they understand properly, they select the method, but if they don’t understand then we tell them that you can be coming so that we can be injecting you, even if its Sayana, yeah…

Interviewer: mmm (showing that one is following the conversation)

Respondent: Unless if they accept that they have understood…

Interviewer: mmmh (showing that ne is following the conversation)

Respondent: This method, the way that you can self-inject…

Title: MZs_SCSI_3.docx

Descriptor Info:	District: Mzimba South	Target Population: TP3 - DMPA-SC SI clients	Respondent type: DMPA-SC SI client	Aware of SI: Yes - had heard of SI before	Coding phase: 1. Single coded	Client marital status: Married	Client age group: 30-39 years	Time since trained: N/A - client

Codes Applied:	C3.2.2 - Lack of inclination or confidence to inject, even after training

Interviewer: would you tell me your experience with what you were taught on self-injection?

Respondent: the counselling was all well it’s just us we had fears that it cannot be possible to self-inject. Like for me I asked them to inject me because I was not confident. But I was told it was meant for self-injection. During the second visit, they counselled us again and this time I understood and managed to self-inject

Title: KK_SCPI_6.docx

Descriptor Info:	District: Nkhotakota	Target Population: TP2 - DMPA-IM/DMPA-SC PI clients	Respondent type: DMPA-SC PI client	Aware of SI: Yes - had heard of SI before	Coding phase: 1. Single coded	Client marital status: Married	Client age group: 20-29 years	Time since trained: N/A - client

Codes Applied:	C3.3.3 - Poor (insufficient/ judgemental) counselling/training	C3.2.1 - Lack of SI practice during training	C3.2.2 - Lack of inclination or confidence to inject, even after training	C3.1.2 - Confidence/comfort to inject after counselling/ training	C1.2.2 - Fear of SI (idea or reality)

INTERVIEWER: okay. Are there any new things in the video that you have never heard about?

RESPONDENT: aaaah. No. there is nothing new.

INTERVIEWER: Nothing new?

RESPONDENT: mmmh.

INTERVIEWER: What do you think are the most important things that are in the video?

RESPONDENT: The idea to inject yourself, Sayana.

INTERVIEWER: mmmhu. What about it?

RESPONDENT: The most important thing is the injection; the way I have seen it as the woman explained that at home…when you collect three injections you will take a year before you go back for the same.

INTERVIEWER: okay. Are there things you remember to have been taught by the providers but are not in the video? Are there things that the provider or may be a nurse taught you about Sayana but are not there in the video?

RESPONDENT: Aaah no.

INTERVIEWER: All of them?

RESPONDENT: mmmh.

INTERVIEWER: What else do you think women need to know about Sayana before they start using it? What else do women need to know before they start using Sayana?

RESPONDENT: They need to know how to open the medicine, how to shake it the way they do. Yes, they need to know about that and also how to inject themselves before they collect it. They need to follow everything as it has been taught by the provider; we need to follow it before we collect Sayana.

INTERVIEWER: mmmhu. Now that you have finished watching the video, have your thoughts about Sayana changed?

RESPONDENT: Yes, they have changed.

INTERVIEWER: Changed, how?

RESPONDENT: Have changed… here they have explained that… we should say the fears I had are gone.

INTERVIEWER: okay.

RESPONDENT: I was afraid. However, the way it has been taught here about shaking and everything and what to do when you reach that day my thoughts have changed and there are no more worries.

INTERVIEWER: oh…okay. As you have watched the video, all the fears are gone?

RESPONDENT: Yes. they are gone.

INTERVIEWER: Is it different from how the nurse or HSAS taught you?

RESPONDENT: A little different.

INTERVIEWER: How little is it different?

RESPONDENT: As they were teaching, they did not remove the Sayana.

INTERVIEWER: ka…

RESPONDENT: They did not show us the injection. They were just explaining theoretically. They did not demonstrate the way it is demonstrated in the video.

INTERVIEWER: ok.

RESPONDENT: yes. They did not take the injection they were just explaining theoretically.

INTERVIEWER: okay.

RESPONDENT: eyetu. Sure.

Title: KK_SCSI_2.docx

Descriptor Info:	District: Nkhotakota	Target Population: TP3 - DMPA-SC SI clients	Respondent type: DMPA-SC SI client	Aware of SI: Yes - had heard of SI before	Coding phase: 1. Single coded	Client marital status: Divorced/ separated/ widowed	Client age group: 20-29 years	Time since trained: N/A - client

Codes Applied:	C5.2.1 - Discontinuation of SI (including reasons)	C1.2.2 - Fear of SI (idea or reality)	C3.2.2 - Lack of inclination or confidence to inject, even after training

INTERVIEWER: Can you describe your experience giving your first self-injection at home

RESPONDENT: I had fever

INTERVIEWER: You had fever?

RESPONDENT: Yes

INTERVIEWER: Did you have concerns or fears about doing your first seif- injection and how did you overcome them?

RESPONDENT: Yes

INTERVIEWER: You had fears

RESPONDENT: Yes, I had fears

INTERVIEWER: Why were you afraid yet you managed to do self-injection at the hospital?

RESPONDENT: My concerns were that I did not mensurate for a month after I did my first self-injection

Title: KK_HSA_3.docx

Descriptor Info:	District: Nkhotakota	Target Population: TP1 - Providers/HSAs	Respondent type: HSA	Aware of SI: Yes - had heard of SI before	Coding phase: 3. Triple coded	Client marital status: N/A - provider	Client age group: N/A - provider	Time since trained: A year or more ago

Codes Applied:	C1.2.2 - Fear of SI (idea or reality)	C3.2.2 - Lack of inclination or confidence to inject, even after training

INTERVIEWER: Alright. Do women have any concerns about self-injection?

RESPONDENT: No. women who…women who have accepted, the ones we have trained and we are convinced that these ones can self-inject, we give them. But the ones we see that these ones cannot do it, they are the ones with the concerns for self-injection. But the ones we have trained…because before we inject them, we first counsel and train them.

INTERVIEWER: the women with the concerns, what type of concerns do they have about self-injection? What exactly worries them?

RESPONDENT: The concern is about…you know for a person to self-inject. The concerns are not about the injection or the drug no. but about if a person can really self-inject. That’s the main concern. They are afraid and worried.

Title: KK_HSA_3.docx

Descriptor Info:	District: Nkhotakota	Target Population: TP1 - Providers/HSAs	Respondent type: HSA	Aware of SI: Yes - had heard of SI before	Coding phase: 3. Triple coded	Client marital status: N/A - provider	Client age group: N/A - provider	Time since trained: A year or more ago

Codes Applied:	C3.2.2 - Lack of inclination or confidence to inject, even after training	C5.2.2 - Clients incorrectly / failing to administer at home

INTERVIEWER: What questions would you ask her?

RESPONDENT: I would ask why she said yes to Sayana when she knew that she will not use it…you will not be able to self-inject. But right now I have never encountered a woman who brings back Sayana. But those who come to us are the ones who have forgotten how to self-inject and they opt for for PI; We have only encountered the ones who fail to self-inject and we inject them.

Title: KK_HSA_3.docx

Descriptor Info:	District: Nkhotakota	Target Population: TP1 - Providers/HSAs	Respondent type: HSA	Aware of SI: Yes - had heard of SI before	Coding phase: 3. Triple coded	Client marital status: N/A - provider	Client age group: N/A - provider	Time since trained: A year or more ago

Codes Applied:	C3.2.2 - Lack of inclination or confidence to inject, even after training	C1.2.2 - Fear of SI (idea or reality)

INTERVIEWER: How about those who say no after the training. What reasons do they give?

RESPONDENT: The reason why they say no to the method is because they will not be able to self-inject.

INTERVIEWER: Fear?

RESPONDENT: fear.

Title: KK_SCPI_4.docx

Descriptor Info:	District: Nkhotakota	Target Population: TP2 - DMPA-IM/DMPA-SC PI clients	Respondent type: DMPA-SC PI client	Aware of SI: Yes - had heard of SI before	Coding phase: 2. Double coded	Client marital status: Never married	Client age group: 20-29 years	Time since trained: N/A - client

Codes Applied:	C3.2.2 - Lack of inclination or confidence to inject, even after training

INTERVIEWER: When you get here at the hospital what happened, when you come here for contraceptions, what happens? Explain to me, what happens?

RESPONDENT: First, they take our weight. They take the health passport and start calling names then we go there individually. They ask you the kind of contraception that you need so if its Sayana, they ask you if you inject yourself if you are not able to do it on your own you also state. Then they teach you. If you fail, as I did, I failed (giggles). They gave me the injection to inject myself. Therefore, I said no, I have not done it before. Whenever I come here, they inject me, I tell them to inject me so whether it is on the arm they inject me. Of course, they tell me to do it myself. We do it in this way. However, for me to get the needle and pierce my skin am very afraid. So I said just inject me, they said no wait outside that is when they sent me here.

Title: KK_SCPI_4.docx

Descriptor Info:	District: Nkhotakota	Target Population: TP2 - DMPA-IM/DMPA-SC PI clients	Respondent type: DMPA-SC PI client	Aware of SI: Yes - had heard of SI before	Coding phase: 2. Double coded	Client marital status: Never married	Client age group: 20-29 years	Time since trained: N/A - client

Codes Applied:	C3.2.2 - Lack of inclination or confidence to inject, even after training	C3.3.3 - Poor (insufficient/ judgemental) counselling/training

RESPONDENT: They were asking us, they were checking the names against the register, we should say, they took a calendar, yes. We did not discuss it properly because I said I would not be able to inject myself. I was asked if I could inject myself and I said no. The next question was on the number of times I have used contraceptions and I said this is my third. And she said who injects you when you get it, so I said I go to the people who know how to inject themselves. So they said I should learn to inject myself, and they started teaching me. But when I took the syringe to inject myself, I failed to do it. So they said wait outside your friends should also be assisted.

Title: KK_SCPI_4.docx

Descriptor Info:	District: Nkhotakota	Target Population: TP2 - DMPA-IM/DMPA-SC PI clients	Respondent type: DMPA-SC PI client	Aware of SI: Yes - had heard of SI before	Coding phase: 2. Double coded	Client marital status: Never married	Client age group: 20-29 years	Time since trained: N/A - client

Codes Applied:	C3.3.3 - Poor (insufficient/ judgemental) counselling/training	C3.2.2 - Lack of inclination or confidence to inject, even after training

INTERVIEWER: mmmhu. You want to tell me that when you come you get Sayana, how many do they give you?

RESPONDENT: Here at the hospital?

INTERVIEWER: mmmmh.

RESPONDENT: Then it is two, sometimes one, but when you say you inject yourself, they just give you to inject yourself and there you go.

INTERVIEWER: When you get here who injects…

RESPONDENT: the doctor injects me on the arm

INTERVIEWER: The doctor is the one who injects you.

RESPONDENT: Yes, on the arm. We just do this and they inject us. When the date they told you comes you come.

INTERVIEWER: Are they really injecting you with Sayana?

RESPONDENT: I do not know. It is in a packet…packet.

INTERVIEWER: How does it look like?

RESPONDENT: It is white.

INTERVIEWER: mmmhu.

RESPONDENT: In addition, the tube is short with a bulge.

INTERVIEWER: This, [showing her a picture of Sayana]

RESPONDENT: Yes, it is this one.

INTERVIEWER: Now when you come…

RESPONDENT: It is as round as this one. And inside of it are white drugs.

INTERVIEWER: When you come every time, the doctor injects you?

RESPONDENT: (giggles).

INTERVIEWER: Right?

RESPONDENT: yes. The first one was injected on my arm, and they said this is a self-injecting injection, we don’t do it for each other.

INTERVIEWER: As you have come today, are they going to inject you?

RESPONDENT: Today I have failed.

INTERVIEWER: Are they going to inject you?

RESPONDENT: No they have not.

INTERVIEWER: Oh, okay

RESPONDENT: mmmmh. They have not injected me.

INTERVIEWER: okay. You said this is the third time?

RESPONDENT: mmmmh.

INTERVIEWER: For the first and second occasions, the doctor injected you, and this is the third round?

RESPONDENT: Yes

INTERVIEWER: okay.

RESPONDENT: mmmh.

Title: MZs_IM_5.docx

Descriptor Info:	District: Mzimba South	Target Population: TP2 - DMPA-IM/DMPA-SC PI clients	Respondent type: DMPA-IM client	Aware of SI: Yes - had heard of SI before	Coding phase: 1. Single coded	Client marital status: Married	Client age group: 20-29 years	Time since trained: N/A - client

Codes Applied:	C3.2.2 - Lack of inclination or confidence to inject, even after training

Interviewer: after the provider counseled you, did you accept to use self-injection

Respondent: I accepted but I asked what if I made a mistake when self-injecting and get pregnant. So he said we would do nothing about it ‘giggles’

Title: MZs_HSA_3.docx

Descriptor Info:	District: Mzimba South	Target Population: TP1 - Providers/HSAs	Respondent type: HSA	Aware of SI: Yes - had heard of SI before	Coding phase: 3. Triple coded	Client marital status: N/A - provider	Client age group: N/A - provider	Time since trained: Less than a year ago

Codes Applied:	C2.1.2 - Reduced trips to facility	C3.2.2 - Lack of inclination or confidence to inject, even after training	C8.1 - Male involvement, general	C4 - Storage of unused units

Q.17 Interviewer: in your view, what are the main factors that would support a client to take up the self- injection option with DMPA-SC?

Respondent: distance where she comes from, if it is very far, she says I have to get Sayana so that I should come I should not be coming here frequently to take almost a year before I come here. The other one could be I heard there is a new vaccine which whereby people inject themselves so I would like to be one of those so today I want to get that Sayana we say here it is we will train you after being trained we see what you do then later on you will be given to carry at home. So she says ok it’s easy for me to stay at home and come back at a later date instead of coming here every 3 months. But for the barriers, others would say that one I will have problems where to keep because my husband will either throw away or my children will may take it or I can’t do… I can’t do. You train her you say now I have finished have you understood she says yes do it then she says I can’t make it myself just give me so she says give me I have understood but I cannot take it home you have to give me yah.

Title: MZs_SCSI_2.docx

Descriptor Info:	District: Mzimba South	Target Population: TP3 - DMPA-SC SI clients	Respondent type: DMPA-SC SI client	Aware of SI: Yes - had heard of SI before	Coding phase: 3. Triple coded	Client marital status: Married	Client age group: Under 20 (adolescent)	Time since trained: N/A - client

Codes Applied:	C3.2.2 - Lack of inclination or confidence to inject, even after training	C5.1.5 - Provider availability/ willingness to retrain	C88 - Client great quotes

Q9. Interviewer: Can you describe your experience of being trained in self injection? And who trained you

Respondent: The training was good even though at first it was difficult for us to accept that we can do it but after some time we were able to do it. The good thing is that the provider said that whenever we have a question, we free to visit the facility for help.

Title: ZA_HSA_3.docx

Descriptor Info:	District: Zomba	Target Population: TP1 - Providers/HSAs	Respondent type: HSA	Aware of SI: Yes - had heard of SI before	Coding phase: 1. Single coded	Client marital status: N/A - provider	Client age group: N/A - provider	Time since trained: A year or more ago

Codes Applied:	C3.2.2 - Lack of inclination or confidence to inject, even after training

Interviewer: Alright, have you faced any other problems when giving out the self-injection family planning injection in the past three months?

Respondent: Ahhh the problems are like some of the women were like they won’t manage to inject themselves they should just be injected right at the hospital

Title: ZA_SCSI_4.docx

Descriptor Info:	District: Zomba	Target Population: TP3 - DMPA-SC SI clients	Respondent type: DMPA-SC SI client	Aware of SI: Yes - had heard of SI before	Coding phase: 2. Double coded	Client marital status: Married	Client age group: 30-39 years	Time since trained: N/A - client

Codes Applied:	C3.2.2 - Lack of inclination or confidence to inject, even after training	C5.2.2 - Clients incorrectly / failing to administer at home	C5.1.2 - Clients correctly administering SI at home	C3.1.2 - Confidence/comfort to inject after counselling/ training

Interviewer: Alright, ah when the covid-19 pandemic started have you seen any changes on accessing contraceptives?

Respondent: No I have not seen any changes because I have seen that it’s the same.

Interviewer: mmmm (showing that one is following the conversation)

Respondent: Even though they encouraged us to get injectables.

Interviewer: mmm (showing that one is following the conversation)

Respondent: But some fail to self-inject, they come back to the facility to have the injection administered to them. Those of us who are able to self-inject get the injectables and go home with it.

Interviewer: Alright.

Respondent: Sure.

Title: MZs_FAC_1.docx

Descriptor Info:	District: Mzimba South	Target Population: TP1 - Providers/HSAs	Respondent type: Facility-based provider	Aware of SI: Yes - had heard of SI before	Coding phase: 3. Triple coded	Client marital status: N/A - provider	Client age group: N/A - provider	Time since trained: A year or more ago

Codes Applied:	P3.1.4 - Provider sufficient support to counsel/train on SI	C3.2.2 - Lack of inclination or confidence to inject, even after training

Interviewer: Okay, have you or your colleagues discuss this about the Sayana press that a woman can inject herself, have you ever discuss it with your colleagues?

Respondent: Yah, in many occasions.

Interviewer: What was your discussion like, can you share?

Respondent: Yah, we know that may be in some scenarios a woman may not convincing to do it in the right way and the way you are teaching her and I think she cannot do by herself, so those we put on providers injection and we need to supervise another visit. So those are the issues sometimes we discuss that no this client was very nervous she couldn’t do the way I taught her or she was not comfortable actually to do it herself and she says no still inject me.

Interviewer: Mmmmh.

Respondent: So those are the issues sometimes we discuss with our friends that may be next she will get used with this.

Title: MZs_FAC_1.docx

Descriptor Info:	District: Mzimba South	Target Population: TP1 - Providers/HSAs	Respondent type: Facility-based provider	Aware of SI: Yes - had heard of SI before	Coding phase: 3. Triple coded	Client marital status: N/A - provider	Client age group: N/A - provider	Time since trained: A year or more ago

Codes Applied:	C1.2.2 - Fear of SI (idea or reality)	C1.1.1 - Positive client attitudes to SI	C3.2.2 - Lack of inclination or confidence to inject, even after training

Interviewer: In your experience as a provider, what is the response when you explain to clients that they could self-inject this method?

Respondent: Yah some they can refuse differently, they show fear and they are reluctant but some they are eager that they can actually do it.

Interviewer: Mmmmmh.

Respondent: Yah, so you see those mixed options from the client.

Title: MZs_FAC_1.docx

Descriptor Info:	District: Mzimba South	Target Population: TP1 - Providers/HSAs	Respondent type: Facility-based provider	Aware of SI: Yes - had heard of SI before	Coding phase: 3. Triple coded	Client marital status: N/A - provider	Client age group: N/A - provider	Time since trained: A year or more ago

Codes Applied:	P3.3 - Counselling/ training approach, general	C3.2.2 - Lack of inclination or confidence to inject, even after training	C1.2.2 - Fear of SI (idea or reality)

Interviewer: In your view what are the main factors that would discourage clients to take up the self-injection option with DMPA-SC?

Respondent: If they haven`t agree with the husband they will find it difficult to say now where am I going to keep this at home, so if they haven`t agreed with the husband then that can be the major problem. It can also be the fear of unknown to say I cannot do it, she has no confident in herself. Also counselling, if you haven`t counselled to her satisfaction or you haven`t demonstrated well then it can also affect that.

Title: MZs_FAC_1.docx

Descriptor Info:	District: Mzimba South	Target Population: TP1 - Providers/HSAs	Respondent type: Facility-based provider	Aware of SI: Yes - had heard of SI before	Coding phase: 3. Triple coded	Client marital status: N/A - provider	Client age group: N/A - provider	Time since trained: A year or more ago

Codes Applied:	P3.4 - Time to counsel/train, general	C3.2.2 - Lack of inclination or confidence to inject, even after training

Interviewer: Alright. Does the length of training influence whether a woman choose to take up self-injection?

Respondent: No

Interviewer: I just want to know that when you spent alit of time with client, does it influence whether a woman can choose to take up self-injection?

Respondent: Yah, time factors is also very important because as we are saying it goes with confidence and demonstrations it really need to have much time to seat and demonstrate to her. So say you are in a hurry and you have just bra..bra…bra..(not counselling it very well), she might not have that confidence, so we need to take much of time to explain then she will say I can do it.

Title: MZs_SCPI_3.docx

Descriptor Info:	District: Mzimba South	Target Population: TP2 - DMPA-IM/DMPA-SC PI clients	Respondent type: DMPA-SC PI client	Aware of SI: Yes - had heard of SI before	Coding phase: 1. Single coded	Client marital status: Married	Client age group: 20-29 years	Time since trained: N/A - client

Codes Applied:	C3.2.2 - Lack of inclination or confidence to inject, even after training

Interviewer: Did you get a chance to practice?

Respondent: No

Interviewer: For example giving you a condom filled with sand or sugar?

Respondent: Nothing like that happened.

Title: MZs_SCPI_3.docx

Descriptor Info:	District: Mzimba South	Target Population: TP2 - DMPA-IM/DMPA-SC PI clients	Respondent type: DMPA-SC PI client	Aware of SI: Yes - had heard of SI before	Coding phase: 1. Single coded	Client marital status: Married	Client age group: 20-29 years	Time since trained: N/A - client

Codes Applied:	C3.2.2 - Lack of inclination or confidence to inject, even after training

Q15. Interviewer: Did you try self-injection at least once?

Respondent: No

Interviewer: What stopped you from trying self-injection after your training?

Respondent: I had some fears that I might not be able to do it on my own.

Interviewer: So, did you go to the provider for help?

Respondent: No

Interviewer: Why?

Respondent: Because I had this feeling that I cannot do it myself.
