# Supplementary material for: Health care provider and client experiences of counselling on depot medroxyprogesterone acetate subcutaneous (DMPA-SC) for self-injection in Malawi
Source: PLOS Glob Public Health. 2023 Nov 30;3(11):e0002057. doi: 10.1371/journal.pgph.0002057 (PMC10688740; doi:10.1371/journal.pgph.0002057)
Supplement: S1 Data — (ZIP) [file pgph.0002057.s001.zip › S2/C3.3 couns vs video PAIM only.doc]

Title: KK_IM_3.docx

Descriptor Info:	District: Nkhotakota	Target Population: TP2 - DMPA-IM/DMPA-SC PI clients	Respondent type: DMPA-IM client	Aware of SI: Yes - had heard of SI before	Coding phase: 1. Single coded	Client marital status: Divorced/ separated/ widowed	Client age group: 20-29 years	Time since trained: N/A - client

Codes Applied:	C3.3 - Counselling / training approach for injectable users

INTERVIEWER: What happens at the facility /with the HSA? How much time does the provider spend with you and what do they say?

RESPONDENT: When we get to the health center, they usually ask us how we have coped up with the previous contraceptive that we took, I would say they take us through a counselling session. So if anyone wants to change the method she can do so, whether you want to get no-plant or to maintain the previous one depending on your experience with the previous contraceptive.

INTERVIEWER: Alright, so that means every visit you make the provider offers a counselling session

RESPONDENT: Yes

INTERVIEWER: So one has a choice?

RESPONDENT: Yes

Title: KK_IM_3.docx

Descriptor Info:	District: Nkhotakota	Target Population: TP2 - DMPA-IM/DMPA-SC PI clients	Respondent type: DMPA-IM client	Aware of SI: Yes - had heard of SI before	Coding phase: 1. Single coded	Client marital status: Divorced/ separated/ widowed	Client age group: 20-29 years	Time since trained: N/A - client

Codes Applied:	C3.3 - Counselling / training approach for injectable users

INTERVIEWER: You heard from the health center did they train you

RESPONDENT: Yes, we were trained that there are different types of Family planning methods, one is injected by the provider, then another one would inject herself, then here are also pills and no-plant.

INTERVIEWER: So what about the one where one can inject herself, did they train you on how it works, or they just mentioned about it.

RESPONDENT: Ye they did mention but since I was not interested, I just heard that one can inject herself

INTERVIEWER: Ok

RESPONDENT: They said anyone can access it, you hold and shake it then you open at the end where the needle is placed. Then you inject and press to release the medicine.

Title: KK_IM_3.docx

Descriptor Info:	District: Nkhotakota	Target Population: TP2 - DMPA-IM/DMPA-SC PI clients	Respondent type: DMPA-IM client	Aware of SI: Yes - had heard of SI before	Coding phase: 1. Single coded	Client marital status: Divorced/ separated/ widowed	Client age group: 20-29 years	Time since trained: N/A - client

Codes Applied:	C3.3 - Counselling / training approach for injectable users

INTERVIEWER: Alright, have you spoken about DMPA -SC for self-injection with a provider. Either during your visit today or in the past?

RESPONDENT: Yes , is it like counselling?

INTERVIEWER: Yes

RESPONDENT: We have discussed before

INTERVIEWER: Would you please tell me more about those conversation? How did the provider describe it to you? Did they tell you all the pros and cons?

RESPONDENT: They said it is a good method one can take home and inject herself thereby reducing the visits to the hospital. But the only problem may be continuous period. But this may happen to some people and not others depending on how one’s boy responds.

Title: MZs_IM_8.docx

Descriptor Info:	District: Mzimba South	Target Population: TP2 - DMPA-IM/DMPA-SC PI clients	Respondent type: DMPA-IM client	Aware of SI: Yes - had heard of SI before	Coding phase: 1. Single coded	Client marital status: Married	Client age group: 20-29 years	Time since trained: N/A - client

Codes Applied:	C3.3 - Counselling / training approach for injectable users

Interviewer: what happens when you arrive at the facility?

Respondent: when we come here at the facility, we wait until they start the clinic at 8 am. We queue before the clinic starts. Then we start entering one by one.

Interviewer: how long does the provider take with each one of you?

Respondent: they start the clinic at 8 am. We enter the room one by one by we don’t take much time

Title: MZs_IM_8.docx

Descriptor Info:	District: Mzimba South	Target Population: TP2 - DMPA-IM/DMPA-SC PI clients	Respondent type: DMPA-IM client	Aware of SI: Yes - had heard of SI before	Coding phase: 1. Single coded	Client marital status: Married	Client age group: 20-29 years	Time since trained: N/A - client

Codes Applied:	C3.3 - Counselling / training approach for injectable users	C3.2.1 - Lack of SI practice during training	C5.1.3 - Resources/follow up materials (e.g. calendar) to aid timely re-injection	C6 - Waste management

Interviewer: now I would like to talk about new method which is called Sayana which a woman self-inject. For those who cannot manage to self-inject the provider inject. Have you ever heard of this method of family planning?

TIME STAMP: 24:24MIN- 36: 34 MIN

Respondent: My friends once told me about the self-injection. But I had already taken Depo-IM which I did not encounter any challenges. I was just wondering how it could be because I had already taken one.

Interviewer: would you take Sayana for self-injection if they tell you that you can?

Respondent: yes, I can take depending with the counselling they can give me

Interviewer: you said you have talked about this method of self-injection from the provider. Tell me more what you said from the start to the time they made a decision to self-inject?

Respondent: when we were here, we were told that there is now a new method of family planning whereby a woman will self-inject. We were told that it was not difficult to self-inject and that we should not have fears. It would help because sometimes providers are not always available. so we will just get and keep it at a good place.

Interviewer: I would like you to tell me from the start how they counselled you?

Respondent: they told us that it is a good method. They told us the benefits of this method. Then they called us to self-inject. They assured us that it was not difficult to self-inject. But I was not comfortable to get it

Interviewer: Did they counsel you on the procedure of self-injection, on the benefits and disadvantages of using the method?

Respondent: they gave us Sayana, cotton to take home. They told us to first clean the area then shake the content. Then to press on the neck, then to pinch the skin and inject. After that then you throw the used needle into the toilet. Then sign on the date on the calendar. Then to bring the calendar to the provider as a proof that you self-injected

Interviewer: you were told to show the calendar?

Respondent: yes

Interviewer: you said after using you threw in…?

Respondent: in the toilet

Interviewer: did you accept to self-inject?

Respondent: no

Interviewer: why not?

Respondent: I thought if I get Sayana it will be difficult for me to go back to Depo-IM it would be like I mix things

Interviewer: did you not ask the provider if it was possible for you to get Sayana?

Respondent: no I did not ask

Interviewer: if they had counselled you on Sayana, would you self-inject?

Respondent: if well counselled I can

Interviewer: did the provider took enough time on counselling?

Respondent: yes, those who had questions were given a chance to ask

Interviewer: were you given a chance to practice before self-injecting?

Respondent: no we were told to pinch our skin and self-inject

Interviewer: were you not given a chance to try it on a condom before self-injecting?

Respondent: no

Interviewer: what were you given to take home after self-injecting?

Respondent: yes, they told us that those who choose to self-inject we will give them something to take home

Interviewer: what sort of things?

Respondent: 2 vials of Sayana and a calendar

Interviewer: how many vials of Sayana did they supply?

Respondent: 2 one was used to self-inject right here at the facility

Title: MZs_IM_2.docx

Descriptor Info:	District: Mzimba South	Target Population: TP2 - DMPA-IM/DMPA-SC PI clients	Respondent type: DMPA-IM client	Aware of SI: Yes - had heard of SI before	Coding phase: 3. Triple coded	Client marital status: Married	Client age group: 30-39 years	Time since trained: N/A - client

Codes Applied:	C3.3 - Counselling / training approach for injectable users

Interviewer: What happens at the facility and how much does the provider spend with you?

Respondent: As of now they don`t spend a lot of time. They just ask us some questions for them to know if the method we are using works well. They have a book which they use when counseling us and after that they do the injection and thus all. At first, we had one day of family planning and we used to wait for a long time before being assisted, but right now right now once you have come for family planning they ask ask you few questions and explain to use about the method thus all.

Title: MZs_IM_2.docx

Descriptor Info:	District: Mzimba South	Target Population: TP2 - DMPA-IM/DMPA-SC PI clients	Respondent type: DMPA-IM client	Aware of SI: Yes - had heard of SI before	Coding phase: 3. Triple coded	Client marital status: Married	Client age group: 30-39 years	Time since trained: N/A - client

Codes Applied:	C3.3 - Counselling / training approach for injectable users	C1.2.1 - Negative client attitudes to SI

Interviewer: Like you are with the provider and you have come for family planning which is injectable, has the doctor ever explain to you about self-inject?

Respondent: Yes, when I came for providers injection

Interviewer: Thus what I want you to explain to me

Respondent: When I came for providers re-injection, the doctor explained to me that we have a new family planning method where we teach women and those who think that they can be able to self-inject, we give them so that they can be doing it. It works the same as the one you have been using though there is a different.

Interviewer: Did he mention the differences?

Respondent: I can’t explain because I did not pay attention but he said there is a different, he also said that the one I am using with providers injection its different with this one but this one they will give it to me so that I can be injecting myself and for those that their husbands do not allow them to use contraceptive they will benefit from this one.

Interviewer: You have said that you did discuss it with the provider, but then you did not pay attention

Respondent: Yes.

Interviewer: Why didn`t you pay attention?

Respondent: It’s because I don’t want to be convinced with a new method, yet I tried to switch to Jadelle but did not work on me thus why I did not pay attention cause the one I am using works well on me, so I don’t want to hear other methods.

Interviewer: Did the provider tell you the good and bad thing about this self-injection method?

Respondent: Aaaaaa…… he only mention about the good ones, he did not say anything about the bad ones. These doctors they always say the good ones, yet they know that it cannot work well for everyone.

Interviewer: So what are the good things he mention?

Respondent: He did explain just that I did not pay attention, but he said women can be injecting themselves at home and this will reduce regular visit to the hospital.

Interviewer: So you have said that the provider did explain to you but you did……

Respondent: Yes, I did not pay attention because I already have one method that fits me.

Interviewer: Can you tell me on how a woman can self-inject herself if you can remember?

Respondent: Aaaa…. I can’t remember.

Interviewer: Thanks a lot, because you have said that the provider did explain to you but did not pay attention.

Respondent: Mmmmm, (yes)

Title: ZA_IM_2.docx

Descriptor Info:	District: Zomba	Target Population: TP2 - DMPA-IM/DMPA-SC PI clients	Respondent type: DMPA-IM client	Aware of SI: Yes - had heard of SI before	Coding phase: 1. Single coded	Client marital status: Married	Client age group: 30-39 years	Time since trained: N/A - client

Codes Applied:	C3.3 - Counselling / training approach for injectable users

Interviewer: Alright, thank you, what happens when you get to the hospital for your injection?

Respondent: When we get to the hospital the first thing we are given a health talk, we start with a prayer then the health talk, when it’s time to start getting the injection they tell us that we cannot get into the doctors room with other clients, they have to talk to us one on one because maybe other people might have questions that they want to ask, so they may be scared or they may be shy and they may not afford to ask the questions in a group, so we meet with the person who injects us and we tell them about our issues and then they help and us and we go back home.

Interviewer: Alright, how long does this probably take, how long does your meeting with the health professional take?

Respondent: It doesn’t take long.

Interviewer: Approximately how minutes do you take?

Respondent: Maybe five minutes, if you have questions, you can take even ten minutes so that your questions get addressed.

Title: ZA_IM_2.docx

Descriptor Info:	District: Zomba	Target Population: TP2 - DMPA-IM/DMPA-SC PI clients	Respondent type: DMPA-IM client	Aware of SI: Yes - had heard of SI before	Coding phase: 1. Single coded	Client marital status: Married	Client age group: 30-39 years	Time since trained: N/A - client

Codes Applied:	C3.3 - Counselling / training approach for injectable users

Interviewer: Alright, can you tell me more about this discussion, how did the health worker describe about this injection?

Respondent: They told us that no one is forced to take this injection, they also told us that, everyone can decide freely on their own to take the injection or not, a person can choose to either inject themselves or they can choose to be injected by the health worker. That’s what we talked about.

Interviewer: Alright, did the health workers tell you about the pros and cons of the self-injection injectables?

Respondent: Yes, they told us that the injection is good, the self-injection.

Interviewer: Alright, did they say anything about the side effects of this injection? Maybe problems or difficulties you may face either physically or any other problems?

Respondent: No, they did not say anything on that, they just explained about the benefits, maybe that you may inject yourself and experience some side effects, when you inject yourself and you experience any problems you need to come to the hospital and meet the providers and explain to them.

Title: ZA_IM_2.docx

Descriptor Info:	District: Zomba	Target Population: TP2 - DMPA-IM/DMPA-SC PI clients	Respondent type: DMPA-IM client	Aware of SI: Yes - had heard of SI before	Coding phase: 1. Single coded	Client marital status: Married	Client age group: 30-39 years	Time since trained: N/A - client

Codes Applied:	C3.3 - Counselling / training approach for injectable users

Interviewer: I was saying that you said that you have talked to the providers about self-injection option for the injectable contraceptive the way that you can inject yourself, can you explain to me about your experiences on the training?

Respondent: Yes, someone was asking me what I felt when I injected myself, did the injection work the way that it does when I get it from a provider? I told them that the injection does not have any problem and there was any problem that I have encountered while using it.

Title: KK_IM_5.docx

Descriptor Info:	District: Nkhotakota	Target Population: TP2 - DMPA-IM/DMPA-SC PI clients	Respondent type: DMPA-IM client	Aware of SI: No - never heard of SI before	Coding phase: 3. Triple coded	Client marital status: Divorced/ separated/ widowed	Client age group: Under 20 (adolescent)	Time since trained: N/A - client

Codes Applied:	C3.3 - Counselling / training approach for injectable users

INTERVIEWER: What happens at the facility /with the HSA? How much time does the provider spend with you and what do they say? I recall you said you get injected by the HSA, so how long do they take to assist you?

RESPONDENT: They don’t take long; upon arrival they ask us if we want to get injected and if we say yes, they get their tools and inject us.

INTERVIEWER: Aright, it seems to be a simple job hence it doesn’t take long

RESPONDENT: Yes

Title: ZA_IM_1.docx

Descriptor Info:	District: Zomba	Target Population: TP2 - DMPA-IM/DMPA-SC PI clients	Respondent type: DMPA-IM client	Aware of SI: Yes - had heard of SI before	Coding phase: 1. Single coded	Client marital status: Married	Client age group: 40-49 years	Time since trained: N/A - client

Codes Applied:	C3.3 - Counselling / training approach for injectable users	C3.3.3 - Poor (insufficient/ judgemental) counselling/training

Interviewer: Thank you very much, what happens at the facility? How long do providers take before they start helping you?

Respondent: When we get here at the hospital, we explain to the providers that we have come to access family planning services.

Interviewer: Mmmhhh

Respondent: So they take our health passport and look at the date we were supposed to come. If the dates are matching, the take the health passport and fill in the register. The start with injecting us then they write in the health passports when we are supposed to return to the hospital to get our injectable.

Interviewer: When you come to the hospital, how long does it take for providers to inject you, write in your health passport and fill in the register? How long does this take?

Respondent: This really doesn’t take time; about 5 to 10 mins everything is done.

Title: ZA_IM_1.docx

Descriptor Info:	District: Zomba	Target Population: TP2 - DMPA-IM/DMPA-SC PI clients	Respondent type: DMPA-IM client	Aware of SI: Yes - had heard of SI before	Coding phase: 1. Single coded	Client marital status: Married	Client age group: 40-49 years	Time since trained: N/A - client

Codes Applied:	C3.3 - Counselling / training approach for injectable users	C1.2.2 - Fear of SI (idea or reality)	C3.1.2 - Confidence/comfort to inject after counselling/ training	C2.1.2 - Reduced trips to facility	C3.2.1 - Lack of SI practice during training	C3.4 - Time to train clients

Interviewer: How did you first hear about it?

Respondent: When I first heard this, I was nervous that would I be able to inject myself because we were told that we have to inject ourselves below the belly button or on the thigh. I was afraid about the belly button thinking I would inject my intestines or anything else. We were then properly taught on how to inject ourselves.

Interviewer: Mmmhh

Respondent: So ahh I accepted it and I used it.

Interviewer: Do you know anyone who is using self-injection?

Respondent: Self injection is currently out of stock, so I would not know

Interviewer: Mmmmhhh

Respondent: Yes

Interviewer: At the time it was available did you know anyone who was using it?

Respondent: Yes, I knew someone who was using it at that time

Interviewer: Alright, alright, have you spoken about DMPA-SC for self-injection with any provider?

Respondent: Yes, we have spoken about it

Interviewer: Mmmmhhh

Respondent: We were taught on how we can use the injection, how we can inject as well as the days when we are supposed to wait till the injection is ineffective in our bodies so that we can re inject the second one.

Interviewer: Can you tell me more about those conversations? How did the provider describe it to you? Tell me in detail what you were told?

Respondent: The provider explained that with this type of injection everyone is supposed to self-inject and the position is either below the belly button or on the thigh. So they explained on how to inject as well as the days we are supposed to wait for the medicine to be effective when re injecting.

Interviewer: Mmmmhhh

Respondent: They explained that when we first inject ourselves we should wait for 90days which is three months. After the 90 days we should inject and so on.

Interviewer: Mmmhhh alright, did they tell you the pros and cons of self-injection?

Respondent: They told us about the pros that sometimes people get lazy or get busy to get injected by provider. With self-injection there are no problems to inject yourself. These are the pros

Interviewer: Did they explain the cons of self-injection?

Respondent: No, they did not explain

Time stamp: 19 mins 38 secs

Interviewer: Alright thank you very much. As a result of the discussion you had with a provider, did you agree to try self-injection?

Respondent: Yes, I agreed and I tried it

Interviewer: Mmmmhhh What influenced you to try the self-injection?

Respondent: I saw that self-injection was a way that I could freely self-inject. The places where I inject are places were am free and no one is there.

Interviewer: What influenced you to self-inject?

Respondent: I was influenced with the fact that I want to family plan and avoid getting pregnant

Interviewer: Alright, alright, can you describe your experience of being trained in self-injection?

Respondent: The time they were explaining about self-injection

Interviewer: mmmhhh

Respondent: I was nervous that they are possibilities of hurting myself as well as not fully injecting due to pain as well as the medicine not getting inside. Once I fully understood I found that I accepted it and I started self-injecting without any difficulties

Interviewer: Alright, alright, in your view do you feel the provider spent enough time training you?

Respondent: Yes, the time was adequate

Interviewer: Did you get the chance to practice on what you were taught? For instance, pricking a condom filled with sugar or sand?

Respondent: No, we didn’t do that

Interviewer: Alright, did you self-inject?

Respondent: Yes

Interviewer: How can you describe the friendliness of the training you received?

Respondent: The friendliness of the training on self-injection?

Interviewer: Mmmmhhh

Respondent: It was easy because it was an easy way, it’s not like it was hard to understand when being taught. It was quite easy, and we practiced and got it.

Interviewer: Yes, what were you given to take home after your training for instance how many injectables?

Respondent: 3

Interviewer: Were you given any paper with instructions on how to self-inject at home?

Respondent: No, the just explained on how we can self-inject

Interviewer: Alright, were you given a calendar?

Respondent: Yes, we were given a calendar

Interviewer: So you got all these?

Respondent: Yes, we got them at that time

Title: KK_SCPI_6.docx

Descriptor Info:	District: Nkhotakota	Target Population: TP2 - DMPA-IM/DMPA-SC PI clients	Respondent type: DMPA-SC PI client	Aware of SI: Yes - had heard of SI before	Coding phase: 1. Single coded	Client marital status: Married	Client age group: 20-29 years	Time since trained: N/A - client

Codes Applied:	C3.3 - Counselling / training approach for injectable users	C3.4 - Time to train clients

INTERVIEWER: mmmhu. When you get here at the hospital on the day that you are supposed to get the injection, what happens?

RESPONDENT: When we arrive, we get on the scale. After the scale, we go into the room where the nurse is. The nurse takes the details and asks us some questions thereafter we are injected.

INTERVIEWER: Okay then…

RESPONDENT: The nurse also asks us if we have had any blood tests, and when it is our time, the nurse advises us to take some blood tests first.

INTERVIEWER: mmmhu. Apart from asking you about blood tests…how long, does the nurse take to help you? When you arrive, how long do you wait?

RESPONDENT: Today I arrived at eight, and everything was fast by half past eight I was injected.

Time stamp: (20:25).

INTERVIEWER: When you go to the nurse, you said what happens.

RESPONDENT: She tests us with something here, she asks us what problems we are experiencing with it, and we answer depending on what you are experiencing then the nurse takes those details down and then we get injected.

INTERVIEWER: okay.

RESPONDENT: mmmh.

INTERVIEWER: Now, I would like to disc

Title: KK_SCPI_6.docx

Descriptor Info:	District: Nkhotakota	Target Population: TP2 - DMPA-IM/DMPA-SC PI clients	Respondent type: DMPA-SC PI client	Aware of SI: Yes - had heard of SI before	Coding phase: 1. Single coded	Client marital status: Married	Client age group: 20-29 years	Time since trained: N/A - client

Codes Applied:	C1.2.2 - Fear of SI (idea or reality)	C3.3 - Counselling / training approach for injectable users	C3.3.3 - Poor (insufficient/ judgemental) counselling/training

INTERVIEWER: mmmhu. You said you have discussed the self-injecting contraception with HSAs before?

RESPONDENT: mmmmh.

INTERVIEWER: Have you discussed it today?

RESPONDENT: Today we did not discuss but the nurse…we discussed with the nurse.

INTERVIEWER: What was the nurse saying?

RESPONDENT: Was saying, “Why do you still want us to inject you”? Why are you not getting the self-injecting one?

INTERVIEWER: okay.

RESPONDENT: yes

INTERVIEWER: The nurse asked you why…

RESPONDENT: Why are you not injecting yourself?

INTERVIEWER: You want me to be injecting you?

RESPONDENT: Yes

INTERVIEWER: So what did you say?

RESPONDENT: I said I have never taken the self-injecting type before.

INTERVIEWER: mmmhu

RESPONDENT: However, when they say…they just inject me I do not take the injection. Nevertheless, I should I want; I can take the injection and inject myself at home.

INTERVIEWER: What stops you from getting the self-injective injection as of now?

RESPONDENT: Encourages me?

INTERVIEWER: What makes you fail? You said should you want, you can get it.

RESPONDENT: mmmmmh.

INTERVIEWER: Why do you not get the self-injecting one to inject yourself at home but the doctor should be injecting you?

RESPONDENT: (Laughs) aaah what about just some fears? It was some fear to inject myself, but now I have observed that a lot of women some of them did not even go to school are doing it for themselves then I can inject myself also. Back then I was afraid, I was afraid but not anymore.

Title: KK_SCPI_6.docx

Descriptor Info:	District: Nkhotakota	Target Population: TP2 - DMPA-IM/DMPA-SC PI clients	Respondent type: DMPA-SC PI client	Aware of SI: Yes - had heard of SI before	Coding phase: 1. Single coded	Client marital status: Married	Client age group: 20-29 years	Time since trained: N/A - client

Codes Applied:	C3.3 - Counselling / training approach for injectable users

INTERVIEWER: How many times have you discussed Sayana with the HSAs?

RESPONDENT: It could be three times.

INTERVIEWER: Three times?

RESPONDENT: mmmh.

INTERVIEWER: Would you tell me about these discussions? What did they explain to you, how, what were they saying?

RESPONDENT: They were saying the self-injective contraception is good because you will start in a good time from home to the hospital and you will start in a good time from the hospital going home and get home in good time. When you get it, there are no delays because they do not inject you, you will inject yourself at home. They say whether you want two when you reach the date you inject yourself and you will not come here and the other one you will use on another date.

INTERVIEWER: mmmhu.

RESPONDENT: mmmh.

INTERVIEWER: Did they tell you the bad sides of Sayana?

RESPONDENT: aaah they did not…

INTERVIEWER: They did not explain, they just explained the good side?

RESPONDENT: Yes. The advantages and how to administer it.

INTERVIEWER: okay. Now, as they were explaining this to you, did you feel being judged at all? When I say judged I mean to be scorned, may be mocked. Did you feel being scorned or judged?

RESPONDENT: Those feelings are not there.

INTERVIEWER: None?

RESPONDENT: mmmmh.

Title: KK_SCPI_6.docx

Descriptor Info:	District: Nkhotakota	Target Population: TP2 - DMPA-IM/DMPA-SC PI clients	Respondent type: DMPA-SC PI client	Aware of SI: Yes - had heard of SI before	Coding phase: 1. Single coded	Client marital status: Married	Client age group: 20-29 years	Time since trained: N/A - client

Codes Applied:	C3.3 - Counselling / training approach for injectable users	C3.3.2 - Good (sufficient/ non-judgemental) counselling/training

INTERVIEWER: okay. Would you explain your experience learning about Sayana? What have you experienced while learning about Sayana from HSAs?

RESPONDENT: There are no problems at all, only thanksgiving that everything they taught us was well received. There are not any problems.

INTERVIEWER: okay. Do you think the HSAs took enough time teaching you?

RESPONDENT: No

INTERVIEWER: mmmmh?

RESPONDENT: By enough you mean each time we come to be taught?

INTERVIEWER: mmmh. Were they taking enough time teaching you Sayana?

RESPONDENT: Oh yes, they were really teaching us.

INTERVIEWER: Enough time?

RESPONDENT: aa[yes].

INTERVIEWER: Until you thought, you had understood?

RESPONDENT: mmmh.

INTERVIEWER: okay. So when teaching you were they filling a condom with sugar or sand to imitate a thigh or stomach?

Time stamp: (30:11)

RESPONDENT: mmmh

INTERVIEWER: Did the HSAs do this as they were teaching you?

RESPONDENT: No they did not do

INTERVIEWER: They did not do.

RESPONDENT: mmmmh.

INTERVIEWER: Did you think the lessons were simple?

RESPONDENT: aaaah, No, they are not simple.

INTERVIEWER: Not easy?

RESPONDENT: mmmhu.

INTERVIEWER: Why is that?

RESPONDENT: Its difficulties comes in because they want …they …(giggles)

INTERVIEWER: Explain, I am listening, they want.

RESPONDENT: (giggle)

INTERVIEWER: These lessons are easy. Why are they easy?

RESPONDENT: You mean easy when learning?

INTERVIEWER: yes

RESPONDENT: The HSAs?

INTERVIEWER: mmmh.

RESPONDENT: It is easy.

INTERVIEWER: How about to your side?

RESPONDENT: It is easy (laughs)

INTERVIEWER: Why?

RESPONDENT: It is easy because we also answer.

INTERVIEWER: You answer?

RESPONDENT mmmh.

INTERVIEWER: mmmhu.

RESPONDENT: We answer when asked a question.

Title: KK_SCPI_4.docx

Descriptor Info:	District: Nkhotakota	Target Population: TP2 - DMPA-IM/DMPA-SC PI clients	Respondent type: DMPA-SC PI client	Aware of SI: Yes - had heard of SI before	Coding phase: 2. Double coded	Client marital status: Never married	Client age group: 20-29 years	Time since trained: N/A - client

Codes Applied:	C3.3 - Counselling / training approach for injectable users

INTERVIEWER: When you get here at the hospital what happened, when you come here for contraceptions, what happens? Explain to me, what happens?

RESPONDENT: First, they take our weight. They take the health passport and start calling names then we go there individually. They ask you the kind of contraception that you need so if its Sayana, they ask you if you inject yourself if you are not able to do it on your own you also state. Then they teach you. If you fail, as I did, I failed (giggles). They gave me the injection to inject myself. Therefore, I said no, I have not done it before. Whenever I come here, they inject me, I tell them to inject me so whether it is on the arm they inject me. Of course, they tell me to do it myself. We do it in this way. However, for me to get the needle and pierce my skin am very afraid. So I said just inject me, they said no wait outside that is when they sent me here.

INTERVIEWER: okay. How long do they take to assist you?

RESPONDENT: The health personnel?

INTERVIEWER: mmmmh

RESPONDENT: They do not take time.

INTERVIEWER: okay. Alright.

RESPONDENT: They are fast enough.

Title: KK_SCPI_4.docx

Descriptor Info:	District: Nkhotakota	Target Population: TP2 - DMPA-IM/DMPA-SC PI clients	Respondent type: DMPA-SC PI client	Aware of SI: Yes - had heard of SI before	Coding phase: 2. Double coded	Client marital status: Never married	Client age group: 20-29 years	Time since trained: N/A - client

Codes Applied:	C1.1.3 - Awareness of DMPA-SC SI	C3.3 - Counselling / training approach for injectable users

INTERVIEWER: Have you ever discussed Sayana with a doctor or a nurse?

RESPONDENT: mmmh.

INTERVIEWER: Have you ever discussed with them?

RESPONDENT: mmmh mmmh [no]

INTERVIEWER: Have you discussed Sayana with them today?

RESPONDENT: yes.

INTERVIEWER: In the past?

RESPONDENT: In the past?

INTERVIEWER: mmmh.

RESPONDENT: Maybe they were saying it in public, maybe they were doing it before we arrived; we could just go straight for scale and thereafter the injection.

INTERVIEWER: okay. What were these health personnel saying about Sayana?

RESPONDENT: The way they were saying it

INTERVIEWER: Yes here, I thought you said you discussed Sayana just today.

RESPONDENT: Yes, we have discussed.

INTERVIEWER: What were they saying?

RESPONDENT: They were asking us, they were checking the names against the register, we should say, they took a calendar, yes. We did not discuss it properly because I said I would not be able to inject myself. I was asked if I could inject myself and I said no. The next question was on the number of times I have used contraceptions and I said this is my third. And she said who injects you when you get it, so I said I go to the people who know how to inject themselves. So they said I should learn to inject myself, and they started teaching me. But when I took the syringe to inject myself, I failed to do it. So they said wait outside your friends should also be assisted.

Title: KK_SCPI_4.docx

Descriptor Info:	District: Nkhotakota	Target Population: TP2 - DMPA-IM/DMPA-SC PI clients	Respondent type: DMPA-SC PI client	Aware of SI: Yes - had heard of SI before	Coding phase: 2. Double coded	Client marital status: Never married	Client age group: 20-29 years	Time since trained: N/A - client

Codes Applied:	C3.3 - Counselling / training approach for injectable users

INTERVIEWER: Would you explain what the HSAs explained about Sayana?

RESPONDENT: That question will be difficult for me to answer because I have never heard them explain Sayana.

INTERVIEWER: You have never heard anything?

RESPONDENT: yes. We just come here to get the contraception, maybe they explained at first, but we were not around given that we do not come here every day.

INTERVIEWER: mmmhu.

RESPONDENT: mmmh.

INTERVIEWER: Now, you have never heard the doctor explain something about Sayana?

RESPONDENT: They were saying there self-injecting Sayana.

INTERVIEWER: We do this and that.

RESPONDENT: Yes, they were teaching, were taking it.

Time stamp: (30:01)

INTERVIEWER: Yes, they were teaching you?

RESPONDENT: Yes, shaking.

INTERVIEWER: What were they saying when teaching you?

RESPONDENT: They were saying, take this, open it, tear it and it is white inside. After tearing it, you take it out and shake. Then they were saying we should hold it in the middle then they said pull it out and inject yourself.

INTERVIEWER: okay.

RESPONDENT: Just that when you have problems understanding, that is it.

INTERVIEWER: (giggles) No your head does not have any problem

RESPONDENT: (giggles) ah.

INTERVIEWER: Did they tell you the good and bad sides of Sayana?

RESPONDENT: No

INTERVIEWER: Bad

RESPONDENT: No they did not tell me anything.

INTERVIEWER: Okay, as they were telling you this, did you feel judged in any way? When I say judged you know what I mean right?

RESPONDENT: mmmh mmmh[no].

INTERVIEWER: maybe mocking you, did you feel that?

RESPONDENT: No, they did not say anything.

INTERVIEWER: okay. You mean to say that; they did not tell you what the advantages of Sayana are?

RESPONDENT: On that one, I do not know what the advantages of Sayana are?

INTERVIEWER: They did not tell you?

RESPONDENT: No they did not…

INTERVIEWER: The bad side?

RESPONDENT: What?

INTERVIEWER: They did not the bad side also?

RESPONDENT: No. You might the one to tell me.

INTERVIEWER: (laughs) no no, am not a doctor.

RESPONDENT: (laughs) You can explain the method, for us we just get them.

INTERVIEWER: Okay?

RESPONDENT: Yes

Title: KK_SCPI_4.docx

Descriptor Info:	District: Nkhotakota	Target Population: TP2 - DMPA-IM/DMPA-SC PI clients	Respondent type: DMPA-SC PI client	Aware of SI: Yes - had heard of SI before	Coding phase: 2. Double coded	Client marital status: Never married	Client age group: 20-29 years	Time since trained: N/A - client

Codes Applied:	C3.3 - Counselling / training approach for injectable users	C3.1.1 - Practice of SI during training

INTERVIEWER: Were you given an opportunity to try what you were taught?

RESPONDENT: I tried and I failed to do it.

INTERVIEWER: So you just tried on your arm, or you were given… because some were given a condom full of sugar to try. Where did you try?

RESPONDENT: On the arm

INTERVIEWER: Oh... Here right?

RESPONDENT: Yes, on the thigh

INTERVIEWER: On the thigh

RESPONDENT: mmmmh.

INTERVIEWER: mmmhu. What did you think about these lessons?

RESPONDENT: The lessons?

INTERVIEWER: Were the lessons easy or difficult?

RESPONDENT: The lessons about contraceptions?

INTERVIEWER: The lessons

RESPONDENT: The lessons are simple, could say they are, but sometimes we may say are difficult.

INTERVIEWER: They are simple, in what way?

RESPONDENT: When you get it, as long as you hand is steady and you inject yourself, you get used.

Title: ZA_IM_6.docx

Descriptor Info:	District: Zomba	Target Population: TP2 - DMPA-IM/DMPA-SC PI clients	Respondent type: DMPA-IM client	Aware of SI: Yes - had heard of SI before	Coding phase: 1. Single coded	Client marital status: Married	Client age group: 30-39 years	Time since trained: N/A - client

Codes Applied:	C3.3 - Counselling / training approach for injectable users

Interviewer: Alright. Ah so when you get here what happens so that you find the help you need.

Respondent: When we get here, we get in line.

Interviewer: Mmhh

Respondent: When we get in line, they take our books and call per person to enter so that they help us.

Interviewer: Mhhm Alright, ah how long do the health counselors take to help you out?

Respondent: I! sometimes it takes a while because they have to counsel us first.

Interviewer: Mmhhmm

Respondent: Mmhhmm

Interviewer: Alright, anything more to add?

Respondent: No.

Title: ZA_SCPI_3.docx

Descriptor Info:	District: Zomba	Target Population: TP2 - DMPA-IM/DMPA-SC PI clients	Respondent type: DMPA-SC PI client	Aware of SI: No - never heard of SI before	Coding phase: 3. Triple coded	Client marital status: Never married	Client age group: Under 20 (adolescent)	Time since trained: N/A - client

Codes Applied:	C3.3 - Counselling / training approach for injectable users

Interviewer: Alright, what happens when you get to the HSAs?

Respondent: We tell them that we have come to access contraceptives.

Interviewer: mmm (showing that one is following the conversation)

Respondent: Yes

Interviewer: Alright, how long do they take while they are helping you and what do they say?

Respondent: It doesn’t last for more than an hour

Interviewer: It doesn’t last for more than one hour?

Respondent: yes.

Interviewer: Alright, what do they say the moment that you get there?

Respondent: They give us counselling concerning contraceptives.

Interviewer: mmm (showing that one is following the conversation)

Respondent: That we want to access

Interviewer: Alright, ah

Respondent: So, for us we also have some question to ask them…

Title: MZs_IM_5.docx

Descriptor Info:	District: Mzimba South	Target Population: TP2 - DMPA-IM/DMPA-SC PI clients	Respondent type: DMPA-IM client	Aware of SI: Yes - had heard of SI before	Coding phase: 1. Single coded	Client marital status: Married	Client age group: 20-29 years	Time since trained: N/A - client

Codes Applied:	C3.3 - Counselling / training approach for injectable users

Interviewer: where did you hear about self-injection first?

Respondent: I heard it from the provider here at the facility. they told me that there is new Depo for self-injection. I asked questions if it was possible for someone to self-inject. The provider said he would counsel on how to go about it. You will follow the instructions we shall give you. So I was told that they will counsel me on how to self-inject. On the next visit they gave me self-injection. I was told to self-inject at my convenient time. The previous day after self-injecting I had abdominal cramps and started menses before my dates.

Title: MZs_IM_6.docx

Descriptor Info:	District: Mzimba South	Target Population: TP2 - DMPA-IM/DMPA-SC PI clients	Respondent type: DMPA-IM client	Aware of SI: Yes - had heard of SI before	Coding phase: 1. Single coded	Client marital status: Married	Client age group: Under 20 (adolescent)	Time since trained: N/A - client

Codes Applied:	C0.4 - Access to or process of obtaining FP services	C3.3 - Counselling / training approach for injectable users

Interviewer: Would you tell me how you accessed this method you are using?

Respondent: How I accessed it?

Interviewer: Mmmmh (agreeing)

Respondent: When we reach the facility…[stammer] the provider explained to us the pros and cons of the family planning method then they inject me and record in my health passport book and give me the next date of appointment. When I am due, I come for subsequent doses.

Interviewer: When did you start receiving the first dose if you can recall

Respondent: It was last year I think in November.

Interviewer: Before coming to the health facility to receive services did you do anything at home?

Respondent: Concerning what?

Interviewer: Concerning your household. What did you do before coming to the facility?

Respondent: I do household chores. Then I take a bathe that’s when I come here.

Interviewer: How do you come here?

Respondent: I use a hired bicycle because it’s a bit far from where I come from

Interviewer: How long does it take for you to travel from your home to this facility

Respondent: Mmmmmh (thinking out loud) about 30 minutes when I used the hired bicycle

Interviewer: Mmmmh (thinking out loud) what happens when you come here to get the family planning method you use?

Respondent: They start with health talk. after that they administer

Interviewer: Would you explain detail what happens from the time you arrive

Respondent: When we arrive, they greet us. We also greet them. Then we give them the health passport book then they check, and they then administer

TIME STAMP 2: FROM 10 MINUTES 13SEC

Interviewer: Mmmhu (agreeing) when they are giving you a talk, do they spend enough time with you?

Respondent: Yes, they take time and ask questions as well

Interviewer: Mmmhu (agreeing) how many minutes do they take

Respondent: From the time they start the clinic?

Interviewer: Mmmh (agreeing)

Respondent: Most of them it takes them hours

Title: MZs_IM_6.docx

Descriptor Info:	District: Mzimba South	Target Population: TP2 - DMPA-IM/DMPA-SC PI clients	Respondent type: DMPA-IM client	Aware of SI: Yes - had heard of SI before	Coding phase: 1. Single coded	Client marital status: Married	Client age group: Under 20 (adolescent)	Time since trained: N/A - client

Codes Applied:	C3.3 - Counselling / training approach for injectable users	C3.1.1 - Practice of SI during training	C1.1.3 - Awareness of DMPA-SC SI	C5.2.3 - Lack of resources/follow up materials to aid timely re-injection	C1.2.2 - Fear of SI (idea or reality)

Interviewer: Mmmhu (agreeing) aaaah (thinking out loud) now I would like to talk about another type of type of injectable family planning method maybe you have heard of it before. It is also called Sayana Press. It is similar to intramuscular Depo only that with Sayana the woman self-inject. How you ever heard of this family planning method?

Respondent: Yes

Interviewer: Where did you hear it from?

Respondent: At the health facility

Interviewer: At the facility? after hearing that a woman will self-inject what was your reaction?

Respondent: At first, I had fears since I had never used before

Interviewer: When you first heard about it. What specific information did you hear about it?

Respondent: They explained to us

Interviewer: What did they say specifically?

Respondent: I heard that there was a new injectable family planning whereby a woman will self-inject, and they taught us how they self-inject

Interviewer: Do you know anyone who uses this method?

Respondent: Eeeh (agreeing) there are some I know)

Interviewer: Aaah (thinking out loud) have you ever talked with the provider on this new method of self-inject?

Respondent: No I haven’t talked to them about it

Interviewer: Have you ever talked to them in previous visits?

Respondent: No I haven’t talked to them on self-inject

Interviewer: Haven’t you talked to them on self-inject?

Respondent: Mmmmh (denying)

Interviewer: But at first, I asked you said you have ever heard about self-inject from the providers

Respondent: I thought you said me asking them. But they really talked about it but I have never used self-inject I use intramuscular Depo

Interviewer: How many times did he talk about it?

Respondent: A couple of times

Interviewer: Did they mention it when you come for family planning services or anytime you visit the health facility

Respondent: When I come for family planning clinic

Interviewer: So would you tell me what was discussed with the provider in detail what you discussed about Sayana Press?

Respondent: They told us that this method is good because you use it at home there in no need to frequently visit the health facility so long as they teach us on how to do it at home

Interviewer: Mmmh (agreeing) did they ever mentioned on the pros and cons of using Sayana Press?

Respondent: The advantages is what I have already said. Overall they said it does not have any cons

Interviewer: They said no cons for using it? Did you feel judged when they were teaching you?

Respondent: Judged?

Interviewer: Yes, like being rude to you

Respondent: No

Interviewer: After they taught you about Sayana Press that a woman will be required to self-inject, aaaah (thinking out loud) did you ever agreed to self-inject at home?

Respondent: Yes

Interviewer: Mmmmhu (agreeing) what made you agree to use it

Respondent: Because I’ve seen that all methods are safe so that’s why I agreed to try it

Interviewer: What else motivated you?

Respondent: Another factor is that I was motivated by my neighbor who also uses it

Interviewer: Mmmh (agreeing) ….aaaaah (thinking out loud) have you ever used this method of self-inject?

Respondent: No I haven’t

Interviewer: You haven’t used it before

Respondent: No

Interviewer: Would you tell me your experiences during the lessons on Sayana Press by the providers. What were the lessons like on this method of self-inject?

Respondent: Quite moment …seemed not to have understood the question

Interviewer: Did they spend enough time with you

Respondent: Yes, they spent enough time

Interviewer: Did they give you a chance to practice

Respondent: Yes, they did

Interviewer: Did you self-inject? What did you use to practice?

Respondent: They gave us a chance like me I practiced on my body

Interviewer: The lessons which you got would you describe it? Was it easy or difficult?

Respondent: It was not easy

Interviewer: It wasn’t easy?

Respondent: Yes, especially to self-inject. It was the first experience, so it was not easy

Interviewer: Mmmhu (agreeing) after self-injecting, what else did they give you to take home

Respondent: They gave us condoms

Interviewer: What else were you given?

Respondent: Sayana

Interviewer: How many doses?

Respondent: They gave us 2 doses for self-injection

Interviewer: What else were you given?

Respondent: That’s all

Title: ZA_IM_4.docx

Descriptor Info:	District: Zomba	Target Population: TP2 - DMPA-IM/DMPA-SC PI clients	Respondent type: DMPA-IM client	Aware of SI: Yes - had heard of SI before	Coding phase: 1. Single coded	Client marital status: Married	Client age group: Under 20 (adolescent)	Time since trained: N/A - client

Codes Applied:	C3.3 - Counselling / training approach for injectable users

Interviewer: Alright thank you what happens uhm when you come here at the hospital for the injection, what really happens? Would you explain in detail what it happens when you reach here at the hospital?

Respondent: Mmh when we reach, they test your blood pressure, when they do the test you submit the hospital passport when it’s your turn you enter inside they help you then you leave

Interviewer: Alright what about the nurses how long does it take for them to help you? (Baby calls the mother)

Respondent: Counselling?

Interviewer: (Baby calls the mother) When you come at the hospital how long does it take to get all the help from the nurses?

Respondent: It depends on how the people are when they are a lot of people it takes time and when they are few (Baby screams while calling the mother) it doesn’t take long.

Title: ZA_IM_4.docx

Descriptor Info:	District: Zomba	Target Population: TP2 - DMPA-IM/DMPA-SC PI clients	Respondent type: DMPA-IM client	Aware of SI: Yes - had heard of SI before	Coding phase: 1. Single coded	Client marital status: Married	Client age group: Under 20 (adolescent)	Time since trained: N/A - client

Codes Applied:	C3.3 - Counselling / training approach for injectable users

Interviewer: Alright do you know anyone who is using this self-injectable family planning method?

Respondent: Mmm I don’t know anyone

(Baby calls the mother) Interviewer: Have you ever talked about this injection method of family planning with the nurses?

Respondent: Mmm mmmmh we have never talked about it

Title: MZs_IM_7.docx

Descriptor Info:	District: Mzimba South	Target Population: TP2 - DMPA-IM/DMPA-SC PI clients	Respondent type: DMPA-IM client	Aware of SI: Yes - had heard of SI before	Coding phase: 1. Single coded	Client marital status: Divorced/ separated/ widowed	Client age group: Under 20 (adolescent)	Time since trained: N/A - client

Codes Applied:	C3.3 - Counselling / training approach for injectable users	C3.3.3 - Poor (insufficient/ judgemental) counselling/training

Interviewer: you don’t know anyone? Have you ever talked with your provider about Sayana?

Respondent: no

Interviewer: today’s clinic, did he mention about Sayana?

Respondent: no

Title: KK_IM_7.docx

Descriptor Info:	District: Nkhotakota	Target Population: TP2 - DMPA-IM/DMPA-SC PI clients	Respondent type: DMPA-IM client	Aware of SI: Yes - had heard of SI before	Coding phase: 1. Single coded	Client marital status: Married	Client age group: 30-39 years	Time since trained: N/A - client

Codes Applied:	C3.3 - Counselling / training approach for injectable users

INTERVIEWER: Okay. So what happens at the facility when you come for your injection?

RESPONDENT: When I arrive, the first thing that we do is to check for our body weight at [not clear]. after that, they collect our health passports and call our names. We go there, get the injection and they give back our health passports.

INTERVIEWER: Okay. So how much time does the provider take to help you?

RESPONDENT: Aaah not that much time

INTERVIEWER: Okay. Everything goes well?

RESPONDENT: Everything goes well.

Title: KK_IM_7.docx

Descriptor Info:	District: Nkhotakota	Target Population: TP2 - DMPA-IM/DMPA-SC PI clients	Respondent type: DMPA-IM client	Aware of SI: Yes - had heard of SI before	Coding phase: 1. Single coded	Client marital status: Married	Client age group: 30-39 years	Time since trained: N/A - client

Codes Applied:	C3.3 - Counselling / training approach for injectable users

INTERVIEWER: Now I understand (laughing). Have you ever discussed about DMPA-SC for self-injection with a provider, either today or in the past?

RESPONDENT: Mmmmh mmmh [no]. I should not lie; I have never discussed with health providers.

INTERVIEWER: mmmh

RESPONDENT: ah ah[no]

INTERVIEWER: How about when you come for a visit here?

RESPONDENT: When we come here and when we meet them inside, we discuss.

INTERVIEWER: okay.

RESPONDENT: mmmhu

INTERVIEWER: What do you discuss?

RESPONDENT: They say that for those who want there are self-injectables. And those who are interested should meet them either at the facility or in the community [HSA’s], and they will train them.

INTERVIEWER: So how do they explain to you? What happens for them to start explaining?

RESPONDENT: (laughs) eeh. When they get inside, they ask us women for anyone… (laughs)

INTERVIEWER: yes anyone…

RESPONDENT: Those who what contraception for example injectables for both the provider and self, they are available. That is how they explain.

INTERVIEWER: Did they tell you the pros and cons of this injectable?

RESPONDENT: No they did not. They were just encouraging us not to give birth like pigs. We should be using contraceptives for the child to grow healthy.

INTERVIEWER: What did they say a woman will benefit from using self-injection?

RESPONDENT: Aaaaaah [long one], I don’t know because that method…

INTERVIEWER: But you have said that gave you Sayana right? Right?

RESPONDENT: Yes.

INTERVIEWER: some other time, right?

RESPONDENT: Sure.

INTERVIEWER: So they gave you without telling you the pros and cons?

RESPONDENT: Mmmhu. They did not explain to me the advantages and its disadvantages.

INTERVIEWER: Why? So they just gave you with no explanation?

RESPONDENT: They were just giving us because we wanted it so that our children should be protected. That is why we were accepting for our protection.

INTERVIEWER: Mmmmmhu. So when they were explaining to you about Sayana and how it’s supposed to be done, did you ever feel judged during the discussions? When I say being judged do you know what I mean?

RESPONDENT: mmmmh mmmmh [no].

INTERVIEWER: Maybe being mocked.

RESPONDENT: Okay.

INTERVIEWER: Or saying some words that are not pleasant.

RESPONDENT: no, never.

INTERVIEWER: okay.

RESPONDENT: Sure.

Title: KK_IM_7.docx

Descriptor Info:	District: Nkhotakota	Target Population: TP2 - DMPA-IM/DMPA-SC PI clients	Respondent type: DMPA-IM client	Aware of SI: Yes - had heard of SI before	Coding phase: 1. Single coded	Client marital status: Married	Client age group: 30-39 years	Time since trained: N/A - client

Codes Applied:	C3.3 - Counselling / training approach for injectable users

INTERVIEWER: Mmmhu. When you came back after your first attempt, they told you to self-inject while they were watching? Mmmh?

RESPONDENT: They did not say I should self-inject…

INTERVIEWER: I mean you have failed to self-inject at home, the next day you came back here. You brought back the Sayana right? So did they tell you to try again?

RESPONDENT: Yes.

INTERVIEWER: Then you did.

RESPONDENT: They said do this, and we were doing that. And they said wondered why we failed at home. It is because when you are alone at home, you become afraid, and the providers are used to it.

INTERVIEWER: You managed to self-inject here?

RESPONDENT: Here?

INTERVIEWER: eeeh. Yes.

RESPONDENT: Yes, I managed and they were saying why did you fail (laughs). And they told us to go home, and you will get Sayana on your next visit because by the you will be able to do it.

INTERVIEWER: okay.

RESPONDENT: mmmh.

INTERVIEWER: chabwino. Alright.

RESPONDENT: On my next visit, there was a stockout.

Title: ZA_IM_5.docx

Descriptor Info:	District: Zomba	Target Population: TP2 - DMPA-IM/DMPA-SC PI clients	Respondent type: DMPA-IM client	Aware of SI: Yes - had heard of SI before	Coding phase: 2. Double coded	Client marital status: Married	Client age group: 30-39 years	Time since trained: N/A - client

Codes Applied:	C3.3 - Counselling / training approach for injectable users

Respondent: When we come here at the facility, we don’t spend so much time. No

Interviewer: Mmmm (showing that one is following the conversation)

Respondent: We don’t take time; they welcome us and assist us then we what…

Title: ZA_IM_5.docx

Descriptor Info:	District: Zomba	Target Population: TP2 - DMPA-IM/DMPA-SC PI clients	Respondent type: DMPA-IM client	Aware of SI: Yes - had heard of SI before	Coding phase: 2. Double coded	Client marital status: Married	Client age group: 30-39 years	Time since trained: N/A - client

Codes Applied:	C3.3 - Counselling / training approach for injectable users

Interviewer: Thank you, what happens here at the facility, how long does the provider take and what do they say when they are helping you?

Respondent: Scale yes when we get here, first thing we get on the scale

Interviewer: Mmmm (showing that one is following the conversation)

Respondent: They see and then after that they will check our BP

Interviewer: Yes

Respondent: They will check, then after checking, then they will call one client at a time

Title: ZA_IM_5.docx

Descriptor Info:	District: Zomba	Target Population: TP2 - DMPA-IM/DMPA-SC PI clients	Respondent type: DMPA-IM client	Aware of SI: Yes - had heard of SI before	Coding phase: 2. Double coded	Client marital status: Married	Client age group: 30-39 years	Time since trained: N/A - client

Codes Applied:	C3.3 - Counselling / training approach for injectable users

Respondent: That time when it was available yeah

Interviewer: Mmmm (showing that one is following the conversation)

Respondent: When we go to the hospital at the clinic, they would explain to us

Interviewer: Mmmm (showing that one is following the conversation)

Respondent: The procedure and how to do it, they would explain that.

Interviewer: Mmmm (showing that one is following the conversation)

Respondent: Yes

Interviewer: Alright so it was often, right?

Respondent: Yes

Interviewer: Alright, can you tell me more about the discussion you had with the providers?

Respondent: The discussion that I was having with the providers was about how we can self-inject

Interviewer: Mmmm (showing that one is following the conversation)

Respondent: The discussion was that when we want to take the self-injection contraceptive

Interviewer: Mmmm (showing that one is following the conversation)

Respondent: Because that time the Depo was not available

Interviewer: Mmmm (showing that one is following the conversation)

Respondent: So the Sayana was the one more popular those months

Interviewer: Mmmm (showing that one is following the conversation)

Respondent: So when we get there, they would tell us that this is the injection, but I don’t what? I don’t inject it on you it is supposed to be self-injected

Interviewer: Mmmm (showing that one is following the conversation)

Respondent: So we say that ah okay, and they give us, when they give us, they show us the syringe, that this is the syringe.

Interviewer: Mmmm (showing that one is following the conversation)

Respondent: When you get it, and you want to use it

Interviewer: Mmmm (showing that one is following the conversation)

Respondent: We don’t inject the way that we do with Depo

Interviewer: Mmmm (showing that one is following the conversation)

Respondent: No

Interviewer: Yah

Respondent: We inject under the navel or on the thigh

Interviewer: Mmmm (showing that one is following the conversation)

Respondent: But also when we want to inject, we don’t hold it straight like we do with Depo.

TIME INTO THE INTERVIEW: 20Mins

Interviewer: Mmmm (showing that one is following the conversation)

Respondent: When we want to inject

Interviewer: Mmmm (showing that one is following the conversation)

Respondent: We hold it in a slanting way.

Interviewer: Mmmm (showing that one is following the conversation)

Respondent: When we are about to use it, when you want the liquid to come out and get into the body, they told us how to open it

Interviewer: Mmmm (showing that one is following the conversation)

Respondent: How we can open it, when they give you the unit since the liquid in in the unit

Interviewer: Yah

Respondent: So on this side there is like as I have said a date for expiration

Interviewer: Mmmm (showing that one is following the conversation)

Respondent: We remove that

Interviewer: Mmmm (showing that one is following the conversation)

Respondent: Tear it and remove it, then we shake it, we shake it, after that on the top on the unit where there is the cover, before we remove it, because it is hard just removing it like that.

Interviewer: Yah yah yah

Respondent: So we push it inside and open it

Interviewer: Mmmm (showing that one is following the conversation)

Respondent: And then what

Interviewer: Self-inject

Respondent: Yes

Interviewer: Alright, did they tell you the pros and cons about this injectable?

Respondent: Ah the self-injectable the problem is that it’s not a problem you just need the right procedure like the way that they told us.

Interviewer: Mmmm (showing that one is following the conversation)

Respondent: You need to understand

Interviewer: Yah

Title: ZA_IM_5.docx

Descriptor Info:	District: Zomba	Target Population: TP2 - DMPA-IM/DMPA-SC PI clients	Respondent type: DMPA-IM client	Aware of SI: Yes - had heard of SI before	Coding phase: 2. Double coded	Client marital status: Married	Client age group: 30-39 years	Time since trained: N/A - client

Codes Applied:	C3.3 - Counselling / training approach for injectable users

Interviewer: Alright, did they explain to you about the problems that you might experience in your body when you are using the injectable?

Respondent: No they did not.

Title: ZA_IM_5.docx

Descriptor Info:	District: Zomba	Target Population: TP2 - DMPA-IM/DMPA-SC PI clients	Respondent type: DMPA-IM client	Aware of SI: Yes - had heard of SI before	Coding phase: 2. Double coded	Client marital status: Married	Client age group: 30-39 years	Time since trained: N/A - client

Codes Applied:	C3.3 - Counselling / training approach for injectable users

Interviewer: Mmmm (showing that one is following the conversation)

Respondent: They said that a self-injectable method has been introduced

Interviewer: Mmmm (showing that one is following the conversation)

Respondent: So we will bring this injectable so that you may see it.

Interviewer: You may see it

Respondent: You should see how you can use it, they encouraged us maybe for some months.

Interviewer: Mmmm (showing that one is following the conversation)

Respondent: Eh so when we went to the antenatal clinic, they would tell us

Interviewer: Alright ah were you given a chance to try it on your own on what you were trained? For example, practicing on a condom filled with sugar or soil, did they do this to you?

Respondent: Ah no

Title: ZA_IM_5.docx

Descriptor Info:	District: Zomba	Target Population: TP2 - DMPA-IM/DMPA-SC PI clients	Respondent type: DMPA-IM client	Aware of SI: Yes - had heard of SI before	Coding phase: 2. Double coded	Client marital status: Married	Client age group: 30-39 years	Time since trained: N/A - client

Codes Applied:	C3.3 - Counselling / training approach for injectable users

Interviewer: Alright, so they did not give you things like injection units to take back home for self-injection, or maybe calendar and a paper of instructions detailing how a woman can self-inject?

Respondent: The paper is what we saw.

Interviewer: Alright.

Respondent: Yeah because of a large number of clients so for them to give us all it was like, because we have a lot of people at the clinic.

Interviewer: Mmmm (showing that one is following the conversation)

Respondent: So they just sowed us the paper of instructions and we could see it.

Interviewer: Alright.

Respondent: Yes.

Title: ZA_IM_9.docx

Descriptor Info:	District: Zomba	Target Population: TP2 - DMPA-IM/DMPA-SC PI clients	Respondent type: DMPA-IM client	Aware of SI: Yes - had heard of SI before	Coding phase: 1. Single coded	Client marital status: Married	Client age group: 20-29 years	Time since trained: N/A - client

Codes Applied:	C3.3 - Counselling / training approach for injectable users

Interviewer: So how does it go, what do health counsellors do to help you out? When you get to the hospital, what do you do first?

Respondent: When we get at the hospital, they advise us then we go get assisted.

Title: ZA_IM_9.docx

Descriptor Info:	District: Zomba	Target Population: TP2 - DMPA-IM/DMPA-SC PI clients	Respondent type: DMPA-IM client	Aware of SI: Yes - had heard of SI before	Coding phase: 1. Single coded	Client marital status: Married	Client age group: 20-29 years	Time since trained: N/A - client

Codes Applied:	C3.3 - Counselling / training approach for injectable users	C1.1.3 - Awareness of DMPA-SC SI

Interviewer: Mmhmmm alright alright. Thank you so much. Now I would like to talk to you about a different type of injection contraception, which you might have heard of or not. Have you heard before of a contraception injection you inject yourself?

Respondent: We only hear about it.

Interviewer: Mmh.

Respondent: Mmh.

Interviewer: Alright. Where did you hear about it?

Respondent: We heard about it from this hospital,

Interviewer: Mmh.

Respondent: People say there is a new injection.

Interviewer: Sorry. What people?

Respondent: Doctors, they say you will be self-injecting.

Interviewer: Mmh.

Respondent: Mmh.

Interviewer: Do doctors advise?

Respondent: They do advise us.

Interviewer: Eeh.

Respondent: That there is a new injection you will have to inject yourselves with.

Interviewer: Mmh.

Respondent: Mmh.

Title: ZA_IM_9.docx

Descriptor Info:	District: Zomba	Target Population: TP2 - DMPA-IM/DMPA-SC PI clients	Respondent type: DMPA-IM client	Aware of SI: Yes - had heard of SI before	Coding phase: 1. Single coded	Client marital status: Married	Client age group: 20-29 years	Time since trained: N/A - client

Codes Applied:	C3.3 - Counselling / training approach for injectable users	C3.3.3 - Poor (insufficient/ judgemental) counselling/training

Interviewer: Alright. You talked about the self-injecting method. Did you talk with health counsellors about the self-injecting contraception method?

Respondent: No

Interviewer: You have not talked about it with your health counsellors?

Respondent: Mmh.

Interviewer: Mmhmm.

Title: MZs_IM_1.docx

Descriptor Info:	District: Mzimba South	Target Population: TP2 - DMPA-IM/DMPA-SC PI clients	Respondent type: DMPA-IM client	Aware of SI: Yes - had heard of SI before	Coding phase: 1. Single coded	Client marital status: Married	Client age group: 30-39 years	Time since trained: N/A - client

Codes Applied:	C1.1.3 - Awareness of DMPA-SC SI	C3.3 - Counselling / training approach for injectable users

Q9. Interviewer: Have you heard about a type of injectables contraceptive that a woman can inject herself (without a provider)?

Respondent: yes, I have heard about it

Interviewer: Where have you heard about it?

Respondent: I heard it from an HSA

Interviewer: What did you first heard about it?

Respondent: I took it as a good method though in my mind I already have Depo the one I have been using since, so I did not pay attention to it.

Interviewer: Do you know anyone who is using self-injection method?

Respondent: Yes.

Interviewer: You said you heard about self-injection from an HSA?

Respondent: Yes. Though we were talking about Depo, the method I am using, then the provider mention self-injection.

Interviewer: So you did not discuss it with the provider nut rather you just heard it from him?

Respondent: Yes, before they start providing the injection, they do the counseling. Counselling id done in all the methods that they provide and then as clients we choose the method that we want. But for those who have chosen self-injection they are the ones who undergo the process on how they can inject themselves and what they can expect as they are injecting themselves. For us who have chosen providers injection we don’t go for self-injection counseling thus why I am saying that I have never met the provider for self-injection method.

Interviewer: I want to have a clear understanding. You when the provider counsel family planning methods, she/he counsel different methods and after the counseling thus when the clients choose the method they want to use and for those who have chosen self-injection they are the ones who undergo the training session for them to learn how to inject themselves?

Respondent: Yes

Title: KK_IM_1.docx

Descriptor Info:	District: Nkhotakota	Target Population: TP2 - DMPA-IM/DMPA-SC PI clients	Respondent type: DMPA-IM client	Aware of SI: No - never heard of SI before	Coding phase: 3. Triple coded	Client marital status: Married	Client age group: Under 20 (adolescent)	Time since trained: N/A - client

Codes Applied:	C3.3 - Counselling / training approach for injectable users

INTERVIEWER: What happens at the facility/with ethe HSA? How much time does the provider spend with you and what do they say?

RESPONDENT: When we get inside to get injected,

INTERVIEWER: Yes

RESPONDENT: After we get injected, we are told not to have sex with our partner for 3 days

INTERVIEWER: Alright, so you get counselled when you visit the health center?

RESPONDENT: yes

INTERVIEWER: Alright, Do you get counselled by the HSA?

RESPONDENT: Yes

Title: ZA_SCPI_8.docx

Descriptor Info:	District: Zomba	Target Population: TP2 - DMPA-IM/DMPA-SC PI clients	Respondent type: DMPA-SC PI client	Aware of SI: Yes - had heard of SI before	Coding phase: 1. Single coded	Client marital status:	Client age group:	Time since trained: N/A - client

Codes Applied:	C3.3 - Counselling / training approach for injectable users

Interviewer: alright what happens when you get to the facility to get injected?

Respondent: when we get here, we find many people

Interviewer: mmmm (showing that one is following the conversation)

Respondent: so we wait until it’s our turn

Interviewer: mmmm (showing that one is following the conversation)

Respondent: we don’t go back without being assisted

Interviewer: alright

Respondent: we wait

Interviewer: How long do the providers take?

Respondent: they take up to 12 o’clock

Interviewer: mmmm (showing that one is following the conversation)

Respondent: yes

Interviewer: alright, how many minutes do you spend with them while they are helping you?

Respondent: maybe up to 3 minutes.

Interviewer: mmmm (showing that one is following the conversation)

Respondent: while they are helping us.

Title: ZA_SCPI_8.docx

Descriptor Info:	District: Zomba	Target Population: TP2 - DMPA-IM/DMPA-SC PI clients	Respondent type: DMPA-SC PI client	Aware of SI: Yes - had heard of SI before	Coding phase: 1. Single coded	Client marital status:	Client age group:	Time since trained: N/A - client

Codes Applied:	C3.3 - Counselling / training approach for injectable users

Interviewer: alright, you have told me that the providers told you about the self-injecting contraceptive

Respondent: yes

Interviewer: ah how many times have you talked about the self-injecting contraceptive with the providers?

Respondent: only once

Interviewer: alright.

Respondent: on the day that I was due for reinjection.

Interviewer: alright

Respondent: yes

Interviewer: Can you tell me more about your discussion?

Respondent: in this discussion I was just told that when I come again in October.

Interviewer: mmmm (showing that one is following the conversation)

Respondent: we will train you

Interviewer: mmmm (showing that one is following the conversation)

Respondent: we will train you on how you can self-inject.

Interviewer: alright.

Respondent: so when we went back, they told us that they are busy with some work.

Interviewer: mmmm (showing that one is following the conversation)

Respondent: so we were not able to get the injectable.

Interviewer: okay

Respondent: yes

Interviewer: Alright, did they tell you about the pros and cons of this injectable?

Respondent: they said that we can be able to use the injectable for 5 years.

Interviewer: Alright, did you ever feel judged in these conversations?

Respondent: no I did not feel judged in any way.

Interviewer: alright.

Respondent: yes

Interviewer: Alright, as a result of the discussion that you had with the provider did you agree to try self-injection?

Respondent: I just agreed that in the coming month you will train me and give me to try it.

Interviewer: alright.

Respondent: so they said that yeah that’s possible,

Title: ZA_SCPI_8.docx

Descriptor Info:	District: Zomba	Target Population: TP2 - DMPA-IM/DMPA-SC PI clients	Respondent type: DMPA-SC PI client	Aware of SI: Yes - had heard of SI before	Coding phase: 1. Single coded	Client marital status:	Client age group:	Time since trained: N/A - client

Codes Applied:	C3.3 - Counselling / training approach for injectable users

Interviewer: Alright, as far as you remember, did the provider teach you anything that was not in the video?

Respondent: eyes for the injectable they just told us that when you inject yourself…

Interviewer: mmmm (showing that one is following the conversation)

Respondent: so we asked them, can we be able to self-inject? So they told us that they will plan to give us thorough training…

Interviewer: mmmm (showing that one is following the conversation)

Respondent: when we train you, you can be self-injecting, if you have fears then you can tell anyone at home to inject you.

Interviewer: alright

Respondent: you will inject yourself either on the thigh or on the stomach area

Interviewer: mmmm (showing that one is following the conversation)

Respondent: yes

Interviewer: alright

Respondent: yes

Title: MZs_SCPI_3.docx

Descriptor Info:	District: Mzimba South	Target Population: TP2 - DMPA-IM/DMPA-SC PI clients	Respondent type: DMPA-SC PI client	Aware of SI: Yes - had heard of SI before	Coding phase: 1. Single coded	Client marital status: Married	Client age group: 20-29 years	Time since trained: N/A - client

Codes Applied:	C3.3 - Counselling / training approach for injectable users

Interviewer: What happens at the facility?

Respondent: We do tell them that we have come for family planning, and they do give us.

Interviewer: Mmmmh (okay)

Respondent: And also, they do partial counselling and ask us some questions and we do tell them our experience upon using it.

Interviewer: Anything else?

Respondent: Nothing.

Title: MZs_SCPI_3.docx

Descriptor Info:	District: Mzimba South	Target Population: TP2 - DMPA-IM/DMPA-SC PI clients	Respondent type: DMPA-SC PI client	Aware of SI: Yes - had heard of SI before	Coding phase: 1. Single coded	Client marital status: Married	Client age group: 20-29 years	Time since trained: N/A - client

Codes Applied:	C3.3 - Counselling / training approach for injectable users

Interviewer: Do you know anyone using self- injection?

Respondent: No, because we are coming from different areas.

Q10. Interviewer: How many times have discussed self-injection method with the provider?

Respondent: Three times.

Interviewer: Can you tell me more about the conversation?

Respondent: The doctor said that if we agree that we can self-inject then they are going to give us one packet of Sayana to inject ourself, right before him, then after seeing that we have done it well, he is going to give us 3 packets of Sayana for us to take home so that we can be injecting ourself after 3 months up until we finish all the 3 packets and come back to the facility for refill.

Interviewer: Did the provider tell you on how you can keep it?

Respondent: Yes, he did, he said that we should be keeping it at a safe place where no one can access it.

Interviewer: Why did he say that you should keep it where no one can see it?

Respondent: So that the children cannot have access to it and play with it which is not good.

Interviewer: Did they provider tell you on how you can dispose used syringe?

Respondent: He said that we should be throwing ii inside the toilet

Interviewer: He said that you should be throwing inside the toilet?

Respondent: Yes.

Interviewer: Did you ever feel judged during these conversations?

Respondent: No

Q11. Interviewer: Then why didn’t you go for it?

Respondent: The reason is that by that time I was afraid that I cannot be able to do it.

Interviewer: Okay.

Respondent: And also the only thing I knew was that it’s only the health workers who can inject us.

Interviewer: But the provider explained to you on how you can self-inject?

Respondent: Mmmmmh (yes)

Interviewer: Then why didn’t you try self-injection?

Respondent: It was because I was afraid to self-inject.

Interviewer: Did you tell him that you had fear so that he would have repeated the process?

Respondent: No

Interviewer: But you was afraid?

Respondent: Yes. Thinking that after injecting ourself, maybe the medicine will not come out.

Interviewer: Was that the reason why you did not opt for self-injection?

Respondent: Yes.

Interviewer: Are you still scared?

Respondent: No, right now I don’t have any fear.

Interviewer: And if I can give you to go and do it at home, are you going to accept it?

Respondent: Yes, I can.

Interviewer: Why do you want to try now?

Respondent: Because I have been seeing the way the doctors do it.

Interviewer: At first, didn’t you see them?

Respondent: I was but I was not paying attention and am sure I can do it.

Interviewer: Alright.

Title: MZs_IM_4.docx

Descriptor Info:	District: Mzimba South	Target Population: TP2 - DMPA-IM/DMPA-SC PI clients	Respondent type: DMPA-IM client	Aware of SI: Yes - had heard of SI before	Coding phase: 2. Double coded	Client marital status: Married	Client age group: 30-39 years	Time since trained: N/A - client

Codes Applied:	C3.3 - Counselling / training approach for injectable users

Interviewer: What happens at the facility?

Respondent: When we reach here as a family planning day, we usually wait-there is a waiting place.

Interviewer: Mmmmmh….(following).

Respondent: The first thing we do is that we wash our hands and then put on our mask, and we go sit on the waiting room - where the providers get our health passport books while asking when was the last time, we get the injection and also for how long have we been using the method and then we get the injection. Lastly, they do ask if we have ever met any problem with the method, if the answer is no, then they do document in our health passport book the next date we are supposed to visit.

Interviewer: You said they do ask you if you have ever met any problem with the method, so if you say yes, you are experiencing some problems with the method, what do they do?

Respondent: If we say yes, they do help us by giving us the medication.

Interviewer: How much time does the provider spend with you?

Respondent: Its less than an hour.

Title: MZs_IM_4.docx

Descriptor Info:	District: Mzimba South	Target Population: TP2 - DMPA-IM/DMPA-SC PI clients	Respondent type: DMPA-IM client	Aware of SI: Yes - had heard of SI before	Coding phase: 2. Double coded	Client marital status: Married	Client age group: 30-39 years	Time since trained: N/A - client

Codes Applied:	C3.3 - Counselling / training approach for injectable users

Interviewer: Did the provider explain to you how this Sayana injection works?

Respondent: Mmmmm.(yes)

Interviewer: Can you tell me more about the conversation you had with the provider?

Respondent: The provider said that with this method you can only inject yourself in the upper leg (patchafu) and around the stomach.

Interviewer: Did the provider tell you the good things about this method?

Respondent: The provider said that this method is very good that a woman can self-inject herself at home without facing any problem

Interviewer: Mmmmmh…(following)

Respondent: And he also said that after injection we can throw the used things inside the toilet

Interviewer: Was it the provider who said that?

Respondent: Yes, he said we should be throwing it inside the toilet or we can find something and put the used thing inside and then we dig a pit and put it there.

Interviewer: What else did the provider tell you about self-injection method?

Respondent: You mean what we need to do?

Interviewer: Yes, what you need to do?

Respondent: The provider said that this Sayana method is very [missing word] and a woman can do it at home freely.

Interviewer: Mmmmh (following),

Respondent: Yes

Interviewer: So did you feel ever judged during these conversations?

Respondent: No, actually I decided to use it so that I should have a feel on how it works.

Q11. Interviewer: As a result of the discussion you had with the provider, did you agree to try self-injection?

Respondent: Yes, I did though it was only once.

Interviewer: Mmmmmh….(following)

Respondent: Yes

Interviewer: Then why did you stop using it?

Respondent: After using the first Sayana that I was given, I went back to the provider to get another doses but I was told that Sayana is out of stock, and I came back the other day and I was also told the same thing, then I later decided that I should just get Depo, for me to avoid pregnancy.

Q12. Interviewer: Can you describe your experience of being trained in self-injection?

Respondent: You mean the good ones or the bad ones?

Interviewer: Both good and the bad ones

Respondent: The good thing about this method is that it gives a woman time to other things freely

Interviewer: Mmmmmh (following)

Respondent: And the bad thing, I don’t know because I only used it once

Interviewer: Did the provider spent enough time in training you?

Respondent: Yes, he did.

Interviewer: Did you get the chance to practice?

Respondent: Yes

Interviewer: How did you practice the method?

Respondent: One woman volunteers herself that we should practice on her body

Interviewer: Did the provider gave you a condom filled with sand or sugar for you inject as part of the training session?

Respondent: Yes, he did.

Interviewer: Explain what happened?

Respondent: He took a condom and it was filled with sand

Interviewer: And after that what did he do?

Respondent: Then he took a syringe and inject the condom that was filled with sand.

Interviewer: You have said that you’re one of the women volunteered herself that you should practice on her body, so between the condom and the women which one did the provider started with?

Respondent: He started with the condom that was filled with sand and then he ended with the woman.

Interviewer: Would you describe the friendship of the training?

Respondent: You meant the way we were trained with Sayana?

Interviewer: Yes

Respondent: We learnt that this Sayana self-injection method is just as the same as Depo, only that the syringe for Sayana is very small

Interviewer: Really?

Respondent: Yes

Interviewer: Do you have anything else to add?

Respondent: (Quiet………) If we are doing it at home, we have enough time to shake the medicine very well and do the injection.

Title: KK_IM_2.docx

Descriptor Info:	District: Nkhotakota	Target Population: TP2 - DMPA-IM/DMPA-SC PI clients	Respondent type: DMPA-IM client	Aware of SI: Yes - had heard of SI before	Coding phase: 2. Double coded	Client marital status: Married	Client age group: 30-39 years	Time since trained: N/A - client

Codes Applied:	C3.3 - Counselling / training approach for injectable users

INTERVIEWER: When you get to the hospital, what happens? The day for an injection like today, you came here today for an injection, right?

RESPONDENT: mmmh.

INTERVIEWER: What happens?

RESPONDENT: When we get here at the hospital, the doctor gets us…the doctor gets the cards. And they check the date. Once the date is checked, you are told whether it’s truly your date for an injection or not. If it’s not your date they tell you to come back on that date and if it is your date, they tell you that you will be injected.

INTERVIEWER: okay. Now the whole process after confirming your date, how does it progress thereafter?

RESPONDENT: They also tell us the other date for us to come for another injection.

INTERVIEWER: Eh they tell you another date.

RESPONDENT: They write the date in … for example today is fourteenth, when it is a self-injecting method, right? When it is a self-injecting method, they make sure to tell you that you should come back on fourteenth for another injection. While the method that the doctor injects us, they alternate the dates. Let us say they inject us today on fourteenth, maybe they will inject you again on 22 January.

INTERVIEWER: Okay, alright. On this one, what I mainly want to know is the assistance that you get when you come here. What really happens up to the point when you are injected and ready to go back home.

RESPONDENT: The doctor welcomes us very well.

INTERVIEWER: mmmhu. What happens?

RESPONDENT: (laughs).

INTERVIEWER: They welcome you well, but what happens? You get here, they collect the cards then thereafter?

RESPONDENT: After the injection, they advise us

INTERVIEWER: Yes, tell me that

RESPONDENT: (laughs).

INTERVIEWER: (laughs) Tell me that

RESPONDENT: They tell us, the injection I have given you, you should take three days before having sex.

INTERVIEWER: okay.

RESPONDENT: mmmhu.

INTERVIEWER: When you come here, how long does it take you to be assisted?

RESPONDENT: No, we do not take long here.

INTERVIEWER: You do not take long?

RESPONDENT: mmmh.

Title: KK_IM_2.docx

Descriptor Info:	District: Nkhotakota	Target Population: TP2 - DMPA-IM/DMPA-SC PI clients	Respondent type: DMPA-IM client	Aware of SI: Yes - had heard of SI before	Coding phase: 2. Double coded	Client marital status: Married	Client age group: 30-39 years	Time since trained: N/A - client

Codes Applied:	C3.3 - Counselling / training approach for injectable users

INTERVIEWER: We are continuing. Now… we would like to discuss another injection contraceptive method; you might have heard about it or not.

RESPONDENT: mmmmmhu.

INTERVIEWER: Now, have you ever heard about the self-injecting contraceptive method that women inject themselves at home?

RESPONDENT: Yes, I have heard about it.

INTERVIEWER: Where did you hear about it from?

RESPONDENT: I heard about from the area I come from.

INTERVIEWER: In your area?

RESPONDENT: mmmmhu.

INTERVIEWER: What were they saying?

RESPONDENT: aaah, they were saying the self-injecting method, you are given an injection at the hospital, right. When they give you, the one to inject yourself, you open the whatchamacallit, the one that you sort of press. And inject yourself, where you inject it on your thigh or here.

INTERVIEWER: Mmmh. That is what the people in your area were saying? Now as for you, is there anyone you know using this method? Anyone using this method?

RESPONDENT: Someone, my neighbor in the village.

INTERVIEWER: Your neighbor?

RESPONDENT: mmmh.

INTERVIEWER: Okay. have you ever discussed this method before, whether today or sometime back with a doctor or an HSA?

RESPONDENT: Aaah no. As I have already said that right here the doctors just tries to enlighten us.

INTERVIEWER: Do they tell you about how this method works?

RESPONDENT: Yes.

INTERVIEWER: Okay. How many times has the doctor told you about this method?

RESPONDENT: Aah for me I heard about it once. Because the time I was pregnant, I moved to salima. So I heard about it here once.

INTERVIEWER: Only once, you heard the doctor talking about it? Telling women about it?

RESPONDENT: (laughs) Yes. He was advising us

(20:00)

INTERVIEWER: When advising you, what was the doctor saying?

RESPONDENT: The doctor was saying, I will be explaining right?

INTERVIEWER: Ye, I would like to know everything that you were told.

RESPONDENT: (laughs). The doctor was advising us to saying, being pregnant as you are, it is not proper to just stay home after delivery knowing well that you easily get pregnant. It is good for you if you know that you easily get pregnant to come to the hospital the moment you note your first period after delivery, at the hospital sometimes they say after six weeks you should go and get contraceptives. Now as a woman, you choose your method. There is an injection to inject yourself at home. When you come here at the hospital, we will give you an injection to inject yourself at home. You will inject yourself whether on your thigh or here. Yes, whether you want to put a Norplant for three or five years, whether you want the doctor to inject you, when we inject you, it takes three months for you to receive another injection.

INTERVIEWER: mmmhu. Okay, now as they were telling you about Sayana, they told you about its advantages right, did they tell about the good and bad sides of Sayana?

RESPONDENT: Yes, we were told that the advantages of Sayana and this other injection are not exactly different.

INTERVIEWER: With the other injection?

RESPONDENT: mmmh, not really different.

INTERVIEWER: mmmhu. The advantages and disadvantages are all the same.

RESPONDENT: yes, yes.

INTERVIEWER: Oh, they said so?

RESPONDENT: Yes

INTERVIEWER: Okay. as they were telling you this, did you feel judged, when I say judged you know what I mean right? May be to say, they are mocking me or what?

RESPONDENT: okay.

INTERVIEWER: mmmh. Did you have any feeling of being judged?

RESPONDENT: No, there is nothing.

INTERVIEWER: Nothing, it was okay?

RESPONDENT: mmmh.

Title: KK_IM_2.docx

Descriptor Info:	District: Nkhotakota	Target Population: TP2 - DMPA-IM/DMPA-SC PI clients	Respondent type: DMPA-IM client	Aware of SI: Yes - had heard of SI before	Coding phase: 2. Double coded	Client marital status: Married	Client age group: 30-39 years	Time since trained: N/A - client

Codes Applied:	C2.2.1 - Stockouts/stocks near expiry dates	C3.3 - Counselling / training approach for injectable users

INTERVIEWER: mmmhu. You have told me that you had discussed with the doctor and the HSAs right. Did you try to inject yourself to accept that you will take this method?

RESPONDENT: No

INTERVIEWER: Why?

RESPONDENT: I did not find it at the hospital that time.

INTERVIEWER: It was not there…

RESPONDENT: The self-injecting one.

INTERVIEWER: Okay…you should have…

RESPONDENT: The one I found is the one for you to be injected by the doctor

INTERVIEWER: Okay. Had you found it…

RESPONDENT: I would have taken it.

INTERVIEWER: You would have taken it?

RESPONDENT: mmmh.

INTERVIEWER: Now, what did the doctor tell you? Did you tell the doctor that you would want to take?

RESPONDENT: Yes. I had wanted to change the method right. I wanted to get a five-year Norplant, but the five-year Norplant was not available as well at that time. Then I said I would get the self-injecting one which they said the stock had ended the day before.

INTERVIEWER: and they…

RESPONDENT: And the one available is for you to be injected right here, so I waited, and I was injected.

INTERVIEWER: Did the doctor say when the injection you wanted would be available, what did the doctor say?

RESPONDENT: The doctor said I would be called to come get it whenever another stock arrived.

Title: KK_IM_2.docx

Descriptor Info:	District: Nkhotakota	Target Population: TP2 - DMPA-IM/DMPA-SC PI clients	Respondent type: DMPA-IM client	Aware of SI: Yes - had heard of SI before	Coding phase: 2. Double coded	Client marital status: Married	Client age group: 30-39 years	Time since trained: N/A - client

Codes Applied:	C3.3 - Counselling / training approach for injectable users	C3.3.3 - Poor (insufficient/ judgemental) counselling/training

INTERVIEWER: The lessons…the doctor already gave you. Again, here you have watched the video right. Is there anything new in the video that you were not taught? Okay, I should not say the doctor only, is there anything that you have never heard whether at home that is in the video?

RESPONDENT: Yes, it is there

INTERVIEWER: what is that?

RESPONDENT: Aaah I did not know that you are given the self-injecting injection after 9 months, I thought they give you after a year. I had never heard about that before. I thought, once you are given the injections at the hospital it takes a year before you go back. That part which I already explained that if one comes far away from the hospital, you save distance.

INTERVIEWER: Distance

RESPONDENT: Yes, but I did not know that it is 9 months.

INTERVIEWER: mmmmhu.

RESPONDENT: mmmh.

INTERVIEWER: okay, is there anything else, that you have never heard about but is in the video?

RESPONDENT: Nothing else, that is all.

INTERVIEWER: That is all?

RESPONDENT: mmmmh.

Title: KK_IM_8.docx

Descriptor Info:	District: Nkhotakota	Target Population: TP2 - DMPA-IM/DMPA-SC PI clients	Respondent type: DMPA-IM client	Aware of SI: No - never heard of SI before	Coding phase: 1. Single coded	Client marital status: Married	Client age group: 20-29 years	Time since trained: N/A - client

Codes Applied:	C3.3 - Counselling / training approach for injectable users

INTERVIEWER: Mmmhu. What happens when you arrive at the hospital? It’s your day to get an injection, what happen when you arrive?

RESPONDENT: The nurses are the ones who inject us and sometimes we find them doing other work. There are four days in a week for family planning. The days are Wednesday, Thursday…oh the days should be three. From Wednesday to Friday. And on these days, on Wednesday, there are also other activities, on Thursday its under-five clinic and other activities. And when we arrive and tell them that we are here for contraceptives, they tell us to wait for them because they are doing other things. And sometimes we go home late because we were waiting for them to finish what they were doing.

INTERVIEWER: okay. How long do you wait for them?

RESPONDENT: Sometimes we wait for an hour.

INTERVIEWER: Okay. So, how do they assist you when you arrive? Can you explain to me what happens up to the point of you being injected?

RESPONDENT: When we arrive, they give us a place to sit. Everyone who has come for family planning is supposed to sit there. We wait until they finish what they are doing for us to get help.

INTERVIEWER: Mmmhu. How do they help you?

RESPONDENT: One person at a time goes in the room.
