# Supplementary material for: Health care provider and client experiences of counselling on depot medroxyprogesterone acetate subcutaneous (DMPA-SC) for self-injection in Malawi
Source: PLOS Glob Public Health. 2023 Nov 30;3(11):e0002057. doi: 10.1371/journal.pgph.0002057 (PMC10688740; doi:10.1371/journal.pgph.0002057)
Supplement: S1 Data — (ZIP) [file pgph.0002057.s001.zip › S2/C3.3 couns vs video SI only.doc]

Title: KK_SCSI_5.docx

Descriptor Info:	District: Nkhotakota	Target Population: TP3 - DMPA-SC SI clients	Respondent type: DMPA-SC SI client	Aware of SI: Yes - had heard of SI before	Coding phase: 2. Double coded	Client marital status: Married	Client age group: 20-29 years	Time since trained: N/A - client

Codes Applied:	C3.3 - Counselling / training approach for injectable users	C3.3.2 - Good (sufficient/ non-judgemental) counselling/training

Alright. Did you fee judged during your training? Judged it’s like someone talks bad of you. Did anything like that happen?

RESPONDENT: No

Title: MZs_SCSI_3.docx

Descriptor Info:	District: Mzimba South	Target Population: TP3 - DMPA-SC SI clients	Respondent type: DMPA-SC SI client	Aware of SI: Yes - had heard of SI before	Coding phase: 1. Single coded	Client marital status: Married	Client age group: 30-39 years	Time since trained: N/A - client

Codes Applied:	C1.1.3 - Awareness of DMPA-SC SI	C3.3 - Counselling / training approach for injectable users

Interviewer: now I would like us to discuss about your experience with self-injection. Where did you first heard about Sayana, self-injection?

Respondent: it was here at the facility on the day we came for Depo IM. They said it is the same as Depo IM.

Title: MZs_SCSI_3.docx

Descriptor Info:	District: Mzimba South	Target Population: TP3 - DMPA-SC SI clients	Respondent type: DMPA-SC SI client	Aware of SI: Yes - had heard of SI before	Coding phase: 1. Single coded	Client marital status: Married	Client age group: 30-39 years	Time since trained: N/A - client

Codes Applied:	C3.3 - Counselling / training approach for injectable users

nterviewer: would you tell me your experience with what you were taught on self-injection?

Respondent: the counselling was all well it’s just us we had fears that it cannot be possible to self-inject. Like for me I asked them to inject me because I was not confident. But I was told it was meant for self-injection. During the second visit, they counselled us again and this time I understood and managed to self-inject

Interviewer: how did they say you should self-inject?

Respondent: on the calendar they said we should turn it and touch on the cup. Then we should shake it until it mixes well. Then we press on the neck until it produces a sound ‘theee’. Then to inject on the thighs or on the sides of the abdomen. We pinch the skin and then inject.

Interviewer: then?

Respondent: we were told to discard in a tin with a lid. After all, doses we should give the tin to the provider for safe disposal

Title: MZs_SCSI_3.docx

Descriptor Info:	District: Mzimba South	Target Population: TP3 - DMPA-SC SI clients	Respondent type: DMPA-SC SI client	Aware of SI: Yes - had heard of SI before	Coding phase: 1. Single coded	Client marital status: Married	Client age group: 30-39 years	Time since trained: N/A - client

Codes Applied:	C3.3 - Counselling / training approach for injectable users

[video plays]

Interviewer: Is there anything new in the video?

Respondent: yes

Interviewer: what is new?

Respondent: she was using a paper to teach

Title: MZs_SCSI_3.docx

Descriptor Info:	District: Mzimba South	Target Population: TP3 - DMPA-SC SI clients	Respondent type: DMPA-SC SI client	Aware of SI: Yes - had heard of SI before	Coding phase: 1. Single coded	Client marital status: Married	Client age group: 30-39 years	Time since trained: N/A - client

Codes Applied:	C3.3 - Counselling / training approach for injectable users

Interviewer: after watching the video, what do you think about the counselling you received?

Respondent: our friend was taught on what we are supposed to know

Title: MZs_SCSI_1.docx

Descriptor Info:	District: Mzimba South	Target Population: TP3 - DMPA-SC SI clients	Respondent type: DMPA-SC SI client	Aware of SI: Yes - had heard of SI before	Coding phase: 1. Single coded	Client marital status: Married	Client age group: Under 20 (adolescent)	Time since trained: N/A - client

Codes Applied:	C3.3 - Counselling / training approach for injectable users

Q8. Interviewer: Where did you first hear about contraceptive methods that you could inject yourself and what was your reaction

Respondent: I heard from doctors who came from Mzimba hospital

Interviewer: You have said that you heard from doctors who came from Mzimba district can you tell me more what happened?

Respondent: The doctors came and put us into different groups, and they asked if there was someone who wanted family planning and they asked us ‘what type of family planning do you want’ and then women mentioned different methods of family planning and as for me I choose injectables

Interviewer: Mmmmh (following)

Respondent: Then they gave us one injectable to us to inject ourselves while they were watching so that they should see if we’re doing it right, and they gave me 3 more injectable for me to us at home

Title: MZs_SCSI_1.docx

Descriptor Info:	District: Mzimba South	Target Population: TP3 - DMPA-SC SI clients	Respondent type: DMPA-SC SI client	Aware of SI: Yes - had heard of SI before	Coding phase: 1. Single coded	Client marital status: Married	Client age group: Under 20 (adolescent)	Time since trained: N/A - client

Codes Applied:	C3.3 - Counselling / training approach for injectable users

Q9. Interviewer: Can you explain your experience if being trained in self – injection, to start please explain who trained you?

Respondent: Mmmmh, as of now I can’t remember all because it’s been long

Interviewer: Anything you can remember?

Respondent: I only remember the way they said we can open the syringe and also how we can inject ourselves

Interviewer: What did they say about it?

Respondent: They said that before we start anything we need to wash hands and then we should take the pack of the syringe (Sayana) and tear it and then take the needle and before we inject ourselves, we need to shake the syringe and find the place where you want to inject either around the stomach or upper leg

Title: KK_SCSI_2.docx

Descriptor Info:	District: Nkhotakota	Target Population: TP3 - DMPA-SC SI clients	Respondent type: DMPA-SC SI client	Aware of SI: Yes - had heard of SI before	Coding phase: 1. Single coded	Client marital status: Divorced/ separated/ widowed	Client age group: 20-29 years	Time since trained: N/A - client

Codes Applied:	C1.1.3 - Awareness of DMPA-SC SI	C3.3 - Counselling / training approach for injectable users

INTERVIEWER: Where did you first hear about contraceptives methods that you could inject yourself , and what was your reaction. Firstly where did you hear that.

RESPONDENT: Firstly I heard from the hospital

INTERVIEWER: Were you counselled here at the hospital?

RESPONDENT: Yes, we were advised that there is a new FP method where one can inject herself

Title: KK_SCSI_2.docx

Descriptor Info:	District: Nkhotakota	Target Population: TP3 - DMPA-SC SI clients	Respondent type: DMPA-SC SI client	Aware of SI: Yes - had heard of SI before	Coding phase: 1. Single coded	Client marital status: Divorced/ separated/ widowed	Client age group: 20-29 years	Time since trained: N/A - client

Codes Applied:	C3.3 - Counselling / training approach for injectable users	C3.3.2 - Good (sufficient/ non-judgemental) counselling/training	C3.1.1 - Practice of SI during training	C3.4 - Time to train clients

NTERVIEWER: Thank you. The next question is, would you please describe your experience of being trained in self injection? To start with please describe who trained you.

RESPONDET: I was trained by the HSA

INTERVIEWER: It was the HSA not so

RESPONDENT: Yes

INTERVIEWER: So would you please describe what has been your experience of being trained in self-injection

RESPONDENT: We were told that after injecting ourselves we need to keep the injections safe. After injecting ourselves we should put the injection in a bottle, then after finish all the dosage we should take back to the hospital as we go to collect the next injection, to show the health personnel to prove that we used it instead of keeping it and deny the opportunity for others to access the injection.

INTERVIEWER: What did the HSA explain about self-injection, I think you have already explained something on this one

RESPOINDENT : Yes

INTERVIEWER: Is there anything that you forgot? that the HSA explained to you about self-injection

RESPONDENT: How to hold it

INTERVIEWER: yes

RESPONDENT: This is how we hold Sayana (showing the interviewer using hands) then we shake it, after shaking it we press its neck and then the needle is pushed inside then we hold the place where we want to inject ourselves and then we inject the needle. The moment we rearise that the needle is inside the skin then we press the medicine until all is finished then we start pulling out the needle little by little.

INTERVIEWER: Thanks you so much this is well explained.

RESPONDENT: Thanks

INTERVIEWER: Did you feel the provider spent enough time training you?

REPONDENT: No we were trained twice

INTERVIEWER: they just trained you twice, but was it enough time?

RESPONDENT: Yes, it was enough time

INTERVIEWER: Maybe it was just, they just took some time

RESPONDENT: Yes

INTERVIEWER: Did you get the chance to practice what you were trained?

RESPONDNET: Yes

Title: KK_SCSI_3.doc

Descriptor Info:	District: Nkhotakota	Target Population: TP3 - DMPA-SC SI clients	Respondent type: DMPA-SC SI client	Aware of SI: Yes - had heard of SI before	Coding phase: 1. Single coded	Client marital status: Married	Client age group: 20-29 years	Time since trained: N/A - client

Codes Applied:	C3.3 - Counselling / training approach for injectable users

INTERVIEWER: Go [talking to the respondent]. Okay, can you describe your experience in being trained in self-injection. you should tell me who trained you and how the training was conducted.

RESPONDENT: An HSA from our community trained us. And the training was good.

INTERVIEWER: okay.

RESPONDENT: Yes. There was nobody who said otherwise.

INTERVIEWER: When you say the training was good, what made it to be good?

RESPONDENT: What we heard about the Sayana method, we thought that it was good.

INTERVIEWER: Mmmhu. What did the HSA explain to you?

RESPONDENT: The HSA explained that it’s the same with Depo injection there is no difference because the amount of drugs is the same.

INTERVIEWER: okay.

RESPONDENT: yes

INTERVIEWER: then

RESPONDENT: (laughs). yes

INTERVIEWER: After that, what did he/she do?

RESPONDENT: after that?

INTERVIEWER: mmmh. After the HSA explained that, what did he do?

RESPONDENT: He/she encouraged us to not be afraid of Sayana because if you have no side effects with Depo, it will be the same with Sayana. There is no difference. And encouraged women to go for Sayana.

INTERVIEWER: that one.

RESPONDENT: that one.

INTERVIEWER: what happened when you came here?

RESPONDENT: When I came here for my injection, there was a stock out of Depo and Sayana was available. I told myself that I will not go back because there is no Depo. Just give me Sayana I will self-inject.

INTERVIEWER: mmmhu.

RESPONDENT: yes.

INTERVIEWER: you self-injected?

RESPONDENT: mmmh.

INTERVIEWER: How did they train you? Who trained you here? The first place was for an HSA, and then you came here. Who trained you?

RESPONDENT: I don’t know the ones who trains us here.

INTERVIEWER: mmmmh.

RESPONDENT: yes.

INTERVIEWER: what were they saying?

RESPONDENT: About Sayana, they said that it is the same training from the HSA, there is no difference. But they said that it differs from person to person, that was the only difference. Some do heavy periods while others they don’t. I self-injected and I was worried that I will have long period. And then everything was okay until my next visit date.

INTERVIEWER: mmmhu.

RESPONDENT: sure.

INTERVIEWER: So the actual training on self-injection, what did they say? What happened?

RESPONDENT: They said we hold this thing and then we shake. After shaking you hold your skin where it is soft. Whether you will inject here or here, it is up to you [pointing the thigh and area around belly button]. When you hold, take the thing and inject and squeeze for all the drugs to go in.

INTERVIEWER: mmmhu.

RESPONDENT: yes.

INTERVIEWER: and then you did that?

RESPONDENT: yes (giggles).

Title: KK_SCSI_3.doc

Descriptor Info:	District: Nkhotakota	Target Population: TP3 - DMPA-SC SI clients	Respondent type: DMPA-SC SI client	Aware of SI: Yes - had heard of SI before	Coding phase: 1. Single coded	Client marital status: Married	Client age group: 20-29 years	Time since trained: N/A - client

Codes Applied:	C3.3 - Counselling / training approach for injectable users	C3.3.2 - Good (sufficient/ non-judgemental) counselling/training	C3.1.1 - Practice of SI during training

NTERVIEWER: You came here right, and you were trained…the training from the HSA was all about Sayana and what it’s all about and here you were actually trained on how you can self-inject. Did you get the chance to practice on what you were trained about?

RESPONDENT: mmmmh.

INTERVIEWER: what were you doing in this practice session?

RESPONDENT: (laughs). Practicing what they were doing.

INTERVIEWER: mmmhu.

RESPONDENT: hold like this and then inject.

INTERVIEWER: Ok. Did they give you a chance to…because when others are conducting the training, they use a condom filled with sugar as a thigh or around the abdomen and a person should try on that. Did that happen?

RESPONDENT: mmmh mmmh[no].

INTERVIEWER: Did they train you using the condom or the actual thigh?

RESPONDENT: real thigh.

INTERVIEWER: yours?

RESPONDENT: yes

INTERVIEWER: it was a group session or one to one?

RESPONDENT: one to one.

INTERVIEWER: one to one?

RESPONDENT: mmmh.

INTERVIEWER: okay. When they were training you, were you judged, when I say judged do you know the meaning?

RESPONDENT: mmmh mmmh [no]

INTERVIEWER: Maybe mocking you. Did you feel any judgement during the training?

RESPONDENT: no.

INTERVIEWER: everything went well?

RESPONDENT: mmmmh.

INTERVIEWER: okay. So they gave you a chance to try self-injection and you did very well, right?

RESPONDENT: mmmmh.

Title: KK_SCSI_4.docx

Descriptor Info:	District: Nkhotakota	Target Population: TP3 - DMPA-SC SI clients	Respondent type: DMPA-SC SI client	Aware of SI: Yes - had heard of SI before	Coding phase: 2. Double coded	Client marital status: Married	Client age group: Under 20 (adolescent)	Time since trained: N/A - client

Codes Applied:	C3.3 - Counselling / training approach for injectable users	C3.1.1 - Practice of SI during training

INTERVIEWER: Alright, as we continue with our discussions; would you please describe your experience of being trained in self-injection? How did the provider describe self-injection to you?

RESPONDENT: They said get the injection tear it and shake it well and press at the middle and remove the cover in the front and then inject yourself on the thigh.

INTERVIEWER: Is that what they said?

RESPONDENT: After inject yourself then press squeeze the medicine and pull the injection slowly and release the thigh.

INTERVIEWER: Ok that’s what was explained?

RESPONDENT: Yes

INTERVIEWER: Who trained you the doctor at the facility or the HSA?

RESPONDENT: The HSA

Time stamp: (20 minutes 5 seconds)

INTERVIEWER: the HSA? Alright, did you feel the provider spent enough time training you?

RESPONDENT: No they didn’t take too much time

INTERVIEWER: They didn’t too much time, did you get the chance to practice?

RESPONDENT: yes

INTERVIEWER: You got the chance?

RESPONDENT: Yes

INTERVIEWER: For you to try and you did it?

RESPONDENT: Yes

INTERVIEWER: Please raise your voice, did you ever feel judged during the training?

RESPONDENT: (Participant not sure)

INTERVIEWER: Did you ever feel judged during the training

RESPONDENT: No

Title: KK_SCSI_4.docx

Descriptor Info:	District: Nkhotakota	Target Population: TP3 - DMPA-SC SI clients	Respondent type: DMPA-SC SI client	Aware of SI: Yes - had heard of SI before	Coding phase: 2. Double coded	Client marital status: Married	Client age group: Under 20 (adolescent)	Time since trained: N/A - client

Codes Applied:	C3.3 - Counselling / training approach for injectable users

NTERVIEWER: No I would like to hear what you teach them form what you have seen from the video

RESPONDENT: (Participants seems not interested to answer)

INTERVIEWER: What is it that was explained?

RESPONDENT: They said, we hold the injection and tear it form an open and then we shake it

INTERVIEWER: Yes

RESPONDENT: If the medicine is spilling then we are supposed to put back in the paper and take it back to the provider for an exchange.

Title: ZA_SCSI_3.docx

Descriptor Info:	District: Zomba	Target Population: TP3 - DMPA-SC SI clients	Respondent type: DMPA-SC SI client	Aware of SI: Yes - had heard of SI before	Coding phase: 3. Triple coded	Client marital status: Married	Client age group: 40-49 years	Time since trained: N/A - client

Codes Applied:	C3.1.1 - Practice of SI during training	C3.1.2 - Confidence/comfort to inject after counselling/ training	C3.3 - Counselling / training approach for injectable users

Interviewer: Okay. So when did you start using the self-injection method?

Respondent: I started in 2020 after we were given the chance to learn about self-injection and I attempted it right there where it was a success so I was told that if I meet any problems, I should call the hospital. The following day after I was given the medication, the health official called me and asked me if I had come across any problems, I told her that I didn’t meet any problems. So she told me to go to the hospital so that they can give me more injections.

Title: ZA_SCSI_3.docx

Descriptor Info:	District: Zomba	Target Population: TP3 - DMPA-SC SI clients	Respondent type: DMPA-SC SI client	Aware of SI: Yes - had heard of SI before	Coding phase: 3. Triple coded	Client marital status: Married	Client age group: 40-49 years	Time since trained: N/A - client

Codes Applied:	C3.3 - Counselling / training approach for injectable users	C3.1.1 - Practice of SI during training

Interviewer: Okay. So what made you stop going to the hospital to get injected and start injecting yourself?

Respondent: We were taught how to inject ourselves; we were asked if would manage and I agreed and attempted to inject myself while the HSA was watching, she confirmed that I had done it well and could manage to do it on my own. I have been injecting myself for 2 years now.

Title: ZA_SCSI_3.docx

Descriptor Info:	District: Zomba	Target Population: TP3 - DMPA-SC SI clients	Respondent type: DMPA-SC SI client	Aware of SI: Yes - had heard of SI before	Coding phase: 3. Triple coded	Client marital status: Married	Client age group: 40-49 years	Time since trained: N/A - client

Codes Applied:	C3.3 - Counselling / training approach for injectable users

Interviewer: Can you explain to me your experiences when being taught the self-injection. Firstly, please explain to me what they taught you.

Respondent: They taught us and asked if we could manage. A few of us agreed and we were taken to a separate room where we were asked to try it out. Since I am left-handed, I failed to inject myself on my left arm, so I asked them to give me another option and they told me I can inject myself on my stomach. I sat down and I reached for my belly button where I injected myself and the medicine went in. so they asked me if it was going to be possible and I saw that I would do it. Some were really afraid if they would be able to do it, some thought that by injecting on the stomach they would accidentally inject the intestines. But I saw that the way I had injected myself, I didn’t reach my intestines at all.

Interviewer: Do you think the health officials took enough time in teaching you about the self-injection method?

Respondent: Yes, I think they did because if they had not, we would not have been convinced enough to try it on our own take part in it. We also learnt the things we were supposed to do and we were able to do them showing that they took their time to teach us about it.

Title: ZA_SCSI_3.docx

Descriptor Info:	District: Zomba	Target Population: TP3 - DMPA-SC SI clients	Respondent type: DMPA-SC SI client	Aware of SI: Yes - had heard of SI before	Coding phase: 3. Triple coded	Client marital status: Married	Client age group: 40-49 years	Time since trained: N/A - client

Codes Applied:	C3.3 - Counselling / training approach for injectable users

Interviewer: Okay, thank you very much. Did you feel any judgements from the people who were teaching you about the self-injection method?

Respondent: Yes, they advised us and told us the right procedure to follow, we were made aware of the advantages and the disadvantages as well of using the method of family planning. So we chose which we wanted since everybody chooses what interests them

Interviewer: Okay, in your opinion did the health officials give any judgements towards you?

Respondent: Yes, they did. They advised us on what we were supposed to do and gave advice on the procedure we were supposed to carry out in order to make sure the self-injection was successful. We were also advised to talk to our husbands about it so that they can be able to support us and remind on the dates we are supposed to inject ourselves. Telling our husbands would also help us to be free and open to inject ourselves at home freely.

Title: ZA_SCSI_3.docx

Descriptor Info:	District: Zomba	Target Population: TP3 - DMPA-SC SI clients	Respondent type: DMPA-SC SI client	Aware of SI: Yes - had heard of SI before	Coding phase: 3. Triple coded	Client marital status: Married	Client age group: 40-49 years	Time since trained: N/A - client

Codes Applied:	C3.3 - Counselling / training approach for injectable users	C3.3.2 - Good (sufficient/ non-judgemental) counselling/training

Interviewer: Thank you very much for watching this video. Is there anything that you saw new in this video?

Respondent: Nothing.

Interviewer: Everything in this video was explained to you?

Respondent: Yes.

Interviewer: Which things in this video do you think are more important?

Respondent: It has served as a reminder to us about the things we learnt. Also some of us were not give the calendars. We just take note of the days that we were given, and we inject ourselves on that day as the video has said which we were also taught by the HSA of our area.

Interviewer: Anything else?

Respondent: No

Interviewer: Is there anything that you were taught but it isn’t there in this video?

Respondent: No, everything is there.

Title: ZA_SCSI_2.docx

Descriptor Info:	District: Zomba	Target Population: TP3 - DMPA-SC SI clients	Respondent type: DMPA-SC SI client	Aware of SI: Yes - had heard of SI before	Coding phase: 1. Single coded	Client marital status:	Client age group:	Time since trained: N/A - client

Codes Applied:	C3.3 - Counselling / training approach for injectable users

Interviewer: can you describe your experience of being trained in self injection?

Respondent: ask the question again

Interviewer: I want you to tell me what you have experienced…

Respondent: like problems?

Interviewer: Being trained either problems or good things while being trained how to self-inject

Respondent: I did not experience any problems

Interviewer: Alright.

Respondent: I did not experience any problems with DMPA-SC

Interviewer: Alright, who trained you?

Respondent: I was trained by the HSAs from this hospital

Title: ZA_SCSI_2.docx

Descriptor Info:	District: Zomba	Target Population: TP3 - DMPA-SC SI clients	Respondent type: DMPA-SC SI client	Aware of SI: Yes - had heard of SI before	Coding phase: 1. Single coded	Client marital status:	Client age group:	Time since trained: N/A - client

Codes Applied:	C3.3 - Counselling / training approach for injectable users

nterviewer: Thank you so much, ah after watching this video, are there any new things that you have never heard before?

Respondent: No there is nothing new.

Interviewer: Alright, what do you think are the most important things in this video?

Respondent: The most important things in this video.

Interviewer: Mmmm (showing acceptance)

Respondent: We have seen how you can self-inject.

Interviewer: Mmmm (showing that one is following the conversation)

Respondent: The way that they counsel. I was not given some of these counsel I am just hearing them now.

Interviewer: Alright.

Respondent: Yes.

Interviewer: In the video that we have just watched are there any things that the provider said in your training but you did not see them in it?

Respondent: No, what we see in the video is what was said by the providers

Title: ZA_SCSI_5.docx

Descriptor Info:	District: Zomba	Target Population: TP3 - DMPA-SC SI clients	Respondent type: DMPA-SC SI client	Aware of SI: Yes - had heard of SI before	Coding phase: 3. Triple coded	Client marital status: Married	Client age group: 30-39 years	Time since trained: N/A - client

Codes Applied:	C3.3 - Counselling / training approach for injectable users

Interviewer: Ah now I want to talk to you about your experiences in using DMPA SC for self-injection, where did you first hear about Sayana?

Respondent: When we came here, they explained to us

Interviewer: Mmm (showing that one is following the conversation)

Respondent: When I was getting the Depo

Interviewer: Alright

Respondent: They said that there is a new method Sayana

Interviewer: Mmm (showing that one is following the conversation)

Respondent: Which you can self-inject.

Interviewer: Mmm (showing that one is following the conversation)

Respondent: When you are home, so we will train you here and then you will get it.

Interviewer: Mmm (showing that one is following the conversation)

Respondent: You will take it home.

Interviewer: Alright

Respondent: So when you are home you can self-inject, after that, in three months’ time you will self-inject again

Interviewer: Alright.

Respondent: When you do that, after three months you will again self-inject

Title: ZA_SCSI_5.docx

Descriptor Info:	District: Zomba	Target Population: TP3 - DMPA-SC SI clients	Respondent type: DMPA-SC SI client	Aware of SI: Yes - had heard of SI before	Coding phase: 3. Triple coded	Client marital status: Married	Client age group: 30-39 years	Time since trained: N/A - client

Codes Applied:	C3.3 - Counselling / training approach for injectable users

Interviewer: Can you tell me your experiences in being trained how to use DMPA SC? Firstly I want you to tell me who trained you.

Respondent: Ah this injection, the person who trained me…

Interviewer: Mmm (showing that one is following the conversation)

Respondent: Is the HSA

Interviewer: Alright.

Respondent: That’s the one who trained me

Interviewer: Alright.

Respondent: They said when we give you this injection and you get home.

Interviewer: Mmm (showing that one is following the conversation)

Respondent: When you get home, before you start self-injecting.

Interviewer: Mmm (showing that one is following the conversation)

Respondent: Or opening.

Interviewer: Mmm (showing that one is following the conversation)

Respondent: You should shake the injection unit.

Interviewer: Mmm (showing that one is following the conversation)

Respondent: We should shake it

Interviewer: Yah

Respondent: And then press it down in order to activate, and then we pinch below the navel.

Interviewer: Mmm (showing that one is following the conversation)

Respondent: And do like this

Interviewer: Mmm (showing that one is following the conversation)

Respondent: And then we inject

Title: MZs_SCSI_2.docx

Descriptor Info:	District: Mzimba South	Target Population: TP3 - DMPA-SC SI clients	Respondent type: DMPA-SC SI client	Aware of SI: Yes - had heard of SI before	Coding phase: 3. Triple coded	Client marital status: Married	Client age group: Under 20 (adolescent)	Time since trained: N/A - client

Codes Applied:	C3.3 - Counselling / training approach for injectable users

Q7. Interviewer: Thanks a lot, as you have said before you started self-injection you have been using providers injection, did you find it difficult or not?

Respondent: No it was easy, but when I came for providers injection it’s when they told us that it’s out of stock, rather they told us there is new method and provider said that with this method we can be able to inject ourselves.

Title: MZs_SCSI_2.docx

Descriptor Info:	District: Mzimba South	Target Population: TP3 - DMPA-SC SI clients	Respondent type: DMPA-SC SI client	Aware of SI: Yes - had heard of SI before	Coding phase: 3. Triple coded	Client marital status: Married	Client age group: Under 20 (adolescent)	Time since trained: N/A - client

Codes Applied:	C3.3 - Counselling / training approach for injectable users

Interviewer: How did the provider describe self-injection to you?

Respondent: The provider said this method is just the same as the providers injection just that with this one the client is supposed to inject themselves after counselling.

Interviewer: Can you explain to me on how the provider instructed you to self-inject?

Respondent: Yes, this Sayana press injection is in a pack where you need to take the pack and tear it and it has only two places where we can inject – Tummy (abdomen) and Thigh. So the provider said that after tearing the Uniject then we find something like a bottle with medicine then we should put it straight and then upside down then we shake it and after that we press the top cover that means we have open it, after opening it we need to touch [pinch] where we are going to inject and then we do the injection while pressing the bottle so that all the medicine should come out.

Interviewer: Mmmmm (following)

Respondent: Then after removing the syringe we need to keep it and bring it to the facility because we do not have a good place where we can throw them.

Interviewer: Did they tell you where you can keep the used ones?

Respondent: They said that we should be putting it inside the plastic bag and cover it then we should keep it away from the children.

Interviewer: Okay, do you have anything that you can add?

Respondent: They also said that we should be keeping the medicine at a place where children cannot reach, and we should not put at a place where there is moisture or a place where there is no direct sunlight.

Interviewer: Mmmmm (following)

Respondent: They told us that we should be keeping the medicine at a good place.

Title: MZs_SCSI_5.docx

Descriptor Info:	District: Mzimba South	Target Population: TP3 - DMPA-SC SI clients	Respondent type: DMPA-SC SI client	Aware of SI: Yes - had heard of SI before	Coding phase: 1. Single coded	Client marital status: Married	Client age group: 20-29 years	Time since trained: N/A - client

Codes Applied:	C3.3 - Counselling / training approach for injectable users	C3.1 - Enablers, general/other	C1.1.3 - Awareness of DMPA-SC SI

Interviewer: Where did you hear about this method of self-inject?

Respondent: It was here at the health facility. I just came here to start family planning because my third child had grown a bit so I wanted to avoid another pregnancy since I have had a flesh scar from caesarean section. Of course I first heard from my friends that they are self-injecting but I did not see what it looked like. So when I came here I gave them my health passport book. Then they started calling one by one. They told us they only had self-inject where a woman self-inject on the stomach or thighs. They taught us on how to self-inject. So I accepted to get it since I had come for family planning so I self-injected and went home

Interviewer: What motivated you to get self-inject method?

Respondent: I did not have any choice because it was the only option. I took time to self-inject because I thought it was a bigger needle which the provider uses to inject. But because I didn’t have a choice, I just took it. But because I did not experience any problems, I came for the second dose, and they told us they had few in stock so we self-injected right here at the facility. The third time it was out of stock, so I was given Depo Provera

Title: MZs_SCSI_5.docx

Descriptor Info:	District: Mzimba South	Target Population: TP3 - DMPA-SC SI clients	Respondent type: DMPA-SC SI client	Aware of SI: Yes - had heard of SI before	Coding phase: 1. Single coded	Client marital status: Married	Client age group: 20-29 years	Time since trained: N/A - client

Codes Applied:	C3.3 - Counselling / training approach for injectable users	C0.4 - Access to or process of obtaining FP services

Interviewer: Who first taught you on how to self-inject?

Respondent: I can’t know their name, but it was one of the providers

Interviewer: Was it a provider who taught you at first

Respondent: Yes

Interviewer: Did your friends ever taught you

Respondent: I came here at the facility that’s where I heard all about self-inject method of family planning.

Interviewer: Were you given anything to take home?

Respondent: As I said earlier on, no. we were just taught on how to self-inject. We were not given to self-inject at home. On the third visit we found that it was out of stock

Title: MZs_SCSI_5.docx

Descriptor Info:	District: Mzimba South	Target Population: TP3 - DMPA-SC SI clients	Respondent type: DMPA-SC SI client	Aware of SI: Yes - had heard of SI before	Coding phase: 1. Single coded	Client marital status: Married	Client age group: 20-29 years	Time since trained: N/A - client

Codes Applied:	C3.3 - Counselling / training approach for injectable users

Interviewer: Were you given a chance to practice before self-injecting

Respondent: Before self-injecting. They taught us the whole procedure and then we self-injected

Interviewer: Did the provider use any aid to self-inject, were you given any aid to practice?

Respondent: No

Interviewer: No?

Respondent: They just taught us how to do it then we did it

Interviewer: Did you feel judged during the counselling?

Respondent: Judged?

Interviewer: Yes

Respondent: We felt judged we were taught on the whole process on self-injection

Interviewer: What if you didn’t understand, were they judgmental?

Respondent: Asking questions

Interviewer: Being judged for not grasping faster?

Respondent: We were able to ask questions like will the needle not remain under the skin? They were able to reassure us that that won’t happen. We also asked on how to hold the needle they were able to demonstrate to us.

Title: KK_SCSI_1.docx

Descriptor Info:	District: Nkhotakota	Target Population: TP3 - DMPA-SC SI clients	Respondent type: DMPA-SC SI client	Aware of SI: Yes - had heard of SI before	Coding phase: 3. Triple coded	Client marital status: Never married	Client age group: Under 20 (adolescent)	Time since trained: N/A - client

Codes Applied:	C3.3 - Counselling / training approach for injectable users

INTERVIEWER: Okay, alright. Can you describe your experience of being trained in self-injection? You should explain how you were trained

Time stamp: (20:08)

RESPONDENT: how I was trained by…by a nurse right?

INTERVIEWER: mmmmh.

RESPONDENT: He/she said that I should tear the sachet that contains Sayana. There is a dotted line, and they tell us to open there. When you open the sachet, they tell us to hold the drug and shake.

INTERVIEWER: mmmmhu.

RESPONDENT: Mmmhu. You should shake and shake and then you have to close…before you start shaking you have to close. We close first and then shake and there after open. There after hold your thigh and inject yourself.

INTERVIEWER: mmmhu.

RESPONDENT: Mmm. They told us.

INTERVIEWER: Who trained you?

RESPONDENT: Nurse [name] trained us.

INTERVIEWER: [name]?

RESPONDENT: mmmh.

INTERVIEWER: Mmmhu. How did he/she describe about Sayana? The day you decided to use this method, when you arrived, how did he describe this to you?

RESPONDENT: They explained that this is one of the methods of family planning. Mmmhu, we inject once in every three months. You are given some so that you should self-inject after three months. A client self-injects. They just give you to self-inject at home and you are required to self-inject after every three months because you are given some so that after every three months you should inject.

Title: KK_SCSI_1.docx

Descriptor Info:	District: Nkhotakota	Target Population: TP3 - DMPA-SC SI clients	Respondent type: DMPA-SC SI client	Aware of SI: Yes - had heard of SI before	Coding phase: 3. Triple coded	Client marital status: Never married	Client age group: Under 20 (adolescent)	Time since trained: N/A - client

Codes Applied:	C3.3 - Counselling / training approach for injectable users

INTERVIEWER: During the training, did you feel judged, when I say judged you know what I mean right? Maybe mocking you? Did you feel judged during your training on self-injection?

RESPONDENT: Yes. They would say that you are a failure because you are not getting it right. We do like this and this. It is like you have made a mistake because you are not doing what they are telling you to. Maybe you did not get what they were saying, they judge you and ask you how you how they should say it so that you should understand.

INTERVIEWER: okay.

RESPONDENT: Yes.

Title: ZA_SCSI_4.docx

Descriptor Info:	District: Zomba	Target Population: TP3 - DMPA-SC SI clients	Respondent type: DMPA-SC SI client	Aware of SI: Yes - had heard of SI before	Coding phase: 2. Double coded	Client marital status: Married	Client age group: 30-39 years	Time since trained: N/A - client

Codes Applied:	C3.3 - Counselling / training approach for injectable users

Interviewer: Alright, ah can you describe our experiences of being trained in self-injection? First of all I want you to tell me who trained you?

Respondent: The HSA trained me.

Interviewer: mmm (showing that one is following the conversation)

Respondent: From this hospital

Interviewer: mmm (showing that one is following the conversation)

Respondent: The day that we came.

Title: ZA_SCSI_4.docx

Descriptor Info:	District: Zomba	Target Population: TP3 - DMPA-SC SI clients	Respondent type: DMPA-SC SI client	Aware of SI: Yes - had heard of SI before	Coding phase: 2. Double coded	Client marital status: Married	Client age group: 30-39 years	Time since trained: N/A - client

Codes Applied:	C3.3 - Counselling / training approach for injectable users

terviewer: mmm (showing that one is following the conversation)

Respondent: When we got into the consultation room, we spent more time with the provider.

Interviewer: mmm (showing that one is following the conversation)

Respondent: so the provider took the injection and shook it

Interviewer: mmm (showing that one is following the conversation)

Respondent: and shared with us how to use it.

Interviewer: mmm (showing that one is following the conversation)

Respondent: And position it, after doing that they would tell us to self-inject, yourself so that when going back home you should have been known how to self-inject. So I saw that for me they took enough time that everyone was trained and knew that when I go home, I will do this.

Interviewer: Alright.

Respondent: Sure.

Interviewer: Alright, did they give you the chance to practice self-injecting?

Respondent: mmm (showing that one is following the conversation)

Interviewer: Did they give you a chance to use things like injecting on a condom filled with sugar or sand so that you could practice?

Respondent: No

Interviewer: Alright, during the time that they were telling you about this injectable, did you feel judged in any way?

Respondent: No

Title: MZs_SCSI_4.docx

Descriptor Info:	District: Mzimba South	Target Population: TP3 - DMPA-SC SI clients	Respondent type: DMPA-SC SI client	Aware of SI: Yes - had heard of SI before	Coding phase: 2. Double coded	Client marital status: Married	Client age group: 20-29 years	Time since trained: N/A - client

Codes Applied:	C3.3 - Counselling / training approach for injectable users

Interviewer: Would you tell me what you experienced with Sayana Press. Firstly tell me who taught you?

Respondent: It’s the HSA who taught me

Interviewer: What did you experience after you started using Sayana

Respondent: I experienced… (stammer) ( moment of silent)

Interviewer: What did you like when you started using Sayana Press. What did they say on how to use Sayana Press when you were taught?

Respondent: The time we came they explained to us, but it’s been a while for me to remember details

Interviewer: Explain to me what you recall

Respondent: This method of family planning (Sayana Press) is good (recording paused visitor entered the room) the provider just gives options so it’s for us to choose.

Interviewer: Mmmmh

Respondent: That’s all I can say

Title: MZs_SCSI_4.docx

Descriptor Info:	District: Mzimba South	Target Population: TP3 - DMPA-SC SI clients	Respondent type: DMPA-SC SI client	Aware of SI: Yes - had heard of SI before	Coding phase: 2. Double coded	Client marital status: Married	Client age group: 20-29 years	Time since trained: N/A - client

Codes Applied:	C3.3 - Counselling / training approach for injectable users

nterviewer: I would like you to explain what they said from the start up to the point you self –injected

Respondent: Firstly, we open the content there is a mark where you open. Then we press it hard and shake the content. After shaking then we self-inject and squeeze all the content then take the injection out.

Interviewer: Mmmhuuu

Respondent: Yes

Interviewer: After that?

Respondent: Then we take used materials and throw them in the toilet because we do not have safety boxes at home. (Points at the sharp container)

Interviewer: Mmmh

Respondent: Yes

Interviewer: Did they say you should throw in the toilet

Respondent: Yes, they did

Interviewer: In your view, did they spend enough time explaining to you?

Respondent: No they did not spend more time

Interviewer: Mmmhu (agreeing) did they give you a chance to practice on how to self-inject?

Respondent: Yes, the very same day they taught us on how to self-inject it’s the same day we self-injected

Interviewer: Did they give you anything to practice on self-injecting?

Respondent: It was a day we were due for injection

Interviewer: After teaching you, did they give you anything to practice on self-injecting? Or not?

Respondent: No they did not provide us with anything we went straight to self-inject

Interviewer: When they were teaching you, did you feel judged by providers?

Respondent: We did not feel judged but just had fears on how to self-inject since it’s not an easy procedure

Title: ZA_SCSI_1.docx

Descriptor Info:	District: Zomba	Target Population: TP3 - DMPA-SC SI clients	Respondent type: DMPA-SC SI client	Aware of SI: Yes - had heard of SI before	Coding phase: 1. Single coded	Client marital status: Married	Client age group: 40-49 years	Time since trained: N/A - client

Codes Applied:	C3.3 - Counselling / training approach for injectable users

Interviewer: Okay, okay thanks a lot, so can you explain what your experience of being trained in self injection? Firstly, explain how they taught you.

Respondent: When we arrived, they started teaching us saying first you remove the injection then pull the plastic finally inject yourself then discard it thanks.
