# Supplementary material for: Health care provider and client experiences of counselling on depot medroxyprogesterone acetate subcutaneous (DMPA-SC) for self-injection in Malawi
Source: PLOS Glob Public Health. 2023 Nov 30;3(11):e0002057. doi: 10.1371/journal.pgph.0002057 (PMC10688740; doi:10.1371/journal.pgph.0002057)
Supplement: S1 Data — (ZIP) [file pgph.0002057.s001.zip › S2/C3.3.1 recos couns PAIM only.doc]

Title: KK_IM_3.docx

Descriptor Info:	District: Nkhotakota	Target Population: TP2 - DMPA-IM/DMPA-SC PI clients	Respondent type: DMPA-IM client	Aware of SI: Yes - had heard of SI before	Coding phase: 1. Single coded	Client marital status: Divorced/ separated/ widowed	Client age group: 20-29 years	Time since trained: N/A - client

Codes Applied:	C3.3.1 - Recos for what should be covered in counselling / training

INTERVIEWER: Alright, what else do you think women need to know about self-injection before trying it?

RESPONDENT: they need to be trained first

INTERVIEWER: to be trained?

RESPONDENT: they need to be trained before trying the method

INTERVIEWER: What exactly should they get trained on

RESPONDENT: They should be trained on how to inject themselves, because they may just get the injections without knowing how best to use it.

INTERVIEWER: Without proper understanding?

RESPONDENT: without proper understanding

Title: MZs_IM_8.docx

Descriptor Info:	District: Mzimba South	Target Population: TP2 - DMPA-IM/DMPA-SC PI clients	Respondent type: DMPA-IM client	Aware of SI: Yes - had heard of SI before	Coding phase: 1. Single coded	Client marital status: Married	Client age group: 20-29 years	Time since trained: N/A - client

Codes Applied:	C3.3.1 - Recos for what should be covered in counselling / training	C1.1.1 - Positive client attitudes to SI	C2.1.2 - Reduced trips to facility

Interviewer: what should a woman planning to get Sayana for self-injection know?

Respondent: how to hold a needle. It is difficult to do it if you have not been taught. But if they do it is easy.

Interviewer: what exactly are they supposed to know?

Respondent: she should be counselled

Interviewer: after watching the video, has your perception on self-injection changed?

Respondent: yes, I have learnt some new things. The way the woman explained self-injection is good especially in this era of COVID-19 and also you have time at home to do your things. It also reduces costs of travelling.

Title: MZs_IM_8.docx

Descriptor Info:	District: Mzimba South	Target Population: TP2 - DMPA-IM/DMPA-SC PI clients	Respondent type: DMPA-IM client	Aware of SI: Yes - had heard of SI before	Coding phase: 1. Single coded	Client marital status: Married	Client age group: 20-29 years	Time since trained: N/A - client

Codes Applied:	C2.1.2 - Reduced trips to facility	C3.3.1 - Recos for what should be covered in counselling / training	C1.2.2 - Fear of SI (idea or reality)

Interviewer: have you ever used self-injection?

Respondent: no

Interviewer: why not?

Respondent: I had fears on self-injecting

Interviewer: did you ever ask the provider to help you on your fears?

Respondent: no

Interviewer: do you think women on Depo-IM might be more interested with Sayana?

Respondent: yes, a lot

Interviewer: why do you think many switch to self-injection?

Respondent: they see it as a way of protecting themselves from COVID-19 because those who get Depo-IM come in large numbers

Interviewer: do you think different women may have different views on self-injection?

Respondent: yes, because it depends with the understanding of each one of them

Interviewer: what do you think should a woman know before getting self-injection?

Respondent: she just needs to be counselled that’s when she would know

Title: MZs_IM_8.docx

Descriptor Info:	District: Mzimba South	Target Population: TP2 - DMPA-IM/DMPA-SC PI clients	Respondent type: DMPA-IM client	Aware of SI: Yes - had heard of SI before	Coding phase: 1. Single coded	Client marital status: Married	Client age group: 20-29 years	Time since trained: N/A - client

Codes Applied:	C3.3.1 - Recos for what should be covered in counselling / training

Interviewer: we have come to the end of our interview. Do you have anything to add?

Respondent: can I mix Depo-IM with Sayana Press

Interviewer: these methods are similar. They have the same mode of action. The only difference is that Depo is provided by the provider whilst Sayana is for self-injection. If you are interested ask your provider on the next date of appointment.

Respondent: ok thank you

Title: MZs_IM_2.docx

Descriptor Info:	District: Mzimba South	Target Population: TP2 - DMPA-IM/DMPA-SC PI clients	Respondent type: DMPA-IM client	Aware of SI: Yes - had heard of SI before	Coding phase: 3. Triple coded	Client marital status: Married	Client age group: 30-39 years	Time since trained: N/A - client

Codes Applied:	C3.3.1 - Recos for what should be covered in counselling / training

Interviewer: What else do you think women would like to find out about this method before finding out?

Respondent: They should first know the good thing about the method and also how it works.

Interviewer: Do you have anything else?

Respondent: No.

Q17. Interviewer: We have now come to an end of the questions I had for you today. Do you have any other thoughts or information you would like to share on the topics we have discussed today?

Respondent: I want to know why do you encourage that after self-injection we are not supposed to dispose needles in the toilet.

Interviewer: Thanks for your question, that needle is not something that cannot be rotted thus we are advised not to throw it in the toilet or any other place. If you throw it in the toilet and the toilet drop or descend the needle will still be there because it’s something that cannot be rotten. Another reason is that, if you give it back the used materials to the provider, that means the provider will have proof that you used the method.

Title: MZs_IM_2.docx

Descriptor Info:	District: Mzimba South	Target Population: TP2 - DMPA-IM/DMPA-SC PI clients	Respondent type: DMPA-IM client	Aware of SI: Yes - had heard of SI before	Coding phase: 3. Triple coded	Client marital status: Married	Client age group: 30-39 years	Time since trained: N/A - client

Codes Applied:	C3.3.1 - Recos for what should be covered in counselling / training

Interviewer: Do you have any final question before we finish?

Respondent: Since I have been using this method where the provider is the one who inject me should I wait for me to get this self-inject?

Interviewer: Yes, if you want to start using self-inject you need to wait for your date and then you will come at the facility and tell them that you want to start using self-injection, then they will do the counselling and tell you all that is required for you to start injecting yourself.

Title: ZA_IM_2.docx

Descriptor Info:	District: Zomba	Target Population: TP2 - DMPA-IM/DMPA-SC PI clients	Respondent type: DMPA-IM client	Aware of SI: Yes - had heard of SI before	Coding phase: 1. Single coded	Client marital status: Married	Client age group: 30-39 years	Time since trained: N/A - client

Codes Applied:	C3.3.1 - Recos for what should be covered in counselling / training

Interviewer: Are there any questions before we part?

Respondent: Yes, I have a question, is it possible that a person can be injected with Sayana here at the hospital?

Interviewer: Yes, here at the hospital it is also possible that if you can’t inject yourself at home, maybe some people are afraid that they may injure themselves, they may just come here at the hospital as you always do, and you can be injected by a provider.

Respondent: Alright

Title: KK_IM_5.docx

Descriptor Info:	District: Nkhotakota	Target Population: TP2 - DMPA-IM/DMPA-SC PI clients	Respondent type: DMPA-IM client	Aware of SI: No - never heard of SI before	Coding phase: 3. Triple coded	Client marital status: Divorced/ separated/ widowed	Client age group: Under 20 (adolescent)	Time since trained: N/A - client

Codes Applied:	C3.3.1 - Recos for what should be covered in counselling / training

INTERVIEWER: What else do you think women need to know about self-injection before trying it? What is it that they may be interested to know as explained, if the doctor may explain about this method as I have just done? And some women would like to use this method, what is it that they would want to know about this self-injection method?

RESPONDENT: The way things are

INTERVIEWER: What do you mean by the way things are?

RESPONDENT: The way the injection works

INTERVIEWER: How the injection is used?

RESPONDENT: Yes

INTERVIEWER: What else would you be interested to know before making a choice to start using the method I need to know this and that?

RESPONDENT: I should know how to inject myself

INTERVIEWER: How to inject yourself? what else, you explained something on Depo what did you say are some of the effects when one is using it

RESPONDENT: They get injected

INTERVIEWER: No you said when using it to some it does something and to some it doesn’t

RESPONDENT: Yes

INTERVIEWER: Yes so, I want to know, what is it that you may want to know about this method?

RESPONDENT: Eeee (participant not sure what to respond) If one gets injected will she have prolonged periods or not

INTERVIEWER: Yes

RESPONDENT: If she may find out that she is having prolonged period she may

INTERVIEWER: In short you are trying to say if there are pros and cons to this method not so

RESPONDENT: Yes

Title: ZA_IM_1.docx

Descriptor Info:	District: Zomba	Target Population: TP2 - DMPA-IM/DMPA-SC PI clients	Respondent type: DMPA-IM client	Aware of SI: Yes - had heard of SI before	Coding phase: 1. Single coded	Client marital status: Married	Client age group: 40-49 years	Time since trained: N/A - client

Codes Applied:	C1.1.1 - Positive client attitudes to SI	C3.3.1 - Recos for what should be covered in counselling / training	C2.1.2 - Reduced trips to facility

Interviewer: What else do you think women need to know about self-injection before trying it?

Respondent: They are supposed to the taught about the benefits because you take time without going to the hospital. You will have the injection with you at your house, when time arises to self-inject you do it. You take take before returning to the hospital to get more self-injection kits

Interviewer: Alright thank you. After watching the video, would you say you feel the same, more positive, or more negative about self-injection?

[Kids making noise in the background]

Respondent: No, they didn’t change I still want self-injection

Title: KK_SCPI_6.docx

Descriptor Info:	District: Nkhotakota	Target Population: TP2 - DMPA-IM/DMPA-SC PI clients	Respondent type: DMPA-SC PI client	Aware of SI: Yes - had heard of SI before	Coding phase: 1. Single coded	Client marital status: Married	Client age group: 20-29 years	Time since trained: N/A - client

Codes Applied:	C3.3.1 - Recos for what should be covered in counselling / training

NTERVIEWER: mmmhu Okay, thank you. After you failed to inject yourself, did you ask the doctor or the nurse any question?

RESPONDENT: Yes, I asked.

INTERVIEWER: What were the questions you asked?

RESPONDENT: I wanted to know if it was possible for the needle to break in the skin may be one does not know how to handle it, so the doctor said it cannot break.

INTERVIEWER: mmmhu.

RESPONDENT: yes

INTERVIEWER: So after watching this video, has that question been answered?

RESPONDENT: Yes, it has been answered.

Title: KK_SCPI_4.docx

Descriptor Info:	District: Nkhotakota	Target Population: TP2 - DMPA-IM/DMPA-SC PI clients	Respondent type: DMPA-SC PI client	Aware of SI: Yes - had heard of SI before	Coding phase: 2. Double coded	Client marital status: Never married	Client age group: 20-29 years	Time since trained: N/A - client

Codes Applied:	C3.3.1 - Recos for what should be covered in counselling / training

INTERVIEWER: What do you think women need to know about Sayana before they start using it?

RESPONDENT: They need to know what is taught, when they get it, and open it and shaking it before injecting themselves.

INTERVIEWER: mmmmhu.

RESPONDENT: The chemicals, when they get to the point of opening, and open it then inject themselves.

INTERVIEWER: okay.

RESPONDENT: Yes

Title: KK_SCPI_4.docx

Descriptor Info:	District: Nkhotakota	Target Population: TP2 - DMPA-IM/DMPA-SC PI clients	Respondent type: DMPA-SC PI client	Aware of SI: Yes - had heard of SI before	Coding phase: 2. Double coded	Client marital status: Never married	Client age group: 20-29 years	Time since trained: N/A - client

Codes Applied:	C3.3.1 - Recos for what should be covered in counselling / training

INTERVIEWER: Anything, a comment may be…

RESPONDENT: All I wanted to know are the good and bad sides of Sayana

INTERVIEWER: You should ask …just ask the doctor. I told you that am just a researcher.

RESPONDENT: mmmh.

Title: ZA_SCPI_3.docx

Descriptor Info:	District: Zomba	Target Population: TP2 - DMPA-IM/DMPA-SC PI clients	Respondent type: DMPA-SC PI client	Aware of SI: No - never heard of SI before	Coding phase: 3. Triple coded	Client marital status: Never married	Client age group: Under 20 (adolescent)	Time since trained: N/A - client

Codes Applied:	C3.3.1 - Recos for what should be covered in counselling / training

Respondent: This contraception is for a year?

Interviewer: eh

Respondent: The whole year?

Title: ZA_SCPI_3.docx

Descriptor Info:	District: Zomba	Target Population: TP2 - DMPA-IM/DMPA-SC PI clients	Respondent type: DMPA-SC PI client	Aware of SI: No - never heard of SI before	Coding phase: 3. Triple coded	Client marital status: Never married	Client age group: Under 20 (adolescent)	Time since trained: N/A - client

Codes Applied:	C3.3.1 - Recos for what should be covered in counselling / training

Interviewer: what do you think women need to know before they say that ah, I’ll use this method or that I won’t use this method…

Respondent: (Laughs) This injectable?

Interviewer: Yes, the self-injectable.

Respondent: They might want to know a lot about the injectable.

Title: ZA_SCPI_3.docx

Descriptor Info:	District: Zomba	Target Population: TP2 - DMPA-IM/DMPA-SC PI clients	Respondent type: DMPA-SC PI client	Aware of SI: No - never heard of SI before	Coding phase: 3. Triple coded	Client marital status: Never married	Client age group: Under 20 (adolescent)	Time since trained: N/A - client

Codes Applied:	C3.3.1 - Recos for what should be covered in counselling / training

Interviewer: Alright, ah thank you so much because of having the time to take part in this study, let me thank you for…

Respondent: Let me ask something…

Interviewer: Alright.

Respondent: Where on the body do you inject yourself, on the arm or the thigh the way that they were doing it there (Pointing at the job aids)?

Interviewer: You inject yourself either on the thigh, or on the stomach area but not on the navel, so you can inject yourself here (pointing at the lower abdomen area) or here on the side, you just have to do it where there are a lot of fats because when you inject yourself this contraception, I have already said that the needle is small it goes in the fats. But the functioning, everything is the same with the injection that you get here at the hospital depo. The doses in this one is the same, the only difference is that women can self-inject, sure. So, you can inject yourself on the stomach or the thigh where there are more fats.

Respondent: Okay.

Interviewer: Sure.

Respondent: So, this contraceptive you can be able to have your menstrual periods?

Interviewer: The menstrual cycles are just the same way they will be when you take the provider administered injection here at the facility. Sure, because the doses that are in the injection are similar with the one that you get from here.

Respondent: mmmm (showing that one is following the conversation)

Interviewer: Meaning that everything is the same, the only difference is that the needle for Depo is longer because it gets to the muscles and for Sayana it gets to the fats, so it is a short needle.

Respondent: Okay.

Interviewer: Sure.

Respondent: Is this injection available?

Interviewer: At this facility, we need to ask the providers here, but in the hospitals, it is available and also from HSAs in the community.

Title: ZA_SCPI_3.docx

Descriptor Info:	District: Zomba	Target Population: TP2 - DMPA-IM/DMPA-SC PI clients	Respondent type: DMPA-SC PI client	Aware of SI: No - never heard of SI before	Coding phase: 3. Triple coded	Client marital status: Never married	Client age group: Under 20 (adolescent)	Time since trained: N/A - client

Codes Applied:	C3.3.1 - Recos for what should be covered in counselling / training	C0.2.2 - Negative community/client perspectives on FP

Interviewer: Sure, are there any other questions?

Respondent: Yes.

Interviewer: Ask please.

Respondent: When a woman takes the injectable contraceptives, is it true that they will never conceive in the future?

Title: MZs_IM_5.docx

Descriptor Info:	District: Mzimba South	Target Population: TP2 - DMPA-IM/DMPA-SC PI clients	Respondent type: DMPA-IM client	Aware of SI: Yes - had heard of SI before	Coding phase: 1. Single coded	Client marital status: Married	Client age group: 20-29 years	Time since trained: N/A - client

Codes Applied:	C3.3.1 - Recos for what should be covered in counselling / training

Interviewer: what should a woman know before getting self-injection?

Respondent: (giggles, stammers, giggles)

Interviewer: what should a woman know before self-injecting?

Respondent: should ask questions like is the self-injection and Depo-IM similar. What is the mode of action? We ask questions so that they explain to us before we make a choice

Title: MZs_IM_6.docx

Descriptor Info:	District: Mzimba South	Target Population: TP2 - DMPA-IM/DMPA-SC PI clients	Respondent type: DMPA-IM client	Aware of SI: Yes - had heard of SI before	Coding phase: 1. Single coded	Client marital status: Married	Client age group: Under 20 (adolescent)	Time since trained: N/A - client

Codes Applied:	C3.3.1 - Recos for what should be covered in counselling / training

Interviewer: What do you think is crucial for women intending to use self-inject need to know before getting it

Respondent: The whole procedure of self-injecting

Interviewer: Mmmhu (agreeing) what else?

Respondent: And the whole procedure

Interviewer: What do you mean

Respondent: I mean like keeping track of the dates

Title: MZs_IM_6.docx

Descriptor Info:	District: Mzimba South	Target Population: TP2 - DMPA-IM/DMPA-SC PI clients	Respondent type: DMPA-IM client	Aware of SI: Yes - had heard of SI before	Coding phase: 1. Single coded	Client marital status: Married	Client age group: Under 20 (adolescent)	Time since trained: N/A - client

Codes Applied:	C1.1.1 - Positive client attitudes to SI	C2.1.2 - Reduced trips to facility	C3.3.1 - Recos for what should be covered in counselling / training

Interviewer: [interrupted by a visitor] as you have watched the video. Do you think other women who gets provider Depo would opt for self-inject after watching this video?

Respondent: Yes

Interviewer: Why?

Respondent: Because it is well explained

Interviewer: What else?

Respondent: They will be attracted by the fact that they will reduce the number of visits to the facility

Interviewer: Mmmh [thinking out loud] what else?

Respondent: That’s all

Interviewer: What do you think is the most crucial information for women who take this method of self-injection

Respondent: They have to know the procedure for using it because if they don’t have adequate information, they might end up doing it wrongly at home but if they are given all the information, they can use it easily

Interviewer: What about for adolescents?

Respondent: The benefit to them is that they will protect themselves from getting pregnant

Title: ZA_IM_4.docx

Descriptor Info:	District: Zomba	Target Population: TP2 - DMPA-IM/DMPA-SC PI clients	Respondent type: DMPA-IM client	Aware of SI: Yes - had heard of SI before	Coding phase: 1. Single coded	Client marital status: Married	Client age group: Under 20 (adolescent)	Time since trained: N/A - client

Codes Applied:	C3.3.1 - Recos for what should be covered in counselling / training

Interviewer: Alright what do you think women would want to know about this family planning method before they make choice?

Respondent: They have to know there is a new injection

Interviewer: Is there anything else?

Respondent: No

Interviewer: Please add something my lady

Respondent: I don’t have any ideas

Title: MZs_IM_7.docx

Descriptor Info:	District: Mzimba South	Target Population: TP2 - DMPA-IM/DMPA-SC PI clients	Respondent type: DMPA-IM client	Aware of SI: Yes - had heard of SI before	Coding phase: 1. Single coded	Client marital status: Divorced/ separated/ widowed	Client age group: Under 20 (adolescent)	Time since trained: N/A - client

Codes Applied:	C3.3.1 - Recos for what should be covered in counselling / training

Interviewer: what do women need to know before getting Sayana for self-injection?

Respondent: to know

Interviewer: yes

Respondent: own their own they cannot know may be if I tell them

Interviewer: what would you tell them?

Respondent: I will tell them that there is a new method of self-injection

Interviewer: what do you think they need to know before they get self-injection?

Respondent: they need to know what you taught me

Interviewer: what did I say?

Respondent: what you explained

Interviewer: may you tell me what I said?

Respondent: I would tell them that they after using this method it’s not difficult to get pregnant again but also, they give you to use home for 9 months

Interviewer: what are they supposed to know, may be let me repeat what I said... [repeated the teaching]… after hearing that what do you think a woman need to know before getting Sayana for self-injection?

Respondent: she needs to know what you explained to me

Interviewer: ok so what is it that I have explained?

Respondent: you said some may have headache, some nausea, some might not have menses, some might have irregular menses. You also said they will self-inject again after 3 months

Title: KK_IM_7.docx

Descriptor Info:	District: Nkhotakota	Target Population: TP2 - DMPA-IM/DMPA-SC PI clients	Respondent type: DMPA-IM client	Aware of SI: Yes - had heard of SI before	Coding phase: 1. Single coded	Client marital status: Married	Client age group: 30-39 years	Time since trained: N/A - client

Codes Applied:	C3.3.1 - Recos for what should be covered in counselling / training

INTERVIEWER: What else do you think women need to know about self-injection before trying it?

RESPONDENT: They have to first go to a provider to understand what should be done.

INTERVIEWER: okay.

RESPONDENT: mmmhu.

INTERVIEWER: alright. So you have watched the video, right?

RESPONDENT: mmmmh.

Title: ZA_IM_5.docx

Descriptor Info:	District: Zomba	Target Population: TP2 - DMPA-IM/DMPA-SC PI clients	Respondent type: DMPA-IM client	Aware of SI: Yes - had heard of SI before	Coding phase: 2. Double coded	Client marital status: Married	Client age group: 30-39 years	Time since trained: N/A - client

Codes Applied:	C2.1.2 - Reduced trips to facility	C3.3.1 - Recos for what should be covered in counselling / training

Interviewer: Alright, what do you think are the most important things in the video?

Respondent: The most important things in the video like they have said that it is reducing

Interviewer: Mmmm (showing that one is following the conversation)

Respondent: Going to the hospital more often because when you get the injection then they will give you three units, it will last you for 9 months.

Interviewer: Mmmm (showing that one is following the conversation)

Respondent: 9 months yeah

Interviewer: Mmmm (showing that one is following the conversation)

Respondent: Yes, which is like a year, just remaining with two months to end the year.

Interviewer: Mmmm (showing that one is following the conversation)

Respondent: Just remaining with a few months in the future.

Interviewer: Yah yah

Respondent: Yes.

Interviewer: Alright.

Respondent: So in a year you can just be coming to the hospital only two times.

Interviewer: Mmmm (showing that one is following the conversation)

Respondent: Yeah

Title: ZA_IM_5.docx

Descriptor Info:	District: Zomba	Target Population: TP2 - DMPA-IM/DMPA-SC PI clients	Respondent type: DMPA-IM client	Aware of SI: Yes - had heard of SI before	Coding phase: 2. Double coded	Client marital status: Married	Client age group: 30-39 years	Time since trained: N/A - client

Codes Applied:	C3.3.1 - Recos for what should be covered in counselling / training

Interviewer: Alright, what else do you think women need to know about DMPA SC before they start using it?

Respondent: What they need to know before they start using DMPA SC is that. My little child is moving around outside. (looking outside the window)

Interviewer: Okay (starts talking about the child and stops recording)

(RECORDING RESUMES)

Interviewer: Alright, let’s continue

Respondent: Is that women like these need to…

DOOR OPENS AND IMMEDIATELY CLOSES

TIME INTO THE INTERVIEW: 40Mins

Respondent: …to sit down with them

Interviewer: Mmmm (showing that one is following the conversation)

Respondent: Encourage them like they were encouraging in the video because they encouraged us but some of the women said they would find this method hard

Interviewer: Alright.

Respondent: But we can, we can encourage them for example the mobility.

Interviewer: Mmmm (showing that one is following the conversation)

Respondent: Since we will stay after months they will come to the facility

Interviewer: Alright.

Respondent: Some women forget the date.

Interviewer: Mmmm (showing that one is following the conversation)

Respondent: But for Sayana you are able to know that I got the injection on this particular date

Interviewer: Mmmm (showing that one is following the conversation)

Respondent: You still are able to remember like the way we do with medication

Interviewer: Mmmm (showing that one is following the conversation)

Respondent: The same way with this we are also able to remember.

Interviewer: Alright.

Respondent: Maybe even when you go to the hospital, they say that the dosage is not available and you should wait that they should be available, so the good thing with this after we encouraged our friends that this is a good method because when we get the units then we are sorted for many months ahead.

Interviewer: Alright.

Respondent: Yeah.

Title: ZA_IM_5.docx

Descriptor Info:	District: Zomba	Target Population: TP2 - DMPA-IM/DMPA-SC PI clients	Respondent type: DMPA-IM client	Aware of SI: Yes - had heard of SI before	Coding phase: 2. Double coded	Client marital status: Married	Client age group: 30-39 years	Time since trained: N/A - client

Codes Applied:	C3.3.1 - Recos for what should be covered in counselling / training

Interviewer: Mmmm (showing that one is following the conversation), alright, alright. Ah now we have reached the end of our discussion and also the questions that I had today, is there anything that you would like to add on what we have shared?

Respondent: I have only a few things to add.

Interviewer: Mmmm (showing that one is following the conversation)

Respondent: My addition is more of an encouragement, because other women yeah

Interviewer: Mmmm (showing that one is following the conversation)

Respondent: Some women due to ignorance do not like the method.

Interviewer: Mmmm (showing that one is following the conversation)

Respondent: They say I cannot get this injection I can’t do this; such women are a lot.

Interviewer: Mmmm (showing that one is following the conversation)

Respondent: But I just see that its ignorance for them

Interviewer: Yah yah

Respondent: They have fears, you are supposed to remove fears until you have tried the thing

Interviewer: Until you have tried it

Respondent: Until you have tried it

Interviewer: Mmmm (showing that one is following the conversation)

Respondent: Afraid that when I do this, what problems could I experience.

Interviewer: Yah yah

Respondent: Yes

Interviewer: Alright, ah thank you for having the time to take part in this study, let me thank you on what you have helped me with. Like I said in the beginning your responses will be transcribed and they will be made anonymous, and they will be analyzed together with other responses from other participants in the study, do you have any questions before we finish?

Respondent: Ah my question is that I just wanted to ask…

Interviewer: Mmmm (showing that one is following the conversation)

Respondent: This injectable.

Interviewer: Mmmm (showing that one is following the conversation)

Respondent: Some people say that when you use it a lot you are no longer able to conceive

Interviewer: Mmmm (showing that one is following the conversation)

Respondent: I think about this question most of the times, but I forget to ask it…

Interviewer: Mmmm (showing that one is following the conversation)

Respondent: So I wanted to ask, some people say that yeah with Depo you can permanently stop conceiving, and some say that you do not stop conceiving, so that when I was asking what about periods, like as of now I don’t bleed anymore I just move around like a man, so that’s when I was asking am I not at risk?

Interviewer: No, you are not at any risk, because let’s assume that a woman has stopped using the injectable, since bodies are different, some will stop using the injection today and when they sleep with their partner, they may get pregnant only a few days after, and for some they can stop using the injection today and they take months ahead before they can pregnant, that is due to having different bodies. Some women can stay for the whole year after they stop using contraceptives without getting pregnant but it does not mean that they will no longer conceive for the rest of their reproductive life, no it is just the difference in the time that one will get pregnant again, some will take a longer time, it’s like the body is getting back to its normal state before you started using the injectables since there is a substance in the injection that makes the body to prevent getting pregnant so the uterus is in a state that it cannot accommodate pregnancy, we can say that the walls of the uterus are thin that pregnancy cannot be there. But when you stop using the injection the uterus gets back to a state where you can get pregnant. So the difference is just the body that for some will experience a headache, some will be okay, some will have excessive bleeding and some will just have light bleeding, so it just depends on the body.

Respondent: Alright.

Interviewer: Sure.

Respondent: Ah I have understood.

Interviewer: Thank you

Title: ZA_IM_9.docx

Descriptor Info:	District: Zomba	Target Population: TP2 - DMPA-IM/DMPA-SC PI clients	Respondent type: DMPA-IM client	Aware of SI: Yes - had heard of SI before	Coding phase: 1. Single coded	Client marital status: Married	Client age group: 20-29 years	Time since trained: N/A - client

Codes Applied:	C3.3.1 - Recos for what should be covered in counselling / training

Interviewer: So what else do you think women would like to know about the method before they decide to use it? What do you think women would want to know about the contraception method before they decide?

Respondent: Mhmm, they would know.

Interviewer: Mmh, as I have explained. What do you think they should be told?

Respondent: They will be told by health counsellors.

Interviewer: Yes, what will health counsellors tell women?

Respondent: They will be telling the women to come get the self-injecting method here at the hospital.

Interviewer: Mmh. So what are the thing the health counsellor are supposed to tell the women? (Outside noises; birds and a house fly) how can we convince women to use this method? What can we tell them?

Respondent: We could convince them by calling for a meeting, organize like a meeting, right?

Interviewer: Mmh.

Respondent: And tell them that, “iiih there is a new self-injecting injection here at the hospital”

Title: ZA_IM_9.docx

Descriptor Info:	District: Zomba	Target Population: TP2 - DMPA-IM/DMPA-SC PI clients	Respondent type: DMPA-IM client	Aware of SI: Yes - had heard of SI before	Coding phase: 1. Single coded	Client marital status: Married	Client age group: 20-29 years	Time since trained: N/A - client

Codes Applied:	C3.3.1 - Recos for what should be covered in counselling / training

Interviewer: Alright, thank you so much. So what help do you think women need to get to start using this method?

Respondent: When coming here they should be telling the women.

Interviewer: Mmh.

Respondent: They should be telling them that there are contraception methods.

Interviewer: Mmh.

Respondent: Which you self-inject. So women will understand right?

Interviewer: Mmh.

Respondent: They will be coming

Interviewer: Mmh.

Respondent: To get.

Interviewer: But the time they told you, you did not come. (Respondent Smiles) So what exactly should they be telling you to come get this method?

Respondent: when we come here to get contraceptives, they should be telling us.

Title: MZs_IM_1.docx

Descriptor Info:	District: Mzimba South	Target Population: TP2 - DMPA-IM/DMPA-SC PI clients	Respondent type: DMPA-IM client	Aware of SI: Yes - had heard of SI before	Coding phase: 1. Single coded	Client marital status: Married	Client age group: 30-39 years	Time since trained: N/A - client

Codes Applied:	C3.3.1 - Recos for what should be covered in counselling / training	C1.1.3 - Awareness of DMPA-SC SI	C8.1.1 - Decision making with partner

Interviewer: If it is okay by you, I would like to show you some pictures of this injectable form of contraception that can be self-injected to understand your thoughts and reactions. This method is currently introduced in Malawi so we want to see if it is something Malawian women would be interested in. Are you okay for me to show you the pictures?

Respondent: Yes.

Interviewer: Thanks.

Now administering DMPA-SC. This method is currently available in public health facilities and HSAs, but not from community-based distribution agents nor from youth centre or pharmacies’

Q16. Interviewer: After hearing this information do you think that women who are already using injectables methods might be more , less or equally interested in self injection option?

Respondent: As of now they are a lot of women who opt for self-injection.

Interviewer: You say they are many?

Respondent: Yes

Interviewer: If you say they are many can you compare those who uses providers injection?

Respondent: Those who opt for self-injection can be more than those who opt for providers injection.

Interviewer: Do you think some women will be more interested than others?

Respondent: Yes

Interviewer: Why do think some will be interested than others?

Respondent: The way the method can respond to them., for example two women might choose same method of family planning but one person might be bleeding too much while the other one no and other would say am experiencing headache while the other no.

Interviewer: What else do you think women would like to find out about this method before making a decision?

Respondent: They should first discuss it with their husbands before making a final decision about this method.

Interviewer: Do have anything to add?

Respondent: They should also find out if the method has any side effects before choosing it.

Title: KK_IM_1.docx

Descriptor Info:	District: Nkhotakota	Target Population: TP2 - DMPA-IM/DMPA-SC PI clients	Respondent type: DMPA-IM client	Aware of SI: No - never heard of SI before	Coding phase: 3. Triple coded	Client marital status: Married	Client age group: Under 20 (adolescent)	Time since trained: N/A - client

Codes Applied:	C3.3.1 - Recos for what should be covered in counselling / training

INTERVIEWER: Alright fine. What else do you think women would like to find out about this method before making a decision.? What do you think women would want to know about this new method before making a decision?

RESPONDENT: They should know all about this method.

INTERVIEWER: They should know about this method like what?

RESPONDENT: How to do self-injection

INTERVIEWER: Self-injection? What else

RESPONDENT: How to administer it

INTERVIEWER: What else?

RESPONDENT: The dates to administer the other doses

INTERVIEWER: Is that all they would want to know before taking the method?

RESPONDENT: Yes

Title: ZA_SCPI_8.docx

Descriptor Info:	District: Zomba	Target Population: TP2 - DMPA-IM/DMPA-SC PI clients	Respondent type: DMPA-SC PI client	Aware of SI: Yes - had heard of SI before	Coding phase: 1. Single coded	Client marital status:	Client age group:	Time since trained: N/A - client

Codes Applied:	C3.3.1 - Recos for what should be covered in counselling / training

Interviewer: What else do you think women need to know about self-injection before trying it?

Respondent: they can know that, since most people spread rumors that the injection brings so many problems…

Interviewer: mmmm (showing that one is following the conversation)

Respondent: But when you get to know more about the injectable after hearing from the providers you find that the injectable has not any problems and the fears can be dispelled, and you can end up using it with no problems.

Interviewer: mmmm (showing that one is following the conversation)

Respondent: yes

Title: ZA_SCPI_8.docx

Descriptor Info:	District: Zomba	Target Population: TP2 - DMPA-IM/DMPA-SC PI clients	Respondent type: DMPA-SC PI client	Aware of SI: Yes - had heard of SI before	Coding phase: 1. Single coded	Client marital status:	Client age group:	Time since trained: N/A - client

Codes Applied:	C3.3.1 - Recos for what should be covered in counselling / training

Interviewer: Have you tried using DMPA-SC for self-injection for more than once?

Respondent: no I have never tired it.

Interviewer: alright.

Respondent: yes

Interviewer: Ah why have you not tried it?

Respondent: because I was not trained properly, I did not receive the proper training.

Interviewer: mmmm (showing that one is following the conversation)

Respondent: but they also did not explain that this is how you perform self-injection very well.

Interviewer: mmmmm (showing that one is following the conversation)

Respondent: they also did not talk about the consequences.

Interviewer: alright.

Respondent: yes

Title: ZA_IM_7.docx

Descriptor Info:	District: Zomba	Target Population: TP2 - DMPA-IM/DMPA-SC PI clients	Respondent type: DMPA-IM client	Aware of SI: No - never heard of SI before	Coding phase: 1. Single coded	Client marital status: Never married	Client age group: 20-29 years	Time since trained: N/A - client

Codes Applied:	C3.3.1 - Recos for what should be covered in counselling / training

Interviewer: Alright what do you think women would like to know about this method before they decide to go for it?

Respondent: Mmmmmh I don’t know.

Title: MZs_IM_4.docx

Descriptor Info:	District: Mzimba South	Target Population: TP2 - DMPA-IM/DMPA-SC PI clients	Respondent type: DMPA-IM client	Aware of SI: Yes - had heard of SI before	Coding phase: 2. Double coded	Client marital status: Married	Client age group: 30-39 years	Time since trained: N/A - client

Codes Applied:	C3.3.1 - Recos for what should be covered in counselling / training

Q13. Interviewer: Was there any new information in the video that you had no heard before?

Respondent: No

Interviewer: What do you think are the most important things covered in the video?

Respondent: The information that is there in proving training session to a client for self-injection method. The other thing is that a woman will be able to stay at home her children for some time without visiting the facility.

Interviewer: If someone cab just watch this video, what would be the important thing that she could get from the video?

Respondent: The important thing is that after injecting yourself make sure that you keep the used syringe in the bottle and keep it well then bring it back to the provider whenever you are to get another medication/method.

Title: MZs_IM_4.docx

Descriptor Info:	District: Mzimba South	Target Population: TP2 - DMPA-IM/DMPA-SC PI clients	Respondent type: DMPA-IM client	Aware of SI: Yes - had heard of SI before	Coding phase: 2. Double coded	Client marital status: Married	Client age group: 30-39 years	Time since trained: N/A - client

Codes Applied:	C3.3.1 - Recos for what should be covered in counselling / training

Interviewer: Thanks. What else do you think women are supposed to know to know before choosing the method?

Respondent: The woman is supposed to know how to open the Sayana packet and then shake it very well so that the method should be well missed and then they should choose a place where they want to self-inject.

Interviewer: is there anything you want to add?

Respondent: The other thing is that after the injection a woman should make sure that she has kept the used syringe in a bottle and bring back to the provider.

Title: KK_IM_2.docx

Descriptor Info:	District: Nkhotakota	Target Population: TP2 - DMPA-IM/DMPA-SC PI clients	Respondent type: DMPA-IM client	Aware of SI: Yes - had heard of SI before	Coding phase: 2. Double coded	Client marital status: Married	Client age group: 30-39 years	Time since trained: N/A - client

Codes Applied:	C3.3.1 - Recos for what should be covered in counselling / training

INTERVIEWER: Now, as for you, what do you think are the important points that are in this video?

RESPONDENT: There are a few things, here, the important things are as I have explained that once you are given the injection to inject yourself at home, it is what I have explained that you save other things right? More especially with the coming of COVID 19 pandemic and also saving distance.

Title: KK_IM_2.docx

Descriptor Info:	District: Nkhotakota	Target Population: TP2 - DMPA-IM/DMPA-SC PI clients	Respondent type: DMPA-IM client	Aware of SI: Yes - had heard of SI before	Coding phase: 2. Double coded	Client marital status: Married	Client age group: 30-39 years	Time since trained: N/A - client

Codes Applied:	C3.3.1 - Recos for what should be covered in counselling / training

INTERVIEWER: What do you think women need to know before they start using Sayana? What do women need to know?

RESPONDENT: okay. Okay. Women need to know these methods… come again on the question, what does it say?

INTERVIEWER: What is it that you think women need to know about Sayana before they start using it? What does a woman need to know about this method of Sayana before using it?

RESPONDENT: There is need for them to receive some advice

INTERVIEWER: mmmhu.

RESPONDENT: From the doctor or any health personnel.

INTERVIEWER: okay.

RESPONDENT: mmmh.

Title: KK_IM_2.docx

Descriptor Info:	District: Nkhotakota	Target Population: TP2 - DMPA-IM/DMPA-SC PI clients	Respondent type: DMPA-IM client	Aware of SI: Yes - had heard of SI before	Coding phase: 2. Double coded	Client marital status: Married	Client age group: 30-39 years	Time since trained: N/A - client

Codes Applied:	C3.3.1 - Recos for what should be covered in counselling / training

INTERVIEWER: Do you have some last questions before we part?

RESPONDENT: I have a question.

INTERVIEWER: Please ask.

RESPONDENT: (laughs) As I was injected, should I want to put a Norplant is it possible?

Title: KK_IM_8.docx

Descriptor Info:	District: Nkhotakota	Target Population: TP2 - DMPA-IM/DMPA-SC PI clients	Respondent type: DMPA-IM client	Aware of SI: No - never heard of SI before	Coding phase: 1. Single coded	Client marital status: Married	Client age group: 20-29 years	Time since trained: N/A - client

Codes Applied:	C1.1.1 - Positive client attitudes to SI	C3.3.1 - Recos for what should be covered in counselling / training

INTERVIEWER: Alright. What else do you think women would like to know about this method before making a decision?

RESPONDENT: You would like to know the procedure on how to use Sayana and for you to understand the end result of using Sayana.

INTERVIEWER: okay.

RESPONDENT: sure.

INTERVIEWER: so with how I have explained to you. I have explained and you have understood right?

RESPONDENT: I have understood.

INTERVIEWER: you have understood?

RESPONDENT: yes.

INTERVIEWER: are you interested? Okay, when is your next visit for an injection?

RESPONDENT: December.

INTERVIEWER: December. Are you interested to start using this method…to be trained?

RESPONDENT: Yes, I am interested. I will come early so that I should be in health talk. I want them to explain to me everything including because when choosing a method, we want to know the good and the bad of that method. Nurses explains to us that every method has a good and bad part. And you choose depending on that.
