# Supplementary material for: Health care provider and client experiences of counselling on depot medroxyprogesterone acetate subcutaneous (DMPA-SC) for self-injection in Malawi
Source: PLOS Glob Public Health. 2023 Nov 30;3(11):e0002057. doi: 10.1371/journal.pgph.0002057 (PMC10688740; doi:10.1371/journal.pgph.0002057)
Supplement: S1 Data — (ZIP) [file pgph.0002057.s001.zip › S2/C3.3.1 recos couns SI only.doc]

Title: KK_SCSI_5.docx

Descriptor Info:	District: Nkhotakota	Target Population: TP3 - DMPA-SC SI clients	Respondent type: DMPA-SC SI client	Aware of SI: Yes - had heard of SI before	Coding phase: 2. Double coded	Client marital status: Married	Client age group: 20-29 years	Time since trained: N/A - client

Codes Applied:	C3.3.1 - Recos for what should be covered in counselling / training

INTERVIEWER: Alright there is nothing new? What do you think are the most important things?

RESPONDENT: The most important thing is the injection that will be used to inject ourselves

INTERVIEWER: What else

RESPONDENT: The other thing is the calendar that we should be using it

Title: KK_SCSI_5.docx

Descriptor Info:	District: Nkhotakota	Target Population: TP3 - DMPA-SC SI clients	Respondent type: DMPA-SC SI client	Aware of SI: Yes - had heard of SI before	Coding phase: 2. Double coded	Client marital status: Married	Client age group: 20-29 years	Time since trained: N/A - client

Codes Applied:	C3.3.1 - Recos for what should be covered in counselling / training

INTERVIEWER: Alright, What else do you think women needs to know about self -injection? In addition to what has been explained in the video what is it that women should know about self -injection?

RESPONDENT: Yes, to encourage them and give them advice in our women groups about taking self-injection

INTERVIEWER: Ok

RESPONDENT: We may be able to give our friends advice

Title: MZs_SCSI_3.docx

Descriptor Info:	District: Mzimba South	Target Population: TP3 - DMPA-SC SI clients	Respondent type: DMPA-SC SI client	Aware of SI: Yes - had heard of SI before	Coding phase: 1. Single coded	Client marital status: Married	Client age group: 30-39 years	Time since trained: N/A - client

Codes Applied:	C3.3.1 - Recos for what should be covered in counselling / training

Interviewer: do you think women from your community are more interested with self-injection?

Respondent: they have interest but what is needed is good counselling for them to be convinced

Title: MZs_SCSI_3.docx

Descriptor Info:	District: Mzimba South	Target Population: TP3 - DMPA-SC SI clients	Respondent type: DMPA-SC SI client	Aware of SI: Yes - had heard of SI before	Coding phase: 1. Single coded	Client marital status: Married	Client age group: 30-39 years	Time since trained: N/A - client

Codes Applied:	C3.3.1 - Recos for what should be covered in counselling / training	C8.1 - Male involvement, general

Interviewer: we have come to the end of this interview. Do you have anything to add?

Respondent: I think providers should have time to market and counsel on self-injection in the gatherings that happen in the community. This will help spread the right messages and clear the misconception that women have. Like my husband at first, he did not want me to use family planning but after the counselling he supported the idea

Title: MZs_SCSI_1.docx

Descriptor Info:	District: Mzimba South	Target Population: TP3 - DMPA-SC SI clients	Respondent type: DMPA-SC SI client	Aware of SI: Yes - had heard of SI before	Coding phase: 1. Single coded	Client marital status: Married	Client age group: Under 20 (adolescent)	Time since trained: N/A - client

Codes Applied:	C3.3.1 - Recos for what should be covered in counselling / training

nterviewer: What do you think are the most important things covered in the video?

Respondent: Concerning Sayana?

Interviewer: About Sayana the one you think is very important

Respondent: Injecting yourself

Interviewer: What is the important thing about injecting yourself?

Respondent: By giving us calendars I think that’s a very important thing on self – injection

Interviewer: The way you watched the video the only important thing is the calendar?

Respondent: Yes, the only important is the calendar

Title: MZs_SCSI_1.docx

Descriptor Info:	District: Mzimba South	Target Population: TP3 - DMPA-SC SI clients	Respondent type: DMPA-SC SI client	Aware of SI: Yes - had heard of SI before	Coding phase: 1. Single coded	Client marital status: Married	Client age group: Under 20 (adolescent)	Time since trained: N/A - client

Codes Applied:	C3.3.1 - Recos for what should be covered in counselling / training	A6.1.3 - Questions/ potential counselling content

nterviewer: The way you have seen the calendar what specifically do you think young adolescent might need to know about self - injection

Respondent: Those who are using it or those who are not?

Interviewer: Both those who have never used it or are using it

Respondent: For those who have been using it together we show each other and watch over each other whenever injecting the method but those that have never used, I think nothing.

Title: KK_SCSI_2.docx

Descriptor Info:	District: Nkhotakota	Target Population: TP3 - DMPA-SC SI clients	Respondent type: DMPA-SC SI client	Aware of SI: Yes - had heard of SI before	Coding phase: 1. Single coded	Client marital status: Divorced/ separated/ widowed	Client age group: 20-29 years	Time since trained: N/A - client

Codes Applied:	C3.3.1 - Recos for what should be covered in counselling / training

INTERVIEWER: Now you have seen the video, how do you feel about the training you received from the provider?

RESPONDENT: I can see that whatever we were trained is true, sometimes when were told something we feel its not be right , but now I believe that whatever were trained is really true.

INTERVIEWER: Are you agreeing with it?

RESPONDENT: Yes

Title: KK_SCSI_2.docx

Descriptor Info:	District: Nkhotakota	Target Population: TP3 - DMPA-SC SI clients	Respondent type: DMPA-SC SI client	Aware of SI: Yes - had heard of SI before	Coding phase: 1. Single coded	Client marital status: Divorced/ separated/ widowed	Client age group: 20-29 years	Time since trained: N/A - client

Codes Applied:	C3.3.1 - Recos for what should be covered in counselling / training

NTERVIWER: My last question, do you have any thoughts or information you would like to share on the topic we have discussed today

RESPONDENT : I may have a question

INTERVIEWER: Alright

RESPONDENT: Some people say that when you are taking Sayana you can stop doing your menses for 2 years and some say; after self-injection a woman should stay twelve days before sleeping with her husband is this true

Title: KK_SCSI_3.doc

Descriptor Info:	District: Nkhotakota	Target Population: TP3 - DMPA-SC SI clients	Respondent type: DMPA-SC SI client	Aware of SI: Yes - had heard of SI before	Coding phase: 1. Single coded	Client marital status: Married	Client age group: 20-29 years	Time since trained: N/A - client

Codes Applied:	C3.3.1 - Recos for what should be covered in counselling / training

NTERVIEWER: What do you think are the most important things covered in the video? What are the most important things?

RESPONDENT: The most important thing is that when you have self-injected and as you wait for the drugs to kick in, they have to give you condoms.

INTERVIEWER: okay. What are the other things that are important in the video?

RESPONDENT: I have just seen that one.

Title: KK_SCSI_3.doc

Descriptor Info:	District: Nkhotakota	Target Population: TP3 - DMPA-SC SI clients	Respondent type: DMPA-SC SI client	Aware of SI: Yes - had heard of SI before	Coding phase: 1. Single coded	Client marital status: Married	Client age group: 20-29 years	Time since trained: N/A - client

Codes Applied:	C3.3.1 - Recos for what should be covered in counselling / training

NTERVIEWER: What else do you think women need to know about self-injection? What else do women need to know about Sayana?

RESPONDENT: Women have to know how Sayana is and also how to hold it when shaking.

INTERVIEWER: mmmhu.

RESPONDENT: yes.

INTERVIEWER: Why are they supposed to know this?

RESPONDENT: Because it is a method they are interested in to be using

INTERVIEWER: mmmmmhu.

RESPONDENT: sure.

Title: KK_SCSI_4.docx

Descriptor Info:	District: Nkhotakota	Target Population: TP3 - DMPA-SC SI clients	Respondent type: DMPA-SC SI client	Aware of SI: Yes - had heard of SI before	Coding phase: 2. Double coded	Client marital status: Married	Client age group: Under 20 (adolescent)	Time since trained: N/A - client

Codes Applied:	C3.3.1 - Recos for what should be covered in counselling / training

INTERVIEWER: What do you think are the most important things covered in the video?

RESPONDENT: The injection

INTERVIEWER: What’s wrong with e injection? Why is it important if you feel it’s important?

RESPONDENT: Its important because we use it for self- injection

INTERVIEWER: What else did you that its important, a lot has been explained in the video? There was some advice, there was pros and cons of self-injection. So from all that what is it that you feel it’s important?

RESPONDENT: Family planning is important

INTERVIEWER: Is that the only point you have heard?

RESPONDENT: yes

Title: ZA_SCSI_3.docx

Descriptor Info:	District: Zomba	Target Population: TP3 - DMPA-SC SI clients	Respondent type: DMPA-SC SI client	Aware of SI: Yes - had heard of SI before	Coding phase: 3. Triple coded	Client marital status: Married	Client age group: 40-49 years	Time since trained: N/A - client

Codes Applied:	C1.1.1 - Positive client attitudes to SI	C2.1.2 - Reduced trips to facility	C88 - Client great quotes	C3.3.1 - Recos for what should be covered in counselling / training

Interviewer: What other thing do you think women need to know about self-injection?

Respondent: What women can know about this method is that it reduces the time we spend going to the hospital. For example, I and the women from my area have to come a long way just to reach the hospital. It is hard for us to make time for other farming activities and coming to the hospital, on top of that some of us do some peace works, so it is difficult to divide our time. In the end, we end up postponing the days we are supposed to visit the hospital. All this can however be avoided if we have the injections at our homes.

Interviewer: So what are women supposed to know about the self-injection method?

Respondent: They should know that this method saves one’s time of visiting the hospital. So it is a good method.

Title: ZA_SCSI_2.docx

Descriptor Info:	District: Zomba	Target Population: TP3 - DMPA-SC SI clients	Respondent type: DMPA-SC SI client	Aware of SI: Yes - had heard of SI before	Coding phase: 1. Single coded	Client marital status:	Client age group:	Time since trained: N/A - client

Codes Applied:	C3.3.1 - Recos for what should be covered in counselling / training

Interviewer: Alright what else do you think women need to know about DMPA SC?

Respondent: The things that they should know?

Interviewer: Yes, which things do you think the women also need to know?

Respondent: women need to know things like how to inject yourself

Interviewer: mmmm (showing that one is following the conversation)

Respondent: they should also follow the instructions that are given by the providers.

Interviewer: Alright.

Respondent: The way that they have indicated in the video.

Interviewer: mmhhh

Respondent: Yes

Interviewer: After you finished watching the video what do you think about the training that you received from the providers on DMPA SC?

Respondent: Ah the advice that I have gotten from this, in the video is that you need to inject yourself after every three months, you should follow the instructions given by the providers.

Interviewer: mmmm (showing that one is following the conversation)

Respondent: You also need to inject yourself by following the instructions

Interviewer: mmmm (showing that one is following the conversation)

Respondent: yes

Title: ZA_SCSI_2.docx

Descriptor Info:	District: Zomba	Target Population: TP3 - DMPA-SC SI clients	Respondent type: DMPA-SC SI client	Aware of SI: Yes - had heard of SI before	Coding phase: 1. Single coded	Client marital status:	Client age group:	Time since trained: N/A - client

Codes Applied:	C3.3.1 - Recos for what should be covered in counselling / training	A6.1.3 - Questions/ potential counselling content

Interviewer: Alright, what else do you think young girls need to know?

Respondent: Concerning DMPA SC?

Interviewer: Yes

Respondent: They should know the pros of this injection if they are younger.

Interviewer: mmmm (showing that one is following the conversation)

Respondent: They should know the pros and cons.

Title: ZA_SCSI_2.docx

Descriptor Info:	District: Zomba	Target Population: TP3 - DMPA-SC SI clients	Respondent type: DMPA-SC SI client	Aware of SI: Yes - had heard of SI before	Coding phase: 1. Single coded	Client marital status:	Client age group:	Time since trained: N/A - client

Codes Applied:	C3.3.1 - Recos for what should be covered in counselling / training	C8.3 - Discretion of injectables/SI

The girls that are below 18, do you think they would like this method?

Respondent: I thought people say that only those who are 18 and above should use contraceptives…

Interviewer: No

Respondent: What’s the correct age for starting to use contraceptives?

Interviewer: Those who have started experiencing menstrual periods. Do you think young girls would be attracted to this method?

Respondent: It can appeal to them because some of them, this method is good that you don’t need anyone to inject you, people cannot know that you are using contraceptives.

Interviewer: Alright.

Respondent: Yes

Title: ZA_SCSI_2.docx

Descriptor Info:	District: Zomba	Target Population: TP3 - DMPA-SC SI clients	Respondent type: DMPA-SC SI client	Aware of SI: Yes - had heard of SI before	Coding phase: 1. Single coded	Client marital status:	Client age group:	Time since trained: N/A - client

Codes Applied:	C3.3.1 - Recos for what should be covered in counselling / training

Respondent: I have a question.

Interviewer: mmm

Respondent: DMPA SC.

Interviewer: mmmm (showing that one is following the conversation)

Respondent: Does it have any other problems in the body?

Interviewer: The problems that you have seen in the video are what women experience, but since the bodies are different some women experience the problems, and some do not. Some also experience a few of the problems some only one because of having different bodies.

Respondent: mmmm (agreeing)

Interviewer: yes

Respondent: I heard that contraceptives have their own problems, when one grows up you start having problems like BP, anemia, is this true?

Title: ZA_SCSI_5.docx

Descriptor Info:	District: Zomba	Target Population: TP3 - DMPA-SC SI clients	Respondent type: DMPA-SC SI client	Aware of SI: Yes - had heard of SI before	Coding phase: 3. Triple coded	Client marital status: Married	Client age group: 30-39 years	Time since trained: N/A - client

Codes Applied:	C1.1.1 - Positive client attitudes to SI	C3.3.1 - Recos for what should be covered in counselling / training

Interviewer: Ah What things do you think are the most important in this video?

Respondent: The most important thing in this video is that it is good when you self-inject because you don’t have any concerns.

Interviewer: Mmm (showing that one is following the conversation)

Respondent: You will sleep with a man without worrying that you will get pregnant.

Interviewer: Mmm (showing that one is following the conversation)

Respondent: Eh so that’s the most important thing

Title: ZA_SCSI_5.docx

Descriptor Info:	District: Zomba	Target Population: TP3 - DMPA-SC SI clients	Respondent type: DMPA-SC SI client	Aware of SI: Yes - had heard of SI before	Coding phase: 3. Triple coded	Client marital status: Married	Client age group: 30-39 years	Time since trained: N/A - client

Codes Applied:	C3.3.1 - Recos for what should be covered in counselling / training	C2.1.2 - Reduced trips to facility

Interviewer: What other thigs do you think women need to know about self-injection?

Respondent: Women need to know that we are encouraging them that there is a new injectable Sayana which you can self-inject.

Interviewer: Mmm (showing that one is following the conversation)

Respondent: So you can just go and find out more about it at the hospital.

Interviewer: Mmm (showing that one is following the conversation)

Respondent: If you like it, you can access it or you can choose not to.

Interviewer: Mmm (showing that one is following the conversation)

Respondent: So the women when they come here, we see that they access it

Interviewer: Alright.

Respondent: They say that its good. It does not keep you busy

Interviewer: Mmm (showing that one is following the conversation)

Respondent: Yes so many women are accessing it

Title: ZA_SCSI_5.docx

Descriptor Info:	District: Zomba	Target Population: TP3 - DMPA-SC SI clients	Respondent type: DMPA-SC SI client	Aware of SI: Yes - had heard of SI before	Coding phase: 3. Triple coded	Client marital status: Married	Client age group: 30-39 years	Time since trained: N/A - client

Codes Applied:	C3.3.1 - Recos for what should be covered in counselling / training

Interviewer: Let me also thank you on what you have shared with me ah as I have said all your responses will be transcribed anonymously and they will be analysed together with the responses from other participants in the study. Do you have any questions before we finish?

Respondent: I have a question

Interviewer: Mmm (showing that one is following the conversation)

Respondent: The question is, the way that you have seen it

Interviewer: Eh

Respondent: DMPA-IM and all these other methods I have used.

Interviewer: Mmm (showing that one is following the conversation)

Respondent: And in choosing this one

Interviewer: Mmm (showing that one is following the conversation)

Respondent: Is it good or bad?

Interviewer: Sayana is a good method because the way you have seen that you spend the whole year without coming to the hospital concerning family planning

Title: MZs_SCSI_2.docx

Descriptor Info:	District: Mzimba South	Target Population: TP3 - DMPA-SC SI clients	Respondent type: DMPA-SC SI client	Aware of SI: Yes - had heard of SI before	Coding phase: 3. Triple coded	Client marital status: Married	Client age group: Under 20 (adolescent)	Time since trained: N/A - client

Codes Applied:	C3.3.1 - Recos for what should be covered in counselling / training

Interviewer: What do you think are the most important things covered in the video?

Respondent: The most important things I have seen is that you can stay on a year without visiting the facility.

Interviewer: What else do you think women need to know about self-injection?

Respondent: The things that women have to know is the counselling we are receiving from the providers.

Title: MZs_SCSI_2.docx

Descriptor Info:	District: Mzimba South	Target Population: TP3 - DMPA-SC SI clients	Respondent type: DMPA-SC SI client	Aware of SI: Yes - had heard of SI before	Coding phase: 3. Triple coded	Client marital status: Married	Client age group: Under 20 (adolescent)	Time since trained: N/A - client

Codes Applied:	C3.3.1 - Recos for what should be covered in counselling / training	C3.3.2 - Good (sufficient/ non-judgemental) counselling/training

Interviewer: The way you have seen this video, how do you feel about the training you received from the provider?

Respondent: The way I have seen the video there is nothing, may be because sometimes you forgot but the way I have seen it, what is needed is the remainder.

Title: MZs_SCSI_2.docx

Descriptor Info:	District: Mzimba South	Target Population: TP3 - DMPA-SC SI clients	Respondent type: DMPA-SC SI client	Aware of SI: Yes - had heard of SI before	Coding phase: 3. Triple coded	Client marital status: Married	Client age group: Under 20 (adolescent)	Time since trained: N/A - client

Codes Applied:	C3.3.1 - Recos for what should be covered in counselling / training	A6.1.3 - Questions/ potential counselling content	A3.1.4 - Community/"People"

Interviewer: What specifically do you think younger adolescents might need to know about self-injection? Bellow aged 18

Respondent: Young girls need to know the good thing about this method and also if there is some side effect about the method they need to know before making a decision, the good and the bad ones.

Interviewers: Alright.

Respondent: Others say that if someone starts using family planning while she has never given birth, she will not be able to have a child, so with this mentality they are supposed to go to the facility and ask for a clarification.

Title: MZs_SCSI_5.docx

Descriptor Info:	District: Mzimba South	Target Population: TP3 - DMPA-SC SI clients	Respondent type: DMPA-SC SI client	Aware of SI: Yes - had heard of SI before	Coding phase: 1. Single coded	Client marital status: Married	Client age group: 20-29 years	Time since trained: N/A - client

Codes Applied:	C3.3.1 - Recos for what should be covered in counselling / training

Interviewer: Aaah (thinking out loud) what do you think a woman should know before getting Sayana for self-injection?

Respondent: What you need to know before going?

Interviewer: Mmmh (agreeing)

Respondent: You need to go after 3 days of finishing menstrual period and without sexual intercourse with a man

Title: KK_SCSI_1.docx

Descriptor Info:	District: Nkhotakota	Target Population: TP3 - DMPA-SC SI clients	Respondent type: DMPA-SC SI client	Aware of SI: Yes - had heard of SI before	Coding phase: 3. Triple coded	Client marital status: Never married	Client age group: Under 20 (adolescent)	Time since trained: N/A - client

Codes Applied:	C3.3.1 - Recos for what should be covered in counselling / training

INTERVIEWER: Okay. What do you think are the most important things in the video? On what was being explained, what are the most important things?

RESPONDENT: The most important?

INTERVIEWER: mmmhu

Time stamp: (30:56)

RESPONDENT: It is important, let us take for example, after using Sayana, putting it at a safe place so that we can later on take it to the hospital for proper disposal.

INTERVIEWER: mmmhu.

RESPONDENT: The other thing is, it is ish… the need for [laughs, she was thinking of a proper response]

INTERVIEWER: Go on, those are your thoughts, they are your thoughts. Do not worry about anything that is what you think. You have told me about how to take care of Sayana after self-injecting yourself.

RESPONDENT: Because they say, we should take care of it and I have mentioned the need to shake the contents…to make sure that the medicine is well mixed right, yes, thereafter you inject yourself. I have observed that it’s important to shake the medicine because without it the medicine may not get mixed.

Title: KK_SCSI_1.docx

Descriptor Info:	District: Nkhotakota	Target Population: TP3 - DMPA-SC SI clients	Respondent type: DMPA-SC SI client	Aware of SI: Yes - had heard of SI before	Coding phase: 3. Triple coded	Client marital status: Never married	Client age group: Under 20 (adolescent)	Time since trained: N/A - client

Codes Applied:	C3.3.1 - Recos for what should be covered in counselling / training

INTERVIEWER: Mmmhu. Now to you, what do you think women are supposed to know about self-injecting contraceptive method? What else do women need to know about about this contraceptive method of …self-injecting Sayana. What else do women need to know?

RESPONDENT: mmmhu [paused before answering, she was thinking] another thing that women need to know about Sayana method?

INTERVIEWER: mmmhu.

RESPONDENT: (laughs). They are supposed to know that this method is effective for three months only right, that after the injection ways off they have to go to the hospital and get another one.

INTERVIEWER: Mmmhu. Now you have watched the video, right?

RESPONDENT: mmmh.

Title: KK_SCSI_1.docx

Descriptor Info:	District: Nkhotakota	Target Population: TP3 - DMPA-SC SI clients	Respondent type: DMPA-SC SI client	Aware of SI: Yes - had heard of SI before	Coding phase: 3. Triple coded	Client marital status: Never married	Client age group: Under 20 (adolescent)	Time since trained: N/A - client

Codes Applied:	C3.3.1 - Recos for what should be covered in counselling / training	A6.1.3 - Questions/ potential counselling content

INTERVIEWER: Now, what do you think young girls below the age of 18 right, need to know about Sayana contraceptive method? These girls, what is it that they need to know, those that are below the age of 18 right? These girls, what do they need to know?

RESPONDENT: About this method?

INTERVIEWER: Mmmh about Sayana

RESPONDENT: mmmhu [sigh, did not get the question]. Come again on that one

INTERVIEWER: Okay, what are the things that you think young girls below the age of 18 are supposed to know about Sayana contraceptive method? What is it that these girls are supposed to know?

RESPONDENT: They need to know that there is Sayana contraceptive method that can protect someone from getting an unwanted pregnancy.

Title: ZA_SCSI_4.docx

Descriptor Info:	District: Zomba	Target Population: TP3 - DMPA-SC SI clients	Respondent type: DMPA-SC SI client	Aware of SI: Yes - had heard of SI before	Coding phase: 2. Double coded	Client marital status: Married	Client age group: 30-39 years	Time since trained: N/A - client

Codes Applied:	C3.3.2 - Good (sufficient/ non-judgemental) counselling/training	C3.3.1 - Recos for what should be covered in counselling / training

Interviewer: Alright, what do you think are the most important things covered in the video?

Respondent: Most important things?

Interviewer: mmmmm (saying yes)

Respondent: it’s what they have encouraged, self-injecting, shaking the package

Interviewer: mmm (showing that one is following the conversation)

Respondent: yes, but also if you feel any problems in your body you should rush to the hospital.

Interviewer: mmm (showing that one is following the conversation)

Respondent: Those are the important things.

Interviewer: Alright, ah as far as you remember, are there any things in the video that the provider taught you and did not see them in the video?

Respondent: Ah no, everything that we were trained is in the video.

Interviewer: Alright.

Respondent: mmm (showing that one is following the conversation)

Title: ZA_SCSI_4.docx

Descriptor Info:	District: Zomba	Target Population: TP3 - DMPA-SC SI clients	Respondent type: DMPA-SC SI client	Aware of SI: Yes - had heard of SI before	Coding phase: 2. Double coded	Client marital status: Married	Client age group: 30-39 years	Time since trained: N/A - client

Codes Applied:	C3.3.1 - Recos for what should be covered in counselling / training

nterviewer: What else do you think women need to know about self-injection before they start using?

TIME INTO THE INTERVIEW: 20Mins 53Secs

Respondent: What they need to know like training them, training us about the body pains…

Interviewer: mmm (showing that one is following the conversation)

Respondent: Maybe gaining weight.

Interviewer: eh

Respondent: Headache

Interviewer: mmm (showing that one is following the conversation)

Respondent: Some feel some heaviness in the legs…

Interviewer: mmm (showing that one is following the conversation)

Respondent some feel pain in their feet.

Interviewer: mmm (showing that one is following the conversation)

Respondent: training them about this method so that when they see those side effects, they shouldn’t see like there is any problem that they should go to the hospital with and seek medical attention.

Interviewer: mmm (showing that one is following the conversation)

Respondent: Or that is a start of some illness, but they should know that it’s just the side effect of the injection. The injection is working

Title: ZA_SCSI_4.docx

Descriptor Info:	District: Zomba	Target Population: TP3 - DMPA-SC SI clients	Respondent type: DMPA-SC SI client	Aware of SI: Yes - had heard of SI before	Coding phase: 2. Double coded	Client marital status: Married	Client age group: 30-39 years	Time since trained: N/A - client

Codes Applied:	C3.3.1 - Recos for what should be covered in counselling / training

nterviewer. Alright, thank you so much for taking your time to take part in this study, I really appreciate your inputs. As I have said at the beginning your responses that you have shared with me will be transcribed anonymized and analyzed along with those from other participants. Do you have any questions before we finish?

Respondent: Ah my question is that, you have said that in the camera, in the video they said that some women are gaining weight

Interviewer: Yes.

Respondent: Some are having a headache.

Interviewer: Yes.

Respondent: Some are experiencing body pains

Interviewer: Yes

Respondent: So let’s assume that one has gained weight.

Interviewer: Yes

Respondent: What problems can you face as a result?

Interviewer: gaining weight is just like how everyone gains weight, yeah…

Respondent: mmmm (showing that one is following the conversation)

Interviewer: maybe it could be from eating too much food, maybe their bodies are just that way, so we cannot say that there is a problem specifically coming from the injection that one has encountered a problem because of the injection. Let’s look at the gaining weight…

Respondent: mmmm (showing that one is following the conversation)

Interviewer: but one needs to watch themselves.

Respondent: mmmm (showing that one is following the conversation)

Interviewer: your diet

Respondent: eh

Interviewer: so that you don’t have problems with your body, but there are no problems that come that we can connect to the injection.

Respondent: mmmm

Interviewer: It’s just like the way any person gains weight like for example men

Respondent: eh

Interviewer: Yes.

Respondent: Some women say that our men are saying that our bodies have changed we don’t feel the same way in bed, some are saying that its better they go and find other women. What happens for people to be saying this?

Interviewer: These are just false beliefs, but any family planning method, for example pills and the like does not change the way a person feels when they are having sex with their partner, the only one that can feel like something has changed is the condom.

Respondent: Mmmm mmmm (showing that one is following the conversation)

Interviewer: Because the condom is like you are putting on something external to your body.

Respondent: Yes.

Interviewer: Not the way it normally is other times. So what people say that they feel a difference when having sex is just a false belief.

Respondent: Mmmm (showing that one is following the conversation)

Interviewer: The same way people have false beliefs for example about the corona vaccine.

Respondent: Yes

Interviewer: That people will not be able to conceive, they are just myths which are not true.

Respondent: Alright, I have understood you I thank you for answering my questions.

Interviewer: Thank you

Respondent: Mmmm

Interviewer: Thank you

Title: MZs_SCSI_4.docx

Descriptor Info:	District: Mzimba South	Target Population: TP3 - DMPA-SC SI clients	Respondent type: DMPA-SC SI client	Aware of SI: Yes - had heard of SI before	Coding phase: 2. Double coded	Client marital status: Married	Client age group: 20-29 years	Time since trained: N/A - client

Codes Applied:	C3.3.3 - Poor (insufficient/ judgemental) counselling/training	C3.3.1 - Recos for what should be covered in counselling / training

nterviewer: Upon watching the video, what do you think is the most important thing you are supposed to know before self-injecting?

Respondent: How to use it

Interviewer: What else would you like to know as one of the women who is receiving self-inject method?

Respondent: To know the intervals for the doses on the calendar which are written by the provider. To wash hands before self-inject. Then open the contents and how to self-inject. Although they mentioned of opening a lid in the video, for us it was never mentioned. They also did not tell me to alternate the thighs when self-injecting I was doing it in one side of my thigh. Maybe I did not understand but I am very sure I followed what I was told.

Title: ZA_SCSI_1.docx

Descriptor Info:	District: Zomba	Target Population: TP3 - DMPA-SC SI clients	Respondent type: DMPA-SC SI client	Aware of SI: Yes - had heard of SI before	Coding phase: 1. Single coded	Client marital status: Married	Client age group: 40-49 years	Time since trained: N/A - client

Codes Applied:	C3.3.1 - Recos for what should be covered in counselling / training

Interviewer: What do you think the most important things in this video?

Respondent: Only two things

Interviewer: Mmmmmmmm

Respondent: If you have travelled and your date is due, you can just inject yourself and others hide it from their husbands so using self-injection he cannot be very much suspicious.

Interviewer: Okay, is there anything you remember?

Respondent: No, it’s only that.

Interviewer: Aaah okay. What do think women should know concerning self-injection method of planning?

Respondent: What they should know is you might travel a long distance so if your date is due then you can just inject yourself.
