# Supplementary material for: Health care provider and client experiences of counselling on depot medroxyprogesterone acetate subcutaneous (DMPA-SC) for self-injection in Malawi
Source: PLOS Glob Public Health. 2023 Nov 30;3(11):e0002057. doi: 10.1371/journal.pgph.0002057 (PMC10688740; doi:10.1371/journal.pgph.0002057)
Supplement: S1 Data — (ZIP) [file pgph.0002057.s001.zip › S2/C3.3.2 client percept good couns.doc]

Title: KK_IM_3.docx

Descriptor Info:	District: Nkhotakota	Target Population: TP2 - DMPA-IM/DMPA-SC PI clients	Respondent type: DMPA-IM client	Aware of SI: Yes - had heard of SI before	Coding phase: 1. Single coded	Client marital status: Divorced/ separated/ widowed	Client age group: 20-29 years	Time since trained: N/A - client

Codes Applied:	C3.2.2 - Lack of inclination or confidence to inject, even after training	C3.3.2 - Good (sufficient/ non-judgemental) counselling/training

INTERVIEWER: did you ever feel judged during these conversations?

RESPONDENT: No I have not

INTERVIEWER: As a result of the discussion, you had with the provider, did you agree to try self-injection?

RESPONDENT: Yes, I agreed but I did not take the method. But I recall they said it is a good method since anyone can use it, however, I have not used it so far.

INTERVIEWER: You have tried the method, but you agreed that you can use it

RESPONDENT: Ye I agreed that I can use it

Title: KK_IM_3.docx

Descriptor Info:	District: Nkhotakota	Target Population: TP2 - DMPA-IM/DMPA-SC PI clients	Respondent type: DMPA-IM client	Aware of SI: Yes - had heard of SI before	Coding phase: 1. Single coded	Client marital status: Divorced/ separated/ widowed	Client age group: 20-29 years	Time since trained: N/A - client

Codes Applied:	C3.3.2 - Good (sufficient/ non-judgemental) counselling/training

INTERVIEWER: Alright, as far as you remember, did the provider teach you anything that was not in the video.

RESPONDENT: No I saw everything

INTERVIEWER: Ok you mean everything that the provider trained you was covered in the video not so?

RESPONDENT: Yes

Title: MZs_IM_8.docx

Descriptor Info:	District: Mzimba South	Target Population: TP2 - DMPA-IM/DMPA-SC PI clients	Respondent type: DMPA-IM client	Aware of SI: Yes - had heard of SI before	Coding phase: 1. Single coded	Client marital status: Married	Client age group: 20-29 years	Time since trained: N/A - client

Codes Applied:	C3.3.2 - Good (sufficient/ non-judgemental) counselling/training

Interviewer: have you seen anything new in this video that you were not told?

Respondent: yes, I have

Interviewer: would you tell me?

Respondent: in the video they told the woman to dispose the used needle in a bottle closed with a lid and bring them to the provider

Interviewer: is it different from what you were told?

Respondent: maybe we were told but I was not paying much attention

Title: ZA_IM_2.docx

Descriptor Info:	District: Zomba	Target Population: TP2 - DMPA-IM/DMPA-SC PI clients	Respondent type: DMPA-IM client	Aware of SI: Yes - had heard of SI before	Coding phase: 1. Single coded	Client marital status: Married	Client age group: 30-39 years	Time since trained: N/A - client

Codes Applied:	C3.3.2 - Good (sufficient/ non-judgemental) counselling/training

Interviewer: Alright, did you feel judged when you were discussing about self-injection with the providers?

Respondent: No

Title: ZA_IM_2.docx

Descriptor Info:	District: Zomba	Target Population: TP2 - DMPA-IM/DMPA-SC PI clients	Respondent type: DMPA-IM client	Aware of SI: Yes - had heard of SI before	Coding phase: 1. Single coded	Client marital status: Married	Client age group: 30-39 years	Time since trained: N/A - client

Codes Applied:	C3.3.2 - Good (sufficient/ non-judgemental) counselling/training

Interviewer: Okay, Alright, I wanted to know if the training you received was comprehensive or as there something else that needed to be clarified?

Respondent: No, the training was okay.

Title: ZA_IM_2.docx

Descriptor Info:	District: Zomba	Target Population: TP2 - DMPA-IM/DMPA-SC PI clients	Respondent type: DMPA-IM client	Aware of SI: Yes - had heard of SI before	Coding phase: 1. Single coded	Client marital status: Married	Client age group: 30-39 years	Time since trained: N/A - client

Codes Applied:	C3.3.2 - Good (sufficient/ non-judgemental) counselling/training

Interviewer: Thank you, now after watching the video I wanted to ask you if there is anything new that you saw in this video that you have never heard.

Respondent: No there is nothing new.

Title: ZA_IM_1.docx

Descriptor Info:	District: Zomba	Target Population: TP2 - DMPA-IM/DMPA-SC PI clients	Respondent type: DMPA-IM client	Aware of SI: Yes - had heard of SI before	Coding phase: 1. Single coded	Client marital status: Married	Client age group: 40-49 years	Time since trained: N/A - client

Codes Applied:	C5.2.3 - Lack of resources/follow up materials to aid timely re-injection	C3.3.2 - Good (sufficient/ non-judgemental) counselling/training

Interviewer: Was there any new information in the video that you had not heard before?

Respondent: No, there is nothing new. Most of the things were explained to us by providers.

Interviewer: Was there any new information in the video that you had not heard before?

Respondent: For instance, we were not given the calendar. They just told us that when you self-inject for the first one, it will take 90 days and then the next one.

Interviewer: Didn’t you earlier mention that you got a calendar?

Respondent: A verbal calendar

Interviewer: You were not given?

Respondent: No we were not given, it was a verbal calendar explaining that after 90 days you should do it, just reading it out. But here there is a calendar from washing hands to self-injecting. This is what has caught my eye because you would know what to do when you forget. You would know at which stage you are and the next stage.

Interviewer: Alright alright, is there anything that the provider did not give you and is in the video?

Respondent: No, there is nothing else just the calendar.

Title: KK_SCPI_6.docx

Descriptor Info:	District: Nkhotakota	Target Population: TP2 - DMPA-IM/DMPA-SC PI clients	Respondent type: DMPA-SC PI client	Aware of SI: Yes - had heard of SI before	Coding phase: 1. Single coded	Client marital status: Married	Client age group: 20-29 years	Time since trained: N/A - client

Codes Applied:	C3.3 - Counselling / training approach for injectable users	C3.3.2 - Good (sufficient/ non-judgemental) counselling/training

NTERVIEWER: okay. Would you explain your experience learning about Sayana? What have you experienced while learning about Sayana from HSAs?

RESPONDENT: There are no problems at all, only thanksgiving that everything they taught us was well received. There are not any problems.

INTERVIEWER: okay. Do you think the HSAs took enough time teaching you?

RESPONDENT: No

INTERVIEWER: mmmmh?

RESPONDENT: By enough you mean each time we come to be taught?

INTERVIEWER: mmmh. Were they taking enough time teaching you Sayana?

RESPONDENT: Oh yes, they were really teaching us.

INTERVIEWER: Enough time?

RESPONDENT: aa[yes].

INTERVIEWER: Until you thought, you had understood?

RESPONDENT: mmmh.

INTERVIEWER: okay. So when teaching you were they filling a condom with sugar or sand to imitate a thigh or stomach?

Time stamp: (30:11)

RESPONDENT: mmmh

INTERVIEWER: Did the HSAs do this as they were teaching you?

RESPONDENT: No they did not do

INTERVIEWER: They did not do.

RESPONDENT: mmmmh.

INTERVIEWER: Did you think the lessons were simple?

RESPONDENT: aaaah, No, they are not simple.

INTERVIEWER: Not easy?

RESPONDENT: mmmhu.

INTERVIEWER: Why is that?

RESPONDENT: Its difficulties comes in because they want …they …(giggles)

INTERVIEWER: Explain, I am listening, they want.

RESPONDENT: (giggle)

INTERVIEWER: These lessons are easy. Why are they easy?

RESPONDENT: You mean easy when learning?

INTERVIEWER: yes

RESPONDENT: The HSAs?

INTERVIEWER: mmmh.

RESPONDENT: It is easy.

INTERVIEWER: How about to your side?

RESPONDENT: It is easy (laughs)

INTERVIEWER: Why?

RESPONDENT: It is easy because we also answer.

INTERVIEWER: You answer?

RESPONDENT mmmh.

INTERVIEWER: mmmhu.

RESPONDENT: We answer when asked a question.

Title: KK_SCPI_4.docx

Descriptor Info:	District: Nkhotakota	Target Population: TP2 - DMPA-IM/DMPA-SC PI clients	Respondent type: DMPA-SC PI client	Aware of SI: Yes - had heard of SI before	Coding phase: 2. Double coded	Client marital status: Never married	Client age group: 20-29 years	Time since trained: N/A - client

Codes Applied:	C3.3.2 - Good (sufficient/ non-judgemental) counselling/training

INTERVIEWER: Okay, as they were telling you this, did you feel judged in any way? When I say judged you know what I mean right?

RESPONDENT: mmmh mmmh[no].

INTERVIEWER: maybe mocking you, did you feel that?

RESPONDENT: No, they did not say anything.

Title: KK_SCPI_4.docx

Descriptor Info:	District: Nkhotakota	Target Population: TP2 - DMPA-IM/DMPA-SC PI clients	Respondent type: DMPA-SC PI client	Aware of SI: Yes - had heard of SI before	Coding phase: 2. Double coded	Client marital status: Never married	Client age group: 20-29 years	Time since trained: N/A - client

Codes Applied:	C3.3.2 - Good (sufficient/ non-judgemental) counselling/training

INTERVIEWER: Thank you, now let us finish

RESPONDENT: mmmmmh.

INTERVIEWER: aaa Is there anything new in the video that you have never heard about?

RESPONDENT: No

INTERVIEWER: mmmh?

RESPONDENT: There is none.

INTERVIEWER: Everything that is in the video is what the doctor told you?

RESPONDENT: It is what they were saying the

Title: KK_SCPI_4.docx

Descriptor Info:	District: Nkhotakota	Target Population: TP2 - DMPA-IM/DMPA-SC PI clients	Respondent type: DMPA-SC PI client	Aware of SI: Yes - had heard of SI before	Coding phase: 2. Double coded	Client marital status: Never married	Client age group: 20-29 years	Time since trained: N/A - client

Codes Applied:	C3.3.2 - Good (sufficient/ non-judgemental) counselling/training

INTERVIEWER: Concerning the things that you remember, is there anything that you remember to have been taught by the doctor, but it is not there in the video?

RESPONDENT: No, I think it is the same, the way it was taught there and the way it was done in the video.

INTERVIEWER: mmmmhu.

RESPONDENT: yea.

Title: MZs_IM_6.docx

Descriptor Info:	District: Mzimba South	Target Population: TP2 - DMPA-IM/DMPA-SC PI clients	Respondent type: DMPA-IM client	Aware of SI: Yes - had heard of SI before	Coding phase: 1. Single coded	Client marital status: Married	Client age group: Under 20 (adolescent)	Time since trained: N/A - client

Codes Applied:	C3.3.2 - Good (sufficient/ non-judgemental) counselling/training

Interviewer: So like I said that I will ask you some few questions after watching this video. After watching the video, did you get something new which was not mentioned by the provider?

Respondent: No [giggles] nothing new to me

Interviewer: Were all these taught?

Respondent: They explained to us. About the calendar, etc. all was said

Interviewer: What do you think is more crucial in this video?

Respondent: That when you use this method you use it whilst home so there is less burden on costs of travelling to the facility

Interviewer: Mmmh (agreeing) do you remember anything that was taught by the provider which was not mentioned in this video?

Respondent: Mmmmh no all was said

Title: KK_IM_7.docx

Descriptor Info:	District: Nkhotakota	Target Population: TP2 - DMPA-IM/DMPA-SC PI clients	Respondent type: DMPA-IM client	Aware of SI: Yes - had heard of SI before	Coding phase: 1. Single coded	Client marital status: Married	Client age group: 30-39 years	Time since trained: N/A - client

Codes Applied:	C3.3.2 - Good (sufficient/ non-judgemental) counselling/training

INTERVIEWER: Okay. How would you describe the friendliness of the training?

RESPONDENT: They were easy.

INTERVIEWER: okay.

RESPONDENT: mmmh.

Title: KK_IM_7.docx

Descriptor Info:	District: Nkhotakota	Target Population: TP2 - DMPA-IM/DMPA-SC PI clients	Respondent type: DMPA-IM client	Aware of SI: Yes - had heard of SI before	Coding phase: 1. Single coded	Client marital status: Married	Client age group: 30-39 years	Time since trained: N/A - client

Codes Applied:	C3.3.2 - Good (sufficient/ non-judgemental) counselling/training

INTERVIEWER: Was there any new information in the video that you had had not heard before?

RESPONDENT: no

INTERVIEWER: mmmh.

RESPONDENT: There was no new information.

INTERVIEWER: You have heard everything before and you have discussed with providers?

RESPONDENT: mmmmh.

Title: KK_IM_7.docx

Descriptor Info:	District: Nkhotakota	Target Population: TP2 - DMPA-IM/DMPA-SC PI clients	Respondent type: DMPA-IM client	Aware of SI: Yes - had heard of SI before	Coding phase: 1. Single coded	Client marital status: Married	Client age group: 30-39 years	Time since trained: N/A - client

Codes Applied:	C3.3.2 - Good (sufficient/ non-judgemental) counselling/training

NTERVIEWER: Yes, can you explain?

RESPONDENT: I should say (giggles). I should say that this a good method. With what I have seen in the video and what the providers were telling us, it is good. Yes, and there are no differences [between the video and providers].

INTERVIEWER: Tell me the truth. What is in the video and the provider did not tell you.

RESPONDENT: Nothing. Everything is the same.

INTERVIEWER: What about the provider did teach you but was not in the video?

RESPONDENT: There is nothing. What I have seen in the video, the provider did the exact same thing, there is no difference. Maybe for us it was because it was the first time. That is why I was confused.

INTERVIEWER: Mmmh what is…but you said they they did not show you the drug. Did the provider do this?

RESPONDENT: aah in terms of the drug, the provider did that.

INTERVIEWER: mmmhu.

RESPONDENT: Yea.

Title: ZA_IM_5.docx

Descriptor Info:	District: Zomba	Target Population: TP2 - DMPA-IM/DMPA-SC PI clients	Respondent type: DMPA-IM client	Aware of SI: Yes - had heard of SI before	Coding phase: 2. Double coded	Client marital status: Married	Client age group: 30-39 years	Time since trained: N/A - client

Codes Applied:	C3.3.2 - Good (sufficient/ non-judgemental) counselling/training

Interviewer: Alright, can you tell me how easy was the training that you received?

Respondent: How easy was the training?

Interviewer: Mmmm (showing that one is following the conversation), was the training clear, or were there other things that were not clear?

Respondent: The injectable, the way that they explained to us, yeah

Interviewer: Mmmm (showing that one is following the conversation)

Respondent: We understood, because they would repeat what they were training us more times.

Interviewer: Mmmm (showing that one is following the conversation)

Respondent: So we were able to understand

Interviewer: Mmmm (showing that one is following the conversation)

Respondent: But like on my side yeah

Interviewer: Mmmm (showing that one is following the conversation)

Respondent: The part that I did not understand was where I had to inject under the navel.

Interviewer: Mmmm (showing that one is following the conversation)

Respondent: That’s where I was confused a lot, I could not understand well.

Interviewer: Alright.

Respondent: But the one on the thigh was not confusing.

Title: ZA_IM_5.docx

Descriptor Info:	District: Zomba	Target Population: TP2 - DMPA-IM/DMPA-SC PI clients	Respondent type: DMPA-IM client	Aware of SI: Yes - had heard of SI before	Coding phase: 2. Double coded	Client marital status: Married	Client age group: 30-39 years	Time since trained: N/A - client

Codes Applied:	C3.3.2 - Good (sufficient/ non-judgemental) counselling/training

Interviewer: Thank you ah is there anything new in the video that you haven’t heard in the past?

Respondent: No

Title: ZA_IM_5.docx

Descriptor Info:	District: Zomba	Target Population: TP2 - DMPA-IM/DMPA-SC PI clients	Respondent type: DMPA-IM client	Aware of SI: Yes - had heard of SI before	Coding phase: 2. Double coded	Client marital status: Married	Client age group: 30-39 years	Time since trained: N/A - client

Codes Applied:	C3.3.2 - Good (sufficient/ non-judgemental) counselling/training

Interviewer: Alright, from what you remember are there things that you were trained but you did not see in the video?

Respondent: Ah what they have explained is what they told us in the training.

Interviewer: Alright.

Respondent: Like injecting under the navel you cannot see but on the thigh.

Interviewer: Mmmm (showing that one is following the conversation)

Respondent: At the beginning we were scared that ah we cannot manage

Interviewer: Mmmm (showing that one is following the conversation)

Respondent: But still after they encouraged us like they were doing our friend in the video…

Interviewer: Mmmm (showing that one is following the conversation)

Respondent: We also gotten used.

Title: ZA_SCPI_8.docx

Descriptor Info:	District: Zomba	Target Population: TP2 - DMPA-IM/DMPA-SC PI clients	Respondent type: DMPA-SC PI client	Aware of SI: Yes - had heard of SI before	Coding phase: 1. Single coded	Client marital status:	Client age group:	Time since trained: N/A - client

Codes Applied:	C3.3.2 - Good (sufficient/ non-judgemental) counselling/training

Interviewer: Alright, did you ever feel judged in these conversations?

Respondent: no I did not feel judged in any way.

Interviewer: alright.

Title: ZA_SCPI_8.docx

Descriptor Info:	District: Zomba	Target Population: TP2 - DMPA-IM/DMPA-SC PI clients	Respondent type: DMPA-SC PI client	Aware of SI: Yes - had heard of SI before	Coding phase: 1. Single coded	Client marital status:	Client age group:	Time since trained: N/A - client

Codes Applied:	C3.3.2 - Good (sufficient/ non-judgemental) counselling/training

Interviewer: Alright, can you explain to me your experience in being trained in DMPA-SC?

Respondent: There is not any bad thing I experienced, they just told me that the injectable can either be self-injected or by the provider.

Interviewer: mmmm (showing that one is following the conversation)

Respondent: yes

Title: ZA_SCPI_8.docx

Descriptor Info:	District: Zomba	Target Population: TP2 - DMPA-IM/DMPA-SC PI clients	Respondent type: DMPA-SC PI client	Aware of SI: Yes - had heard of SI before	Coding phase: 1. Single coded	Client marital status:	Client age group:	Time since trained: N/A - client

Codes Applied:	C3.3.2 - Good (sufficient/ non-judgemental) counselling/training

Interviewer: Alright, how would you describe the friendliness of the training that you received?

Respondent: it was friendlier and simple

Interviewer: Alright, Were you given anything to take back home after the training?

Title: ZA_SCPI_8.docx

Descriptor Info:	District: Zomba	Target Population: TP2 - DMPA-IM/DMPA-SC PI clients	Respondent type: DMPA-SC PI client	Aware of SI: Yes - had heard of SI before	Coding phase: 1. Single coded	Client marital status:	Client age group:	Time since trained: N/A - client

Codes Applied:	C3.3.2 - Good (sufficient/ non-judgemental) counselling/training

Interviewer: alright, was there any new information in the video that you have not heard before?

Respondent: no there is not any new information.

Title: MZs_SCPI_3.docx

Descriptor Info:	District: Mzimba South	Target Population: TP2 - DMPA-IM/DMPA-SC PI clients	Respondent type: DMPA-SC PI client	Aware of SI: Yes - had heard of SI before	Coding phase: 1. Single coded	Client marital status: Married	Client age group: 20-29 years	Time since trained: N/A - client

Codes Applied:	C3.3.2 - Good (sufficient/ non-judgemental) counselling/training

Interviewer: Did you ever feel judged during these conversations?

Respondent: No

Title: MZs_SCPI_3.docx

Descriptor Info:	District: Mzimba South	Target Population: TP2 - DMPA-IM/DMPA-SC PI clients	Respondent type: DMPA-SC PI client	Aware of SI: Yes - had heard of SI before	Coding phase: 1. Single coded	Client marital status: Married	Client age group: 20-29 years	Time since trained: N/A - client

Codes Applied:	C3.3.2 - Good (sufficient/ non-judgemental) counselling/training

Interviewer: How would you describe the friendliness of the training you received?

Respondent: The training was okay and good.

Interviewer: Why do you say the training was friendly?

Respondent: It’s because we were able to get what he was saying about the Sayana, thus why am saying it was simple and friendly.

Title: MZs_SCPI_3.docx

Descriptor Info:	District: Mzimba South	Target Population: TP2 - DMPA-IM/DMPA-SC PI clients	Respondent type: DMPA-SC PI client	Aware of SI: Yes - had heard of SI before	Coding phase: 1. Single coded	Client marital status: Married	Client age group: 20-29 years	Time since trained: N/A - client

Codes Applied:	C3.3.2 - Good (sufficient/ non-judgemental) counselling/training

Q13. Interviewer: Was there any new information in the video that you had not heard before?

Respondent: No

Interviewer: Were you told everything in the video?

Respondent: Yes.

Title: MZs_SCPI_3.docx

Descriptor Info:	District: Mzimba South	Target Population: TP2 - DMPA-IM/DMPA-SC PI clients	Respondent type: DMPA-SC PI client	Aware of SI: Yes - had heard of SI before	Coding phase: 1. Single coded	Client marital status: Married	Client age group: 20-29 years	Time since trained: N/A - client

Codes Applied:	C3.3.2 - Good (sufficient/ non-judgemental) counselling/training

Interviewer: The way you have seen the video, did the provider teach you anything that was not in the video?

Respondent: No

Interviewer: What else do you think women need to know about self-injection before trying it?

Respondent: [Quiet…….], I think the way they provide counsel it’s okay.

Interviewer: Is there anything that you can add?

Respondent: There is nothing to add.

Q14. Interviewer: After seeing the video, would you say you feel the same, more positive, or more negative about self-injection?

Respondent: It’s the same with the way the provider ex

Title: MZs_IM_4.docx

Descriptor Info:	District: Mzimba South	Target Population: TP2 - DMPA-IM/DMPA-SC PI clients	Respondent type: DMPA-IM client	Aware of SI: Yes - had heard of SI before	Coding phase: 2. Double coded	Client marital status: Married	Client age group: 30-39 years	Time since trained: N/A - client

Codes Applied:	C3.3.2 - Good (sufficient/ non-judgemental) counselling/training

Interviewer: So did you feel ever judged during these conversations?

Respondent: No, actually I decided to use it so that I should have a feel on how it works.

Title: MZs_IM_4.docx

Descriptor Info:	District: Mzimba South	Target Population: TP2 - DMPA-IM/DMPA-SC PI clients	Respondent type: DMPA-IM client	Aware of SI: Yes - had heard of SI before	Coding phase: 2. Double coded	Client marital status: Married	Client age group: 30-39 years	Time since trained: N/A - client

Codes Applied:	C3.3.2 - Good (sufficient/ non-judgemental) counselling/training

Q13. Interviewer: Was there any new information in the video that you had no heard before?

Respondent: No

Title: MZs_IM_4.docx

Descriptor Info:	District: Mzimba South	Target Population: TP2 - DMPA-IM/DMPA-SC PI clients	Respondent type: DMPA-IM client	Aware of SI: Yes - had heard of SI before	Coding phase: 2. Double coded	Client marital status: Married	Client age group: 30-39 years	Time since trained: N/A - client

Codes Applied:	C3.3.2 - Good (sufficient/ non-judgemental) counselling/training

Interviewer: The way you have seen in the video, do you think there is anything that the provider teach you but it is not in the video?

Respondent: No

Interviewer: According to the video, where can a woman inject herself?

Respondent: A woman can self-inject on the stomach and also on the upper leg (patchafu) .

Interviewer: But where did the provider say you can self-inject?

Respondent: The provider said we have three places where we can self-inject but, in the video, they have said we have only two places where we can self-inject.

Interviewer: Is that the only different that you have seen?

Respondent: Yes

Title: KK_IM_2.docx

Descriptor Info:	District: Nkhotakota	Target Population: TP2 - DMPA-IM/DMPA-SC PI clients	Respondent type: DMPA-IM client	Aware of SI: Yes - had heard of SI before	Coding phase: 2. Double coded	Client marital status: Married	Client age group: 30-39 years	Time since trained: N/A - client

Codes Applied:	C3.3.2 - Good (sufficient/ non-judgemental) counselling/training

INTERVIEWER: Okay. as they were telling you this, did you feel judged, when I say judged you know what I mean right? May be to say, they are mocking me or what?

RESPONDENT: okay.

INTERVIEWER: mmmh. Did you have any feeling of being judged?

RESPONDENT: No, there is nothing.

INTERVIEWER: Nothing, it was okay?

RESPONDENT: mmmh.

Title: KK_SCSI_3.doc

Descriptor Info:	District: Nkhotakota	Target Population: TP3 - DMPA-SC SI clients	Respondent type: DMPA-SC SI client	Aware of SI: Yes - had heard of SI before	Coding phase: 1. Single coded	Client marital status: Married	Client age group: 20-29 years	Time since trained: N/A - client

Codes Applied:	C3.4 - Time to train clients	C3.3.2 - Good (sufficient/ non-judgemental) counselling/training

NTERVIEWER: Okay, alright. Did you feel the provider spent enough time training you? Was the time spent training you enough?

RESPONDENT: yes.

INTERVIEWER: why was it enough?

RESPONDENT: we spent a lot of time at that place.

INTERVIEWER: you understood what they trained you?

RESPONDENT: I understood everything they trained me about.

Title: KK_SCSI_3.doc

Descriptor Info:	District: Nkhotakota	Target Population: TP3 - DMPA-SC SI clients	Respondent type: DMPA-SC SI client	Aware of SI: Yes - had heard of SI before	Coding phase: 1. Single coded	Client marital status: Married	Client age group: 20-29 years	Time since trained: N/A - client

Codes Applied:	C3.3 - Counselling / training approach for injectable users	C3.3.2 - Good (sufficient/ non-judgemental) counselling/training	C3.1.1 - Practice of SI during training

NTERVIEWER: You came here right, and you were trained…the training from the HSA was all about Sayana and what it’s all about and here you were actually trained on how you can self-inject. Did you get the chance to practice on what you were trained about?

RESPONDENT: mmmmh.

INTERVIEWER: what were you doing in this practice session?

RESPONDENT: (laughs). Practicing what they were doing.

INTERVIEWER: mmmhu.

RESPONDENT: hold like this and then inject.

INTERVIEWER: Ok. Did they give you a chance to…because when others are conducting the training, they use a condom filled with sugar as a thigh or around the abdomen and a person should try on that. Did that happen?

RESPONDENT: mmmh mmmh[no].

INTERVIEWER: Did they train you using the condom or the actual thigh?

RESPONDENT: real thigh.

INTERVIEWER: yours?

RESPONDENT: yes

INTERVIEWER: it was a group session or one to one?

RESPONDENT: one to one.

INTERVIEWER: one to one?

RESPONDENT: mmmh.

INTERVIEWER: okay. When they were training you, were you judged, when I say judged do you know the meaning?

RESPONDENT: mmmh mmmh [no]

INTERVIEWER: Maybe mocking you. Did you feel any judgement during the training?

RESPONDENT: no.

INTERVIEWER: everything went well?

RESPONDENT: mmmmh.

INTERVIEWER: okay. So they gave you a chance to try self-injection and you did very well, right?

RESPONDENT: mmmmh.

Title: KK_SCSI_3.doc

Descriptor Info:	District: Nkhotakota	Target Population: TP3 - DMPA-SC SI clients	Respondent type: DMPA-SC SI client	Aware of SI: Yes - had heard of SI before	Coding phase: 1. Single coded	Client marital status: Married	Client age group: 20-29 years	Time since trained: N/A - client

Codes Applied:	C3.3.2 - Good (sufficient/ non-judgemental) counselling/training

NTERVIEWER: Thank you. Was there any new information in the video that you have not heard before?

RESPONDENT: no. I have heard everything.

INTERVIEWER: everything. The HSA told you everything?

RESPONDENT: yes.

Title: KK_SCSI_4.docx

Descriptor Info:	District: Nkhotakota	Target Population: TP3 - DMPA-SC SI clients	Respondent type: DMPA-SC SI client	Aware of SI: Yes - had heard of SI before	Coding phase: 2. Double coded	Client marital status: Married	Client age group: Under 20 (adolescent)	Time since trained: N/A - client

Codes Applied:	C3.3.2 - Good (sufficient/ non-judgemental) counselling/training

INTERVIEWER: Please raise your voice, did you ever feel judged during the training?

RESPONDENT: (Participant not sure)

INTERVIEWER: Did you ever feel judged during the training

RESPONDENT: No

Title: KK_SCSI_4.docx

Descriptor Info:	District: Nkhotakota	Target Population: TP3 - DMPA-SC SI clients	Respondent type: DMPA-SC SI client	Aware of SI: Yes - had heard of SI before	Coding phase: 2. Double coded	Client marital status: Married	Client age group: Under 20 (adolescent)	Time since trained: N/A - client

Codes Applied:	C3.3.2 - Good (sufficient/ non-judgemental) counselling/training

INTERVIEWER: As far as you remember, did the provider teach you anything that was not in the video

RESPONDENT: Yes, there is

INTERVIEWER: You mean there is something that the provider trained you, but you did not see it in the video

RESPONDENT: No

INTERVIEWER: There none?

RESPONDENT: Yes

INTERVIEWER: Alright, what else do you think women should now about self-injection? Anything that you feel women should know?

RESPONDENT: Nothing

INTERVIEWER: there is nothing that women should, do u think what is in the video is enough?

RESPONDENT: Yes

INTERVIEWER: Now you have seen the video, how do you feel about the training you received from the provider? Was the training good, did the provider share everything? Or else they missed something. what would you say after watching the video?

RESPONDENT: They spoke everything

INTERVIEWER: The spoke everything that means the training was well done

RESPONDENT: Yes

Title: ZA_SCSI_3.docx

Descriptor Info:	District: Zomba	Target Population: TP3 - DMPA-SC SI clients	Respondent type: DMPA-SC SI client	Aware of SI: Yes - had heard of SI before	Coding phase: 3. Triple coded	Client marital status: Married	Client age group: 40-49 years	Time since trained: N/A - client

Codes Applied:	C3.3 - Counselling / training approach for injectable users	C3.3.2 - Good (sufficient/ non-judgemental) counselling/training

Interviewer: Thank you very much for watching this video. Is there anything that you saw new in this video?

Respondent: Nothing.

Interviewer: Everything in this video was explained to you?

Respondent: Yes.

Interviewer: Which things in this video do you think are more important?

Respondent: It has served as a reminder to us about the things we learnt. Also some of us were not give the calendars. We just take note of the days that we were given, and we inject ourselves on that day as the video has said which we were also taught by the HSA of our area.

Interviewer: Anything else?

Respondent: No

Interviewer: Is there anything that you were taught but it isn’t there in this video?

Respondent: No, everything is there.

Title: ZA_SCSI_3.docx

Descriptor Info:	District: Zomba	Target Population: TP3 - DMPA-SC SI clients	Respondent type: DMPA-SC SI client	Aware of SI: Yes - had heard of SI before	Coding phase: 3. Triple coded	Client marital status: Married	Client age group: 40-49 years	Time since trained: N/A - client

Codes Applied:	C3.3.2 - Good (sufficient/ non-judgemental) counselling/training	C2.1.2 - Reduced trips to facility	C1.2.1 - Negative client attitudes to SI	C1.1.1 - Positive client attitudes to SI

Interviewer: After finishing watching this video, what do you feel about the teaching you got?

Time stamp: 30:46

Respondent: After watching, I am now convinced that what we learnt is actually true and they also told that the things explained in the video happen. At first, I thought they were just trying to reduce their work or burden us with the job of injecting ourselves. Now I have been encouraged that this method is actually helpful since it helps reduce our trips to the hospital since we can check the calendars for our days and inject ourselves. This method is good, and a woman can successfully do family planning using it.

Title: ZA_SCSI_2.docx

Descriptor Info:	District: Zomba	Target Population: TP3 - DMPA-SC SI clients	Respondent type: DMPA-SC SI client	Aware of SI: Yes - had heard of SI before	Coding phase: 1. Single coded	Client marital status:	Client age group:	Time since trained: N/A - client

Codes Applied:	C3.3.2 - Good (sufficient/ non-judgemental) counselling/training

Interviewer: Alright, when you were being trained on how to self-inject did you feel judged in any way?

Respondent: No they did not judge us.

Title: ZA_SCSI_2.docx

Descriptor Info:	District: Zomba	Target Population: TP3 - DMPA-SC SI clients	Respondent type: DMPA-SC SI client	Aware of SI: Yes - had heard of SI before	Coding phase: 1. Single coded	Client marital status:	Client age group:	Time since trained: N/A - client

Codes Applied:	C3.3.2 - Good (sufficient/ non-judgemental) counselling/training

Interviewer: What do you think about the training that you received from the HSAs after you watched the video?

Respondent: My thought…

Interviewer: The training that you received, what do you think about this training after you have watched the video, was the training sufficient?

Respondent: No, the training that we received is similar to what is in the video.

Interviewer: Alright.

Respondent: That’s how the training was

Title: ZA_SCSI_5.docx

Descriptor Info:	District: Zomba	Target Population: TP3 - DMPA-SC SI clients	Respondent type: DMPA-SC SI client	Aware of SI: Yes - had heard of SI before	Coding phase: 3. Triple coded	Client marital status: Married	Client age group: 30-39 years	Time since trained: N/A - client

Codes Applied:	C3.3.2 - Good (sufficient/ non-judgemental) counselling/training	C3.1.2 - Confidence/comfort to inject after counselling/ training

Interviewer: Did you feel judged when you were being trained?

Respondent: No they just trained us like that

Interviewer: Mmm (showing that one is following the conversation)

Respondent: They trained us, and we got home, and we were able to do what we were taught and we saw that it worked.

Interviewer: Alright.

Respondent: So when I came here, I just told them that what you trained me I have been able to do

Title: ZA_SCSI_5.docx

Descriptor Info:	District: Zomba	Target Population: TP3 - DMPA-SC SI clients	Respondent type: DMPA-SC SI client	Aware of SI: Yes - had heard of SI before	Coding phase: 3. Triple coded	Client marital status: Married	Client age group: 30-39 years	Time since trained: N/A - client

Codes Applied:	C3.3.2 - Good (sufficient/ non-judgemental) counselling/training	C5.1.3 - Resources/follow up materials (e.g. calendar) to aid timely re-injection

Interviewer: In as far as you remember are there any things that the provider trained you but you did not see them in the video?

Respondent: Ah no

Interviewer: Alright.

Respondent: Because the calendar was given the one that we have seen in the video.

Interviewer: Alright.

Respondent: Yes, the things that you need to do, they told me

Title: ZA_SCSI_5.docx

Descriptor Info:	District: Zomba	Target Population: TP3 - DMPA-SC SI clients	Respondent type: DMPA-SC SI client	Aware of SI: Yes - had heard of SI before	Coding phase: 3. Triple coded	Client marital status: Married	Client age group: 30-39 years	Time since trained: N/A - client

Codes Applied:	C3.3.2 - Good (sufficient/ non-judgemental) counselling/training

Interviewer: Alright, after watching the video, what do you think of the training that you received from the providers on Sayana?

Respondent: After watching the video I have seen that it is similar to what was said at the training.

Title: ZA_SCSI_5.docx

Descriptor Info:	District: Zomba	Target Population: TP3 - DMPA-SC SI clients	Respondent type: DMPA-SC SI client	Aware of SI: Yes - had heard of SI before	Coding phase: 3. Triple coded	Client marital status: Married	Client age group: 30-39 years	Time since trained: N/A - client

Codes Applied:	C3.3.2 - Good (sufficient/ non-judgemental) counselling/training	C1.1.1 - Positive client attitudes to SI

Interviewer: Alright, I want to know ah do you think the providers gave you all the support you needed while using DMPA SC?

Respondent: The providers gave me enough support and I am grateful to them even up until now…

Interviewer: Alright.

Respondent: On what they did for me

Interviewer: Mmm (showing that one is following the conversation)

Respondent: Yes, because they did good things for me and I am grateful

Interviewer: Alright.

Respondent: They did a good thing to let us know about Sayana

Interviewer: Mmm (showing that one is following the conversation)

Respondent: Yes

Interviewer: Alright

Respondent: We did not know that there was a new method , we just knew about Depo.

Interviewer: Mmm (showing that one is following the conversation)

Respondent: So the coming of Sayana, we are happy and grateful.

Title: MZs_SCSI_2.docx

Descriptor Info:	District: Mzimba South	Target Population: TP3 - DMPA-SC SI clients	Respondent type: DMPA-SC SI client	Aware of SI: Yes - had heard of SI before	Coding phase: 3. Triple coded	Client marital status: Married	Client age group: Under 20 (adolescent)	Time since trained: N/A - client

Codes Applied:	C3.3.2 - Good (sufficient/ non-judgemental) counselling/training

Interviewer: Did you ever feel judged during your training?

Respondent: No

Title: MZs_SCSI_2.docx

Descriptor Info:	District: Mzimba South	Target Population: TP3 - DMPA-SC SI clients	Respondent type: DMPA-SC SI client	Aware of SI: Yes - had heard of SI before	Coding phase: 3. Triple coded	Client marital status: Married	Client age group: Under 20 (adolescent)	Time since trained: N/A - client

Codes Applied:	C3.3.2 - Good (sufficient/ non-judgemental) counselling/training

Q10. Was there any new information in the video that you had not heard before?

Respondent: No, there is nothing new.

Title: MZs_SCSI_2.docx

Descriptor Info:	District: Mzimba South	Target Population: TP3 - DMPA-SC SI clients	Respondent type: DMPA-SC SI client	Aware of SI: Yes - had heard of SI before	Coding phase: 3. Triple coded	Client marital status: Married	Client age group: Under 20 (adolescent)	Time since trained: N/A - client

Codes Applied:	C3.3.1 - Recos for what should be covered in counselling / training	C3.3.2 - Good (sufficient/ non-judgemental) counselling/training

Interviewer: The way you have seen this video, how do you feel about the training you received from the provider?

Respondent: The way I have seen the video there is nothing, may be because sometimes you forgot but the way I have seen it, what is needed is the remainder.

Title: MZs_SCSI_5.docx

Descriptor Info:	District: Mzimba South	Target Population: TP3 - DMPA-SC SI clients	Respondent type: DMPA-SC SI client	Aware of SI: Yes - had heard of SI before	Coding phase: 1. Single coded	Client marital status: Married	Client age group: 20-29 years	Time since trained: N/A - client

Codes Applied:	C3.3.2 - Good (sufficient/ non-judgemental) counselling/training

Interviewer: Like I said I have some questions based on the video you have just seen

Respondent: Mmmh (agreeing)

Interviewer: Is there anything new in the video that the provider did not talk about during counselling

Respondent: Nothing new

Title: KK_SCSI_1.docx

Descriptor Info:	District: Nkhotakota	Target Population: TP3 - DMPA-SC SI clients	Respondent type: DMPA-SC SI client	Aware of SI: Yes - had heard of SI before	Coding phase: 3. Triple coded	Client marital status: Never married	Client age group: Under 20 (adolescent)	Time since trained: N/A - client

Codes Applied:	C3.3.2 - Good (sufficient/ non-judgemental) counselling/training	C5.2.3 - Lack of resources/follow up materials to aid timely re-injection

INTERVIEWER: But it’s not an exam. Mmmhu, was there new things in the video that you had not heard before? In this video that you have watched, are there new things that you have not heard before?

RESPONDENT: They are no new things, but the only difference is about calendar…calendar which are not provided. So it is difficult for us to remember our dates.

INTERVIEWER: okay.

RESPONDENT: Because they don’t provide us with calendars. They just give us Sayana. So it is difficult. But there is no new thing that I have seen.

INTERVIEWER: okay. You no everything except for calendar?

RESPONDENT: mmh.

Title: KK_SCSI_1.docx

Descriptor Info:	District: Nkhotakota	Target Population: TP3 - DMPA-SC SI clients	Respondent type: DMPA-SC SI client	Aware of SI: Yes - had heard of SI before	Coding phase: 3. Triple coded	Client marital status: Never married	Client age group: Under 20 (adolescent)	Time since trained: N/A - client

Codes Applied:	C3.3.2 - Good (sufficient/ non-judgemental) counselling/training

INTERVIEWER: Now, depending on what you remember right, is there anything that the nurse had taught you, but you have not seen it in the video?

RESPONDENT: Anything that the nurse had taught me but I cannot see it in the video?

INTERVIEWER: mmmmh.

RESPONDENT: I think the content is the same.

INTERVIEWER: It is the same?

RESPONDENT: mmmh.

Title: KK_SCSI_1.docx

Descriptor Info:	District: Nkhotakota	Target Population: TP3 - DMPA-SC SI clients	Respondent type: DMPA-SC SI client	Aware of SI: Yes - had heard of SI before	Coding phase: 3. Triple coded	Client marital status: Never married	Client age group: Under 20 (adolescent)	Time since trained: N/A - client

Codes Applied:	C3.3.2 - Good (sufficient/ non-judgemental) counselling/training

INTERVIEWER: Now, what do you think about the lessons that you received from HSAs? Compare between the video and the the lessons you got from HSAs.

RESPONDENT: I think it is all the the same, because when I watch the video and look at what we were taught by HSAs, it is all the same.

Title: ZA_SCSI_4.docx

Descriptor Info:	District: Zomba	Target Population: TP3 - DMPA-SC SI clients	Respondent type: DMPA-SC SI client	Aware of SI: Yes - had heard of SI before	Coding phase: 2. Double coded	Client marital status: Married	Client age group: 30-39 years	Time since trained: N/A - client

Codes Applied:	C3.3.2 - Good (sufficient/ non-judgemental) counselling/training

Respondent: we van manage to do this the way that they trained us because they trained us well.

Title: ZA_SCSI_4.docx

Descriptor Info:	District: Zomba	Target Population: TP3 - DMPA-SC SI clients	Respondent type: DMPA-SC SI client	Aware of SI: Yes - had heard of SI before	Coding phase: 2. Double coded	Client marital status: Married	Client age group: 30-39 years	Time since trained: N/A - client

Codes Applied:	C3.3.2 - Good (sufficient/ non-judgemental) counselling/training

Interviewer: Alright, during the time that they were telling you about this injectable, did you feel judged in any way?

Respondent: No

Title: ZA_SCSI_4.docx

Descriptor Info:	District: Zomba	Target Population: TP3 - DMPA-SC SI clients	Respondent type: DMPA-SC SI client	Aware of SI: Yes - had heard of SI before	Coding phase: 2. Double coded	Client marital status: Married	Client age group: 30-39 years	Time since trained: N/A - client

Codes Applied:	C3.3.2 - Good (sufficient/ non-judgemental) counselling/training	C3.3.1 - Recos for what should be covered in counselling / training

Interviewer: Alright, what do you think are the most important things covered in the video?

Respondent: Most important things?

Interviewer: mmmmm (saying yes)

Respondent: it’s what they have encouraged, self-injecting, shaking the package

Interviewer: mmm (showing that one is following the conversation)

Respondent: yes, but also if you feel any problems in your body you should rush to the hospital.

Interviewer: mmm (showing that one is following the conversation)

Respondent: Those are the important things.

Interviewer: Alright, ah as far as you remember, are there any things in the video that the provider taught you and did not see them in the video?

Respondent: Ah no, everything that we were trained is in the video.

Interviewer: Alright.

Respondent: mmm (showing that one is following the conversation)

Title: ZA_SCSI_4.docx

Descriptor Info:	District: Zomba	Target Population: TP3 - DMPA-SC SI clients	Respondent type: DMPA-SC SI client	Aware of SI: Yes - had heard of SI before	Coding phase: 2. Double coded	Client marital status: Married	Client age group: 30-39 years	Time since trained: N/A - client

Codes Applied:	C3.3.2 - Good (sufficient/ non-judgemental) counselling/training

Interviewer: Alright, after seeing this video what do you think about the training that you received from the providers?

Respondent: After I watched the video and the training that I received from the providers…

Interviewer: mmm (showing that one is following the conversation)

Respondent: I can see that am able to do everything in order and there is no problem and the providers trained us well

Interviewer: Alright

Respondent: mmmm (showing agreement)

Title: MZs_SCSI_4.docx

Descriptor Info:	District: Mzimba South	Target Population: TP3 - DMPA-SC SI clients	Respondent type: DMPA-SC SI client	Aware of SI: Yes - had heard of SI before	Coding phase: 2. Double coded	Client marital status: Married	Client age group: 20-29 years	Time since trained: N/A - client

Codes Applied:	C1.2.2 - Fear of SI (idea or reality)	C3.3.2 - Good (sufficient/ non-judgemental) counselling/training

Interviewer: When they were teaching you, did you feel judged by providers?

Respondent: We did not feel judged but just had fears on how to self-inject since it’s not an easy procedure

Title: ZA_SCSI_1.docx

Descriptor Info:	District: Zomba	Target Population: TP3 - DMPA-SC SI clients	Respondent type: DMPA-SC SI client	Aware of SI: Yes - had heard of SI before	Coding phase: 1. Single coded	Client marital status: Married	Client age group: 40-49 years	Time since trained: N/A - client

Codes Applied:	C3.3.2 - Good (sufficient/ non-judgemental) counselling/training

Interviewer: Did you feel judged the time you were being taught?

Respondent: No, we were just taught fine furthermore they just encouraged us.

Title: ZA_SCSI_1.docx

Descriptor Info:	District: Zomba	Target Population: TP3 - DMPA-SC SI clients	Respondent type: DMPA-SC SI client	Aware of SI: Yes - had heard of SI before	Coding phase: 1. Single coded	Client marital status: Married	Client age group: 40-49 years	Time since trained: N/A - client

Codes Applied:	C3.3.2 - Good (sufficient/ non-judgemental) counselling/training

Interviewer: Is there anything new to you?

Respondent: No they aren’t.

Title: ZA_SCSI_1.docx

Descriptor Info:	District: Zomba	Target Population: TP3 - DMPA-SC SI clients	Respondent type: DMPA-SC SI client	Aware of SI: Yes - had heard of SI before	Coding phase: 1. Single coded	Client marital status: Married	Client age group: 40-49 years	Time since trained: N/A - client

Codes Applied:	C3.3.2 - Good (sufficient/ non-judgemental) counselling/training

Interviewer: Thanks. So can you explain to me with reference to the video how similar is it to what you learnt?

Respondent: It’s more less the same nothing much different.

Title: KK_SCSI_5.docx

Descriptor Info:	District: Nkhotakota	Target Population: TP3 - DMPA-SC SI clients	Respondent type: DMPA-SC SI client	Aware of SI: Yes - had heard of SI before	Coding phase: 2. Double coded	Client marital status: Married	Client age group: 20-29 years	Time since trained: N/A - client

Codes Applied:	C3.3 - Counselling / training approach for injectable users	C3.3.2 - Good (sufficient/ non-judgemental) counselling/training

Alright. Did you fee judged during your training? Judged it’s like someone talks bad of you. Did anything like that happen?

RESPONDENT: No

Title: KK_SCSI_5.docx

Descriptor Info:	District: Nkhotakota	Target Population: TP3 - DMPA-SC SI clients	Respondent type: DMPA-SC SI client	Aware of SI: Yes - had heard of SI before	Coding phase: 2. Double coded	Client marital status: Married	Client age group: 20-29 years	Time since trained: N/A - client

Codes Applied:	C3.3.2 - Good (sufficient/ non-judgemental) counselling/training

INTERVIEWER: So, we will continue with our questions as explained earlier on

RESPONDENT: Ok

INTERVIEWER: Was there any new information on the video that you had not heard before? And What was it?

RESPONDENT: There is nothing new

INTERVIEWER: Alright there is nothing new? What do you think are the most important things?

RESPONDENT: The most important thing is the injection that will be used to inject ourselves

INTERVIEWER: What else

RESPONDENT: The other thing is the calendar that we should be using it

INTERVIEWER: Is there anything else that you have heard in the video

RESPONDENT: No

INTERVIEWER: Alright, As far as you remember did the provider teach you anything that was not shown in the video?

RESPONDENT: No I saw everything

INTERVIEWER: What you were trained?

RESPONDENT: What I have seen is all I was trained, I have not seen anything new

Title: KK_SCSI_5.docx

Descriptor Info:	District: Nkhotakota	Target Population: TP3 - DMPA-SC SI clients	Respondent type: DMPA-SC SI client	Aware of SI: Yes - had heard of SI before	Coding phase: 2. Double coded	Client marital status: Married	Client age group: 20-29 years	Time since trained: N/A - client

Codes Applied:	C3.3.2 - Good (sufficient/ non-judgemental) counselling/training

INTERVIEWER: Now you have seen the video, how do you feel about the training you received from the provider?

RESPONDENT: I think it’s a good thing

INTERVIEWER: Ok, Was the training good?

RESPONDENT: Yes, the training was good

INTERVIEWER: Ok the training was good because all you have in the video is what you were trained on

RESPONDENT: Yes, it is the same

Title: MZs_SCSI_3.docx

Descriptor Info:	District: Mzimba South	Target Population: TP3 - DMPA-SC SI clients	Respondent type: DMPA-SC SI client	Aware of SI: Yes - had heard of SI before	Coding phase: 1. Single coded	Client marital status: Married	Client age group: 30-39 years	Time since trained: N/A - client

Codes Applied:	C3.3.2 - Good (sufficient/ non-judgemental) counselling/training

Interviewer: did you feel judged during counselling?

Respondent: not at all

Title: MZs_SCSI_1.docx

Descriptor Info:	District: Mzimba South	Target Population: TP3 - DMPA-SC SI clients	Respondent type: DMPA-SC SI client	Aware of SI: Yes - had heard of SI before	Coding phase: 1. Single coded	Client marital status: Married	Client age group: Under 20 (adolescent)	Time since trained: N/A - client

Codes Applied:	C3.3.2 - Good (sufficient/ non-judgemental) counselling/training

Interviewer: Did you feel judged during your training?

Respondent: No

Title: MZs_SCSI_1.docx

Descriptor Info:	District: Mzimba South	Target Population: TP3 - DMPA-SC SI clients	Respondent type: DMPA-SC SI client	Aware of SI: Yes - had heard of SI before	Coding phase: 1. Single coded	Client marital status: Married	Client age group: Under 20 (adolescent)	Time since trained: N/A - client

Codes Applied:	C3.3.2 - Good (sufficient/ non-judgemental) counselling/training

Q10. Interviewer: Was there any new information in the video that you have not heard about?

Respondent: Not at all

Title: KK_SCSI_2.docx

Descriptor Info:	District: Nkhotakota	Target Population: TP3 - DMPA-SC SI clients	Respondent type: DMPA-SC SI client	Aware of SI: Yes - had heard of SI before	Coding phase: 1. Single coded	Client marital status: Divorced/ separated/ widowed	Client age group: 20-29 years	Time since trained: N/A - client

Codes Applied:	C3.3 - Counselling / training approach for injectable users	C3.3.2 - Good (sufficient/ non-judgemental) counselling/training	C3.1.1 - Practice of SI during training	C3.4 - Time to train clients

NTERVIEWER: Thank you. The next question is, would you please describe your experience of being trained in self injection? To start with please describe who trained you.

RESPONDET: I was trained by the HSA

INTERVIEWER: It was the HSA not so

RESPONDENT: Yes

INTERVIEWER: So would you please describe what has been your experience of being trained in self-injection

RESPONDENT: We were told that after injecting ourselves we need to keep the injections safe. After injecting ourselves we should put the injection in a bottle, then after finish all the dosage we should take back to the hospital as we go to collect the next injection, to show the health personnel to prove that we used it instead of keeping it and deny the opportunity for others to access the injection.

INTERVIEWER: What did the HSA explain about self-injection, I think you have already explained something on this one

RESPOINDENT : Yes

INTERVIEWER: Is there anything that you forgot? that the HSA explained to you about self-injection

RESPONDENT: How to hold it

INTERVIEWER: yes

RESPONDENT: This is how we hold Sayana (showing the interviewer using hands) then we shake it, after shaking it we press its neck and then the needle is pushed inside then we hold the place where we want to inject ourselves and then we inject the needle. The moment we rearise that the needle is inside the skin then we press the medicine until all is finished then we start pulling out the needle little by little.

INTERVIEWER: Thanks you so much this is well explained.

RESPONDENT: Thanks

INTERVIEWER: Did you feel the provider spent enough time training you?

REPONDENT: No we were trained twice

INTERVIEWER: they just trained you twice, but was it enough time?

RESPONDENT: Yes, it was enough time

INTERVIEWER: Maybe it was just, they just took some time

RESPONDENT: Yes

INTERVIEWER: Did you get the chance to practice what you were trained?

RESPONDNET: Yes

Title: KK_SCSI_2.docx

Descriptor Info:	District: Nkhotakota	Target Population: TP3 - DMPA-SC SI clients	Respondent type: DMPA-SC SI client	Aware of SI: Yes - had heard of SI before	Coding phase: 1. Single coded	Client marital status: Divorced/ separated/ widowed	Client age group: 20-29 years	Time since trained: N/A - client

Codes Applied:	C3.3.2 - Good (sufficient/ non-judgemental) counselling/training

Was there any new information on the video that you had not heard before? And what was it?

RESPONDENT: Nothing

INTERVIEWER: All you have watched on the video you have heard it before?

RESPONDENT: yes

Title: KK_SCSI_5.docx

Descriptor Info:	District: Nkhotakota	Target Population: TP3 - DMPA-SC SI clients	Respondent type: DMPA-SC SI client	Aware of SI: Yes - had heard of SI before	Coding phase: 2. Double coded	Client marital status: Married	Client age group: 20-29 years	Time since trained: N/A - client

Codes Applied:	C3.3 - Counselling / training approach for injectable users	C3.3.2 - Good (sufficient/ non-judgemental) counselling/training

Alright. Did you feel judged during your training? Judged it’s like someone talks bad of you. Did anything like that happen?

RESPONDENT: No
