# Supplementary material for: Health care provider and client experiences of counselling on depot medroxyprogesterone acetate subcutaneous (DMPA-SC) for self-injection in Malawi
Source: PLOS Glob Public Health. 2023 Nov 30;3(11):e0002057. doi: 10.1371/journal.pgph.0002057 (PMC10688740; doi:10.1371/journal.pgph.0002057)
Supplement: S1 Data — (ZIP) [file pgph.0002057.s001.zip › S2/C3.3.3 client percept poor couns.doc]

Title: KK_IM_3.docx

Descriptor Info:	District: Nkhotakota	Target Population: TP2 - DMPA-IM/DMPA-SC PI clients	Respondent type: DMPA-IM client	Aware of SI: Yes - had heard of SI before	Coding phase: 1. Single coded	Client marital status: Divorced/ separated/ widowed	Client age group: 20-29 years	Time since trained: N/A - client

Codes Applied:	C3.3.3 - Poor (insufficient/ judgemental) counselling/training	C3.2.1 - Lack of SI practice during training

INTERVIEWER: Alright, next question is: would you please describe your experience of being trained in self-injection? What was your experience? For instance, did the provider spent enough time training you?

RESPONDENT: It did not take much time; I would not say I understood the method deeply no

INTERVIEWER: You were not fully trained?

RESPONDENT: Yes, I would not say I know it 100% then I might be lying.

INTERVIEWER: You did not undergo the training fully? Did you get any chance to practice on what you were trained?

RESPONDENT: No

INTERVIEWER: you did not?

RESPONDENT: Yes

INTERVIEWER: Ok, how would you describe the friendliness of the training you received. I recall you said you did not undergo a full training? Like explaining in details on how the method works.

RESPONDENT: Yes

Title: KK_IM_3.docx

Descriptor Info:	District: Nkhotakota	Target Population: TP2 - DMPA-IM/DMPA-SC PI clients	Respondent type: DMPA-IM client	Aware of SI: Yes - had heard of SI before	Coding phase: 1. Single coded	Client marital status: Divorced/ separated/ widowed	Client age group: 20-29 years	Time since trained: N/A - client

Codes Applied:	C3.3.3 - Poor (insufficient/ judgemental) counselling/training

INTERVIEWER: We are now proceeding with our discussions; was there any new information in the video that you had not heard before? What was it?

RESPONDENT: Not really there is nothing new, however the component on how to administer the injection was a bit new since I was not trained in detailed on how I can do self-injection.

INTERVIEWER: Ok so the provider did not clearly explain on how to do self-injection

RESPONDENT: Yes

INTERVIEWER: In the video it is clearly explained on how one can do self-injection

RESPONDENT: Yes

INTERVIEWER: Alright, What do you think are the most important things covered in the video?

RESPONDENT: I think it’s… it’s the way the method is administered since one has to take it home.

INTERVIEWER: What else?

RESPONDENT: The other think that I have learnt how the method works

INTERVIEWER: Like how it the FP method works

RESPONDENT: Yes, how it works

Title: ZA_IM_1.docx

Descriptor Info:	District: Zomba	Target Population: TP2 - DMPA-IM/DMPA-SC PI clients	Respondent type: DMPA-IM client	Aware of SI: Yes - had heard of SI before	Coding phase: 1. Single coded	Client marital status: Married	Client age group: 40-49 years	Time since trained: N/A - client

Codes Applied:	C3.3 - Counselling / training approach for injectable users	C3.3.3 - Poor (insufficient/ judgemental) counselling/training

Interviewer: Thank you very much, what happens at the facility? How long do providers take before they start helping you?

Respondent: When we get here at the hospital, we explain to the providers that we have come to access family planning services.

Interviewer: Mmmhhh

Respondent: So they take our health passport and look at the date we were supposed to come. If the dates are matching, the take the health passport and fill in the register. The start with injecting us then they write in the health passports when we are supposed to return to the hospital to get our injectable.

Interviewer: When you come to the hospital, how long does it take for providers to inject you, write in your health passport and fill in the register? How long does this take?

Respondent: This really doesn’t take time; about 5 to 10 mins everything is done.

Title: KK_SCPI_6.docx

Descriptor Info:	District: Nkhotakota	Target Population: TP2 - DMPA-IM/DMPA-SC PI clients	Respondent type: DMPA-SC PI client	Aware of SI: Yes - had heard of SI before	Coding phase: 1. Single coded	Client marital status: Married	Client age group: 20-29 years	Time since trained: N/A - client

Codes Applied:	C1.2.2 - Fear of SI (idea or reality)	C3.3 - Counselling / training approach for injectable users	C3.3.3 - Poor (insufficient/ judgemental) counselling/training

INTERVIEWER: mmmhu. You said you have discussed the self-injecting contraception with HSAs before?

RESPONDENT: mmmmh.

INTERVIEWER: Have you discussed it today?

RESPONDENT: Today we did not discuss but the nurse…we discussed with the nurse.

INTERVIEWER: What was the nurse saying?

RESPONDENT: Was saying, “Why do you still want us to inject you”? Why are you not getting the self-injecting one?

INTERVIEWER: okay.

RESPONDENT: yes

INTERVIEWER: The nurse asked you why…

RESPONDENT: Why are you not injecting yourself?

INTERVIEWER: You want me to be injecting you?

RESPONDENT: Yes

INTERVIEWER: So what did you say?

RESPONDENT: I said I have never taken the self-injecting type before.

INTERVIEWER: mmmhu

RESPONDENT: However, when they say…they just inject me I do not take the injection. Nevertheless, I should I want; I can take the injection and inject myself at home.

INTERVIEWER: What stops you from getting the self-injective injection as of now?

RESPONDENT: Encourages me?

INTERVIEWER: What makes you fail? You said should you want, you can get it.

RESPONDENT: mmmmmh.

INTERVIEWER: Why do you not get the self-injecting one to inject yourself at home but the doctor should be injecting you?

RESPONDENT: (Laughs) aaah what about just some fears? It was some fear to inject myself, but now I have observed that a lot of women some of them did not even go to school are doing it for themselves then I can inject myself also. Back then I was afraid, I was afraid but not anymore.

Title: KK_SCPI_6.docx

Descriptor Info:	District: Nkhotakota	Target Population: TP2 - DMPA-IM/DMPA-SC PI clients	Respondent type: DMPA-SC PI client	Aware of SI: Yes - had heard of SI before	Coding phase: 1. Single coded	Client marital status: Married	Client age group: 20-29 years	Time since trained: N/A - client

Codes Applied:	C3.3.3 - Poor (insufficient/ judgemental) counselling/training	C3.2.1 - Lack of SI practice during training	C3.2.2 - Lack of inclination or confidence to inject, even after training	C3.1.2 - Confidence/comfort to inject after counselling/ training	C1.2.2 - Fear of SI (idea or reality)

NTERVIEWER: okay. Are there any new things in the video that you have never heard about?

RESPONDENT: aaaah. No. there is nothing new.

INTERVIEWER: Nothing new?

RESPONDENT: mmmh.

INTERVIEWER: What do you think are the most important things that are in the video?

RESPONDENT: The idea to inject yourself, Sayana.

INTERVIEWER: mmmhu. What about it?

RESPONDENT: The most important thing is the injection; the way I have seen it as the woman explained that at home…when you collect three injections you will take a year before you go back for the same.

INTERVIEWER: okay. Are there things you remember to have been taught by the providers but are not in the video? Are there things that the provider or may be a nurse taught you about Sayana but are not there in the video?

RESPONDENT: Aaah no.

INTERVIEWER: All of them?

RESPONDENT: mmmh.

INTERVIEWER: What else do you think women need to know about Sayana before they start using it? What else do women need to know before they start using Sayana?

RESPONDENT: They need to know how to open the medicine, how to shake it the way they do. Yes, they need to know about that and also how to inject themselves before they collect it. They need to follow everything as it has been taught by the provider; we need to follow it before we collect Sayana.

INTERVIEWER: mmmhu. Now that you have finished watching the video, have your thoughts about Sayana changed?

RESPONDENT: Yes, they have changed.

INTERVIEWER: Changed, how?

RESPONDENT: Have changed… here they have explained that… we should say the fears I had are gone.

INTERVIEWER: okay.

RESPONDENT: I was afraid. However, the way it has been taught here about shaking and everything and what to do when you reach that day my thoughts have changed and there are no more worries.

INTERVIEWER: oh…okay. As you have watched the video, all the fears are gone?

RESPONDENT: Yes. they are gone.

INTERVIEWER: Is it different from how the nurse or HSAS taught you?

RESPONDENT: A little different.

INTERVIEWER: How little is it different?

RESPONDENT: As they were teaching, they did not remove the Sayana.

INTERVIEWER: ka…

RESPONDENT: They did not show us the injection. They were just explaining theoretically. They did not demonstrate the way it is demonstrated in the video.

INTERVIEWER: ok.

RESPONDENT: yes. They did not take the injection they were just explaining theoretically.

INTERVIEWER: okay.

RESPONDENT: eyetu. Sure.

Title: KK_SCPI_4.docx

Descriptor Info:	District: Nkhotakota	Target Population: TP2 - DMPA-IM/DMPA-SC PI clients	Respondent type: DMPA-SC PI client	Aware of SI: Yes - had heard of SI before	Coding phase: 2. Double coded	Client marital status: Never married	Client age group: 20-29 years	Time since trained: N/A - client

Codes Applied:	C3.2.2 - Lack of inclination or confidence to inject, even after training	C3.3.3 - Poor (insufficient/ judgemental) counselling/training

RESPONDENT: They were asking us, they were checking the names against the register, we should say, they took a calendar, yes. We did not discuss it properly because I said I would not be able to inject myself. I was asked if I could inject myself and I said no. The next question was on the number of times I have used contraceptions and I said this is my third. And she said who injects you when you get it, so I said I go to the people who know how to inject themselves. So they said I should learn to inject myself, and they started teaching me. But when I took the syringe to inject myself, I failed to do it. So they said wait outside your friends should also be assisted.

Title: KK_SCPI_4.docx

Descriptor Info:	District: Nkhotakota	Target Population: TP2 - DMPA-IM/DMPA-SC PI clients	Respondent type: DMPA-SC PI client	Aware of SI: Yes - had heard of SI before	Coding phase: 2. Double coded	Client marital status: Never married	Client age group: 20-29 years	Time since trained: N/A - client

Codes Applied:	C3.3.3 - Poor (insufficient/ judgemental) counselling/training	C3.2.2 - Lack of inclination or confidence to inject, even after training

NTERVIEWER: mmmhu. You want to tell me that when you come you get Sayana, how many do they give you?

RESPONDENT: Here at the hospital?

INTERVIEWER: mmmmh.

RESPONDENT: Then it is two, sometimes one, but when you say you inject yourself, they just give you to inject yourself and there you go.

INTERVIEWER: When you get here who injects…

RESPONDENT: the doctor injects me on the arm

INTERVIEWER: The doctor is the one who injects you.

RESPONDENT: Yes, on the arm. We just do this and they inject us. When the date they told you comes you come.

INTERVIEWER: Are they really injecting you with Sayana?

RESPONDENT: I do not know. It is in a packet…packet.

INTERVIEWER: How does it look like?

RESPONDENT: It is white.

INTERVIEWER: mmmhu.

RESPONDENT: In addition, the tube is short with a bulge.

INTERVIEWER: This, [showing her a picture of Sayana]

RESPONDENT: Yes, it is this one.

INTERVIEWER: Now when you come…

RESPONDENT: It is as round as this one. And inside of it are white drugs.

INTERVIEWER: When you come every time, the doctor injects you?

RESPONDENT: (giggles).

INTERVIEWER: Right?

RESPONDENT: yes. The first one was injected on my arm, and they said this is a self-injecting injection, we don’t do it for each other.

INTERVIEWER: As you have come today, are they going to inject you?

RESPONDENT: Today I have failed.

INTERVIEWER: Are they going to inject you?

RESPONDENT: No they have not.

INTERVIEWER: Oh, okay

RESPONDENT: mmmmh. They have not injected me.

INTERVIEWER: okay. You said this is the third time?

RESPONDENT: mmmmh.

INTERVIEWER: For the first and second occasions, the doctor injected you, and this is the third round?

RESPONDENT: Yes

INTERVIEWER: okay.

RESPONDENT: mmmh.

Title: ZA_IM_6.docx

Descriptor Info:	District: Zomba	Target Population: TP2 - DMPA-IM/DMPA-SC PI clients	Respondent type: DMPA-IM client	Aware of SI: Yes - had heard of SI before	Coding phase: 1. Single coded	Client marital status: Married	Client age group: 30-39 years	Time since trained: N/A - client

Codes Applied:	C3.3.3 - Poor (insufficient/ judgemental) counselling/training

Interviewer: Alright, have you ever talked about the self-injecting contraceptive method with your health counselors?

Respondent: No, I have not talked about it with my health counselors maybe because I got here late, and they already talked about it.

Title: MZs_IM_5.docx

Descriptor Info:	District: Mzimba South	Target Population: TP2 - DMPA-IM/DMPA-SC PI clients	Respondent type: DMPA-IM client	Aware of SI: Yes - had heard of SI before	Coding phase: 1. Single coded	Client marital status: Married	Client age group: 20-29 years	Time since trained: N/A - client

Codes Applied:	C3.3.3 - Poor (insufficient/ judgemental) counselling/training	C5.2.3 - Lack of resources/follow up materials to aid timely re-injection

Interviewer: I have a few questions based on the video. Have you seen anything new in the video?

Respondent: we were not given a calendar that’s the new thing I saw. The dates for next dose were recorded in our health passport book. We were not given a chance to ask questions like in the video. She just counselled us and demonstrated how to self-inject. If the counselling was like that one, we would have easily understood.

Interviewer: was there anything that the provider told you that is missing in the video?

Respondent: yes, we were told to use condoms the first 5 days which is missing in the video

Title: ZA_IM_4.docx

Descriptor Info:	District: Zomba	Target Population: TP2 - DMPA-IM/DMPA-SC PI clients	Respondent type: DMPA-IM client	Aware of SI: Yes - had heard of SI before	Coding phase: 1. Single coded	Client marital status: Married	Client age group: Under 20 (adolescent)	Time since trained: N/A - client

Codes Applied:	C3.3.3 - Poor (insufficient/ judgemental) counselling/training

(Baby calls the mother) Interviewer: Have you ever talked about this injection method of family planning with the nurses?

Respondent: Mmm mmmmh we have never talked about it

Title: MZs_IM_7.docx

Descriptor Info:	District: Mzimba South	Target Population: TP2 - DMPA-IM/DMPA-SC PI clients	Respondent type: DMPA-IM client	Aware of SI: Yes - had heard of SI before	Coding phase: 1. Single coded	Client marital status: Divorced/ separated/ widowed	Client age group: Under 20 (adolescent)	Time since trained: N/A - client

Codes Applied:	C3.3 - Counselling / training approach for injectable users	C3.3.3 - Poor (insufficient/ judgemental) counselling/training

Interviewer: you don’t know anyone? Have you ever talked with your provider about Sayana?

Respondent: no

Interviewer: today’s clinic, did he mention about Sayana?

Respondent: no

Title: KK_IM_7.docx

Descriptor Info:	District: Nkhotakota	Target Population: TP2 - DMPA-IM/DMPA-SC PI clients	Respondent type: DMPA-IM client	Aware of SI: Yes - had heard of SI before	Coding phase: 1. Single coded	Client marital status: Married	Client age group: 30-39 years	Time since trained: N/A - client

Codes Applied:	C3.3.3 - Poor (insufficient/ judgemental) counselling/training

NTERVIEWER: Did they tell you the pros and cons of this injectable?

RESPONDENT: No they did not. They were just encouraging us not to give birth like pigs. We should be using contraceptives for the child to grow healthy.

INTERVIEWER: What did they say a woman will benefit from using self-injection?

RESPONDENT: Aaaaaah [long one], I don’t know because that method…

INTERVIEWER: But you have said that gave you Sayana right? Right?

RESPONDENT: Yes.

INTERVIEWER: some other time, right?

RESPONDENT: Sure.

INTERVIEWER: So they gave you without telling you the pros and cons?

RESPONDENT: Mmmhu. They did not explain to me the advantages and its disadvantages.

INTERVIEWER: Why? So they just gave you with no explanation?

RESPONDENT: They were just giving us because we wanted it so that our children should be protected. That is why we were accepting for our protection.

Title: ZA_IM_5.docx

Descriptor Info:	District: Zomba	Target Population: TP2 - DMPA-IM/DMPA-SC PI clients	Respondent type: DMPA-IM client	Aware of SI: Yes - had heard of SI before	Coding phase: 2. Double coded	Client marital status: Married	Client age group: 30-39 years	Time since trained: N/A - client

Codes Applied:	C3.3.3 - Poor (insufficient/ judgemental) counselling/training

Respondent: But like on my side yeah

Interviewer: Mmmm (showing that one is following the conversation)

Respondent: The part that I did not understand was where I had to inject under the navel.

Interviewer: Mmmm (showing that one is following the conversation)

Respondent: That’s where I was confused a lot, I could not understand well.

Interviewer: Alright.

Respondent: But the one on the thigh was not confusing.

Title: ZA_IM_9.docx

Descriptor Info:	District: Zomba	Target Population: TP2 - DMPA-IM/DMPA-SC PI clients	Respondent type: DMPA-IM client	Aware of SI: Yes - had heard of SI before	Coding phase: 1. Single coded	Client marital status: Married	Client age group: 20-29 years	Time since trained: N/A - client

Codes Applied:	C3.3 - Counselling / training approach for injectable users	C3.3.3 - Poor (insufficient/ judgemental) counselling/training

Interviewer: Alright. You talked about the self-injecting method. Did you talk with health counsellors about the self-injecting contraception method?

Respondent: No

Interviewer: You have not talked about it with your health counsellors?

Respondent: Mmh.

Interviewer: Mmhmm.

Title: ZA_SCPI_8.docx

Descriptor Info:	District: Zomba	Target Population: TP2 - DMPA-IM/DMPA-SC PI clients	Respondent type: DMPA-SC PI client	Aware of SI: Yes - had heard of SI before	Coding phase: 1. Single coded	Client marital status:	Client age group:	Time since trained: N/A - client

Codes Applied:	C3.3.3 - Poor (insufficient/ judgemental) counselling/training

Interviewer: Have you tried using DMPA-SC for self-injection for more than once?

Respondent: no I have never tired it.

Interviewer: alright.

Respondent: yes

Interviewer: Ah why have you not tried it?

Respondent: because I was not trained properly, I did not receive the proper training.

Interviewer: mmmm (showing that one is following the conversation)

Respondent: but they also did not explain that this is how you perform self-injection very well.

Interviewer: mmmmm (showing that one is following the conversation)

Respondent: they also did not talk about the consequences.

Interviewer: alright.

Respondent: yes

Title: MZs_SCPI_3.docx

Descriptor Info:	District: Mzimba South	Target Population: TP2 - DMPA-IM/DMPA-SC PI clients	Respondent type: DMPA-SC PI client	Aware of SI: Yes - had heard of SI before	Coding phase: 1. Single coded	Client marital status: Married	Client age group: 20-29 years	Time since trained: N/A - client

Codes Applied:	C3.3.3 - Poor (insufficient/ judgemental) counselling/training

Interviewer: What happens at the facility?

Respondent: We do tell them that we have come for family planning, and they do give us.

Interviewer: Mmmmh (okay)

Respondent: And also, they do partial counselling and ask us some questions and we do tell them our experience upon using it.

Interviewer: Anything else?

Respondent: Nothing.

Title: KK_IM_2.docx

Descriptor Info:	District: Nkhotakota	Target Population: TP2 - DMPA-IM/DMPA-SC PI clients	Respondent type: DMPA-IM client	Aware of SI: Yes - had heard of SI before	Coding phase: 2. Double coded	Client marital status: Married	Client age group: 30-39 years	Time since trained: N/A - client

Codes Applied:	C3.3 - Counselling / training approach for injectable users	C3.3.3 - Poor (insufficient/ judgemental) counselling/training

INTERVIEWER: The lessons…the doctor already gave you. Again, here you have watched the video right. Is there anything new in the video that you were not taught? Okay, I should not say the doctor only, is there anything that you have never heard whether at home that is in the video?

RESPONDENT: Yes, it is there

INTERVIEWER: what is that?

RESPONDENT: Aaah I did not know that you are given the self-injecting injection after 9 months, I thought they give you after a year. I had never heard about that before. I thought, once you are given the injections at the hospital it takes a year before you go back. That part which I already explained that if one comes far away from the hospital, you save distance.

INTERVIEWER: Distance

RESPONDENT: Yes, but I did not know that it is 9 months.

INTERVIEWER: mmmmhu.

RESPONDENT: mmmh.

INTERVIEWER: okay, is there anything else, that you have never heard about but is in the video?

RESPONDENT: Nothing else, that is all.

INTERVIEWER: That is all?

RESPONDENT: mmmmh.

Title: KK_IM_2.docx

Descriptor Info:	District: Nkhotakota	Target Population: TP2 - DMPA-IM/DMPA-SC PI clients	Respondent type: DMPA-IM client	Aware of SI: Yes - had heard of SI before	Coding phase: 2. Double coded	Client marital status: Married	Client age group: 30-39 years	Time since trained: N/A - client

Codes Applied:	C3.3.3 - Poor (insufficient/ judgemental) counselling/training

NTERVIEWER: As they were teaching you about Sayana, what were they using?

RESPONDENT: Honestly, at that time it was just talking to us, was very busy because he was alone.

INTERVIEWER: okay.

RESPONDENT: Was injecting women and was tending to pregnant women. Yes, so she just enlightened us because she had too much work.

INTERVIEWER: Oh. So she did not even demonstrate.

RESPONDENT: No

INTERVIEWER: She just explained

RESPONDENT: eya. Yes

Title: MZs_SCSI_5.docx

Descriptor Info:	District: Mzimba South	Target Population: TP3 - DMPA-SC SI clients	Respondent type: DMPA-SC SI client	Aware of SI: Yes - had heard of SI before	Coding phase: 1. Single coded	Client marital status: Married	Client age group: 20-29 years	Time since trained: N/A - client

Codes Applied:	P3.2.1 - Lack of IEC/demo materials for counselling/training

Interviewer: Is there anything that you have seen in the video that your provider did not use when counselling

Respondent: I did not see a calendar

Interviewer: What else did you not see?

Respondent: All is similar even the procedure on how to self-inject. It’s only the calendar which I did not see.

Title: KK_SCSI_2.docx

Descriptor Info:	District: Nkhotakota	Target Population: TP3 - DMPA-SC SI clients	Respondent type: DMPA-SC SI client	Aware of SI: Yes - had heard of SI before	Coding phase: 1. Single coded	Client marital status: Divorced/ separated/ widowed	Client age group: 20-29 years	Time since trained: N/A - client

Codes Applied:	C3.3.3 - Poor (insufficient/ judgemental) counselling/training

NTERVIEWER: Did you ever feel judged during your training. Did you get what I said. Did you ever feel judged like someone talking in a rude way?

RESPONDENT Yes

INTERVIEWER: they talked very rude to you?

RESPONDENT: Yes

Title: KK_SCSI_3.doc

Descriptor Info:	District: Nkhotakota	Target Population: TP3 - DMPA-SC SI clients	Respondent type: DMPA-SC SI client	Aware of SI: Yes - had heard of SI before	Coding phase: 1. Single coded	Client marital status: Married	Client age group: 20-29 years	Time since trained: N/A - client

Codes Applied:	C3.3.3 - Poor (insufficient/ judgemental) counselling/training

NTERVIEWER: Now you have seen the video, how do you feel about the training you received from your provider? You have to compare the two, provider training and the video. What do you feel about the training you received from your provider?

RESPONDENT: The training I received from my provider is different because they have said that some get fat, some menstruate, some stomachache and some headache. They did not explain this.

INTERVIEWER: they did not explain that?

RESPONDENT: mmmm [no]

INTERVIEWER: but it’s in the video?

RESPONDENT: it’s in the video.

INTERVIEWER: Okay but you you said they told you that the contents of the drugs are the same?

RESPONDENT: yes.

INTERVIEWER: but they did not proceed to this point?

RESPONDENT: they did not.

Title: ZA_SCSI_5.docx

Descriptor Info:	District: Zomba	Target Population: TP3 - DMPA-SC SI clients	Respondent type: DMPA-SC SI client	Aware of SI: Yes - had heard of SI before	Coding phase: 3. Triple coded	Client marital status: Married	Client age group: 30-39 years	Time since trained: N/A - client

Codes Applied:	C3.3.3 - Poor (insufficient/ judgemental) counselling/training

Interviewer: Ah after watching the video, is there anything new that you have not heard before?

Respondent: Yes, I have seen some new things.

Interviewer: Aright, like what?

Respondent: Like having a headache

Interviewer: Mmm (showing that one is following the conversation)

Respondent: Feeling dizzy

Title: MZs_SCSI_2.docx

Descriptor Info:	District: Mzimba South	Target Population: TP3 - DMPA-SC SI clients	Respondent type: DMPA-SC SI client	Aware of SI: Yes - had heard of SI before	Coding phase: 3. Triple coded	Client marital status: Married	Client age group: Under 20 (adolescent)	Time since trained: N/A - client

Codes Applied:	C3.3.3 - Poor (insufficient/ judgemental) counselling/training

Interviewer: There is nothing new?

Respondent: Yes, only that in this video a client was given Sayana for 1 year without visiting the facility

Title: ZA_SCSI_4.docx

Descriptor Info:	District: Zomba	Target Population: TP3 - DMPA-SC SI clients	Respondent type: DMPA-SC SI client	Aware of SI: Yes - had heard of SI before	Coding phase: 2. Double coded	Client marital status: Married	Client age group: 30-39 years	Time since trained: N/A - client

Codes Applied:	C3.3.3 - Poor (insufficient/ judgemental) counselling/training

Interviewer: Ah thank you, after we have watched this video, is there anything new that you have not heard before?

Respondent: ah the new information maybe about the headache

Interviewer: mmm (showing that one is following the conversation)

Respondent: Or maybe the other things

Title: MZs_SCSI_4.docx

Descriptor Info:	District: Mzimba South	Target Population: TP3 - DMPA-SC SI clients	Respondent type: DMPA-SC SI client	Aware of SI: Yes - had heard of SI before	Coding phase: 2. Double coded	Client marital status: Married	Client age group: 20-29 years	Time since trained: N/A - client

Codes Applied:	C3.3.3 - Poor (insufficient/ judgemental) counselling/training

Is there anything new that you have heard in the video that you were not told before?

Respondent: Yes

Interviewer: Ok, would you mention them

Respondent: Firstly, the information that our friend received is not the same information we were told.

Interviewer: What was different?

Respondent: In the video they differentiated the self-inject and provider injected saying the self-injection is easier because we self- inject at home. However our friend received much better counselling on her fears.

Interviewer: Mhuuu

Respondent: For us we were given Sayana Press to self-inject and the calendar without detailed explanation

Interviewer: Anything else that you were not told that you have heard in the video?

Respondent: (attending to her child as he was crying) oooh I just forgot

Interviewer: What about on disposal…

Respondent: Oooh yes, we were told to through the used injection in the toilets whilst our friend was told to through it in a Frozy bottle and bring it back to the facility.

Title: MZs_SCSI_4.docx

Descriptor Info:	District: Mzimba South	Target Population: TP3 - DMPA-SC SI clients	Respondent type: DMPA-SC SI client	Aware of SI: Yes - had heard of SI before	Coding phase: 2. Double coded	Client marital status: Married	Client age group: 20-29 years	Time since trained: N/A - client

Codes Applied:	C3.3.3 - Poor (insufficient/ judgemental) counselling/training	C3.3.1 - Recos for what should be covered in counselling / training

nterviewer: Upon watching the video, what do you think is the most important thing you are supposed to know before self-injecting?

Respondent: How to use it

Interviewer: What else would you like to know as one of the women who is receiving self-inject method?

Respondent: To know the intervals for the doses on the calendar which are written by the provider. To wash hands before self-inject. Then open the contents and how to self-inject. Although they mentioned of opening a lid in the video, for us it was never mentioned. They also did not tell me to alternate the thighs when self-injecting I was doing it in one side of my thigh. Maybe I did not understand but I am very sure I followed what I was told.

Title: ZA_SCSI_1.docx

Descriptor Info:	District: Zomba	Target Population: TP3 - DMPA-SC SI clients	Respondent type: DMPA-SC SI client	Aware of SI: Yes - had heard of SI before	Coding phase: 1. Single coded	Client marital status: Married	Client age group: 40-49 years	Time since trained: N/A - client

Codes Applied:	C6 - Waste management	C3.3.3 - Poor (insufficient/ judgemental) counselling/training

Interviewer: What do you do with the remaining injections?

Respondent: Discard it

Interviewer: Where do you discard it

Respondent: Toilet

Interviewer: Okay, where did the medical assistant tell you to discard?

Respondent: Told us to throw away in the toilet not just anyhow.

Interviewer: Mmmmmmm, how about in the video?

Respondent: In the video they are discarding in a box

Title: ZA_HSA_1.docx

Descriptor Info:	District: Zomba	Target Population: TP1 - Providers/HSAs	Respondent type: HSA	Aware of SI: Yes - had heard of SI before	Coding phase: 1. Single coded	Client marital status: N/A - provider	Client age group: N/A - provider	Time since trained: Less than a year ago

Codes Applied:	C3.3.3 - Poor (insufficient/ judgemental) counselling/training	P3.1.2 - Provider skill/ability to effectively counsel on SI

Interviewer: In your view, what are the main factors that would discourage clients to take up the self-injection option?

Respondent: If they don’t have enough information

Interviewer: Mmmh

Respondent: If they come, we tell them that it is possible, and you will manage it

Interviewer: Alright

Respondent: If they don’t have enough information they would go back and say, “I won’t be able to self-inject”.
