# Supplementary material for: Health care provider and client experiences of counselling on depot medroxyprogesterone acetate subcutaneous (DMPA-SC) for self-injection in Malawi
Source: PLOS Glob Public Health. 2023 Nov 30;3(11):e0002057. doi: 10.1371/journal.pgph.0002057 (PMC10688740; doi:10.1371/journal.pgph.0002057)
Supplement: S1 Data — (ZIP) [file pgph.0002057.s001.zip › S2/C3.4 client percept time to train.doc]

Title: KK_IM_3.docx

Descriptor Info:	District: Nkhotakota	Target Population: TP2 - DMPA-IM/DMPA-SC PI clients	Respondent type: DMPA-IM client	Aware of SI: Yes - had heard of SI before	Coding phase: 1. Single coded	Client marital status: Divorced/ separated/ widowed	Client age group: 20-29 years	Time since trained: N/A - client

Codes Applied:	C3.4 - Time to train clients

INTERVIEWER: Alright, next question is: would you please describe your experience of being trained in self-injection? What was your experience? For instance, did the provider spent enough time training you?

RESPONDENT: It did not take much time; I would not say I understood the method deeply no

Title: KK_SCSI_5.docx

Descriptor Info:	District: Nkhotakota	Target Population: TP3 - DMPA-SC SI clients	Respondent type: DMPA-SC SI client	Aware of SI: Yes - had heard of SI before	Coding phase: 2. Double coded	Client marital status: Married	Client age group: 20-29 years	Time since trained: N/A - client

Codes Applied:	C3.4 - Time to train clients

INTERVIEWER: Alright it was the nurse? Did you feel the nurse spent enough time training you or else it was just for few moments?

RESPONDENT: They took some good minutes

INTERVIEWER: Ok

RESPONDENT: For us to understand

Title: ZA_HSA_2.docx

Descriptor Info:	District: Zomba	Target Population: TP1 - Providers/HSAs	Respondent type: HSA	Aware of SI: Yes - had heard of SI before	Coding phase: 3. Triple coded	Client marital status: N/A - provider	Client age group: N/A - provider	Time since trained: Less than a year ago

Codes Applied:	P3.4 - Time to counsel/train, general	C3.4 - Time to train clients

Interviewer: Does that influence whether some women might choose to take up self-injection or not?

Respondent: ah When the training is longer…

Interviewer: mmmhh (showing that one is following the conversation)

Respondent: eh It makes some clients not to understand about the methods of family planning…

Interviewer: mmm (showing that one is following the conversation)

Respondent: They become bored yeah…

Interviewer: mmm (showing that one is following the conversation)

Respondent: Ah but when the training, when you tell them that the training will take 15 minutes or 20 minutes, it is enough to tell the client the main important points. Because there are some cases where you might prolong and keep on repeating the things that you have already mentioned, and you keep repeating them…

Interviewer: mmm (showing that one is following the conversation)

Respondent: The clients become bored with that…

Title: MZs_SCSI_3.docx

Descriptor Info:	District: Mzimba South	Target Population: TP3 - DMPA-SC SI clients	Respondent type: DMPA-SC SI client	Aware of SI: Yes - had heard of SI before	Coding phase: 1. Single coded	Client marital status: Married	Client age group: 30-39 years	Time since trained: N/A - client

Codes Applied:	C3.4 - Time to train clients

Interviewer: did they take enough time to counsel?

Respondent: yes, they took their time. We were able to ask all the questions we had

Title: ZA_IM_2.docx

Descriptor Info:	District: Zomba	Target Population: TP2 - DMPA-IM/DMPA-SC PI clients	Respondent type: DMPA-IM client	Aware of SI: Yes - had heard of SI before	Coding phase: 1. Single coded	Client marital status: Married	Client age group: 30-39 years	Time since trained: N/A - client

Codes Applied:	C3.4 - Time to train clients

Interviewer: Alright, in your experience, did the provider who was training you on self-injection take enough time to train you about this?

Respondent: Yes, it was enough time.

Title: MZs_SCSI_1.docx

Descriptor Info:	District: Mzimba South	Target Population: TP3 - DMPA-SC SI clients	Respondent type: DMPA-SC SI client	Aware of SI: Yes - had heard of SI before	Coding phase: 1. Single coded	Client marital status: Married	Client age group: Under 20 (adolescent)	Time since trained: N/A - client

Codes Applied:	C3.4 - Time to train clients

Interviewer: Did you feel the provider spent enough time to train you?

Respondent: No, they did not spend enough time.

Interviewer: Was the time they spent on you okay or not okay?

Respondent: It was okay

Title: ZA_IM_1.docx

Descriptor Info:	District: Zomba	Target Population: TP2 - DMPA-IM/DMPA-SC PI clients	Respondent type: DMPA-IM client	Aware of SI: Yes - had heard of SI before	Coding phase: 1. Single coded	Client marital status: Married	Client age group: 40-49 years	Time since trained: N/A - client

Codes Applied:	C3.3 - Counselling / training approach for injectable users	C1.2.2 - Fear of SI (idea or reality)	C3.1.2 - Confidence/comfort to inject after counselling/ training	C2.1.2 - Reduced trips to facility	C3.2.1 - Lack of SI practice during training	C3.4 - Time to train clients

Interviewer: How did you first hear about it?

Respondent: When I first heard this, I was nervous that would I be able to inject myself because we were told that we have to inject ourselves below the belly button or on the thigh. I was afraid about the belly button thinking I would inject my intestines or anything else. We were then properly taught on how to inject ourselves.

Interviewer: Mmmhh

Respondent: So ahh I accepted it and I used it.

Interviewer: Do you know anyone who is using self-injection?

Respondent: Self injection is currently out of stock, so I would not know

Interviewer: Mmmmhhh

Respondent: Yes

Interviewer: At the time it was available did you know anyone who was using it?

Respondent: Yes, I knew someone who was using it at that time

Interviewer: Alright, alright, have you spoken about DMPA-SC for self-injection with any provider?

Respondent: Yes, we have spoken about it

Interviewer: Mmmmhhh

Respondent: We were taught on how we can use the injection, how we can inject as well as the days when we are supposed to wait till the injection is ineffective in our bodies so that we can re inject the second one.

Interviewer: Can you tell me more about those conversations? How did the provider describe it to you? Tell me in detail what you were told?

Respondent: The provider explained that with this type of injection everyone is supposed to self-inject and the position is either below the belly button or on the thigh. So they explained on how to inject as well as the days we are supposed to wait for the medicine to be effective when re injecting.

Interviewer: Mmmmhhh

Respondent: They explained that when we first inject ourselves we should wait for 90days which is three months. After the 90 days we should inject and so on.

Interviewer: Mmmhhh alright, did they tell you the pros and cons of self-injection?

Respondent: They told us about the pros that sometimes people get lazy or get busy to get injected by provider. With self-injection there are no problems to inject yourself. These are the pros

Interviewer: Did they explain the cons of self-injection?

Respondent: No, they did not explain

Time stamp: 19 mins 38 secs

Interviewer: Alright thank you very much. As a result of the discussion you had with a provider, did you agree to try self-injection?

Respondent: Yes, I agreed and I tried it

Interviewer: Mmmmhhh What influenced you to try the self-injection?

Respondent: I saw that self-injection was a way that I could freely self-inject. The places where I inject are places were am free and no one is there.

Interviewer: What influenced you to self-inject?

Respondent: I was influenced with the fact that I want to family plan and avoid getting pregnant

Interviewer: Alright, alright, can you describe your experience of being trained in self-injection?

Respondent: The time they were explaining about self-injection

Interviewer: mmmhhh

Respondent: I was nervous that they are possibilities of hurting myself as well as not fully injecting due to pain as well as the medicine not getting inside. Once I fully understood I found that I accepted it and I started self-injecting without any difficulties

Interviewer: Alright, alright, in your view do you feel the provider spent enough time training you?

Respondent: Yes, the time was adequate

Interviewer: Did you get the chance to practice on what you were taught? For instance, pricking a condom filled with sugar or sand?

Respondent: No, we didn’t do that

Interviewer: Alright, did you self-inject?

Respondent: Yes

Interviewer: How can you describe the friendliness of the training you received?

Respondent: The friendliness of the training on self-injection?

Interviewer: Mmmmhhh

Respondent: It was easy because it was an easy way, it’s not like it was hard to understand when being taught. It was quite easy, and we practiced and got it.

Interviewer: Yes, what were you given to take home after your training for instance how many injectables?

Respondent: 3

Interviewer: Were you given any paper with instructions on how to self-inject at home?

Respondent: No, the just explained on how we can self-inject

Interviewer: Alright, were you given a calendar?

Respondent: Yes, we were given a calendar

Interviewer: So you got all these?

Respondent: Yes, we got them at that time

Title: KK_SCPI_6.docx

Descriptor Info:	District: Nkhotakota	Target Population: TP2 - DMPA-IM/DMPA-SC PI clients	Respondent type: DMPA-SC PI client	Aware of SI: Yes - had heard of SI before	Coding phase: 1. Single coded	Client marital status: Married	Client age group: 20-29 years	Time since trained: N/A - client

Codes Applied:	C3.3 - Counselling / training approach for injectable users	C3.4 - Time to train clients

NTERVIEWER: mmmhu. When you get here at the hospital on the day that you are supposed to get the injection, what happens?

RESPONDENT: When we arrive, we get on the scale. After the scale, we go into the room where the nurse is. The nurse takes the details and asks us some questions thereafter we are injected.

INTERVIEWER: Okay then…

RESPONDENT: The nurse also asks us if we have had any blood tests, and when it is our time, the nurse advises us to take some blood tests first.

INTERVIEWER: mmmhu. Apart from asking you about blood tests…how long, does the nurse take to help you? When you arrive, how long do you wait?

RESPONDENT: Today I arrived at eight, and everything was fast by half past eight I was injected.

Time stamp: (20:25).

INTERVIEWER: When you go to the nurse, you said what happens.

RESPONDENT: She tests us with something here, she asks us what problems we are experiencing with it, and we answer depending on what you are experiencing then the nurse takes those details down and then we get injected.

INTERVIEWER: okay.

RESPONDENT: mmmh.

INTERVIEWER: Now, I would like to disc

Title: KK_SCSI_2.docx

Descriptor Info:	District: Nkhotakota	Target Population: TP3 - DMPA-SC SI clients	Respondent type: DMPA-SC SI client	Aware of SI: Yes - had heard of SI before	Coding phase: 1. Single coded	Client marital status: Divorced/ separated/ widowed	Client age group: 20-29 years	Time since trained: N/A - client

Codes Applied:	C3.3 - Counselling / training approach for injectable users	C3.3.2 - Good (sufficient/ non-judgemental) counselling/training	C3.1.1 - Practice of SI during training	C3.4 - Time to train clients

NTERVIEWER: Thank you. The next question is, would you please describe your experience of being trained in self injection? To start with please describe who trained you.

RESPONDET: I was trained by the HSA

INTERVIEWER: It was the HSA not so

RESPONDENT: Yes

INTERVIEWER: So would you please describe what has been your experience of being trained in self-injection

RESPONDENT: We were told that after injecting ourselves we need to keep the injections safe. After injecting ourselves we should put the injection in a bottle, then after finish all the dosage we should take back to the hospital as we go to collect the next injection, to show the health personnel to prove that we used it instead of keeping it and deny the opportunity for others to access the injection.

INTERVIEWER: What did the HSA explain about self-injection, I think you have already explained something on this one

RESPOINDENT : Yes

INTERVIEWER: Is there anything that you forgot? that the HSA explained to you about self-injection

RESPONDENT: How to hold it

INTERVIEWER: yes

RESPONDENT: This is how we hold Sayana (showing the interviewer using hands) then we shake it, after shaking it we press its neck and then the needle is pushed inside then we hold the place where we want to inject ourselves and then we inject the needle. The moment we rearise that the needle is inside the skin then we press the medicine until all is finished then we start pulling out the needle little by little.

INTERVIEWER: Thanks you so much this is well explained.

RESPONDENT: Thanks

INTERVIEWER: Did you feel the provider spent enough time training you?

REPONDENT: No we were trained twice

INTERVIEWER: they just trained you twice, but was it enough time?

RESPONDENT: Yes, it was enough time

INTERVIEWER: Maybe it was just, they just took some time

RESPONDENT: Yes

INTERVIEWER: Did you get the chance to practice what you were trained?

RESPONDNET: Yes

Title: KK_SCPI_4.docx

Descriptor Info:	District: Nkhotakota	Target Population: TP2 - DMPA-IM/DMPA-SC PI clients	Respondent type: DMPA-SC PI client	Aware of SI: Yes - had heard of SI before	Coding phase: 2. Double coded	Client marital status: Never married	Client age group: 20-29 years	Time since trained: N/A - client

Codes Applied:	C3.4 - Time to train clients

INTERVIEWER: okay. How long do they take to assist you?

RESPONDENT: The health personnel?

INTERVIEWER: mmmmh

RESPONDENT: They do not take time.

INTERVIEWER: okay. Alright.

RESPONDENT: They are fast enough.

Title: KK_SCPI_4.docx

Descriptor Info:	District: Nkhotakota	Target Population: TP2 - DMPA-IM/DMPA-SC PI clients	Respondent type: DMPA-SC PI client	Aware of SI: Yes - had heard of SI before	Coding phase: 2. Double coded	Client marital status: Never married	Client age group: 20-29 years	Time since trained: N/A - client

Codes Applied:	C3.4 - Time to train clients

NTERVIEWER: Now, I want you to explain to me what you have experienced, when learning the self-injecting method called Sayana. I want you to explain what you have experienced. In your view, did the doctor take enough time teaching you, how to use Sayana and also the pros and cons…

RESPONDENT: They do not take a long…very short time.

INTERVIEWER: Do you think this time is enough?

RESPONDENT: On that one, I would not know.

INTERVIEWER: No in your view, do you think this time was enough to you?

RESPONDENT: Maybe it was enough (giggles) for us we thought that may be that is as far as it goes. Because you would not really tell the doctor what to do.

INTERVIEWER: Now to you, I just want to know, was the time long or short?

RESPONDENT: as not enough

INTERVIEWER: Was short?

RESPONDENT: mmmmh.

INTERVIEWER: Why do you think it was short?

RESPONDENT: Depending on the lessons, it was a short lesson, when they take you to be taught it was ending there, it was not taking long.

INTERVIEWER: okay.

RESPONDENT: yes.

Title: KK_SCSI_3.doc

Descriptor Info:	District: Nkhotakota	Target Population: TP3 - DMPA-SC SI clients	Respondent type: DMPA-SC SI client	Aware of SI: Yes - had heard of SI before	Coding phase: 1. Single coded	Client marital status: Married	Client age group: 20-29 years	Time since trained: N/A - client

Codes Applied:	C3.4 - Time to train clients	C3.3.2 - Good (sufficient/ non-judgemental) counselling/training

NTERVIEWER: Okay, alright. Did you feel the provider spent enough time training you? Was the time spent training you enough?

RESPONDENT: yes.

INTERVIEWER: why was it enough?

RESPONDENT: we spent a lot of time at that place.

INTERVIEWER: you understood what they trained you?

RESPONDENT: I understood everything they trained me about.

Title: KK_SCSI_4.docx

Descriptor Info:	District: Nkhotakota	Target Population: TP3 - DMPA-SC SI clients	Respondent type: DMPA-SC SI client	Aware of SI: Yes - had heard of SI before	Coding phase: 2. Double coded	Client marital status: Married	Client age group: Under 20 (adolescent)	Time since trained: N/A - client

Codes Applied:	C3.4 - Time to train clients

INTERVIEWER: the HSA? Alright, did you feel the provider spent enough time training you?

RESPONDENT: No they didn’t take too much time

Title: MZs_IM_5.docx

Descriptor Info:	District: Mzimba South	Target Population: TP2 - DMPA-IM/DMPA-SC PI clients	Respondent type: DMPA-IM client	Aware of SI: Yes - had heard of SI before	Coding phase: 1. Single coded	Client marital status: Married	Client age group: 20-29 years	Time since trained: N/A - client

Codes Applied:	C5 - Reinjection	C3.4 - Time to train clients

Interviewer: did they spend enough time on counselling?

Respondent: no we were many of us here, so they spent few minutes on each one of us, so I went to our HSA in our community for proper counselling

Title: MZs_IM_5.docx

Descriptor Info:	District: Mzimba South	Target Population: TP2 - DMPA-IM/DMPA-SC PI clients	Respondent type: DMPA-IM client	Aware of SI: Yes - had heard of SI before	Coding phase: 1. Single coded	Client marital status: Married	Client age group: 20-29 years	Time since trained: N/A - client

Codes Applied:	C3.4 - Time to train clients

Interviewer: do you feel you received enough counselling from the provider?

Respondent: (giggles) no she was rushing she seemed to have other things to do

Title: ZA_SCSI_2.docx

Descriptor Info:	District: Zomba	Target Population: TP3 - DMPA-SC SI clients	Respondent type: DMPA-SC SI client	Aware of SI: Yes - had heard of SI before	Coding phase: 1. Single coded	Client marital status:	Client age group:	Time since trained: N/A - client

Codes Applied:	C3.4 - Time to train clients

nterviewer: Did you feel that the provider spent enough time training you?

Respondent: Yes, they took almost an hour

Interviewer: Okay.

Respondent: It elapsed while they talked about this method of family planning.

Interviewer: Alright.

Respondent: Yes,

Title: ZA_IM_4.docx

Descriptor Info:	District: Zomba	Target Population: TP2 - DMPA-IM/DMPA-SC PI clients	Respondent type: DMPA-IM client	Aware of SI: Yes - had heard of SI before	Coding phase: 1. Single coded	Client marital status: Married	Client age group: Under 20 (adolescent)	Time since trained: N/A - client

Codes Applied:	C3.4 - Time to train clients

Interviewer: Alright what about the nurses how long does it take for them to help you? (Baby calls the mother)

Respondent: Counselling?

Interviewer: (Baby calls the mother) When you come at the hospital how long does it take to get all the help from the nurses?

Respondent: It depends on how the people are when they are a lot of people it takes time and when they are few (Baby screams while calling the mother) it doesn’t take long.

Title: ZA_SCSI_5.docx

Descriptor Info:	District: Zomba	Target Population: TP3 - DMPA-SC SI clients	Respondent type: DMPA-SC SI client	Aware of SI: Yes - had heard of SI before	Coding phase: 3. Triple coded	Client marital status: Married	Client age group: 30-39 years	Time since trained: N/A - client

Codes Applied:	C3.4 - Time to train clients

Interviewer: Alright do you think the provider took enough time training you about self-injection?

Respondent: Ah they did not take a long time,

Interviewer: Mmm (showing that one is following the conversation)

Respondent: They just took… maybe 1 hour did not elapse

Interviewer: Alright.

Respondent: Yes, it’s been a long time ago

Title: MZs_SCSI_2.docx

Descriptor Info:	District: Mzimba South	Target Population: TP3 - DMPA-SC SI clients	Respondent type: DMPA-SC SI client	Aware of SI: Yes - had heard of SI before	Coding phase: 3. Triple coded	Client marital status: Married	Client age group: Under 20 (adolescent)	Time since trained: N/A - client

Codes Applied:	C5.1.3 - Resources/follow up materials (e.g. calendar) to aid timely re-injection	C3.4 - Time to train clients

Interviewer: Did you think that provider spent enough time training you?

Respondent: Yes, and after counselling they also gave us calendar

Interviewer: What was the calendar for?

Respondent: We should be recording the dates for us to know the next date of injection. They also told us that after the injection we should count 13 weeks, that means we will do the injection again on week 13 and we should note that date for easy to remember.

Title: KK_IM_7.docx

Descriptor Info:	District: Nkhotakota	Target Population: TP2 - DMPA-IM/DMPA-SC PI clients	Respondent type: DMPA-IM client	Aware of SI: Yes - had heard of SI before	Coding phase: 1. Single coded	Client marital status: Married	Client age group: 30-39 years	Time since trained: N/A - client

Codes Applied:	C3.4 - Time to train clients

NTERVIEWER: Can you describe your experience of being trained in self-injection? Did the provider spend enough time training you?

RESPONDENT: aaah [yes].

INTERVIEWER: Mmmm. They spend enough time training you?

RESPONDENT: aaah yes, they do.

INTERVIEWER: okay.

RESPONDENT: Sure.

INTERVIEWER: Good training?

RESPONDENT: Yes.

Title: ZA_IM_5.docx

Descriptor Info:	District: Zomba	Target Population: TP2 - DMPA-IM/DMPA-SC PI clients	Respondent type: DMPA-IM client	Aware of SI: Yes - had heard of SI before	Coding phase: 2. Double coded	Client marital status: Married	Client age group: 30-39 years	Time since trained: N/A - client

Codes Applied:	C3.4 - Time to train clients

Interviewer: Alright, can you tell me your experience while being trained on DMPA-SC? Ah maybe I should just say that, do you think the providers took enough time training you?

Respondent: No they did not take a long time, when the method came, they started telling us at the antenatal clinic about it

Title: MZs_SCSI_5.docx

Descriptor Info:	District: Mzimba South	Target Population: TP3 - DMPA-SC SI clients	Respondent type: DMPA-SC SI client	Aware of SI: Yes - had heard of SI before	Coding phase: 1. Single coded	Client marital status: Married	Client age group: 20-29 years	Time since trained: N/A - client

Codes Applied:	C3.4 - Time to train clients

Interviewer: Did the provider took enough time to teach you on self-injection

Respondent: Yes, and it was one to one. They asked if we could manage to self-inject it wasn’t by force since it was the only method that was available on that day. I gave consent. They taught us on the procedure and we self-injected

Title: KK_SCSI_1.docx

Descriptor Info:	District: Nkhotakota	Target Population: TP3 - DMPA-SC SI clients	Respondent type: DMPA-SC SI client	Aware of SI: Yes - had heard of SI before	Coding phase: 3. Triple coded	Client marital status: Never married	Client age group: Under 20 (adolescent)	Time since trained: N/A - client

Codes Applied:	C3.4 - Time to train clients

INTERVIEWER: Mmmhu. Did you feel that the nurse spent enough time training you?

RESPONDENT: Aaah, the time…the time was enough.

INTERVIEWER: okay.

RESPONDENT: Yes.

Title: ZA_IM_9.docx

Descriptor Info:	District: Zomba	Target Population: TP2 - DMPA-IM/DMPA-SC PI clients	Respondent type: DMPA-IM client	Aware of SI: Yes - had heard of SI before	Coding phase: 1. Single coded	Client marital status: Married	Client age group: 20-29 years	Time since trained: N/A - client

Codes Applied:	C3.4 - Time to train clients

Interviewer: Alright alright, thank you so much. What happens when you get here at the hospital? How long does it take for you to be assisted here at the hospital?

Respondent: We stay a while; we take time because there are a lot of people.

Interviewer: Mmh.

Respondent: To help each and every one we stay a lot of minutes.

Interviewer: So how many minutes do you stay here at the hospital for a person to get assisted?

Respondent: 1 hour.

Interviewer: It can take an hour?

Respondent: Mmmh.

Title: ZA_SCSI_4.docx

Descriptor Info:	District: Zomba	Target Population: TP3 - DMPA-SC SI clients	Respondent type: DMPA-SC SI client	Aware of SI: Yes - had heard of SI before	Coding phase: 2. Double coded	Client marital status: Married	Client age group: 30-39 years	Time since trained: N/A - client

Codes Applied:	C3.4 - Time to train clients

Interviewer: mmm (showing that one is following the conversation)

Respondent: Sure

Interviewer: Alright ah, did you feel the provider spent enough time training you about DMPA-SC?

Respondent: Ah I feel like they took enough time because it’s not like we were just entering the consultation room the way we use to when getting DMPA-IM.

Interviewer: mmm (showing that one is following the conversation)

Respondent: When we got into the consultation room, we spent more time with the provider.

Interviewer: mmm (showing that one is following the conversation)

Respondent: so the provider took the injection and shook it

Interviewer: mmm (showing that one is following the conversation)

Respondent: and shared with us how to use it.

Interviewer: mmm (showing that one is following the conversation)

Respondent: And position it, after doing that they would tell us to self-inject, yourself so that when going back home you should have been known how to self-inject. So I saw that for me they took enough time that everyone was trained and knew that when I go home, I will do this.

Title: ZA_SCPI_8.docx

Descriptor Info:	District: Zomba	Target Population: TP2 - DMPA-IM/DMPA-SC PI clients	Respondent type: DMPA-SC PI client	Aware of SI: Yes - had heard of SI before	Coding phase: 1. Single coded	Client marital status:	Client age group:	Time since trained: N/A - client

Codes Applied:	C3.4 - Time to train clients

Interviewer: alright, how many minutes do you spend with them while they are helping you?

Respondent: maybe up to 3 minutes.

Interviewer: mmmm (showing that one is following the conversation)

Title: ZA_SCPI_8.docx

Descriptor Info:	District: Zomba	Target Population: TP2 - DMPA-IM/DMPA-SC PI clients	Respondent type: DMPA-SC PI client	Aware of SI: Yes - had heard of SI before	Coding phase: 1. Single coded	Client marital status:	Client age group:	Time since trained: N/A - client

Codes Applied:	C3.4 - Time to train clients

Interviewer: Alright, do you think the providers took enough time to train you in DMPA-SC?

Respondent: no they did not take enough time

Interviewer: mmmm (showing that one is following the conversation)

Respondent: YES

Title: MZs_SCSI_4.docx

Descriptor Info:	District: Mzimba South	Target Population: TP3 - DMPA-SC SI clients	Respondent type: DMPA-SC SI client	Aware of SI: Yes - had heard of SI before	Coding phase: 2. Double coded	Client marital status: Married	Client age group: 20-29 years	Time since trained: N/A - client

Codes Applied:	C3.4 - Time to train clients

Interviewer: In your view, did they spend enough time explaining to you?

Respondent: No they did not spend more time

Title: MZs_SCPI_3.docx

Descriptor Info:	District: Mzimba South	Target Population: TP2 - DMPA-IM/DMPA-SC PI clients	Respondent type: DMPA-SC PI client	Aware of SI: Yes - had heard of SI before	Coding phase: 1. Single coded	Client marital status: Married	Client age group: 20-29 years	Time since trained: N/A - client

Codes Applied:	C3.4 - Time to train clients

Interviewer: Did you feel the provider spent enough time training you?

Respondent: Yes.

Title: ZA_SCSI_1.docx

Descriptor Info:	District: Zomba	Target Population: TP3 - DMPA-SC SI clients	Respondent type: DMPA-SC SI client	Aware of SI: Yes - had heard of SI before	Coding phase: 1. Single coded	Client marital status: Married	Client age group: 40-49 years	Time since trained: N/A - client

Codes Applied:	C3.4 - Time to train clients

Interviewer: In your thinking, did they take enough time teaching you?

Respondent: They didn’t take long rather it just depended on everyone’s interest.

Interviewer: As for you, how long did it take?

Respondent: As for me it didn’t take just a day then I was good to go.

Title: MZs_IM_4.docx

Descriptor Info:	District: Mzimba South	Target Population: TP2 - DMPA-IM/DMPA-SC PI clients	Respondent type: DMPA-IM client	Aware of SI: Yes - had heard of SI before	Coding phase: 2. Double coded	Client marital status: Married	Client age group: 30-39 years	Time since trained: N/A - client

Codes Applied:	C3.4 - Time to train clients

Interviewer: How much time does the provider spend with you?

Respondent: Its less than an hour.

Title: MZs_IM_4.docx

Descriptor Info:	District: Mzimba South	Target Population: TP2 - DMPA-IM/DMPA-SC PI clients	Respondent type: DMPA-IM client	Aware of SI: Yes - had heard of SI before	Coding phase: 2. Double coded	Client marital status: Married	Client age group: 30-39 years	Time since trained: N/A - client

Codes Applied:	C3.4 - Time to train clients

Interviewer: Did the provider spent enough time in training you?

Respondent: Yes, he did.

Title: KK_IM_2.docx

Descriptor Info:	District: Nkhotakota	Target Population: TP2 - DMPA-IM/DMPA-SC PI clients	Respondent type: DMPA-IM client	Aware of SI: Yes - had heard of SI before	Coding phase: 2. Double coded	Client marital status: Married	Client age group: 30-39 years	Time since trained: N/A - client

Codes Applied:	C3.4 - Time to train clients	C1.1.1 - Positive client attitudes to SI

INTERVIEWER: All right, now, in your own view, on your discussion with the doctor, do you think the time was enough for you to discuss Sayana?

RESPONDENT: No, the doctor did not take much time because Sayana was not available

INTERVIEWER: Was not available? But you were taught anyhow?

RESPONDENT: We were just told that these two methods are not really different.

INTERVIEWER: mmmhu. What do you think about the lessons that you received?

RESPONDENT: About Sayana?

INTERVIEWER: mmmhu. Yes, that.

RESPONDENT: aaah to me it was good.

INTERVIEWER: it was good?

RESPONDENT: mmmmh to us women who use contraceptives.

INTERVIEWER: okay.

RESPONDENT: yes
