# Supplementary material for: Health care provider and client experiences of counselling on depot medroxyprogesterone acetate subcutaneous (DMPA-SC) for self-injection in Malawi
Source: PLOS Glob Public Health. 2023 Nov 30;3(11):e0002057. doi: 10.1371/journal.pgph.0002057 (PMC10688740; doi:10.1371/journal.pgph.0002057)
Supplement: S1 Data — (ZIP) [file pgph.0002057.s001.zip › S2/P1.1.1 provider positive percept.doc]

Title: ZA_HSA_2.docx

Descriptor Info:	District: Zomba	Target Population: TP1 - Providers/HSAs	Respondent type: HSA	Aware of SI: Yes - had heard of SI before	Coding phase: 3. Triple coded	Client marital status: N/A - provider	Client age group: N/A - provider	Time since trained: Less than a year ago

Codes Applied:	C2.1.1 - Community availability of injectables/SI	C2.1.2 - Reduced trips to facility	P1.1.1 - Positive provider attitudes towards SI	P88 - Provider great quotes

Interviewer: ah do you think injectable contraceptives are more common than the other methods you mentioned?

Respondent: Okay, these methods are very easy to find

Interviewer: mmm (Showing that one is following the conversation)

Respondent: Because most of the HSAs in the communities have been trained in self-injection…

Interviewer: mmm (Showing that one is following the conversation)

Respondent: So, we serve most of the clients in the communities so that they don’t have to spend so much time coming to the facility.

Interviewer: mmm (Showing that one is following the conversation)

Respondent: Eh, we are able to serve so many clients in the communities different from these other methods like Norplant which require that there should be someone properly trained in providing those methods.

Title: ZA_HSA_2.docx

Descriptor Info:	District: Zomba	Target Population: TP1 - Providers/HSAs	Respondent type: HSA	Aware of SI: Yes - had heard of SI before	Coding phase: 3. Triple coded	Client marital status: N/A - provider	Client age group: N/A - provider	Time since trained: Less than a year ago

Codes Applied:	P1.1.1 - Positive provider attitudes towards SI	P3.1.1 - Reduced provider workload

Interviewer: What are your thoughts on the training that you received, any good things or bad things on this training?

Respondent: There were some good things.

Interviewer: mmm (Showing that one is following the conversation)

Respondent: Yes, there were some good things, that we will be relieved of some work even in the villages because starting from the time we started, people started coming in few numbers because, for example when we gave them the injection today, …

Interviewer: mmm (Showing that one is following the conversation)

Respondent: It means they will come again in 2022, a day like this one…

Title: ZA_HSA_2.docx

Descriptor Info:	District: Zomba	Target Population: TP1 - Providers/HSAs	Respondent type: HSA	Aware of SI: Yes - had heard of SI before	Coding phase: 3. Triple coded	Client marital status: N/A - provider	Client age group: N/A - provider	Time since trained: Less than a year ago

Codes Applied:	P1.1.1 - Positive provider attitudes towards SI	P3.1.1 - Reduced provider workload	P4.1.3 - Provider availability/ willingness to repeat SI training	P4.1.1 - Follow up materials for client to take home

Interviewer: Ah to women at this facility and also in this community. How did you feel when you first heard of the possibility of women self-injecting?

Respondent: I received the news well…

Interviewer: mmm (showing agreement)

Respondent: …because of this possibility I saw that the work that we have at the facility and in the communities would be reduced since the clients will be trained and will be given the responsibility that they can be able to serve themselves, they just need to come and collect the injection on the days that we have advised them to do so, since we also provide them with calendars, they inject themselves four times a year…

Interviewer: mmm (agreement)

Respondent: …when they have finished the four doses they are supposed to come, we also provide them with calendars which means that they are able to follow the schedule properly…

Interviewer: Alright

Respondent: Sure, if they were not able to follow through the instructions then they are allowed to come back so that they can seek more instruction from us on how we advised them at the beginning, then we are able to explain to them again thoroughly.

Interviewer: mmm (showing that one is following the conversation)

Respondent: Yes.

Interviewer: Alright

Respondent: …so there is reduced congestion here at the facility and also in the community where we work. The reduced congestion does not mean that people are no longer using family planning, they are using family planning, but DMPA-SC seems like a longer method of family planning since the clients are injecting themselves at home…

Title: ZA_HSA_2.docx

Descriptor Info:	District: Zomba	Target Population: TP1 - Providers/HSAs	Respondent type: HSA	Aware of SI: Yes - had heard of SI before	Coding phase: 3. Triple coded	Client marital status: N/A - provider	Client age group: N/A - provider	Time since trained: Less than a year ago

Codes Applied:	P1.1.1 - Positive provider attitudes towards SI

Respondent: (laughs) eh There was a problem…

Interviewer: eh

Respondent: But he understood because I explained to him, yeah, but for some men they are just very rigid…

Interviewer: mmm (showing that one is following the conversation)

Respondent: This is a good method that can help a lot of women.

Interviewer: mmmhh (showing that one is following the conversation)

Respondent: Yeah, because the problems of motherhood are mostly experienced by women different from us men…

Interviewer: yah yah (Agreeing)

Respondent: Because for us we just impregnate them and tell them to go…

Interviewer: mmm (showing that one is following the conversation)

Respondent: We just come back in the morning when the baby is already born (laughs)

Title: KK_HSA_1.docx

Descriptor Info:	District: Nkhotakota	Target Population: TP1 - Providers/HSAs	Respondent type: HSA	Aware of SI: Yes - had heard of SI before	Coding phase: 2. Double coded	Client marital status: N/A - provider	Client age group: N/A - provider	Time since trained: A year or more ago

Codes Applied:	P1.1.1 - Positive provider attitudes towards SI

Interviewer: So the first question, what do you do in providing family planning in your community? Role in providing family planning in your community?

Respondent: In terms of family planning, when we are going to the community, whether we are going to do a different type of work, but still we are required to explain information about family planning to women more importantly about Sayana press, because when we do not tell them this information, maybe women walk long distances to reach at this health centre. But if we are going to do any type of job that we do in the village, we tell them and we also take the Sayana with us so that these people should be helped.

Title: KK_HSA_1.docx

Descriptor Info:	District: Nkhotakota	Target Population: TP1 - Providers/HSAs	Respondent type: HSA	Aware of SI: Yes - had heard of SI before	Coding phase: 2. Double coded	Client marital status: N/A - provider	Client age group: N/A - provider	Time since trained: A year or more ago

Codes Applied:	P1.1.1 - Positive provider attitudes towards SI	P3.1.5 - Ease of training clients in SI	P2.1.2 - Sufficient original provider training	P4.3 - Follow up approach	C7 - Resupply

Interviewer: What did you…what did you think of the training?

Respondent: The training is good. And also this Sayana, because of the long distance that women have to travel, when you counsel them and when the women understand, it is very easy for women to administer the method at home. When they have run out of Sayana, they come to you before their actual visit date. Let’s say you have given them three, yee when they inject the second dose, before they inject the third dose, they come and explain that they are remaining with one. So we tell them to come and get other doses when they inject the third dose. Or else when we are in the community, and we have Sayana, we give them other doses.

Interviewer: mmmh.

Respondent: So yeah. It is a very good method for women. they like it in our communities.

Interviewer: okay

Respondent: mmmh

Title: KK_HSA_1.docx

Descriptor Info:	District: Nkhotakota	Target Population: TP1 - Providers/HSAs	Respondent type: HSA	Aware of SI: Yes - had heard of SI before	Coding phase: 2. Double coded	Client marital status: N/A - provider	Client age group: N/A - provider	Time since trained: A year or more ago

Codes Applied:	P1.1.1 - Positive provider attitudes towards SI	P88 - Provider great quotes	P2.1.1 - Refresher training/mentorship	C0.2.1 - Positive community/client perspectives on FP

Interviewer: So…the concerns that you had then right? When you first heard and now, have these concerns changed?

Respondent: Yes they have changed because then we thought that this was a burden. The very first days of this, you could counsel these women and the women could say no I can not manage, I can not manage. And we could just inject them. See? When Sayana is for a woman to self inject. So after they visited us and helped us in some other areas, they just polished us in counselling on how we can chat with the women for us to convince them to start self injection. And then we saw that there was a change. When we arrive, we were counselling them, and during counselling you had maybe two or three women who self inject themselves. And there you could ask who has ever self injected? These people were role models to their friends

(10:37).

Interviewer: mmmhu

Respondent: Yes. sure

Title: KK_HSA_1.docx

Descriptor Info:	District: Nkhotakota	Target Population: TP1 - Providers/HSAs	Respondent type: HSA	Aware of SI: Yes - had heard of SI before	Coding phase: 2. Double coded	Client marital status: N/A - provider	Client age group: N/A - provider	Time since trained: A year or more ago

Codes Applied:	P1.2.1 - Negative provider attitudes towards SI	C1.2.2 - Fear of SI (idea or reality)	P1.1.1 - Positive provider attitudes towards SI	P88 - Provider great quotes

Interviewer: So with colleagues, maybe your fellow HSA’s did you discuss when you first heard of Sayana. Did you discuss?

Respondent: yes.

Interviewer: What did you discuss?

Respondent: About the challenges. We were saying that it is not possible because it is a new thing. We were saying that it will not be possible because women are even afraid of injection…they are afraid of tetanus vaccine for pregnant women. They run. And they should say a family planning method people should be self injecting, it can not be possible. So it was like an argument. But when we went for training and started providing, we saw that it was what, it was working.

Title: KK_HSA_1.docx

Descriptor Info:	District: Nkhotakota	Target Population: TP1 - Providers/HSAs	Respondent type: HSA	Aware of SI: Yes - had heard of SI before	Coding phase: 2. Double coded	Client marital status: N/A - provider	Client age group: N/A - provider	Time since trained: A year or more ago

Codes Applied:	P3.1.1 - Reduced provider workload	P1.1.1 - Positive provider attitudes towards SI

Interviewer: Mmmhu. So you as a provider right, how easy or difficult is it to train a woman to self-inject. To teach her so that she should be self-injecting. How difficult or easy is it?

Respondent: It is not difficult and it is one way for you as a provider to have less work.

Interviewer: mmmhu.

Respondent: Mmmhu. Because let us say it is her first time…the first time you have injected her and the second one you also have to inject her, the third, fourth. Each and every time you should be injecting her. But when you train her and you observe when she is first injecting herself, it means she has reduced your work.

Interviewer: okay

Respondent: mmmh

Title: ZA_HSA_5.docx

Descriptor Info:	District: Zomba	Target Population: TP1 - Providers/HSAs	Respondent type: HSA	Aware of SI: Yes - had heard of SI before	Coding phase: 2. Double coded	Client marital status: N/A - provider	Client age group: N/A - provider	Time since trained: Less than a year ago

Codes Applied:	P1.1.1 - Positive provider attitudes towards SI	C1.1.1 - Positive client attitudes to SI

Respondent: But for people, because I was in Nkhotakota

Interviewer: Mmm (showing that one is following the conversation)

Respondent: When I was in Nkhotakota and I then came here I don’t know whether they had been trained, but some have been doing the trainings, yah.

Interviewer: Mmm (showing that one is following the conversation)

Respondent: They did not know anything, so I started training them

TIME INTO THE INTERVIEW: 10Mins 1Sec

Interviewer: Mmm (showing that one is following the conversation)

Respondent: Now they have accepted it, now most women are accessing this method, there was nothing difficult in the training

Title: ZA_HSA_5.docx

Descriptor Info:	District: Zomba	Target Population: TP1 - Providers/HSAs	Respondent type: HSA	Aware of SI: Yes - had heard of SI before	Coding phase: 2. Double coded	Client marital status: N/A - provider	Client age group: N/A - provider	Time since trained: Less than a year ago

Codes Applied:	P1.1.1 - Positive provider attitudes towards SI

Interviewer: Am interested to know your experiences in providing DMPA-SC in your community, when you or your friends first heard about the possibility of self-injection, how did you feel?

Respondent: When we heard that women can self-inject?

Interviewer: Yes

Respondent: I felt good

Interviewer: Alright.

Respondent: Because it reduces our workload

Interviewer: Mmm (showing that one is following the conversation)

Respondent: Like I have said that we had 160 clients.

Interviewer: Mmm (showing that one is following the conversation)

Respondent: We also have to serve 100 children

Interviewer: Mmm (showing that one is following the conversation)

Respondent: And also 100 family planning clients

Interviewer: Mmm (showing that one is following the conversation)

Respondent: And we were the two of us

Interviewer: Mmm (showing that one is following the conversation)

Respondent: So, when there are more women who self-inject

Interviewer: Eh

Respondent: Then they will just come and collect, and they will not show up again for an entire year. For them to come back, it means the gap they create we can be serving other clients.

Interviewer: Mmm (showing that one is following the conversation)

Respondent: Sure

Title: ZA_HSA_5.docx

Descriptor Info:	District: Zomba	Target Population: TP1 - Providers/HSAs	Respondent type: HSA	Aware of SI: Yes - had heard of SI before	Coding phase: 2. Double coded	Client marital status: N/A - provider	Client age group: N/A - provider	Time since trained: Less than a year ago

Codes Applied:	P1.1.1 - Positive provider attitudes towards SI

Interviewer: Alright, on what you have told me, did you discuss this with your friends or colleagues … on how you felt when you heard that women could self-inject?

Respondent: Yes, I discussed with my friends because when I got here, I was the one who started telling them and advising them that there is this new method and women don’t have to struggle to get to the facility , you will just be doing it at home.

Interviewer: Mmm (showing that one is following the conversation)

Respondent: Some were denying it, and now they are saying to me that they think the method is good since they get it and stay at home for a long time

Interviewer: Mmm (showing that one is following the conversation)

Respondent: Yes so, I also talked with my colleagues that we should give out this method up until that time we run out of calendars.

Interviewer: Mmm (showing that one is following the conversation)

Respondent: There were no calendars available

Interviewer: Yah

Respondent: So, it was hard for me to help the clients. Eh sometimes I would just write on the packs the dates that they are supposed to inject themselves. Because of no availability of calendars, I have now seen that the calendars are available, and they called me that the calendars are now here that’s when I came collect and distribute to the women.

Interviewer: Alright. Did you have any concerns when you heard that women could self-inject?

Respondent: Ah I did not have any concerns

Title: KK_HSA_5.docx

Descriptor Info:	District: Nkhotakota	Target Population: TP1 - Providers/HSAs	Respondent type: HSA	Aware of SI: Yes - had heard of SI before	Coding phase: 1. Single coded	Client marital status: N/A - provider	Client age group: N/A - provider	Time since trained: A year or more ago

Codes Applied:	P2.2 - Barriers, general/other	P1.1.1 - Positive provider attitudes towards SI	P2.1.2 - Sufficient original provider training

INTERVIEWER: Overall, what did you think of the training?

RESPONDENT: I feel the training is so good, helpful, perfect but this group called CBDA’s

INTERVIEWER: Yes

RESPONDENT: The ones who does distribution of condoms, if possible, they should also be trained in this method, as we may be able to reach more clients. Because it seems a lot of clients are now pushing to using this method.

INTERVIEWER: Alright

RESPONDENT: So we have a lot of clients interested in this method

INTERVIEWER: Alright

RESPONDENT: As such I find myself to busy attending to a lot of clients

INTERVIEWER: Okay

RESPONDENT: Sure sure

INTERVIEWER: Alright alright

RESPONDENT: It’s because of the opportunity to have some dose to take home

Title: KK_HSA_5.docx

Descriptor Info:	District: Nkhotakota	Target Population: TP1 - Providers/HSAs	Respondent type: HSA	Aware of SI: Yes - had heard of SI before	Coding phase: 1. Single coded	Client marital status: N/A - provider	Client age group: N/A - provider	Time since trained: A year or more ago

Codes Applied:	P1.2.1 - Negative provider attitudes towards SI	P1.1.1 - Positive provider attitudes towards SI	C1.2.2 - Fear of SI (idea or reality)	C1.1.3 - Awareness of DMPA-SC SI

INTERVIEWER: …. when you and your colleagues first heard that DMPA-SC, could be injected by women themselves, what was your reaction? And has the opinion of you/your colleagues on self-injection changed over time?

RESPONDENT: When we first heard we were so worried

INTERVIEWER: Mhhh

RESPONDENT: That not many women will manage to do that, considering the type of injection ( Depo) that we have administering to our clients. So we though would women be able to inject themselves? So after the training and seeing the size of the injection we noted that its possible. We can see that ore clients are interested in this method and that for those who have started using it they are able to self-inject so well on their own.

INTERVIEWER: Ok

RESPONDENT: For those who were so scared and afraid are the ones who are encouraging their friends to use this method. That’s the position as of now about DMPA-SC

Title: KK_HSA_5.docx

Descriptor Info:	District: Nkhotakota	Target Population: TP1 - Providers/HSAs	Respondent type: HSA	Aware of SI: Yes - had heard of SI before	Coding phase: 1. Single coded	Client marital status: N/A - provider	Client age group: N/A - provider	Time since trained: A year or more ago

Codes Applied:	P1.1.1 - Positive provider attitudes towards SI	C1.1.3 - Awareness of DMPA-SC SI

INTERVIEWER: Have you and your colleagues discussed this?

RESPONDENT: WE discuss this with my colleagues

INTERVIEWER: Are you supportive of the idea of women administering their own contraceptives?

RESPONDENT: Yes, we are very much supportive, since most of the

INTERVIEWER: Yes

RESPONDENT: Who are afraid to inject themselves, we do ask some clients to come and show their friends how self-inject is done. This encourages the other clients seeing their fellow woman being able to do that and they also try to do self-inject. It is through this that more women have been strengthened to take self-injection.

INTERVIEWER: Alright

RESPONDENT: Sure sure

Title: KK_HSA_5.docx

Descriptor Info:	District: Nkhotakota	Target Population: TP1 - Providers/HSAs	Respondent type: HSA	Aware of SI: Yes - had heard of SI before	Coding phase: 1. Single coded	Client marital status: N/A - provider	Client age group: N/A - provider	Time since trained: A year or more ago

Codes Applied:	C1.1.1 - Positive client attitudes to SI	C2.1.2 - Reduced trips to facility	C1.1.3 - Awareness of DMPA-SC SI	P1.1.1 - Positive provider attitudes towards SI

INTERVIEWER: What specifically has influenced your opinion changing or not changing regarding DMPA-SC?

RESPONDENT: Yes, there is something that has influenced my opinion.

INTERVIEWER: Ok

RESPONDENT: The thing that has changed my opinion is the way women have welcome method. A thing we thought would not turn this way.

INTERVIEWER: Ok.

RESPONDENT: We have noticed that it is very possible. Most of the people in this community are farmers

INTERVIEWER: Okay

RESPONDENT: There has been fights in most of the homes during rainy season as their wives have been spending much of their time at the hospital. However, upon the coming of DMPA-SC the few women who started to take this method had communicated to their friends about this it, that it doesn’t require one to be visiting a facility now and then, that is why they have been home most of the times. This has prompted some more clients to come and try this method. In no time we just noticed that more women are coming for this method as it gives them ample time to do other things at home.

Title: KK_HSA_5.docx

Descriptor Info:	District: Nkhotakota	Target Population: TP1 - Providers/HSAs	Respondent type: HSA	Aware of SI: Yes - had heard of SI before	Coding phase: 1. Single coded	Client marital status: N/A - provider	Client age group: N/A - provider	Time since trained: A year or more ago

Codes Applied:	P1.1.1 - Positive provider attitudes towards SI	C1.1.1 - Positive client attitudes to SI	P3.2.2 - Stockouts/stocks near expiry dates	P2.2 - Barriers, general/other

INTERVIEWER: Do you have any other thoughts or information if at all you may have forgotten something during our discussions.

RESPONDENT: Much have already been said

INTERVIEWER: Alright

RESPONDENT: In my community most women they like to use this method over the other methods.

INTERVIEWER: Ok

RESPONDENT: They are scrambling for it compared in the, we just request that those who are supplying this method should continue with regular and enough supplies.

INTERVIEWER: Alright

RESPONDENT: We have some areas where new HSA’s have just been deployed, that means the tie DMPA-SC was being introduced these areas were missed as a result the method has not been fully introduced yet a lot of clients are interested to take this method.

INTERVIEWER: Alright

RESPONDENT: That’s it, sure sure

Title: MZs_HSA_1.docx

Descriptor Info:	District: Mzimba South	Target Population: TP1 - Providers/HSAs	Respondent type: HSA	Aware of SI: Yes - had heard of SI before	Coding phase: 2. Double coded	Client marital status: N/A - provider	Client age group: N/A - provider	Time since trained:

Codes Applied:	P1.1.1 - Positive provider attitudes towards SI	C2.1.2 - Reduced trips to facility	P4.3 - Follow up approach

Interviewer: So tell me about it. The good as well as the bad ones that someone can come across upon using Sayana self-injection and also how can a women self-inject herself.

Respondent: A women who uses Sayana self-injection will only be visiting the facility once a year and this method is very convenient because once a woman chooses to use this method the chances of getting pregnant are very low.

Interviewer: Mmmmh. (following)

Respondent: Yes. And this Sayana self-injection will give a chance to women to do other things since they will only be visiting the facility once a year. We also tell them that with this method women can experience lighter or heavy periods or sometimes no periods at all. But if women can experience overflowing of periods, then she should visit the facility immediately for help.

Title: MZs_HSA_1.docx

Descriptor Info:	District: Mzimba South	Target Population: TP1 - Providers/HSAs	Respondent type: HSA	Aware of SI: Yes - had heard of SI before	Coding phase: 2. Double coded	Client marital status: N/A - provider	Client age group: N/A - provider	Time since trained:

Codes Applied:	P1.1.1 - Positive provider attitudes towards SI	P3.1.5 - Ease of training clients in SI

Interviewer: Has opinion you/colleagues on self-injection changed over time?

Respondent: Yes, it has changed through the training we received and also by seeing the needle itself, we have seen that women do like it and they are saying that this one is simple than DEPO.

Interviewer: Did you/do you have any concerns about this idea?

Respondent: There is no concerns.

Interviewer: Why don’t you have any concern?

Respondent: There is no concerns because women do use it very well.

Interviewer: Alright.

Respondent: Mmmmm yes.

Interviewer: What specifically has influenced your opinion to change?

Respondent: Since the training we received and upon the provision of Sayana to women, we have seen that its simple to self-inject and the needle is just okay.

Title: MZs_HSA_1.docx

Descriptor Info:	District: Mzimba South	Target Population: TP1 - Providers/HSAs	Respondent type: HSA	Aware of SI: Yes - had heard of SI before	Coding phase: 2. Double coded	Client marital status: N/A - provider	Client age group: N/A - provider	Time since trained:

Codes Applied:	P1.1.1 - Positive provider attitudes towards SI	C2.1.2 - Reduced trips to facility	C1.1.1 - Positive client attitudes to SI	C3.1 - Enablers, general/other

Q17. Interviewer: In your view, what are the main factors that would support a client to take up the self-injection option with DMPA-SC?

Respondent: The main factor is that it saves time to a client by not visiting the facility regularly and spending her time on other things

Interviewer: Mmmmh (following)

Respondent: Yes

Interviewer: Do you have anything to add?

Respondent: The Sayana needle is very small thus why women do accept to self-inject.

Title: KK_FAC_1.docx

Descriptor Info:	District: Nkhotakota	Target Population: TP1 - Providers/HSAs	Respondent type: Facility-based provider	Aware of SI: Yes - had heard of SI before	Coding phase: 3. Triple coded	Client marital status: N/A - provider	Client age group: N/A - provider	Time since trained: A year or more ago

Codes Applied:	P3.1.1 - Reduced provider workload	P1.1.1 - Positive provider attitudes towards SI

RESPONDENT: The good parts are many

INTERVIEWER: Okay

RESPONDENT: The reason is when you provide Sayana you are guaranteed to meet the client after some time.

INTERVIEWER: yes

RESPONDENT: Unlike Depo, the provider has to meet the client every three months to administer it to her. Whilst with Sayana we give the client doses you will see her again after a year. Thereby reducing work load.

Title: KK_FAC_1.docx

Descriptor Info:	District: Nkhotakota	Target Population: TP1 - Providers/HSAs	Respondent type: Facility-based provider	Aware of SI: Yes - had heard of SI before	Coding phase: 3. Triple coded	Client marital status: N/A - provider	Client age group: N/A - provider	Time since trained: A year or more ago

Codes Applied:	P1.2.1 - Negative provider attitudes towards SI	P1.1.1 - Positive provider attitudes towards SI

INTERVIEWER: Alright, alright, I am still interested to know more, thought you seem to know more and you have shared that, But I am still interested to know your experience of providing DMPA-SC at the facility. I would like to know when you/ your colleagues first heard that DMPA-SC, could be injected by women themselves, what was your reaction? And has the opinion of you /your colleges on self-injection changed over time? How was your reaction when you first heard and now after time has passed has your opinion changed?

RESPONDENT: Of course, when I first heard about it, I was doubting as to whether Malawian women can manage to inject themselves. This thought came up because I was considering how the women reacts when one is injecting them, they are always afraid. So I was thinking, is this doer able that one can inject herself . But with passing of time, we see clients coming to request for the method.

INTERVIEWER: okay

RESPONDENT: May I have Sayana.

Title: KK_FAC_1.docx

Descriptor Info:	District: Nkhotakota	Target Population: TP1 - Providers/HSAs	Respondent type: Facility-based provider	Aware of SI: Yes - had heard of SI before	Coding phase: 3. Triple coded	Client marital status: N/A - provider	Client age group: N/A - provider	Time since trained: A year or more ago

Codes Applied:	P1.1.1 - Positive provider attitudes towards SI

INTERVIEWER: So how do you manage that?

RESPONDENT: When training the clients, we give them an example of patients with sugar disease. That these patients they do inject themselves every day morning and evening. So we counsel them that if someone is able to inject herself every day, how would you fail to inject yourself only once in three months. Hence most of the clients understands. We also explain to them that the injection used for this method is very small that cannot hurt them

INTERVIEWER: Yes

RESPONDENT: It very small hence it cannot cause much pain when used on someone’s body

Title: MZs_HSA_5.docx

Descriptor Info:	District: Mzimba South	Target Population: TP1 - Providers/HSAs	Respondent type: HSA	Aware of SI: Yes - had heard of SI before	Coding phase: 1. Single coded	Client marital status: N/A - provider	Client age group: N/A - provider	Time since trained: A year or more ago

Codes Applied:	P1.1.1 - Positive provider attitudes towards SI	P3.1.1 - Reduced provider workload

Interviewer: now I would like to know your experience as a provider for Sayana here at the facility and at your catchment area. When you first heard about Sayana that a woman will self-inject, what was your reaction with your colleagues?

Respondent: for me I saw it as a good development because it would reduce workload since we are also involved in IMCI. So for those who gets Sayana we do not take time we just remind them and provide them for self-injection

Interviewer: what did you discuss with your workmates upon hearing about self-injection?

TIME STAMP 13:13 MIN- 20:54 MIN

Respondent: with my fellow HSA’s?

Interviewer: yes, and other workmates?

Respondent: we had discussed that it would reduce workload. So we were asking questions to nurses because we were working together

Title: MZs_HSA_5.docx

Descriptor Info:	District: Mzimba South	Target Population: TP1 - Providers/HSAs	Respondent type: HSA	Aware of SI: Yes - had heard of SI before	Coding phase: 1. Single coded	Client marital status: N/A - provider	Client age group: N/A - provider	Time since trained: A year or more ago

Codes Applied:	P1.1.1 - Positive provider attitudes towards SI	P1.2.1 - Negative provider attitudes towards SI	C1.2.2 - Fear of SI (idea or reality)

Interviewer: did you have any concerns or questions about a woman self-injecting?

Respondent: we were worried if women would manage to self-inject because most of them have fears when we inject them. So at the training we saw a woman self-inject on the abdomen we had fears that it would go deep. But after the training we saw that it was all well

Interviewer: after providing Sayana for self-injection have your perception changed? Do you still have fears?

Respondent: the fears are now gone because we waited for the feedback from women, but it was all well.

Title: MZs_HSA_5.docx

Descriptor Info:	District: Mzimba South	Target Population: TP1 - Providers/HSAs	Respondent type: HSA	Aware of SI: Yes - had heard of SI before	Coding phase: 1. Single coded	Client marital status: N/A - provider	Client age group: N/A - provider	Time since trained: A year or more ago

Codes Applied:	P1.1.1 - Positive provider attitudes towards SI	C1.1.1 - Positive client attitudes to SI

Interviewer: what is your experience in providing Sayana?

Respondent: from the time I started administering, a lot of people like Sayana. Many have changed from other methods of family planning. They like it because the injection is smaller like the one for BCG compared to Depo IM syringe.

Title: MZs_HSA_5.docx

Descriptor Info:	District: Mzimba South	Target Population: TP1 - Providers/HSAs	Respondent type: HSA	Aware of SI: Yes - had heard of SI before	Coding phase: 1. Single coded	Client marital status: N/A - provider	Client age group: N/A - provider	Time since trained: A year or more ago

Codes Applied:	P1.1.1 - Positive provider attitudes towards SI	C2.1.2 - Reduced trips to facility	C1.1.1 - Positive client attitudes to SI

Interviewer: we are now going to talk about what makes other women choose Sayana compared to others. In your view, what makes other women choose Sayana compared to others?

Respondent: like I have already said it is easy to use you do not visit the facility regularly. It is easy to self-inject.

Title: KK_HSA_3.docx

Descriptor Info:	District: Nkhotakota	Target Population: TP1 - Providers/HSAs	Respondent type: HSA	Aware of SI: Yes - had heard of SI before	Coding phase: 3. Triple coded	Client marital status: N/A - provider	Client age group: N/A - provider	Time since trained: A year or more ago

Codes Applied:	C1.1.1 - Positive client attitudes to SI	P1.1.1 - Positive provider attitudes towards SI	C5.1.4 - Timely injection due to not having to rely on external factors

INTERVIEWER: Firstly, I would like to ask…to ask some questions to understand more about your role in providing family planning services in your community.

RESPONDENT: Mmmmh. Yes, I do provide family planning services in my community. First, we begin with teaching then family planning methods for example self-injectable. Aah a lot of them like the method and it is a success. This method is good because sometimes when the women are on this method, we help then on the next dates for them to self-inject. When we teach them, those who have managed to self-inject or those who have agreed to this method, we also see if they will really be able to self-inject if they take the drugs home. A lot of people like this method because sometimes when they need our services, they don’t find us. But when we give them, they help themselves when it’s time to self-inject.

Title: KK_HSA_3.docx

Descriptor Info:	District: Nkhotakota	Target Population: TP1 - Providers/HSAs	Respondent type: HSA	Aware of SI: Yes - had heard of SI before	Coding phase: 3. Triple coded	Client marital status: N/A - provider	Client age group: N/A - provider	Time since trained: A year or more ago

Codes Applied:	P1.1.1 - Positive provider attitudes towards SI	C1.1.1 - Positive client attitudes to SI

INTERVIEWER: So on the training, what were the good parts of the training?

RESPONDENT: this training was good because the method of self-injection, they…the women they are helped because when we provide the method to them, they help themselves without any problem because…the method is easily available. But for them to come to the hospital maybe some travel long distances and to come to the hospital the nurse to help them get that method might be busy with other patients and clients. But when HSAs are in the community, it’s a good method. They have been praising the method because they are getting help easily.

Title: KK_HSA_3.docx

Descriptor Info:	District: Nkhotakota	Target Population: TP1 - Providers/HSAs	Respondent type: HSA	Aware of SI: Yes - had heard of SI before	Coding phase: 3. Triple coded	Client marital status: N/A - provider	Client age group: N/A - provider	Time since trained: A year or more ago

Codes Applied:	P1.2.1 - Negative provider attitudes towards SI	P1.1.1 - Positive provider attitudes towards SI

INTERVIEWER: Alright. We are still discussing. So here I am interested in your experiences of providing DMPA-SC in your community. So when you have first heard there is another injectable who…it’s still Depo right? But a woman can what, self-inject after training. What was your reaction?

RESPONDENT: As an…as an HSA, I was worried because we thought that it was an injection to sterilize women or that when a woman is injected, she will not get pregnant. I was worried and also people in the community were worried. It reached a point where people understood that it’s a good injectable although some complain of backache, legs ache, stomach ache, menstruation stops maybe for 2 months or 3 months. But those doubts are disappearing.

Title: KK_HSA_3.docx

Descriptor Info:	District: Nkhotakota	Target Population: TP1 - Providers/HSAs	Respondent type: HSA	Aware of SI: Yes - had heard of SI before	Coding phase: 3. Triple coded	Client marital status: N/A - provider	Client age group: N/A - provider	Time since trained: A year or more ago

Codes Applied:	P1.2.1 - Negative provider attitudes towards SI	P1.1.1 - Positive provider attitudes towards SI

INTERVIEWER: How about with your colleagues right, maybe with HSA’s or any colleagues, when you heard that DMPA-SC could be self-injected by a woman or an adolescent, a girl right. What did you discuss?

RESPONDENT: mmmh

INTERVIEWER: What were you discussing?

RESPONDENT: We were discussing that DMPA-SC is a very good injectable although some at first thought that it is a very dangerous and can destroy their life. But with time, they realized that it’s a very good injectable…It is a good method.

Title: KK_HSA_3.docx

Descriptor Info:	District: Nkhotakota	Target Population: TP1 - Providers/HSAs	Respondent type: HSA	Aware of SI: Yes - had heard of SI before	Coding phase: 3. Triple coded	Client marital status: N/A - provider	Client age group: N/A - provider	Time since trained: A year or more ago

Codes Applied:	P1.1.1 - Positive provider attitudes towards SI

INTERVIEWER: Are you now in support of women administering their own contraception? Are you in support?

RESPONDENT: eeeh. Yes.

INTERVIEWER: motani? How?

RESPONDENT: We encourage them according to the importance of this method. We encourage them because when they are on this method they have time as they don’t have to walk long distances to a health facility to get an injectable because we have these methods in the community and we assist them right there and they have enough time to prepare.

Title: KK_HSA_3.docx

Descriptor Info:	District: Nkhotakota	Target Population: TP1 - Providers/HSAs	Respondent type: HSA	Aware of SI: Yes - had heard of SI before	Coding phase: 3. Triple coded	Client marital status: N/A - provider	Client age group: N/A - provider	Time since trained: A year or more ago

Codes Applied:	P1.2.1 - Negative provider attitudes towards SI	P1.1.1 - Positive provider attitudes towards SI	C2.1.2 - Reduced trips to facility	C2.1.1 - Community availability of injectables/SI

INTERVIEWER: In its first stages before it was implemented, when you had just heard. Were you in support that Sayana should be introduced?

RESPONDENT: it was difficult for us to accept it because we did not know its end results. But after it was introduced, we saw that it was a good thing and then we accepted it.

INTERVIEWER: mmmhu. So what made you change your mind? What made you to accept it?

RESPONDENT: What made me change my mind was that I realized that Sayana is a good method because with other methods, women were walking long distances to access contraceptives. But now a lot of women see the importance of this method because they access it right there in their community, in the field.

Title: KK_HSA_3.docx

Descriptor Info:	District: Nkhotakota	Target Population: TP1 - Providers/HSAs	Respondent type: HSA	Aware of SI: Yes - had heard of SI before	Coding phase: 3. Triple coded	Client marital status: N/A - provider	Client age group: N/A - provider	Time since trained: A year or more ago

Codes Applied:	C5.1.4 - Timely injection due to not having to rely on external factors	P1.1.1 - Positive provider attitudes towards SI	C7 - Resupply

INTERVIEWER: Alright. We are now going towards the end, and I would like to know your perspectives on…on why you think clients may or may not choose the self-injection option. What are the factors that would support a client to take-up DMPA-SC?

RESPONDENT: The factors that can encourage women to take-up self-injection are that with Sayana, they have it at home. It’s different from Depo because Depo is with an HSA while with Sayana, a client who has accepted and has passed the training of self-injection has the drug at home. And the other factor for taking up self-injection is that it is readily available to them. The method is easily accessible/to get. Another good factor is that one.

INTERVIEWER: chifukwa china. Another factor.

RESPONDENT: Another factor…but that is a strong factor that it is easily accessible. They have the contraceptive at home.

Title: ZA_HSA_4.docx

Descriptor Info:	District: Zomba	Target Population: TP1 - Providers/HSAs	Respondent type: HSA	Aware of SI: Yes - had heard of SI before	Coding phase: 1. Single coded	Client marital status: N/A - provider	Client age group: N/A - provider	Time since trained: A year or more ago

Codes Applied:	P1.1.1 - Positive provider attitudes towards SI	P3.1.1 - Reduced provider workload	C2.1.2 - Reduced trips to facility

Interviewer: Aah in your community when you first heard about the self-injection how did you and your colleagues feel?

Respondent: I felt very good because it minimized our workload and also time spent by women coming here to get the injection. They have time to do other things because they will just inject themselves doing other things whilst coming here is time consuming.

Interviewer: Has your opinion or your colleagues on self-injection changed over time?

Respondent: What kind of change?

Interviewer: You said that you accepted this method and it’s good, did your thoughts change since administering this injection?

Respondent: No, they did not change

Title: ZA_HSA_4.docx

Descriptor Info:	District: Zomba	Target Population: TP1 - Providers/HSAs	Respondent type: HSA	Aware of SI: Yes - had heard of SI before	Coding phase: 1. Single coded	Client marital status: N/A - provider	Client age group: N/A - provider	Time since trained: A year or more ago

Codes Applied:	P1.1.1 - Positive provider attitudes towards SI	P3.2.2 - Stockouts/stocks near expiry dates

Interviewer: Mmh! Alright alright were you or are you still having concerns about the self-injection.

Respondent: Aaah I don’t have any concern it’s just that when we don’t have the injection it’s when they complain

Interviewer: Alright

Respondent: Mmmh

Interviewer: Is there anything that have made you to change your perception about the self-injection method?

Respondent: Aaah! There’s nothing that has changed my perception

Interviewer: Alright alright! What made you not to change your perception?

Respondent: When these women are receiving the injection, they appreciate it saying its good, some saying it is challenging. The thing that made me not to change my perception it’s because even Depo women were saying the same some saying it is good some saying its bad, so I just saw that it’s the same.

Interviewer: Alright thank you! What has the uptake of self-injection been like in this community the past three months?

Respondent: Aaah in the last 2 months it expired so if there is time, we had it is this month

Interviewer: Mmmh

Respondent: Yes

Interviewer: Alright alright! So, for how long have you not administered the injection in the community?

Respondent: Like for me it has been 2 months

Interviewer: 2 months?

Respondent: Yes

Title: ZA_HSA_4.docx

Descriptor Info:	District: Zomba	Target Population: TP1 - Providers/HSAs	Respondent type: HSA	Aware of SI: Yes - had heard of SI before	Coding phase: 1. Single coded	Client marital status: N/A - provider	Client age group: N/A - provider	Time since trained: A year or more ago

Codes Applied:	C2.1.2 - Reduced trips to facility	P1.1.1 - Positive provider attitudes towards SI	C4 - Storage of unused units

Interviewer: What do you think is the reason why women may go for the self-injecting contraception method?

Respondent: They choose the method to save time, the way this vaccine is injected is simple as compared to Depo, the storage is easy.

Title: KK_HSA_4.docx

Descriptor Info:	District: Nkhotakota	Target Population: TP1 - Providers/HSAs	Respondent type: HSA	Aware of SI: Yes - had heard of SI before	Coding phase: 1. Single coded	Client marital status: N/A - provider	Client age group: N/A - provider	Time since trained: A year or more ago

Codes Applied:	P1.1.1 - Positive provider attitudes towards SI	P3.1.1 - Reduced provider workload	C2.1.2 - Reduced trips to facility	P2.1.2 - Sufficient original provider training

INTERVIEWER: Alright thank you so much. To continue with our conversation, I would like to ask you some questions about your experience with being trained in DMPA-SC. The first question on this topic is; may you please tell me a bit about the training you received in providing DMPA-SC? Overall what did you think of the training?

RESPONDENT: From the time we received the training in DMPA-SC for self-injection

INTERVIEWER: Yes

RESPONDENT: In would say that after the training I felt it was so good that I did undergone such training because as an HSA, it meant that I have added knowledge in another type family planning method. Secondly, I also considered the distance that most clients travel to come and meet me just to take contraceptives. Furthermore, the fact that the method do require clients to take some more units home to use afterwards, I felt this will do me good since my work load will reduce because I won’t be meeting clients regularly as before.

Title: KK_HSA_4.docx

Descriptor Info:	District: Nkhotakota	Target Population: TP1 - Providers/HSAs	Respondent type: HSA	Aware of SI: Yes - had heard of SI before	Coding phase: 1. Single coded	Client marital status: N/A - provider	Client age group: N/A - provider	Time since trained: A year or more ago

Codes Applied:	P1.1.1 - Positive provider attitudes towards SI

RESPONDENT: I was also considering how we store the medicine at a facility. However, after the training and was back in my community, the only problem that in noted was on clients getting used to self-injection of which now it’s no longer an issue.

INTERVIEWER: Alright

RESPONDENT: I would say it two weeks or so for me to sees that clients are getting used to the fact that self-injection is indeed possible.

INTERVIEWER: Alright.

RESPONDENT: As of now, looking at how interested more people are in this method we cannot that self-injection is no longer a problem like before.

RESPONDENT: Yes

Title: KK_HSA_4.docx

Descriptor Info:	District: Nkhotakota	Target Population: TP1 - Providers/HSAs	Respondent type: HSA	Aware of SI: Yes - had heard of SI before	Coding phase: 1. Single coded	Client marital status: N/A - provider	Client age group: N/A - provider	Time since trained: A year or more ago

Codes Applied:	P1.1.1 - Positive provider attitudes towards SI

INTERVIEWER: Are you supportive of the idea of women administering their own contraceptives?

RESPONDENT: Yes, we are more supportive to both, those who are already using the method so that they continue and not drop on the way. In addition, we still have a lot of women who are not yet using this method; hence we are also working tersely convincing more women to start using this method.

INTERVIEWER: Alright

RESPONDENT: Sure

Title: KK_HSA_4.docx

Descriptor Info:	District: Nkhotakota	Target Population: TP1 - Providers/HSAs	Respondent type: HSA	Aware of SI: Yes - had heard of SI before	Coding phase: 1. Single coded	Client marital status: N/A - provider	Client age group: N/A - provider	Time since trained: A year or more ago

Codes Applied:	P1.1.1 - Positive provider attitudes towards SI	C1.1.1 - Positive client attitudes to SI	C8.3 - Discretion of injectables/SI	C5.1.4 - Timely injection due to not having to rely on external factors	C2.1.2 - Reduced trips to facility

INTERVIEWER: Ok, would you say the trend in DMPA-S uptake for self-injection has changed over time? What factors do you think have influenced this trend? From the time you introduced the method to now and please explain how the change has been.

RESPONDENT: I will explain more on a better change.

INTERVIEWER: Ok fine

RESPONDENT: Things have really changed for better

INTERVIEWER: Alright

RESPONDENT: As providers as well as clients we can see that there is a great change from the time the method was introduced compared to now in that; for the injection that was being administered by the provider every three months

INTERVIEWER: Yes

RESPONDENT: but for the new method After initial injection, the client is given extra units to use at home. This has led to more women getting interested in this method since it reduces visits that a client is supposed to make for her to go access the contraceptive.

INTERVIEWER: Alright

RESPONDENT: As such she is able to do other chores at home instead of spending much of her time at a facility. The other reason could be that the method has privacy, in that the client may use the method for a period of a year without visiting the facility ad people won’t notice that she is on taking any family planning method, because she will not be visiting the HSA.

INTERVIEWER: Yes, true

RESPONDENT: Yes

Title: MZs_HSA_4.docx

Descriptor Info:	District: Mzimba South	Target Population: TP1 - Providers/HSAs	Respondent type: HSA	Aware of SI: Yes - had heard of SI before	Coding phase: 1. Single coded	Client marital status: N/A - provider	Client age group: N/A - provider	Time since trained:

Codes Applied:	P1.1.1 - Positive provider attitudes towards SI

Interviewer: have you ever discussed this method with your peers or fellows?

Respondent: yes, we sometimes have discussions on this method.

Interviewer: what did you discuss?

Respondent: we discuss the advantages of self-injection. The Sayana need is different from the one of Depo since it is smaller. This encourages clients not to use Depo which has a long needle.

Interviewer: do you encourage the clients to use the injectable methods?

Respondent: yes.

Title: MZs_HSA_4.docx

Descriptor Info:	District: Mzimba South	Target Population: TP1 - Providers/HSAs	Respondent type: HSA	Aware of SI: Yes - had heard of SI before	Coding phase: 1. Single coded	Client marital status: N/A - provider	Client age group: N/A - provider	Time since trained:

Codes Applied:	P1.1.1 - Positive provider attitudes towards SI

Interviewer: did or do you have any worries about this method?

Respondent: at first yes, but not now.

Interviewer: is there any factor which has made you to change or not to change you perception of self-injectables?

Respondent: yes, the size of the needle because I did not know what type of the need used and how the whole process goes about. But with the training I do not have such worries.

Title: MZs_HSA_4.docx

Descriptor Info:	District: Mzimba South	Target Population: TP1 - Providers/HSAs	Respondent type: HSA	Aware of SI: Yes - had heard of SI before	Coding phase: 1. Single coded	Client marital status: N/A - provider	Client age group: N/A - provider	Time since trained:

Codes Applied:	C1.1.1 - Positive client attitudes to SI	P1.1.1 - Positive provider attitudes towards SI

Q17. Interviewer: we will hear your views on why women can choose or not choose the self-injection method. In your opinion which factors can enable women to adopt self-injection?

Respondent: self-injection syringe has a needle which does not cause much pain and that the method has a reasonable expiration duration. this is different from the pills method in which the client is required to take pills every day.

Interviewer: are you saying that a single injection is effective for three months?

Respondent: yes.

Interviewer: do you have anything to add?

Respondent: no.

Title: ZA_FAC_1.docx

Descriptor Info:	District: Zomba	Target Population: TP1 - Providers/HSAs	Respondent type: Facility-based provider	Aware of SI: Yes - had heard of SI before	Coding phase: 2. Double coded	Client marital status: N/A - provider	Client age group: N/A - provider	Time since trained: Less than a year ago

Codes Applied:	P1.1.1 - Positive provider attitudes towards SI

Respondent: eh but by and by we just take it as a take home message. After we preached it to the women and trained them

Interviewer: mmmm (showing that one is following the conversation)

Respondent: Then receive feedback from them some were verbalizing that I think it's not painful like Depo, be cause for Depo when we inject them some say that they will not be able to cook nsima

Interviewer: mmmm (showing that one is following the conversation)

Respondent: eh they were complaining that they arm gets swollen after getting Depo

Interviewer: mmmm (showing that one is following the conversation)

Respondent: but for Sayana they were saying that the arm was not painful, so I began to understand about the physiological makeup of the area where the injection is administered on

Interviewer: mmmm (showing that one is following the conversation)

Respondent: I think the area does not have many nerves, so I understood it well

Interviewer: yah yah

Respondent: it was a little bit some resistance yah when I saw that OK, that's when I understood the feedback that Sayana is not painful, but at first

Interviewer: alright

Respondent: yah

Interviewer: So we can say that after some time your perspective has changed, right?

Respondent: yes, there was a renew of mind

Interviewer: alright,

Respondent: yes

Interviewer: I want to know, you have told me that you had some resistance, did you talk about this this your work colleagues?

Respondent: no,

Interviewer: mmmm (showing that one is following the conversation)

Respondent: we did not talk about it

Interviewer: alright

Respondent: eh

Interviewer: alright, you have said that you had doubts about the possibility of women self-injecting.

Respondent: mmmm (showing that one is following the conversation)

Interviewer: After some time, can you say that you can encourage women to self-inject?

Respondent: absolutely

Interviewer: alright

Respondent: that’s the perfect method.

Title: ZA_FAC_1.docx

Descriptor Info:	District: Zomba	Target Population: TP1 - Providers/HSAs	Respondent type: Facility-based provider	Aware of SI: Yes - had heard of SI before	Coding phase: 2. Double coded	Client marital status: N/A - provider	Client age group: N/A - provider	Time since trained: Less than a year ago

Codes Applied:	P1.1.1 - Positive provider attitudes towards SI

Interviewer: alright

Respondent: when every new method is introduced there is research backing the method

Interviewer: mmmm (showing that one is following the conversation)

Respondent: eh, after the research we will be told that we need to change and do things this new way

Interviewer: yah yah

Respondent: we also act like agents of receptance of feedback

Interviewer: mmmm (showing that one is following the conversation)

Respondent: from them

Interviewer: alright

Respondent: that’s why we have some forms that we are supposed to fill when there are complications, or ma side effects whatever we are supposed to record them and keep them, so I was keen to receive feedback from them especially from the first team that we trained

Title: ZA_FAC_1.docx

Descriptor Info:	District: Zomba	Target Population: TP1 - Providers/HSAs	Respondent type: Facility-based provider	Aware of SI: Yes - had heard of SI before	Coding phase: 2. Double coded	Client marital status: N/A - provider	Client age group: N/A - provider	Time since trained: Less than a year ago

Codes Applied:	P1.1.1 - Positive provider attitudes towards SI

Respondent: of course there was some resistance, and they had fears because injection its injection

Interviewer: yah yah

Respondent: nobody is happy to receive an injection, no, but by and by I saw that the clients came and said that they use Sayana, so you see that ah I think things are working now

Interviewer: yah yah

Respondent: and some of them could tell their friends that they should get Sayana and not Depo

Interviewer: mmmm (showing that one is following the conversation)

Respondent: Depo is painful and Sayana is not, that’s what they could say.

Interviewer: mmmm (showing that one is following the conversation)

Respondent: So I could see that the research that was done benefitted because the implementation is working on the ground,

Title: ZA_FAC_1.docx

Descriptor Info:	District: Zomba	Target Population: TP1 - Providers/HSAs	Respondent type: Facility-based provider	Aware of SI: Yes - had heard of SI before	Coding phase: 2. Double coded	Client marital status: N/A - provider	Client age group: N/A - provider	Time since trained: Less than a year ago

Codes Applied:	P1.1.1 - Positive provider attitudes towards SI	P3.1.1 - Reduced provider workload

Interviewer: Alright, how much DMPA-SC is accessed for self-injection at this hospital?

Respondent: if we could use a scale of out of 10

Interviewer: eh in every 10 women

Respondent: we can out it at 7

Interviewer: alright

Respondent: they access it because we give them the information

Interviewer: mmmm (showing that one is following the conversation)

Respondent: we always know that when we miss the information, then the quality of the service will be compromised.

Interviewer: yah yah

Respondent: yah because if you can see, in most of the facilities including ours.

Interviewer: mmmm (showing that one is following the conversation)

Respondent: the space is not enough; the rooms are few

Interviewer: mmmm (showing that one is following the conversation)

Respondent: so we saw that the introduction of Sayana will do us good, congestion will no longer be there

Interviewer: mmmm (showing that one is following the conversation)

Respondent: because for many women we will inject them, and we will not see them again for the next three months.

Interviewer: mmmm (showing that one is following the conversation)

Respondent: so it will be almost one year if we include the three injection units, we have given them for self-injection

Interviewer: mmmm (showing that one is following the conversation)

Respondent: so those women will just be coming to the OPD to seek treatment of diseases and they will not be coming to the family planning clinic, that means on the issue of congestion, we are relieved.

Title: ZA_FAC_1.docx

Descriptor Info:	District: Zomba	Target Population: TP1 - Providers/HSAs	Respondent type: Facility-based provider	Aware of SI: Yes - had heard of SI before	Coding phase: 2. Double coded	Client marital status: N/A - provider	Client age group: N/A - provider	Time since trained: Less than a year ago

Codes Applied:	P1.1.1 - Positive provider attitudes towards SI	C1.1.3 - Awareness of DMPA-SC SI

Interviewer: alright

Respondent: when they elf-inject it was motivating other women, so the result was that out of the people that we see we can put it at 7 out of 10

Interviewer: alright.

Respondent: eh only two are the ones saying that when we come back the next time, we will get DMPA-SC for self-injection.

Interviewer: alright.

Respondent: that’s what was happening

Interviewer: Alright, has the trend of uptake of DMPA-SC for self-injection changed over time since it was introduced?

Respondent: mmm come again

Interviewer: ah have you observed any change in the number of women who say that they will take DMPA-SC for self-injection after some time?

Respondent: yes, I can say that there is a change

Interviewer: mmmm (showing that one is following the conversation)

Respondent: it has changed after some time especially in the first three months after Sayana was introduced.

Interviewer: mmmm (showing that one is following the conversation)

Respondent: we were facing a problem, the graph increasing in a decreasing way with some resistance

Interviewer: mmmm (showing that one is following the conversation)

Respondent: but after those first three months we were now on track

Interviewer: mmmm (showing that one is following the conversation)

Respondent: when every client came, they would say that I want to collect my Sayana doses and go

Interviewer: mmmm (showing that one is following the conversation)

Respondent: as I have said, we cannot separate family planning and culture, when they went back to the communities, they would preach about Sayana

Interviewer: mmmm (showing that one is following the conversation)

Respondent: so when the clients came, they started seeing family planning as Sayana

Interviewer: mmmm (showing that one is following the conversation)

Respondent: they would say, I have also come, and I want to collect my Sayana and self-inject, so for the clients it was already preached out there in the communities.

Interviewer: mmmm (showing that one is following the conversation)

Respondent: they would come here to only access what they had planned in their mind so we could not struggle, we could actually see that Depo was becoming less popular, since we were saying that Depo is for the facility and the HSAs when they do their clinics. But for Sayana you are free to take because its user friendly, you can easily store it, you can self-inject, and things like those.

Interviewer: mmmm (showing that one is following the conversation)

Respondent: so we observed that the clients were driving towards self-injection, eh but because of those hindrance we saw that the progress just dropped like bomb.

Title: ZA_FAC_2.docx

Descriptor Info:	District: Zomba	Target Population: TP1 - Providers/HSAs	Respondent type: Facility-based provider	Aware of SI: Yes - had heard of SI before	Coding phase: 3. Triple coded	Client marital status: N/A - provider	Client age group: N/A - provider	Time since trained: Less than a year ago

Codes Applied:	P2.1.2 - Sufficient original provider training	P1.1.1 - Positive provider attitudes towards SI	P3.1.1 - Reduced provider workload

Interviewer: Overall, what do you think about the training you had?

Respondent: Okay, the training was successful to me because as a provider, the way we give depo it’s quite different with the way we give Sayana press. So the training was successful to me because I learnt a lot of things, the things which I learnt there which can reduce my work load because if I teach my clients about the self-injection, they can inject themselves at their homes which will make my work to be reduced.

Interviewer: Anything else you’d like to add?

Respondent: About Sayana press?

Interviewer: About the training you received; what do you think about it?

Respondent: Okay, mmm, to my thinking, if I compare the way we do when providing depo and Sayana. I find it simple and easier to give Sayana than depo.

Title: ZA_FAC_2.docx

Descriptor Info:	District: Zomba	Target Population: TP1 - Providers/HSAs	Respondent type: Facility-based provider	Aware of SI: Yes - had heard of SI before	Coding phase: 3. Triple coded	Client marital status: N/A - provider	Client age group: N/A - provider	Time since trained: Less than a year ago

Codes Applied:	P1.1.1 - Positive provider attitudes towards SI	P3.1.1 - Reduced provider workload

Interviewer: I am curious to know about the experiences you encounter when administering the self-injection family planning method at this hospital or at an outreach. When you and your friends first found out that it was possible for females to apply the self-injection method of family planning on their own, how did you feel?

Respondent: I was happy and I accepted the idea since it means that the workload has now been reduced.

Interviewer: Okay fine. So have your thoughts towards the self-injection family planning method changed recently?

Respondent: No, they haven’t changed.

Interviewer: Everything is the same nothing has changed?

Respondent: Yes.

Interviewer: When with your friends, perhaps fellow nurses, do you discuss the Sayana press family planning method?

Respondent: Yes, we do. We also encourage each other that if the family planning method is not appearing in the register, especially the self-injectable one, we encourage each other that we should be teaching the people so that it can be seen that we are doing our job.

Title: ZA_FAC_2.docx

Descriptor Info:	District: Zomba	Target Population: TP1 - Providers/HSAs	Respondent type: Facility-based provider	Aware of SI: Yes - had heard of SI before	Coding phase: 3. Triple coded	Client marital status: N/A - provider	Client age group: N/A - provider	Time since trained: Less than a year ago

Codes Applied:	P88 - Provider great quotes	C2.1.2 - Reduced trips to facility	P1.1.1 - Positive provider attitudes towards SI

Interviewer: Thank you very much. Is there any reason why you have changed or unchanged views towards the method?

Respondent: The reason for the change is that before the Sayana method was introduced, women after getting the depo method would go home and come back after 3months but with the introduction of the Sayana method once a woman gets it, she will have to visit the hospital again the following year which is also like a long-term method since the woman stays at home and comes back the other year.

Title: MZs_HSA_3.docx

Descriptor Info:	District: Mzimba South	Target Population: TP1 - Providers/HSAs	Respondent type: HSA	Aware of SI: Yes - had heard of SI before	Coding phase: 3. Triple coded	Client marital status: N/A - provider	Client age group: N/A - provider	Time since trained: Less than a year ago

Codes Applied:	P1.1.1 - Positive provider attitudes towards SI	P3.1.1 - Reduced provider workload

Q.5 Interviewer: When you and your colleagues first heard that DMPA-SC could be injected by women themselves, what was your reaction?

Respondent: our reaction was we accepted that one because it could ease us instead of every three months people coming when we give them, we instruct them they can stay at home for almost a year yah! So they were easing partly our job yah!

Interviewer: anything else?

Respondent: the…way…we…were…. briefing them I should say taking tha tho the community that they should do this the clients we had to reduce our job in the way that most of them could carry to their homes but very few who could not understand they were the people we were supposed to give but those who could understand we said they will come after a year soo we were to see some people at longer period. Yah!

Interviewer: did you have any concerns about this idea?

Respondent:…. Yah that’s where we we knew that anybody is able to do what is told. So then we said if we can tell a clients they can do what if me a health worker failing to do so I have to continue yah…it give me courage.

Title: MZs_HSA_3.docx

Descriptor Info:	District: Mzimba South	Target Population: TP1 - Providers/HSAs	Respondent type: HSA	Aware of SI: Yes - had heard of SI before	Coding phase: 3. Triple coded	Client marital status: N/A - provider	Client age group: N/A - provider	Time since trained: Less than a year ago

Codes Applied:	C8.1 - Male involvement, general	C8.3 - Discretion of injectables/SI	P1.1.1 - Positive provider attitudes towards SI

Interviewer: were you/are supportive of the idea of women injecting administering their own contraceptive?

Respondent: yes…because most of them coming every month every three months they were having problems with either their husbands or them coming here they were known by other people that this one is getting may be family planning so the.. those fears with the women they come out of their place because they could just come once then they come the other time it was time taken for them to come back.

Title: KK_HSA_2.docx

Descriptor Info:	District: Nkhotakota	Target Population: TP1 - Providers/HSAs	Respondent type: HSA	Aware of SI: Yes - had heard of SI before	Coding phase: 2. Double coded	Client marital status: N/A - provider	Client age group: N/A - provider	Time since trained: A year or more ago

Codes Applied:	P1.1.1 - Positive provider attitudes towards SI

INTERVIEWER: Are you supportive of the idea of women administering their own contraceptives?

RESPONDENT: Yes very much

INTERVIEWER: Your so supportive?

RESPONDENT: Mhhh

Title: KK_HSA_2.docx

Descriptor Info:	District: Nkhotakota	Target Population: TP1 - Providers/HSAs	Respondent type: HSA	Aware of SI: Yes - had heard of SI before	Coding phase: 2. Double coded	Client marital status: N/A - provider	Client age group: N/A - provider	Time since trained: A year or more ago

Codes Applied:	P1.1.1 - Positive provider attitudes towards SI	C2.1.2 - Reduced trips to facility

INTERVIEWER: Mhh so would you say that the trend in DMPA-SC uptake of injection has changed over time. If so what factors do you think have influenced this trend.

RESPONDENT: Yes there has been a change for instance if one takes Depo she would be visiting the facility every three months whilst DMPA _Sc its only once a year. Hence she would prefer Sayana.

Title: KK_HSA_2.docx

Descriptor Info:	District: Nkhotakota	Target Population: TP1 - Providers/HSAs	Respondent type: HSA	Aware of SI: Yes - had heard of SI before	Coding phase: 2. Double coded	Client marital status: N/A - provider	Client age group: N/A - provider	Time since trained: A year or more ago

Codes Applied:	P1.1.1 - Positive provider attitudes towards SI	C5.1.4 - Timely injection due to not having to rely on external factors	P3.2.2 - Stockouts/stocks near expiry dates

INTERVIEWER: Alright, now am on point where you have counselled a client on DMPA-SC what do you think would attract a client to use the Sayana method since there is also Depo. What is it that attracts women the most?

RESPONDENT: aaa sometimes we come across incidences where Depo is out of stock

INTERVIEWER: Yes

RESPONDENT: Whilst in case of Sayana one is given a stock for a year, compared to Depo which would require a client to come again after three months and then she find out that Depo is out of stock.

INTERVIEWER: Okay so that that’s what attracts women the most not so

RESPONDENT: Yes

Title: ZA_HSA_3.docx

Descriptor Info:	District: Zomba	Target Population: TP1 - Providers/HSAs	Respondent type: HSA	Aware of SI: Yes - had heard of SI before	Coding phase: 1. Single coded	Client marital status: N/A - provider	Client age group: N/A - provider	Time since trained: A year or more ago

Codes Applied:	P1.1.1 - Positive provider attitudes towards SI

Interviewer: Okay, how did you or your colleagues react when you first heard that it is possible for a woman inject herself a family planning injection?

Respondent: I felt great because our workload reduced, and it mean that the number of people who were coming here reduced as well because they were able to help themselves at home. The number of people who were coming here for Sayana reduced hence we were less occupied.

Title: ZA_HSA_3.docx

Descriptor Info:	District: Zomba	Target Population: TP1 - Providers/HSAs	Respondent type: HSA	Aware of SI: Yes - had heard of SI before	Coding phase: 1. Single coded	Client marital status: N/A - provider	Client age group: N/A - provider	Time since trained: A year or more ago

Codes Applied:	P1.1.1 - Positive provider attitudes towards SI

Interviewer: Alright thank you very much, what are your thoughts of the self-injecting family planning injection, aahh going back did your thoughts on the self-injecting family planning injection change lately?

Respondent: No my thoughts did not change because even the clients preferred Sayana because they were able to inject themselves when their day is due and any other time, they feel free to inject themselves, so our thoughts were that even many people choose this Sayana press family planning method.

Interviewer: Alright thank you very much.

Respondent: So in our thoughts we saw that in this case we can be able to control population country wide.

Title: ZA_HSA_3.docx

Descriptor Info:	District: Zomba	Target Population: TP1 - Providers/HSAs	Respondent type: HSA	Aware of SI: Yes - had heard of SI before	Coding phase: 1. Single coded	Client marital status: N/A - provider	Client age group: N/A - provider	Time since trained: A year or more ago

Codes Applied:	P1.1.1 - Positive provider attitudes towards SI

Interviewer: Mhhhmmm... when you are with your friends what to you talk about concerning self-injection Sayana family planning method?

Respondent: We discuss that it is good that Sayana press was introduced because we no longer get busy giving family planning services hence it reduced our work load.

Interviewer: As an individual do you encourage the possibility of a woman to inject herself with family planning injection?

Respondent: Yes, I encourage.

Title: ZA_HSA_3.docx

Descriptor Info:	District: Zomba	Target Population: TP1 - Providers/HSAs	Respondent type: HSA	Aware of SI: Yes - had heard of SI before	Coding phase: 1. Single coded	Client marital status: N/A - provider	Client age group: N/A - provider	Time since trained: A year or more ago

Codes Applied:	P1.1.1 - Positive provider attitudes towards SI

Interviewer: Alright, is there anything that made you change your mind set on the self-injecting family planning Jackson?

Respondent: Ahhh there is nothing that made me change my thoughts, I still think the method is fine,

Title: ZA_HSA_6.docx

Descriptor Info:	District: Zomba	Target Population: TP1 - Providers/HSAs	Respondent type: HSA	Aware of SI: Yes - had heard of SI before	Coding phase: 1. Single coded	Client marital status: N/A - provider	Client age group: N/A - provider	Time since trained:

Codes Applied:	P1.2.1 - Negative provider attitudes towards SI	P1.1.1 - Positive provider attitudes towards SI	P3.4 - Time to counsel/train, general

Interviewer: Alright, I am curious on what you have experienced in providing self-injection contraceptive at this facility and in your community. Ah when you and your colleagues first heard about the possibility of a woman self-injecting, how did you feel?

Respondent: at first, I thought that it would be difficult.

Interviewer: mmmm (showing that one is following the conversation)

Respondent: But after we did the practical it showed that the number of women who self-inject is starting to increase gradually. But most of them were worried that since a person is self-injecting they thought that they will not be doing it right, so there still required counselling, that the trainer should have enough counselling so that when they have been trained they should get the correct information because sometimes maybe the training that we do is very fast due to a time factor, but if the ones being trained, that we have people with great skill who can also train their friends it can be helpful so that there should be no problems.

Title: ZA_HSA_6.docx

Descriptor Info:	District: Zomba	Target Population: TP1 - Providers/HSAs	Respondent type: HSA	Aware of SI: Yes - had heard of SI before	Coding phase: 1. Single coded	Client marital status: N/A - provider	Client age group: N/A - provider	Time since trained:

Codes Applied:	P88 - Provider great quotes	P1.1.1 - Positive provider attitudes towards SI

Interviewer: alright, do you encourage women on self-injection?

Respondent: ah yes, a lot, it’s possible.

Interviewer: mmmm (showing that one is following the conversation)

Respondent: even up to composing songs about self-injection family planning

Interviewer: ya yah yah

Respondent: we are ready we can compose music and sing in the group

Interviewer: mmmm (showing that one is following the conversation)

Respondent: the clines could be attracted to the music and follow what we are saying.

Interviewer: alright.

Respondent: sure

Title: ZA_HSA_6.docx

Descriptor Info:	District: Zomba	Target Population: TP1 - Providers/HSAs	Respondent type: HSA	Aware of SI: Yes - had heard of SI before	Coding phase: 1. Single coded	Client marital status: N/A - provider	Client age group: N/A - provider	Time since trained:

Codes Applied:	P1.1.1 - Positive provider attitudes towards SI	P3.1.1 - Reduced provider workload

Interviewer: When you heard about the possibility of women self-injecting, did you have any concerns?

Respondent: at first the concern was not that much

Interviewer: mmmm (showing that one is following the conversation)

Respondent: but I saw that it was a chance that we should have reduced work.

Interviewer: mmmm (showing that one is following the conversation)

Respondent: because if we were targeting to meet 100 people

Interviewer: mmmm (showing that one is following the conversation)

Respondent: and among the 100 maybe 30 people have accessed the self-injecting option

Interviewer: mmmm (showing that one is following the conversation)

Respondent: it means we will be meeting only 70 people for the whole year… which shows that some of our work has been reduced and we will be focusing on other work

Title: ZA_HSA_6.docx

Descriptor Info:	District: Zomba	Target Population: TP1 - Providers/HSAs	Respondent type: HSA	Aware of SI: Yes - had heard of SI before	Coding phase: 1. Single coded	Client marital status: N/A - provider	Client age group: N/A - provider	Time since trained:

Codes Applied:	C1.1.1 - Positive client attitudes to SI	P1.1.1 - Positive provider attitudes towards SI

Interviewer: What factors do you think have influenced to this trend?

Respondent: ah I see that it has risen because the clients say that the syringe for DMPA-SC is small.

Interviewer: mmmm (showing that one is following the conversation)

Respondent: and it is not painful, and because we do not inject inter-muscular yeah

Interviewer: mmmm (showing that one is following the conversation)

Respondent: they see that it will not cause them any problems in their body different from the other type of injections which are 0.5 or injection like Depo.

Interviewer: mmmm (showing that one is following the conversation)

Respondent: eh so I see that currently the clients think that it has advantages, and it is not painful

Title: MZs_FAC_2.docx

Descriptor Info:	District: Mzimba South	Target Population: TP1 - Providers/HSAs	Respondent type: Facility-based provider	Aware of SI: Yes - had heard of SI before	Coding phase: 2. Double coded	Client marital status: N/A - provider	Client age group: N/A - provider	Time since trained:

Codes Applied:	P1.1.1 - Positive provider attitudes towards SI	C5.1.4 - Timely injection due to not having to rely on external factors

Interviewer: Were you or are supportive of the idea of women administering their own contraception?

Respondent: Yes, I do support them so that during rainy season they should just be injecting themselves and concentrating on their farms.

Title: MZs_FAC_2.docx

Descriptor Info:	District: Mzimba South	Target Population: TP1 - Providers/HSAs	Respondent type: Facility-based provider	Aware of SI: Yes - had heard of SI before	Coding phase: 2. Double coded	Client marital status: N/A - provider	Client age group: N/A - provider	Time since trained:

Codes Applied:	P1.1.1 - Positive provider attitudes towards SI

Q17. Interviewer: In your view, what are the main factors that would support a client to take up the self-injection option with DMPA-SC?

Respondent: The method is good since it makes a client not to visit the facility regularly. The only challenge can be to those who cannot inject themselves

Interviewer: Mmmmmhh….? (Really)

Respondent: Yes, thus the problem.

Interviewer: Alright. In your view, what are the main factors that would support a client to take up the self-injection option?

Respondent: Less number of visiting the facility

Interviewer: What else?

Respondent: To avoid pregnancy

Title: MZs_HSA_6.docx

Descriptor Info:	District: Mzimba South	Target Population: TP1 - Providers/HSAs	Respondent type: HSA	Aware of SI: Yes - had heard of SI before	Coding phase: 1. Single coded	Client marital status: N/A - provider	Client age group: N/A - provider	Time since trained: A year or more ago

Codes Applied:	P1.1.1 - Positive provider attitudes towards SI	P3.1.1 - Reduced provider workload	C2.1.2 - Reduced trips to facility

Interviewer: When you first heard that Sayana is for self-injection, what was your reaction with your colleagues.

Respondent: it was a good thing because we would provide a woman with Sayana for self-injection then she would not be coming to the provider frequently

Interviewer: what was your reaction before starting providing after hearing the rumors that women will self-inject

Respondent: it was a welcome development

Interviewer: did you talk with your colleagues at this facility upon hearing that women will self-inject at their homes

Respondent: we were wondering what kind of the method it was for a woman to self-inject. So we had agreed to go to the training to learn more about it.

Title: KK_HSA_6.docx

Descriptor Info:	District: Nkhotakota	Target Population: TP1 - Providers/HSAs	Respondent type: HSA	Aware of SI: Yes - had heard of SI before	Coding phase: 1. Single coded	Client marital status: N/A - provider	Client age group: N/A - provider	Time since trained: A year or more ago

Codes Applied:	P2.3 - Provider training content	P3.1.1 - Reduced provider workload	P1.1.1 - Positive provider attitudes towards SI

RESPONDENT: They trained us on how we can inject a woman and also how we can train a woman to self-inject Sayana. It was also to our advantage because they reduce our work load of injecting women. When they trained us, we understood and went into our communities training people. The women are self-injecting and now our job is just to update.

INTERVIEWER: Okay.

RESPONDENT: mmmh.

INTERVIEWER: What were the good parts of the training?

RESPONDENT: As an HSA, the good part of the training was that I learnt a lot for example how I can inject a person and how I can train them. Those were some of the things I learned.

Title: KK_HSA_6.docx

Descriptor Info:	District: Nkhotakota	Target Population: TP1 - Providers/HSAs	Respondent type: HSA	Aware of SI: Yes - had heard of SI before	Coding phase: 1. Single coded	Client marital status: N/A - provider	Client age group: N/A - provider	Time since trained: A year or more ago

Codes Applied:	P1.1.1 - Positive provider attitudes towards SI

RESPONDENT: At the same time, I thought that the government was reducing our workload. But after I went for training, I realized that it was good.

Title: KK_HSA_6.docx

Descriptor Info:	District: Nkhotakota	Target Population: TP1 - Providers/HSAs	Respondent type: HSA	Aware of SI: Yes - had heard of SI before	Coding phase: 1. Single coded	Client marital status: N/A - provider	Client age group: N/A - provider	Time since trained: A year or more ago

Codes Applied:	P1.1.1 - Positive provider attitudes towards SI

INTERVIEWER: Have these opinions change over time?

RESPONDENT: I realized that I am now comfortable and the women are also comfortable. It is very important for a person to self-inject and there is nothing to be afraid of.

Title: KK_HSA_6.docx

Descriptor Info:	District: Nkhotakota	Target Population: TP1 - Providers/HSAs	Respondent type: HSA	Aware of SI: Yes - had heard of SI before	Coding phase: 1. Single coded	Client marital status: N/A - provider	Client age group: N/A - provider	Time since trained: A year or more ago

Codes Applied:	P1.1.1 - Positive provider attitudes towards SI	A6.1.2 - Potential concerns about SI	C3.1.2 - Confidence/comfort to inject after counselling/ training

INTERVIEWER: mmmh. Did you discuss with your colleagues about this when you first heard?

RESPONDENT: We discussed with my colleagues in the community if it was going to work. We had opinions and one of them was that it is possible depending on how we will be trained and how we will be training the women. And then we saw that it worked and people were self-injecting. It’s only a few women maybe a young mother of 16 or 17, are the ones when trained, they still remain afraid. But the majority responds responds positively when trained.

Title: KK_HSA_6.docx

Descriptor Info:	District: Nkhotakota	Target Population: TP1 - Providers/HSAs	Respondent type: HSA	Aware of SI: Yes - had heard of SI before	Coding phase: 1. Single coded	Client marital status: N/A - provider	Client age group: N/A - provider	Time since trained: A year or more ago

Codes Applied:	C5.2.1 - Discontinuation of SI (including reasons)	C8.1 - Male involvement, general	P1.1.1 - Positive provider attitudes towards SI

INTERVIEWER: This about the women, right? How about the challenges that you have faced since you started providing DMPA-SC?

RESPONDENT: aaaa, I have not faced any challenges.

INTERVIEWER: everything is perfect.

RESPONDENT: Aaah everything is okay. But some men are saying that because of this injection, it is reducing their sexual drive. I told them that it is not possible for the drugs to affect them because they were nit injected. But maybe the sexual drive might decrease in women and not in men. Also women were telling me that sexual drive is decreasing, they have stomach aches, headaches and some back aches. And we tell them to go to the hospital If they have this [roble.

Title: KK_HSA_6.docx

Descriptor Info:	District: Nkhotakota	Target Population: TP1 - Providers/HSAs	Respondent type: HSA	Aware of SI: Yes - had heard of SI before	Coding phase: 1. Single coded	Client marital status: N/A - provider	Client age group: N/A - provider	Time since trained: A year or more ago

Codes Applied:	P1.1.1 - Positive provider attitudes towards SI	C5.1.4 - Timely injection due to not having to rely on external factors

INTERVIEWER: The challenge that you have explained here is that…

RESPONDENT: Sometimes visiting the clients. Some maybe they have complaints like what I have already said. Some they miss their appointment date. I might book a particular number of people, but 6 or 10 might not show up. You follow up on these ones that did not show up. Some may say, I was sick or I went to the village for a funeral. Which is making the Sayana method to be simple and easy because whether they are sick or they have travelled, they have the drug and they will just self-inject.

Title: MZs_FAC_1.docx

Descriptor Info:	District: Mzimba South	Target Population: TP1 - Providers/HSAs	Respondent type: Facility-based provider	Aware of SI: Yes - had heard of SI before	Coding phase: 3. Triple coded	Client marital status: N/A - provider	Client age group: N/A - provider	Time since trained: A year or more ago

Codes Applied:	P1.2.1 - Negative provider attitudes towards SI	P1.1.1 - Positive provider attitudes towards SI

Interviewer: Overall, what did you think of the training how was it- you have said it was a good one, is there anything you would like to add about the training?

Respondent: It was a good training and it was an exciting one because even to us we were somehow surprise that you can teach someone to inject herself.

Interviewer: Mmmmmmh.

Respondent: Yah, so we say aaah, but will people understand this but once we went for training we really understood that it was just a simple procedures that anyone can do and it was really exciting and everybody was excited.

Title: MZs_FAC_1.docx

Descriptor Info:	District: Mzimba South	Target Population: TP1 - Providers/HSAs	Respondent type: Facility-based provider	Aware of SI: Yes - had heard of SI before	Coding phase: 3. Triple coded	Client marital status: N/A - provider	Client age group: N/A - provider	Time since trained: A year or more ago

Codes Applied:	P1.1.1 - Positive provider attitudes towards SI

Interviewer: And what was your reaction when you first head that a woman can inject herself?

Respondent: After going throw the training and seeing that I can even convince somebody to do it and actually seeing women doing it, no it was an exciting one.

Interviewer: Alright.

Respondent: Yah (yes).

Interviewer: So has the opinion of you or your colleague on self-injection changed over time?

Respondent: Yah it has really changed because now we are able to tell the women and those who are doubting or fearing we are there to convince them that no actually it is very simple.

Interviewer: Mmmmmh.

Respondent: And they agree to listen to what we are telling them actually they also see that they can do it and they can actually inject themselves. So the opinion has really changed.

Title: MZs_FAC_1.docx

Descriptor Info:	District: Mzimba South	Target Population: TP1 - Providers/HSAs	Respondent type: Facility-based provider	Aware of SI: Yes - had heard of SI before	Coding phase: 3. Triple coded	Client marital status: N/A - provider	Client age group: N/A - provider	Time since trained: A year or more ago

Codes Applied:	P1.1.1 - Positive provider attitudes towards SI	P3.1.1 - Reduced provider workload

Interviewer: Were you /are you supportive of the idea of a woman administering her own contraception?

Respondent: Yah, am for it actually.

Interviewer: Why?

Respondent: It reduces the burden to us as providers cause once we teach her once and she has gotten the content we give her this for a year which means may not report back for a year.

Interviewer: Mmmmmh

Respondent: So we know we may not have frequently visit to the hospital.

Title: MZs_FAC_1.docx

Descriptor Info:	District: Mzimba South	Target Population: TP1 - Providers/HSAs	Respondent type: Facility-based provider	Aware of SI: Yes - had heard of SI before	Coding phase: 3. Triple coded	Client marital status: N/A - provider	Client age group: N/A - provider	Time since trained: A year or more ago

Codes Applied:	P88 - Provider great quotes	P1.1.1 - Positive provider attitudes towards SI	P3.1.5 - Ease of training clients in SI

Interviewer: Okay, thank you so much. Aaaah you have said something like your opinion or your colleagues opinion on self-injection has changed

Respondent: Mmmmh (yes)

Interviewer: So I want to understand specifically what has influence your opinion to change?

Respondent: First is the simplicity of the injection itself is quiet that now I know that with good counselling anybody can understand it and even the woman that have received this thing they are willingly taking it so I really know that they do understand it that it is a simple thing and they can do it.

Interviewer: Mmmmh

Respondent: The simplicity is that the way they simplified that injection is the simple thing

Title: MZs_FAC_1.docx

Descriptor Info:	District: Mzimba South	Target Population: TP1 - Providers/HSAs	Respondent type: Facility-based provider	Aware of SI: Yes - had heard of SI before	Coding phase: 3. Triple coded	Client marital status: N/A - provider	Client age group: N/A - provider	Time since trained: A year or more ago

Codes Applied:	P1.1.1 - Positive provider attitudes towards SI	P88 - Provider great quotes

Q 19. Interviewer: Okay. We have now come to the end of the questions I had for you today, I just want to know if you have any other thoughts or information you would like to share on the topics we have discussed today?

Respondent: I think personally I feel extra excitement when am giving Sayana injection and am demonstrating to women because some of them they will show that they are doubting, so actually when I have DEPO and Sayana in stock I really very good to demonstrate to the client so that they should be able to understand and convince them that they can do it by themselves and when am done with the demonstration and they are now doing it, it is one of the encouraging part to me that I have taught them and they are doing it themselves the family planning methods.

Title: ZA_HSA_1.docx

Descriptor Info:	District: Zomba	Target Population: TP1 - Providers/HSAs	Respondent type: HSA	Aware of SI: Yes - had heard of SI before	Coding phase: 1. Single coded	Client marital status: N/A - provider	Client age group: N/A - provider	Time since trained: Less than a year ago

Codes Applied:	P1.1.1 - Positive provider attitudes towards SI	P3.1.1 - Reduced provider workload

Respondent: Our thoughts are that we have to decrease the number of people who come to the hospital. Once they learn on how to self-inject, they will be able to self-inject when they go home.

Interviewer: Alright alright

Respondent: That means there won’t be long queues of people seeking family planning methods

Interviewer: You mentioned that you went for training. On the training you had, what were the good parts of the training?

Respondent: The good things we learnt?

Interviewer: Yes, what are the good things of the training

Respondent: It was training us to decongest the hospital because when the day arrives they self-inject at home.

Title: ZA_HSA_1.docx

Descriptor Info:	District: Zomba	Target Population: TP1 - Providers/HSAs	Respondent type: HSA	Aware of SI: Yes - had heard of SI before	Coding phase: 1. Single coded	Client marital status: N/A - provider	Client age group: N/A - provider	Time since trained: Less than a year ago

Codes Applied:	P1.1.1 - Positive provider attitudes towards SI	P3.1.1 - Reduced provider workload

Interviewer: Alright Alright, thank you very much. We are now moving into the third topic. I am interested in your experience of providing DMPA-SC in the community. When you and your colleagues first heard of the possibility of a woman to self-inject, what was your reaction?

Respondent: We were happy that it is now possible, queues will be shorter here at the hospital

Interviewer: Umm

Respondent: When people self-inject

Title: ZA_HSA_1.docx

Descriptor Info:	District: Zomba	Target Population: TP1 - Providers/HSAs	Respondent type: HSA	Aware of SI: Yes - had heard of SI before	Coding phase: 1. Single coded	Client marital status: N/A - provider	Client age group: N/A - provider	Time since trained: Less than a year ago

Codes Applied:	P1.1.1 - Positive provider attitudes towards SI

Interviewer: Do you and your colleagues talk about anything regarding this type of injectables?

Respondent: Yes, we do

Interviewer: What do you talk about?

Respondent: That is a good injectable as it is small and easy to store. Furthermore, it takes 3 months to re inject. You cannot forget as compared to pills as others forget. Once you get injected it will take 3 months to re inject.

Interviewer: Are you supportive of the idea of women administering their own contraception?

Respondent: Yes I am supportive

Title: MZs_HSA_2.docx

Descriptor Info:	District: Mzimba South	Target Population: TP1 - Providers/HSAs	Respondent type: HSA	Aware of SI: Yes - had heard of SI before	Coding phase: 1. Single coded	Client marital status: N/A - provider	Client age group: N/A - provider	Time since trained: A year or more ago

Codes Applied:	P1.1.1 - Positive provider attitudes towards SI	P3.1.1 - Reduced provider workload	C5.1.4 - Timely injection due to not having to rely on external factors

Interviewer: what are your thoughts on the training you received

Respondent: it’s a very good development to us. Because if we teach women to self-inject Sayana she takes it home and we have time to do other things because she comes again after a year

Interviewer: mmmh (agreeing) what were the important thing of the training

Respondent: about Sayana

Interviewer: mmmmh (thinking out loud) about the training that you received that you saw

Respondent: may be its what I have already said. But Sayana is good for women who travel to South Africa because they take for the whole year. Even for the rest of the women they take about a year before we see each other again.

Title: MZs_HSA_2.docx

Descriptor Info:	District: Mzimba South	Target Population: TP1 - Providers/HSAs	Respondent type: HSA	Aware of SI: Yes - had heard of SI before	Coding phase: 1. Single coded	Client marital status: N/A - provider	Client age group: N/A - provider	Time since trained: A year or more ago

Codes Applied:	C5.1.4 - Timely injection due to not having to rely on external factors	P3.1.1 - Reduced provider workload	P1.1.1 - Positive provider attitudes towards SI

Interviewer: mmmmh (agreeing) now I would like to hear about your experiences as someone who provides Sayana Press for self-injection here at the facility and at the community. After you heard that women will self-inject what was your reaction

Respondent: I was happy because it would reduce number of clients seen

Interviewer: what did you and your colleague discuss about Sayana Press

Respondent: we saw it as a good method which would reduce workload since women would get dosage for one year at home. mmmmh (agreeing to the point)

Interviewer: what else

Respondent: it is a good method for women who travel to South Africa because they take a dose for one year

Interviewer: were you in support of the idea of women to self-inject

Respondent: yes

Title: MZs_HSA_2.docx

Descriptor Info:	District: Mzimba South	Target Population: TP1 - Providers/HSAs	Respondent type: HSA	Aware of SI: Yes - had heard of SI before	Coding phase: 1. Single coded	Client marital status: N/A - provider	Client age group: N/A - provider	Time since trained: A year or more ago

Codes Applied:	P1.1.1 - Positive provider attitudes towards SI

Interviewer: did you have any concerns that women will self-inject

Respondent: I did not necessarily have any concerns because we would teach these women before they self-inject then they practice for those who have problems we assist them until they are also capable

Title: KK_FAC_2.docx

Descriptor Info:	District: Nkhotakota	Target Population: TP1 - Providers/HSAs	Respondent type: Facility-based provider	Aware of SI: Yes - had heard of SI before	Coding phase: 1. Single coded	Client marital status: N/A - provider	Client age group: N/A - provider	Time since trained: A year or more ago

Codes Applied:	P1.1.1 - Positive provider attitudes towards SI	P1.2.1 - Negative provider attitudes towards SI	C1.2.2 - Fear of SI (idea or reality)

INTERVIEWER: mmmmhu. We are proceeding. Now I am interested in your experiences of providing Sayana at this facility. So when you first heard that DMPA-SC, women will be self-injecting, how did you feel?

RESPONDENT: At first, aaa I was happy by the thought of a woman injecting herself. But I was interested to know how they will be self-injecting because this is a body and an injection is very painful. Yeah, so when we were trained on how a woman can self-inject, it was interesting for us to continue this work.

INTERVIEWER: mmmmhu.

RESPONDENT: yes.

INTERVIEWER: What did you discuss with your colleagues when you heard about this?

RESPONDENT: With my colleagues, everyone was interested to go for training because we really wanted to go for the self-injection training because we were not providing this method at the facility. Everyone was interested if they could go to the training to see what happens.

INTERVIEWER: mmmmhu.

RESPONDENT: mmmh.

INTERVIEWER: What about before you knew that there will be a training, what were you discussing with your colleagues about Sayana which women will be self-injecting?

RESPONDENT: mmmmhu.

INTERVIEWER: What were you discussing?

RESPONDENT: We were discussing that with the coming in of this method, we will see what will happen, because a lot of women are afraid to self-inject before they are trained.
