# Supplementary material for: Health care provider and client experiences of counselling on depot medroxyprogesterone acetate subcutaneous (DMPA-SC) for self-injection in Malawi
Source: PLOS Glob Public Health. 2023 Nov 30;3(11):e0002057. doi: 10.1371/journal.pgph.0002057 (PMC10688740; doi:10.1371/journal.pgph.0002057)
Supplement: S1 Data — (ZIP) [file pgph.0002057.s001.zip › S2/P1.2.1 provider negative percept.doc]

Title: ZA_HSA_2.docx

Descriptor Info:	District: Zomba	Target Population: TP1 - Providers/HSAs	Respondent type: HSA	Aware of SI: Yes - had heard of SI before	Coding phase: 3. Triple coded	Client marital status: N/A - provider	Client age group: N/A - provider	Time since trained: Less than a year ago

Codes Applied:	P1.2.1 - Negative provider attitudes towards SI	C1.2.2 - Fear of SI (idea or reality)	C3.1.2 - Confidence/comfort to inject after counselling/ training

Interviewer: Ah and I wanted to know the first time that you heard about this injection that women can self-inject, did you have any concerns on the possibility of women to self-inject?

Respondent: Yes, I had concerns, it was that for most people to self-inject, even any type of injection, we tend to be scared, the same thing when at the hospital they tell you that we will give you an injection, we are scared and concerned…

Interviewer: Mmmm (Showing that one is following the conversation)

Respondent: The same concerns and fears we had them, we thought, will these women accept to self-inject? most of them are not as brave, but now these women no longer have those fears because at first, they thought the injection was painful…

Interviewer: mmm (Showing agreement)

Respondent: But after they were trained and they started using the injection, they appreciated it a lot.

Title: KK_HSA_1.docx

Descriptor Info:	District: Nkhotakota	Target Population: TP1 - Providers/HSAs	Respondent type: HSA	Aware of SI: Yes - had heard of SI before	Coding phase: 2. Double coded	Client marital status: N/A - provider	Client age group: N/A - provider	Time since trained: A year or more ago

Codes Applied:	P1.2.1 - Negative provider attitudes towards SI

Interviewer: Alright. Thank you. I am now interested in your experience in providing this family planning method of self injection in your area.

Respondent: Mmmhu. Only…

Interviewer: So I want…I will ask you questions about what?

Respondent: The experiences.

Interviewer: The experiences. When you first heard that there is an injection that women could be self injecting themselves right?

Respondent: mmmhu

Interviewer: What was your reaction when you first heard?

Respondent: Eee. When we first heard, we thought that it was impossible for these women to self-inject themselves. But when we were trained, we saw that it is possible.

Interviewer: mmmhu.

Respondent: Mmmmhu. But our worry was that, it is even problematic to inject a health worker. So will it be possible for a woman to self inject? So we thought that it was a difficult thing.

Interviewer: mmm

Respondent: mmmm

Title: KK_HSA_1.docx

Descriptor Info:	District: Nkhotakota	Target Population: TP1 - Providers/HSAs	Respondent type: HSA	Aware of SI: Yes - had heard of SI before	Coding phase: 2. Double coded	Client marital status: N/A - provider	Client age group: N/A - provider	Time since trained: A year or more ago

Codes Applied:	P1.2.1 - Negative provider attitudes towards SI	C1.2.2 - Fear of SI (idea or reality)	P1.1.1 - Positive provider attitudes towards SI	P88 - Provider great quotes

Interviewer: So with colleagues, maybe your fellow HSA’s did you discuss when you first heard of Sayana. Did you discuss?

Respondent: yes.

Interviewer: What did you discuss?

Respondent: About the challenges. We were saying that it is not possible because it is a new thing. We were saying that it will not be possible because women are even afraid of injection…they are afraid of tetanus vaccine for pregnant women. They run. And they should say a family planning method people should be self injecting, it can not be possible. So it was like an argument. But when we went for training and started providing, we saw that it was what, it was working.

Title: KK_HSA_1.docx

Descriptor Info:	District: Nkhotakota	Target Population: TP1 - Providers/HSAs	Respondent type: HSA	Aware of SI: Yes - had heard of SI before	Coding phase: 2. Double coded	Client marital status: N/A - provider	Client age group: N/A - provider	Time since trained: A year or more ago

Codes Applied:	P88 - Provider great quotes	P3.2.3 - Complexity/difficulty of training clients in SI	P2.1.1 - Refresher training/mentorship	P1.2.1 - Negative provider attitudes towards SI	P3.3 - Counselling/ training approach, general

Interviewer: Alright. What is your experience, based on what you have seen since you started providing Sayana to women in your community, aaah what do women say when you explain that they can self-inject?

Respondent: At first women they…they…they doubt and they say that it is not possible for them to what, to self-inject. And we struggle with them. You counsel them and when you are done and ask is there anyone? aah I cant. All of them would say no. when that happens you as a provider, you have just wasted your time right, counselling them and everything eh…eh…by the end of that time everyone is saying no and you have to inject them. it could have been better to just begin…just inject them. But after some time, when they came for supervision they helped us. You should be doing it like this and here you do this, you will see you will have more clients, and that is when they told us that when doing group counselling, and there are two or three women, those ones should be examples to their friends. When we began using that system, we saw that it was working. When you train them, then because its women only, Mrs So-so-so you inject yourself. Your friends here are saying they cannot self-inject. So we will begin with you. And then you will give one woman who has self-injected before. And then she will inject on her thigh. You take pills…you also take the remaining 3 doses and off she goes. The second one will also do the same thing. And then the remaining ones will say oh it is possible and painless and it is short or at home you can do it. And then I saw that I now had what,

Interviewer: mmmmhu

Respondent: mmmh

Title: KK_HSA_5.docx

Descriptor Info:	District: Nkhotakota	Target Population: TP1 - Providers/HSAs	Respondent type: HSA	Aware of SI: Yes - had heard of SI before	Coding phase: 1. Single coded	Client marital status: N/A - provider	Client age group: N/A - provider	Time since trained: A year or more ago

Codes Applied:	P1.2.1 - Negative provider attitudes towards SI	P1.1.1 - Positive provider attitudes towards SI	C1.2.2 - Fear of SI (idea or reality)	C1.1.3 - Awareness of DMPA-SC SI

INTERVIEWER: … when you and your colleagues first heard that DMPA-SC, could be injected by women themselves, what was your reaction? And has the opinion of you/your colleagues on self-injection changed over time?

RESPONDENT: When we first heard we were so worried

INTERVIEWER: Mhhh

RESPONDENT: That not many women will manage to do that, considering the type of injection ( Depo) that we have administering to our clients. So we though would women be able to inject themselves? So after the training and seeing the size of the injection we noted that its possible. We can see that ore clients are interested in this method and that for those who have started using it they are able to self-inject so well on their own.

INTERVIEWER: Ok

RESPONDENT: For those who were so scared and afraid are the ones who are encouraging their friends to use this method. That’s the position as of now about DMPA-SC

Title: MZs_HSA_1.docx

Descriptor Info:	District: Mzimba South	Target Population: TP1 - Providers/HSAs	Respondent type: HSA	Aware of SI: Yes - had heard of SI before	Coding phase: 2. Double coded	Client marital status: N/A - provider	Client age group: N/A - provider	Time since trained:

Codes Applied:	P1.2.1 - Negative provider attitudes towards SI

Q5. Interviewer: When you or your colleagues first heard that DMPA-SC, could be injected by the women themselves, what was your reaction?

Respondent: It was a difficult thing thinking that will a woman allow to inject herself? Cause the way they react when being injected by the provider, so, for her to self-inject mmmm, they cannot manage.

Interviewer: Have you or your colleagues discussed about this?

Respondent: Yes, we did. Saying that will they allow to self-inject? Cause they feel reluctant whenever we inject them, so telling them to inject themselves will it work?

Title: MZs_HSA_1.docx

Descriptor Info:	District: Mzimba South	Target Population: TP1 - Providers/HSAs	Respondent type: HSA	Aware of SI: Yes - had heard of SI before	Coding phase: 2. Double coded	Client marital status: N/A - provider	Client age group: N/A - provider	Time since trained:

Codes Applied:	A5.1 - Covert/ discreet use	C8.1 - Male involvement, general	P1.2.1 - Negative provider attitudes towards SI

Interviewer: Are women not comfortable or not confident to inject themselves?

Respondent: Women are comfortable

Interviewer: What about adolescent?

Respondent: Adolescent are not confident

Interviewer: Why adolescent are not confident?

Respondent: I think it’s because the adolescent do take family planning method in secret and if they carry it at home parents will know that they are using family planning thus why they are not confident.

Interviewer: What those adolescent aged 18 or 19 and they are married, will they be not comfortable

Respondent: These also cannot be comfortable because their husband do not allow them to use family planning method because they want to start bearing children and if they can be using it secretly then they will not be confident

Title: KK_FAC_1.docx

Descriptor Info:	District: Nkhotakota	Target Population: TP1 - Providers/HSAs	Respondent type: Facility-based provider	Aware of SI: Yes - had heard of SI before	Coding phase: 3. Triple coded	Client marital status: N/A - provider	Client age group: N/A - provider	Time since trained: A year or more ago

Codes Applied:	P1.2.1 - Negative provider attitudes towards SI	P1.1.1 - Positive provider attitudes towards SI

INTERVIEWER: Alright, alright, I am still interested to know more, thought you seem to know more and you have shared that, But I am still interested to know your experience of providing DMPA-SC at the facility. I would like to know when you/ your colleagues first heard that DMPA-SC, could be injected by women themselves, what was your reaction? And has the opinion of you /your colleges on self-injection changed over time? How was your reaction when you first heard and now after time has passed has your opinion changed?

RESPONDENT: Of course, when I first heard about it, I was doubting as to whether Malawian women can manage to inject themselves. This thought came up because I was considering how the women reacts when one is injecting them, they are always afraid. So I was thinking, is this doer able that one can inject herself . But with passing of time, we see clients coming to request for the method.

INTERVIEWER: okay

RESPONDENT: May I have Sayana.

Title: KK_FAC_1.docx

Descriptor Info:	District: Nkhotakota	Target Population: TP1 - Providers/HSAs	Respondent type: Facility-based provider	Aware of SI: Yes - had heard of SI before	Coding phase: 3. Triple coded	Client marital status: N/A - provider	Client age group: N/A - provider	Time since trained: A year or more ago

Codes Applied:	P1.2.1 - Negative provider attitudes towards SI

INTERVIEWER: Okay, so have you and your friends discussed about this?

RESPONDENT: No

INTERVIEWER: You have never discussed not so? Alright you said there is nothing that has changed?

RESPONDENT: Yes

Title: MZs_HSA_5.docx

Descriptor Info:	District: Mzimba South	Target Population: TP1 - Providers/HSAs	Respondent type: HSA	Aware of SI: Yes - had heard of SI before	Coding phase: 1. Single coded	Client marital status: N/A - provider	Client age group: N/A - provider	Time since trained: A year or more ago

Codes Applied:	P1.1.1 - Positive provider attitudes towards SI	P1.2.1 - Negative provider attitudes towards SI	C1.2.2 - Fear of SI (idea or reality)

Interviewer: did you have any concerns or questions about a woman self-injecting?

Respondent: we were worried if women would manage to self-inject because most of them have fears when we inject them. So at the training we saw a woman self-inject on the abdomen we had fears that it would go deep. But after the training we saw that it was all well

Interviewer: after providing Sayana for self-injection have your perception changed? Do you still have fears?

Respondent: the fears are now gone because we waited for the feedback from women, but it was all well.

Title: MZs_HSA_5.docx

Descriptor Info:	District: Mzimba South	Target Population: TP1 - Providers/HSAs	Respondent type: HSA	Aware of SI: Yes - had heard of SI before	Coding phase: 1. Single coded	Client marital status: N/A - provider	Client age group: N/A - provider	Time since trained: A year or more ago

Codes Applied:	P1.2.1 - Negative provider attitudes towards SI	P3.1.1 - Reduced provider workload	P3.2.2 - Stockouts/stocks near expiry dates

Interviewer: thanks you so much we have come to the end of our interview. If you have anything to say, go ahead

Respondent: when Sayana was introduced we had fears that women may not manage to self-inject. We also thought they would disrespect us if they inject themselves. After using it we have seen that there is reduced workload on our side. However, Sayana is out of stock women are complaining because they have gone back to Depo IM. Some they go back without getting an alternative which is risking unwanted pregnancies.

Interviewer: thank you so much for your time. Like I said this shall be transcribed and we shall maintain confidentiality. Do you have any question?

Respondent: we got a training on Sayana but now it is out of stock.

Title: KK_HSA_3.docx

Descriptor Info:	District: Nkhotakota	Target Population: TP1 - Providers/HSAs	Respondent type: HSA	Aware of SI: Yes - had heard of SI before	Coding phase: 3. Triple coded	Client marital status: N/A - provider	Client age group: N/A - provider	Time since trained: A year or more ago

Codes Applied:	P1.2.1 - Negative provider attitudes towards SI	P1.1.1 - Positive provider attitudes towards SI

INTERVIEWER: Alright. We are still discussing. So here I am interested in your experiences of providing DMPA-SC in your community. So when you have first heard there is another injectable who…it’s still Depo right? But a woman can what, self-inject after training. What was your reaction?

RESPONDENT: As an…as an HSA, I was worried because we thought that it was an injection to sterilize women or that when a woman is injected, she will not get pregnant. I was worried and also people in the community were worried. It reached a point where people understood that it’s a good injectable although some complain of backache, legs ache, stomach ache, menstruation stops maybe for 2 months or 3 months. But those doubts are disappearing.

Title: KK_HSA_3.docx

Descriptor Info:	District: Nkhotakota	Target Population: TP1 - Providers/HSAs	Respondent type: HSA	Aware of SI: Yes - had heard of SI before	Coding phase: 3. Triple coded	Client marital status: N/A - provider	Client age group: N/A - provider	Time since trained: A year or more ago

Codes Applied:	P1.2.1 - Negative provider attitudes towards SI	P1.1.1 - Positive provider attitudes towards SI

INTERVIEWER: How about with your colleagues right, maybe with HSA’s or any colleagues, when you heard that DMPA-SC could be self-injected by a woman or an adolescent, a girl right. What did you discuss?

RESPONDENT: mmmh

INTERVIEWER: What were you discussing?

RESPONDENT: We were discussing that DMPA-SC is a very good injectable although some at first thought that it is a very dangerous and can destroy their life. But with time, they realized that it’s a very good injectable…It is a good method.

Title: KK_HSA_3.docx

Descriptor Info:	District: Nkhotakota	Target Population: TP1 - Providers/HSAs	Respondent type: HSA	Aware of SI: Yes - had heard of SI before	Coding phase: 3. Triple coded	Client marital status: N/A - provider	Client age group: N/A - provider	Time since trained: A year or more ago

Codes Applied:	P1.2.1 - Negative provider attitudes towards SI	P1.1.1 - Positive provider attitudes towards SI	C2.1.2 - Reduced trips to facility	C2.1.1 - Community availability of injectables/SI

INTERVIEWER: In its first stages before it was implemented, when you had just heard. Were you in support that Sayana should be introduced?

RESPONDENT: it was difficult for us to accept it because we did not know its end results. But after it was introduced, we saw that it was a good thing and then we accepted it.

INTERVIEWER: mmmhu. So what made you change your mind? What made you to accept it?

RESPONDENT: What made me change my mind was that I realized that Sayana is a good method because with other methods, women were walking long distances to access contraceptives. But now a lot of women see the importance of this method because they access it right there in their community, in the field.

Title: KK_HSA_3.docx

Descriptor Info:	District: Nkhotakota	Target Population: TP1 - Providers/HSAs	Respondent type: HSA	Aware of SI: Yes - had heard of SI before	Coding phase: 3. Triple coded	Client marital status: N/A - provider	Client age group: N/A - provider	Time since trained: A year or more ago

Codes Applied:	C8.1 - Male involvement, general	C0.2.2 - Negative community/client perspectives on FP	P1.2.1 - Negative provider attitudes towards SI

INTERVIEWER: What role do you think the women’s husbands or for adolescents we can say boyfriends right, their loves ones play in making a decision to take-up self-injection.

RESPONDENT: Adolescents.

INTERVIEWER: Yes, adolescents or husbands

Time stamp: (50:00).

RESPONDENT: The role they play?

INTERVIEWER: Mmmmh. What role do they take…what role do you think the women’s husbands/partners play the the decision to take up Sayana?

RESPONDENT: A lot on men do not take part in this decision.

INTERVIEWER: Why?

RESPONDENT: Because when women use Sayana, sexual activity reduces. Sexual activity between the reduces and man dislike this method

Title: KK_HSA_3.docx

Descriptor Info:	District: Nkhotakota	Target Population: TP1 - Providers/HSAs	Respondent type: HSA	Aware of SI: Yes - had heard of SI before	Coding phase: 3. Triple coded	Client marital status: N/A - provider	Client age group: N/A - provider	Time since trained: A year or more ago

Codes Applied:	C8.2 - Covert/ open use of injectables/SI	C8.1 - Male involvement, general	P1.2.1 - Negative provider attitudes towards SI

INTERVIEWER: Mmmhu. How about covert use contraceptives; does it discourage clients to take up Sayana?

RESPONDENT: Covert use of method…covert

INTERVIEWER: Without the knowledge of their husbands.

RESPONDENT: Mmmmh. Yes, that is why most of them does not take up this method they opt for provider injected. Because to take the drugs home, maybe the husband will see them, and it will cause problems in their marriage.

INTERVIEWER: mmmhu.

RESPONDENT: mmmh.

INTERVIEWER: how about…

RESPONDENT: The women want to take up the method but because they are afraid of their husbands that when they see this, there will be chaos.

INTERVIEWER: Mmmhu. You have said that there is reduced sexual activity, aaa aome they…

RESPONDENT: The body becomes weak.

Title: KK_HSA_4.docx

Descriptor Info:	District: Nkhotakota	Target Population: TP1 - Providers/HSAs	Respondent type: HSA	Aware of SI: Yes - had heard of SI before	Coding phase: 1. Single coded	Client marital status: N/A - provider	Client age group: N/A - provider	Time since trained: A year or more ago

Codes Applied:	P1.2.1 - Negative provider attitudes towards SI

INTERVIEWER: Thank you so much, I would like now to understand something. I am interested in your experience of providing DMPA-SC in this community. So here comes the question; when you and your colleagues first heard that DMPA-SC, could be injected by women themselves, what was your reaction? And has the opinion of you/your colleagues on self-injection changed over time?

RESPONDENT: Ok, That’s very true the very first time I heard about Sayana was during the training. And the first thing that came into my mind was can someone be able to inject herself?

INTERVIEWER: Ok

RESPONDENT: The other thing that bothered me the most was on storage. I was thinking if we give clients the medicine will they not inject themselves after its damaged. Because I could compare the way we store medicines here at the hospital and how a client would store at home.

INTERVIEWER: That’s very true

Title: KK_HSA_4.docx

Descriptor Info:	District: Nkhotakota	Target Population: TP1 - Providers/HSAs	Respondent type: HSA	Aware of SI: Yes - had heard of SI before	Coding phase: 1. Single coded	Client marital status: N/A - provider	Client age group: N/A - provider	Time since trained: A year or more ago

Codes Applied:	P1.2.1 - Negative provider attitudes towards SI

INTERVIEWER: Alright, have you and your colleagues discussed this? After hearing about this

RESPONDENT: Yes, we discussed about this, that self-injection is not an easy thing. Furthermore, we were considering that women are used to be injected by the provider, so will she manage to do that if we tell to do so? Another thing we were looking into is that women generally are not a strong character. So will they manage to be strong enough to inject themselves. Those are some of the things we were discussing.

INTERVIEWER: Alright

RESPONDENT: Sure

Title: MZs_HSA_4.docx

Descriptor Info:	District: Mzimba South	Target Population: TP1 - Providers/HSAs	Respondent type: HSA	Aware of SI: Yes - had heard of SI before	Coding phase: 1. Single coded	Client marital status: N/A - provider	Client age group: N/A - provider	Time since trained:

Codes Applied:	P1.2.1 - Negative provider attitudes towards SI	C1.1.1 - Positive client attitudes to SI	C1.2.2 - Fear of SI (idea or reality)	P3.5 - Data capture, general

Q5. Interviewer: I would like to hear about your experiences when offering Sayana in this area. When you heard about the self-injection method what was your reaction?

Respondent: what I thought of first is how this development will be received by the client. This was so because, most people are afraid of being injected, as such how would they feel of self-injection? Thus, I had doubts that self-injection will be effective.

Title: ZA_FAC_1.docx

Descriptor Info:	District: Zomba	Target Population: TP1 - Providers/HSAs	Respondent type: Facility-based provider	Aware of SI: Yes - had heard of SI before	Coding phase: 2. Double coded	Client marital status: N/A - provider	Client age group: N/A - provider	Time since trained: Less than a year ago

Codes Applied:	P1.2.1 - Negative provider attitudes towards SI

Interviewer: I'm interested in your experiences when providing DMPA-SC, what was your reaction when you first head that women can self-inject?

Respondent: okay can you come again

Interviewer: okay I wanted to know about your first time hearing of the possibility that women can self-inject, how did you feel?

Respondent: okay I had resistance

Interviewer : mmmm (showing that one is following the conversation)

Respondent: especially on my side because I started thinking of what can happen since an injection is an injection

Interviewer: mmmm (showing that one is following the conversation)

Respondent: we talk of the side effects that may come, there is pain, there is redness, we know that when an injection is getting on the body there are some things that happen

Interviewer: mmmm (showing that one is following the conversation)

Respondent: so I was thinking of a typical woman, she has gone back home behind that mountain to self-inject

Interviewer: mmmm (showing that one is following the conversation)

Respondent: what is happening, for some of them they even require an anesthesia when we want to inject them

Interviewer: yah yah

Respondent: so to me I had some resistance at the beginning after knowing that a woman can self-inject

Interviewer: mmmm (showing that one is following the conversation)

Respondent: it is different from the drug addicts those ones are addicted

Interviewer: mmmm (showing that one is following the conversation)

Respondent: they don't have the fear

Interviewer: yah yah

Respondent: for the women I thought there would be some resistance, if a provider like me had some resistance on the first time of hearing.

Interviewer: mmmm (showing that one is following the conversation)

Respondent: I also knew that for the women there could be resistance

Title: ZA_FAC_2.docx

Descriptor Info:	District: Zomba	Target Population: TP1 - Providers/HSAs	Respondent type: Facility-based provider	Aware of SI: Yes - had heard of SI before	Coding phase: 3. Triple coded	Client marital status: N/A - provider	Client age group: N/A - provider	Time since trained: Less than a year ago

Codes Applied:	C3.1.2 - Confidence/comfort to inject after counselling/ training	P4.1.3 - Provider availability/ willingness to repeat SI training	P1.2.1 - Negative provider attitudes towards SI	P3.1.2 - Provider skill/ability to effectively counsel on SI

Interviewer: Do women have any fears concerning their ability to apply the drug themselves?

Respondent: Yes, some are worried. They say, “Will I manage this? Will I not get pregnant? What if I get lost when administering the drug?” these things make us providers ask questions like, “will these people really manage to do this on their own since they are doubting?”, “Do they really qualify to be given the drugs so as to administer them on their own?”. So here is where we sense this. But when the person seems to understand and is able to explain the things well, then we’re sure that this person will do it properly and it will be effective.

Interviewer: So how do you deal with these worries? I’ve shown you that am worried and unable to administer the self-injection on my own, how do you deal with it?

Respondent: We re-do the teaching and try to find out why the fears of self-injecting are coming? So they respond, for example, “the way the bottle is supposed to be held..., or maybe on the stomach the place where am supposed to inject how do I do it?”. So we re-explain the process and how they’re supposed to do it. We ask, “is it clear?”, “is there a question?”

Title: KK_HSA_2.docx

Descriptor Info:	District: Nkhotakota	Target Population: TP1 - Providers/HSAs	Respondent type: HSA	Aware of SI: Yes - had heard of SI before	Coding phase: 2. Double coded	Client marital status: N/A - provider	Client age group: N/A - provider	Time since trained: A year or more ago

Codes Applied:	P1.2.1 - Negative provider attitudes towards SI	C1.2.2 - Fear of SI (idea or reality)

INTERVIEWER: Alright fine, aaa we are now moving to another topic. Am interested to know your experiences of providing DMPA-SC, at the facility hence the following question is, when you/your colleagues first heard that DMPA-SC, could be injected by women themselves, what was your reaction? When you first heard about this, what was your reaction. And has the opinion of you/your colleges on self injection changed over time. The first time you heard that women will be injecting DMPA-SA themselves how did you react. I understand to heard about this a year ago, so has your opinion changed.

RESPONDENT: aaa When I first heard, I was really worried, my worry was mainly about community as to whether they would welcome the idea since they are used to have health personnel injecting them. But now for them to be injecting themselves it was something tough.

INTERVIEWER: Mhhhh Mhhh now what is your opinion?

RESPONDENT: My opinion is that they should bring a different method other than this one.

INTERVIEWER: They should bring a new method?

RESPONDENT: Yes another method

INTERVIEWER: What type of method would it be, since we already have Depo where women are injected by health personnel and this one woman are supposed to inject themselves?

RESPONDENT: Yes, the only problem is fear, that most of them are afraid to inject themselves very few of them are so courageous to inject themselves.

INTERVIEWER: Have you and your colleagues discussed about your reaction and opinion on this?

RESPONDENT: aaa no we have never discussed

Title: KK_HSA_2.docx

Descriptor Info:	District: Nkhotakota	Target Population: TP1 - Providers/HSAs	Respondent type: HSA	Aware of SI: Yes - had heard of SI before	Coding phase: 2. Double coded	Client marital status: N/A - provider	Client age group: N/A - provider	Time since trained: A year or more ago

Codes Applied:	P1.2.1 - Negative provider attitudes towards SI	C1.2.2 - Fear of SI (idea or reality)

INTERVIEWER: counselling the client how has been the reaction after you explain to them that the method will be administered by themselves. Like you previously explained that when you heard about self injection method you had some reservations about community members how they would welcome the idea.

RESPONDENT: aaa they normally have fear that if the administer on their own the won’t be able to exhaust the whole medicine inside the injection

Title: KK_HSA_2.docx

Descriptor Info:	District: Nkhotakota	Target Population: TP1 - Providers/HSAs	Respondent type: HSA	Aware of SI: Yes - had heard of SI before	Coding phase: 2. Double coded	Client marital status: N/A - provider	Client age group: N/A - provider	Time since trained: A year or more ago

Codes Applied:	C1.2.1 - Negative client attitudes to SI	P1.2.1 - Negative provider attitudes towards SI

INTERVIEWER: Do women have any concerns about self injection? If yes what concerns do they have and how do you manage them.

RESPONDENT: The concerns that they have is doubt, they think by administering the injection themselves they may get pregnant.

INTERVIEWER: hahaha doubting themselves.

RESPONDENT: yes doubting because they believe that if the injection is administered by the health personnel I cannot get pregnant.

Title: ZA_HSA_3.docx

Descriptor Info:	District: Zomba	Target Population: TP1 - Providers/HSAs	Respondent type: HSA	Aware of SI: Yes - had heard of SI before	Coding phase: 1. Single coded	Client marital status: N/A - provider	Client age group: N/A - provider	Time since trained: A year or more ago

Codes Applied:	P1.2.1 - Negative provider attitudes towards SI

Interviewer: Alright, do you have any fears concerning a woman being able to inject herself?

Respondent: Ahhh the fears are still there thus thinking about if the person is not going to forget what she has been taught when she arrives at home? And if she has injected herself, is she going to store the syringe in the right way? Is the story okay such that children at home will not be playing with the syringe?

Title: ZA_HSA_6.docx

Descriptor Info:	District: Zomba	Target Population: TP1 - Providers/HSAs	Respondent type: HSA	Aware of SI: Yes - had heard of SI before	Coding phase: 1. Single coded	Client marital status: N/A - provider	Client age group: N/A - provider	Time since trained:

Codes Applied:	P1.2.1 - Negative provider attitudes towards SI	P2.2 - Barriers, general/other	P2.3 - Provider training content

Interviewer: Alright. What are your thoughts on the training you received?

Respondent: ah this training that we received I can say that we should be grateful, the training was good indeed, because of course we already knew about family planning.

Interviewer: mmmm (showing that one is following the conversation)

Respondent: but the way that Sayana was…

Interviewer: mmmm (showing that one is following the conversation)

Respondent with the way the tube was, we thought it was not as effective

Interviewer: mmmm (showing that one is following the conversation)

Respondent: But when we followed the procedure properly it shows that it helped us a lot and also currently it is a reliable method.

Interviewer: Alright. Ah were there any challenges while you were receiving this training?

Respondent: ah the challenges were during the time we were doing practical. It seemed that when people injected the Sayana

Interviewer: mmmm (showing that one is following the conversation)

Respondent: there were some few drops remaining in the tube

Interviewer: mmmm (showing that one is following the conversation)

Respondent: So, to us during that time we were concerned that the dose, the correct mls that should get in the body maybe they are what?

Interviewer: they are remaining.

Respondent: they are remaining, when we finished the practical and after being taught that this is how it works…

Interviewer: but the client has received the method without any problems?

Respondent: we see that those who have accessed the method in the community there is no one who came back with a problem that I have used Sayana, and I am pregnant.

Interviewer: alright alright.

Respondent: sure

Title: ZA_HSA_6.docx

Descriptor Info:	District: Zomba	Target Population: TP1 - Providers/HSAs	Respondent type: HSA	Aware of SI: Yes - had heard of SI before	Coding phase: 1. Single coded	Client marital status: N/A - provider	Client age group: N/A - provider	Time since trained:

Codes Applied:	P1.2.1 - Negative provider attitudes towards SI	P1.1.1 - Positive provider attitudes towards SI	P3.4 - Time to counsel/train, general

Interviewer: Alright, I am curious on what you have experienced in providing self-injection contraceptive at this facility and in your community. Ah when you and your colleagues first heard about the possibility of a woman self-injecting, how did you feel?

Respondent: at first, I thought that it would be difficult.

Interviewer: mmmm (showing that one is following the conversation)

Respondent: But after we did the practical it showed that the number of women who self-inject is starting to increase gradually. But most of them were worried that since a person is self-injecting they thought that they will not be doing it right, so there still required counselling, that the trainer should have enough counselling so that when they have been trained they should get the correct information because sometimes maybe the training that we do is very fast due to a time factor, but if the ones being trained, that we have people with great skill who can also train their friends it can be helpful so that there should be no problems.

Title: ZA_HSA_6.docx

Descriptor Info:	District: Zomba	Target Population: TP1 - Providers/HSAs	Respondent type: HSA	Aware of SI: Yes - had heard of SI before	Coding phase: 1. Single coded	Client marital status: N/A - provider	Client age group: N/A - provider	Time since trained:

Codes Applied:	P1.2.1 - Negative provider attitudes towards SI

Interviewer: Alright, ah I want to hear your thoughts on what you think ah why would women chose DMPA-SC? What factors do you think would motivate women to access DMPA-SC?

Respondent: Ah as a method which has just been introduced even when we were being trained as providers.

Interviewer: mmmm (showing that one is following the conversation)

Respondent: most had the thought that of concern that ah this could be difficult yeah

Interviewer: yah

Respondent: even in the community or the people from the village on their own yeah

Interviewer: mmmm (showing that one is following the conversation)

Respondent: They could still have concerns or maybe lack some belief, that is this method effective that I should take it and go home with it?

Title: MZs_FAC_2.docx

Descriptor Info:	District: Mzimba South	Target Population: TP1 - Providers/HSAs	Respondent type: Facility-based provider	Aware of SI: Yes - had heard of SI before	Coding phase: 2. Double coded	Client marital status: N/A - provider	Client age group: N/A - provider	Time since trained:

Codes Applied:	P1.2.1 - Negative provider attitudes towards SI	C1.2.2 - Fear of SI (idea or reality)

Q5. Interviewer: When you or your colleagues first heard that DMPA–SC, could be injected by women themselves, what was your reaction?

Respondent: I thought it would be very difficult for us to convince a village woman to inject herself and on this I was very resistant

Interviewer: Mmmmmmmhhh? (like really?)

Respondent: Mmmm (yes), because when we want to inject them they say shaaaaaa…. (showing some fear) injection – injection? Meaning they don`t want to be injected, thus why I was resistant saying that will they be able to inject themselves?

Interviewer: And has the opinion of you or your colleagues changed over time?

Respondent: It has changed but not much cause others still comes for provider’s injection, so we can say there is a little change.

Interviewer: You say there is a little change?

Respondent: Mmmmmmm (yes).

Title: MZs_FAC_2.docx

Descriptor Info:	District: Mzimba South	Target Population: TP1 - Providers/HSAs	Respondent type: Facility-based provider	Aware of SI: Yes - had heard of SI before	Coding phase: 2. Double coded	Client marital status: N/A - provider	Client age group: N/A - provider	Time since trained:

Codes Applied:	P1.2.1 - Negative provider attitudes towards SI

Interviewer: Have you and your colleagues discussed this?

Respondent: Yes, we do discuss with HSA’s, saying that even though others have been able to understand it, still more we have other women who find it difficult.

Title: KK_HSA_6.docx

Descriptor Info:	District: Nkhotakota	Target Population: TP1 - Providers/HSAs	Respondent type: HSA	Aware of SI: Yes - had heard of SI before	Coding phase: 1. Single coded	Client marital status: N/A - provider	Client age group: N/A - provider	Time since trained: A year or more ago

Codes Applied:	P1.2.1 - Negative provider attitudes towards SI

INTERVIEWER: alright. I am now interested in your experience of providing DMPA-SC in your community?

RESPONDENT: mmmmh.

INTERVIEWER: When you first heard that DMPA-SC could be injected by women themselves, what was your reaction?

RESPONDENT: I was afraid. Can a woman inject herself? I was afraid that it will be difficult for them. At the same time, I thought that the government was reducing our workload. But after I went for training, I realized that it was good. Because I was thinking that women will not be able to do it, and some will lie that they have self-injected while they haven’t. I was very afraid.

INTERVIEWER: okay.

RESPONDENT: yes.

Title: MZs_FAC_1.docx

Descriptor Info:	District: Mzimba South	Target Population: TP1 - Providers/HSAs	Respondent type: Facility-based provider	Aware of SI: Yes - had heard of SI before	Coding phase: 3. Triple coded	Client marital status: N/A - provider	Client age group: N/A - provider	Time since trained: A year or more ago

Codes Applied:	P1.2.1 - Negative provider attitudes towards SI	P1.1.1 - Positive provider attitudes towards SI

Interviewer: Overall, what did you think of the training how was it- you have said it was a good one, is there anything you would like to add about the training?

Respondent: It was a good training and it was an exciting one because even to us we were somehow surprise that you can teach someone to inject herself.

Interviewer: Mmmmmmh.

Respondent: Yah, so we say aaah, but will people understand this but once we went for training we really understood that it was just a simple procedures that anyone can do and it was really exciting and everybody was excited.

Title: MZs_FAC_1.docx

Descriptor Info:	District: Mzimba South	Target Population: TP1 - Providers/HSAs	Respondent type: Facility-based provider	Aware of SI: Yes - had heard of SI before	Coding phase: 3. Triple coded	Client marital status: N/A - provider	Client age group: N/A - provider	Time since trained: A year or more ago

Codes Applied:	P1.2.1 - Negative provider attitudes towards SI

Q5. Interviewer: When you or your colleagues first heard that DMPA-SC, could be injected by women themselves, what was your reaction?

Respondent: Yah, as I said already it was a doubting one to say but can a woman inject herself since we were thinking of anybody to say how can you inject yourself because we associate with pain and the fear of an injection already. We were doubting how successful this thing will be.

Title: MZs_FAC_1.docx

Descriptor Info:	District: Mzimba South	Target Population: TP1 - Providers/HSAs	Respondent type: Facility-based provider	Aware of SI: Yes - had heard of SI before	Coding phase: 3. Triple coded	Client marital status: N/A - provider	Client age group: N/A - provider	Time since trained: A year or more ago

Codes Applied:	P1.2.1 - Negative provider attitudes towards SI	C6 - Waste management	C4 - Storage of unused units

Interviewer: Did you/do you have any concerns about this idea?

Respondent: Of self-injection?

Interviewer: Yes

Respondent: Ahhhhh… yah.. kkkkkkkkk, sometimes you are not sure how will they keep the medication at home of course we do tell them but you know the communities with different people, but sometimes we are not sure if they are going to follow exactly what we have taught them and these are the concerns we have sometimes.

Interviewer: Anything else about the concerns?

Respondent: The main issue is the storage of the medication and also may be the used injectables to say what if the children reach them or may be use and injured themselves, so those are the issues but unfortunately I haven’t heard any since we started but those are our concerns usually.

Title: KK_FAC_2.docx

Descriptor Info:	District: Nkhotakota	Target Population: TP1 - Providers/HSAs	Respondent type: Facility-based provider	Aware of SI: Yes - had heard of SI before	Coding phase: 1. Single coded	Client marital status: N/A - provider	Client age group: N/A - provider	Time since trained: A year or more ago

Codes Applied:	P1.1.1 - Positive provider attitudes towards SI	P1.2.1 - Negative provider attitudes towards SI	C1.2.2 - Fear of SI (idea or reality)

INTERVIEWER: mmmmhu. We are proceeding. Now I am interested in your experiences of providing Sayana at this facility. So when you first heard that DMPA-SC, women will be self-injecting, how did you feel?

RESPONDENT: At first, aaa I was happy by the thought of a woman injecting herself. But I was interested to know how they will be self-injecting because this is a body and an injection is very painful. Yeah, so when we were trained on how a woman can self-inject, it was interesting for us to continue this work.

INTERVIEWER: mmmmhu.

RESPONDENT: yes.

INTERVIEWER: What did you discuss with your colleagues when you heard about this?

RESPONDENT: With my colleagues, everyone was interested to go for training because we really wanted to go for the self-injection training because we were not providing this method at the facility. Everyone was interested if they could go to the training to see what happens.

INTERVIEWER: mmmmhu.

RESPONDENT: mmmh.

INTERVIEWER: What about before you knew that there will be a training, what were you discussing with your colleagues about Sayana which women will be self-injecting?

RESPONDENT: mmmmhu.

INTERVIEWER: What were you discussing?

RESPONDENT: We were discussing that with the coming in of this method, we will see what will happen, because a lot of women are afraid to self-inject before they are trained.
