# Supplementary material for: Health care provider and client experiences of counselling on depot medroxyprogesterone acetate subcutaneous (DMPA-SC) for self-injection in Malawi
Source: PLOS Glob Public Health. 2023 Nov 30;3(11):e0002057. doi: 10.1371/journal.pgph.0002057 (PMC10688740; doi:10.1371/journal.pgph.0002057)
Supplement: S1 Data — (ZIP) [file pgph.0002057.s001.zip › S2/P3.1 oth enablers of prov integration.doc]

Title: ZA_HSA_2.docx

Descriptor Info:	District: Zomba	Target Population: TP1 - Providers/HSAs	Respondent type: HSA	Aware of SI: Yes - had heard of SI before	Coding phase: 3. Triple coded	Client marital status: N/A - provider	Client age group: N/A - provider	Time since trained: Less than a year ago

Codes Applied:	P3.2.2 - Stockouts/stocks near expiry dates	P3.1 - Enablers, general/other	P2.1.1 - Refresher training/mentorship

Interviewer: Alright, are there any good things about the support that you said you received?

Respondent: Yes

Interviewer: Can you share these with me?

Respondent: There were good things, we knew why we were experiencing the Sayana stock-outs

Interviewer: mmm (showing that one is following the conversation)

Respondent: But also, when its available…

Interviewer: mmm (Showing that one is following the conversation)

Respondent: How can we administer it…

Interviewer: Alright.

Respondent: Yes, the DHO emphasized that he will take the responsibility of making sure that Sayana is available…

Interviewer: mm (Showing that one is following the conversation)

Respondent: Yes, so it seems the questions that we had were addressed by the one that represented the DHO in that meeting, they gave us an assurance that they will try to do so…

Interviewer: mmm (Showing that one is following the conversation)

Respondent: Even though it will be available in small quantities, but when they start giving us enough funding for buying Sayana.

Interviewer: mmm (showing that one is following the conversation)

Respondent: Yes, because they communicated to us that the central medical stores were not given a program that they should be bringing Sayana…

Title: KK_HSA_5.docx

Descriptor Info:	District: Nkhotakota	Target Population: TP1 - Providers/HSAs	Respondent type: HSA	Aware of SI: Yes - had heard of SI before	Coding phase: 1. Single coded	Client marital status: N/A - provider	Client age group: N/A - provider	Time since trained: A year or more ago

Codes Applied:	P2.1.1 - Refresher training/mentorship	P3.1 - Enablers, general/other

INTERVIEWER: Alright, since your initial training in DMPA-SC, have you received any follow up training, supervision visits, monitoring visits, or any other form of support specifically focused on DMPA-SC?

RESPONDENT: Yes, we have had

INTERVIEWER: You have had?

RESPONDENT: Eee supervision, aaa mentorship idachikapo

RESPONDENT: Yes, supervision and mentorship

INTERVIEWER: Okay

RESPONDENT: We have had it last year where visitors from district hospital and nurses were our mentors. We were told to hold the distribution of the contraceptives until a carrier talk was done. we were the ones providing counselling and family planning methods.

INTERVIEWER: Alright fine what were the good part of the training?

RESPONDENT: All the errors were addressed

INTERVIEWER: alright

RESPONDENT: Some areas were also looked into and addressed like lack of supplies, and delay in provision of the supplies. After this supervisory visit things changed, we do now have regular supply and also the supplies come in good tie.

INTERVIEWER: Fine

RESPONDENT: The visit enabled discovery of some issues that were not considered to be problems. During the visit we were also drilled on report writing and also, we were able to identify a focal person for the community as well as for a facility. The focal person was responsible for compilation and submission of the report

Title: MZs_HSA_1.docx

Descriptor Info:	District: Mzimba South	Target Population: TP1 - Providers/HSAs	Respondent type: HSA	Aware of SI: Yes - had heard of SI before	Coding phase: 2. Double coded	Client marital status: N/A - provider	Client age group: N/A - provider	Time since trained:

Codes Applied:	P3.1 - Enablers, general/other

Interviewer: Thanks. What do you think make women to take-up DMPA-SC for self-injection?

Respondent: I think it’s the way Sayana was made, the needle is very small and short, and they can’t feel pain compared to DEPO and I think thus the reason they end up taking the self-injection method.

Title: ZA_HSA_4.docx

Descriptor Info:	District: Zomba	Target Population: TP1 - Providers/HSAs	Respondent type: HSA	Aware of SI: Yes - had heard of SI before	Coding phase: 1. Single coded	Client marital status: N/A - provider	Client age group: N/A - provider	Time since trained: A year or more ago

Codes Applied:	P3.1 - Enablers, general/other	P4.2.2 - Lack of support from others for follow up

Interviewer: Okay thank you so much. We have reached the end of our discussion. Do you have any thoughts you can share on what we have discussed today?

Respondent: Yes. I would like those working in the health sector to be coming during outreach days to help us in talking with the women because we as HSAs we do go alone.

Title: ZA_FAC_1.docx

Descriptor Info:	District: Zomba	Target Population: TP1 - Providers/HSAs	Respondent type: Facility-based provider	Aware of SI: Yes - had heard of SI before	Coding phase: 2. Double coded	Client marital status: N/A - provider	Client age group: N/A - provider	Time since trained: Less than a year ago

Codes Applied:	P3.1 - Enablers, general/other	C1.1.3 - Awareness of DMPA-SC SI

Interviewer: What do women say when you tell them about self-injecting?

Respondent: they say that it’s not painful

Interviewer: yah yah

Respondent: at first use they will have doubts

Interviewer: mmmm (showing that one is following the conversation)

Respondent: when they come on the second visit, they are more confident.

Interviewer: oho

Respondent: so we just counsel them even if the stock is not available

Interviewer: What do you think appeals more to women about DMPA-SC?

Respondent: yah I think the community leaders whom they idolize

Interviewer: mmmm (showing that one is following the conversation)

Respondent: in the communities

Interviewer: yah yah

Respondent: in every catchment population there is a focal point whom people listen to when they communicate something.

Interviewer: mmmm (showing that one is following the conversation)

Respondent: from the experience that I have

Interviewer: mmmm (showing that one is following the conversation)

Respondent: we agreed that even if we spend one hour doing counselling, we should make sure that the group understand what we are telling them so that they can be mobilizing their communities on Sayana when they go back

Interviewer: yah yah

Respondent: upon hearing that Sayana is not painful we started encouraging the women to take it up

Interviewer: alright

Respondent: so thoroughly health education helps us to mobilize the communities to encourage each other to take up the commodity

Interviewer: mmmm (showing that one is following the conversation)

Respondent: the mobilisers make our work very easy

Title: ZA_FAC_2.docx

Descriptor Info:	District: Zomba	Target Population: TP1 - Providers/HSAs	Respondent type: Facility-based provider	Aware of SI: Yes - had heard of SI before	Coding phase: 3. Triple coded	Client marital status: N/A - provider	Client age group: N/A - provider	Time since trained: Less than a year ago

Codes Applied:	P3.1 - Enablers, general/other	P3.2.2 - Stockouts/stocks near expiry dates

Interviewer: To talk about supply, have you ever had any shortages in the supply of the self-injection medicine?

Respondent: No. We have had no shortages. The drugs are plenty.

Title: ZA_FAC_2.docx

Descriptor Info:	District: Zomba	Target Population: TP1 - Providers/HSAs	Respondent type: Facility-based provider	Aware of SI: Yes - had heard of SI before	Coding phase: 3. Triple coded	Client marital status: N/A - provider	Client age group: N/A - provider	Time since trained: Less than a year ago

Codes Applied:	C1.1.3 - Awareness of DMPA-SC SI	P3.1 - Enablers, general/other

Interviewer: In your opinion, is there anything that can help you in counselling women who use the self-injection method?

Respondent: Yes. Sometimes we use the women who already use the self-injection method. We ask, “is there anyone here who uses the self-injection method?” and some stand up and admit that they use this method. These people are asked to share their experiences as in, what challenges they are facing during their use of the method. These people act as motivation to the others, and they feel that if their friends are able to use this method then they can as well

Interviewer: Is there anything else which can help you and use to give the counselling?

Respondent: Yes, there is need to work together with the healthy officials. Since they go into communities where they sensitize the community. As such, many women show up here at the hospital.

Title: KK_HSA_2.docx

Descriptor Info:	District: Nkhotakota	Target Population: TP1 - Providers/HSAs	Respondent type: HSA	Aware of SI: Yes - had heard of SI before	Coding phase: 2. Double coded	Client marital status: N/A - provider	Client age group: N/A - provider	Time since trained: A year or more ago

Codes Applied:	P3.1 - Enablers, general/other

INTERVIEWER: Alright fine, is there anything that may be helpful for the provision of Sayana method?

RESPONDENT: That would definitely be needed especially if we could be conducting drama groups and sometimes distribution of leaflets for clients to be reading at home would be helpful.

(30MINTUES AND 35 SECONDS)

INTERVIEWER: Yes

RESPONDENT: These are so helpful

Title: ZA_HSA_6.docx

Descriptor Info:	District: Zomba	Target Population: TP1 - Providers/HSAs	Respondent type: HSA	Aware of SI: Yes - had heard of SI before	Coding phase: 1. Single coded	Client marital status: N/A - provider	Client age group: N/A - provider	Time since trained:

Codes Applied:	P3.1 - Enablers, general/other	P3.2 - Barrier, general/other	P3.4 - Time to counsel/train, general

Interviewer: alright would the counselling differ if you are meeting the clients in the community or at the facility?

Respondent: ah it is a bit different.

Interviewer: mmmm (showing that one is following the conversation)

Respondent: because when we meet them in the community, they are ready that the providers are coming.

Interviewer: mmmm (showing that one is following the conversation)

Respondent: what am saying is that past 8 AM thy have already started gathering at the place of meeting.

Interviewer: yah yah

Respondent: but that time we have not yet started off from the facility.

Interviewer: mmmm (showing that one is following the conversation)

Respondent: they wait for us until we …

Interviewer: until you get there

Respondent: meaning that they are ready, and they are just waiting for us

Interviewer: mmmm (showing that one is following the conversation)

Respondent: at the facility, even though we are at the facility due to a large number of tasks to perform you tend to find that the people become bored, they see that you have been told to do other things

Interviewer: mmmm (showing that one is following the conversation)

Respondent: and you haven’t attended to them, it is a bit different with the way it is in the community

Interviewer: alright.

Respondent: for example, in the community it is possible to find 150 women,

Interviewer: yah

Respondent: they are waiting at one place for the providers, which shows that they don’t have a problem, and when its one day I the month they are very sure that the providers will come on such such a date

Title: ZA_HSA_6.docx

Descriptor Info:	District: Zomba	Target Population: TP1 - Providers/HSAs	Respondent type: HSA	Aware of SI: Yes - had heard of SI before	Coding phase: 1. Single coded	Client marital status: N/A - provider	Client age group: N/A - provider	Time since trained:

Codes Applied:	P3.1 - Enablers, general/other

Respondent: they need enough training and counselling so that they know that this method is correct.

Interviewer: alright

Respondent: If it was also possible if we had people that in every three months or maybe six months those that have been using DMPA-SC like to help in the training

Interviewer: yah

Respondent: yes, like those who are accessing DMPA-SC in their area to help in the training so that they should give thoughts on how they see it so that the other clients should learn from them someone whom they know from the community maybe than someone from the hospital.

Title: ZA_HSA_6.docx

Descriptor Info:	District: Zomba	Target Population: TP1 - Providers/HSAs	Respondent type: HSA	Aware of SI: Yes - had heard of SI before	Coding phase: 1. Single coded	Client marital status: N/A - provider	Client age group: N/A - provider	Time since trained:

Codes Applied:	C8.1 - Male involvement, general	P3.1 - Enablers, general/other

Interviewer: What role do you think men whose wives’ access DMPA-SC need to play on the decision?

Respondent: ah currently the problem, due to the change of things, most gatherings with men in the community are very hard to happen because the men are not available.

Interviewer: okay

Respondent: and also, other procedures we think that they can be possible that when women are coming to access the services that affect their family should be coming with their husbands, but this also seems like

Interviewer: its hard

Respondent: it is hard even for pregnant women to come together with their husbands it also seems that the number is low, in every 50 women maybe only 5 are the ones that can come with their husbands, and the others will not...

Interviewer: they will not come with them

Respondent: it seems that the message from the hospital is not reaching the men, most women are the ones that are receiving the messages because when they are going to the clinic with the child

Interviewer: mmmm (showing that one is following the conversation)

Respondent: they are the ones receiving most of the messages.

Interviewer: alright

Respondent: so, I think that maybe if it could be possible that we had enough resources organizing a gathering for men only so that the messages can reach them.

Interviewer: alright

Respondent: or maybe we could use a few men, let’s say 10 men from 5 villages, every village should have 2 men, we could brief them or even train them about the methods so that they can also speak to other men in their respective villages, this could help with the number of men who have the information about contraceptives since they will know about the advantages of contraceptives.

Title: MZs_FAC_2.docx

Descriptor Info:	District: Mzimba South	Target Population: TP1 - Providers/HSAs	Respondent type: Facility-based provider	Aware of SI: Yes - had heard of SI before	Coding phase: 2. Double coded	Client marital status: N/A - provider	Client age group: N/A - provider	Time since trained:

Codes Applied:	P3.1 - Enablers, general/other

Interviewer: What specifically has your opinion changing or not changing? As you have said that your opinion has changed

Respondent: Mmmmmmm (yes)

Interviewer: What has made it to change?

Respondent: Since we have other women who has already started injecting themselves that means others will also have confidence to inject themselves and with time a lot of women will start using self-injection

Title: KK_FAC_2.docx

Descriptor Info:	District: Nkhotakota	Target Population: TP1 - Providers/HSAs	Respondent type: Facility-based provider	Aware of SI: Yes - had heard of SI before	Coding phase: 1. Single coded	Client marital status: N/A - provider	Client age group: N/A - provider	Time since trained: A year or more ago

Codes Applied:	P3.3 - Counselling/ training approach, general	P3.1 - Enablers, general/other

INTERVIEWER: In your view, what are the factors that would support a client to take up the self-injection option? What can encourage them to take up Sayana?

RESPONDENT: The first thing that can…I will say reasons because it is not just one.

INTERVIEWER: … Yes, even if its five reasons.

RESPONDENT: What would support them to take up Sayana is firstly when they have been explained to about the problems they will face when they take up Sayana. This is because what makes clients not to choose a method are side effects or problems that arise because of contraception methods. If she is not well explained to about the side effects, she just mentions anything for example my head was itching it is because of Sayana. This was happening on my body, and it is because of Sayana. The second factor that can encourage them is that there should be continuous supply of Sayana. Not that you have supply for two months and then the next month there is stock out. Because of this, instead for clients to come and ask for this method, they just say that they are always in stock out and people forget about the method.

INTERVIEWER: mmmmhu.

RESPONDENT: yeah.

Title: KK_FAC_2.docx

Descriptor Info:	District: Nkhotakota	Target Population: TP1 - Providers/HSAs	Respondent type: Facility-based provider	Aware of SI: Yes - had heard of SI before	Coding phase: 1. Single coded	Client marital status: N/A - provider	Client age group: N/A - provider	Time since trained: A year or more ago

Codes Applied:	P3.1 - Enablers, general/other

INTERVIEWER: okay. Let us assume that it is my first time to hear about Sayana or other women’s first time to hear about Sayana, what do you think can encourage them or influence them to take up this method? What can it be?

RESPONDENT: What can influence them to take up this method of contraception is a message.

INTERVIEWER: mmmmmhu.

RESPONDENT: Yeah, the first factor is a message. They should know about Sayana whether by DHO or NGO’s like you, and also explaining about this method on the radio. Also us from OPD, maternity, conducting health talk on these methods.
