# Supplementary material for: Health care provider and client experiences of counselling on depot medroxyprogesterone acetate subcutaneous (DMPA-SC) for self-injection in Malawi
Source: PLOS Glob Public Health. 2023 Nov 30;3(11):e0002057. doi: 10.1371/journal.pgph.0002057 (PMC10688740; doi:10.1371/journal.pgph.0002057)
Supplement: S1 Data — (ZIP) [file pgph.0002057.s001.zip › S2/P3.1.1 reduced provider workload.doc]

Title: ZA_HSA_2.docx

Descriptor Info:	District: Zomba	Target Population: TP1 - Providers/HSAs	Respondent type: HSA	Aware of SI: Yes - had heard of SI before	Coding phase: 3. Triple coded	Client marital status: N/A - provider	Client age group: N/A - provider	Time since trained: Less than a year ago

Codes Applied:	P1.1.1 - Positive provider attitudes towards SI	P3.1.1 - Reduced provider workload

Interviewer: What are your thoughts on the training that you received, any good things or bad things on this training?

Respondent: There were some good things.

Interviewer: mmm (Showing that one is following the conversation)

Respondent: Yes, there were some good things, that we will be relieved of some work even in the villages because starting from the time we started, people started coming in few numbers because, for example when we gave them the injection today, …

Interviewer: mmm (Showing that one is following the conversation)

Respondent: It means they will come again in 2022, a day like this one…

Title: ZA_HSA_2.docx

Descriptor Info:	District: Zomba	Target Population: TP1 - Providers/HSAs	Respondent type: HSA	Aware of SI: Yes - had heard of SI before	Coding phase: 3. Triple coded	Client marital status: N/A - provider	Client age group: N/A - provider	Time since trained: Less than a year ago

Codes Applied:	P1.1.1 - Positive provider attitudes towards SI	P3.1.1 - Reduced provider workload	P4.1.3 - Provider availability/ willingness to repeat SI training	P4.1.1 - Follow up materials for client to take home

Interviewer: Ah to women at this facility and also in this community. How did you feel when you first heard of the possibility of women self-injecting?

Respondent: I received the news well…

Interviewer: mmm (showing agreement)

Respondent: …because of this possibility I saw that the work that we have at the facility and in the communities would be reduced since the clients will be trained and will be given the responsibility that they can be able to serve themselves, they just need to come and collect the injection on the days that we have advised them to do so, since we also provide them with calendars, they inject themselves four times a year…

Interviewer: mmm (agreement)

Respondent: …when they have finished the four doses they are supposed to come, we also provide them with calendars which means that they are able to follow the schedule properly…

Interviewer: Alright

Respondent: Sure, if they were not able to follow through the instructions then they are allowed to come back so that they can seek more instruction from us on how we advised them at the beginning, then we are able to explain to them again thoroughly.

Interviewer: mmm (showing that one is following the conversation)

Respondent: Yes.

Interviewer: Alright

Respondent: …so there is reduced congestion here at the facility and also in the community where we work. The reduced congestion does not mean that people are no longer using family planning, they are using family planning, but DMPA-SC seems like a longer method of family planning since the clients are injecting themselves at home…

Title: ZA_HSA_2.docx

Descriptor Info:	District: Zomba	Target Population: TP1 - Providers/HSAs	Respondent type: HSA	Aware of SI: Yes - had heard of SI before	Coding phase: 3. Triple coded	Client marital status: N/A - provider	Client age group: N/A - provider	Time since trained: Less than a year ago

Codes Applied:	P3.1.1 - Reduced provider workload

Interviewer: Have your opinions changed after the passing of time?

Respondent: My opinions?

Interviewer: Yes, from the time that you heard, up to now, about the possibility of women to self-inject.

Respondent: No, there is nothing that has changed.

Interviewer: Alright

Respondent: Because everything is just okay.

Interviewer: mm (Showing that one is following)

Respondent: Yes, for the women instead of them finding time to come to the hospital, they can just self-inject right at home, and they can continue doing other chores, so DMPA-SC (Sayana press) is helping our clients not to travel longer distances or for more days to access family planning methods.

Interviewer: Alright.

Respondent: But when they go once and collect Sayana for use in a longer period, they will inject themselves first here at the facility and then take three more doses that they can use at home, this means that they can be able to perform their chores without disruptions.

Title: KK_HSA_1.docx

Descriptor Info:	District: Nkhotakota	Target Population: TP1 - Providers/HSAs	Respondent type: HSA	Aware of SI: Yes - had heard of SI before	Coding phase: 2. Double coded	Client marital status: N/A - provider	Client age group: N/A - provider	Time since trained: A year or more ago

Codes Applied:	P3.1.1 - Reduced provider workload	P1.1.1 - Positive provider attitudes towards SI

Interviewer: Mmmhu. So you as a provider right, how easy or difficult is it to train a woman to self-inject. To teach her so that she should be self-injecting. How difficult or easy is it?

Respondent: It is not difficult and it is one way for you as a provider to have less work.

Interviewer: mmmhu.

Respondent: Mmmhu. Because let us say it is her first time…the first time you have injected her and the second one you also have to inject her, the third, fourth. Each and every time you should be injecting her. But when you train her and you observe when she is first injecting herself, it means she has reduced your work.

Interviewer: okay

Respondent: mmmh

Title: ZA_HSA_5.docx

Descriptor Info:	District: Zomba	Target Population: TP1 - Providers/HSAs	Respondent type: HSA	Aware of SI: Yes - had heard of SI before	Coding phase: 2. Double coded	Client marital status: N/A - provider	Client age group: N/A - provider	Time since trained: Less than a year ago

Codes Applied:	P3.1.1 - Reduced provider workload	C1.1.3 - Awareness of DMPA-SC SI

Respondent: Currently our job, since women are now accepting self-injection, the acceptance rate is slow

Interviewer: Mmm (showing that one is following the conversation)

Respondent: When they see their friends doing it, they are also doing it.

Interviewer: They are trying it

Respondent: They are also trying it, yes, there are some who are still not accepting but still I know that the way am working by next year I want every woman to be self-injecting

Interviewer: Alright.

Respondent: Because we are having many clients

Interviewer: Mmm (showing that one is following the conversation)

Respondent: Who want to access family planning.

Interviewer: Mmm (showing that one is following the conversation)

Respondent: So, you would find 100 people, so you will see that some of these things are taking so much of our time in our work.

Interviewer: Mmm (showing that one is following the conversation)

Respondent: So, at the end you are finding yourself very tired.

Title: ZA_HSA_5.docx

Descriptor Info:	District: Zomba	Target Population: TP1 - Providers/HSAs	Respondent type: HSA	Aware of SI: Yes - had heard of SI before	Coding phase: 2. Double coded	Client marital status: N/A - provider	Client age group: N/A - provider	Time since trained: Less than a year ago

Codes Applied:	P3.1.1 - Reduced provider workload

Respondent: Because it reduces our workload

Interviewer: Mmm (showing that one is following the conversation)

Respondent: Like I have said that we had 160 clients.

Interviewer: Mmm (showing that one is following the conversation)

Respondent: We also have to serve 100 children

Interviewer: Mmm (showing that one is following the conversation)

Respondent: And also 100 family planning clients

Interviewer: Mmm (showing that one is following the conversation)

Respondent: And we were the two of us

Interviewer: Mmm (showing that one is following the conversation)

Respondent: So, when there are more women who self-inject

Interviewer: Eh

Respondent: Then they will just come and collect, and they will not show up again for an entire year. For them to come back, it means the gap they create we can be serving other clients.

Title: KK_HSA_5.docx

Descriptor Info:	District: Nkhotakota	Target Population: TP1 - Providers/HSAs	Respondent type: HSA	Aware of SI: Yes - had heard of SI before	Coding phase: 1. Single coded	Client marital status: N/A - provider	Client age group: N/A - provider	Time since trained: A year or more ago

Codes Applied:	C1.2.2 - Fear of SI (idea or reality)	C1.1.3 - Awareness of DMPA-SC SI	C8.1 - Male involvement, general	C2.1.2 - Reduced trips to facility	P3.1.1 - Reduced provider workload

INTERVIEWER: Would you say the trend in DMPA-S uptake for self-injection has changed over time? What factors do you think have influenced this trend?

RESPONDENT: Yes, there has been a good change, the first one being that those women who were very much afraid to inject themselves are now able to do so on their own, because of how they have been strengthened by their fellow women are doing it.

INTERVIEWER: Alright

RESPONDENT: It has reduced tension in the homes by taking away bad thought that men sed to think that their wives were coming to the hospital not for treatment or family planning but were interested in male doctors.

INTERVIEWER: [interviewer laugh]

RESPONDENT: And male HSA’s. But now most of them know that there is a new method that allows their wives to visit the hospital only once in 6 months. And if they do it’s because a child is sick, whilst in the past women used to visit the hospital regularly that caused fights in the homes. They thought the nurses or HSA’s were delaying their wives deliberately, not knowing that the counselling process takes time. But now all that is history, hence men are encouraging their wives to go for this method so that most of the times are home. This change has done good as our work load has reduced than in the past that we used to have a lot of clients at once.

INTERVIEWER: Okay

RESPONDENT: These are some of the changes that has taken pace in this community.

Title: KK_FAC_1.docx

Descriptor Info:	District: Nkhotakota	Target Population: TP1 - Providers/HSAs	Respondent type: Facility-based provider	Aware of SI: Yes - had heard of SI before	Coding phase: 3. Triple coded	Client marital status: N/A - provider	Client age group: N/A - provider	Time since trained: A year or more ago

Codes Applied:	P3.1.1 - Reduced provider workload	P1.1.1 - Positive provider attitudes towards SI

RESPONDENT: The good parts are many

INTERVIEWER: Okay

RESPONDENT: The reason is when you provide Sayana you are guaranteed to meet the client after some time.

INTERVIEWER: yes

RESPONDENT: Unlike Depo, the provider has to meet the client every three months to administer it to her. Whilst with Sayana we give the client doses you will see her again after a year. Thereby reducing work load.

Title: MZs_HSA_5.docx

Descriptor Info:	District: Mzimba South	Target Population: TP1 - Providers/HSAs	Respondent type: HSA	Aware of SI: Yes - had heard of SI before	Coding phase: 1. Single coded	Client marital status: N/A - provider	Client age group: N/A - provider	Time since trained: A year or more ago

Codes Applied:	C8.2 - Covert/ open use of injectables/SI	C8.1 - Male involvement, general	C8.3 - Discretion of injectables/SI	P3.1.1 - Reduced provider workload

Interviewer: what were the good things at the training?

Respondent: the training was good. We were taught about Sayana which is a good method for those who come to the facility behind their husbands back and those who were shy to come to the facility. it also would help to save time.

Title: MZs_HSA_5.docx

Descriptor Info:	District: Mzimba South	Target Population: TP1 - Providers/HSAs	Respondent type: HSA	Aware of SI: Yes - had heard of SI before	Coding phase: 1. Single coded	Client marital status: N/A - provider	Client age group: N/A - provider	Time since trained: A year or more ago

Codes Applied:	P1.1.1 - Positive provider attitudes towards SI	P3.1.1 - Reduced provider workload

Interviewer: now I would like to know your experience as a provider for Sayana here at the facility and at your catchment area. When you first heard about Sayana that a woman will self-inject, what was your reaction with your colleagues?

Respondent: for me I saw it as a good development because it would reduce workload since we are also involved in IMCI. So for those who gets Sayana we do not take time we just remind them and provide them for self-injection

Interviewer: what did you discuss with your workmates upon hearing about self-injection?

TIME STAMP 13:13 MIN- 20:54 MIN

Respondent: with my fellow HSA’s?

Interviewer: yes, and other workmates?

Respondent: we had discussed that it would reduce workload. So we were asking questions to nurses because we were working together

Title: MZs_HSA_5.docx

Descriptor Info:	District: Mzimba South	Target Population: TP1 - Providers/HSAs	Respondent type: HSA	Aware of SI: Yes - had heard of SI before	Coding phase: 1. Single coded	Client marital status: N/A - provider	Client age group: N/A - provider	Time since trained: A year or more ago

Codes Applied:	P1.2.1 - Negative provider attitudes towards SI	P3.1.1 - Reduced provider workload	P3.2.2 - Stockouts/stocks near expiry dates

Interviewer: thanks you so much we have come to the end of our interview. If you have anything to say, go ahead

Respondent: when Sayana was introduced we had fears that women may not manage to self-inject. We also thought they would disrespect us if they inject themselves. After using it we have seen that there is reduced workload on our side. However, Sayana is out of stock women are complaining because they have gone back to Depo IM. Some they go back without getting an alternative which is risking unwanted pregnancies.

Interviewer: thank you so much for your time. Like I said this shall be transcribed and we shall maintain confidentiality. Do you have any question?

Respondent: we got a training on Sayana but now it is out of stock.

Title: ZA_HSA_4.docx

Descriptor Info:	District: Zomba	Target Population: TP1 - Providers/HSAs	Respondent type: HSA	Aware of SI: Yes - had heard of SI before	Coding phase: 1. Single coded	Client marital status: N/A - provider	Client age group: N/A - provider	Time since trained: A year or more ago

Codes Applied:	P1.1.1 - Positive provider attitudes towards SI	P3.1.1 - Reduced provider workload	C2.1.2 - Reduced trips to facility

Interviewer: Aah in your community when you first heard about the self-injection how did you and your colleagues feel?

Respondent: I felt very good because it minimized our workload and also time spent by women coming here to get the injection. They have time to do other things because they will just inject themselves doing other things whilst coming here is time consuming.

Interviewer: Has your opinion or your colleagues on self-injection changed over time?

Respondent: What kind of change?

Interviewer: You said that you accepted this method and it’s good, did your thoughts change since administering this injection?

Respondent: No, they did not change

Title: KK_HSA_4.docx

Descriptor Info:	District: Nkhotakota	Target Population: TP1 - Providers/HSAs	Respondent type: HSA	Aware of SI: Yes - had heard of SI before	Coding phase: 1. Single coded	Client marital status: N/A - provider	Client age group: N/A - provider	Time since trained: A year or more ago

Codes Applied:	P1.1.1 - Positive provider attitudes towards SI	P3.1.1 - Reduced provider workload	C2.1.2 - Reduced trips to facility	P2.1.2 - Sufficient original provider training

INTERVIEWER: Alright thank you so much. To continue with our conversation, I would like to ask you some questions about your experience with being trained in DMPA-SC. The first question on this topic is; may you please tell me a bit about the training you received in providing DMPA-SC? Overall what did you think of the training?

RESPONDENT: From the time we received the training in DMPA-SC for self-injection

INTERVIEWER: Yes

RESPONDENT: In would say that after the training I felt it was so good that I did undergone such training because as an HSA, it meant that I have added knowledge in another type family planning method. Secondly, I also considered the distance that most clients travel to come and meet me just to take contraceptives. Furthermore, the fact that the method do require clients to take some more units home to use afterwards, I felt this will do me good since my work load will reduce because I won’t be meeting clients regularly as before.

Title: MZs_HSA_4.docx

Descriptor Info:	District: Mzimba South	Target Population: TP1 - Providers/HSAs	Respondent type: HSA	Aware of SI: Yes - had heard of SI before	Coding phase: 1. Single coded	Client marital status: N/A - provider	Client age group: N/A - provider	Time since trained:

Codes Applied:	P3.1.1 - Reduced provider workload

Respondent: Further, when the clients have been taught how to self-inject, it lessens our job when they come back again since it is like revision of what we have already taught them

Title: MZs_HSA_4.docx

Descriptor Info:	District: Mzimba South	Target Population: TP1 - Providers/HSAs	Respondent type: HSA	Aware of SI: Yes - had heard of SI before	Coding phase: 1. Single coded	Client marital status: N/A - provider	Client age group: N/A - provider	Time since trained:

Codes Applied:	P2.2.1 - Lack of/insufficient refresher training/mentorship	P3.1.1 - Reduced provider workload	P3.2.2 - Stockouts/stocks near expiry dates

Interviewer: what are your thoughts on this training?

Respondent: my view is that quite some time has passed since we were trained as such, we may forget some elements. Therefore, there is need for fresher courses because our job concerns the health life of human beings. I also would like to urge the providers to make accessible the Sayana which is currently out of stock. This is so because when clients use Sayana it lessens our job.

Title: MZs_HSA_4.docx

Descriptor Info:	District: Mzimba South	Target Population: TP1 - Providers/HSAs	Respondent type: HSA	Aware of SI: Yes - had heard of SI before	Coding phase: 1. Single coded	Client marital status: N/A - provider	Client age group: N/A - provider	Time since trained:

Codes Applied:	P3.1.1 - Reduced provider workload	P2.1.2 - Sufficient original provider training

Interviewer: what are the benefits of this training?

Respondent: this training has helped us to make the client easily understand on how to use the method. In so doing our job is lessened and it reduces queues. we also have gained knowledge of the method since before we were not aware of the method. Further, we were given an allowance which was helpful.

Title: ZA_FAC_1.docx

Descriptor Info:	District: Zomba	Target Population: TP1 - Providers/HSAs	Respondent type: Facility-based provider	Aware of SI: Yes - had heard of SI before	Coding phase: 2. Double coded	Client marital status: N/A - provider	Client age group: N/A - provider	Time since trained: Less than a year ago

Codes Applied:	P1.1.1 - Positive provider attitudes towards SI	P3.1.1 - Reduced provider workload

Interviewer: Alright, how much DMPA-SC is accessed for self-injection at this hospital?

Respondent: if we could use a scale of out of 10

Interviewer: eh in every 10 women

Respondent: we can out it at 7

Interviewer: alright

Respondent: they access it because we give them the information

Interviewer: mmmm (showing that one is following the conversation)

Respondent: we always know that when we miss the information, then the quality of the service will be compromised.

Interviewer: yah yah

Respondent: yah because if you can see, in most of the facilities including ours.

Interviewer: mmmm (showing that one is following the conversation)

Respondent: the space is not enough; the rooms are few

Interviewer: mmmm (showing that one is following the conversation)

Respondent: so we saw that the introduction of Sayana will do us good, congestion will no longer be there

Interviewer: mmmm (showing that one is following the conversation)

Respondent: because for many women we will inject them, and we will not see them again for the next three months.

Interviewer: mmmm (showing that one is following the conversation)

Respondent: so it will be almost one year if we include the three injection units, we have given them for self-injection

Interviewer: mmmm (showing that one is following the conversation)

Respondent: so those women will just be coming to the OPD to seek treatment of diseases and they will not be coming to the family planning clinic, that means on the issue of congestion, we are relieved.

Title: ZA_FAC_2.docx

Descriptor Info:	District: Zomba	Target Population: TP1 - Providers/HSAs	Respondent type: Facility-based provider	Aware of SI: Yes - had heard of SI before	Coding phase: 3. Triple coded	Client marital status: N/A - provider	Client age group: N/A - provider	Time since trained: Less than a year ago

Codes Applied:	P2.1.2 - Sufficient original provider training	P1.1.1 - Positive provider attitudes towards SI	P3.1.1 - Reduced provider workload

Interviewer: Overall, what do you think about the training you had?

Respondent: Okay, the training was successful to me because as a provider, the way we give depo it’s quite different with the way we give Sayana press. So the training was successful to me because I learnt a lot of things, the things which I learnt there which can reduce my work load because if I teach my clients about the self-injection, they can inject themselves at their homes which will make my work to be reduced.

Interviewer: Anything else you’d like to add?

Respondent: About Sayana press?

Interviewer: About the training you received; what do you think about it?

Respondent: Okay, mmm, to my thinking, if I compare the way we do when providing depo and Sayana. I find it simple and easier to give Sayana than depo.

Title: ZA_FAC_2.docx

Descriptor Info:	District: Zomba	Target Population: TP1 - Providers/HSAs	Respondent type: Facility-based provider	Aware of SI: Yes - had heard of SI before	Coding phase: 3. Triple coded	Client marital status: N/A - provider	Client age group: N/A - provider	Time since trained: Less than a year ago

Codes Applied:	P1.1.1 - Positive provider attitudes towards SI	P3.1.1 - Reduced provider workload

Interviewer: I am curious to know about the experiences you encounter when administering the self-injection family planning method at this hospital or at an outreach. When you and your friends first found out that it was possible for females to apply the self-injection method of family planning on their own, how did you feel?

Respondent: I was happy and I accepted the idea since it means that the workload has now been reduced.

Interviewer: Okay fine. So have your thoughts towards the self-injection family planning method changed recently?

Respondent: No, they haven’t changed.

Interviewer: Everything is the same nothing has changed?

Respondent: Yes.

Interviewer: When with your friends, perhaps fellow nurses, do you discuss the Sayana press family planning method?

Respondent: Yes, we do. We also encourage each other that if the family planning method is not appearing in the register, especially the self-injectable one, we encourage each other that we should be teaching the people so that it can be seen that we are doing our job.

Title: MZs_HSA_3.docx

Descriptor Info:	District: Mzimba South	Target Population: TP1 - Providers/HSAs	Respondent type: HSA	Aware of SI: Yes - had heard of SI before	Coding phase: 3. Triple coded	Client marital status: N/A - provider	Client age group: N/A - provider	Time since trained: Less than a year ago

Codes Applied:	P1.1.1 - Positive provider attitudes towards SI	P3.1.1 - Reduced provider workload

Q.5 Interviewer: When you and your colleagues first heard that DMPA-SC could be injected by women themselves, what was your reaction?

Respondent: our reaction was we accepted that one because it could ease us instead of every three months people coming when we give them, we instruct them they can stay at home for almost a year yah! So they were easing partly our job yah!

Interviewer: anything else?

Respondent: the…way…we…were…. briefing them I should say taking tha tho the community that they should do this the clients we had to reduce our job in the way that most of them could carry to their homes but very few who could not understand they were the people we were supposed to give but those who could understand we said they will come after a year soo we were to see some people at longer period. Yah!

Interviewer: did you have any concerns about this idea?

Respondent:…. Yah that’s where we we knew that anybody is able to do what is told. So then we said if we can tell a clients they can do what if me a health worker failing to do so I have to continue yah…it give me courage.

Title: ZA_HSA_3.docx

Descriptor Info:	District: Zomba	Target Population: TP1 - Providers/HSAs	Respondent type: HSA	Aware of SI: Yes - had heard of SI before	Coding phase: 1. Single coded	Client marital status: N/A - provider	Client age group: N/A - provider	Time since trained: A year or more ago

Codes Applied:	C2.1.2 - Reduced trips to facility	P3.1.1 - Reduced provider workload

Interviewer: Alright, what are your thoughts about the training you got?

Respondent: From what I learnt, I believe Sayana press reduced the number of people in hospitals since people inject themselves at home and sometimes we give them three syringes to use themselves at home and that reduced queues in the hospital and also enhanced safety as people do not meet this prevent getting the corona virus.

Title: ZA_HSA_3.docx

Descriptor Info:	District: Zomba	Target Population: TP1 - Providers/HSAs	Respondent type: HSA	Aware of SI: Yes - had heard of SI before	Coding phase: 1. Single coded	Client marital status: N/A - provider	Client age group: N/A - provider	Time since trained: A year or more ago

Codes Applied:	P3.1.1 - Reduced provider workload

Interviewer: What were the good parts about the training you had?

Respondent: What excited me about the training is that we had less work because we taught the people how to inject themselves, that meant we had a lesser number of people to inject thus less work.

Title: ZA_HSA_3.docx

Descriptor Info:	District: Zomba	Target Population: TP1 - Providers/HSAs	Respondent type: HSA	Aware of SI: Yes - had heard of SI before	Coding phase: 1. Single coded	Client marital status: N/A - provider	Client age group: N/A - provider	Time since trained: A year or more ago

Codes Applied:	P3.1.1 - Reduced provider workload

Interviewer: Okay, how did you or your colleagues react when you first heard that it is possible for a woman inject herself a family planning injection?

Respondent: I felt great because our workload reduced, and it mean that the number of people who were coming here reduced as well because they were able to help themselves at home. The number of people who were coming here for Sayana reduced hence we were less occupied.

Title: ZA_HSA_3.docx

Descriptor Info:	District: Zomba	Target Population: TP1 - Providers/HSAs	Respondent type: HSA	Aware of SI: Yes - had heard of SI before	Coding phase: 1. Single coded	Client marital status: N/A - provider	Client age group: N/A - provider	Time since trained: A year or more ago

Codes Applied:	P3.1.1 - Reduced provider workload

Interviewer: Mhhhmmm... when you are with your friends what to you talk about concerning self-injection Sayana family planning method?

Respondent: We discuss that it is good that Sayana press was introduced because we no longer get busy giving family planning services hence it reduced our work load.

Title: ZA_HSA_6.docx

Descriptor Info:	District: Zomba	Target Population: TP1 - Providers/HSAs	Respondent type: HSA	Aware of SI: Yes - had heard of SI before	Coding phase: 1. Single coded	Client marital status: N/A - provider	Client age group: N/A - provider	Time since trained:

Codes Applied:	P1.1.1 - Positive provider attitudes towards SI	P3.1.1 - Reduced provider workload

Interviewer: When you heard about the possibility of women self-injecting, did you have any concerns?

Respondent: at first the concern was not that much

Interviewer: mmmm (showing that one is following the conversation)

Respondent: but I saw that it was a chance that we should have reduced work.

Interviewer: mmmm (showing that one is following the conversation)

Respondent: because if we were targeting to meet 100 people

Interviewer: mmmm (showing that one is following the conversation)

Respondent: and among the 100 maybe 30 people have accessed the self-injecting option

Interviewer: mmmm (showing that one is following the conversation)

Respondent: it means we will be meeting only 70 people for the whole year… which shows that some of our work has been reduced and we will be focusing on other work

Title: MZs_HSA_6.docx

Descriptor Info:	District: Mzimba South	Target Population: TP1 - Providers/HSAs	Respondent type: HSA	Aware of SI: Yes - had heard of SI before	Coding phase: 1. Single coded	Client marital status: N/A - provider	Client age group: N/A - provider	Time since trained: A year or more ago

Codes Applied:	P1.1.1 - Positive provider attitudes towards SI	P3.1.1 - Reduced provider workload	C2.1.2 - Reduced trips to facility

Interviewer: When you first heard that Sayana is for self-injection, what was your reaction with your colleagues.

Respondent: it was a good thing because we would provide a woman with Sayana for self-injection then she would not be coming to the provider frequently

Interviewer: what was your reaction before starting providing after hearing the rumors that women will self-inject

Respondent: it was a welcome development

Interviewer: did you talk with your colleagues at this facility upon hearing that women will self-inject at their homes

Respondent: we were wondering what kind of the method it was for a woman to self-inject. So we had agreed to go to the training to learn more about it.

Title: KK_HSA_6.docx

Descriptor Info:	District: Nkhotakota	Target Population: TP1 - Providers/HSAs	Respondent type: HSA	Aware of SI: Yes - had heard of SI before	Coding phase: 1. Single coded	Client marital status: N/A - provider	Client age group: N/A - provider	Time since trained: A year or more ago

Codes Applied:	P2.3 - Provider training content	P3.1.1 - Reduced provider workload	P1.1.1 - Positive provider attitudes towards SI

RESPONDENT: They trained us on how we can inject a woman and also how we can train a woman to self-inject Sayana. It was also to our advantage because they reduce our work load of injecting women. When they trained us, we understood and went into our communities training people. The women are self-injecting and now our job is just to update.

INTERVIEWER: Okay.

RESPONDENT: mmmh.

INTERVIEWER: What were the good parts of the training?

RESPONDENT: As an HSA, the good part of the training was that I learnt a lot for example how I can inject a person and how I can train them. Those were some of the things I learned.

Title: KK_HSA_6.docx

Descriptor Info:	District: Nkhotakota	Target Population: TP1 - Providers/HSAs	Respondent type: HSA	Aware of SI: Yes - had heard of SI before	Coding phase: 1. Single coded	Client marital status: N/A - provider	Client age group: N/A - provider	Time since trained: A year or more ago

Codes Applied:	P3.1.1 - Reduced provider workload

RESPONDENT: At the same time, I thought that the government was reducing our workload

Title: MZs_FAC_1.docx

Descriptor Info:	District: Mzimba South	Target Population: TP1 - Providers/HSAs	Respondent type: Facility-based provider	Aware of SI: Yes - had heard of SI before	Coding phase: 3. Triple coded	Client marital status: N/A - provider	Client age group: N/A - provider	Time since trained: A year or more ago

Codes Applied:	P1.1.1 - Positive provider attitudes towards SI	P3.1.1 - Reduced provider workload

Interviewer: Were you /are you supportive of the idea of a woman administering her own contraception?

Respondent: Yah, am for it actually.

Interviewer: Why?

Respondent: It reduces the burden to us as providers cause once we teach her once and she has gotten the content we give her this for a year which means may not report back for a year.

Interviewer: Mmmmmh

Respondent: So we know we may not have frequently visit to the hospital.

Title: ZA_HSA_1.docx

Descriptor Info:	District: Zomba	Target Population: TP1 - Providers/HSAs	Respondent type: HSA	Aware of SI: Yes - had heard of SI before	Coding phase: 1. Single coded	Client marital status: N/A - provider	Client age group: N/A - provider	Time since trained: Less than a year ago

Codes Applied:	P1.1.1 - Positive provider attitudes towards SI	P3.1.1 - Reduced provider workload

Respondent: Our thoughts are that we have to decrease the number of people who come to the hospital. Once they learn on how to self-inject, they will be able to self-inject when they go home.

Interviewer: Alright alright

Respondent: That means there won’t be long queues of people seeking family planning methods

Interviewer: You mentioned that you went for training. On the training you had, what were the good parts of the training?

Respondent: The good things we learnt?

Interviewer: Yes, what are the good things of the training

Respondent: It was training us to decongest the hospital because when the day arrives they self-inject at home.

Title: ZA_HSA_1.docx

Descriptor Info:	District: Zomba	Target Population: TP1 - Providers/HSAs	Respondent type: HSA	Aware of SI: Yes - had heard of SI before	Coding phase: 1. Single coded	Client marital status: N/A - provider	Client age group: N/A - provider	Time since trained: Less than a year ago

Codes Applied:	P1.1.1 - Positive provider attitudes towards SI	P3.1.1 - Reduced provider workload

Interviewer: Alright Alright, thank you very much. We are now moving into the third topic. I am interested in your experience of providing DMPA-SC in the community. When you and your colleagues first heard of the possibility of a woman to self-inject, what was your reaction?

Respondent: We were happy that it is now possible, queues will be shorter here at the hospital

Interviewer: Umm

Respondent: When people self-inject

Title: MZs_HSA_2.docx

Descriptor Info:	District: Mzimba South	Target Population: TP1 - Providers/HSAs	Respondent type: HSA	Aware of SI: Yes - had heard of SI before	Coding phase: 1. Single coded	Client marital status: N/A - provider	Client age group: N/A - provider	Time since trained: A year or more ago

Codes Applied:	P1.1.1 - Positive provider attitudes towards SI	P3.1.1 - Reduced provider workload	C5.1.4 - Timely injection due to not having to rely on external factors

Interviewer: what are your thoughts on the training you received

Respondent: it’s a very good development to us. Because if we teach women to self-inject Sayana she takes it home and we have time to do other things because she comes again after a year

Interviewer: mmmh (agreeing) what were the important thing of the training

Respondent: about Sayana

Interviewer: mmmmh (thinking out loud) about the training that you received that you saw

Respondent: may be its what I have already said. But Sayana is good for women who travel to South Africa because they take for the whole year. Even for the rest of the women they take about a year before we see each other again.

Title: MZs_HSA_2.docx

Descriptor Info:	District: Mzimba South	Target Population: TP1 - Providers/HSAs	Respondent type: HSA	Aware of SI: Yes - had heard of SI before	Coding phase: 1. Single coded	Client marital status: N/A - provider	Client age group: N/A - provider	Time since trained: A year or more ago

Codes Applied:	C5.1.4 - Timely injection due to not having to rely on external factors	P3.1.1 - Reduced provider workload	P1.1.1 - Positive provider attitudes towards SI

Interviewer: mmmmh (agreeing) now I would like to hear about your experiences as someone who provides Sayana Press for self-injection here at the facility and at the community. After you heard that women will self-inject what was your reaction

Respondent: I was happy because it would reduce number of clients seen

Interviewer: what did you and your colleague discuss about Sayana Press

Respondent: we saw it as a good method which would reduce workload since women would get dosage for one year at home. mmmmh (agreeing to the point)

Interviewer: what else

Respondent: it is a good method for women who travel to South Africa because they take a dose for one year

Interviewer: were you in support of the idea of women to self-inject

Respondent: yes

Title: MZs_HSA_2.docx

Descriptor Info:	District: Mzimba South	Target Population: TP1 - Providers/HSAs	Respondent type: HSA	Aware of SI: Yes - had heard of SI before	Coding phase: 1. Single coded	Client marital status: N/A - provider	Client age group: N/A - provider	Time since trained: A year or more ago

Codes Applied:	P3.2.2 - Stockouts/stocks near expiry dates	P3.1.1 - Reduced provider workload

Interviewer: so has your view about Sayana changed, earlier you mentioned that you were happy with the coming of Sayana because it would reduce workload. Now with the passing of time from the time you started providing to now, has your views changed on Sayana Press self-inject

Respondent: the change is that when we had Sayana we had reduced workload but now we see a lot of clients

Interviewer: I would like to know the proportion of women who take Sayana for self-inject at this facility

Respondent: it’s been out of stock

Interviewer: what do you mean

Respondent: because we do not have Sayana it’s been a year now without Sayana

Title: KK_FAC_2.docx

Descriptor Info:	District: Nkhotakota	Target Population: TP1 - Providers/HSAs	Respondent type: Facility-based provider	Aware of SI: Yes - had heard of SI before	Coding phase: 1. Single coded	Client marital status: N/A - provider	Client age group: N/A - provider	Time since trained: A year or more ago

Codes Applied:	P2.3 - Provider training content	P3.1.1 - Reduced provider workload	C2.1.2 - Reduced trips to facility	P3.1.5 - Ease of training clients in SI

INTERVIEWER: Aaa we are continuing. I will ask you questions on your experiences during DMPA-SC training.

RESPONDENT: mmmhu.

INTERVIEWER: Aaah, can you tell me a little bit about the training you received in providing DMPA-SC? What training did you receive?

RESPONDENT: The training that we received regarding the self-injectable is how to counsel a client. On this on the counselling also training them on how they can self-inject and also how the self-injecting kit is so that when self-injecting they should not face any problems.

INTERVIEWER: okay.

RESPONDENT: mmmh.

INTERVIEWER: Alright. So what were the good parts of the training? [someone came in and disturbed us] continue, you can continue. Yeah.

RESPONDENT: Where were we?

INTERVIEWER: I asked about, wait [he came in again]. I asked you about the good parts of the training…training about Sayana?

RESPONDENT: Okay. There are a lot of good things; firstly, the kit for injecting the Sayana drug is very interesting because it has different parts that interests when learning. The other is that through that training, it reduced our workload because when we teach them and they accept to be self-injecting, we can give them up to four doses including the one they have injected at the facility. Which means they will have fewer visits to the hospital and also we have reduced our work load.

INTERVIEWER: Okay.

RESPONDENT: mmmh.

INTERVIEWER: Mmmhu. If there could be another training for instance right, what would you like to be improved in the training?

Time stamp: (10:09)

RESPONDENT: In my view, that training was just fine.

INTERVIEWER: mmmhu.

RESPONDENT: Because after the training we came to the facility and we started providing, we did not face any problems.

INTERVIEWER: okay.

RESPONDENT: yes.

INTERVIEWER: everything was…

RESPONDENT: And the questions that people were asking we were answering them very well.
