# Supplementary material for: Health care provider and client experiences of counselling on depot medroxyprogesterone acetate subcutaneous (DMPA-SC) for self-injection in Malawi
Source: PLOS Glob Public Health. 2023 Nov 30;3(11):e0002057. doi: 10.1371/journal.pgph.0002057 (PMC10688740; doi:10.1371/journal.pgph.0002057)
Supplement: S1 Data — (ZIP) [file pgph.0002057.s001.zip › S2/P3.1.2 provider couns skill-ability.doc]

Title: ZA_HSA_2.docx

Descriptor Info:	District: Zomba	Target Population: TP1 - Providers/HSAs	Respondent type: HSA	Aware of SI: Yes - had heard of SI before	Coding phase: 3. Triple coded	Client marital status: N/A - provider	Client age group: N/A - provider	Time since trained: Less than a year ago

Codes Applied:	P3.1.2 - Provider skill/ability to effectively counsel on SI

Respondent: It’s not hard for the clients to understand this method.

Interviewer: Alright.

Respondent: And when we come to DMPA-SC we explain to them that Sayana is the same as DMPA-IM

Interviewer: Yes

Respondent: Yes, the effectiveness is the same and there is no any difference.

Interviewer: mmm (Showing that one is following the conversation)

Respondent: Yes, the days of effectiveness for DMPA-SC are the same as those for DMPA-IM, the only difference is that for DMPA-SC you have to self-inject…

Title: ZA_HSA_2.docx

Descriptor Info:	District: Zomba	Target Population: TP1 - Providers/HSAs	Respondent type: HSA	Aware of SI: Yes - had heard of SI before	Coding phase: 3. Triple coded	Client marital status: N/A - provider	Client age group: N/A - provider	Time since trained: Less than a year ago

Codes Applied:	P3.3 - Counselling/ training approach, general	P3.1.2 - Provider skill/ability to effectively counsel on SI

Interviewer: Alright, let’s assume that a woman has an interest in using the injectable contraceptive…

Respondent: mmm (showing that they are following the conversation)

Interviewer: How would you explain their options?

Respondent: Okay, we explain to them according to…, have you said only injectable contraceptive, right?

Interviewer: Yes.

Respondent: okay, aahh, we explain to them how the injectable works, yes, the other one when it’s the self-injecting one it’s the same as the other one there is no difference….

Interviewer: eh

Respondent: but maybe we just explain to them how the injectable can function in women’s bodies….

Interviewer: mmm (showing that one is following that conversation)

Respondent: its different from one client to the next.

Interviewer: Alright.

Respondent: some of the clients take the injection, and they don’t experience any menstrual cycles, for those clients we tell them that everything for them is perfect, there is no any problem.

Interviewer: Alright.

Respondent: some of the clients take the injection and they experience erratic bleeding during menstruation cycles.

Interviewer: mmm (showing that one is following the conversation)

Respondent: Yes, so we tell them that there is a problem…

Interviewer: mmm (showing that one is following the conversation)

Respondent: So, they need to come to the hospital to explain to the health worker.

Interviewer: Alright.

Respondent: Yes, so that the doctor can help them.

Interviewer: mmm (showing that one is following the conversation)

Respondent: some of them experience rapid bleeding during menstrual cycles.

Interviewer: mmm (showing that one is following the conversation)

Respondent: The same for these ones they can, they can come t the facility and seek medical attention, some of the clients who use this method experience minor issues for example, stomach-aches nearing the time that they will experience their menstrual periods, for these clients we tell them that it happens.

Interviewer: mmm (showing that one is following the conversation)

Respondent: yes, it’s the possibility that maybe this time something was about to happen, but it has not happened. …

Title: ZA_HSA_2.docx

Descriptor Info:	District: Zomba	Target Population: TP1 - Providers/HSAs	Respondent type: HSA	Aware of SI: Yes - had heard of SI before	Coding phase: 3. Triple coded	Client marital status: N/A - provider	Client age group: N/A - provider	Time since trained: Less than a year ago

Codes Applied:	P3.3 - Counselling/ training approach, general	C0.2 - Community and client perspectives on FP - general	P3.1.2 - Provider skill/ability to effectively counsel on SI

Respondent: so, most of these clients are living in the remote areas where there are people with different beliefs, some of them can advise their friends good things, and some of them can mislead their friends. Some of them say that when you inject yourself with this injection you’re no longer going to conceive. When these women get to us, we explain to them properly, we tell them that its all safe and there is not any problem with the Depo injection and Sayana press, the only difference is the way that they are packaged, yeah…

Interviewer: mmm (showing that one is following the conversation)

Respondent: Yes, but all of these are the same.

Interviewer: Alright.

Respondent: Yes so, we tell them that when they have questions or any problem, they should be coming to us before they believe what they hear from the people in their communities, we can explain to them properly…

Title: ZA_HSA_5.docx

Descriptor Info:	District: Zomba	Target Population: TP1 - Providers/HSAs	Respondent type: HSA	Aware of SI: Yes - had heard of SI before	Coding phase: 2. Double coded	Client marital status: N/A - provider	Client age group: N/A - provider	Time since trained: Less than a year ago

Codes Applied:	P3.1.3 - IEC/demo materials for counselling/training	P3.1.2 - Provider skill/ability to effectively counsel on SI

Interviewer: Alright, ah in your job of training the women in using Sayana Press, do you use any resources for example job aids like filling a condom with sugar or sand that you should use in training the women, do you use any job aids?

Respondent: We use a small tree branch yeah

Interviewer: Mmm (showing that one is following the conversation)

Respondent: So, we train them with that

Interviewer: Mmm (showing that one is following the conversation)

Respondent: How they can hold the thigh

Interviewer: Mmm (showing that one is following the conversation)

Respondent: The way they can self-inject up to the point where they can release the thigh properly, because the first time is always hard.

Interviewer: Mmm (showing that one is following the conversation)

Respondent: We have trained them and given them, you will then see that they are saying the liquid is not coming out, that time they have already injected themselves. So, we have to start explaining again that we have to activate the injection unit first and open it, so we try to illustrate that with a stick

Interviewer: Mmm (showing that one is following the conversation)

Respondent: Eh

Title: KK_HSA_5.docx

Descriptor Info:	District: Nkhotakota	Target Population: TP1 - Providers/HSAs	Respondent type: HSA	Aware of SI: Yes - had heard of SI before	Coding phase: 1. Single coded	Client marital status: N/A - provider	Client age group: N/A - provider	Time since trained: A year or more ago

Codes Applied:	P3.4 - Time to counsel/train, general	P3.1.2 - Provider skill/ability to effectively counsel on SI	P3.2 - Barrier, general/other	P4.1.3 - Provider availability/ willingness to repeat SI training

INTERVIEWER: Does the length of a training influence whether some women choose to take up self-injection?

RESPONDENT: The length of a training

INTERVIEWER: The process that takes in counselling the client does it influence whether some women choose to take up self-injection?

RESPONDENT: Yes, it does

INTERVIEWER: How does it influence?

RESPONDENT: When a client receives the right information through counselling

INTERVIEWER: Yes

RESPONDENT: Ndi pomweso amakhla ndi kuthekera kotenga

RESPONDENT: That’s when she has the urge to take the method. For instance, if the provider has counselled the clients so well in details, it becomes so easy for the clients when it comes for them to try to self-inject. But in cases where the provider does counsel the clients in a hurry without checking whether they are clear with the process. It becomes a problem for them when doing the trial injection.

INTERVIEWER: Alright

RESPONDENT: You may find some who will get the doses to take home, but because they did not understand well during counselling, they will just keep the units without using them. You may feel that thigs are fine, until when CBDA meets you to explain that your client is failing to use the injection you gave her, it’s when you discover that she just opened the unit. When asked she will tell you that the medication could not come out. Hence you start the counselling process all over again.

INTERVIEWER: Alright (Laugh)

RESPONDENT: Sure

Title: KK_FAC_1.docx

Descriptor Info:	District: Nkhotakota	Target Population: TP1 - Providers/HSAs	Respondent type: Facility-based provider	Aware of SI: Yes - had heard of SI before	Coding phase: 3. Triple coded	Client marital status: N/A - provider	Client age group: N/A - provider	Time since trained: A year or more ago

Codes Applied:	P3.1.2 - Provider skill/ability to effectively counsel on SI

INTERVIEWER: How many options would you counsel them? and in what order

RESPONDENT: When someone would like to take injectable, we explain that we do have two types of injectables. There is one that a client has to inject herself and the one that is administered by the provider. So we normally start explaining on the self-injection one that the advantage of this one is that a client who choses this method will have reduced visits to the facility because we give you doses that will last you for a year. Whilst if you take the one that is administered by the provider you need to be coming here every three months to get injected, Which means you will visit the facility four times. But for the first one the client will be home for a year.

Title: KK_FAC_1.docx

Descriptor Info:	District: Nkhotakota	Target Population: TP1 - Providers/HSAs	Respondent type: Facility-based provider	Aware of SI: Yes - had heard of SI before	Coding phase: 3. Triple coded	Client marital status: N/A - provider	Client age group: N/A - provider	Time since trained: A year or more ago

Codes Applied:	P3.1.2 - Provider skill/ability to effectively counsel on SI	P3.3 - Counselling/ training approach, general

INTERVIEWER: So you earlier explained that most women are afraid to inject themselves

RESPONDENT: Yes

INTERVIEWER: So how do you manage that?

RESPONDENT: When training the clients, we give them an example of patients with sugar disease. That these patients they do inject themselves every day morning and evening. So we counsel them that if someone is able to inject herself every day, how would you fail to inject yourself only once in three months. Hence most of the clients understands. We also explain to them that the injection used for this method is very small that cannot hurt them

INTERVIEWER: Yes

RESPONDENT: It very small hence it cannot cause much pain when used on someone’s body

Title: KK_HSA_4.docx

Descriptor Info:	District: Nkhotakota	Target Population: TP1 - Providers/HSAs	Respondent type: HSA	Aware of SI: Yes - had heard of SI before	Coding phase: 1. Single coded	Client marital status: N/A - provider	Client age group: N/A - provider	Time since trained: A year or more ago

Codes Applied:	P3.1.2 - Provider skill/ability to effectively counsel on SI

INTERVIEWER: Alright, lets proceed What in your experience, is the response when you explain to clients that they could self-inject this method.

RESPONDENT: When we explain about self-injection to most of the women,

INTERVIEWER: Yes

RESPONDENT: In the first place most of the women think it something too hard to handle.

INTERVIEWER: Ok

RESPONDENT: As such most of them would still want us to administer it to them.

INTERVIEWER: Ok

RESPONDENT: But we do not allow them because we tell them that the essence of this method is that the client should be able to do self-injection. Hence, we make sure that we encourage them to do it themselves by siting examples of some women who are also doing it and they are managing so well. We also tell them that since it’s the first time, but if they will do for the second time they will find it easy. After getting to understand that, and they try to inject themselves they would even say we were just scared to it on our own but its not as hard as we thought in the first place.

Time stamp: (40 minutes 6 seconds)

INTERVIEWER: Its easy (Both interviewer and participant laugh)

RESPONDENT: Easy

Title: MZs_HSA_4.docx

Descriptor Info:	District: Mzimba South	Target Population: TP1 - Providers/HSAs	Respondent type: HSA	Aware of SI: Yes - had heard of SI before	Coding phase: 1. Single coded	Client marital status: N/A - provider	Client age group: N/A - provider	Time since trained:

Codes Applied:	P3.1.2 - Provider skill/ability to effectively counsel on SI

Interviewer: do the clients have worries about self-injection?

Respondent: yes, they do.

Interviewer: which worries do they have and how do you deal with them?

Respondent: the clients worry about how to self-inject. However, when they learn about it the worry is solved because we explain to them that the needle is not painful and does not cause any problems. This is included in counselling.

Title: MZs_HSA_4.docx

Descriptor Info:	District: Mzimba South	Target Population: TP1 - Providers/HSAs	Respondent type: HSA	Aware of SI: Yes - had heard of SI before	Coding phase: 1. Single coded	Client marital status: N/A - provider	Client age group: N/A - provider	Time since trained:

Codes Applied:	P3.1.2 - Provider skill/ability to effectively counsel on SI

Q13. Interviewer: how simple or difficult is it to teach self-injection?

Respondent: it is difficult because we were born differently hence different understanding. Others may discourage their friends. However, as counsellors we use techniques in counselling different sets of people.

Title: ZA_FAC_1.docx

Descriptor Info:	District: Zomba	Target Population: TP1 - Providers/HSAs	Respondent type: Facility-based provider	Aware of SI: Yes - had heard of SI before	Coding phase: 2. Double coded	Client marital status: N/A - provider	Client age group: N/A - provider	Time since trained: Less than a year ago

Codes Applied:	P3.1.2 - Provider skill/ability to effectively counsel on SI	C1.2.2 - Fear of SI (idea or reality)

Respondent: so we agreed that when providing health talks, we should make sure that we emphasize and iron out every fear

Interviewer: mmmm (showing that one is following the conversation)

Respondent: the fear that is there is that they are scared that they will not be able to do it the first time, they will not know how to store it, we make sure that we understand where that fear is coming from, and we should help them to address those fears.

Title: ZA_FAC_2.docx

Descriptor Info:	District: Zomba	Target Population: TP1 - Providers/HSAs	Respondent type: Facility-based provider	Aware of SI: Yes - had heard of SI before	Coding phase: 3. Triple coded	Client marital status: N/A - provider	Client age group: N/A - provider	Time since trained: Less than a year ago

Codes Applied:	P3.3 - Counselling/ training approach, general	P3.1.2 - Provider skill/ability to effectively counsel on SI	C4 - Storage of unused units

Interviewer: Okay thanks, we are now entering another section. In this section, I want you to explain how you provide counsel for a woman who is considering to start using the self-injection method? Please explain in detail the steps you would follow in the provision of the counsel. Firstly, how would you greet a woman?

Respondent: A woman who has come to get family planning?

Interviewer: Yes.

Respondent: When they come, we welcome them, “you are welcome here, how are you?”, “am fine and you?”, “am fine as well, how may I help you?”	We move straight to that question because the women receive health education outside before they come inside to meet with the nurse one on one. So they respond, “I want to take the self-injection family planning method”. We ask them what they know about the family planning method. Some explain according to how they got it from the health education they received previously. So we just add more on that information by telling them the procedure when administering the method. We further explain to them by showing them the difference between the self-injection method which stays in the body for 3 months and the depo method. We also enlighten them that taking the self-injection family planning method does not mean they are protected from sexually transmitted diseases for example H.I.V. or syphilis or gonorrhea. We advise them to also be using condoms to avoid sexually transmitted diseases. We also show them the drugs and explain how they are supposed to administer the drugs. Precisely, they need to wash hands, open the injection package, then mix the drugs by shaking the injection for a quarter minute using the neck till the drug is properly mixed. After that we tell them to ensure that the bottle of drugs is not cracked or leaking. If for example, one changes her mind to do it later, perhaps she is busy with something, but they already shook the drug, we tell them to make sure they shake it again at the time they are ready to administer the drug. To administer, there is need to close the gap between the neck and the lid of the bottled drug. Then hold the bottle at the neck and point the needle of the injection upwards to prevent it from spilling out. The lid has to be properly closed because failure to do so makes pushing the drugs from the injection hard. Reaching this point, after removing the lid, the lid will not be put back and then the drug is administered into the body. This is done at 3 different positions in the body; the stomach, the thigh or behind the hand where there is more muscle. So we tell them that after shaking the drug, you administer where you prefer. If it’s the stomach, you will push the drug into the skin. Then you will have to hold the neck of the injection till it reaches the skin. Push the injection close to the skin till it touches the skin. Push the drug into the body little by little till the whole drugs gets into the skin. Pushing the drug at one time may cause pain when administering it. The place where the drug is administered is not supposed to be rubbed. The injection that was used can be put in a carton or a box and handed over to the responsible health official since they have the correct knowledge on how to dispose them.

Title: ZA_FAC_2.docx

Descriptor Info:	District: Zomba	Target Population: TP1 - Providers/HSAs	Respondent type: Facility-based provider	Aware of SI: Yes - had heard of SI before	Coding phase: 3. Triple coded	Client marital status: N/A - provider	Client age group: N/A - provider	Time since trained: Less than a year ago

Codes Applied:	C3.1.2 - Confidence/comfort to inject after counselling/ training	P4.1.3 - Provider availability/ willingness to repeat SI training	P1.2.1 - Negative provider attitudes towards SI	P3.1.2 - Provider skill/ability to effectively counsel on SI

Interviewer: Do women have any fears concerning their ability to apply the drug themselves?

Respondent: Yes, some are worried. They say, “Will I manage this? Will I not get pregnant? What if I get lost when administering the drug?” these things make us providers ask questions like, “will these people really manage to do this on their own since they are doubting?”, “Do they really qualify to be given the drugs so as to administer them on their own?”. So here is where we sense this. But when the person seems to understand and is able to explain the things well, then we’re sure that this person will do it properly and it will be effective.

Interviewer: So how do you deal with these worries? I’ve shown you that am worried and unable to administer the self-injection on my own, how do you deal with it?

Respondent: We re-do the teaching and try to find out why the fears of self-injecting are coming? So they respond, for example, “the way the bottle is supposed to be held..., or maybe on the stomach the place where am supposed to inject how do I do it?”. So we re-explain the process and how they’re supposed to do it. We ask, “is it clear?”, “is there a question?”

Title: MZs_HSA_3.docx

Descriptor Info:	District: Mzimba South	Target Population: TP1 - Providers/HSAs	Respondent type: HSA	Aware of SI: Yes - had heard of SI before	Coding phase: 3. Triple coded	Client marital status: N/A - provider	Client age group: N/A - provider	Time since trained: Less than a year ago

Codes Applied:	P3.1.2 - Provider skill/ability to effectively counsel on SI

Q10. Interviewer: Assuming the client was only interested in taking up an injectable option, how would you explain their options?

Respondent: about the options because there is Depo and there is Sayana. So Depo she has to get Depo if she would like to get Depo Depo will take 3 months it’s just the same like Sayana its all 3 months but there is Depo has to be taken with me as a provider she cannot get that one at home but Sayana has got an advantage of getting it home so long as she understands what I will tell her on how to get that Sayana. So that one will make her stay at home instead of coming here every 3 months but depo would like her to be coming every 3 months but Sayana will mmmmh she has to stay there for almost a year.

Title: ZA_HSA_3.docx

Descriptor Info:	District: Zomba	Target Population: TP1 - Providers/HSAs	Respondent type: HSA	Aware of SI: Yes - had heard of SI before	Coding phase: 1. Single coded	Client marital status: N/A - provider	Client age group: N/A - provider	Time since trained: A year or more ago

Codes Applied:	P3.1.2 - Provider skill/ability to effectively counsel on SI	C1.2.2 - Fear of SI (idea or reality)

Interviewer: Do women have concerns about the possibility of them injecting themselves? Respondent: They do have fears like will they inject themselves correctly? or will they inject the right place? so yes, they have fears.

Interviewer: Alright alright, How do you deal with their concerns?

Respondent: We explain to them in a way that they are able to fully understand and by counseling. All this is to help them get rid of their fears so that they can be able to inject themselves

Title: ZA_HSA_6.docx

Descriptor Info:	District: Zomba	Target Population: TP1 - Providers/HSAs	Respondent type: HSA	Aware of SI: Yes - had heard of SI before	Coding phase: 1. Single coded	Client marital status: N/A - provider	Client age group: N/A - provider	Time since trained:

Codes Applied:	P3.1.2 - Provider skill/ability to effectively counsel on SI	P3.5.1 - Trends/numbers of SI

Interviewer: alright. Alright, how has been the trend of uptake for DMPA-SC been at this hospital and also in the community let’s just say for the past three months?

Respondent: ah truth be told, when we have the resources at this facility. When we tell them that we want contraceptive methods we are going in the communities …

Interviewer: mmmm (showing that one is following the conversation)

Respondent: …we don’t have any problem if the supplies are enough.

Interviewer: alright, alright. So maybe in the past three months what can we say the uptake trend has been, for those women who use Sayana?

Respondent: we can say 40 percent of the women whom we have been meeting in the clinics.

Interviewer: mmmm (showing that one is following the conversation)

Respondent: they take Sayana

Title: MZs_FAC_2.docx

Descriptor Info:	District: Mzimba South	Target Population: TP1 - Providers/HSAs	Respondent type: Facility-based provider	Aware of SI: Yes - had heard of SI before	Coding phase: 2. Double coded	Client marital status: N/A - provider	Client age group: N/A - provider	Time since trained:

Codes Applied:	P3.1.2 - Provider skill/ability to effectively counsel on SI

Q12B. Interviewer: In your experience, what is the response when you explain to clients that they could self-inject this method?

Respondent: They show some resistant, even though we do the counselling and also show them the needle that this is very small, and it can be just the same as the way you do tattoo (phini)

Interviewer: Mmmmhh (following)

Respondent: So this one you only do it once in a month while the tattoo you do it twice or more than that and you can do it, but still more they find it difficult.

Interviewer: Alright.

Respondent: But after counselling, they end up taking the method and inject themselves and they even say that its simple.

Interviewer: So all these that you have mentioned, do you think they are the ones that makes them to go for Sayana?

Respondent: Yes

Interviewer: Why do you think so?

Respondent: This is so because the needle is small even though at first, they seem as if they cannot manage to do it but later on they say that its simple and they can do.

Title: KK_HSA_6.docx

Descriptor Info:	District: Nkhotakota	Target Population: TP1 - Providers/HSAs	Respondent type: HSA	Aware of SI: Yes - had heard of SI before	Coding phase: 1. Single coded	Client marital status: N/A - provider	Client age group: N/A - provider	Time since trained: A year or more ago

Codes Applied:	P3.1.2 - Provider skill/ability to effectively counsel on SI

INTERVIEWER: What about those with concerns about self-injection, how do you manage them?

RESPONDENT: It takes good counselling. Do you see this woman? Yes, was she injected by an HSA? No. we also tell them that the needle is small comparing with Depo Provera. When you show the the two needles, they say this is long and this small and easy just like malaria test kit. When we counsel her, they understand although they might still be afraid. When they understand on counselling, everything that is happening now id straight forward. But when they did not understand during counselling, when they go home, they come back and tell you that I made a mistake and destroyed the drug. You just know that she has failed to self-inject. We do the counselling again. we give them Sayana and watch them do what she was doing. You show them what is supposed to be done and they also have to explain to you what you explained to them. There are some women who are shy who do not want HSAs to see their thighs. But when we counsel them, they loosen up. And then they the woman does everything. She injects herself and off she goes.

Title: MZs_FAC_1.docx

Descriptor Info:	District: Mzimba South	Target Population: TP1 - Providers/HSAs	Respondent type: Facility-based provider	Aware of SI: Yes - had heard of SI before	Coding phase: 3. Triple coded	Client marital status: N/A - provider	Client age group: N/A - provider	Time since trained: A year or more ago

Codes Applied:	P3.1.2 - Provider skill/ability to effectively counsel on SI

Q 10. Interviewer: Assuming the client was only interested in talking up an injectable option, how would you explain their options?

Respondent: Okay, aaaa…once the woman has come and we have introduced to her and she is welcome and we are warm to her now we introduce to her the injectables that are available, we already know that we have Sayana which is self-injection, and we have DEPO which will be given as providers injection, so we actually introduce to the client that we have these two options – DEPO which you will be coming here every three months to get an injection, but apart from that we have self- Injection which we are going to teach you to do it yourself under our supervision cause its your first time and once we are convinced that you have done it rightly then we supply you with the rest of the drugs for a year.

Title: MZs_FAC_1.docx

Descriptor Info:	District: Mzimba South	Target Population: TP1 - Providers/HSAs	Respondent type: Facility-based provider	Aware of SI: Yes - had heard of SI before	Coding phase: 3. Triple coded	Client marital status: N/A - provider	Client age group: N/A - provider	Time since trained: A year or more ago

Codes Applied:	P3.3 - Counselling/ training approach, general	P3.1.2 - Provider skill/ability to effectively counsel on SI

Interviewer: Do women have concerns about self-injection?

Respondent: Yah there are some questions they could ask, mainly they are some think that how can I do it myself? What about the pain, so they will say may it is painful and they will not manage to complete the drug and how we are going to do it, but once we have taught they are convinced that is not as painful as we thought and also is not as difficult as we thought.

Interviewer: Mmmmh

Respondent: So, before they were many questions that we attend to but after we have taught them I think it answers them that it is just a simple procedure.

Title: MZs_FAC_1.docx

Descriptor Info:	District: Mzimba South	Target Population: TP1 - Providers/HSAs	Respondent type: Facility-based provider	Aware of SI: Yes - had heard of SI before	Coding phase: 3. Triple coded	Client marital status: N/A - provider	Client age group: N/A - provider	Time since trained: A year or more ago

Codes Applied:	P3.3 - Counselling/ training approach, general	P3.1.2 - Provider skill/ability to effectively counsel on SI	C1.1.1 - Positive client attitudes to SI

Q 17. Interviewer: In your view, what are the main factors that would support a client to take up the self-injection option with DMPA-SC?

Respondent: Okay, mainly the counselling itself may influence the client that I think I can take it, the way you have demonstrated to the client it can give confidence to the client that she can actually take it.

Interviewer: Mmmmh

Respondent: And if she has also seen others injecting themselves, I think she can also say that I can also do it.

Title: ZA_HSA_1.docx

Descriptor Info:	District: Zomba	Target Population: TP1 - Providers/HSAs	Respondent type: HSA	Aware of SI: Yes - had heard of SI before	Coding phase: 1. Single coded	Client marital status: N/A - provider	Client age group: N/A - provider	Time since trained: Less than a year ago

Codes Applied:	C1.2.2 - Fear of SI (idea or reality)	P3.1.2 - Provider skill/ability to effectively counsel on SI	P88 - Provider great quotes	P3.3.2 - Inclusion of practice opportunities for clients in training

Interviewer: Alright, do women have any concerns about self-injection?

Respondent: Concerns? Others fear wrongly injecting themselves

Interviewer: Mmmmh

Respondent: There are not worrisome concerns, fear is dominant. Once we watch them self-inject, they manage to do it

Interviewer: Alright, how do you overcome these women’s concerns. How do you remove the concerns?

Respondent: We just encourage them that nothing is impossible, and you will manage. We have closed watched you and you have now managed

Interviewer: Mmmh

Respondent: You will be able to manage

Title: ZA_HSA_1.docx

Descriptor Info:	District: Zomba	Target Population: TP1 - Providers/HSAs	Respondent type: HSA	Aware of SI: Yes - had heard of SI before	Coding phase: 1. Single coded	Client marital status: N/A - provider	Client age group: N/A - provider	Time since trained: Less than a year ago

Codes Applied:	C3.3.3 - Poor (insufficient/ judgemental) counselling/training	P3.1.2 - Provider skill/ability to effectively counsel on SI

Interviewer: In your view, what are the main factors that would discourage clients to take up the self-injection option?

Respondent: If they don’t have enough information

Interviewer: Mmmh

Respondent: If they come, we tell them that it is possible, and you will manage it

Interviewer: Alright

Respondent: If they don’t have enough information they would go back and say, “I won’t be able to self-inject”.
