# Supplementary material for: Health care provider and client experiences of counselling on depot medroxyprogesterone acetate subcutaneous (DMPA-SC) for self-injection in Malawi
Source: PLOS Glob Public Health. 2023 Nov 30;3(11):e0002057. doi: 10.1371/journal.pgph.0002057 (PMC10688740; doi:10.1371/journal.pgph.0002057)
Supplement: S1 Data — (ZIP) [file pgph.0002057.s001.zip › S2/P3.1.3+P3.2.1 prov use of IEC-demo materials.doc]

Title: ZA_HSA_2.docx

Descriptor Info:	District: Zomba	Target Population: TP1 - Providers/HSAs	Respondent type: HSA	Aware of SI: Yes - had heard of SI before	Coding phase: 3. Triple coded	Client marital status: N/A - provider	Client age group: N/A - provider	Time since trained: Less than a year ago

Codes Applied:	P3.2.1 - Lack of IEC/demo materials for counselling/training	P4.2.1 - Lack of follow up materials for client to take home

Interviewer: In this task that you have explained of counselling a client of DMPA-SC, do you use any resources that can help you do the job of counselling the women?

Respondent: It is supposed to be that way.

Interviewer: mmm (showing that one is following)

Respondent: Yes, we call them teaching aids when we are at school…

Interviewer: mmm (Showing that one is following the conversation)

Respondent: But at the moment we are supposed to have things like calendars, yeah…

Interviewer: mmm (showing agreement)

Respondent: we use calendars…

Interviewer: mmm (Showing agreement)

Respondent: But the resources to use to perform our job right now are not available…

Interviewer: Alright

Respondent: But these resources are important a lot, because most people easily understand when they are able to see the things that they are being trained in how it is operating, eh…

Title: ZA_HSA_2.docx

Descriptor Info:	District: Zomba	Target Population: TP1 - Providers/HSAs	Respondent type: HSA	Aware of SI: Yes - had heard of SI before	Coding phase: 3. Triple coded	Client marital status: N/A - provider	Client age group: N/A - provider	Time since trained: Less than a year ago

Codes Applied:	P3.2.1 - Lack of IEC/demo materials for counselling/training	P3.1.3 - IEC/demo materials for counselling/training

Interviewer: Are there any problems that you are facing when training women who take self-injection?

Respondent: There are problems, we don’t have the teaching aids to use in training the clients…

Interviewer: Alright.

Respondent: Yes

Interviewer: Alright.

Respondent: but if the teaching aids were available…

Interviewer: mmm (showing that one is following the conversation)

Respondent: Yes, so if those are available the women can be trained properly, because some of these things we just say them without demonstrating with the teaching aids like posters…

Interviewer: mmm (showing that one is following the conversation)

Respondent: Yes, we try to ask them that do you see this, do you see what this one is doing, so this is how it is done, if the calendars and posters are available to help in training the women, our work could be very easy…

Title: ZA_HSA_2.docx

Descriptor Info:	District: Zomba	Target Population: TP1 - Providers/HSAs	Respondent type: HSA	Aware of SI: Yes - had heard of SI before	Coding phase: 3. Triple coded	Client marital status: N/A - provider	Client age group: N/A - provider	Time since trained: Less than a year ago

Codes Applied:	P4.2.1 - Lack of follow up materials for client to take home	P3.2.1 - Lack of IEC/demo materials for counselling/training

Interviewer: Do you think there is anything that can help you in providing training to women who use self-injectables?

Respondent: Yes, we need the posters…

Interviewer: mmm (showing that one is following the conversation)

Respondent: Posters for training them…

Interviewer: mm (showing that one is following the conversation)

Respondent: And also, the calendars, because most of the times we don’t have the calendars. We can have the injectables but no calendars, these are the ones that we use for training clients, every client that has come, yeah…

Interviewer: mmm (showing that one is following the conversation)

Respondent: We have trained them, and they have accepted, we are supposed to provide them with a calendar so that they can be able to follow where the third month is ending on.

Interviewer: mmm (Showing that one is following the conversation)

Respondent: So, they circle where the third month ends, and she repeats the same every three months until the whole year…

Interviewer: yah

Respondent: Should end, so the calendar is very important…

Title: KK_HSA_1.docx

Descriptor Info:	District: Nkhotakota	Target Population: TP1 - Providers/HSAs	Respondent type: HSA	Aware of SI: Yes - had heard of SI before	Coding phase: 2. Double coded	Client marital status: N/A - provider	Client age group: N/A - provider	Time since trained: A year or more ago

Codes Applied:	P3.1.3 - IEC/demo materials for counselling/training	P4.1.1 - Follow up materials for client to take home

Interviewer: So when training them right, do you use any resources?

Respondent: Yes, when training them we use the calendar. On the calendar there are pictures showing how a person can do…poyenera kuyika, to open the medicine etc. below the calendar they put it there. Above it is a calendar with 12 months the way a calendar is. Below is where they put what...that. let’s say that you opened the drug, you are supposed to wash your hands, and it is like a reminder to who? The client on that…on that calendar. Right. So it happens that you have trained her, but she is a person and she forgets, if she knows how to read she is able to see that this step, I have washed my hands, mmmu. I have shaken and opened, and now I am supposed to open. After opening I should shake, and after shaking I should…I should choose the place I want to do the injection.

Interviewer: mmmh

Respondent: mmmh

Title: KK_HSA_1.docx

Descriptor Info:	District: Nkhotakota	Target Population: TP1 - Providers/HSAs	Respondent type: HSA	Aware of SI: Yes - had heard of SI before	Coding phase: 2. Double coded	Client marital status: N/A - provider	Client age group: N/A - provider	Time since trained: A year or more ago

Codes Applied:	P3.2.1 - Lack of IEC/demo materials for counselling/training

Interviewer: Is there anything that could help you with training the women in self-injection?

Respondent: Mmmh (thinking). The same issue of demonstrations. Because right now we just say hold the thigh like this. The condoms, of course we have them in supply at the health centre we asked them. But they said that sugar which makes the condom to feel like a body, they can not provide. And they said that every HSA, should find for him/herself.

Interviewer: mmmh

Respondent: yes.

Interviewer: okay

Respondent: Sure.

Title: ZA_HSA_5.docx

Descriptor Info:	District: Zomba	Target Population: TP1 - Providers/HSAs	Respondent type: HSA	Aware of SI: Yes - had heard of SI before	Coding phase: 2. Double coded	Client marital status: N/A - provider	Client age group: N/A - provider	Time since trained: Less than a year ago

Codes Applied:	P3.2.1 - Lack of IEC/demo materials for counselling/training

Interviewer: Aright, do you sue any job aids in providing counselling to the clients?

Respondent: Ah right now we don’t have the job aids, we just explain it to them verbally.

Interviewer: Alright.

Respondent: Yeah, but we need to have the job aids

Interviewer: Mmm (showing that one is following the conversation)

Respondent: So that maybe the women can be able to see and have a choice that this is the method that has appealed to me, than just telling them verbally about the methods.

Title: ZA_HSA_5.docx

Descriptor Info:	District: Zomba	Target Population: TP1 - Providers/HSAs	Respondent type: HSA	Aware of SI: Yes - had heard of SI before	Coding phase: 2. Double coded	Client marital status: N/A - provider	Client age group: N/A - provider	Time since trained: Less than a year ago

Codes Applied:	P3.1.3 - IEC/demo materials for counselling/training	P3.1.2 - Provider skill/ability to effectively counsel on SI

Interviewer: Alright, ah in your job of training the women in using Sayana Press, do you use any resources for example job aids like filling a condom with sugar or sand that you should use in training the women, do you use any job aids?

Respondent: We use a small tree branch yeah

Interviewer: Mmm (showing that one is following the conversation)

Respondent: So, we train them with that

Interviewer: Mmm (showing that one is following the conversation)

Respondent: How they can hold the thigh

Interviewer: Mmm (showing that one is following the conversation)

Respondent: The way they can self-inject up to the point where they can release the thigh properly, because the first time is always hard.

Interviewer: Mmm (showing that one is following the conversation)

Respondent: We have trained them and given them, you will then see that they are saying the liquid is not coming out, that time they have already injected themselves. So, we have to start explaining again that we have to activate the injection unit first and open it, so we try to illustrate that with a stick

Interviewer: Mmm (showing that one is following the conversation)

Respondent: Eh

Title: ZA_HSA_5.docx

Descriptor Info:	District: Zomba	Target Population: TP1 - Providers/HSAs	Respondent type: HSA	Aware of SI: Yes - had heard of SI before	Coding phase: 2. Double coded	Client marital status: N/A - provider	Client age group: N/A - provider	Time since trained: Less than a year ago

Codes Applied:	P3.1.3 - IEC/demo materials for counselling/training

Interviewer: Is there anything that can support you in providing counselling to women on DMPA-SC?

Respondent: The charts would help us a lot.

Interviewer: Alright

Respondent: So that we can show the women, not only for Sayana but even for condoms and the pills.

Interviewer: Mmm (showing that one is following the conversation)

Respondent: People believe when they see.

Interviewer: Yah yah

Respondent: Yeah, showing them how it works so that the women can have an informed choice.

Interviewer: Mmm (showing that one is following the conversation)

Respondent: Yeah

Title: KK_HSA_5.docx

Descriptor Info:	District: Nkhotakota	Target Population: TP1 - Providers/HSAs	Respondent type: HSA	Aware of SI: Yes - had heard of SI before	Coding phase: 1. Single coded	Client marital status: N/A - provider	Client age group: N/A - provider	Time since trained: A year or more ago

Codes Applied:	P3.1.3 - IEC/demo materials for counselling/training

INTERVIEWER: I would like to ask you some questions about your experience with being trained in DMPA-SC. May you please tell me a bit about the training you received in providing DMPA-SC?

RESPONDENT: WE did the training for two days

INTERVIEWER: Alright

RESPONDENT: The training was conducted soon after we had started a training on Depo, hence we were told that it was a continuation to that. I feel the training was fine since the people who were undergoing the training could easily grasp the content. This is different from the clients because most of them are full of fear.

INTERVIEWER: Ok

RESPONDENT: Sure sure though during training we use a condom full of sand as a sample for them to try how to inject but it still takes them time to get used to it to enable them do it on their own.

Title: KK_HSA_5.docx

Descriptor Info:	District: Nkhotakota	Target Population: TP1 - Providers/HSAs	Respondent type: HSA	Aware of SI: Yes - had heard of SI before	Coding phase: 1. Single coded	Client marital status: N/A - provider	Client age group: N/A - provider	Time since trained: A year or more ago

Codes Applied:	P3.1.3 - IEC/demo materials for counselling/training

INTERVIEWER: Would you use any material/ job aid?

RESPONDENT: Yes, during our counselling sessions we normally have cards, some books that have pictures of all family planning methods. When we are talking about a particular method for instance a condom we point out on the picture. When we are talking of the injectable methods, we show the picture of the set of the injection like the injection and the bottle that contains the medicine. So during counselling we normally use the counselling cards and and posters.

Title: KK_HSA_5.docx

Descriptor Info:	District: Nkhotakota	Target Population: TP1 - Providers/HSAs	Respondent type: HSA	Aware of SI: Yes - had heard of SI before	Coding phase: 1. Single coded	Client marital status: N/A - provider	Client age group: N/A - provider	Time since trained: A year or more ago

Codes Applied:	P3.1.3 - IEC/demo materials for counselling/training

INTERVIEWER: Is there anything that could help you with training clients in SI?

RESPONDENT: Yes

INTERVIEWER: Like what

RESPONDENT: Like calendars they are so helpful

INTERVIEWER: Ok

RESPONDENT: Apart from it being a calendar but the other side is full of instructions. It contains details on the steps on how self-injection should be done and also it has some pictures that help the clients to see the process. We also use the pictures when counselling clients, we tell to be checking the pictures whilst counselling them. It is only that at certain point we had the units without calendars hence some clients collected that doses without calendars.

INTERVIEWER: Ok

RESPONDENT: Those were some of the problems noted

Title: KK_HSA_5.docx

Descriptor Info:	District: Nkhotakota	Target Population: TP1 - Providers/HSAs	Respondent type: HSA	Aware of SI: Yes - had heard of SI before	Coding phase: 1. Single coded	Client marital status: N/A - provider	Client age group: N/A - provider	Time since trained: A year or more ago

Codes Applied:	P3.2.1 - Lack of IEC/demo materials for counselling/training	P3.1.3 - IEC/demo materials for counselling/training

INTERVIEWER: What about things like leaflets, reminders, re-injection, calendars. Do you have enough of these?

RESPONDENT: That’s what I just said, if we have them available, we give out to our clients. And they do help us a lot as we do or work.

INTERVIEWER: Ok

RESPONDENT: It helps especially when they are alone, they are able to refer to the instruction sheet. It becomes a problem when we have a shortfall of the calendars, sometimes we have more units than calendars and vice versa. We sometimes experience availability of units without calendar, or we may have mis-used the calendars ourselves. This makes our work a bit difficult

Title: MZs_HSA_1.docx

Descriptor Info:	District: Mzimba South	Target Population: TP1 - Providers/HSAs	Respondent type: HSA	Aware of SI: Yes - had heard of SI before	Coding phase: 2. Double coded	Client marital status: N/A - provider	Client age group: N/A - provider	Time since trained:

Codes Applied:	P3.1.3 - IEC/demo materials for counselling/training

Q13. Interviewer: How easy or difficult it is to train clients in DMPA-SC for self-injection?

Respondent: The only problem we encounter when counselling them is the resistance they show at first before self-injection but after trying they show interest, and they do like the method.

Interviewer: Do you use any resources during the counselling like condom filled with sand or sugar?

Respondent: As for me most of the times I use condom filled with sand for demonstration for us to know if they can do it.

Interviewer: What about to the adolescent? What resources do you use during counselling?

Respondent: We use the same materials for both adults as well as adolescents.

Title: MZs_HSA_1.docx

Descriptor Info:	District: Mzimba South	Target Population: TP1 - Providers/HSAs	Respondent type: HSA	Aware of SI: Yes - had heard of SI before	Coding phase: 2. Double coded	Client marital status: N/A - provider	Client age group: N/A - provider	Time since trained:

Codes Applied:	P3.2.1 - Lack of IEC/demo materials for counselling/training

Interviewer: Is there anything that could help you with training clients in SI?

Respondent: Mostly we need flip charts, cause as of now the flip charts we have there is no Sayana Press. So if they can add Sayana press in the flip chart it can be better and easy for us in training them.

Interviewer: Anything else?

Respondent: Nothing.

Title: KK_FAC_1.docx

Descriptor Info:	District: Nkhotakota	Target Population: TP1 - Providers/HSAs	Respondent type: Facility-based provider	Aware of SI: Yes - had heard of SI before	Coding phase: 3. Triple coded	Client marital status: N/A - provider	Client age group: N/A - provider	Time since trained: A year or more ago

Codes Applied:	P3.2.1 - Lack of IEC/demo materials for counselling/training

RESPONDENT: Like in what area, of course training materials for demonstration has been a problem, because we normally use a condom filled with sugar. There is nothing like tools for demonstration apart from the sugar that is filled in a condom hence illustration becomes tough.

INTERVIEWER: Okay

RESPONDENT: U cannot use it for some months you need to destroy it and make another sample

Title: KK_FAC_1.docx

Descriptor Info:	District: Nkhotakota	Target Population: TP1 - Providers/HSAs	Respondent type: Facility-based provider	Aware of SI: Yes - had heard of SI before	Coding phase: 3. Triple coded	Client marital status: N/A - provider	Client age group: N/A - provider	Time since trained: A year or more ago

Codes Applied:	P3.1.3 - IEC/demo materials for counselling/training

RESPONDENT: So I suggest that a human-like doll should be available.

INTERVIEWER:

RESPONDENT: That should have actual sites for demonstration that we can easily show the clients how things are done.

Title: KK_FAC_1.docx

Descriptor Info:	District: Nkhotakota	Target Population: TP1 - Providers/HSAs	Respondent type: Facility-based provider	Aware of SI: Yes - had heard of SI before	Coding phase: 3. Triple coded	Client marital status: N/A - provider	Client age group: N/A - provider	Time since trained: A year or more ago

Codes Applied:	P3.2.1 - Lack of IEC/demo materials for counselling/training

INTERVIEWER: Alright, for instance you would like to counsel a client how do you go about it?

RESPONDENT: Okay counselling is done, let me say that we are now sed to what to counsel our clients on

INTERVIEWER: Alright I see

RESPONDENT: Initially we used to have flip charts that have information on FP methods , but now since we do not have so we just explain to our clients on the methods available at our facility.

Title: KK_FAC_1.docx

Descriptor Info:	District: Nkhotakota	Target Population: TP1 - Providers/HSAs	Respondent type: Facility-based provider	Aware of SI: Yes - had heard of SI before	Coding phase: 3. Triple coded	Client marital status: N/A - provider	Client age group: N/A - provider	Time since trained: A year or more ago

Codes Applied:	P3.1.3 - IEC/demo materials for counselling/training

INTERVIEWER: Alright. You seem to remember a lot of stuff, in terms of materials or tools do you use them during trainings?

RESPONDENT: Yes , we do have a poster that explains family planning methods that we offer at this facility and how each method works.

Title: KK_FAC_1.docx

Descriptor Info:	District: Nkhotakota	Target Population: TP1 - Providers/HSAs	Respondent type: Facility-based provider	Aware of SI: Yes - had heard of SI before	Coding phase: 3. Triple coded	Client marital status: N/A - provider	Client age group: N/A - provider	Time since trained: A year or more ago

Codes Applied:	P3.2.1 - Lack of IEC/demo materials for counselling/training

INTERVIEWER: Alright, is there anything that would help you in training clients in SI?

RESPONDENT: Of course like Sayana, I feel it does not have enough training materials like posters

INTERVIEWER: Okay posters?

RESPONDENT: Yes, some how women find it easier when visual aids are used so that they are able to see how the methods works. Because they are able to remember easily when they see the pictures. As such I feel Sayana lacks posters.

Title: KK_FAC_1.docx

Descriptor Info:	District: Nkhotakota	Target Population: TP1 - Providers/HSAs	Respondent type: Facility-based provider	Aware of SI: Yes - had heard of SI before	Coding phase: 3. Triple coded	Client marital status: N/A - provider	Client age group: N/A - provider	Time since trained: A year or more ago

Codes Applied:	P3.1.3 - IEC/demo materials for counselling/training

INTERVIEWER: We have reached the end of our discussions that we had. But I know you may have questions or any other information you would like to share on the topics we have discussed, or there may be some questions you feel I did not ask and you want to say something especially on Sayana method? You may do so.

RESPONDENT: I would like to say something on the issue of posters

INTERVIEWER: Yes

RESPONDENT: Those things assist us as we give out information to our clients so that it’s easy to understand it.

INTERVIEWER: Ok

RESPONDENT: Its different from one to be told we inject on the thigh, but at least if we have a real sample where one is able to

INTERVIEWER: Yes

RESPONDENT: If there can be that provision to be available in the facilities

Title: MZs_HSA_5.docx

Descriptor Info:	District: Mzimba South	Target Population: TP1 - Providers/HSAs	Respondent type: HSA	Aware of SI: Yes - had heard of SI before	Coding phase: 1. Single coded	Client marital status: N/A - provider	Client age group: N/A - provider	Time since trained: A year or more ago

Codes Applied:	P3.1.3 - IEC/demo materials for counselling/training

Interviewer: you have talked of the demo pen, what do you use when counselling about Sayana?

Respondent: we use card and a calendar

Interviewer: what else?

Respondent: counselling card

Title: MZs_HSA_5.docx

Descriptor Info:	District: Mzimba South	Target Population: TP1 - Providers/HSAs	Respondent type: HSA	Aware of SI: Yes - had heard of SI before	Coding phase: 1. Single coded	Client marital status: N/A - provider	Client age group: N/A - provider	Time since trained: A year or more ago

Codes Applied:	P3.1.3 - IEC/demo materials for counselling/training

Interviewer: what would assist you in the counselling of a woman on Sayana Press?

Respondent: if we would have flipcharts and a special card for Sayana because the one we use has all methods of family planning. So for us to emphasize we need a special card for Sayana so that women understand better.

Title: KK_HSA_3.docx

Descriptor Info:	District: Nkhotakota	Target Population: TP1 - Providers/HSAs	Respondent type: HSA	Aware of SI: Yes - had heard of SI before	Coding phase: 3. Triple coded	Client marital status: N/A - provider	Client age group: N/A - provider	Time since trained: A year or more ago

Codes Applied:	P3.2.1 - Lack of IEC/demo materials for counselling/training

INTERVIEWER: mmhu. job aids during counselling?

RESPONDENT: Job aids…we don’t use any materials…any materials but what has been provided to us, what we have like Sayana and Depo.

Title: KK_HSA_3.docx

Descriptor Info:	District: Nkhotakota	Target Population: TP1 - Providers/HSAs	Respondent type: HSA	Aware of SI: Yes - had heard of SI before	Coding phase: 3. Triple coded	Client marital status: N/A - provider	Client age group: N/A - provider	Time since trained: A year or more ago

Codes Applied:	P3.1.3 - IEC/demo materials for counselling/training	P3.2.1 - Lack of IEC/demo materials for counselling/training

INTERVIEWER: So is there anything that could help you in training/counselling the women in SI? Is there anything that you feel can help when conducting the training?

RESPONDENT: [paused he was thinking about the question] When counselling them? What can help are maybe posters.

INTERVIEWER: okay.

RESPONDENT: Mmmmmhu. The posters help when explaining. They are able to see the stages you are in your explanation. Another thing are audios right? You have to record the explanations and follow that because you get tired when you are the only one who is speaking.

INTERVIEWER: Okay, you want an availability of an audio?

RESPONDENT: mmmh.

Title: KK_HSA_3.docx

Descriptor Info:	District: Nkhotakota	Target Population: TP1 - Providers/HSAs	Respondent type: HSA	Aware of SI: Yes - had heard of SI before	Coding phase: 3. Triple coded	Client marital status: N/A - provider	Client age group: N/A - provider	Time since trained: A year or more ago

Codes Applied:	P3.1.3 - IEC/demo materials for counselling/training

INTERVIEWER: Mmmhu. Let’s say that you are…you have told me that you train about 6 or 7 women for 2hours right? How easy or difficult is it for a client to understand when she is supposed to do (self-injection process)?

RESPONDENT: We do practicals

INTERVIEWR: okay.

RESPONDENT: mmmmh.

INTERVIEWER: you practice

RESPONDENT: we practice for example we use a certain sample…

INTERVIEWER: Mmmh. What is sample? mmmh

RESPONDENT: We use sugar and put it in a certain tube, right? We put sugar in that plastic paper and then we practice by injecting that plastic. And all the women see that. The sample helps us because they have first to try on that sample and thereafter, they have to try on themselves. And then we compare the sample and how they are injecting themselves and see where they are doing wrong, and we help them to improve on that. And the ones who have passed we congratulate them and tell them to continue at home. But for the ones who have failed, we tell them.

Title: ZA_HSA_4.docx

Descriptor Info:	District: Zomba	Target Population: TP1 - Providers/HSAs	Respondent type: HSA	Aware of SI: Yes - had heard of SI before	Coding phase: 1. Single coded	Client marital status: N/A - provider	Client age group: N/A - provider	Time since trained: A year or more ago

Codes Applied:	P3.1.5 - Ease of training clients in SI	P3.2.1 - Lack of IEC/demo materials for counselling/training

Interviewer: Ohh alright fine. How is difficult or (not difficult) to train the women on the self-injecting method?

Respondent: It is not difficult. It’s just that the women have fears because we just tell them how they are supposed to be doing it step by step.

Interviewer: Alright alright. Do you use any other resources for example those that help condoms full of sugar or sand to make the women easily understand what is being said?

Respondent: I have never done that before using the condom, I just explain the things to them.

Title: KK_HSA_4.docx

Descriptor Info:	District: Nkhotakota	Target Population: TP1 - Providers/HSAs	Respondent type: HSA	Aware of SI: Yes - had heard of SI before	Coding phase: 1. Single coded	Client marital status: N/A - provider	Client age group: N/A - provider	Time since trained: A year or more ago

Codes Applied:	P3.1.3 - IEC/demo materials for counselling/training

INTERVIEWER: Is there anything that could help you with training clients in SI?

RESPONDENT: Yes

INTERVIEWER: Like what?

RESPONDENT: What helps us the most are like health talks, because it shows that,

INTERVIEWER: Alright.

RESPONDENT: Because though women may come for outpatient services, we still do health talks on family planning methods.

INTERVIEWER: Alright

RESPONDENT: WE also feel that the one who came up with an idea of introducing the calendar did a commendable job because on the other side of the calendar we do have instructions So for those women who are able to read they use that information at home on their own to remind themselves of what we were counselling them .

INTERVIEWER: Yes

RESPONDENT: Sure

Title: MZs_HSA_4.docx

Descriptor Info:	District: Mzimba South	Target Population: TP1 - Providers/HSAs	Respondent type: HSA	Aware of SI: Yes - had heard of SI before	Coding phase: 1. Single coded	Client marital status: N/A - provider	Client age group: N/A - provider	Time since trained:

Codes Applied:	P3.1.3 - IEC/demo materials for counselling/training

Respondent: … in teaching them self-injection, we demonstrate to the client how to do the injection so that the client should know that it is possible and advantageous for them to self-inject.

Title: MZs_HSA_4.docx

Descriptor Info:	District: Mzimba South	Target Population: TP1 - Providers/HSAs	Respondent type: HSA	Aware of SI: Yes - had heard of SI before	Coding phase: 1. Single coded	Client marital status: N/A - provider	Client age group: N/A - provider	Time since trained:

Codes Applied:	P3.1.3 - IEC/demo materials for counselling/training

Interviewer: do you use any aids in explaining these methods to the clients for better understanding?

Respondent: yes, I use materials such as condoms and flip charts. In explaining about Sayana I take a condom and put sand in it.

Interviewer: how about to the youth?

Respondent: we also use the same material.

Interviewer: Mmmmh

Respondent: yes.

Title: MZs_HSA_4.docx

Descriptor Info:	District: Mzimba South	Target Population: TP1 - Providers/HSAs	Respondent type: HSA	Aware of SI: Yes - had heard of SI before	Coding phase: 1. Single coded	Client marital status: N/A - provider	Client age group: N/A - provider	Time since trained:

Codes Applied:	P4.1.1 - Follow up materials for client to take home	P3.2.1 - Lack of IEC/demo materials for counselling/training

Interviewer: thank you. Q15. Interviewer: when the client has chosen self-injection do you offer any additional services?

Respondent: No. we only provide calendars.

Interviewer: are the calendars enough?

Respondent: no.

Interviewer: are the clients comfortable in receiving the calendars?

Respondent: some clients refuse to receive the calendars because they are afraid that their husbands may see it.

Title: ZA_FAC_1.docx

Descriptor Info:	District: Zomba	Target Population: TP1 - Providers/HSAs	Respondent type: Facility-based provider	Aware of SI: Yes - had heard of SI before	Coding phase: 2. Double coded	Client marital status: N/A - provider	Client age group: N/A - provider	Time since trained: Less than a year ago

Codes Applied:	P3.1.3 - IEC/demo materials for counselling/training

Interviewer: Alright, do you use any resources when training women in DMPA-SC?

Respondent: yes

Interviewer: Alright what resources?

Respondent: Calendars

Interviewer: yah

Respondent: we make sure to have the calendars and we also use some of the methods as resources.

Title: ZA_FAC_1.docx

Descriptor Info:	District: Zomba	Target Population: TP1 - Providers/HSAs	Respondent type: Facility-based provider	Aware of SI: Yes - had heard of SI before	Coding phase: 2. Double coded	Client marital status: N/A - provider	Client age group: N/A - provider	Time since trained: Less than a year ago

Codes Applied:	P3.1.3 - IEC/demo materials for counselling/training	P4.1.1 - Follow up materials for client to take home

Interviewer: Do you use any resources in giving counselling on DMPA-SC?

Respondent: yah apart from the methods

Interviewer: mmmm (showing that one is following the conversation)

Respondent: we are supposed to use the methods, the Sayana units, calendars

Interviewer: mmmm (showing that one is following the conversation)

Respondent: calendars are for wen they inject themselves to circle their dates

Interviewer: mmmm (showing that one is following the conversation)

Respondent: we have the calendars, so we show them maybe they don’t know how to write so we help them out

Interviewer: mmmm (showing that one is following the conversation)

Respondent: eh those things are supposed to be there

Interviewer: alright

Respondent: eh

Interviewer: What about other resources like filling a condom with sand or sugar, do you use those resources?

Respondent: yes, we use that in the health talk demonstrations

Interviewer: mmmm (showing that one is following the conversation)

Respondent: eh we are supposed to demonstrate how the person self-injects.

Interviewer: mmmm (showing that one is following the conversation)

Respondent: we don’t do the demonstration on the woman but on an object, an object like filling up a condom with sand and then inject it, but the women play down that demonstration.

Interviewer: mmmm (showing that one is following the conversation)

Respondent: so we then say that I can demonstrate this on one woman and the woman will give feedback to the others.

Interviewer: mmmm (showing that one is following the conversation)

Respondent: so the resources help us but most of the times the women are reluctant that this is not a real thing, and they want a demonstration on the actual body.

Interviewer: eh eh

Respondent: so we don’t rely on it too much we just use when we want to show how to pinch the skin and inject slowly

Title: ZA_FAC_1.docx

Descriptor Info:	District: Zomba	Target Population: TP1 - Providers/HSAs	Respondent type: Facility-based provider	Aware of SI: Yes - had heard of SI before	Coding phase: 2. Double coded	Client marital status: N/A - provider	Client age group: N/A - provider	Time since trained: Less than a year ago

Codes Applied:	P3.1.3 - IEC/demo materials for counselling/training

Interviewer: Ah is there anything you think can help you in providing counselling to the women who want DMPA-SC?

Respondent: if we had a projector,

Interviewer: mmmm (showing that one is following the conversation)

Respondent: it could help us

Interviewer: eh eh

Respondent: if we could also hear testimonies from the women on their experiences with DMPA-SC.

Interviewer: mmmm (showing that one is following the conversation)

Respondent: so that could help us a lot

Interviewer: mmmm (showing that one is following the conversation)

Respondent: for the adolescents if we could

Title: ZA_FAC_2.docx

Descriptor Info:	District: Zomba	Target Population: TP1 - Providers/HSAs	Respondent type: Facility-based provider	Aware of SI: Yes - had heard of SI before	Coding phase: 3. Triple coded	Client marital status: N/A - provider	Client age group: N/A - provider	Time since trained: Less than a year ago

Codes Applied:	P3.1.3 - IEC/demo materials for counselling/training

Interviewer: Okay. So when explaining to people, do you use any materials when giving counselling the clients?

Respondent: Yes, we do.

Interviewer: What materials do you use?

Respondent: We have a flip chart; we have a certain book which is kept in this room on this table. We use this book when doing one to one counselling. But when doing group counselling, we take all the methods and show the clients. For instance, Sayana looks like this, loop looks like this, Jadelle, level plant looks like this. This is when we’re delivering group counselling.

Title: ZA_FAC_2.docx

Descriptor Info:	District: Zomba	Target Population: TP1 - Providers/HSAs	Respondent type: Facility-based provider	Aware of SI: Yes - had heard of SI before	Coding phase: 3. Triple coded	Client marital status: N/A - provider	Client age group: N/A - provider	Time since trained: Less than a year ago

Codes Applied:	P3.1.3 - IEC/demo materials for counselling/training

Interviewer: Do you use any equipment which help you to teach the people?

Respondent: Yes, as I said earlier, we have tools which we use. For example, we show them how to put on a condom, we show them and demonstrate, we also show then the methods and explain to them how they operate.

Interviewer: So for Sayana do you demonstrate using a condom filled with sugar or sand for better understanding of the teaching that you are giving?

Respondent: We use the one filled with sand not sugar

Interviewer: So you use the one filled with sand?

Respondent: Yes.

Title: ZA_FAC_2.docx

Descriptor Info:	District: Zomba	Target Population: TP1 - Providers/HSAs	Respondent type: Facility-based provider	Aware of SI: Yes - had heard of SI before	Coding phase: 3. Triple coded	Client marital status: N/A - provider	Client age group: N/A - provider	Time since trained: Less than a year ago

Codes Applied:	P3.1.3 - IEC/demo materials for counselling/training	C8.2 - Covert/ open use of injectables/SI

Interviewer: Are women free to take these, for example calendars?

Respondent: Yes, they are.

Interviewer: How about those who hide it from their partners? Are they free to also take these things?

Respondent: No, for those women it is hard for them to get those things.

Title: ZA_FAC_2.docx

Descriptor Info:	District: Zomba	Target Population: TP1 - Providers/HSAs	Respondent type: Facility-based provider	Aware of SI: Yes - had heard of SI before	Coding phase: 3. Triple coded	Client marital status: N/A - provider	Client age group: N/A - provider	Time since trained: Less than a year ago

Codes Applied:	P3.1.3 - IEC/demo materials for counselling/training

Respondent: Provider was demonstrating counselling using a guide

Title: MZs_HSA_3.docx

Descriptor Info:	District: Mzimba South	Target Population: TP1 - Providers/HSAs	Respondent type: HSA	Aware of SI: Yes - had heard of SI before	Coding phase: 3. Triple coded	Client marital status: N/A - provider	Client age group: N/A - provider	Time since trained: Less than a year ago

Codes Applied:	P3.1.3 - IEC/demo materials for counselling/training

Interviewer: would you use any materials/ job aids?

Respondent: yes. We use job aid, family planning book

Title: MZs_HSA_3.docx

Descriptor Info:	District: Mzimba South	Target Population: TP1 - Providers/HSAs	Respondent type: HSA	Aware of SI: Yes - had heard of SI before	Coding phase: 3. Triple coded	Client marital status: N/A - provider	Client age group: N/A - provider	Time since trained: Less than a year ago

Codes Applied:	P3.2.1 - Lack of IEC/demo materials for counselling/training

Interviewer: Do you use any resources (e.g. job aids, condoms filled with sugar/sand etc.) to help the client understand and practice self-injection?

Respondent: yes. We have got… but because we have no Sayana…we have to get condoms Sayana Depo then……Sayana we could …. talk on Sayana itself because it is not in the job aid yah because that one has come very recent. So all other methods will be taught on job aid then Sayana when it comes to Sayana we have to show her exactly what Sayana is so that we are doing as a job aid at the same time as you are talking. Now we are at this step there was I should say it’s a job aid but it had a calendar somewhere that what I always… it’s a calendar but it has drawings. It can act as a job aid I should say because they have drawn everything what is happening until she gets Sayana.

Title: KK_HSA_2.docx

Descriptor Info:	District: Nkhotakota	Target Population: TP1 - Providers/HSAs	Respondent type: HSA	Aware of SI: Yes - had heard of SI before	Coding phase: 2. Double coded	Client marital status: N/A - provider	Client age group: N/A - provider	Time since trained: A year or more ago

Codes Applied:	P3.2.1 - Lack of IEC/demo materials for counselling/training

INTERVIEWER: Like what for instance you are counseling the client on injectable method.

RESPONDENT: In the past we had posters that we were using, I have remembered something, we also had a screening card that we used to show the client whilst counseling them.

(20MINUTES AND 10 SECONDS)

INTERVIEWER: Eyaa

RESPONDENT: But now such aids are not available are missing

INTERVIEWER: Okay

RESPONDENT: Sure

Title: ZA_HSA_3.docx

Descriptor Info:	District: Zomba	Target Population: TP1 - Providers/HSAs	Respondent type: HSA	Aware of SI: Yes - had heard of SI before	Coding phase: 1. Single coded	Client marital status: N/A - provider	Client age group: N/A - provider	Time since trained: A year or more ago

Codes Applied:	P3.1.3 - IEC/demo materials for counselling/training

Interviewer: Alright alright, do you use any materials?

Respondent: When counselling?

Interviewer: Uhhmmm…

Respondent: Yes, there are pamphlets, flip charts which we can use for teaching

Interviewer: Alright, aside from the pamphlets and flip charts, is there anything else that helps you when doing your job? Please explain

Respondent: Yes, we also use the thing that looks like a penis

Title: ZA_HSA_3.docx

Descriptor Info:	District: Zomba	Target Population: TP1 - Providers/HSAs	Respondent type: HSA	Aware of SI: Yes - had heard of SI before	Coding phase: 1. Single coded	Client marital status: N/A - provider	Client age group: N/A - provider	Time since trained: A year or more ago

Codes Applied:	P3.1.3 - IEC/demo materials for counselling/training

Interviewer: How difficult is it to train a woman how to use the self-injection method?

Respondent: It is not hard to train since we use demonstrations, we start by teaching them then after that we use demonstrations which is easy

Interviewer: What other things do you use to help you with the demonstrations?

Respondent: Only the flip charts as I already mentioned

Interviewer: Alright, is there anything else that you use?

Respondent: No

Interviewer: Alright, what do you use for adolescents?

Respondent: Same thing we use flip charts together with health talks that are explained in an efficient way

Title: ZA_HSA_3.docx

Descriptor Info:	District: Zomba	Target Population: TP1 - Providers/HSAs	Respondent type: HSA	Aware of SI: Yes - had heard of SI before	Coding phase: 1. Single coded	Client marital status: N/A - provider	Client age group: N/A - provider	Time since trained: A year or more ago

Codes Applied:	P3.1.3 - IEC/demo materials for counselling/training

Interviewer: Alright thank you very much, Is there anything that can help you in providing training to women about the self-injection family planning method?

Respondent: Yes, we are the only ones who can help ourselves by explaining to clients in a way that they understand and also using flip charts together with demonstrations

Title: ZA_HSA_3.docx

Descriptor Info:	District: Zomba	Target Population: TP1 - Providers/HSAs	Respondent type: HSA	Aware of SI: Yes - had heard of SI before	Coding phase: 1. Single coded	Client marital status: N/A - provider	Client age group: N/A - provider	Time since trained: A year or more ago

Codes Applied:	P3.1.3 - IEC/demo materials for counselling/training	C3.1.2 - Confidence/comfort to inject after counselling/ training	P3.3.2 - Inclusion of practice opportunities for clients in training

Interviewer: Alright, Do the women feel uncomfortable to inject themselves?

Respondent: Ahhhh... Many women do not feel uncomfortable about it since we teach them enough, we also use all the available resources to teach and they inject themselves first whole we are watching them, so they don't feel uncomfortable doing it.

Interviewer: Alright alright, what about adolescents?

Respondent: Same goes for the young women because they are free and comfortable because we teach them enough and during demonstration

Title: ZA_HSA_6.docx

Descriptor Info:	District: Zomba	Target Population: TP1 - Providers/HSAs	Respondent type: HSA	Aware of SI: Yes - had heard of SI before	Coding phase: 1. Single coded	Client marital status: N/A - provider	Client age group: N/A - provider	Time since trained:

Codes Applied:	P3.1.3 - IEC/demo materials for counselling/training	P3.2.1 - Lack of IEC/demo materials for counselling/training	P3.3.2 - Inclusion of practice opportunities for clients in training

Interviewer: Alright, ah in this job of training women on DMPA-SC, do you use any resources for example job aids like filling a condom with soil and practice self-injection on it, do you use any job aids?

Respondent: yes, we use, the time that we were using we took like a flexaform yeah and practiced self-injection on it how it works.

Interviewer: alright.

Respondent: that’s how we were doing when we just started, but now it seems like most of the clients have known how it works yeah

Interviewer: mmmm (showing that one is following the conversation)

Respondent: the past two months we didn’t take any resources to use in training.

Title: ZA_HSA_6.docx

Descriptor Info:	District: Zomba	Target Population: TP1 - Providers/HSAs	Respondent type: HSA	Aware of SI: Yes - had heard of SI before	Coding phase: 1. Single coded	Client marital status: N/A - provider	Client age group: N/A - provider	Time since trained:

Codes Applied:	P3.2.1 - Lack of IEC/demo materials for counselling/training	P4.2.1 - Lack of follow up materials for client to take home

Interviewer: Alright, is there anything else that can support you in providing training to the women in DMPA-SC?

Respondent: If we could have flip charts which have the procedure on how we can train… or even pamphlets, the books that have pages that show the procedure.

Interviewer: yah yah

Respondent: it can help us

Interviewer: Alright, when a woman comes to access contraceptives and they have chosen DMPA-SC, do you provide them with any support in addition to the training?

Respondent: no, it’s just the contraceptive method that they have received

Interviewer: alright

Respondent: and we don’t give out anything else extra

Interviewer: alright

Respondent: sure

Interviewer: ah you don’t give out things like maybe a paper of instructions detailing how a woman can self-inject, or maybe a calendar showing the reinjection day?

Respondent: they said that those things will be sticked, but currently we don’t have them

Interviewer: alright.

Respondent: I don’t know, since we are a group here, we are more than 30 of us

Interviewer: eh

Respondent: I don’t know if others have received those.

Title: MZs_FAC_2.docx

Descriptor Info:	District: Mzimba South	Target Population: TP1 - Providers/HSAs	Respondent type: Facility-based provider	Aware of SI: Yes - had heard of SI before	Coding phase: 2. Double coded	Client marital status: N/A - provider	Client age group: N/A - provider	Time since trained:

Codes Applied:	C2.1.2 - Reduced trips to facility	P3.1.3 - IEC/demo materials for counselling/training

Interviewer: Mmmmmhh (following). What were the good parts of the training?

Respondent: During the training, we was given a flip chart and on this flip chart everything was well demonstrated, and because of this flip chart it has been easy to train women. And the needle is very small which makes the client to feel that they can do it and with this Sayana method clients will only be visiting the facility once a year once they have made a decision of self – injecting themselves and even our job has been reduced since we will be meeting them once a year rather than each and every 3 months.

Interviewer: Alright.

Respondent: Mmmmm. (yes)

Title: MZs_FAC_2.docx

Descriptor Info:	District: Mzimba South	Target Population: TP1 - Providers/HSAs	Respondent type: Facility-based provider	Aware of SI: Yes - had heard of SI before	Coding phase: 2. Double coded	Client marital status: N/A - provider	Client age group: N/A - provider	Time since trained:

Codes Applied:	P3.1.3 - IEC/demo materials for counselling/training

Interviewer: Mmmmh (following)

Respondent: Once we are done with this then we bring out the flip chart and explaining to to her on how she can inject herself at home.

Interviewer: Alright

Respondent: Sure

Interviewer: You have mention things that can help the client when self-injecting?

Respondent: Yes

Interviewer: Is there other things that you can explain when counselling?

Respondent: We use flip chart and also showing her the places where she can self-inject and on how she can do it.

Interviewer: Mmmmm (following)

Respondent: Yes, not only flip chart

Title: MZs_FAC_2.docx

Descriptor Info:	District: Mzimba South	Target Population: TP1 - Providers/HSAs	Respondent type: Facility-based provider	Aware of SI: Yes - had heard of SI before	Coding phase: 2. Double coded	Client marital status: N/A - provider	Client age group: N/A - provider	Time since trained:

Codes Applied:	P3.1.3 - IEC/demo materials for counselling/training

Interviewer: Alright. Is there anything that could help you with training clients in SI?

Respondent: I think flip chart is okay

Interviewer: Is it enough?

Respondent: Mmmmm… (thinking), yes it`s enough.

Title: MZs_HSA_6.docx

Descriptor Info:	District: Mzimba South	Target Population: TP1 - Providers/HSAs	Respondent type: HSA	Aware of SI: Yes - had heard of SI before	Coding phase: 1. Single coded	Client marital status: N/A - provider	Client age group: N/A - provider	Time since trained: A year or more ago

Codes Applied:	P3.3 - Counselling/ training approach, general	P3.3.2 - Inclusion of practice opportunities for clients in training	P3.1.3 - IEC/demo materials for counselling/training	P4.1.1 - Follow up materials for client to take home

Interviewer: now I would like you to tell me how you would counsel a woman or an adolescent who has come to get injectable. I would like you to tell me from the time they come to the clinic until you finish with them. How do you greet a client when she has come to you?

Respondent: first we greet them; we introduce ourselves because many of them do not know us. we tell them that we are family planning providers. We tell them that whatever we discuss is kept confidential. Those who are free introduce themselves, where they are coming from and that they came to get family planning methods. Then we start counselling them using the counselling cards. Even if they choose Sayana we teach them on all methods. Then we get our checklist and ask the client questions. If she gets Sayana we wash our hands first. We counsel them on how to self-inject. Then we wash hands, put on a mask, then you are ready. We get the vial and show them. We counsel them on the mode of action of Sayana. We also give then dates for the next dose. Then we start demonstrating the procedure. We shake the vial slowly and touch it on the neck and close it. The closing opens the contents. Then we show them the area to inject on the abdomen, arm or thigh to pinch the skin and then self-Inject all the contents. We advise them not to rub on the area. They follow this procedure after us. we then give them some for home and a calendar. We circle the dates they are supposed to self-inject on the next doses.

Interviewer: how many methods would you counsel this woman

Respondent: come again

Interviewer: how many methods of injectable would you counsel this woman

Respondent: when she comes to me that she wants injectable, I would tell her that we have another method Sayana which a woman self-inject. So a woman will make a decision on which method she would want like self-injection

Interviewer: what questions would you ask during the counselling process

Respondent: have you ever had migraine headache? High blood pressure? When was your last menstrual period? Are you suspecting that you are pregnant? We ask many questions which are on the checklist.

Interviewer: apart from Sayana, which other method are you going to counsel this woman

Respondent: on family planning

Interviewer: yes, on the injectable methods

Respondent: I will counsel her on Depo IM which is given by the provider and Sayana for self-injection. So she will choose

Interviewer: after she chooses

Respondent: if she chooses let’s say Sayana for self-injection then I will counsel her on how to self-inject then supply Sayana with a calendar to take home. If she came with a spouse, we counsel them together.

Interviewer: what do you use when counselling

Respondent: like I have already said we use a counselling card

Interviewer: do you use anything else

Respondent: no, just a counselling card

Title: MZs_HSA_6.docx

Descriptor Info:	District: Mzimba South	Target Population: TP1 - Providers/HSAs	Respondent type: HSA	Aware of SI: Yes - had heard of SI before	Coding phase: 1. Single coded	Client marital status: N/A - provider	Client age group: N/A - provider	Time since trained: A year or more ago

Codes Applied:	P3.1.3 - IEC/demo materials for counselling/training

Interviewer: what type of aids do you use when counselling

Respondent: we demonstrate using the Sayana vial

Interviewer: what do you use to demonstrate

Respondent: I use tomato at my catchment area. I have someone with hearing problems. So I just took tomato to demonstrate

Title: KK_HSA_6.docx

Descriptor Info:	District: Nkhotakota	Target Population: TP1 - Providers/HSAs	Respondent type: HSA	Aware of SI: Yes - had heard of SI before	Coding phase: 1. Single coded	Client marital status: N/A - provider	Client age group: N/A - provider	Time since trained: A year or more ago

Codes Applied:	P3.1.3 - IEC/demo materials for counselling/training

INTERVIEWER: When doing counselling, do you use any resources that would help in your explanations?

RESPONDENT: Yes, we have books we use when teaching about family planning. We also have calendars that explains how a woman should self-inject and the areas for the injection. If it is a provider injecting the, they see that we do it on the arm, but if it is self-injection, it is either on the thigh or the area around the belly button. We also have counselling cards we use. The woman explains according to what is on the card for example, a woman is washing her hands, tearing off the packet for the drug, and then she is self-injecting. We teach what is in the books and counselling cards.

INTERVIEWER: ok.

RESPONDENT: sure.

Title: KK_HSA_6.docx

Descriptor Info:	District: Nkhotakota	Target Population: TP1 - Providers/HSAs	Respondent type: HSA	Aware of SI: Yes - had heard of SI before	Coding phase: 1. Single coded	Client marital status: N/A - provider	Client age group: N/A - provider	Time since trained: A year or more ago

Codes Applied:	P3.1.3 - IEC/demo materials for counselling/training

INTERVIEWER: Do you use any resources to help the client understand?

RESPONDENT: It is just counselling. We tell them in details.

INTERVIEWER: mmmhu.

RESPONDENT: yea.

INTERVIEWER: For example using a condom filled with sugar, do you do that?

RESPONDENT: Yes, we do. We fill a condom with sugar and then we use both Sayana and Depo Provera needles. We explain to a client, you see how I have done it, this is like your body. And then we tell her to do like what I did. And then she will say it is easy. After that, she will do the self-injection.

INTERVIEWER: Do you use the condom all the time you are training clients on Sayana?

RESPONDENT: We mostly use it with new clients because new clients are difficult to train because they are blank. But for them I already trained, we just conclude. We use sugar with condoms on new clients and some Sayana for demonstration.

Title: KK_HSA_6.docx

Descriptor Info:	District: Nkhotakota	Target Population: TP1 - Providers/HSAs	Respondent type: HSA	Aware of SI: Yes - had heard of SI before	Coding phase: 1. Single coded	Client marital status: N/A - provider	Client age group: N/A - provider	Time since trained: A year or more ago

Codes Applied:	P3.2.1 - Lack of IEC/demo materials for counselling/training

INTERVIEWER: mmmhu. Is there anything that can help you in training clients about Sayana?

RESPONDENT: aaah what I know is about counselling cards they are good they gave us but they have to add some more and other items. The other items maybe the ministry of health can support us with so that clients should see what they are being trained on.

INTERVIEWER: what are these items?

RESPONDENT: Mmmmh, for example, what you are saying about sugar and condoms. There are some things that the government can buy.

INTERVIEWER: okay.

RESPONDENT: mmmhu.

Title: KK_HSA_6.docx

Descriptor Info:	District: Nkhotakota	Target Population: TP1 - Providers/HSAs	Respondent type: HSA	Aware of SI: Yes - had heard of SI before	Coding phase: 1. Single coded	Client marital status: N/A - provider	Client age group: N/A - provider	Time since trained: A year or more ago

Codes Applied:	P3.2.1 - Lack of IEC/demo materials for counselling/training

RESPONDENT: Alright. If they could give us more training resources for example counselling cards and more calendars to give to each woman so that they should be able to see what they are supposed to do because there are illustrations, and some know how to read

Title: MZs_FAC_1.docx

Descriptor Info:	District: Mzimba South	Target Population: TP1 - Providers/HSAs	Respondent type: Facility-based provider	Aware of SI: Yes - had heard of SI before	Coding phase: 3. Triple coded	Client marital status: N/A - provider	Client age group: N/A - provider	Time since trained: A year or more ago

Codes Applied:	P3.1.3 - IEC/demo materials for counselling/training

Interviewer: Alright. Would you use any materials or job aid when counselling on to inject themselves?

Respondent: Yah, we usually do

Interviewer: What could be these materials?

Respondent: With Sayana press we already have the demonstration part on the Calendar there are pictures and instructions there which we use. On DEPO we usually have the flip chart which we show her how it is done.

Interviewer: Mmmmh.

Respondent: We usually follow those.

Interviewer: Anything else that you can add about the material/job aid?

Respondent: On injectables, we can show them that this is the the simple thing we are talking about and this is the Sayana we are talking about and on DEPO we say that this is the syringe we are talking about and you can compare.

Title: MZs_FAC_1.docx

Descriptor Info:	District: Mzimba South	Target Population: TP1 - Providers/HSAs	Respondent type: Facility-based provider	Aware of SI: Yes - had heard of SI before	Coding phase: 3. Triple coded	Client marital status: N/A - provider	Client age group: N/A - provider	Time since trained: A year or more ago

Codes Applied:	P3.2.1 - Lack of IEC/demo materials for counselling/training

Interviewer: Alright. Do you use any resources like job aid, condom filled with sugar/sand to help the client understand and practice self-injection?

Respondent: We are supposed to be doing that and we used to but at this moment we are not using it and at this moment we just using job aid.

Interviewer: Why are you not using it?

Respondent: Aaaaaah, I think after the sugars, the condoms we were using, we could not get another sugar and condoms.

Interviewer: Okay.

Respondent: Yah, so…… I think I should not cheat you, we are not using it anymore.

Title: ZA_HSA_1.docx

Descriptor Info:	District: Zomba	Target Population: TP1 - Providers/HSAs	Respondent type: HSA	Aware of SI: Yes - had heard of SI before	Coding phase: 1. Single coded	Client marital status: N/A - provider	Client age group: N/A - provider	Time since trained: Less than a year ago

Codes Applied:	P3.2.1 - Lack of IEC/demo materials for counselling/training

Interviewer: In explaining do you use any materials/job aids?

Respondent: I have never used

Interviewer: You have never used it?

Respondent: Mmmhh, on my side

Title: ZA_HSA_1.docx

Descriptor Info:	District: Zomba	Target Population: TP1 - Providers/HSAs	Respondent type: HSA	Aware of SI: Yes - had heard of SI before	Coding phase: 1. Single coded	Client marital status: N/A - provider	Client age group: N/A - provider	Time since trained: Less than a year ago

Codes Applied:	P3.2.1 - Lack of IEC/demo materials for counselling/training

Interviewer: When training clients on how to self-inject do you use any resources that can help illustrate how they can self-inject such as a condom filled with sugar or sand?

Respondent: We just explain it to them, but we have never tried it

Interviewer: To help them better understand the training?

Respondent: Ahhh not here, we did it at the training. We have never tried it here in the community

Title: MZs_HSA_2.docx

Descriptor Info:	District: Mzimba South	Target Population: TP1 - Providers/HSAs	Respondent type: HSA	Aware of SI: Yes - had heard of SI before	Coding phase: 1. Single coded	Client marital status: N/A - provider	Client age group: N/A - provider	Time since trained: A year or more ago

Codes Applied:	P3.1.3 - IEC/demo materials for counselling/training

Interviewer: you said you would counsel on Depo and Sayana. Would you use any aids during the counselling?

Respondent: I would use a chart. We have charts which we use as well as Sayana and Depo

Title: MZs_HSA_2.docx

Descriptor Info:	District: Mzimba South	Target Population: TP1 - Providers/HSAs	Respondent type: HSA	Aware of SI: Yes - had heard of SI before	Coding phase: 1. Single coded	Client marital status: N/A - provider	Client age group: N/A - provider	Time since trained: A year or more ago

Codes Applied:	P3.1.3 - IEC/demo materials for counselling/training

Interviewer: do you use any aid when teaching women apart from Sayana

Respondent: we use a chart for family planning methods

Interviewer: what else

Respondent: we have…(thinking out loud)...on Sayana we take Sayana and other things. We also take pills, Depo Provera. that’s what I can say

Title: MZs_HSA_2.docx

Descriptor Info:	District: Mzimba South	Target Population: TP1 - Providers/HSAs	Respondent type: HSA	Aware of SI: Yes - had heard of SI before	Coding phase: 1. Single coded	Client marital status: N/A - provider	Client age group: N/A - provider	Time since trained: A year or more ago

Codes Applied:	P3.1.3 - IEC/demo materials for counselling/training

Interviewer: do you need any aids when counselling a woman on Sayana Press

Respondent: we are supposed to have materials to aid us in the counselling

Interviewer: what do you mean? May you clarify?

Respondent: on self-injection we need to have something to practice on like Tomato. That how we do it

Title: KK_FAC_2.docx

Descriptor Info:	District: Nkhotakota	Target Population: TP1 - Providers/HSAs	Respondent type: Facility-based provider	Aware of SI: Yes - had heard of SI before	Coding phase: 1. Single coded	Client marital status: N/A - provider	Client age group: N/A - provider	Time since trained: A year or more ago

Codes Applied:	P3.1.3 - IEC/demo materials for counselling/training

INTERVIEWER: Mmmhu. So when conducting counselling that there is Depo and Sayana injections, right, do you any materials/job aids?

RESPONDENT: yes.

INTERVIEWER: like what?

RESPONDENT: The actual drugs, aaa…what can I say, needle (giggle), needle. syringe

INTERVIEWER: syringe (laughs).

RESPONDENT: syringe. When we say syringe, we are talking about Depo.

INTERVIEWER: aha.

RESPONDENT: Syringe and drugs, we are talking about Sayana.

INTERVIEWER: okay.

RESPONDENT: aaa there are also flip charts that we use.

INTERVIEWER: mmmmhu.

RESPONDENT: yeah.

Title: KK_FAC_2.docx

Descriptor Info:	District: Nkhotakota	Target Population: TP1 - Providers/HSAs	Respondent type: Facility-based provider	Aware of SI: Yes - had heard of SI before	Coding phase: 1. Single coded	Client marital status: N/A - provider	Client age group: N/A - provider	Time since trained: A year or more ago

Codes Applied:	P3.3 - Counselling/ training approach, general	P3.1.5 - Ease of training clients in SI	P3.1.3 - IEC/demo materials for counselling/training

INTERVIEWER: Alright. How difficult or easy is it to train a woman to self-inject? How difficult or easy is it to train a woman about Sayana?

RESPONDENT: Teaching a woman about Sayana is not difficult according to me.

INTERVIEWER: How about self-injecting, the process of training them to self-inject is it easy or difficult?

RESPONDENT: The process is not difficult to teach them. But the practical part of self-injecting is up to that person. That is where we keep on repeating until she gets it. Yeah.

INTERVIEWER: Do you use any resources when teaching them self-injection?

RESPONDENT: We use flexa foams

INTERVIEWER: okay.

RESPONDENT: mmmh.

INTERVIEWER: What happens with these flexa foams?

RESPONDENT: flexa foam?

INTERVIEWER: mmmh.

RESPONDENT: We take the Sayana syringe and then we explain to them that you should hold the syringe like this on the neck. Don’t hold on the bulb because it has a bulb. They should not hold that part because if they do, Sayana will spill.

INTERVIEWER: mmmhu.

RESPONDENT: Yeah. And we tell them to put the syringe at an angle they can see. They are supposed to inject above the belly button or on a left or right thigh.

INTERVIEWER: mmmhu.

RESPONDENT: Yeah. But if they are being injected by the provider, it’s on the arm.

INTERVIEWER: Okay. Do you use condoms filled with sugar when training the women?

RESPONDENT: We don’t use condoms filled with sugar. We just use them as a demonstration.

INTERVIEWER: Okay. What do you put inside the condom?

RESPONDENT: condom?

INTERVIEWER: Yes…no…I mean…when others are training clients, they fill a condom with sugar for the women to try as if they are injecting the Sayana. Do you use the condom?

RESPONDENT: Like a condom?

INTERVIEWER: mmmmh.

RESPONDENT: No we don’t use a condom.

INTERVIEWER: You don’t?

RESPONDENT: mmm mmm [no].

INTERVIEWER: But you say you demonstrate with a flexa foam?

RESPONDENT: mmmh a flexa foam.

INTERVIEWER: The flexa foam in this case is a thigh?

RESPONDENT: Yes, a small one.

INTERVIEWER: okay.

RESPONDENT: mmmh.

Title: KK_FAC_2.docx

Descriptor Info:	District: Nkhotakota	Target Population: TP1 - Providers/HSAs	Respondent type: Facility-based provider	Aware of SI: Yes - had heard of SI before	Coding phase: 1. Single coded	Client marital status: N/A - provider	Client age group: N/A - provider	Time since trained: A year or more ago

Codes Applied:	P3.1.3 - IEC/demo materials for counselling/training

INTERVIEWER: Mmmhu, alright. Is there anything that could help you with training clients in Sayana? Is there anything that can help you with training the clients?

RESPONDENT: Aaah in terms of the training, we have the resources. We have Sayana and also flip charts.
