# Supplementary material for: Health care provider and client experiences of counselling on depot medroxyprogesterone acetate subcutaneous (DMPA-SC) for self-injection in Malawi
Source: PLOS Glob Public Health. 2023 Nov 30;3(11):e0002057. doi: 10.1371/journal.pgph.0002057 (PMC10688740; doi:10.1371/journal.pgph.0002057)
Supplement: S1 Data — (ZIP) [file pgph.0002057.s001.zip › S2/P3.1.4 provider support to couns.doc]

Title: ZA_FAC_1.docx

Descriptor Info:	District: Zomba	Target Population: TP1 - Providers/HSAs	Respondent type: Facility-based provider	Aware of SI: Yes - had heard of SI before	Coding phase: 2. Double coded	Client marital status: N/A - provider	Client age group: N/A - provider	Time since trained: Less than a year ago

Codes Applied:	P3.1.4 - Provider sufficient support to counsel/train on SI

Respondent: upon hearing that Sayana is not painful we started encouraging the women to take it up

Interviewer: alright

Respondent: so thoroughly health education helps us to mobilize the communities to encourage each other to take up the commodity

Interviewer: mmmm (showing that one is following the conversation)

Respondent: the mobilisers make our work very easy

Title: MZs_HSA_6.docx

Descriptor Info:	District: Mzimba South	Target Population: TP1 - Providers/HSAs	Respondent type: HSA	Aware of SI: Yes - had heard of SI before	Coding phase: 1. Single coded	Client marital status: N/A - provider	Client age group: N/A - provider	Time since trained: A year or more ago

Codes Applied:	P3.4 - Time to counsel/train, general	P3.1.4 - Provider sufficient support to counsel/train on SI

Interviewer: how long does it take to counsel a client on Sayana

Respondent: it depends on how a client understand. It takes about 30 minutes or more

Interviewer: for those who understand slowly how long does it take?

Respondent: 30-45 minutes

Interviewer: those who understands faster?

Respondent: 25 minutes some 15 minutes

Interviewer: is there a difference when you counsel adolescents

Respondent: no

Interviewer: do you have enough time to counsel a woman or an adolescent

Respondent: yes

Interviewer: what about on your busy days

Respondent: yes. We just divide the time and the clinics. We may schedule family planning clinic after 12 so that we have enough time for counselling

Title: MZs_FAC_1.docx

Descriptor Info:	District: Mzimba South	Target Population: TP1 - Providers/HSAs	Respondent type: Facility-based provider	Aware of SI: Yes - had heard of SI before	Coding phase: 3. Triple coded	Client marital status: N/A - provider	Client age group: N/A - provider	Time since trained: A year or more ago

Codes Applied:	P3.1.4 - Provider sufficient support to counsel/train on SI	C3.2.2 - Lack of inclination or confidence to inject, even after training

Interviewer: Okay, have you or your colleagues discuss this about the Sayana press that a woman can inject herself, have you ever discuss it with your colleagues?

Respondent: Yah, in many occasions.

Interviewer: What was your discussion like, can you share?

Respondent: Yah, we know that may be in some scenarios a woman may not convincing to do it in the right way and the way you are teaching her and I think she cannot do by herself, so those we put on providers injection and we need to supervise another visit. So those are the issues sometimes we discuss that no this client was very nervous she couldn’t do the way I taught her or she was not comfortable actually to do it herself and she says no still inject me.

Interviewer: Mmmmh.

Respondent: So those are the issues sometimes we discuss with our friends that may be next she will get used with this.

Title: MZs_FAC_1.docx

Descriptor Info:	District: Mzimba South	Target Population: TP1 - Providers/HSAs	Respondent type: Facility-based provider	Aware of SI: Yes - had heard of SI before	Coding phase: 3. Triple coded	Client marital status: N/A - provider	Client age group: N/A - provider	Time since trained: A year or more ago

Codes Applied:	P3.4 - Time to counsel/train, general	P3.1.4 - Provider sufficient support to counsel/train on SI	P3.1.5 - Ease of training clients in SI

Q 13. Interviewer: How easy or difficult it to train client in DMPA-SC for self-injection?

Respondent: Is not so difficult only that it really need time.

Interviewer: Mmmmmm.

Respondent: To explain to the client, you need to have uninterrupted time and also sometimes nurses are busy and a client come and say I need injection and you are busy, then you just refer them to the HSA`s to assist because you need to sit down and start explain all the procedures up until you are convinced that she can do it. So it’s not that difficult but you just need time to do it.

Title: MZs_FAC_1.docx

Descriptor Info:	District: Mzimba South	Target Population: TP1 - Providers/HSAs	Respondent type: Facility-based provider	Aware of SI: Yes - had heard of SI before	Coding phase: 3. Triple coded	Client marital status: N/A - provider	Client age group: N/A - provider	Time since trained: A year or more ago

Codes Applied:	P3.1.4 - Provider sufficient support to counsel/train on SI

Interviewer: Alright. Is there anything that could help you in training clients in SI?

Respondent: In terms of resources or?

Interviewer: Anything, any help that you may need in training the client

Respondent: Yah, luckily we have HSAs around which we usually refer if we feel we are busy as nurses and they do it.

Interviewer: Alright. Is that all?

Respondent: Yes.

Title: KK_FAC_2.docx

Descriptor Info:	District: Nkhotakota	Target Population: TP1 - Providers/HSAs	Respondent type: Facility-based provider	Aware of SI: Yes - had heard of SI before	Coding phase: 1. Single coded	Client marital status: N/A - provider	Client age group: N/A - provider	Time since trained: A year or more ago

Codes Applied:	P3.3 - Counselling/ training approach, general	P3.1.4 - Provider sufficient support to counsel/train on SI

INTERVIEWER: mmmhu. Do you feel you have enough time to conduct these trainings?

RESPONDENT: I feel that it’s enough.

INTERVIEWER: it’s enough?

RESPONDENT: mmmmmh.

INTERVIEWER: Do you conduct these trainings in a group or one to one?

RESPONDENT: Firstly, there is group counselling and from the group counselling, we go to individual counselling to a person who has chosen…

INTERVIEWER: mmmhu.

RESPONDENT: Explaining to her about the method.

INTERVIEWER: okay.

RESPONDENT: mmmhu.

INTERVIEWER: What about on busy days, does it still take 15 minutes to conduct the training on self-injection? Is it still the same 15 minutes?

RESPONDENT: yes, we divide the work.

INTERVIEWER: okay.

RESPONDENT: mmmmh.

INTERVIEWER: How do you divide the work?

RESPONDENT: Others come because of emergencies maybe they are sick. We first assist those ones so that they should go and for us to have a good number.

INTERVIEWER: okay.

RESPONDENT: mmmhu.
