# Supplementary material for: Health care provider and client experiences of counselling on depot medroxyprogesterone acetate subcutaneous (DMPA-SC) for self-injection in Malawi
Source: PLOS Glob Public Health. 2023 Nov 30;3(11):e0002057. doi: 10.1371/journal.pgph.0002057 (PMC10688740; doi:10.1371/journal.pgph.0002057)
Supplement: S1 Data — (ZIP) [file pgph.0002057.s001.zip › S2/P3.1.5+P3.2.3 prov percept ease-diff couns.doc]

Title: ZA_HSA_2.docx

Descriptor Info:	District: Zomba	Target Population: TP1 - Providers/HSAs	Respondent type: HSA	Aware of SI: Yes - had heard of SI before	Coding phase: 3. Triple coded	Client marital status: N/A - provider	Client age group: N/A - provider	Time since trained: Less than a year ago

Codes Applied:	P2.3 - Provider training content	P3.1.5 - Ease of training clients in SI	P3.3 - Counselling/ training approach, general

Interviewer: The time that you received this training, ah I wanted you to tell me a little bit about the training that you received…

Respondent: mmmm (showing that he is following)

Interviewer: …Relating to providing this method of family planning which women are able to self-inject.

Respondent: Alright, eh ah, most of it was about how we can train a woman on how they can be able to self-inject.

Interviewer: mmm (Showing that one is following the conversation)

Respondent: For most of the women didn’t go to school, but we are able to communicate with them and teach them that this is how we handle the injection (Explains while demonstrating with gestures), there is this little pot, so when we want to self-inject, we pinch the skin a little bit, inject, and then when we inject ah, we stop pinching the skin and then squeeze the little pot where the liquid is…

Interviewer: mmm (Showing that one is following the conversation)

Respondent: Yes, so that the injection gets to all the right places.

Title: ZA_HSA_2.docx

Descriptor Info:	District: Zomba	Target Population: TP1 - Providers/HSAs	Respondent type: HSA	Aware of SI: Yes - had heard of SI before	Coding phase: 3. Triple coded	Client marital status: N/A - provider	Client age group: N/A - provider	Time since trained: Less than a year ago

Codes Applied:	P3.4 - Time to counsel/train, general	P3.3.1 - Differences in counselling/training approach by population group	P3.2.3 - Complexity/difficulty of training clients in SI	A6 - Adol Perspectives/preferences for DMPA-SC SI

Interviewer: Alright, does the time that you spend training a client in DMPA-SC differ from one client to the next?

Respondent: Yes, they can be different.

Interviewer: mmm (showing that one is following the conversation)

Respondent: Because understanding is different. There are some people who can easily understand and some who take time to understand, they keep asking questions so that they can fully understand. So, the other ones we take more time with them even 45 minutes which I have mentioned may not be enough, yes, because the client wants to know more so we try to be with the for more time, but most of the times the time is between 30 to 45 minutes…

Interviewer: Alright.

Respondent: Sure, sure.

Interviewer: Alright, what about for adolescents.?

Respondent: mmm (showing that they are following the conversation)

Interviewer: Does the time that you take to train them differ compared to older women?

Respondent: Yes, it is possible like that, it can be like that, yes because girls, it is difficult that they should understand faster yeah,

Interviewer: mmm (showing that one is following the conversation)

Respondent: Compared to an older woman, because older women already know the things, and maybe they have already heard the things from somebody else, but mostly the adolescents don’t know most of the information relating to family planning…

Interviewer: Alright.

Respondent: Yes, if we are to do shortcut for those ones it is difficult…

Interviewer: mmm (showing that one is following the conversation)

Respondent: …if the find us we are required to be with them for the appropriate length of time to train them properly so that they appreciate the things that we have trained them…

Interviewer: mmm (showing that one is following the conversation)

Respondent: The we give them the family planning methods…

Title: KK_HSA_1.docx

Descriptor Info:	District: Nkhotakota	Target Population: TP1 - Providers/HSAs	Respondent type: HSA	Aware of SI: Yes - had heard of SI before	Coding phase: 2. Double coded	Client marital status: N/A - provider	Client age group: N/A - provider	Time since trained: A year or more ago

Codes Applied:	P1.1.1 - Positive provider attitudes towards SI	P3.1.5 - Ease of training clients in SI	P2.1.2 - Sufficient original provider training	P4.3 - Follow up approach	C7 - Resupply

Interviewer: What did you…what did you think of the training?

Respondent: The training is good. And also this Sayana, because of the long distance that women have to travel, when you counsel them and when the women understand, it is very easy for women to administer the method at home. When they have run out of Sayana, they come to you before their actual visit date. Let’s say you have given them three, yee when they inject the second dose, before they inject the third dose, they come and explain that they are remaining with one. So we tell them to come and get other doses when they inject the third dose. Or else when we are in the community, and we have Sayana, we give them other doses.

Interviewer: mmmh.

Respondent: So yeah. It is a very good method for women. they like it in our communities.

Interviewer: okay

Respondent: mmmh

Title: KK_HSA_1.docx

Descriptor Info:	District: Nkhotakota	Target Population: TP1 - Providers/HSAs	Respondent type: HSA	Aware of SI: Yes - had heard of SI before	Coding phase: 2. Double coded	Client marital status: N/A - provider	Client age group: N/A - provider	Time since trained: A year or more ago

Codes Applied:	P88 - Provider great quotes	P3.2.3 - Complexity/difficulty of training clients in SI	P2.1.1 - Refresher training/mentorship	P1.2.1 - Negative provider attitudes towards SI	P3.3 - Counselling/ training approach, general

Interviewer: Alright. What is your experience, based on what you have seen since you started providing Sayana to women in your community, aaah what do women say when you explain that they can self-inject?

Respondent: At first women they…they…they doubt and they say that it is not possible for them to what, to self-inject. And we struggle with them. You counsel them and when you are done and ask is there anyone? aah I cant. All of them would say no. when that happens you as a provider, you have just wasted your time right, counselling them and everything eh…eh…by the end of that time everyone is saying no and you have to inject them. it could have been better to just begin…just inject them. But after some time, when they came for supervision they helped us. You should be doing it like this and here you do this, you will see you will have more clients, and that is when they told us that when doing group counselling, and there are two or three women, those ones should be examples to their friends. When we began using that system, we saw that it was working. When you train them, then because its women only, Mrs So-so-so you inject yourself. Your friends here are saying they cannot self-inject. So we will begin with you. And then you will give one woman who has self-injected before. And then she will inject on her thigh. You take pills…you also take the remaining 3 doses and off she goes. The second one will also do the same thing. And then the remaining ones will say oh it is possible and painless and it is short or at home you can do it. And then I saw that I now had what,

Interviewer: mmmmhu

Respondent: mmmh

Title: KK_HSA_1.docx

Descriptor Info:	District: Nkhotakota	Target Population: TP1 - Providers/HSAs	Respondent type: HSA	Aware of SI: Yes - had heard of SI before	Coding phase: 2. Double coded	Client marital status: N/A - provider	Client age group: N/A - provider	Time since trained: A year or more ago

Codes Applied:	P3.1.5 - Ease of training clients in SI	P3.3 - Counselling/ training approach, general

Interviewer: How about the process for…for self injecting right? how do you think it is easy or difficult to train?

Respondent: Aah it is not difficult.

Interviewer: mmmhu

Respondent: Mmmh. Because when you train her, and when you open right, you tell her that she is free to self-inject the area around the belly button. Right hand, left or up here. Or this or that thigh. So when the woman says aah, I will inject on the thigh, you just help her that we hold Sayana like this. And the leg; we also hold the thigh like this.

Interviewer: mmmh

Respondent: yes

Title: ZA_HSA_5.docx

Descriptor Info:	District: Zomba	Target Population: TP1 - Providers/HSAs	Respondent type: HSA	Aware of SI: Yes - had heard of SI before	Coding phase: 2. Double coded	Client marital status: N/A - provider	Client age group: N/A - provider	Time since trained: Less than a year ago

Codes Applied:	P3.2.3 - Complexity/difficulty of training clients in SI	P3.2 - Barrier, general/other

Interviewer: Alright, would you need to be supported on your work of providing Sayana Press?

Respondent: Yes, I would need the support especially the help would be like for these people to understand us it is very hard

Interviewer: Mmm (showing that one is following the conversation)

Respondent: Let me not lie, so for you to reach out to many women you need one on one sessions yeah

Interviewer: Mmm (showing that one is following the conversation)

Respondent: You should be able to explain to the client properly and also use a model who is using the method properly, so that maybe the women should easily receive it and they should make their friends to accept the method, maybe helping out those ones

Interviewer: Mmm (showing that one is following the conversation)

Respondent: They could be curious, and they could appreciate that this is okay and there are no problems, we cannot be administering the injection to each woman individually

Interviewer: Mmm (showing that one is following the conversation)

Respondent: But also, for those people who like the method, we should recruit those that they can assist their friends.

Interviewer: Mmm (showing that one is following the conversation)

Respondent: They should understand it

Interviewer: Alright.

Respondent: Yes

Title: ZA_HSA_5.docx

Descriptor Info:	District: Zomba	Target Population: TP1 - Providers/HSAs	Respondent type: HSA	Aware of SI: Yes - had heard of SI before	Coding phase: 2. Double coded	Client marital status: N/A - provider	Client age group: N/A - provider	Time since trained: Less than a year ago

Codes Applied:	C1.1.1 - Positive client attitudes to SI	C1.1.3 - Awareness of DMPA-SC SI	P3.2.3 - Complexity/difficulty of training clients in SI

Respondent: But it’s not a thing where you teach, and people understand it

Interviewer: Mmm (showing that one is following the conversation)

Respondent: You know people in the communities find it difficult to understand properly

Interviewer: It’s difficult

Respondent: It’s difficult so whenever a few of them accept it, and some of them bother you about it then you know that …

Interviewer: They are accepting it

Respondent: They are accepting it

Title: ZA_HSA_5.docx

Descriptor Info:	District: Zomba	Target Population: TP1 - Providers/HSAs	Respondent type: HSA	Aware of SI: Yes - had heard of SI before	Coding phase: 2. Double coded	Client marital status: N/A - provider	Client age group: N/A - provider	Time since trained: Less than a year ago

Codes Applied:	P3.1.5 - Ease of training clients in SI

Interviewer: Alright, how easy or difficult is it to train women in Sayana Press

Respondent: It’s not difficult.

Interviewer: Alright.

Respondent: It needs that you take the women step by step, you should act like you are injecting yourself

Interviewer: Mmm (showing that one is following the conversation)

Respondent: You should be there and there should be a good relationship between yourself and the women.

Interviewer: Mmm (showing that one is following the conversation)

Respondent: It’s not hard because when your relationship with the women is not good it means it cannot what

Interviewer: It cannot work

Respondent: You also need to make sure that you blend with the community

Interviewer: Mmm (showing that one is following the conversation)

Respondent: And then you can tell them what you need to tell them, that way they start believing you.

Interviewer: Mmm (showing that one is following the conversation)

Respondent: Eh

Title: KK_HSA_5.docx

Descriptor Info:	District: Nkhotakota	Target Population: TP1 - Providers/HSAs	Respondent type: HSA	Aware of SI: Yes - had heard of SI before	Coding phase: 1. Single coded	Client marital status: N/A - provider	Client age group: N/A - provider	Time since trained: A year or more ago

Codes Applied:	P3.1.5 - Ease of training clients in SI	P3.2.3 - Complexity/difficulty of training clients in SI

INTERVIEWER: Alright, how easy or difficult is it to train a client in DMPA-SC for self-injection

RESPONDENT: It’s easy when it is in a group counselling

INTERVIEWER: Ok

RESPONDENT: As it happens that in a group you may found few individuals who started some time back, so it becomes do easy

INTERVIEWER: Alright

RESPONDENT: But for if you have new clients, it becomes so difficult

INTERVIEWER: Alright

RESPONDENT: Because some say I may get noticed when I go home. Some few individuals have shared to say after doing a self-injection a dimple is created on an injected place but after some time the dimple fades away.

Title: KK_HSA_5.docx

Descriptor Info:	District: Nkhotakota	Target Population: TP1 - Providers/HSAs	Respondent type: HSA	Aware of SI: Yes - had heard of SI before	Coding phase: 1. Single coded	Client marital status: N/A - provider	Client age group: N/A - provider	Time since trained: A year or more ago

Codes Applied:	P3.1.5 - Ease of training clients in SI	P3.3.1 - Differences in counselling/training approach by population group

INTERVIEWER: Do you think having previous experience with injectables makes a woman more or less likely to consider self-injection?

RESPONDENT: Having previous experience does help, for instance, during counselling if we have clients who started the method some time time, you will hear them commenting that they know the stuff already hence they do not need to go through the counselling process. So if you try to give them some few questions regarding the method you will know that they are indeed responding to each one of them, this makes the work simple because you know the counselling process will be easy and does not take a lot of time. Whilst if you have new clients it takes long to counsel them

INTERVIEWER: Alright

RESPONDENT: Sure

Title: MZs_HSA_1.docx

Descriptor Info:	District: Mzimba South	Target Population: TP1 - Providers/HSAs	Respondent type: HSA	Aware of SI: Yes - had heard of SI before	Coding phase: 2. Double coded	Client marital status: N/A - provider	Client age group: N/A - provider	Time since trained:

Codes Applied:	P1.1.1 - Positive provider attitudes towards SI	P3.1.5 - Ease of training clients in SI

Interviewer: Has opinion you/colleagues on self-injection changed over time?

Respondent: Yes, it has changed through the training we received and also by seeing the needle itself, we have seen that women do like it and they are saying that this one is simple than DEPO.

Interviewer: Did you/do you have any concerns about this idea?

Respondent: There is no concerns.

Interviewer: Why don’t you have any concern?

Respondent: There is no concerns because women do use it very well.

Interviewer: Alright.

Respondent: Mmmmm yes.

Interviewer: What specifically has influenced your opinion to change?

Respondent: Since the training we received and upon the provision of Sayana to women, we have seen that its simple to self-inject and the needle is just okay.

Title: MZs_HSA_1.docx

Descriptor Info:	District: Mzimba South	Target Population: TP1 - Providers/HSAs	Respondent type: HSA	Aware of SI: Yes - had heard of SI before	Coding phase: 2. Double coded	Client marital status: N/A - provider	Client age group: N/A - provider	Time since trained:

Codes Applied:	P3.1.5 - Ease of training clients in SI	C1.2.2 - Fear of SI (idea or reality)	C3.1.2 - Confidence/comfort to inject after counselling/ training

Q12B. Interviewer: In your experience what is the response when you explain to clients that they could self-inject this method?

Respondent: At first, they show some resistance that they cannot manage to self-inject, but after the counselling and ask them to do it while we are watching them at least they find it simple and easy.

Title: MZs_HSA_1.docx

Descriptor Info:	District: Mzimba South	Target Population: TP1 - Providers/HSAs	Respondent type: HSA	Aware of SI: Yes - had heard of SI before	Coding phase: 2. Double coded	Client marital status: N/A - provider	Client age group: N/A - provider	Time since trained:

Codes Applied:	P3.1.5 - Ease of training clients in SI	C1.2.2 - Fear of SI (idea or reality)	C3.1.2 - Confidence/comfort to inject after counselling/ training

Interviewer: Do women have concerns about self-injection?

Respondent: At first yes, but once we have counsel them on how they can self-inject themselves they no longer have concerns.

Interviewer: Alright.

Respondent: They only have concerns before the counselling.

Title: MZs_HSA_1.docx

Descriptor Info:	District: Mzimba South	Target Population: TP1 - Providers/HSAs	Respondent type: HSA	Aware of SI: Yes - had heard of SI before	Coding phase: 2. Double coded	Client marital status: N/A - provider	Client age group: N/A - provider	Time since trained:

Codes Applied:	P3.4 - Time to counsel/train, general	P3.1.5 - Ease of training clients in SI	P3.2.3 - Complexity/difficulty of training clients in SI

Q14. Interviewer: How long on average does it take to train a client on DMPA-SC?

Respondent: Mmmmmm… (thinking), 30 minutes but sometimes 30-45 minutes.

Interviewer: Why are you saying sometimes?

Respondent: It’s because clients are different, others it’s very easy to learn while others they are not

Interviewer: Alright. What about to the adolescent?

Respondent: It’s the same as adults

Interviewer: Is it the same?

Respondent: Yes.

Interviewer: Do you feel you have enough time to conduct these trainings?

Respondent: Yes.

Title: KK_FAC_1.docx

Descriptor Info:	District: Nkhotakota	Target Population: TP1 - Providers/HSAs	Respondent type: Facility-based provider	Aware of SI: Yes - had heard of SI before	Coding phase: 3. Triple coded	Client marital status: N/A - provider	Client age group: N/A - provider	Time since trained: A year or more ago

Codes Applied:	P3.1.5 - Ease of training clients in SI	P3.3.1 - Differences in counselling/training approach by population group	P3.2.3 - Complexity/difficulty of training clients in SI

INTERVIEWER: Alright fine, how easy or difficult is it to train clients in DMPA for self-injection

RESPONDENT: The understanding of adults is a bit complex

INTERVIEWER: Okay

RESPONDENT: Hence it becomes difficult for them to grasp the information easily as such you have to say it again and again. It’s different from adolescents when you train them, they capture the info easily. However, we try our best that if a client chose a method we should drill her until she understands it.

INTERVIEWER: What about specifically for adolescents?

RESPONDENT: I have explained they are so fast to understand something

Title: KK_FAC_1.docx

Descriptor Info:	District: Nkhotakota	Target Population: TP1 - Providers/HSAs	Respondent type: Facility-based provider	Aware of SI: Yes - had heard of SI before	Coding phase: 3. Triple coded	Client marital status: N/A - provider	Client age group: N/A - provider	Time since trained: A year or more ago

Codes Applied:	C1.1.2 - Baseline correct knowledge/ awareness of injectables	P3.3.1 - Differences in counselling/training approach by population group	P3.1.5 - Ease of training clients in SI

INTERVIEWER: Do you think having previous experience with injectables makes a woman more less likely to consider self-injection

RESPONDENT: To have what?

INTERVIEWER: Having previous experience like knowledge, for instance in the past the client was taking depo so with such kind of experience

RESPONDENT: Okay

INTERVIEWER: can it help a woman to choose or not to choose self-injection?

RESPONDENT: It helps since one has knowledge in injectables already

INTERVIEWER: Okay

RESPONDENT: So it feels like you are just shifting someone from what she already knew to a similar concepts but just with few changes in that the other one will require you to inject yourself though it also works in three months. Hence it becomes easily because it’s just that you have added a little information on what she already knew.

INTERVIEWER: It differs from one who have not heard about injectables before.

RESPONDENT: Yes

Title: MZs_HSA_5.docx

Descriptor Info:	District: Mzimba South	Target Population: TP1 - Providers/HSAs	Respondent type: HSA	Aware of SI: Yes - had heard of SI before	Coding phase: 1. Single coded	Client marital status: N/A - provider	Client age group: N/A - provider	Time since trained: A year or more ago

Codes Applied:	P3.2.3 - Complexity/difficulty of training clients in SI	P3.1.5 - Ease of training clients in SI

Interviewer: how easy or difficult was it to counsel women and adolescents on Sayana for self-injection?

Respondent: for adolescents it is easy to teach them because most of the information they had already learnt at school so for me I just add and emphasize the point. For those who have no education it is difficult even for demonstration of a demo pen they find it as a taboo

Title: MZs_HSA_5.docx

Descriptor Info:	District: Mzimba South	Target Population: TP1 - Providers/HSAs	Respondent type: HSA	Aware of SI: Yes - had heard of SI before	Coding phase: 1. Single coded	Client marital status: N/A - provider	Client age group: N/A - provider	Time since trained: A year or more ago

Codes Applied:	P3.2.3 - Complexity/difficulty of training clients in SI

Interviewer: what about older women?

Respondent: it is difficult for those who have not attained any education

Title: KK_HSA_3.docx

Descriptor Info:	District: Nkhotakota	Target Population: TP1 - Providers/HSAs	Respondent type: HSA	Aware of SI: Yes - had heard of SI before	Coding phase: 3. Triple coded	Client marital status: N/A - provider	Client age group: N/A - provider	Time since trained: A year or more ago

Codes Applied:	P3.2.3 - Complexity/difficulty of training clients in SI	P4.2.3 - Provider lack of availability/ hesitancy to repeat SI training	C5.2.4 - Lack of provider willingness/availability to retrain

INTERVIEWER: Alright. In your own view, how easy or difficult is it to train a client for self-injection?

RESPONDENT: mmmmh.

INTERVIEWER: How easy or difficult is it?

RESPONDENT: The difficult part is that for these ones who have failed to self-inject, we take these as problems because we tell them to come back another day. If they have failed to self-inject, we inject them. But the problem comes in when they come again and they find us busy with other things, that is where the problem is. There is no problem for the ones who have passed because we have given them all the requirements; they have a calendar as a pointer that from here you will inject yourself here, you see. The women know what to do

Title: ZA_HSA_4.docx

Descriptor Info:	District: Zomba	Target Population: TP1 - Providers/HSAs	Respondent type: HSA	Aware of SI: Yes - had heard of SI before	Coding phase: 1. Single coded	Client marital status: N/A - provider	Client age group: N/A - provider	Time since trained: A year or more ago

Codes Applied:	P3.1.5 - Ease of training clients in SI

Interviewer: Alright in addition have you encountered any challenges in providing self-injection?

Respondent: From people

Interviewer: No when you administer

Respondent: Aah are no there’s no challenges that I have encountered

Title: ZA_HSA_4.docx

Descriptor Info:	District: Zomba	Target Population: TP1 - Providers/HSAs	Respondent type: HSA	Aware of SI: Yes - had heard of SI before	Coding phase: 1. Single coded	Client marital status: N/A - provider	Client age group: N/A - provider	Time since trained: A year or more ago

Codes Applied:	P3.1.5 - Ease of training clients in SI	P3.2.1 - Lack of IEC/demo materials for counselling/training

Interviewer: Ohh alright fine. How is difficult or (not difficult) to train the women on the self-injecting method?

Respondent: It is not difficult. It’s just that the women have fears because we just tell them how they are supposed to be doing it step by step.

Interviewer: Alright alright. Do you use any other resources for example those that help condoms full of sugar or sand to make the women easily understand what is being said?

Respondent: I have never done that before using the condom, I just explain the things to them.

Title: KK_HSA_4.docx

Descriptor Info:	District: Nkhotakota	Target Population: TP1 - Providers/HSAs	Respondent type: HSA	Aware of SI: Yes - had heard of SI before	Coding phase: 1. Single coded	Client marital status: N/A - provider	Client age group: N/A - provider	Time since trained: A year or more ago

Codes Applied:	P3.2.3 - Complexity/difficulty of training clients in SI

INTERVIEWER: Alright fine, have you faced any challenge providing DMPA-SC in the past three months?

RESPONDENT: Yes, we had some problems as said earlier, the method was a bit new to clients.

INTERVIEWER: Ok

RESPONDENT: Some clients still had problems with self-injection in that during the first injection they managed so well to do it since the provider was present. But after given a take away unit, they could not manage to do it on their own.

INTERVIEWER: Ok

RESPONDENT: Hence they had to come back for assistance

INTERVIEWER: Ok

RESPONDENT: We used to come across such incidences very often in the earlier days but now it has reduced.

INTERVIEWER: Alright

Title: MZs_HSA_4.docx

Descriptor Info:	District: Mzimba South	Target Population: TP1 - Providers/HSAs	Respondent type: HSA	Aware of SI: Yes - had heard of SI before	Coding phase: 1. Single coded	Client marital status: N/A - provider	Client age group: N/A - provider	Time since trained:

Codes Applied:	P3.2.3 - Complexity/difficulty of training clients in SI

Respondent: some problems faced were that some clients had difficulties to understand about self-injection

Title: ZA_FAC_1.docx

Descriptor Info:	District: Zomba	Target Population: TP1 - Providers/HSAs	Respondent type: Facility-based provider	Aware of SI: Yes - had heard of SI before	Coding phase: 2. Double coded	Client marital status: N/A - provider	Client age group: N/A - provider	Time since trained: Less than a year ago

Codes Applied:	P3.2.3 - Complexity/difficulty of training clients in SI	C5.2.2 - Clients incorrectly / failing to administer at home	C4 - Storage of unused units

Interviewer: Alright, are there any problems that you have faced in providing DMPASC in the last three months in addition to the stock out issue?

Respondent: the problem that I have observed is that it required a client who could understand

Interviewer: mmmm (showing that one is following the conversation)

Respondent: for a client who has difficulty in listening and remembering what to do it’s an issue now because I have received complaints like my injection unit has been messed up

Interviewer: mmmm (showing that one is following the conversation)

Respondent: mine is broken

Interviewer: mmmm (showing that one is following the conversation)

Respondent: mine has been eaten by rats, so such issues required that when you advise the client on storage of DMPA-SC, they should understand and follow that, but for those clients who did not listen, you’d wonder for example someone who has come to get medication and we have given them pills in the pill pack

Interviewer: mmmm (showing that one is following the conversation)

Respondent: If you follow that client at home, you’ll find that the pill pack is already dirty, but it has just been given to them on the same day.

Interviewer: yah yah

Respondent: Some will finish the whole dosage and the pill pack looks just as clean. So it requires someone who understands and should practice what has been said.

Interviewer: mmmm (showing that one is following the conversation)

Respondent: the complaints that we have received are that before the client injects themselves

Interviewer: mmmm (showing that one is following the conversation)

Respondent: they would find that they have broken the tube in the injection unit

Interviewer: mmmm (showing that one is following the conversation)

Respondent: and the liquid in the tube has been lost. They would come and say that they want another injection unit since the one that they were given at first have been destroyed.

Interviewer: mmmm (showing that one is following the conversation)

Respondent: after we probed into those cases, we found that before injecting themselves they had inflated the tube.

Interviewer: mmmm (showing that one is following the conversation)

Respondent: so it requires that the client should be someone who listens and understands. Some are concerned that they have fears, things like those.

Interviewer: mmmm (showing that one is following the conversation)

Respondent: eh but they managed to self-inject

Interviewer: alright.

Respondent: eya those are the small things, maybe the feedback I can say we have encountered so far

Title: ZA_FAC_1.docx

Descriptor Info:	District: Zomba	Target Population: TP1 - Providers/HSAs	Respondent type: Facility-based provider	Aware of SI: Yes - had heard of SI before	Coding phase: 2. Double coded	Client marital status: N/A - provider	Client age group: N/A - provider	Time since trained: Less than a year ago

Codes Applied:	P3.2.3 - Complexity/difficulty of training clients in SI	P3.4 - Time to counsel/train, general

Interviewer: Alright, have you experienced any problems in training women on DMPA-SC?

Respondent: yah I think the many questions, when we start training women on Sayana we just know that we will spend more time with them due to the fears that are there.

Interviewer: yah yah

Respondent: since it’s a new thing and the clients cannot just accept it on the word go.

Interviewer: mmmm (showing that one is following the conversation)

Respondent: the client already knows about the other methods, but for Sayana they say that it’s to them and you need to take time on it, but still more there is a benefit in that.

Title: ZA_FAC_2.docx

Descriptor Info:	District: Zomba	Target Population: TP1 - Providers/HSAs	Respondent type: Facility-based provider	Aware of SI: Yes - had heard of SI before	Coding phase: 3. Triple coded	Client marital status: N/A - provider	Client age group: N/A - provider	Time since trained: Less than a year ago

Codes Applied:	C1.1.3 - Awareness of DMPA-SC SI	P88 - Provider great quotes	P3.2.3 - Complexity/difficulty of training clients in SI

Interviewer: Okay, fine. How has the administration of the self-injection method been at this facility or in the surrounding communities where you conduct an outreach?

Respondent: In the beginning, it was hard and most women were failing to understand the concept but reaching this far, most women are able to do it on their own as some come and ask for the self-injection claiming they already do it on their own. Some also come to ask for the method claiming to have heard it from others. This shows that the message about self-injection method is spreading to more communities which was a challenge at first.

Title: ZA_FAC_2.docx

Descriptor Info:	District: Zomba	Target Population: TP1 - Providers/HSAs	Respondent type: Facility-based provider	Aware of SI: Yes - had heard of SI before	Coding phase: 3. Triple coded	Client marital status: N/A - provider	Client age group: N/A - provider	Time since trained: Less than a year ago

Codes Applied:	P3.2.3 - Complexity/difficulty of training clients in SI

Interviewer: Okay, thank you very much. How hard or simple is it to teach women to administer the drug on their own through self-injecting?

Respondent: It is hard since the level of understanding is different among the women because for some women to teach them to reach a point where they have understood it takes time while others understand quickly. So we make sure that health education happens every day. In addition, both group and individual counselling should not be done with the mindset that one is rushing, because there may be confusion. So we try to assess the women through individual counselling to know if it will be possible to do it or not.

Interviewer: How hard is it to teach the youth this method?

Respondent: Most youth mostly don’t have time. It happens that sometimes the time we’re giving the talk, they’re rushing to do their own things and they sometimes try to avoid people them. So what we do is we gather all the youth together so we can help them as the youth on their own. Sometimes being free to express oneself in a group can be a challenge because they think, “If so and so sees me they will report them to their homes. So we want to priotize the youth so we can help them quickly, separately so that the goal is achieved.

Title: MZs_HSA_3.docx

Descriptor Info:	District: Mzimba South	Target Population: TP1 - Providers/HSAs	Respondent type: HSA	Aware of SI: Yes - had heard of SI before	Coding phase: 3. Triple coded	Client marital status: N/A - provider	Client age group: N/A - provider	Time since trained: Less than a year ago

Codes Applied:	P3.2.3 - Complexity/difficulty of training clients in SI

Q. 13 Interviewer: how easy or difficult is it to train clients in DMPA-SC for self-injection?

Respondent: [moment of silence] ….of course it is easy but it becomes difficult when they come to practice. Yah you can talk to her you train her but when you say now can you provide yourself… then that’s why there comes now a problem but after injecting themselves the one who does it they say aah this is simple just give me I will go and do.

Title: KK_HSA_2.docx

Descriptor Info:	District: Nkhotakota	Target Population: TP1 - Providers/HSAs	Respondent type: HSA	Aware of SI: Yes - had heard of SI before	Coding phase: 2. Double coded	Client marital status: N/A - provider	Client age group: N/A - provider	Time since trained: A year or more ago

Codes Applied:	P3.2.3 - Complexity/difficulty of training clients in SI

INTERVIEWER: Mhh alright, are women uncomfortable / not confident to inject themselves?

RESPONDENT: Yes they seem to be uncomfortable since the places where they are supposed to inject themselves are secrete places. Hence they feel should one see my secret parts. the main problem is most of the service providers are men and we are dealing with women, there won't be a big problem if its women to women but in most cases counselling is done by us men hence it becomes difficult on that part.

INTERVIEWER: Mhhh alright

RESPONDENT: There is a problem for sure

INTERVIEWER: Mhhh alright

RESPONDENT: It becomes so difficult especially when you tell them to show the place where they need to inject themselves. Even if you can explain that its okay there is nothing wrong to expose such parts of their body

Title: ZA_HSA_3.docx

Descriptor Info:	District: Zomba	Target Population: TP1 - Providers/HSAs	Respondent type: HSA	Aware of SI: Yes - had heard of SI before	Coding phase: 1. Single coded	Client marital status: N/A - provider	Client age group: N/A - provider	Time since trained: A year or more ago

Codes Applied:	P3.1.5 - Ease of training clients in SI

Interviewer: How difficult is it to train a woman how to use the self-injection method?

Respondent: It is not hard to train since we use demonstrations

Title: MZs_FAC_2.docx

Descriptor Info:	District: Mzimba South	Target Population: TP1 - Providers/HSAs	Respondent type: Facility-based provider	Aware of SI: Yes - had heard of SI before	Coding phase: 2. Double coded	Client marital status: N/A - provider	Client age group: N/A - provider	Time since trained:

Codes Applied:	P3.1.5 - Ease of training clients in SI

Respondent: But after counselling, they end up taking the method and inject themselves and they even say that its simple.

Interviewer: So all these that you have mentioned, do you think they are the ones that makes them to go for Sayana?

Respondent: Yes

Interviewer: Why do you think so?

Respondent: This is so because the needle is small even though at first, they seem as if they cannot manage to do it but later on they say that its simple and they can do.

Interviewer: Do woman have any concerns about self-injection?

Respondent: Aaaaaa… (thinking), if you do the counselling very well, they do not have any concerns as long as they have understood every procedure on how they can self-inject and also know the next date to self- inject in doing so they will not forget.

Title: MZs_FAC_2.docx

Descriptor Info:	District: Mzimba South	Target Population: TP1 - Providers/HSAs	Respondent type: Facility-based provider	Aware of SI: Yes - had heard of SI before	Coding phase: 2. Double coded	Client marital status: N/A - provider	Client age group: N/A - provider	Time since trained:

Codes Applied:	P3.2.3 - Complexity/difficulty of training clients in SI

Interviewer: Are women uncomfortable/not confident to inject themselves?

Respondent: Mmmmm…. (yes), some clients find it difficult to inject themselves

Title: MZs_HSA_6.docx

Descriptor Info:	District: Mzimba South	Target Population: TP1 - Providers/HSAs	Respondent type: HSA	Aware of SI: Yes - had heard of SI before	Coding phase: 1. Single coded	Client marital status: N/A - provider	Client age group: N/A - provider	Time since trained: A year or more ago

Codes Applied:	P3.1.5 - Ease of training clients in SI	C1.1.2 - Baseline correct knowledge/ awareness of injectables	A1.1 - Awareness of DMPA-SC SI

Interviewer: how easy or difficult is it to counsel a client on Sayana for self-injection

Respondent: it is easy because most of them have already made a decision at home. So when you are counselling you understand each other well

Interviewer: what about adolescents

Respondent: similarly, they already had made the decision at home. So you easily understand each other.

Title: MZs_HSA_6.docx

Descriptor Info:	District: Mzimba South	Target Population: TP1 - Providers/HSAs	Respondent type: HSA	Aware of SI: Yes - had heard of SI before	Coding phase: 1. Single coded	Client marital status: N/A - provider	Client age group: N/A - provider	Time since trained: A year or more ago

Codes Applied:	P3.1.5 - Ease of training clients in SI

Interviewer: do those who were on Depo IM easily switch to Sayana compared to others on other methods of family planning

Respondent: it just depends on the counselling you give. If well counselled,

Interviewer: do those who were already on Depo easily switch to Sayana

Respondent: yes, because they had already received a similar counselling

Title: KK_HSA_6.docx

Descriptor Info:	District: Nkhotakota	Target Population: TP1 - Providers/HSAs	Respondent type: HSA	Aware of SI: Yes - had heard of SI before	Coding phase: 1. Single coded	Client marital status: N/A - provider	Client age group: N/A - provider	Time since trained: A year or more ago

Codes Applied:	P3.1.5 - Ease of training clients in SI	C2.1.2 - Reduced trips to facility	C1.1.1 - Positive client attitudes to SI	C8.3 - Discretion of injectables/SI

INTERVIEWER: Why do you think Sayana is the most common among women?

RESPONDENT: Mmmhu, Sayana is the most common because when we counsel the women, they feel that it is an easy method for them. There are also some who are on contraceptives without the knowledge of the husband and they feel that when we train them, it is easy for them to hide the drugs when we give them. But for the provider injectable, there is no privacy. It is also good because when we train them and they understand, sometimes they ask for more doses maybe for 9 months because during the rainy season, they have to cross rivers to get to an HSA. It’s only a handful that are provider injected these days. Most women are self-injecting at home. It is simple than Depo Provera and that is why it is common.

Title: KK_HSA_6.docx

Descriptor Info:	District: Nkhotakota	Target Population: TP1 - Providers/HSAs	Respondent type: HSA	Aware of SI: Yes - had heard of SI before	Coding phase: 1. Single coded	Client marital status: N/A - provider	Client age group: N/A - provider	Time since trained: A year or more ago

Codes Applied:	P3.2.3 - Complexity/difficulty of training clients in SI	A6 - Adol Perspectives/preferences for DMPA-SC SI	P3.3.1 - Differences in counselling/training approach by population group

INTERVIEWER: How easy or difficult is it to train clients in DMPA-SC?

RESPONDENT: Most of the women have experienced a lot of pain especially during child birth than adolescents. Training an adolescent is difficult than a woman because she knows that the pain will be for a short while than that of child birth. They understand fast than girls.

INTERVIEWER: okay.

RESPONDENT: yea.

Title: MZs_FAC_1.docx

Descriptor Info:	District: Mzimba South	Target Population: TP1 - Providers/HSAs	Respondent type: Facility-based provider	Aware of SI: Yes - had heard of SI before	Coding phase: 3. Triple coded	Client marital status: N/A - provider	Client age group: N/A - provider	Time since trained: A year or more ago

Codes Applied:	P88 - Provider great quotes	P1.1.1 - Positive provider attitudes towards SI	P3.1.5 - Ease of training clients in SI

Interviewer: Okay, thank you so much. Aaaah you have said something like your opinion or your colleagues opinion on self-injection has changed

Respondent: Mmmmh (yes)

Interviewer: So I want to understand specifically what has influence your opinion to change?

Respondent: First is the simplicity of the injection itself is quiet that now I know that with good counselling anybody can understand it and even the woman that have received this thing they are willingly taking it so I really know that they do understand it that it is a simple thing and they can do it.

Interviewer: Mmmmh

Respondent: The simplicity is that the way they simplified that injection is the simple thing

Title: MZs_FAC_1.docx

Descriptor Info:	District: Mzimba South	Target Population: TP1 - Providers/HSAs	Respondent type: Facility-based provider	Aware of SI: Yes - had heard of SI before	Coding phase: 3. Triple coded	Client marital status: N/A - provider	Client age group: N/A - provider	Time since trained: A year or more ago

Codes Applied:	P3.4 - Time to counsel/train, general	P3.1.4 - Provider sufficient support to counsel/train on SI	P3.1.5 - Ease of training clients in SI

Q 13. Interviewer: How easy or difficult it to train client in DMPA-SC for self-injection?

Respondent: Is not so difficult only that it really need time.

Interviewer: Mmmmmm.

Respondent: To explain to the client, you need to have uninterrupted time and also sometimes nurses are busy and a client come and say I need injection and you are busy, then you just refer them to the HSA`s to assist because you need to sit down and start explain all the procedures up until you are convinced that she can do it. So it’s not that difficult but you just need time to do it.

Title: ZA_HSA_1.docx

Descriptor Info:	District: Zomba	Target Population: TP1 - Providers/HSAs	Respondent type: HSA	Aware of SI: Yes - had heard of SI before	Coding phase: 1. Single coded	Client marital status: N/A - provider	Client age group: N/A - provider	Time since trained: Less than a year ago

Codes Applied:	P3.2.3 - Complexity/difficulty of training clients in SI	P3.1.5 - Ease of training clients in SI

Interviewer: Alright thank you very much, how easy or difficult is it to train clients in DMPA-SC for self-injection

Respondent: It all depends on the person; others find it easy to understand. Whilst others say that they won’t be able to manage please inject me will be coming to get injected.

Interviewer: Alright

Respondent: It all depends on the person

Title: MZs_HSA_2.docx

Descriptor Info:	District: Mzimba South	Target Population: TP1 - Providers/HSAs	Respondent type: HSA	Aware of SI: Yes - had heard of SI before	Coding phase: 1. Single coded	Client marital status: N/A - provider	Client age group: N/A - provider	Time since trained: A year or more ago

Codes Applied:	P3.2.3 - Complexity/difficulty of training clients in SI

Interviewer: how easy or difficult is it to teach a woman who has come to get family planning methods

Respondent: its only difficult when a woman can’t understand what we teach. Some are afraid to self-inject. After teaching them we ask them to self-inject under direct observation. So most of them do not really have fears they manage to self-inject.

Title: KK_FAC_2.docx

Descriptor Info:	District: Nkhotakota	Target Population: TP1 - Providers/HSAs	Respondent type: Facility-based provider	Aware of SI: Yes - had heard of SI before	Coding phase: 1. Single coded	Client marital status: N/A - provider	Client age group: N/A - provider	Time since trained: A year or more ago

Codes Applied:	P2.3 - Provider training content	P3.1.1 - Reduced provider workload	C2.1.2 - Reduced trips to facility	P3.1.5 - Ease of training clients in SI

INTERVIEWER: Aaa we are continuing. I will ask you questions on your experiences during DMPA-SC training.

RESPONDENT: mmmhu.

INTERVIEWER: Aaah, can you tell me a little bit about the training you received in providing DMPA-SC? What training did you receive?

RESPONDENT: The training that we received regarding the self-injectable is how to counsel a client. On this on the counselling also training them on how they can self-inject and also how the self-injecting kit is so that when self-injecting they should not face any problems.

INTERVIEWER: okay.

RESPONDENT: mmmh.

INTERVIEWER: Alright. So what were the good parts of the training? [someone came in and disturbed us] continue, you can continue. Yeah.

RESPONDENT: Where were we?

INTERVIEWER: I asked about, wait [he came in again]. I asked you about the good parts of the training…training about Sayana?

RESPONDENT: Okay. There are a lot of good things; firstly, the kit for injecting the Sayana drug is very interesting because it has different parts that interests when learning. The other is that through that training, it reduced our workload because when we teach them and they accept to be self-injecting, we can give them up to four doses including the one they have injected at the facility. Which means they will have fewer visits to the hospital and also we have reduced our work load.

INTERVIEWER: Okay.

RESPONDENT: mmmh.

INTERVIEWER: Mmmhu. If there could be another training for instance right, what would you like to be improved in the training?

Time stamp: (10:09)

RESPONDENT: In my view, that training was just fine.

INTERVIEWER: mmmhu.

RESPONDENT: Because after the training we came to the facility and we started providing, we did not face any problems.

INTERVIEWER: okay.

RESPONDENT: yes.

INTERVIEWER: everything was…

RESPONDENT: And the questions that people were asking we were answering them very well.

Title: KK_FAC_2.docx

Descriptor Info:	District: Nkhotakota	Target Population: TP1 - Providers/HSAs	Respondent type: Facility-based provider	Aware of SI: Yes - had heard of SI before	Coding phase: 1. Single coded	Client marital status: N/A - provider	Client age group: N/A - provider	Time since trained: A year or more ago

Codes Applied:	P3.1.5 - Ease of training clients in SI

INTERVIEWER: Okay, alright. What in your experience is the response when you explain to clients that they could self-inject at home? When it is their first time, what do they say?

Time stamp: (30:16)

RESPONDENT: Oh, at first the response is, self-injecting? It is difficult I cannot manage to self-inject. Then we continue by saying that self-injecting is difficult and also not difficult. But if we explain to you, you will see that it is not difficult. Then we explain to them and also how Sayana works. Then they say, I should just try and they self-inject. And then I ask them if it will be difficult to self-inject at home because they have done it here, and they say no. so what should we do? Just give me some I will self-inject at home.

INTERVIEWER: mmmmhu.

RESPONDENT: Yea. That’s how it happens.

Title: KK_FAC_2.docx

Descriptor Info:	District: Nkhotakota	Target Population: TP1 - Providers/HSAs	Respondent type: Facility-based provider	Aware of SI: Yes - had heard of SI before	Coding phase: 1. Single coded	Client marital status: N/A - provider	Client age group: N/A - provider	Time since trained: A year or more ago

Codes Applied:	P3.3 - Counselling/ training approach, general	P3.1.5 - Ease of training clients in SI	P3.1.3 - IEC/demo materials for counselling/training

INTERVIEWER: Alright. How difficult or easy is it to train a woman to self-inject? How difficult or easy is it to train a woman about Sayana?

RESPONDENT: Teaching a woman about Sayana is not difficult according to me.

INTERVIEWER: How about self-injecting, the process of training them to self-inject is it easy or difficult?

RESPONDENT: The process is not difficult to teach them. But the practical part of self-injecting is up to that person. That is where we keep on repeating until she gets it. Yeah.

INTERVIEWER: Do you use any resources when teaching them self-injection?

RESPONDENT: We use flexa foams

INTERVIEWER: okay.

RESPONDENT: mmmh.

INTERVIEWER: What happens with these flexa foams?

RESPONDENT: flexa foam?

INTERVIEWER: mmmh.

RESPONDENT: We take the Sayana syringe and then we explain to them that you should hold the syringe like this on the neck. Don’t hold on the bulb because it has a bulb. They should not hold that part because if they do, Sayana will spill.

INTERVIEWER: mmmhu.

RESPONDENT: Yeah. And we tell them to put the syringe at an angle they can see. They are supposed to inject above the belly button or on a left or right thigh.

INTERVIEWER: mmmhu.

RESPONDENT: Yeah. But if they are being injected by the provider, it’s on the arm.

INTERVIEWER: Okay. Do you use condoms filled with sugar when training the women?

RESPONDENT: We don’t use condoms filled with sugar. We just use them as a demonstration.

INTERVIEWER: Okay. What do you put inside the condom?

RESPONDENT: condom?

INTERVIEWER: Yes…no…I mean…when others are training clients, they fill a condom with sugar for the women to try as if they are injecting the Sayana. Do you use the condom?

RESPONDENT: Like a condom?

INTERVIEWER: mmmmh.

RESPONDENT: No we don’t use a condom.

INTERVIEWER: You don’t?

RESPONDENT: mmm mmm [no].

INTERVIEWER: But you say you demonstrate with a flexa foam?

RESPONDENT: mmmh a flexa foam.

INTERVIEWER: The flexa foam in this case is a thigh?

RESPONDENT: Yes, a small one.

INTERVIEWER: okay.

RESPONDENT: mmmh.
