# Supplementary material for: Health care provider and client experiences of counselling on depot medroxyprogesterone acetate subcutaneous (DMPA-SC) for self-injection in Malawi
Source: PLOS Glob Public Health. 2023 Nov 30;3(11):e0002057. doi: 10.1371/journal.pgph.0002057 (PMC10688740; doi:10.1371/journal.pgph.0002057)
Supplement: S1 Data — (ZIP) [file pgph.0002057.s001.zip › S2/P3.2 oth barriers to prov integration.doc]

Title: ZA_HSA_2.docx

Descriptor Info:	District: Zomba	Target Population: TP1 - Providers/HSAs	Respondent type: HSA	Aware of SI: Yes - had heard of SI before	Coding phase: 3. Triple coded	Client marital status: N/A - provider	Client age group: N/A - provider	Time since trained: Less than a year ago

Codes Applied:	P3.2 - Barrier, general/other

Respondent: Yes, because there are some things that seem to have been forgotten among us…

Interviewer: mmm (Showing that one is following the conversation)

Respondent: Yes, maybe we are just doing short cuts because we have forgotten….

Interviewer: mmm (showing that one is following the conversation))

Respondent: Yes, so if we have the refresher courses more often, yeah, they could encourage us in our work…

Interviewer: mmm (Showing agreement)

Respondent: Yeah, this is very important, for example some of our fellow HSAs, even just administering DMPA-IM… yeah…

Interviewer: mmm (showing agreement)

Respondent: Some of the HSAs in the communities they say that we have had enough of this, eh it seems that the pay is not enough (Laughs)

Title: ZA_HSA_2.docx

Descriptor Info:	District: Zomba	Target Population: TP1 - Providers/HSAs	Respondent type: HSA	Aware of SI: Yes - had heard of SI before	Coding phase: 3. Triple coded	Client marital status: N/A - provider	Client age group: N/A - provider	Time since trained: Less than a year ago

Codes Applied:	A5.2 - Stigma/judgement	P3.2 - Barrier, general/other

Respondent: But when we spend some time with them in their first visit, we inject them, the second time we also inject them, because we don’t just tell them to go and inject themselves, no…

Interviewer: mmm (showing that one is following the conversation)

Respondent: But we first see the clients who have the capability that they have accepted the training, and they accept that I will be able to self-inject at home…

Interviewer: mmm (showing that one is following the conversation)

Respondent: Yes so, we still continue, even though the person has come, and we have trained them…

Interviewer: mmm (showing that one is following the conversation)

Respondent: If it’s the case that they are not satisfied that they can be able to self-inject, when they come again, we are able to tell them, when they accept that now, because some of them do things because their friends have done them…

Title: ZA_HSA_2.docx

Descriptor Info:	District: Zomba	Target Population: TP1 - Providers/HSAs	Respondent type: HSA	Aware of SI: Yes - had heard of SI before	Coding phase: 3. Triple coded	Client marital status: N/A - provider	Client age group: N/A - provider	Time since trained: Less than a year ago

Codes Applied:	P3.4 - Time to counsel/train, general	P3.2 - Barrier, general/other

Interviewer: In your opinion, do you think you have enough time to provide training to DMPA-SC clients?

Respondent: Ah, time is not always enough, like we say some should come in the morning, some in the late afternoon hours, at least those clients that come in the late afternoon hours we don’t have time, here at the facility we don’t knock of late due to transport issues, yeah…

Interviewer: Yah yah

Respondent: Yes, so when the vehicle comes to pick us from the facility you will find that around 2PM or 3PM we are knocking off…

Interviewer: mm (showing that one is following the conversation)

Respondent: Yes, so starting from 2PM, 3PM up to 4:30PM or 5:00PM …

Interviewer: eh

Respondent: .is the time that we, the clients come, we got them used to that time…

Interviewer: mmm (showing that one is following the conversation)

Respondent: So, it depends what time the client has come to the facility, some of the clients come at dusk…

Interviewer: mm (showing that one is following the conversation)

Respondent: Yes, so at dusk it is very hard for us to train the client. But we are still able to assist the client because some of the clients come late because they already know the methods, yeah…

Interviewer: mm (Showing that one is following the conversation)

Respondent: and some of them have jobs, they go to work early and knock off late so we accept them, that they can come late here at the facility. But if they need to be trained and they want to know more…

Interviewer: mm (showing that one is following the conversation)

Respondent: We can still discuss with them,

Interviewer: mmmhh (showing that one is following the conversation)

Respondent: Sure, sure.

Interviewer: Alright alright, ah on the days that you are pre-occupied on your job, does the time that you spend with the clients on training them differ? Just comparing with the other days when you are not pre-occupied.

Respondent: ah to be honest, the thing is like that but in this area, we are not that busy a lot…

Interviewer: mm (showing that one is following the conversation)

Respondent: No.

Interviewer: Alright

Respondent: so, we have the time to chat with the clients…

Title: KK_HSA_1.docx

Descriptor Info:	District: Nkhotakota	Target Population: TP1 - Providers/HSAs	Respondent type: HSA	Aware of SI: Yes - had heard of SI before	Coding phase: 2. Double coded	Client marital status: N/A - provider	Client age group: N/A - provider	Time since trained: A year or more ago

Codes Applied:	P3.2 - Barrier, general/other

Interviewer: Mmmhu. Apart from this refresher right, have you received any other follow up training concerned with DMSC-SC in the community?

Respondent: Mmmh mmmh. We said back then that we need bags right. So that let’s say they should give us bags because when you are in the field, you need to take good care of your things. The registers are big but they did not…there is no answer until today.

Interviewer: mmmhu

Respondent: mmh. The register is quite big. Every time you are going out, you have to carry it or put it on the bicycle, as a result, it does not take long to be destroyed. At least if there was a bag for you to be carrying the register, maybe it can last long.

Interviewer: mmmmmhu.

Respondent: Yes.

Title: KK_HSA_1.docx

Descriptor Info:	District: Nkhotakota	Target Population: TP1 - Providers/HSAs	Respondent type: HSA	Aware of SI: Yes - had heard of SI before	Coding phase: 2. Double coded	Client marital status: N/A - provider	Client age group: N/A - provider	Time since trained: A year or more ago

Codes Applied:	P2.1.1 - Refresher training/mentorship	P3.2 - Barrier, general/other

Interviewer: So what did you do at this refresher?

Respondent: At this refresher they chose…at first they chose representatives from different hospitals. A person should stand up and explain how they do their work. After that one, another one should also explain and another one also explained…in the end, they summarized and showed us our shortfalls. Yes.

Interviewer: mmmmhu.

Respondent: yes

Title: KK_HSA_1.docx

Descriptor Info:	District: Nkhotakota	Target Population: TP1 - Providers/HSAs	Respondent type: HSA	Aware of SI: Yes - had heard of SI before	Coding phase: 2. Double coded	Client marital status: N/A - provider	Client age group: N/A - provider	Time since trained: A year or more ago

Codes Applied:	P3.2 - Barrier, general/other

Interviewer: So what sort of support do you need? Apart from the bags for the registers, any other support that you need?

Respondent: Transport because the catchment is huge. Other HSA’s have a population of maybe 1000 or 1000 below. While others have 2000 above per one HSA. So when the catchment is scattered, it is difficult to to reach others in good time because it is the same job. But if you have transport, you can go around in your catchment.

Interviewer: mmmh

Respondent: yes

Interviewer: okay

Respondent: right

Title: KK_HSA_1.docx

Descriptor Info:	District: Nkhotakota	Target Population: TP1 - Providers/HSAs	Respondent type: HSA	Aware of SI: Yes - had heard of SI before	Coding phase: 2. Double coded	Client marital status: N/A - provider	Client age group: N/A - provider	Time since trained: A year or more ago

Codes Applied:	P3.2 - Barrier, general/other

Interviewer: Alright. We have come to the end of our discussion. Do you have any thoughts or anything that you would like to share on what we have discussed?

Respondent: Mmmmh (thinking). The thoughts are that; it is better that you have come, you have heard how we discuss with these people right?

Interviewer: mmmh.

Respondent: ….in the community how we are using this method. But what is required is that sometimes like the way we work in the field right, when they come for supervision or the bosses, on our work station, where we conduct our services from. They should be coming for supervision because that encourages us and they see that these people are indeed working. There is really a need for that bicycle or those bags. look at how they are keeping their things. But a lot of them when they do this, that’s it. Yes, although this goes to where it is supposed to right... but for us we deal with peoples’ lives.

Interviewer: mmmhu.

Respondent: Yes. A provider is also supposed to feel good. Yes, because of this they will sell the services to the community attractively.

Title: ZA_HSA_5.docx

Descriptor Info:	District: Zomba	Target Population: TP1 - Providers/HSAs	Respondent type: HSA	Aware of SI: Yes - had heard of SI before	Coding phase: 2. Double coded	Client marital status: N/A - provider	Client age group: N/A - provider	Time since trained: Less than a year ago

Codes Applied:	P3.2.3 - Complexity/difficulty of training clients in SI	P3.2 - Barrier, general/other

Interviewer: Alright, would you need to be supported on your work of providing Sayana Press?

Respondent: Yes, I would need the support especially the help would be like for these people to understand us it is very hard

Interviewer: Mmm (showing that one is following the conversation)

Respondent: Let me not lie, so for you to reach out to many women you need one on one sessions yeah

Interviewer: Mmm (showing that one is following the conversation)

Respondent: You should be able to explain to the client properly and also use a model who is using the method properly, so that maybe the women should easily receive it and they should make their friends to accept the method, maybe helping out those ones

Interviewer: Mmm (showing that one is following the conversation)

Respondent: They could be curious, and they could appreciate that this is okay and there are no problems, we cannot be administering the injection to each woman individually

Interviewer: Mmm (showing that one is following the conversation)

Respondent: But also, for those people who like the method, we should recruit those that they can assist their friends.

Interviewer: Mmm (showing that one is following the conversation)

Respondent: They should understand it

Interviewer: Alright.

Respondent: Yes

Title: ZA_HSA_5.docx

Descriptor Info:	District: Zomba	Target Population: TP1 - Providers/HSAs	Respondent type: HSA	Aware of SI: Yes - had heard of SI before	Coding phase: 2. Double coded	Client marital status: N/A - provider	Client age group: N/A - provider	Time since trained: Less than a year ago

Codes Applied:	P3.2 - Barrier, general/other

Interviewer: Alright, are there any other areas you need support in?

Respondent: On the issue of Sayana?

Interviewer: Yes

Respondent: Yes, they could be a need for more support

Interviewer: Mmm (showing that one is following the conversation)

Respondent: We don’t have bags

Interviewer: Okay

Respondent: And also, the villages we work in are very far

Interviewer: Mmm (showing that one is following the conversation)

Respondent: We have transportation problems

Interviewer: Mmm (showing that one is following the conversation)

Respondent: For example, like yesterday, if we could not have gone then we have disadvantaged many women.

Interviewer: Mmm (showing that one is following the conversation)

Respondent: Because yesterday we met almost 160 women

Interviewer: Mmm (showing that one is following the conversation)

Respondent: Those were just the ones accessing family planning and we inject them with Sayana

Interviewer: Eh

Respondent: Some of them take for self-injection which shows that more women want this method.

Interviewer: Mmm (showing that one is following the conversation)

Respondent: But maybe the failures on our side like on the issue of transportation

Interviewer: Mmm (showing that one is following the conversation)

Respondent: But also bags to carry our resources in

Interviewer: Mmm (showing that one is following the conversation)

Respondent: (pointing at the bag on the table) as you can see here, I have used this small bag

Interviewer: Yah

Respondent: Eh, yes

Interviewer: Alright

Respondent: Sure

Title: KK_HSA_5.docx

Descriptor Info:	District: Nkhotakota	Target Population: TP1 - Providers/HSAs	Respondent type: HSA	Aware of SI: Yes - had heard of SI before	Coding phase: 1. Single coded	Client marital status: N/A - provider	Client age group: N/A - provider	Time since trained: A year or more ago

Codes Applied:	P3.4 - Time to counsel/train, general	P3.1.2 - Provider skill/ability to effectively counsel on SI	P3.2 - Barrier, general/other	P4.1.3 - Provider availability/ willingness to repeat SI training

INTERVIEWER: Does the length of a training influence whether some women choose to take up self-injection?

RESPONDENT: The length of a training

INTERVIEWER: The process that takes in counselling the client does it influence whether some women choose to take up self-injection?

RESPONDENT: Yes, it does

INTERVIEWER: How does it influence?

RESPONDENT: When a client receives the right information through counselling

INTERVIEWER: Yes

RESPONDENT: Ndi pomweso amakhla ndi kuthekera kotenga

RESPONDENT: That’s when she has the urge to take the method. For instance, if the provider has counselled the clients so well in details, it becomes so easy for the clients when it comes for them to try to self-inject. But in cases where the provider does counsel the clients in a hurry without checking whether they are clear with the process. It becomes a problem for them when doing the trial injection.

INTERVIEWER: Alright

RESPONDENT: You may find some who will get the doses to take home, but because they did not understand well during counselling, they will just keep the units without using them. You may feel that thigs are fine, until when CBDA meets you to explain that your client is failing to use the injection you gave her, it’s when you discover that she just opened the unit. When asked she will tell you that the medication could not come out. Hence you start the counselling process all over again.

INTERVIEWER: Alright (Laugh)

RESPONDENT: Sure

Title: KK_HSA_3.docx

Descriptor Info:	District: Nkhotakota	Target Population: TP1 - Providers/HSAs	Respondent type: HSA	Aware of SI: Yes - had heard of SI before	Coding phase: 3. Triple coded	Client marital status: N/A - provider	Client age group: N/A - provider	Time since trained: A year or more ago

Codes Applied:	P3.2 - Barrier, general/other

RESPONDENT: Mmmmhu. Also you have asked me about what we could have loved to be done to improve, I have remembered one thing that according to distance or hard to reach areas, we are supposed to have good stationery for example bags and bicycle because we walk long distances to reach people.

INTERVIEWER: mmmmhu.

RESPONDENT: mmmh.

INTERVIEWER: So you are telling me that you have not received any other training since the initial training?

RESPONDENT: mmmh.

INTERVIEWER: So you would like support…would you like any support?

RESPONDENT: Yes, we need support as it is important.

INTERVIEWER: What kind of support?

RESPONDENT: Support for example logistics on how we are supposed to travel to reach people in villages like hard-to-reach areas. And also we have few stationery and bags to carry things and the drugs we have like Sayana, we are supposed to put in good carrier bags to reach people in a good condition.

Title: KK_HSA_3.docx

Descriptor Info:	District: Nkhotakota	Target Population: TP1 - Providers/HSAs	Respondent type: HSA	Aware of SI: Yes - had heard of SI before	Coding phase: 3. Triple coded	Client marital status: N/A - provider	Client age group: N/A - provider	Time since trained: A year or more ago

Codes Applied:	P3.2 - Barrier, general/other

INTERVIEWER: Alright. Do women have any concerns about self-injection?

RESPONDENT: No. women who…women who have accepted, the ones we have trained and we are convinced that these ones can self-inject, we give them. But the ones we see that these ones cannot do it, they are the ones with the concerns for self-injection. But the ones we have trained…because before we inject them, we first counsel and train them.

Title: KK_HSA_4.docx

Descriptor Info:	District: Nkhotakota	Target Population: TP1 - Providers/HSAs	Respondent type: HSA	Aware of SI: Yes - had heard of SI before	Coding phase: 1. Single coded	Client marital status: N/A - provider	Client age group: N/A - provider	Time since trained: A year or more ago

Codes Applied:	P3.2 - Barrier, general/other

INTERVIEWER: Alright, next question, you have already answered it, it’s about the concerns that most women have when they are first introduced to the method.

RESPONDENT: Yes

INTERVIEWER: Next question; how easy or difficult is it to train a client in DMPA-SC for self-injection? Is it difficult? If it so how difficult is it?

RESPONDENT: Yes, I would say it may be de difficult. The difficult part is that self-injection is done on three areas in the woman’s body.

INTERVIEWER: Alright.

RESPONDENT: The first place is on the thigh, the second one is at the back of the arm and lastly is around the navel. And the provider is a man who is counselling a woman who is married (participant laugh) that this method we also inject around the navel, hence she is supposed to pull up the clothes for you to show her how it’s done.

INTERVIEWER: At a particular place.

RESPONDENT: This method we may also administer it on the thigh, it’s not just a matter of explaining. Then you find that the woman would tell you she wants to do self-injection on the stomach and she had to pull down her clothes.

INTERVIEWER: Yes, true.

RESPONDENT: Yes

INTERVIEWER: Ok

RESPONDENT: This sometimes becomes a problem, however, they know that as health personnel we are obliged to keep secret of any issues about our clients.

INTERVIEWER: Ok

RESPONDENT: But now they don’t take is as a problem per se. Because nowadays some male doctors are assisting them in delivery.

INTERVIEWER: In giving birth.

RESPONDENT: So for us as male workers we are just skeptical, that may be the clients may feel uncomfortable that the provider had told me to undress for me to do self-injection.

INTERVIEWER: Yes

RESPONDENT: But by the look of things we feel they don’t take it as a problem

Title: KK_HSA_4.docx

Descriptor Info:	District: Nkhotakota	Target Population: TP1 - Providers/HSAs	Respondent type: HSA	Aware of SI: Yes - had heard of SI before	Coding phase: 1. Single coded	Client marital status: N/A - provider	Client age group: N/A - provider	Time since trained: A year or more ago

Codes Applied:	P3.2 - Barrier, general/other

INTERVIEWER: Alright, are women uncomfortable to inject themselves? After counselling them on how the method works and now you are telling them to try self-injection, since the parts for injection are the thighs and some private places. Do you see them uncomfortable to inject themselves?

RESPONDENT: Yes, as already explained that self-injection is done in private areas of the women’s body

INTERVIEWER: True

RESPONDENT: You will hear stories like the clothes are somehow tight, but the truth is they feel uncomfortable to take out the clothes to do self-injection.

Title: MZs_HSA_4.docx

Descriptor Info:	District: Mzimba South	Target Population: TP1 - Providers/HSAs	Respondent type: HSA	Aware of SI: Yes - had heard of SI before	Coding phase: 1. Single coded	Client marital status: N/A - provider	Client age group: N/A - provider	Time since trained:

Codes Applied:	P3.5 - Data capture, general	P3.2 - Barrier, general/other

Interviewer: is there anything which can help you in offering self-injection services?

Respondent: yes, we sometimes need a big bag to carry the supplies when going in the community.

Interviewer: apart from the bag is there anything which can help?

Respondent: yes, there are somethings in the register which we do not offer such as loop and Implanon as such if the register can be shortened to not include these may be helpful. We also need means of transport such as bikes. We may also need raincoats and umbrella during rainy season.

Interviewer: Mmmmh.

Title: ZA_FAC_1.docx

Descriptor Info:	District: Zomba	Target Population: TP1 - Providers/HSAs	Respondent type: Facility-based provider	Aware of SI: Yes - had heard of SI before	Coding phase: 2. Double coded	Client marital status: N/A - provider	Client age group: N/A - provider	Time since trained: Less than a year ago

Codes Applied:	P3.2 - Barrier, general/other

Interviewer: Alright, the training that you received on DMPASC, can you explain to me what was good or bad about the training?

Respondent: I think that if the training, could have been important especially on the part of information

Interviewer: mmmm (showing that one is following the conversation)

Respondent: the information that was provided to the providers show us that most of the people who received the information don't use the commodity

Interviewer: mmmm (showing that one is following the conversation)

Respondent: I would have loved if the information was also available to the resource persons

Interviewer: mmmm (showing that one is following the conversation)

Respondent: resource person

Interviewer: yah

Respondent: because when we provide the women with the method and they come back with feedback

Interviewer: mmmm (showing that one is following the conversation)

Respondent: we try to address the feedback but because we don't have resource persons who can give testimonies of their use and experience of Sayana

Interviewer: mmmm (showing that one is following the conversation)

Respondent: this is how it is, sometimes this is how I felt, for example here at the facility we may have a nurse who has used Norplant and she shows to the clients

Interviewer: yah

Respondent: ma provider but for this commodity Sayana, even the providers

Interviewer: mmmm (showing that one is following the conversation)

Respondent: ah I'm not seeing a lot of resource persons; we are lacking resource persons whom we can display to the community so that the women should have confidence that the method is just OK

Interviewer: mmmm (showing that one is following the conversation)

Respondent: the same way that we do with loop some are able to stand up and say that I have accessed loop and this is my 7th year I have never faced any problem. The biggest problem I see is the lack of resource persons. I don't know if the research about that has happened of course there are side effects and other things that we talk about but still the gap that is there a fear, if we had resource persons especially those that come from the community in the groups of women. the healthy surveillance assistance in their catchment areas they have volunteers if we could lobby the people like those to start using these methods, for example Depo, if they could use that one so that they could be a resource person on in their community then we can address the issues that are there, Some things are easily understood when it is someone from the community who has spoken on them and not a provider from the facility

Interviewer: yah

Respondent: eh I think that gap could have helped us

Title: ZA_HSA_6.docx

Descriptor Info:	District: Zomba	Target Population: TP1 - Providers/HSAs	Respondent type: HSA	Aware of SI: Yes - had heard of SI before	Coding phase: 1. Single coded	Client marital status: N/A - provider	Client age group: N/A - provider	Time since trained:

Codes Applied:	P3.2 - Barrier, general/other

Interviewer: Ah would you want to be assisted in any way?

Respondent: ah the support would be required

Interviewer: mmmm (showing that one is following the conversation)

Respondent: especially sometimes when we are meeting with community volunteers those who can help us mobilize the communities

Interviewer: mmmm (showing that one is following the conversation)

Respondent: if they also had a chance of being trained like the way that we are receiving.

Interviewer: mmmm (showing that one is following the conversation)

Respondent: it could also be helpful that they help us in mobilizing the people on the ground

Interviewer: yah yah

Respondent: It could be helpful that maybe the message should reach the people without delay so that they can also have the knowledge on how the program is.

Title: ZA_HSA_6.docx

Descriptor Info:	District: Zomba	Target Population: TP1 - Providers/HSAs	Respondent type: HSA	Aware of SI: Yes - had heard of SI before	Coding phase: 1. Single coded	Client marital status: N/A - provider	Client age group: N/A - provider	Time since trained:

Codes Applied:	P3.1 - Enablers, general/other	P3.2 - Barrier, general/other	P3.4 - Time to counsel/train, general

Interviewer: alright would the counselling differ if you are meeting the clients in the community or at the facility?

Respondent: ah it is a bit different.

Interviewer: mmmm (showing that one is following the conversation)

Respondent: because when we meet them in the community, they are ready that the providers are coming.

Interviewer: mmmm (showing that one is following the conversation)

Respondent: what am saying is that past 8 AM thy have already started gathering at the place of meeting.

Interviewer: yah yah

Respondent: but that time we have not yet started off from the facility.

Interviewer: mmmm (showing that one is following the conversation)

Respondent: they wait for us until we …

Interviewer: until you get there

Respondent: meaning that they are ready, and they are just waiting for us

Interviewer: mmmm (showing that one is following the conversation)

Respondent: at the facility, even though we are at the facility due to a large number of tasks to perform you tend to find that the people become bored, they see that you have been told to do other things

Interviewer: mmmm (showing that one is following the conversation)

Respondent: and you haven’t attended to them, it is a bit different with the way it is in the community

Interviewer: alright.

Respondent: for example, in the community it is possible to find 150 women,

Interviewer: yah

Respondent: they are waiting at one place for the providers, which shows that they don’t have a problem, and when its one day I the month they are very sure that the providers will come on such such a date

Title: MZs_HSA_6.docx

Descriptor Info:	District: Mzimba South	Target Population: TP1 - Providers/HSAs	Respondent type: HSA	Aware of SI: Yes - had heard of SI before	Coding phase: 1. Single coded	Client marital status: N/A - provider	Client age group: N/A - provider	Time since trained: A year or more ago

Codes Applied:	P3.2.2 - Stockouts/stocks near expiry dates	P3.2 - Barrier, general/other

Interviewer: did you have challenges due to covid-19 on providing Sayana

Respondent: with the coming of covid-19 we did not have enough supplies to prove Sayana. We had few gloves at this facility which were prioritized to other departments. So this made us advocate Sayana for self-injection only injecting those who wouldn’t manage after counseling. We were washing our hands and sanitizing.

Interviewer: any other challenge that you met

Respondent: the main problem is that it is out of stock

Title: MZs_HSA_6.docx

Descriptor Info:	District: Mzimba South	Target Population: TP1 - Providers/HSAs	Respondent type: HSA	Aware of SI: Yes - had heard of SI before	Coding phase: 1. Single coded	Client marital status: N/A - provider	Client age group: N/A - provider	Time since trained: A year or more ago

Codes Applied:	P3.2 - Barrier, general/other

Interviewer: do you need extra aids for counselling clients

Respondent: yes, like I said for my catchment area I have someone who uses sign language which I am not familiar with. We have difficulties in understanding one another

Title: KK_HSA_6.docx

Descriptor Info:	District: Nkhotakota	Target Population: TP1 - Providers/HSAs	Respondent type: HSA	Aware of SI: Yes - had heard of SI before	Coding phase: 1. Single coded	Client marital status: N/A - provider	Client age group: N/A - provider	Time since trained: A year or more ago

Codes Applied:	P3.2 - Barrier, general/other

INTERVIEWER: Have you faced any challenges in providing DMPA-SC for the last 3 months?

RESPONDENT: mmmhu. The challenges

INTERVIEWER: what are the challenges?

RESPONDENT: The challenges that these women are facing are that on the spot where they have self-injected, after some time, it looks like there is a hole which is different from Depo Provera. They inject on a flat skin but after some time, a hole develops. Because of this, women are worried. I took a step further and asked, they said that maybe it’s how they are self-injecting. Two, we were told that once a woman self-inject, she should not touch the place. But I think when they inject, they should be touching the spot to help the drugs move into the body. But during training they said that we should not be doing that. But I think it is necessary to do that because the end result of not doing it is what is happening, the spot develops a hole in 2 to 3 months. On her next appointment, she will show me and say I changed a spot and injected on this side, it’s the same outcome [HE LOOKED CONCERNED AND WORRIED]. Maybe if we are going to have a review meeting, I will ask about this. Maybe it’s because of the drugs or the touching. Apart from my community, my colleagues are also complaining about this.

INTERVIEWER: okay.

RESPONDENT: mmh.

Title: ZA_SCPI_8.docx

Descriptor Info:	District: Zomba	Target Population: TP2 - DMPA-IM/DMPA-SC PI clients	Respondent type: DMPA-SC PI client	Aware of SI: Yes - had heard of SI before	Coding phase: 1. Single coded	Client marital status:	Client age group:	Time since trained: N/A - client

Codes Applied:	P3.2 - Barrier, general/other

Respondent: so when we went back, they told us that they are busy with some work.

Interviewer: mmmm (showing that one is following the conversation)

Respondent: so we were not able to get the injectable.
