# Supplementary material for: Health care provider and client experiences of counselling on depot medroxyprogesterone acetate subcutaneous (DMPA-SC) for self-injection in Malawi
Source: PLOS Glob Public Health. 2023 Nov 30;3(11):e0002057. doi: 10.1371/journal.pgph.0002057 (PMC10688740; doi:10.1371/journal.pgph.0002057)
Supplement: S1 Data — (ZIP) [file pgph.0002057.s001.zip › S2/P3.2.2 stockouts + prov couns.doc]

Title: ZA_HSA_2.docx

Descriptor Info:	District: Zomba	Target Population: TP1 - Providers/HSAs	Respondent type: HSA	Aware of SI: Yes - had heard of SI before	Coding phase: 3. Triple coded	Client marital status: N/A - provider	Client age group: N/A - provider	Time since trained: Less than a year ago

Codes Applied:	P3.2.2 - Stockouts/stocks near expiry dates	P88 - Provider great quotes

Respondent: Yes, but the difficult things that are there is that currently there is a stock-out of Sayana.

Interviewer: mmmm (Showing that one is following the conversation)

Respondent: When its available, yeah, when its available maybe at the DHO only a few, so mostly we are just administering DMPA-IM which is bringing back the congestion that used to be there in the communities.

Title: ZA_HSA_2.docx

Descriptor Info:	District: Zomba	Target Population: TP1 - Providers/HSAs	Respondent type: HSA	Aware of SI: Yes - had heard of SI before	Coding phase: 3. Triple coded	Client marital status: N/A - provider	Client age group: N/A - provider	Time since trained: Less than a year ago

Codes Applied:	P3.2.2 - Stockouts/stocks near expiry dates

Respondent: ah Another thing is that because of the Sayana stock-outs

Interviewer: eh eh

Respondent: Has made people to come to ask about the Sayana, they say “you just started your program and now you are saying that there are no supplies for the commodity, we are used to self-inject at home.”

Interviewer: Yes.

Respondent: We are being busy just like we used to be in the past before Sayana came because of the stock-out.

Title: ZA_HSA_2.docx

Descriptor Info:	District: Zomba	Target Population: TP1 - Providers/HSAs	Respondent type: HSA	Aware of SI: Yes - had heard of SI before	Coding phase: 3. Triple coded	Client marital status: N/A - provider	Client age group: N/A - provider	Time since trained: Less than a year ago

Codes Applied:	P3.2.2 - Stockouts/stocks near expiry dates

Respondent: The method had a good start since it was introduced, then it experienced some slow progress because of the stock-outs that were experienced.

Title: ZA_HSA_2.docx

Descriptor Info:	District: Zomba	Target Population: TP1 - Providers/HSAs	Respondent type: HSA	Aware of SI: Yes - had heard of SI before	Coding phase: 3. Triple coded	Client marital status: N/A - provider	Client age group: N/A - provider	Time since trained: Less than a year ago

Codes Applied:	P3.2.2 - Stockouts/stocks near expiry dates	P3.1 - Enablers, general/other	P2.1.1 - Refresher training/mentorship

Interviewer: Alright, are there any good things about the support that you said you received?

Respondent: Yes

Interviewer: Can you share these with me?

Respondent: There were good things, we knew why we were experiencing the Sayana stock-outs

Interviewer: mmm (showing that one is following the conversation)

Respondent: But also, when its available…

Interviewer: mmm (Showing that one is following the conversation)

Respondent: How can we administer it…

Interviewer: Alright.

Respondent: Yes, the DHO emphasized that he will take the responsibility of making sure that Sayana is available…

Interviewer: mm (Showing that one is following the conversation)

Respondent: Yes, so it seems the questions that we had were addressed by the one that represented the DHO in that meeting, they gave us an assurance that they will try to do so…

Interviewer: mmm (Showing that one is following the conversation)

Respondent: Even though it will be available in small quantities, but when they start giving us enough funding for buying Sayana.

Interviewer: mmm (showing that one is following the conversation)

Respondent: Yes, because they communicated to us that the central medical stores were not given a program that they should be bringing Sayana…

Title: ZA_HSA_2.docx

Descriptor Info:	District: Zomba	Target Population: TP1 - Providers/HSAs	Respondent type: HSA	Aware of SI: Yes - had heard of SI before	Coding phase: 3. Triple coded	Client marital status: N/A - provider	Client age group: N/A - provider	Time since trained: Less than a year ago

Codes Applied:	P3.2.2 - Stockouts/stocks near expiry dates	C7 - Resupply

Respondent: some of them when they saw that we were experiencing stock-outs, they started worrying, so we tell them that when its available again we will continue, your chores and activities at home should not be affected because you have to come to the hospital to access family planning.

Title: ZA_HSA_2.docx

Descriptor Info:	District: Zomba	Target Population: TP1 - Providers/HSAs	Respondent type: HSA	Aware of SI: Yes - had heard of SI before	Coding phase: 3. Triple coded	Client marital status: N/A - provider	Client age group: N/A - provider	Time since trained: Less than a year ago

Codes Applied:	P3.2.2 - Stockouts/stocks near expiry dates

Interviewer: But I also wanted to know, in the past three months, how has the uptake of DMPA-SC for self-injection been like at this facility but also in the community where you work?

Respondent: Recently it didn’t happen because the commodity has recently not been available.

Interviewer: Alright.

Respondent: Now it’s been almost five to six months since we started experiencing the shortages.

Title: ZA_HSA_2.docx

Descriptor Info:	District: Zomba	Target Population: TP1 - Providers/HSAs	Respondent type: HSA	Aware of SI: Yes - had heard of SI before	Coding phase: 3. Triple coded	Client marital status: N/A - provider	Client age group: N/A - provider	Time since trained: Less than a year ago

Codes Applied:	P3.2.2 - Stockouts/stocks near expiry dates	P3.3 - Counselling/ training approach, general

Interviewer: Have you faced any difficulties in providing DMPA-SC?

Respondent: No, we have not faced any difficulties.

Interviewer: Alright.

Respondent: Mostly it’s because the commodity has not been available

Interviewer: mmm (Showing agreement)

Respondent: It hasn’t been available, the clients that were coming were being given DMPA_IM.

Interviewer: Alright.

Respondent: Yes, no difficulties have been faced.

Title: ZA_HSA_2.docx

Descriptor Info:	District: Zomba	Target Population: TP1 - Providers/HSAs	Respondent type: HSA	Aware of SI: Yes - had heard of SI before	Coding phase: 3. Triple coded	Client marital status: N/A - provider	Client age group: N/A - provider	Time since trained: Less than a year ago

Codes Applied:	P3.2.2 - Stockouts/stocks near expiry dates

Interviewer: Ah since the commodity was introduced here in Malawi, how has the trend of uptake been, has it changed overtime?

Respondent: The change is due to the shortages that are there…

Interviewer: mmm (showing agreement)

Respondent: Yes, but if it was available, people have accepted this commodity and they are used to it…

Interviewer: mmmm (Showing agreement)

Respondent: Yes

Interviewer: Alright.

Respondent: so, the commodity is the same as DMPA-IM, the only difference is that DMPA-SC is not available now…

Title: ZA_HSA_2.docx

Descriptor Info:	District: Zomba	Target Population: TP1 - Providers/HSAs	Respondent type: HSA	Aware of SI: Yes - had heard of SI before	Coding phase: 3. Triple coded	Client marital status: N/A - provider	Client age group: N/A - provider	Time since trained: Less than a year ago

Codes Applied:	P3.2.2 - Stockouts/stocks near expiry dates

Interviewer: Now we have come to the end of the questions that I had for you today, but before we part, ah do you have any other thoughts or maybe anything that you may want to share with me on what we have discussed?

Respondent: ah the main thing is the availability of Sayana.

Interviewer: mmmm (showing that one is following the conversation)

Respondent: Yeah, if this commodity is available the way that Depo is,

Interviewer: mmm (showing that one is following the conversation)

Respondent: Yea because for us, when we are ordering, we order here…

Interviewer: mmmm (showing that one is following the conversation)

Respondent: We can be ordering all the two commodities.

Title: ZA_HSA_2.docx

Descriptor Info:	District: Zomba	Target Population: TP1 - Providers/HSAs	Respondent type: HSA	Aware of SI: Yes - had heard of SI before	Coding phase: 3. Triple coded	Client marital status: N/A - provider	Client age group: N/A - provider	Time since trained: Less than a year ago

Codes Applied:	C1.1.1 - Positive client attitudes to SI	C5.1.4 - Timely injection due to not having to rely on external factors	C5.2.1 - Discontinuation of SI (including reasons)	P3.2.2 - Stockouts/stocks near expiry dates	C2.1.2 - Reduced trips to facility	C7 - Resupply

Respondent: According to the preferences of the clients in the communities, some prefer Sayana…

Interviewer: mmmhhh (showing that one is following the conversation)

Respondent: … and some prefer Depo

Interviewer: mmmmmhhh (showing that one is following the conversation)

Respondent: Yes, so when all the commodities are available, especially Sayana is available without hustles…

Interviewer: mmmhhh (showing that one is following the conversation)

Respondent: Yeah, it means it will give assurance to the clients because they will be coming to access the commodities…

Interviewer: mmmm (showing that one is following the conversation)

Respondent: Because when the commodity has run out, and we tell them that we will give them Depo, that Sayana is not available…

Interviewer: mmm (showing that one is following the conversation)

Respondent: yeah, so some of the clients are discouraged, they say it was good with Sayana, should we be coming often to the facility, we are working women…

Interviewer: mmm (Showing that one is following the conversation)

Respondent: If we have the dosage, we just inject ourselves after we come back from work, or before we go to work in the morning, we just inject ourselves when we are due for the injection.

Interviewer: mmmm (showing that one is following the conversation)

Respondent: Yeah, so you should try and find for us, then we say I will try to find if it is available at the DHO, I don’t know how the DHO does it, but the one available there is not enough…

Title: KK_HSA_1.docx

Descriptor Info:	District: Nkhotakota	Target Population: TP1 - Providers/HSAs	Respondent type: HSA	Aware of SI: Yes - had heard of SI before	Coding phase: 2. Double coded	Client marital status: N/A - provider	Client age group: N/A - provider	Time since trained: A year or more ago

Codes Applied:	P3.2.2 - Stockouts/stocks near expiry dates	P3.3.2 - Inclusion of practice opportunities for clients in training

Interviewer: Mmmhu. So in your community. What has been the uptake of Sayana in the past three months…so…mmmm we are in October right? Mmmh. So we should say September, august, what has…what has been the uptake of Sayana for the past 3 months?

Respondent: Aaah, for the past 3 months, the uptake was a little bit good but we now have a…what can we say? Stock out? For Sayana

Interviewer: okay

Respondent: So we are using DMPA. So DMPA, a health worker is supposed to inject a client. You see? But if it is Sayana, you just train them, and then counsel them, then they self inject. The first dose, they inject in your presence, then the remaining doses you give them to self inject. But for this no matter the circumstance, we inject them for three months. It can not be possible for them to take home DMPA.so because of this shortfall that is why…maybe the uptake has decreased.[not clear] October…October we did not finish. So on the past three months, it is the last two months that we have used DMPA. The first month in these three months is the one we used Sayana.

Interviewer: Which means in July it was available?

Respondent: mmmh

Title: KK_HSA_1.docx

Descriptor Info:	District: Nkhotakota	Target Population: TP1 - Providers/HSAs	Respondent type: HSA	Aware of SI: Yes - had heard of SI before	Coding phase: 2. Double coded	Client marital status: N/A - provider	Client age group: N/A - provider	Time since trained: A year or more ago

Codes Applied:	P3.2.2 - Stockouts/stocks near expiry dates

Interviewer: And what are the reasons that can discourage women to take up this self injection option? What can…discourage them?

Respondent: What can discourage them is frequent stockouts of the drug. Like what has…happened now. Mmhu. Women are now used that we use Sayana. And when an HSA is coming here in the field even if they are coming for other issues, they still have Sayana who is readily available. But now there is stockout of Sayana. We only have DMPA. And women are like no, that injection pains a lot. Aah as for me this one no, but Sayana. And the woman will not be on any method and she get pregnant

Interviewer: mmmhu.

Respondent: what if Sayana is readily available and there are no stockouts….

Title: ZA_HSA_5.docx

Descriptor Info:	District: Zomba	Target Population: TP1 - Providers/HSAs	Respondent type: HSA	Aware of SI: Yes - had heard of SI before	Coding phase: 2. Double coded	Client marital status: N/A - provider	Client age group: N/A - provider	Time since trained: Less than a year ago

Codes Applied:	C0.2.1 - Positive community/client perspectives on FP	P3.2.2 - Stockouts/stocks near expiry dates

Interviewer: Among the methods that you have mentioned are there any that are more popular than others? Among these methods?

Respondent: The ones that people like more?

Interviewer: Yes yes

Respondent: Sayana is more popular

Interviewer: Mmm (showing that one is following the conversation)

Respondent: Because when a in the community there was this other group of women who said that we don’t want Depo, but we want Sayana

Interviewer: Mmm (showing that one is following the conversation)

Respondent: When I asked them why, they said Sayana is a better option

Interviewer: Mmm (showing that one is following the conversation)

Respondent: Because I do not bleed a lot.

Interviewer: Mmm (showing that one is following the conversation)

Respondent: It’s just the way their bodies are yeah

Interviewer: Mmm (showing that one is following the conversation)

Respondent: So right now, most of them

Interviewer: Mmm (showing that one is following the conversation)

Respondent: I can see that they are accepting especially the area where I am responsible for

Interviewer: Mmm (showing that one is following the conversation)

Respondent: They are accepting it, like yesterday I went there, and I have given 5 women Sayana that they should self-inject

Interviewer: Mmm (showing that one is following the conversation)

Respondent: But some are still asking me because I did not have the Sayana yeah

Interviewer: Mmm (showing that one is following the conversation)

Respondent: Actually, today I have collected more

Interviewer: Okay

Title: ZA_HSA_5.docx

Descriptor Info:	District: Zomba	Target Population: TP1 - Providers/HSAs	Respondent type: HSA	Aware of SI: Yes - had heard of SI before	Coding phase: 2. Double coded	Client marital status: N/A - provider	Client age group: N/A - provider	Time since trained: Less than a year ago

Codes Applied:	P3.2.2 - Stockouts/stocks near expiry dates

Interviewer: Alright, how has the uptake trend been like for Sayana Press at this facility but also in the community for the past three months?

Respondent: Recently we had stock-outs, and Sayana expired yeah

Interviewer: Mmm (showing that one is following the conversation)

Respondent: So that’s why am saying that people were complaining, Sayana expired so we were distributing out Depo

Interviewer: Mmm (showing that one is following the conversation)

Respondent: That’s the one we were administering to the clients

Interviewer: Mmm (showing that one is following the conversation)

Respondent: So, the clients were inquiring, so we had those issues, this is now when Am going to give them

Interviewer: Mmm (showing that one is following the conversation)

Respondent: Because Sayana had expired.

Interviewer: Okay

Respondent: Yes

Title: ZA_HSA_5.docx

Descriptor Info:	District: Zomba	Target Population: TP1 - Providers/HSAs	Respondent type: HSA	Aware of SI: Yes - had heard of SI before	Coding phase: 2. Double coded	Client marital status: N/A - provider	Client age group: N/A - provider	Time since trained: Less than a year ago

Codes Applied:	P3.2.2 - Stockouts/stocks near expiry dates

Respondent: Okay like yesterday I have given five women.

Interviewer: Mmm (showing that one is following the conversation)

Respondent: I turned back the others because the Sayana that I had yeah …

Interviewer: Mmm (showing that one is following the conversation)

Respondent: … was a little so we just had to inject them ourselves

Interviewer: Mmm (showing that one is following the conversation)

Respondent: ma date so if we give these ones or to inject them it means it won’t work, its better if we inject them so that maybe we will go back and give them more once we get the stock

Title: ZA_HSA_5.docx

Descriptor Info:	District: Zomba	Target Population: TP1 - Providers/HSAs	Respondent type: HSA	Aware of SI: Yes - had heard of SI before	Coding phase: 2. Double coded	Client marital status: N/A - provider	Client age group: N/A - provider	Time since trained: Less than a year ago

Codes Applied:	P3.2.2 - Stockouts/stocks near expiry dates	P3.5.1 - Trends/numbers of SI

Interviewer: Alright, what can you say about the trend of uptake of DMPA-SC after some time? Has the trend changed?

Respondent: The uptake?

Interviewer: Are more women …

Respondent: The women accessing it?

Interviewer: Mmm (showing that one is following the conversation)

Respondent: More women are accessing it, currently, As I have said there was a gap recently

Interviewer: Mmm (showing that one is following the conversation)

Respondent: Because there was no supply of Sayana, currently I have noticed that more women are taking it for sure.

Interviewer: Mmm (showing that one is following the conversation)

Respondent: Because that time when I started training them, I gave more women, but I did not continue with them because of the stock out

Title: ZA_HSA_5.docx

Descriptor Info:	District: Zomba	Target Population: TP1 - Providers/HSAs	Respondent type: HSA	Aware of SI: Yes - had heard of SI before	Coding phase: 2. Double coded	Client marital status: N/A - provider	Client age group: N/A - provider	Time since trained: Less than a year ago

Codes Applied:	P3.2.2 - Stockouts/stocks near expiry dates

Interviewer: Alright, for clients like these ones, did you give then additional counselling after you injected them?

Respondent: We gave her, she was solid that she wanted to be self-injecting, but since we had a stock out of Sayana

Interviewer: Eh

Respondent: We were unable to continue with her on self-injection

Interviewer: Alright

Respondent: But since she wanted to self-inject, I would have loved...

Interviewer: Mmm (showing that one is following the conversation)

Respondent: If she got trained until she was able to self-inject, but since the stock is here when the client comes back, she will be assisted to self-inject

Interviewer: Alright.

Respondent: Eh

Title: KK_HSA_5.docx

Descriptor Info:	District: Nkhotakota	Target Population: TP1 - Providers/HSAs	Respondent type: HSA	Aware of SI: Yes - had heard of SI before	Coding phase: 1. Single coded	Client marital status: N/A - provider	Client age group: N/A - provider	Time since trained: A year or more ago

Codes Applied:	P3.2.2 - Stockouts/stocks near expiry dates

INTERVIEWER: What has the uptake of DMPA-SC been like in this community in the past three months?

RESPONDENT: The uptake has been great, though we have a slight problem in that the units that we had expired.

INTERVIEWER: Alright

RESPONDENT: We realized a bit late that the cartons of supplied that we counted on had only a life span of 1 month, they would expire in a month. Hence, we had to communicate so fast

INTERVIEWER: Ok

RESPONDENT: With the district hospital to bring us another lot and collect the one that was about to expire. Unfortunately, when bringing the ither lot they had also included the one that we had returned, so there was confusion.

INTERVIEWER: Ok

RESPONDENT: But we manage to resolve that.

INTERVIEWER: Have you faced any challenge providing DMPA-SC in the last three months?

RESPONDENT: The main challenge in the past three months was about shortage of supplies due to the stocks that were about to expire. So after reporting to the coordinator at the district were advised that our stock that was about to expire will be collected and be used at the district, whilst we get some new stocks from the closest hospital that had some stock with a long shelf-span.

Title: KK_HSA_5.docx

Descriptor Info:	District: Nkhotakota	Target Population: TP1 - Providers/HSAs	Respondent type: HSA	Aware of SI: Yes - had heard of SI before	Coding phase: 1. Single coded	Client marital status: N/A - provider	Client age group: N/A - provider	Time since trained: A year or more ago

Codes Applied:	P1.1.1 - Positive provider attitudes towards SI	C1.1.1 - Positive client attitudes to SI	P3.2.2 - Stockouts/stocks near expiry dates	P2.2 - Barriers, general/other

INTERVIEWER: Do you have any other thoughts or information if at all you may have forgotten something during our discussions.

RESPONDENT: Much have already been said

INTERVIEWER: Alright

RESPONDENT: In my community most women they like to use this method over the other methods.

INTERVIEWER: Ok

RESPONDENT: They are scrambling for it compared in the, we just request that those who are supplying this method should continue with regular and enough supplies.

INTERVIEWER: Alright

RESPONDENT: We have some areas where new HSA’s have just been deployed, that means the tie DMPA-SC was being introduced these areas were missed as a result the method has not been fully introduced yet a lot of clients are interested to take this method.

INTERVIEWER: Alright

RESPONDENT: That’s it, sure sure

Title: MZs_HSA_1.docx

Descriptor Info:	District: Mzimba South	Target Population: TP1 - Providers/HSAs	Respondent type: HSA	Aware of SI: Yes - had heard of SI before	Coding phase: 2. Double coded	Client marital status: N/A - provider	Client age group: N/A - provider	Time since trained:

Codes Applied:	P3.2.2 - Stockouts/stocks near expiry dates

Respondent: Because it has been long since we were trained and we have forgotten and also since Sayana is out of stock for some time now, it’s possible for us to forget some parts of the training on provision of Sayana press.

Title: MZs_HSA_1.docx

Descriptor Info:	District: Mzimba South	Target Population: TP1 - Providers/HSAs	Respondent type: HSA	Aware of SI: Yes - had heard of SI before	Coding phase: 2. Double coded	Client marital status: N/A - provider	Client age group: N/A - provider	Time since trained:

Codes Applied:	P3.2.2 - Stockouts/stocks near expiry dates	P3.5.1 - Trends/numbers of SI

Q6. Interviewer: What has the uptake of DMPA-SC been like in this community?

Respondent: For the past 3 months clients are not coming Sayana press cause it’s out of stock.

Interviewer: Mmmmmm. (following)

Respondent: Yes, we don’t have Sayana.

Interviewer: Have you faced any challenge in providing DMPA-SC in the last 3 months like Covid 19?

Respondent: No.

Interviewer: What proportion of DMPA-SC provided is for self-injection? You have said that for the past 3 months Sayana is out of stock.

Respondent: Yes.

Interviewer: So I want you to compare the time you had Sayana, how many women came for self-injection?

Respondent: There is a different, the time Sayana was in stock we used to have a lot of women opt for self-injection.

Interviewer: Would you say the trend in DMPA-SC uptake for self-injection has changed over the time?

Respondent: Yes.

Interviewer: What factors do you think has influence this trend?

Respondent: Lack of Sayana press at the facility.

Title: MZs_HSA_1.docx

Descriptor Info:	District: Mzimba South	Target Population: TP1 - Providers/HSAs	Respondent type: HSA	Aware of SI: Yes - had heard of SI before	Coding phase: 2. Double coded	Client marital status: N/A - provider	Client age group: N/A - provider	Time since trained:

Codes Applied:	P3.2.2 - Stockouts/stocks near expiry dates

Respondent: The other reason might be lack of Sayana press since it’s out of stock. Women may not have confident in self-injection because once they have started using it and if they come for refill, we told them that Sayana is out of stock, as a result they will not be confident to use it.

Title: KK_FAC_1.docx

Descriptor Info:	District: Nkhotakota	Target Population: TP1 - Providers/HSAs	Respondent type: Facility-based provider	Aware of SI: Yes - had heard of SI before	Coding phase: 3. Triple coded	Client marital status: N/A - provider	Client age group: N/A - provider	Time since trained: A year or more ago

Codes Applied:	P3.2.2 - Stockouts/stocks near expiry dates

RESPONDENT: But with passing of time, we see clients coming to request for the method.

INTERVIEWER: okay

RESPONDENT: May I have Sayana. But recently it was out of stock since the one available were expiring in August if am wrong.

INTERVIEWER: Okay

RESPONDENT: So we were not giving out to take home as we wanted to use the stock available. Hence, we were advising clients to have other doses to take home in the next visit, when the new stock is available.

Title: KK_FAC_1.docx

Descriptor Info:	District: Nkhotakota	Target Population: TP1 - Providers/HSAs	Respondent type: Facility-based provider	Aware of SI: Yes - had heard of SI before	Coding phase: 3. Triple coded	Client marital status: N/A - provider	Client age group: N/A - provider	Time since trained: A year or more ago

Codes Applied:	P3.2.2 - Stockouts/stocks near expiry dates

INTERVIEWER: What has the uptake DMPA-SC been like at the facility in the past three months.

RESPONDENT: For the past three months, the issue is the one I earlier mentioned that we had a lot of Sayana that was about to expire it should be August, so we could not give it to our clients to take home because we knew that by the time they want to take the next dose it may have expired.

INTERVIEWER: It has expired.

RESPONDENT: So the past three months has not been promising even our reports show very few clients, or I may just say we do not have any clients indicating a take some dose home due to expiration issue.

Title: KK_FAC_1.docx

Descriptor Info:	District: Nkhotakota	Target Population: TP1 - Providers/HSAs	Respondent type: Facility-based provider	Aware of SI: Yes - had heard of SI before	Coding phase: 3. Triple coded	Client marital status: N/A - provider	Client age group: N/A - provider	Time since trained: A year or more ago

Codes Applied:	C2.2.1 - Stockouts/stocks near expiry dates	P3.2.2 - Stockouts/stocks near expiry dates

INTERVIEWER: Alright fine. Our last question, in your view what are the main factors that would discourage clients to take the self-injection option with DMPA-SC? Factors that can discourage them.

RESPONDENT: The issue is the one that I said at some point on stockouts, this affects so much

INTERVIEWER: Okay

RESPONDENT: Yes, like the time we hard short life Sayana , we had clients asking to take it but since the expiry date was so close

INTERVIEWER: Yes

RESPONDENT: So we could not give out the method since we knew that the medication would expire at home

INTERVIEWER: Yes

RESPONDENT: So when procuring the medicines we should consider expiry dates.

INTERVIEWER: Ok

RESPONDENT: Sure

INTERVIEWER: Fine

RESPONDENT: Because if a client comes to request for the method and we tell them we have expired stuff they get disappointed, then next time they will not have the courage to ask for it again.

INTERVIEWER: So it means the client would go back to depo?

RESPONDENT: Yes, to the previous one depo

Title: MZs_HSA_5.docx

Descriptor Info:	District: Mzimba South	Target Population: TP1 - Providers/HSAs	Respondent type: HSA	Aware of SI: Yes - had heard of SI before	Coding phase: 1. Single coded	Client marital status: N/A - provider	Client age group: N/A - provider	Time since trained: A year or more ago

Codes Applied:	P3.2.2 - Stockouts/stocks near expiry dates

Interviewer: for the past 3 months, have you met any challenges on Sayana?

Respondent: as of the past we do not have Sayana

Interviewer: when did you last run out of it?

Respondent: I think in February. From march it was out of stock

Title: MZs_HSA_5.docx

Descriptor Info:	District: Mzimba South	Target Population: TP1 - Providers/HSAs	Respondent type: HSA	Aware of SI: Yes - had heard of SI before	Coding phase: 1. Single coded	Client marital status: N/A - provider	Client age group: N/A - provider	Time since trained: A year or more ago

Codes Applied:	P1.2.1 - Negative provider attitudes towards SI	P3.1.1 - Reduced provider workload	P3.2.2 - Stockouts/stocks near expiry dates

Interviewer: thanks you so much we have come to the end of our interview. If you have anything to say, go ahead

Respondent: when Sayana was introduced we had fears that women may not manage to self-inject. We also thought they would disrespect us if they inject themselves. After using it we have seen that there is reduced workload on our side. However, Sayana is out of stock women are complaining because they have gone back to Depo IM. Some they go back without getting an alternative which is risking unwanted pregnancies.

Interviewer: thank you so much for your time. Like I said this shall be transcribed and we shall maintain confidentiality. Do you have any question?

Respondent: we got a training on Sayana but now it is out of stock.

Title: KK_HSA_3.docx

Descriptor Info:	District: Nkhotakota	Target Population: TP1 - Providers/HSAs	Respondent type: HSA	Aware of SI: Yes - had heard of SI before	Coding phase: 3. Triple coded	Client marital status: N/A - provider	Client age group: N/A - provider	Time since trained: A year or more ago

Codes Applied:	P3.2.2 - Stockouts/stocks near expiry dates

INTERVIEWER: what do you love could have been improved in terms of the training apart from that the days were not enough?

RESPONDENT: What I would like to be improved is that when training us and when it came to giving us supplies, sometimes they are in small amounts mmmh. they are not enough.

INTERVIEWER: mmmh

RESPONDENT: What I have seen is that, when we counselled the people, they loved the method, but the supplies are now not enough

Title: KK_HSA_3.docx

Descriptor Info:	District: Nkhotakota	Target Population: TP1 - Providers/HSAs	Respondent type: HSA	Aware of SI: Yes - had heard of SI before	Coding phase: 3. Triple coded	Client marital status: N/A - provider	Client age group: N/A - provider	Time since trained: A year or more ago

Codes Applied:	P3.2.2 - Stockouts/stocks near expiry dates	C7 - Resupply

INTERVIEWER: What has the uptake of DMPA-SC been like in your community in the past three months?

RESPONDENT: In the past three months the uptake of Sayana was not good because we had Sayana that aaaa [thinking] expired in July. Women on this method were not happy because they like the method. Another one expired in July and another batch in October. In short it was difficult.

INTERVIEWER: Which means you now have Sayana in stock?

RESPONDENT: We have a stockout of Sayana.

INTERVIEWER: since July…July?

REPONDENT: Mmmmh August, September, October. The Sayana in store and at the pharmacy is expired. We are in stockout. Women are receiving Depo. A lot of women on Sayana are worried. mmmh

INTERVIEWER: Okay. When Sayana is in stock, how much do you have?

RESPONDENT: When Sayana is in stock, it depends with the population of women you have in your community. So you have Sayana according to the number of your clients.

Title: ZA_HSA_4.docx

Descriptor Info:	District: Zomba	Target Population: TP1 - Providers/HSAs	Respondent type: HSA	Aware of SI: Yes - had heard of SI before	Coding phase: 1. Single coded	Client marital status: N/A - provider	Client age group: N/A - provider	Time since trained: A year or more ago

Codes Applied:	P1.1.1 - Positive provider attitudes towards SI	P3.2.2 - Stockouts/stocks near expiry dates

Interviewer: Mmh! Alright alright were you or are you still having concerns about the self-injection.

Respondent: Aaah I don’t have any concern it’s just that when we don’t have the injection it’s when they complain

Interviewer: Alright

Respondent: Mmmh

Interviewer: Is there anything that have made you to change your perception about the self-injection method?

Respondent: Aaah! There’s nothing that has changed my perception

Interviewer: Alright alright! What made you not to change your perception?

Respondent: When these women are receiving the injection, they appreciate it saying its good, some saying it is challenging. The thing that made me not to change my perception it’s because even Depo women were saying the same some saying it is good some saying its bad, so I just saw that it’s the same.

Interviewer: Alright thank you! What has the uptake of self-injection been like in this community the past three months?

Respondent: Aaah in the last 2 months it expired so if there is time, we had it is this month

Interviewer: Mmmh

Respondent: Yes

Interviewer: Alright alright! So, for how long have you not administered the injection in the community?

Respondent: Like for me it has been 2 months

Interviewer: 2 months?

Respondent: Yes

Title: ZA_HSA_4.docx

Descriptor Info:	District: Zomba	Target Population: TP1 - Providers/HSAs	Respondent type: HSA	Aware of SI: Yes - had heard of SI before	Coding phase: 1. Single coded	Client marital status: N/A - provider	Client age group: N/A - provider	Time since trained: A year or more ago

Codes Applied:	P3.2.2 - Stockouts/stocks near expiry dates	P3.5.1 - Trends/numbers of SI

Interviewer: Alright on ratio of 100 women who receive Sayana how many inject themselves

Respondent: Mmmh like in my community there are only 3 women which I know well are getting the self-injection while the others prefer to be injected.

Interviewer: Mmmm

Interviewer: That means it’s just the 3 of them?

Respondent: In my community

Interviewer: How many are they?

Respondent: That get injected with Sayana

Interviewer: Mmmh

Respondent: I cannot really say how many they are because the time they started administering Sayana, Depo was stopped so that means all those who were receiving Depo started receiving Sayana by compulsory

Interviewer: Mmmh

Respondent: I cannot really say that such number are people who gets Sayana injectable because the Depo injectable have just been administered last month. All this time it was Sayana.

Interviewer: It’s just the 3 who get themselves injected

Respondent: Yes yes

Title: ZA_HSA_4.docx

Descriptor Info:	District: Zomba	Target Population: TP1 - Providers/HSAs	Respondent type: HSA	Aware of SI: Yes - had heard of SI before	Coding phase: 1. Single coded	Client marital status: N/A - provider	Client age group: N/A - provider	Time since trained: A year or more ago

Codes Applied:	P3.2.2 - Stockouts/stocks near expiry dates

Interviewer: Alright thank you so which can you say that the uptake of the self-injected contraceptive have changed lately?

Respondent: Aah!! It changed because it was scarce, it was not found in communities since we take from the hospital

Interviewer: Eeeh you take from the hospital?

Respondent: When we go to the community

Interviewer: When you go to the community, has there been a change in women who self-inject?

Respondent: No it has not changed; they were also troubled since in there was none here as they expired.

Interviewer: What do you do with the expired injectables?

Respondent: We leave the expired ones here at the hospital, we don’t take them

Interviewer: How do you help these women?

Respondent: You mean when theirs has finished?

Interviewer: Yes

Respondent: Oho we tell them they should go to Matawale here they just get Depo

Interviewer: Do we have hope that they will get injected?

Respondent: Now they will get it

Interviewer: The self-injecting ones or?

Respondent: The self-injecting ones, they told me they got if from got if from Matawale

Title: KK_HSA_4.docx

Descriptor Info:	District: Nkhotakota	Target Population: TP1 - Providers/HSAs	Respondent type: HSA	Aware of SI: Yes - had heard of SI before	Coding phase: 1. Single coded	Client marital status: N/A - provider	Client age group: N/A - provider	Time since trained: A year or more ago

Codes Applied:	P3.2.2 - Stockouts/stocks near expiry dates

INTERVIEWER: Alright, what could have been improved? You have responded to the second question already by explaining about the good side of the method.

RESPONDENT: Yes

INTERVIEWER: So what could have been improved? You have responded to the second question already by explaining about the good side of the method, from the training that you did?

RESPONDENT: yes, I feel that the most important thing that would need to improve is the availability of supplies. I recall that during our training we were told to give the client extra units to take home to be used after three months or so.

INTERVIEWER: Ok

RESPONDENT: However, in most cases we don’t have enough stock as a result we just allow the client to do self-injection once right here at a facility. And she is told to come at a later date to check if we have more stocks so she can collect some to take home.

Title: KK_HSA_4.docx

Descriptor Info:	District: Nkhotakota	Target Population: TP1 - Providers/HSAs	Respondent type: HSA	Aware of SI: Yes - had heard of SI before	Coding phase: 1. Single coded	Client marital status: N/A - provider	Client age group: N/A - provider	Time since trained: A year or more ago

Codes Applied:	P2.1.1 - Refresher training/mentorship	P3.2.2 - Stockouts/stocks near expiry dates

RESPONDENT: We were just recoding that the client had the first injection. So during the supervision it’s when we were told also to record the stocks given to the client to use home for such a particular period.

INTERVIEWER: Alright

RESPONDENT: On that one I would say it helped us so much, however, we still request provision of enough stocks, this one has to be addressed.

Title: MZs_HSA_4.docx

Descriptor Info:	District: Mzimba South	Target Population: TP1 - Providers/HSAs	Respondent type: HSA	Aware of SI: Yes - had heard of SI before	Coding phase: 1. Single coded	Client marital status: N/A - provider	Client age group: N/A - provider	Time since trained:

Codes Applied:	P2.2.1 - Lack of/insufficient refresher training/mentorship	P3.1.1 - Reduced provider workload	P3.2.2 - Stockouts/stocks near expiry dates

Interviewer: what are your thoughts on this training?

Respondent: my view is that quite some time has passed since we were trained as such, we may forget some elements. Therefore, there is need for fresher courses because our job concerns the health life of human beings. I also would like to urge the providers to make accessible the Sayana which is currently out of stock. This is so because when clients use Sayana it lessens our job.

Title: MZs_HSA_4.docx

Descriptor Info:	District: Mzimba South	Target Population: TP1 - Providers/HSAs	Respondent type: HSA	Aware of SI: Yes - had heard of SI before	Coding phase: 1. Single coded	Client marital status: N/A - provider	Client age group: N/A - provider	Time since trained:

Codes Applied:	P3.2.2 - Stockouts/stocks near expiry dates

Interviewer: what difficulties have you faced in providing this service for the past two months?

Respondent: the unavailability of Sayana since most clients like it. Such unavailability has caused difficulties

Interviewer: alright

Respondent: three months now has passed since we had Sayana.

Title: MZs_HSA_4.docx

Descriptor Info:	District: Mzimba South	Target Population: TP1 - Providers/HSAs	Respondent type: HSA	Aware of SI: Yes - had heard of SI before	Coding phase: 1. Single coded	Client marital status: N/A - provider	Client age group: N/A - provider	Time since trained:

Codes Applied:	P3.2.2 - Stockouts/stocks near expiry dates

Q19. Interviewer: Do you have any additional views on what we have discussed?

Respondent: no, mine is just a word of thanks that we conducted this interview but now three months have passed since we last had Sayana…

Title: ZA_FAC_1.docx

Descriptor Info:	District: Zomba	Target Population: TP1 - Providers/HSAs	Respondent type: Facility-based provider	Aware of SI: Yes - had heard of SI before	Coding phase: 2. Double coded	Client marital status: N/A - provider	Client age group: N/A - provider	Time since trained: Less than a year ago

Codes Applied:	P3.2.2 - Stockouts/stocks near expiry dates	C1.1.3 - Awareness of DMPA-SC SI

Respondent: the second most popular method would be Sayana but it hard its own challenges in the middle of this year.

Interviewer: yah yah

Respondent: due to those challenges the clients were coming to ask about the method

Interviewer: mmmm (showing that one is following the conversation)

Respondent: so I cannot place it on top two

Title: ZA_FAC_1.docx

Descriptor Info:	District: Zomba	Target Population: TP1 - Providers/HSAs	Respondent type: Facility-based provider	Aware of SI: Yes - had heard of SI before	Coding phase: 2. Double coded	Client marital status: N/A - provider	Client age group: N/A - provider	Time since trained: Less than a year ago

Codes Applied:	P3.2.2 - Stockouts/stocks near expiry dates

Respondent: another thing that I have observed is that yes indeed we were trained but it looks like on the ground it's not the reality for example the availability of Sayana

Interviewer: mmmm (showing that one is following the conversation)

Respondent: we could be trained we come back vibrant and we will start training the clients but you will find that the commodity is not available, So what are we doing. So it just seems like we are trained in theory but here we are expecting that we will be doing the practical things on the ground, but the things are not yet ready.

Interviewer: mmmm (showing that one is following the conversation)

Respondent: I would have loved that before the trainings the resources should be ready

Interviewer: mmmm (showing that one is following the conversation)

Respondent: not like people are studying the training but the resources are not available

Interviewer: mmmm (showing that one is following the conversation)

Respondent: it means that we have trained the clients in DMPA-SC, I don't know if it is now available but in the recent past it was not available

Interviewer: mmmm (showing that one is following the conversation)

Respondent: we still train, we just agreed that we can just jump they part for DMPA-SC Because the clients may ask that they want to see the commodity, so on the part of resources I would have loved that if there should be a training the resources should also be available

Interviewer: Alright

Respondent: yes yes

Title: ZA_FAC_1.docx

Descriptor Info:	District: Zomba	Target Population: TP1 - Providers/HSAs	Respondent type: Facility-based provider	Aware of SI: Yes - had heard of SI before	Coding phase: 2. Double coded	Client marital status: N/A - provider	Client age group: N/A - provider	Time since trained: Less than a year ago

Codes Applied:	P3.2.2 - Stockouts/stocks near expiry dates

Interviewer: Yah alright, how has the trend of uptake of DMPASC been like at this facility for the past three months?

Respondent: mmm so far for the past three months it has been bad

Interviewer: mmmm (showing that one is following the conversation)

Respondent: because we had a Sayana stock out.

Interviewer: mmmm (showing that one is following the conversation)

Respondent: eh but when you talk of the previous last quarter

Interviewer: mmmm (showing that one is following the conversation)

Respondent: when we look at the urban facilities

Interviewer: mmmm (showing that one is following the conversation)

Respondent: There is [NAME], there is our facility, am saying this because I attended a DMPASC refresher training

Interviewer: mmmm (showing that one is following the conversation)

Respondent: so the data showed that at least we are going towards something like 60, 70 percent

Interviewer: mmmm (showing that one is following the conversation)

Respondent: yeah, we are indeed providing Sayana at this facility.

Interviewer: alright,

Respondent: but it dropped because of stock out

Interviewer: ah okay

Respondent: yes

Title: ZA_FAC_2.docx

Descriptor Info:	District: Zomba	Target Population: TP1 - Providers/HSAs	Respondent type: Facility-based provider	Aware of SI: Yes - had heard of SI before	Coding phase: 3. Triple coded	Client marital status: N/A - provider	Client age group: N/A - provider	Time since trained: Less than a year ago

Codes Applied:	P3.1 - Enablers, general/other	P3.2.2 - Stockouts/stocks near expiry dates

Interviewer: To talk about supply, have you ever had any shortages in the supply of the self-injection medicine?

Respondent: No. We have had no shortages. The drugs are plenty.

Title: MZs_HSA_3.docx

Descriptor Info:	District: Mzimba South	Target Population: TP1 - Providers/HSAs	Respondent type: HSA	Aware of SI: Yes - had heard of SI before	Coding phase: 3. Triple coded	Client marital status: N/A - provider	Client age group: N/A - provider	Time since trained: Less than a year ago

Codes Applied:	P3.2.2 - Stockouts/stocks near expiry dates

Q6. Interviewer: What has uptake of DMPA-SC been like at this facility/ in this community in the past three months?

Respondent: because now its almost we should say its almost over three months we don’t have that Sayana yah… there is no Sayana coming we…returned back to Depo… Depo. Yah Sayana is coming we don’t know what is problem. We feel they targeted the DHO for piloting or we don’t know but……they have not they have not more more Sayana and the dates for expiry they were within that Sayana which came had the range of either September or October expiring so it expired.

Interviewer: What other challenges have you faced?

Respondent: it is… aah on a provision…saa. Sayana we don’t receive we don’t know why.

Interviewer: would you say the trend in DMPA-SC uptake for self-injection has changed over time?

Respondent: aaaah…people were were in need of it but it was not there it is not there they still need when we tell them that there is no Sayana most of them they are disappointed because they were now used to say they should just collect and give inject themselves in the villages.

Interviewer: would you say the trend in DMPA-SC uptake for self-injection has changed over time?

Respondent: yes… because they are forced to say they have come for that service but its not there but they need the Sayana. They say we need Sayana but we say we don’t have Sayana so they have to get Depo.

Title: KK_HSA_2.docx

Descriptor Info:	District: Nkhotakota	Target Population: TP1 - Providers/HSAs	Respondent type: HSA	Aware of SI: Yes - had heard of SI before	Coding phase: 2. Double coded	Client marital status: N/A - provider	Client age group: N/A - provider	Time since trained: A year or more ago

Codes Applied:	P3.2.2 - Stockouts/stocks near expiry dates

INTERVIEWER: Alright, the next question is what has the uptake of DMPA-SC been like at the facility in the past three month. This DMPA-SC how has been its uptake in your community in the past three month.

RESPONDENT: In the past three months DMPA-SC was being administered by since that time up to now it’s out of stock.

INTERVIEWER: Ohooo so you mean it’s out of stock?

RESPONDENT: yaah

INTERVIEWER: So we can say that the major problem is that DMPA-SC is out of stock?

RESPONDENT: Yes

INTERVIEWER: mhh alright, so if we compare self -injection and the one injected by health personnel like Depo, what’s the proportion like?

RESPONDENT: aa what normally happens is when DMPA-SA is available, Depo is out of stock and vice versa hence its difficult to compare the proportion.

INTERVIEWER: Mhhh ohh fine so in short we can say your unable to detect the proportion because of that?

RESPONDENT: yaaa

INTERVIEWER: Mhh so would you say that the trend in DMPA-SC uptake of injection has changed over time. If so what factors do you think have influenced this trend.

RESPONDENT: Yes there has been a change for instance if one takes Depo she would be visiting the facility every three months whilst DMPA _Sc its only once a year. Hence she would prefer Sayana.

INTERVIEWER: So mainly what would you say has changed is it that more women are taking DMPA-SC or not?

RESPONDENT: They have increased

INTERVIEWER: The number has increase for those taking Sayana?

RESPONDENT: Yes

Title: KK_HSA_2.docx

Descriptor Info:	District: Nkhotakota	Target Population: TP1 - Providers/HSAs	Respondent type: HSA	Aware of SI: Yes - had heard of SI before	Coding phase: 2. Double coded	Client marital status: N/A - provider	Client age group: N/A - provider	Time since trained: A year or more ago

Codes Applied:	P1.1.1 - Positive provider attitudes towards SI	C5.1.4 - Timely injection due to not having to rely on external factors	P3.2.2 - Stockouts/stocks near expiry dates

INTERVIEWER: Alright, now am on point where you have counselled a client on DMPA-SC what do you think would attract a client to use the Sayana method since there is also Depo. What is it that attracts women the most?

RESPONDENT: aaa sometimes we come across incidences where Depo is out of stock

INTERVIEWER: Yes

RESPONDENT: Whilst in case of Sayana one is given a stock for a year, compared to Depo which would require a client to come again after three months and then she find out that Depo is out of stock.

INTERVIEWER: Okay so that that’s what attracts women the most not so

RESPONDENT: Yes

Title: ZA_HSA_3.docx

Descriptor Info:	District: Zomba	Target Population: TP1 - Providers/HSAs	Respondent type: HSA	Aware of SI: Yes - had heard of SI before	Coding phase: 1. Single coded	Client marital status: N/A - provider	Client age group: N/A - provider	Time since trained: A year or more ago

Codes Applied:	P3.2.2 - Stockouts/stocks near expiry dates

Interviewer: Alright thank you very much, ever since you had training on self-injection family planning method have you had any other support in addition to the training?

Respondent: No, we haven’t had any other support concerning family planning or Sayana press.

Interviewer: Alright, ahh do you think you need any support?

Respondent: Yes, we would like to be assisted.

Interviewer: Where exactly do you see that there is a need for assistance?

Respondent: Uhhmmm because by the time we had our training there was Sayana press but nowadays we don’t have it anymore so it is like we went back to Depo IM which we were injecting before Sayana hence our workload has increased again so we would like Sayana to be available again.

Title: ZA_HSA_3.docx

Descriptor Info:	District: Zomba	Target Population: TP1 - Providers/HSAs	Respondent type: HSA	Aware of SI: Yes - had heard of SI before	Coding phase: 1. Single coded	Client marital status: N/A - provider	Client age group: N/A - provider	Time since trained: A year or more ago

Codes Applied:	P3.2.2 - Stockouts/stocks near expiry dates

Interviewer: Have you given out Sayana in the past three months?

Respondent: We have not been able to provide Sayana since we have not had it for a long time, it must be between January or February when it went out of stock.

Title: ZA_HSA_3.docx

Descriptor Info:	District: Zomba	Target Population: TP1 - Providers/HSAs	Respondent type: HSA	Aware of SI: Yes - had heard of SI before	Coding phase: 1. Single coded	Client marital status: N/A - provider	Client age group: N/A - provider	Time since trained: A year or more ago

Codes Applied:	P3.2.2 - Stockouts/stocks near expiry dates

Interviewer: Alright thank you very much, we have reached the end of this interview, are there any thoughts that you would like to share with me concerning everything we have discussed today?

Respondent: Ahhh... As I have already said that time method of using Sayana press is greatly appreciated by the community because it is a simple way but my request to you is that Sayana should start being found again since we have not had it here in a long time so, please consider us

Interviewer: Alright alright thank you very much

Title: ZA_HSA_3.docx

Descriptor Info:	District: Zomba	Target Population: TP1 - Providers/HSAs	Respondent type: HSA	Aware of SI: Yes - had heard of SI before	Coding phase: 1. Single coded	Client marital status: N/A - provider	Client age group: N/A - provider	Time since trained: A year or more ago

Codes Applied:	P3.2.2 - Stockouts/stocks near expiry dates

Respondent: Yes, my last question is if that will Sayana come back in the hospitals

Interviewer: Yes, the Sayana will return to the hospitals because even the government is aware of the existing gaps as such, they will fix this problem

Respondent: Thank you very much

Title: ZA_HSA_6.docx

Descriptor Info:	District: Zomba	Target Population: TP1 - Providers/HSAs	Respondent type: HSA	Aware of SI: Yes - had heard of SI before	Coding phase: 1. Single coded	Client marital status: N/A - provider	Client age group: N/A - provider	Time since trained:

Codes Applied:	P3.2.2 - Stockouts/stocks near expiry dates

Interviewer: Alright, did you and your friends discuss this?

Respondent: ah we have been discussing.

Interviewer: mmmm (showing that one is following the conversation)

Respondent: but sometimes maybe when we went on the ground, after discussing and we go on the ground to do the work, it would happen that the supplies that we have discussed

Interviewer: mmmm (showing that one is following the conversation)

Respondent: maybe the office doesn’t have

Interviewer: mmmm (showing that one is following the conversation)

Respondent: it showed that the office like the DHO’s, on its own it fails to have the resources which are required for our work on the ground. So, it would happen that on the ground it changes due to the resources that we have.

Interviewer: yah yah yah

Respondent: so that may disturb our programs. Let’s just assume that we have discussed that lets mobilize the community about Sayana.

Interviewer: mmmm (showing that one is following the conversation)

Respondent: but you will find that here we only have maybe, at the health center we don’t have, at the DHO they also don’t have

Interviewer: yah yah yah

Respondent: so, it all seems like a mess

Title: ZA_HSA_6.docx

Descriptor Info:	District: Zomba	Target Population: TP1 - Providers/HSAs	Respondent type: HSA	Aware of SI: Yes - had heard of SI before	Coding phase: 1. Single coded	Client marital status: N/A - provider	Client age group: N/A - provider	Time since trained:

Codes Applied:	P3.2.2 - Stockouts/stocks near expiry dates	P2.2 - Barriers, general/other	C2.1.1 - Community availability of injectables/SI

Interviewer: Alright, what factors do you think prevent women from using DMPA-SC?

Respondent: Stock outs of the commodity, it seems that it’s a major factor

Interviewer: mmmm (showing that one is following the conversation)

Respondent: ah and maybe the cancellation of clinics in the communities can be another factor

Interviewer: mmmm (showing that one is following the conversation)

Respondent: another factor is the HSAs that have been trained, if these are not available at the community clinic in a particular week

Interviewer: eh

Respondent: If only those that have gone to the community clinic are the ones that were not trained, this can affect the uptake of DMPA-SC, if all the HSAs are trained it means the clinics will still be done even if some HSAs are busy. It could be that 5 HSAs were supposed to go to the community clinic but two out of the five have been invited to a TB program, it means only three will go to the community clinic and if these three have not been trained in DMPA-SC and only the two that have been called to a different program were trained, then it could be a mess

Title: MZs_FAC_2.docx

Descriptor Info:	District: Mzimba South	Target Population: TP1 - Providers/HSAs	Respondent type: Facility-based provider	Aware of SI: Yes - had heard of SI before	Coding phase: 2. Double coded	Client marital status: N/A - provider	Client age group: N/A - provider	Time since trained:

Codes Applied:	P3.2.2 - Stockouts/stocks near expiry dates

Interviewer: Would you like more support?

Respondent: Laughing……… the training was okay, and we got all the information, but the problem is that we stopped for some time.

Interviewer: Mmmmmm… (following)

Respondent: [Quiet]…. but I think am okay. Only that the provision of Sayana has been on and off [out of stock], and what is needed is that once we have started providing it, we should continue instead of telling them that as of now we don’t have rather injecting her with Depo as a result it shoes as if we are not helping at all.

Interviewer: Alright.

Respondent: Mmmmmm…. (yes)

Title: MZs_FAC_2.docx

Descriptor Info:	District: Mzimba South	Target Population: TP1 - Providers/HSAs	Respondent type: Facility-based provider	Aware of SI: Yes - had heard of SI before	Coding phase: 2. Double coded	Client marital status: N/A - provider	Client age group: N/A - provider	Time since trained:

Codes Applied:	P3.2.2 - Stockouts/stocks near expiry dates	P3.5.1 - Trends/numbers of SI

Q6. Interviewer: What has the uptake of DMPA – SC been like at this facility in the past 3 months?

Respondent: I don’t know since I have been out for some time, and it was out of stock (not clear though)

Interviewer: Have you faced any challenges in providing DMPA–SC in the last 3 months? As you have said Sayana was out of stock, is there anything else?

Respondent: Since we have clients who started injecting themselves but because we had out of stock, clients may feel discouraged, and this may result as if we have failed as providers.

Interviewer: Mmmmmhh … (following)

Respondent: Mmmmm (yes)

Interviewer: Would you say the trend in DMPA –SC uptake for self-injection has changed over time?

Respondent: Yes, the trend has changed only that Sayana has been out of stock for some time now, but despite this stock out the figures were increasing.

Title: MZs_FAC_2.docx

Descriptor Info:	District: Mzimba South	Target Population: TP1 - Providers/HSAs	Respondent type: Facility-based provider	Aware of SI: Yes - had heard of SI before	Coding phase: 2. Double coded	Client marital status: N/A - provider	Client age group: N/A - provider	Time since trained:

Codes Applied:	P3.2.2 - Stockouts/stocks near expiry dates

Interviewer: Alright. We have come to an end of the questions I had for you today. Do you have any other thoughts or information you would like to share on the topic we have discussed today?

Respondent: Quiet…

Interviewer: Other thoughts or information that you would like to share

Respondent: The problem is that clients are not that familiar with the method like Depo

Interviewer: Mmmmmmm. (yes)

Respondent: We have head that this Depo will phase out and we will only have Sayana, yet the same Sayana it has been out of stock for some time now and clients were about to get used with the method but unfortunately it is out of stock and a client who comes for re-fill we tell them that there is no Sayana in steady we will just inject you Depo. So may be the government should help us so that Sayana should always be in stock every time and once clients have made a decision of using it if they come for re-fill, they should always find it.

Title: MZs_HSA_6.docx

Descriptor Info:	District: Mzimba South	Target Population: TP1 - Providers/HSAs	Respondent type: HSA	Aware of SI: Yes - had heard of SI before	Coding phase: 1. Single coded	Client marital status: N/A - provider	Client age group: N/A - provider	Time since trained: A year or more ago

Codes Applied:	P2.1.1 - Refresher training/mentorship	P3.2.2 - Stockouts/stocks near expiry dates

Interviewer: what kind of support would you want from the supervision team

Respondent: they should remind us, since it’s been a while, we took the training so they should remind us. They should also bring Sayana it’s been a while since it was out of stock

Title: MZs_HSA_6.docx

Descriptor Info:	District: Mzimba South	Target Population: TP1 - Providers/HSAs	Respondent type: HSA	Aware of SI: Yes - had heard of SI before	Coding phase: 1. Single coded	Client marital status: N/A - provider	Client age group: N/A - provider	Time since trained: A year or more ago

Codes Applied:	P3.2.2 - Stockouts/stocks near expiry dates	P3.5.1 - Trends/numbers of SI

Interviewer: in the past 3 months, out of 100 women how many took Sayana for self-injection

Respondent: as I have already said it’s been out of stock

Interviewer: is Sayana and Depo IM all out of stock

Respondent: all are out of stock. But if we would check in the register in three months out of 100 women who chose Sayana almost 85 or I should say 80 or below would choose self-injection

Interviewer: you mean the remaining 20 would ask you to inject them

Respondent: yes

Title: MZs_HSA_6.docx

Descriptor Info:	District: Mzimba South	Target Population: TP1 - Providers/HSAs	Respondent type: HSA	Aware of SI: Yes - had heard of SI before	Coding phase: 1. Single coded	Client marital status: N/A - provider	Client age group: N/A - provider	Time since trained: A year or more ago

Codes Applied:	P3.2.2 - Stockouts/stocks near expiry dates	P3.2 - Barrier, general/other

Interviewer: did you have challenges due to covid-19 on providing Sayana

Respondent: with the coming of covid-19 we did not have enough supplies to prove Sayana. We had few gloves at this facility which were prioritized to other departments. So this made us advocate Sayana for self-injection only injecting those who wouldn’t manage after counseling. We were washing our hands and sanitizing.

Interviewer: any other challenge that you met

Respondent: the main problem is that it is out of stock

Title: MZs_HSA_6.docx

Descriptor Info:	District: Mzimba South	Target Population: TP1 - Providers/HSAs	Respondent type: HSA	Aware of SI: Yes - had heard of SI before	Coding phase: 1. Single coded	Client marital status: N/A - provider	Client age group: N/A - provider	Time since trained: A year or more ago

Codes Applied:	P3.2.2 - Stockouts/stocks near expiry dates	C2.1.2 - Reduced trips to facility	C5.1.4 - Timely injection due to not having to rely on external factors

Interviewer: with passing of time, did the demand for Sayana increase?

Respondent: the demand kept increasing because of thee counselling we were giving them so many understood and chose Sayana because they would get for a year and stay home which reduces the costs of travelling.

Interviewer: what increased the demand for Sayana?

Respondent: the counselling we gave them

Interviewer: what else

Respondent: women were happy with the idea of self-injecting at their homes. They could come to the provider and found him not their so self-injecting at home solved that challenge. Women are complaining now that it is out of stock

Title: KK_HSA_6.docx

Descriptor Info:	District: Nkhotakota	Target Population: TP1 - Providers/HSAs	Respondent type: HSA	Aware of SI: Yes - had heard of SI before	Coding phase: 1. Single coded	Client marital status: N/A - provider	Client age group: N/A - provider	Time since trained: A year or more ago

Codes Applied:	P3.2.2 - Stockouts/stocks near expiry dates

NTERVIEWER: okay. You did that training, but what could have been improved on the training?

Time stamp: (10:05)

RESPONDENT: aaaaaah [thinking]

INTERVIEWER: what they should improve on?

RESPONDENT: All the parts of the training were good. But what I can say now is about the shortage of supply. Sometimes we are out of stock of the drug - as a result women are sent back. I would like to ask that we should have more stock in the community. Sometimes we are out of stock for one to two months. This is a weakness from the office where Sayana is sent from. That should be looked upon.

Title: KK_HSA_6.docx

Descriptor Info:	District: Nkhotakota	Target Population: TP1 - Providers/HSAs	Respondent type: HSA	Aware of SI: Yes - had heard of SI before	Coding phase: 1. Single coded	Client marital status: N/A - provider	Client age group: N/A - provider	Time since trained: A year or more ago

Codes Applied:	P3.2.2 - Stockouts/stocks near expiry dates	P3.5.1 - Trends/numbers of SI

INTERVIEWER: mmmhu. What has the uptake of DMPA-SC been like in your community for the past three months?

RESPONDENT: It is good. And in all the months it was good except for the month of September when we had stock out. People were coming and sent back home. But from August…October everything is fine and people are coming for Sayana,

Title: KK_HSA_6.docx

Descriptor Info:	District: Nkhotakota	Target Population: TP1 - Providers/HSAs	Respondent type: HSA	Aware of SI: Yes - had heard of SI before	Coding phase: 1. Single coded	Client marital status: N/A - provider	Client age group: N/A - provider	Time since trained: A year or more ago

Codes Applied:	P3.2.2 - Stockouts/stocks near expiry dates

NTERVIEWER: Have you faced any challenges with Sayana from August, September and October?

RESPONDENT: aaah, ayi. Aaah, ayi. Palibenso mavuto ambiri amene ndakumna nawo. Aaaah, no. I have not faced any challenges.

INTERVIEWER: okay. You have said that…

RESPONDENT: just stock outs.

INTERVIEWER: oh ok. When was this stock out?

Time stamp: (20:05)

RESPONDENT: mmmmh, September

INTERVIEWER: September

RESPONDENT: September.

Title: KK_HSA_6.docx

Descriptor Info:	District: Nkhotakota	Target Population: TP1 - Providers/HSAs	Respondent type: HSA	Aware of SI: Yes - had heard of SI before	Coding phase: 1. Single coded	Client marital status: N/A - provider	Client age group: N/A - provider	Time since trained: A year or more ago

Codes Applied:	P3.2.2 - Stockouts/stocks near expiry dates

INTERVIEWER: We have come to the end of the discussion we had today. Do you have any thoughts or information on the topics we have discussed?

RESPONDENT: Alright. I would like to thank you for this discussion. I would like to ask the ministry of health to help us people in hard-to-reach areas for example HSA’s so that we should be having enough stock of drugs because when we are put of stock, women who were doing good on this method, they become lazy because they keep on coming and we send them back because of stock outs. It could be better if the supply of drugs could be increased in our areas.

Title: MZs_FAC_1.docx

Descriptor Info:	District: Mzimba South	Target Population: TP1 - Providers/HSAs	Respondent type: Facility-based provider	Aware of SI: Yes - had heard of SI before	Coding phase: 3. Triple coded	Client marital status: N/A - provider	Client age group: N/A - provider	Time since trained: A year or more ago

Codes Applied:	P3.2.2 - Stockouts/stocks near expiry dates	P3.5.1 - Trends/numbers of SI

Q6. Interviewer: Alright. I just want to know may be out of 100 clients who have come for Sayana press, how many of the clients out of 100 who have come for self-injection?

Respondent: Out of 100?

Interviewer: Yes.

Respondent: Mmmm..

Interviewer: In the past 3 months.

Respondent: In the past 3 months unfortunately eeeeee, Sayana has been very scarce.

Interviewer: Mmmmmh.

Respondent: Soooo…, but in general when we had full stock of Sayana out of 100 I can put at 65.

Interviewer: For self-injection?

Respondent: Yes for self-injection

Title: MZs_FAC_1.docx

Descriptor Info:	District: Mzimba South	Target Population: TP1 - Providers/HSAs	Respondent type: Facility-based provider	Aware of SI: Yes - had heard of SI before	Coding phase: 3. Triple coded	Client marital status: N/A - provider	Client age group: N/A - provider	Time since trained: A year or more ago

Codes Applied:	P3.2.2 - Stockouts/stocks near expiry dates	C1.1.3 - Awareness of DMPA-SC SI

Interviewer: Wow. Have faced any challenges in providing DMPA-SC in the last 3 months? For example may be because of covid 19 or stock out?

Respondent: Aaaaah, mainly stock outs and it is really hindering it because somebody comes for this method and may be its stock out and some even come to say I want this method cause may be because they have heard it from their friends and they come and you say now I think we don’t have, but I think stock out is the main challenges we are facing , but otherwise we haven’t met any challenges.

Title: ZA_HSA_1.docx

Descriptor Info:	District: Zomba	Target Population: TP1 - Providers/HSAs	Respondent type: HSA	Aware of SI: Yes - had heard of SI before	Coding phase: 1. Single coded	Client marital status: N/A - provider	Client age group: N/A - provider	Time since trained: Less than a year ago

Codes Applied:	P3.2.2 - Stockouts/stocks near expiry dates

Interviewer: Alright thank you very much. What has the uptake of DMPA_SC been like at in this community

Respondent: Ahh, a lot of people have been taking it

Interviewer: Umm

Respondent: They love it a lot

Interviewer: You however mentioned that you stopped at this hospital

Respondent: Umm, when going for outreach we take depo

Interviewer: Umm

Respondent: Sayana has gone missing, a few months have gone without us taking Sayana. We take Depo instead

Interviewer: Umm

Respondent: Umm

Interviewer: We are talking about Sayana, how is the uptake currently?

Respondent: We haven’t had Sayana am not sure if it has come

Interviewer: Umm

Respondent: We use Depo; however people also take Sayana a lot

Interviewer: Oh ok, alright

Respondent: If its available, we inject Sayana

Interviewer: Have you faced any challenges in providing DMPA-SC in the last 3 months

Respondent: We haven’t been administering Sayana for the past 3 months because we did not have it

Interviewer: Nothing?

Title: ZA_HSA_1.docx

Descriptor Info:	District: Zomba	Target Population: TP1 - Providers/HSAs	Respondent type: HSA	Aware of SI: Yes - had heard of SI before	Coding phase: 1. Single coded	Client marital status: N/A - provider	Client age group: N/A - provider	Time since trained: Less than a year ago

Codes Applied:	P3.2.2 - Stockouts/stocks near expiry dates	P3.5.1 - Trends/numbers of SI

Interviewer: Alright thank you very much. Roughly what proportion of DMPA-SC provided for self-injection? We are saying that you give out the two types of injectables. As so the Sayana, for instance you have given out 10, how many people would get Sayana and out of those how many would self-inject?

Respondent: Self inject?

Interviewer: Umm

Respondent: I would say that a lot of them are afraid

Interviewer: Umm

Respondent: To self-inject

Interviewer: Umm

Respondent: Most of them say that we should inject then. Out of 10 people 4 would agree to self-inject

Interviewer: Alright thank you very much

Respondent: We still encourage them to learn on how to self-inject

Interviewer: Alright, has the trend in self-injection changed over time?

Respondent: That will be very hard for me as we haven’t had any for the past 3 months

Interviewer: Umm, alright does it mean that things have changed?

Respondent: Yes, because it is not available

Title: ZA_HSA_1.docx

Descriptor Info:	District: Zomba	Target Population: TP1 - Providers/HSAs	Respondent type: HSA	Aware of SI: Yes - had heard of SI before	Coding phase: 1. Single coded	Client marital status: N/A - provider	Client age group: N/A - provider	Time since trained: Less than a year ago

Codes Applied:	P3.2.2 - Stockouts/stocks near expiry dates

Interviewer: Alright thank you very much, we have now come to the end of the questions I had for you today. Do you have any other thoughts or information you would like to share on the topics we have discussed today?

Respondent: Is Sayana available, is it available at the DHO?

Title: MZs_HSA_2.docx

Descriptor Info:	District: Mzimba South	Target Population: TP1 - Providers/HSAs	Respondent type: HSA	Aware of SI: Yes - had heard of SI before	Coding phase: 1. Single coded	Client marital status: N/A - provider	Client age group: N/A - provider	Time since trained: A year or more ago

Codes Applied:	P2.1.2 - Sufficient original provider training	P3.2.2 - Stockouts/stocks near expiry dates

Interviewer: the way the training was conducted, what do you think would have done better

Respondent: generally all was well and after the training we started providing. However the main challenge is that it is now out of stock we don’t know why

Title: MZs_HSA_2.docx

Descriptor Info:	District: Mzimba South	Target Population: TP1 - Providers/HSAs	Respondent type: HSA	Aware of SI: Yes - had heard of SI before	Coding phase: 1. Single coded	Client marital status: N/A - provider	Client age group: N/A - provider	Time since trained: A year or more ago

Codes Applied:	P3.2.2 - Stockouts/stocks near expiry dates

Interviewer: what do you think could have been done better during the supervision

Respondent: they would have brought Sayana because right now it’s out of stock

Title: MZs_HSA_2.docx

Descriptor Info:	District: Mzimba South	Target Population: TP1 - Providers/HSAs	Respondent type: HSA	Aware of SI: Yes - had heard of SI before	Coding phase: 1. Single coded	Client marital status: N/A - provider	Client age group: N/A - provider	Time since trained: A year or more ago

Codes Applied:	P3.2.2 - Stockouts/stocks near expiry dates

Interviewer: has your views about Sayana Press changed with time

Respondent: yes, because Sayana is now out of stock and women who were using have been put back on Depo

Title: MZs_HSA_2.docx

Descriptor Info:	District: Mzimba South	Target Population: TP1 - Providers/HSAs	Respondent type: HSA	Aware of SI: Yes - had heard of SI before	Coding phase: 1. Single coded	Client marital status: N/A - provider	Client age group: N/A - provider	Time since trained: A year or more ago

Codes Applied:	P3.2.2 - Stockouts/stocks near expiry dates	P3.1.1 - Reduced provider workload

Interviewer: so has your view about Sayana changed, earlier you mentioned that you were happy with the coming of Sayana because it would reduce workload. Now with the passing of time from the time you started providing to now, has your views changed on Sayana Press self-inject

Respondent: the change is that when we had Sayana we had reduced workload but now we see a lot of clients

Interviewer: I would like to know the proportion of women who take Sayana for self-inject at this facility

Respondent: it’s been out of stock

Interviewer: what do you mean

Respondent: because we do not have Sayana it’s been a year now without Sayana

Title: MZs_HSA_2.docx

Descriptor Info:	District: Mzimba South	Target Population: TP1 - Providers/HSAs	Respondent type: HSA	Aware of SI: Yes - had heard of SI before	Coding phase: 1. Single coded	Client marital status: N/A - provider	Client age group: N/A - provider	Time since trained: A year or more ago

Codes Applied:	P3.5 - Data capture, general	P3.2.2 - Stockouts/stocks near expiry dates

Interviewer: how easy or difficult was it to get data on Sayana Press and at the same time Depo Provera

Respondent: the only challenge so far is that we were recording a lot of women for Depo since Sayana is out of stock

Interviewer: the time you had Sayana, did you have any challenges in recording

Respondent: no

Interviewer: were you recording all clients? Did you have any missing data?

Respondent: we were able to record all

Interviewer: did you have adequate forms

Respondent: yes

Interviewer: did you have adequate registers

Respondent: yes

Interviewer: is the data you record consistent and without errors all the time

Respondent: we always record correctly

Interviewer: are you able to differentiate records for Sayana Press and Depo IM

Respondent: yes

Title: MZs_HSA_2.docx

Descriptor Info:	District: Mzimba South	Target Population: TP1 - Providers/HSAs	Respondent type: HSA	Aware of SI: Yes - had heard of SI before	Coding phase: 1. Single coded	Client marital status: N/A - provider	Client age group: N/A - provider	Time since trained: A year or more ago

Codes Applied:	P3.2.2 - Stockouts/stocks near expiry dates

Interviewer: in your view, what would discourage women from using Sayana Press

Respondent: the main reason is that Sayana is out of stock, so most women are back to Depo Provera. This is a challenge

Interviewer: what else

Respondent: mmmmh (thinking out loud) that’s all

Title: MZs_HSA_2.docx

Descriptor Info:	District: Mzimba South	Target Population: TP1 - Providers/HSAs	Respondent type: HSA	Aware of SI: Yes - had heard of SI before	Coding phase: 1. Single coded	Client marital status: N/A - provider	Client age group: N/A - provider	Time since trained: A year or more ago

Codes Applied:	P3.2.2 - Stockouts/stocks near expiry dates

Interviewer: we have now come to the end of our interview, maybe you have something to share with me you can do so

Respondent: you came to do a research with an emphasis on Sayana Press. Why is Sayana out of stock?

Title: KK_FAC_2.docx

Descriptor Info:	District: Nkhotakota	Target Population: TP1 - Providers/HSAs	Respondent type: Facility-based provider	Aware of SI: Yes - had heard of SI before	Coding phase: 1. Single coded	Client marital status: N/A - provider	Client age group: N/A - provider	Time since trained: A year or more ago

Codes Applied:	P3.2.2 - Stockouts/stocks near expiry dates	C5.2.2 - Clients incorrectly / failing to administer at home

INTERVIEWER: Mmmhu. Have you faced any challenges in providing DMPA-SC in the last 3 months? Have you faced any challenges?

RESPONDENT: The last three months? [asked the question in a low tone he was thinking].

INTERVIEWER: Mmmh, it should be from August…mmm, July, August, September. Have you faced any challenges?

RESPONDENT: okay. Although I don’t remember the exact period, a certain woman was given four units, including the one she injected at the facility. She injected the others and said that one was damaged. That’s the challenge.

INTERVIEWER: Okay. That is the challenge that you have faced?

RESPONDENT: Second, we have stock outs of Sayana. Which means demand is still high.

INTERVIEWER: stock out?

RESPONDENT: Yes stock out.

INTERVIEWER: mmmmhu. The past three months.

RESPONDENT: And if we go beyond the past three months, we had a very serious stockout in which Sayana wasn’t available at this facility.

INTERVIEWER: okay. Which means for the past three months there was stock out but you could have Sayana every now and then?

RESPONDENT: yes.

INTERVIEWER: Okay. But in the past, you could not find Sayana.

RESPONDENT: Yes, in the past.

Title: KK_FAC_2.docx

Descriptor Info:	District: Nkhotakota	Target Population: TP1 - Providers/HSAs	Respondent type: Facility-based provider	Aware of SI: Yes - had heard of SI before	Coding phase: 1. Single coded	Client marital status: N/A - provider	Client age group: N/A - provider	Time since trained: A year or more ago

Codes Applied:	C5.2.1 - Discontinuation of SI (including reasons)	P3.2.2 - Stockouts/stocks near expiry dates

INTERVIEWER: Okay. Now I just want to know the factors you think would discourage clients to take up Sayana. Why can they be discouraged not to take up this method?

RESPONDENT: we have also already discussed about this question.

INTERVIEWER: mmmmhu.

RESPONDENT: The reasons why women can be discouraged to use Sayana is firstly if there will be shortage of supply. Secondly, if they will not get an explanation on the side effects...the problems they can face when using Sayana although they do not happen to everyone. If they will not get a proper explanation, they will go back home and say that Sayana is doing this and that to me. Also if there is a problem, the client should be assisted properly because what they need is that when they explain their problem, they have to be counselled and helped and there after tell her that not all problems that you experience are Sayana related. Most of the clients say that when they are asked they say they have body pains because of a contraception method or maybe I am menstruating because of contraception whilst it is the normal monthly period. We need to re-think on these issues.
