# Supplementary material for: Health care provider and client experiences of counselling on depot medroxyprogesterone acetate subcutaneous (DMPA-SC) for self-injection in Malawi
Source: PLOS Glob Public Health. 2023 Nov 30;3(11):e0002057. doi: 10.1371/journal.pgph.0002057 (PMC10688740; doi:10.1371/journal.pgph.0002057)
Supplement: S1 Data — (ZIP) [file pgph.0002057.s001.zip › S2/P3.3 couns approach FAC only.doc]

Title: KK_FAC_1.docx

Descriptor Info:	District: Nkhotakota	Target Population: TP1 - Providers/HSAs	Respondent type: Facility-based provider	Aware of SI: Yes - had heard of SI before	Coding phase: 3. Triple coded	Client marital status: N/A - provider	Client age group: N/A - provider	Time since trained: A year or more ago

Codes Applied:	P3.3 - Counselling/ training approach, general

INTERVIEWER: Alright, are there any methods that you do not usually counsel clients about? If so why

RESPONDENT: We encourage women to take any method they would like to; it depends on one’s choice.

Title: KK_FAC_1.docx

Descriptor Info:	District: Nkhotakota	Target Population: TP1 - Providers/HSAs	Respondent type: Facility-based provider	Aware of SI: Yes - had heard of SI before	Coding phase: 3. Triple coded	Client marital status: N/A - provider	Client age group: N/A - provider	Time since trained: A year or more ago

Codes Applied:	P3.3 - Counselling/ training approach, general

INTERVIEWER: Ok in this section of discussions, I would like you to take me through how you counsel a client who is considering an injection contraceptive. To begin with, how would you greet the client?

RESPONDENT: Ok

INTERVIEWER: Yes

RESPONDENT: When clients are here, we welcome them as a group

INTERVIEWER: Ok

RESPONDENT: We counsel them as a group and give them information on how all the family planning methods works as a group. Whilst in a group we don’t really ask them what type of FP have they chosen. But when they are now in a room where they take their FP methods that’s when we ask them which method they want. Now from the method chosen we go deeper in counselling the client because it is the method that the client will use for some time. And then we tell the client the good side and effects of the choice made.

INTERVIEWER: Alright, for instance you would like to counsel a client how do you go about it?

RESPONDENT: Okay counselling is done, let me say that we are now sed to what to counsel our clients on

INTERVIEWER: Alright I see

RESPONDENT: Initially we used to have flip charts that have information on FP methods , but now since we do not have so we just explain to our clients on the methods available at our facility.

INTERVIEWER: Alright

RESPONDENT: Sure

INTERVIEWER: Would you ask the clients any question?

RESPONDENT: Yes, after counselling we ask them to explain on the methods that they have understood and the good side and defects of those methods.

INTERVIEWER: Let’s assume the client was only interested I taking up an injection option, how would you explain their options?

RESPONDENT: Likeee ( participants did not get the question and was trying to figure out on what to answer)

INTERVIEWER: How many options would you counsel them? and in what order

RESPONDENT: When someone would like to take injectable, we explain that we do have two types of injectables. There is one that a client has to inject herself and the one that is administered by the provider. So we normally start explaining on the self-injection one that the advantage of this one is that a client who choses this method will have reduced visits to the facility because we give you doses that will last you for a year. Whilst if you take the one that is administered by the provider you need to be coming here every three months to get injected, Which means you will visit the facility four times. But for the first one the client will be home for a year.

Title: KK_FAC_1.docx

Descriptor Info:	District: Nkhotakota	Target Population: TP1 - Providers/HSAs	Respondent type: Facility-based provider	Aware of SI: Yes - had heard of SI before	Coding phase: 3. Triple coded	Client marital status: N/A - provider	Client age group: N/A - provider	Time since trained: A year or more ago

Codes Applied:	P3.1.2 - Provider skill/ability to effectively counsel on SI	P3.3 - Counselling/ training approach, general

INTERVIEWER: So you earlier explained that most women are afraid to inject themselves

RESPONDENT: Yes

INTERVIEWER: So how do you manage that?

RESPONDENT: When training the clients, we give them an example of patients with sugar disease. That these patients they do inject themselves every day morning and evening. So we counsel them that if someone is able to inject herself every day, how would you fail to inject yourself only once in three months. Hence most of the clients understands. We also explain to them that the injection used for this method is very small that cannot hurt them

INTERVIEWER: Yes

RESPONDENT: It very small hence it cannot cause much pain when used on someone’s body

Title: KK_FAC_1.docx

Descriptor Info:	District: Nkhotakota	Target Population: TP1 - Providers/HSAs	Respondent type: Facility-based provider	Aware of SI: Yes - had heard of SI before	Coding phase: 3. Triple coded	Client marital status: N/A - provider	Client age group: N/A - provider	Time since trained: A year or more ago

Codes Applied:	P3.4 - Time to counsel/train, general	P3.3 - Counselling/ training approach, general

INTERVIEWER: How long on average does it train a client in DMPA-SC?

RESPONDENT: We take a maximum of ten minutes for someone to clearly understand

INTERVIEWER: Yes

RESPONDENT: The bigger part is already done during group counselling

INTERVIEWER: Yes

RESPONDENT: In a counselling room we mainly dwell on technical part of it like teaching on how one can effectively inject herself. Though during group counselling we do some demonstration but when we have the client alone we go deeper so that they master the method properly.

INTERVIEWER: What about for adolescents whom you said they easily capture information, how would you compare the time you spend with then in counselling with adults .

RESPONDENT: For the adolescents it little time

INTERVIEWER: Its little time not so?

RESPONDENT: Yes

INTERVIEWER: Do you feel you have enough time to conduct these trainings? what about on busy days?

RESPONDENT: If always have time since the client is here for help

INTERVIEWER: Sure but is the time always enough to train the client’s when they visit the facility?

RESPONDENT: may be 5 to 6 people at time. For example Thursday is a busy day since it’s a family planning day hence we have a lot of work to do.

Title: KK_FAC_1.docx

Descriptor Info:	District: Nkhotakota	Target Population: TP1 - Providers/HSAs	Respondent type: Facility-based provider	Aware of SI: Yes - had heard of SI before	Coding phase: 3. Triple coded	Client marital status: N/A - provider	Client age group: N/A - provider	Time since trained: A year or more ago

Codes Applied:	P3.3 - Counselling/ training approach, general	P3.4 - Time to counsel/train, general

INTERVIEWER: Does the length of the training influence whether some women choose to take up self-injection?

RESPONDENT: It is a bit tricky to know how much time is spend on a particular method since counselling is done in a group

INTERVIEWER: Ok

RESPONDENT: because we train women in a group and then makes a choice on the method they want.

INTERVIEWER: Ok the from that point?

RESPONDENT: Yes

Title: ZA_FAC_1.docx

Descriptor Info:	District: Zomba	Target Population: TP1 - Providers/HSAs	Respondent type: Facility-based provider	Aware of SI: Yes - had heard of SI before	Coding phase: 2. Double coded	Client marital status: N/A - provider	Client age group: N/A - provider	Time since trained: Less than a year ago

Codes Applied:	P3.3 - Counselling/ training approach, general

Interviewer: Alright, among the methods that you have mentioned, are there any methods that you do not encourage use?

Respondent: if we say that we don’t encourage use a lot

Interviewer: mmmm (showing that one is following the conversation)

Respondent: we will be wrong, what we don’t encourage is that women should not be using contraceptives.

Interviewer: mmmm (showing that one is following the conversation)

Respondent: that we don’t encourage

Interviewer: mmmm (showing that one is following the conversation)

Respondent: but we try to give out information so that the person should not say that I have failed to get contraceptives because all the methods don’t suit me.

Interviewer: mmmm (showing that one is following the conversation)

Respondent: we try to give her the information that when she says that I have problems with Depo

Interviewer: mmmm (showing that one is following the conversation)

Respondent: the pills should be OK for her

Interviewer: mmmm (showing that one is following the conversation)

Respondent: or I have problems with pills, condoms should be available

Interviewer: mmmm (showing that one is following the conversation)

Respondent: or say I find problems with condoms, then Norplant is available.

Interviewer: mmmm (showing that one is following the conversation)

Respondent: so what we don't encourage is not accessing family planning

Interviewer: mmmm (showing that one is following the conversation)

Respondent: but family planning is what we encourage

Interviewer: alright

Respondent: yes

Title: ZA_FAC_1.docx

Descriptor Info:	District: Zomba	Target Population: TP1 - Providers/HSAs	Respondent type: Facility-based provider	Aware of SI: Yes - had heard of SI before	Coding phase: 2. Double coded	Client marital status: N/A - provider	Client age group: N/A - provider	Time since trained: Less than a year ago

Codes Applied:	P3.3 - Counselling/ training approach, general	P3.3.2 - Inclusion of practice opportunities for clients in training

Respondent: when we train the client, we tell her that OK it's up to you either we inject you or you self-inject since there are two options. so when the client is trained and sees that it has been demonstrated that she can be able to self-inject

Interviewer: mmmm (showing that one is following the conversation)

Respondent: they are supposed to self-inject while we are there and observing them

Interviewer: alright

Respondent: there is a specific location on the boarding that they are supposed to inject on, on the thigh, around the navel

Title: ZA_FAC_1.docx

Descriptor Info:	District: Zomba	Target Population: TP1 - Providers/HSAs	Respondent type: Facility-based provider	Aware of SI: Yes - had heard of SI before	Coding phase: 2. Double coded	Client marital status: N/A - provider	Client age group: N/A - provider	Time since trained: Less than a year ago

Codes Applied:	P3.3 - Counselling/ training approach, general	P0 - General method mix / method provision

Interviewer: Thank you, ah now I would like you to talk me through how you counsel a client who is considering an injectable contraceptive. I would like as much detail as possible, so please break down the steps you would take in counselling this client, to begin how would you greet them, or how would you welcome them?

Respondent: okay (clears throat) okay so when the client comes normally since this is an OPD clinic, there are other clients that have also come for other services like neonatal clinic, postnatal services and other services.

Interviewer: mmmm (showing that one is following the conversation)

Respondent we make sure there is a health talk which is brief and general

Interviewer: mmmm (showing that one is following the conversation)

Respondent: after that we separate them based on the service they have come to seek, for example those who have come for safe motherhood go to this room,

Interviewer: mmmm (showing that one is following the conversation)

Respondent: when they come here, including men when they happen to show up, because some of the women are accompanied by their husbands.

Interviewer: mmmm (showing that one is following the conversation)

Respondent: so we introduce ourselves

Interviewer: mmmm (showing that one is following the conversation)

Respondent: after introducing ourselves, our name and who you are, you then tell them that they should be free in everything that we will discuss here because some of them have come here with their own issues and their moods are not normal.

Interviewer: mmmm (showing that one is following the conversation)

Respondent: so you make sure that the women are flexible, if they are not flexible you try to at least shift their attention

Interviewer: mmmm (showing that one is following the conversation)

Respondent: like we ask anyone to lead us into a song

Interviewer: mmmm (showing that one is following the conversation)

Respondent: after we pray, before we start every health talk, we are supposed to pray, but if we have already prayed during the general health talk then we just go on. After the song is done, or after motivating them with anything, something like an ice breaker with morale, you always read their mood and you observe how it is,

Interviewer: mmmm (showing that one is following the conversation)

Respondent: you can know that the clients that have come today are very free.

Interviewer: mmmm (showing that one is following the conversation)

Respondent: so for those ones you know that you won’t take time with them because you know that the rapport

Interviewer: is already good

Respondent: its already good, so first of all you need to build a good rapport

Interviewer: mmmm (showing that one is following the conversation)

Respondent: When you are sure that the rapport is good then you welcome them and say that welcome to the clinic, a lot happens here, this room is for family planning, this room is for doing check-ups, this room is for youth friendly services,

TIME INTO THE INTERIEW: 30Mins

Interviewer: mmmm (showing that one is following the conversation)

Respondent: then you start to focus on the area that you are interested in that day, the service that you want to focus on like family planning, so we will start about family planning that at this facility we have these family planning services that we provide

Interviewer: alright.

Respondent: we have Norplant, it lasts for three years, we have pills, the pills are of three groups

Interviewer: mmmm (showing that one is following the conversation)

Respondent: we have injectables, there are two groups of injectables, there is Sayana, there is Depo which we already know, there is also another method loop, this one we insert it on the cervix

Interviewer: mmmm (showing that one is following the conversation)

Respondent: after that you start explaining more about each of those methods.

Interviewer: mmmm (showing that one is following the conversation)

Respondent: you explain more about each, for example loop last for 10 years.

Interviewer: mmmm (showing that one is following the conversation)

Respondent: this one is inserted on the cervix

Interviewer: mmmm (showing that one is following the conversation)

Respondent: it is easy to insert it

Interviewer: mmmm (showing that one is following the conversation)

Respondent: so we make sure to take a sample of loop and give them to see it, if they hold it themselves, they will be aware of the material it is made from, that it’s not metal that it could be painful.

Interviewer: mmmm (showing that one is following the conversation)

Respondent: so they are supposed to at least they should feel

Interviewer: mmmm (showing that one is following the conversation)

Respondent: as you are talking about the method the lop for demonstration is going around

Interviewer: mmmm (showing that one is following the conversation)

Respondent: and then you tell them that when you insert this loop

Interviewer: mmmm (showing that one is following the conversation)

Respondent: since it is a foreign body and it has been introduced in your body, sometimes you may feel a stomachache

Interviewer: mmmm (showing that one is following the conversation)

Respondent: because the body has received something external and new

Interviewer: mmmm (showing that one is following the conversation)

Respondent: but this happens only for a few days

Interviewer: mmmm (showing that one is following the conversation)

Respondent: and sometimes this requires good hygiene practices

Interviewer: mmmm (showing that one is following the conversation)

Respondent: and you need to make sure you check yourself that the loop should show as you are bathing, some people may have rumors that women who use loop are not sweet in bed, so make sure that the things that the people hear and haven’t heard

Interviewer: mmmm (showing that one is following the conversation)

Respondent: you’ve included them in your talk

Interviewer: yah yah

Respondent: after explaining about this method, you will come to another method.

Interviewer: mmmm (showing that one is following the conversation)

Respondent: this is an injectable, and we call it Depo.

Interviewer: mmmm (showing that one is following the conversation)

Respondent: after mentioning Depo, you will also show Sayana, the liquid inside is the same, they look similar but currently the Malawi government and the ministry of health and the WHO have introduced this injection due to the difficulties that we used to face

Interviewer: mmmm (showing that one is following the conversation)

Respondent: you need to let the people know that they are the same, secondly the slight difference that is there.

Interviewer: mmmm (showing that one is following the conversation)

Respondent: You need to tell them that the injectable is taken every three months, this injectable is supposed to be administered to you by a provider, for this one Sayana, we can inject you or you can also self-inject.

Interviewer: yah

Respondent: that’s another difference

Interviewer: mmmm (showing that one is following the conversation)

Respondent: you are supposed to get the injection every three months

Interviewer: mmmm (showing that one is following the conversation)

Respondent: but what is here is the medication, you need to tell them that, then you will bring another method, these are pills, you need to take them every day, every other day at the same time

Interviewer: mmmm (showing that one is following the conversation)

Respondent: most of the time it’s better to tale them in the evening, because sometimes the pills can make you feel dizzy, so it is better to take them in the evening

Interviewer: yah yah.

Respondent: but they don’t bring any problem, they just protect from getting pregnant.

Interviewer: mmmm (showing that one is following the conversation)

Respondent: you then come to another method, these are condoms, these are for men, they wear them before they have sex. They will do foreplay and then when ready to have sex they will put on the condom.

Interviewer: alright.

Respondent: yeah, after we explain that we tell them that these methods have inducements while these ones don’t have, these methods are long term methods while these are short term methods,

Interviewer: mmmm (showing that one is following the conversation)

Respondent: we tell them that they chose the method depending on their future plans

Interviewer: yah

Respondent: as we are doing that then we are splitting the women so that everyone can focus on the method that appeals to them.

Interviewer: mmmm (showing that one is following the conversation)

Respondent: we don’t force the client but guide them the direction that they need to take.

Interviewer: mmmm (showing that one is following the conversation)

Respondent: we tell them that the methods will protect them from pregnancy, but they will not protect them from STIs except for the condoms

Interviewer: mmmm (showing that one is following the conversation)

Respondent: But some of these methods do not protect from STIs then we tell them that some people come with side effects

Interviewer: yah yah

Respondent: some come that they are experiencing spotting and some experience heavy bleeding

Interviewer: mmmm (showing that one is following the conversation)

Respondent: those who experience heavy bleeding should find us at the facility.

Interviewer: mmmm (showing that one is following the conversation)

Respondent: some people gain weight

Interviewer: mmmm (showing that one is following the conversation)

Respondent: some people that were slim, we make sure that when we communicate these things they should not sound like a burden.

Interviewer: mmmm (showing that one is following the conversation)

Respondent: but we should explain it the way that it is

Interviewer: mmmm (showing that one is following the conversation)

Respondent: some people are slim, but they still want to gain weight, gaining weight is not a problem.

Interviewer: mmmm (showing that one is following the conversation)

Respondent: we make sure that we address these issues

Interviewer: mmmm (showing that one is following the conversation)

Respondent: what people want cannot be common, so some people may have migraine headache

Interviewer: mmmm (showing that one is following the conversation)

Respondent: eh some may experience dizziness.

Interviewer: mmmm (showing that one is following the conversation)

Respondent: eh some may have heart palpitations

Interviewer: yah

Respondent: we make sure to explain misconceptions, we ask them what things they have heard about contraceptives, so they are able to tell us the things they have heard

Interviewer: mmmm (showing that one is following the conversation)

Respondent: we then iron out those misconceptions and tell them that someone who’s on family planning can also be sick and it’s not due to the use of family planning.

Interviewer: alright

Respondent: yes

Interviewer: Do you ask them any questions as you are telling them this?

Respondent: yes, firstly

Interviewer: mmmm (showing that one is following the conversation)

Respondent: before we tell them about the methods

Interviewer: mmmm (showing that one is following the conversation)

Respondent: we need to know of the prerequisite knowledge they have

Interviewer: mmmm (showing that one is following the conversation)

Respondent: what do they know about family planning.

Interviewer: yah yah

Respondent: what do they say and think

Interviewer: mmmm (showing that one is following the conversation)

Respondent: we then confirm what they say

Interviewer: mmmm (showing that one is following the conversation)

Respondent: that’s indeed what family planning is.

Interviewer: mmmm (showing that one is following the conversation)

Respondent: everyone speaks on what they think family planning is

Interviewer: mmmm (showing that one is following the conversation)

Respondent: or if they heard of the problems that are there

Interviewer: mmmm (showing that one is following the conversation)

Respondent: iwo aja ndiye they begin to say the problems they have heard, then we train them on the correct information and iron out the incorrect things

Interviewer: yah yah

Respondent: things like those we then ask them if there is anyone with experience with those methods to speak out on how they found it when they were using

Interviewer: yah yah

Respondent: the resource person helps to clear out the incorrect things

Interviewer: mmmm (showing that one is following the conversation)

Respondent: so we try to involve them

Interviewer: mmmm (showing that one is following the conversation)

Respondent: even those that are shy

Interviewer: yah yah

Respondent: they can still stand and speak from their own experience

Interviewer: Alright, do you use any resources when training women in DMPA-SC?

Respondent: yes

Interviewer: Alright what resources?

Respondent: Calendars

Interviewer: yah

Title: ZA_FAC_2.docx

Descriptor Info:	District: Zomba	Target Population: TP1 - Providers/HSAs	Respondent type: Facility-based provider	Aware of SI: Yes - had heard of SI before	Coding phase: 3. Triple coded	Client marital status: N/A - provider	Client age group: N/A - provider	Time since trained: Less than a year ago

Codes Applied:	P3.3 - Counselling/ training approach, general

Interviewer: Okay, thank you very much. What does uh, your typical day look like? Your typical day at work, what do you do when you get to work?

Respondent: When I get to work, first thing, I do health education; I educate my clients about the methods, the indications, the side effects, the complications, so that the client should know more about the methods and should be able to make decisions on their own, to make the decision on which family planning method they’re going to take. Yeah.

Title: ZA_FAC_2.docx

Descriptor Info:	District: Zomba	Target Population: TP1 - Providers/HSAs	Respondent type: Facility-based provider	Aware of SI: Yes - had heard of SI before	Coding phase: 3. Triple coded	Client marital status: N/A - provider	Client age group: N/A - provider	Time since trained: Less than a year ago

Codes Applied:	P3.3 - Counselling/ training approach, general

Interviewer: Thank you very much. Out of the methods that you have explained about, are there any methods that you do not usually counsel clients about?

Respondent: Yeah, like the one we use the beads, the cycle beads. I don’t usually give health education on that because it’s very sensitive you know the cycle can change anytime so if I dwell much on the cycle beat, I think there can be much pregnancies. So I don’t dwell much on the cycle beads.

Interviewer: Okay.is there anything you would like to add on that as well?

Respondent: Eheh, another method that I don’t like talking about it’s the, they say the ‘withdrawal method’; the natural family planning method. So I don’t dwell much on that because its 50/50. If I teach them, the chances of getting pregnant is very high.

Interviewer: okay. Any other method that you did not talk about?

Respondent: No

Title: ZA_FAC_2.docx

Descriptor Info:	District: Zomba	Target Population: TP1 - Providers/HSAs	Respondent type: Facility-based provider	Aware of SI: Yes - had heard of SI before	Coding phase: 3. Triple coded	Client marital status: N/A - provider	Client age group: N/A - provider	Time since trained: Less than a year ago

Codes Applied:	P3.3 - Counselling/ training approach, general

Interviewer: Are you someone who encourages women to do the self-injection method?

Respondent: Yes.

Interviewer: How do you do this?

Respondent: By teaching them. When teaching we also demonstrate to them so they can determine if they can manage to do it on their own or not.

Title: ZA_FAC_2.docx

Descriptor Info:	District: Zomba	Target Population: TP1 - Providers/HSAs	Respondent type: Facility-based provider	Aware of SI: Yes - had heard of SI before	Coding phase: 3. Triple coded	Client marital status: N/A - provider	Client age group: N/A - provider	Time since trained: Less than a year ago

Codes Applied:	P3.3 - Counselling/ training approach, general	P3.1.2 - Provider skill/ability to effectively counsel on SI	C4 - Storage of unused units

Interviewer: Okay thanks, we are now entering another section. In this section, I want you to explain how you provide counsel for a woman who is considering to start using the self-injection method? Please explain in detail the steps you would follow in the provision of the counsel. Firstly, how would you greet a woman?

Respondent: A woman who has come to get family planning?

Interviewer: Yes.

Respondent: When they come, we welcome them, “you are welcome here, how are you?”, “am fine and you?”, “am fine as well, how may I help you?”	We move straight to that question because the women receive health education outside before they come inside to meet with the nurse one on one. So they respond, “I want to take the self-injection family planning method”. We ask them what they know about the family planning method. Some explain according to how they got it from the health education they received previously. So we just add more on that information by telling them the procedure when administering the method. We further explain to them by showing them the difference between the self-injection method which stays in the body for 3 months and the depo method. We also enlighten them that taking the self-injection family planning method does not mean they are protected from sexually transmitted diseases for example H.I.V. or syphilis or gonorrhea. We advise them to also be using condoms to avoid sexually transmitted diseases. We also show them the drugs and explain how they are supposed to administer the drugs. Precisely, they need to wash hands, open the injection package, then mix the drugs by shaking the injection for a quarter minute using the neck till the drug is properly mixed. After that we tell them to ensure that the bottle of drugs is not cracked or leaking. If for example, one changes her mind to do it later, perhaps she is busy with something, but they already shook the drug, we tell them to make sure they shake it again at the time they are ready to administer the drug. To administer, there is need to close the gap between the neck and the lid of the bottled drug. Then hold the bottle at the neck and point the needle of the injection upwards to prevent it from spilling out. The lid has to be properly closed because failure to do so makes pushing the drugs from the injection hard. Reaching this point, after removing the lid, the lid will not be put back and then the drug is administered into the body. This is done at 3 different positions in the body; the stomach, the thigh or behind the hand where there is more muscle. So we tell them that after shaking the drug, you administer where you prefer. If it’s the stomach, you will push the drug into the skin. Then you will have to hold the neck of the injection till it reaches the skin. Push the injection close to the skin till it touches the skin. Push the drug into the body little by little till the whole drugs gets into the skin. Pushing the drug at one time may cause pain when administering it. The place where the drug is administered is not supposed to be rubbed. The injection that was used can be put in a carton or a box and handed over to the responsible health official since they have the correct knowledge on how to dispose them.

Title: ZA_FAC_2.docx

Descriptor Info:	District: Zomba	Target Population: TP1 - Providers/HSAs	Respondent type: Facility-based provider	Aware of SI: Yes - had heard of SI before	Coding phase: 3. Triple coded	Client marital status: N/A - provider	Client age group: N/A - provider	Time since trained: Less than a year ago

Codes Applied:	P3.3 - Counselling/ training approach, general

Interviewer: I’ve come to you with the desire to take the self-injection method, how can you explain to me about the other choices which I have that I can also choose at this hospital? You talked about pills and others. How can you advise me about those methods too?

Respondent: Okay, the issue is that we teach the different family planning methods, and it is the choice of the woman herself to select which she prefers because we tell them both the advantages of a particular family planning method as well as the disadvantage. We also explain to them the that it’s not everyone who encounters problems with the method for instance, the Sayana press method. Some people, however, find themselves having prolonged or frequent menstrual cycles. We tell them that if they menstruate for more than 8 days, they should visit the hospital. Some however do not menstruate at all, for some they have irregular (inconsistent) menstrual cycles during the use of this method. For all these cases there is no problem except for those who have prolonged or frequent menstrual cycles.

Interviewer: Assuming I have come to your facility desiring to get self-injection method, can you tell me about the other methods of family planning?

Respondent: Yes. We explain all the methods of family planning. At first, we begin with group counselling where we explain all the methods of family planning in general, we try to find out the information you know about the different family methods, the advantages and disadvantages of the family planning methods, the problems we may encounter when doing the different family planning methods. So depending on the information the women get from the nurses, they make a decision on which family planning to take.

Time stamp: 30:15

Interviewer: Okay. So when counselling the individual you also talk about all the methods?

Respondent: Yes. When counselling individually, we give room to the client to choose which method of family planning which she prefers based on the information previously given during group counselling. For example, a client chooses Sayana method, we explain how the method works, it makes the womb to tot be prepared for pregnancy. Therefore, if the client is getting the family planning method, they are protected from getting pregnant. However, the client is also reminded that the family planning method does not protect her from sexually transmitted diseases such as H.I.V., syphilis or gonorrhea.

Title: ZA_FAC_2.docx

Descriptor Info:	District: Zomba	Target Population: TP1 - Providers/HSAs	Respondent type: Facility-based provider	Aware of SI: Yes - had heard of SI before	Coding phase: 3. Triple coded	Client marital status: N/A - provider	Client age group: N/A - provider	Time since trained: Less than a year ago

Codes Applied:	P3.3 - Counselling/ training approach, general

Interviewer: Which things can you explain when counselling clients?

Respondent: Before or after the person has chosen the family planning method?

Interviewer: I’ve come to you as I said, looking for the self- injection method. What are things you can explain in your counselling?

Respondent: Okay, the counselling we give when a person comes depends on the way the method works, the advantages, the shortfalls that a person may come across while using the method. That is what we tell the person

Title: ZA_FAC_2.docx

Descriptor Info:	District: Zomba	Target Population: TP1 - Providers/HSAs	Respondent type: Facility-based provider	Aware of SI: Yes - had heard of SI before	Coding phase: 3. Triple coded	Client marital status: N/A - provider	Client age group: N/A - provider	Time since trained: Less than a year ago

Codes Applied:	C8.1 - Male involvement, general	P3.3 - Counselling/ training approach, general

Interviewer: Fine, what role do you think the loved ones of the women play in the decision?

Respondent: This requires that the partners of the women should escort them when they come to get antenatal services. Such that when we’re teaching the women, they both hear the message and hence remind each other at home on how to apply the drugs depending on the counsel they have been given at the hospital.

Title: MZs_FAC_2.docx

Descriptor Info:	District: Mzimba South	Target Population: TP1 - Providers/HSAs	Respondent type: Facility-based provider	Aware of SI: Yes - had heard of SI before	Coding phase: 2. Double coded	Client marital status: N/A - provider	Client age group: N/A - provider	Time since trained:

Codes Applied:	P3.3 - Counselling/ training approach, general

Q1. Interviewer: Can you tell me a little about your role in providing family planning at this facility?

Respondent: Family planning in general or just Sayana Press?

Interviewer: Family planning in general.

Respondent: In general okay, we have different methods of family planning. If we have clients who have come for family planning, we gather them together and give them a health talk as a group.

Interviewer: Okay.

Respondent: And after that we give health talk as individual while showing them all the methods of family planning.

Interviewer: Mmmmmmh …… (following)

Respondent: And then the client should choose the method.

Interviewer: Mmmmmmhh…. (following).

Respondent: After the client has choose the method she wants to use, then we give her a health talk about the method she has chosen.

Interviewer: Mmmmmhh …… (following).

Respondent: But sometimes it happens that, we have one or two clients we tell them to wait for others maybe they will come so that we can have a group counseling. But if they are not coming then thus okay, we give them counselling and later on if we have new clients we also counsel them.

Interviewer: Mmmmmh…., (following)

Respondent: Sure.

Title: MZs_FAC_2.docx

Descriptor Info:	District: Mzimba South	Target Population: TP1 - Providers/HSAs	Respondent type: Facility-based provider	Aware of SI: Yes - had heard of SI before	Coding phase: 2. Double coded	Client marital status: N/A - provider	Client age group: N/A - provider	Time since trained:

Codes Applied:	P3.3 - Counselling/ training approach, general

Interviewer: Alright. Are there any methods that you do not usually counsel clients about?

Respondent: Of course we do discourage those that have more children that instead of using Implanon why don’t you just go for kuseka and we are just unikira you the decision to go for it is yours. So we can’t say we discourage them but we unikira them.

Interviewer: Mmmmmmh…. (following)

Respondent: Mmmmmmm

Interviewer: Alright.

Respondent: Sure

Title: MZs_FAC_2.docx

Descriptor Info:	District: Mzimba South	Target Population: TP1 - Providers/HSAs	Respondent type: Facility-based provider	Aware of SI: Yes - had heard of SI before	Coding phase: 2. Double coded	Client marital status: N/A - provider	Client age group: N/A - provider	Time since trained:

Codes Applied:	P3.3 - Counselling/ training approach, general

Q8. Interviewer: If a client has made a decision of considering an injectable contraceptive, how would you greet the client?

Respondent: When a client comes, we great her and tell her you are welcome at this facility feel free.

Interviewer: Mmmmm (following)

Respondent: Then we tell her that I am (Name), working at this facility. How can I help you? Then the client will start explaining the reasons for her visit, if she says, I have come for family planning, then we continue our discussion. Should I continue?

Interviewer: Yes continue.

Respondent: Mmmmmmm… (thinking), if the client say she wants family planning (injectables) then we ask her have you ever used family planning? Then the client may say no or yes but I was using pills, so I want to change. The next step is to find out why she wants to change from pills to injectables, then the client will explain that maybe she was experiencing heavy bleeding.

Interviewer: Mmmmm (following)

Respondent: Mmmmmm… (thinking), then from there if we have more than one client, we do put them together so that we can give them a group counselling.

Interviewer: Okay.

Respondent: Mmmmmmm. (yes)

Interviewer: Then after that?

Respondent: After that, we start the group counselling telling them all the methods that we offer at this facility, and this is done outside. After telling them all the methods that we offer at this facility and then we tell them what is family planning and the advantages of using it. And then the goodness of each method, even though sometimes we skip some of the steps because family planning has a lot of benefits, like benefit to the mother (the one using), to the child, the husband even to the country.

Interviewer: Mmmmmmh… (following).

Respondent: And then we start explaining to them one by one using the flip chart, and we show the method to her and ask her if she knows it and if she says yes then we ask her what type of family planning method is this, then if she says injectables , how do you know that its injectables? Then she might say that she have seen a nurse injecting the woman thus when I will continue with the counselling on how this method works.

Interviewer: Mmmm... (following)

Respondent: And then we continue by telling the client the advantages and the disadvantages about the method

Interviewer: Okay

Respondent: And we do this on all the method

Interviewer: Let’s now talk about a client who has chosen Sayana Press

Respondent: Mmmmmh (okay)

Interviewer: Yes

Respondent: If the client says that she wants Sayana Press then we do explain to her that it is a good method of family planning and if there is 100 people who have used Sayana the possibility that they can get pregnant its only 2

Interviewer: Mmmmmmm (Alright)

Respondent: We also do explain about ovulation on how it works so that someone should not get pregnant.

Interviewer: Mmmmm (alright)

Respondent: Yes. Then we tell them advantages of Sayana compared to Depo that with this one you will be visiting the facility every 3 months while this Sayana will only visit the facility once a year. Or if you choose jakison you will be taking it every day and with these pills you can easily forget. With Sayana method once you have injected or being injected by the provider then thus okay you will just document on the calendar on the next months that you are going to inject.

Interviewer: Mmmmmm (following)

Respondent: Yes, and then…. mmmmm… (thinking)

Interviewer: You can continue

Respondent: Kikikiki… (laughing)… and then we should tell the client the that this Sayana method it only protect pregnancy but not HIV/AIDS, but you want to be protected you need to use condoms every time you are having sex.

Title: MZs_FAC_1.docx

Descriptor Info:	District: Mzimba South	Target Population: TP1 - Providers/HSAs	Respondent type: Facility-based provider	Aware of SI: Yes - had heard of SI before	Coding phase: 3. Triple coded	Client marital status: N/A - provider	Client age group: N/A - provider	Time since trained: A year or more ago

Codes Applied:	P0 - General method mix / method provision	P3.3 - Counselling/ training approach, general

Interviewer: First of all I would like to ask you some questions to understand more about your role in providing family planning services at this facility.

Q1. Interviewer: Can you tell me a little about your role in providing family planning at this facility?

Respondent: Okay, we do provide family planning at this facility on two days and that is Mondays and Tuesdays. Mondays are for short term family methods and Tuesdays are for long term family methods, so in a typical day of family planning clinic we usually welcome mothers who has come for family planning methods. We do counselling and generally all the methods has to be presented to them and at the end we see what they have chosen from the family planning list we have counselled them.

Interviewer: Yes

Respondent: Yah(yes), from there we start giving family planning method individually and also continuation of counselling to the specific family planning they have chosen and after giving the method we give them the next appointment date, so thus how we counsel and give family planning methods is done.

Title: MZs_FAC_1.docx

Descriptor Info:	District: Mzimba South	Target Population: TP1 - Providers/HSAs	Respondent type: Facility-based provider	Aware of SI: Yes - had heard of SI before	Coding phase: 3. Triple coded	Client marital status: N/A - provider	Client age group: N/A - provider	Time since trained: A year or more ago

Codes Applied:	P3.3 - Counselling/ training approach, general

Interviewer: In this section I would like you to talk to through how you counsel a client who is considering an injectable contraceptive. I would like as much as detail as possible, so please break down the steps you would take in counselling this client.

Q8. Interviewer: To begin, how would you greet the client and what would you say?

Respondent: Yah, the first thing you have to be warm. She should feel that she is welcome just by your expression of course the language goes with that, so welcome her and you have also to introduce yourself to her to say that you are the provider and you mention your name and you actually tell her to feel free in that room and that you are going to explain and provide the method. So you have to show that you know the method you are providing and you are on top of things because if she takes that you are also very limited in information and limited in the procedure itself she may think that she is not in the safe hands.

Interviewer: Mmmmm.

Respondent: Yah.

Q9. Interviewer: Alright. Then how would you start the counselling session? After welcoming her, making sure that she is in safe hands and she feels that she is welcomed and you have introduced yourself and then how would you start counselling session?

Respondent: Okay, you could confirm now to say she has really chosen to be given Sayana self- injection course it is when you have given her a general counselling and now she has opted for Sayana injection and now you do one to one counselling and you confirm if she has really chosen the method and if she confirms then you start telling her in details how the method works.

Interviewer: Alright. Would you ask them any questions?

Respondent: Yah, it is possible to ask course you may say that have you ever heard this before and if she says yes then you ask have you ever heard anything concerning this method though they are some misconception sometimes, so its really important to ask her some questions.

Interviewer: Thanks.

Q 10. Interviewer: Assuming the client was only interested in talking up an injectable option, how would you explain their options?

Respondent: Okay, aaaa…once the woman has come and we have introduced to her and she is welcome and we are warm to her now we introduce to her the injectables that are available, we already know that we have Sayana which is self-injection, and we have DEPO which will be given as providers injection, so we actually introduce to the client that we have these two options – DEPO which you will be coming here every three months to get an injection, but apart from that we have self- Injection which we are going to teach you to do it yourself under our supervision cause its your first time and once we are convinced that you have done it rightly then we supply you with the rest of the drugs for a year.

Interviewer: Mmmmmh.

Respondent: And it is an advantage to you that may not be coming frequent to the hospital to get an injection every three months.

Interviewer: Alright. Do you have anything else to say?

Respondent: As I said the stress is to convince the woman that we have this injection that you do by yourself once you have done it rightly we supply you with a one year supply apart from the usual injection which she will be coming every three months which is DEPO.

Title: MZs_FAC_1.docx

Descriptor Info:	District: Mzimba South	Target Population: TP1 - Providers/HSAs	Respondent type: Facility-based provider	Aware of SI: Yes - had heard of SI before	Coding phase: 3. Triple coded	Client marital status: N/A - provider	Client age group: N/A - provider	Time since trained: A year or more ago

Codes Applied:	P3.3 - Counselling/ training approach, general	P3.1.2 - Provider skill/ability to effectively counsel on SI

Interviewer: Do women have concerns about self-injection?

Respondent: Yah there are some questions they could ask, mainly they are some think that how can I do it myself? What about the pain, so they will say may it is painful and they will not manage to complete the drug and how we are going to do it, but once we have taught they are convinced that is not as painful as we thought and also is not as difficult as we thought.

Interviewer: Mmmmh

Respondent: So, before they were many questions that we attend to but after we have taught them I think it answers them that it is just a simple procedure.

Title: MZs_FAC_1.docx

Descriptor Info:	District: Mzimba South	Target Population: TP1 - Providers/HSAs	Respondent type: Facility-based provider	Aware of SI: Yes - had heard of SI before	Coding phase: 3. Triple coded	Client marital status: N/A - provider	Client age group: N/A - provider	Time since trained: A year or more ago

Codes Applied:	P3.3 - Counselling/ training approach, general	P3.1.2 - Provider skill/ability to effectively counsel on SI	C1.1.1 - Positive client attitudes to SI

Q 17. Interviewer: In your view, what are the main factors that would support a client to take up the self-injection option with DMPA-SC?

Respondent: Okay, mainly the counselling itself may influence the client that I think I can take it, the way you have demonstrated to the client it can give confidence to the client that she can actually take it.

Interviewer: Mmmmh

Respondent: And if she has also seen others injecting themselves, I think she can also say that I can also do it.

Title: MZs_FAC_1.docx

Descriptor Info:	District: Mzimba South	Target Population: TP1 - Providers/HSAs	Respondent type: Facility-based provider	Aware of SI: Yes - had heard of SI before	Coding phase: 3. Triple coded	Client marital status: N/A - provider	Client age group: N/A - provider	Time since trained: A year or more ago

Codes Applied:	P3.3 - Counselling/ training approach, general	C3.2.2 - Lack of inclination or confidence to inject, even after training	C1.2.2 - Fear of SI (idea or reality)

Interviewer: In your view what are the main factors that would discourage clients to take up the self-injection option with DMPA-SC?

Respondent: If they haven`t agree with the husband they will find it difficult to say now where am I going to keep this at home, so if they haven`t agreed with the husband then that can be the major problem. It can also be the fear of unknown to say I cannot do it, she has no confident in herself. Also counselling, if you haven`t counselled to her satisfaction or you haven`t demonstrated well then it can also affect that.

Title: KK_FAC_2.docx

Descriptor Info:	District: Nkhotakota	Target Population: TP1 - Providers/HSAs	Respondent type: Facility-based provider	Aware of SI: Yes - had heard of SI before	Coding phase: 1. Single coded	Client marital status: N/A - provider	Client age group: N/A - provider	Time since trained: A year or more ago

Codes Applied:	P3.3 - Counselling/ training approach, general

INTERVIEWER: Okay. We have reached another section. In this section, I would like you to talk me through how you counsel a client who is considering an injectable contraceptive. I want to know so I will ask you a couple of questions.

RESPONDENT: alright.

INTERVIEWER: A client has arrived at the facility; how do you greet the client?

RESPONDENT: Firstly, we greet the client. After the greeting, we explain to her about yourself who is providing the contraception methods. After that, you ask her name, and she will tell you. And then we ask her the service she wants from us.

INTERVIEWER: mmmmhu.

RESPONDENT: yeah.

INTERVIEWER: Alright. How do you start counselling? You have welcomed her, greeted her etc., right? How do you begin counselling?

RESPONDENT: We begin from the problem she has come for or from a service that she wants. Then we explain to her that we will discuss with her about family planning. But we ask her if she has ever used contraception methods.

INTERVIEWER: okay.

RESPONDENT: Mmmhu. Or if she has never used a contraception method. So when we ask that, it gives us a picture on where to start from.

INTERVIEWER: mmmmhu.

RESPONDENT: yea.

INTERVIEWER: You have told me about two questions, right? What other questions do you ask apart from these ones?

RESPONDENT: I first explained about the type of service she wants right?

INTERVIEWER: mmmmh.

RESPONDENT: yeah, the type of contraception method that they want right?

INTERVIEWER: mmmmh.

RESPONDENT: So when they explain about that, we ask her if she has ever heard about contraception.

INTERVIEWER: okay.

RESPONDENT: mmmmh.

INTERVIEWER: alright.

RESPONDENT: And where she heard from where form friends or where ever.

INTERVIEWER: Okay, alright. Let us assume that a client was only interested in taking up an injectable option, how would you explain the options she has on the injectable? She wants an injectable right? How will you explain the choices she has on the injectable?

RESPONDENT: Okay. On the injectable, we first explain to her the types of the injectable that there is Depo injected by the provider and there is Sayana foe self-injection.

INTERVIEWER: mmmmhu.

RESPONDENT: Yeah. So we will explain to her the pros and cons of the method, or I should just say how it works. How the injection works and also how she can self-inject and the problems she might face when using this method.

INTERVIEWER: mmmmhu.

RESPONDENT: mmmmh.

INTERVIEWER: Between Depo and Sayana, which method do you first explain? Which method do you first explain between Depo and Sayana? Which method do you first explain?

RESPONDENT: It depends with the type of method she has chosen. Because when a person has chosen Depo, you cannot explain about Sayana, no.

INTERVIEWER: okay.

RESPONDENT: Yes. She has chosen this method. She has received all the counselling and she is saying that I want Sayana, we go straight to the method she wants.

INTERVIEWER: mmmmhu.

RESPONDENT: yea.

INTERVIEWER: Alright. What about when you are explaining that there is this, this and that method. Between Depo and Sayana, which method do you mention first?

RESPONDENT: we first mention Sayana.

INTERVIEWER: you start with Sayana?

RESPONDENT: mmmmh.

INTERVIEWER: before you explain Depo?

RESPONDENT: Mmmhu. So that we should promote this method.

INTERVIEWER: Mmmhu. So when conducting counselling that there is Depo and Sayana injections, right, do you any materials/job aids?

RESPONDENT: yes.

INTERVIEWER: like what?

RESPONDENT: The actual drugs, aaa…what can I say, needle (giggle), needle. syringe

INTERVIEWER: syringe (laughs).

RESPONDENT: syringe. When we say syringe, we are talking about Depo.

INTERVIEWER: aha.

RESPONDENT: Syringe and drugs, we are talking about Sayana.

INTERVIEWER: okay.

RESPONDENT: aaa there are also flip charts that we use.

INTERVIEWER: mmmmhu.

RESPONDENT: yeah.

Title: KK_FAC_2.docx

Descriptor Info:	District: Nkhotakota	Target Population: TP1 - Providers/HSAs	Respondent type: Facility-based provider	Aware of SI: Yes - had heard of SI before	Coding phase: 1. Single coded	Client marital status: N/A - provider	Client age group: N/A - provider	Time since trained: A year or more ago

Codes Applied:	P3.3 - Counselling/ training approach, general	P3.1.5 - Ease of training clients in SI	P3.1.3 - IEC/demo materials for counselling/training

INTERVIEWER: Alright. How difficult or easy is it to train a woman to self-inject? How difficult or easy is it to train a woman about Sayana?

RESPONDENT: Teaching a woman about Sayana is not difficult according to me.

INTERVIEWER: How about self-injecting, the process of training them to self-inject is it easy or difficult?

RESPONDENT: The process is not difficult to teach them. But the practical part of self-injecting is up to that person. That is where we keep on repeating until she gets it. Yeah.

INTERVIEWER: Do you use any resources when teaching them self-injection?

RESPONDENT: We use flexa foams

INTERVIEWER: okay.

RESPONDENT: mmmh.

INTERVIEWER: What happens with these flexa foams?

RESPONDENT: flexa foam?

INTERVIEWER: mmmh.

RESPONDENT: We take the Sayana syringe and then we explain to them that you should hold the syringe like this on the neck. Don’t hold on the bulb because it has a bulb. They should not hold that part because if they do, Sayana will spill.

INTERVIEWER: mmmhu.

RESPONDENT: Yeah. And we tell them to put the syringe at an angle they can see. They are supposed to inject above the belly button or on a left or right thigh.

INTERVIEWER: mmmhu.

RESPONDENT: Yeah. But if they are being injected by the provider, it’s on the arm.

INTERVIEWER: Okay. Do you use condoms filled with sugar when training the women?

RESPONDENT: We don’t use condoms filled with sugar. We just use them as a demonstration.

INTERVIEWER: Okay. What do you put inside the condom?

RESPONDENT: condom?

INTERVIEWER: Yes…no…I mean…when others are training clients, they fill a condom with sugar for the women to try as if they are injecting the Sayana. Do you use the condom?

RESPONDENT: Like a condom?

INTERVIEWER: mmmmh.

RESPONDENT: No we don’t use a condom.

INTERVIEWER: You don’t?

RESPONDENT: mmm mmm [no].

INTERVIEWER: But you say you demonstrate with a flexa foam?

RESPONDENT: mmmh a flexa foam.

INTERVIEWER: The flexa foam in this case is a thigh?

RESPONDENT: Yes, a small one.

INTERVIEWER: okay.

RESPONDENT: mmmh.

Title: KK_FAC_2.docx

Descriptor Info:	District: Nkhotakota	Target Population: TP1 - Providers/HSAs	Respondent type: Facility-based provider	Aware of SI: Yes - had heard of SI before	Coding phase: 1. Single coded	Client marital status: N/A - provider	Client age group: N/A - provider	Time since trained: A year or more ago

Codes Applied:	P3.3 - Counselling/ training approach, general	P3.1.4 - Provider sufficient support to counsel/train on SI

INTERVIEWER: mmmhu. Do you feel you have enough time to conduct these trainings?

RESPONDENT: I feel that it’s enough.

INTERVIEWER: it’s enough?

RESPONDENT: mmmmmh.

INTERVIEWER: Do you conduct these trainings in a group or one to one?

RESPONDENT: Firstly, there is group counselling and from the group counselling, we go to individual counselling to a person who has chosen…

INTERVIEWER: mmmhu.

RESPONDENT: Explaining to her about the method.

INTERVIEWER: okay.

RESPONDENT: mmmhu.

INTERVIEWER: What about on busy days, does it still take 15 minutes to conduct the training on self-injection? Is it still the same 15 minutes?

RESPONDENT: yes, we divide the work.

INTERVIEWER: okay.

RESPONDENT: mmmmh.

INTERVIEWER: How do you divide the work?

RESPONDENT: Others come because of emergencies maybe they are sick. We first assist those ones so that they should go and for us to have a good number.

INTERVIEWER: okay.

RESPONDENT: mmmhu.

Title: KK_FAC_2.docx

Descriptor Info:	District: Nkhotakota	Target Population: TP1 - Providers/HSAs	Respondent type: Facility-based provider	Aware of SI: Yes - had heard of SI before	Coding phase: 1. Single coded	Client marital status: N/A - provider	Client age group: N/A - provider	Time since trained: A year or more ago

Codes Applied:	P3.3 - Counselling/ training approach, general	C6 - Waste management

INTERVIEWER: Mmmmhu. Do women feel comfortable taking these things home?

RESPONDENT: They feel comfortable because we we explain to them on how they can dispose used syringes. We tell them to find a bottle of frozzy or any other bottle and dispose in there. They should also keep away from children because they might get hurt. On the next appointment they have to bring that…they bring that and we burn it.

INTERVIEWER: mmmhu.

RESPONDENT: mmmh.

Title: KK_FAC_2.docx

Descriptor Info:	District: Nkhotakota	Target Population: TP1 - Providers/HSAs	Respondent type: Facility-based provider	Aware of SI: Yes - had heard of SI before	Coding phase: 1. Single coded	Client marital status: N/A - provider	Client age group: N/A - provider	Time since trained: A year or more ago

Codes Applied:	P3.3 - Counselling/ training approach, general	P3.1 - Enablers, general/other

INTERVIEWER: In your view, what are the factors that would support a client to take up the self-injection option? What can encourage them to take up Sayana?

RESPONDENT: The first thing that can…I will say reasons because it is not just one.

INTERVIEWER: … Yes, even if its five reasons.

RESPONDENT: What would support them to take up Sayana is firstly when they have been explained to about the problems they will face when they take up Sayana. This is because what makes clients not to choose a method are side effects or problems that arise because of contraception methods. If she is not well explained to about the side effects, she just mentions anything for example my head was itching it is because of Sayana. This was happening on my body, and it is because of Sayana. The second factor that can encourage them is that there should be continuous supply of Sayana. Not that you have supply for two months and then the next month there is stock out. Because of this, instead for clients to come and ask for this method, they just say that they are always in stock out and people forget about the method.

INTERVIEWER: mmmmhu.

RESPONDENT: yeah.
