# Supplementary material for: Health care provider and client experiences of counselling on depot medroxyprogesterone acetate subcutaneous (DMPA-SC) for self-injection in Malawi
Source: PLOS Glob Public Health. 2023 Nov 30;3(11):e0002057. doi: 10.1371/journal.pgph.0002057 (PMC10688740; doi:10.1371/journal.pgph.0002057)
Supplement: S1 Data — (ZIP) [file pgph.0002057.s001.zip › S2/P3.3 couns approach HSA only.doc]

Title: ZA_HSA_2.docx

Descriptor Info:	District: Zomba	Target Population: TP1 - Providers/HSAs	Respondent type: HSA	Aware of SI: Yes - had heard of SI before	Coding phase: 3. Triple coded	Client marital status: N/A - provider	Client age group: N/A - provider	Time since trained: Less than a year ago

Codes Applied:	P3.3 - Counselling/ training approach, general

Interviewer: Sure, can you tell me a little bit about your role in providing family planning services at this hospital and in this area?

Respondent: Okay, first of all we are supposed to give advice to the people so that they know what family planning is.

Interviewer: mmmm (Showing that one is following the conversation)

Respondent: What are the benefits of family planning, yes, so when we give them the advice about the family planning methods and they approve, for example Sayana press, we are able to inject them right there if they feel like they cannot be able to inject themselves, eh, for those that have accepted that they can inject themselves we encourage them so that they can be injecting themselves at their homes.

Interviewer: Alright

Respondent: But if they seem not to understand something, we allow them to, we encourage them to ask questions so that they fully understand the things that we are discussing.

Title: ZA_HSA_2.docx

Descriptor Info:	District: Zomba	Target Population: TP1 - Providers/HSAs	Respondent type: HSA	Aware of SI: Yes - had heard of SI before	Coding phase: 3. Triple coded	Client marital status: N/A - provider	Client age group: N/A - provider	Time since trained: Less than a year ago

Codes Applied:	P3.3 - Counselling/ training approach, general

Respondent: Most of the times when we see that we have given them our counselling…

Interviewer: mmm (Showing that one is following the conversation)

Respondent: When we ask them that, the way that we have trained you and taught you about these family planning methods, which one have you chosen or has appealed the most to you…

Interviewer: Mmmm (Showing agreement)

Respondent: Then most of them chose DMPA-IM or DMPA-SC (Sayana press) when these methods are available.

Interviewer: Okay.

Respondent: Yes, so that we can administer the DMPA-IM to them, if we do that, we will also tell them that after three months they should come back since they cannot be able to perform self-injection with DMPA-IM.

Interviewer: Eh

Respondent: But when its Sayana some are able to agree that with the way that you have injected me I can also be able to inject myself at home.

Title: ZA_HSA_2.docx

Descriptor Info:	District: Zomba	Target Population: TP1 - Providers/HSAs	Respondent type: HSA	Aware of SI: Yes - had heard of SI before	Coding phase: 3. Triple coded	Client marital status: N/A - provider	Client age group: N/A - provider	Time since trained: Less than a year ago

Codes Applied:	P3.3 - Counselling/ training approach, general	P0 - General method mix / method provision

Interviewer: Are there any other family planning methods that you don’t like to encourage women to use?

Respondent: Yes, the natural ones, eh these are mostly encouraged by church members (religious people)

Interviewer: mmm (Showing that one is following the conversation)

Respondent: But for us when we saw those methods, most people have used these methods, but they have failed, they found that they could not follow the methods properly and as a result they got pregnant, and some have kids.

Interviewer: Alright.

Respondent: So, for these methods we see that in the modern day…

Interviewer: mmmm (Showing that someone is following the conversation)

Respondent: Its different from the old days, people were able to use the traditional methods, but currently in this modern day we are not encouraging the use of those methods.

Title: ZA_HSA_2.docx

Descriptor Info:	District: Zomba	Target Population: TP1 - Providers/HSAs	Respondent type: HSA	Aware of SI: Yes - had heard of SI before	Coding phase: 3. Triple coded	Client marital status: N/A - provider	Client age group: N/A - provider	Time since trained: Less than a year ago

Codes Applied:	P2.3 - Provider training content	P3.1.5 - Ease of training clients in SI	P3.3 - Counselling/ training approach, general

Interviewer: The time that you received this training, ah I wanted you to tell me a little bit about the training that you received…

Respondent: mmmm (showing that he is following)

Interviewer: …Relating to providing this method of family planning which women are able to self-inject.

Respondent: Alright, eh ah, most of it was about how we can train a woman on how they can be able to self-inject.

Interviewer: mmm (Showing that one is following the conversation)

Respondent: For most of the women didn’t go to school, but we are able to communicate with them and teach them that this is how we handle the injection (Explains while demonstrating with gestures), there is this little pot, so when we want to self-inject, we pinch the skin a little bit, inject, and then when we inject ah, we stop pinching the skin and then squeeze the little pot where the liquid is…

Interviewer: mmm (Showing that one is following the conversation)

Respondent: Yes, so that the injection gets to all the right places.

Title: ZA_HSA_2.docx

Descriptor Info:	District: Zomba	Target Population: TP1 - Providers/HSAs	Respondent type: HSA	Aware of SI: Yes - had heard of SI before	Coding phase: 3. Triple coded	Client marital status: N/A - provider	Client age group: N/A - provider	Time since trained: Less than a year ago

Codes Applied:	P3.2.2 - Stockouts/stocks near expiry dates	P3.3 - Counselling/ training approach, general

Interviewer: Have you faced any difficulties in providing DMPA-SC?

Respondent: No, we have not faced any difficulties.

Interviewer: Alright.

Respondent: Mostly it’s because the commodity has not been available

Interviewer: mmm (Showing agreement)

Respondent: It hasn’t been available, the clients that were coming were being given DMPA_IM.

Interviewer: Alright.

Respondent: Yes, no difficulties have been faced.

Title: ZA_HSA_2.docx

Descriptor Info:	District: Zomba	Target Population: TP1 - Providers/HSAs	Respondent type: HSA	Aware of SI: Yes - had heard of SI before	Coding phase: 3. Triple coded	Client marital status: N/A - provider	Client age group: N/A - provider	Time since trained: Less than a year ago

Codes Applied:	P3.3 - Counselling/ training approach, general

Interviewer: Thank you, ah now I would like you to talk to me through how you counsel a client who is considering an injectable contraceptive. I would like as much detail as possible, so please break down the steps you would take in counselling this client.

Respondent: mmhh (Showing that he is following the conversation)

Interviewer: To begin, how do you greet the client? How do you welcome them when they get to the facility or when they find you at home in the community?

Respondent: Alright, ah we greet them.

Interviewer: mmmhh (Showing agreement)

Respondent: and welcome them, maybe we ask them “how is home?”

Interviewer: mmm (Showing agreement)

Respondent: Yes, how can I help you?

Interviewer: mm (Showing agreement)

Respondent: Yes, how should I help you?

Interviewer: mmmm (Showing Agreement)

Respondent: Yes, so they answer that I am here to seek family planning services.

Interviewer: mmm (Showing agreement)

Respondent: So, when the client is a frequent user of family planning, that they already know about family planning…

Interviewer: mmmmhh (Showing agreement)

Respondent: Ah, we just encourage the client, but if the client is a new user of family planning, we give the counselling…

Interviewer: mmmm (Showing that one is following the conversation)

Respondent: We counsel the client everything about family planning and the methods.

Interviewer: mmhh (Showing that one is following the conversation)

Respondent: yes, even though we don’t provide Norplant.

Interviewer: mmm (Showing that one is following the conversation)

Respondent: But still, we counsel them on that highlighting to the client about the advantages of the methods…

Interviewer: Mmmm (showing that one is following the conversation)

Respondent: If they have chosen that ah for me, I feel that I should chose Norplant…

Interviewer: mmm (Showing that one is following the conversation)

Respondent: They have all the freedom, and we give them what they want, if they want injectables then we tell them that right now we have DMPA-IM injectable and we explain how DMPA-IM works, we tell them that we will inject you and it will be effective for three months, and the advantage is that you cannot get pregnant, yeah

Interviewer: mmm (Agreeing)

Respondent: Yes, it’s only a few women that can get pregnant while they are using family planning methods.

Interviewer: mmm (Showing agreement)

Respondent: Yes, so, this one seems like is the method that most of the clients don’t know it, yeah…

Interviewer: mmm (showing that one is following the conversation)

Respondent: It’s not hard for the clients to understand this method.

Interviewer: Alright.

Respondent: And when we come to DMPA-SC we explain to them that Sayana is the same as DMPA-IM

Interviewer: Yes

Respondent: Yes, the effectiveness is the same and there is no any difference.

Interviewer: mmm (Showing that one is following the conversation)

Respondent: Yes, the days of effectiveness for DMPA-SC are the same as those for DMPA-IM, the only difference is that for DMPA-SC you have to self-inject…

Interviewer: mmm (Showing agreement)

Respondent: …at home, here at the facility we will inject you tight now, then we train them how they can self-inject the way that I have already explained that at first, they should check the expiry date of the commodity…

Interviewer: mmmhh (Showing agreement)

Respondent: yes, when they check the expiry date if it is not yet expired, we tell them to tear the package…

Interviewer: mmm (showing that one is following the conversation)

Respondent: When they tear the package, we train them first then we inject them right there, our commodity is administered through the arm…

Interviewer: eh eh (Showing agreement)

Respondent: Yes, so we tell them that the areas to inject themselves are on the tummy around the navel and on the thigh…

Interviewer: mmm (Showing agreement)

Respondent: Yes, then when they want to inject themselves, they hold the., they open, yeah…

Interviewer: mmm (Agreeing)

Respondent: then they hold the small pot containing the medicine, at the bottom there is a small pot that is connected to the needle, yeah …

Interviewer: mmm (Showing that one is following the conversation)

Respondent: Yes, then they, for them to open the bottle they are supposed to hold the cover and press it down, then the bottle opens…

Interviewer: Mmmm (Showing that one is following the conversation)

Respondent: Then they remove the cover, yeah…

Interviewer: mmmm (showing agreement)

Respondent: And do that…

Interviewer: mmm (Showing that one is following the conversation)

Respondent: Then they hold and pinch the skin either on the thigh or on the tummy surrounding the navel then they inject the Sayana, when they inject, they will squeeze the small pot, then they will .., let me just say before they squeeze the small pot they stop the pinching of the skin, yeah…

Interviewer: mmm (showing that one is following the conversation)

Respondent: When they stop pinching, and release the skin so that the medicine from the injection should circulate properly…

Interviewer: mmm (showing that one is following the conversation)

Respondent: When they squeeze the small pot, they will remove the needle and they will dispose it off…

Title: ZA_HSA_2.docx

Descriptor Info:	District: Zomba	Target Population: TP1 - Providers/HSAs	Respondent type: HSA	Aware of SI: Yes - had heard of SI before	Coding phase: 3. Triple coded	Client marital status: N/A - provider	Client age group: N/A - provider	Time since trained: Less than a year ago

Codes Applied:	P3.3 - Counselling/ training approach, general

Respondent: Then the client if they have understood we allow them to ask questions, if they have understood, if there is something that they didn’t understand well we let them ask us questions…

Interviewer: Alright.

Respondent: when it’s the case that they have understood…

Interviewer: mmm (Showing that one is following the conversation)

Respondent: oh alright, we inject them. When we inject them, for Sayana we ask them for the second time, so we were saying that this one you will self-inject at home…

Interviewer: mmm (showing that one is following the conversation)

Respondent: can you tell me how you will be injecting yourself? So, the client is able to explain the way that we have explained to them, so from that we are able to know that this client will…

Interviewer: self-inject…

Respondent: be able to self-inject…

Respondent: When Sayana is available in right quantities we even take something, whatever, that we can demonstrate the way that the client can self-inject, yeah…

Interviewer: mmm (showing agreement)

Respondent: we can use tomato, or we can collect sand and fill it is a paper so that we demonstrate how we can self-inject, the degrees of injecting on the skin are 45, 90 something between that, but the needle is supposed to be in that position (Demonstrating on the arm with fingers the angle that the needle has to be) …

Interviewer: mmm (showing that one is following the conversation)

Respondent: Yes…

Interviewer: Alright.

Respondent: so, when the client seems to have explained everything properly, we are satisfied that this client has…

Interviewer: Understood.

Respondent: Understood, but also if the client goes home and its almost time to re-inject …

Interviewer: mmm (showing that one is following the conversation)

Respondent: the third month

Interviewer: mmm (showing that one is following the conversation)

Respondent: But the client feels like they are not sure, we allow them to come back to us so that we explain to them again…

Interviewer: mm (showing that one is following the conversation)

Respondent: we inject them again if the client does not bring the…

Interviewer: uniject.

Respondent: Yes, but if they have understood they can say that I just forgot a little here where you have now explained, now its okay am going back home I will e able to inject myself, yeah…

Title: ZA_HSA_2.docx

Descriptor Info:	District: Zomba	Target Population: TP1 - Providers/HSAs	Respondent type: HSA	Aware of SI: Yes - had heard of SI before	Coding phase: 3. Triple coded	Client marital status: N/A - provider	Client age group: N/A - provider	Time since trained: Less than a year ago

Codes Applied:	P3.3 - Counselling/ training approach, general	P3.1.2 - Provider skill/ability to effectively counsel on SI

Interviewer: Alright, let’s assume that a woman has an interest in using the injectable contraceptive…

Respondent: mmm (showing that they are following the conversation)

Interviewer: How would you explain their options?

Respondent: Okay, we explain to them according to…, have you said only injectable contraceptive, right?

Interviewer: Yes.

Respondent: okay, aahh, we explain to them how the injectable works, yes, the other one when it’s the self-injecting one it’s the same as the other one there is no difference….

Interviewer: eh

Respondent: but maybe we just explain to them how the injectable can function in women’s bodies….

Interviewer: mmm (showing that one is following that conversation)

Respondent: its different from one client to the next.

Interviewer: Alright.

Respondent: some of the clients take the injection, and they don’t experience any menstrual cycles, for those clients we tell them that everything for them is perfect, there is no any problem.

Interviewer: Alright.

Respondent: some of the clients take the injection and they experience erratic bleeding during menstruation cycles.

Interviewer: mmm (showing that one is following the conversation)

Respondent: Yes, so we tell them that there is a problem…

Interviewer: mmm (showing that one is following the conversation)

Respondent: So, they need to come to the hospital to explain to the health worker.

Interviewer: Alright.

Respondent: Yes, so that the doctor can help them.

Interviewer: mmm (showing that one is following the conversation)

Respondent: some of them experience rapid bleeding during menstrual cycles.

Interviewer: mmm (showing that one is following the conversation)

Respondent: The same for these ones they can, they can come t the facility and seek medical attention, some of the clients who use this method experience minor issues for example, stomach-aches nearing the time that they will experience their menstrual periods, for these clients we tell them that it happens.

Interviewer: mmm (showing that one is following the conversation)

Respondent: yes, it’s the possibility that maybe this time something was about to happen, but it has not happened. …

Title: ZA_HSA_2.docx

Descriptor Info:	District: Zomba	Target Population: TP1 - Providers/HSAs	Respondent type: HSA	Aware of SI: Yes - had heard of SI before	Coding phase: 3. Triple coded	Client marital status: N/A - provider	Client age group: N/A - provider	Time since trained: Less than a year ago

Codes Applied:	P3.3 - Counselling/ training approach, general	C0.2 - Community and client perspectives on FP - general	P3.1.2 - Provider skill/ability to effectively counsel on SI

Respondent: so, most of these clients are living in the remote areas where there are people with different beliefs, some of them can advise their friends good things, and some of them can mislead their friends. Some of them say that when you inject yourself with this injection you’re no longer going to conceive. When these women get to us, we explain to them properly, we tell them that its all safe and there is not any problem with the Depo injection and Sayana press, the only difference is the way that they are packaged, yeah…

Interviewer: mmm (showing that one is following the conversation)

Respondent: Yes, but all of these are the same.

Interviewer: Alright.

Respondent: Yes so, we tell them that when they have questions or any problem, they should be coming to us before they believe what they hear from the people in their communities, we can explain to them properly…

Title: ZA_HSA_2.docx

Descriptor Info:	District: Zomba	Target Population: TP1 - Providers/HSAs	Respondent type: HSA	Aware of SI: Yes - had heard of SI before	Coding phase: 3. Triple coded	Client marital status: N/A - provider	Client age group: N/A - provider	Time since trained: Less than a year ago

Codes Applied:	C1.1.3 - Awareness of DMPA-SC SI	P3.3 - Counselling/ training approach, general

Respondent: Yes, when they hear that one of their friends, I do self-inject, they ask how do you do it? So, the friends explain to them that this is how we do it, when they explain you will hear them saying that I also need to use DMPA-SC so that I should what…

Interviewer: I should self-inject.

Respondent: I should self-inject, yes because some things happen in groups, yeah…

Interviewer: mmm (showing that one is following the conversation)

Respondent: Yes, so in groups most people learn faster than when they are being trained individually because in groups, they are able to see their friends, th

Title: ZA_HSA_2.docx

Descriptor Info:	District: Zomba	Target Population: TP1 - Providers/HSAs	Respondent type: HSA	Aware of SI: Yes - had heard of SI before	Coding phase: 3. Triple coded	Client marital status: N/A - provider	Client age group: N/A - provider	Time since trained: Less than a year ago

Codes Applied:	C8.1 - Male involvement, general	P3.3 - Counselling/ training approach, general

Interviewer: Ah, some women…

Respondent: eh

Interviewer: Use contraception covertly,

Respondent: eh

Interviewer: Do you think this influences their decision that they should use self-injection?

Respondent: eh (PAUSE) especially for those men that deny their spouses…

Interviewer: mmm (showing that one is following the conversation)

Respondent: That they should use family planning.

Interviewer: mmm (showing that one is following the conversation)

Respondent: When they take the doses, the woman is able to self-inject without the husband knowing…

Interviewer: Without them knowing.

Respondent: without them knowing…

Interviewer: mmm (showing that one is following the conversation)

Respondent: There is this scenario that happened somewhere yeah …

Interviewer: mmm (showing that one is following the conversation)

Respondent: A woman came for family planning; the husband was just following her without knowing what services the woman wanted to access…

Interviewer: Family planning.

Respondent: When the man found out, he just came and grabbed the woman’s health passport and teared it…

Interviewer: mmm (showing that one is following the conversation)

Respondent: The woman’s book.

Interviewer: mmm (showing that one is following the conversation)

Respondent: He also destroyed the uniject

Interviewer: mmm (showing that one is following the conversation)

Respondent: Yeah so, I sat the man down for some time and discussed with him, the good thing was that I chat with him…

Interviewer: mmm (showing that one is following the conversation)

Respondent: I asked him, why have you done this? He says that, ah I don’t want my wife to be on family planning, but the wife had already bore five kids…

Interviewer: mmm (showing that one is following the conversation)

Respondent: And he wants another child.

Interviewer: mmm (showing that one is following the conversation)

Respondent: So, I sat down with him and discussed the benefits of having children that your family can be able to take care of yeah…

Interviewer: mmm (showing that one is following the conversation)

Respondent: Yeah, so for now we say that the limit is four children.

Interviewer: mmm (showing that one is following the conversation)

Respondent: Yeah, but six going up no, so the man understood…

Interviewer: mmm (showing that one is following the conversation)

Respondent: so, the way it is now you can administer the injection on her…

Interviewer: mmmm (showing that one is following the conversation)

Respondent: (laughs) eh There was a problem…

Interviewer: eh

Respondent: But he understood because I explained to him, yeah, but for some men they are just very rigid…

Interviewer: mmm (showing that one is following the conversation)

Respondent: This is a good method that can help a lot of women.

Interviewer: mmmhh (showing that one is following the conversation)

Respondent: Yeah, because the problems of motherhood are mostly experienced by women different from us men…

Interviewer: yah yah (Agreeing)

Respondent: Because for us we just impregnate them and tell them to go…

Interviewer: mmm (showing that one is following the conversation)

Respondent: We just come back in the morning when the baby is already born (laughs)

Title: ZA_HSA_2.docx

Descriptor Info:	District: Zomba	Target Population: TP1 - Providers/HSAs	Respondent type: HSA	Aware of SI: Yes - had heard of SI before	Coding phase: 3. Triple coded	Client marital status: N/A - provider	Client age group: N/A - provider	Time since trained: Less than a year ago

Codes Applied:	C3.2.2 - Lack of inclination or confidence to inject, even after training	C5.2.2 - Clients incorrectly / failing to administer at home	P3.3 - Counselling/ training approach, general	P4.1.3 - Provider availability/ willingness to repeat SI training

Interviewer: Alright, are the women that you serve uncomfortable or not confident to inject themselves?

Respondent: Some are uncomfortable, but only a few.

Interviewer: mmmm (showing that one is following the conversation)

Respondent: Those ones are the ones who when its time we are giving counselling about self-injection…

Interviewer: mmm (showing that one is following the conversation)

Respondent: Some of them are those that have completely failed to self-inject…

Interviewer: mmm (showing that one is following the conversation)

Respondent: They say that, ah I cannot manage…

Interviewer: I can not manage…

Respondent: I cannot manage, eh, those that cannot manage we just try to guide them and another time when they come back but we don’t force them that they should self-inject because if we do that, they might end up taking the doses and not use them…

Interviewer: mmmm (showing agreement)

Respondent: Yea, without using them, or they might just switch to another facility and get the provider administered injection there and they won’t use the doses that we have given them at this facility.

Interviewer: mmmm (showing that one is following the conversation)

Respondent: Yeah, and another client may end up lacking the doses while the other client is just keeping the doses without using them…

Interviewer: mmm (showing that one is following the conversation)

Respondent: So, we want that at first the client has been guided, and when they understand properly, they select the method, but if they don’t understand then we tell them that you can be coming so that we can be injecting you, even if its Sayana, yeah…

Interviewer: mmm (showing that one is following the conversation)

Respondent: Unless if they accept that they have understood…

Interviewer: mmmh (showing that ne is following the conversation)

Respondent: This method, the way that you can self-inject…

Title: KK_HSA_1.docx

Descriptor Info:	District: Nkhotakota	Target Population: TP1 - Providers/HSAs	Respondent type: HSA	Aware of SI: Yes - had heard of SI before	Coding phase: 2. Double coded	Client marital status: N/A - provider	Client age group: N/A - provider	Time since trained: A year or more ago

Codes Applied:	P3.3 - Counselling/ training approach, general

Interviewer: Okay. Are there any methods that you do not encourage clients about?

Respondent: no

Interviewer: So that women should use those methods?

Respondent: We encourage all methods.

Interviewer: okay

Respondent: Yes.

Title: KK_HSA_1.docx

Descriptor Info:	District: Nkhotakota	Target Population: TP1 - Providers/HSAs	Respondent type: HSA	Aware of SI: Yes - had heard of SI before	Coding phase: 2. Double coded	Client marital status: N/A - provider	Client age group: N/A - provider	Time since trained: A year or more ago

Codes Applied:	P3.3 - Counselling/ training approach, general

Interviewer: What was the uptake?

Respondent: No people do…when you train them…..because when you counsel the woman, it requires that you have given her the first dose right? Inject your self. We have trained them, their friends will be like role models they will self inject. And then them, I will be able to do it what what. And they will come and you will see that they have self injected she...she…she has done well in all the procedures. So you give her 3 or four doses. Mmhu, but when she is afraid, you just give her one dose so that next time you should also watch her. At her next visit you also counsel her so that she should be fully she should not be afraid. So those who came the previous month they take more doses because they are now well trained. The women also tell each other that vaccine…the Sayana injection is not difficult and not painful. I have self injected and it is easy when they train you. So when these people are informing each other, lets say 15 women have come, you should give each one of them 3 doses, at least the what…

Interviewer: It’s increasing?

Respondent: Yes they are taking more doses.

Interviewer: Okay.

Respondent: Yes.

Interviewer: So roughly right? What proportion of Sayana…Sayana press is for self-injection?

Respondent: We give a woman four doses for a year.

Interviewer: 4?

Title: KK_HSA_1.docx

Descriptor Info:	District: Nkhotakota	Target Population: TP1 - Providers/HSAs	Respondent type: HSA	Aware of SI: Yes - had heard of SI before	Coding phase: 2. Double coded	Client marital status: N/A - provider	Client age group: N/A - provider	Time since trained: A year or more ago

Codes Applied:	P3.3 - Counselling/ training approach, general

Interviewer: Mmmhu we are proceeding. In this section I want you to explain how you…how you counsel a client who is considering an injectable contraceptive right about how you do the counselling. You said counselling…you give yes. So I want to know how you do it. I will ask you questions so that you should explain to me.

Respondent: alright

Interviewer: Mmmhu. Firstly, a woman has arrived, how do you welcome them?

Respondent: When a woman arrives, we welcome her…we greet her. After greeting her then we tell her that I am an HSA and my name is this and I stay at that place. And the woman should also be free, I am Mrs. Ngana. They have said it right?

Interviewer: mmmh.

Respondent: How can I help you? I have come because I want a contraceptive method. What method do you want? I want an injectable, Sayana. So before we give her a method, we explain to her all the methods so that it should be to her to choose what she has really liked. Because some come with an already made decision from home that I want this method because she just heard when people were chatting, so I will just chose the same Sayana.

Interviewer: Mmmhu. So…how do you start the counselling?

Respondent: the first one is, one on one

Interviewer: mmmh

Respondent: Like maybe a one woman has come. And there is another one you do group counselling. Yes, so when she has come alone, the woman you...you greet her and you ask why she has visited and when she tells you you begin…if she wants the contraceptives, you explain all the methods. Because she came with a mind that I want an injectable, you explain the others as one way of selling the methods right, everything. The good and bad and they function. And after that the woman says I want Sayana and there you explain to her in detail about the method and the you give her.

Interviewer: okay.

Respondent: Yes.

Interviewer: So you have told me that you ask questions right?

Respondent: mmmhu.

Interviewer: What questions do you ask?

Respondent: We have questions we use…I’ve just forgotten a certain guide. Yes, like how we do with DMPA. Have you ever suffered from headache? Have you ever fainted? Different questions. So we also use that as a checklist before we give her…before her choice of Sayana.

Title: KK_HSA_1.docx

Descriptor Info:	District: Nkhotakota	Target Population: TP1 - Providers/HSAs	Respondent type: HSA	Aware of SI: Yes - had heard of SI before	Coding phase: 2. Double coded	Client marital status: N/A - provider	Client age group: N/A - provider	Time since trained: A year or more ago

Codes Applied:	P3.3 - Counselling/ training approach, general

Interviewer: So…let us imagine that a woman has come right?

Respondent: mmmh

Interviewer: She wants family planning. She has shown interest that she only wants an injectable right? How…how…how do you explain to her the different options that are available? She has come and she has told you that she wants family planning and she has told you that she wants an injectable right?

Respondent: mmmh.

Interviewer: How do you explain to have the different options that are available?

Respondent: Yes, that where I said that the woman has already come with a mind that I am going for an injection yes, I want Sayana. Right? So when you explain all the methods, when you explain to the woman. There is loop, there is a method of pills, there is Norplant, there is an injectable, there are pills etc., you explain. So on every method you tell her the good and bad side. So when you sell the methods to the woman, she will weigh in her mind, and that is when she makes a choice. She came with a decision that she wants Sayana right?

Interviewer: mmmh.

Respondent: But because she has changed her mind with the way you explained or maybe she has thought that maybe this method I was not sure, what if I choose loop for 3 years.

Interviewer: mmmh

Respondent: Yes.

Interviewer: Okay. So this person right?

Respondent: mmmh

Interviewer: She has come aaah, I want an injectable right? How are you going to explain to her the options she has on these injections?

Respondent: Oh. When…when… when she explains that I want an injectable, so you repeat. You will tell her the good side of Sayana. The good and the bad you see? We explain. At the end of that counselling, you are supposed to give that woman, the Sayana she wants. Yes, for her to inject herself.

Interviewer: But there are different types of these injectables right?

Respondent: Yes.

Interviewer: So you will explain…you will explain which injectable?

Respondent: Saya…all of it, it should be her choice. We will tell her about DMPA, its us HSA’s who always injects and also Sayana because Sayana, the drug and syringe are combined. But for the other one, the drug and syringe are separated and this one a woman does not inject herself. She requires someone to inject her who is a provider, you see?

Interviewer: mmmh

Respondent: So you explain all the methods and then the woman makes a choice that I want Sayana because you have explained everything. We explain well how we do it right? DMPA requires that every three months you should be going where?

Interviewer: The hospital

Respondent: While this one you will be doing it yourself and she should understand when you teach her.

Interviewer: mmmhu

Respondent: mmmh

Title: KK_HSA_1.docx

Descriptor Info:	District: Nkhotakota	Target Population: TP1 - Providers/HSAs	Respondent type: HSA	Aware of SI: Yes - had heard of SI before	Coding phase: 2. Double coded	Client marital status: N/A - provider	Client age group: N/A - provider	Time since trained: A year or more ago

Codes Applied:	P3.3 - Counselling/ training approach, general

Interviewer: So during counselling, what do you explain to her when counselling?

Respondent: During counselling, we explain the good and bad about the method. Yes, the good and bad or the problems the person can face when she is using that method. So when she…when you explain to then and when they come across these problems, they know that if I have heavy periods, the HSA explained to me so I have to go to the hospital because you counselled her. When a woman comes, you can not inject her before you explain to her and the you say, go I am tired. The end result is that the woman will be at home but confused. Maybe she will face the problems we are saying here for example heavy periods. She will not make a decision to go to the hospital. Maybe she will not be menstruating, she will think that it is a problem. But when you have been counselled, she knows that oh the HSA already told me that some menstruate and some does not, see. So they know.

Title: KK_HSA_1.docx

Descriptor Info:	District: Nkhotakota	Target Population: TP1 - Providers/HSAs	Respondent type: HSA	Aware of SI: Yes - had heard of SI before	Coding phase: 2. Double coded	Client marital status: N/A - provider	Client age group: N/A - provider	Time since trained: A year or more ago

Codes Applied:	P88 - Provider great quotes	P3.2.3 - Complexity/difficulty of training clients in SI	P2.1.1 - Refresher training/mentorship	P1.2.1 - Negative provider attitudes towards SI	P3.3 - Counselling/ training approach, general

Interviewer: Alright. What is your experience, based on what you have seen since you started providing Sayana to women in your community, aaah what do women say when you explain that they can self-inject?

Respondent: At first women they…they…they doubt and they say that it is not possible for them to what, to self-inject. And we struggle with them. You counsel them and when you are done and ask is there anyone? aah I cant. All of them would say no. when that happens you as a provider, you have just wasted your time right, counselling them and everything eh…eh…by the end of that time everyone is saying no and you have to inject them. it could have been better to just begin…just inject them. But after some time, when they came for supervision they helped us. You should be doing it like this and here you do this, you will see you will have more clients, and that is when they told us that when doing group counselling, and there are two or three women, those ones should be examples to their friends. When we began using that system, we saw that it was working. When you train them, then because its women only, Mrs So-so-so you inject yourself. Your friends here are saying they cannot self-inject. So we will begin with you. And then you will give one woman who has self-injected before. And then she will inject on her thigh. You take pills…you also take the remaining 3 doses and off she goes. The second one will also do the same thing. And then the remaining ones will say oh it is possible and painless and it is short or at home you can do it. And then I saw that I now had what,

Interviewer: mmmmhu

Respondent: mmmh

Title: KK_HSA_1.docx

Descriptor Info:	District: Nkhotakota	Target Population: TP1 - Providers/HSAs	Respondent type: HSA	Aware of SI: Yes - had heard of SI before	Coding phase: 2. Double coded	Client marital status: N/A - provider	Client age group: N/A - provider	Time since trained: A year or more ago

Codes Applied:	P3.1.5 - Ease of training clients in SI	P3.3 - Counselling/ training approach, general

Interviewer: How about the process for…for self injecting right? how do you think it is easy or difficult to train?

Respondent: Aah it is not difficult.

Interviewer: mmmhu

Respondent: Mmmh. Because when you train her, and when you open right, you tell her that she is free to self-inject the area around the belly button. Right hand, left or up here. Or this or that thigh. So when the woman says aah, I will inject on the thigh, you just help her that we hold Sayana like this. And the leg; we also hold the thigh like this.

Interviewer: mmmh

Respondent: yes

Title: KK_HSA_1.docx

Descriptor Info:	District: Nkhotakota	Target Population: TP1 - Providers/HSAs	Respondent type: HSA	Aware of SI: Yes - had heard of SI before	Coding phase: 2. Double coded	Client marital status: N/A - provider	Client age group: N/A - provider	Time since trained: A year or more ago

Codes Applied:	P3.3 - Counselling/ training approach, general	P88 - Provider great quotes

Respondent: Mmmh, if it is an outreach, let us assume that started with the kids with the kids who came sick. You are dealing with the kids, and the women are increasing in number. Mmmh, there will be 6 or 7 women. So you will just counsel them together and inject them. And another phase comes, those who are late, you take them aside and assist them. Which means you will not take time for…a lot of time.

Interviewer: How about busy days?

Respondent: During busy days, we do shortcuts (laughs).

Interviewer: (laughs) eh eh eh

Respondent: During busy days, we also do the same thing. We start one session before this session. We wait for at least the women to be 10 or 8 or 7, where you feel that this a good number. You just teach them in one kick and then give them the methods. But for you to start one on one and being a busy day, it does not work.

Interviewer: okay.

Respondent: yes.

Title: KK_HSA_1.docx

Descriptor Info:	District: Nkhotakota	Target Population: TP1 - Providers/HSAs	Respondent type: HSA	Aware of SI: Yes - had heard of SI before	Coding phase: 2. Double coded	Client marital status: N/A - provider	Client age group: N/A - provider	Time since trained: A year or more ago

Codes Applied:	P4.1.3 - Provider availability/ willingness to repeat SI training	P3.3 - Counselling/ training approach, general	C5.2.2 - Clients incorrectly / failing to administer at home

Interviewer: Alright. So she has her doses. Some time she…what if she comes to you and she still has some and she failed to inject maybe because of some reasons right? What can you tell her?

Respondent: This happens. They come. Oh doctor, eh it went missing. but we explained during counselling. You have to keep at this place, Sayana is supposed to be kept like this this this. Sometimes they come and say I injected the other doses, but the last one I did but…some of the drug did not come out, some spilled. Ah how was it spilled because that time you self injected here you did very well. We face those problems. We still counsel them that what is required is seriousness on the use of the drug. Like what I was saying that some forget that we are supposed to open the drug, and then press. You see? They forget and they just open like this. So no matter how you inject, even if you try the whole thigh, provided if you have not pressed like this, the drug will not come out. So you find that they are coming, the injection could not work, this and that. Which means that is wastage, that one has been destroyed. Yes, so we…we…

Interviewer: What questions do you ask?

Respondent: What happened so that it should not open? What did you do? And then you let her explain. So you polish where she had a problem. And then she will know, ooh this is where I failed, but you told me. Yes from now whenever you want to self inject, do like this. Or you should be storing at such a place with an intention that the medicine should be safe, should be safe this vaccine for….

Interviewer: This one failed to open.

Title: KK_HSA_1.docx

Descriptor Info:	District: Nkhotakota	Target Population: TP1 - Providers/HSAs	Respondent type: HSA	Aware of SI: Yes - had heard of SI before	Coding phase: 2. Double coded	Client marital status: N/A - provider	Client age group: N/A - provider	Time since trained: A year or more ago

Codes Applied:	P3.3 - Counselling/ training approach, general	P4.1.3 - Provider availability/ willingness to repeat SI training

But some one has come because of a different reason she has some left. What questions do you ask her?

Respondent: Oh she is left with some injection?

Interviewer: Mmh, she...she…she…you gave her three doses, but she is left with some, what questions do you ask her?

Respondent: Alright. For us to know that this one has some left, we…we…when we give them, she self injects one dose right there right? And she will be given 3. When coming for the next visit, she is supposed to bring leftovers (used syringes).

Interviewer: okay

Respondent: mmmh mmmh

Interviewer: So what questions do you ask?

Respondent: Firstly when coming they bring…Herself she…because we already tell tell them mmmmh. I am giving you three because your home is far and you have showed an interest you can inject yourself and you are not afraid. When coming after the dosses are finished, we teach them that when they inject, they should keep in a tight plastic bottle, we can say that their safety box in the village.

Interviewer: mmmmh

Respondent: so when coming they are supposed to bring those three things you gave…you gave them. If they have not finished…

Interviewer: They have brought two.

Respondent: Two. You ask. And she also starts telling you before you ask her because she knows she has made a mistake. Sorry counsellor, the other one my neighbor asked for one, I gave my neba you see (laughs).

Interviewer: mmmh

Respondent: Yes. Here is the other one I did not inject I failed to open. So you see all the things.

Interviewer: Mmmh. So this woman, do you inject her your self or you counsel her. Let us assume this one who failed to open, what do you do with her?

Respondent: we ask her what happened for you to fail to open…for the injection not to open. You have tried different ways but the injection is not…the injection is not opening. Aah I did like this. Eeh...so you listen to what she is explaining and then you see that this one made a mistake on that…so you take another one and teach her again where she had forgotten. we said that…or sometimes you give her another one. Madam, here it is, can you open so that I should see. And she has to start explaining. We open, and then we hold here, and we shake the drug. And then we remove the cup and then we do this on our thigh. What have she forgotten? You know that oh she did not press so that the drug should come out. So you tell her that this is where the problem is. You have to press like this so that the drug should come out. When you touch here, the drug will come out before you inject but you should hold here, so do that. Then the woman is…she has done it you are just observing, yes she will inject herself and it is done.

Title: ZA_HSA_5.docx

Descriptor Info:	District: Zomba	Target Population: TP1 - Providers/HSAs	Respondent type: HSA	Aware of SI: Yes - had heard of SI before	Coding phase: 2. Double coded	Client marital status: N/A - provider	Client age group: N/A - provider	Time since trained: Less than a year ago

Codes Applied:	P3.3 - Counselling/ training approach, general

Respondent: the family planning services that we provide in our area are like training the clients on family planning, the advantages, but also when there is a new method for example Sayana, we train women on which are the better methods to access, for example for Sayana they will spend more time before coming to the facility.

Interviewer: Mmm (showing that one is following the conversation)

Title: ZA_HSA_5.docx

Descriptor Info:	District: Zomba	Target Population: TP1 - Providers/HSAs	Respondent type: HSA	Aware of SI: Yes - had heard of SI before	Coding phase: 2. Double coded	Client marital status: N/A - provider	Client age group: N/A - provider	Time since trained: Less than a year ago

Codes Applied:	P3.3 - Counselling/ training approach, general

Interviewer: Alright, alright, among the methods that you have mentioned is there any method that you don’t encourage?

Respondent: No, every method we encourage it, according to the preferences of the client.

Interviewer: Mmm (showing that one is following the conversation)

Respondent: Since we were created different.

Interviewer: Mmm (showing that one is following the conversation)

Respondent: Some you will inject them with Depo or Sayana and they will complain about other things

Interviewer: Yah

Respondent: For some it’s just very okay with them

Interviewer: Mmm (showing that one is following the conversation)

Respondent: So, we are able to recommend another method to them either pills, some say that they are having heavy bleeding, so we tell them to stop using Sayana or Depo and recommend the pills to them

Interviewer: Mmm (showing that one is following the conversation)

Respondent: When it fails that’s when we tell them to come to the facility where they can explore more other methods and they can be assisted.

Interviewer: Mmm (showing that one is following the conversation)

Respondent: Eh

Title: ZA_HSA_5.docx

Descriptor Info:	District: Zomba	Target Population: TP1 - Providers/HSAs	Respondent type: HSA	Aware of SI: Yes - had heard of SI before	Coding phase: 2. Double coded	Client marital status: N/A - provider	Client age group: N/A - provider	Time since trained: Less than a year ago

Codes Applied:	P2.3 - Provider training content	P3.3 - Counselling/ training approach, general

Interviewer: Alright, alright, ah now I want to ask you about your experiences being trained in DMPA-SC or Sayana Press. Could you tell me a bit about your training in providing DMPA-SC?

Respondent: How we were trained?

Interviewer: Yes

Respondent: Sayana is different from Depo, how I was trained

Interviewer: Mmm (showing that one is following the conversation)

Respondent: Because for Sayana, when one gets the injection today then they have to count up to three months

Interviewer: Mmm (showing that one is following the conversation)

Respondent: They will have to inject the same day as the one they got injected today.

Respondent: But for this other one, they give us charts in which we see

Interviewer: Mmm (showing that one is following the conversation)

Respondent: That’s the difference, with Depo, so when you tell the woman she is able to understand, so we write for them properly and tell them that this is the date

Interviewer: Mmm (showing that one is following the conversation)

Respondent: Yes, we were also trained how we can administer Sayana.

Interviewer: Mmm (showing that one is following the conversation)

Respondent: They trained us that when we are administering it as a provider

Interviewer: Mmm (showing that one is following the conversation)

Respondent: We are supposed to inject at the back of the arm

Interviewer: Mmm (showing that one is following the conversation)

Respondent: But the needle, the syringe, should not face upwards, but it should be a bit down

Interviewer: Mmm (showing that one is following the conversation)

Respondent: That’s how they trained us, but also for women who chose to self-inject we should tell them properly they should sit down, and lift their thigh

Interviewer: Mmm (showing that one is following the conversation)

Respondent: Then she should shake the injection unit, or at first, she should check the expiry date

Interviewer: Mmm (showing that one is following the conversation)

Respondent: That when is this injection unit am about to use expiring

Interviewer: Mmm (showing that one is following the conversation)

Respondent: But she should also have a bottle which when she finishes injecting herself…

Interviewer: Dispose in

Respondent: Dispose in, she should wash hands properly

Interviewer: Mmm (showing that one is following the conversation)

Respondent: And take the Sayana and shake it for almost a minute so that the liquid inside can mix properly, and then she should hold the bottle containing Sayana, upright and then activate the unit

Interviewer: Mmm (showing that one is following the conversation)

Respondent: So that the unit should open

Interviewer: Mmm (showing that one is following the conversation)

Respondent: And then she should lift her thigh, and inject squeeze slowly, until all the liquid inside has run out into the body.

Interviewer: Mmm (showing that one is following the conversation)

Respondent: Eh

Interviewer: Alright.

Respondent: That’s what I was trained

Title: ZA_HSA_5.docx

Descriptor Info:	District: Zomba	Target Population: TP1 - Providers/HSAs	Respondent type: HSA	Aware of SI: Yes - had heard of SI before	Coding phase: 2. Double coded	Client marital status: N/A - provider	Client age group: N/A - provider	Time since trained: Less than a year ago

Codes Applied:	P3.3 - Counselling/ training approach, general

Interviewer: Yah, alright, ah now I want you to explain to me how you counsel clients that are considering Sayana Press. Ah I wanted to know as much detail as possible, so please break it down in steps that you would take in counselling this client. To begin, how do you greet them? How do you welcome them?

Respondent: Okay, when a woman comes that they want to access family planning we just don’t start that we have Sayana

Interviewer: Mmm (showing that one is following the conversation)

Respondent: We welcome her, after that we ask her how can we help you? Ah I want to access family planning

Interviewer: Mmm (showing that one is following the conversation)

Respondent: So, I take her book and see what methods she has been using, so she will explain and why she likes that method, what difficulties has she encountered with the method.

Interviewer: Mmm (showing that one is following the conversation)

Respondent: While using that method

Interviewer: Alright.

Respondent: So she will explain everything, if the problems she is facing are fatal for example excessive bleeding which are risky yeah

Interviewer: Yes

Respondent: We tell her that with the way things are you need to go to the facility so that they should examine you or maybe they should change the method for you

TIME INTO THE INTERVIEW: 21Mins 34Secs

Interviewer: Alright, ah how would you start the counselling session?

Respondent: These women, if it’s a new client that they have just started. They have accepted that they want to start using Sayana, I start by telling her the benefits or advantages of Sayana

Interviewer: Mmm (showing that one is following the conversation)

Respondent: A woman takes so much time before coming back to the hospital, and due to the coronavirus, you don’t have to be coming to the hospital more often. When we tell her about that we also tell her that maybe there are other problems for example migraine headache or heart problems

Interviewer: Mmm (showing that one is following the conversation)

Respondent: High blood pressure, things like those we ask her so that we should know

Interviewer: Assuming that the client was only interested in injectables, how would you explain of the options that they have?

Respondent: Concerning what?

Interviewer: Concerning injectable contraceptives

Respondent: Can you ask again the question?

Interviewer: I was saying that let’s assume that a woman who use using family planning was interested in injectable contraceptives, so I wanted you to tell me how you can counsel her the options that she has that she can access.

Respondent: Okay the methods, ah when women have the interest that they want to access injectables, we first ask them what she has heard about family planning if she has any information...

Interviewer: Mmm (showing that one is following the conversation)

Respondent: When she explains to us we start telling her the truth about injectables, now when she as accepted or maybe she has some fears depending on some of the information we have shared with them like excessive bleeding or light bleeding they may have some fear, so we are able to tell her that there are also other methods like pills, you can also take pills every day, there is also the natural method we also explain that to them.

Interviewer: Mmm (showing that one is following the conversation)

Respondent: Which works according to how one’s menstrual cycles works, we also explain that, so it is now up to the client to choose also according to how long she wants to avoid pregnancy

Interviewer: Aright, do you sue any job aids in providing counselling to the clients?

Title: KK_HSA_5.docx

Descriptor Info:	District: Nkhotakota	Target Population: TP1 - Providers/HSAs	Respondent type: HSA	Aware of SI: Yes - had heard of SI before	Coding phase: 1. Single coded	Client marital status: N/A - provider	Client age group: N/A - provider	Time since trained: A year or more ago

Codes Applied:	P3.3 - Counselling/ training approach, general

INTERVIEWER: I would like as much as possible, so please break down steps you would take in counselling this client. To begin with, how would you greet the client? Wat would you say

RESPONDENT: Firstly, we welcome the client, then greet her in a Malawian way. How are you? Then she responds am doing good and you. Then I would introduce myself and tell her my position or tell her the job that I do. I then ask them if they need any help so they can explain.

INTERVIEWER: Ok

RESPONDENT: The other thing that we tell them is to be assured of privacy, because that is one of our responsibility to keep secret of any information about our client. Such explanations put them at ease so that they are able to give us all they had and share with us.

INTERVIEWER: Alright

RESPONDENT: Yes

INTERVIEWER: Next how would you start counselling

RESPONDENT: To begin with, we introduce ourself by telling them that as an HSA it doesn’t mean that am a teacher, because a teacher and an HSA are two different professionals. As an HSA am there to guide them on the right decisions that they should make when it comes to family planning. Hence a guide them through advice, in the process taking note of how much they know about family planning so that in know how I can help them.

INTERVIEWER: Would you ask them any questions? What would you ask them?

RESPONDENT: The type of questions we normally ask incudes; do you the meaning of family planning? Does she know how contraceptives works? Do they know the good or bad side of family planning methods? So the information we gather during questioning time, that helps us on how best we can counsel our client.

INTERVIEWER: Alright

RESPONDENT: Yes

INTERVIEWER: Lets assume the client was only interested

RESPONDENT: Yes

INTERVIEWER: In taking up an injectable option,

RESPONDENT: Ok

INTERVIEWER: How would you explain their options? How many options would you counsel them on? In what order?

RESPONDENT: Whenever we are counselling our clients, we tell them all the options that we provide. We also explain to them how each options works, where they can access it, the good side of it, and talk against any misleading information they may have heard about a particular option. And we also tell them on the effectiveness of each option.

INTERVIEWER: Alright

RESPONDENT: We are now able to discuss further after that. And when a client chooses a particular option, we dwell much and give more details on such a particular option for instance one has chosen injectable, we now talk more on that.

INTERVIEWER: Ok

RESPONDENT: We give them a detailed information about the injectables that we have two types of them. We the explain more on how each works, where to access it, including information on the new method that has just been introduced. On the new one we make sure that we tell them on it advantages over the other one (IM), and what are the problems with the previous method? This one is dependent on the provider, because you may visit a facility for this method, but you may encounter a delay in that you find the nurse is busy, or she has gone to a workshop or the HSA has gone for a different clinic. Hence you have to wait for the provider to come back and administer to you. Whilst with the new method you just collect once and you administer it yourself at home.

INTERVIEWER: Alright.

RESPONDENT: They administer it themselves at home, they reduce visits one can make to a facility. That what we dwell much on, not forgetting to enlighten them that as much as it is one of the best method in avoiding unwanted pregnancy but it cannot protect them from contracting sexual transmitted diseases such a s HIV. So these are some of the issues we discuss with our clients.

INTERVIEWER: Okay

RESPONDENT: Sure

INTERVIEWER: Would you use any material/ job aid?

RESPONDENT: Yes, during our counselling sessions we normally have cards, some books that have pictures of all family planning methods. When we are talking about a particular method for instance a condom we point out on the picture. When we are talking of the injectable methods, we show the picture of the set of the injection like the injection and the bottle that contains the medicine. So during counselling we normally use the counselling cards and and posters.

Title: MZs_HSA_1.docx

Descriptor Info:	District: Mzimba South	Target Population: TP1 - Providers/HSAs	Respondent type: HSA	Aware of SI: Yes - had heard of SI before	Coding phase: 2. Double coded	Client marital status: N/A - provider	Client age group: N/A - provider	Time since trained:

Codes Applied:	P3.3 - Counselling/ training approach, general

Interviewer: Are there any method that you do not usually counsel clients about?

Respondent: As an HSA we do encourage them on all the methods, and we do tell them that all the methods are good.

Title: MZs_HSA_1.docx

Descriptor Info:	District: Mzimba South	Target Population: TP1 - Providers/HSAs	Respondent type: HSA	Aware of SI: Yes - had heard of SI before	Coding phase: 2. Double coded	Client marital status: N/A - provider	Client age group: N/A - provider	Time since trained:

Codes Applied:	P3.3 - Counselling/ training approach, general	P0 - General method mix / method provision

Q8. Interviewers: To begin, how would you greet the client? The client has made her decision that she wants to take injectables, how would you counsel that client? How would you start and what can you say?

Respondent: The first thing we do is to welcome her and introduce myself that I am (Name), am here providing family planning methods.

Interviewer: Mmmmmmm (following)

Respondent: Yes. And after the introduction, we ask what has brought her and from there if she say, I want family planning methods and others they will even say I want injectables.

Interviewer: Mmmmmm. (yes following)

Respondent: Then we start explaining to them all the family planning methods we are offering.

Interviewer: Yes.

Respondent: And after counselling all the methods, we told her to choose the one she wants. Then we counsel again for her to have a clear understanding and ask her some questions for us to know if the method she has opt for is suitable for her or not.

Interviewer: Mmmmmh, (following)

Respondent: And then if we see that the method is suitable for her then we give her the method.

Q9. Interviewer: You have said that you ask her questions for you to know if the client is suitable for the method or not, can you tell me the type of question that you can ask her:

Respondent: Most of the times we ask questions concerning her life if she is suffering from any disease like BP, or if she has ever suffered Jaundice (river cirrhosis) or maybe she varicose veins.

Interviewer: Mmmmmh (following)

Respondent: Things that can be burden for her to take up that method of family planning.

Interviewer: Alright.

Respondent: Others maybe I can forgot cause we have a guidance which we use when counselling them.

Q10. Interviewer: Alright. Let’s assume the client was only interested in talking up an injectable option, how would you explain their option?

Respondent: Mmmmm (yes)

Interviewer: The client says I have come for injectables, so I want you to explain to me on how you can counsel this client?

Respondent: Okay, I will start by telling her that we now have two ways of using injectables. We have provider injection as well as self-injection, on self-injection we counsel you on how you can self-inject and then give you so that you can self-inject at home.

Interviewer: Mmmmh (following)

Respondent: Yes.

Interviewer: How many options would you counsel them, would you counsel them only that method?

Respondent: Aaaaah... (thinking), we counsel them all the methods that we are offering for them to make their choice.

Interviewer: Mmmmh (following)

Respondent: Yes.

Interviewer: Alright, Would you use any materials during counselling?

Respondent: During counselling we also show them pictures of the method and the way it works, things like a man’s penis, condoms and the like.

Interviewer: Mmmmmmh (following)

Respondent: Condoms are also family planning methods.

Interviewer: You said you show them something like a man’s penis when you are counselling what method of FP?

Respondent: We use it whenever we are counselling [on] male family planning methods.

Interviewer: You have said that you show them the method when you are counseling them, do you have anything else that you show them or use during counselling?

Respondent: Nothing else.

Title: MZs_HSA_5.docx

Descriptor Info:	District: Mzimba South	Target Population: TP1 - Providers/HSAs	Respondent type: HSA	Aware of SI: Yes - had heard of SI before	Coding phase: 1. Single coded	Client marital status: N/A - provider	Client age group: N/A - provider	Time since trained: A year or more ago

Codes Applied:	P3.3 - Counselling/ training approach, general

Interviewer: we are now going to talk about counselling on a woman who has made a choice to get injectable. I would like you to tell me how you would counsel this client from reception to the point where she gets the injectable. So I would like you to tell me how you would greet this woman or adolescent?

Respondent: if a woman or an adolescent come to me, I will provide a place to sit and greet her. She will tell me why she has visited. So if she tells me she would like to get injectable, I will tell her that we have 2 types. I have Depo IM and Sayana for self-injection. So a woman would say I want Sayana for self-injection so I will counsel her on how it works, its physiology on pregnancy prevention, and what she may encounter after getting Sayana, and to come back if she sees any side effect.

Interviewer: would you ask her any questions?

Respondent: yes

Interviewer: what type of questions?

Respondent: we have a checklist which guide us on the questions to ask. Have you ever had migraine headache? Do you have high blood pressure? Do you have diabetes? So if she says yes, it means she is not eligible for the method. If not, then she is eligible for the method

Interviewer: do you use any aid when counselling?

Respondent: yes

Interviewer: what aids do you use?

Respondent: we have a booklet which has all family planning methods because we counsel on all methods and a counselling card which has images

Interviewer: counselling card?

Respondent: counselling card

Title: MZs_HSA_5.docx

Descriptor Info:	District: Mzimba South	Target Population: TP1 - Providers/HSAs	Respondent type: HSA	Aware of SI: Yes - had heard of SI before	Coding phase: 1. Single coded	Client marital status: N/A - provider	Client age group: N/A - provider	Time since trained: A year or more ago

Codes Applied:	P3.3 - Counselling/ training approach, general

Interviewer: for those who told you that they cannot manage to self-inject, what did you do?

Respondent: we were just reassuring them then ask them to self-injection. Those who had fears on self-injecting on the abdomen to do it on the thighs

Title: KK_HSA_3.docx

Descriptor Info:	District: Nkhotakota	Target Population: TP1 - Providers/HSAs	Respondent type: HSA	Aware of SI: Yes - had heard of SI before	Coding phase: 3. Triple coded	Client marital status: N/A - provider	Client age group: N/A - provider	Time since trained: A year or more ago

Codes Applied:	P3.3 - Counselling/ training approach, general

INTERVIEWER: As HSA’s, are there other methods that you do not counsel clients about?

RESPONDENT: No. because on all the methods that we counsel/teach on, the method that we provide for example injectables are easily available to them and they accept it. But we still counsel them on all the other methods for example injectables, implant or pills and other methods. Those methods they feel that we should help them with, we do that. But for the other methods, they are assisted at the health facility.

Title: KK_HSA_3.docx

Descriptor Info:	District: Nkhotakota	Target Population: TP1 - Providers/HSAs	Respondent type: HSA	Aware of SI: Yes - had heard of SI before	Coding phase: 3. Triple coded	Client marital status: N/A - provider	Client age group: N/A - provider	Time since trained: A year or more ago

Codes Applied:	P3.3 - Counselling/ training approach, general

RESPONDENT: We encourage them according to the importance of this method. We encourage them because when they are on this method they have time as they don’t have to walk long distances to a health facility to get an injectable because we have these methods in the community and we assist them right there and they have enough time to prepare.

Title: KK_HSA_3.docx

Descriptor Info:	District: Nkhotakota	Target Population: TP1 - Providers/HSAs	Respondent type: HSA	Aware of SI: Yes - had heard of SI before	Coding phase: 3. Triple coded	Client marital status: N/A - provider	Client age group: N/A - provider	Time since trained: A year or more ago

Codes Applied:	P3.3 - Counselling/ training approach, general

INTERVIEWER: I would like to know in detail so please break down the steps you would take when…when counselling a client. To begin with, a woman has arrived, how do you greet her?

RESPONDENT: Firstly, like mu clinic, when a woman arrives…when women arrive, we greet them. Mmh, after greeting them, I tell them my name, I am sososo, introductions right. Then we counsel the woman about the contraceptive method. Its good and bad side...what to expect when she is on this method.

INTERVIEWER: mmmhu.

RESPONDENT: Yes, when some are on Sayana, they stop menstruating, they experience pain…stomach ache or back ache so we tell them that it’s not really a problem. If it persists, we refer them to health centre to meet a doctor. But it’s not really a problem. And it’s just a few with a report of those side effects.

INTERVIEWER: Okay. Alright. So you have greeted her right? Mmmh…how would you start the counselling session?

RESPONDENT: mmmhu.

INTERVIEWER: How do you start counselling session?

RESPONDENT: Injectable counselling?

INTERVIEWER: mmmmh mmmmh [saying no]

RESPONDENT: DMPA-SC or

INTERVIEWER: She has come; she wants a contraceptive method.

RESPONDENT: mmh.

INTERVIEWER: How do you start a counselling session?

RESPONDENT: Okay. Aaaa when a woman has visited and she wants a contraceptive method, firstly, we ask her where she is from, her age, maternal health. Mmmh?

INTERVIEWR: mmmhu

RESPONDENT: Yes. On maternal health, we mean the number of times she has given birth, how many kids she has and how many are alive, and we document in our register. We also explain to her the results of contraceptives. If she is a new client, we explain to her and if she is already our client, we also explain to her. We ask her about motherhood, where she lives, her age and why she wants the contraceptive.

INTERVIEWER: mmmh.

RESPONDENT: mmmh.

INTERVIEWER: Let’s say that you are in your community on a particular day, right? Aaah a client has come right? And she is only interested in taking up an injectable right?

RESPONDENT: mmmh.

INTERVIEWER: How would you explain to her the different options she has?

RESPONDENT: mmmhu.

INTERVIEWER: mmh. Did you get me?

RESPONDENT: Aah her different choices?

INTERVIEWR: This woman has visited…I right. I am on contraceptives, and I I have visited you in your community I want an injectable right? How can you explain to me the options I have?

RESPONDENT: mmmh.

INTERVIEWER: mmmh.

RESPONDENT: Firstly, we explain the benefits of the injectable for example Sayana. We train her how she can self-inject. If she fails, then we inject her. If she is able to according to what we have trained her, we give her the Sayana. We give her 3 sachets so that she should self-inject on the dates we will tell her after every 3 months. If she likes this method and if she will be able to self-inject, we give her Sayana. If she says she will not be able to self-inject, as an HSA, I record in her health passport that on such a date she should come at the same place where I conduct my under five clinic or ICCM.

INTERVIEWER: mmmhu

RESPONDENT: mmmh.

INTERVIEWER: Alright. This woman right, she needs an injectable. But she does not know the types of injectables right?

RESPONDENT: mmmhu.

INTERVIEWER: How many methods will you counsel her on and in what order?

RESPONDENT: alright. There are…

INTERVIEWER: On the injectables right?

RESPONDENT: On injectable contraceptives, there is Sayana for self-injection and Depo. We also provide pills. When we counsel her, the method she will choose, we will counsel her about that method for example Sayana. We will train her the procedure and also about the side effects of Sayana so that when she will experience the signs, she should know that it’s the Sayana. mmmmh

Title: KK_HSA_3.docx

Descriptor Info:	District: Nkhotakota	Target Population: TP1 - Providers/HSAs	Respondent type: HSA	Aware of SI: Yes - had heard of SI before	Coding phase: 3. Triple coded	Client marital status: N/A - provider	Client age group: N/A - provider	Time since trained: A year or more ago

Codes Applied:	P3.3 - Counselling/ training approach, general

INTERVIEWER: When counselling her, what information do you cover?

RESPONDENT: What I can tell her during counselling is the importance of injectables and also the importance of the contraceptive? When we explain all that and when she accepts, we will give her or inject her.

INTERVIEWER: okay.

RESPONDENT: mmmhu.

INTERVIEWER: So you will give her for self-injection or you will inject her?

RESPONDENT: Yes.

INTERVIEWER: Alright. So this woman wants Depo…and she has said I want an injectable, you have to inject me, right? During counselling…the options that she has, in what order do you tell her?

RESPONDENT: The choices about the method she wants?

INETRVIEWER: yes

RESPONDENT: Her choices?

INTERVIEWR: yes.

RESPONDENT: It’s up to her when we explain all the methods, it’s up to her to choose.

INTERVIEWER: okay. When explaining to her, in what order do you explain the injectable?

RESPONDENT: The one we start with?

INTERVIEWER: Mmmh. What injectable do you first explain…okay how should I explain. We have two types of injectables right? So in what order do you explain the injectables? Which one do you start with?

INTERVIEWER:

RESPONDENT: Aaah as I have already said that if we have Sayana mmh we will explain about Sayana. Id don’t have Sayana we have Depo; we will explain the method that we have. Whether Depo or Sayana. If Sayana is out of stock, we bring Depo and vice versa. We explain to her the injectable we have on that day/time

Title: KK_HSA_3.docx

Descriptor Info:	District: Nkhotakota	Target Population: TP1 - Providers/HSAs	Respondent type: HSA	Aware of SI: Yes - had heard of SI before	Coding phase: 3. Triple coded	Client marital status: N/A - provider	Client age group: N/A - provider	Time since trained: A year or more ago

Codes Applied:	C1.1.1 - Positive client attitudes to SI	P3.3 - Counselling/ training approach, general

INTERVIEWR: Alright. When you explain to women about Sayana, and the possibility of self-injection at home, what is their response?

RESPONDENT: After explaining to them, most of them would say that it is a good method…but those who would say they cannot self-inject, we would inject them. But we would give Sayana to someone who would say that she will be able to self-inject.

Title: KK_HSA_3.docx

Descriptor Info:	District: Nkhotakota	Target Population: TP1 - Providers/HSAs	Respondent type: HSA	Aware of SI: Yes - had heard of SI before	Coding phase: 3. Triple coded	Client marital status: N/A - provider	Client age group: N/A - provider	Time since trained: A year or more ago

Codes Applied:	P3.4 - Time to counsel/train, general	P3.3 - Counselling/ training approach, general

INTERVIEWER: How long does it take right, when a woman comes? How long does it take to train this client for you to know that this client will self-inject? How long does it take?

RESPONDENT: Okay. Aaa depending on how we train the women. We don’t have a specific time that maybe 2hours, 3hours, 4hours. But it depends on the training and the practical session if the women will be able to self-inject. So we cannot say that maybe 2hours, 3hours. But it depends on how serious they are and if they are interested…

INTERVIEWER: Yes continue

RESPONDENT: That these ones will be able to self-inject.

INTERVIEWER: But on average…because you have trained a lot of women, right?

RESPONDENT: mmmh.

INETRVIEWER: How long do you think it takes?

RESPONDENT: 2hours.

INTERVIEWER: 2hours?

RESPONDENT: mmmh.

INTERVIEWR: One woman?

RESPONDENT: mmmh.

INTERVIEWER: To be able to self-inject?

RESPONDENT: mmmmh.

INTERVIEWER: mmm. Do you feel you have…do you feel you have enough time…do you have enough time to conduct these trainings?

RESPONDENT: Yes, we have enough time because when we arrive for example at 8 o’clock, we know that we will be there until 2 o’clock. I finish training all the women within these hours.

INTERVIEWER: What happens on busy days? You are in your community and it’s a busy day, what happens?

RESPONDENT: Okay. The day I am in my community to do other work, it’s the same day I am working with under-five children, right? When I am done with the underfive clinic for example those with malaria and other issues, I also spare sometime for women on family planning. So maybe I can meet 5 or 6 women depending on the ones who want contraceptives. And I know that now I have this number of women. These 2 hours are for all the women. I do not counsel them separately for 2hours each. If there are 5 or 6 or 10 women, I train them together. when I counsel then together, I also make time to see if see them individuality if they will be able to self-inject. “Can you do it? Try I should see” [asking the client] We have resources for practice. The 2hours is enough to train the women in group counselling.

Title: ZA_HSA_4.docx

Descriptor Info:	District: Zomba	Target Population: TP1 - Providers/HSAs	Respondent type: HSA	Aware of SI: Yes - had heard of SI before	Coding phase: 1. Single coded	Client marital status: N/A - provider	Client age group: N/A - provider	Time since trained: A year or more ago

Codes Applied:	P3.3 - Counselling/ training approach, general	C8.3 - Discretion of injectables/SI

Interviewer: Alright thank you so much!! Now we are going to the next section. In this section I Would like for you to talk me through how you counsel a client who is considering an injectable contraceptive. I would like as much detail as possible, so please break down the steps you would take in counselling this client

Respondent: We have a chart that we use but we received this chart when we got the Depo, so they are about 13 questions.

Interviewer: Alright let me just go back a little bit. Can you tell me how you greet a woman who just came?

Respondent: When a woman comes we greet them either in the morning or afternoon, how are you? I’m fine and you. How can we help you? Yes are you using any family planning method? You want to start using family planning methods? Yes. How many children do you have? Aaah one , aaah two. Aaaah I don’t have a child.

Interviewer: Yes yes

Respondent: So maybe in a joking way you want to start getting contraceptives without a baby why talking to them like this. Iiih maybe the husband is not mine. So we tell them that we just administer the injection they know the truth themselves. We teach them the benefits and disadvantage about contraceptive. There are several methods of contraceptive there’s injection and pills and implant.

Interviewer: You said implant?

Respondent: All these are methods of family planning there’s loop but the ones we administer in the community are injections and pills sometimes. So which one will you choose? Aah I want the injection, so we give them

Interviewer: Mmmh

Respondent: If you are in your period you keep menstruating but eventually you stop

Interviewer: Mmmh

Respondent: Then when it stops when you come for the next time you start again some menstruate every month but with Depo sometimes you just spot then they can differentiate. We explain to the women so maybe they should have knowledge about contraceptives. So we tell them the importance of family planning that when you have a small baby you take care of the baby with all of your heart you don’t have stress and also chores are done without problems. When the baby grows say 5yrs or 6yrs you are free to leave the baby in the hands of neighbor’s, but under 5 children are hard to leave them things like those.

Interviewer: Alright thank you. Assuming the client was only interested in taking up an injectable option, how would you explain their options?

Respondent: Options like?

Interviewer: Methods that are recommended

Respondent: Oooh but I want the injection, i have interest in the injection

Respondent: We tell them that injection is good because whether you sneak out from home or you don’t you want the neighbor to know that you get the vaccine you are free but also ooh I forgot the other point.

Interviewer: Take your time.

Respondent: Ooh the importance of injection is if you want to start getting babies again you just stop from the pregnant again. It works like that.

Interviewer: Mmmmh okay fine. So when you are telling the women about the contraception methods, do you also tell them about the other contraception methods that you have, or you just focus on the method which the woman has chosen. For example, let’s say the woman wants to get the injection, do you only tell her more about the method she has chosen or?

Respondent: When the women have chosen the injection method, we also tell them that there are two types of injection thus Depo and Sayana. “There are also pills if you might be in need of them”. Some women may say that I may not need pills because am afraid of taking pills each and every day or some say that they may easily forget to take the pills some other days. Therefore, they just opt for that injection method. So, they may ask about the difference between Depo and Sayana. We tell them that there are not any differences between the two as their function is all the same only that the injection process or the place of injection is different. So now the women choose now whether to go for Depo or Sayana

Title: ZA_HSA_4.docx

Descriptor Info:	District: Zomba	Target Population: TP1 - Providers/HSAs	Respondent type: HSA	Aware of SI: Yes - had heard of SI before	Coding phase: 1. Single coded	Client marital status: N/A - provider	Client age group: N/A - provider	Time since trained: A year or more ago

Codes Applied:	P3.3 - Counselling/ training approach, general

Interviewer: Ohh! Thank you so much. Do you use any other resources which may help you in counselling the women about the family planning methods? Do you use any resource?

Respondent: Mmm we don’t have resources.

Interviewer: But you talked of a chart.

Respondent: Ohh yes, the chart is only used to those who have just started using the contraception methods.

Interviewer: Ohh it’s for those who have just started?

Respondent: Yes, those who have not used any contraception method before. So yes we use the chart to ask them questions (question 1-13). Are you breastfeeding a six months old child?

Interviewer: Ummm

Respondent: Yes… no… or six weeks. After having sex with your husband, do you discharge blood from your private part? ... No… Or when you are just staying, do you feel some pains from the other side of your legs? ... No… Or do you have swollen parts?

Interviewer: Ohh Okay fine. Thank you! Thank you so much! What are some of the things you explain to the women when counselling them about the contraception methods?

Respondent: We tell the women about the advantages as well as the disadvantages of the methods.

Interviewer: Ohh! Is there anything else?

Respondent: Aaaah…

Interviewer: Like how the methods function?

Respondent: Oh yes. Like for those who have just started using the contraception methods, we also give the condoms so that by the time she has been injected, she should have about 10 days that when she will have sex with her husband on these days, they should use a condom. So yeah, they are given condoms.

Interviewer: Ohh okay, so that the injection should start functioning well in the body?

Respondent: Yes, because the woman can get the injection and them tomorrow, she may have sex with a man and get pregnant. She may later on say that the injection is useless.

Interviewer: Thank you so much! Will the counselling you give to those women using the injection method be different with how you advised them in the past?

Respondent: No. It’s not different because it is the same injection or you just the woman that here are the functions of Sayana and its just the same as how you were using depo, there is no difference.

Interviewer: Okay. How many contraception methods can you counsel the women on? And in what way?

Respondent: Counselling them? Like for me, I advise them on the contraception methods that we provide, and we also tell them that if they feel like they have had many children, it’s better to go for tubal ligation.

Title: ZA_HSA_4.docx

Descriptor Info:	District: Zomba	Target Population: TP1 - Providers/HSAs	Respondent type: HSA	Aware of SI: Yes - had heard of SI before	Coding phase: 1. Single coded	Client marital status: N/A - provider	Client age group: N/A - provider	Time since trained: A year or more ago

Codes Applied:	P4.1.1 - Follow up materials for client to take home	P3.3 - Counselling/ training approach, general

Interviewer: Okay. Thank you so much. Do you give any other additional help to those women who have opted for the self-injecting method?

Respondent: When the women have come for the injection, we give them the injection which they will inject themselves for three times and then we give them dates of which we have calendars. We also give them cotton swabs and we tell them to fine a tin of which after injecting themselves, they should throw what they have used in it.

Interviewer: Alright. Are the materials you give the women enough?

Respondent: Yes

Interviewer: Do the women get comfortable taking these?

Respondent: Yes, they do because they do accept what they are supposed to do.

Title: ZA_HSA_4.docx

Descriptor Info:	District: Zomba	Target Population: TP1 - Providers/HSAs	Respondent type: HSA	Aware of SI: Yes - had heard of SI before	Coding phase: 1. Single coded	Client marital status: N/A - provider	Client age group: N/A - provider	Time since trained: A year or more ago

Codes Applied:	P3.3 - Counselling/ training approach, general

Interviewer: Will the women get encouraged with the length of training?

Respondent: No. Sometimes they get bored with the lessons.

Title: KK_HSA_4.docx

Descriptor Info:	District: Nkhotakota	Target Population: TP1 - Providers/HSAs	Respondent type: HSA	Aware of SI: Yes - had heard of SI before	Coding phase: 1. Single coded	Client marital status: N/A - provider	Client age group: N/A - provider	Time since trained: A year or more ago

Codes Applied:	P3.3 - Counselling/ training approach, general

RESPONDENT: So it’s up to her to choose the type of contraceptive she wants. So for instance, she chooses Sayana

INTERVIEWER: Yes

RESPONDENT: The self-injection one

INTERVIEWER: Yes

RESPONDENT: We ae now supposed to explain in details how she can do self-injection, and the period through which the method works that is from the time she injects herself to the next injection.

INTERVIEWER: Alright

RESPONDENT: We are also supposed to tell her the good side and bad side of the method.

INTERVIEWER: Alright

RESPONDENT: So if she chooses Sayana we go into details about the method she has chosen.

INTERVIEWER: Alright

RESPONDENT: However, we still explain to her on the other methods but not in details as the one she has chosen.

INTERVIEWER: Alright

RESPONDENT: After counselling the client gets convinced about the method that she wants to take

INTERVIEWER: Ok

RESPONDENT: Then we assist the client how to do self-injection later on we explain to her about the things that she may encounter as she is using the method, for example side effects.

INTERVIEWER: Alright

RESPONDENT: Ma side effects Are things that may come along as the client is using the method, for example prolonged periods.

INTERVIEWER: Ok

RESPONDENT: We advise the client that, if she noted that she having prolonged periods she should come at a facility to meet the doctor or the HSA.

INTERVIEWER: Alright.

RESPONDENT: Incase the clients meets us we counsel her and refer her to the nurse at a facility.

INTERVIEWER: Alright

RESPONDENT: Because normally we assist our clients right in the community

INTERVIEWER: Thank you so much.

RESPONDENT: Yes

Title: KK_HSA_4.docx

Descriptor Info:	District: Nkhotakota	Target Population: TP1 - Providers/HSAs	Respondent type: HSA	Aware of SI: Yes - had heard of SI before	Coding phase: 1. Single coded	Client marital status: N/A - provider	Client age group: N/A - provider	Time since trained: A year or more ago

Codes Applied:	P3.3 - Counselling/ training approach, general

INTERVIEWER: Thanks you so much for such a detailed explanation. Are there any methods that you do not usually counsel clients about? why?

RESPONDENT: WE know that there are a number of options and for us we counsel all of them

INTERVIEWER: Alright

RESPONDENT: Because normally when a client comes to us to take a family planning method, we do explain all the methods. But I depend on the likeness of the clients which type of method does she want to use.

INTERVIEWER: Alright

RESPONDENT: So depending on the choices that most people are taking, we just make sure that we respect the method that our client has taken, consider in that may be after counselling that’s the one she liked the most. However, we make sure that counsel her well on the choice she has made.

Title: KK_HSA_4.docx

Descriptor Info:	District: Nkhotakota	Target Population: TP1 - Providers/HSAs	Respondent type: HSA	Aware of SI: Yes - had heard of SI before	Coding phase: 1. Single coded	Client marital status: N/A - provider	Client age group: N/A - provider	Time since trained: A year or more ago

Codes Applied:	P3.3 - Counselling/ training approach, general

INTERVIEWER: As we proceed with our conversation on this topic This time around, I would like for you to take me through how you counsel a client who is considering and injectable contraceptive. I would like as much as possible, so please break down steps you would take in counselling this client. To begin with, how would you greet the client? What would you say?

RESPONDENT: Ok Firstly, when a client’s visits us in the community in normal circumstances she is supposed to knock

INTERVIEWER: That’s true

RESPONDENT: After a knock, we allow her to get in and we give her a seat and the we greet her.

INTERVIEWER: Alright

RESPONDENT: How are you? Am fine and you. I am also fine. May I help you? Yes, I am here to take a contraceptive. Then we take her through all the options that are available. We do not just give her a method that we she uses, but we try to counsel her on all that are available.

INTERVIEWER: [missing?]

RESPONDENT: We are supposed to counsel her on all options that are available.

INTERVIEER: Alright

RESPONDENT: After explaining to her on all options that we have the client now choses the method she is interested in.

INTERVIEWER: Ok

RESPONDENT: after making a choice, for instance she has chosen Sayana Press, we need to explain to her all about the method like it’s a method that one injects herself. We are also supposed to tell her how long will the method be effective in her body.

INTERVIEWER: Alright

RESPONDENT: But before administering the method we have to ask her some questions

INTERVIEWER: Alright

RESPONDENT: We normally have a checklist of questions that allows a client to be on family planning.

INTERVIEWER: Alright

RESPONDENT: After administering the questions to the client, we able to know whether she is eligible for contraception or not depending on her responses. If she responds so well to the questions, we are set to give the method of her choice. However, before that we also explain to her on the side effects of the method she has taken.

INTERVIEWER: Ok

RESPONDENT: For instance, when women are using most for these family planning methods, they may stop mensuration. Hence, we need to let the client know about all this so that if she encounters such a thing whilst using the method she will not get surprised

INTERVIEWER: Alright

RESPONDENT: But things like these will not cause any problem in your body.

INTERVIEWER: Alright

RESPONDENT: But there may be some things that you may encounter whilst using the method like continuous bleeding. In such cases you are not supposed to be comfortable at home.

INTERVIEWER: Ok

RESPONDENT: You are supposed to come and report to us. After receiving such reports, we make arrangements that the client go to a facility and meet the nurse. (30 minutes 10 seconds)

INTERVIEWER: Alright

RESPONDENT: Furthermore, we also advise the client that the method she has taken will not protect her from contracting sexual transmitted diseases

INTERVIEWER: True.

RESPONDENT: This method is only effective on issues of pregnancy, but whenever you want to have sexual intercourse with someone whom you are not married to, you should make sure you use a condom.

INTERVIEWER: Alright.

RESPONDENT: If the client is accessing the family planning method for the first time. We are supposed to provide her with a backup because the medication does not start working instantly taking it.

INTERVIEWER: Alright

RESPONDENT: That’s when we are set and free to give the client a contraceptive that she has chosen.

INTERVIEWER: Alright

RESPONDENT: Let me stop a bit.

Title: KK_HSA_4.docx

Descriptor Info:	District: Nkhotakota	Target Population: TP1 - Providers/HSAs	Respondent type: HSA	Aware of SI: Yes - had heard of SI before	Coding phase: 1. Single coded	Client marital status: N/A - provider	Client age group: N/A - provider	Time since trained: A year or more ago

Codes Applied:	P3.3 - Counselling/ training approach, general

INTERVIEWER: Alright, at some point you mentioned that before you offer the client option to choose from, you have a list of questions that you administer to her in order to check if she is fit for any family planning method. May you please share with me some of the questions that you usually ask.

RESPONDENT: Do you faint?

INTERVIEWER: Alright

RESPONDENT: Have you ever had migraine headache

INTERVIEWER: Alright

RESPONDENT: These are some of the questions that we usually ask.

INTERVIEWER: Ok Fine. Let’s assume the client was only interested in taking up an injectable option, how would you explain their options? How many options would you counsel them on? In what order? I recall you started explaining of the options that are available, how would you explain about the options that are available. I know you may have already explained a lot, but you can just add up what you may have left out.

RESPONDENT: Alright, we really supposed to explain all the options that are available, for example we may mentions options like self-injection, pills, condoms and the natural remedy of breastfeeding

INTERVIEWER: Ok fine

RESPONDENT: Also we have loop

INTERVIEWER: Yes

RESPONDENT: These are some of the options that were normally explain to them.

Title: KK_HSA_4.docx

Descriptor Info:	District: Nkhotakota	Target Population: TP1 - Providers/HSAs	Respondent type: HSA	Aware of SI: Yes - had heard of SI before	Coding phase: 1. Single coded	Client marital status: N/A - provider	Client age group: N/A - provider	Time since trained: A year or more ago

Codes Applied:	P3.3 - Counselling/ training approach, general

INTERVIEWER: Alright, would you use any material/ job aid? As you are providing the services

RESPONDENT: Yes, when conducting counselling services we are supposed to have a counselling card book, which contains pictures of all the options that I have mention above.

INTERVIEWER: OK

RESPONDENT: Yes, and in our local language we call it a book that is used when counselling women who have come to take contraceptives.

INTERVIEWER: Alright

RESPONDENT: Yes, since the book contains all the options that I have mentioned, when counselling the client, we are supposed to open page by page so that the client should be able to see these options that are being explained.

INTERVIEWER: Alright

RESPONDENT: As we are showing our clients the options at the same time we are supposed to tell her how each option works and the side effects. WE go to another option s we tell her how it works and tell her the good side and bad side of it, and also how effective is the option in terms of preventing her from getting pregnancy, since the options have different percentages.

INTERVIEWER: Alright

RESPONDENT: Yes, some are more effective like they may be as higher as 90 something percent.

INTERVIEWER: Alright fine

RESPONDENT: Some are less effective with lesser percentage, so the client has a choice to make depending on the one that she sees is more effective to her that can prevent her from getting pregnancy.

Title: KK_HSA_4.docx

Descriptor Info:	District: Nkhotakota	Target Population: TP1 - Providers/HSAs	Respondent type: HSA	Aware of SI: Yes - had heard of SI before	Coding phase: 1. Single coded	Client marital status: N/A - provider	Client age group: N/A - provider	Time since trained: A year or more ago

Codes Applied:	P3.3 - Counselling/ training approach, general

INTERVIEWER: Alright, If a client you had previously trained in SI returned to you without having completed her injection for some reason, what would your response be? What questions would you ask her? Are clear?

RESPONDENT: Yes, am clear. If we have been visited by a client who have not yet finished the doses that she was given to administered at home. We normally enquire first what has made her to come back to us before she has finished the dose, and we take our time to listen to her so that we can get the reasons for her coming. It may be that she may have encountered something as she is taking the contraceptive.

INTERVIEWER: Ok

RESPONDENT: That’s why I said we are supposed to listen to her concerns first.

INTERVIEWER: Alright

RESPONDENT: For example, she may come with issues of what she has been encountering whilst using the method.

INTERVIEWER: Ok

RESPONDENT: The method

INTERVIEWER: Ok

RESPONDENT: Hence we are supposed to remind them of the things that we mentioned during our counselling sessions. Since she may have brought issues concerning prolonged periods of which we had talked about, but she may have forgotten what we said.

INTERVIEWER: Ok

RESPONDENT: So we are supposed to [call?] our client back and remind her that we had discussed such issues during counseling.

INTERVIEWER: Ok

RESPONDENT: that if we encounter such thing we need to do this and that

INTERVIEWER: Ok

RESPONDENT: So for example is she comes with a concern about prolonged periods, that since I started using this method, I have been going through this. It may be that she may have forgotten about wat you told her on what to do in such cases. Hence you remind her that with her concern she is supposed to go to a facility and meet the nurse.

Title: MZs_HSA_4.docx

Descriptor Info:	District: Mzimba South	Target Population: TP1 - Providers/HSAs	Respondent type: HSA	Aware of SI: Yes - had heard of SI before	Coding phase: 1. Single coded	Client marital status: N/A - provider	Client age group: N/A - provider	Time since trained:

Codes Applied:	P3.3 - Counselling/ training approach, general

Interviewer: are there any methods which you do not encourage the client to use?

Respondent: no, we put emphasis on all methods.

Interviewer: thank you.

Title: MZs_HSA_4.docx

Descriptor Info:	District: Mzimba South	Target Population: TP1 - Providers/HSAs	Respondent type: HSA	Aware of SI: Yes - had heard of SI before	Coding phase: 1. Single coded	Client marital status: N/A - provider	Client age group: N/A - provider	Time since trained:

Codes Applied:	P3.3 - Counselling/ training approach, general

Interviewer: what are the other problems you faced when Sayana was available?

Respondent: some problems faced were that some clients had difficulties to understand about self-injection. However, we try in teaching them in groups such that other clients just come for Sayana because they already know how to use it.

Title: MZs_HSA_4.docx

Descriptor Info:	District: Mzimba South	Target Population: TP1 - Providers/HSAs	Respondent type: HSA	Aware of SI: Yes - had heard of SI before	Coding phase: 1. Single coded	Client marital status: N/A - provider	Client age group: N/A - provider	Time since trained:

Codes Applied:	P3.3 - Counselling/ training approach, general	C2.1.2 - Reduced trips to facility	C5.1.1 - Continuation of SI (including reasons)

Interviewer: do you have any difficulties in differentiating self-injection form other injectables?

Respondent: most people prefer self-injection because they do not like to travel to the facility. There was one client who said that by using Depo she hard an abnormal menstruation while Sayana does not.

Interviewer: how did you interact with this client?

Respondent: I explained to the client that the two methods are similar. I asked her if she went to the hospital after experiencing such condition? She also explained that she believed that it was the Depo method which made her menstruate abnormally while Sayana did not. So I advised her to go to the hospital and seek clarification of whether this was caused by the method or not.

Title: MZs_HSA_4.docx

Descriptor Info:	District: Mzimba South	Target Population: TP1 - Providers/HSAs	Respondent type: HSA	Aware of SI: Yes - had heard of SI before	Coding phase: 1. Single coded	Client marital status: N/A - provider	Client age group: N/A - provider	Time since trained:

Codes Applied:	P3.3 - Counselling/ training approach, general

Q8. Interviewer: in this section I will ask what advice do you give to women who want to use the injectables. As such you should expansively explain the processes. firstly, how do you greet the client?

Respondent: we first welcome and greet the client. Thereafter, I introduce myself as a health counsellor. Then we ask how we may help her. I also ask how or from whom she knew about the methods. She may then respond. Thereafter, I introduce the family planning methods which we have at this facility and show to her how each method works. From there the client is given chance to choose a method. I also highlight on the advantages and disadvantages of the method chosen including how the method works. From there I wash hands and inject the client.

Q9. Interviewer: how do you begin counselling? Do you ask the client any questions?

Respondent: yes, I ask the client questions to inquire what the client knows about the method.

Interviewer: what questions can you ask?

Respondent: I first ask how he came across the information about family planning methods.

Interviewer: Mmmmmh

Respondent: the client may say that she heard from her neighbor. Others say that the midwives told them that six months after giving birth they should be on family planning. From there I begin explaining about the methods which we have.

Interviewer: Mmmmmh

Respondent: from there the client also asks questions and we shed more light so that she should not have difficulties where ever she goes.

Title: MZs_HSA_4.docx

Descriptor Info:	District: Mzimba South	Target Population: TP1 - Providers/HSAs	Respondent type: HSA	Aware of SI: Yes - had heard of SI before	Coding phase: 1. Single coded	Client marital status: N/A - provider	Client age group: N/A - provider	Time since trained:

Codes Applied:	P3.3 - Counselling/ training approach, general

Q10. Interviewer: how can you explain to a client who has interested in the injectable method the methods available?

Respondent: I explain the advantages and disadvantages of the method. I also explain that we have two types of the injectable methods which are self-injection and the one injected by the professional. We also explain the duration of each injection and when to inject again. So the person chooses whether to adopt self-injection or not. I also explain on other types of family planning and then I repeat explaining the method which the client has chosen.

Interviewer: may you please shed more light on the injectable method?

Respondent: I explain that the first injection takes about seven days to start working and it has a duration of three months. Then I explain how the injected drug works in the body. For instance, it hardens the some bodily fluids and prevents one from getting pregnant.

Interviewer: on how many methods can you counsel them and in what order?

Respondent: I do not understand the question

Interviewer: on how many methods can you offer your counselling skills and in what order?

Respondent: I explain two or three methods. the first one is Depo which has a duration of three months. then Sayana which is a self-injectable if the client chooses so. a client who has chosen to self-inject is taught on how to do so and one can inject on the abdomen or upper leg. We tell them to shake the drug first before injecting and how to dispose of the material used. The client is given the drugs to self-inject at home. However, they are advised that if they face any problem they should come back to the facility.

Title: MZs_HSA_4.docx

Descriptor Info:	District: Mzimba South	Target Population: TP1 - Providers/HSAs	Respondent type: HSA	Aware of SI: Yes - had heard of SI before	Coding phase: 1. Single coded	Client marital status: N/A - provider	Client age group: N/A - provider	Time since trained:

Codes Applied:	P3.3 - Counselling/ training approach, general	P4.1.3 - Provider availability/ willingness to repeat SI training

Q16. Interviewer: if a client comes while she has not finished using the self-injections what can you advise them?

Respondent: they explain the problems they have faced.

Interviewer: Mmmmh.

Respondent: so we try to find the cause of their problem. Then we refer them to the hospital if need be. If they are over menstruating or suspect that they are pregnant we also refer them.

Interviewer: if a client says she cannot manage to self-inject, can you manage to inject her or you can give her other counsel?

Respondent: I have not yet encountered such client.

Interviewer: what if it happens?

Respondent: if such situation can happen, I will know that the counsel I gave her was not effective and the client did not understand. So I can explain again to the client on how to self-inject. If she still does not understand I will do the injection for her.

Interviewer: alright.

Title: MZs_HSA_3.docx

Descriptor Info:	District: Mzimba South	Target Population: TP1 - Providers/HSAs	Respondent type: HSA	Aware of SI: Yes - had heard of SI before	Coding phase: 3. Triple coded	Client marital status: N/A - provider	Client age group: N/A - provider	Time since trained: Less than a year ago

Codes Applied:	P3.3 - Counselling/ training approach, general

Interviewer: are there any methods that you do not usually counsel clients about? why?

Respondent: no….(moment of silence) we counsel all methods, but they have to choose which to take. Yah!

Title: MZs_HSA_3.docx

Descriptor Info:	District: Mzimba South	Target Population: TP1 - Providers/HSAs	Respondent type: HSA	Aware of SI: Yes - had heard of SI before	Coding phase: 3. Triple coded	Client marital status: N/A - provider	Client age group: N/A - provider	Time since trained: Less than a year ago

Codes Applied:	P3.3 - Counselling/ training approach, general

Q.8 Interviewer: to begin, how would you greet the client?

Respondent: [giggles]…we welcome that client, come in she comes in. have a sit we give how are you we greet her greetings we make greetings then from there I introduce myself I ask her who she is later on I ask her why she come to me yes then later on she says I have come to get the family planning method yah. Then From there I will start to ask what she knows about family planning method. Or she has been getting from somewhere or she is a new client. Soooo she… she will talk what she knows from there I have to come in to say ok this is of course you are not wrong in the way but I have to tell what she is supposed to know. This is like this this is like this. So what she said and what I have told her she will make them put them together then from there I will say from now these are the family planning method which I provide one by one, one by one from there after finishing I have to give her a space whereby she has to choose which method she would like to get. Then after choosing that method then I have to say because I told her more methods eti? Now she has chosen I have to go back non that topic then explain again so that she will understands on what will happen after getting that one. Then from there that’s when I can give her that method. Of course I am hiding maybe because we are talking in English. Of course what am talking is it understandable to those who are getting ENGLISH?

Interviewer: Yes you are free to speak in Tumbuka.

Interviewer: would you ask them any questions? What would you ask them?

Respondent: aaaaah… have you ever taken any family planning methods? Do you have mmmm… maybe I should say do you have BP? Or you have got…..heart attack somewhere? Or………how how long mmmmmm……….have you slept with…..your husband within the days? Or…when did you last get the family planning if she is an…oooh….eeeeeh….subsequent client if she was somewhere she got that one and now she is coming to me now then I should say when? Because when I give her she may go while she had a break with that because she might have taken some weeks before coming to you. So I have to ask her if that was her date to get or she had a break yah.

Q10. Interviewer: Assuming the client was only interested in taking up an injectable option, how would you explain their options?

Respondent: about the options because there is Depo and there is Sayana. So Depo she has to get Depo if she would like to get Depo Depo will take 3 months it’s just the same like Sayana its all 3 months but there is Depo has to be taken with me as a provider she cannot get that one at home but Sayana has got an advantage of getting it home so long as she understands what I will tell her on how to get that Sayana. So that one will make her stay at home instead of coming here every 3 months but depo would like her to be coming every 3 months but Sayana will mmmmh she has to stay there for almost a year.

Title: MZs_HSA_3.docx

Descriptor Info:	District: Mzimba South	Target Population: TP1 - Providers/HSAs	Respondent type: HSA	Aware of SI: Yes - had heard of SI before	Coding phase: 3. Triple coded	Client marital status: N/A - provider	Client age group: N/A - provider	Time since trained: Less than a year ago

Codes Applied:	P3.3 - Counselling/ training approach, general

Interviewer: what kind of information would you cover in counselling?

TIME STAMP 32:21- 44-20MIN

Respondent: yes.. if that client is new she has to choose to get a back up or not it depends on how she feels you are getting this one today and you have some days to go before you meet your husband . So are you able to wait for that period or you can get other methods like condoms? So she has to say you give me or you don’t give me. When she gets that one either her body will choose to have three things; the first one will be either to may do menstruation or she may stop or she might be bleeding heavily. So when she bleeds heavily she has to come to the health center or to come back so that she should be given some other family planning method so that she should reduce that menstruation period. Yah.

Title: MZs_HSA_3.docx

Descriptor Info:	District: Mzimba South	Target Population: TP1 - Providers/HSAs	Respondent type: HSA	Aware of SI: Yes - had heard of SI before	Coding phase: 3. Triple coded	Client marital status: N/A - provider	Client age group: N/A - provider	Time since trained: Less than a year ago

Codes Applied:	C8.1 - Male involvement, general	P3.3 - Counselling/ training approach, general

Interviewer: what role do you think the woman husband/ partner plays in the decision?

Respondent: those who are able when they come together, they say you get this one I will do it for you mmmhu because ether the woman will say aaaaah… koma Sayana ine sindingatenge… I shuu…I can’t get I have understood what you have said but I can’t do it I can’t give it myself so the husband if he is there, he says no I have understood what they have said. Then I will give you. Then before giving them you have to also interview that husband, did he get what I said? Is he true that… is it true he has understood what I have said? Others will say aaah let me give you meaning her what her wife let me give you. So when he gives, we know kuti ok he has given her it means he can do it at home. Yah

Title: KK_HSA_2.docx

Descriptor Info:	District: Nkhotakota	Target Population: TP1 - Providers/HSAs	Respondent type: HSA	Aware of SI: Yes - had heard of SI before	Coding phase: 2. Double coded	Client marital status: N/A - provider	Client age group: N/A - provider	Time since trained: A year or more ago

Codes Applied:	P3.3 - Counselling/ training approach, general

INTERVIEWER: Okay we are continuing with our discussions, in this section I would you to take me through, how you counsel a client who is considering an injection contraception. I would like as much details as possible, so please break down the steps the steps you would take in counseling this client. To begin, how would you greet the client.

RESPONDENT: When a client comes you greet her, and then you ask her do you need any help and then she answers yes I do. Then you ask her, what help can I offer you? Then she replies, I want to take a family planning method. That’s when you explain to her all the options we have on family planning methods. Then when she chooses a method like let’s say Sayana, then we take her through the method how it’s administered.

INTERVIEWER: Alright fine, I think you also answered the next question where I wanted to know how you would counsel the client on the family planning method that she chooses.

RESPONDENT: Yaa

INTERVIEWER: Alright, now let’s say a client was only interested to take an injectable option, how would you explain to her on the options available

RESPONDENT: Mhhh

INTERVIEWER: For instance how many options would you counsel her and in what order.

RESPONDENT: We are able to present to her all the injectable methods and then you explain to her the good side of injectable methods and we also take her through the effects that the method may have.

INTERVIEWER: sure

RESPONDENT: But when you face some problems you should not be scared of anything just come and meet the Clinician for help.

INTERVIEWER: Mhh okay, so as you counsel the client do you use any material/job aid

RESPONDENT: Yes we do

INTERVIEWER: Like what for instance you are counseling the client on injectable method.

RESPONDENT: In the past we had posters that we were using, I have remembered something, we also had a screening card that we used to show the client whilst counseling them.

(20MINUTES AND 10 SECONDS)

INTERVIEWER: Eyaa

RESPONDENT: But now such aids are not available are missing

INTERVIEWER: Okay

RESPONDENT: Sure

Title: KK_HSA_2.docx

Descriptor Info:	District: Nkhotakota	Target Population: TP1 - Providers/HSAs	Respondent type: HSA	Aware of SI: Yes - had heard of SI before	Coding phase: 2. Double coded	Client marital status: N/A - provider	Client age group: N/A - provider	Time since trained: A year or more ago

Codes Applied:	P3.4 - Time to counsel/train, general	P3.3 - Counselling/ training approach, general

INTERVIEWER: How easy or difficult is it to train clients in DMPA-SC for self injection

RESPONDENT: aaaa mostly the most difficult part is the questions that the clients asks since they want to know more

INTERVIEWER: But in your experience do you find it difficult or not to counsel clients on self injection

RESPONDENT: no I don’t

INTERVIEWER: For you to counsel them

RESPONDENT No we don’t have difficulties

INTERVIEWER: I recall you said you said there are job aids that you use in your job.

RESPONDENT Yes

INTERVIEWER: How long on average does it take to train clients in DMPA-SC

RESPONDENT : The most painful thing is that women do not come in groups, you may find that there is only one who is present and you start counselling her, as you are about to finish you find out that there is also another client who wants counselling as well. Hence you have to start all over again

INTERVIEWER: Mhhh Mhh

RESPONDENT : And sometimes you just find yourselves doing short cuts

INTERVIEWER: Hehehe repeating the same process again and again.

RESPONDENT : yaah

INTERVIEWER: Do you feel you have enough time to conduct these trainings? And what about on busy days and you have more clients?

RESPONDENT: In that case one would not have enough time, unless when we are doing under five clinics then we target the mothers and counsel them on family planning methods.

INTERVIEWER: Alright

RESPONDENT: Sure doing under five clinics they get counselling in large numbers which is better than at the health centre.

INTERVIEWER: Alright

RESPONDENT : Yes, because it becomes so difficult when dealing with one client at a time than in a group. This is why nurses don’t like doing family planning counselling.

INTERVIEWER: Mhhhh

RESPONDENT: They say they can't be doing the same things again and again.

Title: KK_HSA_2.docx

Descriptor Info:	District: Nkhotakota	Target Population: TP1 - Providers/HSAs	Respondent type: HSA	Aware of SI: Yes - had heard of SI before	Coding phase: 2. Double coded	Client marital status: N/A - provider	Client age group: N/A - provider	Time since trained: A year or more ago

Codes Applied:	P3.3 - Counselling/ training approach, general

INTERVIEWER: We are about to wrap up our discussion, because we are left with two questions only. And now I would like to hear your perspective on why you think. On this topic we only have two questions; firstly in your own opinion, what are the main factors that would support a client to take up the self-injection option with DMPA-SC

RESPONDENT: Let me start with the previous question aaa one need to move with time

INTERVIEWER: Yes

RESPONDENT: In the first place we need to move with time, women should be able to learn on how others are failing on different issues. Hence you may advise the client by giving her an example on how others are doing badly in family planning.

INTERVIEWER: Mhhh

RESPONDENT: And if you don’t take family planning methods you will definitely be like them

INTERVIEWER: Yes

RESPONDENT: Firstly we have village banks happening around, you will find that your friends will be attending such gathering but you will not be able to do so. in addition you find that you will not be able to do developmental activities

INTERVIEWER: What role do you think the women's husband/partner plays in the decision?

RESPONDENT: Amhhh some men do encourage their wives, sometimes we counsel families by advising them on the dangers of bearing more children since any problem that happens to a woman will affect the whole family

INTERVIEWER: Mhhh

RESPONDENT: Which mean the whole family is affected

INTERVIEWER: Mhhh

RESPONDENT: And it very difficult develop your family so that people can admire you.

Title: ZA_HSA_3.docx

Descriptor Info:	District: Zomba	Target Population: TP1 - Providers/HSAs	Respondent type: HSA	Aware of SI: Yes - had heard of SI before	Coding phase: 1. Single coded	Client marital status: N/A - provider	Client age group: N/A - provider	Time since trained: A year or more ago

Codes Applied:	P3.3 - Counselling/ training approach, general	C2.1.2 - Reduced trips to facility	C5.1.4 - Timely injection due to not having to rely on external factors

Interviewer: In what ways do you encourage?

Respondent: We encourage them by teaching them that they can inject themselves at their free time at home and the clients also get excited because it is simpler for them than setting out a day to come to the hospital which is tiresome and takes the whole morning up to noon hence they can do other things instead of doing one thing.

Title: ZA_HSA_3.docx

Descriptor Info:	District: Zomba	Target Population: TP1 - Providers/HSAs	Respondent type: HSA	Aware of SI: Yes - had heard of SI before	Coding phase: 1. Single coded	Client marital status: N/A - provider	Client age group: N/A - provider	Time since trained: A year or more ago

Codes Applied:	P3.3 - Counselling/ training approach, general

Interviewer: Alright thank you very much. In this section I would like for you to talk me through how you counsel a client who is considering an injectable contraceptive. I would like as much detail as possible, so please break down the steps you would take in counselling this client. Firstly how do you greet the woman? For instance, if I were the one that came to you how would you greet me?

Time stamp: 19:42

Respondent: I would say you're welcome, sit down and feel free, mmmhhh if you have any problem be sure we will help you and if there are other things about family planning methods that you do not understand please ask and you will be helped, but how are you?

Interviewer: Thank you very much, I am fine, how are you?

Respondent: I am fine

Interviewer: After the greetings, how would you start telling me or counselling me about the method?

Respondent: I would ask the person how I may be of assistance to them and they would tell me that they want to start using a Sayana family planning method and so I would tell them that there are two ways first bring I inject them and the other being the way in which they inject themselves and if they choose the one where they have to inject themselves then I would take the Sayana to demonstrate how they can I next [inject] themselves

Title: ZA_HSA_3.docx

Descriptor Info:	District: Zomba	Target Population: TP1 - Providers/HSAs	Respondent type: HSA	Aware of SI: Yes - had heard of SI before	Coding phase: 1. Single coded	Client marital status: N/A - provider	Client age group: N/A - provider	Time since trained: A year or more ago

Codes Applied:	P3.3 - Counselling/ training approach, general

Interviewer: Alright alright, let's say a woman is interested in using the Sayana method, how would you explain to her the other methods available for her to use?

Respondent: Interested in what?

Interviewer: They were interested in using the self-injection method, how would you explain the other methods available for them to use? For instance, if I came to you looking for a self-injection method how would you tell me about the other family planning methods available for me to use?

Respondent: About the injection….

Interviewer: I want an injection and I have come to you; how can you explain to me about the other family planning methods available for me to use that are offered in this hospital? Respondent: Oooowooh…

Interviewer: But I want the one to do with injections

Respondent: Oowooh so I would tell them that there are other methods for family planning

Interviewer: Mmmmmh

Respondent: I would tell them that they can use pills or the man would get vasectomized or that they can use condoms and there are male and female condoms available. There are also long-lasting methods where you can choose whether you want to be using a family planning method for three or five years, all this can be explained to them.

Title: ZA_HSA_3.docx

Descriptor Info:	District: Zomba	Target Population: TP1 - Providers/HSAs	Respondent type: HSA	Aware of SI: Yes - had heard of SI before	Coding phase: 1. Single coded	Client marital status: N/A - provider	Client age group: N/A - provider	Time since trained: A year or more ago

Codes Applied:	P3.3 - Counselling/ training approach, general

Interviewer: Alright thank you very much, What information do you tell a person when telling them about the family planning method?

Respondent: Which things?

Interviewer: I have come to you looking for an injection, what things would you tell me when counselling me?

Respondent: Uhhumm I can start by telling them the importance of the injection like the use of this injection is ... I can also tell them how to store the medicine when they reach home

Interviewer: Alright thank you very much

Respondent: I would also ask them if they suffer from any diseases that may prevent them from taking the injection

Interviewer: Alright the way I asked the other question, I expected you to talk about the self-injection method because I asked how you would advise me if I wanted to use that method, did you talk about it?

Respondent: I understood as though you meant other ways available in addition to the self-injection method and that is why I was talking about the long-term methods.

Title: ZA_HSA_3.docx

Descriptor Info:	District: Zomba	Target Population: TP1 - Providers/HSAs	Respondent type: HSA	Aware of SI: Yes - had heard of SI before	Coding phase: 1. Single coded	Client marital status: N/A - provider	Client age group: N/A - provider	Time since trained: A year or more ago

Codes Applied:	P3.3 - Counselling/ training approach, general

Interviewer: Alright, are there any other questions that you would ask me?

Respondent: Yes, why did Sayana disappear as fast as it came?

Interviewer: No I meant the questions you would ask me if I came to you after failing to inject myself. What questions would you ask me?

Respondent: Yes, I would ask that...hmmm... How would you fail to inject after all I taught you everything and you also injected yourself while I was seeing in the first place so what changed?

Title: ZA_HSA_6.docx

Descriptor Info:	District: Zomba	Target Population: TP1 - Providers/HSAs	Respondent type: HSA	Aware of SI: Yes - had heard of SI before	Coding phase: 1. Single coded	Client marital status: N/A - provider	Client age group: N/A - provider	Time since trained:

Codes Applied:	P3.3 - Counselling/ training approach, general

Respondent: firstly, we greet them, hello women, welcome and be free. Today we would like to talk about Sayana

Interviewer: mmmm (showing that one is following the conversation)

Respondent: Sayana is one method of family planning just like other methods are

Interviewer: mmmm (showing that one is following the conversation)

Respondent: Sayana is available to every woman who is in childbearing age.

Interviewer: mmmm (showing that one is following the conversation)

Respondent: the main reason is to protect ourselves from unwanted pregnancy

Interviewer: yah yah

Respondent: so, because of this reason, when we get Sayana we check when you are supposed to come back again…

Interviewer: mmmm (showing that one is following the conversation)

Respondent: when you get Sayana you’re supposed to come back of such such a day.

Interviewer: mmmm (showing that one is following the conversation)

Respondent: what you should know is that when you are getting Sayana there are some things that can happen in your body since the bodies are different.

Interviewer: mmmm (showing that one is following the conversation)

Respondent: everyone has a different body from the next person.

Interviewer: ya yah

Respondent: we inject Sayana on the arm, this is how we inject it… but we are also encouraging women to access Sayana so that they can self-inject at home.

Interviewer: alright.

Respondent: those that will access this method today we will record you in our book so that we should know that in a certain village this particular person accessed Sayana on a particular day so that we whenever we meet to access the method, we should know how many of you are there in your community.

Interviewer: mmmm (showing that one is following the conversation)

Respondent: But Sayana does not protect you from sexually transmitted diseases, when you have accessed Sayana it does not mean that when you go out there you can just sleep with anyone else because you can contract HIV or even other STIs.

Interviewer: mmmm (showing that one is following the conversation)

Respondent: Sayana is aimed at protecting women from unwanted pregnancies until the time that you want to have a child.

Interviewer: mmmm (showing that one is following the conversation)

Respondent: but also, for women that are in childbearing age you should have good health

Interviewer: mmmm (showing that one is following the conversation)

Respondent: if you might have anu questions then you can ask, and we ca discuss right here.

Interviewer: mmmm (showing that one is following the conversation)

Respondent: those who might have questions concerning Sayana and will not feel comfortable to ask in a group you are allowed to meet the HSAs so that they can explain to you one on one.

Interviewer: Alright, you’ve told me that ask them if they have questions then they can comfortably ask you, do you ask these women any questions?

Respondent: when we have finished explaining, we ask them, what have we said Sayana is?

Interviewer: mmmm (showing that one is following the conversation)

Respondent: and what does Sayana do?

Interviewer: alright.

Respondent: how long does it take when someone has accessed Sayana?

Interviewer: mmmm (showing that one is following the conversation)

Respondent: we have explained the good thing about Sayana, what did we say? What about the problems that one can encounter after they use Sayana, what should they do?

Interviewer: yah

Respondent: and what are the problems?

Interviewer: alright.

Respondent: yes, so that they may follow what we have explained.

Interviewer: mmmm (showing that one is following the conversation)

Respondent: and also, why do we want to get the names of the people who have accessed Sayana.

Interviewer: mmmm (showing that one is following the conversation)

Respondent: yes, with the wish that the clients should have the information about the things we have explained from the beginning and also, they have a part on what they hear and also adding on to what we can, if they do not explain well, we still thank them because they have taken the part of answering.

Interviewer: mmmm (showing that one is following the conversation)

Respondent: then we correct the areas that the client got wrong so that we explain correctly so that the message should be correct.

Interviewer: alright.

Respondent: sure.

Interviewer: Let’s assume that a client is interested in accessing injectables, what options can you explain to the client that they have?

Respondent: ah firstly, we will explain about the good things about injectables and the problems that are there.

Interviewer: mmmm (showing that one is following the conversation)

Respondent: we will explain the good things about Sayana and f there could be problems

Interviewer: mmmm (showing that one is following the conversation)

Respondent: Sayana but mainly we can explain that they should access Sayana option

Interviewer: mmmm (showing that one is following the conversation)

Respondent: we will explain the good things about Sayana so that their curiosity should be on this program that we want for them

Interviewer: mmmm (showing that one is following the conversation)

Respondent: for example, when you access Sayana you have 12 months until you come back to the facility and meet someone.

Interviewer: yah yah

Respondent: you will be storing it yourself at home.

Interviewer: mmmm (showing that one is following the conversation)

Respondent: for reinjection days you will be remembering yourself and you will know who has all the equipment that you need. When we take the other injectable options, you will find that you have failed to come to the facility because of transportation issues.

Interviewer: mmmm (showing that one is following the conversation)

Respondent: so, for a person to receive the injectable option they will fail to come, so for them to have balancing on the benefits of the methods according to the way that we have explained to them.

Title: ZA_HSA_6.docx

Descriptor Info:	District: Zomba	Target Population: TP1 - Providers/HSAs	Respondent type: HSA	Aware of SI: Yes - had heard of SI before	Coding phase: 1. Single coded	Client marital status: N/A - provider	Client age group: N/A - provider	Time since trained:

Codes Applied:	P3.3 - Counselling/ training approach, general

Interviewer: Alright, would the way that you counsel a client differ if they have used injectables before?

Respondent: ah of course there could be a difference, when we balance Sayana

Interviewer: mmmm (showing that one is following the conversation)

Respondent: then there could be a difference, because the first injectable the one that we provide like Depo, is administered by the provider

Interviewer: yah

Respondent: for the other one then they can self-inject, so there is a difference

Interviewer: yaya

Respondent: another difference is that this other one is kept at the hospital by the providers, the other one can be kept by the client themselves, that’s another difference. The provider administered one has a specific place for injection for the community clinic then we inject on the arm, when they come to the facility then the nurses inject them on the thigh or maybe on the butt according to their technic

Interviewer: yah

Respondent: meaning that for those that received the injection at the facility and were injected on the thigh when they go to the village and are told about injecting on the arm they think like its different.

Title: ZA_HSA_6.docx

Descriptor Info:	District: Zomba	Target Population: TP1 - Providers/HSAs	Respondent type: HSA	Aware of SI: Yes - had heard of SI before	Coding phase: 1. Single coded	Client marital status: N/A - provider	Client age group: N/A - provider	Time since trained:

Codes Applied:	P3.3 - Counselling/ training approach, general	P3.4 - Time to counsel/train, general

Interviewer: Do you think that the length of the training on DMPA-SC can influence the decision to get DMPA-SC?

Respondent: Ah firstly, no

Interviewer: mmmm (showing that one is following the conversation)

Respondent: because we have seen other women they just come to the clinic, they did not have any program to access family planning but because of the training that they have heard, they have heard about the advantages in the training

Interviewer: yah

Respondent: you will see that they have now accessed the methods that same day, so we can see that there is a big advantage

Interviewer: mmmm (showing that one is following the conversation)

Respondent: when the training as been delivered to the women properly

Title: MZs_HSA_6.docx

Descriptor Info:	District: Mzimba South	Target Population: TP1 - Providers/HSAs	Respondent type: HSA	Aware of SI: Yes - had heard of SI before	Coding phase: 1. Single coded	Client marital status: N/A - provider	Client age group: N/A - provider	Time since trained: A year or more ago

Codes Applied:	P0 - General method mix / method provision

Interviewer: I would like to ask you questions concerning your work as a family planning provider. I would like to know what your day is like when you go to work at the facility and at the community

Respondent: when I am at my catchment area, I provide Depo and Sayana Press. Clients come at my house for Sayana Press.

Interviewer: what do you do as a provider. Whether it is at the facility or community I would like you to tell me from the time you start the clinic up to the end

Respondent: my day is good. Like I said clients come to my place. By the time I am starting the clinic they are already there. So firstly I wash my hands. Then I put on my mask in this era of covid-19. Then I welcome the clients. Then they start entering one by one. I counsel them on family planning methods. Then they make a decision on the method they prefer. When they choose Sayana we also counsel both the woman and the spouse on how to self-inject at home. We tell them on the dates and a site for injection on the arm, abdomen or thigh.

Interviewer: tell me until you finish the clinic

Respondent: clients still come for family planning. We counsel them on Sayana mode of action, how to self-inject, on storage to keep out of reach of children and in a closed bottle. We do this until the clinic is over. Even if the clients come in large numbers, we help them all.

Title: MZs_HSA_6.docx

Descriptor Info:	District: Mzimba South	Target Population: TP1 - Providers/HSAs	Respondent type: HSA	Aware of SI: Yes - had heard of SI before	Coding phase: 1. Single coded	Client marital status: N/A - provider	Client age group: N/A - provider	Time since trained: A year or more ago

Codes Applied:	P3.3 - Counselling/ training approach, general	P3.3.2 - Inclusion of practice opportunities for clients in training	P3.1.3 - IEC/demo materials for counselling/training	P4.1.1 - Follow up materials for client to take home

Interviewer: now I would like you to tell me how you would counsel a woman or an adolescent who has come to get injectable. I would like you to tell me from the time they come to the clinic until you finish with them. How do you greet a client when she has come to you?

Respondent: first we greet them; we introduce ourselves because many of them do not know us. we tell them that we are family planning providers. We tell them that whatever we discuss is kept confidential. Those who are free introduce themselves, where they are coming from and that they came to get family planning methods. Then we start counselling them using the counselling cards. Even if they choose Sayana we teach them on all methods. Then we get our checklist and ask the client questions. If she gets Sayana we wash our hands first. We counsel them on how to self-inject. Then we wash hands, put on a mask, then you are ready. We get the vial and show them. We counsel them on the mode of action of Sayana. We also give then dates for the next dose. Then we start demonstrating the procedure. We shake the vial slowly and touch it on the neck and close it. The closing opens the contents. Then we show them the area to inject on the abdomen, arm or thigh to pinch the skin and then self-Inject all the contents. We advise them not to rub on the area. They follow this procedure after us. we then give them some for home and a calendar. We circle the dates they are supposed to self-inject on the next doses.

Interviewer: how many methods would you counsel this woman

Respondent: come again

Interviewer: how many methods of injectable would you counsel this woman

Respondent: when she comes to me that she wants injectable, I would tell her that we have another method Sayana which a woman self-inject. So a woman will make a decision on which method she would want like self-injection

Interviewer: what questions would you ask during the counselling process

Respondent: have you ever had migraine headache? High blood pressure? When was your last menstrual period? Are you suspecting that you are pregnant? We ask many questions which are on the checklist.

Interviewer: apart from Sayana, which other method are you going to counsel this woman

Respondent: on family planning

Interviewer: yes, on the injectable methods

Respondent: I will counsel her on Depo IM which is given by the provider and Sayana for self-injection. So she will choose

Interviewer: after she chooses

Respondent: if she chooses let’s say Sayana for self-injection then I will counsel her on how to self-inject then supply Sayana with a calendar to take home. If she came with a spouse, we counsel them together.

Interviewer: what do you use when counselling

Respondent: like I have already said we use a counselling card

Interviewer: do you use anything else

Respondent: no, just a counselling card

Title: KK_HSA_6.docx

Descriptor Info:	District: Nkhotakota	Target Population: TP1 - Providers/HSAs	Respondent type: HSA	Aware of SI: Yes - had heard of SI before	Coding phase: 1. Single coded	Client marital status: N/A - provider	Client age group: N/A - provider	Time since trained: A year or more ago

Codes Applied:	P3.3 - Counselling/ training approach, general

RESPONDENT: We were trained on different types of contraceptives. The injection and another one is the one that has just been introduced which women are self-injecting. When a woman approaches us, we ask her questions depending on the method they want. We explain about Depo and Sayana and the woman makes a choice. We also explain both the good and the bad of the methods because all methods have good and bad sides.

INTERVIEWER: yes, mmmh.

RESPONDENT: That’s how drugs are in the body.

Title: KK_HSA_6.docx

Descriptor Info:	District: Nkhotakota	Target Population: TP1 - Providers/HSAs	Respondent type: HSA	Aware of SI: Yes - had heard of SI before	Coding phase: 1. Single coded	Client marital status: N/A - provider	Client age group: N/A - provider	Time since trained: A year or more ago

Codes Applied:	P3.3 - Counselling/ training approach, general

INTERVIEWER: Are there any methods that you do not usually counsel clients about?

RESPONDENT: Aaaah, as an HSA, I don’t have a method that I can say I will not counsel a client on. When a person wants contraceptives, we counsel her on all the methods and she makes a choice on the method she will use.

INTERVIEWER: okay.

RESPONDENT: yeah.

Title: KK_HSA_6.docx

Descriptor Info:	District: Nkhotakota	Target Population: TP1 - Providers/HSAs	Respondent type: HSA	Aware of SI: Yes - had heard of SI before	Coding phase: 1. Single coded	Client marital status: N/A - provider	Client age group: N/A - provider	Time since trained: A year or more ago

Codes Applied:	P3.3 - Counselling/ training approach, general

INTERVIEWER: We are proceeding. In this section, I would like for you to explain to me in detail how you counsel a client who is considering an injectable. you have to explain in detail. I will ask you questions, and you have to break down the steps you take in counselling, clearly.

RESPONDENT: alright.

INTERVIEWER: To begin with, when a woman arrives, how do you greet this client?

RESPONDENT: Okay. When women come to our under-five clinic, where my work also includes family planning, we combine family planning and under-five. We have medicine we distribute in the community for under-five. So we combine those who have come for CCM and family planning. The first thing is for you and the client to know each other. For example, you have come to me, you mention your name and I will also do the same. And then ask politely why she has visited, and she will explain what she wants. You assure her of privacy and tell her to feel free. Then she will tell you that she wants contraceptives. We explain to her about all the contraceptive methods that are available and also the ones that we HSAs provide. And the client might choose Sayana. And then we now counsel her on the method she has chosen including its advantage and disadvantages. But we cannot focus on the bad side because she will not choose the method, but we should explain to her the advantages of the method. Then she understood and makes her choice. And then we train her on this method.

INTERVIEWER: okay.

RESPONDENT: yea.

INTERVIEWER: Alright. So you normally ask them questions, right?

RESPONDENT: mmmh[yes]

INTERVIEWER: What type of questions do you ask?

RESPONDENT: We can ask questions. Why have you chosen this method? some say that my mother used this method back then and she did not face any problem up to the time she stopped using the contraceptive. Oh ok, she did not tell you any challenges? She did not but some were saying they have heavy period and an irregular menstrual circle. And we explain to her that this normally happens in the first three months gets better from 4 months. The client might also explain that she wants the contraceptive because she does not want to have a lot of children. And she wants to have a child spacing of 4 to 5 years. This is some of the things that women explain.

Title: KK_HSA_6.docx

Descriptor Info:	District: Nkhotakota	Target Population: TP1 - Providers/HSAs	Respondent type: HSA	Aware of SI: Yes - had heard of SI before	Coding phase: 1. Single coded	Client marital status: N/A - provider	Client age group: N/A - provider	Time since trained: A year or more ago

Codes Applied:	P3.3 - Counselling/ training approach, general

INTERVIEWER: Alright. Let us assume that a client was only interested in taking up an injectable option. For example, I am interested in an injectable. How would you explain to her the options she has?

RESPONDENT: oh, she has directly mentioned an injectable?

INTERVIEWER: mmmhu. How will you explain the options she has?

RESPONDENT: Yea. I will ask her why she has opted for an injectable when there are a lot of contraceptive methods. She will explain her reasons because women have different reasons. She might say I have chosen this provider injected. I will tell her it is a good method but I will also explain to her about Sayana and tell her that the Sayana method is sometimes good for example in the rainy season and the other seasons. Maybe the HSA will travel on your visit day, but when you have Sayana you will self-inject. We explain both the method she has chosen and the one she has not. We also explain the advantages of the method she has not chosen.

INTERVIEWER: mmmhu. You have said you will explain…

RESPONDENT: the method she wants; we will assist her.

INTERVIEWER: Depo…

RESPONDENT: it is good, and this is how it works…

INTERVIEWER: Sayana.

RESPONDENT: It is good because a client will be self-injecting at home. When it rains and the rivers have overflooded, you will have maybe 2 doses to keep at home. But the methods are the same.

INTERVIEWER: mmmhu.

RESPONDENT: yes.

INTERVIEWER: What is the first method you explain to her between the two?

RESPONDENT: Aaah, when a client comes, we tell her all the contraceptive methods. Whether there are 6 or 20 methods. We explain everything she needs to know about all the methods including the side effects for example irregular menstrual circle in the first three months. And it is up to you to choose the method which is appealing to you according to its benefits. And then she will choose for example an injectable. Then we explain that the injection we the providers will do it, but there is also another injectable for self-injection

INTERVIEWER: okay.

RESPONDENT: yea.

INTERVIEWER: there are two types of injections, right?

RESPONDENT: two, Sayana and Depo Provera.

INTERVIEWER: in what order do you counsel her on the injectable?

RESPONDENT: I have already explained that there are a lot of contraceptive methods and you cannot just go direct on two methods.

INTERVIEWER: Aaah we are…the question I am asking you is, I am asking you on these two methods. A client has come and said she wants an injection and you are supposed to explain to her about the other methods.

RESPONDENT: …explain... she wants these two?

INTERVIEWER: Which one will you first explain to her?

RESPONDENT: I will first start with the one she has chosen. I will ask her why she has chosen me to inject her. After giving her explanation, I will tell her there is also another method called Sayana Press and it works like this. She can change her mind to choose the second method according to how I have conducted my counselling.

INTERVIEWER: ok

RESPONDENT: yea

Title: KK_HSA_6.docx

Descriptor Info:	District: Nkhotakota	Target Population: TP1 - Providers/HSAs	Respondent type: HSA	Aware of SI: Yes - had heard of SI before	Coding phase: 1. Single coded	Client marital status: N/A - provider	Client age group: N/A - provider	Time since trained: A year or more ago

Codes Applied:	P3.3 - Counselling/ training approach, general

INTERVIEWER: You have trained a woman and you have given her 3 doses. On her appointment date, she has returned to you without completed one injection for some reasons known to her. What would your reaction be?

RESPONDENT: I will ask her why she did not do self-injection with the drug and she wants some more. Maybe there might be a reason. Maybe the husband told her not to or she was afraid. Some they can self-inject and then after some time, they may be afraid again. I am afraid to self-inject because where I injected, there is a hole. We just train her again until she will self-inject again. because Sayana is for self-injection.

Title: ZA_HSA_1.docx

Descriptor Info:	District: Zomba	Target Population: TP1 - Providers/HSAs	Respondent type: HSA	Aware of SI: Yes - had heard of SI before	Coding phase: 1. Single coded	Client marital status: N/A - provider	Client age group: N/A - provider	Time since trained: Less than a year ago

Codes Applied:	P3.3 - Counselling/ training approach, general

Interviewer: Alright alright thank you very much. Are there any methods that you do not usually counsel clients about?

Respondent: No, there is no method that we do not counsel about.

Title: ZA_HSA_1.docx

Descriptor Info:	District: Zomba	Target Population: TP1 - Providers/HSAs	Respondent type: HSA	Aware of SI: Yes - had heard of SI before	Coding phase: 1. Single coded	Client marital status: N/A - provider	Client age group: N/A - provider	Time since trained: Less than a year ago

Codes Applied:	P3.3 - Counselling/ training approach, general

Interviewer: Thank you very much, has your opinion towards self-injection changed overtime?

Respondent: Opinion?

Interviewer: Your opinion

Respondent: No it has changed

Interviewer: Umm

Respondent: Those with fears need to be counselled so that they can be injecting themselves at home

Title: ZA_HSA_1.docx

Descriptor Info:	District: Zomba	Target Population: TP1 - Providers/HSAs	Respondent type: HSA	Aware of SI: Yes - had heard of SI before	Coding phase: 1. Single coded	Client marital status: N/A - provider	Client age group: N/A - provider	Time since trained: Less than a year ago

Codes Applied:	P3.3 - Counselling/ training approach, general

Interviewer: What way do you support them?

Respondent: We encourage them that when their due date arrives, they won’t need to travel a long distance. They will do it at home

Interviewer: Yes

Respondent: When their day arrives, they will self-inject at home. They won’t lose time coming here at the hospital. Further to this it doesn’t take long

Title: ZA_HSA_1.docx

Descriptor Info:	District: Zomba	Target Population: TP1 - Providers/HSAs	Respondent type: HSA	Aware of SI: Yes - had heard of SI before	Coding phase: 1. Single coded	Client marital status: N/A - provider	Client age group: N/A - provider	Time since trained: Less than a year ago

Codes Applied:	P3.3 - Counselling/ training approach, general

Interviewer: Alright alright, we are now going into another section. In this section I would like for you to talk me through how you counsel a client who is considering an injectable contraceptive. I would like as much detail as possible so please break down the steps you would take in counselling a client. To begin, how would you greet the client?

Respondent: Who has come?

Interviewer: Yes

Respondent: We welcome and take them to the office; we cannot counsel them in public

Interviewer: Umm

Respondent: We then greet them and tell them that they are welcome

Interviewer: Am sorry you have an office in the community?

Respondent: Oh, in the community (giggles)

Interviewer: No, it’s just in general how you can counsel a person. I have come to you looking for family planning methods, how would you counsel me?

Respondent: When you come we will ask you of the method you want to get

Interviewer: Ummmhh

Respondent: For instance, you might tell us that you want the injection or pills. You will get the method that you want be it injection or pills, this will be in accordance with what you want.

Interviewer: Alright, how would you start counselling someone?

Respondent: Counselling?

Interviewer: As earlier explained I have come to you wanting family planning, how would you counsel me?

Respondent: (Giggles) I won’t be able to answer this

Interviewer: In addition to the counselling, you offer before they start family planning, the clients have come

Respondent: Yes

Interviewer: At the family planning clinic, before giving them the method

Respondent: Yes

Interviewer: What they have come for instance they have chosen

Respondent: Yes

Interviewer: What do you do?

Respondent: Oh, ok alright, we counsel then in accordance with the method they want. That when they get this method (noise in the background). For instance, with Sayana, others menstruate while others don’t menstruate with it. Others take time to get pregnant, it all depends on the person. Others say that they stopped using it last month but got pregnant the following month. We also counsel them that Sayana only protects against unplanned pregnancy, it does not offer protection against sexually transmitted diseases

Interviewer: Umm

Respondent: This is the counselling we would offer

Interviewer: Are there any questions you would ask when offering counselling?

Respondent: Questions?

Interviewer: Mmmh

Respondent: No, we just inform them that if there is continuous menstruation they should come to the hospital

Interviewer: Mmmh

Respondent: Eeh

Interviewer: Alright, is there any question that you would ask them?

Respondent: Aaah………. i have never asked any question

Interviewer: We are moving forward, assuming a woman was only interested in taking up an injectable option, how would you explain their options?

Respondent: What options?

Interviewer: Different methods that are available

Respondent: Relating to family planning

Interviewer: Mmmh

Respondent: (Giggles)

Interviewer: I have come (respondent giggles) I want an injection

Respondent: Yes

Interviewer: Would you explain about the other methods that are available

Interviewer 2: When a woman comes to the hospital looking for family planning methods

Time stamp: 17:11

Respondent: Mmmh

Interviewer: They are not told only about 1 method; you teach them different methods.

Respondent: Mmmh. Oh ok (giggles) This question will be hard for me to respond in terms of family planning I was only trained in Sayana

Interviewer: Mmmh

Respondent: I haven’t been trained in the others

Interviewer: Mmmh

Respondent: Yes

Interviewer: Even other methods (very windy and blurry)

Respondent: We tell them that there is loop

Interviewer: Mmmh

Respondent: There is Jadelle

Interviewer: Mmmh

Respondent: Yes

Interviewer: Mmmmh

Respondent: There are pills, we still tell though I am not able to explain in detail as I don’t know them

Title: MZs_HSA_2.docx

Descriptor Info:	District: Mzimba South	Target Population: TP1 - Providers/HSAs	Respondent type: HSA	Aware of SI: Yes - had heard of SI before	Coding phase: 1. Single coded	Client marital status: N/A - provider	Client age group: N/A - provider	Time since trained: A year or more ago

Codes Applied:	P3.3 - Counselling/ training approach, general

Interviewer: what is your day like when you come to the health facility as well as at the community when you go for work. You can start with the health facility then at the community. Tell me what you do

Respondent: when we come here, we first meet the client we teach them about all family planning methods that are certified in Malawi that we have in a booklet which we use when teaching. Then the client makes an informed decision to choose the type of the method which she wants. The same in our community we start with teaching them on family planning methods that are certified in Malawi then they choose the one they want.

Title: MZs_HSA_2.docx

Descriptor Info:	District: Mzimba South	Target Population: TP1 - Providers/HSAs	Respondent type: HSA	Aware of SI: Yes - had heard of SI before	Coding phase: 1. Single coded	Client marital status: N/A - provider	Client age group: N/A - provider	Time since trained: A year or more ago

Codes Applied:	P3.3 - Counselling/ training approach, general

Interviewer: is there any other methods that you do not teach clients

Respondent: no we teach all methods

Interviewer: you mention all?

Respondent: we tell them all. If they opt for other methods like jadelle which we do not provide we refer them to the clinic because we do not provide at the community

Title: MZs_HSA_2.docx

Descriptor Info:	District: Mzimba South	Target Population: TP1 - Providers/HSAs	Respondent type: HSA	Aware of SI: Yes - had heard of SI before	Coding phase: 1. Single coded	Client marital status: N/A - provider	Client age group: N/A - provider	Time since trained: A year or more ago

Codes Applied:	P2.3 - Provider training content	P3.3 - Counselling/ training approach, general

Interviewer: now I would like to ask you question on the training you received on Sayana Press. aaaah (thinking out loud) would you tell me about the training you receive on Sayana Press

Respondent: we were taught that firstly the client should wash hands with soap. Then they should open the packet. After opening the packet of Sayana then they should shake it. Then they squeeze it on the neck. They told us this is the neck (demonstrating). The other hand touching the upper part. This push the needle into the contents. Then they prepare the area to inject if its self-injection. If it’s me administering I prepare and inject on the arm. After administering aaaah (thinking out loud) I dispose the needle in a safety box but when it’s at home we tell them to dispose in a tin and keep it away from the reach of children.

Interviewer: is there anything you want to add?

Respondent: no

Title: MZs_HSA_2.docx

Descriptor Info:	District: Mzimba South	Target Population: TP1 - Providers/HSAs	Respondent type: HSA	Aware of SI: Yes - had heard of SI before	Coding phase: 1. Single coded	Client marital status: N/A - provider	Client age group: N/A - provider	Time since trained: A year or more ago

Codes Applied:	P3.3 - Counselling/ training approach, general

Interviewer: now we have come to another part on counselling of a woman who is intending to get injectable family planning methods. Would you take me through the procedure you follow from reception and how you counsel her? Firstly how would you greet a woman who has come to get injectable family planning method

Respondent: we welcome the woman and greet her. I tell her my name and cadre. If she is open, she also introduces herself and tell me why she has visited. If she chooses, we teach her according to the teaching aid. We tell her that choosing family planning method is her personal choice which she made from home before seeing a provider. We were told to teach on all family planning methods that are certified. We teach on all methods and then she chooses. If she chooses Sayana, we then teach in detail on Sayana. We have forms which we use for teaching. We also give her a form for reference during the teaching. We start teaching from the first step whereby a woman is supposed to wash hands for cleanliness before doing anything. Then we give her Sayana and show her where we open and tell her where we hold. After that we tell her to shake (someone entered the room) after opening and holding it and shaking the contents then we tell her to press so that the needle sinks into the contents. Those who self-inject do that on the stomach where there are more fats or on the thighs. We teach them how to do it. After injecting we tell her to dispose the used needle in the sharp box. But when she self-injects at home, she has to put used needles in a tin. When she finishes all the doses to bring the tin for her to get another dosage of Sayana.

Interviewer: I would like to ask what type of counselling would you give to a woman who has chosen injectable method of family planning on such methods

Respondent: we ask if she wants Sayana or Depo

Interviewer: I would like to hear from you what you would tell her

Respondent: we educate her on the benefits of injectable. We also tell her that Sayana and Depo are similar. We tell her that when someone is using these methods it delays ovulation, it makes the lining of the uterus thin, it also makes the mucus thick; that’s how they work. We tell her that the difference between these two is that Depo is given by the provider whilst Sayana she can self-inject at home. We tell her that if she experiences excessive bleeding she should come to the facility for management. We advise her that if she opt for Depo, she can get STIs if she has multiple sexual partners. Family planning methods do not protect from STIs.

Interviewer: what sort of questions would you ask her

Respondent: I would ask if she is following when teaching her. So she would repeat what I told her that way I know we are together
