# Supplementary material for: Health care provider and client experiences of counselling on depot medroxyprogesterone acetate subcutaneous (DMPA-SC) for self-injection in Malawi
Source: PLOS Glob Public Health. 2023 Nov 30;3(11):e0002057. doi: 10.1371/journal.pgph.0002057 (PMC10688740; doi:10.1371/journal.pgph.0002057)
Supplement: S1 Data — (ZIP) [file pgph.0002057.s001.zip › S2/P3.3.1 diff in couns by client group.doc]

Title: ZA_HSA_2.docx

Descriptor Info:	District: Zomba	Target Population: TP1 - Providers/HSAs	Respondent type: HSA	Aware of SI: Yes - had heard of SI before	Coding phase: 3. Triple coded	Client marital status: N/A - provider	Client age group: N/A - provider	Time since trained: Less than a year ago

Codes Applied:	P3.3.1 - Differences in counselling/training approach by population group

Respondent: Ah, we just encourage the client, but if the client is a new user of family planning, we give the counselling…

Interviewer: mmmm (Showing that one is following the conversation)

Respondent: We counsel the client everything about family planning and the methods.

Interviewer: mmhh (Showing that one is following the conversation)

Respondent: yes, even though we don’t provide Norplant.

Interviewer: mmm (Showing that one is following the conversation)

Respondent: But still, we counsel them on that highlighting to the client about the advantages of the methods…

Interviewer: Mmmm (showing that one is following the conversation)

Respondent: If they have chosen that ah for me, I feel that I should chose Norplant…

Interviewer: mmm (Showing that one is following the conversation)

Respondent: They have all the freedom,

Title: ZA_HSA_2.docx

Descriptor Info:	District: Zomba	Target Population: TP1 - Providers/HSAs	Respondent type: HSA	Aware of SI: Yes - had heard of SI before	Coding phase: 3. Triple coded	Client marital status: N/A - provider	Client age group: N/A - provider	Time since trained: Less than a year ago

Codes Applied:	P3.3.1 - Differences in counselling/training approach by population group

Interviewer: Does the counselling session differ in any way based on whether a client is a current user or new user of the injectable contraceptive?

Respondent: eh

Interviewer: But also, for a client whom in the past was already using this method of family planning, dos your counselling differ in any way?

Respondent: Yes, it’s quite different.

Interviewer: mmhh (Showing that one is following the conversation)

Respondent: Because the client that is a current user already knows the information, we give in the counselling session; we just remind them…

Interviewer: mmmhhh (Showing that one is following the conversation)

Respondent: …on some things that they might have forgotten…

Interviewer: mmmhhh (showing agreement)

Respondent: We just remind them that this is how we do it

Interviewer: mmm (Showing agreement)

Respondent: Yes, then we give the, we give them the hep that they want. If they have a question, they are also able to ask. But for the new client we take time with them, yes because for them the commodity is a new thing, yeah…

Interviewer: mmmhh (Showing agreement)

Respondent: So that it sticks to their mind, they should understand it very well, we need to take more time with them…

Title: ZA_HSA_2.docx

Descriptor Info:	District: Zomba	Target Population: TP1 - Providers/HSAs	Respondent type: HSA	Aware of SI: Yes - had heard of SI before	Coding phase: 3. Triple coded	Client marital status: N/A - provider	Client age group: N/A - provider	Time since trained: Less than a year ago

Codes Applied:	P3.3.1 - Differences in counselling/training approach by population group

Interviewer: Does the counselling that you provide differ compared to a client who is older, does the counselling differ in any way?

Respondent: Ah, it is the same.

Interviewer: Alright.

Respondent: Yes, because the way that the methods are used both for an adolescent and an older woman it is just the same.

Interviewer: Alright.

Respondent: Yes.

Interviewer: Alright.

Respondent: Sure.

Interviewer: Does it differ the way that you counsel when you are meeting the clients in the community compared to when you are meeting them here at the facility?

Respondent: No, it is the same.

Title: ZA_HSA_2.docx

Descriptor Info:	District: Zomba	Target Population: TP1 - Providers/HSAs	Respondent type: HSA	Aware of SI: Yes - had heard of SI before	Coding phase: 3. Triple coded	Client marital status: N/A - provider	Client age group: N/A - provider	Time since trained: Less than a year ago

Codes Applied:	P3.4 - Time to counsel/train, general	P3.3.1 - Differences in counselling/training approach by population group	P3.2.3 - Complexity/difficulty of training clients in SI	A6 - Adol Perspectives/preferences for DMPA-SC SI

Interviewer: Alright, does the time that you spend training a client in DMPA-SC differ from one client to the next?

Respondent: Yes, they can be different.

Interviewer: mmm (showing that one is following the conversation)

Respondent: Because understanding is different. There are some people who can easily understand and some who take time to understand, they keep asking questions so that they can fully understand. So, the other ones we take more time with them even 45 minutes which I have mentioned may not be enough, yes, because the client wants to know more so we try to be with the for more time, but most of the times the time is between 30 to 45 minutes…

Interviewer: Alright.

Respondent: Sure, sure.

Interviewer: Alright, what about for adolescents.?

Respondent: mmm (showing that they are following the conversation)

Interviewer: Does the time that you take to train them differ compared to older women?

Respondent: Yes, it is possible like that, it can be like that, yes because girls, it is difficult that they should understand faster yeah,

Interviewer: mmm (showing that one is following the conversation)

Respondent: Compared to an older woman, because older women already know the things, and maybe they have already heard the things from somebody else, but mostly the adolescents don’t know most of the information relating to family planning…

Interviewer: Alright.

Respondent: Yes, if we are to do shortcut for those ones it is difficult…

Interviewer: mmm (showing that one is following the conversation)

Respondent: …if the find us we are required to be with them for the appropriate length of time to train them properly so that they appreciate the things that we have trained them…

Interviewer: mmm (showing that one is following the conversation)

Respondent: The we give them the family planning methods…

Title: KK_HSA_1.docx

Descriptor Info:	District: Nkhotakota	Target Population: TP1 - Providers/HSAs	Respondent type: HSA	Aware of SI: Yes - had heard of SI before	Coding phase: 2. Double coded	Client marital status: N/A - provider	Client age group: N/A - provider	Time since trained: A year or more ago

Codes Applied:	P3.3.1 - Differences in counselling/training approach by population group

Interviewer: Mmmhu. A woman has come, she has used an injectable before. she has come back and she wants an injectable, the way you counsel a client does…do you…does it differ or it’s the same?

(30:05)

Respondent: It’s the same. It is the same in a way that for a woman who has used before right? Let’s assume that 3 years ago, she got an injectable, and then she…she got pregnant and she gave birth. In…in…in the third year, she has come back and said the child has grown its now 6 weeks and she wants contraceptives. You ask her, have you ever used this method? Yes. And when the woman tells you she has used the method before, so you ask her what she knows about the method…what do you know about the injectable? You see? so the woman if she still remembers she says there is this this and that. And what are the side effects, you see? So as a provider, you just tell her the areas the person has what, forgotten.

Interviewer: mmmh.

Respondent: You have to tell her or else add this this and that she is required to follow as she has explained. And then you are now on the same level with the woman.

Interviewer: mmmh.

Respondent: yes.

Interviewer: What about if its an adolescent?

Respondent: If its an adolescent?

Interviewer: mmmh.

Respondent: Eeeh, I should say that on all methods, we still say that its confidential because the adolescents fail to come because maybe providers are not confidential. Let us an 18, 17-year-old wants contraceptives, adolescents come. Firstly, we encourage her that these methods are confidential, yes so that the person should be free. And then you tell her all the methods. Whether she will want condoms or injectables, yes. If it’s an adolescent, we tell her you have been injected, you will not get pregnant, but you will be infected with diseases. We also say this during counselling. Or the whole counselling we use that. So when you use condom and injectable at the same time, it is like double what, protection. You will avoid pregnancy; you will not be infected. And then we have an increased number of adolescents.

Interviewer: mmmh.

Respondent: Yes.

Title: KK_HSA_1.docx

Descriptor Info:	District: Nkhotakota	Target Population: TP1 - Providers/HSAs	Respondent type: HSA	Aware of SI: Yes - had heard of SI before	Coding phase: 2. Double coded	Client marital status: N/A - provider	Client age group: N/A - provider	Time since trained: A year or more ago

Codes Applied:	P3.3.1 - Differences in counselling/training approach by population group	A6.1.1 - Reasons for potential interest in SI

Interviewer: How about the adolescents what…what…what…what problems do they face when learning self-injection?

Respondent: The adolescents for them to learn it is difficult or not because the adolescents are afraid. They are afraid that if I go to that woman or to that HSA, they will report me to who…to my parents. You see? But we tell them that when we assist you here whether it is contraceptives or condoms, it is confidential because it helps your life; you must not be afraid. You see, they should come and we should do the whole counselling process, the right one on how Sayana press works, its importance blablabla. But they have no time for that, they just want a quick one.

Interviewer: mmmhu.

Respondent: yes.

Interviewer: How about the adolescents who are married?

Respondent: It is possible, we counsel them

Title: KK_HSA_1.docx

Descriptor Info:	District: Nkhotakota	Target Population: TP1 - Providers/HSAs	Respondent type: HSA	Aware of SI: Yes - had heard of SI before	Coding phase: 2. Double coded	Client marital status: N/A - provider	Client age group: N/A - provider	Time since trained: A year or more ago

Codes Applied:	C0.3 - Client previous use/experience of any FP method	P3.3.1 - Differences in counselling/training approach by population group

Interviewer: In your view, do you think having previous knowledge of on the use of Sayana. Does it help a woman to choose or not choose this method?

Respondent: No a woman has a right and they choose. Right now, women are free to choose that you should not inject me the old one, give me Sayana.

Interviewer: okay.

Respondent: Yes, they are speaking out.

Interviewer: Alright.

Respondent: Mmmmhu.

Title: ZA_HSA_5.docx

Descriptor Info:	District: Zomba	Target Population: TP1 - Providers/HSAs	Respondent type: HSA	Aware of SI: Yes - had heard of SI before	Coding phase: 2. Double coded	Client marital status: N/A - provider	Client age group: N/A - provider	Time since trained: Less than a year ago

Codes Applied:	P3.3.1 - Differences in counselling/training approach by population group

Interviewer: Alright, would the way you counsel a client on injectables differ if they had used injectables in the past?

Respondent: If they have not used?

Interviewer: Yes

Respondent: That they are new users?

Interviewer: Yes, that they are new users

Respondent: They were using a different method and they want another method, it cannot differ because the way that they told us about injectables is the same information, if we make them differ then we are not doing a good job then it means we are telling the women lies, we should tell her the truth that this is how injectables work not different information, we cannot do that

Interviewer: Alright ah what about if it’s an adolescent compared to an older woman? Would the counselling differ between these two groups?

Respondent: Eh yes it can differ, for older women it means they have passed through a lot, and they know well maybe they have already have children, but for the adolescent it could be different because we will tell her about the pros and cons so that her future is bright and she should be able to continue with school, for the older women we cannot tell them that.

Interviewer: Alright, can the counselling differ if you are talking to a married woman or an unmarried woman?

Respondent: Yes, in the areas that we work in most of the women even though we are giving them family planning they are not married but the counselling is just the same with those that are married, for those who are not married you just tell them that they should protect themselves from pregnancy, but they are not protecting themselves from STIs. So, we need to tell that woman that once you have accessed the contraceptives you also need to protect yourself from STIs you are required to get condoms to protect yourself for you might be at risk of catching STIs

Interviewer: Alright

Respondent: For a married woman she is someone who as a husband we cannot tell her about STIs, yes, she needs to be safe so we also say a bit about that but we know she has a husband, for the unmarried one it is different.

Interviewer: Alright, does the counselling differ when you are meeting clients here at the facility or in the community?

Respondent: Here and the community?

Interviewer: Yes

Respondent: Ah no there is no difference

Interviewer: Alright

Respondent: It is supposed to be the same because if they are being told a different thing here and in the communities then that’s where the questions come in

Interviewer: Mmm (showing that one is following the conversation)

Respondent: I remember sometime back they came

Interviewer: Mmm (showing that one is following the conversation)

Respondent: On the same issue of Depo

Interviewer: Mmm (showing that one is following the conversation)

Respondent: They heard about a certain hospital which I will not mention

Interviewer: Yes

Respondent: At the hospital they were told that when you inject Depo 5 times you should stop using it yeah

Interviewer: Mmm (showing that one is following the conversation)

Respondent: But for us we had told them to inject themselves and protect themselves from pregnancy, but they said but you told us that we should stop, so you see its different.

Interviewer: It’s different

Respondent: It’s different, so there is need that the information they are giving out here should be similar to the we are giving out in the communities. So, there should be no difference.

Title: KK_HSA_5.docx

Descriptor Info:	District: Nkhotakota	Target Population: TP1 - Providers/HSAs	Respondent type: HSA	Aware of SI: Yes - had heard of SI before	Coding phase: 1. Single coded	Client marital status: N/A - provider	Client age group: N/A - provider	Time since trained: A year or more ago

Codes Applied:	P3.3.1 - Differences in counselling/training approach by population group

INTERVIEWER: We are proceeding with our conversation, would the way you counsel a client on injectable differ if they had used injectables in the past?

RESPONDENT: There may be a very slight different in that the other injectable we may talk of places they are supposed to inject, and also the type of injection used and the size of the injection. But in terms of how the injection works and the effectiveness we tell them it’s just the same ( Depo IM and Sayana Press ).

INTERVIEWER: What about if they were an adolescent, were married / unmarried, if they wanted to delay a first pregnancy would the advice differ from the one you would give to an adult and an adolescent?

RESPONDENT: The counselling differs a little bit (clearing throat) The different comes in where for an adult we counsel them mostly on family planning methods that are getting towards cessation of fertility, this depends on the age of our client. Whilst for an adolescent we counsel them more on family planning methods that will just lead to a delay in getting pregnancy like injectables for instance Jadelle. This is done so to ensure that an adolescent should have more information on contraceptives that only delays pregnancy and helps the client to do child spacing. So that she should avoid unwanted pregnancy. She should only have a child after making a sound decision to do so, but not doing it abruptly.

Time stamp: (30minutes 45 seconds)

INTERVIEWER: Alright

RESPONDENT: Sure

INTERVIEWER: Thank you much. What if you were seeing them in the community versus seeing the at a facility would the counselling differ?

RESPONDENT: It differs a little bit because in the community meeting places is a challenge

INTERVIEWER: Ok

RESPONDENT: We do not have conducive environment

INTERVIEWER: Ok

RESPONDENT: We normally meet in churches where we do conduct under-five clinics, so we have to wait to finish the clinic and then start our counselling session. We have to make sure that we create private site for our clients hence we try our best to ensure that all other people are gone.

INTERVIEWER: Ok

RESPONDENT: It is a problem to create privacy in the community. For serious clients they just invite us in their homes, but for the rest of members we do during under-five clinic and village clinics that is so tough. Whilst at a facility we normally have privacy.

Title: KK_HSA_5.docx

Descriptor Info:	District: Nkhotakota	Target Population: TP1 - Providers/HSAs	Respondent type: HSA	Aware of SI: Yes - had heard of SI before	Coding phase: 1. Single coded	Client marital status: N/A - provider	Client age group: N/A - provider	Time since trained: A year or more ago

Codes Applied:	P3.1.5 - Ease of training clients in SI	P3.3.1 - Differences in counselling/training approach by population group

INTERVIEWER: Do you think having previous experience with injectables makes a woman more or less likely to consider self-injection?

RESPONDENT: Having previous experience does help, for instance, during counselling if we have clients who started the method some time time, you will hear them commenting that they know the stuff already hence they do not need to go through the counselling process. So if you try to give them some few questions regarding the method you will know that they are indeed responding to each one of them, this makes the work simple because you know the counselling process will be easy and does not take a lot of time. Whilst if you have new clients it takes long to counsel them

INTERVIEWER: Alright

RESPONDENT: Sure

Title: MZs_HSA_1.docx

Descriptor Info:	District: Mzimba South	Target Population: TP1 - Providers/HSAs	Respondent type: HSA	Aware of SI: Yes - had heard of SI before	Coding phase: 2. Double coded	Client marital status: N/A - provider	Client age group: N/A - provider	Time since trained:

Codes Applied:	P3.3.1 - Differences in counselling/training approach by population group

Q11. Interviewer: Would the way you counsel a client on injectables differ if the client had used injectables before?

Respondent: There is no difference because once a client has come for injectable we do counsel them in details despite that client has used it before or not.

Interviewer: Okay.

Respondent: We are doing this because someone might have used it before, but may she has forgotten.

Interviewer: Alright, what about if they were adolescent, were married/unmarried if they want to delay or a first pregnancy versus space or limit their pregnancies?

Respondent: The counselling is the same, there is no different.

Interviewer: No different?

Respondent: Mmmmmm. (Yes)

Interviewer: What if you are seeing them in the facility, does it differ?

Respondent: It differs because it might happen that we haven`t carry other methods of family planning or the method she wants is out of stock thus why we tell them to come to the facility.

Title: MZs_HSA_1.docx

Descriptor Info:	District: Mzimba South	Target Population: TP1 - Providers/HSAs	Respondent type: HSA	Aware of SI: Yes - had heard of SI before	Coding phase: 2. Double coded	Client marital status: N/A - provider	Client age group: N/A - provider	Time since trained:

Codes Applied:	P3.3.1 - Differences in counselling/training approach by population group

Interviewer: Do you use any resources during the counselling like condom filled with sand or sugar?

Respondent: As for me most of the times I use condom filled with sand for demonstration for us to know if they can do it.

Interviewer: What about to the adolescent? What resources do you use during counselling?

Respondent: We use the same materials for both adults as well as adolescents.

Title: MZs_HSA_1.docx

Descriptor Info:	District: Mzimba South	Target Population: TP1 - Providers/HSAs	Respondent type: HSA	Aware of SI: Yes - had heard of SI before	Coding phase: 2. Double coded	Client marital status: N/A - provider	Client age group: N/A - provider	Time since trained:

Codes Applied:	C0.3 - Client previous use/experience of any FP method	P3.3.1 - Differences in counselling/training approach by population group	A6 - Adol Perspectives/preferences for DMPA-SC SI

Interviewer: Do you think having previous experience with injectables makes a woman less or more likely to consider self-injection?

Respondent: Yes.

Interviewer: Why do you think so?

Respondent: Because they know its advantages and how it works thus why they can choose self- injection option.

Interviewer: What about specifically for adolescent?

Respondent: Its all the same.

Interviewer: You mean there is no different for adolescent?

Respondent: Yes.

Title: KK_FAC_1.docx

Descriptor Info:	District: Nkhotakota	Target Population: TP1 - Providers/HSAs	Respondent type: Facility-based provider	Aware of SI: Yes - had heard of SI before	Coding phase: 3. Triple coded	Client marital status: N/A - provider	Client age group: N/A - provider	Time since trained: A year or more ago

Codes Applied:	P3.3.1 - Differences in counselling/training approach by population group	C1.1.2 - Baseline correct knowledge/ awareness of injectables

INTERVIEWER: Alright fine, would the way you counsel a client on injectables differ if they had used injectables in the past? Let’s assume one was using depo and now she has opted for Sayana , would the counselling differ?

RESPONDENT: Sayana

INTERVIEWER: If it can be different, how can it differ?

RESPONDENT: The counselling would differ a little bit, since for Sayana client is supposed to do self-injection? So we are supposed to tarin the client on how best she will handle or use the injection when she back home. Whilst the other one we administer it right here.

INTERVIEWER: Okay

RESPONDENT: As for this one we need to go into details so that we are sure that she understand so that when she gets back home she does not miss

Title: KK_FAC_1.docx

Descriptor Info:	District: Nkhotakota	Target Population: TP1 - Providers/HSAs	Respondent type: Facility-based provider	Aware of SI: Yes - had heard of SI before	Coding phase: 3. Triple coded	Client marital status: N/A - provider	Client age group: N/A - provider	Time since trained: A year or more ago

Codes Applied:	P3.3.1 - Differences in counselling/training approach by population group

INTERVIEWER: Let’s assume one was using depo and now she has opted for Sayana , would the counselling differ?

RESPONDENT: Sayana

INTERVIEWER: If it can be different, how can it differ?

RESPONDENT: The counselling would differ a little bit, since for Sayana client is supposed to do self-injection? So we are supposed to tarin the client on how best she will handle or use the injection when she back home. Whilst the other one we administer it right here.

INTERVIEWER: Okay

RESPONDENT: As for this one we need to go into details so that we are sure that she understand so that when she gets back home she does not miss it .

INTERVIEWER: Okay fine, What about if they were an adolescent, were married /unmarried, if they wanted to take Sayana would the counselling differ?

RESPONDENT: The counselling will not that much, however, in case of the adolescence we take some time when counselling them.

INTERVIEWER: Okay

RESPONDENT: Because they rush in making up a decision. Hence, we make sure that we go deeper in counselling them so that they make a sound decision. Because some may get the method but they may not use the injection. That’s what most adolescent do.

Time stamp: (20 minutes 27 seconds)

INTERVIEWER: Okay so they may just get the injections?

RESPONDENT: yes, as if they will use it but they do not

INTERVIEWER: okay

RESPONDENT: For instance in youth clubs you find out that most adolescents will get condoms but you note there is an increased number of teen pregnancies. This clearly shows that they don’t use the methods, they just take them home instead.

Title: KK_FAC_1.docx

Descriptor Info:	District: Nkhotakota	Target Population: TP1 - Providers/HSAs	Respondent type: Facility-based provider	Aware of SI: Yes - had heard of SI before	Coding phase: 3. Triple coded	Client marital status: N/A - provider	Client age group: N/A - provider	Time since trained: A year or more ago

Codes Applied:	A6 - Adol Perspectives/preferences for DMPA-SC SI	C8.3 - Discretion of injectables/SI	P3.3.1 - Differences in counselling/training approach by population group

INTERVIEWER: What about adolescents if you give them the same counselling on self-injection what is their response?

RESPONDENT: The adolescents find it so exiting, to them they feel if they take the method no one will know that they are doing Family planning. Bu the issue is the one that I said earlier on that most of the decisions among the adolescents are done out of excitement, but you may find out that they are not using the injections. That what I see as a problem to this age group.

Title: KK_FAC_1.docx

Descriptor Info:	District: Nkhotakota	Target Population: TP1 - Providers/HSAs	Respondent type: Facility-based provider	Aware of SI: Yes - had heard of SI before	Coding phase: 3. Triple coded	Client marital status: N/A - provider	Client age group: N/A - provider	Time since trained: A year or more ago

Codes Applied:	P3.1.5 - Ease of training clients in SI	P3.3.1 - Differences in counselling/training approach by population group	P3.2.3 - Complexity/difficulty of training clients in SI

INTERVIEWER: Alright fine, how easy or difficult is it to train clients in DMPA for self-injection

RESPONDENT: The understanding of adults is a bit complex

INTERVIEWER: Okay

RESPONDENT: Hence it becomes difficult for them to grasp the information easily as such you have to say it again and again. It’s different from adolescents when you train them, they capture the info easily. However, we try our best that if a client chose a method we should drill her until she understands it.

INTERVIEWER: What about specifically for adolescents?

RESPONDENT: I have explained they are so fast to understand something

Title: KK_FAC_1.docx

Descriptor Info:	District: Nkhotakota	Target Population: TP1 - Providers/HSAs	Respondent type: Facility-based provider	Aware of SI: Yes - had heard of SI before	Coding phase: 3. Triple coded	Client marital status: N/A - provider	Client age group: N/A - provider	Time since trained: A year or more ago

Codes Applied:	A6 - Adol Perspectives/preferences for DMPA-SC SI	P3.4 - Time to counsel/train, general	P3.3.1 - Differences in counselling/training approach by population group

INTERVIEWER: What about for adolescents whom you said they easily capture information, how would you compare the time you spend with then in counselling with adults .

RESPONDENT: For the adolescents it little time

INTERVIEWER: Its little time not so?

RESPONDENT: Yes

Title: KK_FAC_1.docx

Descriptor Info:	District: Nkhotakota	Target Population: TP1 - Providers/HSAs	Respondent type: Facility-based provider	Aware of SI: Yes - had heard of SI before	Coding phase: 3. Triple coded	Client marital status: N/A - provider	Client age group: N/A - provider	Time since trained: A year or more ago

Codes Applied:	C1.1.2 - Baseline correct knowledge/ awareness of injectables	P3.3.1 - Differences in counselling/training approach by population group	P3.1.5 - Ease of training clients in SI

INTERVIEWER: Do you think having previous experience with injectables makes a woman more less likely to consider self-injection

RESPONDENT: To have what?

INTERVIEWER: Having previous experience like knowledge, for instance in the past the client was taking depo so with such kind of experience

RESPONDENT: Okay

INTERVIEWER: can it help a woman to choose or not to choose self-injection?

RESPONDENT: It helps since one has knowledge in injectables already

INTERVIEWER: Okay

RESPONDENT: So it feels like you are just shifting someone from what she already knew to a similar concepts but just with few changes in that the other one will require you to inject yourself though it also works in three months. Hence it becomes easily because it’s just that you have added a little information on what she already knew.

INTERVIEWER: It differs from one who have not heard about injectables before.

RESPONDENT: Yes

Title: KK_FAC_1.docx

Descriptor Info:	District: Nkhotakota	Target Population: TP1 - Providers/HSAs	Respondent type: Facility-based provider	Aware of SI: Yes - had heard of SI before	Coding phase: 3. Triple coded	Client marital status: N/A - provider	Client age group: N/A - provider	Time since trained: A year or more ago

Codes Applied:	P3.3.1 - Differences in counselling/training approach by population group	A6 - Adol Perspectives/preferences for DMPA-SC SI

INTERVIEWER: Does that apply to adolescents as well or else its different

RESPONDENT: For adolescent we start from scratch

INTERVIEWER: Okay

RESPONDENT: Because most of them do not have knowledge

INTERVIEWER: yes

RESPONDENT: However, we normally assess the level of their knowledge during counselling, to what extend do they know the stuff. So that helps us to know at what level do we go in giving out information, should we go into details or some have information on the topic in discussion. So one is able to know that through how clients are responding to the questions asked.

Title: KK_FAC_1.docx

Descriptor Info:	District: Nkhotakota	Target Population: TP1 - Providers/HSAs	Respondent type: Facility-based provider	Aware of SI: Yes - had heard of SI before	Coding phase: 3. Triple coded	Client marital status: N/A - provider	Client age group: N/A - provider	Time since trained: A year or more ago

Codes Applied:	A6 - Adol Perspectives/preferences for DMPA-SC SI	P3.3.1 - Differences in counselling/training approach by population group

INTERVIEWER: What about specifically for adolescent, how do they react?

RESPONDENT: in most cases adolescent have no problem

Title: MZs_HSA_5.docx

Descriptor Info:	District: Mzimba South	Target Population: TP1 - Providers/HSAs	Respondent type: HSA	Aware of SI: Yes - had heard of SI before	Coding phase: 1. Single coded	Client marital status: N/A - provider	Client age group: N/A - provider	Time since trained: A year or more ago

Codes Applied:	P3.3.1 - Differences in counselling/training approach by population group

Interviewer: is the counselling for married and unmarried on injectable different?

Respondent: the counselling is similar because the injectable has the same mode of action. The goal is the prevent pregnancy. So the counselling would not be different.

Interviewer: is the counselling different for someone with a child and someone without a child?

Respondent: it might be different because for the one with a child we ask about the age of the child and last menstrual period.

Interviewer: is the counselling different for a client you meet at the facility compared to a client you meet at the community?

Respondent: counselling is the same

Title: KK_HSA_3.docx

Descriptor Info:	District: Nkhotakota	Target Population: TP1 - Providers/HSAs	Respondent type: HSA	Aware of SI: Yes - had heard of SI before	Coding phase: 3. Triple coded	Client marital status: N/A - provider	Client age group: N/A - provider	Time since trained: A year or more ago

Codes Applied:	A6 - Adol Perspectives/preferences for DMPA-SC SI	P3.3.1 - Differences in counselling/training approach by population group

INTERVIEWER: What about adolescents? Girls.

RESPONDENT: Girls?

INTERVIEWER: mmmhu.

RESPONDENT: Girls are not concerned because they know that when we tarin them, they will be able to self-inject. But most girls use. condoms.

Title: KK_HSA_3.docx

Descriptor Info:	District: Nkhotakota	Target Population: TP1 - Providers/HSAs	Respondent type: HSA	Aware of SI: Yes - had heard of SI before	Coding phase: 3. Triple coded	Client marital status: N/A - provider	Client age group: N/A - provider	Time since trained: A year or more ago

Codes Applied:	P3.3.1 - Differences in counselling/training approach by population group

INTERVIEWER: if a client was previously on an injectable…she was on Depo...she is back and she wants to be re-initiated

RESPONDENT: Same Depo?

INTERVIEWER: Yes. Does the counselling differ with a new client?

RESPONDENT: this one was…

INTERVIEWER: she was…

RESPONDENT: She was already on family planning?

INTERVIEWER: yes. On an injectable.

RESPONDENT: injectable

INTERVIEWER: she stopped and she wants to be re-initiated...

RESPONDENT: Again?

INTERVIEWER: The type of counselling that she gets, is it…is it the same with a new client?

RESPONDENT: Yes, it is supposed to be different because this one…the new client and the one who was already on this method. We are supposed to counsel the new client because a new...new client is supposed to be counseled. While the other one we ask her if she has encountered any side effects when using Depo or Sayana. The counselling is the same. But for the new client we are supposed to give her proper counselling. But it is not that different.

INTERVIEWER: But this one stopped and she wants to try the method again. Is the counselling the same with a new client…do you consider her as a new client?

RESPONDENT: Yes, we consider her as a new client because she stopped and she wants to try again. She is considered as a new client and we also do counselling for the second time.

INTERVIEWER: mmmhu.

RESPONDENT: mmmmh.

INTERVIEWER: What about for adolescents. How do you conduct counselling?

RESPONDENT: An adolescent?

INTERVIEWER: mmmh.

RESPONDENT: Do you mean a new client?

INTERVIEWER: yes, an adolescent girl.

RESPONDENT: A girl

INTERVIEWER: she wants contraceptives…

RESPONDENT: ...yes, counselling is counselling.

INTERVIEWER: Maybe she got pregnant. Or maybe she wants to prevent pregnancy.

RESPONDENT: Mmmmh. Counselling is counselling it does not matter if it’s an adolescent, an adult or if she has used contraceptives for some time. The counselling is the same according to how it is supposed to be done.

Title: KK_HSA_3.docx

Descriptor Info:	District: Nkhotakota	Target Population: TP1 - Providers/HSAs	Respondent type: HSA	Aware of SI: Yes - had heard of SI before	Coding phase: 3. Triple coded	Client marital status: N/A - provider	Client age group: N/A - provider	Time since trained: A year or more ago

Codes Applied:	P3.3.1 - Differences in counselling/training approach by population group	A6 - Adol Perspectives/preferences for DMPA-SC SI

INTERVIEWER: What about for adolescents. How do you conduct counselling?

RESPONDENT: An adolescent?

INTERVIEWER: mmmh.

RESPONDENT: Do you mean a new client?

INTERVIEWER: yes, an adolescent girl.

RESPONDENT: A girl

INTERVIEWER: she wants contraceptives…

RESPONDENT: ...yes, counselling is counselling.

INTERVIEWER: Maybe she got pregnant. Or maybe she wants to prevent pregnancy.

RESPONDENT: Mmmmh. Counselling is counselling it does not matter if it’s an adolescent, an adult or if she has used contraceptives for some time. The counselling is the same according to how it is supposed to be done.

Title: ZA_HSA_4.docx

Descriptor Info:	District: Zomba	Target Population: TP1 - Providers/HSAs	Respondent type: HSA	Aware of SI: Yes - had heard of SI before	Coding phase: 1. Single coded	Client marital status: N/A - provider	Client age group: N/A - provider	Time since trained: A year or more ago

Codes Applied:	P3.3.1 - Differences in counselling/training approach by population group	A6 - Adol Perspectives/preferences for DMPA-SC SI

Interviewer: Okay fine. What way do you use when counselling them? Umm can you advise me in a different way had it been I was an adolescent?

Respondent: Yes. I do advise them in a different way because I tell the girl that yes, these are the contraception methods indeed, but you don’t have to use them for a long time because they may affect your future. For example, you still are in school, and then you want to have a child, it’s not going to happen. So, you are not supposed to get used to the methods.

Interviewer: Okay fine. Are there any differences on how you do advise married people and those who are not married?

Respondent: Yes its different because those who are married know what they are doing and why they are doing that so that they may take a long time to have another child or maybe they were facing problems during delivery. So, they may choose the contraception methods. Others say that I am using the contraception methods now up until when I will be infertile. That’s what they say. We may also tell them that “mmm you have been injected for so many times eeh..about 50 times iih! It’s too much!” So they say, “iii no, I just want to get infertile after using the methods for a long time.”

Time stamp: 28:54

Interviewer: Hahahah okay fine. Umm, do you advise people differently depending on their different communities?

Respondent: There is no differences. It’s all the same.

Title: KK_HSA_4.docx

Descriptor Info:	District: Nkhotakota	Target Population: TP1 - Providers/HSAs	Respondent type: HSA	Aware of SI: Yes - had heard of SI before	Coding phase: 1. Single coded	Client marital status: N/A - provider	Client age group: N/A - provider	Time since trained: A year or more ago

Codes Applied:	P3.3.1 - Differences in counselling/training approach by population group	C1.1.2 - Baseline correct knowledge/ awareness of injectables	C0.3 - Client previous use/experience of any FP method

INTERVIEWER: Alright, would the way you counsel a client on injectable differ if they had used injectables in the past? Am I clear?

RESPONDENT: Yes, your clear, Yes, there may be a different as already said that we are now using a new method called Sayana.

INTERVIEWER: ok

RESPONDENT: For the client to differentiate the two we may explain to her that, the new method Sayana have both the medication and injection in the same pack. Meaning that everything is in the same package.

INTERVIEWER: Alright

RESPONDENT: But when we talk of the injection that is administered by the provider, the injection and the bottle that contains the medicine are separate. Secondly, we may also add to say this new one you do self-injection which means you ca do it at home at any time you want, either in the morning or afternoon.

INTERVIEWER: Ok

RESPONDENT: Provided you do it on the indicated date. Those are some of the things that we explain to our client so that she is able to differentiate the two. And when she understands properly she able to note that this is the best method because I will be able to do it at my own time after I finish doing other endeavors.

Title: KK_HSA_4.docx

Descriptor Info:	District: Nkhotakota	Target Population: TP1 - Providers/HSAs	Respondent type: HSA	Aware of SI: Yes - had heard of SI before	Coding phase: 1. Single coded	Client marital status: N/A - provider	Client age group: N/A - provider	Time since trained: A year or more ago

Codes Applied:	P3.3.1 - Differences in counselling/training approach by population group	A6 - Adol Perspectives/preferences for DMPA-SC SI

INTERVIEWER: What about if they were an adolescent, were married / unmarried, if they wanted to delay a first pregnancy would the advice differ from the one you would give to an adult and an adolescent? If you had an adult who have been married and have been taking injectable all along, and the other side you have this adolescent who has just been married or is about to t married. Would the counselling differ?

RESPONDENT: Then counselling would be the same.

INTERVIEWER: Alright

RESPONDENT: But it may just slightly differ because the message is going to two different people, since this other person have been married for some time, whilst an adolescent is taking the method with some intentions behind. Maybe they just want to delay pregnancy so you may tell them that they should take the method so that they should not pregnant whilst young.

INTERVIEWER: Alright

RESPONDENT: Since she is not mature for the pregnancy.

INTERVIEWER: Alright

RESPONDENT: But you may just get contraceptives as you wait for the body to get ready for pregnancy. Whilst for an adult who is married, we cannot tell her about this since she is already matured enough for the pregnancy. That’s how I think the message would differ. But for the rest of the message would go the same.

Title: KK_HSA_4.docx

Descriptor Info:	District: Nkhotakota	Target Population: TP1 - Providers/HSAs	Respondent type: HSA	Aware of SI: Yes - had heard of SI before	Coding phase: 1. Single coded	Client marital status: N/A - provider	Client age group: N/A - provider	Time since trained: A year or more ago

Codes Applied:	P3.3.1 - Differences in counselling/training approach by population group

INTERVIEWER: Alright. What if you were seeing them in the community versus seeing the at a facility would the counselling differ?

RESPONDENT: Counselling would have been the same.

INTERVIEWER: Would be same?

RESPONDENT: Yes

INTERVIEWER: The way you would counsel her in the community and if she comes at a facility.

RESPONDENT: Yes, it will be just the same whether we meet her at a facility or in the community.

INTERVIEWER: Alright

RESPONDENT: Is the same

Title: MZs_HSA_4.docx

Descriptor Info:	District: Mzimba South	Target Population: TP1 - Providers/HSAs	Respondent type: HSA	Aware of SI: Yes - had heard of SI before	Coding phase: 1. Single coded	Client marital status: N/A - provider	Client age group: N/A - provider	Time since trained:

Codes Applied:	P3.3.1 - Differences in counselling/training approach by population group

Q11. Interviewer: how different is it offering counsel on injectables to today’s women as compared to clients in the past?

Respondent: it is different because previously we were only offering Depo. With the coming of Sayana we distinguish to the clients the two methods and their features and their advantages and disadvantages.

Interviewer: how do you explain to a client who is yet to be married and does not want an unplanned pregnancy?

Respondent: I explain to such client on all the methods available here. This includes Depo and Sayana and everything about the methods.

Interviewer: what if you are meeting the client in the community and not at the facility?

Respondent: there can be a difference because the client may be from my colleague’s area as such, I will counsel the client and refer him to my colleague from his area.

Title: ZA_FAC_1.docx

Descriptor Info:	District: Zomba	Target Population: TP1 - Providers/HSAs	Respondent type: Facility-based provider	Aware of SI: Yes - had heard of SI before	Coding phase: 2. Double coded	Client marital status: N/A - provider	Client age group: N/A - provider	Time since trained: Less than a year ago

Codes Applied:	P3.3.1 - Differences in counselling/training approach by population group

Respondent: we need to understand them and help them out depending on the decision that they have made. So we try that, especially for the youth, we should provide them with youth friendly services, and also give information to those that are in school since we are surrounded by schools here like [NAME] primary

Title: ZA_FAC_1.docx

Descriptor Info:	District: Zomba	Target Population: TP1 - Providers/HSAs	Respondent type: Facility-based provider	Aware of SI: Yes - had heard of SI before	Coding phase: 2. Double coded	Client marital status: N/A - provider	Client age group: N/A - provider	Time since trained: Less than a year ago

Codes Applied:	A5.2 - Stigma/judgement	P3.3.1 - Differences in counselling/training approach by population group

Respondent: so for the school going women we start with abstinence; abstinence is also family planning.

Interviewer: mmmm (showing that one is following the conversation)

Respondent: we provide information, and we also provide those methods.

Title: ZA_FAC_1.docx

Descriptor Info:	District: Zomba	Target Population: TP1 - Providers/HSAs	Respondent type: Facility-based provider	Aware of SI: Yes - had heard of SI before	Coding phase: 2. Double coded	Client marital status: N/A - provider	Client age group: N/A - provider	Time since trained: Less than a year ago

Codes Applied:	P3.3.1 - Differences in counselling/training approach by population group

Interviewer: could your counselling differ if you are dealing with a new user of injectables and to an old user?

Respondent: yes, it’s different

Interviewer: mmmm (showing that one is following the conversation)

Respondent: the first part of the counselling is the same.

Interviewer: mmmm (showing that one is following the conversation)

Respondent: if it’s a new user of contraceptives if ask them whether it’s their first time using contraceptives

Interviewer: mmmm (showing that one is following the conversation)

Respondent: if they are just continuing use

Interviewer: yah

Respondent: we will then ask her if she encountered any problems if they are an old user

Interviewer: mmmm (showing that one is following the conversation)

Respondent: we will also explain to them of other methods and their dynamics

Interviewer: mmmm (showing that one is following the conversation)

Respondent: we will give her a full health talk on family planning

Interviewer: mmmm (showing that one is following the conversation)

Respondent: we will also ask her of her prerequisite knowledge

Interviewer: mmmm (showing that one is following the conversation)

Respondent: sometimes the clients use a particular method because someone uses it

Interviewer: mmmm (showing that one is following the conversation)

Respondent: when they say that they want an injectable

Interviewer: eh

Respondent: we also guide them based on what their future plan is

Interviewer: mmmm (showing that one is following the conversation)

Respondent: we don’t direct them on what method to choose we just provide them with guidance

Interviewer: alright. Does the counselling differ for adolescents and older women?

Respondent: yes, it’s different.

Interviewer: mmmm (showing that one is following the conversation)

Respondent: eh especially for adolescents

Interviewer: mmmm (showing that one is following the conversation)

Respondent: when counselling the adolescents we know that some of them have not yet started using contraceptives

Interviewer: mmmm (showing that one is following the conversation)

Respondent: the message is not that everyone should get contraceptives

Interviewer: mmmm (showing that one is following the conversation)

Respondent: for the older women our target is for them to get the methods

Interviewer: yah yah

Respondent: yah we make sure to communicate to the youth those methods that are more private

Interviewer: mmmm (showing that one is following the conversation)

Respondent: yah we make sure that we don’t leave anybody behind

Interviewer: alright.

Respondent: yeah

Title: ZA_FAC_1.docx

Descriptor Info:	District: Zomba	Target Population: TP1 - Providers/HSAs	Respondent type: Facility-based provider	Aware of SI: Yes - had heard of SI before	Coding phase: 2. Double coded	Client marital status: N/A - provider	Client age group: N/A - provider	Time since trained: Less than a year ago

Codes Applied:	P3.3.1 - Differences in counselling/training approach by population group

Interviewer: alright. Does the counselling differ for adolescents and older women?

Respondent: yes, it’s different.

Interviewer: mmmm (showing that one is following the conversation)

Respondent: eh especially for adolescents

Interviewer: mmmm (showing that one is following the conversation)

Respondent: when counselling the adolescents we know that some of them have not yet started using contraceptives

Interviewer: mmmm (showing that one is following the conversation)

Respondent: the message is not that everyone should get contraceptives

Interviewer: mmmm (showing that one is following the conversation)

Respondent: for the older women our target is for them to get the methods

Interviewer: yah yah

Respondent: yah we make sure to communicate to the youth those methods that are more private

Interviewer: mmmm (showing that one is following the conversation)

Respondent: yah we make sure that we don’t leave anybody behind

Interviewer: alright.

Respondent: yeah

Title: ZA_FAC_2.docx

Descriptor Info:	District: Zomba	Target Population: TP1 - Providers/HSAs	Respondent type: Facility-based provider	Aware of SI: Yes - had heard of SI before	Coding phase: 3. Triple coded	Client marital status: N/A - provider	Client age group: N/A - provider	Time since trained: Less than a year ago

Codes Applied:	P3.3.1 - Differences in counselling/training approach by population group	C1.1.2 - Baseline correct knowledge/ awareness of injectables

Interviewer: Can the counselling you give to women who do the self-injection method be different if the woman has used the method in the past?

Respondent: It’s not very different because the main issue is how long the self-injection is used in the body. So we explain to them that the method is basically the same. But the difference is only on the type of bottles and the method of injecting. Otherwise it is the same method of self-injection which works for 3 months so that a person doesn’t get pregnant.

Interviewer: Okay. When the person a client is youthful, whether married or unmarried. If the person wanted to delay pregnancy for the first time or reduce the number of pregnancies. Can you counsel them differently? Assuming a youthful person comes along, do you counsel the two differently?

Respondent: No, we do not differentiate.

Interviewer: A person who is married, unmarried, can you counsel them differently?

Respondent: No. The target is that these people should all know about the dial family planning method. So whether it’s the youth, those married or unmarried we specify that family planning does not protect one from being infected by sexually transmitted diseases. So we also encourage them to be using condoms, but the planning methods are the same in accordance with the counselling we have given. But when they combine the dial loop together with the family planning method which they have chosen, they will be protected from many things.

Interviewer: Okay thank you. Does the counselling you give differ for example at the hospital and in outreaches? Is the counselling the same?

Respondent: It is the same.

Interviewer: It does not differ in any way?

Respondent: No.

Title: MZs_HSA_3.docx

Descriptor Info:	District: Mzimba South	Target Population: TP1 - Providers/HSAs	Respondent type: HSA	Aware of SI: Yes - had heard of SI before	Coding phase: 3. Triple coded	Client marital status: N/A - provider	Client age group: N/A - provider	Time since trained: Less than a year ago

Codes Applied:	P3.3.1 - Differences in counselling/training approach by population group

Q.11 Interviewer: Would the way you counsel the client on injectable differ if they had used injectable in the past?

Respondent: [silent moment]….. because that one is a subsequent the will differ because she is already used to……… that counselling but the new.. the one who is new has to be counselled in all.

Interviewer: what would differ?

Respondent: the time of counselling… mmmh because that one huuuu…knows already what is to be done because when I ask her this one is done like this this one is done like that but the new client will not have anything in her head.

Interviewer: what about when they were married/unmarried?

Respondent: the counselling is just the same. Yah!

Interviewer: what about if they were adolescent?

Respondent: [moment of silence] mmmmh that one has little understanding so we have to repeat repeat what we are telling her so that she should know exactly what will happen after getting that ujeni.

Interviewer: what if it’s an adolescent who have gone to school?

Respondent: no…it will be the same

Interviewer: what if you were seeing them in the community versus seeing them at the facility?

Respondent: no it’s the same…[clears the throat] because they are the same in the way that whatever I tell this one here it should be the same with that client that client it will be the same with that in the community.

Title: KK_HSA_2.docx

Descriptor Info:	District: Nkhotakota	Target Population: TP1 - Providers/HSAs	Respondent type: HSA	Aware of SI: Yes - had heard of SI before	Coding phase: 2. Double coded	Client marital status: N/A - provider	Client age group: N/A - provider	Time since trained: A year or more ago

Codes Applied:	P3.3.1 - Differences in counselling/training approach by population group

INTERVIEWER: would the way you would counsel a client on injectables differ if they had used injectables in the past. For instance in the past the client was on Depo and now with the new method (Sayana) would the counselling differ.

RESPONDENT: Initially she was on Depo?

INTERVIEWER: Yes

RESPONDENT: She was on Depo and now she wants to change and take another method

INTERVIEWER: Mhhh

RESPONDENT: You still have to take her through the process again since as she was using Depo she is the one who noticed the good side and bad side of it since as a provider I may not know exactly how she faced whilst using the method

INTERVIEWER: Assuming she was using Depo and now she would like to go for Sayana would the counselling differ?

RESPONDENT: yaah It would differ

INTERVIEWER: Would it differ?

RESPONDENT: yah yah

INTERVIEWER: How would it differ?

RESPONDENT: It will differ since Depo and Sayana are a bit different specifically where client is supposed to inject herself.

INTERVIEWER: Ohooo so that's the only different but the rest would be the same is that so?

RESPONDENT: yes

INTERVIEWER: What about if the client were an adolescent, were married / unmarried, if they wanted to delay a first pregnancy versus space or limit their pregnancies?

RESPONDENT: aaa when we talk of family planning method, counselling is not that different

INTERVIEWER: okay fine

RESPONDENT: Yes when a client comes for family planning method

INTERVIEWER: Yes

RESPONDENT: That I want to take a family planning method normally they are things that she wants to fulfill before she starts to have children because if I start to bear children I want be able to do some things

INTERVIEWER: Okay fine

RESPONDENT: Sure

INTERVIEWER: What if you were seeing the client in the community versus seeing them at the facility would the counselling process differ.

RESPONDENT: aaa Counselling would be the same

INTERVIEWER: Okay so you mean counselling process would be the same not so

RESPONDENT: Sure

Title: KK_HSA_2.docx

Descriptor Info:	District: Nkhotakota	Target Population: TP1 - Providers/HSAs	Respondent type: HSA	Aware of SI: Yes - had heard of SI before	Coding phase: 2. Double coded	Client marital status: N/A - provider	Client age group: N/A - provider	Time since trained: A year or more ago

Codes Applied:	P3.3.1 - Differences in counselling/training approach by population group	A6 - Adol Perspectives/preferences for DMPA-SC SI

INTERVIEWER: What about specifically for adolescence do you think having previous experience with injectables would make them to choose this method.

RESPONDENT: aaai will go a bit deeper, adolescent also have feelings and there are two things happening at once the feelings in her body and also school. Hence for one to continue with school we advice them to use family planning methods

INTERVIEWER: Okay fine

RESPONDENT : Mhh if you don’t do family planning then you will be home and stop going to school

Title: ZA_HSA_6.docx

Descriptor Info:	District: Zomba	Target Population: TP1 - Providers/HSAs	Respondent type: HSA	Aware of SI: Yes - had heard of SI before	Coding phase: 1. Single coded	Client marital status: N/A - provider	Client age group: N/A - provider	Time since trained:

Codes Applied:	P3.3.1 - Differences in counselling/training approach by population group

Interviewer: Does the way that you counsel women differ if you are meeting with adolescents compared to older women?

Respondent: yeah, it is different, the difference is that the methods for teaching adolescents and those for teaching older women

Interviewer: yah

Respondent: and older women, they are different.

Interviewer: mmmm (showing that one is following the conversation)

Respondent: the adolescents we teach them as if we are of their age or we are their peers.

Interviewer: mmmm (showing that one is following the conversation)

Respondent: So that they should feel like we are the same level even though our activities are, our ages are different.

Interviewer: yah yah

Respondent: but for the older women, we train them to use the resources

Interviewer: mmmm (showing that one is following the conversation)

Respondent: let’s say that they are at the age where they have so much going on, so we make sure to mobilize them and they should not think about what they have left at home, say maybe they have fought with their family at home.

Interviewer: mmmm (showing that one is following the conversation)

Respondent: we sing songs so that they should loosen up, maybe we will tie a cloth in our waist and dance, so with that they are excited that the HSAs make them happy, in the end we provide the information, and it looks that they receive it with gladness. They forget all their issues from home.

Interviewer: mmmm (showing that one is following the conversation)

Respondent: For very older women most of the times it is difficult since they want to access sterilization methods and they meet with the nurses.

Title: ZA_HSA_6.docx

Descriptor Info:	District: Zomba	Target Population: TP1 - Providers/HSAs	Respondent type: HSA	Aware of SI: Yes - had heard of SI before	Coding phase: 1. Single coded	Client marital status: N/A - provider	Client age group: N/A - provider	Time since trained:

Codes Applied:	P3.3.1 - Differences in counselling/training approach by population group

Interviewer: ah does the way that you provide counselling differ if you were speaking to a married or unmarried woman?

Respondent: wen we are in a group of clients that are accessing family planning services, we do not separate them the married and unmarried.

Interviewer: mmmm (showing that one is following the conversation)

Respondent: all of them we take them as women of childbearing age, those that have children for example yeah

Interviewer: mmmm (showing that one is following the conversation)

Respondent: it means all of them are the same, so when we are explaining we don’t ask them why you are getting family planning, yet you’re not married or that your friends that are on this side are married, we mix them all and provide them with the service without looking at their marital status.

Interviewer: alright

Respondent: because all of them are of childbearing age

Title: ZA_HSA_6.docx

Descriptor Info:	District: Zomba	Target Population: TP1 - Providers/HSAs	Respondent type: HSA	Aware of SI: Yes - had heard of SI before	Coding phase: 1. Single coded	Client marital status: N/A - provider	Client age group: N/A - provider	Time since trained:

Codes Applied:	P3.3.1 - Differences in counselling/training approach by population group

Interviewer: Alright, does the time that you take to train women in DMPA-SC differ when you are working with adolescents compared to older women?

Respondent: it cannot be a big difference, because maybe the group of adolescents, 80 percent don’t have an interest in contraceptives

Interviewer: mmmm (showing that one is following the conversation)

Respondent: and they also don’t follow the methods, that’s why they don’t access them yeah.

Interviewer: yah yah

Respondent: so maybe it can take a long time because of questions and other things like that.

Interviewer: alright

Respondent: for the older women, they already know, some of them have already accessed and they just stopped because they bore children and what so own yeah, they already have the knowledge, so it does not take time.

Title: ZA_HSA_6.docx

Descriptor Info:	District: Zomba	Target Population: TP1 - Providers/HSAs	Respondent type: HSA	Aware of SI: Yes - had heard of SI before	Coding phase: 1. Single coded	Client marital status: N/A - provider	Client age group: N/A - provider	Time since trained:

Codes Applied:	P3.3.1 - Differences in counselling/training approach by population group

Interviewer: Do you think having previous knowledge about DMPA-SC can influence whether one chose the method or not?

Respondent: Previous knowledge like?

Interviewer: That they already know about DMPA-SC, do you think that this knowledge can make women to choose DMPA-SC?

Respondent: Ah no everyone who comes to access this method

Interviewer: yah

Respondent: even if she was using other methods in the past

Interviewer: mmmm (showing that one is following the conversation)

Respondent: when they are coming for the second time, we take the as a new client, they’re supposed to be trained again

Interviewer: mmmm (showing that one is following the conversation)

Respondent: they should be trained again that this is how it works just like the way it is with other medications from the hospital, for example for Panadol, when a patient has received Panadol in the past and they have been told about the dosage if they come back to the hospital after one week we will also give them the information about the dosage, so any one who comes to the clinic to get these methods needs to be trained again and be guided on everything because here we take them as a new client.

Title: MZs_FAC_2.docx

Descriptor Info:	District: Mzimba South	Target Population: TP1 - Providers/HSAs	Respondent type: Facility-based provider	Aware of SI: Yes - had heard of SI before	Coding phase: 2. Double coded	Client marital status: N/A - provider	Client age group: N/A - provider	Time since trained:

Codes Applied:	P3.3.1 - Differences in counselling/training approach by population group

Q11. Interviewer: Would the way you counsel a client on injectables differ if they had used injectables in the past?

Respondent: Aaaaa… (thinking), I think it’s all the same. Only that with this Sayana, the client will be self-injecting and she will take a year without visiting the facility. If she has shown that she cannot do it the provider can inject her, while the one she has been using it was only the provider who was injecting her, and she has been visiting the facility every 3 months

Interviewer: What about if they were an adolescent, were married? Unmarried? If they wanted to delay first pregnancy versus space or limit their pregnancy?

Respondent: Will still do the counselling and tell them that they have made a good choice and it will help them to get prepared before having a child or another child

Interviewer: What if you are seeing them in the community versus seeing them at the facility?

Respondent: There will be no different.

Title: MZs_FAC_2.docx

Descriptor Info:	District: Mzimba South	Target Population: TP1 - Providers/HSAs	Respondent type: Facility-based provider	Aware of SI: Yes - had heard of SI before	Coding phase: 2. Double coded	Client marital status: N/A - provider	Client age group: N/A - provider	Time since trained:

Codes Applied:	P3.3.1 - Differences in counselling/training approach by population group	A6 - Adol Perspectives/preferences for DMPA-SC SI

Q13. Interviewer: Alright. How easy or difficult is to train clients on DMPA-SC for self-injection?

Respondent: (Coughing)

Interviewer: Sorry for that.

Respondent: The only problem we encounter it’s when we are giving the counselling on how they can self-inject and also on how they self-inject for the first time.

Interviewer: Okay

Respondent: Sure

Interviewer: What about specifically for adolescents?

Respondent: I think…. Hiccup…, there is no different

Interviewer: Alright.

Respondent: Sure

Title: MZs_HSA_6.docx

Descriptor Info:	District: Mzimba South	Target Population: TP1 - Providers/HSAs	Respondent type: HSA	Aware of SI: Yes - had heard of SI before	Coding phase: 1. Single coded	Client marital status: N/A - provider	Client age group: N/A - provider	Time since trained: A year or more ago

Codes Applied:	P3.3.1 - Differences in counselling/training approach by population group	A6 - Adol Perspectives/preferences for DMPA-SC SI

Interviewer: is the counselling similar for married and unmarried

Respondent: it might be different. We take town for unmarried girls to counsel them and ask questions because most of them have misconceptions from peers. We answer all questions on the misconceptions she has. Similarly married women have misconceptions from peers too. So we spend time on them as well until they understand and make a decision to get the family planning method

Interviewer: is the counselling similar for those with a child and for those who have never conceived

Respondent: the counselling is the same. We use the same counselling card and check list for questions. For the one with a child you would ask how many years she would want to do child spacing. That can be the only difference. For the girl it might be that she is still going to school or stopped but we do not probe much on that so long as she has made the decision herself. Then I provide Sayana for the whole year

Interviewer: is the counselling similar for a client you find at the facility compared to the client who finds you at the community

Respondent: it’s the same

Title: KK_HSA_6.docx

Descriptor Info:	District: Nkhotakota	Target Population: TP1 - Providers/HSAs	Respondent type: HSA	Aware of SI: Yes - had heard of SI before	Coding phase: 1. Single coded	Client marital status: N/A - provider	Client age group: N/A - provider	Time since trained: A year or more ago

Codes Applied:	P3.3.1 - Differences in counselling/training approach by population group

INTERVIEWER: Would the way you counsel a client on injectables differ if they had used injectables before?

RESPONDENT: counselling her?

INTERVIEWER: Yes. She was using before, and she wants to start using again. Is the counselling the same with a new client or it differs?

RESPONDENT: There might be a slight difference because this one has the knowledge. We ask her questions about what happed to her when she was using this method. We also ask her if she has ever used the method before or never. And she tells you she has used it before. You ask her about the challenges she faced, and she will tell you. And then we explain to her everything because she might have forgotten some information. But for a new client, we go deep because they don’t know anything. But for someone who has used before, we might start with questions for example the challenges she was facing. We cannot start from the beginning. What are challenges are you facing as you are using this method?

INTERVIEWER: What if she stopped and she wants to start using again?

RESPONDENT: She wants to start using again, ok. We ask her questions about why she stopped using the method. Oh my child was of age, some I was menstruating every now and then so I decided to stop. So we tell them that a person is supposed to have regular period but when it is irregular, we send her to the health centre.

Title: KK_HSA_6.docx

Descriptor Info:	District: Nkhotakota	Target Population: TP1 - Providers/HSAs	Respondent type: HSA	Aware of SI: Yes - had heard of SI before	Coding phase: 1. Single coded	Client marital status: N/A - provider	Client age group: N/A - provider	Time since trained: A year or more ago

Codes Applied:	P3.3.1 - Differences in counselling/training approach by population group	A6 - Adol Perspectives/preferences for DMPA-SC SI

INTERVIEWER: mmmhu. What about counselling for an adolescent who is not married, does it differ, or it is the same?

RESPONDENT: There can be a slight difference. But also there is no difference because counselling is not supposed to differ. You have to explain from the beginning up to the end. If we differentiate counselling, it is a mistake for us health workers. We explain all contraceptive methods to them. Because adolescents mostly use condoms, but we also explain the other methods. Counselling is the same for everyone

Title: KK_HSA_6.docx

Descriptor Info:	District: Nkhotakota	Target Population: TP1 - Providers/HSAs	Respondent type: HSA	Aware of SI: Yes - had heard of SI before	Coding phase: 1. Single coded	Client marital status: N/A - provider	Client age group: N/A - provider	Time since trained: A year or more ago

Codes Applied:	A6 - Adol Perspectives/preferences for DMPA-SC SI	A5.2 - Stigma/judgement	A88 - Adol. great quotes	P3.3.1 - Differences in counselling/training approach by population group

INTERVIEWER: Do you have adolescents who are on Sayana?

RESPONDENT: Adolescents; girls or boys?

INTERVIEWER: girls.

RESPONDENT: we have adolescent girls.

INTERVIEWER: because Sayana…

RESPONDENT: I have. A group of school girls approached me. I want to be an injectable contraceptive. Why? I first asked her questions. Because I have a boyfriend. A school girl, what are you doing with a boyfriend at your age? In what class are you? Standard 7. Why can’t you stop doing that? Aah I can’t but I was told that there is an injection you can self-inject, or you can inject a person. She said she wanted me to explain to her about contraception methods and I explained to her everything. When you explain to them, most of them opt for condoms. Girls do come and when we counsel them the way we do with their parents, they understand, and they go and self-inject at home. The parents are afraid more than the girls.

Title: KK_HSA_6.docx

Descriptor Info:	District: Nkhotakota	Target Population: TP1 - Providers/HSAs	Respondent type: HSA	Aware of SI: Yes - had heard of SI before	Coding phase: 1. Single coded	Client marital status: N/A - provider	Client age group: N/A - provider	Time since trained: A year or more ago

Codes Applied:	P3.2.3 - Complexity/difficulty of training clients in SI	A6 - Adol Perspectives/preferences for DMPA-SC SI	P3.3.1 - Differences in counselling/training approach by population group

INTERVIEWER: How easy or difficult is it to train clients in DMPA-SC?

RESPONDENT: Most of the women have experienced a lot of pain especially during child birth than adolescents. Training an adolescent is difficult than a woman because she knows that the pain will be for a short while than that of child birth. They understand fast than girls.

INTERVIEWER: okay.

RESPONDENT: yea.

Title: KK_HSA_6.docx

Descriptor Info:	District: Nkhotakota	Target Population: TP1 - Providers/HSAs	Respondent type: HSA	Aware of SI: Yes - had heard of SI before	Coding phase: 1. Single coded	Client marital status: N/A - provider	Client age group: N/A - provider	Time since trained: A year or more ago

Codes Applied:	P3.3.1 - Differences in counselling/training approach by population group

INTERVIEWER: Do you use the condom all the time you are training clients on Sayana?

RESPONDENT: We mostly use it with new clients because new clients are difficult to train because they are blank. But for them I already trained, we just conclude. We use sugar with condoms on new clients and some Sayana for demonstration.

Title: MZs_FAC_1.docx

Descriptor Info:	District: Mzimba South	Target Population: TP1 - Providers/HSAs	Respondent type: Facility-based provider	Aware of SI: Yes - had heard of SI before	Coding phase: 3. Triple coded	Client marital status: N/A - provider	Client age group: N/A - provider	Time since trained: A year or more ago

Codes Applied:	P3.3.1 - Differences in counselling/training approach by population group

Q 11. Interviewer: Alright. I just want to know that the way you can counsel a client on injectables is it differ if they had used injectables in the past?

Respondent: Who has chosen self -injection?

Interviewer: Yes.

Respondent: Yah, it differs.

Interviewer: How?

Respondent: Aaaah because on provider injection where we are the one injecting the client there not much effort on the client but to the provider who do the injection. But for Sayana we need to tell her how to do it herself, how to keep the medication and how to follow the dates, so those are the things and also you are going to say the advantages and the side effects though they are almost the same but you have to say them again .

Interviewer: What about if they were adolescent, were married/unmarried, if they wanted to delay pregnancy versus space or limit their pregnancies how would you counsel them?

Respondent: In that scenario we also need to counsel them those for short term methods and it is easier to stop when they want to have a child they can stop anytime they want and thus the advantages of using short term to them.

Interviewer: What if you were seeing them in the community versus seeing them at the facility, would it differ?

Respondent: No the counselling is the same.

Title: ZA_HSA_1.docx

Descriptor Info:	District: Zomba	Target Population: TP1 - Providers/HSAs	Respondent type: HSA	Aware of SI: Yes - had heard of SI before	Coding phase: 1. Single coded	Client marital status: N/A - provider	Client age group: N/A - provider	Time since trained: Less than a year ago

Codes Applied:	P3.3.1 - Differences in counselling/training approach by population group

Interviewer: The way you would counsel a client on injectables differ if they had used injectables in the past?

Respondent: Counselling?

Interviewer: Mmmmh

Respondent: Since they are already using the injection method, we just have to encourage to remember to come to the hospital when they are due or self-inject at home

Interviewer: Alright, what is they were adolescents or married or unmarried?

Respondent: Married or unmarried (giggles)

Interviewer: Mmmh

Respondent: Counselling wouldn’t be different since they are all taking the same method.

Interviewer: What if you were seeing them in the community versus seeing them at the facility?

Respondent: Here in the community?

Interviewer: Mmmh

Respondent: Aaah, my view is that it would be the same

Title: ZA_HSA_1.docx

Descriptor Info:	District: Zomba	Target Population: TP1 - Providers/HSAs	Respondent type: HSA	Aware of SI: Yes - had heard of SI before	Coding phase: 1. Single coded	Client marital status: N/A - provider	Client age group: N/A - provider	Time since trained: Less than a year ago

Codes Applied:	P3.3.1 - Differences in counselling/training approach by population group	A6 - Adol Perspectives/preferences for DMPA-SC SI

Interviewer: Alright, how do you help adolescents to better understand these things?

Respondent: Adolescents?

Interviewer: Mmmh, or have you ever come across an adolescent who self-injects?

Respondent: No I have never came across any adolescent who self-injects?

Interviewer: Alright alright thank you very much. How long to train a client in DMPA-SC?

Respondent: Ahhh probably 15 mins

Interviewer: Adolescents?

Respondent: Ahhh I have never met an adolescent

Interviewer: By the way you mentioned you have never met an adolescent

Respondent: (Giggles)

Title: MZs_HSA_2.docx

Descriptor Info:	District: Mzimba South	Target Population: TP1 - Providers/HSAs	Respondent type: HSA	Aware of SI: Yes - had heard of SI before	Coding phase: 1. Single coded	Client marital status: N/A - provider	Client age group: N/A - provider	Time since trained: A year or more ago

Codes Applied:	P3.3.1 - Differences in counselling/training approach by population group

Interviewer: would the counselling be similar for a woman on an initial visit versus a woman on subsequent visit

Respondent: no

Interviewer: what would be different

Respondent: for a first-time user we tell her to abstain from sex for the first 7 days. we give her condoms to use in those 7 days. For the one continuing we don’t give her condoms since she is continuing

Interviewer: what about for a married and unmarried woman, is the counselling similar?

Respondent: no

Interviewer: how do you differentiate

Respondent: we tell the unmarried to have condoms because the family planning will simply protect her from pregnancy and not STIs

Interviewer: what if it’s an adolescent is the counselling different from adults

Respondent: its somehow different. An adolescent might have a number of sexual partners. So we tell them that they can get STIs

Title: MZs_HSA_2.docx

Descriptor Info:	District: Mzimba South	Target Population: TP1 - Providers/HSAs	Respondent type: HSA	Aware of SI: Yes - had heard of SI before	Coding phase: 1. Single coded	Client marital status: N/A - provider	Client age group: N/A - provider	Time since trained: A year or more ago

Codes Applied:	P3.3.1 - Differences in counselling/training approach by population group	A6 - Adol Perspectives/preferences for DMPA-SC SI

Interviewer: what about for a married and unmarried woman, is the counselling similar?

Respondent: no

Interviewer: how do you differentiate

Respondent: we tell the unmarried to have condoms because the family planning will simply protect her from pregnancy and not STIs

Interviewer: what if it’s an adolescent is the counselling different from adults

Respondent: its somehow different. An adolescent might have a number of sexual partners. So we tell them that they can get STIs

Title: MZs_HSA_2.docx

Descriptor Info:	District: Mzimba South	Target Population: TP1 - Providers/HSAs	Respondent type: HSA	Aware of SI: Yes - had heard of SI before	Coding phase: 1. Single coded	Client marital status: N/A - provider	Client age group: N/A - provider	Time since trained: A year or more ago

Codes Applied:	C1.2.2 - Fear of SI (idea or reality)	A6 - Adol Perspectives/preferences for DMPA-SC SI	P3.3.1 - Differences in counselling/training approach by population group

Interviewer: what about adolescents?

Respondent: (giggles) they also do not necessarily have fears since we teach them how to use

Interviewer: what do you mean, for those who have fears what makes them so

Respondent: sometimes they do not understand clearly but we give them a chance to ask questions for clarifications. Some women think it’s a big needle but it’s not

Interviewer: mmmmh (thinking out loud) do you have any additions on fears adolescents have?

Respondent: no

Title: KK_FAC_2.docx

Descriptor Info:	District: Nkhotakota	Target Population: TP1 - Providers/HSAs	Respondent type: Facility-based provider	Aware of SI: Yes - had heard of SI before	Coding phase: 1. Single coded	Client marital status: N/A - provider	Client age group: N/A - provider	Time since trained: A year or more ago

Codes Applied:	P3.3.1 - Differences in counselling/training approach by population group	A6 - Adol Perspectives/preferences for DMPA-SC SI

INTERVIEWER: Alright. A client wants a contraception method right. The method that she wants is an injection. So the way you can counsel her, can it differ with a new client or counselling does not differ [did not ask this question the way it was supposed to be asked. I realized after his response and asked again].

RESPONDENT: Counselling is not the same. Counselling differs from person to person. We can say that counselling is the same according to a method. But there are some things that you can explain in one method that you cannot in another method.

INTERVIEWER: okay.

RESPONDENT: mmmmh.

INTERVIEWER: mmmhu.

RESPONDENT: it’s individualized?

INTERVIEWER: okay.

RESPONDENT: mmmmh.

INTERVIEWER: Alright. let us assume that I was on Depo and I stopped maybe for a year. I have come again and I want to start using Depo again right?

RESPONDENT: Depo?

INTERVIEWER: Yes, Depo. So the way you can counsel me, will it be the same with a new client or it will be different because I have used the injection before?

RESPONDENT: It will differ where to begin from. What will differ is where you are going to start from because for a new client, you explain to her about all the methods, and you let her choose the methods you have given her. But for this one who has used an injection before, the target will be on the methods that she wants.

INTERVIEWER: okay.

RESPONDENT: yeah.

INTERVIEWER: How about an adolescent and a woman, will the counselling be same…a girl and a woman, is the counselling the same or it differs?

RESPONDENT: We should say that what we say in counselling is…what can I say? Everything about the method is the same but there are some things that you have discussed with a woman you cannot discuss with an adolescent because they have their own languages, their dressing, they have their own behavior. So a provider is supposed to be like them.

INTERVIEWER: mmmhu.

RESPONDENT: Yea. That is why the message differs between women and adolescents.

INTERVIEWER: mmmh.

RESPONDENT: yeah.

Title: KK_FAC_2.docx

Descriptor Info:	District: Nkhotakota	Target Population: TP1 - Providers/HSAs	Respondent type: Facility-based provider	Aware of SI: Yes - had heard of SI before	Coding phase: 1. Single coded	Client marital status: N/A - provider	Client age group: N/A - provider	Time since trained: A year or more ago

Codes Applied:	P3.3.1 - Differences in counselling/training approach by population group	A6 - Adol Perspectives/preferences for DMPA-SC SI

INTERVIEWER: Alright. Mmmmh, do you teach adolescents about Sayana? Girls who are not married?

RESPONDENT: Not as an individual. But if they come here for contraception methods.

INTERVIEWER: mmmhu.

RESPONDENT: mmmh.
