# Supplementary material for: Health care provider and client experiences of counselling on depot medroxyprogesterone acetate subcutaneous (DMPA-SC) for self-injection in Malawi
Source: PLOS Glob Public Health. 2023 Nov 30;3(11):e0002057. doi: 10.1371/journal.pgph.0002057 (PMC10688740; doi:10.1371/journal.pgph.0002057)
Supplement: S1 Data — (ZIP) [file pgph.0002057.s001.zip › S2/P3.3.2 inclusion of practice opps.doc]

Title: ZA_HSA_2.docx

Descriptor Info:	District: Zomba	Target Population: TP1 - Providers/HSAs	Respondent type: HSA	Aware of SI: Yes - had heard of SI before	Coding phase: 3. Triple coded	Client marital status: N/A - provider	Client age group: N/A - provider	Time since trained: Less than a year ago

Codes Applied:	P3.3.2 - Inclusion of practice opportunities for clients in training

Respondent: When Sayana is available in right quantities we even take something, whatever, that we can demonstrate the way that the client can self-inject, yeah…

Interviewer: mmm (showing agreement)

Respondent: we can use tomato, or we can collect sand and fill it is a paper so that we demonstrate how we can self-inject, the degrees of injecting on the skin are 45, 90 something between that, but the needle is supposed to be in that position (Demonstrating on the arm with fingers the angle that the needle has to be) …

Title: KK_HSA_1.docx

Descriptor Info:	District: Nkhotakota	Target Population: TP1 - Providers/HSAs	Respondent type: HSA	Aware of SI: Yes - had heard of SI before	Coding phase: 2. Double coded	Client marital status: N/A - provider	Client age group: N/A - provider	Time since trained: A year or more ago

Codes Applied:	P3.2.2 - Stockouts/stocks near expiry dates	P3.3.2 - Inclusion of practice opportunities for clients in training

Interviewer: Mmmhu. So in your community. What has been the uptake of Sayana in the past three months…so…mmmm we are in October right? Mmmh. So we should say September, august, what has…what has been the uptake of Sayana for the past 3 months?

Respondent: Aaah, for the past 3 months, the uptake was a little bit good but we now have a…what can we say? Stock out? For Sayana

Interviewer: okay

Respondent: So we are using DMPA. So DMPA, a health worker is supposed to inject a client. You see? But if it is Sayana, you just train them, and then counsel them, then they self inject. The first dose, they inject in your presence, then the remaining doses you give them to self inject. But for this no matter the circumstance, we inject them for three months. It can not be possible for them to take home DMPA.so because of this shortfall that is why…maybe the uptake has decreased.[not clear] October…October we did not finish. So on the past three months, it is the last two months that we have used DMPA. The first month in these three months is the one we used Sayana.

Interviewer: Which means in July it was available?

Respondent: mmmh

Title: KK_HSA_1.docx

Descriptor Info:	District: Nkhotakota	Target Population: TP1 - Providers/HSAs	Respondent type: HSA	Aware of SI: Yes - had heard of SI before	Coding phase: 2. Double coded	Client marital status: N/A - provider	Client age group: N/A - provider	Time since trained: A year or more ago

Codes Applied:	P3.3.2 - Inclusion of practice opportunities for clients in training

Interviewer: Do you use any resources to help them easily understand?

Respondent: We use the same Sayana. We use the same injection. When…we have trained, we take one for…for demonstration.

Interviewer: mmmhu

Respondent: Yes. They shake and open the same one. Everything that a woman does, we use the same. But during our training they made this…what is this…it was a condom, what was that? So that is taken as what, a thigh. And it is loaded with sugar. It is like a person’s thigh or here [showing the area around the abdomen]. So they take the injection and hold what…the condom as a thigh or the abdomen right, and that’s it.

Interviewer: Which means you only train them with the real thigh and not the condom?

Respondent: yes

Interviewer: okay.

Respondent: But when teaching us the technique right, the one who was training us showed us. Maybe us we…we are relaxed. But it was like that.

Interviewer: mmhu.

Respondent: sure.

Title: KK_HSA_1.docx

Descriptor Info:	District: Nkhotakota	Target Population: TP1 - Providers/HSAs	Respondent type: HSA	Aware of SI: Yes - had heard of SI before	Coding phase: 2. Double coded	Client marital status: N/A - provider	Client age group: N/A - provider	Time since trained: A year or more ago

Codes Applied:	P3.3.2 - Inclusion of practice opportunities for clients in training	P4.1.1 - Follow up materials for client to take home

Interviewer: So you have given the women Sayana right, for self injection. You have said that you give her 3 doses. You inject her with one dose?

Respondent: She injects herself while you are observing her.

Interviewer: Yes, while you are observing her, and then you give her 3 doses right?

Respondent: Mmmh, yes for a year.

Interviewer: For a year.

Respondent: But if she is failing we give her one dose. She has self-injected here, we give her one to self-inject at home. So that when she is self-injecting the third one, you should be observing her.

Interviewer: mmmh

Respondent: yes. So that you should see if she is now competent. Is she really self-injecting?

Title: ZA_HSA_5.docx

Descriptor Info:	District: Zomba	Target Population: TP1 - Providers/HSAs	Respondent type: HSA	Aware of SI: Yes - had heard of SI before	Coding phase: 2. Double coded	Client marital status: N/A - provider	Client age group: N/A - provider	Time since trained: Less than a year ago

Codes Applied:	P3.3.2 - Inclusion of practice opportunities for clients in training

Respondent: The way they can self-inject up to the point where they can release the thigh properly, because the first time is always hard.

Interviewer: Mmm (showing that one is following the conversation)

Respondent: We have trained them and given them, you will then see that they are saying the liquid is not coming out, that time they have already injected themselves. So, we have to start explaining again that we have to activate the injection unit first and open it, so we try to illustrate that with a stick

Interviewer: Mmm (showing that one is following the conversation)

Respondent: Eh

Title: ZA_HSA_5.docx

Descriptor Info:	District: Zomba	Target Population: TP1 - Providers/HSAs	Respondent type: HSA	Aware of SI: Yes - had heard of SI before	Coding phase: 2. Double coded	Client marital status: N/A - provider	Client age group: N/A - provider	Time since trained: Less than a year ago

Codes Applied:	P3.3.2 - Inclusion of practice opportunities for clients in training

Respondent: because these days there is covid, we make sure they social distance and I explain to them while they watch and then I give them the units.

Interviewer: Mmm (showing that one is following the conversation)

Respondent: Try and do it, they shake it, and everyone opens it, I then tell them to hold their thigh, holding it then inject. And then they say it’s not hard, it’s not hard.

Interviewer: Yes.

Respondent: That’s when the people understood

Title: KK_FAC_1.docx

Descriptor Info:	District: Nkhotakota	Target Population: TP1 - Providers/HSAs	Respondent type: Facility-based provider	Aware of SI: Yes - had heard of SI before	Coding phase: 3. Triple coded	Client marital status: N/A - provider	Client age group: N/A - provider	Time since trained: A year or more ago

Codes Applied:	C1.2.2 - Fear of SI (idea or reality)	P3.3.2 - Inclusion of practice opportunities for clients in training

INTERVIEWER: Are women uncomfortable to inject themselves, after you train the and one choose the self-injection, I thought they are supposed to try the first on in your presence?

RESPONDENT: Yes, we need to monitor them

RESPONDENT:

INTERVIEWER: Yes, to see whether they are doing it the right way, do you think they are uncomfortable?

RESPONDENT: Yes, they seem nervous in the first trial

INTERVIEWER: Yes

RESPONDENT: Because it’s a new thing most people have never injected themselves

Title: ZA_FAC_1.docx

Descriptor Info:	District: Zomba	Target Population: TP1 - Providers/HSAs	Respondent type: Facility-based provider	Aware of SI: Yes - had heard of SI before	Coding phase: 2. Double coded	Client marital status: N/A - provider	Client age group: N/A - provider	Time since trained: Less than a year ago

Codes Applied:	P3.3 - Counselling/ training approach, general	P3.3.2 - Inclusion of practice opportunities for clients in training

Respondent: when we train the client, we tell her that OK it's up to you either we inject you or you self-inject since there are two options. so when the client is trained and sees that it has been demonstrated that she can be able to self-inject

Interviewer: mmmm (showing that one is following the conversation)

Respondent: they are supposed to self-inject while we are there and observing them

Interviewer: alright

Respondent: there is a specific location on the boarding that they are supposed to inject on, on the thigh, around the navel

Title: ZA_FAC_1.docx

Descriptor Info:	District: Zomba	Target Population: TP1 - Providers/HSAs	Respondent type: Facility-based provider	Aware of SI: Yes - had heard of SI before	Coding phase: 2. Double coded	Client marital status: N/A - provider	Client age group: N/A - provider	Time since trained: Less than a year ago

Codes Applied:	P3.3.2 - Inclusion of practice opportunities for clients in training

Interviewer: What about other resources like filling a condom with sand or sugar, do you use those resources?

Respondent: yes, we use that in the health talk demonstrations

Interviewer: mmmm (showing that one is following the conversation)

Respondent: eh we are supposed to demonstrate how the person self-injects.

Interviewer: mmmm (showing that one is following the conversation)

Respondent: we don’t do the demonstration on the woman but on an object, an object like filling up a condom with sand and then inject it, but the women play down that demonstration.

Interviewer: mmmm (showing that one is following the conversation)

Respondent: so we then say that I can demonstrate this on one woman and the woman will give feedback to the others.

Interviewer: mmmm (showing that one is following the conversation)

Respondent: so the resources help us but most of the times the women are reluctant that this is not a real thing, and they want a demonstration on the actual body.

Interviewer: eh eh

Respondent: so we don’t rely on it too much we just use when we want to show how to pinch the skin and inject slowly

Title: MZs_HSA_3.docx

Descriptor Info:	District: Mzimba South	Target Population: TP1 - Providers/HSAs	Respondent type: HSA	Aware of SI: Yes - had heard of SI before	Coding phase: 3. Triple coded	Client marital status: N/A - provider	Client age group: N/A - provider	Time since trained: Less than a year ago

Codes Applied:	C1.2.1 - Negative client attitudes to SI	C3.1.2 - Confidence/comfort to inject after counselling/ training	P88 - Provider great quotes	P3.3.2 - Inclusion of practice opportunities for clients in training

Q.12b Interviewer: what, in your experience is the response when you explain to clients that they could self-inject this method?

Respondent: most of them they refuse at first they say we don’t know then you say although you don’t know but we can explain to you I tell you what to do at the end you will know what to do. So you start explaining to her you show that chart she looks at that one you move together now we are here now we are here now you can get it. Can you do this one? So you do it she says no I will just do at home but no without practicing you cannot do at home but after I practice here you get it here you will do it at home so those who are unable to do what told them they fail to give themselves even if you can talk to them they will say aaah no aah ahh just give me I will come back and get it here. But those who can catch up early they say aaah let me do. After doing they say aah its simple I will go.

Title: MZs_HSA_3.docx

Descriptor Info:	District: Mzimba South	Target Population: TP1 - Providers/HSAs	Respondent type: HSA	Aware of SI: Yes - had heard of SI before	Coding phase: 3. Triple coded	Client marital status: N/A - provider	Client age group: N/A - provider	Time since trained: Less than a year ago

Codes Applied:	P4.1.3 - Provider availability/ willingness to repeat SI training	P88 - Provider great quotes	C8.1 - Male involvement, general	P3.3.2 - Inclusion of practice opportunities for clients in training

Q.16 Interviewer: if a client you had previously trained in SI returned to you without having completed her injection for some reason, what would your response be?

Respondent: ….she comes back she says I fail…I have to do. If she has said my husband doesn’t… or I don’t have space or I don’t my husband doesn’t allow it, I have to get that one back and start providing a self-provider given.

Interviewer: what question would you ask her?

Respondent: ….when did you get the last one? What has been the reason for you to come back here? So she she will either explain my husband had taken some of it so I have escaped with that one or she might say I was trying to give myself but it didn’t come so I have to know where she had problem aaah(thinking out loud) its either she didn’t push that one enough so even if she can push that one that one will not … take out that Sayana into her body so I have to take that responsibility of doing it as a provider now.

TIME STAMP 54-46- 1:09:31

Interviewer: what if she is still interested to do it at home?

Respondent: I have to re-train her again. Then after re-training her I should give her so that she should give herself here to prove that she can do it at a home but if she is failing again here she says I can’t do but you give me then I I cannot give her I have to say I have to insist on provider grounds not to give her back because even If I train her without doing she will not do it again.

Title: KK_HSA_2.docx

Descriptor Info:	District: Nkhotakota	Target Population: TP1 - Providers/HSAs	Respondent type: HSA	Aware of SI: Yes - had heard of SI before	Coding phase: 2. Double coded	Client marital status: N/A - provider	Client age group: N/A - provider	Time since trained: A year or more ago

Codes Applied:	P3.3.2 - Inclusion of practice opportunities for clients in training

INTERVIEWER: alright fine. what have you observed regarding response after counselling women a family planning method that requires them to inject themselves?

RESPONDENT: Yaa we normally advise them to try and inject themselves after counselling so that when they take the other doses they are able to do on their own when they get back home. instead of injecting them since they want remember to do that when they get back home which is a waste of counseling process

Title: ZA_HSA_3.docx

Descriptor Info:	District: Zomba	Target Population: TP1 - Providers/HSAs	Respondent type: HSA	Aware of SI: Yes - had heard of SI before	Coding phase: 1. Single coded	Client marital status: N/A - provider	Client age group: N/A - provider	Time since trained: A year or more ago

Codes Applied:	P3.1.3 - IEC/demo materials for counselling/training	C3.1.2 - Confidence/comfort to inject after counselling/ training	P3.3.2 - Inclusion of practice opportunities for clients in training

Interviewer: Alright, Do the women feel uncomfortable to inject themselves?

Respondent: Ahhhh... Many women do not feel uncomfortable about it since we teach them enough, we also use all the available resources to teach and they inject themselves first whole we are watching them, so they don't feel uncomfortable doing it.

Interviewer: Alright alright, what about adolescents?

Respondent: Same goes for the young women because they are free and comfortable because we teach them enough and during demonstration

Title: ZA_HSA_6.docx

Descriptor Info:	District: Zomba	Target Population: TP1 - Providers/HSAs	Respondent type: HSA	Aware of SI: Yes - had heard of SI before	Coding phase: 1. Single coded	Client marital status: N/A - provider	Client age group: N/A - provider	Time since trained:

Codes Applied:	P3.1.3 - IEC/demo materials for counselling/training	P3.2.1 - Lack of IEC/demo materials for counselling/training	P3.3.2 - Inclusion of practice opportunities for clients in training

Interviewer: Alright, ah in this job of training women on DMPA-SC, do you use any resources for example job aids like filling a condom with soil and practice self-injection on it, do you use any job aids?

Respondent: yes, we use, the time that we were using we took like a flexaform yeah and practiced self-injection on it how it works.

Interviewer: alright.

Respondent: that’s how we were doing when we just started, but now it seems like most of the clients have known how it works yeah

Interviewer: mmmm (showing that one is following the conversation)

Respondent: the past two months we didn’t take any resources to use in training.

Title: MZs_HSA_6.docx

Descriptor Info:	District: Mzimba South	Target Population: TP1 - Providers/HSAs	Respondent type: HSA	Aware of SI: Yes - had heard of SI before	Coding phase: 1. Single coded	Client marital status: N/A - provider	Client age group: N/A - provider	Time since trained: A year or more ago

Codes Applied:	P3.3 - Counselling/ training approach, general	P3.3.2 - Inclusion of practice opportunities for clients in training	P3.1.3 - IEC/demo materials for counselling/training	P4.1.1 - Follow up materials for client to take home

Interviewer: now I would like you to tell me how you would counsel a woman or an adolescent who has come to get injectable. I would like you to tell me from the time they come to the clinic until you finish with them. How do you greet a client when she has come to you?

Respondent: first we greet them; we introduce ourselves because many of them do not know us. we tell them that we are family planning providers. We tell them that whatever we discuss is kept confidential. Those who are free introduce themselves, where they are coming from and that they came to get family planning methods. Then we start counselling them using the counselling cards. Even if they choose Sayana we teach them on all methods. Then we get our checklist and ask the client questions. If she gets Sayana we wash our hands first. We counsel them on how to self-inject. Then we wash hands, put on a mask, then you are ready. We get the vial and show them. We counsel them on the mode of action of Sayana. We also give then dates for the next dose. Then we start demonstrating the procedure. We shake the vial slowly and touch it on the neck and close it. The closing opens the contents. Then we show them the area to inject on the abdomen, arm or thigh to pinch the skin and then self-Inject all the contents. We advise them not to rub on the area. They follow this procedure after us. we then give them some for home and a calendar. We circle the dates they are supposed to self-inject on the next doses.

Interviewer: how many methods would you counsel this woman

Respondent: come again

Interviewer: how many methods of injectable would you counsel this woman

Respondent: when she comes to me that she wants injectable, I would tell her that we have another method Sayana which a woman self-inject. So a woman will make a decision on which method she would want like self-injection

Interviewer: what questions would you ask during the counselling process

Respondent: have you ever had migraine headache? High blood pressure? When was your last menstrual period? Are you suspecting that you are pregnant? We ask many questions which are on the checklist.

Interviewer: apart from Sayana, which other method are you going to counsel this woman

Respondent: on family planning

Interviewer: yes, on the injectable methods

Respondent: I will counsel her on Depo IM which is given by the provider and Sayana for self-injection. So she will choose

Interviewer: after she chooses

Respondent: if she chooses let’s say Sayana for self-injection then I will counsel her on how to self-inject then supply Sayana with a calendar to take home. If she came with a spouse, we counsel them together.

Interviewer: what do you use when counselling

Respondent: like I have already said we use a counselling card

Interviewer: do you use anything else

Respondent: no, just a counselling card

Title: MZs_FAC_1.docx

Descriptor Info:	District: Mzimba South	Target Population: TP1 - Providers/HSAs	Respondent type: Facility-based provider	Aware of SI: Yes - had heard of SI before	Coding phase: 3. Triple coded	Client marital status: N/A - provider	Client age group: N/A - provider	Time since trained: A year or more ago

Codes Applied:	P88 - Provider great quotes	P3.3.2 - Inclusion of practice opportunities for clients in training

Interviewer: So I just want to know if there is different with the way a client understood the time you were using sugar and sand when counselling them and now that you are no longer use sugar and sand when counselling them, is it differ in terms of understanding?

Respondent: Yah, it can really be different because the demonstration with sugar and condom is to see how a client is doing it directly.

Interviewer: Mmmmm.

Respondent: And she is doing it you teach her and show her how to do it correctly, so it was one part would assure you that the client has understood the whole part.

Interviewer: Mmmmmmh

Respondent: But this time around we are just giving the pictures of illustration showing that you can just do like this, and this is the packet you have to open like this and you have to hold like this and it is shown here and we go step by step up until she understands and she has agreed that she can do it.

Interviewer: Mmmmmh

Respondent: So yah, it cannot give enough chance of a woman to practice sometimes it can lead to a failure in administering the drug at the end and instead of giving her self -injection you just do providers injection so that you should examine her again

Title: ZA_HSA_1.docx

Descriptor Info:	District: Zomba	Target Population: TP1 - Providers/HSAs	Respondent type: HSA	Aware of SI: Yes - had heard of SI before	Coding phase: 1. Single coded	Client marital status: N/A - provider	Client age group: N/A - provider	Time since trained: Less than a year ago

Codes Applied:	C1.2.2 - Fear of SI (idea or reality)	P3.1.2 - Provider skill/ability to effectively counsel on SI	P88 - Provider great quotes	P3.3.2 - Inclusion of practice opportunities for clients in training

Interviewer: Alright, do women have any concerns about self-injection?

Respondent: Concerns? Others fear wrongly injecting themselves

Interviewer: Mmmmh

Respondent: There are not worrisome concerns, fear is dominant. Once we watch them self-inject, they manage to do it

Interviewer: Alright, how do you overcome these women’s concerns. How do you remove the concerns?

Respondent: We just encourage them that nothing is impossible, and you will manage. We have closed watched you and you have now managed

Interviewer: Mmmh

Respondent: You will be able to manage
