# Supplementary material for: Health care provider and client experiences of counselling on depot medroxyprogesterone acetate subcutaneous (DMPA-SC) for self-injection in Malawi
Source: PLOS Glob Public Health. 2023 Nov 30;3(11):e0002057. doi: 10.1371/journal.pgph.0002057 (PMC10688740; doi:10.1371/journal.pgph.0002057)
Supplement: S1 Data — (ZIP) [file pgph.0002057.s001.zip › S2/P3.4 prov percept time to train.doc]

Title: ZA_HSA_2.docx

Descriptor Info:	District: Zomba	Target Population: TP1 - Providers/HSAs	Respondent type: HSA	Aware of SI: Yes - had heard of SI before	Coding phase: 3. Triple coded	Client marital status: N/A - provider	Client age group: N/A - provider	Time since trained: Less than a year ago

Codes Applied:	P3.4 - Time to counsel/train, general

Interviewer: How long on average does it take to train clients in DMPA-SC?

Respondent: Aaahh, the limit could be 45 minutes.

Interviewer: okay

Respondent: 30 to 45 minutes.

Interviewer: mmm (showing that one is following the conversation)

Respondent: This is because we try to give the people enough time so that they can understand and ask questions…

Interviewer: mmm (showing that one is following the conversation)

Respondent: But also, ah, sometimes we ask them to repeat what e have said in the training so that whatever we are teaching them sticks in their mind…

Respondent: Sure.

Title: ZA_HSA_2.docx

Descriptor Info:	District: Zomba	Target Population: TP1 - Providers/HSAs	Respondent type: HSA	Aware of SI: Yes - had heard of SI before	Coding phase: 3. Triple coded	Client marital status: N/A - provider	Client age group: N/A - provider	Time since trained: Less than a year ago

Codes Applied:	P3.4 - Time to counsel/train, general	P3.3.1 - Differences in counselling/training approach by population group	P3.2.3 - Complexity/difficulty of training clients in SI	A6 - Adol Perspectives/preferences for DMPA-SC SI

Interviewer: Alright, does the time that you spend training a client in DMPA-SC differ from one client to the next?

Respondent: Yes, they can be different.

Interviewer: mmm (showing that one is following the conversation)

Respondent: Because understanding is different. There are some people who can easily understand and some who take time to understand, they keep asking questions so that they can fully understand. So, the other ones we take more time with them even 45 minutes which I have mentioned may not be enough, yes, because the client wants to know more so we try to be with the for more time, but most of the times the time is between 30 to 45 minutes…

Interviewer: Alright.

Respondent: Sure, sure.

Interviewer: Alright, what about for adolescents.?

Respondent: mmm (showing that they are following the conversation)

Interviewer: Does the time that you take to train them differ compared to older women?

Respondent: Yes, it is possible like that, it can be like that, yes because girls, it is difficult that they should understand faster yeah,

Interviewer: mmm (showing that one is following the conversation)

Respondent: Compared to an older woman, because older women already know the things, and maybe they have already heard the things from somebody else, but mostly the adolescents don’t know most of the information relating to family planning…

Interviewer: Alright.

Respondent: Yes, if we are to do shortcut for those ones it is difficult…

Interviewer: mmm (showing that one is following the conversation)

Respondent: …if the find us we are required to be with them for the appropriate length of time to train them properly so that they appreciate the things that we have trained them…

Interviewer: mmm (showing that one is following the conversation)

Respondent: The we give them the family planning methods…

Title: ZA_HSA_2.docx

Descriptor Info:	District: Zomba	Target Population: TP1 - Providers/HSAs	Respondent type: HSA	Aware of SI: Yes - had heard of SI before	Coding phase: 3. Triple coded	Client marital status: N/A - provider	Client age group: N/A - provider	Time since trained: Less than a year ago

Codes Applied:	P3.4 - Time to counsel/train, general	P3.2 - Barrier, general/other

Interviewer: In your opinion, do you think you have enough time to provide training to DMPA-SC clients?

Respondent: Ah, time is not always enough, like we say some should come in the morning, some in the late afternoon hours, at least those clients that come in the late afternoon hours we don’t have time, here at the facility we don’t knock of late due to transport issues, yeah…

Interviewer: Yah yah

Respondent: Yes, so when the vehicle comes to pick us from the facility you will find that around 2PM or 3PM we are knocking off…

Interviewer: mm (showing that one is following the conversation)

Respondent: Yes, so starting from 2PM, 3PM up to 4:30PM or 5:00PM …

Interviewer: eh

Respondent: .is the time that we, the clients come, we got them used to that time…

Interviewer: mmm (showing that one is following the conversation)

Respondent: So, it depends what time the client has come to the facility, some of the clients come at dusk…

Interviewer: mm (showing that one is following the conversation)

Respondent: Yes, so at dusk it is very hard for us to train the client. But we are still able to assist the client because some of the clients come late because they already know the methods, yeah…

Interviewer: mm (Showing that one is following the conversation)

Respondent: and some of them have jobs, they go to work early and knock off late so we accept them, that they can come late here at the facility. But if they need to be trained and they want to know more…

Interviewer: mm (showing that one is following the conversation)

Respondent: We can still discuss with them,

Interviewer: mmmhh (showing that one is following the conversation)

Respondent: Sure, sure.

Interviewer: Alright alright, ah on the days that you are pre-occupied on your job, does the time that you spend with the clients on training them differ? Just comparing with the other days when you are not pre-occupied.

Respondent: ah to be honest, the thing is like that but in this area, we are not that busy a lot…

Interviewer: mm (showing that one is following the conversation)

Respondent: No.

Interviewer: Alright

Respondent: so, we have the time to chat with the clients…

Title: ZA_HSA_2.docx

Descriptor Info:	District: Zomba	Target Population: TP1 - Providers/HSAs	Respondent type: HSA	Aware of SI: Yes - had heard of SI before	Coding phase: 3. Triple coded	Client marital status: N/A - provider	Client age group: N/A - provider	Time since trained: Less than a year ago

Codes Applied:	P3.4 - Time to counsel/train, general	C3.4 - Time to train clients

Interviewer: Does that influence whether some women might choose to take up self-injection or not?

Respondent: ah When the training is longer…

Interviewer: mmmhh (showing that one is following the conversation)

Respondent: eh It makes some clients not to understand about the methods of family planning…

Interviewer: mmm (showing that one is following the conversation)

Respondent: They become bored yeah…

Interviewer: mmm (showing that one is following the conversation)

Respondent: Ah but when the training, when you tell them that the training will take 15 minutes or 20 minutes, it is enough to tell the client the main important points. Because there are some cases where you might prolong and keep on repeating the things that you have already mentioned, and you keep repeating them…

Interviewer: mmm (showing that one is following the conversation)

Respondent: The clients become bored with that…

Title: KK_HSA_1.docx

Descriptor Info:	District: Nkhotakota	Target Population: TP1 - Providers/HSAs	Respondent type: HSA	Aware of SI: Yes - had heard of SI before	Coding phase: 2. Double coded	Client marital status: N/A - provider	Client age group: N/A - provider	Time since trained: A year or more ago

Codes Applied:	P3.4 - Time to counsel/train, general

Interviewer: Mmmhu. How long does it take to train…train women who want Sayana for them to self-inject? How long does it take?

Respondent: It takes maybe 20 or 20…20-30 minutes.

Interviewer: One woman?

Respondent: grou…group.

Interviewer: Okay, can you explain

Respondent: When it is group counselling right, or I should just say all of them because you explain the good side of the method and its bad side. And maybe if takes…maybe there are questions or any worries. You want to assist the women. Maybe the actual time is 20 minutes. But if there are more questions, it takes 30 minutes if it is a group. If it is just one, one to one approximately it takes 20 or 15 minutes. It differs because with one person you loose time while if it is a group, you kill two birds with what…the same massage is reaching one person, the same message is reaching 10 people. And you will have more data here and less there.

(40:04)

Interviewer: mmmmhu.

Respondent: sure.

Interviewer: Do you think you have enough time for this training/counselling?

Respondent: mmmhu[responding or saying yes].

Interviewer: It is enough

Respondent: Mmmh, if it is an outreach, let us assume that started with the kids with the kids who came sick. You are dealing with the kids, and the women are increasing in number. Mmmh, there will be 6 or 7 women. So you will just counsel them together and inject them. And another phase comes, those who are late, you take them aside and assist them. Which means you will not take time for…a lot of time.

Interviewer: How about busy days?

Respondent: During busy days, we do shortcuts (laughs).

Interviewer: (laughs) eh eh eh

Respondent: During busy days, we also do the same thing. We start one session before this session. We wait for at least the women to be 10 or 8 or 7, where you feel that this a good number. You just teach them in one kick and then give them the methods. But for you to start one on one and being a busy day, it does not work.

Interviewer: okay.

Respondent: yes.

Title: ZA_HSA_5.docx

Descriptor Info:	District: Zomba	Target Population: TP1 - Providers/HSAs	Respondent type: HSA	Aware of SI: Yes - had heard of SI before	Coding phase: 2. Double coded	Client marital status: N/A - provider	Client age group: N/A - provider	Time since trained: Less than a year ago

Codes Applied:	P3.4 - Time to counsel/train, general

Interviewer: Alright, how long does it take to train the women in DMPA-SC?

Respondent: It does not take so much time.

Interviewer: Alright.

Respondent: Maybe 15 minutes

Interviewer: Alright.

Respondent: Yes.

Interviewer: Alright, ah does the time you take differ when you are training adolescents compared to older women?

Respondent: Yes, it differs

Interviewer: Alright.

Respondent: Adolescents do not take time to understand, they easily understand but the older women take time to understand

Interviewer: Mmm (showing that one is following the conversation)

Respondent: You need to slowly explain to them when you are training them step-by-step…

Interviewer: Eh

Respondent: …until you reach the end

Interviewer: eh, alright

Title: ZA_HSA_5.docx

Descriptor Info:	District: Zomba	Target Population: TP1 - Providers/HSAs	Respondent type: HSA	Aware of SI: Yes - had heard of SI before	Coding phase: 2. Double coded	Client marital status: N/A - provider	Client age group: N/A - provider	Time since trained: Less than a year ago

Codes Applied:	P3.4 - Time to counsel/train, general

Interviewer: Alright, do you think you have enough time to provide training on DMPA-SC?

Respondent: Ah for me the time is not enough. Yeah.

Interviewer: Mmm (showing that one is following the conversation)

Respondent: Because we have so many things to do.

Interviewer: Mmm (showing that one is following the conversation)

Respondent: Like yesterday we were the two of us so as I told you there were 100 family planning clients and others were also around 100

Interviewer: Mmm (showing that one is following the conversation)

Respondent: How many providers...

Interviewer: Two

Respondent: Two, so can you have enough time? You cannot…

Interviewer: Alright

Respondent: Eh

Title: ZA_HSA_5.docx

Descriptor Info:	District: Zomba	Target Population: TP1 - Providers/HSAs	Respondent type: HSA	Aware of SI: Yes - had heard of SI before	Coding phase: 2. Double coded	Client marital status: N/A - provider	Client age group: N/A - provider	Time since trained: Less than a year ago

Codes Applied:	P3.4 - Time to counsel/train, general

Interviewer: Alright does the length of the training session that you give to the women who are considering DMPA-SC influence whether a woman might choose self-injection?

Respondent: We try our best that our training should not take time so that the woman does not become bored because if it takes so much time then they are already thinking about their chores like going to the farm, what will I cook the kids, so when we take time, they become bored and they cannot take it. But we make it fast, and joyful so that they should also be attracted, and they understand what we want to communicate with them yeah

Interviewer: Mmm (showing that one is following the conversation)

Respondent: But when we take a long time then they will not consider it.

Title: KK_HSA_5.docx

Descriptor Info:	District: Nkhotakota	Target Population: TP1 - Providers/HSAs	Respondent type: HSA	Aware of SI: Yes - had heard of SI before	Coding phase: 1. Single coded	Client marital status: N/A - provider	Client age group: N/A - provider	Time since trained: A year or more ago

Codes Applied:	P3.4 - Time to counsel/train, general

INTERVIEWER: How long on average does it take to train a DMPA-SC client?

RESPONDENT: Counselling normally takes 30 to 45 minutes

INTERVIEWER: Is the counselling period for adults same as that of adolescent?

RESPONDENT: Just close

INTERVIEWER: Do you feel you have enough time to conduct these trainings? What about on busy days.

RESPONDENT: We do not have enough time; this is because we do counselling sessions in a group and then we go for one on one. So for busy day it tough only that due an increase in number for the HSA’s we have a relief because we share responsibilities.

Title: KK_HSA_5.docx

Descriptor Info:	District: Nkhotakota	Target Population: TP1 - Providers/HSAs	Respondent type: HSA	Aware of SI: Yes - had heard of SI before	Coding phase: 1. Single coded	Client marital status: N/A - provider	Client age group: N/A - provider	Time since trained: A year or more ago

Codes Applied:	P3.4 - Time to counsel/train, general	P3.1.2 - Provider skill/ability to effectively counsel on SI	P3.2 - Barrier, general/other	P4.1.3 - Provider availability/ willingness to repeat SI training

INTERVIEWER: Does the length of a training influence whether some women choose to take up self-injection?

RESPONDENT: The length of a training

INTERVIEWER: The process that takes in counselling the client does it influence whether some women choose to take up self-injection?

RESPONDENT: Yes, it does

INTERVIEWER: How does it influence?

RESPONDENT: When a client receives the right information through counselling

INTERVIEWER: Yes

RESPONDENT: Ndi pomweso amakhla ndi kuthekera kotenga

RESPONDENT: That’s when she has the urge to take the method. For instance, if the provider has counselled the clients so well in details, it becomes so easy for the clients when it comes for them to try to self-inject. But in cases where the provider does counsel the clients in a hurry without checking whether they are clear with the process. It becomes a problem for them when doing the trial injection.

INTERVIEWER: Alright

RESPONDENT: You may find some who will get the doses to take home, but because they did not understand well during counselling, they will just keep the units without using them. You may feel that thigs are fine, until when CBDA meets you to explain that your client is failing to use the injection you gave her, it’s when you discover that she just opened the unit. When asked she will tell you that the medication could not come out. Hence you start the counselling process all over again.

INTERVIEWER: Alright (Laugh)

RESPONDENT: Sure

Title: MZs_HSA_1.docx

Descriptor Info:	District: Mzimba South	Target Population: TP1 - Providers/HSAs	Respondent type: HSA	Aware of SI: Yes - had heard of SI before	Coding phase: 2. Double coded	Client marital status: N/A - provider	Client age group: N/A - provider	Time since trained:

Codes Applied:	P3.4 - Time to counsel/train, general	P3.1.5 - Ease of training clients in SI	P3.2.3 - Complexity/difficulty of training clients in SI

Q14. Interviewer: How long on average does it take to train a client on DMPA-SC?

Respondent: Mmmmmm… (thinking), 30 minutes but sometimes 30-45 minutes.

Interviewer: Why are you saying sometimes?

Respondent: It’s because clients are different, others it’s very easy to learn while others they are not

Interviewer: Alright. What about to the adolescent?

Respondent: It’s the same as adults

Interviewer: Is it the same?

Respondent: Yes.

Interviewer: Do you feel you have enough time to conduct these trainings?

Respondent: Yes.

Title: MZs_HSA_1.docx

Descriptor Info:	District: Mzimba South	Target Population: TP1 - Providers/HSAs	Respondent type: HSA	Aware of SI: Yes - had heard of SI before	Coding phase: 2. Double coded	Client marital status: N/A - provider	Client age group: N/A - provider	Time since trained:

Codes Applied:	P3.4 - Time to counsel/train, general

Interviewer: Thanks. Does the length of training influence whether a women choose to take up self-injection?

Respondent: Yes, spending much time with the client will help her to understand the whole process on how she can inject herself and it can influence her in taking self-injection option.

Title: KK_FAC_1.docx

Descriptor Info:	District: Nkhotakota	Target Population: TP1 - Providers/HSAs	Respondent type: Facility-based provider	Aware of SI: Yes - had heard of SI before	Coding phase: 3. Triple coded	Client marital status: N/A - provider	Client age group: N/A - provider	Time since trained: A year or more ago

Codes Applied:	P3.4 - Time to counsel/train, general

INTERVIEWER: Okay fine, What about if they were an adolescent, were married /unmarried, if they wanted to take Sayana would the counselling differ?

RESPONDENT: The counselling will not that much, however, in case of the adolescence we take some time when counselling them.

INTERVIEWER: Okay

RESPONDENT: Because they rush in making up a decision. Hence, we make sure that we go deeper in counselling them so that they make a sound decision. Because some may get the method but they may not use the injection. That’s what most adolescent do.

Title: KK_FAC_1.docx

Descriptor Info:	District: Nkhotakota	Target Population: TP1 - Providers/HSAs	Respondent type: Facility-based provider	Aware of SI: Yes - had heard of SI before	Coding phase: 3. Triple coded	Client marital status: N/A - provider	Client age group: N/A - provider	Time since trained: A year or more ago

Codes Applied:	P3.4 - Time to counsel/train, general	P3.3 - Counselling/ training approach, general

INTERVIEWER: How long on average does it train a client in DMPA-SC?

RESPONDENT: We take a maximum of ten minutes for someone to clearly understand

INTERVIEWER: Yes

RESPONDENT: The bigger part is already done during group counselling

INTERVIEWER: Yes

RESPONDENT: In a counselling room we mainly dwell on technical part of it like teaching on how one can effectively inject herself. Though during group counselling we do some demonstration but when we have the client alone we go deeper so that they master the method properly.

INTERVIEWER: What about for adolescents whom you said they easily capture information, how would you compare the time you spend with then in counselling with adults .

RESPONDENT: For the adolescents it little time

INTERVIEWER: Its little time not so?

RESPONDENT: Yes

INTERVIEWER: Do you feel you have enough time to conduct these trainings? what about on busy days?

RESPONDENT: If always have time since the client is here for help

INTERVIEWER: Sure but is the time always enough to train the client’s when they visit the facility?

RESPONDENT: may be 5 to 6 people at time. For example Thursday is a busy day since it’s a family planning day hence we have a lot of work to do.

Title: KK_FAC_1.docx

Descriptor Info:	District: Nkhotakota	Target Population: TP1 - Providers/HSAs	Respondent type: Facility-based provider	Aware of SI: Yes - had heard of SI before	Coding phase: 3. Triple coded	Client marital status: N/A - provider	Client age group: N/A - provider	Time since trained: A year or more ago

Codes Applied:	A6 - Adol Perspectives/preferences for DMPA-SC SI	P3.4 - Time to counsel/train, general	P3.3.1 - Differences in counselling/training approach by population group

INTERVIEWER: What about for adolescents whom you said they easily capture information, how would you compare the time you spend with then in counselling with adults .

RESPONDENT: For the adolescents it little time

INTERVIEWER: Its little time not so?

RESPONDENT: Yes

Title: KK_FAC_1.docx

Descriptor Info:	District: Nkhotakota	Target Population: TP1 - Providers/HSAs	Respondent type: Facility-based provider	Aware of SI: Yes - had heard of SI before	Coding phase: 3. Triple coded	Client marital status: N/A - provider	Client age group: N/A - provider	Time since trained: A year or more ago

Codes Applied:	P3.3 - Counselling/ training approach, general	P3.4 - Time to counsel/train, general

INTERVIEWER: Does the length of the training influence whether some women choose to take up self-injection?

RESPONDENT: It is a bit tricky to know how much time is spend on a particular method since counselling is done in a group

INTERVIEWER: Ok

RESPONDENT: because we train women in a group and then makes a choice on the method they want.

INTERVIEWER: Ok the from that point?

RESPONDENT: Yes

Title: MZs_HSA_5.docx

Descriptor Info:	District: Mzimba South	Target Population: TP1 - Providers/HSAs	Respondent type: HSA	Aware of SI: Yes - had heard of SI before	Coding phase: 1. Single coded	Client marital status: N/A - provider	Client age group: N/A - provider	Time since trained: A year or more ago

Codes Applied:	P3.4 - Time to counsel/train, general

Interviewer: how long do you take to counsel a client?

Respondent: it depends with the understanding of the client. Some grasp the content faster than some

Interviewer: like how many minutes?

Respondent: 20-25 minutes

Interviewer: even with adolescents?

Respondent: it’s less than that

Interviewer: like how many minutes?

Respondent: about 15 minutes you are done

Interviewer: do you have enough time to counsel women and adolescents on self-injection?

Respondent: we divide our time making sure we spend enough time with each client so that we avoid consequences because they might get unwanted pregnancy because of inadequate counselling

Title: KK_HSA_3.docx

Descriptor Info:	District: Nkhotakota	Target Population: TP1 - Providers/HSAs	Respondent type: HSA	Aware of SI: Yes - had heard of SI before	Coding phase: 3. Triple coded	Client marital status: N/A - provider	Client age group: N/A - provider	Time since trained: A year or more ago

Codes Applied:	P3.4 - Time to counsel/train, general	P3.3 - Counselling/ training approach, general

INTERVIEWER: How long does it take right, when a woman comes? How long does it take to train this client for you to know that this client will self-inject? How long does it take?

RESPONDENT: Okay. Aaa depending on how we train the women. We don’t have a specific time that maybe 2hours, 3hours, 4hours. But it depends on the training and the practical session if the women will be able to self-inject. So we cannot say that maybe 2hours, 3hours. But it depends on how serious they are and if they are interested…

INTERVIEWER: Yes continue

RESPONDENT: That these ones will be able to self-inject.

INTERVIEWER: But on average…because you have trained a lot of women, right?

RESPONDENT: mmmh.

INETRVIEWER: How long do you think it takes?

RESPONDENT: 2hours.

INTERVIEWER: 2hours?

RESPONDENT: mmmh.

INTERVIEWR: One woman?

RESPONDENT: mmmh.

INTERVIEWER: To be able to self-inject?

RESPONDENT: mmmmh.

INTERVIEWER: mmm. Do you feel you have…do you feel you have enough time…do you have enough time to conduct these trainings?

RESPONDENT: Yes, we have enough time because when we arrive for example at 8 o’clock, we know that we will be there until 2 o’clock. I finish training all the women within these hours.

INTERVIEWER: What happens on busy days? You are in your community and it’s a busy day, what happens?

RESPONDENT: Okay. The day I am in my community to do other work, it’s the same day I am working with under-five children, right? When I am done with the underfive clinic for example those with malaria and other issues, I also spare sometime for women on family planning. So maybe I can meet 5 or 6 women depending on the ones who want contraceptives. And I know that now I have this number of women. These 2 hours are for all the women. I do not counsel them separately for 2hours each. If there are 5 or 6 or 10 women, I train them together. when I counsel then together, I also make time to see if see them individuality if they will be able to self-inject. “Can you do it? Try I should see” [asking the client] We have resources for practice. The 2hours is enough to train the women in group counselling.

Title: KK_HSA_3.docx

Descriptor Info:	District: Nkhotakota	Target Population: TP1 - Providers/HSAs	Respondent type: HSA	Aware of SI: Yes - had heard of SI before	Coding phase: 3. Triple coded	Client marital status: N/A - provider	Client age group: N/A - provider	Time since trained: A year or more ago

Codes Applied:	P3.4 - Time to counsel/train, general

INTERVIEWER: No. how about the length of the training, this 2hours right? does it influence women to say no to this contraceptive?

RESPONDENT: No. the length of this 2hours does not make women not to take up this method. This is when we are teaching/counselling them about the method. And those who have chosen this method, are the ones we now explain to in detail.

Title: ZA_HSA_4.docx

Descriptor Info:	District: Zomba	Target Population: TP1 - Providers/HSAs	Respondent type: HSA	Aware of SI: Yes - had heard of SI before	Coding phase: 1. Single coded	Client marital status: N/A - provider	Client age group: N/A - provider	Time since trained: A year or more ago

Codes Applied:	P3.4 - Time to counsel/train, general

Interviewer: Alright. Thank you so much. How long does it take train the women taking the self-injecting contraception method?

Respondent: Mmmm it takes a long time, about 10 minutes because there might be a group of them. So, we encourage them about this method and why it is a good method to be using.

Interviewer: Do you think the time is enough to counsel the women?

Respondent: Aaah as for me I feel that the time is enough because these women when they find us in the villages, it’s not that this time is for these lessons, they find us busy with some other things, so we do not have enough time for us to discuss about this method. We usually give them time to come visit us so that we may have enough time to talk about the method. They do not show up at the end of the day. But also, it is different when they visit as in the hospitals even though time is not always enough for us.

Interviewer: Is there any other thing you want to add?

Respondent: Aaaah no.

Title: KK_HSA_4.docx

Descriptor Info:	District: Nkhotakota	Target Population: TP1 - Providers/HSAs	Respondent type: HSA	Aware of SI: Yes - had heard of SI before	Coding phase: 1. Single coded	Client marital status: N/A - provider	Client age group: N/A - provider	Time since trained: A year or more ago

Codes Applied:	P3.4 - Time to counsel/train, general

NTERVIEWER: Alright, how long on average does it take to train a DMPA-SC client?

RESPONDENT: We would put it at 25 to 30 minutes

INTERVIEWER: aright 25 to 30 minutes fine. Do you feel you have enough time to conduct these trainings? What about on busy days.

RESPONDENT: In true sense we don’t have enough time, because if we consider us as HSAs we normally have integrated activates like sometimes we also do village clinics.

INTERVIEWER: Alright

RESPONDENT: It happens that we are conducting under-five clinics abut at the same time the same women would like us to assist then in providing them family planning services.

INTERVIEWER: Ok

RESPONDENT: We don’t have enough time as already explained that, only on counselling we spent 25 to 30 minutes to discuss with the women, and the counsel them and give them contraceptives.

INTERVIEWER: Alright

RESPONDENT: As such I feel we don’t have enough time

INTERVIEWER: Ok

RESPONDENT: Yes

Title: KK_HSA_4.docx

Descriptor Info:	District: Nkhotakota	Target Population: TP1 - Providers/HSAs	Respondent type: HSA	Aware of SI: Yes - had heard of SI before	Coding phase: 1. Single coded	Client marital status: N/A - provider	Client age group: N/A - provider	Time since trained: A year or more ago

Codes Applied:	P3.4 - Time to counsel/train, general

RESPONDENT: But you would note that most women are sticking on one particular method.

INTERVIEWER: Ok

RESPONDENT: It makes send more of our time on the things that women will have nothing to do about. I feel counselling just takes our time because we will explain some things that will not even interest them.

INTERVIEWER: Alright.

Title: MZs_HSA_4.docx

Descriptor Info:	District: Mzimba South	Target Population: TP1 - Providers/HSAs	Respondent type: HSA	Aware of SI: Yes - had heard of SI before	Coding phase: 1. Single coded	Client marital status: N/A - provider	Client age group: N/A - provider	Time since trained:

Codes Applied:	P3.4 - Time to counsel/train, general

Respondent: this exercise is not simple such that sometimes it is very tiresome. We are supposed to go for village outreach, and it is done once a week. Sometimes clients come to our homes when they have missed the designated day or when the due date has arrived before the designated date to access the method. The clients come to our homes since we live in the same catchment area.

Title: MZs_HSA_4.docx

Descriptor Info:	District: Mzimba South	Target Population: TP1 - Providers/HSAs	Respondent type: HSA	Aware of SI: Yes - had heard of SI before	Coding phase: 1. Single coded	Client marital status: N/A - provider	Client age group: N/A - provider	Time since trained:

Codes Applied:	P3.4 - Time to counsel/train, general

Q14. Interviewer: how long does it take to teach clients about self-injection?

Respondent: it takes about 15 to 20 minutes for a person to understand because the process also involves demonstration.

Interviewer: is this time allocated to those already married or the youth?

Respondent: this time is spent mostly with those who are already married. However, sometimes it takes more than 20 minutes to teach and demonstrate self-injection.

Interviewer: how long can it take?

Respondent: May be 30 minutes.

Interviewer: is this 30 minutes spent when counselling the youth or the older clients?

Respondent: this is allocated to the older clients. The youth easily understand.

Interviewer: mmmmh

Respondent: sometimes I can spend more time counselling the youth as compared to counselling the older clients because the latter may have had already heard about the method of family planning. While the youth may only be conversant with the condom method. So we may spend a little more time to teach the other methods.

Interviewer: do you think you have enough time to offer the method?

Respondent: yes

Interviewer: are you sure?

Respondent: yes

Interviewer: is this time enough even during busy days?

Respondent: in the field we may reschedule so that we should meet the clients next time.

Interviewer: Mmmmmh

Respondent: we also tell the busy clients to come again the next day.

Title: ZA_FAC_1.docx

Descriptor Info:	District: Zomba	Target Population: TP1 - Providers/HSAs	Respondent type: Facility-based provider	Aware of SI: Yes - had heard of SI before	Coding phase: 2. Double coded	Client marital status: N/A - provider	Client age group: N/A - provider	Time since trained: Less than a year ago

Codes Applied:	P3.2.3 - Complexity/difficulty of training clients in SI	P3.4 - Time to counsel/train, general

Interviewer: Alright, have you experienced any problems in training women on DMPA-SC?

Respondent: yah I think the many questions, when we start training women on Sayana we just know that we will spend more time with them due to the fears that are there.

Interviewer: yah yah

Respondent: since it’s a new thing and the clients cannot just accept it on the word go.

Interviewer: mmmm (showing that one is following the conversation)

Respondent: the client already knows about the other methods, but for Sayana they say that it’s to them and you need to take time on it, but still more there is a benefit in that.

Title: ZA_FAC_1.docx

Descriptor Info:	District: Zomba	Target Population: TP1 - Providers/HSAs	Respondent type: Facility-based provider	Aware of SI: Yes - had heard of SI before	Coding phase: 2. Double coded	Client marital status: N/A - provider	Client age group: N/A - provider	Time since trained: Less than a year ago

Codes Applied:	P3.4 - Time to counsel/train, general

Interviewer: How long does it take to train women on DMPA-SC?

Respondent: eh initially I think for the first three months

Interviewer: mmmm (showing that one is following the conversation)

Respondent: We would spend almost 45 minutes

Interviewer: mmmm (showing that one is following the conversation)

Respondent: 40 to 45 minutes

Interviewer: yah yah

Respondent: but after the first three months we spend 15 minutes

Interviewer: alright.

Respondent: if its beyond 15 minutes, because there are only a few new clients, in the middle of the training we ask if there are any new clients, those ones we spend more time with since we need to check their preexisting conditions before we give them the contraceptives

Interviewer: mmmm (showing that one is following the conversation)

Respondent: for those that are old clients we don’t spend more time with them since they already know a lot

Interviewer: mmmm (showing that one is following the conversation)

Respondent: 15 minutes is not enough

Interviewer: alright.

Respondent: yes

Title: ZA_FAC_1.docx

Descriptor Info:	District: Zomba	Target Population: TP1 - Providers/HSAs	Respondent type: Facility-based provider	Aware of SI: Yes - had heard of SI before	Coding phase: 2. Double coded	Client marital status: N/A - provider	Client age group: N/A - provider	Time since trained: Less than a year ago

Codes Applied:	P3.4 - Time to counsel/train, general

Interviewer: Do you think you have enough time to provide training on DMPA-SC?

Respondent: exactly we don’t have enough time

Interviewer: alright

Respondent: yes

Title: ZA_FAC_2.docx

Descriptor Info:	District: Zomba	Target Population: TP1 - Providers/HSAs	Respondent type: Facility-based provider	Aware of SI: Yes - had heard of SI before	Coding phase: 3. Triple coded	Client marital status: N/A - provider	Client age group: N/A - provider	Time since trained: Less than a year ago

Codes Applied:	P3.4 - Time to counsel/train, general

Interviewer: How long does it take for you to teach women who do family planning using the self-injection method?

Respondent: 30 minutes.

Interviewer: 30 minutes each person?

Respondent: No, since we’re teaching them as a group. But when its one-to-one counselling it takes 10 to 15 minutes.

Interviewer: Fine. How about for the youth?

Respondent: For the youth, it doesn’t reach 30 minutes, roughly 20 minutes.

Interviewer: In your opinion, do you think the time you have is enough to deliver the counselling?

Respondent: No, it’s not enough. It requires more time maybe 1 hour. But since the place is usually crowded. So to give the healthy talk for one hour let’s say we begin work 8 o’clock to 9 o’clock but we receive a lot of people here. In a week we sometimes have data showing that we have attended to 200 or a hundred and ninety something people.

Title: ZA_FAC_2.docx

Descriptor Info:	District: Zomba	Target Population: TP1 - Providers/HSAs	Respondent type: Facility-based provider	Aware of SI: Yes - had heard of SI before	Coding phase: 3. Triple coded	Client marital status: N/A - provider	Client age group: N/A - provider	Time since trained: Less than a year ago

Codes Applied:	P3.4 - Time to counsel/train, general	A6 - Adol Perspectives/preferences for DMPA-SC SI

Interviewer: Fine. How about for the youth?

Respondent: For the youth, it doesn’t reach 30 minutes, roughly 20 minutes.

Interviewer: In your opinion, do you think the time you have is enough to deliver the counselling?

Title: ZA_FAC_2.docx

Descriptor Info:	District: Zomba	Target Population: TP1 - Providers/HSAs	Respondent type: Facility-based provider	Aware of SI: Yes - had heard of SI before	Coding phase: 3. Triple coded	Client marital status: N/A - provider	Client age group: N/A - provider	Time since trained: Less than a year ago

Codes Applied:	P3.4 - Time to counsel/train, general

Interviewer: Is there a connection between the duration of the counselling and the choice women make to do the self-injection family planning method?

Respondent: It helps since we’re explaining everything needed to know. Thus they make a decision based on enough information.

Title: MZs_HSA_3.docx

Descriptor Info:	District: Mzimba South	Target Population: TP1 - Providers/HSAs	Respondent type: HSA	Aware of SI: Yes - had heard of SI before	Coding phase: 3. Triple coded	Client marital status: N/A - provider	Client age group: N/A - provider	Time since trained: Less than a year ago

Codes Applied:	P3.4 - Time to counsel/train, general	C1.1.2 - Baseline correct knowledge/ awareness of injectables

Q.11 Interviewer: Would the way you counsel the client on injectable differ if they had used injectable in the past?

Respondent: [silent moment]….. because that one is a subsequent the will differ because she is already used to……… that counselling but the new.. the one who is new has to be counselled in all.

Interviewer: what would differ?

Respondent: the time of counselling… mmmh because that one huuuu…knows already what is to be done because when I ask her this one is done like this this one is done like that but the new client will not have anything in her head.

Title: MZs_HSA_3.docx

Descriptor Info:	District: Mzimba South	Target Population: TP1 - Providers/HSAs	Respondent type: HSA	Aware of SI: Yes - had heard of SI before	Coding phase: 3. Triple coded	Client marital status: N/A - provider	Client age group: N/A - provider	Time since trained: Less than a year ago

Codes Applied:	P3.4 - Time to counsel/train, general

Q.14 Interviewer: how long on average does it take to train clients in DMPA-SC?

Respondent: on self- injection…. its about 10-20 minutes yah depending on how that one can understand yah

Interviewer: what about specifically for adolescents?

Respondent: that one can take almost 30 minutes I think because it requires repeating you have to repeat again so that she accept.

Interviewer: Do you have feel you have enough time to conduct these trainings? What about on busy days?

Respondent: yes. [Moment of silence]..on busy days, mmmmh it depends on the busy I have. If requires me to be at this place I can shift…..

Interviewer: is there anything that could help you with training clients in SI?

Respondent: aaah I don’t think so… they are enough

Title: MZs_HSA_3.docx

Descriptor Info:	District: Mzimba South	Target Population: TP1 - Providers/HSAs	Respondent type: HSA	Aware of SI: Yes - had heard of SI before	Coding phase: 3. Triple coded	Client marital status: N/A - provider	Client age group: N/A - provider	Time since trained: Less than a year ago

Codes Applied:	P3.4 - Time to counsel/train, general	C1.1.3 - Awareness of DMPA-SC SI	C2.1.2 - Reduced trips to facility

Interviewer: Does the length of training influence whether some women choose to take up self-injection?

Respondent: yes. Yah! Because the they might be 2 so seeing that the other one is getting Sayana the other one got depo. So when she says what what about this one so we say this is Sayana has got these these advantages it’s like this it’s done like these somebody just gets it at home you will inject it at home so those who got depo somewhere they say aaah no therefore am am also able I can go and get.. [interrupted by noise the recording was paused] ndipitilize? ….where I was, I got Depo but today am hearing about Sayana the advantages which are there to I may stay home for almost a year without coming here now let me try Sayana.

Title: KK_HSA_2.docx

Descriptor Info:	District: Nkhotakota	Target Population: TP1 - Providers/HSAs	Respondent type: HSA	Aware of SI: Yes - had heard of SI before	Coding phase: 2. Double coded	Client marital status: N/A - provider	Client age group: N/A - provider	Time since trained: A year or more ago

Codes Applied:	P3.4 - Time to counsel/train, general	P3.3 - Counselling/ training approach, general

INTERVIEWER: How easy or difficult is it to train clients in DMPA-SC for self injection

RESPONDENT: aaaa mostly the most difficult part is the questions that the clients asks since they want to know more

INTERVIEWER: But in your experience do you find it difficult or not to counsel clients on self injection

RESPONDENT: no I don’t

INTERVIEWER: For you to counsel them

RESPONDENT No we don’t have difficulties

INTERVIEWER: I recall you said you said there are job aids that you use in your job.

RESPONDENT Yes

INTERVIEWER: How long on average does it take to train clients in DMPA-SC

RESPONDENT : The most painful thing is that women do not come in groups, you may find that there is only one who is present and you start counselling her, as you are about to finish you find out that there is also another client who wants counselling as well. Hence you have to start all over again

INTERVIEWER: Mhhh Mhh

RESPONDENT : And sometimes you just find yourselves doing short cuts

INTERVIEWER: Hehehe repeating the same process again and again.

RESPONDENT : yaah

INTERVIEWER: Do you feel you have enough time to conduct these trainings? And what about on busy days and you have more clients?

RESPONDENT: In that case one would not have enough time, unless when we are doing under five clinics then we target the mothers and counsel them on family planning methods.

INTERVIEWER: Alright

RESPONDENT: Sure doing under five clinics they get counselling in large numbers which is better than at the health centre.

INTERVIEWER: Alright

RESPONDENT : Yes, because it becomes so difficult when dealing with one client at a time than in a group. This is why nurses don’t like doing family planning counselling.

INTERVIEWER: Mhhhh

RESPONDENT: They say they can't be doing the same things again and again.

Title: KK_HSA_2.docx

Descriptor Info:	District: Nkhotakota	Target Population: TP1 - Providers/HSAs	Respondent type: HSA	Aware of SI: Yes - had heard of SI before	Coding phase: 2. Double coded	Client marital status: N/A - provider	Client age group: N/A - provider	Time since trained: A year or more ago

Codes Applied:	P3.4 - Time to counsel/train, general

INTERVIEWER: Okay Fine, does the length of training influence whether some women choose to take up self-injection?

RESPONDENT : Ammm it depends

INTERVIEWER: My question was does the length of a training influence whether some women choose to take up self -injection, for instance are the trainings for Sayana long than other family planning methods that affect a woman to choose whether to take Sayana or not

RESPONDENT : All this depends on how the woman is grasping the message. Sometimes you may train a woman in Sayana for very short period of time and you are done, similarly in short period of time you may also train a woman in Depo and be done in a short period of time. In addition it also depends on the person providing counselling to the woman on how best you’re counselling the woman will determine the understanding of the whole concept.

Title: ZA_HSA_3.docx

Descriptor Info:	District: Zomba	Target Population: TP1 - Providers/HSAs	Respondent type: HSA	Aware of SI: Yes - had heard of SI before	Coding phase: 1. Single coded	Client marital status: N/A - provider	Client age group: N/A - provider	Time since trained: A year or more ago

Codes Applied:	P3.4 - Time to counsel/train, general

Interviewer: How long does it take to train a woman?

Respondent: It takes ten to fifteen minutes.

Interviewer: How about when training the adolescents?

Respondent: Same time we also take ten to fifteen minutes

Interviewer: Alright thank you very much, is the time always ten to fifteen minutes? Does it not change?

Respondent: No the amount of time used for teaching does not change but sometimes it does due to questions received from the client and the need for them to fully understand but by the mentioned time both the training and demonstrations are supposed to be complete

Interviewer: Alright, do you think the time is enough for the required training?

Time stamp: 29:56

Respondent: Yes, the time is always enough but as I have already said, the time is adjusted because the client did not understand or if they have questions.

Interviewer: Alright alright, how do you manage work on busy days?

Respondent: On our busy days?

Interviewer: Mhhmmm...

Respondent: You mean us from environmental or??

Interviewer: How do you offer the counselling and training on your busy day?

Respondent: Clients are sent to the nursing department where there is a person with training in the same.

Interviewer: Alright

Respondent: Or we work hand in hand as such they also send clients to our department when they are busy.

Title: ZA_HSA_3.docx

Descriptor Info:	District: Zomba	Target Population: TP1 - Providers/HSAs	Respondent type: HSA	Aware of SI: Yes - had heard of SI before	Coding phase: 1. Single coded	Client marital status: N/A - provider	Client age group: N/A - provider	Time since trained: A year or more ago

Codes Applied:	P3.4 - Time to counsel/train, general

Interviewer: Alright thank you, Does the period of time it takes to train women on how to use the method affect the choice of women on whether or not they should us the self-injection method?

Respondent: You mean the training that we offer them?

Interviewer: Yes

Respondent: No like I have already said that it takes ten to fifteen minutes to finish offering the lessons then it does not stop them from using the m

Title: ZA_HSA_6.docx

Descriptor Info:	District: Zomba	Target Population: TP1 - Providers/HSAs	Respondent type: HSA	Aware of SI: Yes - had heard of SI before	Coding phase: 1. Single coded	Client marital status: N/A - provider	Client age group: N/A - provider	Time since trained:

Codes Applied:	P1.2.1 - Negative provider attitudes towards SI	P1.1.1 - Positive provider attitudes towards SI	P3.4 - Time to counsel/train, general

Interviewer: Alright, I am curious on what you have experienced in providing self-injection contraceptive at this facility and in your community. Ah when you and your colleagues first heard about the possibility of a woman self-injecting, how did you feel?

Respondent: at first, I thought that it would be difficult.

Interviewer: mmmm (showing that one is following the conversation)

Respondent: But after we did the practical it showed that the number of women who self-inject is starting to increase gradually. But most of them were worried that since a person is self-injecting they thought that they will not be doing it right, so there still required counselling, that the trainer should have enough counselling so that when they have been trained they should get the correct information because sometimes maybe the training that we do is very fast due to a time factor, but if the ones being trained, that we have people with great skill who can also train their friends it can be helpful so that there should be no problems.

Title: ZA_HSA_6.docx

Descriptor Info:	District: Zomba	Target Population: TP1 - Providers/HSAs	Respondent type: HSA	Aware of SI: Yes - had heard of SI before	Coding phase: 1. Single coded	Client marital status: N/A - provider	Client age group: N/A - provider	Time since trained:

Codes Applied:	P3.1 - Enablers, general/other	P3.2 - Barrier, general/other	P3.4 - Time to counsel/train, general

Interviewer: alright would the counselling differ if you are meeting the clients in the community or at the facility?

Respondent: ah it is a bit different.

Interviewer: mmmm (showing that one is following the conversation)

Respondent: because when we meet them in the community, they are ready that the providers are coming.

Interviewer: mmmm (showing that one is following the conversation)

Respondent: what am saying is that past 8 AM thy have already started gathering at the place of meeting.

Interviewer: yah yah

Respondent: but that time we have not yet started off from the facility.

Interviewer: mmmm (showing that one is following the conversation)

Respondent: they wait for us until we …

Interviewer: until you get there

Respondent: meaning that they are ready, and they are just waiting for us

Interviewer: mmmm (showing that one is following the conversation)

Respondent: at the facility, even though we are at the facility due to a large number of tasks to perform you tend to find that the people become bored, they see that you have been told to do other things

Interviewer: mmmm (showing that one is following the conversation)

Respondent: and you haven’t attended to them, it is a bit different with the way it is in the community

Interviewer: alright.

Respondent: for example, in the community it is possible to find 150 women,

Interviewer: yah

Respondent: they are waiting at one place for the providers, which shows that they don’t have a problem, and when its one day I the month they are very sure that the providers will come on such such a date

Title: ZA_HSA_6.docx

Descriptor Info:	District: Zomba	Target Population: TP1 - Providers/HSAs	Respondent type: HSA	Aware of SI: Yes - had heard of SI before	Coding phase: 1. Single coded	Client marital status: N/A - provider	Client age group: N/A - provider	Time since trained:

Codes Applied:	P3.4 - Time to counsel/train, general

Interviewer: How easy or difficult has it been training women in DMPA-SC?

Respondent: Ah there are difficulties, it’s not hard to train them, but sometimes since we combine services, let’s say that we have a clinic that you should provide vaccination, you should do growth monitoring for children, maybe you should assess infant nutrition plus you should also provide family planning.

Interviewer: mmmm (showing that one is following the conversation)

Respondent: sometimes the clients become tired because there was another program which they were waiting for

Interviewer: yah yah

Respondent: so sometimes it, if it was possible that we have a special clinic for family planning that we are going just to do family planning services only then it would have not been a big problem, but maybe because the time is not enough, we may spend 15 minutes or even 20 minutes with one client, and sometimes it is very hard.

Title: ZA_HSA_6.docx

Descriptor Info:	District: Zomba	Target Population: TP1 - Providers/HSAs	Respondent type: HSA	Aware of SI: Yes - had heard of SI before	Coding phase: 1. Single coded	Client marital status: N/A - provider	Client age group: N/A - provider	Time since trained:

Codes Applied:	P3.4 - Time to counsel/train, general	C1.2.2 - Fear of SI (idea or reality)

Interviewer: alright, how long does it take to train the clients who would like to access DMPA-SC?

Respondent: Ah if the training is a group training.

Interviewer: mmmm (showing that one is following the conversation)

Respondent: then its 15 to 20 minutes

Interviewer: okay

Respondent: they have already known about everything that is required

Interviewer: yah yah

Respondent: but maybe the technic of, self-injection is the one that takes some time because of fear of self-injecting.

Interviewer: mmmm (showing that one is following the conversation)

Respondent: but it’s not like it can go on for 30 minutes if you are training is a group

Title: ZA_HSA_6.docx

Descriptor Info:	District: Zomba	Target Population: TP1 - Providers/HSAs	Respondent type: HSA	Aware of SI: Yes - had heard of SI before	Coding phase: 1. Single coded	Client marital status: N/A - provider	Client age group: N/A - provider	Time since trained:

Codes Applied:	P3.4 - Time to counsel/train, general

Interviewer: Alright, does the time that you take to train women in DMPA-SC differ when you are working with adolescents compared to older women?

Respondent: it cannot be a big difference, because maybe the group of adolescents, 80 percent don’t have an interest in contraceptives

Interviewer: mmmm (showing that one is following the conversation)

Respondent: and they also don’t follow the methods, that’s why they don’t access them yeah.

Interviewer: yah yah

Respondent: so maybe it can take a long time because of questions and other things like that.

Interviewer: alright

Respondent: for the older women, they already know, some of them have already accessed and they just stopped because they bore children and what so own yeah, they already have the knowledge, so it does not take time.

Interviewer: alright

Respondent: sure

Interviewer: Do you think you have enough time to train women in DMPA-SC?

Respondent: Ah like the way that I have explained

Interviewer: mmmm (showing that one is following the conversation)

Respondent: we can have enough time but because of the fact that we provide the services in one place

Interviewer: yah

Respondent: because of the service combination it confuses with other programs which are important in themselves.

Title: ZA_HSA_6.docx

Descriptor Info:	District: Zomba	Target Population: TP1 - Providers/HSAs	Respondent type: HSA	Aware of SI: Yes - had heard of SI before	Coding phase: 1. Single coded	Client marital status: N/A - provider	Client age group: N/A - provider	Time since trained:

Codes Applied:	P3.3 - Counselling/ training approach, general	P3.4 - Time to counsel/train, general

Interviewer: Do you think that the length of the training on DMPA-SC can influence the decision to get DMPA-SC?

Respondent: Ah firstly, no

Interviewer: mmmm (showing that one is following the conversation)

Respondent: because we have seen other women they just come to the clinic, they did not have any program to access family planning but because of the training that they have heard, they have heard about the advantages in the training

Interviewer: yah

Respondent: you will see that they have now accessed the methods that same day, so we can see that there is a big advantage

Interviewer: mmmm (showing that one is following the conversation)

Respondent: when the training as been delivered to the women properly

Title: MZs_FAC_2.docx

Descriptor Info:	District: Mzimba South	Target Population: TP1 - Providers/HSAs	Respondent type: Facility-based provider	Aware of SI: Yes - had heard of SI before	Coding phase: 2. Double coded	Client marital status: N/A - provider	Client age group: N/A - provider	Time since trained:

Codes Applied:	P3.4 - Time to counsel/train, general

Q14. Interviewer: How long on average does it take to train clients in DMPA-SC for self-injection?

Respondent: It depends if you are counselling client who went to school it’s very easy for them to understand the process but if the client did not attend school it takes a bit longer.

Interviewer: Alright

Respondent: Sometimes we use one or two Uniject for a client to understand it

Interviewer: How long do you take?

Respondent: 30 minutes

Interviewer: So do you think you have enough time?

Respondent: For others it is enough time, but other we spend up to 1hour sometimes even 1hour,30 minutes.

Title: MZs_FAC_2.docx

Descriptor Info:	District: Mzimba South	Target Population: TP1 - Providers/HSAs	Respondent type: Facility-based provider	Aware of SI: Yes - had heard of SI before	Coding phase: 2. Double coded	Client marital status: N/A - provider	Client age group: N/A - provider	Time since trained:

Codes Applied:	P3.4 - Time to counsel/train, general

Interviewer: Does the length of the training influence whether a woman might choose to take up self-injection?

Respondent: No, providers might spend a lot of time so that the client should understand it very well

Title: MZs_HSA_6.docx

Descriptor Info:	District: Mzimba South	Target Population: TP1 - Providers/HSAs	Respondent type: HSA	Aware of SI: Yes - had heard of SI before	Coding phase: 1. Single coded	Client marital status: N/A - provider	Client age group: N/A - provider	Time since trained: A year or more ago

Codes Applied:	P3.4 - Time to counsel/train, general	P3.1.4 - Provider sufficient support to counsel/train on SI

Interviewer: how long does it take to counsel a client on Sayana

Respondent: it depends on how a client understand. It takes about 30 minutes or more

Interviewer: for those who understand slowly how long does it take?

Respondent: 30-45 minutes

Interviewer: those who understands faster?

Respondent: 25 minutes some 15 minutes

Interviewer: is there a difference when you counsel adolescents

Respondent: no

Interviewer: do you have enough time to counsel a woman or an adolescent

Respondent: yes

Interviewer: what about on your busy days

Respondent: yes. We just divide the time and the clinics. We may schedule family planning clinic after 12 so that we have enough time for counselling

Title: KK_HSA_6.docx

Descriptor Info:	District: Nkhotakota	Target Population: TP1 - Providers/HSAs	Respondent type: HSA	Aware of SI: Yes - had heard of SI before	Coding phase: 1. Single coded	Client marital status: N/A - provider	Client age group: N/A - provider	Time since trained: A year or more ago

Codes Applied:	P3.4 - Time to counsel/train, general

INTERVIEWER: How many hours do you take, or minutes for a client to understand?

RESPONDENT: Maximum is 30 minutes. Most of them understand in 30 minutes. Sometimes when a I am not busy and they come to my house, you discuss with her for about 40 minutes. And then she understands and says give me I will be able to self-inject. And some say I will inject at home but we tell them to do it in our presence first because they might fail at home. If you will do it here, it means you will also do it at home. Some they insist that we should just give them they will inject at home and we do. And they come back to tell us that I self-injected and there is no problem

INTERVIEWER: Do you feel you have enough time to do conduct the trainings?

RESPONDENT: Time is there if you are not busy. But if you are busy, you just give her an appointment. But when we are doing under-five clinics, we counsel them in that group because sometimes we have hundred something or two hundred. So we counsel them on all the contraceptive methods and some they get interested maybe one or 2. We ask them what we were telling them at the under-five clinic and they explain. If its girls, we train them for 30 to 40 minutes if we are not busy because shortcuts are not good in contraceptives. They have to understand the advantages and disadvantages.

Title: MZs_FAC_1.docx

Descriptor Info:	District: Mzimba South	Target Population: TP1 - Providers/HSAs	Respondent type: Facility-based provider	Aware of SI: Yes - had heard of SI before	Coding phase: 3. Triple coded	Client marital status: N/A - provider	Client age group: N/A - provider	Time since trained: A year or more ago

Codes Applied:	P3.4 - Time to counsel/train, general	P3.1.4 - Provider sufficient support to counsel/train on SI	P3.1.5 - Ease of training clients in SI

Q 13. Interviewer: How easy or difficult it to train client in DMPA-SC for self-injection?

Respondent: Is not so difficult only that it really need time.

Interviewer: Mmmmmm.

Respondent: To explain to the client, you need to have uninterrupted time and also sometimes nurses are busy and a client come and say I need injection and you are busy, then you just refer them to the HSA`s to assist because you need to sit down and start explain all the procedures up until you are convinced that she can do it. So it’s not that difficult but you just need time to do it.

Title: MZs_FAC_1.docx

Descriptor Info:	District: Mzimba South	Target Population: TP1 - Providers/HSAs	Respondent type: Facility-based provider	Aware of SI: Yes - had heard of SI before	Coding phase: 3. Triple coded	Client marital status: N/A - provider	Client age group: N/A - provider	Time since trained: A year or more ago

Codes Applied:	P3.4 - Time to counsel/train, general

Q 14. Interviewer: Alright. Thank you so much. I just want to know how long on average does it take to train client on DMPA-SC?

Respondent: On average I would say 20 minutes.

Interviewer: Aaaaah, that 20 minutes is it for adults or adolescents?

Respondent: It’s for both, though adolescent can be a bit less and I can put at 15 minutes.

Interviewer: Alright. Do you feel you have enough time to conduct these trainings?

Respondent: Yah, we usually have that time as I said that we usually have specific days for family planning, so if it is a family planning day we usually allocate a nurse saying that you are one who is going to be at family planning today and that day it means she will spend her time in family planning.

Interviewer: Mmmmh

Respondent: Aaaaah, usually group counselling where you counsel them in groups for nurses usually its not a problem but sometimes you find that they come at different time and thus when you find that the time is not enough with the nurses, so they tend to refer but on a specific day for family planning we have enough time.

Interviewer: What about on a busy day?

Respondent: On a busy day thus when challenge comes

Title: MZs_FAC_1.docx

Descriptor Info:	District: Mzimba South	Target Population: TP1 - Providers/HSAs	Respondent type: Facility-based provider	Aware of SI: Yes - had heard of SI before	Coding phase: 3. Triple coded	Client marital status: N/A - provider	Client age group: N/A - provider	Time since trained: A year or more ago

Codes Applied:	P3.4 - Time to counsel/train, general	C3.2.2 - Lack of inclination or confidence to inject, even after training

Interviewer: Alright. Does the length of training influence whether a woman choose to take up self-injection?

Respondent: No

Interviewer: I just want to know that when you spent alit of time with client, does it influence whether a woman can choose to take up self-injection?

Respondent: Yah, time factors is also very important because as we are saying it goes with confidence and demonstrations it really need to have much time to seat and demonstrate to her. So say you are in a hurry and you have just bra..bra…bra..(not counselling it very well), she might not have that confidence, so we need to take much of time to explain then she will say I can do it.

Title: ZA_SCPI_8.docx

Descriptor Info:	District: Zomba	Target Population: TP2 - DMPA-IM/DMPA-SC PI clients	Respondent type: DMPA-SC PI client	Aware of SI: Yes - had heard of SI before	Coding phase: 1. Single coded	Client marital status:	Client age group:	Time since trained: N/A - client

Codes Applied:	P3.4 - Time to counsel/train, general

Respondent: so when we went back, they told us that they are busy with some work.

Interviewer: mmmm (showing that one is following the conversation)

Respondent: so we were not able to get the injectable.

Interviewer: okay

Title: ZA_HSA_1.docx

Descriptor Info:	District: Zomba	Target Population: TP1 - Providers/HSAs	Respondent type: HSA	Aware of SI: Yes - had heard of SI before	Coding phase: 1. Single coded	Client marital status: N/A - provider	Client age group: N/A - provider	Time since trained: Less than a year ago

Codes Applied:	P3.4 - Time to counsel/train, general

Interviewer: Alright alright thank you very much. How long to train a client in DMPA-SC?

Respondent: Ahhh probably 15 mins

Title: ZA_HSA_1.docx

Descriptor Info:	District: Zomba	Target Population: TP1 - Providers/HSAs	Respondent type: HSA	Aware of SI: Yes - had heard of SI before	Coding phase: 1. Single coded	Client marital status: N/A - provider	Client age group: N/A - provider	Time since trained: Less than a year ago

Codes Applied:	P3.4 - Time to counsel/train, general

Interviewer: Do you feel you have enough time to conduct these trainings?

Respondent: To clients, ahh it’s enough

Respondent: Alright

Title: ZA_HSA_1.docx

Descriptor Info:	District: Zomba	Target Population: TP1 - Providers/HSAs	Respondent type: HSA	Aware of SI: Yes - had heard of SI before	Coding phase: 1. Single coded	Client marital status: N/A - provider	Client age group: N/A - provider	Time since trained: Less than a year ago

Codes Applied:	P3.4 - Time to counsel/train, general

Interviewer: Does the length of training influence whether some women choose to take up self-injection?

Respondent: No, there is no relationship

Title: MZs_HSA_2.docx

Descriptor Info:	District: Mzimba South	Target Population: TP1 - Providers/HSAs	Respondent type: HSA	Aware of SI: Yes - had heard of SI before	Coding phase: 1. Single coded	Client marital status: N/A - provider	Client age group: N/A - provider	Time since trained: A year or more ago

Codes Applied:	P3.4 - Time to counsel/train, general

Interviewer: how long does it take to counsel a woman on Sayana Press

Respondent: we counsel a woman on all methods, so it takes about 45 mins for her to ask questions as well

Interviewer: what about on adolescents

Respondent: 45 min

Interviewer: do you feel you have enough time when counselling women and adolescents

Respondent: yes, we do

Interviewer: in your busy days, do you have enough time to counsel on Sayana Press

Respondent: yes, we do

Title: MZs_HSA_2.docx

Descriptor Info:	District: Mzimba South	Target Population: TP1 - Providers/HSAs	Respondent type: HSA	Aware of SI: Yes - had heard of SI before	Coding phase: 1. Single coded	Client marital status: N/A - provider	Client age group: N/A - provider	Time since trained: A year or more ago

Codes Applied:	P3.4 - Time to counsel/train, general	C8.1 - Male involvement, general	C8.2 - Covert/ open use of injectables/SI

Interviewer: does the time you tale to counsel a woman on Sayana Press influences her decision on whether to get Sayana or not

Respondent: not necessarily because when teaching them we provide information on the physiology so it’s not possible for them to have fears or be influenced by other women who had experienced side effects because these side effects vary

Interviewer: I am asking in terms of the time you spend on a woman. Would this affect her decision

Respondent: yes, especially those who come where without telling their husbands might opt for another method which is quicker to access

Title: MZs_HSA_2.docx

Descriptor Info:	District: Mzimba South	Target Population: TP1 - Providers/HSAs	Respondent type: HSA	Aware of SI: Yes - had heard of SI before	Coding phase: 1. Single coded	Client marital status: N/A - provider	Client age group: N/A - provider	Time since trained: A year or more ago

Codes Applied:	P3.4 - Time to counsel/train, general	A6.1.2 - Potential concerns about SI

Interviewer: what about adolescents. What factors would discourage them to use Sayana Press

Respondent: maybe the time we spend teaching them. Most of them see it as time wasting

Title: KK_FAC_2.docx

Descriptor Info:	District: Nkhotakota	Target Population: TP1 - Providers/HSAs	Respondent type: Facility-based provider	Aware of SI: Yes - had heard of SI before	Coding phase: 1. Single coded	Client marital status: N/A - provider	Client age group: N/A - provider	Time since trained: A year or more ago

Codes Applied:	P3.4 - Time to counsel/train, general

INTERVIEWER: Okay, mmmh. How long on average does it take to train clients on DMPA-SC? Approximately, how long does counselling take and also the training?

RESPONDENT: It’s not all of them, but because am just guessing, it should be 7-10 minutes.

INTERVIEWER: 7-10 minutes?

RESPONDENT: mmmh.

INTERVIEWER: which means you do the counselling and also…

RESPONDENT: I am saying…ah ah (no). are we specifically talking about Sayana?

INTERVIEWER: yes.

RESPONDENT: mmmh.

INTERVIEWER: Okay, 7 minutes. This 7 minutes for explaining and also showing her how she can self-inject right?

RESPONDENT: Mmmmmh mmmmh [no]. I am saying you can start self-injecting; this works in this way and the Uniject is like this, in 7 minutes.

INTERVIEWER: okay, 7 minutes.

RESPONDENT: mmmh.

INTERVIEWER: Up to the point she will know how to self-inject, 7 minutes?

RESPONDENT: We are saying that, have you seen this Uniject, yes, this is Sayana and explaining to her how it works. So you can do like this to inject yourself.

INTERVIEWER: mmhu.

RESPONDENT: mmmh.

INTERVIEWER: How about the process of teaching her and she has grasped the idea on how she can inject herself and maybe she has tried self-injecting, how minutes does it take?

RESPONDENT: 15 minutes.

INTERVIEWER: 15.

RESPONDENT: everything.

INTERVIEWER: everything

RESPONDENT: mmmh.

INTERVIEWER: 15 minutes

RESPONDENT: mmmmh 15.
